# An immune-related prognostic signature associated with immune landscape and therapeutic responses in gastric cancer

**DOI:** 10.18632/aging.204534

**Published:** 2023-02-22

**Authors:** Jian-Rong Sun, Chen-Fan Kong, Xiang-Ke Qu, An-Tao Sun, Kun-Peng Zhao, Jin-Hui Sun

**Affiliations:** 1 School of Clinical Medicine, Beijing University of Chinese Medicine, Beijing 100029, China; 2 School of Clinical Medicine, Shanghai University of Traditional Chinese Medicine, Shanghai 201203, China; 3 Department of Hematology, Guang’anmen Hospital, Beijing 100053, China; 4 School of Traditional Chinese Medicine, Gansu University of Chinese Medicine, Lanzhou 730000, China; 5 Department of Gastroenterology, Beijing University of Chinese Medicine Affiliated Dongzhimen Hospital, Beijing 100700, China

**Keywords:** gastric cancer, immune-related signature, prognosis, immune infiltration, immunotherapy

## Abstract

Immune-related genes (IRGs) have attracted attention in recent years as therapeutic targets in various tumors. However, the role of IRGs in gastric cancer (GC) has not been clearly elucidated. This study presents a comprehensive analysis exploring the clinical, molecular, immune, and drug response features characterizing the IRGs in GC. Data were acquired from the TCGA and GEO databases. The Cox regression analyses were performed to develop a prognostic risk signature. The genetic variants, immune infiltration, and drug responses associated with the risk signature were explored using bioinformatics methods. Lastly, the expression of the IRS was verified by qRT-PCR in cell lines. In this manner, an immune-related signature (IRS) was established based on 8 IRGs. According to the IRS, patients were divided into the low-risk group (LRG) and high-risk group (HRG). Compared with the HRG, the LRG was characterized by a better prognosis, high genomic instability, more CD8+ T cell infiltration, greater sensitivity to chemotherapeutic drugs, and greater likelihood of benefiting from the immunotherapy. Moreover, the expression result showed good consistency between the qRT-PCR and TCGA cohort. Our findings provide insights into the specific clinical and immune features underlying the IRS, which may be important for patient treatment.

## INTRODUCTION

Gastric cancer (GC) is the fifth most common malignancy, with 1,089,103 new cases and 768,793 new deaths being reported globally in 2020 alone [1]. Due to the insidious onset of GC and lack of overt early symptoms, most patients are already in the advanced stage at the time of diagnosis, resulting in missed opportunities for surgical resection. Despite the fact that development of treatment approaches for advanced GC has seen much improvement in recent years, the 5-year survival rate of <20% is still clearly unsatisfactory [2]. In addition, the high degree of heterogeneity of GC leads to vastly different prognoses and therapeutic responses. Thus, there is an urgent need to develop prognostic signatures to predict outcomes and guide individualized treatments.

The tumor microenvironment (TME) comprises tumor cells, resident and recruited host cells (cancer-related stromal cells and immune cells), and secreted products of these cells (such as cytokines), which is closely related to the occurrence and progression of tumors [3]. Accumulating evidence suggests that, among the various cell types in the TME, the abundance and type of tumor-infiltrating immune cells significantly influences the outcome of immunotherapy and tumor progression. For instance, high infiltration of T cells is associated with better immune checkpoint inhibitor (ICI) efficacy [4]. The CD8+ Foxp3+ T lymphocyte infiltration is increased with tumor progression as reflected by TNM stage, indicating their important role in GC progression [5]. Thus, the systematic investigation of immune phenotypes within the GC microenvironment represents a promising approach for better understanding the complex antitumor response and to guide effective immunotherapies. Several studies have reported immune-related signatures (IRS) in GC, some of them have explored the prognosis of GC patients by establishing immune-related prognostic models, others have further explored the tumor microenvironment, and still others have performed similar analyses on restricted populations only [6–9]. It is well known that immunotherapy is one of the mainstream modalities in oncology treatment today, but it remains a challenge to differentiate patients with potential immunotherapy response before treatment initiation. The studies on the immunotherapy response and chemotherapy sensitivity in GC have largely not been reported.

In the present study, we aimed to identify and validate a novel immune-related signature and a matching nomogram that can be utilized for predicting prognosis. This prognostic signature was conducive to the identification of immune infiltrating cells in the TME and assessment of immunotherapeutic response. Moreover, this immune-related signature was used for computing the IC50 of chemotherapeutic agents to predict the drug sensitivity.

## MATERIALS AND METHODS

**Figure 1:**
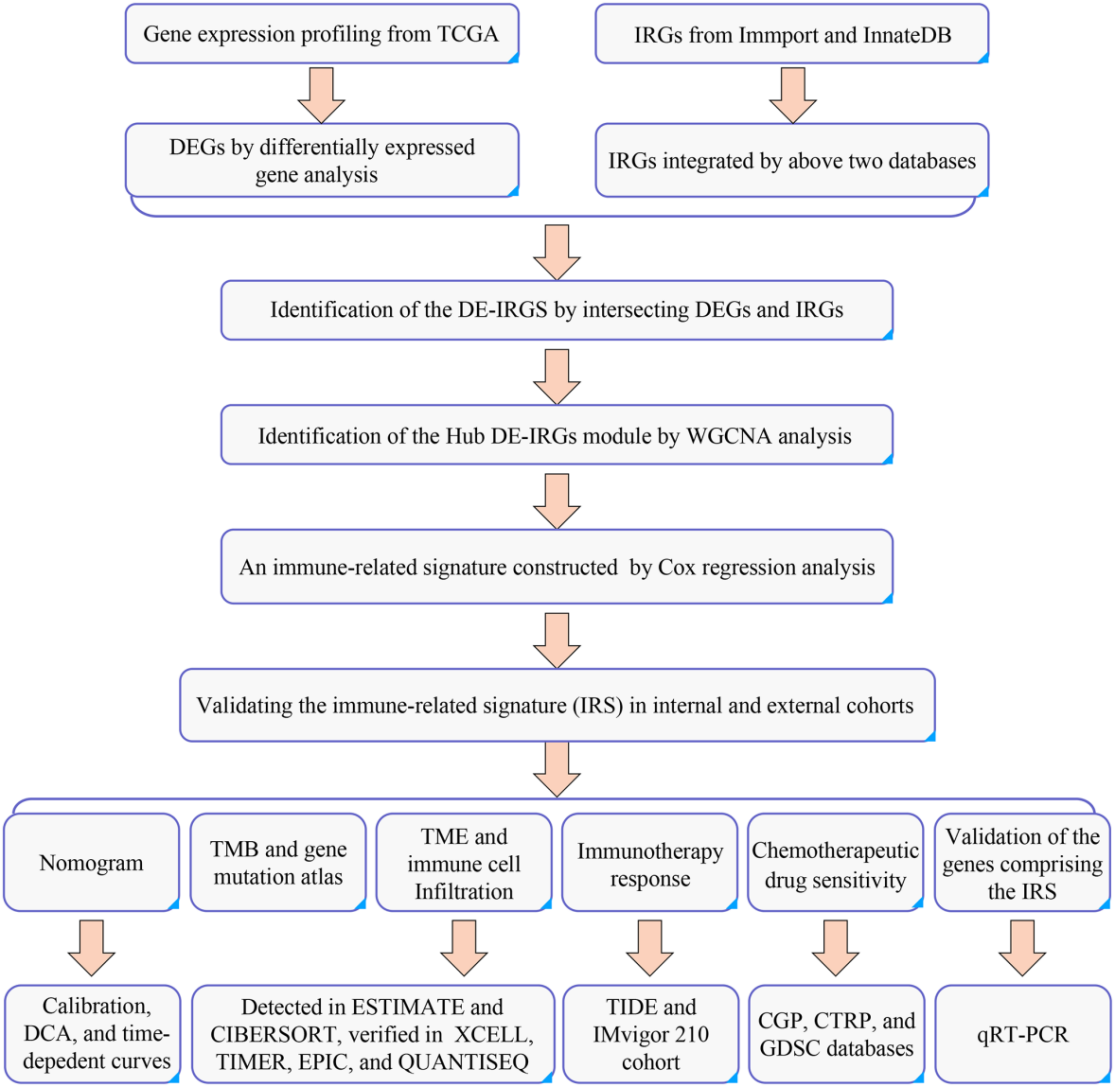
The flowchart of the current study.

A flowchart of this study presented in Figure 1.

### Data acquisition

The transcriptional expression data, somatic mutation patterns, and matching clinical information (including survival time, vital status, age, gender, tumor grade, and pathological stage) of GC patients were obtained from the Cancer Genome Atlas (TCGA, https://portal.gdc.cancer.gov) database (Supplementary Table 1). An independent external GC cohort GSE84437 containing microarray data and corresponding clinical information was downloaded from the Gene Expression Omnibus (GEO, https://www.ncbi.nlm.nih.gov/geo/) database as well (Supplementary Table 1). The gene expression profile and the detailed clinical annotations of an immunotherapy cohort (IMvigor210) were acquired online (http://research-pub.gene.com/IMvigor210CoreBiologies) and utilized to further validate the efficiency of the immune-related signature (Supplementary Table 2). The immune-related gene list was obtained from the ImmPort (https://www.immport.org) and InnateDB (https://www.innatedb.ca) databases (Supplementary Tables 3, 4). As all data were obtained from public databases, ethical approval was not necessary.

### Data processing

For transcriptional expression data from the TCGA database, FPKM data were transformed into TPM (transcripts per kilobase of exon model per million mapped reads) values which is more similar to microarray data, to make them more comparable between different samples [10]. The mRNA Ensembl IDs in the TCGA dataset were switched to gene symbols according to GENCODE (https://www.gencodegenes.org/). For microarray data in GEO, the probe ID in the gene expression dataset was annotated to a gene symbol by platform files (GPL6947 Illumina HumanHT-12 V3.0 expression beadchip). Next, the above datasets were normalized by log2 transformation, and the batch effects were adjusted using the ComBat function in the R3.7.0 software “sva” package [11]. mRNAs with gene expression values of 0 in >90% samples both in TCGA and GEO datasets were excluded, as they were regarded as transcriptional noise. Averaging was performed for mRNAs with more than one-row expression values.

### Differentially expressed gene analysis (DEGA), differentially expressed IRG (DE-IRG) screening and functional enrichment analysis

The R “limma” package was used for identifying differentially expressed genes (DEGs) between the adjacent normal and GC samples. Those with |logFC| > 1 and FDR < 0.05 were regarded as DEGs. Next, the DEGs and IRGs were intersected to obtain DE-IRGs, and a heatmap was used to visualize the gene expression profiles of DEGs and DE-IRGs respectively by R “pheatmap” package.

### GO and KEGG enrichment analysis of DE-IRGs

Gene ontology (GO) and Kyoto Genome Encyclopedia (KEGG) enrichment analyses were conducted for the DE-IRGs via the R “ClusterProfiler” package to detect their underlying biological function. The GO terms and KEGG pathways with *P* < 0.05 were regarded as significant. The above results were visualized by R “ggplot2” and “enrichplot” packages eventually.

### Weighted gene co-expression network analysis (WGCNA) of DE-IRGs

WGCNA is a method for exploring gene expression patterns of multiple samples [12]. Genes with similar expression patterns can be clustered, and the relationship between modules and specific traits or phenotypes can be analyzed. Firstly, Pearson’s correlation matrix was defined based on the interaction coefficients among genes. An adjacency matrix was defined with the threshold of Pearson coefficient exceeding 0.8, which was further used to construct a topological overlap matrix (TOM). Finally, the TOM matrix was applied for determining the co-expression gene modules, in this process modules with statistical significance (*P* < 0.05) were regarded as cancer-related modules, and genes involved in the modules were considered more important and used for subsequent analysis.

### Identification of the immune-related signature (IRS)

In the discovery (TCGA) cohort, univariate Cox regression analysis was first conducted to detect the relationship between IRGs and GC prognosis. IRGs with *P* < 0.05 were regarded as having the potential to build the final prognostic signature. Next, to enhance the robustness of this final prognostic signature, the TCGA cohort was randomly divided into training and test subsets at a ratio of 5 to 5. In train subsets, IRGs with *P* < 0.05 in univariate Cox regression analysis were selected for inclusion in multivariate Cox regression analysis to establish the optimal prognostic signature. Particularly, the robustness of the prognostic signature was validated in the internal cohort (test cohort) and external cohort (validation cohort, GSE84437).

According to the prognostic signature, each patient was given a risk score by the following formula:


$$Risk\;Score=\sum_{k=1}^{n}Coef_{k}\times B_{k}$$

Where Coef_k_ is the coefficient and B_K_ is the normalized expression value of the IRGs included in the prognostic signature. GC patients were assigned to a low-risk group (LRG) or a high-risk group (HRG) according to the cohort-specific median risk score taken as the cut-off value. The performance of risk groups as determined by the risk score of the prognostic signature was evaluated by the Kaplan-Meier method with log-rank testing. Moreover, The ROC curves at 1, 3, and 5 years were plotted and AUC values were computed to estimate the predictive performance of the signature.

In addition, we divided patients into subgroups according to clinical characteristics such as age, pathological stage, etc. We then performed stratified survival analysis to study whether the IRS maintained predictive power in different cohorts.

### Development and validation of a combined IRG-clinical nomogram

A nomogram is a statistical predictive tool that combines multiple prognostic factors to assess the survival probability for individual patients. To investigate whether the IRG prognostic signature possesses independent predictive capacity. First, risk scores together with clinical parameters including age, gender, tumor grade, and pathological stage were investigated by univariate Cox regression analysis to filter prognostic factors in the TCGA cohort. The above variables with *P* < 0.05 were then selected for inclusion in multivariate Cox regression analysis to determine independent prognostic ability. A combined prognostic model consisting of factors with *P* < 0.05 in multivariate Cox regression analysis was compared with the clinical model or IRS model respectively with regard to time-dependent AUC value and calibration curve. Finally, the model was visualized by nomogram and assessed by DCA to detect whether using this nomogram could yield closer associations with clinical net-benefit than other models.

### Gene functions, tumor mutation burden, and somatic mutation profiling in the different risk subgroups

Gene set enrichment analysis (GSEA) is used to evaluate the distribution trend of genes in a predefined gene set in a gene table ranked by their relevance to phenotype, thereby judging their contribution to the phenotype [13]. The functional enrichment of genes in different risk subgroups was investigated in GSEA via the R “clusterProfiler” package. The somatic mutation characteristics of GC in the LRG and HRG were analyzed and visualized using the R “maftool” package. Moreover, the tumor mutation burden (TMB) for each patient was computed as mutations per million bases.

### Correlations between different risk subgroups, TME and immune cell infiltration pattern

The ESTIMATE algorithm is a tool using expression data for the estimation of stromal cells and infiltrating immune cells in malignant tumors to predict tumor purity [14]. Therefore, utilizing ESTIMATE generates three scores: a stromal score (that captures the presence of stroma in tumor tissue), an immune score (that represents the infiltration of immune cells in tumor tissue), and the estimate score (that infers tumor purity) to evaluate the main cell types in the TME. Additionally, CIBERSORT, a deconvolution algorithm supported by R package with default parameters utilizing gene expression profiles to quantify immune infiltration, was used for evaluating the proportions of 22 immune cell types in each GC tumor tissue [15]. As CIBERSORT computes an empirical *P*-value for the deconvolution to denote the accuracy of results, we only retained those samples with a CIBERSORT *P*-value < 0.05 for subsequent analysis. Furthermore, to verify the results of CIBERSORT, other algorithms such as XCELL, TIMER, EPIC, and QUANTISEQ were used for analyzing the tumor infiltration immune cells as well [16–19].

Also, based on the single sample gene set enrichment analysis (ssGSEA) method supported by the R “GSVA” package, the 29 common immune-associated pathways for each sample were given an enrichment score to quantify this. Furthermore, immune cell abundance and their corresponding functional pathways were compared in different risk groups. Finally, survival analysis was performed for immune cells and immune functions respectively to comprehensively explore the relationship between immune infiltration and GC prognosis. The macrophages could play diverse roles in tumorigenesis and progression, such as M1 macrophages (prone to anti-tumor) and M2 macrophages (pro-tumor). Thus, the expression of markers for M0, M1, and M2 macrophages were analyzed in different risk groups. Besides, expression of immune checkpoint molecules PD-1, PD-L1, and CTLA4 in different risk groups were also analyzed. Moreover, due to the cytokine and chemokine are key factors for immune cell recruitment and functions, the expression of them in the risk model was analyzed as well.

### The immunotherapy response and chemotherapeutic drug sensitivity in distinct risk subgroups

The tumor immune dysfunction and exclusion (TIDE) score calculated online (http://tide.dfci.harvard.edu/) was used to predict the immunotherapy response, the higher the TIDE score, the lower immunotherapy response [20]. Moreover, the IMvigor210 cohort was utilized to further verify the efficiency of the IRS in appraising immunotherapy responsiveness.

Based on the three public drug sensitivity databases, CGP (Cancer Genome Project), GDSC (Genomics of Drug Sensitivity in Cancer), and CTRP (Cancer Therapeutics Response Portal), the ridge regression models were built by the pRRophetic algorithm to predict chemotherapeutic responses. Gene expression profiling and risk grouping information were used in the model to estimate the half-maximal inhibitory concentrations (IC50) for each sample [21]. The prediction accuracy was evaluated by 10-fold cross-validation based on each training set. The smaller the IC50 value of the drug, the stronger its ability to inhibit cell growth and the better the effect of cancer treatment. Here, common GC therapeutic drugs, such as cisplatin, oxaliplatin, docetaxel, paclitaxel, 5-fluorouracil (5-Fu), capecitabine, and irinotecan were selected for analysis.

### Immunohistochemical analysis

The protein expression data were acquired from the Human Protein Atlas (HPA) database, a largest and most comprehensive database for evaluating protein distribution in human tissues [22]. The protein expression of the IRGs signature in normal and GC tissues was determined using the immunohistochemical staining images, and then Image J was used to perform the quantitative analysis.

### Validation of the immune-related signature by relative quantitative real-time PCR (qRT-PCR)

The expression levels of genes comprising the immune-relative signature were measured in a GC cell line (HGC-27, human gastric cancer cells) and a control cell line (GES-1, human gastric mucosal epithelial cells). All the cell lines were obtained from the National Infrastructure of Cell Line Resources (Beijing, China) and were in RPMI-1640 (FBS, Gibco, USA), 10% fetal bovine serum (FBS, Gibco, USA), and 1% penicillin/streptomycin (Gibco, Canada). All the cells were cultured at 37°C with 5% CO2. Total RNA was extracted from cells using the RNeasy Mini Kit (Qiagen, USA, Cat. 74104), and reverse transcription was subsequently performed using the 5∗All-in-one RT MasterMix (ABM, USA, Cat. No. G492). qRT-PCR was performed with a SYBR Green Real-time PCR Kit (Keygen Biotech, Nanjing, China, Cat. KGA1339-1) on a QuantStudio 5 Real-Time PCR System (Thermo Fisher Scientific, USA). All experiments were repeated at least three times. The RNA primer sequences are listed in Supplementary Table 5. Relative expression was calculated using the comparative threshold cycle (Ct) method. Simultaneously, the gene expression consisting of the signature was analyzed in the TCGA cohort.

### Statistical analysis

All statistical analyses were conducted via R software (version 3.7.0). Continuous and categorical data were analyzed by Wilcoxon and Chi-square methods respectively. Survival was estimated by the Kaplan-Meier method and the statistical significance was determined by log-rank testing. Correlations between two continuous characteristics were analyzed by the Spearman method. Univariate and multivariate Cox regression analyses were performed using the R “survival” package. The time-dependent AUC value was calculated by the R “timeROC” package, and ROC curves were plotted by R “survivalROC” package. *P* < 0.05 was regarded as statistically significant.

### Data availability statement

The data that support this study are openly available in online repositories and the raw data was uploaded (https://www.jianguoyun.com/c/sd/155e27d/6d60f576ace1d1cc).

## RESULTS

### Identification of DE-IRGs and functional enrichment analysis

**Figure 2:**
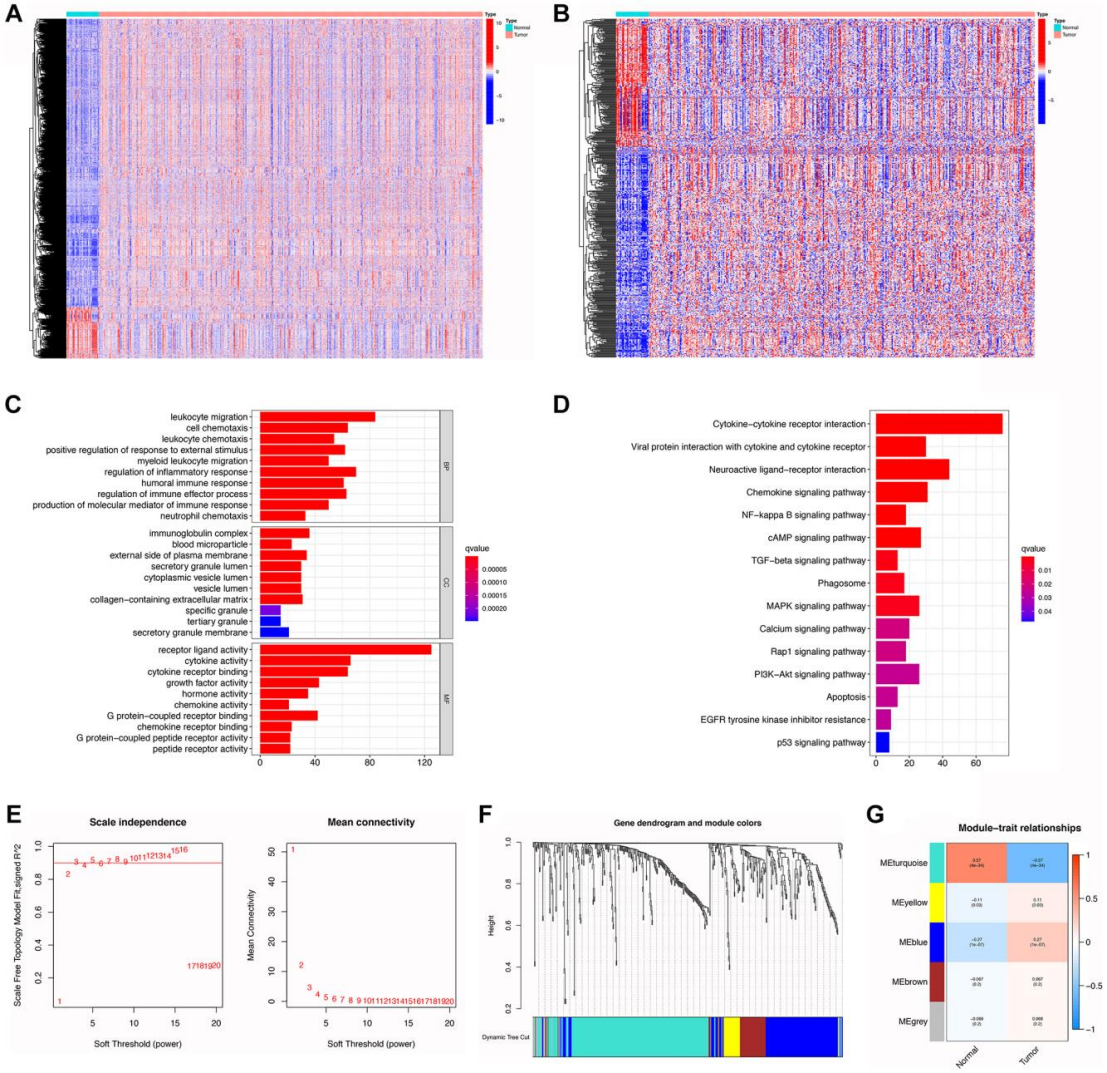
**Identification, functional enrichment, and WGCNA analysis of DE-IRGs.** (**A**, **B**) Heatmap of DEGs and DE-IRGs, respectively. (**C**, **D**) GO enrichment and KEGG analysis for DE-IRGs. (**E**) The scale-free fit index for soft-thresholding powers. Left: the relationship between the soft-threshold and scale-free R2. Right: the relationship between the soft-threshold and mean connectivity. (**F**) Dendrogram and module colors for DE-IRGs. (**G**) The correlations heatmap between modules and sample types.

With the cut-off set as logFC > 1, FDR < 0.05, there were 8833 DEGs identified totally, of which 1335 were down-regulated and 7498 were up-regulated (Supplementary Table 6). By searching ImmPort and InnateDB, 2483 and 1226 IRGs (Supplementary Tables 7, 8) were determined separately as well as 2660 IRGs were obtained by integrating IRGs from the two databases (Supplementary Table 9). Next, overlaps of DEGs and IRGs identified 493 DE-IRGs, comprising 184 that were down-regulated and 309 up-regulated (Supplementary Table 10). As shown in Figure 2A, 2B, there was a clear distinction of DEGs and DE-IRGs between normal and tumor samples.

These 493 DE-IRGs were further analyzed via GO and KEGG to explore their function. In GO enrichment analysis, the biological process (BP) term showed that these DE-IRGs were enriched in leukocyte migration, regulation of the inflammatory response, cell chemotaxis, regulation of immune effector processes, and regulation of responses to external stimuli. The cellular component (CC) terms showed that these DE-IRGs were mainly enriched for the immunoglobulin complex, external side of plasma membrane, and collagen-containing extracellular matrix. Concerning molecular function (MF), these DE-IRGs were enriched in terms of receptor-ligand activity, cytokine activity, cytokine receptor binding, and growth factor activity (Figure 2C). Moreover, KEGG pathway analysis documented that these DE-IRGs were principally enriched in cytokine-cytokine receptor interaction, neuroactive ligand-receptor interaction, chemokine signaling pathway, viral protein interaction with cytokine and cytokine receptor, cAMP signaling pathway, and MAPK signaling pathways (Figure 2D).

### Detecting hub DE-IRGs via WGCNA analysis

DE-IRGs were further selected for WGCNA analysis to distinguish different gene modules and then to identify the hub DE-IRGs. A soft threshold (power value) of 5 was selected to make sure that both the scale-free topology model fit index (R^2^) and mean connectivity were optimal (Figure 2E). A total of 5 modules was obtained by average linkage hierarchical clustering. Each module is shown in a different color in Figure 2F. Among these modules, MEturquoise possessed the highest correlation with GC traits (Figure 2G). There were 255 DE-IRGs included in the filtered MEturquoise module, which were chosen for further analysis (Supplementary Table 11).

### Construction and verification of the immune-related prognostic signature

**Figure 3:**
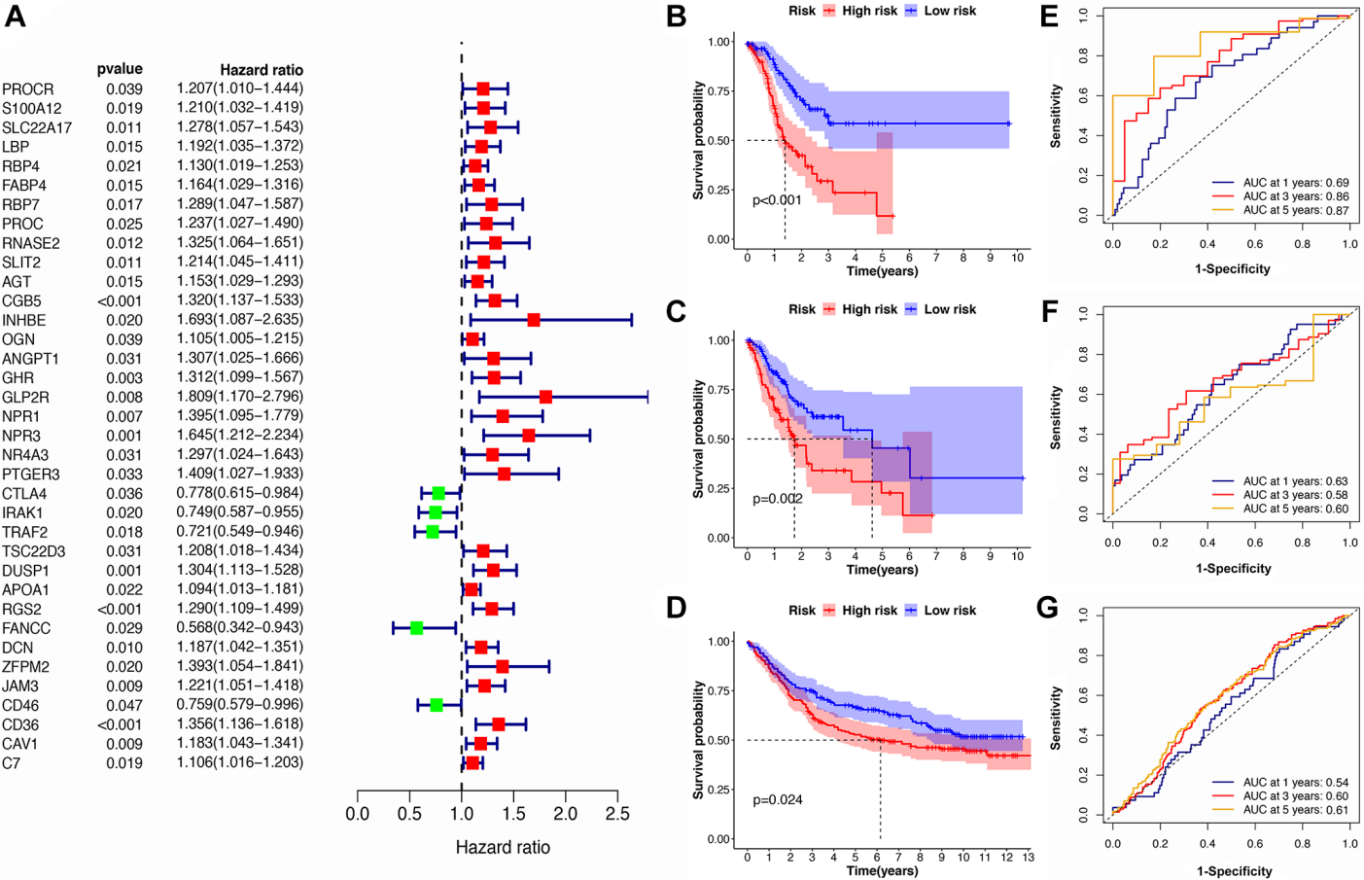
**Identification of the immune-related prognostic signature.** (**A**) The forest plot of univariate Cox regression analysis for IRGs. (**B**–**D**) The Kaplan-Meier survival curves for high- and low-risk groups in train, test, and validation cohorts. (**E**–**G**) The ROC curve for the IRS in train, test, and validation cohorts.

Utilizing univariate Cox regression analysis, a total of 36 genes was identified as being associated with GC prognosis (Figure 3A). We then constructed an 8-gene signature by multivariate Cox regression analysis in the training subset (Supplementary Table 12). The risk scores for each GC patients were calculated as follows:

Risk Score = (0.277) × *RNASE2* + (0.255) × *CGB5* + (0.501) × *INGBE* + (−0.348) × *PTGER3* + (−0.341) × *CTLA4* + (0.260) × *DUSP1* + (0.071) × *APOA1* + (0.234) × *CD36*.

Next, all GC patients were assigned to either a lower or higher risk group: LRG (*n* = 93) and HRG (*n* = 93). The results of Kaplan-Meier analysis and log-rank testing showed that patients in the LRG had a better overall survival rate (OS) than the HRG (Figure 3B). The AUC values of ROC curves for predicting survival outcomes at 1, 3, 5 years were 0.69, 0.86, and 0.87, respectively, demonstrating that the signature possesses good predictive capacity for GC prognosis (Figure 3C). In addition, the results of IRS validation showed that this signature maintain its prognosis predictive power in test subsets and validation cohorts as well (Figure 3D–3G).

Furthermore, we conducted stratified analysis for the IRS based on clinical characteristics such as age, gender, tumor grade, and pathological stage to explore the signature’s robustness. The results showed that patients in the HRG generally have a poor prognosis in different sub-cohorts compared with the LRG, which is consistent with the result from the integrated cohort. This confirms the robustness of this IRS in cohorts with different clinical features (Table 1).

**Table 1:** Cox survival analysis of the IRS in stratified GC cohort.

**Variables**	**TCGA-STAD**				**GSE84437**	
	**Training cohort**		**Test cohort**			
	**HR (95% CI)**	***P* value**	**HR (95% CI)**	***P* value**	**HR (95% CI)**	***P* value**
Age (years)						
≤65	6.46 (2.16–19.31)	<0.001	2.52 (1.13–5.65)	0.025	1.60 (1.01–2.56)	0.047
>65	2.22 (1.19–4.13)	0.012	1.88 (1.03–3.45)	0.040	1.36 (0.88–2.11)	0.170
Gender						
Male	3.19 (1.69–6.02)	<0.001	1.55 (0.85–2.82)	0.150	1.30 (0.94–1.80)	0.107
Female	2.76 (1.05–7.34)	0.040	3.13 (1.38–7.09)	0.006	1.71 (1.07–2.98)	0.036
Tumor grade						
G1/2	2.50 (0.98–6.37)	0.054	1.37 (0.61–3.06)	0.448	-	-
G3/4	2.61 (1.33–5.12)	0.005	2.41 (1.32–4.43)	0.004	-	-
Pathological stage						
Stage I/II	3.20 (1.22–8.36)	0.018	2.03 (1.78–4.63)	0.018	1.65 (1.31–2.65)	0.025
Stage III/IV	2.93 (1.55–5.55)	<0.001	2.02 (1.11–3.69)	0.021	1.83 (1.07–2.69)	0.015
T stage						
T1/2	2.94 (0.73–11.86)	0.130	2.87 (0.91–9.12)	0.073	0.49 (0.12–2.00)	0.326
T3/4	3.27 (1.82–5.87)	<0.001	1.99 (1.17–3.39)	0.011	1.40 (1.06–1.86)	0.019
N stage						
N0	4.32 (1.32–14.18)	0.016	2.39 (1.35–6.69)	0.027	1.98 (1.45–2.14)	0.017
N1–3	2.86 (1.53–5.34)	<0.001	1.86 (1.08–3.20)	0.025	1.37 (1.01–1.84)	0.040
M stage						
M0	3.14 (1.79–5.53)	<0.001	1.79 (1.07–2.99)	0.027	1.48 (1.04–2.11)	0.029
M1	2.45 (0.23–25.77)	0.455	2.76 (0.55–13.90)	0.218	1.21 (0.77–1.86)	0.408

The “-” indicates that the value is not available; Abbreviations: HR: hazard ratio; CI: confidence interval.

### Establishment and validation of an immune-clinical nomogram

**Figure 4:**
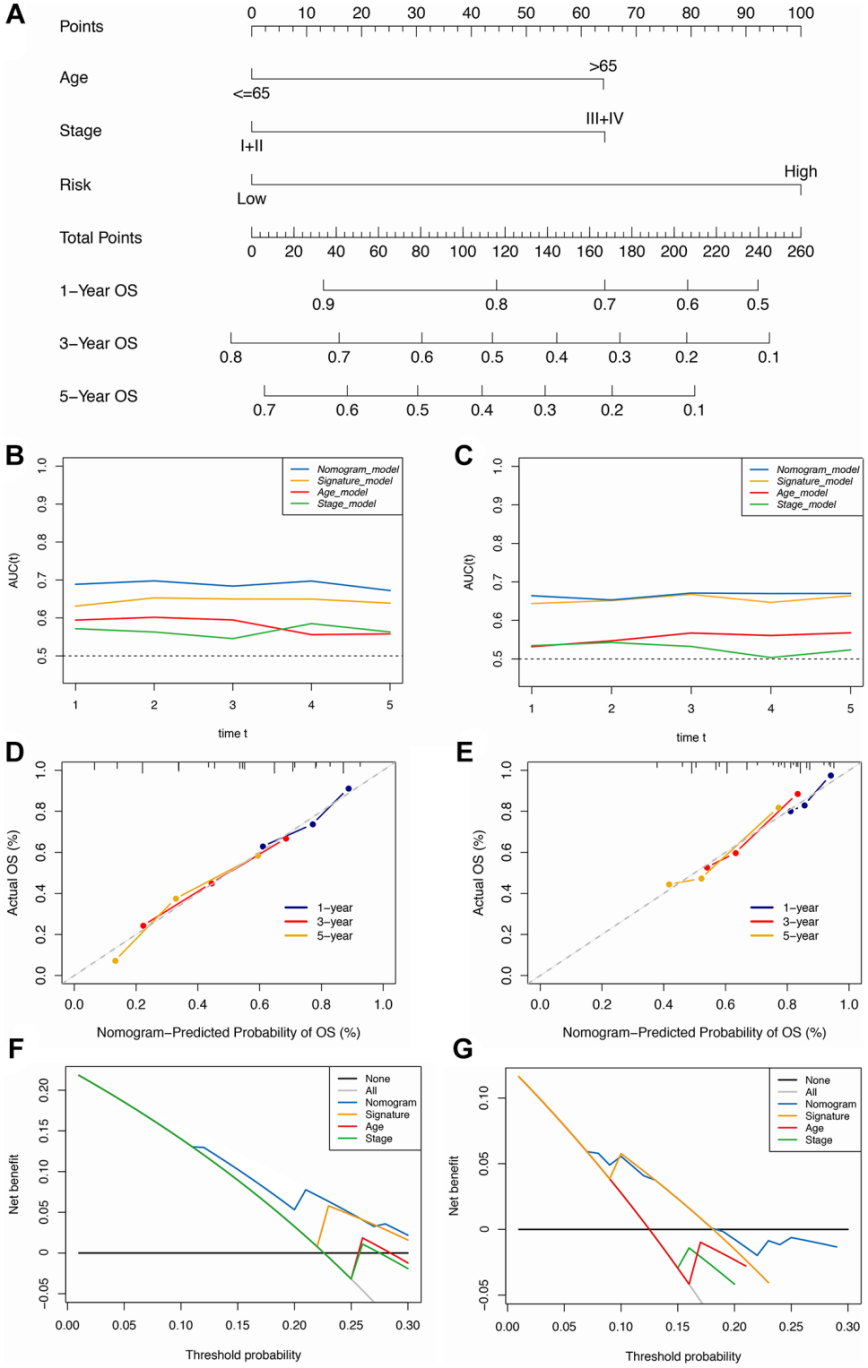
**Construction of the immune-clinical nomogram.** (**A**) The nomogram for predicting 1-year, 3-year, and 5-year OS for GC patients. (**B**, **C**) Time-dependent ROC curves for the nomogram, immune signature, age, and stage models at different time points in the TCGA and GEO datasets. (**D**, **E**) Calibration curves of observed and predicted probabilities for the nomogram in the TCGA and GEO datasets. (**F**, **G**) DCA curves for the nomogram in the TCGA and GEO datasets.

The Cox regression analysis of the discovery cohort showed that age, pathological stage, and risk score were independent prognostic factors for GC patients (Table 2). Therefore, these factors were combined to construct a nomogram for predicting GC patients’ short- and long-term survival rates (Figure 4A). The time-dependent AUC value of the nomogram remained higher than for other models over time, suggesting that combining the IRS with age and pathological stage improved accuracy for predicting survival outcomes (Figure 4B). The calibration curve of the nomogram showed a good performance in consistency between prediction and actual observation, especially for 3-year OS. Also, the DCA curve suggested that the nomogram possessed better value for clinical applications than the other models (Figure 4C, 4D).

**Table 2:** Univariate and multivariate Cox regression analysis for clinical variables.

**Variables**	**Univariate analysis**		**Multivariate analysis**	
	**HR (95% CI)**	***P* value**	**HR (95% CI)**	***P* value**
Age				
≤65	Reference		Reference	
>65	1.03 (1.01–1.04)	0.005	1.04 (1.02–1.05)	<0.001
Gender				
Female	Reference		*-*	*-*
Male	1.23 (0.85–1.76)	0.274	*-*	*-*
Tumor grade				
G1–2	Reference		*-*	*-*
G3–4	1.33 (0.93–1.91)	0.123	*-*	*-*
Pathological stage				
Stage I/II	Reference			
Stage III/IV	1.80 (1.26–2.57)	0.001	1.76 (1.21–2.55)	0.003
T				
T1–2	Reference			
T3–4	1.63 (1.05–2.52)	0.030	1.38 (0.84–2.29)	0.207
N				
N0	Reference		Reference	
N1–3	1.74 (1.15–2.62)	0.008	1.26 (0.72–2.20)	0.415
M				
M0	Reference		Reference	
M1	1.79 (1.09–2.95)	0.022	1.45 (0.85–2.48)	0.178
Risk Score				
Low	Reference		Reference	
High	1.57 (1.37–1.80)	<0.001	1.58 (1.37–1.84)	<0.001

The ‘-’ indicates that the value is not available; Abbreviations: HR: hazard ratio; CI: confidence interval.

In the validation cohort, the time-independent AUC value and calibration curve of the nonogram model maintained its good performance for predicting patients’ OS as well (Figure 4E, 4F). DCA revealed that utilizing the nomogram could bring more clinical net benefit (Figure 4G). Thus, the nomogram comprising IRS and clinical characteristics (pathological stage and age) appeared highly accurate in predicting the short- and long-term OS of GC patients in both the discovery and validation cohorts. The above results demonstrated that using this immune-clinical nomogram for prognosis prediction assistance might result in significant clinical benefit.

### GSEA analysis, TMB, and a gene mutation atlas in different risk subgroups

**Figure 5:**
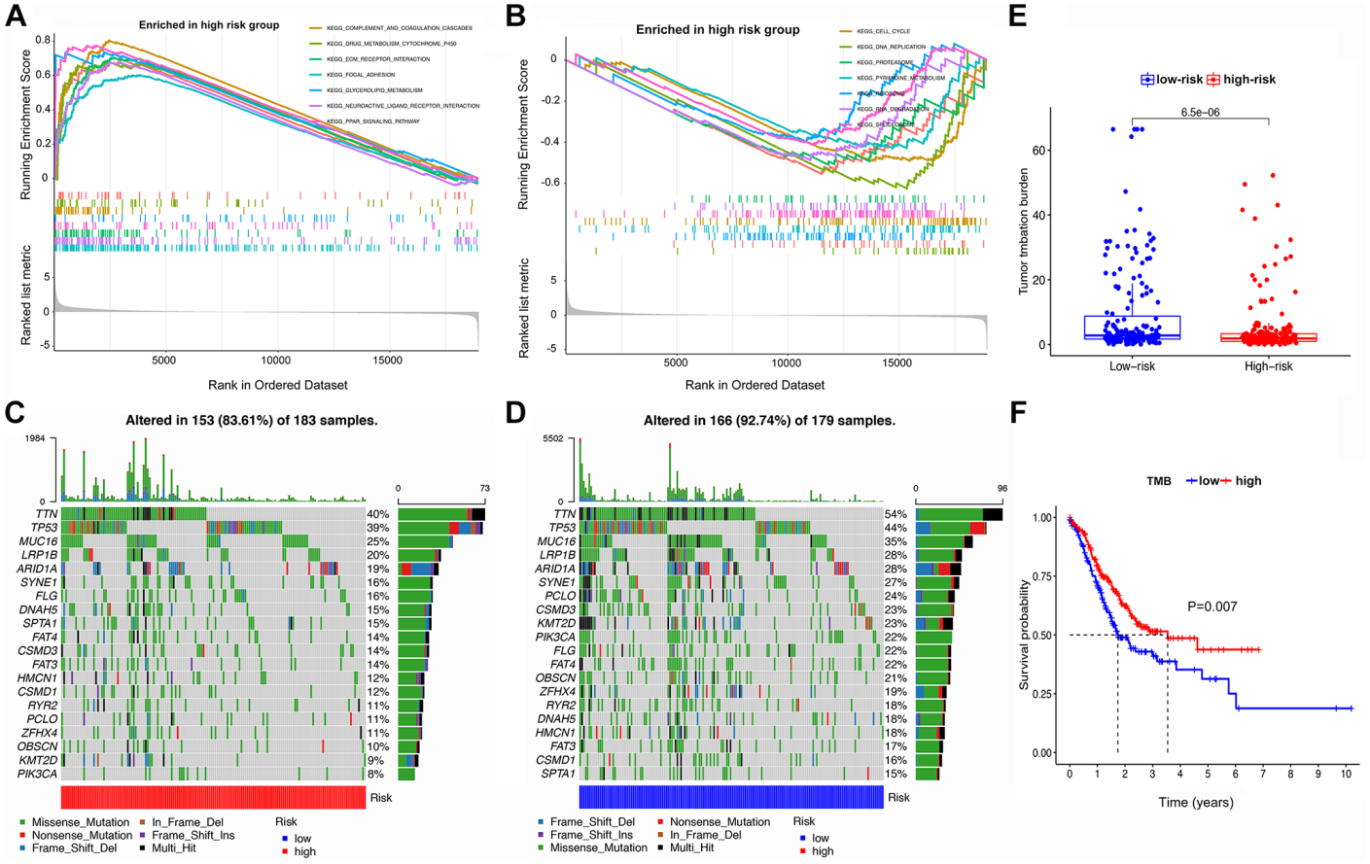
**GSEA, mutational landscape, and TMB in high- and low-risk groups stratified by the IRS.** (**A**, **B**) The enriched pathways for different risk groups based on GSEA analysis (**C**, **D**) Waterfall plot of the top 20 mutant genes in the high- and low-risk groups. (**E**) Box plot for the TMB between the high- and low-risk groups. (**F**) Kaplan-Meier survival curve of high versus low TMB.

As shown in Figure 5A, 5B, GSEA analysis indicated that tumors in the HRG were enriched in complement and coagulation cascades, ECM receptor interaction, focal adhesion, neuroactive ligand-receptor interaction, and PPAR signaling pathway. In contrast, the LRG was enriched in aminoacyl tRNA biosynthesis, cell cycle, DNA replication, pyrimidine metabolism, and spliceosome terms.

Summaries of gene mutation profiles for the different risk groups are shown in the Supplementary Figures 1 and 2, suggesting that the LRG has a higher overall mutation frequency than the HRG. Nonetheless, the top six genes with the highest mutation rates in the LRG were *TTN* (54%), *TP53* (44%), *MUC16* (35%), *LRP1B* (28%), *ARID1A* (28%), and *SYNE1* (27%), which is similar to the HRG with *TTN* (40%), *TP53* (39%), *MUC16* (25%), *LRP1B* (20%), *ARID1A* (19%), and *SYNE1* (16%). The most common mutation type was missense mutation in both LRG and HRG (Figure 5C, 5D).

TMB analysis in the HRG and LRG is shown in Figure 5E, 5F, suggesting that the latter has a higher TMB. In addition, survival analysis of patients stratified for TMB indicated that a high TMB resulted in a better prognosis than a low TMB.

### Analysis of the tumor microenvironment, immune cell infiltration, and expression of markers for macrophages, immune checkpoint, cytokine, and chemokine in different risk groups

**Figure 6:**
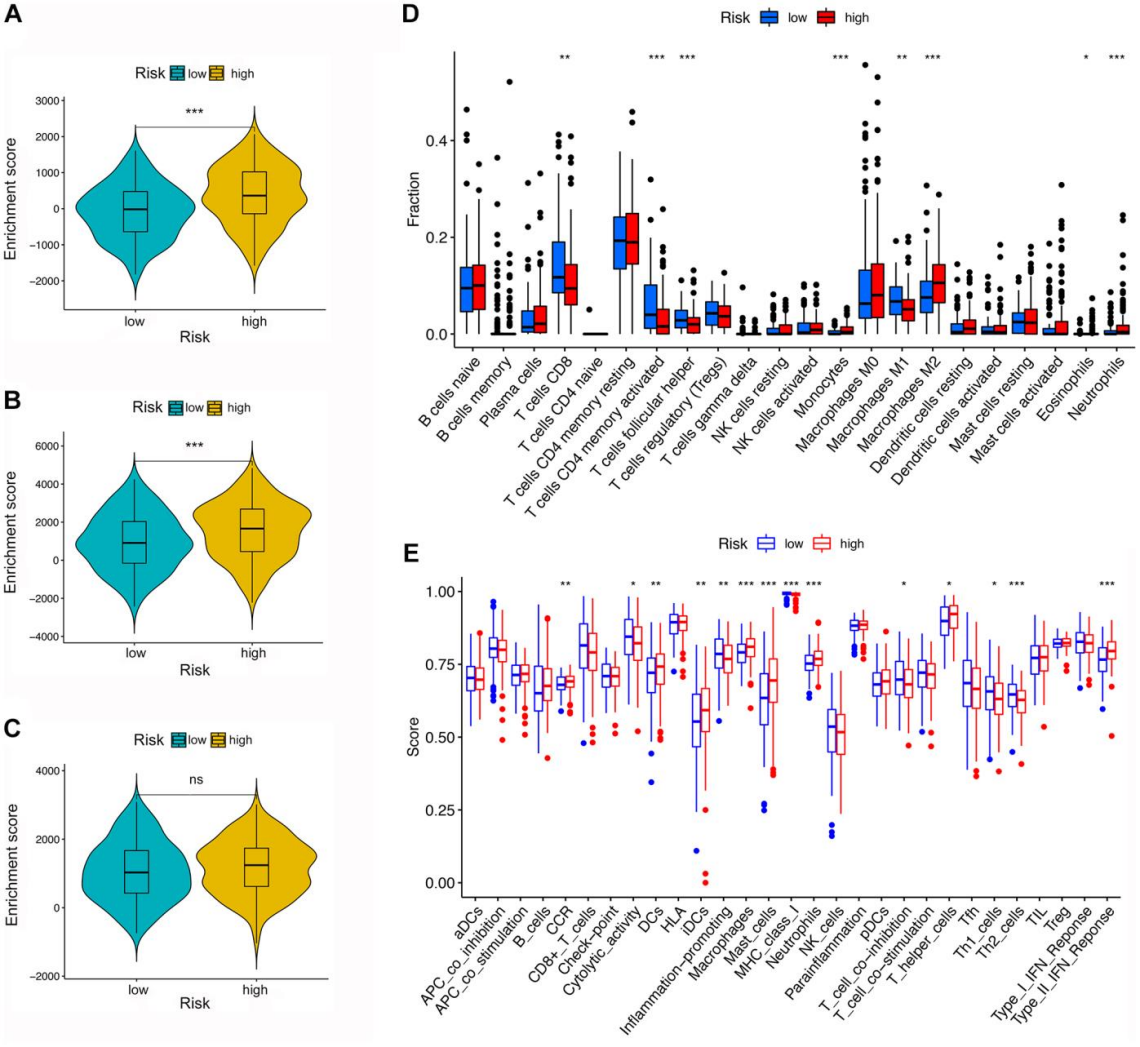
**TME and immune cell infiltration in different risk groups.** (**A**–**C**) TME analysis based on ESTIMATE algorithm. From top to bottom: The stromal score, ESTIMATE score, and immune score. (**D**) Immune cell infiltration in high- and low-risk groups based on the CIBERSORT algorithm. (**E**) Immune-related pathways based on ssGSEA.

First, the difference between the TME and immune cell infiltration was analyzed for LRG and HRG. As shown in Figure 6A–6C, the stromal score and ESTIMATE score were both higher in HRG than LRG, whereas the immune score was not significantly different, indicating that the HRG had a higher proportion of stromal cells than LRG while tumor purity was lower.

**Figure 7:**
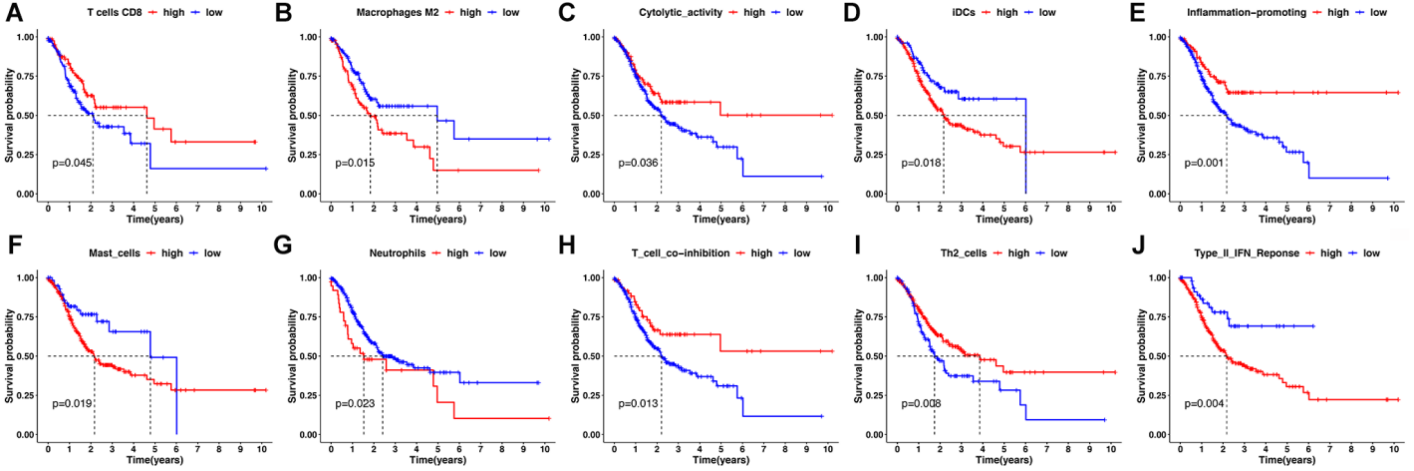
**Kaplan-Meier survival analysis for immune infiltration cell and immune-related pathway.** (**A**, **B**) Kaplan-Meier survival curves for immune infiltrating CD8+ T cells and M2 macrophages. (**C**–**J**) Kaplan-Meier survival curves for immune-related pathway.

Second, by using the CIBERSORT, the abundance of immune cells in each GC sample was estimated and can be seen intuitively in the histogram (Supplementary Figure 3). The number of samples with significant levels of immune cell infiltration in the HRG was greater than in the LRG. Score difference analysis for immune cells’ abundance in different risk groups showed that compared with the HRG, the LRG had higher abundances of CD8+ T cells (*P* < 0.01^**^), CD4+ activated memory T cells (*P* < 0.001^***^), follicular T helper cells (*P* < 0.001^***^), and M1 macrophages (*P* < 0.01^**^). On the other hand, there was a lower abundance of monocytes (*P* < 0.001^***^), M2 macrophages (*P* < 0.001^***^), eosinophils (*P* < 0.05^*^), and neutrophils (*P* < 0.001^***^) (Figure 6D). Notably, the results of XCELL, TIMER, EPIC, and QUANTISEQ showed good consistency with the CIBERSORT (Supplementary Figure 4). Score differences for immune-related functional pathways suggested that the scores for cytolytic activity (*P* < 0.05^*^), inflammation promoting (*P* < 0.01^**^), MHC class I (*P* < 0.001^***^), T cell co-inhibition (*P* < 0.05^*^), Th1 cells (*P* < 0.05^*^), and Th2 cells (*P* < 0.001^***^) were higher in LRG than in HRG. In contrast, the scores for CCR (*P* < 0.01^**^), DCs (*P* < 0.01^**^), iDCs (*P* < 0.01^**^), macrophages (*P* < 0.001^***^), mast cells (*P* < 0.001^***^), neutrophils (*P* < 0.001^***^), T helper cells (*P* < 0.05^*^), and type II IFN responses (*P* < 0.001^***^) were lower in the LRG than in the HRG (Figure 6E). Moreover, survival analysis based on immune cells’ abundance in all samples implied that high infiltration of CD8+ T cells was connected with a favorable prognosis and that high abundance of M2 macrophage was related to an adverse prognosis (Figure 7A, 7B). This confirms that specific immune infiltration patterns have impact on patient prognosis. Survival analysis for immune-associated pathways suggested that high enrichment scores for cytolytic activity, inflammation promoting, T cell co-inhibition, and Th2 cell presence were associated with a favorable prognosis, whereas IDCs, mast cells, neutrophils, and type II IFN responses were associated with adverse prognosis (Figure 7C–7J).

The expression analysis for markers of macrophages indicated that the marker gene of M0 macrophages like CD68 was highly expressed in the high-risk group (Supplementary Figure 5A), CD86 for M1 macrophages was lowly expressed in the high-risk group (Supplementary Figure 5B), similarly, NOS2 tended to be down-regulated in the high-risk group (Supplementary Figure 5C). Moreover, the markers for M2 macrophages like CD163 and CD206 were highly expressed in the high-risk group (Supplementary Figure 5D, 5E). The aforesaid results were consistent with the analysis of tumor infiltration immune cells for macrophages.

**Figure 8:**
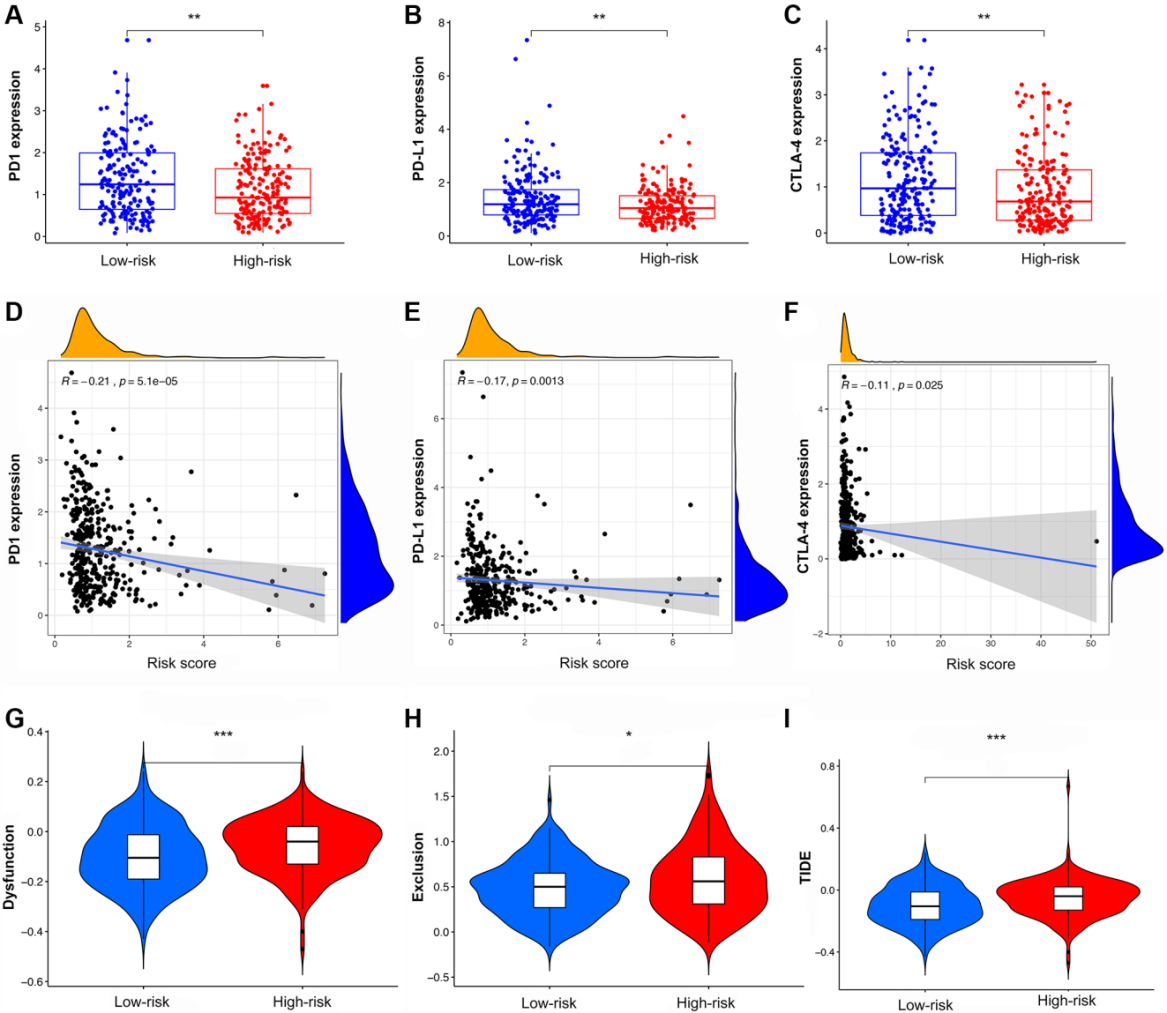
**The analysis of TIDE score and expression of immune checkpoints in high- and low-risk groups.** (**A**–**C**) The expression of PD1, PD-L1, and CTLA4 in different risk groups. (**D**–**F**) The co-expression patterns between immune checkpoints and risk scores. (**G**–**I**) The scores of immune dysfunction, immune exclusion, and TIDE in different risk groups.

Analysis of immune checkpoints showed that PD-1, PD-L1, and CTLA4 were more highly expressed in LRG than in HRG, with decreasing expression as the risk score increased (Figure 8A–8F). The results of cytokine suggested that IFN-γ, IL-21, and OSM had higher expression in LRG than in HRG while IL-1α, IL-1β, IL-10, IL-24, TGF-β, EGF, and VEGF had lower expression in LRG than in HRG (Supplementary Figure 6A–6J). The analysis of chemokine showed that CCL2, CCL7, and CXCL8 were highly expressed in the HRG than in the LRG whereas the CXCL9, CXCL10, and CXCL11 were highly expressed in the LRG than in the HRG (Supplementary Figure 6K–6P).

### Immunotherapy response and chemotherapeutic drug sensitivity prediction based on the IRS to improve the GC patients’ survival

**Figure 9:**
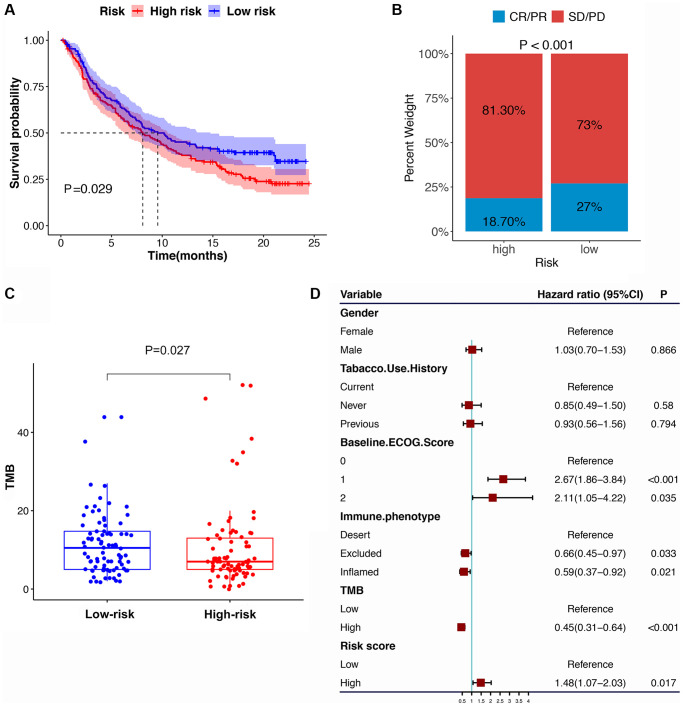
**The IRS in the role of immune checkpoint blocker treatment.** (**A**) Kaplan-Meier survival curve of the high- versus low-risk group in the immunotherapy cohort (IMvigor210 cohort). (**B**) The proportion of immune response to immunotherapy in high versus low-risk group. (**C**) The tumor mutation burden in the immunotherapy cohort was compared among distinct risk groups. (**D**) Multivariate Cox regression analysis of the IRS with features in the immunotherapy cohort.

Compared with the HRG, the LRG presented with lower TIDE scores, indicating that the latter may have a greater response to ICI than the former (Figure 8G–8I). In the immunotherapy cohort, patients in the LRG had a markedly longer survival time (Figure 9A). Compared with HRG, the LRG had a better therapeutic advantage and immunotherapy response (Figure 9B). Moreover, the TMB was significantly elevated in LRG, which is closely linked to immunotherapeutic efficacy (Figure 9C). Also, the association between the IRS and survival on immunotherapy remained statistically significant after taking into account gender, smoking, ECOG score, immune phenotype, and TMB status (Figure 9D).

**Figure 10:**
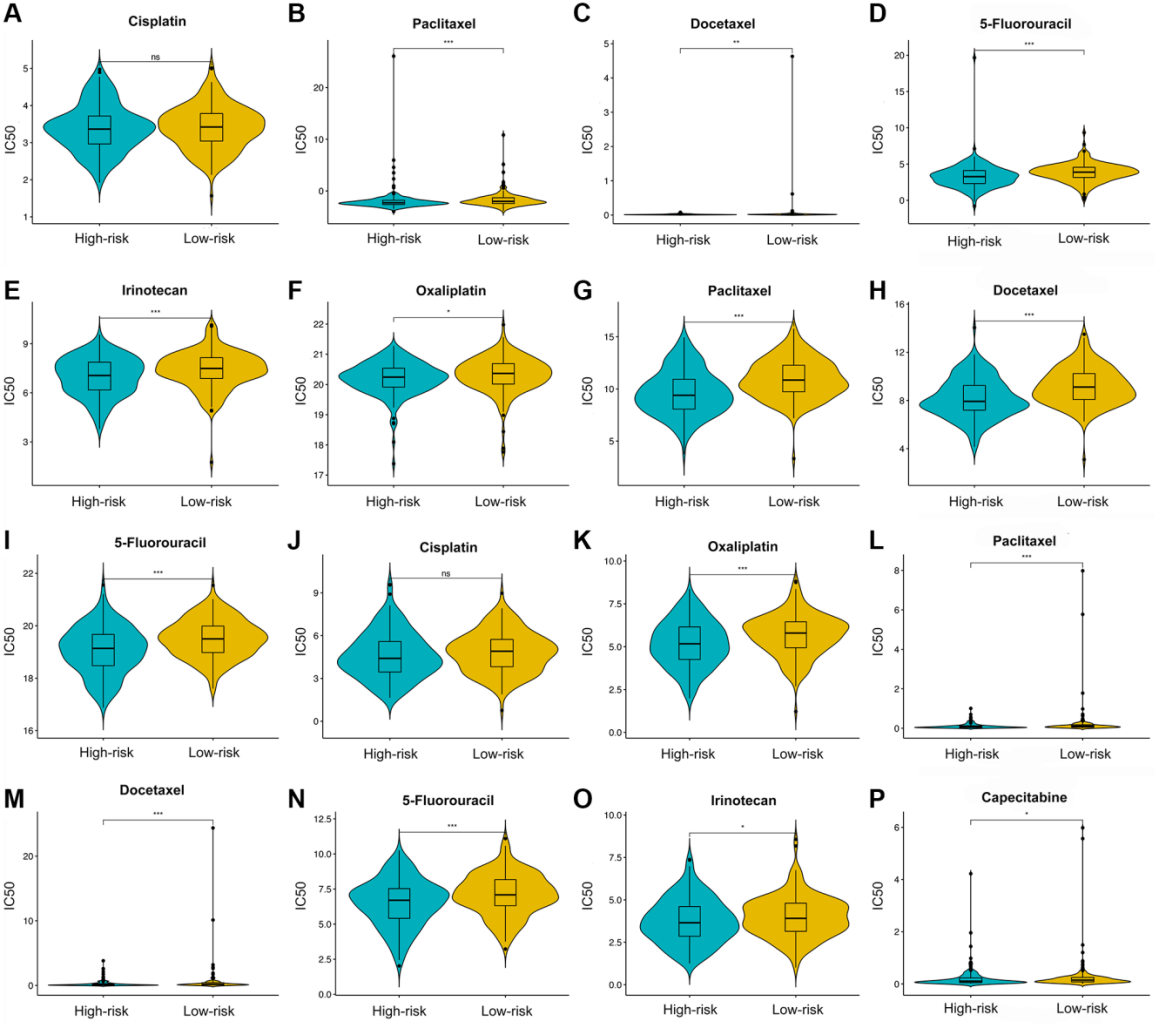
**Chemotherapy drugs’ sensitivity of the high- versus low-risk group.** Differential analysis of IC50 for chemotherapy drugs in CGP (**A**–**E**), CTRP (**F**–**I**), and GDSC (**J**–**P**) databases.

Three drug response-related databases (CGP, GDSC, and CTRP) were utilized to investigate the association between the chemotherapeutic drug sensitivity and the IRS. Results suggested that patients in the LRG are generally more sensitive to chemotherapeutic drugs than those in the HRG (Figure 10).

### Expression of the genes in the immune-related signature

The quantitative analysis of the immunohistochemical images determined that *CD36, DUSP1*, and *PTGER3* showed lower protein expression levels in GC samples than in the adjacent normal tissues while *CGB5* showed high levels in GC samples than in the normal tissues. The expression of *APOA1*, *INHBE*, and *RNASE2* did not differ significantly in gastric cancer and normal samples, and the *CTLA4* was not reported in the database (Supplementary Figures 7, 8).

### Validation of the immune-related signature by qRT-PCR

**Figure 11:**
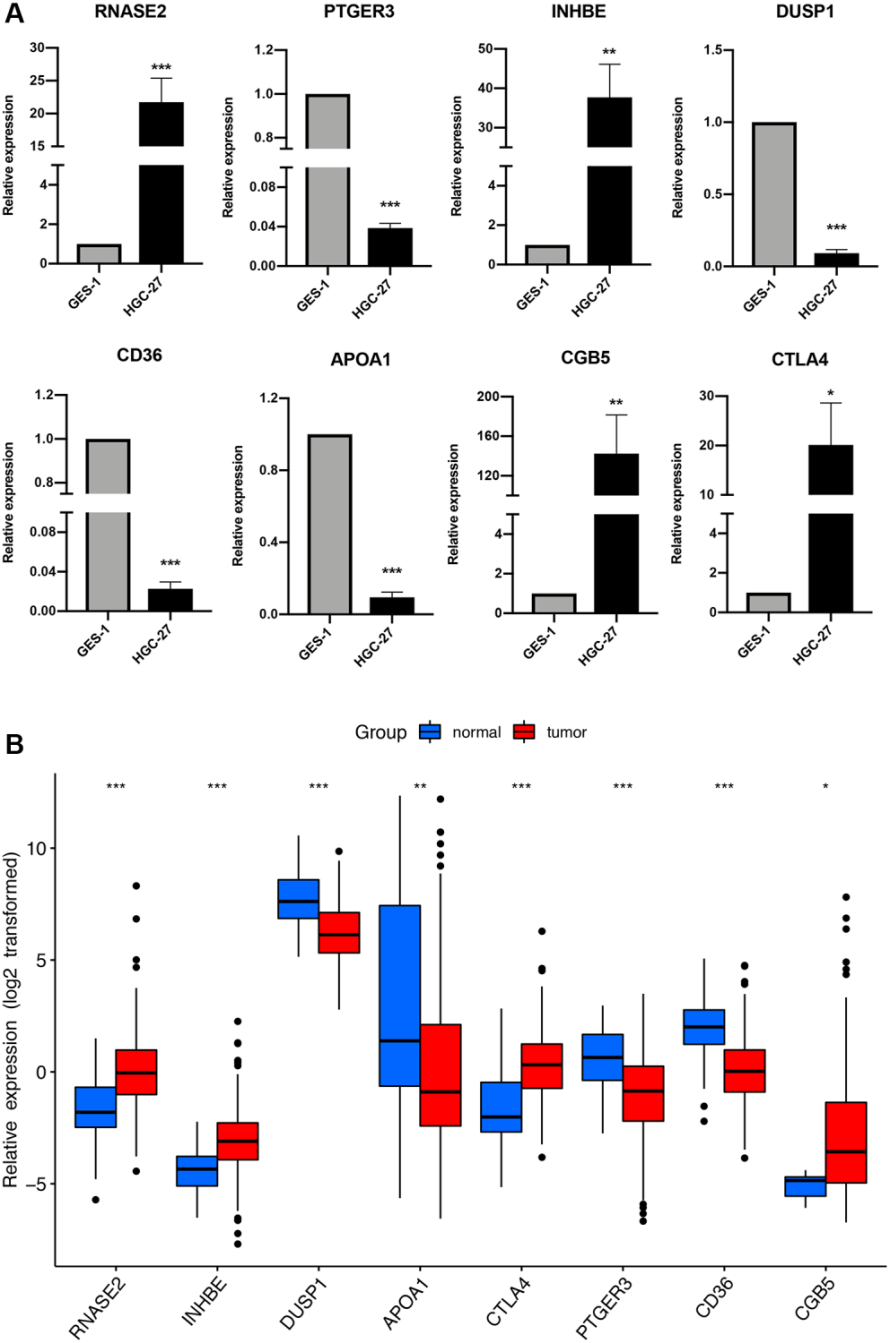
**The expression of the immune-related signature in cell lines and TCGA cohort.** (**A**) qRT-PCR results of the immune-related signature in GC cell lines (HGC-27) and control cell lines (GES-1). ^*^P < 0.05, ^**^P < 0.01, ^***^P < 0.001. (**B**) The expression of the immune-related signature in TCGA cohort.

The expression profiles of the eight genes comprising the prognostic signature were verified in GC and stomach cell lines by qRT-PCR. The result suggested that *RNASE2, INHBE, CGB5*, and *CTLA4* were upregulated in GC cell lines, while *PTGER2, DUSP1, CD36,* and *APOA1* were downregulated (Figure 11A), which showed good consistency with the expression analysis results in TCGA cohort (Figure 11B).

## DISCUSSION

In the current study, we identified the IRGs that were significantly correlated with the prognosis of GC patients. We then constructed an IRS to predict the prognosis of patients assigned to the different risk groups. This signature was combined with clinical characteristics to build a composite nomogram, which exhibited an accurate prediction capacity for GC patients’ prognosis. Finally, we investigated the relationship between the IRS and the somatic mutation, pathway activation, immune cell infiltration, immunotherapy responsiveness, and chemotherapeutic drug sensitivity.

GC is a highly heterogeneous malignant tumor with a poor prognosis. The traditional TNM classification is a common strategy on which to base clinical management but inevitably has limitations. With the development of genomics and precision medicine, molecular signatures based on gene expression levels have been developed to predict clinical outcomes. Several approaches have been applied for distinguishing the risk-stratified subgroups of GC and helping to formulate individual treatment strategies. Nevertheless, probably due to the high heterogeneity of GC, genes comprising published signatures did not overlap, and more novel signatures are still needed. Immune features have been reported to significantly affect the treatment responses and survival of GC patients; thus, several molecular signatures consisting of immune-related genes have been adopted for GC prognosis, and these signatures are usually used to stratify risk groups in patients. However, whether these signatures predict clinical therapeutic responses is still unknown. Moreover, ICIs targeting PD-1, PD-L1, and CTLA4 have been widely utilized and found to significantly prolong survival time in GC patients. Navulizumab in combination with fluorouracil-based and oxaliplatin-based chemotherapy can be a first-line treatment option for patients with advanced HER-2 negative gastric cancer with PD-L1 CPS ≥ 5. The study CheckMmate-649 showed that Navulizumab combined with chemotherapy can significantly improve OS and PFS of GC patients. Pabrolizumab can be used as second-line or follow-up treatment for patients with MSI-H/ dMMR or advanced gastric cancer with high tumor load. The clinical trial revealed that the ORR for these patients was 39.6%, with a 9.9% complete response rate. Besides, dostarlimab-gxly may be used to treat patients with MSI-H/dMMR gastric tumors that have progressed on or after prior treatment, who have no satisfactory alternative treatment options, and who had not previously received a PD-1 or PD-L1 inhibitor. The GARNET trial demonstrated that the ORR was 42%, with a 9% complete response rate. It can be seen that the response rate of GC patients to ICIs treatment is relatively low. There are only a limited group of patients are benefited from the ICIs treatment, thus it remains a challenge to discriminate patients likely to respond well to ICI. Given that, biomarkers predicting patient subpopulations appropriate for ICI treatment warrant intensive investigation.

Previously immune-related gene signatures were mostly established based on the differentially expressed gene sets. Here, we applied the WGCNA analysis to further filter candidate gene markers, which increases the reliability of the final result. After conducting the Cox regression analysis, a final prognostic signature consisting of 8 genes (*RNASE2*, *CGB5*, *INHBE*, *PTGER3*, *CTLA4*, *DUSP1*, *APOA1*, and *CD36*) was constructed. Of these genes, some had previously been reported to play vital roles in cancer. *CD36*, a multi-ligand scavenger receptor expressed on the surface of platelets, adipocytes, hepatocytes, and epithelial cells, was reported to be associated with adverse prognosis and treatment resistance of patients with GC and other solid tumors [23–27]. Additionally, evidence indicated that *CD36* can promote GC cell migration and invasion by inducing c-Myc-dependent DEK transcription, GSK-3β/β-catenin pathway activation, and EMT, suggesting that *CD36* may serve as a novel target in GC [23]. *CTLA4* is one of the most studied immune checkpoints in malignancies, blockade of which has yielded considerable clinical benefits for patients with malignant tumors [28]. Studies showed that *CTLA4* mRNA levels are upregulated in tumor tissues and correlate with a favorable prognosis [29]. *DUSP1* participates in several cellular processes including proliferation, differentiation, and apoptosis. Interestingly, strong expression of *DUSP1* is a favorable prognostic factor in glioma and hepatocellular carcinoma [30, 31]. However, it was also reported to be upregulated in several solid tumors where it facilitated carcinogenesis [32–34], suggesting that the role of *DUSP1* in carcinogenesis could be controversial in different tumors. *CGB5* was reported to be associated with poor prognosis in GC in several bioinformatics studies [35, 36]. Another study suggested that it may promote tumor growth and vascular formation in ovarian cancer via activation of the LHR signal transduction pathway [37]. Previous studies have reported that *PTGER3* is mainly involved in the carcinogenesis of gynecological malignancies [38]. It was found to act as an independent prognostic factor and was associated with poor overall survival in cervical and ovarian cancers [39, 40]. *PTGER3* promotes the tumor cell migration by regulating uPAR expression to affect cervical cancer progression [41]. *APOA1*, a major protein moiety in high-density lipoprotein (HDL) particles, may suppress colorectal tumor progression via regulating the metabolism of intracellular cholesterol [42]. It was found in high amounts in urine from patients with bladder cancer, and utilizing exfoliative urinary cytology in combination with *APOA1* detection increased the sensitivity of diagnosis [43, 44]. Also, studies suggested that *APOA1* might act as an innovative marker in predicting recurrence, development, prognosis, and chemotherapy resistance of some solid tumors [45–47]. There is less data on *INHBE* and *RNASE2* in malignancies, except for some bioinformatics studies reporting their predictive value in cancer prognosis, indicating that more in-depth studies on their biological functions are necessary in the future.

We developed an immune-clinical nomogram consisting of IRS and clinical characteristics (pathological stage and age), which performed well for predicting the prognosis of patients with GC. The DCA curves suggested that combining with the nomogram could yield the most clinical benefit for patients with GC.

The Go enrichment analysis revealed that the activity of cytokines and chemokines, migration of various inflammatory cells, and immune inflammatory responses were significantly enriched in gastric cancer. Previous study revealed that inflammation is a critical component of tumor progression. The inflammatory cells could orchestrate the tumor microenvironment and participant in the neoplastic process, fostering proliferation, survival and migration [48]. Furthermore, the KEGG analysis showed that the DE-IRGs were enriched in multiple cytokine-related signaling pathways such as cytokine-cytokine receptor interaction, chemokine signaling pathway, viral protein interaction with cytokine and cytokine receptor. Previous studies have shown that cytokines can promote or inhibit tumor growth and invasiveness through multiple pathways, thereby affecting gastric cancer progression [49, 50]. Besides, the inflammation-related pathway such as NF-κB also had significant enrichment. Study demonstrated that the activation of NF-κB signaling has been identified as regulating several sporadic and inflammation-associated gastrointestinal tract malignancies [51]. Summarily, the above results suggest that cytokine activation and immune inflammation have an important role in the development of gastric cancer.

The results suggested that the TMB was generally higher in the LRG. The higher the TMB, the more DNA mutations exist and more candidate peptides generated, leading to a greater likelihood of neoantigens being identified by the immune system [52]. Accumulating studies indicate that the TMB is a potential biomarker for immunotherapy response and prognosis in solid tumors [52, 53]. Interestingly, a high TMB did suggested a favorable prognosis for GC patients according to our results. In keeping with the TMB level in distinct risk subgroups, the LRG presented with a greater genetic mutation frequency, suggesting higher levels of tumor heterogeneity. In particular, it was observed that the LRG had a significantly higher frequency of *TTN* mutation. Some studies reported that *TTN* mutation was associated with increased TMB and related to high immunogenicity. Consequently, the patients with mutated *TTN* exhibited a favorable objective response to ICI treatment and longer progression-free survival or overall survival than those with wild-type status [54, 55].

In addition to tumor cells, nontumor cells such as stromal cells and immune cells are present in the GC TME, which decrease tumor purity. The present study revealed a higher proportion of stromal cells as well as lower tumor purity in the HRG. It has been reported that TME-related stromal cells can positively regulate tumor growth and impair host immune responses, and that low tumor purity is associated with an unfavorable prognosis and an immune-evasion phenotype. This suggests that stromal changes in the development of GC might be deleterious [56, 57]. As a critical component of the TME, the distribution of immune cells also varies across risk groups. As the main effector cells, CD8+ T cells play an important role in host defense against cancer. The current study showed that tumors from the LRG had a higher CD8+ T cells content, and that more infiltration of CD8+ T cells correlated with a better prognosis for patients with GC. This is consistent with a high infiltration of CD8+ T cells enhancing the host’s antitumor defense, thereby improving the survival outcomes of GC patients. Activated CD4+ memory T cells and follicular T-helper cells were also copious in the LRG. These cells are likely to bolster the maintenance of a protective immune response against tumor-related antigens, associated with a more favorable prognosis for GC patients in the LRG. Macrophages are an essential component of innate immunity, playing an important role in cancer development and metastasis. Proinflammatory M1 macrophages can phagocytose tumor cells, while anti-inflammatory M2 macrophages promote tumor growth and invasion [58]. This may explain why M1 macrophages were enriched in the LRG while M2 macrophages were clustered in the HRG. Accumulating studies are showing that eosinophils, neutrophils, and monocytes all have disparate effects on cancer progression, encompassing both pro- and anti-tumorigenic roles [59–61]. In the present study, these three immune cell types were more bountiful in the LRG, indicating that they might contribute to the tumor invasion and angiogenesis thereby playing a role in promoting cancer.

Moreover, CCRs can influence the proliferation, invasion, and metastasis of cancer cells and have potential for future immunotherapeutic exploitation [62]. DCs can aid cancer growth and development by facilitating immune tolerance [63]. Mast cells are increased in GC and have been correlated with angiogenesis, and lymph metastasis [64]. The aforesaid immune-related pathways were centered in the HRG, associated with the poor prognosis of GC. Inflammation assists in the proliferation and survival of malignant cells, and stimulates angiogenesis and metastasis, thereby driving tumor initiation, growth, and progression [65]. Inflammation-promoting terms were enriched in the LRG, which might weaken the protective effect. One study pointed out that the primary response to anti-CTLA-4 requires MHC class I expression [66]. In the current study, MHC class I was enriched in the LRG, reflecting a better immunotherapeutic response for the LRG.

Furthermore, we explored the association between IRS and therapeutic responses. ICI treatment has been widely applied for GC, but so far the response rate is relatively low (10–26%) [67]. The present study suggested that TIDE score was lower but the TMB and expression of PD-L1 were higher in LRG. Also, the IRS effectively distinguish patients in the IMvigor210 cohort who benefit from immunotherapy. The aforementioned results implied that patients with GC in LRG might benefit from immunotherapy, so the IRS may have great potential for predicting immunotherapy responsiveness.

Currently, patients with GC still need to receive systematic chemotherapy [68]. Our results suggest that LRG were more susceptive to conventional chemotherapy drugs such as cisplatin, oxaliplatin, docetaxel, paclitaxel, 5-FU, capecitabine, and irinotecan. On the one hand, docetaxel, paclitaxel, irinotecan, and 5-FU are cell cycle-specific drugs. Paclitaxel or docetaxel can stabilize microtubules and impede the mitosis of cancer cells, thereby effectively preventing their proliferation and mediating anti-cancer effects. Irinotecan inhibits topoisomerase 1 and induces DNA single-strand damage, thus blocking DNA replication and affect the cell cycle. 5-FU is an antimetabolite chemotherapeutic drug that inhibits tumor cell proliferation by affecting nucleic acid synthesis. On the other hand, cisplatin and oxaliplatin, cell-cycle-nonspecific drugs, are not affected by the cell cycle phase and kill rapidly dividing cancer cells via disrupting DNA structure. As mentioned above, the LRG shows a considerable enrichment of the proliferation- and metabolism-related pathways such as cell cycle, DNA replication, and pyrimidine metabolism, which might account for their higher sensitivity to chemotherapeutic drugs.

In brief, we established an IRS which had good predictive performance for prognosis of patients with GC. Patients in the HRG had poor prognosis, more enrichment of oncogenic pathways, low TMB and mutation frequency, more basic immune cells, low expression of immune checkpoints, poor response to immunotherapy, and low sensitivity to common chemotherapeutic drugs. Reciprocally, patients in the LRG had a more favorable prognosis, more enrichment of proliferation pathways, high TMB and mutation frequency, more CD8+ T cells, high expression of immune checkpoints, positive responses to immunotherapy, and high sensitivity to the chemotherapeutic agents. A nomogram was constructed which appeared to possess great capability for predicting GC patients’ survival, the application of which might yield more clinical benefit.

The concept of the IRS has been reported before. The strength of the present study is that we developed a robust IRS using TCGA database and further validated it on an external dataset to ensure the confirmability of the analysis results. Simultaneously, we validated the expression differences of prognostic signatures using PCR, and the results were in good agreement with the bioinformatic analysis, indicating that the results of this study have good verifiability. Furthermore, we investigated the TMB, TME, and immune cell infiltration, and analyzed the response to ICIs as well as sensitivity to chemotherapeutic drugs, which offers a perspective for understanding the specific immune characteristics underlying the IRS and may be vital for GC patients. Regardless of the strengths, our study inevitably has several limitations as well. First, data on clinical characteristics such as Her-2 expression, microsatellite instability, chemotherapy and immunotherapy are insufficient in the TCGA database, yet this information might be necessary for perfecting the nomogram. Second, further functional experiments on the IRS are required to validate our silico results. Finally, clinical responses to ICIs and sensitivity to chemotherapeutic drugs should be further verified in clinical cohorts. In summary, the subsequent validation in a large clinical cohort and the use of experiments to validate the molecular function of the prognostic signature will also be the focus of our future research.

In conclusion, this study underlines the importance of IRGs in GC prognosis and establishes an immune-related prognostic signature, which is expected to improve the prediction of GC patient survival together with well-defined TNM staging. Additionally, the clinical outcome, genetic mutations, immune cell infiltration, immunotherapy response, and chemotherapeutic drug sensitivity underlying the signature were also identified. These results lay the foundation for comprehending the role of IRGs and illustrate the underlying clinical implications of IRGs in GC.

## Supplementary Materials

Supplementary Figures

Supplementary Tables 1-2, 5 and 12

Supplementary Table 3

Supplementary Table 4

Supplementary Table 6

Supplementary Table 7

Supplementary Table 8

Supplementary Table 9

Supplementary Table 10

Supplementary Table 11
