# Supplementary material for: An immune-related prognostic signature associated with immune landscape and therapeutic responses in gastric cancer
Source: Aging (Albany NY). 2023 Feb 22;15(4):1074–106. doi: 10.18632/aging.204534 (PMC10008502; doi:10.18632/aging.204534)
Supplement: Supplementary Figures [file aging-15-204534-s001.pdf]

SUPPLEMENTARY FIGURES

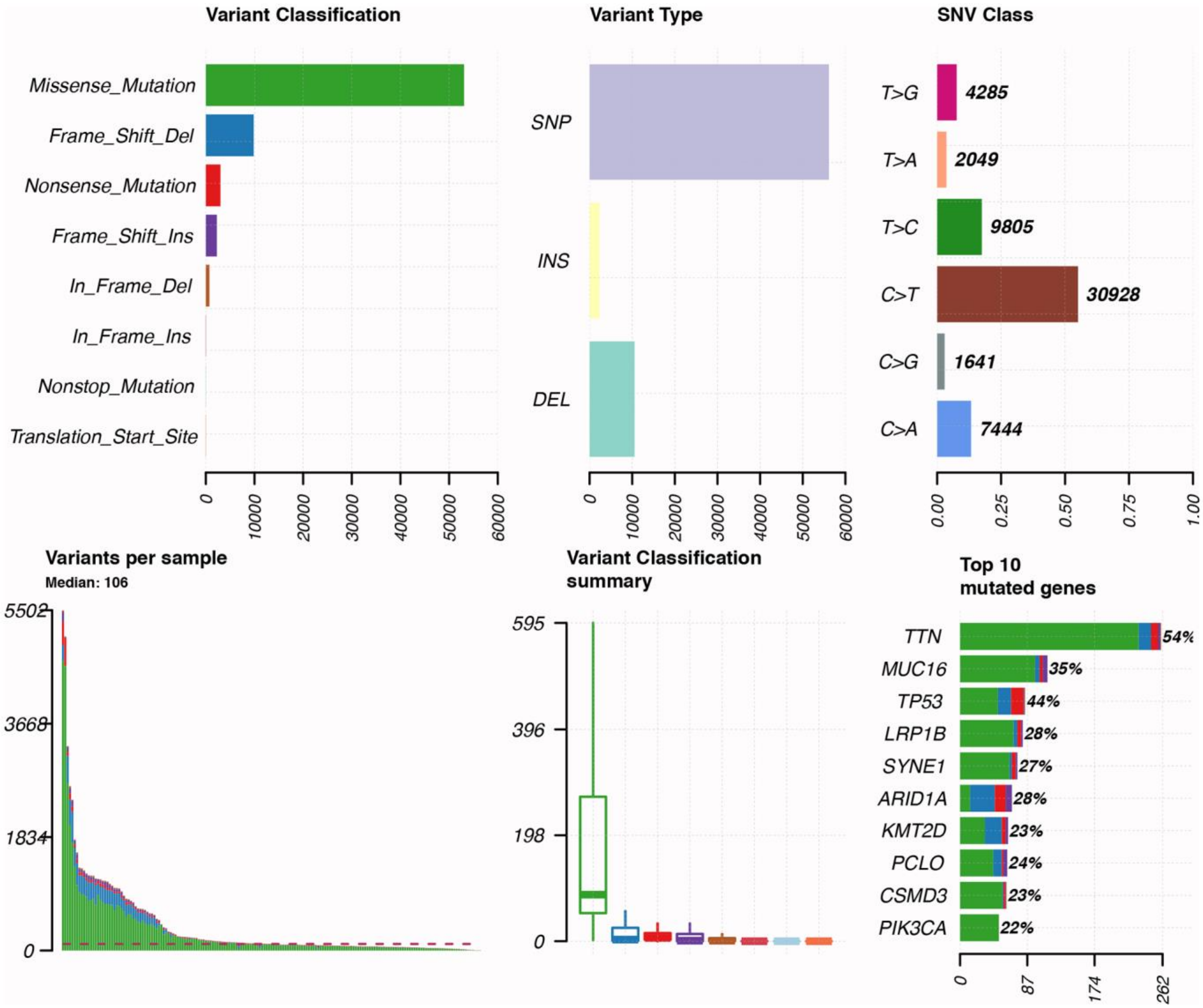

Supplementary Figure 1. Summaries of gene mutation profiles for low-risk group.

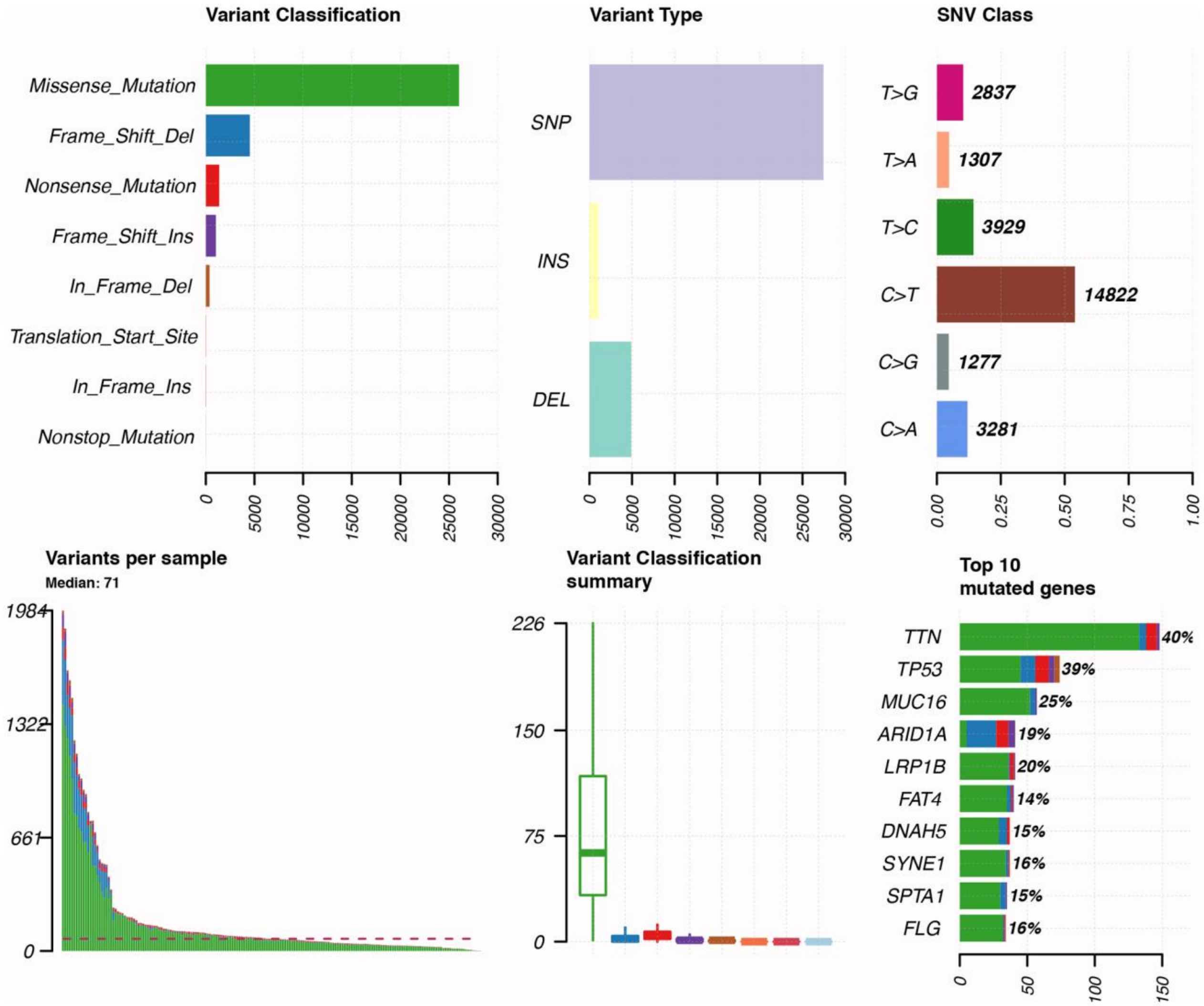

Supplementary Figure 2. Summaries of gene mutation profiles for high-risk group.

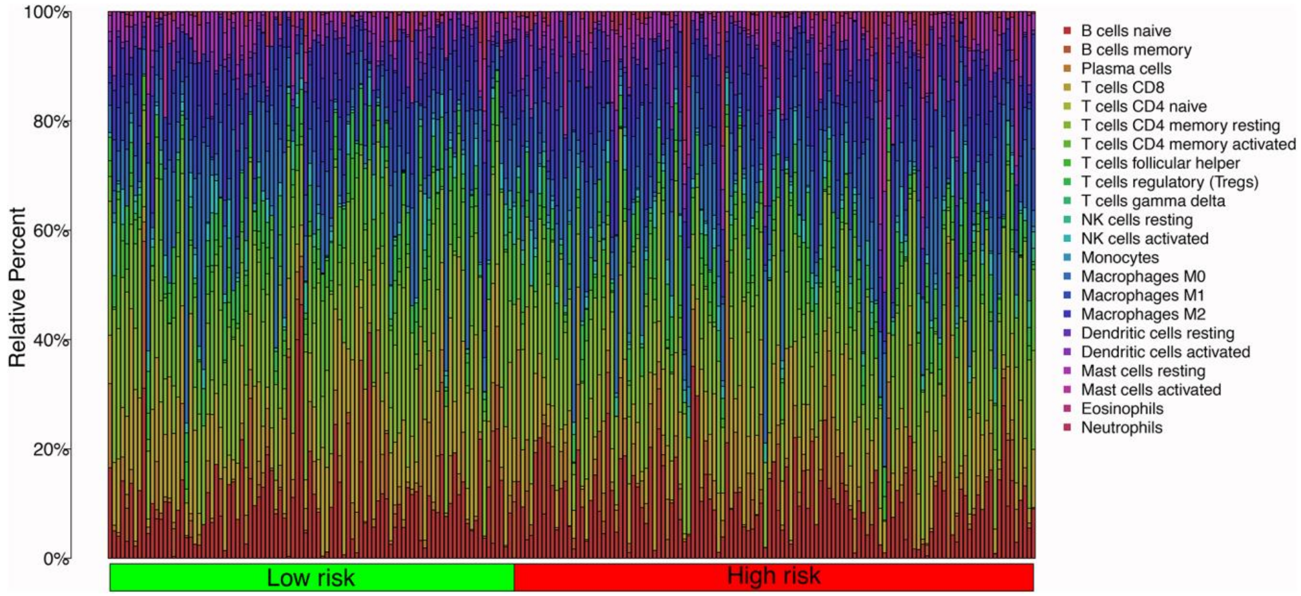

Supplementary Figure 3. The histogram of immune cells' abundance in GC samples.

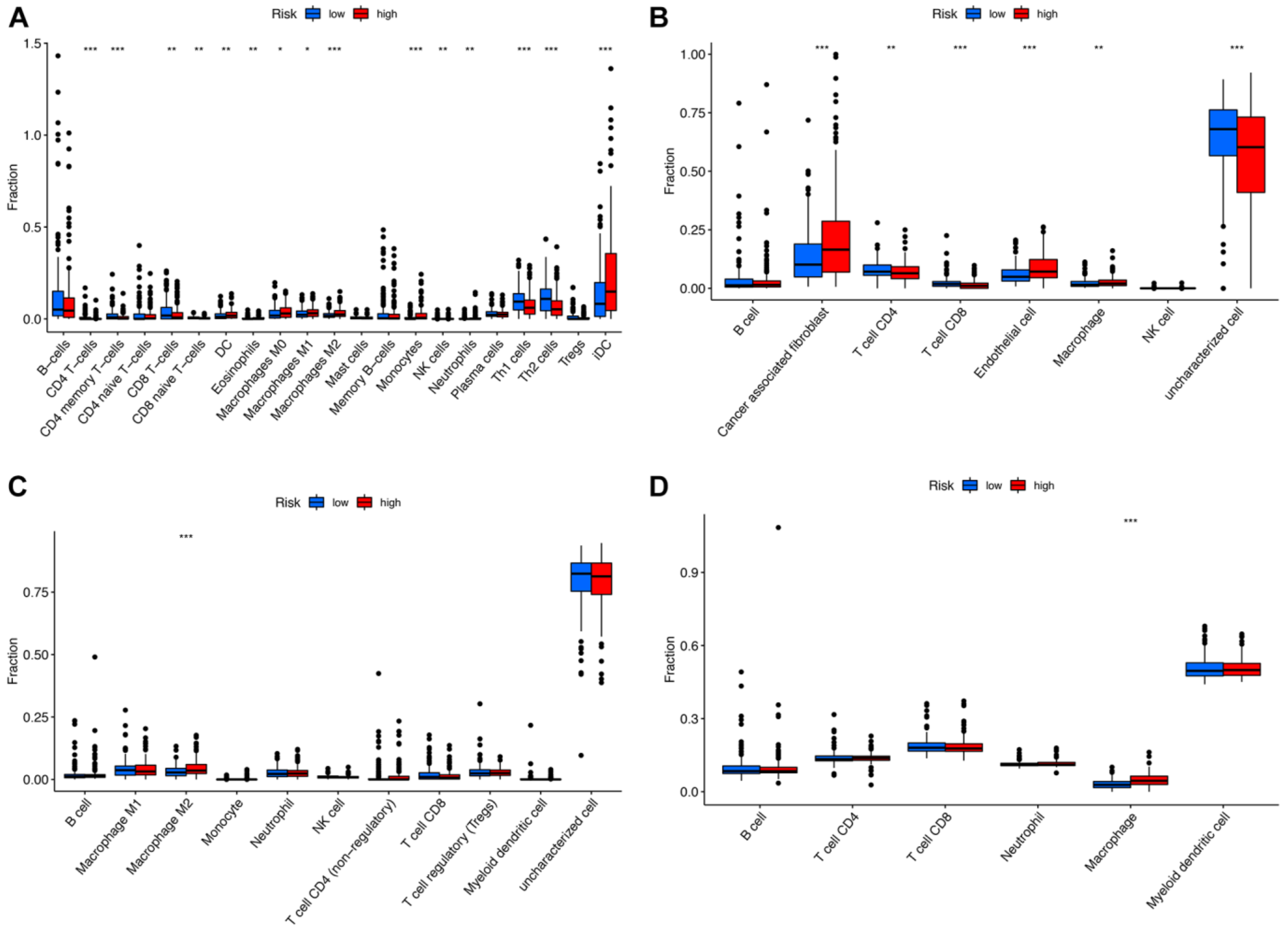

Supplementary Figure 4. The immune cell infiltration in different risk groups analyzed by XCELL (**A**), EPIC (**B**), QUANTISEQ (**C**), and TIMER (**D**).

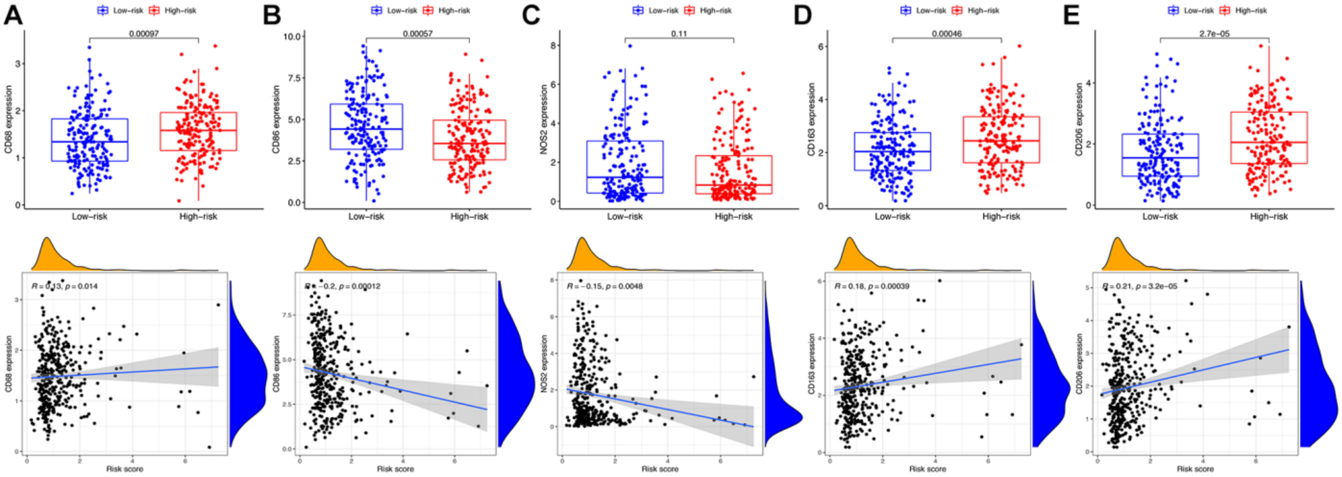

Supplementary Figure 5. The expression of markers for M0 macrophages (**A**), M1 macrophages (**B**, **C**), M2 macrophages (**D**, **E**).

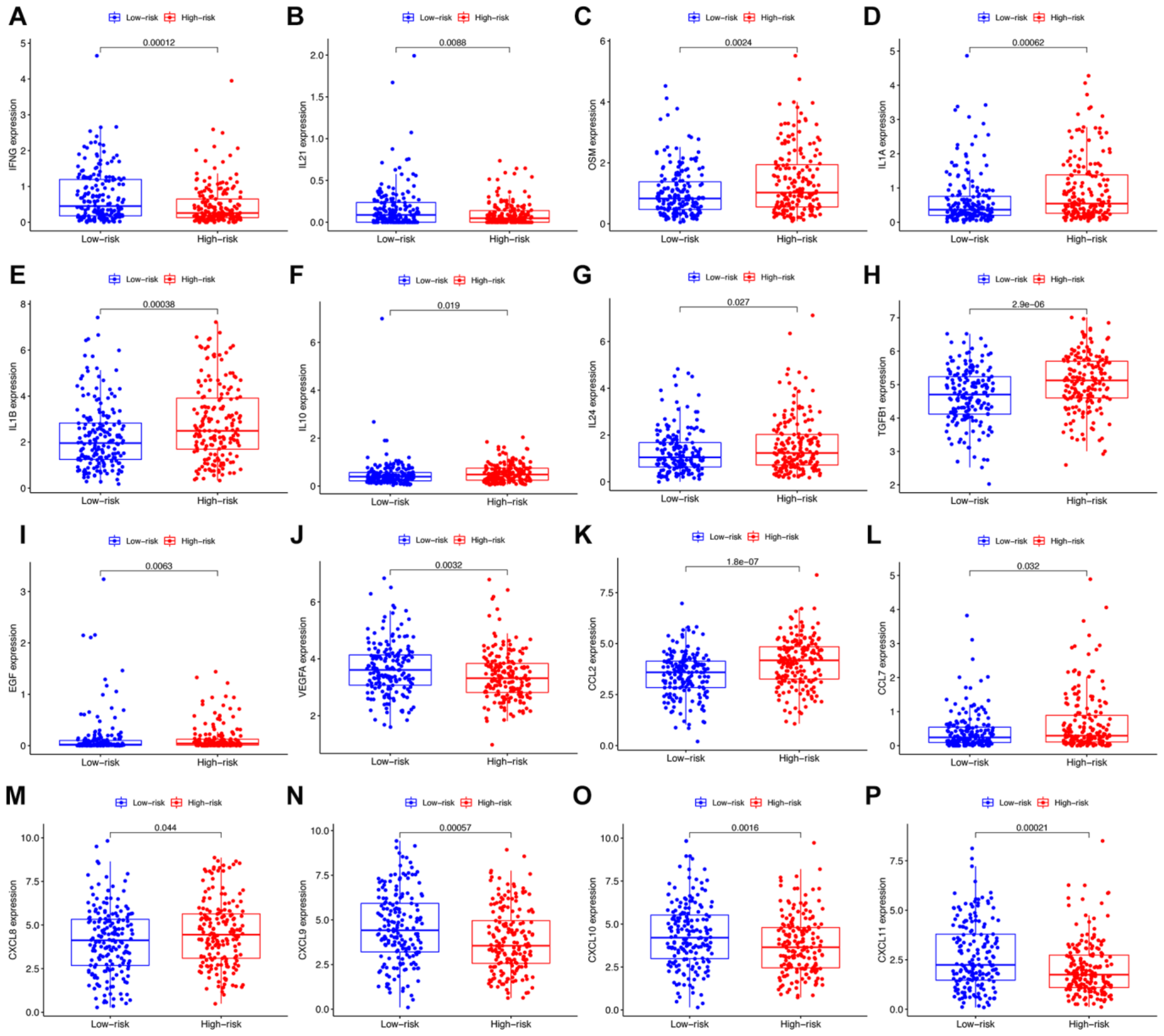

Supplementary Figure 6. The expression of cytokine (**A**–**J**) and chemokine (**K**–**P**).

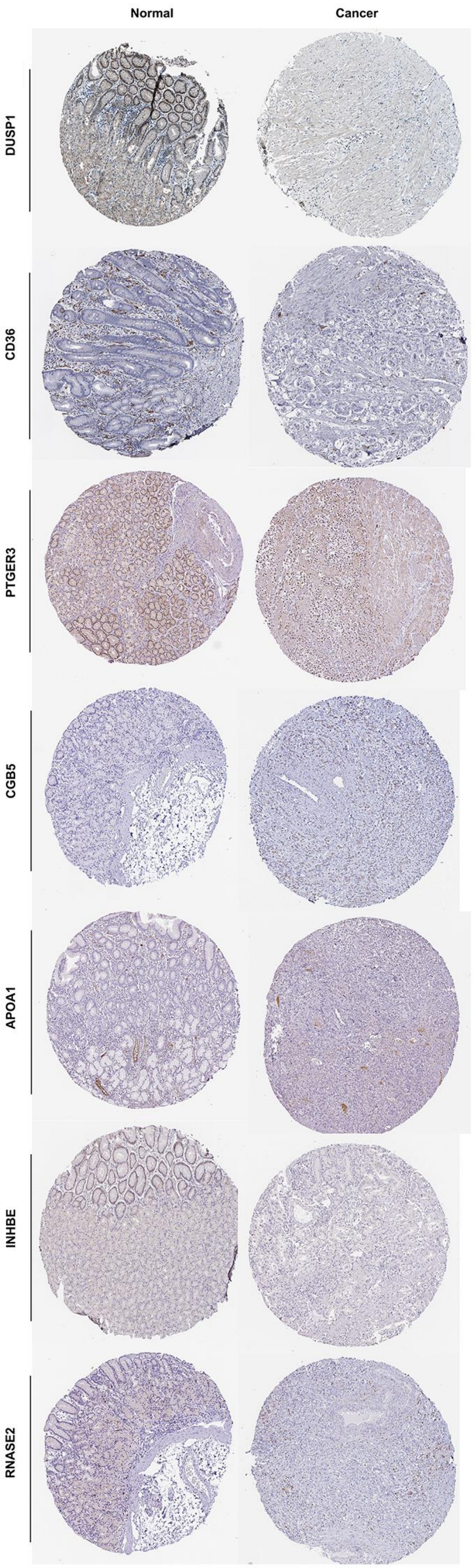

Supplementary Figure 7. The immunohistochemical analysis of the protein expression of the genes in the IRS in the HPA database.

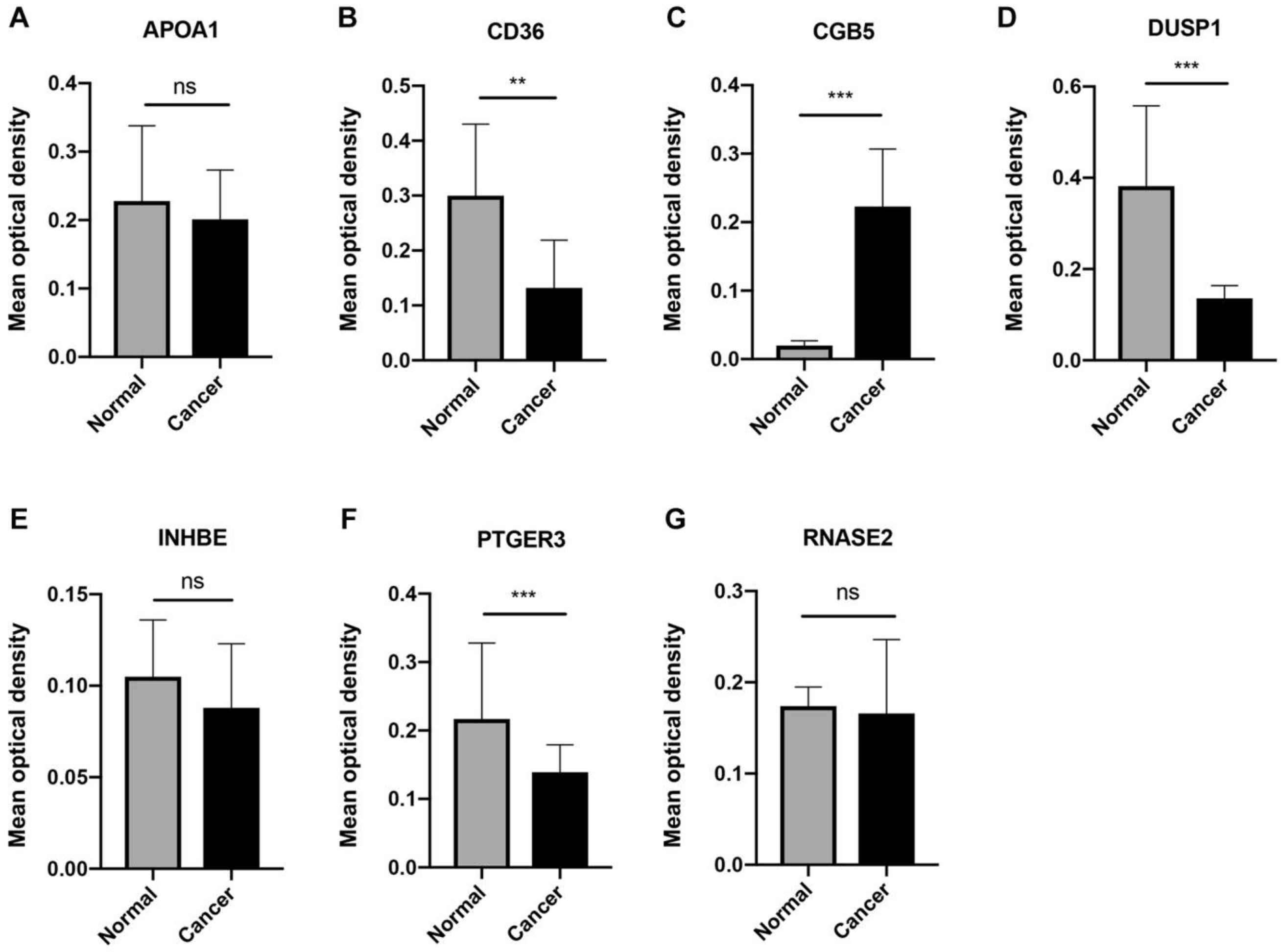

Supplementary Figure 8. The quantitative analysis of immunohistochemical images. The quantitative analysis plots of APOA1 (**A**), CD36 (**B**), CGB5 (**C**), DUSP1 (**D**), INHBE (**E**), PTGER3 (**F**), RNASE2 (**G**). Data are presented as mean ± SD, $^{**}P < 0.01$, $^{***}P < 0.001$, and 'ns' represents no statistical significance.
