# Supplementary material for: An immune-related prognostic signature associated with immune landscape and therapeutic responses in gastric cancer
Source: Aging (Albany NY). 2023 Feb 22;15(4):1074–106. doi: 10.18632/aging.204534 (PMC10008502; doi:10.18632/aging.204534)
Supplement: Supplementary Tables 1-2, 5 and 12 [file aging-15-204534-s002.pdf]

SUPPLEMENTARY TABLES

Supplementary Table 1. The baseline features of GC patients in TCGA and GEO datasets.

Features	TCGA (*n* = 371)	GSE84437 (*n* = 433)
Age, years (mean ± SD)	65.54 ± 10.55	60.06 ± 11.58
Gender		
Male	230	296
Female	141	137
Tumor grade		
G1	25	–
G2	129	–
G3	209	–
G4	8	–
Pathologic stage		
I–II	173	157
III–IV	198	276
Tumor size		
T1	16	11
T2	70	38
T3	161	92
T4	124	292
Lymph node metastasis		
N0	109	80
N1	115	188
N2	72	132
N3	75	33
Metastasis status		
M0	319	347
M1	52	86

The “–” indicates that the value is not available. Abbreviations: HR: hazard ratio; CI: confidence interval.

Supplementary Table 2. The baseline features of patients in IMvigor210 cohort.

Features	*n* = 348
OS, months (mean ± SD)	10.25 ± 7.65
Survival status	
Alive	232
Dead	116
Gender	
Female	76
Male	272
Tobacco. Use. History	209
Current	35
Never	116
Previous	197
Baseline. ECOG. Score	198
0	134
1	196
2	18
Immune. Phenotype	161
Desert	76
Exclude	134
Inflamed	74
Unknown	64
Binary Response	72
CR/PR	68
SD/PD	230
Unknown	50
Mutation burden per MB (mean ± SD)	10.86 ± 9.57

Abbreviations: OS: overall survival; ECOG: eastern cooperative oncology group; CR: complete remission; PR: partial remission; SD: stable disease; PD: progressive disease.

Supplementary Table 5. The primers sequences for mRNAs of the immune-related signature.

Gene name	Forward sequence (5′–3′)	Reverse sequence (5′–3′)
GAPDH	TGCACCACCAACTGCTTAGC	ATCGAGTGAAGGACCTGGC
DUSP1	GTACATCAAGTCCATCTGAC	GGTTCTTCTAGGAGTAGACA
APOA1	AGCTTGCTGAAGGTGGAGGT	ATCGAGTGAAGGACCTGGC
CTLA4	CACAAGGCTCAGCTGAACCT	AGGTGCCCGTGCAGATGGAA
PTGER3	AAGGCCACGGCATCTCAGT	TGATCCCCATAAGCTGAATGG
RNASE2	TGATCCCCATAAGCTGAATGG	ACCATGTTTCCCAGTCTCCG
CD36	ACGGGCTGAGCAAGGTTGA	TTCGTTGGGTGGGTAGATGG
CGB5	GCTACTGCCCCACCATGACC	ATGGACTCGAAGCGCACATC
INHBE	AGCCCTTCCTAGAGCTTAAG	GCTGCAGCCACAGGCC

Supplementary Table 12. The prognostic signature identified by multivariate Cox regression analysis.

Gene	HR (CI 95%)	*P*-value	Coefficient
RNASE2	1.32 (1.03–1.68)	0.026	0.28
CGB5	1.29 (1.10–1.52)	0.002	0.26
INHBE	1.65 (0.98–2.79)	0.061	0.50
PTGER3	0.71 (0.44–1.13)	0.145	−0.35
CTLA4	0.71 (0.55–0.93)	0.012	–.034
DUSP1	1.30 (1.07–1.57)	0.008	0.26
APOA1	1.07 (0.99–1.17)	0.092	0.07
CD36	1.26 (1.00–1.60)	0.055	0.23
