# Supplementary material for: An immune-related prognostic signature associated with immune landscape and therapeutic responses in gastric cancer
Source: Aging (Albany NY). 2023 Feb 22;15(4):1074–106. doi: 10.18632/aging.204534 (PMC10008502; doi:10.18632/aging.204534)
Supplement: Supplementary Table 3 [file aging-15-204534-s003.pdf]

Supplementary Table 3. Detailed information of the immune-related genes in ImmPort database.

Symbol	Database ID	Name	Synonyms	Chromosome
AZGP1	563	alpha-2-glycoprotein 1, zinc-binding	ZA2G|ZAG	7
B2M	567	beta-2-microglobulin	IMD43	15
CALR	811	calreticulin	CRT|HEL-S-99n|RO|SSA|cC1qR	19
CANX	821	calnexin	CNX|IP90|P90	5
CD1A	909	CD1a molecule	CD1|FCB6|HTA1|R4|T6	1
CD1B	910	CD1b molecule	CD1|CD1A|R1	1
CD1C	911	CD1c molecule	BDCA1|CD1|CD1A|R7	1
CD1D	912	CD1d molecule	CD1A|R3|R3G1	1
CD1E	913	CD1e molecule	CD1A|R2	1
CD4	920	CD4 molecule	CD4mut	12
CD8A	925	CD8a molecule	CD8|Leu2|p32	2
CD8B	926	CD8b molecule	CD8B1|LEU2|LY3|LYT3|P37	2
CD74	972	CD74 molecule	DHLAG|HLADG|II|Ia-GAMMA|p33	5
CREB1	1385	cAMP responsive element binding protein 1	CREB|CREB-1	2
CTSB	1508	cathepsin B	APPS|CPSB|RECEUP	8
CTSE	1510	cathepsin E	CATE	1
CTSL	1514	cathepsin L	CATL|CTSL1|MEP	9
CTSS	1520	cathepsin S	-	1
FCER1G	2207	Fc fragment of IgE receptor Ig	FCRG	1
FCGRT	2217	Fc fragment of IgG receptor and transporter	FCRN|alpha-chain	19
PDIA3	2923	protein disulfide isomerase family A member 3	ER60|ERp57|ERp60|ERp61|GRP57|GRP58|HEL-S-269|HEL-S-93n|HsT17083|P58|PI-PLC	15
HFE	3077	homeostatic iron regulator	HFE1|HH|HLA-H|MVCD7|TFQTL2	6
HLA-A	3105	major histocompatibility complex, class I, A	HLAA	6

HLA-B	3106	major histocompatibility complex, class I, B	AS|B-4901|HLAB	6
HLA-C	3107	major histocompatibility complex, class I, C	D6S204|HLA-JY3|HLAC|HLC-C|MHC|PSORS1	6
HLA-DMA	3108	major histocompatibility complex, class II, DM alpha	D6S222E|DMA|HLADM|RING6	6
HLA-DMB	3109	major histocompatibility complex, class II, DM beta	D6S221E|RING7	6
HLA-DOA	3111	major histocompatibility complex, class II, DO alpha	HLA-DNA|HLA-DZA|HLADZ	6
HLA-DOB	3112	major histocompatibility complex, class II, DO beta	DOB|HLA_DOB	6
HLA-DPA1	3113	major histocompatibility complex, class II, DP alpha 1	DP(W3)|DP(W4)|DPA1|HLA-DP1A|HLA-DPB1|HLADP|HLASB|PLT1	6
HLA-DPB1	3115	major histocompatibility complex, class II, DP beta 1	DPB1|HLA-DP|HLA-DP1B|HLA-DPB	6
HLA-DQA1	3117	major histocompatibility complex, class II, DQ alpha 1	CELIAC1|DQ-A1|DQA1|HLA-DQA	6
HLA-DQA2	3118	major histocompatibility complex, class II, DQ alpha 2	DC-alpha|DX-ALPHA|HLA-DCA|HLA-DXA|HLADQA2	6
HLA-DQB1	3119	major histocompatibility complex, class II, DQ beta 1	CELIAC1|HLA-DQB|IDDM1	6
HLA-DRA	3122	major histocompatibility complex, class II, DR alpha	HLA-DRA1	6
HLA-DRB1	3123	major histocompatibility complex, class II, DR beta 1	DRB1|HLA-DR1B|HLA-DRB|SS1	6

HLA-DRB3	3125	major histocompatibility complex, class II, DR beta 3	DRB3|HLA-DPB1|HLA-DR1B|HLA-DR3B	6
HLA-DRB4	3126	major histocompatibility complex, class II, DR beta 4	DR4|DRB4|HLA-DR4B|HLA-DRB4*	6
HLA-DRB5	3127	major histocompatibility complex, class II, DR beta 5	-	6
HLA-E	3133	major histocompatibility complex, class I, E	HLA-6.2|QA1	6
HLA-F	3134	major histocompatibility complex, class I, F	CDA12|HLA-5.4|HLA-CDA12|HLAF	6
HLA-G	3135	major histocompatibility complex, class I, G	MHC-G	6
HLA-H	3136	major histocompatibility complex, class I, H (pseudogene)	HLAHP	6
MR1	3140	major histocompatibility complex, class I-related	HLALS	1
HSPA1A	3303	heat shock protein family A (Hsp70) member 1A	HEL-S-103|HSP70-1|HSP70-1A|HSP70-2|HSP70.1|HSP70.2|HSP70I|HSP72|HSPA1	6
HSPA1B	3304	heat shock protein family A (Hsp70) member 1B	HSP70-1|HSP70-1B|HSP70-2|HSP70.1|HSP70.2|HSP72|HSPA1|HSX70	6
HSPA1L	3305	heat shock protein family A (Hsp70) member 1 like	HSP70-1L|HSP70-HOM|HSP70T|hum70t	6
HSPA2	3306	heat shock protein family A (Hsp70) member 2	HSP70-2|HSP70-3	14
HSPA4	3308	heat shock protein family A (Hsp70) member 4	APG-2|HEL-S-5a|HS24/P52|HSPH2|RY|hsp70|hsp70RY	6

HSPA5	3309	heat shock protein family A (Hsp70) member 5	BIP|GRP78|HEL-S-89n|MIF2	9
HSPA6	3310	heat shock protein family A (Hsp70) member 6	HSP70B'	1
HSPA8	3312	heat shock protein family A (Hsp70) member 8	HEL-33|HEL-S-72p|HSC54|HSC70|HSC71|HSP71|HSP73|HSPA10|LAP-1|LAP1|NIP71	11
HSP90AA1	3320	heat shock protein 90 alpha family class A member 1	EL52|HEL-S-65p|HSP86|HSP89A|HSP90A|HSP90N|HSPC1|HSPCA|HSPCAL1|HSPCAL4|HSPN|Hsp103|Hsp89|Hsp90|LAP-2|LAP2	14
HSP90AB1	3326	heat shock protein 90 alpha family class B member 1	D6S182|HSP84|HSP90B|HSPC2|HSPCB	6
ICAM1	3383	intercellular adhesion molecule 1	BB2|CD54|P3.58	19
IFNA1	3439	interferon alpha 1	IFL|IFN|IFN-ALPHA|IFN-alphaD|IFNA13|IFNA@|leIF D	9
IFNA2	3440	interferon alpha 2	IFN-alpha-2|IFN-alphaA|IFNA|IFNA2B|leIF A	9
IFNA4	3441	interferon alpha 4	IFN-alpha4a|INFA4	9
IFNA5	3442	interferon alpha 5	IFN-alpha-5|IFN-alphaG|INA5|INFA5|leIF G	9
IFNA6	3443	interferon alpha 6	IFN-alphaK	9
IFNA7	3444	interferon alpha 7	IFN-alphaJ|IFNA-J	9
IFNA8	3445	interferon alpha 8	IFN-alphaB	9
IFNA10	3446	interferon alpha 10	IFN-alphaC	9
IFNA13	3447	interferon alpha 13	-	9
IFNA14	3448	interferon alpha 14	IFN-alphaH|LEIF2H	9
IFNA16	3449	interferon alpha 16	IFN-alpha-16|IFN-alphaO	9
IFNA17	3451	interferon alpha 17	IFN-alphaI|IFNA|INFA|LEIF2C1	9
IFNA21	3452	interferon alpha 21	IFN-alphaI|LeIF F|leIF-F	9

IFNG	3458	interferon gamma	IFG|IFI	12
KIR2DL1	3802	killer cell immunoglobulin like receptor, two Ig domains and long cytoplasmic tail 1	CD158A|KIR-K64|KIR221|KIR2DL3|NKAT|NKAT-1|NKAT1|p58.1	19
KIR2DL2	3803	killer cell immunoglobulin like receptor, two Ig domains and long cytoplasmic tail 2	CD158B1|CD158b|NKAT-6|NKAT6|p58.2	19
KIR2DL3	3804	killer cell immunoglobulin like receptor, two Ig domains and long cytoplasmic tail 3	CD158B2|CD158b|GL183|KIR-023GB|KIR-K7b|KIR-K7c|KIR2DL|KIR2DS5|KIRCL23|NKAT|NKAT2|NKAT2A|NKAT2B|p58	19
KIR2DL4	3805	killer cell immunoglobulin like receptor, two Ig domains and long cytoplasmic tail 4	CD158D|G9P|KIR-103AS|KIR-2DL4|KIR103|KIR103AS	19
KIR2DS1	3806	killer cell immunoglobulin like receptor, two Ig domains and short cytoplasmic tail 1	CD158H|CD158a|p50.1	19
KIR2DS3	3808	killer cell immunoglobulin like receptor, two Ig domains and short cytoplasmic tail 3	NKAT7	19
KIR2DS4	3809	killer cell immunoglobulin like receptor, two Ig domains and short cytoplasmic tail 4	CD158I|KIR-2DS4|KIR1D|KIR412|KKA3|NKAT-8|NKAT8	19
KIR2DS5	3810	killer cell immunoglobulin like receptor, two Ig domains and short cytoplasmic tail 5	CD158G|NKAT9	19

KIR3DL1	3811	killer cell immunoglobulin like receptor, three Ig domains and long cytoplasmic tail 1	CD158E1|KIR|KIR3DL1/S1|NKAT-3|NKAT3|NKB1|NKB1B	19
KIR3DL2	3812	killer cell immunoglobulin like receptor, three Ig domains and long cytoplasmic tail 2	3DL2|CD158K|KIR-3DL2|NKAT-4|NKAT4|NKAT4B|p140	19
KLRC1	3821	killer cell lectin like receptor C1	CD159A|NKG2|NKG2A	12
KLRC2	3822	killer cell lectin like receptor C2	CD159c|NKG2-C|NKG2C	12
KLRC3	3823	killer cell lectin like receptor C3	NKG2-E|NKG2E	12
KLRD1	3824	killer cell lectin like receptor D1	CD94	12
LTA	4049	lymphotoxin alpha	LT|TNFB|TNFSF1|TNLG1E	6
CIITA	4261	class II major histocompatibility complex transactivator	C2TA|CIITAIV|MHC2TA|NLRA	16
MICA	100507436	MHC class I polypeptide-related sequence A	MIC-A|PERB11.1	6
MICB	4277	MHC class I polypeptide-related sequence B	PERB11.2	6
NFYA	4800	nuclear transcription factor Y subunit alpha	CBF-A|CBF-B|HAP2|NF-YA	6
NFYB	4801	nuclear transcription factor Y subunit beta	CBF-A|CBF-B|HAP3|NF-YB	12
NFYC	4802	nuclear transcription factor Y subunit gamma	CBF-C|CBFC|H1TF2A|HAP5|HSM|NF-YC	1
LGMN	5641	legumain	AEP|LGMN1|PRSC1	14
PSMB8	5696	proteasome 20S subunit beta 8	ALDD|D6S216|D6S216E|JMP|LMP7|NKJO|PRAAS1|PSMB5i|RING10	6
PSMC1	5700	proteasome 26S subunit, ATPase 1	P26S4|S4|p56	14
PSMC2	5701	proteasome 26S subunit, ATPase 2	MSS1|Nbla10058|S7	7

PSMC3	5702	proteasome 26S subunit, ATPase 3	TBP1	11
PSMC4	5704	proteasome 26S subunit, ATPase 4	MIP224|RPT3|S6|TBP-7|TBP7	19
PSMC5	5705	proteasome 26S subunit, ATPase 5	S8|SUG-1|SUG1|TBP10|TRIP1|p45|p45/SUG	17
PSMC6	5706	proteasome 26S subunit, ATPase 6	SUG2|p42	14
PSMD1	5707	proteasome 26S subunit, non-ATPase 1	P112|Rpn2|S1	2
PSMD2	5708	proteasome 26S subunit, non-ATPase 2	P97|RPN1|S2|TRAP2	3
PSMD3	5709	proteasome 26S subunit, non-ATPase 3	P58|RPN3|S3|TSTA2	17
PSMD4	5710	proteasome 26S subunit, non-ATPase 4	AF|AF-1|ASF|MCB1|Rpn10|S5A|pUB-R5	1
PSMD5	5711	proteasome 26S subunit, non-ATPase 5	S5B	9
PSMD7	5713	proteasome 26S subunit, non-ATPase 7	MOV34|P40|Rpn8|S12	16
PSMD8	5714	proteasome 26S subunit, non-ATPase 8	HEL-S-91n|HIP6|HYPF|Nin1p|Rpn12|S14|p31	19
PSMD10	5716	proteasome 26S subunit, non-ATPase 10	dJ889N15.2|p28|p28(GANK)	X
PSMD11	5717	proteasome 26S subunit, non-ATPase 11	Rpn6|S9|p44.5	17
PSMD13	5719	proteasome 26S subunit, non-ATPase 13	HSPC027|Rpn9|S11|p40.5	11
PSME1	5720	proteasome activator subunit 1	HEL-S-129m|IFI5111|PA28A|PA28alpha|REGalpha	14
PSME1	5720	proteasome activator subunit 1	HEL-S-129m|IFI5111|PA28A|PA28alpha|REGalpha	14
PSME2	5721	proteasome activator subunit 2	PA28B|PA28beta|REGbeta	14
PSME2	5721	proteasome activator subunit 2	PA28B|PA28beta|REGbeta	14

RELB	5971	RELB proto-oncogene, NF-kB subunit	I-REL|IMD53|IREL|REL-B	19
RFX5	5993	regulatory factor X5	-	1
RFXAP	5994	regulatory factor X associated protein	-	13
SLC10A2	6555	solute carrier family 10 member 2	ASBT|IBAT|ISBT|NTCP2|PBAM	13
TAP1	6890	transporter 1, ATP binding cassette subfamily B member	ABC17|ABCB2|APT1|D6S114E|PSF-1|PSF1|RING4|TAP1*0102N|TAP1N	6
TAP2	6891	transporter 2, ATP binding cassette subfamily B member	ABC18|ABCB3|APT2|D6S217E|PSF-2|PSF2|RING11	6
TAPBP	6892	TAP binding protein	NGS17|TAPA|TPN|TPSN	6
THBS1	7057	thrombospondin 1	THBS|THBS-1|TSP|TSP-1|TSP1	15
SEM1	7979	SEM1 26S proteasome complex subunit	C7orf76|DSS1|ECD|SHFD1|SHFM1|SHSF1|Shfdg1	7
KLRC4	8302	killer cell lectin like receptor C4	NKG2-F|NKG2F	12
AP3B1	8546	adaptor related protein complex 3 subunit beta 1	ADTB3|ADTB3A|HPS|HPS2|PE	5
RFXANK	8625	regulatory factor X associated ankyrin containing protein	ANKRA1|BLS|F14150_1|RFX-B	19
PSMD6	9861	proteasome 26S subunit, non-ATPase 6	Rpn7|S10|SGA-113M|p42A|p44S10	3
PSME3	10197	proteasome activator subunit 3	HEL-S-283|Ki|PA28-gamma|PA28G|PA28gamma|REG-GAMMA	17
PSMD14	10213	proteasome 26S subunit, non-ATPase 14	PAD1|POH1|RPN11	2
CLEC4M	10332	C-type lectin domain family 4 member M	CD209L|CD299|DC-SIGN2|DC-SIGNR|DCSIGNR|HP10347|L-SIGN|LSIGN	19
IFI30	10437	IFI30 lysosomal thiol reductase	GILT|IFI-30|IP-30|IP30	19

PROCR	10544	protein C receptor	CCCA|CCD41|EPCR	20
ADRM1	11047	adhesion regulating molecule 1	ARM-1|ARM1|GP110	20
ECPAS	23392	Ecm29 proteasome adaptor and scaffold	ECM29|KIAA0368	9
TRPC4AP	26133	transient receptor potential cation channel subfamily C member 4 associated protein	C20orf188|PPP1R158|TRRP4AP|TRUSS	20
CD209	30835	CD209 molecule	CDSIGN|CLEC4L|DC-SIGN|DC-SIGN1	19
UBXN1	51035	UBX domain protein 1	2B28|SAKS1|UBXD10	11
ERAP1	51752	endoplasmic reticulum aminopeptidase 1	A-LAP|ALAP|APPILS|ARTS-1|ARTS1|ERAAP|ERAAP1|PILS-AP|PILSAP	5
TAPBPL	55080	TAP binding protein like	TAPBP-R|TAPBPR	12
KIR2DL5A	57292	killer cell immunoglobulin like receptor, two Ig domains and long cytoplasmic tail 5A	CD158F|KIR2DL5|KIR2DL5.1|KIR2DL5.3	19
ERAP2	64167	endoplasmic reticulum aminopeptidase 2	L-RAP|LRAP	5
ULBP3	79465	UL16 binding protein 3	N2DL-3|NKG2DL3|RAET1N	6
ULBP2	80328	UL16 binding protein 2	ALCAN-alpha|N2DL2|NKG2DL2|RAET1H|RAET1L	6
ULBP1	80329	UL16 binding protein 1	N2DL-1|NKG2DL1|RAET1I	6
KIR3DL3	115653	killer cell immunoglobulin like receptor, three Ig domains and long cytoplasmic tail 3	CD158Z|KIR3DL7|KIR44|KIRC1	19

RAET1E	135250	retinoic acid early transcript 1E	LETAL|N2DL-4|NKG2DL4|RAET1E2|RL-4|ULBP4|bA350J20.7	6
RAET1L	154064	retinoic acid early transcript 1L	ULBP6	6
UBR1	197131	ubiquitin protein ligase E3 component n-recognin 1	JBS	15
RAET1G	353091	retinoic acid early transcript 1G	ULBP5	6
PDIA2	64714	protein disulfide isomerase family A member 2	PDA2|PDI|PDIP|PDIR	16
HAMP	57817	hepcidin antimicrobial peptide	HEPC|HFE2B|LEAP1|PLTR	19
PI3	5266	peptidase inhibitor 3	ESI|SKALP|WAP3|WFDC14|cementoin	20
CAMP	820	cathelicidin antimicrobial peptide	CAP-18|CAP18|CRAMP|FALL-39|FALL39|HSD26|LL37	3
DEFB4A	1673	defensin beta 4A	BD-2|DEFB-2|DEFB102|DEFB2|DEFB4|HBD-2|SAP1	8
PPBP	5473	pro-platelet basic protein	B-TG1|Beta-TG|CTAP-III|CTAP3|CTAPIII|CXCL7|LA-PF4|LDGF|MDGF|NAP-2|PBP|SCYB7|TC1|TC2|TGB|TGB1|THBGB|THBGB1	4
REG3G	130120	regenerating family member 3 gamma	LPPM429|PAPIB|PAP-1B|PAP1B|PAPIB|REG III|REG-III|UNQ429	2
CXCL14	9547	C-X-C motif chemokine ligand 14	BMAC|BRAK|KEC|KS1|MIP-2g|MIP2G|NJAC|SCYB14	5
CXCL16	58191	C-X-C motif chemokine ligand 16	CXCLG16|SR-PSOX|SRPSOX	17

SLPI	6590	secretory leukocyte peptidase inhibitor	ALK1|ALP|BLPI|HUSI|HUSI-I|MPI|WAP4|WFDC4	20
CXCL8	3576	C-X-C motif chemokine ligand 8	GCP-1|GCP1|IL8|LECT|LUCT|LYNAP|MDNCF|MONAP|NAF|NAP-1|NAP1|SCYB8	4
CXCL10	3627	C-X-C motif chemokine ligand 10	C7|IFI10|INP10|IP-10|SCYB10|crg-2|gIP-10|mob-1	4
CXCL9	4283	C-X-C motif chemokine ligand 9	CMK|Humig|MIG|SCYB9|crg-10	4
CXCL5	6374	C-X-C motif chemokine ligand 5	ENA-78|SCYB5	4
CXCL11	6373	C-X-C motif chemokine ligand 11	H174|I-TAC|IP-9|IP9|SCYB11|SCYB9B|b-R1	4
CXCL6	6372	C-X-C motif chemokine ligand 6	CKA-3|GCP-2|GCP2|SCYB6	4
CXCL1	2919	C-X-C motif chemokine ligand 1	FSP|GRO1|GROa|MGSA|MGSA-a|NAP-3|SCYB1	4
CXCL12	6387	C-X-C motif chemokine ligand 12	IRH|PBSF|SCYB12|SDF1|TLSF|TPAR1	10
CXCL13	10563	C-X-C motif chemokine ligand 13	ANGIE|ANGIE2|BCA-1|BCA1|BLC|BLR1L|SCYB13	4
CXCL2	2920	C-X-C motif chemokine ligand 2	CINC-2a|GRO2|GROb|MGSA-b|MIP-2a|MIP2|MIP2A|SCYB2	4
PF4	5196	platelet factor 4	CXCL4|PF-4|SCYB4	4
XCL1	6375	X-C motif chemokine ligand 1	ATAC|LPTN|LTN|SCM-1|SCM-1a|SCM1|SCM1A|SCYC1	1
CXCL3	2921	C-X-C motif chemokine ligand 3	CINC-2b|GRO3|GROg|MIP-2b|MIP2B|SCYB3	4

DEFB103B	55894	defensin beta 103B	BD-3|DEFB-3|DEFB103|DEFB3|HBD-3|HBD3|HBP-3|HBP3	8
CCL13	6357	C-C motif chemokine ligand 13	CKb10|MCP-4|NCC-1|NCC1|SCYA13|SCYL1	17
CCL1	6346	C-C motif chemokine ligand 1	I-309|P500|SCYA1|SISe|TCA3	17
DEFB1	1672	defensin beta 1	BD1|DEFB-1|DEFB101|HBD1	8
CCL8	6355	C-C motif chemokine ligand 8	HC14|MCP-2|MCP2|SCYA10|SCYA8	17
ELANE	1991	elastase, neutrophil expressed	ELA2|GE|HLE|HNE|NE|PMN-E|SCN1	19
DEFB103A	414325	defensin beta 103A	BD-3|DEFB-3|DEFB103|DEFB3|HBD3|HBP-3|HBP3	8
DEFA3	1668	defensin alpha 3	DEF3|HNP-3|HNP3|HP-3|HP3	8
DEFA1	1667	defensin alpha 1	DEF1|DEFA2|HNP-1|HP-1|HP1|MRS	8
TMSB10	9168	thymosin beta 10	MIG12|TB10	2
DEFA6	1671	defensin alpha 6	DEF6|HD-6	8
DEFA5	1670	defensin alpha 5	DEF5|HD-5	8
DEFA4	1669	defensin alpha 4	DEF4|HNP-4|HP-4|HP4	8
LCN2	3934	lipocalin 2	24p3|MSFI|NGAL|p25	9
LCN1	3933	lipocalin 1	PMFA|TLC|TP|VEGP	9
COLEC10	10584	collectin subfamily member 10	3MC3|CL-34|CLL1	8
BPI	671	bactericidal permeability increasing protein	BPIFD1|rBPI	20
S100A9	6280	S100 calcium binding protein A9	60B8AG|CAGB|CFAG|CGLB|L1AG|LIAG|MAC387|MIF|MRP14|NIF|P14	1
S100A8	6279	S100 calcium binding protein A8	60B8AG|CAGA|CFAG|CGLA|CP-10|L1Ag|MA387|MIF|MRP8|NIF|P8	1
DCD	117159	dermcidin	AIDD|DCD-1|DSEP|HCAP|PIF	12

LCN6	158062	lipocalin 6	LCN5|UNQ643|hLcn5	9
S100A12	6283	S100 calcium binding protein A12	CAAF1|CAGC|CGRP|ENRAGE|MRP-6|MRP6|p6	1
HTN3	3347	histatin 3	HIS2|HTN2|HTN5|PB	4
LCN8	138307	lipocalin 8	EP17|LCN5	9
DEFA1B	728358	defensin alpha 1B	HNP-1|HP-1|HP1	8
CCR10	2826	C-C motif chemokine receptor 10	GPR2	17
CELA1	1990	chymotrypsin like elastase 1	ELA1	12
DEFB106A	245909	defensin beta 106A	BD-6|DEFB-6|DEFB106	8
PENK	5179	proenkephalin	PE|PENK-A	8
BPIFC	254240	BPI fold containing family C	BPIL2	22
MMP12	4321	matrix metallopeptidase 12	HME|ME|MME|MMP-12	11
BPIFB6	128859	BPI fold containing family B member 6	BPIL3|LPLUNC6	20
LEAP2	116842	liver enriched antimicrobial peptide 2	LEAP-2	5
SFTPD	6441	surfactant protein D	COLEC7|PSP-D|SFTP4|SP-D	10
LCN9	392399	lipocalin 9	HEL129	9
BPIFB2	80341	BPI fold containing family B member 2	BPIL1|C20orf184|LPLUNC2|RYSR|dJ726C3.2	20
PTGDS	5730	prostaglandin D2 synthase	L-PGDS|LPGDS|PDS|PGD2|PGDS|PGDS2	9
TMSB4X	7114	thymosin beta 4 X-linked	FX|PTMB4|TB4X|TMSB4	X
PGLYRP1	8993	peptidoglycan recognition protein 1	PGLYRP|PGRP|PGRP-S|PGRPS|TAG7|TNFSF3L	19
ZC3HAV1	56829	zinc finger CCCH-type containing, antiviral 1	ARTD13|FLB6421|PARP13|ZAP|ZC3H2|ZC3HDC2	7
TMSB15A	11013	thymosin beta 15a	TMSB15|TMSB15B|TMSL8|TMSNB|Tb15|TbNB	X

S100B	6285	S100 calcium binding protein B	NEF|S100|S100-B|S100beta	21
S100A13	6284	S100 calcium binding protein A13	-	1
S100A6	6277	S100 calcium binding protein A6	2A9|5B10|CABP|CACY|PRA|S10A6	1
DEFB119	245932	defensin beta 119	DEFB-19|DEFB-20|DEFB120|DEFB20|ESC42-RELA|ESC42-RELB	20
DEFB107A	245910	defensin beta 107A	BD-7|DEFB-7|DEFB107	8
DEFB105A	245908	defensin beta 105A	BD-5|DEFB-5|DEFB105	8
SERPIND1	3053	serpin family D member 1	D22S673|HC2|HCF2|HCII|HLS2|LS2|THPH10	22
DEFB129	140881	defensin beta 129	C20orf87|DEFB-29|DEFB29|bA530N10.3|hBD-29	20
DEFB127	140850	defensin beta 127	C20orf73|DEF-27|DEFB-27|DEFB27|bA530N10.2|hBD-27	20
S100P	6286	S100 calcium binding protein P	MIG9	4
S100A7	6278	S100 calcium binding protein A7	PSOR1|S100A7c	1
DEFB104A	140596	defensin beta 104A	BD-4|DEFB-4|DEFB104|DEFB4|hBD-4	8
DEFB126	81623	defensin beta 126	C20orf8|DEFB-26|DEFB26|HBD26|bA530N10.1|hBD-26	20
DEFB106B	503841	defensin beta 106B	BD-6|DEFB-6	8
DEFB104B	503618	defensin beta 104B	BD-4|DEFB-4|hBD-4	8
DEFB107B	503614	defensin beta 107B	HsT21816	8
PGLYRP3	114771	peptidoglycan recognition protein 3	PGLYRPIalpha|PGRP-Ialpha|PGRPIA	1
PGLYRP2	114770	peptidoglycan recognition protein 2	HMFT0141|PGLYRPL|PGRP-L|PGRPL|TAGL-like|tagL|tagL-alpha|tagl-beta	19

S100A10	6281	S100 calcium binding protein A10	42C|ANX2L|ANX2LG|CAL1L|CLP11|Ca[1]|GP11|P11|p10	1
S100A2	6273	S100 calcium binding protein A2	CAN19|S100L	1
DEFB125	245938	defensin beta 125	DEFB-25	20
DEFB123	245936	defensin beta 123	DEFB-23|DEFB23|ESC42-RELD	20
DEFB105B	504180	defensin beta 105B	BD-5|DEFB-5	8
DEFB132	400830	defensin beta 132	BD-32|DEFB-32|DEFB32|HEL-75|KFLL827|UNQ827	20
BPIFB3	359710	BPI fold containing family B member 3	C20orf185|LPLUNC3|RYA3	20
LCN12	286256	lipocalin 12	-	9
PGLYRP4	57115	peptidoglycan recognition protein 4	PGLYRPIbeta|PGRP-Ibeta|PGRPIB|SBBI67	1
S100A11	6282	S100 calcium binding protein A11	HEL-S-43|MLN70|S100C	1
S100A5	6276	S100 calcium binding protein A5	S100D	1
S100A3	6274	S100 calcium binding protein A3	S100E	1
S100A1	6271	S100 calcium binding protein A1	S100|S100-alpha|S100A	1
DEFB128	245939	defensin beta 128	DEFB-28|DEFB28|hBD-28	20
DEFB108B	245911	defensin beta 108B	DEFB-8|hBD-8	11
HTN1	3346	histatin 1	HIS1	4
LMBR1L	55716	limb development membrane protein 1 like	LIMR	12
S100A7A	338324	S100 calcium binding protein A7A	NICE-2|NICE2|S100A15|S100A7L1|S100A7f	1
DEFB118	117285	defensin beta 118	C20orf63|DEFB-18|ESC42|ESP13.6	20
COLEC12	81035	collectin subfamily member 12	CLP1|NSR2|SCARA4|SRCL	18
TMSB4Y	9087	thymosin beta 4 Y-linked	TB4Y	Y
DEFB131A	644414	defensin beta 131A	DEFB-31|DEFB131	4

DEFB134	613211	defensin beta 134	-	8
DEFB130A	245940	defensin beta 130A	DEFB-30|DEFB130|DEFB130L|DEFB30	8
DEFB124	245937	defensin beta 124	DEFB-24	20
DEFB121	245934	defensin beta 121	DEFB21|ESC42RELC	20
DEFB116	245930	defensin beta 116	DEFB-16	20
DEFB115	245929	defensin beta 115	DEFB-15	20
DEFB114	245928	defensin beta 114	DEFB-14|DEFB14	6
DEFB113	245927	defensin beta 113	DEFB-13	6
DEFB112	245915	defensin beta 112	DEFB-12	6
DEFB110	245913	defensin beta 110	DEFB-10|DEFB-11|DEFB111	6
TMSB15B	286527	thymosin beta 15B	TMSB15A|TMSL8|TMSNB|Tbeta15b	X
DEFB133	403339	defensin beta 133	-	6
S100Z	170591	S100 calcium binding protein Z	Gm625|S100-zeta	5
MAVS	57506	mitochondrial antiviral signaling protein	CARDIF|IPS-1|IPS1|VISA	20
TMSB4XP8	7117	TMSB4X pseudogene 8	TMSL3	4
S100A14	57402	S100 calcium binding protein A14	BCMP84|S100A15	1
LCN10	414332	lipocalin 10	-	9
S100A16	140576	S100 calcium binding protein A16	AAG13|DT1P1A7|S100F	1
DEFB136	613210	defensin beta 136	DEFB137	8
DEFB135	613209	defensin beta 135	DEFB136	8
DEFB117	245931	defensin beta 117 (pseudogene)	DEFB-17	20
DEFB110	245913	defensin beta 110	DEFB-10|DEFB-11|DEFB111	6
ZC3HAV1L	92092	zinc finger CCCH-type containing, antiviral 1 like	C7orf39	7
S100A7L2	645922	S100 calcium binding protein A7 like 2	S100a7b	1
MBL3P	50639	mannose-binding lectin family member 3, pseudogene	COLEC2|MBL	10
DEFB4B	100289462	defensin beta 4B	DEFB4P	8

BPIFB4	149954	BPI fold containing family B member 4	C20orf186|LPLUNC4|RY2G5|dJ726C3.5	20
IFNAR1	3454	interferon alpha and beta receptor subunit 1	AVP|IFN-alpha-REC|IFNAR|IFNBR|IFRC	21
AZU1	566	azurocidin 1	AZAMP|AZU|CAP37|HBP|HUMAZUR|NAZC|hHBP	19
DEFB131B	100129216	defensin beta 131B	-	11
DEFA1A3	613253	defensin alpha 1 and alpha 3, variable copy number locus	DEFA1|DEFA3|DEFT1P	8
LCN1P1	286310	lipocalin 1 pseudogene 1	LCN1L1|bA430N14.2	9
S100G	795	S100 calcium binding protein G	CABP|CABP1|CABP9K|CALB3	X
DEFA7P	724067	defensin alpha 7, pseudogene	DEFA7	8
DEFB130B	100133267	defensin beta 130B	-	8
DEFB108F	100133128	defensin beta 108F (pseudogene)	DEFB108P5	4
DEFB131C	100128174	defensin beta 131C (pseudogene)	-	8
TCHHL1	126637	trichohyalin like 1	S100A17|THHL1	1
TINAGL1	64129	tubulointerstitial nephritis antigen like 1	ARG1|LCN7|LIECG3|TINAGRP	1
IFNGR1	3459	interferon gamma receptor 1	CD119|IFNGR|IMD27A|IMD27B	6
SLC22A17	51310	solute carrier family 22 member 17	24p3R|BOCT|BOIT|NGALR|NGALR2|NGALR3|hBOIT	14
WFIKKN1	117166	WAP, follistatin/kazal, immunoglobulin, kunitz and netrin domain containing 1	C16orf12|RJD2|WFDC20A|WFIKKN	16
WFDC2	10406	WAP four-disulfide core domain 2	EDDM4|HE4|WAP5|dJ461P17.6	20
IL6	3569	interleukin 6	BSF-2|BSF2|CDF|HGF|HSF|IFN-beta-2|IFNB2|IL-6	7
UMODL1	89766	uromodulin like 1	-	21

TGFB1	7040	transforming growth factor beta 1	CED|DPD1|IBDIMDE|LAP|TGF-beta1|TGFB|TGFbeta	19
PF4V1	5197	platelet factor 4 variant 1	CXCL4L1|CXCL4V1|PF4-ALT|PF4A|SCYB4V1	4
MMP9	4318	matrix metallopeptidase 9	CLG4B|GELB|MANDP2|MMP-9	20
ANOS1	3730	anosmin 1	ADMLX|HH1|HHA|KAL|KAL1|KALIG-1|KMS|WFDC19	X
TLR4	7099	toll like receptor 4	ARMD10|CD284|TLR-4|TOLL	9
IFNG	3458	interferon gamma	IFG|IFI	12
SPAG11B	10407	sperm associated antigen 11B	EDDM2B|EP2|EP2C|EP2D|HE2|HE2C|SPAG11|SPAG11A	8
A2M	2	alpha-2-macroglobulin	A2MD|CPAMD5|FWP007|S863-7	12
CTSL	1514	cathepsin L	CATL|CTSL1|MEP	9
NFKB1	4790	nuclear factor kappa B subunit 1	CVID12|EBP-1|KBF1|NF-kB|NF-kB1|NF-kappa-B1|NF-kappaB|NF-kappabeta|NFKB-p105|NFKB-p50|NFkappaB	4
APOBEC3(	60489	apolipoprotein B mRNA editing enzyme catalytic subunit 3G	A3G|ARCD|ARP-9|ARP9|CEM-15|CEM15|MDS019|bK150C2.7|dJ494G10.1	22
FABP6	2172	fatty acid binding protein 6	I-15P|I-BABP|I-BALB|I-BAP|ILBP|ILBP3|ILLBP	5
NOD2	64127	nucleotide binding oligomerization domain containing 2	ACUG|BLAU|BLAUS|CARD15|CD|CLR16.3|IBD1|NLRC2|NOD2B|PSORAS1|YAOS	16
MBL2	4153	mannose binding lectin 2	COLEC1|HSMBPC|MBL|MBL2D|MBP|MBP-C|MBP1|MBPD	10

SFTPA1	653509	surfactant protein A1	COLEC4|PSAP|PSP-A|PSPA|SFTP1|SFTPA1B|SP-A|SP-A1|SP-A1 beta|SP-A1 delta|SP-A1 epsilon|SP-A1 gamma|SPA|SPA1	10
RBP1	5947	retinol binding protein 1	CRABP-I|CRBP|CRBP1|CRBPI|RBPC	3
TLR2	7097	toll like receptor 2	CD282|TIL4	4
SLC40A1	30061	solute carrier family 40 member 1	FPN1|HFE4|IREG1|MST079|MSTP079|MTP1|SLC11A3	2
PLAU	5328	plasminogen activator, urokinase	ATF|BDPLT5|QPD|UPA|URK|u-PA	10
IL1B	3553	interleukin 1 beta	IL-1|IL1-BETA|IL1F2|IL1beta	2
PAEP	5047	progestagen associated endometrial protein	GD|GdA|GdF|GdS|PAEG|PEP|PP14|ZIF-1	9
HJV	148738	hemojuvelin BMP co-receptor	HFE2|HFE2A|JH|RGMC	1
MUC5AC	4586	mucin 5AC, oligomeric mucus/gel-forming	MUC5|TBM|leB|mucin	11
CTSS	1520	cathepsin S	-	1
OBP2A	29991	odorant binding protein 2A	LCN13|OBP|OBP2C|OBPIIa|hOBPIIa	9
PLTP	5360	phospholipid transfer protein	BPIFE|HDLCQ9	20
MX1	4599	MX dynamin like GTPase 1	IFI-78K|IFI78|MX|MxA|lncMX1-215	21
DDX58	23586	DExD/H-box helicase 58	RIG-I|RIG1|RIGI|RLR-1|SGMRT2	9
IFNL1	282618	interferon lambda 1	IL-29|IL29	19
IRF3	3661	interferon regulatory factor 3	IIAE7	19
SFTPA2	729238	surfactant protein A2	COLEC5|PSAP|PSP-A|PSPA|SFTP1|SFTPA2B|SP-2A|SP-A|SPA2|SPAII	10
LPA	4018	lipoprotein(a)	AK38|APOA|LP	6

LBP	3929	lipopolysaccharide binding protein	BPIFD2	20
RBP4	5950	retinol binding protein 4	MCOPCB10|RDCCAS	10
SFTPA1	653509	surfactant protein A1	COLEC4|PSAP|PSP-A|PSPA|SFTP1|SFTPA1B|SP-A|SP-A1|SP-A1 beta|SP-A1 delta|SP-A1 epsilon|SP-A1 gamma|SPA|SPA1	10
NOX4	50507	NADPH oxidase 4	KOX|KOX-1|RENOX	11
LTF	4057	lactotransferrin	GIG12|HEL110|HLF2|LF	3
IFNB1	3456	interferon beta 1	IFB|IFF|IFN-beta|IFNB	9
RBP5	83758	retinol binding protein 5	CRBP-III|CRBP3|CRBPIII|HRBPiso	12
FABP7	2173	fatty acid binding protein 7	B-FABP|BLBP|FABPB|MRG	6
FABP5	2171	fatty acid binding protein 5	E-FABP|EFABP|KFABP|PA-FABP|PAFABP	8
FABP3	2170	fatty acid binding protein 3	FABP11|H-FABP|M-FABP|MDGI|O-FABP	1
FABP2	2169	fatty acid binding protein 2	FABPI|I-FABP	4
FABP4	2167	fatty acid binding protein 4	A-FABP|AFABP|ALBP|HEL-S-104|aP2	8
R3HDML	140902	R3H domain containing like	dJ881L22.3	20
BPIFA3	128861	BPI fold containing family A member 3	C20orf71|SPLUNC3	20
BPIFB1	92747	BPI fold containing family B member 1	C20orf114|LPLUNC1	20
OASL	8638	2'-5'-oligoadenylate synthetase like	OASL1|OASLd|TRIP-14|TRIP14|p59 OASL|p59-OASL|p59OASL	12
CRABP2	1382	cellular retinoic acid binding protein 2	CRABP-II|RBP6	1

CRABP1	1381	cellular retinoic acid binding protein 1	CRABP|CRABP-I|CRABPI|RBP5	15
RBP7	116362	retinol binding protein 7	CRABP4|CRBP4|CRBPIV	1
DUOX1	53905	dual oxidase 1	LNOX1|NOXEF1|THOX1	15
OBP2B	29989	odorant binding protein 2B	LCN14|OBPIIb	9
RBP2	5948	retinol binding protein 2	CRABP-II|CRBP2|CRBPII|RBPC2	3
LCN15	389812	lipocalin 15	PRO6093|UNQ2541	9
CETP	1071	cholesteryl ester transfer protein	BPIFF|HDLCQ10	16
FABP12	646486	fatty acid binding protein 12	-	8
FABP9	646480	fatty acid binding protein 9	PERF|PERF15|T-FABP|TLBP	8
BPIFA1	51297	BPI fold containing family A member 1	LUNX|NASG|PLUNC|SPLUNC1|SPURT|bA49G10.5	20
LCNL1	401562	lipocalin like 1	-	9
C8G	733	complement C8 gamma chain	C8C	9
SPAG11A	653423	sperm associated antigen 11A	EDDM2A|HE2	8
PI15	51050	peptidase inhibitor 15	CRISP8|P24TI|P25TI	8
NOX1	27035	NADPH oxidase 1	GP91-2|MOX1|NOH-1|NOH1	X
PMP2	5375	peripheral myelin protein 2	CMT1G|FABP8|M-FABP|MP2|P2	8
APOD	347	apolipoprotein D	-	3
ORM2	5005	orosomucoid 2	AGP-B|AGP-B'|AGP2	9
ORM1	5004	orosomucoid 1	AGP-A|AGP1|HEL-S-153w|ORM	9
TNF	7124	tumor necrosis factor	DIF|TNF-alpha|TNFA|TNFSF2|TNLG1F	6
CTSG	1511	cathepsin G	CATG|CG	14
PRTN3	5657	proteinase 3	ACPA|AGP7|C-ANCA|CANCA|MBN|MBT|NP-4|NP4|P29|PR-3|PR3	19

MAPK1	5594	mitogen-activated protein kinase 1	ERK|ERK-2|ERK2|ERT1|MAPK2|P42MAPK|PRKM1|PRKM2|p38|p40|p41|p41mapk|p42-MAPK	22
PML	5371	PML nuclear body scaffold	MYL|PP8675|RNF71|TRIM19	15
AEN	64782	apoptosis enhancing nuclease	ISG20L1|pp12744	15
CYBB	1536	cytochrome b-245 beta chain	AMCBX2|CGD|GP91-1|GP91-PHOX|GP91PHOX|IMD34|NOX2|p91-PHOX	X
BPIFA2	140683	BPI fold containing family A member 2	C20orf70|PSP|SPLUNC2|bA49G10.1	20
ISG20	3669	interferon stimulated exonuclease gene 20	CD25|HEM45	15
BCL3	602	BCL3 transcription coactivator	BCL4|D19S37	19
ISG20L2	81875	interferon stimulated exonuclease gene 20 like 2	HSD38	1
NOX5	79400	NADPH oxidase 5	-	15
NOX3	50508	NADPH oxidase 3	GP91-3|MOX-2	6
DUOX2	50506	dual oxidase 2	LNOX2|NOXEF2|P138-TOX|TDH6|THOX2	15
TLR3	7098	toll like receptor 3	CD283|IIAE2	4
TFRC	7037	transferrin receptor	CD71|IMD46|T9|TFR|TFR1|TR|TRFR|p90	3
IFIH1	64135	interferon induced with helicase C domain 1	AGS7|Hlcd|IDDM19|MDA-5|MDA5|RLR-2|SGMRT1	2
LRP1	4035	LDL receptor related protein 1	A2MR|APOER|APR|CD91|IGFBP-3R|IGFBP3R|IGFBP3R1|KPA|LRP|LRP1A|TGFBR5	12
TRIM5	85363	tripartite motif containing 5	RNF88|TRIM5alpha	11
IDO1	3620	indoleamine 2,3-dioxygenase 1	IDO|IDO-1|INDO	8

GDF15	9518	growth differentiation factor 15	GDF-15|MIC-1|MIC1|NAG-1|PDF|PLAB|PTGFB	19
NEDD4	4734	NEDD4 E3 ubiquitin protein ligase	NEDD4-1|RPF1	15
ADIPOQ	9370	adiponectin, C1Q and collagen domain containing	ACDC|ACRP30|ADIPQTL1|ADPN|APM-1|APM1|GBP28	3
STAT3	6774	signal transducer and activator of transcription 3	ADMIO|ADMIO1|APRF|HIES	17
STAT1	6772	signal transducer and activator of transcription 1	CANDF7|IMD31A|IMD31B|IMD31C|ISGF-3|STAT91	2
IFNL2	282616	interferon lambda 2	IL-28A|IL28A	19
SOCS3	9021	suppressor of cytokine signaling 3	ATOD4|CIS3|Cish3|SOCS-3|SSI-3|SSI3	17
SEMG1	6406	semenogelin 1	CT103|SEMG|SGI|dJ172H20.2	20
TNFSF10	8743	TNF superfamily member 10	APO2L|Apo-2L|CD253|TL2|TNLG6A|TRAIL	3
CCL20	6364	C-C motif chemokine ligand 20	CKb4|Exodus|LARC|MIP-3-alpha|MIP-3a|MIP3A|SCYA20|ST38	2
SOCS1	8651	suppressor of cytokine signaling 1	CIS1|CISH1|JAB|SOCS-1|SSI-1|SSI1|TIP-3|TIP3	16
RNASEL	6041	ribonuclease L	PRCA1|RNS4	1
IRF1	3659	interferon regulatory factor 1	IRF-1|MAR	5
IL15	3600	interleukin 15	IL-15	4
APOBEC3l	200316	apolipoprotein B mRNA editing enzyme catalytic subunit 3F	A3F|ARP8|BK150C2.4.MRNA|KA6	22
PLAAT4	5920	phospholipase A and acyltransferase 4	HRASLS4|HRSL4|PLA1/2-3|PLAAT-4|RARRES3|RIG1|TIG3	11
CHIT1	1118	chitinase 1	CHI3|CHIT|CHITD	1

IFNA1	3439	interferon alpha 1	IFL|IFN|IFN-ALPHA|IFN-alphaD|IFNA13|IFNA@|leIF D	9
CD40	958	CD40 molecule	Bp50|CDW40|TNFRSF5|p50	20
TLR7	51284	toll like receptor 7	TLR7-like	X
PPIA	5478	peptidylprolyl isomerase A	CYPA|CYPH|HEL-S-69p	7
HFE	3077	homeostatic iron regulator	HFE1|HH|HLA-H|MVCD7|TFQTL2	6
ZYX	7791	zyxin	ESP-2|HED-2	7
NLRX1	79671	NLR family member X1	CLR11.3|DLNB26|NOD26|NOD5|NOD9	11
PGC	5225	progastricsin	PEPC|PGII	6
VEGFA	7422	vascular endothelial growth factor A	MVCD1|VEGF|VPF	6
IKBKE	9641	inhibitor of nuclear factor kappa B kinase subunit epsilon	IKK-E|IKK-i|IKKE|IKKI	1
ISG15	9636	ISG15 ubiquitin like modifier	G1P2|IFI15|IMD38|IP17|UCRP|hUCRP	1
DHX58	79132	DExH-box helicase 58	D11LGP2|D11lgp2e|LGP2|RLR-3	17
TNFAIP3	7128	TNF alpha induced protein 3	A20|AISBL|OTUD7C|TNFA1P2	6
TFR2	7036	transferrin receptor 2	HFE3|TFRC2	7
FCN2	2220	ficolin 2	EBP-37|FCNL|P35|ficolin-2	9
MUC4	4585	mucin 4, cell surface associated	ASGP|HSA276359|MUC-4	3
F2R	2149	coagulation factor II thrombin receptor	CF2R|HTR|PAR-1|PAR1|TR	5
ELN	2006	elastin	ADCL1|SVAS|WBS|WS	7
IL27	246778	interleukin 27	IL-27|IL-27A|IL27A|IL27p28|IL30|p28	16
MAPT	4137	microtubule associated protein tau	DDPAC|FTDP-17|MAPTL|MSTD|MTBT1|MTBT2|PPND|PPP1R103|TAU	17
LYZ	4069	lysozyme	LYZF1|LZM	12

CCL5	6352	C-C motif chemokine ligand 5	D17S136E|RANTES|SCYA5|SIS-delta|SISd|TCP228|eoCP	17
LEP	3952	leptin	LEPD|OB|OBS	7
CYLD	1540	CYLD lysine 63 deubiquitinase	BRSS|CDMT|CYLD1|CYLDI|EAC|MFT|MFT1|SBS|TEM|USPL2	16
KLKB1	3818	kallikrein B1	KLK3|PKK|PKKD|PPK	4
CST4	1472	cystatin S	-	20
CSRP1	1465	cysteine and glycine rich protein 1	CRP|CRP1|CSRP|CYRP|D1S181E|HEL-141|HEL-S-286	1
MAPK14	1432	mitogen-activated protein kinase 14	CSBP|CSBP1|CSBP2|CSPB1|EXIP|Mxi2|PRKM14|PRKM15|RK|SAPK2A|p38|p38ALPHA	6
JUN	3725	Jun proto-oncogene, AP-1 transcription factor subunit	AP-1|AP1|c-Jun|cJUN|p39	1
ITGAV	3685	integrin subunit alpha V	CD51|MSK8|VNRA|VTNR	2
IRF5	3663	interferon regulatory factor 5	SLEB10	7
CCR6	1235	C-C motif chemokine receptor 6	BN-1|C-C CKR-6|CC-CKR-6|CCR-6|CD196|CKR-L3|CKRL3|CMKBR6|DCR2|DRY6|GPR29|GPRCY4|STRL22	6
IL12B	3593	interleukin 12B	CLMF|CLMF2|IL-12B|IMD28|IMD29|NKSF|NKSF2	5
TLR8	51311	toll like receptor 8	CD288	X
GNLY	10578	granulysin	D2S69E|LAG-2|LAG2|NKG5|TLA519	2
CD81	975	CD81 molecule	CVID6|S5.7|TAPA1|TSPAN28	11
EIF2AK2	5610	eukaryotic translation initiation factor 2 alpha kinase 2	EIF2AK1|LEUDEN|PKR|PPP1R83|PRKR	2
APOM	55937	apolipoprotein M	G3a|HSPC336|NG20|apo-M	6

CACYBP	27101	calcyclin binding protein	GIG5|PNAS-107|S100A6BP|SIP	1
NOD1	10392	nucleotide binding oligomerization domain containing 1	CARD4|CLR7.1|NLRC1	7
MAPK8	5599	mitogen-activated protein kinase 8	JNK|JNK-46|JNK1|JNK1A2|JNK21B1/2|PRKM8|SAPK1|SAPK1c	10
MAPK3	5595	mitogen-activated protein kinase 3	ERK-1|ERK1|ERT2|HS44KDAP|HUMKER1A|P44ERK1|P44MAPK|PRKM3|p44-ERK1|p44-MAPK	16
BST2	684	bone marrow stromal cell antigen 2	CD317|TETHERIN	19
BPHL	670	biphenyl hydrolase like	BPH-RP|MCNAA|VACVASE	6
PLA2G2A	5320	phospholipase A2 group IIA	MOM1|PLA2|PLA2B|PLA2L|PLA2S|PLAS1|sPLA2	1
GRN	2896	granulin precursor	CLN11|GEP|GP88|PCDGF|PEPI|PGRN	17
NEWENTR	192343	-	-	-
PDGFRA	5156	platelet derived growth factor receptor alpha	CD140A|PDGFR-2|PDGFR2	4
GNAI1	2770	G protein subunit alpha i1	Gi	7
WNT5A	7474	Wnt family member 5A	hWNT5A	3
FURIN	5045	furin, paired basic amino acid cleaving enzyme	FUR|PACE|PCSK3|SPC1	15
ADAR	103	adenosine deaminase RNA specific	ADAR1|AGS6|DRADA|DSH|DSRAD|G1P1|IFI-4|IFI4|K88DSRBP|P136	1
TYK2	7297	tyrosine kinase 2	IMD35|JTK1	19
NOS2	4843	nitric oxide synthase 2	HEP-NOS|INOS|NOS|NOS2A	17

TRAF3	7187	TNF receptor associated factor 3	CAP-1|CAP1|CD40bp|CRAF1|IIAE5|LAP1|RNF118	14
TPT1	7178	tumor protein, translationally-controlled 1	HRF|TCTP|p02|p23	13
TPM2	7169	tropomyosin 2	AMCD1|DA1|DA2B|DA2B4|HEL-S-273|NEM4|TMSB	9
NEO1	4756	neogenin 1	IGDCC2|NGN|NTN1R2	15
AHNAK	79026	AHNAK nucleoprotein	AHNAKRS|PM227	11
TLR1	7096	toll like receptor 1	CD281|TIL|TIL. LPRS5|rsc786	4
TK2	7084	thymidine kinase 2	MTDPS2|MTTK|PEOB3|SCA31	16
PRDX2	7001	peroxiredoxin 2	HEL-S-2a|NKEF-B|NKEFB|PRP|PRX2|PRXII|PTX1|TDPX1|TPX1|TSA	19
MX2	4600	MX dynamin like GTPase 2	MXB	21
FGF2	2247	fibroblast growth factor 2	BFGF|FGF-2|FGFB|HBGF-2	4
FGA	2243	fibrinogen alpha chain	Fib2	4
TCF7L2	6934	transcription factor 7 like 2	TCF-4|TCF4	10
F2RL1	2150	F2R like trypsin receptor 1	GPR11|PAR2	5
TKFC	26007	triokinase and FMN cyclase	DAK|NET45|TKFCD	11
MSR1	4481	macrophage scavenger receptor 1	CD204|SCARA1|SR-A|SR-AI|SR-AII|SR-AIII|SRA|phSR1|phSR2	8
NFKBIZ	64332	NFKB inhibitor zeta	IKBZ|INAP|MAIL	3
LMBR1	64327	limb development membrane protein 1	ACHP|C7orf2|DIF14|LSS|PPD2|THYP|TPT|ZRS	7
EPPIN	57119	epididymal peptidase inhibitor	CT71|CT72|SPINLW1|WAP7|WFDC7|dJ461P17.2	20
SRC	6714	SRC proto-oncogene, non-receptor tyrosine kinase	ASV|SRC1|THC6|c-SRC|p60-Src	20

MPO	4353	myeloperoxidase	-	17
ELAVL1	1994	ELAV like RNA binding protein 1	ELAV1|HUR|Hua|MelG	19
ROBO3	64221	roundabout guidance receptor 3	HGPPS|HGPPS1|HGPS|RBIG1|RIG1	11
SP1	6667	Sp1 transcription factor	-	12
SOD1	6647	superoxide dismutase 1	ALS|ALS1|HEL-S-44|IPOA|SOD|STAHP|hSod1|homodimer	21
PDF	64146	peptide deformylase, mitochondrial	-	16
DLL4	54567	delta like canonical Notch ligand 4	AOS6|delta4|hdelta2	15
ECD	11319	ecdysoneless cell cycle regulator	GCR2|HSGT1|SGT1	10
SLC11A1	6556	solute carrier family 11 member 1	LSH|NRAMP|NRAMP1	2
DMBT1	1755	deleted in malignant brain tumors 1	GP340|SAG|SALSA|muclin	10
STING1	340061	stimulator of interferon response cGAMP interactor 1	ERIS|MITA|MPYS|NET23|SAVI|STING|STING-beta|TMEM173|hMITA|hSTING	5
SKIV2L	6499	Ski2 like RNA helicase	170A|DDX13|HLP|SKI2|SKI2W|SKIV2|SKIV2L1|THES2	6
SEMG2	6407	semenogelin 2	SGII	20
LTA	4049	lymphotoxin alpha	LT|TNFB|TNFSF1|TNLG1E	6
DES	1674	desmin	CDCD3|CSM1|CSM2|LGMD1D|LGMD1E|LGMD2R	2
DCK	1633	deoxycytidine kinase	-	4
DAXX	1616	death domain associated protein	BING2|DAP6|EAP1|SMIM40	6
TNFRSF10	8797	TNF receptor superfamily member 10a	APO2|CD261|DR4|TRAILR-1|TRAILR1	8

TNFRSF10	8795	TNF receptor superfamily member 10b	CD262|DR5|KILLER|KILLER/DR5|TRAIL-R2|TRAILR2|TRICK2|TRICK2A|TRICK2B|TRICKB|ZTNFR9	8
EED	8726	embryonic ectoderm development	COGIS|HEED|WAIT1	11
CCL4	6351	C-C motif chemokine ligand 4	ACT2|AT744.1|G-26|HC21|LAG-1|LAG1|MIP-1-beta|MIP1B|MIP1B1|SCYA2|SCYA4	17
LIMS1	3987	LIM zinc finger domain containing 1	PINCH|PINCH-1|PINCH1	2
LALBA	3906	lactalbumin alpha	LYZG	12
APOBEC3l	164668	apolipoprotein B mRNA editing enzyme catalytic subunit 3H	A3H|ARP-10|ARP10	22
TMPRSS6	164656	transmembrane serine protease 6	IRIDA|MT2	22
SPINK5	11005	serine peptidase inhibitor Kazal type 5	LEKTI|LETKI|NETS|NS|VAKTI	5
MARCO	8685	macrophage receptor with collagenous structure	SCARA2|SR-A6	2
BECN1	8678	beclin 1	ATG6|VPS30|beclin 1	17
TNFSF11	8600	TNF superfamily member 11	CD254|ODF|OPGL|OPTB2|RANKL|TNLG6B|TRANCE|hRANKL2|sOdf	13
KNG1	3827	kininogen 1	BDK|BK|HMWK|KNG	3
CSK	1445	C-terminal Src kinase	-	15
KLRK1	22914	killer cell lectin like receptor K1	CD314|D12S2489E|KLR|NKG2-D|NKG2D	12
KCNH2	3757	potassium voltage-gated channel subfamily H member 2	ERG-1|ERG1|H-ERG|HERG|HERG1|Kv11.1|LQT2|SQT1	7

JUND	3727	JunD proto-oncogene, AP-1 transcription factor subunit	AP-1	19
JAK1	3716	Janus kinase 1	JAK1A|JAK1B|JTK3	1
CREB1	1385	cAMP responsive element binding protein 1	CREB|CREB-1	2
CLDN4	1364	claudin 4	CPE-R|CPER|CPETR|CPETR1|WBSCR8|hCPE-R	7
CCL28	56477	C-C motif chemokine ligand 28	CCK1|MEC|SCYA28	5
RNASE3	6037	ribonuclease A family member 3	ECP|RAF1|RNS3	14
RN7SL1	6029	RNA component of signal recognition particle 7SL1	7L1a|7SL|RN7SL|RNSRP1	14
IRF7	3665	interferon regulatory factor 7	IMD39|IRF-7|IRF-7H|IRF7A|IRF7B|IRF7C|IRF7H	11
IREB2	3658	iron responsive element binding protein 2	ACO3|IRE-BP 2|IRE-BP2|IRP2|IRP2AD|NDCAMA	15
ILK	3611	integrin linked kinase	HEL-S-28|ILK-1|ILK-2|P59|p59ILK	11
IL18	3606	interleukin 18	IGIF|IL-18|IL-1g|IL1F4	11
IL17A	3605	interleukin 17A	CTLA-8|CTLA8|IL-17|IL-17A|IL17	6
LTB4R	1241	leukotriene B4 receptor	BLT1|BLTR|CMKRL1|GPR16|LTB4R1|LTBR1|P2RY7|P2Y7	14
APOBEC3A	200315	apolipoprotein B mRNA editing enzyme catalytic subunit 3A	A3A|ARP3|PHRBN|bK150C2.1	22
MASP2	10747	mannan binding lectin serine peptidase 2	MAP19|MASP-2|MASP1P1|sMAP	1
TRIM27	5987	tripartite motif containing 27	RFP|RNF76	6

RELA	5970	RELA proto-oncogene, NF-kB subunit	CMCU|NFKB3|p65	11
IL7R	3575	interleukin 7 receptor	CD127|CDW127|IL-7R-alpha|IL7RA|ILRA	5
IL1A	3552	interleukin 1 alpha	IL-1 alpha|IL-1A|IL1|IL1-ALPHA|IL1F1	2
PTX3	5806	pentraxin 3	TNFAIP5|TSG-14	3
IFNAR2	3455	interferon alpha and beta receptor subunit 2	IFN-R|IFN-alpha-REC|IFNABR|IFNARB|IMD45	21
IFN1@	3438	-	IFNA	9
SYTL1	84958	synaptotagmin like 1	JFC1|SLP1	1
APOBEC3(	27350	apolipoprotein B mRNA editing enzyme catalytic subunit 3C	A3C|APOBEC1L|ARDC2|ARDC4|ARP5|PBI|bK150C2.3	22
DDX17	10521	DEAD-box helicase 17	P72|RH70	22
PTGS2	5743	prostaglandin-endoperoxide synthase 2	COX-2|COX2|GRIPGHS|PGG/HS|PGHS-2|PHS-2|hCox-2	1
HTR1A	3350	5-hydroxytryptamine receptor 1A	5-HT-1A|5-HT1A|5HT1a|ADRB2RL1|ADRBRL1|G-21|PFMCD	5
SEPTIN7	989	septin 7	CDC10|CDC3|NBLA02942|SEPT7|SEPT7A	7
CD40LG	959	CD40 ligand	CD154|CD40L|HIGM1|IGM|IMD3|T-BAM|TNFSF5|TRAP|gp39|hCD40L	X
CD14	929	CD14 molecule	-	5
CD8A	925	CD8a molecule	CD8|Leu2|p32	2
CD4	920	CD4 molecule	CD4mut	12
MASP1	5648	mannan binding lectin serine peptidase 1	3MC1|CRARF|CRARF1|MAP1|MASP|MASP3|MAp44|PRSS5|RaRF	3
PROC	5624	protein C, inactivator of coagulation factors Va and VIIIa	APC|PC|PROC1|THPH3|THPH4	2
MAP2K2	5605	mitogen-activated protein kinase kinase 2	CFC4|MAPKK2|MEK2|MKK2|PRKMK2	19

MAP2K1	5604	mitogen-activated protein kinase kinase 1	CFC3|MAPKK1|MEK1|MKK1|PRKMK1	15
HRG	3273	histidine rich glycoprotein	HPRG|HRGP|THPH11	3
NDRG1	10397	N-myc downstream regulated 1	CAP43|CMT4D|DRG-1|DRG1|GC4|HMSNL|NDR1|NMSL|PROXY1|RIT42|RTP|TARG1|TDD5	8
IRF9	10379	interferon regulatory factor 9	IRF-9|ISGF3|ISGF3G|p48	14
TRIM22	10346	tripartite motif containing 22	GPSTAF50|RNF94|STAF50	11
LANCL1	10314	LanC like 1	GPR69A|p40	2
PPP4C	5531	protein phosphatase 4 catalytic subunit	PP-X|PP4|PP4C|PPH3|PPP4|PPX	16
HMOX1	3162	heme oxygenase 1	HMOX1D|HO-1|HSP32|bK286B10	22
HMGB1	3146	high mobility group box 1	HMG-1|HMG1|HMG3|SBP-1	13
HLA-B	3106	major histocompatibility complex, class I, B	AS|B-4901|HLAB	6
RNASE7	84659	ribonuclease A family member 7	RAE1	14
ABCC4	10257	ATP binding cassette subfamily C member 4	MOAT-B|MOATB|MRP4	13
HGF	3082	hepatocyte growth factor	DFNB39|F-TCF|HGFB|HPTA|SF	7
HDAC1	3065	histone deacetylase 1	GON-10|HD1|KDAC1|RPD3|RPD3L1	1
IFNLR1	163702	interferon lambda receptor 1	CRF2/12|IFNLR|IL-28R1|IL28RA|LICR2	1
PLSCR1	5359	phospholipid scramblase 1	MMTRA1B	3
B2M	567	beta-2-microglobulin	IMD43	15
BACH2	60468	BTB domain and CNC homolog 2	BTBD25|IMD60	6

TANK	10010	TRAF family member associated NFKB activator	I-TRAF|ITRAF|TRAF2	2
PIK3CG	5294	phosphatidylinositol-4,5-bisphosphate 3-kinase catalytic subunit gamma	PI3CG|PI3K|PI3Kgamma|PIK3|p110gamma|p120-PI3K	7
ARRB1	408	arrestin beta 1	ARB1|ARR1	11
RSAD2	91543	radical S-adenosyl methionine domain containing 2	2510004L01Rik|cig33|cig5|vig1	2
STAB2	55576	stabilin 2	FEEL2|FELE-2|FELL2|FEX2|HARE|SCARH1	12
TBK1	29110	TANK binding kinase 1	FTDALS4|IIAE8|NAK|T2K	12
PDYN	5173	prodynorphin	ADCA|PENKB|SCA23	20
PDGFRB	5159	platelet derived growth factor receptor beta	CD140B|IBGC4|IMF1|JTK12|KOGS|PDGFR|PDGFR-1|PDGFR1|PENTT	5
PDCD1	5133	programmed cell death 1	CD279|PD-1|PD1|SLEB2|hPD-1|hPD-l|hSLE1	2
PCSK2	5126	proprotein convertase subtilisin/kexin type 2	NEC 2|NEC-2|NEC2|PC2|SPC2	20
PCSK1	5122	proprotein convertase subtilisin/kexin type 1	BMIQ12|NEC1|PC1|PC3|SPC3	5
ARG2	384	arginase 2	-	14
AQP9	366	aquaporin 9	AQP-9|HsT17287|SSC1|T17287	15
FASLG	356	Fas ligand	ALPS1B|APT1LG1|APTL|CD178|CD95-L|CD95L|FASL|TNFSF6|TNLG1A	1
APOH	350	apolipoprotein H	B2G1|B2GP1|BG	17
BIRC5	332	baculoviral IAP repeat containing 5	API4|EPR-1	17
ANXA6	309	annexin A6	ANX6|CBP68|CPB-II|p68|p70	5

IL22	50616	interleukin 22	IL-21|IL-22|IL-D110|IL-TIF|ILTIF|TIFIL-23|TIFa|zcyto18	12
VTN	7448	vitronectin	V75|VN|VNT	17
VIM	7431	vimentin	-	10
VCAM1	7412	vascular cell adhesion molecule 1	CD106|INCAM-100	1
PRDX1	5052	peroxiredoxin 1	MSP23|NKEF-A|NKEFA|PAG|PAGA|PAGB|PRX1|PRXI|TDPX2	1
GFAP	2670	glial fibrillary acidic protein	ALXDRD	17
GBP2	2634	guanylate binding protein 2	-	1
ALB	213	albumin	HSA|PRO0883|PRO0903|PRO1341	4
SLC29A3	55315	solute carrier family 29 member 3	ENT3|HCLAP|HJCD|PHID	10
OAS1	4938	2'-5'-oligoadenylate synthetase 1	E18/E16|IFI-4|OIAS|OIASI	12
AGER	177	advanced glycosylation end-product specific receptor	RAGE|SCARJ1	6
UNC93B1	81622	unc-93 homolog B1, TLR signaling regulator	IIAE1|UNC93|UNC93B|Unc-93B1	11
TNFSF4	7292	TNF superfamily member 4	CD134L|CD252|GP34|OX-40L|OX4OL|TNLG2B|TXGP1	1
NOS1	4842	nitric oxide synthase 1	IHPS1|N-NOS|NC-NOS|NOS|bNOS|nNOS	12
ACTG1	71	actin gamma 1	ACT|ACTG|DFNA20|DFNA26|HEL-176	17
ACTA1	58	actin alpha 1, skeletal muscle	ACTA|ASMA|CFTD|CFTD1|CFTDM|MPFD|NEM1|NEM2|NEM3|SHPM	1
ACO1	48	aconitase 1	ACONS|HEL60|IREB1|IREBP|IREBP1|IRP1	9
SERPINA3	12	serpin family A member 3	AACT|ACT|GIG24|GIG25	14

CXCR1	3577	C-X-C motif chemokine receptor 1	C-C|C-C-CKR-1|CD128|CD181|CDw128a|CKR-1|CMKAR1|IL8R1|IL8RA|IL8RBA	2
CCL15	6359	C-C motif chemokine ligand 15	HCC-2|HMRP-2B|LKN-1|LKN1|MIP-1 delta|MIP-1D|MIP-5|MRP-2B|NCC-3|NCC3|SCYA15|SCYL3|SY15	17
CCL14	6358	C-C motif chemokine ligand 14	CC-1|CC-3|CKB1|HCC-1|HCC-1(1-74)|HCC-1/HCC-3|HCC-3|MCIF|NCC-2|NCC2|SCYA14|SCYL2|SY14	17
CCL4	6351	C-C motif chemokine ligand 4	ACT2|AT744.1|G-26|HC21|LAG-1|LAG1|MIP-1-beta|MIP1B|MIP1B1|SCYA2|SCYA4	17
CCL16	6360	C-C motif chemokine ligand 16	CKb12|HCC-4|ILINCK|LCC-1|LEC|LMC|Mtn-1|NCC-4|NCC4|SCYA16|SCYL4	17
CCL19	6363	C-C motif chemokine ligand 19	CKb11|ELC|MIP-3b|MIP3B|SCYA19	9
CCL13	6357	C-C motif chemokine ligand 13	CKb10|MCP-4|NCC-1|NCC1|SCYA13|SCYL1	17
CCL18	6362	C-C motif chemokine ligand 18	AMAC-1|AMAC1|CKb7|DC-CK1|DCCK1|MIP-4|PARC|SCYA18	17
CCL17	6361	C-C motif chemokine ligand 17	A-152E5.3|ABCD-2|SCYA17|TARC	16
CCL26	10344	C-C motif chemokine ligand 26	IMAC|MIP-4a|MIP-4alpha|SCYA26|TSC-1	7

CCL22	6367	C-C motif chemokine ligand 22	A-152E5.1|ABCD-1|DC/B-CK|MDC|SCYA22|STCP-1	16
CCR3	1232	C-C motif chemokine receptor 3	C C CKR3|CC-CKR-3|CD193|CKR 3|CKR3|CMKBR3	3
CCL28	56477	C-C motif chemokine ligand 28	CCK1|MEC|SCYA28	5
CCL4L1	388372	C-C motif chemokine ligand 4 like 1	AT744.2|CCL4L|LAG-1|LAG1|MIP-1-beta|SCYA4L|SCYA4L1|SCYA4L2	17
ACKR2	1238	atypical chemokine receptor 2	CCBP2|CCR10|CCR9|CMKBR9|D6|hD6	3
CCR7	1236	C-C motif chemokine receptor 7	BLR2|CC-CKR-7|CCR-7|CD197|CDw197|CMKBR7|EBI1	17
CCL27	10850	C-C motif chemokine ligand 27	ALP|CTACK|CTAK|ESKINE|ILC|PESKY|SCYA27	9
CCR8	1237	C-C motif chemokine receptor 8	CC-CKR-8|CCR-8|CDw198|CKRL1|CMKBR8|CMKBRL2|CY6|GPRCY6|TER1	3
ACKR4	51554	atypical chemokine receptor 4	CC-CKR-11|CCBP2|CCR-11|CCR10|CCR11|CCRL1|CCX CKR|CCX-CKR|CKR-11|PPR1|VSHK1	3
CCR10	2826	C-C motif chemokine receptor 10	GPR2	17
CCL2	6347	C-C motif chemokine ligand 2	GDCF-2|HC11|HSMCR30|MCAF|MCP-1|MCP1|SCYA2|SMC-CF	17
CCL21	6366	C-C motif chemokine ligand 21	6Ckine|CKb9|ECL|SCYA21|SLC|TCA4	9
CCL7	6354	C-C motif chemokine ligand 7	FIC|MARC|MCP-3|MCP3|NC28|SCYA6|SCYA7	17

CCL5	6352	C-C motif chemokine ligand 5	D17S136E|RANTES|SCYA5|SIS-delta|SISd|TCP228|eoCP	17
CCL3	6348	C-C motif chemokine ligand 3	G0S19-1|LD78ALPHA|MIP-1-alpha|MIP1A|SCYA3	17
CCL20	6364	C-C motif chemokine ligand 20	CKb4|Exodus|LARC|MIP-3-alpha|MIP-3a|MIP3A|SCYA20|ST38	2
CCL11	6356	C-C motif chemokine ligand 11	SCYA11	17
CCR5	1234	C-C motif chemokine receptor 5	CC-CKR-5|CCCKR5|CCR-5|CD195|CKR-5|CKR5|CMKBR5|IDDM22	3
CCL23	6368	C-C motif chemokine ligand 23	CK-BETA-8|CKb8|Ckb-8|Ckb-8-1|MIP-3|MIP3|MPIF-1|SCYA23|hmrp-2a	17
CCL25	6370	C-C motif chemokine ligand 25	Ckb15|SCYA25|TECK	19
CCL1	6346	C-C motif chemokine ligand 1	I-309|P500|SCYA1|SISe|TCA3	17
CCL3L3	414062	C-C motif chemokine ligand 3 like 3	464.2|D17S1718|G0S19-2|LD78|LD78BETA|SCYA3L|SCYA3L1	17
CCL4L2	9560	C-C motif chemokine ligand 4 like 2	AT744.2|CCL4L|SCYA4L|SCYQ4L2	17
CXCL12	6387	C-X-C motif chemokine ligand 12	IRH|PBSF|SCYB12|SDF1|TLSF|TPAR1	10
XCL1	6375	X-C motif chemokine ligand 1	ATAC|LPTN|LTN|SCM-1|SCM-1a|SCM1|SCM1A|SCYC1	1
CCL8	6355	C-C motif chemokine ligand 8	HC14|MCP-2|MCP2|SCYA10|SCYA8	17

CCL3L1	6349	C-C motif chemokine ligand 3 like 1	464.2|D17S1718|G0S19-2|LD78|LD78-beta(1-70)|LD78BETA|MIP1AP|SCYA3L|SCYA3L1	17
CCR1	1230	C-C motif chemokine receptor 1	CD191|CKR-1|CKR1|CMKBR1|HM145|MIP1aR|SCYAR1	3
CCL24	6369	C-C motif chemokine ligand 24	Ckb-6|MPIF-2|MPIF2|SCYA24	7
XCL2	6846	X-C motif chemokine ligand 2	SCM-1b|SCM1B|SCYC2	1
CXCL1	2919	C-X-C motif chemokine ligand 1	FSP|GRO1|GROa|MGSA|MGSA-a|NAP-3|SCYB1	4
CXCL10	3627	C-X-C motif chemokine ligand 10	C7|IFI10|INP10|IP-10|SCYB10|crg-2|gIP-10|mob-1	4
CXCR4	7852	C-X-C motif chemokine receptor 4	CD184|D2S201E|FB22|HM89|HSY3RR|LAP-3|LAP3|LCR1|LESTR|NPY3R|NPYR|NPYRL|NPYY3R|WHIM|WHIMS	2
CXCL2	2920	C-X-C motif chemokine ligand 2	CINC-2a|GRO2|GROb|MGSA-b|MIP-2a|MIP2|MIP2A|SCYB2	4
CXCR6	10663	C-X-C motif chemokine receptor 6	BONZO|CD186|STRL33|TYMSTR	3
CCR4	1233	C-C motif chemokine receptor 4	CC-CKR-4|CD194|CKR4|CMKBR4|ChemR13|HGCN:14099|K5-5	3
CXCL11	6373	C-X-C motif chemokine ligand 11	H174|I-TAC|IP-9|IP9|SCYB11|SCYB9B|b-R1	4
TAFA5	25817	TAFA chemokine like family member 5	FAM19A5|QLLK5208|TAFA-5|UNQ5208	22
TAFA3	284467	TAFA chemokine like family member 3	FAM19A3|TAFA-3	1

TAFA4	151647	TAFA chemokine like family member 4	FAM19A4|TAFA-4	3
TAFA1	407738	TAFA chemokine like family member 1	FAM19A1|TAFA-1	3
TAFA2	338811	TAFA chemokine like family member 2	FAM19A2|TAFA-2	12
CCL15-CC	348249	CCL15-CCL14 readthrough (NMD candidate)	CCL15|HCC-2|LKN-1|MIP-5|MIP5|Mrp-2b|NCC-3|NCC3|SCYA15	17
IL6	3569	interleukin 6	BSF-2|BSF2|CDF|HGF|HSF|IFN-beta-2|IFNB2|IL-6	7
TNF	7124	tumor necrosis factor	DIF|TNF-alpha|TNFA|TNFSF2|TNLG1F	6
IL1B	3553	interleukin 1 beta	IL-1|IL1-BETA|IL1F2|IL1beta	2
IL18	3606	interleukin 18	IGIF|IL-18|IL-1g|IL1F4	11
PTK2B	2185	protein tyrosine kinase 2 beta	CADTK|CAKB|FADK2|FAK2|PKB|PTK|PYK2|RAFTK	8
VEGFA	7422	vascular endothelial growth factor A	MVCD1|VEGF|VPF	6
IL4	3565	interleukin 4	BCGF-1|BCGF1|BSF-1|BSF1|IL-4	5
CDH1	999	cadherin 1	Arc-1|BCDS1|CD324|CDHE|ECAD|LCAM|UVO	16
CD40	958	CD40 molecule	Bp50|CDW40|TNFRSF5|p50	20
DEFB103B	55894	defensin beta 103B	BD-3|DEFB-3|DEFB103|DEFB3|HBD-3|HBD3|HBP-3|HBP3	8
F2RL1	2150	F2R like trypsin receptor 1	GPR11|PAR2	5
MMP9	4318	matrix metallopeptidase 9	CLG4B|GELB|MANDP2|MMP-9	20

LTBP1	4052	latent transforming growth factor beta binding protein 1	-	2
DEFB4A	1673	defensin beta 4A	BD-2|DEFB-2|DEFB102|DEFB2|DEFB4|HBD-2|SAP1	8
TNFSF10	8743	TNF superfamily member 10	APO2L|Apo-2L|CD253|TL2|TNLG6A|TRAIL	3
IL13	3596	interleukin 13	IL-13|P600	5
IL10	3586	interleukin 10	CSIF|GVHDS|IL-10|IL10A|TGIF	1
IL2	3558	interleukin 2	IL-2|TCGF|lymphokine	4
PPARG	5468	peroxisome proliferator activated receptor gamma	CIMT1|GLM1|NR1C3|PPARG1|PPARG2|PPARG5|PPARgamma	3
FGR	2268	FGR proto-oncogene, Src family tyrosine kinase	SRC2|c-fgr|c-src2|p55-Fgr|p55c-fgr|p58-Fgr|p58c-fgr	1
MIF	4282	macrophage migration inhibitory factor	GIF|GLIF|MMIF	22
CRP	1401	C-reactive protein	PTX1	1
JAK2	3717	Janus kinase 2	JTK10|THCYT3	9
IL1A	3552	interleukin 1 alpha	IL-1 alpha|IL-1A|IL1|IL1-ALPHA|IL1F1	2
PTK2	5747	protein tyrosine kinase 2	FADK|FAK|FAK1|FRNK|PPP1R71|p125FAK|pp125FAK	8
PTGDR	5729	prostaglandin D2 receptor	AS1|ASRT1|DP|DP1|PTGDR1	14
CD86	942	CD86 molecule	B7-2|B7.2|B70|CD28LG2|LAB72	3
HCK	3055	HCK proto-oncogene, Src family tyrosine kinase	JTK9|p59Hck|p61Hck	20
ARRB1	408	arrestin beta 1	ARB1|ARR1	11
GNAI1	2770	G protein subunit alpha i1	Gi	7
VDR	7421	vitamin D receptor	NR1I1|PPP1R163	12
OLR1	4973	oxidized low density lipoprotein receptor 1	CLEC8A|LOX1|LOXIN|SCARE1|SLOX1	12

GRK2	156	G protein-coupled receptor kinase 2	ADRBK1|BARK1|BETA-ARK1	11
TXK	7294	TXK tyrosine kinase	BTKL|PSCTK5|PTK4|RLK|TKL	4
RNASE2	6036	ribonuclease A family member 2	EDN|RAF3|RNS2	14
CD79A	973	CD79a molecule	IGA|MB-1	19
CD79B	974	CD79b molecule	AGM6|B29|IGB	17
LYN	4067	LYN proto-oncogene, Src family tyrosine kinase	JTK8|p53Lyn|p56Lyn	8
SYK	6850	spleen associated tyrosine kinase	p72-Syk	9
BTK	695	Bruton tyrosine kinase	AGMX1|AT|ATK|BPK|IGHD3|IMD1|PSCTK1|XLA	X
BLNK	29760	B cell linker	AGM4|BASH|BLNK-S|LY57|SLP-65|SLP65|bca	10
VAV3	10451	vav guanine nucleotide exchange factor 3	-	1
VAV1	7409	vav guanine nucleotide exchange factor 1	VAV	19
VAV2	7410	vav guanine nucleotide exchange factor 2	VAV-2	9
RAC1	5879	Rac family small GTPase 1	MIG5|MRD48|Rac-1|TC-25|p21-Rac1	7
RAC2	5880	Rac family small GTPase 2	EN-7|Gx|HSPC022|p21-Rac2	22
RAC3	5881	Rac family small GTPase 3	-	17
PPP3CA	5530	protein phosphatase 3 catalytic subunit alpha	ACCIID|CALN|CALNA|CALNA1|CCN1|CNA1|IECEE|IECEE1|PPP2B	4
PPP3CB	5532	protein phosphatase 3 catalytic subunit beta	CALNA2|CALNB|CNA2|PP2Bbeta	10
PPP3CC	5533	protein phosphatase 3 catalytic subunit gamma	CALNA3|CNA3|PP2Bgamma	8
CHP1	11261	calcineurin like EF-hand protein 1	CHP|SLC9A1BP|SPAX9|Sid470p|p22|p24	15

PPP3R1	5534	protein phosphatase 3 regulatory subunit B, alpha	CALNB1|CNB|CNB1	2
PPP3R2	5535	protein phosphatase 3 regulatory subunit B, beta	PPP3RL	9
CHP2	63928	calcineurin like EF-hand protein 2	-	16
NFAT5	10725	nuclear factor of activated T cells 5	NF-AT5|NFATL1|NFATZ|OREBP|TONEBP	16
NFATC1	4772	nuclear factor of activated T cells 1	NF-ATC|NF-ATc1.2|NFAT2|NFATc	18
NFATC2	4773	nuclear factor of activated T cells 2	NFAT1|NFATP	20
NFATC3	4775	nuclear factor of activated T cells 3	NF-AT4c|NFAT4|NFATX	16
NFATC4	4776	nuclear factor of activated T cells 4	NF-AT3|NF-ATC4|NFAT3	14
HRAS	3265	HRas proto-oncogene, GTPase	C-BAS/HAS|C-H-RAS|C-HA-RAS1|CTLO|H-RASIDX|HAMSV|HRAS1|RASH1|p21ras	11
KRAS	3845	KRAS proto-oncogene, GTPase	'C-K-RAS|C-K-RAS|CFC2|K-RAS2A|K-RAS2B|K-RAS4A|K-RAS4B|K-Ras|K-Ras 2|KI-RAS|KRAS1|KRAS2|NS|NS3|OES|RALD|RASK2|c-Ki-ras|c-Ki-ras2	12
NRAS	4893	NRAS proto-oncogene, GTPase	ALPS4|CMNS|N-ras|NCMS|NRAS1|NS6	1
FOS	2353	Fos proto-oncogene, AP-1 transcription factor subunit	AP-1|C-FOS|p55	14
JUN	3725	Jun proto-oncogene, AP-1 transcription factor subunit	AP-1|AP1|c-Jun|cJUN|p39	1

CARD11	84433	caspase recruitment domain family member 11	BENTA|BIMP3|CARMA1|IMD11|IMD11A|PPBL	7
BCL10	8915	BCL10 immune signaling adaptor	CARMEN|CIPER|CLAP|IMD37|c-E10|mE10	1
MALT1	10892	MALT1 paracaspase	IMD12|MLT|MLT1|PCASP1	18
CHUK	1147	component of inhibitor of nuclear factor kappa B kinase complex	IKBKA|IKK-alpha|IKK1|IKKA|NFKBIKA|TCF16	10
IKBKB	3551	inhibitor of nuclear factor kappa B kinase subunit beta	IKK-beta|IKK2|IKKB|IMD15|IMD15A|IMD15B|NFKBIKB	8
IKBKG	8517	inhibitor of nuclear factor kappa B kinase regulatory subunit gamma	AMCBX1|EDAID1|FIP-3|FIP3|Fip3p|IKK-gamma|IKKAP1|IKKG|IMD33|IP|IP1|IP2|IPD2|NEMO|ZC2HC9	X
NFKB1	4790	nuclear factor kappa B subunit 1	CVID12|EBP-1|KBF1|NF-kB|NF-kB1|NF-kappa-B1|NF-kappaB|NF-kappabeta|NFKB-p105|NFKB-p50|NFkappaB	4
RELA	5970	RELA proto-oncogene, NF-kB subunit	CMCU|NFKB3|p65	11
NFKBIA	4792	NFKB inhibitor alpha	EDAID2|IKBA|MAD-3|NFKBI	14
NFKBIB	4793	NFKB inhibitor beta	IKBB|TRIP9	19
NFKBIE	4794	NFKB inhibitor epsilon	IKBE	6
CD81	975	CD81 molecule	CVID6|S5.7|TAPA1|TSPAN28	11
CD19	930	CD19 molecule	B4|CVID3	16
CR2	1380	complement C3d receptor 2	C3DR|CD21|CR|CVID7|SLEB9	1
PIK3R5	23533	phosphoinositide-3-kinase regulatory subunit 5	F730038I15Rik|FOAP-2|P101-PI3K|p101	17
PIK3R1	5295	phosphoinositide-3-kinase regulatory subunit 1	AGM7|GRB1|IMD36|p85|p85-ALPHA	5

PIK3R2	5296	phosphoinositide-3-kinase regulatory subunit 2	MPPH|MPPH1|P85B|p85|p85-BETA	19
PIK3R3	8503	phosphoinositide-3-kinase regulatory subunit 3	p55|p55-GAMMA|p55PIK	1
PIK3CA	5290	phosphatidylinositol-4,5-bisphosphate 3-kinase catalytic subunit alpha	CLAPO|CLOVE|CWS5|MCAP|MCM|MCMTC|PI3K|PI3K-alpha|p110-alpha	3
PIK3CB	5291	phosphatidylinositol-4,5-bisphosphate 3-kinase catalytic subunit beta	P110BETA|PI3K|PI3KBETA|PIK3C1	3
PIK3CD	5293	phosphatidylinositol-4,5-bisphosphate 3-kinase catalytic subunit delta	APDS|IMD14|P110DELTA|PI3K|p110D	1
PIK3CG	5294	phosphatidylinositol-4,5-bisphosphate 3-kinase catalytic subunit gamma	PI3CG|PI3K|PI3Kgamma|PIK3|p110gamma|p120-PI3K	7
AKT3	10000	AKT serine/threonine kinase 3	MPPH|MPPH2|PKB-GAMMA|PKBG|PRKBG|RAC-PK-gamma|RAC-gamma|STK-2	1
AKT1	207	AKT serine/threonine kinase 1	AKT|CWS6|PKB|PKB-ALPHA|PRKBA|RAC|RAC-ALPHA	14
AKT2	208	AKT serine/threonine kinase 2	HIHGHH|PKBB|PKBBETA|PRKBB|RAC-BETA	19
GSK3B	2932	glycogen synthase kinase 3 beta	-	3
INPP5D	3635	inositol polyphosphate-5-phosphatase D	SHIP|SHIP-1|SHIP1|SIP-145|hp51CN|p150Ship	2
CD22	933	CD22 molecule	SIGLEC-2|SIGLEC2	19
CD72	971	CD72 molecule	CD72b|LYB2	9
PTPN6	5777	protein tyrosine phosphatase non-receptor type 6	HCP|HCPH|HPTP1C|PTP-1C|SH-PTP1|SHP-1|SHP-1L|SHP1	12

LILRB3	11025	leukocyte immunoglobulin like receptor B3	CD85A|HL9|ILT-5|ILT5|LILRA6|LIR-3|LIR3|PIR-B|PIRB	19
FCGR2B	2213	Fc fragment of IgG receptor IIb	CD32|CD32B|FCG2|FCGR2|FCGR2C|FcRII-c|IGFR2	1
RASGRP3	25780	RAS guanyl releasing protein 3	GRP3	2
PLCG2	5336	phospholipase C gamma 2	APLAID|FCAS3|PLC-IV|PLC-gamma-2	16
PRKCB	5579	protein kinase C beta	PKC-beta|PKCB|PKCI(2)|PKCbeta|PRKCB1|PRKCB2	16
IFITM1	8519	interferon induced transmembrane protein 1	9-27|CD225|DSPA2a|IFI17|LEU13	11
IGH	3492	immunoglobulin heavy locus	IGD1|IGH.1@|IGH@|IGHD@|IGHDY1|IGHJ|IGHJ@|IGHV|IGHV@	14
IGHA1	3493	immunoglobulin heavy constant alpha 1	IgA1	14
IGHA2	3494	immunoglobulin heavy constant alpha 2 (A2m marker)	-	14
IGHD	3495	immunoglobulin heavy constant delta	-	14
IGHD1-1	28510	immunoglobulin heavy diversity 1-1	IGHD11	14
IGHD1-14	28508	immunoglobulin heavy diversity 1-14 (non-functional)	DM2|IGHD114	14
IGHD1-20	28507	immunoglobulin heavy diversity 1-20	IGHD120	14
IGHD1-26	28506	immunoglobulin heavy diversity 1-26	IGHD126	14
IGHD1-7	28509	immunoglobulin heavy diversity 1-7	DM1|IGHD17	14
IGHD2-15	28503	immunoglobulin heavy diversity 2-15	D2|IGHD215	14
IGHD2-2	28505	immunoglobulin heavy diversity 2-2	IGHD22	14

IGHD2-21	28502	immunoglobulin heavy diversity 2-21	IGHD221	14
IGHD2-8	28504	immunoglobulin heavy diversity 2-8	DLR1|IGHD28	14
IGHD3-10	28499	immunoglobulin heavy diversity 3-10	DXP'1|IGHD310	14
IGHD3-16	28498	immunoglobulin heavy diversity 3-16	IGHD316	14
IGHD3-22	28497	immunoglobulin heavy diversity 3-22	IGHD322	14
IGHD3-3	28501	immunoglobulin heavy diversity 3-3	DXP4|IGHD33	14
IGHD3-9	28500	immunoglobulin heavy diversity 3-9	DXP1|IGHD39	14
IGHD4-11	28495	immunoglobulin heavy diversity 4-11 (non-functional)	DA1|IGHD411	14
IGHD4-17	28494	immunoglobulin heavy diversity 4-17	IGHD417	14
IGHD4-23	28493	immunoglobulin heavy diversity 4-23 (non-functional)	IGHD423	14
IGHD4-4	28496	immunoglobulin heavy diversity 4-4	DA4|IGHD44	14
IGHD5-12	28491	immunoglobulin heavy diversity 5-12	DK1|IGHD512	14
IGHD5-18	28490	immunoglobulin heavy diversity 5-18	IGHD518	14
IGHD5-24	28489	immunoglobulin heavy diversity 5-24 (non-functional)	IGHD524	14
IGHD5-5	28492	immunoglobulin heavy diversity 5-5	DK4|IGHD55	14
IGHD6-13	28487	immunoglobulin heavy diversity 6-13	DN1|IGHD613	14
IGHD6-19	28486	immunoglobulin heavy diversity 6-19	IGHD619	14
IGHD6-25	28485	immunoglobulin heavy diversity 6-25	IGHD625	14
IGHD6-6	28488	immunoglobulin heavy diversity 6-6	D(N4)|IGHD66	14

IGHD7-27	28484	immunoglobulin heavy diversity 7-27	DHQ52|IGHD727	14
IGHE	3497	immunoglobulin heavy constant epsilon	IgE	14
IGHG1	3500	immunoglobulin heavy constant gamma 1 (G1m marker)	-	14
IGHG2	3501	immunoglobulin heavy constant gamma 2 (G2m marker)	-	14
IGHG3	3502	immunoglobulin heavy constant gamma 3 (G3m marker)	IgG3	14
IGHG4	3503	immunoglobulin heavy constant gamma 4 (G4m marker)	-	14
IGHJ1	28483	immunoglobulin heavy joining 1	JH1	14
IGHJ2	28481	immunoglobulin heavy joining 2	JH2	14
IGHJ3	28479	immunoglobulin heavy joining 3	JH3b	14
IGHJ4	28477	immunoglobulin heavy joining 4	JH4b	14
IGHJ5	28476	immunoglobulin heavy joining 5	JH5b	14
IGHJ6	28475	immunoglobulin heavy joining 6	JH6b	14
IGHM	3507	immunoglobulin heavy constant mu	AGM1|MU|VH	14
IGH	3492	immunoglobulin heavy locus	IGD1|IGH.1@|IGH@|IGHD@|IGHDY1|IGHJ|IGHJ@|IGHV|IGHV@	14
IGHV1-18	28468	immunoglobulin heavy variable 1-18	IGHV118	14
IGHV1-2	28474	immunoglobulin heavy variable 1-2	IGHV12|V35	14
IGHV1-24	28467	immunoglobulin heavy variable 1-24	IGHV124|VH	14
IGHV1-3	28473	immunoglobulin heavy variable 1-3	IGHV13|VI-3B	14

IGHV1-45	28466	immunoglobulin heavy variable 1-45	IGHV145|VH	14
IGHV1-46	28465	immunoglobulin heavy variable 1-46	IGHV146	14
IGHV1-58	28464	immunoglobulin heavy variable 1-58	IGHV158|VH	14
IGHV1-69	28461	immunoglobulin heavy variable 1-69	IGHV1-E|IGHV169|IGHV1E	14
IGHV1-8	28472	immunoglobulin heavy variable 1-8	IGHV18	14
IGHV1-38-[illegible]	28460	immunoglobulin heavy variable 1-38-4 (non-functional)	IGHV1-C|IGHV1C	14
IGHV1-69-[illegible]	28458	immunoglobulin heavy variable 1-69-2	IGHV1-F|IGHV1F	14
IGHV2-26	28455	immunoglobulin heavy variable 2-26	IGHV226|VH	14
IGHV2-5	28457	immunoglobulin heavy variable 2-5	IGHV25|VH	14
IGHV2-70	28454	immunoglobulin heavy variable 2-70	IGHV270|VH	14
IGHV3-11	28450	immunoglobulin heavy variable 3-11	IGHV311|VH	14
IGHV3-13	28449	immunoglobulin heavy variable 3-13	IGHV313	14
IGHV3-15	28448	immunoglobulin heavy variable 3-15	IGHV315|VH	14
IGHV3-16	28447	immunoglobulin heavy variable 3-16 (non-functional)	IGHV316|VH	14
IGHV3-20	28445	immunoglobulin heavy variable 3-20	IGHV320|VH	14
IGHV3-21	28444	immunoglobulin heavy variable 3-21	IGHV321|VH	14
IGHV3-23	28442	immunoglobulin heavy variable 3-23	DP47|IGHV323|V3-23|VH26	14

IGHV3-30	28439	immunoglobulin heavy variable 3-30	IGHV330|VH	14
IGHV3-30-[illegible]	57290	immunoglobulin heavy variable 3-30-3	IGHV3-3|IGHV3303	14
IGHV3-30-[illegible]	89770	immunoglobulin heavy variable 3-30-5	IGHV3-3|IGHV3305	14
IGHV3-33	28434	immunoglobulin heavy variable 3-33	IGHV333|VH	14
IGHV3-35	28432	immunoglobulin heavy variable 3-35 (non-functional)	IGHV335|VH	14
IGHV3-38	28429	immunoglobulin heavy variable 3-38 (non-functional)	IGHV338|VH	14
IGHV3-43	28426	immunoglobulin heavy variable 3-43	IGHV343|VH	14
IGHV3-48	28424	immunoglobulin heavy variable 3-48	IGHV348|VH	14
IGHV3-49	28423	immunoglobulin heavy variable 3-49	IGHV349|VH	14
IGHV3-53	28420	immunoglobulin heavy variable 3-53	IGHV353|VH	14
IGHV3-64	28414	immunoglobulin heavy variable 3-64	IGHV364|VH	14
IGHV3-66	28412	immunoglobulin heavy variable 3-66	IGHV366|VH	14
IGHV3-7	28452	immunoglobulin heavy variable 3-7	IGHV37|VH	14
IGHV3-72	28410	immunoglobulin heavy variable 3-72	IGHV372|VH	14
IGHV3-73	28409	immunoglobulin heavy variable 3-73	IGHV373|VH	14
IGHV3-74	28408	immunoglobulin heavy variable 3-74	IGHV374|VH	14
IGHV3-9	28451	immunoglobulin heavy variable 3-9	IGHV39|VH	14

IGHV3-38-:	28404	immunoglobulin heavy variable 3-38-3 (non-functional)	IGHV3-D|IGHV3D	14
IGHV3-69-	28402	immunoglobulin heavy variable 3-69-1 (pseudogene)	IGH|IGHM|IGHV|IGHV3-11|IGHV3-H|IGHV3H|IgVH	14
IGHV4-28	28400	immunoglobulin heavy variable 4-28	IGHV428|VH	14
IGHV4-30-	28399	immunoglobulin heavy variable 4-30-1	IGHV4-3	14
IGHV4-30-:	28398	immunoglobulin heavy variable 4-30-2	IGHV4-3|IGHV4302	14
IGHV4-30-	28397	immunoglobulin heavy variable 4-30-4	IGHV4-3|IGHV4304	14
IGHV4-31	28396	immunoglobulin heavy variable 4-31	IGHV431	14
IGHV4-34	28395	immunoglobulin heavy variable 4-34	IGHV434|VH	14
IGHV4-39	28394	immunoglobulin heavy variable 4-39	IGHV439|VH	14
IGHV4-4	28401	immunoglobulin heavy variable 4-4	IGHV44|VH	14
IGHV4-59	28392	immunoglobulin heavy variable 4-59	IGHV459|VH	14
IGHV4-61	28391	immunoglobulin heavy variable 4-61	IGHV461|VH	14
IGHV4-38-:	28389	immunoglobulin heavy variable 4-38-2	IGHV4-B|IGHV4B	14
IGHV5-51	28388	immunoglobulin heavy variable 5-51	IGHV551|VH	14
IGHV5-10-	28386	immunoglobulin heavy variable 5-10-1	IGHV5-A|IGHV5A	14
IGHV6-1	28385	immunoglobulin heavy variable 6-1	IGHV61|VH	14
IGHV7-4-1	57289	immunoglobulin heavy variable 7-4-1	IGHV7-41|IGHV741	14

IGHV7-81	28378	immunoglobulin heavy variable 7-81 (non-functional)	IGHV781	14
IGK	50802	immunoglobulin kappa locus	IGK@	2
IGKC	3514	immunoglobulin kappa constant	HCAK1|IGKCD|Km	2
IGKDEL	3515	immunoglobulin kappa deleting element or like	IGKDE	2
IGKJ	7842	-	IGKJ@	2
IGKJ1	28950	immunoglobulin kappa joining 1	J1	2
IGKJ2	28949	immunoglobulin kappa joining 2	J2	2
IGKJ3	28948	immunoglobulin kappa joining 3	J3	2
IGKJ4	28947	immunoglobulin kappa joining 4	J4	2
IGKJ5	28946	immunoglobulin kappa joining 5	J5	2
IGKV@	3519	-	IGKV|IGKV1|IGKV1@|IGKV2|IGKV2@|IGKV3|IGKV3@	2
IGKV1-12	28940	immunoglobulin kappa variable 1-12	IGKV112|L19	2
IGKV1-13	28939	immunoglobulin kappa variable 1-13	IGKV113|L18	2
IGKV1-16	28938	immunoglobulin kappa variable 1-16	IGKV116|L1	2
IGKV1-17	28937	immunoglobulin kappa variable 1-17	A30|IGKV117	2
IGKV1-27	28935	immunoglobulin kappa variable 1-27	A20|IGKV127	2
IGKV1-33	28933	immunoglobulin kappa variable 1-33	IGKV133|O18	2
IGKV1-37	28931	immunoglobulin kappa variable 1-37 (non-functional)	IGKV137|O14	2
IGKV1-39	28930	immunoglobulin kappa variable 1-39	IGKV139|O12|O12a	2
IGKV1-5	28299	immunoglobulin kappa variable 1-5	IGKV|IGKV15|L12|L12a|V1	2

IGKV1-6	28943	immunoglobulin kappa variable 1-6	IGKV16|L11	2
IGKV1-8	28942	immunoglobulin kappa variable 1-8	IGKV18|L9	2
IGKV1-9	28941	immunoglobulin kappa variable 1-9	IGKV19|L8	2
IGKV1D-12	28903	immunoglobulin kappa variable 1D-12	IGKV1D12|L19	2
IGKV1D-13	28902	immunoglobulin kappa variable 1D-13	IGKV1D13|L18	2
IGKV1D-16	28901	immunoglobulin kappa variable 1D-16	IGKV1D16|L15|L15a	2
IGKV1D-17	28900	immunoglobulin kappa variable 1D-17	IGKV1D17|L14	2
IGKV1D-33	28896	immunoglobulin kappa variable 1D-33	IGKV1D33|O8	2
IGKV1D-37	28894	immunoglobulin kappa variable 1D-37 (non-functional)	IGKV1D37|O4	2
IGKV1D-39	28893	immunoglobulin kappa variable 1D-39	IGKV1D39|O2	2
IGKV1D-42	28892	immunoglobulin kappa variable 1D-42 (non-functional)	IGKV1D42|L22	2
IGKV1D-43	28891	immunoglobulin kappa variable 1D-43	IGKV1D43|L23|L23a	2
IGKV1D-8	28904	immunoglobulin kappa variable 1D-8	IGKV1D8|L24|L24a	2
IGKV2-24	28923	immunoglobulin kappa variable 2-24	A23|IGKV224	2
IGKV2-28	28921	immunoglobulin kappa variable 2-28	A19|IGKV228	2
IGKV2-30	28919	immunoglobulin kappa variable 2-30	A17|IGKV230	2
IGKV2-40	28916	immunoglobulin kappa variable 2-40	IGKV240|O11|O11a	2
IGKV2D-24	28885	immunoglobulin kappa variable 2D-24 (non-functional)	A7|IGKV2D24	2

IGKV2D-28	28883	immunoglobulin kappa variable 2D-28	A3|IGKV2D28	2
IGKV2D-29	28882	immunoglobulin kappa variable 2D-29	A2a|A2c|IGKV2D29	2
IGKV2D-30	28881	immunoglobulin kappa variable 2D-30	A1|IGKV2D30	2
IGKV2D-40	28878	immunoglobulin kappa variable 2D-40	IGKV2D40|O1	2
IGKV3-11	28914	immunoglobulin kappa variable 3-11	IGKV311|L6	2
IGKV3-15	28913	immunoglobulin kappa variable 3-15	IGKV315|L2	2
IGKV3-20	28912	immunoglobulin kappa variable 3-20	13K18|A27|IGKV320	2
IGKV3-7	28915	immunoglobulin kappa variable 3-7 (non-functional)	IGKV37|L10|L10a|Vh	2
IGKV3D-11	28876	immunoglobulin kappa variable 3D-11	IGKV3D11|L20	2
IGKV3D-15	28875	immunoglobulin kappa variable 3D-15	IGKV3D15|L16|L16a|L16b|L16c	2
IGKV3D-20	28874	immunoglobulin kappa variable 3D-20	A11|A11a|IGKV3D20	2
IGKV3D-7	28877	immunoglobulin kappa variable 3D-7	IGKV3D7|L25	2
IGKV4-1	28908	immunoglobulin kappa variable 4-1	B3|IGKV41	2
IGKV5-2	28907	immunoglobulin kappa variable 5-2	B2|IGKV52	2
IGKV6-21	28906	immunoglobulin kappa variable 6-21 (non-functional)	A26|IGKV621	2
IGKV6D-21	28870	immunoglobulin kappa variable 6D-21 (non-functional)	A10|IGKV6D21	2
IGKV6D-41	28869	immunoglobulin kappa variable 6D-41 (non-functional)	A14	2
IGL	3535	immunoglobulin lambda locus	IGL@|IGLC6	22

IGLC1	3537	immunoglobulin lambda constant 1	IGLC	22
IGLC2	3538	immunoglobulin lambda constant 2	IGLC	22
IGLC3	3539	immunoglobulin lambda constant 3 (Kern-Oz+ marker)	IGLC	22
IGLC6	3542	immunoglobulin lambda constant 6	IGLC	22
IGLC7	28834	immunoglobulin lambda constant 7	C7	22
IGLJ	8217	-	IGLJ@	22
IGLJ1	28833	immunoglobulin lambda joining 1	J1	22
IGLJ2	28832	immunoglobulin lambda joining 2	J2	22
IGLJ3	28831	immunoglobulin lambda joining 3	J3	22
IGLJ4	28830	immunoglobulin lambda joining 4 (non-functional)	-	22
IGLJ5	28829	immunoglobulin lambda joining 5 (non-functional)	-	22
IGLJ6	28828	immunoglobulin lambda joining 6	-	22
IGLJ7	28827	immunoglobulin lambda joining 7	J7	22
IGLV@	3546	-	IGLV	22
IGLV1-36	28826	immunoglobulin lambda variable 1-36	IGLV136|V1-11	22
IGLV1-40	28825	immunoglobulin lambda variable 1-40	IGLV140|V1-13	22
IGLV1-44	28823	immunoglobulin lambda variable 1-44	IGLV144|V1-16	22
IGLV1-47	28822	immunoglobulin lambda variable 1-47	IGLV147|V1-17	22
IGLV1-50	28821	immunoglobulin lambda variable 1-50 (non-functional)	IGLV150|V1-18	22
IGLV1-51	28820	immunoglobulin lambda variable 1-51	IGLV151|V1-19	22
IGLV10-54	28772	immunoglobulin lambda variable 10-54	IGLV1054|V1-20	22

IGLV11-55	28770	immunoglobulin lambda variable 11-55 (non-functional)	IGLV1155|V4-6	22
IGLV2-11	28816	immunoglobulin lambda variable 2-11	IGLV211|V1-3	22
IGLV2-14	28815	immunoglobulin lambda variable 2-14	IGLV214|V1-4	22
IGLV2-18	28814	immunoglobulin lambda variable 2-18	IGLV218|V1-5	22
IGLV2-23	28813	immunoglobulin lambda variable 2-23	IGLV223|V1-7	22
IGLV2-33	28811	immunoglobulin lambda variable 2-33 (non-functional)	IGLV233|V1-9	22
IGLV2-8	28817	immunoglobulin lambda variable 2-8	IGLV28|V1-2	22
IGLV3-1	28809	immunoglobulin lambda variable 3-1	IGLV31|V2-1	22
IGLV3-10	28803	immunoglobulin lambda variable 3-10	IGLV310|V2-7	22
IGLV3-12	28802	immunoglobulin lambda variable 3-12	IGLV312|V2-8	22
IGLV3-16	28799	immunoglobulin lambda variable 3-16	IGLV316|V2-11	22
IGLV3-19	28797	immunoglobulin lambda variable 3-19	IGLV319|V2-13|VL3L	22
IGLV3-21	28796	immunoglobulin lambda variable 3-21	IGLV321|V2-14	22
IGLV3-22	28795	immunoglobulin lambda variable 3-22	IGLV322|V2-15	22
IGLV3-25	28793	immunoglobulin lambda variable 3-25	IGLV325|V2-17	22
IGLV3-27	28791	immunoglobulin lambda variable 3-27	IGLV327|V2-19	22
IGLV3-32	28787	immunoglobulin lambda variable 3-32 (non-functional)	IGLV332|V2-23P	22

IGLV3-9	28804	immunoglobulin lambda variable 3-9	IGLV39|V2-6	22
IGLV4-3	28786	immunoglobulin lambda variable 4-3	IGLV43|V5-1	22
IGLV4-60	28785	immunoglobulin lambda variable 4-60	IGLV460|V5-4	22
IGLV4-69	28784	immunoglobulin lambda variable 4-69	IGLV469|V5-6	22
IGLV5-37	28783	immunoglobulin lambda variable 5-37	IGLV537|V4-1	22
IGLV5-39	28782	immunoglobulin lambda variable 5-39	IGLV539	22
IGLV5-45	28781	immunoglobulin lambda variable 5-45	IGLV545|V4-2	22
IGLV5-48	28780	immunoglobulin lambda variable 5-48 (non-functional)	IGLV548|V4-3	22
IGLV5-52	28779	immunoglobulin lambda variable 5-52	IGLV552|V4-4	22
IGLV6-57	28778	immunoglobulin lambda variable 6-57	IGLV657|V1-22	22
IGLV7-43	28776	immunoglobulin lambda variable 7-43	IGLV743|V3-2	22
IGLV7-46	28775	immunoglobulin lambda variable 7-46	IGLV746|V3-3	22
IGLV8-61	28774	immunoglobulin lambda variable 8-61	IGLV861|V3-4	22
IGLV9-49	28773	immunoglobulin lambda variable 9-49	IGLV949|V5-2	22
C3	718	complement C3	AHUS5|ARMD9|ASP|C3a|C3b|CPAMD1|HEL-S-62p	19
C5	727	complement C5	C5D|C5a|C5b|CPAMD4|ECLZB	9
CAMP	820	cathelicidin antimicrobial peptide	CAP-18|CAP18|CRAMP|FALL-39|FALL39|HSD26|LL37	3

CCL1	6346	C-C motif chemokine ligand 1	I-309|P500|SCYA1|SISe|TCA3	17
CCL11	6356	C-C motif chemokine ligand 11	SCYA11	17
CCL13	6357	C-C motif chemokine ligand 13	CKb10|MCP-4|NCC-1|NCC1|SCYA13|SCYL1	17
CCL14	6358	C-C motif chemokine ligand 14	CC-1|CC-3|CKB1|HCC-1|HCC-1(1-74)|HCC-1/HCC-3|HCC-3|MCIF|NCC-2|NCC2|SCYA14|SCYL2|SY14	17
CCL15-CC	348249	CCL15-CCL14 readthrough (NMD candidate)	CCL15|HCC-2|LKN-1|MIP-5|MIP5|Mrp-2b|NCC-3|NCC3|SCYA15	17
CCL15	6359	C-C motif chemokine ligand 15	HCC-2|HMRP-2B|LKN-1|LKN1|MIP-1 delta|MIP-1D|MIP-5|MRP-2B|NCC-3|NCC3|SCYA15|SCYL3|SY15	17
CCL16	6360	C-C motif chemokine ligand 16	CKb12|HCC-4|ILINCK|LCC-1|LEC|LMC|Mtn-1|NCC-4|NCC4|SCYA16|SCYL4	17
CCL17	6361	C-C motif chemokine ligand 17	A-152E5.3|ABCD-2|SCYA17|TARC	16
CCL18	6362	C-C motif chemokine ligand 18	AMAC-1|AMAC1|CKb7|DC-CK1|DCCK1|MIP-4|PARC|SCYA18	17
CCL19	6363	C-C motif chemokine ligand 19	CKb11|ELC|MIP-3b|MIP3B|SCYA19	9
CCL2	6347	C-C motif chemokine ligand 2	GDCF-2|HC11|HSMCR30|MCAF|MCP-1|MCP1|SCYA2|SMC-CF	17

CCL20	6364	C-C motif chemokine ligand 20	CKb4|Exodus|LARC|MIP-3-alpha|MIP-3a|MIP3A|SCYA20|ST38	2
CCL21	6366	C-C motif chemokine ligand 21	6Ckine|CKb9|ECL|SCYA21|SLC|TCA4	9
CCL22	6367	C-C motif chemokine ligand 22	A-152E5.1|ABCD-1|DC/B-CK|MDC|SCYA22|STCP-1	16
CCL23	6368	C-C motif chemokine ligand 23	CK-BETA-8|CKb8|Ckb-8|Ckb-8-1|MIP-3|MIP3|MPIF-1|SCYA23|hmrp-2a	17
CCL24	6369	C-C motif chemokine ligand 24	Ckb-6|MPIF-2|MPIF2|SCYA24	7
CCL25	6370	C-C motif chemokine ligand 25	Ckb15|SCYA25|TECK	19
CCL26	10344	C-C motif chemokine ligand 26	IMAC|MIP-4a|MIP-4alpha|SCYA26|TSC-1	7
CCL27	10850	C-C motif chemokine ligand 27	ALP|CTACK|CTAK|ESKINE|ILC|PESKY|SCYA27	9
CCL28	56477	C-C motif chemokine ligand 28	CCK1|MEC|SCYA28	5
CCL3	6348	C-C motif chemokine ligand 3	G0S19-1|LD78ALPHA|MIP-1-alpha|MIP1A|SCYA3	17
CCL3L1	6349	C-C motif chemokine ligand 3 like 1	464.2|D17S1718|G0S19-2|LD78|LD78-beta(1-70)|LD78BETA|MIP1AP|SCYA3L|SCYA3L1	17
CCL3P1	390788	C-C motif chemokine ligand 3 pseudogene 1	CCL3L2|G0S19-3|LD78gamma|SCYA3L2	17
CCL3L3	414062	C-C motif chemokine ligand 3 like 3	464.2|D17S1718|G0S19-2|LD78|LD78BETA|SCYA3L|SCYA3L1	17

CCL4	6351	C-C motif chemokine ligand 4	ACT2|AT744.1|G-26|HC21|LAG-1|LAG1|MIP-1-beta|MIP1B|MIP1B1|SCYA2|SCYA4	17
CCL4L2	9560	C-C motif chemokine ligand 4 like 2	AT744.2|CCL4L|SCYA4L|SCYQ4L2	17
CCL4L1	388372	C-C motif chemokine ligand 4 like 1	AT744.2|CCL4L|LAG-1|LAG1|MIP-1-beta|SCYA4L|SCYA4L1|SCYA4L2	17
CCL5	6352	C-C motif chemokine ligand 5	D17S136E|RANTES|SCYA5|SIS-delta|SISd|TCP228|eoCP	17
CCL7	6354	C-C motif chemokine ligand 7	FIC|MARC|MCP-3|MCP3|NC28|SCYA6|SCYA7	17
CCL8	6355	C-C motif chemokine ligand 8	HC14|MCP-2|MCP2|SCYA10|SCYA8	17
CKLF	51192	chemokine like factor	C32|CKLF1|CKLF2|CKLF3|CKLF4|HSPC224|UCK-1	16
CMA1	1215	chymase 1	CYH|MCT1|chymase	14
CTSG	1511	cathepsin G	CATG|CG	14
CX3CL1	6376	C-X3-C motif chemokine ligand 1	ABCD-3|C3Xkine|CXC3|CXC3C|NTN|NTT|SCYD1|fractalkine|neurotactin	16
CXCL1	2919	C-X-C motif chemokine ligand 1	FSP|GRO1|GROa|MGSA|MGSA-a|NAP-3|SCYB1	4
CXCL10	3627	C-X-C motif chemokine ligand 10	C7|IFI10|INP10|IP-10|SCYB10|crg-2|gIP-10|mob-1	4
CXCL11	6373	C-X-C motif chemokine ligand 11	H174|I-TAC|IP-9|IP9|SCYB11|SCYB9B|b-R1	4
CXCL12	6387	C-X-C motif chemokine ligand 12	IRH|PBSF|SCYB12|SDF1|TLSF|TPAR1	10
CXCL13	10563	C-X-C motif chemokine ligand 13	ANGIE|ANGIE2|BCA-1|BCA1|BLC|BLR1L|SCYB13	4

CXCL14	9547	C-X-C motif chemokine ligand 14	BMAC|BRAK|KEC|KS1|MIP-2g|MIP2G|NJAC|SCYB14	5
CXCL16	58191	C-X-C motif chemokine ligand 16	CXCLG16|SR-PSOX|SRPSOX	17
CXCL17	284340	C-X-C motif chemokine ligand 17	DMC|Dcip1|UNQ473|VCC-1|VCC1	19
CXCL2	2920	C-X-C motif chemokine ligand 2	CINC-2a|GRO2|GROb|MGSA-b|MIP-2a|MIP2|MIP2A|SCYB2	4
CXCL3	2921	C-X-C motif chemokine ligand 3	CINC-2b|GRO3|GROg|MIP-2b|MIP2B|SCYB3	4
CXCL5	6374	C-X-C motif chemokine ligand 5	ENA-78|SCYB5	4
CXCL6	6372	C-X-C motif chemokine ligand 6	CKA-3|GCP-2|GCP2|SCYB6	4
CXCL9	4283	C-X-C motif chemokine ligand 9	CMK|Humig|MIG|SCYB9|crg-10	4
CCN1	3491	cellular communication network factor 1	CYR61|GIG1|IGFBP10	1
DEFA1	1667	defensin alpha 1	DEF1|DEFA2|HNP-1|HP-1|HP1|MRS	8
DEFA3	1668	defensin alpha 3	DEF3|HNP-3|HNP3|HP-3|HP3	8
DEFA5	1670	defensin alpha 5	DEF5|HD-5	8
DEFB1	1672	defensin beta 1	BD1|DEFB-1|DEFB101|HBD1	8
DEFB103B	55894	defensin beta 103B	BD-3|DEFB-3|DEFB103|DEFB3|HBD-3|HBD3|HBP-3|HBP3	8
DEFB104A	140596	defensin beta 104A	BD-4|DEFB-4|DEFB104|DEFB4|hBD-4	8
DEFB4A	1673	defensin beta 4A	BD-2|DEFB-2|DEFB102|DEFB2|DEFB4|HBD-2|SAP1	8
EDN1	1906	endothelin 1	ARCND3|ET1|HDLCQ7|PPET1|QME	6

EDN2	1907	endothelin 2	ET-2|ET2|PPET2	1
EDN3	1908	endothelin 3	ET-3|ET3|HSCR4|PPET3|WS4B	20
FGF10	2255	fibroblast growth factor 10	-	5
FGF2	2247	fibroblast growth factor 2	BFGF|FGF-2|FGFB|HBGF-2	4
HTN3	3347	histatin 3	HIS2|HTN2|HTN5|PB	4
CXCL8	3576	C-X-C motif chemokine ligand 8	GCP-1|GCP1|IL8|LECT|LUCT|LYNAP|MDNCF|MONAP|NAF|NAP-1|NAP1|SCYB8	4
LECT2	3950	leukocyte cell derived chemotaxin 2	chm-II|chm2	5
PF4	5196	platelet factor 4	CXCL4|PF-4|SCYB4	4
PF4V1	5197	platelet factor 4 variant 1	CXCL4L1|CXCL4V1|PF4-ALT|PF4A|SCYB4V1	4
PLAU	5328	plasminogen activator, urokinase	ATF|BDPLT5|QPD|UPA|URK|u-PA	10
PPBP	5473	pro-platelet basic protein	B-TG1|Beta-TG|CTAP-III|CTAP3|CTAPIII|CXCL7|LA-PF4|LDGF|MDGF|NAP-2|PBP|SCYB7|TC1|TC2|TGB|TGB1|THBGB|THBGB1	4
PPBPP1	728045	pro-platelet basic protein pseudogene 1	PPBPL1|TGB2	4
PROK2	60675	prokineticin 2	BV8|HH4|KAL4|MIT1|PK2	3
RNASE2	6036	ribonuclease A family member 2	EDN|RAF3|RNS2	14
SAA1	6288	serum amyloid A1	PIG4|SAA|SAA2|TP53I4	11
SAA2	6289	serum amyloid A2	SAA|SAA1	11
SBDS	51119	SBDS ribosome maturation factor	CGI-97|SDS|SWDS	7

SEMA3A	10371	semaphorin 3A	COLL1|HH16|Hsema-I|Hsema-III|SEMA1|SEMAD|SEMAIII|SEMAL|SemD|coll-1	7
SEMA3B	7869	semaphorin 3B	LUCA-1|SEMA5|SEMAA|SemA|semaV	3
SEMA3C	10512	semaphorin 3C	SEMAE|SemE	7
SEMA3D	223117	semaphorin 3D	Sema-Z2|coll-2	7
SEMA3E	9723	semaphorin 3E	M-SEMAH|M-SemaK|SEMAH|coll-5	7
SEMA3F	6405	semaphorin 3F	SEMA-IV|SEMA4|SEMAK	3
SEMA3G	56920	semaphorin 3G	sem2	3
SEMA4A	64218	semaphorin 4A	CORD10|RP35|SEMAB|SEMB	1
SEMA4B	10509	semaphorin 4B	SEMAC|SemC	15
SEMA4C	54910	semaphorin 4C	M-SEMA-F|SEMACL1|SEMAF|SEMAI	2
SEMA4D	10507	semaphorin 4D	A8|BB18|C9orf164|CD100|COLL4|GR3|M-sema-G|SEMAJ|coll-4	9
SEMA4F	10505	ssemaphorin 4F	M-SEMA|PRO2353|S4F|SEMAM|SEMAW|m-Sema-M	2
SEMA4G	57715	semaphorin 4G	-	10
SEMA5A	9037	semaphorin 5A	SEMAF|semF	5
SEMA5B	54437	semaphorin 5B	SEMAG|SemG	3
SEMA6A	57556	semaphorin 6A	HT018|SEMA|SEMA6A1|SEMAQ|VIA	5
SEMA6B	10501	semaphorin 6B	EPM11|SEM-SEMA-Y|SEMA-VIB|SEMAN|semaZ	19
SEMA6C	10500	semaphorin 6C	SEMAY|m-SemaY|m-SemaY2	1
SEMA6D	80031	semaphorin 6D	-	15
SEMA7A	8482	semaphorin 7A (John Milton Hagen blood group)	CD108|CDw108|H-SEMA-K1|H-Sema-L|JMH|SEMAK1|SEMAL	15
SLIT1	6585	slit guidance ligand 1	MEGF4|SLIL1|SLIT-1|SLIT3	10

SLIT2	9353	slit guidance ligand 2	SLIL3|Slit-2	4
TNC	3371	tenascin C	150-225|DFNA56|GMEM|GP|HXB|JI|TN|TN-C	9
TYMP	1890	thymidine phosphorylase	ECGF|ECGF1|MEDPS1|MNGIE|MTDPS1|PDECGF|TP|hPD-ECGF	22
XCL1	6375	X-C motif chemokine ligand 1	ATAC|LPTN|LTN|SCM-1|SCM-1a|SCM1|SCM1A|SCYC1	1
XCL2	6846	X-C motif chemokine ligand 2	SCM-1b|SCM1B|SCYC2	1
C5AR1	728	complement C5a receptor 1	C5A|C5AR|C5R1|CD88	19
ACKR2	1238	atypical chemokine receptor 2	CCBP2|CCR10|CCR9|CMKBR9|D6|hD6	3
CCR1	1230	C-C motif chemokine receptor 1	CD191|CKR-1|CKR1|CMKBR1|HM145|MIP1aR|SCYAR1	3
CCR10	2826	C-C motif chemokine receptor 10	GPR2	17
CCR3	1232	C-C motif chemokine receptor 3	C C CKR3|CC-CKR-3|CD193|CKR3|CKR3|CMKBR3	3
CCR4	1233	C-C motif chemokine receptor 4	CC-CKR-4|CD194|CKR4|CMKBR4|ChemR13|HGCN:14099|K5-5	3
CCR5	1234	C-C motif chemokine receptor 5	CC-CKR-5|CCCKR5|CCR-5|CD195|CKR-5|CKR5|CMKBR5|IDDM22	3
CCR6	1235	C-C motif chemokine receptor 6	BN-1|C-C CKR-6|CC-CKR-6|CCR-6|CD196|CKR-L3|CKRL3|CMKBR6|DCR2|DRY6|GPR29|GPRCY4|STRL22	6

CCR7	1236	C-C motif chemokine receptor 7	BLR2|CC-CKR-7|CCR-7|CD197|CDw197|CMKBR7|EBI1	17
CCR8	1237	C-C motif chemokine receptor 8	CC-CKR-8|CCR-8|CDw198|CKRL1|CMKBR8|CMKBRL2|CY6|GPRCY6|TER1	3
CCR9	10803	C-C motif chemokine receptor 9	CC-CKR-9|CDw199|GPR-9-6|GPR28	3
ACKR4	51554	atypical chemokine receptor 4	CC-CKR-11|CCBP2|CCR-11|CCR10|CCR11|CCRL1|CCX CKR|CCX-CKR|CKR-11|PPR1|VSHK1	3
CCRL2	9034	C-C motif chemokine receptor like 2	ACKR5|CKRX|CRAM|CRAM-A|CRAM-B|HCR	3
CMKLR1	1240	chemerin chemokine-like receptor 1	CHEMERINR|ChemR23|DEZ|RVER1	12
CX3CR1	1524	C-X3-C motif chemokine receptor 1	CCRL1|CMKBRL1|CMKDR1|GPR13|GPRV28|V28	3
CXCR3	2833	C-X-C motif chemokine receptor 3	CD182|CD183|CKR-L2|CMKAR3|GPR9|IP10-R|Mig-R|MigR	X
CXCR4	7852	C-X-C motif chemokine receptor 4	CD184|D2S201E|FB22|HM89|HSY3RR|LAP-3|LAP3|LCR1|LESTR|NPY3R|NPYR|NPYRL|NPYY3R|WHIM|WHIMS	2
CXCR5	643	C-X-C motif chemokine receptor 5	BLR1|CD185|MDR15	11
CXCR6	10663	C-X-C motif chemokine receptor 6	BONZO|CD186|STRL33|TYMSTR	3
ACKR3	57007	atypical chemokine receptor 3	CMKOR1|CXC-R7|CXCR-7|CXCR7|GPR159|RDC-1|RDC1	2

CYSLTR1	10800	cysteinyl leukotriene receptor 1	CYSLT1|CYSLT1R|CYSLTR|HMTMF81	X
CYSLTR2	57105	cysteinyl leukotriene receptor 2	CYSLT2|CYSLT2R|GPCR21|HG57|HPN321|KPG_011|PSEC0146|hGPCR21	13
ACKR1	2532	atypical chemokine receptor 1 (Duffy blood group)	CCBP1|CD234|DARC|DARC/ACKR1|Dfy|FY|GPD|GpFy|WBCQ1	1
EDNRA	1909	endothelin receptor type A	ET-A|ETA|ETA-R|ETAR|ETRA|MFDA|hET-AR	4
EDNRB	1910	endothelin receptor type B	ABCDS|ET-B|ET-BR|ETB|ETB1|ETBR|ETRB|HSCR|HSCR2|WS4A	13
FPR1	2357	formyl peptide receptor 1	FMLP|FPR	19
FPR2	2358	formyl peptide receptor 2	ALXR|FMLP-R-II|FMLPX|FPR2A|FPRH1|FPRH2|FPRL1|HM63|LXA4R	19
FPR2	2358	formyl peptide receptor 2	ALXR|FMLP-R-II|FMLPX|FPR2A|FPRH1|FPRH2|FPRL1|HM63|LXA4R	19
GPR17	2840	G protein-coupled receptor 17	-	2
GPR32	2854	G protein-coupled receptor 32	RVDR1	19
GPR33	2856	G protein-coupled receptor 33	-	14
PTGDR2	11251	prostaglandin D2 receptor 2	CD294|CRTH2|DL1R|DP2|GPR44	11
C5AR2	27202	complement component 5a receptor 2	C5L2|GPF77|GPR77	19
CXCR1	3577	C-X-C motif chemokine receptor 1	C-C|C-C-CKR-1|CD128|CD181|CDw128a|CKR-1|CMKAR1|IL8R1|IL8RA|IL8RBA	2
CXCR2	3579	C-X-C motif chemokine receptor 2	CD182|CDw128b|CMKAR2|IL8R2|IL8RA|IL8RB	2
LTB4R	1241	leukotriene B4 receptor	BLT1|BLTR|CMKRL1|GPR16|LTB4R1|LTBR1|P2RY7|P2Y7	14

LTB4R2	56413	leukotriene B4 receptor 2	BLT2|BLTR2|JULF 2|KPG_004|LTB4-R 2|LTB4-R2|NOP9	14
PLAUR	5329	plasminogen activator, urokinase receptor	CD87|U-PAR|UPAR|URKR	19
PLXNA1	5361	plexin A1	NOV|NOVP|PLEXI N-A1|PLXN1	3
PLXNA2	5362	plexin A2	OCT|PLXN2	1
PLXNA3	55558	plexin A3	6.3|HSSEXGENE|P LXN3|PLXN4|XAP-6	X
PLXNA4	91584	plexin A4	FAYV2820|PLEXA 4|PLXNA4A|PLXN A4B|PRO34003	7
PLXNB1	5364	plexin B1	PLEXIN-B1|PLXN5|SEP	3
PLXNB2	23654	plexin B2	MM1|Nbla00445|PL EXB2|dJ402G11.3	22
PLXNB3	5365	plexin B3	PLEXB3|PLEXR|PL XN6	X
PLXNC1	10154	plexin C1	CD232|PLXN-C1|VESPR	12
PLXND1	23129	plexin D1	PLEXD1	3
PTAFR	5724	platelet activating factor receptor	PAFR	1
ROBO1	6091	roundabout guidance receptor 1	DUTT1|SAX3	3
ROBO2	6092	roundabout guidance receptor 2	SAX3	3
ROBO3	64221	roundabout guidance receptor 3	HGPPS|HGPPS1|H GPS|RBIG1|RIG1	11
RXFP3	51289	relaxin family peptide receptor 3	GPCR135|RLN3R1| RXFPR3|SALPR	5
XCR1	2829	X-C motif chemokine receptor 1	CCXCR1|GPR5	3
ADIPOQ	9370	adiponectin, C1Q and collagen domain containing	ACDC|ACRP30|AD IPQTL1|ADPN|AP M-1|APM1|GBP28	3
ADM	133	adrenomedullin	AM|PAMP	11
ADM2	79924	adrenomedullin 2	AM2|dJ579N16.4	22
AGRP	181	agouti related neuropeptide	AGRT|ART|ASIP2	16
AGT	183	angiotensinogen	ANHU|SERPINA8|h FLT1	1
AMBN	258	ameloblastin	AI1F	4

AMELX	265	amelogenin X-linked	AI1E|AIH1|ALGN|AMG|AMGL|AMGX	X
AMH	268	anti-Mullerian hormone	MIF|MIS	19
ANGPTL5	253935	angiopoietin like 5	-	11
ANGPTL7	10218	angiopoietin like 7	AngX|CDT6|dJ647M16.1	1
APLN	8862	apelin	APEL|XNPEP2	X
AREG	374	amphiregulin	AR|AREGB|CRDGF|SDGF	4
MANF	7873	mesencephalic astrocyte derived neurotrophic factor	ARMET|ARP	3
CDNF	441549	cerebral dopamine neurotrophic factor	ARMETL1	10
ARTN	9048	artemin	ART|ENOVIN|EVN|NBN	1
AVP	551	arginine vasopressin	ADH|ARVP|AVP-NPII|AVRP|VP	20
AZU1	566	azurocidin 1	AZAMP|AZU|CAP37|HBP|HUMAZUR|NAZC|hHBP	19
BDNF	627	brain derived neurotrophic factor	ANON2|BULN2	11
BMP1	649	bone morphogenetic protein 1	OI13|PCOLC|PCP|PCP2|TLD	8
BMP10	27302	bone morphogenetic protein 10	-	2
BMP15	9210	bone morphogenetic protein 15	GDF9B|ODG2|POF4	X
BMP2	650	bone morphogenetic protein 2	BDA2|BMP2A|SSFSC	20
BMP3	651	bone morphogenetic protein 3	BMP-3A	4
BMP4	652	bone morphogenetic protein 4	BMP2B|BMP2B1|MCOPS6|OFC11|ZYME	14
BMP5	653	bone morphogenetic protein 5	-	6
BMP6	654	bone morphogenetic protein 6	VGR|VGR1	6

BMP7	655	bone morphogenetic protein 7	OP-1	20
BMP8A	353500	bone morphogenetic protein 8a	OP-2	1
BMP8B	656	bone morphogenetic protein 8b	BMP8|OP2	1
BTC	685	betacellulin	-	4
MYDGF	56005	myeloid derived growth factor	C19orf10|EUROIMAGE1875335|IL25|IL27|IL27w|R33729_1|SF20	19
C3	718	complement C3	AHUS5|ARMD9|ASP|C3a|C3b|CPAMD1|HEL-S-62p	19
C5	727	complement C5	C5D|C5a|C5b|CPAMD4|ECLZB	9
CALCA	796	calcitonin related polypeptide alpha	CALC1|CGRP|CGRP-I|CGRP-alpha|CGRP1|CT|KC|PCT	11
CALCB	797	calcitonin related polypeptide beta	CALC2|CGRP-II|CGRP2	11
CAMP	820	cathelicidin antimicrobial peptide	CAP-18|CAP18|CRAMP|FALL-39|FALL39|HSD26|LL37	3
CAT	847	catalase	-	11
CCK	885	cholecystokinin	-	3
CCL1	6346	C-C motif chemokine ligand 1	I-309|P500|SCYA1|SISe|TCA3	17
CCL11	6356	C-C motif chemokine ligand 11	SCYA11	17
CCL13	6357	C-C motif chemokine ligand 13	CKb10|MCP-4|NCC-1|NCC1|SCYA13|SCYL1	17
CCL14	6358	C-C motif chemokine ligand 14	CC-1|CC-3|CKB1|HCC-1|HCC-1(1-74)|HCC-1/HCC-3|HCC-3|MCIF|NCC-2|NCC2|SCYA14|SCYL2|SY14	17

CCL15-CC[illegible]	348249	CCL15-CCL14 readthrough (NMD candidate)	CCL15|HCC-2|LKN-1|MIP-5|MIP5|Mrp-2b|NCC-3|NCC3|SCYA15	17
CCL15	6359	C-C motif chemokine ligand 15	HCC-2|HMRP-2B|LKN-1|LKN1|MIP-1 delta|MIP-1D|MIP-5|MRP-2B|NCC-3|NCC3|SCYA15|SCYL3|SY15	17
CCL16	6360	C-C motif chemokine ligand 16	CKb12|HCC-4|ILINCK|LCC-1|LEC|LMC|Mtn-1|NCC-4|NCC4|SCYA16|SCYL4	17
CCL17	6361	C-C motif chemokine ligand 17	A-152E5.3|ABCD-2|SCYA17|TARC	16
CCL18	6362	C-C motif chemokine ligand 18	AMAC-1|AMAC1|CKb7|DC-CK1|DCCK1|MIP-4|PARC|SCYA18	17
CCL19	6363	C-C motif chemokine ligand 19	CKb11|ELC|MIP-3b|MIP3B|SCYA19	9
CCL2	6347	C-C motif chemokine ligand 2	GDCF-2|HC11|HSMCR30|MCAF|MCP-1|MCP1|SCYA2|SMC-CF	17
CCL20	6364	C-C motif chemokine ligand 20	CKb4|Exodus|LARC|MIP-3-alpha|MIP-3a|MIP3A|SCYA20|ST38	2
CCL21	6366	C-C motif chemokine ligand 21	6Ckine|CKb9|ECL|SCYA21|SLC|TCA4	9
CCL22	6367	C-C motif chemokine ligand 22	A-152E5.1|ABCD-1|DC/B-CK|MDC|SCYA22|STCP-1	16
CCL23	6368	C-C motif chemokine ligand 23	CK-BETA-8|CKb8|Ckb-8|Ckb-8-1|MIP-3|MIP3|MPIF-1|SCYA23|hmrp-2a	17

CCL24	6369	C-C motif chemokine ligand 24	Ckb-6|MPIF-2|MPIF2|SCYA24	7
CCL25	6370	C-C motif chemokine ligand 25	Ckb15|SCYA25|TECK	19
CCL26	10344	C-C motif chemokine ligand 26	IMAC|MIP-4a|MIP-4alpha|SCYA26|TSC-1	7
CCL27	10850	C-C motif chemokine ligand 27	ALP|CTACK|CTAK|ESKINE|ILC|PESKY|SCYA27	9
CCL28	56477	C-C motif chemokine ligand 28	CCK1|MEC|SCYA28	5
CCL3	6348	C-C motif chemokine ligand 3	G0S19-1|LD78ALPHA|MIP-1-alpha|MIP1A|SCYA3	17
CCL3L1	6349	C-C motif chemokine ligand 3 like 1	464.2|D17S1718|G0S19-2|LD78|LD78-beta(1-70)|LD78BETA|MIP1AP|SCYA3L|SCYA3L1	17
CCL3P1	390788	C-C motif chemokine ligand 3 pseudogene 1	CCL3L2|G0S19-3|LD78gamma|SCYA3L2	17
CCL3L3	414062	C-C motif chemokine ligand 3 like 3	464.2|D17S1718|G0S19-2|LD78|LD78BETA|SCYA3L|SCYA3L1	17
CCL4	6351	C-C motif chemokine ligand 4	ACT2|AT744.1|G-26|HC21|LAG-1|LAG1|MIP-1-beta|MIP1B|MIP1B1|SCYA2|SCYA4	17
CCL4L2	9560	C-C motif chemokine ligand 4 like 2	AT744.2|CCL4L|SCYA4L|SCYQ4L2	17
CCL4L1	388372	C-C motif chemokine ligand 4 like 1	AT744.2|CCL4L|LAG-1|LAG1|MIP-1-beta|SCYA4L|SCYA4L1|SCYA4L2	17
CCL5	6352	C-C motif chemokine ligand 5	D17S136E|RANTES|SCYA5|SIS-delta|SISd|TCP228|eoCP	17

CCL7	6354	C-C motif chemokine ligand 7	FIC|MARC|MCP-3|MCP3|NC28|SCYA6|SCYA7	17
CCL8	6355	C-C motif chemokine ligand 8	HC14|MCP-2|MCP2|SCYA10|SCYA8	17
CD320	51293	CD320 molecule	8D6|8D6A|TCBLR|TCN2R	19
CD40LG	959	CD40 ligand	CD154|CD40L|HIGM1|IGM|IMD3|T-BAM|TNFSF5|TRAP|gp39|hCD40L	X
CD70	970	CD70 molecule	CD27-L|CD27L|CD27LG|LPFS3|TNFSF7|TNLG8A	19
ADA2	51816	adenosine deaminase 2	ADGF|CECR1|IDGFL|PAN|SNEDS|VAIHS	22
CER1	9350	cerberus 1, DAN family BMP antagonist	DAND4	9
CGA	1081	glycoprotein hormones, alpha polypeptide	CG-ALPHA|FSHA|GPA1|GPHA1|GPHa|HCG|LHA|TSHA	6
CGB3	1082	chorionic gonadotropin subunit beta 3	CGB|CGB5|CGB7|CGB8|hCGB	19
CGB1	114335	chorionic gonadotropin subunit beta 1	-	19
CGB2	114336	chorionic gonadotropin subunit beta 2	-	19
CGB5	93659	chorionic gonadotropin subunit beta 5	CGB|HCG|hCGB	19
CGB7	94027	chorionic gonadotropin subunit beta 7	CG-beta-a|CGB6	19
CGB8	94115	chorionic gonadotropin subunit beta 8	-	19
CHGA	1113	chromogranin A	CGA	14
CHGB	1114	chromogranin B	SCG1	20
CKLF	51192	chemokine like factor	C32|CKLF1|CKLF2|CKLF3|CKLF4|HSPC224|UCK-1	16

CLCF1	23529	cardiotrophin like cytokine factor 1	BSF-3|BSF3|CISS2|CLC|NNT-1|NNT1|NR6	11
CLEC11A	6320	C-type lectin domain containing 11A	CLECSF3|LSLCL|P47|SCGF	19
CMA1	1215	chymase 1	CYH|MCT1|chymase	14
CMTM1	113540	CKLF like MARVEL transmembrane domain containing 1	CKLFH|CKLFH1|CKLFSF1	16
CMTM2	146225	CKLF like MARVEL transmembrane domain containing 2	CKLFSF2	16
CMTM3	123920	CKLF like MARVEL transmembrane domain containing 3	BNAS2|CKLFSF3	16
CMTM4	146223	CKLF like MARVEL transmembrane domain containing 4	CKLFSF4	16
CMTM5	116173	CKLF like MARVEL transmembrane domain containing 5	CKLFSF5	14
CMTM6	54918	CKLF like MARVEL transmembrane domain containing 6	CKLFSF6|PRO2219	3
CMTM7	112616	CKLF like MARVEL transmembrane domain containing 7	CKLFSF7	3
CMTM8	152189	CKLF like MARVEL transmembrane domain containing 8	CKLFSF8|CKLFSF8-V2	3
CNTF	1270	ciliary neurotrophic factor	HCNTF	11
CORT	1325	cortistatin	CST-14|CST-17|CST-29	1

CRH	1392	corticotropin releasing hormone	CRF|CRH1	8
CSF1	1435	colony stimulating factor 1	CSF-1|MCSF	1
CSF2	1437	colony stimulating factor 2	CSF|GMCSF	5
CSF3	1440	colony stimulating factor 3	C17orf33|CSF3OS|GCSF	17
CSH1	1442	chorionic somatomammotropin hormone 1	CS-1|CSA|CSMT|GHB3|PL|hCS-1|hCS-A	17
CSH2	1443	chorionic somatomammotropin hormone 2	CS-2|CSB|GHB1|PL|hCS-B	17
CSHL1	1444	chorionic somatomammotropin hormone like 1	CS-5|CSHP1|CSL|GHB4|hCS-L	17
CSPG5	10675	chondroitin sulfate proteoglycan 5	NGC	3
CTF1	1489	cardiotrophin 1	CT-1|CT1	16
CCN2	1490	cellular communication network factor 2	CTGF|HCS24|IGFBP8|NOV2	6
CTSG	1511	cathepsin G	CATG|CG	14
CX3CL1	6376	C-X3-C motif chemokine ligand 1	ABCD-3|C3Xkine|CXC3|CXC3C|NTN|NTT|SCYD1|fractalkine|neurotactin	16
CXCL1	2919	C-X-C motif chemokine ligand 1	FSP|GRO1|GROa|MGSA|MGSA-a|NAP-3|SCYB1	4
CXCL10	3627	C-X-C motif chemokine ligand 10	C7|IFI10|INP10|IP-10|SCYB10|crg-2|gIP-10|mob-1	4
CXCL11	6373	C-X-C motif chemokine ligand 11	H174|I-TAC|IP-9|IP9|SCYB11|SCYB9B|b-R1	4
CXCL12	6387	C-X-C motif chemokine ligand 12	IRH|PBSF|SCYB12|SDF1|TLSF|TPAR1	10
CXCL13	10563	C-X-C motif chemokine ligand 13	ANGIE|ANGIE2|BCA-1|BCA1|BLC|BLR1L|SCYB13	4
CXCL14	9547	C-X-C motif chemokine ligand 14	BMAC|BRAK|KEC|KS1|MIP-2g|MIP2G|NJAC|SCYB14	5

CXCL16	58191	C-X-C motif chemokine ligand 16	CXCLG16|SR-PSOX|SRPSOX	17
CXCL17	284340	C-X-C motif chemokine ligand 17	DMC|Dcip1|UNQ473|VCC-1|VCC1	19
CXCL2	2920	C-X-C motif chemokine ligand 2	CINC-2a|GRO2|GROb|MGSA-b|MIP-2a|MIP2|MIP2A|SCYB2	4
CXCL3	2921	C-X-C motif chemokine ligand 3	CINC-2b|GRO3|GROg|MIP-2b|MIP2B|SCYB3	4
CXCL5	6374	C-X-C motif chemokine ligand 5	ENA-78|SCYB5	4
CXCL6	6372	C-X-C motif chemokine ligand 6	CKA-3|GCP-2|GCP2|SCYB6	4
CXCL9	4283	C-X-C motif chemokine ligand 9	CMK|Humig|MIG|SCYB9|crg-10	4
CCN1	3491	cellular communication network factor 1	CYR61|GIG1|IGFBP10	1
DEFA1	1667	defensin alpha 1	DEF1|DEFA2|HNP-1|HP-1|HP1|MRS	8
DEFA3	1668	defensin alpha 3	DEF3|HNP-3|HNP3|HP-3|HP3	8
DEFA5	1670	defensin alpha 5	DEF5|HD-5	8
DEFB1	1672	defensin beta 1	BD1|DEFB-1|DEFB101|HBD1	8
DEFB103B	55894	defensin beta 103B	BD-3|DEFB-3|DEFB103|DEFB3|HBD-3|HBD3|HBP-3|HBP3	8
DEFB104A	140596	defensin beta 104A	BD-4|DEFB-4|DEFB104|DEFB4|hBD-4	8
DEFB4A	1673	defensin beta 4A	BD-2|DEFB-2|DEFB102|DEFB2|DEFB4|HBD-2|SAP1	8
DKK1	22943	dickkopf WNT signaling pathway inhibitor 1	DKK-1|SK	10
EBI3	10148	Epstein-Barr virus induced 3	IL-27B|IL27B|IL35B	19

EDN1	1906	endothelin 1	ARCND3|ET1|HDLCQ7|PPET1|QME	6
EDN2	1907	endothelin 2	ET-2|ET2|PPET2	1
EDN3	1908	endothelin 3	ET-3|ET3|HSCR4|PPET3|WS4B	20
EGF	1950	epidermal growth factor	HOMG4|URG	4
EPGN	255324	epithelial mitogen	ALGV3072|EPG|PRO9904	4
EPO	2056	erythropoietin	DBAL|ECYT5|EP|MVCD2	7
EREG	2069	epiregulin	EPR|ER|Ep	4
ESM1	11082	endothelial cell specific molecule 1	endocan	5
FAM3B	54097	FAM3 metabolism regulating signaling molecule B	2-21|C21orf11|C21orf76|ORF9|PANDER|PRED44	21
FAM3C	10447	FAM3 metabolism regulating signaling molecule C	GS3786|ILEI	7
FAM3D	131177	FAM3 metabolism regulating signaling molecule D	EF7|OIT1	3
FASLG	356	Fas ligand	ALPS1B|APT1LG1|APTL|CD178|CD95-L|CD95L|FASL|TNFSF6|TNLG1A	1
FGF1	2246	fibroblast growth factor 1	AFGF|ECGF|ECGF-beta|ECGFA|ECGFB|FGF-1|FGF-alpha|FGFA|GLIO703|HBGF-1|HBGF1	5
FGF10	2255	fibroblast growth factor 10	-	5
FGF11	2256	fibroblast growth factor 11	FGF-11|FHF-3|FHF3	17
FGF12	2257	fibroblast growth factor 12	EIEE47|FGF12B|FHF1	3
FGF13	2258	fibroblast growth factor 13	FGF-13|FGF2|FHF-2|FHF2|LINC00889	X
FGF14	2259	fibroblast growth factor 14	FGF-14|FHF-4|FHF4|SCA27	13
FGF16	8823	fibroblast growth factor 16	FGF-16|MF4	X

FGF17	8822	fibroblast growth factor 17	FGF-13|FGF-17|HH20	8
FGF18	8817	fibroblast growth factor 18	FGF-18|ZFGF5	5
FGF19	9965	fibroblast growth factor 19	-	11
FGF2	2247	fibroblast growth factor 2	BFGF|FGF-2|FGFB|HBGF-2	4
FGF20	26281	fibroblast growth factor 20	FGF-20|RHDA2	8
FGF21	26291	fibroblast growth factor 21	-	19
FGF22	27006	fibroblast growth factor 22	-	19
FGF23	8074	fibroblast growth factor 23	ADHR|FGFN|HFTC2|HPDR2|HYPF|PHPTC	12
FGF3	2248	fibroblast growth factor 3	HBGF-3|INT2	11
FGF4	2249	fibroblast growth factor 4	FGF-4|HBGF-4|HST|HST-1|HSTF-1|HSTF1|K-FGF|KFGF	11
FGF5	2250	fibroblast growth factor 5	HBGF-5|Smag-82|TCMGLY	4
FGF6	2251	fibroblast growth factor 6	HBGF-6|HST2	12
FGF7	2252	fibroblast growth factor 7	HBGF-7|KGF	15
FGF8	2253	fibroblast growth factor 8	AIGF|FGF-8|HBGF-8|HH6|KAL6	10
FGF9	2254	fibroblast growth factor 9	FGF-9|GAF|HBFG-9|HBGF-9|SYNS3	13
VEGFD	2277	vascular endothelial growth factor D	FIGF|VEGF-D	X
FIGNL2	401720	fidgetin like 2	-	12
FLT3LG	2323	fms related receptor tyrosine kinase 3 ligand	FL|FLG3L|FLT3L	19
FSHB	2488	follicle stimulating hormone subunit beta	HH24	11
GAL	51083	galanin and GMAP prepropeptide	ETL8|GAL-GMAP|GALN|GLNN|GMAP	11
GALP	85569	galanin like peptide	-	19
GAST	2520	gastrin	GAS	17

GCG	2641	glucagon	GLP-1|GLP1|GLP2|GRPP	2
GDF1	2657	growth differentiation factor 1	CERS1|CHTD6|DORV|DTGA3|LAG1|LASS1|RAI|UOG1	19
GDF10	2662	growth differentiation factor 10	BIP|BMP-3b|BMP3B	10
GDF11	10220	growth differentiation factor 11	BMP-11|BMP11	12
GDF15	9518	growth differentiation factor 15	GDF-15|MIC-1|MIC1|NAG-1|PDF|PLAB|PTGFB	19
GDF2	2658	growth differentiation factor 2	BMP-9|BMP9|HHT5	10
GDF3	9573	growth differentiation factor 3	KFS3|MCOP7|MCOPCB6	12
GDF5	8200	growth differentiation factor 5	BDA1C|BMP-14|BMP14|CDMP1|DUPANS|LAP-4|LAP4|OS5|SYM1B|SYNS2	20
GDF6	392255	growth differentiation factor 6	BMP-13|BMP13|CDMP2|KFM|KFS|KFS1|KFSL|SGM1|SYNS4	8
GDF7	151449	growth differentiation factor 7	BMP12	2
GDF9	2661	growth differentiation factor 9	POF14	5
GDNF	2668	glial cell derived neurotrophic factor	ATF|ATF1|ATF2|HFB1-GDNF|HSCR3	5
GH1	2688	growth hormone 1	GH|GH-N|GHB5|GHN|IGHD1A|IGHD1B|IGHD2|hGH-N	17
GH2	2689	growth hormone 2	GH-V|GHB2|GHL|GHV|hGH-V	17
GHRH	2691	growth hormone releasing hormone	GHRF|GRF|INN	20
GHRL	51738	ghrelin and obestatin prepropeptide	MTLRP	3

GIP	2695	gastric inhibitory polypeptide	-	17
GKN1	56287	gastrokine 1	AMP18|BRICD1|CA11|FOV|foveolin	2
GMFB	2764	glia maturation factor beta	GMF	14
GMFG	9535	glia maturation factor gamma	GMF-GAMMA	19
GNRH1	2796	gonadotropin releasing hormone 1	GNRH|GRH|LHRH|LNRH	8
GNRH2	2797	gonadotropin releasing hormone 2	GnRH-II|LH-RHII	20
GPHA2	170589	glycoprotein hormone subunit alpha 2	A2|GPA2|ZSIG51	11
GPHB5	122876	glycoprotein hormone subunit beta 5	B5|GPB5|ZLUT1	14
GPI	2821	glucose-6-phosphate isomerase	AMF|GNPI|NLK|PGI|PHI|SA-36|SA36	19
GREM1	26585	gremlin 1, DAN family BMP antagonist	C15DUPq|CKTSF1B1|CRAC1|CRCS4|DAND2|DRM|DUP15q|GREMLIN|HMPS|HMPS1|IHG-2|MPSH|PIG2	15
GREM2	64388	gremlin 2, DAN family BMP antagonist	CKTSF1B2|DAND3|PRDC|STHAG9	1
GRN	2896	granulin precursor	CLN11|GEP|GP88|PCDGF|PEPI|PGRN	17
GRP	2922	gastrin releasing peptide	BN|GRP-10|preproGRP|proGRP	18
GUCA2A	2980	guanylate cyclase activator 2A	GCAP-I|GUCA2|STARA	1
HAMP	57817	hepcidin antimicrobial peptide	HEPC|HFE2B|LEAP1|PLTR	19
HBEGF	1839	heparin binding EGF like growth factor	DTR|DTS|DTSF|HEGFL	5
HDGF	3068	heparin binding growth factor	HMG1L2	1
HDGFL3	50810	HDGF like 3	CGI-142|HDGF-2|HDGF2|HDGFRP3|HRP-3	15

HGF	3082	hepatocyte growth factor	DFNB39|F-TCF|HGFB|HPTA|SF	7
HTN3	3347	histatin 3	HIS2|HTN2|HTN5|PB	4
IAPP	3375	islet amyloid polypeptide	DAP|IAP	12
IFNA1	3439	interferon alpha 1	IFL|IFN|IFN-ALPHA|IFN-alphaD|IFNA13|IFNA@|leIF D	9
IFNA10	3446	interferon alpha 10	IFN-alphaC	9
IFNA13	3447	interferon alpha 13	-	9
IFNA14	3448	interferon alpha 14	IFN-alphaH|LEIF2H	9
IFNA16	3449	interferon alpha 16	IFN-alpha-16|IFN-alphaO	9
IFNA17	3451	interferon alpha 17	IFN-alphaI|IFNA|INFA|LEIF2C1	9
IFNA2	3440	interferon alpha 2	IFN-alpha-2|IFN-alphaA|IFNA|IFNA2B|leIF A	9
IFNA21	3452	interferon alpha 21	IFN-alphaI|LeIF F|leIF-F	9
IFNA4	3441	interferon alpha 4	IFN-alpha4a|INFA4	9
IFNA5	3442	interferon alpha 5	IFN-alpha-5|IFN-alphaG|INA5|INFA5|leIF G	9
IFNA6	3443	interferon alpha 6	IFN-alphaK	9
IFNA7	3444	interferon alpha 7	IFN-alphaJ|IFNA-J	9
IFNA8	3445	interferon alpha 8	IFN-alphaB	9
IFNB1	3456	interferon beta 1	IFB|IFF|IFN-beta|IFNB	9
IFNE	338376	interferon epsilon	IFN-E|IFNE1|IFNT1|INFE1|PRO655	9
IFNG	3458	interferon gamma	IFG|IFI	12
IFNK	56832	interferon kappa	IFNT1|INFE1	9
IFNW1	3467	interferon omega 1	-	9
IGF1	3479	insulin like growth factor 1	IGF|IGF-I|IGFI|MGF	12
IGF2	3481	insulin like growth factor 2	C11orf43|GRDF|IGF-II|PP9974	11
IL10	3586	interleukin 10	CSIF|GVHDS|IL-10|IL10A|TGIF	1
IL11	3589	interleukin 11	AGIF|IL-11	19
IL12A	3592	interleukin 12A	CLMF|IL-12A|NFSK|NKSF1|P35	3

IL12B	3593	interleukin 12B	CLMF|CLMF2|IL-12B|IMD28|IMD29|NKSF|NKSF2	5
IL13	3596	interleukin 13	IL-13|P600	5
IL15	3600	interleukin 15	IL-15	4
IL16	3603	interleukin 16	LCF|NIL16|PRIL16|prIL-16	15
IL17A	3605	interleukin 17A	CTLA-8|CTLA8|IL-17|IL-17A|IL17	6
IL17B	27190	interleukin 17B	IL-17B|IL-20|NIRF|ZCYTO7	5
IL17C	27189	interleukin 17C	CX2|IL-17C	16
IL17D	53342	interleukin 17D	IL-17D	13
IL17F	112744	interleukin 17F	CANDF6|IL-17F|ML-1|ML1	6
IL18	3606	interleukin 18	IGIF|IL-18|IL-1g|IL1F4	11
IL19	29949	interleukin 19	IL-10C|MDA1|NG.1|ZMDA1	1
IL1A	3552	interleukin 1 alpha	IL-1 alpha|IL-1A|IL1|IL1-ALPHA|IL1F1	2
IL1B	3553	interleukin 1 beta	IL-1|IL1-BETA|IL1F2|IL1beta	2
IL1F10	84639	interleukin 1 family member 10	FIL1-theta|FKSG75|IL-1HY2|IL-38|IL1-theta|IL1HY2	2
IL36RN	26525	interleukin 36 receptor antagonist	FIL1|FIL1(DELTA)|FIL1D|IL-36Ra|IL1F5|IL1HY1|IL1L1|IL1RP3|IL36RA|PSORP|PSORS14	2
IL36A	27179	interleukin 36 alpha	FIL1|FIL1(EPSILON)|FIL1E|IL-1F6|IL1(EPSILON)|IL1F6	2
IL37	27178	interleukin 37	FIL1|FIL1(ZETA)|FIL1Z|IL-1F7|IL-1H|IL-1H4|IL-1RP1|IL-37|IL1F7|IL1H4|IL1RP1	2

IL36B	27177	interleukin 36 beta	FIL1|FIL1-(ETA)|FIL1H|FILI-(ETA)|IL-1F8|IL-1H2|IL1-ETA|IL1F8|IL1H2	2
IL36G	56300	interleukin 36 gamma	IL-1F9|IL-1H1|IL-1RP2|IL1E|IL1F9|IL1H1|IL1RP2	2
IL1RN	3557	interleukin 1 receptor antagonist	DIRA|ICIL-1RA|IL-1RN|IL-1ra|IL-1ra3|IL1F3|IL1RA|IRAP|MVCD4	2
IL2	3558	interleukin 2	IL-2|TCGF|lymphokine	4
IL20	50604	interleukin 20	IL-20|IL10D|ZCYTO10	1
IL21	59067	interleukin 21	CVID11|IL-21|Za11	4
IL22	50616	interleukin 22	IL-21|IL-22|IL-D110|IL-TIF|ILTIF|TIFIL-23|TIFa|zcyto18	12
IL23A	51561	interleukin 23 subunit alpha	IL-23|IL-23A|IL23P19|P19|SGRF	12
IL24	11009	interleukin 24	C49A|FISP|IL10B|MDA7|MOB5|ST16	1
IL25	64806	interleukin 25	IL17E	14
IL26	55801	interleukin 26	AK155|IL-26	12
IL27	246778	interleukin 27	IL-27|IL-27A|IL27A|IL27p28|IL30|p28	16
IFNL2	282616	interferon lambda 2	IL-28A|IL28A	19
IFNL3	282617	interferon lambda 3	IFN-lambda-3|IFN-lambda-4|IL-28B|IL-28C|IL28B|IL28C	19
IFNL1	282618	interferon lambda 1	IL-29|IL29	19
IL3	3562	interleukin 3	IL-3|MCGF|MULTI-CSF	5
IL31	386653	interleukin 31	IL-31	12

IL32	9235	interleukin 32	IL-32alpha|IL-32beta|IL-32delta|IL-32gamma|NK4|TAIF|TAIFa|TAIFb|TAIFc|TAIFd	16
IL33	90865	interleukin 33	C9orf26|DVS27|IL1F11|NF-HEV|NFEHEV	9
IL34	146433	interleukin 34	C16orf77|IL-34	16
IL4	3565	interleukin 4	BCGF-1|BCGF1|BSF-1|BSF1|IL-4	5
IL5	3567	interleukin 5	EDF|IL-5|TRF	5
IL6	3569	interleukin 6	BSF-2|BSF2|CDF|HGF|HSF|IFN-beta-2|IFNB2|IL-6	7
IL6ST	3572	interleukin 6 signal transducer	CD130|CDW130|GP130|HIES4|IL-6RB|sGP130	5
IL7	3574	interleukin 7	IL-7	8
CXCL8	3576	C-X-C motif chemokine ligand 8	GCP-1|GCP1|IL8|LECT|LUCT|LYNAP|MDNCF|MONAP|NAF|NAP-1|NAP1|SCYB8	4
IL9	3578	interleukin 9	HP40|IL-9|P40	5
INHA	3623	inhibin subunit alpha	-	2
INHBA	3624	inhibin subunit beta A	EDF|FRP	7
INHBB	3625	inhibin subunit beta B	-	2
INHBC	3626	inhibin subunit beta C	IHBC	12
INHBE	83729	inhibin subunit beta E	-	12
INS	3630	insulin	IDDM|IDDM1|IDDM2|ILPR|IRDN|MODY10|PNDM4	11
INS-IGF2	723961	INS-IGF2 readthrough	INSIGF	11
INSL3	3640	insulin like 3	RLF|RLNL|ley-I-L	19
INSL4	3641	insulin like 4	EPIL|PLACENTIN	9
INSL5	10022	insulin like 5	PRO182|UNQ156	1
INSL6	11172	insulin like 6	RIF1	9

JAG1	182	jagged canonical Notch ligand 1	AGS|AGS1|AHD|AWS|CD339|DCHE|HJ1|JAGL1	20
JAG2	3714	jagged canonical Notch ligand 2	HJ2|SER2	14
FGF7P6	387628	fibroblast growth factor 7 pseudogene 6	KGFLP1	9
FGF7P3	654466	fibroblast growth factor 7 pseudogene 3	KGFLP2	9
KITLG	4254	KIT ligand	DCUA|DFNA69|FPH2|FPHH|KL-1|Kitl|MGF|SCF|SF|SHEP7|SLF	12
KL	9365	klotho	HFTC3	13
LACRT	90070	lacritin	-	12
LECT2	3950	leukocyte cell derived chemotaxin 2	chm-II|chm2	5
LEFTY1	10637	left-right determination factor 1	LEFTB|LEFTYB	1
LEFTY2	7044	left-right determination factor 2	EBAF|LEFTA|LEFTYA|TGFB4	1
LEP	3952	leptin	LEPD|OB|OBS	7
LHB	3972	luteinizing hormone subunit beta	CGB4|HH23|LSH-B|LSH-beta	19
LIF	3976	LIF interleukin 6 family cytokine	CDF|DIA|HILDA|MLPLI	22
LRSAM1	90678	leucine rich repeat and sterile alpha motif containing 1	CMT2P|RIFLE|TAL	9
LTA	4049	lymphotoxin alpha	LT|TNFB|TNFSF1|TNLG1E	6
LTB	4050	lymphotoxin beta	TNFC|TNFSF3|TNLG1C|p33	6
LTBP1	4052	latent transforming growth factor beta binding protein 1	-	2
LTBP2	4053	latent transforming growth factor beta binding protein 2	C14orf141|GLC3D|LTBP3|MSPKA|MSTP031|WMS3	14
LTBP3	4054	latent transforming growth factor beta binding protein 3	DASS|GPHYSD3|LTBP-3|LTBP2|STHAG6|pp6425	11

LTBP4	8425	latent transforming growth factor beta binding protein 4	ARCL1C|LTBP-4|LTBP4L|LTBP4S	19
MDK	4192	midkine	ARAP|MK|NEGF2	11
MIA	8190	MIA SH3 domain containing	CD-RAP	19
MIF	4282	macrophage migration inhibitory factor	GIF|GLIF|MMIF	22
MLN	4295	motilin	-	6
MSTN	2660	myostatin	GDF8|MSLHP	2
NAMPT	10135	nicotinamide phosphoribosyltransferase	1110035O14Rik|PBEF|PBEF1|VF|VISFATIN	7
NDP	4693	norrin cystine knot growth factor NDP	EVR2|FEVR|ND	X
NENF	29937	neudesin neurotrophic factor	CIR2|SCIRP10|SPUF	1
NGF	4803	nerve growth factor	Beta-NGF|HSAN5|NGFB	1
NMB	4828	neuromedin B	-	15
NODAL	4838	nodal growth differentiation factor	HTX5	10
CCN3	4856	cellular communication network factor 3	IBP-9|IGFBP-9|IGFBP9|NOV|NOVh	8
NPFF	8620	neuropeptide FF-amide peptide precursor	FMRFAL	12
NPPA	4878	natriuretic peptide A	ANF|ANP|ATFB6|ATRST2|CDD|CDD-ANF|CDP|PND	1
NPPB	4879	natriuretic peptide B	BNP	1
NPPC	4880	natriuretic peptide C	CNP|CNP2	2
NPY	4852	neuropeptide Y	PYY4	7
NRG1	3084	neuregulin 1	ARIA|GGF|GGF2|HGL|HRG|HRG1|HRGA|MST131|MSTP131|NDF|NRG1-IT2|SMDF	8
NRG2	9542	neuregulin 2	DON1|HRG2|NTAK	5
NRG3	10718	neuregulin 3	HRG3|pro-NRG3	10
NRG4	145957	neuregulin 4	HRG4	15
NRTN	4902	neurturin	NTN	19

NTF3	4908	neurotrophin 3	HDNF|NGF-2|NGF2|NT-3|NT3	12
NTF4	4909	neurotrophin 4	GLC10|GLC1O|NT-4|NT-4/5|NT-5|NT4|NT5|NTF5	19
NTS	4922	neurotensin	NMN-125|NN|NT|NT/N|NTS1	12
NUDT6	11162	nudix hydrolase 6	ASFGF2|FGF-AS|FGF2AS|GFG-1|GFG1	4
OGN	4969	osteoglycin	OG|OIF|SLRR3A	9
OSGIN1	29948	oxidative stress induced growth inhibitor 1	BDGI|OKL38	16
OSM	5008	oncostatin M	-	22
OSTN	344901	osteocrin	MUSCLIN	3
OXT	5020	oxytocin/neurophysin I prepropeptide	OT|OT-NPI|OXT-NPI	20
ENDOU	8909	endonuclease, poly(U) specific	P11|PP11|PRSS26	12
PDGFA	5154	platelet derived growth factor subunit A	PDGF-A|PDGF1	7
PDGFB	5155	platelet derived growth factor subunit B	IBGC5|PDGF-2|PDGF2|SIS|SSV|c-sis	22
PDGFC	56034	platelet derived growth factor C	FALLOTEIN|SCDGF	4
PDGFD	80310	platelet derived growth factor D	IEGF|MSTP036|SCDGF-B|SCDGFB	11
PDGFRA	5156	platelet derived growth factor receptor alpha	CD140A|PDGFR-2|PDGFR2	4
PDGFRB	5159	platelet derived growth factor receptor beta	CD140B|IBGC4|IMF1|JTK12|KOGS|PDGFR|PDGFR-1|PDGFR1|PENTT	5
PDGFRL	5157	platelet derived growth factor receptor like	PDGRL|PRLTS	8
PDYN	5173	prodynorphin	ADCA|PENKB|SCA23	20
PENK	5179	proenkephalin	PE|PENK-A	8
PF4	5196	platelet factor 4	CXCL4|PF-4|SCYB4	4
PF4V1	5197	platelet factor 4 variant 1	CXCL4L1|CXCL4V1|PF4-ALT|PF4A|SCYB4V1	4

PGF	5228	placental growth factor	D12S1900|PGFL|PIGF|PLGF|PlGF-2|SHGC-10760	14
PLAU	5328	plasminogen activator, urokinase	ATF|BDPLT5|QPD|UPA|URK|u-PA	10
PMCH	5367	pro-melanin concentrating hormone	MCH|ppMCH	12
PNOC	5368	prepronociceptin	N/OFQ|NOP|OFQ|PPNOC|ppN/OFQ	8
POMC	5443	proopiomelanocortin	ACTH|CLIP|LPH|MSH|NPP|OBAIRH|POC	2
PPBP	5473	pro-platelet basic protein	B-TG1|Beta-TG|CTAP-III|CTAP3|CTAPIII|CXCL7|LA-PF4|LDGF|MDGF|NAP-2|PBP|SCYB7|TC1|TC2|TGB|TGB1|THBGB|THBGB1	4
PPBPP1	728045	pro-platelet basic protein pseudogene 1	PPBPL1|TGB2	4
PPBPP2	10895	pro-platelet basic protein pseudogene 2	PPBPL2|SPBPBP	4
PPY	5539	pancreatic polypeptide	PNP|PP	17
PRL	5617	prolactin	GHA1	6
PRLH	51052	prolactin releasing hormone	PRH|PRRP	2
PROK1	84432	prokineticin 1	EGVEGF|PK1|PRK1	1
PROK2	60675	prokineticin 2	BV8|HH4|KAL4|MIT1|PK2	3
PSPN	5623	persephin	PSP	19
PTH	5741	parathyroid hormone	FIH1|PTH1	11
PTH2	113091	parathyroid hormone 2	TIP39	19
PTHLH	5744	parathyroid hormone like hormone	BDE2|HHM|PLP|PTHR|PTHRP	12

PTN	5764	pleiotrophin	HARP|HB-GAM|HBBM|HBGF-8|HBGF8|HBNF|HBNF-1|NEGF1|OSF-1	7
PYY	5697	peptide YY	PYY-I|PYY1	17
QRFP	347148	pyroglutamylated RFamide peptide	26RFa|P518	9
RABEP1	9135	rabaptin, RAB GTPase binding effector protein 1	RAB5EP|RABPT5	17
RABEP2	79874	rabaptin, RAB GTPase binding effector protein 2	FRA	16
REG1A	5967	regenerating family member 1 alpha	ICRF|P19|PSP|PSPS|PSPS1|PTP|REG	2
RETN	56729	resistin	ADSF|FIZZ3|RETN1|RSTN|XCP1	19
RETNLB	84666	resistin like beta	FIZZ1|FIZZ2|HXCP2|RELM-beta|RELMb|RELMbeta|XCP2	3
RLN1	6013	relaxin 1	H1|H1RLX|RLXH1|bA12D24.3.1|bA12D24.3.2	9
RLN2	6019	relaxin 2	H2|H2-RLX|RLXH2|bA12D24.1.1|bA12D24.1.2	9
RLN3	117579	relaxin 3	H3|RXN3|ZINS4|insl7	19
RNASE2	6036	ribonuclease A family member 2	EDN|RAF3|RNS2	14
S100A6	6277	S100 calcium binding protein A6	2A9|5B10|CABP|CACY|PRA|S10A6	1
SAA1	6288	serum amyloid A1	PIG4|SAA|SAA2|TP53I4	11
SAA2	6289	serum amyloid A2	SAA|SAA1	11
SBDS	51119	SBDS ribosome maturation factor	CGI-97|SDS|SWDS	7
SCG2	7857	secretogranin II	CHGC|EM66|SN|SgII	2
SCGB3A1	92304	secretoglobin family 3A member 1	HIN-1|HIN1|LU105|PnSP-2|UGRP2	5
SCT	6343	secretin	-	11

AIMP1	9255	aminoacyl tRNA synthetase complex interacting multifunctional protein 1	EMAP2|EMAPII|HLD3|SCYE1|p43	4
SECTM1	6398	secreted and transmembrane 1	K12|SECTM	17
SEMA3A	10371	semaphorin 3A	COLL1|HH16|Hsema-I|Hsema-III|SEMA1|SEMAD|SEMAIII|SEMAL|SemD|coll-1	7
SEMA3B	7869	semaphorin 3B	LUCA-1|SEMA5|SEMAA|SemA|semaV	3
SEMA3C	10512	semaphorin 3C	SEMAE|SemE	7
SEMA3D	223117	semaphorin 3D	Sema-Z2|coll-2	7
SEMA3E	9723	semaphorin 3E	M-SEMAH|M-SemaK|SEMAH|coll-5	7
SEMA3F	6405	semaphorin 3F	SEMA-IV|SEMA4|SEMAK	3
SEMA3G	56920	semaphorin 3G	sem2	3
SEMA4A	64218	semaphorin 4A	CORD10|RP35|SEMAB|SEMB	1
SEMA4B	10509	semaphorin 4B	SEMAC|SemC	15
SEMA4C	54910	semaphorin 4C	M-SEMA-F|SEMACL1|SEMAF|SEMAI	2
SEMA4D	10507	semaphorin 4D	A8|BB18|C9orf164|CD100|COLL4|GR3|M-sema-G|SEMAJ|coll-4	9
SEMA4F	10505	ssemaphorin 4F	M-SEMA|PRO2353|S4F|SEMAM|SEMAW|m-Sema-M	2
SEMA4G	57715	semaphorin 4G	-	10
SEMA5A	9037	semaphorin 5A	SEMAF|semF	5
SEMA5B	54437	semaphorin 5B	SEMAG|SemG	3
SEMA6A	57556	semaphorin 6A	HT018|SEMA|SEMA6A1|SEMAQ|VIA	5
SEMA6B	10501	semaphorin 6B	EPM11|SEM-SEMA-Y|SEMA-VIB|SEMAN|semaZ	19
SEMA6C	10500	semaphorin 6C	SEMAY|m-SemaY|m-SemaY2	1

SEMA6D	80031	semaphorin 6D	-	15
SEMA7A	8482	semaphorin 7A (John Milton Hagen blood group)	CD108|CDw108|H-SEMA-K1|H-Sema-L|JMH|SEMAK1|SEMAL	15
SLIT1	6585	slit guidance ligand 1	MEGF4|SLIL1|SLIT-1|SLIT3	10
SLIT2	9353	slit guidance ligand 2	SLIL3|Slit-2	4
SLURP1	57152	secreted LY6/PLAUR domain containing 1	ANUP|ARS|ArsB|LY6-MT|LY6LS|MDM	8
SPP1	6696	secreted phosphoprotein 1	BNSP|BSPI|ETA-1|OPN	4
SST	6750	somatostatin	SMST	3
STC1	6781	stanniocalcin 1	STC	8
STC2	8614	stanniocalcin 2	STC-2|STCRP	5
TAC1	6863	tachykinin precursor 1	Hs.2563|NK2|NKNA|NPK|TAC2	7
TDGF1	6997	teratocarcinoma-derived growth factor 1	CR|CR-1|CRGF|CRIPTO	3
TDGF1P3	6998	teratocarcinoma-derived growth factor 1 pseudogene 3	CR-3|CRIPTO|CRIPTO-3|CRIPTO3|TDGF1|TDGF2|TDGF3	X
TG	7038	thyroglobulin	AITD3|TGN	8
TGFA	7039	transforming growth factor alpha	TFGA	2
TGFB1	7040	transforming growth factor beta 1	CED|DPD1|IBDIMDE|LAP|TGF-beta1|TGFB|TGFbeta	19
TGFB2	7042	transforming growth factor beta 2	G-TSF|LDS4|TGF-beta2	1
TGFB3	7043	transforming growth factor beta 3	ARVD|ARVD1|LDS5|RNHF|TGF-beta3	14
THPO	7066	thrombopoietin	MGDF|MKCSF|ML|MPLLG|THCYT1|TPO	3
TNC	3371	tenascin C	150-225|DFNA56|GMEM|GP|HXB|JI|TN|TN-C	9
TNF	7124	tumor necrosis factor	DIF|TNF-alpha|TNFA|TNFSF2|TNLG1F	6

TNFRSF11	4982	TNF receptor superfamily member 11b	OCIF|OPG|PDB5|TR1	8
TNFSF10	8743	TNF superfamily member 10	APO2L|Apo-2L|CD253|TL2|TNLG6A|TRAIL	3
TNFSF11	8600	TNF superfamily member 11	CD254|ODF|OPGL|OPTB2|RANKL|TNLG6B|TRANCE|hRANKL2|sOdf	13
TNFSF12	8742	TNF superfamily member 12	APO3L|DR3LG|TNLG4A|TWEAK	17
TNFSF13	8741	TNF superfamily member 13	APRIL|CD256|TALL-2|TALL2|TNLG7B|TRDL-1|UNQ383/PRO715|ZTNF2	17
TNFSF13B	10673	TNF superfamily member 13b	BAFF|BLYS|CD257|DTL|TALL-1|TALL1|THANK|TNFSF20|TNLG7A|ZTNF4	13
TNFSF14	8740	TNF superfamily member 14	CD258|HVEML|LIGHT|LTg	19
TNFSF15	9966	TNF superfamily member 15	TL1|TL1A|TNLG1B|VEGI|VEGI192A	9
TNFSF18	8995	TNF superfamily member 18	AITRL|GITRL|TL6|TNLG2A|hGITRL	1
TNFSF4	7292	TNF superfamily member 4	CD134L|CD252|GP34|OX-40L|OX4OL|TNLG2B|TXGP1	1
TNFSF8	944	TNF superfamily member 8	CD153|CD30L|CD30LG|TNLG3A	9
TNFSF9	8744	TNF superfamily member 9	4-1BB-L|CD137L|TNLG5A	19
TOR2A	27433	torsin family 2 member A	TORP1	9
TRH	7200	thyrotropin releasing hormone	Pro-TRH|TRF	3
TSHB	7252	thyroid stimulating hormone subunit beta	TSH-B|TSH-BETA	1
TSLP	85480	thymic stromal lymphopoietin	-	5
TXLNA	200081	taxilin alpha	IL14|TXLN	1
TYMP	1890	thymidine phosphorylase	ECGF|ECGF1|MEDPS1|MNGIE|MTDPS1|PDECGF|TP|hPD-ECGF	22

UCN	7349	urocortin	UI|UROC	2
UCN2	90226	urocortin 2	SRP|UCN-II|UCNI|UR|URP	3
UCN3	114131	urocortin 3	SCP|SPC|UCNIII	10
UTS2	10911	urotensin 2	PRO1068|U-II|UCN2|UII	1
UTS2B	257313	urotensin 2B	U2B|URP|UTS2D	3
VEGFA	7422	vascular endothelial growth factor A	MVCD1|VEGF|VPF	6
VEGFB	7423	vascular endothelial growth factor B	VEGFL|VRF	11
VEGFC	7424	vascular endothelial growth factor C	Flt4-L|LMPH1D|LMPHM4|VRP	4
VGF	7425	VGF nerve growth factor inducible	SCG7|SgVII	7
VIP	7432	vasoactive intestinal peptide	PHM27	6
XCL1	6375	X-C motif chemokine ligand 1	ATAC|LPTN|LTN|SCM-1|SCM-1a|SCM1|SCM1A|SCYC1	1
XCL2	6846	X-C motif chemokine ligand 2	SCM-1b|SCM1B|SCYC2	1
ACVR1B	91	activin A receptor type 1B	ACTRIB|ACVRLK4|ALK4|SKR2	12
ACVR1C	130399	activin A receptor type 1C	ACVRLK7|ALK7	2
ACVR2A	92	activin A receptor type 2A	ACTRII|ACVR2	2
ACVR2B	93	activin A receptor type 2B	ACTRIIB|ActR-IIB|HTX4	3
ACVRL1	94	activin A receptor like type 1	ACVRLK1|ALK-1|ALK1|HHT|HHT2|ORW2|SKR3|TSR-I	12
ADCYAP1	117	ADCYAP receptor type I	PAC1|PAC1R|PACAPR|PACAPRI	7
ADIPOR1	51094	adiponectin receptor 1	ACDCR1|CGI-45|CGI45|PAQR1|TESBP1A	1
ADIPOR2	79602	adiponectin receptor 2	ACDCR2|PAQR2	12
ADRB1	153	adrenoceptor beta 1	ADRB1R|B1AR|BETA1AR|FNSS2|RHR	10

ADRB2	154	adrenoceptor beta 2	ADRB2R|ADRBR|B2AR|BAR|BETA2AR	5
AGTR1	185	angiotensin II receptor type 1	AG2S|AGTR1B|AT1|AT1AR|AT1B|AT1BR|AT1R|AT2R1|HAT1R	3
AGTR2	186	angiotensin II receptor type 2	AT2|ATGR2|MRX88	X
AMHR2	269	anti-Mullerian hormone receptor type 2	AMHR|MISR2|MISRII|MRII	12
ANGPT1	284	angiopoietin 1	AGP1|AGPT|ANG1	8
ANGPT4	51378	angiopoietin 4	ANG3|ANG4	20
ANGPTL1	9068	angiopoietin like 1	ANG3|ANGPT3|ARP1|AngY|UNQ162|dJ595C2.2	1
ANGPTL2	23452	angiopoietin like 2	ARP2|HARP	9
ANGPTL3	27329	angiopoietin like 3	ANG-5|ANGPT5|ANL3|FHBL2	1
ANGPTL4	51129	angiopoietin like 4	ARP4|FIAF|HARP|HFARP|NL2|PGAR|TGQTL|UNQ171|pp1158	19
ANGPTL6	83854	angiopoietin like 6	AGF|ARP5	19
APLNR	187	apelin receptor	AGTRL1|APJ|APJR|HG11	11
AR	367	androgen receptor	AIS|AR8|DHTR|HUMARA|HYSP1|KD|NR3C4|SBMA|SMAX1|TFM	X
AVPR1A	552	arginine vasopressin receptor 1A	AVPR V1a|AVPR1|V1aR	12
AVPR1B	553	arginine vasopressin receptor 1B	AVPR3|V1bR	1
AVPR2	554	arginine vasopressin receptor 2	ADHR|DI1|DIR|DIR3|NDI|V2R	X
BMPR1A	657	bone morphogenetic protein receptor type 1A	10q23del|ACVRLK3|ALK3|CD292|SKR5	10
BMPR1B	658	bone morphogenetic protein receptor type 1B	ALK-6|ALK6|AMDD|BDA1D|BDA2|CDw293	4

BMPR2	659	bone morphogenetic protein receptor type 2	BMPR-II|BMPR3|BMR2|BRK-3|POVD1|PPH1|T-ALK	2
BRD8	10902	bromodomain containing 8	SMAP|SMAP2|p120	5
C3AR1	719	complement C3a receptor 1	AZ3B|C3AR|HNFAG09	12
C5AR1	728	complement C5a receptor 1	C5A|C5AR|C5R1|CD88	19
CALCR	799	calcitonin receptor	CRT|CT-R|CTR|CTR1	7
CALCRL	10203	calcitonin receptor like receptor	CGRPR|CRLR|LMPHM8	2
ACKR2	1238	atypical chemokine receptor 2	CCBP2|CCR10|CCR9|CMKBR9|D6|hD6	3
CCR1	1230	C-C motif chemokine receptor 1	CD191|CKR-1|CKR1|CMKBR1|HM145|MIP1aR|SCYAR1	3
CCR10	2826	C-C motif chemokine receptor 10	GPR2	17
CCR3	1232	C-C motif chemokine receptor 3	C C CKR3|CC-CKR-3|CD193|CKR3|CKR3|CMKBR3	3
CCR4	1233	C-C motif chemokine receptor 4	CC-CKR-4|CD194|CKR4|CMKBR4|ChemR13|HGCN:14099|K5-5	3
CCR5	1234	C-C motif chemokine receptor 5	CC-CKR-5|CCCKR5|CCR-5|CD195|CKR-5|CKR5|CMKBR5|IDDM22	3
CCR6	1235	C-C motif chemokine receptor 6	BN-1|C-C CKR-6|CC-CKR-6|CCR-6|CD196|CKR-L3|CKRL3|CMKBR6|DCR2|DRY6|GPR29|GPRCY4|STRL22	6
CCR7	1236	C-C motif chemokine receptor 7	BLR2|CC-CKR-7|CCR-7|CD197|CDw197|CMKBR7|EBI1	17

CCR8	1237	C-C motif chemokine receptor 8	CC-CKR-8|CCR-8|CDw198|CKRL1|CMKBR8|CMKBRL2|CY6|GPRCY6|TER1	3
CCR9	10803	C-C motif chemokine receptor 9	CC-CKR-9|CDw199|GPR-9-6|GPR28	3
ACKR4	51554	atypical chemokine receptor 4	CC-CKR-11|CCBP2|CCR-11|CCR10|CCR11|CCRL1|CCX CKR|CCX-CKR|CKR-11|PPR1|VSHK1	3
CCRL2	9034	C-C motif chemokine receptor like 2	ACKR5|CKRX|CRAM|CRAM-A|CRAM-B|HCRBp50|CDW40|TNFRSF5|p50	3
CD40	958	CD40 molecule	Bp50|CDW40|TNFRSF5|p50	20
CMKLR1	1240	chemerin chemokine-like receptor 1	CHEMERINR|ChemR23|DEZ|RVER1	12
CNTFR	1271	ciliary neurotrophic factor receptor	-	9
CRHR1	1394	corticotropin releasing hormone receptor 1	CRF-R|CRF-R-1|CRF-R1|CRF1|CRFR-1|CRFR1|CRH-R-1|CRH-R1|CRHR|CRHR1L	17
CRHR2	1395	corticotropin releasing hormone receptor 2	CRF-RB|CRF2|CRFR2|HM-CRF	7
CRIM1	51232	cysteine rich transmembrane BMP regulator 1	CRIM-1|S52	2
CRLF1	9244	cytokine receptor like factor 1	CISS|CISS1|CLF|CLF-1|NR6|zcytor5	19
CRLF2	64109	cytokine receptor like factor 2	CRL2|CRLF2Y|TSLPR	X|Y
CRLF3	51379	cytokine receptor like factor 3	CREME-9|CREME9|CRLM9|CYTOR4|FRWS|p48.2	17
CSF1R	1436	colony stimulating factor 1 receptor	BANDDOS|C-FMS|CD115|CSF-1R|CSFR|FIM2|FMS|HDLS|M-CSF-R	5

CSF2RA	1438	colony stimulating factor 2 receptor subunit alpha	CD116|CDw116|CSF2R|CSF2RAX|CSF2RAY|CSF2RX|CSF2RY|GM-CSF-R-alpha|GMCSFR|GMCSFR-alpha|GMR|GMR-alpha|SMDP4|alphaGMR	X|Y
CSF2RB	1439	colony stimulating factor 2 receptor subunit beta	CD131|CDw131|IL3RB|IL5RB|SMDP5|betaGMR	22
CSF3R	1441	colony stimulating factor 3 receptor	CD114|GCSFR|SCN7	1
CX3CR1	1524	C-X3-C motif chemokine receptor 1	CCRL1|CMKBRL1|CMKDR1|GPR13|GPRV28|V28	3
CXCR3	2833	C-X-C motif chemokine receptor 3	CD182|CD183|CKR-L2|CMKAR3|GPR9|IP10-R|Mig-R|MigR	X
CXCR4	7852	C-X-C motif chemokine receptor 4	CD184|D2S201E|FB22|HM89|HSY3RR|LAP-3|LAP3|LCR1|LESTR|NPY3R|NPYR|NPYRL|NPYY3R|WHIM|WHIMS	2
CXCR5	643	C-X-C motif chemokine receptor 5	BLR1|CD185|MDR15	11
CXCR6	10663	C-X-C motif chemokine receptor 6	BONZO|CD186|STRL33|TYMSTR	3
ACKR3	57007	atypical chemokine receptor 3	CMKOR1|CXC-R7|CXCR-7|CXCR7|GPR159|RDC-1|RDC1	2
CYSLTR1	10800	cysteinyl leukotriene receptor 1	CYSLT1|CYSLT1R|CYSLTR|HMTMF81	X
CYSLTR2	57105	cysteinyl leukotriene receptor 2	CYSLT2|CYSLT2R|GPCR21|HG57|HPN321|KPG_011|PSEC0146|hGPCR21	13
ACKR1	2532	atypical chemokine receptor 1 (Duffy blood group)	CCBP1|CD234|DARC|DARC/ACKR1|Dfy|FY|GPD|GpFy|WBCQ1	1

EDNRA	1909	endothelin receptor type A	ET-A|ETA|ETA-R|ETAR|ETRA|MFDA|hET-AR	4
EDNRB	1910	endothelin receptor type B	ABCDS|ET-B|ET-BR|ETB|ETB1|ETBR|ETRB|HSCR|HSCR2|WS4A	13
EGFR	1956	epidermal growth factor receptor	ERBB|ERBB1|HER1|NISBD2|PIG61|mENA	7
ENG	2022	endoglin	END|HHT1|ORW1	9
EPOR	2057	erythropoietin receptor	EPO-R	19
ESR1	2099	estrogen receptor 1	ER|ESR|ESRA|ESTRR|Era|NR3A1	6
ESR2	2100	estrogen receptor 2	ER-BETA|ESR-BETA|ESRB|ESTRB|Erb|NR3A2|ODG8	14
ESRRA	2101	estrogen related receptor alpha	ERR1|ERRa|ERRalpha|ESRL1|NR3B1	11
ESRRB	2103	estrogen related receptor beta	DFNB35|ERR beta-2|ERR2|ERRb|ERR beta2|ESRL2|NR3B2	14
ESRRG	2104	estrogen related receptor gamma	ERR-gamma|ERR3|ERRg|ERRgamma|NR3B3	1
FGFR1	2260	fibroblast growth factor receptor 1	BFGFR|CD331|CEK|ECCL|FGFBR|FGFR-1|FLG|FLT-2|FLT2|HBGFR|HH2|HRTFDS|KAL2|N-SAM|OGD|bFGF-R-1	8
FGFR2	2263	fibroblast growth factor receptor 2	BBDS|BEK|BFR-1|CD332|CEK3|CFD1|ECT1|JWS|K-SAM|KGFR|TK14|TK25	10
FGFR3	2261	fibroblast growth factor receptor 3	ACH|CD333|CEK2|HSFGFR3EX|JTK4	4
FGFR4	2264	fibroblast growth factor receptor 4	CD334|JTK2|TKF	5
FGFRL1	53834	fibroblast growth factor receptor like 1	FGFR-5|FGFR5|FHFR	4
FLT1	2321	fms related receptor tyrosine kinase 1	FLT|FLT-1|VEGFR-1|VEGFR1	13

FLT3	2322	fms related receptor tyrosine kinase 3	CD135|FLK-2|FLK2|STK1	13
FLT4	2324	fms related receptor tyrosine kinase 4	CHTD7|FLT-4|FLT41|LMPH1A|LMPHM1|PCL|VEGFR-3|VEGFR3	5
FPR1	2357	formyl peptide receptor 1	FMLP|FPR	19
FPR2	2358	formyl peptide receptor 2	ALXR|FMLP-R-II|FMLPX|FPR2A|FPRH1|FPRH2|FPRL1|HM63|LXA4R	19
FPR2	2358	formyl peptide receptor 2	ALXR|FMLP-R-II|FMLPX|FPR2A|FPRH1|FPRH2|FPRL1|HM63|LXA4R	19
FSHR	2492	follicle stimulating hormone receptor	FSHR1|FSHRO|LGR1|ODG1	2
GALR2	8811	galanin receptor 2	GAL2-R|GALNR2|GALR-2	17
GALR3	8484	galanin receptor 3	-	22
GCGR	2642	glucagon receptor	GGR|GL-R	17
GHR	2690	growth hormone receptor	GHBP|GHIP	5
GHRHR	2692	growth hormone releasing hormone receptor	GHRFR|GRFR|IGHD1B|IGHD4	7
GHSR	2693	growth hormone secretagogue receptor	GHDP	3
GIPR	2696	gastric inhibitory polypeptide receptor	PGQTL2	19
GLP1R	2740	glucagon like peptide 1 receptor	GLP-1|GLP-1-R|GLP-1R	6
GLP2R	9340	glucagon like peptide 2 receptor	-	17
GNRHR	2798	gonadotropin releasing hormone receptor	GNRHR1|GRHR|HH7|LHRHR|LRHR	4
GPER1	2852	G protein-coupled estrogen receptor 1	CEPR|CMKRL2|DRY12|FEG-1|GPCR-Br|GPER|GPR30|LERGU|LERGU2|LyGPR|mER	7
GPR17	2840	G protein-coupled receptor 17	-	2

GPR32	2854	G protein-coupled receptor 32	RVDR1	19
GPR33	2856	G protein-coupled receptor 33	-	14
PTGDR2	11251	prostaglandin D2 receptor 2	CD294|CRTH2|DL1R|DP2|GPR44	11
C5AR2	27202	complement component 5a receptor 2	C5L2|GPF77|GPR77	19
HNF4A	3172	hepatocyte nuclear factor 4 alpha	FRTS4|HNF4|HNF4a7|HNF4a8|HNF4a9|HNF4alpha|MODY|MODY1|NR2A1|NR2A21|TCF|TCF14	20
HNF4G	3174	hepatocyte nuclear factor 4 gamma	NR2A2|NR2A3	8
HTR3A	3359	5-hydroxytryptamine receptor 3A	5-HT-3|5-HT3A|5-HT3R|5HT3R|HTR3	11
HTR3B	9177	5-hydroxytryptamine receptor 3B	5-HT3B	11
HTR3C	170572	5-hydroxytryptamine receptor 3C	-	3
HTR3D	200909	5-hydroxytryptamine receptor 3D	5HT3D	3
HTR3E	285242	5-hydroxytryptamine receptor 3E	5-HT3-E|5-HT3E|5-HT3c1	3
IFNAR1	3454	interferon alpha and beta receptor subunit 1	AVP|IFN-alpha-REC|IFNAR|IFNBR|IFRC	21
IFNAR2	3455	interferon alpha and beta receptor subunit 2	IFN-R|IFN-alpha-REC|IFNABR|IFNARB|IMD45	21
IFNGR1	3459	interferon gamma receptor 1	CD119|IFNGR|IMD27A|IMD27B	6
IFNGR2	3460	interferon gamma receptor 2	AF-1|IFGR2|IFNGT1|IMD28	21
IGF1R	3480	insulin like growth factor 1 receptor	CD221|IGFIR|IGFR|JTK13	15
IGF2R	3482	insulin like growth factor 2 receptor	CD222|CI-M6PR|CIMPR|M6P-R|M6P/IGF2R|MPR300|MPR1|MPR300|MPRI	6

IL10RA	3587	interleukin 10 receptor subunit alpha	CD210|CD210a|CDW210A|HIL-10R|IL-10R1|IL10R	11
IL10RB	3588	interleukin 10 receptor subunit beta	CDW210B|CRF2-4|CRFB4|D21S58|D21S66|IL-10R2	21
IL11RA	3590	interleukin 11 receptor subunit alpha	CRSDA	9
IL12RB1	3594	interleukin 12 receptor subunit beta 1	CD212|IL-12R-BETA1|IL12RB|IMD30	19
IL12RB2	3595	interleukin 12 receptor subunit beta 2	-	1
IL13RA1	3597	interleukin 13 receptor subunit alpha 1	CD213A1|CT19|IL-13Ra|NR4	X
IL13RA2	3598	interleukin 13 receptor subunit alpha 2	CD213A2|CT19|IL-13R|IL13BP	X
IL15RA	3601	interleukin 15 receptor subunit alpha	CD215	10
IL2RB	3560	interleukin 2 receptor subunit beta	CD122|IL15RB|IMD63|P70-75	22
IL17RA	23765	interleukin 17 receptor A	CANDF5|CD217|CDw217|IL-17RA|IL17R|IMD51|hIL-17R	22
IL17RB	55540	interleukin 17 receptor B	CRL4|EVI27|IL17BR|IL17RH1	3
IL17RC	84818	interleukin 17 receptor C	CANDF9|IL17-RL|IL17RL	3
IL17RD	54756	interleukin 17 receptor D	HH18|IL-17RD|IL17RLM|SEF	3
IL17RE	132014	interleukin 17 receptor E	-	3
IL18R1	8809	interleukin 18 receptor 1	CD218a|CDw218a|IL-18R-alpha|IL-18Ralpha|IL-1Rrp|IL18RA|IL18Ralpha2|IL1RRP	2
IL18RAP	8807	interleukin 18 receptor accessory protein	ACPL|CD218b|CDw218b|IL-18R-beta|IL-18RAcP|IL-18Rbeta|IL-1R-7|IL-1R7|IL-1RAcPL|IL18RB	2

IL1R1	3554	interleukin 1 receptor type 1	CD121A|D2S1473|IL-1R-alpha|IL1R|IL1RA|P80	2
IL1R2	7850	interleukin 1 receptor type 2	CD121b|CDw121b|IL-1R-2|IL-1RT-2|IL-1RT2|IL1R2c|IL1RB	2
IL1RAP	3556	interleukin 1 receptor accessory protein	C3orf13|IL-1RAcP|IL1R3	3
IL1RL1	9173	interleukin 1 receptor like 1	DER4|FIT-1|IL33R|ST2|ST2L|ST2V|T1	2
IL1RL2	8808	interleukin 1 receptor like 2	IL-1Rrp2|IL-36R|IL1R-rp2|IL1RRP2	2
IL20RA	53832	interleukin 20 receptor subunit alpha	CRF2-8|IL-20R-alpha|IL-20R1|IL-20RA	6
IL20RB	53833	interleukin 20 receptor subunit beta	DIRS1|FNDC6|IL-20R2	3
IL21R	50615	interleukin 21 receptor	CD360|IMD56|NILR	16
IL22RA1	58985	interleukin 22 receptor subunit alpha 1	CRF2-9|IL22R|IL22R1	1
IL22RA2	116379	interleukin 22 receptor subunit alpha 2	CRF2-10|CRF2-S1|CRF2X|IL-22BP|IL-22R-alpha-2|IL-22RA2|ZCYTOR16	6
IL23R	149233	interleukin 23 receptor	-	1
IL27RA	9466	interleukin 27 receptor subunit alpha	CRL1|IL-27RA|IL27R|TCCR|WSX1|zcytor1	19
IFNLR1	163702	interferon lambda receptor 1	CRF2/12|IFNLR|IL-28R1|IL28RA|LICR2	1
IL2RA	3559	interleukin 2 receptor subunit alpha	CD25|IDDM10|IL2R|IMD41|TCGFR|p55	10
IL2RB	3560	interleukin 2 receptor subunit beta	CD122|IL15RB|IMD63|P70-75	22
IL2RG	3561	interleukin 2 receptor subunit gamma	CD132|CIDX|IL-2RG|IMD4|P64|SCIDX|SCIDX1	X

IL31RA	133396	interleukin 31 receptor A	CRL|CRL3|GLM-R|GLMR|GPL|IL-31RA|PLCA2|PRO21384|hGLM-R	5
IL3RA	3563	interleukin 3 receptor subunit alpha	CD123|IL3R|IL3RAY|IL3RX|IL3RY|hIL-3Ra	X|Y
IL4R	3566	interleukin 4 receptor	CD124|IL-4RA|IL4RA	16
IL5RA	3568	interleukin 5 receptor subunit alpha	CD125|CDw125|HSIL5R3|IL5R	3
IL6R	3570	interleukin 6 receptor	CD126|IL-6R-1|IL-6RA|IL6Q|IL6RA|IL6RQ|gp80	1
IL7R	3575	interleukin 7 receptor	CD127|CDW127|IL-7R-alpha|IL7RA|ILRA	5
CXCR1	3577	C-X-C motif chemokine receptor 1	C-C|C-C-CKR-1|CD128|CD181|CDw128a|CKR-1|CMKAR1|IL8R1|IL8RA|IL8RBA	2
CXCR2	3579	C-X-C motif chemokine receptor 2	CD182|CDw128b|CMKAR2|IL8R2|IL8RA|IL8RB	2
IL9R	3581	interleukin 9 receptor	CD129|IL-9R	X|Y
INSR	3643	insulin receptor	CD220|HHF5	19
KDR	3791	kinase insert domain receptor	CD309|FLK1|VEGFR|VEGFR2	4
LEPR	3953	leptin receptor	CD295|LEP-R|LEPRD|OB-R|OBR	1
LGR4	55366	leucine rich repeat containing G protein-coupled receptor 4	BNMD17|GPR48	11
LGR5	8549	leucine rich repeat containing G protein-coupled receptor 5	FEX|GPR49|GPR67|GRP49|HG38	12
LGR6	59352	leucine rich repeat containing G protein-coupled receptor 6	GPCR|VTS20631	1
LHCGR	3973	luteinizing hormone/choriogonadotropin receptor	HHG|LCGR|LGR2|LH/CG-R|LH/CGR|LHR|LHRHR|LSH-R|ULG5	2

LIFR	3977	LIF receptor subunit alpha	CD118|LIF-R|SJS2|STWS|SWS	5
LTB4R	1241	leukotriene B4 receptor	BLT1|BLTR|CMKRL1|GPR16|LTB4R1|LTBR1|P2RY7|P2Y7	14
LTB4R2	56413	leukotriene B4 receptor 2	BLT2|BLTR2|JULF2|KPG_004|LTB4-R2|LTB4-R2|NOP9	14
LTBR	4055	lymphotoxin beta receptor	D12S370|LT-BETA-R|TNF-R-III|TNFCR|TNFR-RP|TNFR2-RP|TNFR3|TNFRSF3	12
MC1R	4157	melanocortin 1 receptor	CMM5|MSH-R|SHEP2	16
MC2R	4158	melanocortin 2 receptor	ACTHR	18
MC3R	4159	melanocortin 3 receptor	BMIQ9|MC3|MC3-R|OB20|OQTL	20
MC4R	4160	melanocortin 4 receptor	BMIQ20	18
MCHR1	2847	melanin concentrating hormone receptor 1	GPR24|MCH-1R|MCH1R|SLC-1|SLC1	22
MCHR2	84539	melanin concentrating hormone receptor 2	GPR145|GPRv17|MCH-2R|MCH-R2|MCH2|MCH2R|MCHR-2|SLT	6
MET	4233	MET proto-oncogene, receptor tyrosine kinase	AUTS9|DFNB97|HGFR|RCCP2|c-Met	7
MLNR	2862	motilin receptor	GPR38|MTLR1	13
MPL	4352	MPL proto-oncogene, thrombopoietin receptor	C-MPL|CD110|MPLV|THCYT2|THPOR|TPOR	1
MTNR1A	4543	melatonin receptor 1A	MEL-1A-R|MT1	4
MTNR1B	4544	melatonin receptor 1B	FGQTL2|MEL-1B-R|MT2	11
NGFR	4804	nerve growth factor receptor	CD271|Gp80-LNGFR|TNFRSF16|p75(NTR)|p75NTR	17
NMBR	4829	neuromedin B receptor	BB1|BB1R|NMB-R	6
NPR1	4881	natriuretic peptide receptor 1	ANPRA|ANPa|GUC2A|GUCY2A|NPRA	1

NPR3	4883	natriuretic peptide receptor 3	ANP-C|ANPR-C|ANPRC|C5orf23|GUCY2B|NPR-C|NPRC	5
NR0B1	190	nuclear receptor subfamily 0 group B member 1	AHC|AHCH|AHX|DAX-1|DAX1|DSS|GTD|HHG|NROB1|SRXY2	X
NR0B2	8431	nuclear receptor subfamily 0 group B member 2	SHP|SHP1	1
NR1D1	9572	nuclear receptor subfamily 1 group D member 1	EAR1|REVERBA|REVERBalpha|THRA1|THRAL|ear-1|hRev	17
NR1D2	9975	nuclear receptor subfamily 1 group D member 2	BD73|EAR-1R|REVERBB|REVERBbeta|RVR	3
NR1H2	7376	nuclear receptor subfamily 1 group H member 2	LXR-b|LXRB|NER|NER-I|RIP15|UNR	19
NR1H3	10062	nuclear receptor subfamily 1 group H member 3	LXR-a|LXRA|RLD-1	11
NR1H4	9971	nuclear receptor subfamily 1 group H member 4	BAR|FXR|HRR-1|HRR1|PFIC5|RIP14	12
NR1I2	8856	nuclear receptor subfamily 1 group I member 2	BXR|ONR1|PAR|PAR1|PAR2|PARq|PRR|PXR|SAR|SXR	3
NR1I3	9970	nuclear receptor subfamily 1 group I member 3	CAR|CAR1|MB67	1
NR2C1	7181	nuclear receptor subfamily 2 group C member 1	TR2	12
NR2C2	7182	nuclear receptor subfamily 2 group C member 2	TAK1|TR4	3
NR2E1	7101	nuclear receptor subfamily 2 group E member 1	TLL|TLX|XTLL	6
NR2E3	10002	nuclear receptor subfamily 2 group E member 3	ESCS|PNR|RNR|RP37|rd7	15

NR2F1	7025	nuclear receptor subfamily 2 group F member 1	BBOAS|BBSOAS|COUP-TFI|COUPTF1|EAR-3|EAR3|ERBAL3|SVP44|TCFCOUP1|TFCOUP1	5
NR2F2	7026	nuclear receptor subfamily 2 group F member 2	ARP-1|ARP1|CHTD4|COUPTF2|COUPTFB|COUPTFII|NF-E3|SVP40|TFCOUP2	15
NR2F6	2063	nuclear receptor subfamily 2 group F member 6	EAR-2|EAR2|ERBAL2	19
NR3C1	2908	nuclear receptor subfamily 3 group C member 1	GCCR|GCR|GCRST|GR|GRL	5
NR3C2	4306	nuclear receptor subfamily 3 group C member 2	MCR|MLR|MR|NR3C2VIT	4
NR4A1	3164	nuclear receptor subfamily 4 group A member 1	GFRP1|HMR|N10|NAK-1|NGFIB|NP10|NUR77|TR3	12
NR4A2	4929	nuclear receptor subfamily 4 group A member 2	HZF-3|NOT|NURR1|RNR1|TINUR	2
NR4A3	8013	nuclear receptor subfamily 4 group A member 3	CHN|CSMF|MINOR|NOR1|TEC	9
NR5A1	2516	nuclear receptor subfamily 5 group A member 1	AD4BP|ELP|FTZ1|FTZF1|POF7|SF-1|SF1|SPGF8|SRXX4|SRXY3|hSF-1	9
NR5A2	2494	nuclear receptor subfamily 5 group A member 2	B1F|B1F2|CPF|FTF|FTZ-F1|FTZ-F1beta|LRH-1|LRH1|hB1F-2	1
NR6A1	2649	nuclear receptor subfamily 6 group A member 1	CT150|GCNF|GCNF1|NR61|RTR|hGCNF|hRTR	9
NRP1	8829	neuropilin 1	BDCA4|CD304|NP1|NRP|VEGF165R	10
NRP2	8828	neuropilin 2	NP2|NPN2|PRO2714|VEGF165R2	2
OGFR	11054	opioid growth factor receptor	-	20
OPRD1	4985	opioid receptor delta 1	DOP|DOR|DOR1|OPRD	1

OPRK1	4986	opioid receptor kappa 1	K-OR-1|KOP|KOR|KOR-1|KOR1|OPRK	8
OPRL1	4987	opioid related nociceptin receptor 1	KOR-3|KOR3|NOCIR|NOP|NOPr|OOR|OPRL|ORL1	20
OPRM1	4988	opioid receptor mu 1	LMOR|M-OR-1|MOP|MOR|MOR1|OPRM	6
OSMR	9180	oncostatin M receptor	IL-31R-beta|IL-31RB|OSMRB|OSMRbeta|PLCA1	5
OXTR	5021	oxytocin receptor	OT-R	3
PGR	5241	progesterone receptor	NR3C3|PR	11
PGRMC2	10424	progesterone receptor membrane component 2	DG6|PMBP	4
PLAUR	5329	plasminogen activator, urokinase receptor	CD87|U-PAR|UPAR|URKR	19
PLXNA1	5361	plexin A1	NOV|NOVP|PLEXIN-A1|PLXN1	3
PLXNA2	5362	plexin A2	OCT|PLXN2	1
PLXNA3	55558	plexin A3	6.3|HSSEXGENE|PLXN3|PLXN4|XAP-6	X
PLXNA4	91584	plexin A4	FAYV2820|PLEXA4|PLXNA4A|PLXNA4B|PRO34003	7
PLXNB1	5364	plexin B1	PLEXIN-B1|PLXN5|SEP	3
PLXNB2	23654	plexin B2	MM1|Nbla00445|PLEXB2|dJ402G11.3	22
PLXNB3	5365	plexin B3	PLEXB3|PLEXR|PLXN6	X
PLXNC1	10154	plexin C1	CD232|PLXN-C1|VESPR	12
PLXND1	23129	plexin D1	PLEXD1	3
PPARA	5465	peroxisome proliferator activated receptor alpha	NR1C1|PPAR|PPARalpha|hPPAR	22
PPARD	5467	peroxisome proliferator activated receptor delta	FAAR|NR1C2|NUC1|NUCI|NUCII|PPARB	6

PPARG	5468	peroxisome proliferator activated receptor gamma	CIMT1|GLM1|NR1C3|PPARG1|PPARG2|PPARG5|PPARgamma	3
PRLHR	2834	prolactin releasing hormone receptor	GPR10|GR3|PrRPR	10
PRLR	5618	prolactin receptor	HPRL|MFAB|RI-PRLR|hPRLrI	5
PTAFR	5724	platelet activating factor receptor	PAFR	1
PTGDR	5729	prostaglandin D2 receptor	AS1|ASRT1|DP|DP1|PTGDR1	14
PTGDS	5730	prostaglandin D2 synthase	L-PGDS|LPGDS|PDS|PGD2|PGDS|PGDS2	9
PTGER1	5731	prostaglandin E receptor 1	EP1	19
PTGER2	5732	prostaglandin E receptor 2	EP2	14
PTGER3	5733	prostaglandin E receptor 3	EP3|EP3-I|EP3-II|EP3-III|EP3-IV|EP3-VI|EP3e|PGE2-R|lnc003875	1
PTGER4	5734	prostaglandin E receptor 4	EP4|EP4R	5
PTGFR	5737	prostaglandin F receptor	FP	1
PTH1R	5745	parathyroid hormone 1 receptor	EKNS|PFE|PTHR|PTHR1	3
PTH2R	5746	parathyroid hormone 2 receptor	PTHR2	2
RARA	5914	retinoic acid receptor alpha	NR1B1|RAR	17
RARB	5915	retinoic acid receptor beta	HAP|MCOPS12|NR1B2|RARbeta1|RRB2	3
RARG	5916	retinoic acid receptor gamma	NR1B3|RARC	12
ROBO1	6091	roundabout guidance receptor 1	DUTT1|SAX3	3
ROBO2	6092	roundabout guidance receptor 2	SAX3	3
ROBO3	64221	roundabout guidance receptor 3	HGPPS|HGPPS1|HGPS|RBIG1|RIG1	11
RORA	6095	RAR related orphan receptor A	IDDECA|NR1F1|ROR1|ROR2|ROR3|RZR-ALPHA|RZRA	15

RORB	6096	RAR related orphan receptor B	EIG15|NR1F2|ROR-BETA|RZR-BETA|RZRB|bA133M9.1	9
RORC	6097	RAR related orphan receptor C	IMD42|NR1F3|RORG|RZR-GAMMA|RZRG|TOR	1
RXFP1	59350	relaxin family peptide receptor 1	LGR7|RXFPR1	4
RXFP2	122042	relaxin family peptide receptor 2	GPR106|GREAT|INSL3R|LGR8|LGR8.1|RXFPR2	13
RXFP3	51289	relaxin family peptide receptor 3	GPCR135|RLN3R1|RXFPR3|SALPR	5
RXRA	6256	retinoid X receptor alpha	NR2B1	9
RXRB	6257	retinoid X receptor beta	DAUDI6|H-2RIIBP|NR2B2|RCoR-1	6
RXRG	6258	retinoid X receptor gamma	NR2B3|RXRC	1
S1PR1	1901	sphingosine-1-phosphate receptor 1	CD363|CHEDG1|D1S3362|ECGF1|EDG-1|EDG1|S1P1	1
S1PR2	9294	sphingosine-1-phosphate receptor 2	AGR16|DFNB68|EDG-5|EDG5|Gpcr13|H218|LPB2|S1P2	19
SCTR	6344	secretin receptor	SR	2
SDC1	6382	syndecan 1	CD138|SDC|SYND1|syndecan	2
SDC2	6383	syndecan 2	CD362|HSPG|HSPG1|SYND2	8
SDC3	9672	syndecan 3	SDCN|SYND3	1
SDC4	6385	syndecan 4	SYND4	20
SORT1	6272	sortilin 1	Gp95|LDLCQ6|NT3|NTR3	1
SSTR1	6751	somatostatin receptor 1	SRIF-2|SS-1-R|SS1-R|SS1R	14
SSTR2	6752	somatostatin receptor 2	-	17
SSTR5	6755	somatostatin receptor 5	SS-5-R	16
ST2	6761	-	-	11
TACR1	6869	tachykinin receptor 1	NK1R|NKIR|SPR|TAC1R	2
TEK	7010	TEK receptor tyrosine kinase	CD202B|GLC3E|TIE-2|TIE2|VMCM|VMCM1	9

TGFBR1	7046	transforming growth factor beta receptor 1	AAT5|ACVRLK4|ALK-5|ALK5|ESS1|LDS1|LDS1A|LDS2A|MSSE|SKR4|TBR-i|TBRI|TGFR-1|tbetaR-I	9
TGFBR2	7048	transforming growth factor beta receptor 2	AAT3|FAA3|LDS1B|LDS2|LDS2B|MFS2|RIIC|TAAD2|TBR-ii|TBRII|TGFR-2|TGFbeta-RII	3
TGFBR3	7049	transforming growth factor beta receptor 3	BGCAN|betaglycan	1
THRA	7067	thyroid hormone receptor alpha	AR7|CHNG6|EAR7|ERB-T-1|ERBA|ERBA1|NR1A1|THRA1|THRA2|c-ERBA-1	17
THRB	7068	thyroid hormone receptor beta	C-ERBA-2|C-ERBA-BETA|ERBA2|GRTH|NR1A2|PRTH|THR1|THRB1|THRB2	3
TIE1	7075	tyrosine kinase with immunoglobulin like and EGF like domains 1	JTK14|TIE	1
TNFRSF10	8797	TNF receptor superfamily member 10a	APO2|CD261|DR4|TRAILR-1|TRAILR1	8
TNFRSF10	8795	TNF receptor superfamily member 10b	CD262|DR5|KILLER|KILLER/DR5|TRAIL-R2|TRAILR2|TRICK2|TRICK2A|TRICK2B|TRICKB|ZTNFR9	8
TNFRSF10	8794	TNF receptor superfamily member 10c	CD263|DCR1|DCR1-TNFR|LIT|TRAIL-R3|TRAILR3|TRID	8
TNFRSF10	8793	TNF receptor superfamily member 10d	CD264|DCR2|TRAIL-R4|TRAILR4|TRUNDD	8

TNFRSF11	8792	TNF receptor superfamily member 11a	CD265|FEO|LOH18CR1|ODFR|OFE|OPTB7|OSTS|PDB2|RANK|TRANCER	18
TNFRSF12	51330	TNF receptor superfamily member 12A	CD266|FN14|TWEAKR	16
TNFRSF13	23495	TNF receptor superfamily member 13B	CD267|CVID|CVID2|IGAD2|RYZN|TACI|TNFRSF14B	17
TNFRSF13	115650	TNF receptor superfamily member 13C	BAFF-R|BAFFR|BROMIX|CD268|CVID4|prolixin	22
TNFRSF14	8764	TNF receptor superfamily member 14	ATAR|CD270|HVEA|HVEM|LIGHTR|TR2	1
TNFRSF17	608	TNF receptor superfamily member 17	BCM|BCMA|CD269|TNFRSF13A	16
TNFRSF18	8784	TNF receptor superfamily member 18	AITR|CD357|ENERGEN|GITR|GITR-D	1
TNFRSF19	55504	TNF receptor superfamily member 19	TAJ|TAJ-alpha|TRADE|TROY	13
TNFRSF1A	7132	TNF receptor superfamily member 1A	CD120a|FPF|TBP1|TNF-R|TNF-R-I|TNF-R55|TNFAR|TNFR1|TNFR55|TNFR60|p55|p55-R|p60	12
TNFRSF1B	7133	TNF receptor superfamily member 1B	CD120b|TBPII|TNF-R-II|TNF-R75|TNFBR|TNFR1B|TNFR2|TNFR80|p75|p75TNFR	1
TNFRSF21	27242	TNF receptor superfamily member 21	BM-018|CD358|DR6	6
TNFRSF25	8718	TNF receptor superfamily member 25	APO-3|DDR3|DR3|GEF720|LARD|PLEKHG5|TNFRSF12|TR3|TRAMP|WSL-1|WSL-LR	1
TNFRSF4	7293	TNF receptor superfamily member 4	ACT35|CD134|IMD16|OX40|TXGP1L	1

TNFRSF6E	8771	TNF receptor superfamily member 6b	DCR3|DJ583P15.1.1|M68|M68E|TR6	20
TNFRSF8	943	TNF receptor superfamily member 8	CD30|D1S166E|Ki-1	1
TNFRSF9	3604	TNF receptor superfamily member 9	4-1BB|CD137|CDw137|ILA	1
TRHR	7201	thyrotropin releasing hormone receptor	CHNG7|TRH-R	8
TSHR	7253	thyroid stimulating hormone receptor	CHNG1|LGR3|hTSHR-I	14
TUBB3	10381	tubulin beta 3 class III	CDCBM|CDCBM1|CFEOM3|CFEOM3A|FEOM3|TUBB4|beta-4	16
VDR	7421	vitamin D receptor	NR1I1|PPP1R163	12
VIPR1	7433	vasoactive intestinal peptide receptor 1	HVR1|II|PACAP-R-2|PACAP-R2|RDC1|V1RG|VAPC1|VIP-R-1|VIPR|VIRG|VPAC1|VPAC1R|VPCAP1R	3
VIPR2	7434	vasoactive intestinal peptide receptor 2	C16DUPq36.3|DUP7q36.3|PACAP-R-3|PACAP-R3|VIP-R-2|VPAC2|VPAC2R|VPCAP2R	7
XCR1	2829	X-C motif chemokine receptor 1	CCXCR1|GPR5	3
IFNA10	3446	interferon alpha 10	IFN-alphaC	9
IFNA13	3447	interferon alpha 13	-	9
IFNA14	3448	interferon alpha 14	IFN-alphaH|LEIF2H	9
IFNA16	3449	interferon alpha 16	IFN-alpha-16|IFN-alphaO	9
IFNA17	3451	interferon alpha 17	IFN-alphaI|IFNA|INFA|LEIF2C1	9
IFNA2	3440	interferon alpha 2	IFN-alpha-2|IFN-alphaA|IFNA|IFNA2B|leIF A	9
IFNA21	3452	interferon alpha 21	IFN-alphaI|LeIF F|leIF-F	9
IFNA4	3441	interferon alpha 4	IFN-alpha4a|INFA4	9

IFNA5	3442	interferon alpha 5	IFN-alpha-5|IFN-alphaG|INA5|INFA5|leIF G	9
IFNA6	3443	interferon alpha 6	IFN-alphaK	9
IFNA7	3444	interferon alpha 7	IFN-alphaJ|IFNA-J	9
IFNA8	3445	interferon alpha 8	IFN-alphaB	9
IFNB1	3456	interferon beta 1	IFB|IFF|IFN-beta|IFNB	9
IFNE	338376	interferon epsilon	IFN-E|IFNE1|IFNT1|INFE1|PRO655	9
IFNG	3458	interferon gamma	IFG|IFI	12
IFNK	56832	interferon kappa	IFNT1|INFE1	9
IFNW1	3467	interferon omega 1	-	9
IFNAR2	3455	interferon alpha and beta receptor subunit 2	IFN-R|IFN-alpha-REC|IFNABR|IFNARB|IMD45	21
IFNGR1	3459	interferon gamma receptor 1	CD119|IFNGR|IMD27A|IMD27B	6
IFNGR2	3460	interferon gamma receptor 2	AF-1|IFGR2|IFNGT1|IMD28	21
IL11	3589	interleukin 11	AGIF|IL-11	19
IL12A	3592	interleukin 12A	CLMF|IL-12A|NFSK|NKSF1|P35	3
IL12B	3593	interleukin 12B	CLMF|CLMF2|IL-12B|IMD28|IMD29|NKSF|NKSF2	5
IL13	3596	interleukin 13	IL-13|P600	5
IL15	3600	interleukin 15	IL-15	4
IL16	3603	interleukin 16	LCF|NIL16|PRIL16|prIL-16	15
IL17A	3605	interleukin 17A	CTLA-8|CTLA8|IL-17|IL-17A|IL17	6
IL17B	27190	interleukin 17B	IL-17B|IL-20|NIRF|ZCYTO7	5
IL17C	27189	interleukin 17C	CX2|IL-17C	16
IL17D	53342	interleukin 17D	IL-17D	13
IL17F	112744	interleukin 17F	CANDF6|IL-17F|ML-1|ML1	6
IL18	3606	interleukin 18	IGIF|IL-18|IL-1g|IL1F4	11
IL19	29949	interleukin 19	IL-10C|MDA1|NG.1|ZMDA1	1

IL1A	3552	interleukin 1 alpha	IL-1 alpha|IL-1A|IL1|IL1-ALPHA|IL1F1	2
IL1B	3553	interleukin 1 beta	IL-1|IL1-BETA|IL1F2|IL1beta	2
IL1F10	84639	interleukin 1 family member 10	FIL1-theta|FKSG75|IL-1HY2|IL-38|IL1-theta|IL1HY2	2
IL36RN	26525	interleukin 36 receptor antagonist	FIL1|FIL1(DELTA)|FIL1D|IL-36Ra|IL1F5|IL1HY1|IL1L1|IL1RP3|IL36RA|PSORP|PSORS14	2
IL36A	27179	interleukin 36 alpha	FIL1|FIL1(EPSILON)|FIL1E|IL-1F6|IL1(EPSILON)|IL1F6	2
IL37	27178	interleukin 37	FIL1|FIL1(ZETA)|FIL1Z|IL-1F7|IL-1H|IL-1H4|IL-1RP1|IL-37|IL1F7|IL1H4|IL1RP1	2
IL36B	27177	interleukin 36 beta	FIL1|FIL1-(ETA)|FIL1H|FILI-(ETA)|IL-1F8|IL-1H2|IL1-ETA|IL1F8|IL1H2	2
IL36G	56300	interleukin 36 gamma	IL-1F9|IL-1H1|IL-1RP2|IL1E|IL1F9|IL1H1|IL1RP2	2
IL1RN	3557	interleukin 1 receptor antagonist	DIRA|ICIL-1RA|IL-1RN|IL-1ra|IL-1ra3|IL1F3|IL1RA|IRAP|MVCD4	2
IL2	3558	interleukin 2	IL-2|TCGF|lymphokine	4
IL20	50604	interleukin 20	IL-20|IL10D|ZCYTO10	1
IL21	59067	interleukin 21	CVID11|IL-21|Za11	4
IL22	50616	interleukin 22	IL-21|IL-22|IL-D110|IL-TIF|ILTIF|TIFIL-23|TIFa|zcyto18	12

IL23A	51561	interleukin 23 subunit alpha	IL-23|IL-23A|IL23P19|P19|SGRF	12
IL24	11009	interleukin 24	C49A|FISP|IL10B|MDA7|MOB5|ST16	1
IL25	64806	interleukin 25	IL17E	14
IL26	55801	interleukin 26	AK155|IL-26	12
IL27	246778	interleukin 27	IL-27|IL-27A|IL27A|IL27p28|IL30|p28	16
IFNL2	282616	interferon lambda 2	IL-28A|IL28A	19
IFNL3	282617	interferon lambda 3	IFN-lambda-3|IFN-lambda-4|IL-28B|IL-28C|IL28B|IL28C	19
IFNL1	282618	interferon lambda 1	IL-29|IL29	19
IL3	3562	interleukin 3	IL-3|MCGF|MULTI-CSF	5
IL31	386653	interleukin 31	IL-31	12
IL32	9235	interleukin 32	IL-32alpha|IL-32beta|IL-32delta|IL-32gamma|NK4|TAIF|TAIFa|TAIFb|TAIFc|TAIFd	16
IL33	90865	interleukin 33	C9orf26|DVS27|IL1F11|NF-HEV|NFEHEV	9
IL34	146433	interleukin 34	C16orf77|IL-34	16
IL4	3565	interleukin 4	BCGF-1|BCGF1|BSF-1|BSF1|IL-4	5
IL5	3567	interleukin 5	EDF|IL-5|TRF	5
IL6	3569	interleukin 6	BSF-2|BSF2|CDF|HGF|HSF|IFN-beta-2|IFNB2|IL-6	7
IL6ST	3572	interleukin 6 signal transducer	CD130|CDW130|GP130|HIES4|IL-6RB|sGP130	5
IL7	3574	interleukin 7	IL-7	8
CXCL8	3576	C-X-C motif chemokine ligand 8	GCP-1|GCP1|IL8|LECT|LUCT|LYNAP|MDNCF|MONAP|NAF|NAP-1|NAP1|SCYB8	4

IL9	3578	interleukin 9	HP40|IL-9|P40	5
TXLNA	200081	taxilin alpha	IL14|TXLN	1
IL10RA	3587	interleukin 10 receptor subunit alpha	CD210|CD210a|CDW210A|HIL-10R|IL-10R1|IL10R	11
IL10RB	3588	interleukin 10 receptor subunit beta	CDW210B|CRF2-4|CRFB4|D21S58|D21S66|IL-10R2	21
IL11RA	3590	interleukin 11 receptor subunit alpha	CRSDA	9
IL12RB1	3594	interleukin 12 receptor subunit beta 1	CD212|IL-12R-BETA1|IL12RB|IMD30	19
IL12RB2	3595	interleukin 12 receptor subunit beta 2	-	1
IL13RA1	3597	interleukin 13 receptor subunit alpha 1	CD213A1|CT19|IL-13Ra|NR4	X
IL13RA2	3598	interleukin 13 receptor subunit alpha 2	CD213A2|CT19|IL-13R|IL13BP	X
IL15RA	3601	interleukin 15 receptor subunit alpha	CD215	10
IL2RB	3560	interleukin 2 receptor subunit beta	CD122|IL15RB|IMD63|P70-75	22
IL17RA	23765	interleukin 17 receptor A	CANDF5|CD217|CDw217|IL-17RA|IL17R|IMD51|hIL-17R	22
IL17RB	55540	interleukin 17 receptor B	CRL4|EVI27|IL17BR|IL17RH1	3
IL17RC	84818	interleukin 17 receptor C	CANDF9|IL17-RL|IL17RL	3
IL17RD	54756	interleukin 17 receptor D	HH18|IL-17RD|IL17RLM|SEF	3
IL17RE	132014	interleukin 17 receptor E	-	3
IL18R1	8809	interleukin 18 receptor 1	CD218a|CDw218a|IL-18R-alpha|IL-18Ralpha|IL-1Rrp|IL18RA|IL18Ralpha2|IL1RRP	2

IL18RAP	8807	interleukin 18 receptor accessory protein	ACPL|CD218b|CDw218b|IL-18R-beta|IL-18RAcP|IL-18Rbeta|IL-1R-7|IL-1R7|IL-1RAcPL|IL18RB	2
IL1R1	3554	interleukin 1 receptor type 1	CD121A|D2S1473|IL-1R-alpha|IL1R|IL1RA|P80	2
IL1R2	7850	interleukin 1 receptor type 2	CD121b|CDw121b|IL-1R-2|IL-1RT-2|IL-1RT2|IL1R2c|IL1RB	2
IL1RAP	3556	interleukin 1 receptor accessory protein	C3orf13|IL-1RAcP|IL1R3	3
IL1RL1	9173	interleukin 1 receptor like 1	DER4|FIT-1|IL33R|ST2|ST2L|ST2V|T1	2
IL1RL2	8808	interleukin 1 receptor like 2	IL-1Rrp2|IL-36R|IL1R-rp2|IL1RRP2	2
IL20RA	53832	interleukin 20 receptor subunit alpha	CRF2-8|IL-20R-alpha|IL-20R1|IL-20RA	6
IL20RB	53833	interleukin 20 receptor subunit beta	DIRS1|FNDC6|IL-20R2	3
IL21R	50615	interleukin 21 receptor	CD360|IMD56|NILR	16
IL22RA1	58985	interleukin 22 receptor subunit alpha 1	CRF2-9|IL22R|IL22R1	1
IL22RA2	116379	interleukin 22 receptor subunit alpha 2	CRF2-10|CRF2-S1|CRF2X|IL-22BP|IL-22R-alpha-2|IL-22RA2|ZCYTOR16	6
IL23R	149233	interleukin 23 receptor	-	1
IL27RA	9466	interleukin 27 receptor subunit alpha	CRL1|IL-27RA|IL27R|TCCR|WSX1|zcytor1	19
IFNLR1	163702	interferon lambda receptor 1	CRF2/12|IFNLR|IL-28R1|IL28RA|LICR2	1
IL2RA	3559	interleukin 2 receptor subunit alpha	CD25|IDDM10|IL2R|IMD41|TCGFR|p55	10

IL2RB	3560	interleukin 2 receptor subunit beta	CD122|IL15RB|IMD63|P70-75	22
IL2RG	3561	interleukin 2 receptor subunit gamma	CD132|CIDX|IL-2RG|IMD4|P64|SCIDX|SCIDX1	X
IL31RA	133396	interleukin 31 receptor A	CRL|CRL3|GLM-R|GLMR|GPL|IL-31RA|PLCA2|PRO21384|hGLM-R	5
IL3RA	3563	interleukin 3 receptor subunit alpha	CD123|IL3R|IL3RAY|IL3RX|IL3RY|hIL-3Ra	X|Y
IL4R	3566	interleukin 4 receptor	CD124|IL-4RA|IL4RA	16
IL5RA	3568	interleukin 5 receptor subunit alpha	CD125|CDw125|HSIL5R3|IL5R	3
IL6R	3570	interleukin 6 receptor	CD126|IL-6R-1|IL-6RA|IL6Q|IL6RA|IL6RQ|gp80	1
IL7R	3575	interleukin 7 receptor	CD127|CDW127|IL-7R-alpha|IL7RA|ILRA	5
CXCR1	3577	C-X-C motif chemokine receptor 1	C-C|C-C-CKR-1|CD128|CD181|CDw128a|CKR-1|CMKAR1|IL8R1|IL8RA|IL8RBA	2
CXCR2	3579	C-X-C motif chemokine receptor 2	CD182|CDw128b|CMKAR2|IL8R2|IL8RA|IL8RB	2
IL9R	3581	interleukin 9 receptor	CD129|IL-9R	X|Y
ST2	6761	-	-	11
HLA-A	3105	major histocompatibility complex, class I, A	HLAA	6
HLA-B	3106	major histocompatibility complex, class I, B	AS|B-4901|HLAB	6
HLA-C	3107	major histocompatibility complex, class I, C	D6S204|HLA-JY3|HLAC|HLC-C|MHC|PSORS1	6
HLA-E	3133	major histocompatibility complex, class I, E	HLA-6.2|QA1	6
HLA-G	3135	major histocompatibility complex, class I, G	MHC-G	6

KIR3DL1	3811	killer cell immunoglobulin like receptor, three Ig domains and long cytoplasmic tail 1	CD158E1|KIR|KIR3DL1/S1|NKAT-3|NKAT3|NKB1|NKB1B	19
KIR3DL2	3812	killer cell immunoglobulin like receptor, three Ig domains and long cytoplasmic tail 2	3DL2|CD158K|KIR-3DL2|NKAT-4|NKAT4|NKAT4B|p140	19
KIR2DL1	3802	killer cell immunoglobulin like receptor, two Ig domains and long cytoplasmic tail 1	CD158A|KIR-K64|KIR221|KIR2DL3|NKAT|NKAT-1|NKAT1|p58.1	19
KIR2DL2	3803	killer cell immunoglobulin like receptor, two Ig domains and long cytoplasmic tail 2	CD158B1|CD158b|NKAT-6|NKAT6|p58.2	19
KIR2DL3	3804	killer cell immunoglobulin like receptor, two Ig domains and long cytoplasmic tail 3	CD158B2|CD158b|GL183|KIR-023GB|KIR-K7b|KIR-K7c|KIR2DL|KIR2DS5|KIRCL23|NKAT|NKAT2|NKAT2A|NKAT2B|p58	19
KIR2DL4	3805	killer cell immunoglobulin like receptor, two Ig domains and long cytoplasmic tail 4	CD158D|G9P|KIR-103AS|KIR-2DL4|KIR103|KIR103AS	19
KIR2DL5A	57292	killer cell immunoglobulin like receptor, two Ig domains and long cytoplasmic tail 5A	CD158F|KIR2DL5|KIR2DL5.1|KIR2DL5.3	19
KLRC1	3821	killer cell lectin like receptor C1	CD159A|NKG2|NKG2A	12
KLRC2	3822	killer cell lectin like receptor C2	CD159c|NKG2-C|NKG2C	12
KLRC3	3823	killer cell lectin like receptor C3	NKG2-E|NKG2E	12
KLRD1	3824	killer cell lectin like receptor D1	CD94	12

PTPN6	5777	protein tyrosine phosphatase non-receptor type 6	HCP|HCPH|HPTP1C|PTP-1C|SH-PTP1|SHP-1|SHP-1L|SHP1	12
PTPN11	5781	protein tyrosine phosphatase non-receptor type 11	BPTP3|CFC|JMML|METCDS|NS1|PTP-1D|PTP2C|SH-PTP2|SH-PTP3|SHP2	12
ICAM1	3383	intercellular adhesion molecule 1	BB2|CD54|P3.58	19
ICAM2	3384	intercellular adhesion molecule 2	CD102	17
ITGAL	3683	integrin subunit alpha L	CD11A|LFA-1|LFA1A	16
ITGB2	3689	integrin subunit beta 2	CD18|LAD|LCAMB|LFA-1|MAC-1|MF17|MFI7	21
PTK2B	2185	protein tyrosine kinase 2 beta	CADTK|CAKB|FADK2|FAK2|PKB|PTK|PYK2|RAFTK	8
VAV3	10451	vav guanine nucleotide exchange factor 3	-	1
VAV1	7409	vav guanine nucleotide exchange factor 1	VAV	19
VAV2	7410	vav guanine nucleotide exchange factor 2	VAV-2	9
RAC1	5879	Rac family small GTPase 1	MIG5|MRD48|Rac-1|TC-25|p21-Rac1	7
RAC2	5880	Rac family small GTPase 2	EN-7|Gx|HSPC022|p21-Rac2	22
RAC3	5881	Rac family small GTPase 3	-	17
PAK1	5058	p21 (RAC1) activated kinase 1	IDDMSSD|PAKalpha|alpha-PAK|p65-PAK	11
MAP2K1	5604	mitogen-activated protein kinase kinase 1	CFC3|MAPKK1|MEK1|MKK1|PRKMK1	15
MAP2K2	5605	mitogen-activated protein kinase kinase 2	CFC4|MAPKK2|MEK2|MKK2|PRKMK2	19

MAPK1	5594	mitogen-activated protein kinase 1	ERK|ERK-2|ERK2|ERT1|MAPK2|P42MAPK|PRKM1|PRKM2|p38|p40|p41|p41mapk|p42-MAPK	22
MAPK3	5595	mitogen-activated protein kinase 3	ERK-1|ERK1|ERT2|HS44KDAP|HUMKER1A|P44ERK1|P44MAPK|PRKM3|p44-ERK1|p44-MAPK	16
TNF	7124	tumor necrosis factor	DIF|TNF-alpha|TNFA|TNFSF2|TNLG1F	6
CSF2	1437	colony stimulating factor 2	CSF|GMCSF	5
IFNG	3458	interferon gamma	IFG|IFI	12
KIR2DS1	3806	killer cell immunoglobulin like receptor, two Ig domains and short cytoplasmic tail 1	CD158H|CD158a|p50.1	19
KIR2DS3	3808	killer cell immunoglobulin like receptor, two Ig domains and short cytoplasmic tail 3	NKAT7	19
KIR2DS4	3809	killer cell immunoglobulin like receptor, two Ig domains and short cytoplasmic tail 4	CD158I|KIR-2DS4|KIR1D|KIR412|KKA3|NKAT-8|NKAT8	19
KIR2DS5	3810	killer cell immunoglobulin like receptor, two Ig domains and short cytoplasmic tail 5	CD158G|NKAT9	19
NCR2	9436	natural cytotoxicity triggering receptor 2	CD336|LY95|NK-p44|NKP44|dJ149M18.1	6
TYROBP	7305	transmembrane immune signaling adaptor TYROBP	DAP12|KARAP|PLOSL|PLOSL1	19
LCK	3932	LCK proto-oncogene, Src family tyrosine kinase	IMD22|LSK|YT16|p56lck|pp58lck	1

FCGR3A	2214	Fc fragment of IgG receptor IIIa	CD16|CD16A|FCG3|FCGR3|FCGRIII|FCR-10|FCRIII|FCRIIIA|IGFR3|IMD20	1
FCGR3B	2215	Fc fragment of IgG receptor IIIb	CD16|CD16A|CD16b|FCG3|FCGR3|FCGR3A|FCR-10|FCRIII|FCRIIIb	1
NCR1	9437	natural cytotoxicity triggering receptor 1	CD335|LY94|NK-p46|NKP46	19
NCR3	259197	natural cytotoxicity triggering receptor 3	1C7|CD337|LY117|MALS|NKp30	6
FCER1G	2207	Fc fragment of IgE receptor Ig	FCRG	1
CD247	919	CD247 molecule	CD3-ZETA|CD3H|CD3Q|CD3Z|IMD25|T3Z|TCRZ	1
ZAP70	7535	zeta chain of T cell receptor associated protein kinase 70	ADMIO2|IMD48|SRK|STCD|STD|TZK|ZAP-70	2
SYK	6850	spleen associated tyrosine kinase	p72-Syk	9
LCP2	3937	lymphocyte cytosolic protein 2	SLP-76|SLP76	5
LAT	27040	linker for activation of T cells	IMD52|LAT1|pp36	16
PLCG1	5335	phospholipase C gamma 1	NCKAP3|PLC-II|PLC1|PLC148|PLCgamma1	20
PLCG2	5336	phospholipase C gamma 2	APLAID|FCAS3|PLC-IV|PLC-gamma-2	16
SH3BP2	6452	SH3 domain binding protein 2	3BP-2|3BP2|CRBM|CRPM|RES4-23	4
PIK3CA	5290	phosphatidylinositol-4,5-bisphosphate 3-kinase catalytic subunit alpha	CLAPO|CLOVE|CWS5|MCAP|MCM|MCMTC|PI3K|PI3K-alpha|p110-alpha	3
PIK3CB	5291	phosphatidylinositol-4,5-bisphosphate 3-kinase catalytic subunit beta	P110BETA|PI3K|PI3KBETA|PIK3C1	3

PIK3CD	5293	phosphatidylinositol-4,5-bisphosphate 3-kinase catalytic subunit delta	APDS|IMD14|P110DELTA|PI3K|p110D	1
PIK3CG	5294	phosphatidylinositol-4,5-bisphosphate 3-kinase catalytic subunit gamma	PI3CG|PI3K|PI3Kgamma|PIK3|p110gamma|p120-PI3K	7
PIK3R5	23533	phosphoinositide-3-kinase regulatory subunit 5	F730038I15Rik|FOAP-2|P101-PI3K|p101	17
PIK3R1	5295	phosphoinositide-3-kinase regulatory subunit 1	AGM7|GRB1|IMD36|p85|p85-ALPHA	5
PIK3R2	5296	phosphoinositide-3-kinase regulatory subunit 2	MPPH|MPPH1|P85B|p85|p85-BETA	19
PIK3R3	8503	phosphoinositide-3-kinase regulatory subunit 3	p55|p55-GAMMA|p55PIK	1
FYN	2534	FYN proto-oncogene, Src family tyrosine kinase	SLK|SYN|p59-FYN	6
SHC2	25759	SHC adaptor protein 2	SCK|SHCB|SLI	19
SHC4	399694	SHC adaptor protein 4	RaLP|SHCD	15
SHC3	53358	SHC adaptor protein 3	N-Shc|NSHC|RAI|SHCC	9
SHC1	6464	SHC adaptor protein 1	SHC|SHCA	1
GRB2	2885	growth factor receptor bound protein 2	ASH|EGFRBP-GRB2|Grb3-3|MST084|MSTP084|NCKAP2	17
SOS1	6654	SOS Ras/Rac guanine nucleotide exchange factor 1	GF1|GGF1|GINGF|HGF|NS4|SOS-1	2
SOS2	6655	SOS Ras/Rho guanine nucleotide exchange factor 2	NS9|SOS-2	14
HRAS	3265	HRas proto-oncogene, GTPase	C-BAS/HAS|C-H-RAS|C-HA-RAS1|CTLO|H-RASIDX|HAMSV|HRAS1|RASH1|p21ras	11

KRAS	3845	KRAS proto-oncogene, GTPase	'C-K-RAS|C-K-RAS|CFC2|K-RAS2A|K-RAS2B|K-RAS4A|K-RAS4B|K-Ras|K-Ras 2|KI-RAS|KRAS1|KRAS2|NS|NS3|OES|RALD|RASK2|c-Ki-ras|c-Ki-ras2	12
NRAS	4893	NRAS proto-oncogene, GTPase	ALPS4|CMNS|N-ras|NCMS|NRAS1|NS6	1
ARAF	369	A-Raf proto-oncogene, serine/threonine kinase	A-RAF|ARAF1|PKS2|RAFA1	X
BRAF	673	B-Raf proto-oncogene, serine/threonine kinase	B-RAF1|B-raf|BRAF1|NS7|RAFB1	7
RAF1	5894	Raf-1 proto-oncogene, serine/threonine kinase	CMD1NN|CRAF|NS5|Raf-1|c-Raf	3
MICA	100507436	MHC class I polypeptide-related sequence A	MIC-A|PERB11.1	6
MICB	4277	MHC class I polypeptide-related sequence B	PERB11.2	6
ULBP3	79465	UL16 binding protein 3	N2DL-3|NKG2DL3|RAET1N	6
ULBP2	80328	UL16 binding protein 2	ALCAN-alpha|N2DL2|NKG2DL2|RAET1H|RAET1L	6
ULBP1	80329	UL16 binding protein 1	N2DL-1|NKG2DL1|RAET1I	6
KLRK1	22914	killer cell lectin like receptor K1	CD314|D12S2489E|KLR|NKG2-D|NKG2D	12
HCST	10870	hematopoietic cell signal transducer	DAP10|KAP10|PIK3AP	19
CD48	962	CD48 molecule	BCM1|BLAST|BLAST1|MEM-102|SLAMF2|hCD48|mCD48	1

CD244	51744	CD244 molecule	2B4|NAIL|NKR2B4|Nmrk|SLAMF4	1
PPP3CA	5530	protein phosphatase 3 catalytic subunit alpha	ACCIID|CALN|CALNA|CALNA1|CCN1|CNA1|IECEE|IECEE1|PPP2B	4
PPP3CB	5532	protein phosphatase 3 catalytic subunit beta	CALNA2|CALNB|CNA2|PP2Bbeta	10
PPP3CC	5533	protein phosphatase 3 catalytic subunit gamma	CALNA3|CNA3|PP2Bgamma	8
CHP1	11261	calcineurin like EF-hand protein 1	CHP|SLC9A1BP|SPAX9|Sid470p|p22|p24	15
PPP3R1	5534	protein phosphatase 3 regulatory subunit B, alpha	CALNB1|CNB|CNB1	2
PPP3R2	5535	protein phosphatase 3 regulatory subunit B, beta	PPP3RL	9
CHP2	63928	calcineurin like EF-hand protein 2	-	16
NFAT5	10725	nuclear factor of activated T cells 5	NF-AT5|NFATL1|NFATZ|OREBP|TONEBP	16
NFATC1	4772	nuclear factor of activated T cells 1	NF-ATC|NF-ATc1.2|NFAT2|NFATc	18
NFATC2	4773	nuclear factor of activated T cells 2	NFAT1|NFATP	20
NFATC3	4775	nuclear factor of activated T cells 3	NF-AT4c|NFAT4|NFATX	16
NFATC4	4776	nuclear factor of activated T cells 4	NF-AT3|NF-ATC4|NFAT3	14
PRKCA	5578	protein kinase C alpha	AAG6|PKC-alpha|PKCA|PKCI+/-|PKCalpha|PRKACA	17
PRKCB	5579	protein kinase C beta	PKC-beta|PKCB|PKCI(2)|PKCbeta|PRKCB1|PRKCB2	16

PRKCG	5582	protein kinase C gamma	PKC-gamma|PKCC|PKCG|PKCI(3)|PKCgamma|SCA14	19
SH2D1B	117157	SH2 domain containing 1B	EAT2	1
SH2D1A	4068	SH2 domain containing 1A	DSHP|EBVS|IMD5|LYP|MTCP1|SAP|SAP/SH2D1A|XLP|XLPD|XLPD1	X
IFNGR1	3459	interferon gamma receptor 1	CD119|IFNGR|IMD27A|IMD27B	6
IFNGR2	3460	interferon gamma receptor 2	AF-1|IFGR2|IFNGT1|IMD28	21
IFNA1	3439	interferon alpha 1	IFL|IFN|IFN-ALPHA|IFN-alphaD|IFNA13|IFNA@|leIF D	9
IFNA2	3440	interferon alpha 2	IFN-alpha-2|IFN-alphaA|IFNA|IFNA2B|leIF A	9
IFNA4	3441	interferon alpha 4	IFN-alpha4a|INFA4	9
IFNA5	3442	interferon alpha 5	IFN-alpha-5|IFN-alphaG|INA5|INFA5|leIF G	9
IFNA6	3443	interferon alpha 6	IFN-alphaK	9
IFNA7	3444	interferon alpha 7	IFN-alphaJ|IFNA-J	9
IFNA8	3445	interferon alpha 8	IFN-alphaB	9
IFNA10	3446	interferon alpha 10	IFN-alphaC	9
IFNA13	3447	interferon alpha 13	-	9
IFNA14	3448	interferon alpha 14	IFN-alphaH|LEIF2H	9
IFNA16	3449	interferon alpha 16	IFN-alpha-16|IFN-alphaO	9
IFNA17	3451	interferon alpha 17	IFN-alphaI|IFNA|INFA|LEIF2C1	9
IFNA21	3452	interferon alpha 21	IFN-alphaI|LeIF F|leIF-F	9
IFNB1	3456	interferon beta 1	IFB|IFF|IFN-beta|IFNB	9
IFNAR1	3454	interferon alpha and beta receptor subunit 1	AVP|IFN-alpha-REC|IFNAR|IFNBR|IFRC	21
IFNAR2	3455	interferon alpha and beta receptor subunit 2	IFN-R|IFN-alpha-REC|IFNABR|IFNARB|IMD45	21
TNFSF10	8743	TNF superfamily member 10	APO2L|Apo-2L|CD253|TL2|TNLG6A|TRAIL	3

TNFRSF10	8793	TNF receptor superfamily member 10d	CD264|DCR2|TRAIL-R4|TRAILR4|TRUNDD	8
TNFRSF10	8794	TNF receptor superfamily member 10c	CD263|DCR1|DCR1-TNFR|LIT|TRAIL-R3|TRAILR3|TRID	8
TNFRSF10	8795	TNF receptor superfamily member 10b	CD262|DR5|KILLER|KILLER/DR5|TRAIL-R2|TRAILR2|TRICK2|TRICK2A|TRICK2B|TRICKB|ZTNFR9	8
TNFRSF10	8797	TNF receptor superfamily member 10a	APO2|CD261|DR4|TRAILR-1|TRAILR1	8
FASLG	356	Fas ligand	ALPS1B|APT1LG1|APTL|CD178|CD95-L|CD95L|FASL|TNFSF6|TNLG1A	1
FAS	355	Fas cell surface death receptor	ALPS1A|APO-1|APT1|CD95|FAS1|FASTM|TNFRSF6	10
GZMB	3002	granzyme B	C11|CCPI|CGL-1|CGL1|CSP-B|CSPB|CTLA1|CTSGL1|HLP|SECT	14
PRF1	5551	perforin 1	HPLH2|P1|PFP	10
CASP3	836	caspase 3	CPP32|CPP32B|SCA-1	4
BID	637	BH3 interacting domain death agonist	FP497	22
CD3D	915	CD3d molecule	CD3-DELTA|IMD19|T3D	11
CD3E	916	CD3e molecule	IMD18|T3E|TCRE	11
CD3G	917	CD3g molecule	CD3-GAMMA|IMD17|T3G	11
CD247	919	CD247 molecule	CD3-ZETA|CD3H|CD3Q|CD3Z|IMD25|T3Z|TCRZ	1
CD4	920	CD4 molecule	CD4mut	12
CD8A	925	CD8a molecule	CD8|Leu2|p32	2

CD8B	926	CD8b molecule	CD8B1|LEU2|LY3|LYT3|P37	2
PTPRC	5788	protein tyrosine phosphatase receptor type C	B220|CD45|CD45R|GP180|L-CA|LCA|LY5|T200	1
LCK	3932	LCK proto-oncogene, Src family tyrosine kinase	IMD22|LSK|YT16|p56lck|pp58lck	1
FYN	2534	FYN proto-oncogene, Src family tyrosine kinase	SLK|SYN|p59-FYN	6
ZAP70	7535	zeta chain of T cell receptor associated protein kinase 70	ADMIO2|IMD48|SRK|STCD|STD|TZK|ZAP-70	2
LCP2	3937	lymphocyte cytosolic protein 2	SLP-76|SLP76	5
LAT	27040	linker for activation of T cells	IMD52|LAT1|pp36	16
ITK	3702	IL2 inducible T cell kinase	EMT|LPFS1|LYK|PSCTK2	5
TEC	7006	tec protein tyrosine kinase	PSCTK4	4
NCK1	4690	NCK adaptor protein 1	NCK|NCKalpha|nck-1	3
NCK2	8440	NCK adaptor protein 2	GRB4|NCKbeta	2
VAV3	10451	vav guanine nucleotide exchange factor 3	-	1
VAV1	7409	vav guanine nucleotide exchange factor 1	VAV	19
VAV2	7410	vav guanine nucleotide exchange factor 2	VAV-2	9
GRAP2	9402	GRB2 related adaptor protein 2	GADS|GRAP-2|GRB2L|GRBLG|GRID|GRPL|GrbX|Grf40|Mona|P38	22
GRB2	2885	growth factor receptor bound protein 2	ASH|EGFRBP-GRB2|Grb3-3|MST084|MSTP084|NCKAP2	17
PAK1	5058	p21 (RAC1) activated kinase 1	IDDMSSD|PAKalpha|alpha-PAK|p65-PAK	11
PAK2	5062	p21 (RAC1) activated kinase 2	PAK65|PAKgamma	3

PAK3	5063	p21 (RAC1) activated kinase 3	ARA|MRX30|MRX47|OPHN3|PAK-3|PAK3beta|bPAK|beta-PAK	X
PAK4	10298	p21 (RAC1) activated kinase 4	-	19
PAK6	56924	p21 (RAC1) activated kinase 6	PAK5	15
PAK5	57144	p21 (RAC1) activated kinase 5	PAK7	20
RHOA	387	ras homolog family member A	ARH12|ARHA|EDFAOB|RHO12|RHOH12	3
CDC42	998	cell division cycle 42	CDC42Hs|G25K|TKS	1
PPP3CA	5530	protein phosphatase 3 catalytic subunit alpha	ACCIID|CALN|CALNA|CALNA1|CCN1|CNA1|IECEE|IECEE1|PPP2B	4
PPP3CB	5532	protein phosphatase 3 catalytic subunit beta	CALNA2|CALNB|CNA2|PP2Bbeta	10
PPP3CC	5533	protein phosphatase 3 catalytic subunit gamma	CALNA3|CNA3|PP2Bgamma	8
CHP1	11261	calcineurin like EF-hand protein 1	CHP|SLC9A1BP|SPAX9|Sid470p|p22|p24	15
PPP3R1	5534	protein phosphatase 3 regulatory subunit B, alpha	CALNB1|CNB|CNB1	2
PPP3R2	5535	protein phosphatase 3 regulatory subunit B, beta	PPP3RL	9
CHP2	63928	calcineurin like EF-hand protein 2	-	16
NFAT5	10725	nuclear factor of activated T cells 5	NF-AT5|NFATL1|NFATZ|OREBP|TONEBP	16
NFATC1	4772	nuclear factor of activated T cells 1	NF-ATC|NF-ATc1.2|NFAT2|NFATc	18
NFATC2	4773	nuclear factor of activated T cells 2	NFAT1|NFATP	20
NFATC3	4775	nuclear factor of activated T cells 3	NF-AT4c|NFAT4|NFATX	16

NFATC4	4776	nuclear factor of activated T cells 4	NF-AT3|NF-ATC4|NFAT3	14
SOS1	6654	SOS Ras/Rac guanine nucleotide exchange factor 1	GF1|GGF1|GINGF|HGF|NS4|SOS-1	2
SOS2	6655	SOS Ras/Rho guanine nucleotide exchange factor 2	NS9|SOS-2	14
HRAS	3265	HRas proto-oncogene, GTPase	C-BAS/HAS|C-H-RAS|C-HA-RAS1|CTLO|H-RASIDX|HAMSV|HRAS1|RASH1|p21ras	11
KRAS	3845	KRAS proto-oncogene, GTPase	'C-K-RAS|C-K-RAS|CFC2|K-RAS2A|K-RAS2B|K-RAS4A|K-RAS4B|K-Ras|K-Ras 2|KI-RAS|KRAS1|KRAS2|NS|NS3|OES|RALD|RASK2|c-Ki-ras|c-Ki-ras2	12
NRAS	4893	NRAS proto-oncogene, GTPase	ALPS4|CMNS|N-ras|NCMS|NRAS1|NS6	1
FOS	2353	Fos proto-oncogene, AP-1 transcription factor subunit	AP-1|C-FOS|p55	14
JUN	3725	Jun proto-oncogene, AP-1 transcription factor subunit	AP-1|AP1|c-Jun|cJUN|p39	1
CARD11	84433	caspase recruitment domain family member 11	BENTA|BIMP3|CARMA1|IMD11|IMD11A|PPBL	7
BCL10	8915	BCL10 immune signaling adaptor	CARMEN|CIPER|CLAP|IMD37|c-E10|mE10	1
MALT1	10892	MALT1 paracaspase	IMD12|MLT|MLT1|PCASP1	18
CHUK	1147	component of inhibitor of nuclear factor kappa B kinase complex	IKBKA|IKK-alpha|IKK1|IKKA|NFKBIKA|TCF16	10

IKBKB	3551	inhibitor of nuclear factor kappa B kinase subunit beta	IKK-beta|IKK2|IKKB|IMD15|IMD15A|IMD15B|NFKBIKB	8
IKBKG	8517	inhibitor of nuclear factor kappa B kinase regulatory subunit gamma	AMCBX1|EDAID1|FIP-3|FIP3|Fip3p|IKK-gamma|IKKAP1|IKKG|IMD33|IP|IP1|IP2|IPD2|NEMO|ZC2HC9	X
NFKB1	4790	nuclear factor kappa B subunit 1	CVID12|EBP-1|KBF1|NF-kB|NF-kB1|NF-kappa-B1|NF-kappaB|NF-kappabeta|NFKB-p105|NFKB-p50|NFkappaB	4
RELA	5970	RELA proto-oncogene, NF-kB subunit	CMCU|NFKB3|p65	11
NFKBIA	4792	NFKB inhibitor alpha	EDAID2|IKBA|MAD-3|NFKBI	14
NFKBIB	4793	NFKB inhibitor beta	IKBB|TRIP9	19
NFKBIE	4794	NFKB inhibitor epsilon	IKBE	6
CD28	940	CD28 molecule	Tp44	2
ICOS	29851	inducible T cell costimulator	AILIM|CD278|CVID1	2
CD40LG	959	CD40 ligand	CD154|CD40L|HIGM1|IGM|IMD3|T-BAM|TNFSF5|TRAP|gp39|hCD40L	X
PIK3R5	23533	phosphoinositide-3-kinase regulatory subunit 5	F730038I15Rik|FOAP-2|P101-PI3K|p101	17
PIK3R1	5295	phosphoinositide-3-kinase regulatory subunit 1	AGM7|GRB1|IMD36|p85|p85-ALPHA	5
PIK3R2	5296	phosphoinositide-3-kinase regulatory subunit 2	MPPH|MPPH1|P85B|p85|p85-BETA	19
PIK3R3	8503	phosphoinositide-3-kinase regulatory subunit 3	p55|p55-GAMMA|p55PIK	1
PIK3CA	5290	phosphatidylinositol-4,5-bisphosphate 3-kinase catalytic subunit alpha	CLAPO|CLOVE|CWS5|MCAP|MCM|MCMTC|PI3K|PI3K-alpha|p110-alpha	3

PIK3CB	5291	phosphatidylinositol-4,5-bisphosphate 3-kinase catalytic subunit beta	P110BETA|PI3K|PI3KBETA|PIK3C1	3
PIK3CD	5293	phosphatidylinositol-4,5-bisphosphate 3-kinase catalytic subunit delta	APDS|IMD14|P110DELTA|PI3K|p110D	1
PIK3CG	5294	phosphatidylinositol-4,5-bisphosphate 3-kinase catalytic subunit gamma	PI3CG|PI3K|PI3Kgamma|PIK3|p110gamma|p120-PI3K	7
AKT3	10000	AKT serine/threonine kinase 3	MPPH|MPPH2|PKB-GAMMA|PKBG|PRKBG|RAC-PK-gamma|RAC-gamma|STK-2	1
AKT1	207	AKT serine/threonine kinase 1	AKT|CWS6|PKB|PKB-ALPHA|PRKBA|RAC|RAC-ALPHA	14
AKT2	208	AKT serine/threonine kinase 2	HIHGHH|PKBB|PKBBETA|PRKBB|RAC-BETA	19
MAP3K8	1326	mitogen-activated protein kinase kinase kinase 8	AURA2|COT|EST|ESTF|MEKK8|TPL2|Tpl-2|c-COT	10
MAP3K14	9020	mitogen-activated protein kinase kinase kinase 14	FTDCR1B|HS|HSNIK|NIK	17
PDCD1	5133	programmed cell death 1	CD279|PD-1|PD1|SLEB2|hPD-1|hPD-l|hSLE1	2
CTLA4	1493	cytotoxic T-lymphocyte associated protein 4	ALPS5|CD|CD152|CELIAC3|CTLA-4|GRD4|GSE|IDDM12	2
PTPN6	5777	protein tyrosine phosphatase non-receptor type 6	HCP|HCPH|HPTP1C|PTP-1C|SH-PTP1|SHP-1|SHP-1L|SHP1	12
CBLC	23624	Cbl proto-oncogene C	CBL-3|CBL-SL|RNF57	19
CBL	867	Cbl proto-oncogene	C-CBL|CBL2|FRA11B|NSLL|RNF55	11

CBLB	868	Cbl proto-oncogene B	Cbl-b|Nbla00127|RNF56	3
IL2	3558	interleukin 2	IL-2|TCGF|lymphokine	4
IL4	3565	interleukin 4	BCGF-1|BCGF1|BSF-1|BSF1|IL-4	5
IL5	3567	interleukin 5	EDF|IL-5|TRF	5
IL10	3586	interleukin 10	CSIF|GVHDS|IL-10|IL10A|TGIF	1
IFNG	3458	interferon gamma	IFG|IFI	12
CSF2	1437	colony stimulating factor 2	CSF|GMCSF	5
TNF	7124	tumor necrosis factor	DIF|TNF-alpha|TNFA|TNFSF2|TNLG1F	6
CDK4	1019	cyclin dependent kinase 4	CMM3|PSK-J3	12
RASGRP1	10125	RAS guanyl releasing protein 1	CALDAG-GEFI|CALDAG-GEFII|IMD64|RASGRP	15
PDK1	5163	pyruvate dehydrogenase kinase 1	-	2
PLCG1	5335	phospholipase C gamma 1	NCKAP3|PLC-II|PLC1|PLC148|PLCgamma1	20
PRKCQ	5588	protein kinase C theta	PRKCT|nPKC-theta	10
TRAC	28755	T cell receptor alpha constant	IMD7|TCRA|TRA|TRCA	14
TRAJ1	28754	T cell receptor alpha joining 1 (non-functional)	-	14
TRAJ2	28753	T cell receptor alpha joining 2 (non-functional)	-	14
TRAJ3	28752	T cell receptor alpha joining 3	-	14
TRAJ4	28751	T cell receptor alpha joining 4	-	14
TRAJ5	28750	T cell receptor alpha joining 5	-	14
TRAJ6	28749	T cell receptor alpha joining 6	-	14
TRAJ7	28748	T cell receptor alpha joining 7	-	14

TRAJ8	28747	T cell receptor alpha joining 8	-	14
TRAJ9	28746	T cell receptor alpha joining 9	-	14
TRAJ10	28745	T cell receptor alpha joining 10	-	14
TRAJ11	28744	T cell receptor alpha joining 11	-	14
TRAJ12	28743	T cell receptor alpha joining 12	-	14
TRAJ13	28742	T cell receptor alpha joining 13	-	14
TRAJ14	28741	T cell receptor alpha joining 14	-	14
TRAJ15	28740	T cell receptor alpha joining 15	-	14
TRAJ16	28739	T cell receptor alpha joining 16	-	14
TRAJ17	28738	T cell receptor alpha joining 17	-	14
TRAJ18	28737	T cell receptor alpha joining 18	-	14
TRAJ19	28736	T cell receptor alpha joining 19 (non-functional)	-	14
TRAJ20	28735	T cell receptor alpha joining 20	-	14
TRAJ21	28734	T cell receptor alpha joining 21	-	14
TRAJ22	28733	T cell receptor alpha joining 22	-	14
TRAJ23	28732	T cell receptor alpha joining 23	-	14
TRAJ24	28731	T cell receptor alpha joining 24	-	14
TRAJ25	28730	T cell receptor alpha joining 25 (non-functional)	-	14
TRAJ26	28729	T cell receptor alpha joining 26	-	14
TRAJ27	28728	T cell receptor alpha joining 27	-	14
TRAJ28	28727	T cell receptor alpha joining 28	-	14
TRAJ29	28726	T cell receptor alpha joining 29	-	14
TRAJ30	28725	T cell receptor alpha joining 30	-	14
TRAJ31	28724	T cell receptor alpha joining 31	-	14

TRAJ32	28723	T cell receptor alpha joining 32	-	14
TRAJ33	28722	T cell receptor alpha joining 33	-	14
TRAJ34	28721	T cell receptor alpha joining 34	-	14
TRAJ35	28720	T cell receptor alpha joining 35 (non-functional)	-	14
TRAJ36	28719	T cell receptor alpha joining 36	-	14
TRAJ37	28718	T cell receptor alpha joining 37	-	14
TRAJ38	28717	T cell receptor alpha joining 38	-	14
TRAJ39	28716	T cell receptor alpha joining 39	-	14
TRAJ40	28715	T cell receptor alpha joining 40	-	14
TRAJ41	28714	T cell receptor alpha joining 41	-	14
TRAJ42	28713	T cell receptor alpha joining 42	-	14
TRAJ43	28712	T cell receptor alpha joining 43	-	14
TRAJ44	28711	T cell receptor alpha joining 44	-	14
TRAJ45	28710	T cell receptor alpha joining 45	-	14
TRAJ46	28709	T cell receptor alpha joining 46	-	14
TRAJ47	28708	T cell receptor alpha joining 47	-	14
TRAJ48	28707	T cell receptor alpha joining 48	-	14
TRAJ49	28706	T cell receptor alpha joining 49	-	14
TRAJ50	28705	T cell receptor alpha joining 50	-	14
TRAJ52	28703	T cell receptor alpha joining 52	-	14
TRAJ53	28702	T cell receptor alpha joining 53	-	14
TRAJ54	28701	T cell receptor alpha joining 54	-	14
TRAJ56	28699	T cell receptor alpha joining 56	-	14
TRAJ57	28698	T cell receptor alpha joining 57	-	14

TRAJ58	28697	T cell receptor alpha joining 58 (non-functional)	-	14
TRAJ59	28696	T cell receptor alpha joining 59 (non-functional)	-	14
TRAJ61	28694	T cell receptor alpha joining 61 (non-functional)	-	14
TRAV1-1	28693	T cell receptor alpha variable 1-1	TCRAV1S1|TCRAV7S1|TRAV11	14
TRAV1-2	28692	T cell receptor alpha variable 1-2	TCRAV1S2|TCRAV7S2|TRAV12	14
TRAV2	28691	T cell receptor alpha variable 2	TCRAV11S1|TCRAV2S1	14
TRAV3	28690	T cell receptor alpha variable 3	TCRAV16S1|TCRAV3S1	14
TRAV4	28689	T cell receptor alpha variable 4	TCRAV20S1|TCRAV4S1	14
TRAV5	28688	T cell receptor alpha variable 5	TCRAV15S1|TCRAV5S1	14
TRAV7	28686	T cell receptor alpha variable 7	TCRAV7S1	14
TRAV8-1	28685	T cell receptor alpha variable 8-1	TCRAV1S1|TCRAV8S1|TRAV81	14
TRAV8-2	28684	T cell receptor alpha variable 8-2	TCRAV1S5|TCRAV8S2|TRAV82	14
TRAV8-3	28683	T cell receptor alpha variable 8-3	TCRAV1S4|TCRAV8S3|TRAV83	14
TRAV8-4	28682	T cell receptor alpha variable 8-4	TCRAV1S2|TCRAV8S4|TRAV84	14
TRAV8-6	28680	T cell receptor alpha variable 8-6	TCRAV1S3|TCRAV8S6|TRAV86	14
TRAV8-7	28679	T cell receptor alpha variable 8-7 (pseudogene)	TCRAV8S7|TRAV87	14
TRAV9-1	28678	T cell receptor alpha variable 9-1	TCRAV9S1|TRAV91	14
TRAV9-2	28677	T cell receptor alpha variable 9-2	TCRAV22S1|TCRAV9S2|TRAV92	14
TRAV10	28676	T cell receptor alpha variable 10	TCRAV10S1|TCRAV24S1	14
TRAV12-1	28674	T cell receptor alpha variable 12-1	TCRAV12S1|TCRAV2S3|TRAV121	14
TRAV12-2	28673	T cell receptor alpha variable 12-2	TCRAV12S2|TCRAV2S1|TRAV122	14
TRAV12-3	28672	T cell receptor alpha variable 12-3	TCRAV12S3|TCRAV2S2|TRAV123	14
TRAV13-1	28671	T cell receptor alpha variable 13-1	TCRAV13S1|TCRAV8S1|TRAV131	14

TRAV13-2	28670	T cell receptor alpha variable 13-2	TCRAV13S2|TCRAV8S2|TRAV132	14
TRAV14D'	28669	T cell receptor alpha variable 14/delta variable 4	TCRAV6S1-hDV104S1|TRAV14/DV4|hADV14S1	14
TRAV16	28667	T cell receptor alpha variable 16	TCRAV16S1|TCRAV9S1	14
TRAV17	28666	T cell receptor alpha variable 17	TCRAV17S1|TCRAV3S1	14
TRAV18	28665	T cell receptor alpha variable 18	TCRAV18S1	14
TRAV19	28664	T cell receptor alpha variable 19	TCRAV12S1|TCRAV19S1	14
TRAV20	28663	T cell receptor alpha variable 20	TCRAV20S1|TCRAV30S1	14
TRAV21	28662	T cell receptor alpha variable 21	TCRAV21S1|TCRAV23S1	14
TRAV22	28661	T cell receptor alpha variable 22	TCRAV13S1|TCRAV22S1	14
TRAV23D'	28660	T cell receptor alpha variable 23/delta variable 6	TCRAV17S1|TRAV23/DV6|hADV23S1	14
TRAV24	28659	T cell receptor alpha variable 24	TCRAV18S1|TCRAV24S1	14
TRAV25	28658	T cell receptor alpha variable 25	TCRAV25S1|TCRAV32S1	14
TRAV26-1	28657	T cell receptor alpha variable 26-1	TCRAV26S1|TCRAV4S2|TRAV261	14
TRAV26-2	28656	T cell receptor alpha variable 26-2	TCRAV26S2|TCRAV4S1|TRAV262	14
TRAV27	28655	T cell receptor alpha variable 27	TCRAV10S1|TCRAV27S1	14
TRAV29D'	28653	T cell receptor alpha variable 29/delta variable 5	TCRA|TCRAV21S1|TRAV29/DV5|hADV29S1	14
TRAV30	28652	T cell receptor alpha variable 30	TCRAV29S1|TCRAV30S1	14
TRAV34	28648	T cell receptor alpha variable 34	TCRAV26S1|TCRAV34S1	14
TRAV35	28647	T cell receptor alpha variable 35	TCRAV25S1|TCRAV35S1	14
TRAV36D'	28646	T cell receptor alpha variable 36/delta variable 7	TCRAV28S1|TRAV36/DV7|hADV36S1	14
TRAV38-1	28644	T cell receptor alpha variable 38-1	TCRAV14S2|TCRAV38S1|TRAV381	14
TRAV38-2I	28643	T cell receptor alpha variable 38-2/delta variable 8	TCRAV14S1|TRAV382DV8|hADV38S2	14
TRAV39	28642	T cell receptor alpha variable 39	TCRAV27S1|TCRAV39S1	14

TRAV40	28641	T cell receptor alpha variable 40	TCRAV31S1|TCRAV40S1	14
TRAV41	28640	T cell receptor alpha variable 41	TCRAV19S1|TCRAV41S1	14
TRBC1	28639	T cell receptor beta constant 1	BV05S1J2.2|TCRB|TCRBC1	7
TRBC2	28638	T cell receptor beta constant 2	TCRBC2	7
TRBD1	28637	T cell receptor beta diversity 1	TCRBD1	7
TRBD2	28636	T cell receptor beta diversity 2	TCRBD2	7
TRBJ1-1	28635	T cell receptor beta joining 1-1	TCRBJ1S1|TRBJ11	7
TRBJ1-2	28634	T cell receptor beta joining 1-2	TCRBJ1S2|TRBJ12	7
TRBJ1-3	28633	T cell receptor beta joining 1-3	TCRBJ1S3|TRBJ13	7
TRBJ1-4	28632	T cell receptor beta joining 1-4	TCRBJ1S4|TRBJ14	7
TRBJ1-5	28631	T cell receptor beta joining 1-5	TCRBJ1S5|TRBJ15	7
TRBJ1-6	28630	T cell receptor beta joining 1-6	TCRBJ1S6|TRBJ16	7
TRBJ2-1	28629	T cell receptor beta joining 2-1	TCRBJ2S1|TRBJ21	7
TRBJ2-2	28628	T cell receptor beta joining 2-2	TCRBJ2S2|TRBJ22	7
TRBJ2-3	28626	T cell receptor beta joining 2-3	TCRBJ2S3|TRBJ23	7
TRBJ2-4	28625	T cell receptor beta joining 2-4	TCRBJ2S4|TRBJ24	7
TRBJ2-5	28624	T cell receptor beta joining 2-5	TCRBJ2S5|TRBJ25	7
TRBJ2-6	28623	T cell receptor beta joining 2-6	TCRBJ2S6|TRBJ26	7
TRBJ2-7	28622	T cell receptor beta joining 2-7	TCRBJ2S7|TRBJ27	7
TRBV2	28620	T cell receptor beta variable 2	TCRBV22S1A2N1T|TCRBV2S1	7
TRBV3-1	28619	T cell receptor beta variable 3-1	TCRBV3S1|TCRBV9S1A1T|TRBV31	7
TRBV4-1	28617	T cell receptor beta variable 4-1	BV07S1J2.7|TCRBV4S1|TCRBV7S1A1N2T|TRBV41	7
TRBV4-2	28616	T cell receptor beta variable 4-2	TCRBV4S2|TCRBV7S3A2|TCRBV7S3A2T|TRBV42	7
TRBV4-3	28615	T cell receptor beta variable 4-3	TCRBV4S3|TCRBV7S2A1N4T|TRBV43	7

TRBV5-1	28614	T cell receptor beta variable 5-1	TCRBV5S1|TCRBV5S1A1T|TRBV51	7
TRBV5-4	28611	T cell receptor beta variable 5-4	TCRBV5S4|TCRBV5S6A3N2T|TRBV54	7
TRBV5-5	28610	T cell receptor beta variable 5-5	TCRBV5S3A2T|TCRBV5S5|TRBV55	7
TRBV5-6	28609	T cell receptor beta variable 5-6	TCRBV5S2|TCRBV5S6|TRBV56	7
TRBV5-7	28608	T cell receptor beta variable 5-7 (non-functional)	TCRBV5S7|TCRBV5S7P|TRBV57	7
TRBV5-8	28607	T cell receptor beta variable 5-8	TCRBV5S4A2T|TCRBV5S8|TRBV58	7
TRBV6-1	28606	T cell receptor beta variable 6-1	TCRBV13S3|TCRBV6S1|TRBV61	7
TRBV6-2	28605	T cell receptor beta variable 6-2	TCRBV13S2|TCRBV13S2A1T|TCRBV6S2|TRBV62	7
TRBV6-3	28604	T cell receptor beta variable 6-3	TCRBV13S9/13S2A1T|TCRBV6S3|TRBV63	7
TRBV6-4	28603	T cell receptor beta variable 6-4	TCRBV13S5|TCRBV6S4|TRBV64	7
TRBV6-5	28602	T cell receptor beta variable 6-5	TCRBV13S1|TCRBV6S5|TRBV65	7
TRBV6-6	28601	T cell receptor beta variable 6-6	TCRBV13S6A2T|TCRBV6S6|TRBV66	7
TRBV6-7	28600	T cell receptor beta variable 6-7 (non-functional)	TCRBV13S8P|TCRBV6S7|TRBV67	7
TRBV6-8	28599	T cell receptor beta variable 6-8	TCRBV13S7P|TCRBV6S8|TRBV68	7
TRBV6-9	28598	T cell receptor beta variable 6-9	TCRBV13S4|TCRBV6S9|TRBV69	7
TRBV7-2	28596	T cell receptor beta variable 7-2	TCRBV6S5A1N1|TCRBV6S5A2|TCRBV7S2|TRBV72	7
TRBV7-3	28595	T cell receptor beta variable 7-3	TCRBV6S1A1N1|TCRBV7S3|TRBV73	7
TRBV7-4	28594	T cell receptor beta variable 7-4	TCRBV6S8A2T|TCRBV7S4|TRBV74	7
TRBV7-6	28592	T cell receptor beta variable 7-6	TCRBV6S3A1N1T|TCRBV7S6|TRBV76	7

TRBV7-7	28591	T cell receptor beta variable 7-7	TCRBV6S6A2T|TCRBV7S7|TRBV77	7
TRBV7-8	28590	T cell receptor beta variable 7-8	TCRBV6S2A1N1T|TCRBV7S8|TRBV78	7
TRBV7-9	28589	T cell receptor beta variable 7-9	TCRB|TCRBV6S4A1|TCRBV7S9|TRBV79	7
TRBV9	28586	T cell receptor beta variable 9	TCRBV1S1A1N1|TCRBV9S1	7
TRBV10-1	28585	T cell receptor beta variable 10-1	TCRBV10S1|TCRBV12S2|TCRBV12S2A1T|TRBV101	7
TRBV10-2	28584	T cell receptor beta variable 10-2	TCRBV10S2|TCRBV12S3|TRBV102	7
TRBV10-3	28583	T cell receptor beta variable 10-3	TCRBV10S3|TCRBV12S1A1N2|TRBV103	7
TRBV11-1	28582	T cell receptor beta variable 11-1	TCRBV11S1|TCRBV21S1|TRBV111	7
TRBV11-2	28581	T cell receptor beta variable 11-2	TCRBV11S2|TCRBV21S3A2N2T|TRBV112	7
TRBV11-3	28580	T cell receptor beta variable 11-3	TCRBV11S3|TCRBV21S2A2|TRBV113	7
TRBV12-3	28577	T cell receptor beta variable 12-3	TCRBV12S3|TCRBV8S1|TRBV123	7
TRBV12-4	28576	T cell receptor beta variable 12-4	TCRBV12S4|TCRBV8S2A1T|TRBV124	7
TRBV12-5	28575	T cell receptor beta variable 12-5	TCRBV12S5|TCRBV8S3|TRBV125	7
TRBV13	28574	T cell receptor beta variable 13	TCRBV13S1|TCRBV23S1A2T	7
TRBV14	28573	T cell receptor beta variable 14	TCRBV14S1|TCRBV16S1A1N1	7
TRBV15	28572	T cell receptor beta variable 15	TCRBV15S1|TCRBV24S1A3T	7
TRBV16	28571	T cell receptor beta variable 16	BV25S1J1.2|TCRB|TCRBV16S1|TCRBV25S1|TCRBV25S1A2PT	7
TRBV17	28570	T cell receptor beta variable 17 (non-functional)	TCRBV17S1|TCRBV26S1P	7
TRBV18	28569	T cell receptor beta variable 18	TCRBV18S1	7
TRBV19	28568	T cell receptor beta variable 19	TCRBV17S1A1T|TCRBV19S1	7

TRBV20-1	28567	T cell receptor beta variable 20-1	TCRBV20S1|TCRBV2S1|TRBV201	7
TRBV24-1	28563	T cell receptor beta variable 24-1	TCRBV15S1|TCRBV24S1|TRBV241	7
TRBV25-1	28562	T cell receptor beta variable 25-1	TCRBV11S1A1T|TCRBV25S1|TRBV251	7
TRBV27	28560	T cell receptor beta variable 27	TCRBV14S1|TCRBV27S1	7
TRBV28	28559	T cell receptor beta variable 28	TCRBV28S1|TCRBV3S1	7
TRBV29-1	28558	T cell receptor beta variable 29-1	TCRBV29S1|TCRBV4S1A1T|TRBV291	7
TRBV30	28557	T cell receptor beta variable 30	TCRBV20S1A1N2|TCRBV30S1	7
TRDC	28526	T cell receptor delta constant	TCRD	14
TRDD1	28525	T cell receptor delta diversity 1	-	14
TRDD2	28524	T cell receptor delta diversity 2	-	14
TRDD3	28523	T cell receptor delta diversity 3	TCRD	14
TRDJ1	28522	T cell receptor delta joining 1	TCRD	14
TRDJ2	28521	T cell receptor delta joining 2	-	14
TRDJ3	28520	T cell receptor delta joining 3	-	14
TRDJ4	28519	T cell receptor delta joining 4	-	14
TRDV1	28518	T cell receptor delta variable 1	hDV101S1	14
TRDV2	28517	T cell receptor delta variable 2	hDV102S1	14
TRDV3	28516	T cell receptor delta variable 3	hDV103S1	14
TRGV9	6983	T cell receptor gamma variable 9	TCRGV9|TRGC1|V2	7
TRGV8	6982	T cell receptor gamma variable 8	TCRGV8|V1S8	7
TRGV5	6978	T cell receptor gamma variable 5	TCRGV5|V1S5	7
TRGV4	6977	T cell receptor gamma variable 4	TCRGV4|V1S4	7
TRGV3	6976	T cell receptor gamma variable 3	TCRGV3|V1S3	7
TRGV2	6974	T cell receptor gamma variable 2	TCRGV2|VIS2	7

TRGJP2	6972	T cell receptor gamma joining P2	JP2|TCRGJP2	7
TRGJP1	6971	T cell receptor gamma joining P1	JP1|TCRGJP1	7
TRGJP	6970	T cell receptor gamma joining P	JP|TCRGJP	7
TRGJ2	6969	T cell receptor gamma joining 2	J2|TCRGJ2	7
TRGJ1	6968	T cell receptor gamma joining 1	J1|TCRGJ1	7
TRGC2	6967	T cell receptor gamma constant 2	TCRGC2|TRGC2(2X)|TRGC2(3X)	7
TRGC1	6966	T cell receptor gamma constant 1	C1|TCRG|TCRGC1	7
TRAV6	6956	T cell receptor alpha variable 6	TCRAV5S1|TCRAV6S1	14
BMP1	649	bone morphogenetic protein 1	OI13|PCOLC|PCP|PCP2|TLD	8
BMP10	27302	bone morphogenetic protein 10	-	2
BMP15	9210	bone morphogenetic protein 15	GDF9B|ODG2|POF4	X
BMP2	650	bone morphogenetic protein 2	BDA2|BMP2A|SSFSC	20
BMP3	651	bone morphogenetic protein 3	BMP-3A	4
BMP4	652	bone morphogenetic protein 4	BMP2B|BMP2B1|MCOPS6|OFC11|ZYME	14
BMP5	653	bone morphogenetic protein 5	-	6
BMP6	654	bone morphogenetic protein 6	VGR|VGR1	6
BMP7	655	bone morphogenetic protein 7	OP-1	20
BMP8A	353500	bone morphogenetic protein 8a	OP-2	1
BMP8B	656	bone morphogenetic protein 8b	BMP8|OP2	1
GDF1	2657	growth differentiation factor 1	CERS1|CHTD6|DORV|DTGA3|LAG1|LASS1|RAI|UOG1	19

GDF10	2662	growth differentiation factor 10	BIP|BMP-3b|BMP3B	10
GDF11	10220	growth differentiation factor 11	BMP-11|BMP11	12
GDF15	9518	growth differentiation factor 15	GDF-15|MIC-1|MIC1|NAG-1|PDF|PLAB|PTGFB	19
GDF2	2658	growth differentiation factor 2	BMP-9|BMP9|HHT5	10
GDF3	9573	growth differentiation factor 3	KFS3|MCOP7|MCOPCB6	12
GDF5	8200	growth differentiation factor 5	BDA1C|BMP-14|BMP14|CDMP1|DUPANS|LAP-4|LAP4|OS5|SYM1B|SYNS2	20
GDF6	392255	growth differentiation factor 6	BMP-13|BMP13|CDMP2|KFM|KFS|KFS1|KFSL|SGM1|SYNS4	8
GDF7	151449	growth differentiation factor 7	BMP12	2
GDF9	2661	growth differentiation factor 9	POF14	5
GDNF	2668	glial cell derived neurotrophic factor	ATF|ATF1|ATF2|HFB1-GDNF|HSCR3	5
INHA	3623	inhibin subunit alpha	-	2
INHBA	3624	inhibin subunit beta A	EDF|FRP	7
INHBB	3625	inhibin subunit beta B	-	2
INHBC	3626	inhibin subunit beta C	IHBC	12
INHBE	83729	inhibin subunit beta E	-	12
LEFTY1	10637	left-right determination factor 1	LEFTB|LEFTYB	1
LEFTY2	7044	left-right determination factor 2	EBAF|LEFTA|LEFTYA|TGFB4	1
NODAL	4838	nodal growth differentiation factor	HTX5	10

TGFB1	7040	transforming growth factor beta 1	CED|DPD1|IBDIMDE|LAP|TGF-beta1|TGFB|TGFbeta	19
TGFB2	7042	transforming growth factor beta 2	G-TSF|LDS4|TGF-beta2	1
TGFB3	7043	transforming growth factor beta 3	ARVD|ARVD1|LDS5|RNHF|TGF-beta3	14
ACVR1B	91	activin A receptor type 1B	ACTRIB|ACVRLK4|ALK4|SKR2	12
ACVR1C	130399	activin A receptor type 1C	ACVRLK7|ALK7	2
ACVR2A	92	activin A receptor type 2A	ACTRII|ACVR2	2
ACVR2B	93	activin A receptor type 2B	ACTRIIB|ActR-IIB|HTX4	3
ACVRL1	94	activin A receptor like type 1	ACVRLK1|ALK-1|ALK1|HHT|HHT2|ORW2|SKR3|TSR-I	12
AMHR2	269	anti-Mullerian hormone receptor type 2	AMHR|MISR2|MISRII|MRII	12
BMPR1A	657	bone morphogenetic protein receptor type 1A	10q23del|ACVRLK3|ALK3|CD292|SKR5	10
BMPR1B	658	bone morphogenetic protein receptor type 1B	ALK-6|ALK6|AMDD|BDA1D|BDA2|CDw293	4
BMPR2	659	bone morphogenetic protein receptor type 2	BMPR-II|BMPR3|BMR2|BRK-3|POVD1|PPH1|T-ALK	2
TGFBR1	7046	transforming growth factor beta receptor 1	AAT5|ACVRLK4|ALK-5|ALK5|ESS1|LDS1|LDS1A|LDS2A|MSSE|SKR4|TBR-i|TBRI|TGFR-1|tbetaR-I	9
TGFBR2	7048	transforming growth factor beta receptor 2	AAT3|FAA3|LDS1B|LDS2|LDS2B|MFS2|RIIC|TAAD2|TBR-ii|TBRII|TGFR-2|TGFbeta-RII	3

TGFBR3	7049	transforming growth factor beta receptor 3	BGCAN|betaglycan	1
TNFRSF11	4982	TNF receptor superfamily member 11b	OCIF|OPG|PDB5|TR1	8
TNFSF10	8743	TNF superfamily member 10	APO2L|Apo-2L|CD253|TL2|TNLG6A|TRAIL	3
TNFSF11	8600	TNF superfamily member 11	CD254|ODF|OPGL|OPTB2|RANKL|TNLG6B|TRANCE|hRANKL2|sOdf	13
TNFSF12	8742	TNF superfamily member 12	APO3L|DR3LG|TNLG4A|TWEAK	17
TNFSF13	8741	TNF superfamily member 13	APRIL|CD256|TALL-2|TALL2|TNLG7B|TRDL-1|UNQ383/PRO715|ZTNF2	17
TNFSF13B	10673	TNF superfamily member 13b	BAFF|BLYS|CD257|DTL|TALL-1|TALL1|THANK|TNFSF20|TNLG7A|ZTNF4	13
TNFSF14	8740	TNF superfamily member 14	CD258|HVEML|LIGHT|LTg	19
TNFSF15	9966	TNF superfamily member 15	TL1|TL1A|TNLG1B|VEGI|VEGI192A	9
TNFSF18	8995	TNF superfamily member 18	AITRL|GITRL|TL6|TNLG2A|hGITRL	1
TNFSF4	7292	TNF superfamily member 4	CD134L|CD252|GP34|OX-40L|OX4OL|TNLG2B|TXGP1	1
TNFSF8	944	TNF superfamily member 8	CD153|CD30L|CD30LG|TNLG3A	9
TNFSF9	8744	TNF superfamily member 9	4-1BB-L|CD137L|TNLG5A	19
TNFRSF10	8795	TNF receptor superfamily member 10b	CD262|DR5|KILLER|KILLER/DR5|TRAIL-R2|TRAILR2|TRICK2|TRICK2A|TRICK2B|TRICKB|ZTNFR9	8
TNFRSF10	8794	TNF receptor superfamily member 10c	CD263|DCR1|DCR1-TNFR|LIT|TRAIL-R3|TRAILR3|TRID	8

TNFRSF10	8793	TNF receptor superfamily member 10d	CD264|DCR2|TRAIL-R4|TRAILR4|TRUNDD	8
TNFRSF11	8792	TNF receptor superfamily member 11a	CD265|FEO|LOH18CR1|ODFR|OFE|OPTB7|OSTS|PDB2|RANK|TRANCER	18
TNFRSF12	51330	TNF receptor superfamily member 12A	CD266|FN14|TWEAKR	16
TNFRSF13	23495	TNF receptor superfamily member 13B	CD267|CVID|CVID2|IGAD2|RYZN|TACI|TNFRSF14B	17
TNFRSF13	115650	TNF receptor superfamily member 13C	BAFF-R|BAFFR|BROMIX|CD268|CVID4|prolixin	22
TNFRSF14	8764	TNF receptor superfamily member 14	ATAR|CD270|HVEA|HVEM|LIGHTR|TR2	1
TNFRSF17	608	TNF receptor superfamily member 17	BCM|BCMA|CD269|TNFRSF13A	16
TNFRSF18	8784	TNF receptor superfamily member 18	AITR|CD357|ENERGEN|GITR|GITR-D	1
TNFRSF19	55504	TNF receptor superfamily member 19	TAJ|TAJ-alpha|TRADE|TROY	13
TNFRSF1A	7132	TNF receptor superfamily member 1A	CD120a|FPF|TBP1|TNF-R|TNF-R-I|TNF-R55|TNFAR|TNFR1|TNFR55|TNFR60|p55|p55-R|p60	12
TNFRSF1E	7133	TNF receptor superfamily member 1B	CD120b|TBPII|TNF-R-II|TNF-R75|TNFBR|TNFR1B|TNFR2|TNFR80|p75|p75TNFR	1
TNFRSF21	27242	TNF receptor superfamily member 21	BM-018|CD358|DR6	6
TNFRSF25	8718	TNF receptor superfamily member 25	APO-3|DDR3|DR3|GEF720|LARD|PLEKHG5|TNFRSF12|TR3|TRAMP|WSL-1|WSL-LR	1

TNFRSF4	7293	TNF receptor superfamily member 4	ACT35|CD134|IMD16|OX40|TXGP1L	1
TNFRSF6E	8771	TNF receptor superfamily member 6b	DCR3|DJ583P15.1.1|M68|M68E|TR6	20
TNFRSF8	943	TNF receptor superfamily member 8	CD30|D1S166E|Ki-1	1
TNFRSF9	3604	TNF receptor superfamily member 9	4-1BB|CD137|CDw137|ILA	1

database

Category
Antigen_Processing_and_Presentation
Antigen_Processing_and_Presentation
Antigen_Processing_and_Presentation
Antigen_Processing_and_Presentation
Antigen_Processing_and_Presentation
Antigen_Processing_and_Presentation
Antigen_Processing_and_Presentation
Antigen_Processing_and_Presentation
Antigen_Processing_and_Presentation
Antigen_Processing_and_Presentation
Antigen_Processing_and_Presentation
Antigen_Processing_and_Presentation
Antigen_Processing_and_Presentation
Antigen_Processing_and_Presentation
Antigen_Processing_and_Presentation
Antigen_Processing_and_Presentation
Antigen_Processing_and_Presentation
Antigen_Processing_and_Presentation
Antigen_Processing_and_Presentation
Antigen_Processing_and_Presentation
Antigen_Processing_and_Presentation
Antigen_Processing_and_Presentation
Antigen_Processing_and_Presentation

Antigen_Processing_and_Presentation

Antigen_Processing_and_Presentation

Antigen_Processing_and_Presentation

Antigen_Processing_and_Presentation

Antigen_Processing_and_Presentation

Antigen_Processing_and_Presentation

Antigen_Processing_and_Presentation

Antigen_Processing_and_Presentation

Antigen_Processing_and_Presentation

Antigen_Processing_and_Presentation

Antigen_Processing_and_Presentation

Antigen_Processing_and_Presentation

Antigen_Processing_and_Presentation

Antigen_Processing_and_Presentation

Antigen_Processing_and_Presentation

Antigen_Processing_and_Presentation

Antigen_Processing_and_Presentation

Antigen_Processing_and_Presentation

Antigen_Processing_and_Presentation

Antigen_Processing_and_Presentation

Antigen_Processing_and_Presentation

Antigen_Processing_and_Presentation

Antigen_Processing_and_Presentation

Antigen_Processing_and_Presentation

Antigen_Processing_and_Presentation

Antigen_Processing_and_Presentation

Antigen_Processing_and_Presentation

Antigen_Processing_and_Presentation

Antigen_Processing_and_Presentation

Antigen_Processing_and_Presentation

Antigen_Processing_and_Presentation

Antigen_Processing_and_Presentation

Antigen_Processing_and_Presentation

Antigen_Processing_and_Presentation

Antigen_Processing_and_Presentation

Antigen_Processing_and_Presentation

Antigen_Processing_and_Presentation

Antigen_Processing_and_Presentation

Antigen_Processing_and_Presentation

Antigen_Processing_and_Presentation

Antigen_Processing_and_Presentation

Antigen_Processing_and_Presentation

Antigen_Processing_and_Presentation

Antigen_Processing_and_Presentation

Antigen_Processing_and_Presentation

Antigen_Processing_and_Presentation

Antigen_Processing_and_Presentation

Antigen_Processing_and_Presentation

Antigen_Processing_and_Presentation

Antigen_Processing_and_Presentation

Antigen_Processing_and_Presentation

Antigen_Processing_and_Presentation

Antigen_Processing_and_Presentation

Antigen_Processing_and_Presentation

Antigen_Processing_and_Presentation

Antigen_Processing_and_Presentation

Antigen_Processing_and_Presentation

Antigen_Processing_and_Presentation

Antigen_Processing_and_Presentation

Antigen_Processing_and_Presentation

Antigen_Processing_and_Presentation

Antigen_Processing_and_Presentation

Antigen_Processing_and_Presentation

Antigen_Processing_and_Presentation

Antigen_Processing_and_Presentation

Antigen_Processing_and_Presentation

Antigen_Processing_and_Presentation

Antigen_Processing_and_Presentation

Antigen_Processing_and_Presentation

Antigen_Processing_and_Presentation

Antigen_Processing_and_Presentation

Antigen_Processing_and_Presentation

Antigen_Processing_and_Presentation

Antigen_Processing_and_Presentation

Antigen_Processing_and_Presentation

Antigen_Processing_and_Presentation

Antigen_Processing_and_Presentation

Antigen_Processing_and_Presentation

Antigen_Processing_and_Presentation

Antigen_Processing_and_Presentation

Antigen_Processing_and_Presentation

Antigen_Processing_and_Presentation

Antigen_Processing_and_Presentation

Antigen_Processing_and_Presentation

Antigen_Processing_and_Presentation

Antigen_Processing_and_Presentation

Antigen_Processing_and_Presentation

Antigen_Processing_and_Presentation

Antigen_Processing_and_Presentation

Antigen_Processing_and_Presentation

Antigen_Processing_and_Presentation

Antigen_Processing_and_Presentation

Antigen_Processing_and_Presentation

Antigen_Processing_and_Presentation

Antigen_Processing_and_Presentation

Antigen_Processing_and_Presentation

Antigen_Processing_and_Presentation

Antigen_Processing_and_Presentation

Antigen_Processing_and_Presentation

Antigen_Processing_and_Presentation

Antigen_Processing_and_Presentation

Antigen_Processing_and_Presentation

Antigen_Processing_and_Presentation

Antigen_Processing_and_Presentation

Antigen_Processing_and_Presentation

Antigen_Processing_and_Presentation

Antigen_Processing_and_Presentation

Antigen_Processing_and_Presentation

Antigen_Processing_and_Presentation

Antigen_Processing_and_Presentation

Antigen_Processing_and_Presentation

Antigen_Processing_and_Presentation

Antigen_Processing_and_Presentation

Antigen_Processing_and_Presentation

Antigen_Processing_and_Presentation

Antigen_Processing_and_Presentation

Antigen_Processing_and_Presentation

Antigen_Processing_and_Presentation

Antigen_Processing_and_Presentation

Antigen_Processing_and_Presentation

Antigen_Processing_and_Presentation

Antigen_Processing_and_Presentation

Antigen_Processing_and_Presentation

Antigen_Processing_and_Presentation

Antimicrobials

Antimicrobials

Antimicrobials

Antimicrobials

Antimicrobials

Antimicrobials

Antimicrobials

Antimicrobials

Antimicrobials

Antimicrobials

Antimicrobials

Antimicrobials

Antimicrobials

Antimicrobials

Antimicrobials

Antimicrobials

Antimicrobials

Antimicrobials

Antimicrobials

Antimicrobials

Antimicrobials

Antimicrobials

Antimicrobials

Antimicrobials

Antimicrobials

Antimicrobials

Antimicrobials

Antimicrobials

Antimicrobials

Antimicrobials

Antimicrobials

Antimicrobials
Antimicrobials
Antimicrobials

Antimicrobials

Antimicrobials

Antimicrobials

Antimicrobials

Antimicrobials

Antimicrobials

Antimicrobials

Antimicrobials

Antimicrobials

Antimicrobials

Antimicrobials

Antimicrobials

Antimicrobials

Antimicrobials

Antimicrobials

Antimicrobials

Antimicrobials

Antimicrobials

Antimicrobials

Antimicrobials

Antimicrobials

Antimicrobials

Antimicrobials

Antimicrobials

Antimicrobials

Antimicrobials

Antimicrobials

Antimicrobials

Antimicrobials

Antimicrobials

Antimicrobials

Antimicrobials

Antimicrobials

Antimicrobials

Antimicrobials

Antimicrobials

Antimicrobials

Antimicrobials

Antimicrobials

Antimicrobials

Antimicrobials

Antimicrobials

Antimicrobials

Antimicrobials

Antimicrobials

Antimicrobials

Antimicrobials

Antimicrobials

Antimicrobials

Antimicrobials

Antimicrobials

Antimicrobials

Antimicrobials

Antimicrobials

Antimicrobials

Antimicrobials

Antimicrobials

Antimicrobials

Antimicrobials

Antimicrobials

Antimicrobials

Antimicrobials

Antimicrobials

Antimicrobials

Antimicrobials

Antimicrobials

Antimicrobials

Antimicrobials

Antimicrobials

Antimicrobials

Antimicrobials

Antimicrobials

Antimicrobials

Antimicrobials

Antimicrobials

Antimicrobials

Antimicrobials

Antimicrobials

Antimicrobials

Antimicrobials

Antimicrobials

Antimicrobials

Antimicrobials

Antimicrobials

Antimicrobials

Antimicrobials

Antimicrobials

Antimicrobials

Antimicrobials

Antimicrobials

Antimicrobials

Antimicrobials

Antimicrobials

Antimicrobials

Antimicrobials

Antimicrobials

Antimicrobials

Antimicrobials

Antimicrobials

Antimicrobials

Antimicrobials

Antimicrobials

Antimicrobials

Antimicrobials

Antimicrobials

Antimicrobials

Antimicrobials

Antimicrobials

Antimicrobials

Antimicrobials

Antimicrobials

Antimicrobials

Antimicrobials

Antimicrobials

Antimicrobials

Antimicrobials

Antimicrobials

Antimicrobials

Antimicrobials

Antimicrobials

Antimicrobials

Antimicrobials

Antimicrobials

Antimicrobials

Antimicrobials

Antimicrobials

Antimicrobials

Antimicrobials

Antimicrobials

Antimicrobials

Antimicrobials

Antimicrobials

Antimicrobials

Antimicrobials

Antimicrobials

Antimicrobials

Antimicrobials

Antimicrobials

Antimicrobials

Antimicrobials

Antimicrobials

Antimicrobials

Antimicrobials

Antimicrobials

Antimicrobials

Antimicrobials

Antimicrobials

Antimicrobials

Antimicrobials

Antimicrobials

Antimicrobials

Antimicrobials

Antimicrobials

Antimicrobials

Antimicrobials

Antimicrobials

Antimicrobials

Antimicrobials

Antimicrobials

Antimicrobials

Antimicrobials

Antimicrobials

Antimicrobials

Antimicrobials

Antimicrobials

Antimicrobials

Antimicrobials

Antimicrobials

Antimicrobials

Antimicrobials

Antimicrobials

Antimicrobials

Antimicrobials

Antimicrobials

Antimicrobials

Antimicrobials

Antimicrobials

Antimicrobials

Antimicrobials

Antimicrobials

Antimicrobials

Antimicrobials

Antimicrobials

Antimicrobials

Antimicrobials

Antimicrobials

Antimicrobials

Antimicrobials

Antimicrobials

Antimicrobials

Antimicrobials

Antimicrobials
Antimicrobials

Antimicrobials

Antimicrobials

Antimicrobials

Antimicrobials

Antimicrobials

Antimicrobials

Antimicrobials

Antimicrobials

Antimicrobials

Antimicrobials

Antimicrobials

Antimicrobials

Antimicrobials

Antimicrobials

Antimicrobials

Antimicrobials

Antimicrobials

Antimicrobials

Antimicrobials

Antimicrobials

Antimicrobials

Antimicrobials

Antimicrobials

Antimicrobials

Antimicrobials

Antimicrobials

Antimicrobials

Antimicrobials

Antimicrobials

Antimicrobials

Antimicrobials

Antimicrobials

Antimicrobials

Antimicrobials

Antimicrobials

Antimicrobials

Antimicrobials

Antimicrobials

Antimicrobials

Antimicrobials

Antimicrobials

Antimicrobials

Antimicrobials

Antimicrobials

Antimicrobials

Antimicrobials

Antimicrobials

Antimicrobials

Antimicrobials

Antimicrobials

Antimicrobials

Antimicrobials

Antimicrobials

Antimicrobials

Antimicrobials

Antimicrobials

Antimicrobials

Antimicrobials

Antimicrobials

Antimicrobials

Antimicrobials

Antimicrobials

Antimicrobials

Antimicrobials

Antimicrobials

Antimicrobials

Antimicrobials

Antimicrobials

Antimicrobials

Antimicrobials

Antimicrobials

Antimicrobials

Antimicrobials

Antimicrobials

Antimicrobials

Antimicrobials

Antimicrobials

Antimicrobials

Antimicrobials

Antimicrobials

Antimicrobials

Antimicrobials

Antimicrobials

Antimicrobials

Antimicrobials

Antimicrobials

Antimicrobials

Antimicrobials

Antimicrobials

Antimicrobials

Antimicrobials

Antimicrobials

Antimicrobials

Antimicrobials

Antimicrobials

Antimicrobials

Antimicrobials
Antimicrobials
Antimicrobials
Antimicrobials
Antimicrobials
Antimicrobials
Antimicrobials
Antimicrobials
Antimicrobials
Antimicrobials
Antimicrobials
Antimicrobials
Antimicrobials
Antimicrobials
Antimicrobials
Antimicrobials
Antimicrobials
Antimicrobials

Antimicrobials

Antimicrobials

Antimicrobials

Antimicrobials

Antimicrobials

Antimicrobials

Antimicrobials

Antimicrobials

Antimicrobials

Antimicrobials

Antimicrobials

Antimicrobials

Antimicrobials

Antimicrobials

Antimicrobials

Antimicrobials

Antimicrobials

Antimicrobials

Antimicrobials

Antimicrobials

Antimicrobials

Antimicrobials

Antimicrobials

Antimicrobials

Antimicrobials

Antimicrobials

Antimicrobials

Antimicrobials

Antimicrobials

Antimicrobials

Antimicrobials

Antimicrobials

Antimicrobials

Antimicrobials

Antimicrobials

Antimicrobials

Antimicrobials

Antimicrobials

Antimicrobials

Antimicrobials

Antimicrobials

Antimicrobials

Antimicrobials

Antimicrobials

Antimicrobials

Antimicrobials

Antimicrobials

Antimicrobials

Antimicrobials

Antimicrobials

Antimicrobials

Antimicrobials

Antimicrobials

Antimicrobials

Antimicrobials

Antimicrobials

Antimicrobials

Antimicrobials

Antimicrobials

Antimicrobials

Antimicrobials

Antimicrobials

Antimicrobials

Antimicrobials

Antimicrobials

Antimicrobials

Antimicrobials

Antimicrobials

Antimicrobials

Antimicrobials

Antimicrobials

Antimicrobials

Antimicrobials

Antimicrobials

Antimicrobials

Antimicrobials

Antimicrobials

Antimicrobials

Antimicrobials

Antimicrobials

Antimicrobials

Antimicrobials

Antimicrobials

Antimicrobials

Antimicrobials

Antimicrobials

Antimicrobials
Antimicrobials

Antimicrobials

Antimicrobials

Antimicrobials

Antimicrobials

Antimicrobials

Antimicrobials

Antimicrobials

Antimicrobials

Antimicrobials

Antimicrobials

Antimicrobials

Antimicrobials

Antimicrobials

Antimicrobials

Antimicrobials

Antimicrobials

Antimicrobials

Antimicrobials

Antimicrobials

Antimicrobials

Antimicrobials

Antimicrobials

Antimicrobials

Antimicrobials

Antimicrobials

Antimicrobials

Antimicrobials

Antimicrobials

Antimicrobials

Antimicrobials

Antimicrobials

Antimicrobials

Antimicrobials

Antimicrobials

Antimicrobials

Antimicrobials

Antimicrobials

Antimicrobials

Antimicrobials

Antimicrobials

Antimicrobials

Antimicrobials

Antimicrobials

Antimicrobials

Antimicrobials

Antimicrobials

Antimicrobials

Antimicrobials

Antimicrobials

Antimicrobials

Antimicrobials

Antimicrobials

Antimicrobials

Antimicrobials

Antimicrobials

Antimicrobials

Antimicrobials

Antimicrobials

Antimicrobials

Antimicrobials

Antimicrobials

Antimicrobials

Antimicrobials

Antimicrobials

Antimicrobials

Antimicrobials

Antimicrobials

Antimicrobials

Antimicrobials

Antimicrobials

Antimicrobials

Antimicrobials

Antimicrobials

Antimicrobials

Antimicrobials

Antimicrobials

Antimicrobials

Antimicrobials

Antimicrobials

Antimicrobials

Antimicrobials

Antimicrobials

Antimicrobials

Antimicrobials

Antimicrobials

Antimicrobials

Antimicrobials

Antimicrobials

Antimicrobials

Antimicrobials

Antimicrobials

Antimicrobials

Antimicrobials

Antimicrobials

Antimicrobials

Antimicrobials

Antimicrobials

Antimicrobials

Antimicrobials

Antimicrobials

Antimicrobials

Antimicrobials

Antimicrobials

BCRSignalingPathway

BCRSignalingPathway

BCRSignalingPathway

BCRSignalingPathway

BCRSignalingPathway

BCRSignalingPathway

BCRSignalingPathway

BCRSignalingPathway

BCRSignalingPathway

BCRSignalingPathway

BCRSignalingPathway

BCRSignalingPathway

BCRSignalingPathway

BCRSignalingPathway

BCRSignalingPathway

BCRSignalingPathway

BCRSignalingPathway

BCRSignalingPathway

BCRSignalingPathway

BCRSignalingPathway

BCRSignalingPathway

BCRSignalingPathway

BCRSignalingPathway

BCRSignalingPathway

BCRSignalingPathway

BCRSignalingPathway

BCRSignalingPathway

BCRSignalingPathway

BCRSignalingPathway

BCRSignalingPathway

BCRSignalingPathway

BCRSignalingPathway

BCRSignalingPathway

BCRSignalingPathway

BCRSignalingPathway

BCRSignalingPathway

BCRSignalingPathway

BCRSignalingPathway

BCRSignalingPathway

BCRSignalingPathway

BCRSignalingPathway

BCRSignalingPathway

BCRSignalingPathway

BCRSignalingPathway

BCRSignalingPathway

BCRSignalingPathway

BCRSignalingPathway

BCRSignalingPathway

BCRSignalingPathway

BCRSignalingPathway

BCRSignalingPathway

BCRSignalingPathway

BCRSignalingPathway

BCRSignalingPathway

BCRSignalingPathway

BCRSignalingPathway

BCRSignalingPathway

BCRSignalingPathway

BCRSignalingPathway

BCRSignalingPathway

BCRSignalingPathway

BCRSignalingPathway

BCRSignalingPathway

BCRSignalingPathway

BCRSignalingPathway

BCRSignalingPathway

BCRSignalingPathway

BCRSignalingPathway

BCRSignalingPathway

BCRSignalingPathway

BCRSignalingPathway

BCRSignalingPathway

BCRSignalingPathway

BCRSignalingPathway

BCRSignalingPathway

BCRSignalingPathway

BCRSignalingPathway

BCRSignalingPathway

BCRSignalingPathway

BCRSignalingPathway

BCRSignalingPathway

BCRSignalingPathway

BCRSignalingPathway

BCRSignalingPathway

BCRSignalingPathway

BCRSignalingPathway

BCRSignalingPathway

BCRSignalingPathway

BCRSignalingPathway

BCRSignalingPathway

BCRSignalingPathway

BCRSignalingPathway

BCRSignalingPathway

BCRSignalingPathway

BCRSignalingPathway

BCRSignalingPathway

BCRSignalingPathway

BCRSignalingPathway

BCRSignalingPathway

BCRSignalingPathway

BCRSignalingPathway

BCRSignalingPathway

BCRSignalingPathway

BCRSignalingPathway

BCRSignalingPathway

BCRSignalingPathway

BCRSignalingPathway

BCRSignalingPathway

BCRSignalingPathway

BCRSignalingPathway

BCRSignalingPathway

BCRSignalingPathway

BCRSignalingPathway

BCRSignalingPathway

BCRSignalingPathway

BCRSignalingPathway

BCRSignalingPathway

BCRSignalingPathway

BCRSignalingPathway

BCRSignalingPathway

BCRSignalingPathway

BCRSignalingPathway

BCRSignalingPathway

BCRSignalingPathway

BCRSignalingPathway

BCRSignalingPathway

BCRSignalingPathway

BCRSignalingPathway

BCRSignalingPathway

BCRSignalingPathway

BCRSignalingPathway

BCRSignalingPathway

BCRSignalingPathway

BCRSignalingPathway

BCRSignalingPathway

BCRSignalingPathway

BCRSignalingPathway

BCRSignalingPathway

BCRSignalingPathway

BCRSignalingPathway

BCRSignalingPathway

BCRSignalingPathway

BCRSignalingPathway

BCRSignalingPathway

BCRSignalingPathway

BCRSignalingPathway

BCRSignalingPathway

BCRSignalingPathway

BCRSignalingPathway

BCRSignalingPathway

BCRSignalingPathway

BCRSignalingPathway

BCRSignalingPathway

BCRSignalingPathway

BCRSignalingPathway

BCRSignalingPathway

BCRSignalingPathway

BCRSignalingPathway

BCRSignalingPathway

BCRSignalingPathway

BCRSignalingPathway

BCRSignalingPathway

BCRSignalingPathway

BCRSignalingPathway

BCRSignalingPathway

BCRSignalingPathway

BCRSignalingPathway

BCRSignalingPathway

BCRSignalingPathway

BCRSignalingPathway

BCRSignalingPathway

BCRSignalingPathway

BCRSignalingPathway

BCRSignalingPathway

BCRSignalingPathway

BCRSignalingPathway

BCRSignalingPathway

BCRSignalingPathway

BCRSignalingPathway

BCRSignalingPathway

BCRSignalingPathway

BCRSignalingPathway

BCRSignalingPathway

BCRSignalingPathway

BCRSignalingPathway

BCRSignalingPathway

BCRSignalingPathway

BCRSignalingPathway

BCRSignalingPathway

BCRSignalingPathway

BCRSignalingPathway

BCRSignalingPathway

BCRSignalingPathway

BCRSignalingPathway

BCRSignalingPathway

BCRSignalingPathway

BCRSignalingPathway

BCRSignalingPathway

BCRSignalingPathway

BCRSignalingPathway

BCRSignalingPathway

BCRSignalingPathway

BCRSignalingPathway

BCRSignalingPathway

BCRSignalingPathway

BCRSignalingPathway

BCRSignalingPathway

BCRSignalingPathway

BCRSignalingPathway

BCRSignalingPathway

BCRSignalingPathway

BCRSignalingPathway

BCRSignalingPathway

BCRSignalingPathway

BCRSignalingPathway

BCRSignalingPathway

BCRSignalingPathway

BCRSignalingPathway

BCRSignalingPathway

BCRSignalingPathway

BCRSignalingPathway

BCRSignalingPathway

BCRSignalingPathway

BCRSignalingPathway

BCRSignalingPathway

BCRSignalingPathway

BCRSignalingPathway

BCRSignalingPathway

BCRSignalingPathway

BCRSignalingPathway

BCRSignalingPathway

BCRSignalingPathway

BCRSignalingPathway

BCRSignalingPathway

BCRSignalingPathway

BCRSignalingPathway

BCRSignalingPathway

BCRSignalingPathway

BCRSignalingPathway

BCRSignalingPathway

BCRSignalingPathway

BCRSignalingPathway

BCRSignalingPathway

BCRSignalingPathway

BCRSignalingPathway

BCRSignalingPathway

BCRSignalingPathway

BCRSignalingPathway

BCRSignalingPathway

BCRSignalingPathway

BCRSignalingPathway

BCRSignalingPathway

BCRSignalingPathway

BCRSignalingPathway

BCRSignalingPathway

BCRSignalingPathway

BCRSignalingPathway

BCRSignalingPathway

BCRSignalingPathway

BCRSignalingPathway

BCRSignalingPathway

BCRSignalingPathway

BCRSignalingPathway

BCRSignalingPathway

BCRSignalingPathway

BCRSignalingPathway

BCRSignalingPathway

BCRSignalingPathway

BCRSignalingPathway

BCRSignalingPathway

BCRSignalingPathway

BCRSignalingPathway

Chemokines

Chemokines

Chemokines

Chemokines

Chemokines

Chemokines

Chemokines

Chemokines

Chemokines

Chemokines

Chemokines

Chemokines

Chemokines

Chemokines

Chemokines

Chemokines

Chemokines

Chemokines

Chemokines

Chemokines

Chemokines

Chemokines

Chemokines

Chemokines

Chemokines

Chemokines

Chemokines

Chemokines

Chemokines

Chemokines

Chemokines

Chemokines

Chemokines

Chemokines

Chemokines

Chemokines

Chemokines

Chemokines

Chemokines

Chemokines

Chemokines

Chemokines

Chemokines

Chemokines

Chemokines

Chemokines

Chemokines

Chemokines

Chemokines

Chemokines

Chemokines

Chemokines

Chemokines

Chemokines

Chemokines

Chemokines

Chemokines

Chemokines

Chemokines

Chemokines

Chemokines

Chemokines

Chemokines

Chemokines

Chemokines

Chemokines

Chemokines

Chemokines

Chemokines

Chemokines

Chemokines

Chemokines

Chemokines

Chemokines

Chemokines

Chemokines

Chemokines

Chemokines

Chemokines
Chemokines

Chemokines

Chemokines

Chemokines

Chemokines

Chemokines

Chemokines

Chemokines

Chemokines

Chemokines
Chemokines
Chemokines

Chemokines

Chemokines

Chemokines

Chemokines

Chemokines

Chemokines

Chemokines

Chemokines

Chemokines

Chemokines

Chemokines

Chemokine_Receptors

Chemokine_Receptors

Chemokine_Receptors

Chemokine_Receptors

Chemokine_Receptors

Chemokine_Receptors

Chemokine_Receptors

Chemokine_Receptors

Chemokine_Receptors

Chemokine_Receptors

Chemokine_Receptors

Chemokine_Receptors

Chemokine_Receptors

Chemokine_Receptors

Chemokine_Receptors

Chemokine_Receptors

Chemokine_Receptors

Chemokine_Receptors

Chemokine_Receptors

Chemokine_Receptors

Chemokine_Receptors

Chemokine_Receptors

Chemokine_Receptors

Chemokine_Receptors

Chemokine_Receptors

Chemokine_Receptors

Chemokine_Receptors

Chemokine_Receptors

Chemokine_Receptors

Chemokine_Receptors

Chemokine_Receptors

Chemokine_Receptors

Chemokine_Receptors

Chemokine_Receptors

Chemokine_Receptors

Chemokine_Receptors

Chemokine_Receptors

Chemokine_Receptors

Chemokine_Receptors

Chemokine_Receptors

Chemokine_Receptors

Chemokine_Receptors

Chemokine_Receptors

Chemokine_Receptors

Chemokine_Receptors

Chemokine_Receptors

Chemokine_Receptors

Chemokine_Receptors

Chemokine_Receptors

Chemokine_Receptors

Chemokine_Receptors

Chemokine_Receptors

Chemokine_Receptors

Cytokines

Cytokines

Cytokines

Cytokines

Cytokines

Cytokines

Cytokines

Cytokines

Cytokines

Cytokines

Cytokines

Cytokines

Cytokines

Cytokines

Cytokines

Cytokines

Cytokines

Cytokines

Cytokines

Cytokines

Cytokines

Cytokines

Cytokines

Cytokines

Cytokines

Cytokines

Cytokines

Cytokines

Cytokines

Cytokines

Cytokines

Cytokines

Cytokines

Cytokines

Cytokines

Cytokines
Cytokines

Cytokines

Cytokines

Cytokines

Cytokines

Cytokines

Cytokines

Cytokines

Cytokines

Cytokines

Cytokines

Cytokines

Cytokines

Cytokines

Cytokines

Cytokines

Cytokines

Cytokines

Cytokines

Cytokines

Cytokines

Cytokines

Cytokines

Cytokines

Cytokines

Cytokines

Cytokines

Cytokines

Cytokines

Cytokines

Cytokines

Cytokines

Cytokines

Cytokines

Cytokines

Cytokines

Cytokines

Cytokines

Cytokines

Cytokines

Cytokines

Cytokines

Cytokines

Cytokines
Cytokines

Cytokines

Cytokines

Cytokines

Cytokines

Cytokines

Cytokines

Cytokines

Cytokines

Cytokines

Cytokines

Cytokines

Cytokines

Cytokines

Cytokines

Cytokines

Cytokines

Cytokines

Cytokines

Cytokines

Cytokines

Cytokines

Cytokines

Cytokines

Cytokines

Cytokines

Cytokines

Cytokines

Cytokines

Cytokines

Cytokines

Cytokines

Cytokines

Cytokines

Cytokines

Cytokines

Cytokines

Cytokines

Cytokines

Cytokines

Cytokines

Cytokines

Cytokines

Cytokines

Cytokines

Cytokines

Cytokines

Cytokines

Cytokines

Cytokines

Cytokines
Cytokines
Cytokines
Cytokines
Cytokines
Cytokines
Cytokines
Cytokines
Cytokines
Cytokines
Cytokines
Cytokines
Cytokines
Cytokines
Cytokines
Cytokines
Cytokines
Cytokines
Cytokines

Cytokines

Cytokines

Cytokines

Cytokines

Cytokines

Cytokines

Cytokines

Cytokines

Cytokines

Cytokines

Cytokines

Cytokines

Cytokines

Cytokines

Cytokines

Cytokines

Cytokines

Cytokines

Cytokines

Cytokines

Cytokines

Cytokines

Cytokines

Cytokines

Cytokines

Cytokines

Cytokines

Cytokines

Cytokines

Cytokines

Cytokines

Cytokines

Cytokines

Cytokines

Cytokines

Cytokines

Cytokines

Cytokines

Cytokines

Cytokines

Cytokines

Cytokines

Cytokines

Cytokines

Cytokines

Cytokines

Cytokines

Cytokines

Cytokines

Cytokines

Cytokines

Cytokines

Cytokines

Cytokines

Cytokines

Cytokines

Cytokines

Cytokines

Cytokines

Cytokines

Cytokines
Cytokines

Cytokines

Cytokines

Cytokines

Cytokines

Cytokines

Cytokines

Cytokines

Cytokines
Cytokines
Cytokines

Cytokines

Cytokines

Cytokines
Cytokines
Cytokines

Cytokines

Cytokines

Cytokines

Cytokines

Cytokines

Cytokines

Cytokines
Cytokines

Cytokines

Cytokines

Cytokines

Cytokines
Cytokines

Cytokines

Cytokines

Cytokines

Cytokines

Cytokines

Cytokines

Cytokines

Cytokines

Cytokines

Cytokines

Cytokines

Cytokines

Cytokines

Cytokines

Cytokines

Cytokines

Cytokines

Cytokines

Cytokines

Cytokines

Cytokines

Cytokines

Cytokines

Cytokines

Cytokines

Cytokines

Cytokines

Cytokines

Cytokines

Cytokines

Cytokines

Cytokines

Cytokines

Cytokines

Cytokines

Cytokines

Cytokines

Cytokines

Cytokines

Cytokines

Cytokines

Cytokines

Cytokines

Cytokines

Cytokines

Cytokines

Cytokines

Cytokines

Cytokines

Cytokines

Cytokines

Cytokines

Cytokines
Cytokines

Cytokines

Cytokines

Cytokines

Cytokines

Cytokines

Cytokines

Cytokines

Cytokines

Cytokines

Cytokines

Cytokines

Cytokines

Cytokines

Cytokines

Cytokines

Cytokines

Cytokines

Cytokines

Cytokines

Cytokines

Cytokines

Cytokines

Cytokines

Cytokines

Cytokines

Cytokines

Cytokines

Cytokines

Cytokines

Cytokines

Cytokines

Cytokines

Cytokines

Cytokines

Cytokines

Cytokines

Cytokines

Cytokines

Cytokines

Cytokines

Cytokines

Cytokines

Cytokines

Cytokines

Cytokines

Cytokines

Cytokines

Cytokines

Cytokines

Cytokines

Cytokines

Cytokines

Cytokines

Cytokines

Cytokines

Cytokines

Cytokines

Cytokines

Cytokines

Cytokines

Cytokines

Cytokines

Cytokines

Cytokines

Cytokines

Cytokines

Cytokines

Cytokines

Cytokines

Cytokines

Cytokines

Cytokines

Cytokines

Cytokines

Cytokines

Cytokines

Cytokines

Cytokines

Cytokines

Cytokines

Cytokines

Cytokines

Cytokines

Cytokines

Cytokines

Cytokines

Cytokines

Cytokines

Cytokines

Cytokines

Cytokines

Cytokines

Cytokines

Cytokines

Cytokines

Cytokines
Cytokines

Cytokines

Cytokines

Cytokines

Cytokines

Cytokines

Cytokines

Cytokines

Cytokines

Cytokines
Cytokines
Cytokines

Cytokines

Cytokines

Cytokines

Cytokines

Cytokines

Cytokines

Cytokines

Cytokines

Cytokines

Cytokines
Cytokines
Cytokines

Cytokines

Cytokines

Cytokines

Cytokines

Cytokines

Cytokines

Cytokines

Cytokines

Cytokines

Cytokines

Cytokines

Cytokines

Cytokines

Cytokines

Cytokines

Cytokines

Cytokines

Cytokines

Cytokines

Cytokines

Cytokines

Cytokines

Cytokines

Cytokines

Cytokines

Cytokines

Cytokines

Cytokines

Cytokines

Cytokines
Cytokines
Cytokines
Cytokines
Cytokines
Cytokines
Cytokines
Cytokines
Cytokines
Cytokines
Cytokines
Cytokines
Cytokine_Receptors
Cytokine_Receptors
Cytokine_Receptors
Cytokine_Receptors
Cytokine_Receptors
Cytokine_Receptors
Cytokine_Receptors
Cytokine_Receptors
Cytokine_Receptors

Cytokine_Receptors

Cytokine_Receptors

Cytokine_Receptors

Cytokine_Receptors

Cytokine_Receptors
Cytokine_Receptors

Cytokine_Receptors

Cytokine_Receptors

Cytokine_Receptors

Cytokine_Receptors

Cytokine_Receptors
Cytokine_Receptors

Cytokine_Receptors

Cytokine_Receptors

Cytokine_Receptors

Cytokine_Receptors

Cytokine_Receptors

Cytokine_Receptors

Cytokine_Receptors

Cytokine_Receptors

Cytokine_Receptors

Cytokine_Receptors

Cytokine_Receptors

Cytokine_Receptors

Cytokine_Receptors

Cytokine_Receptors

Cytokine_Receptors

Cytokine_Receptors

Cytokine_Receptors

Cytokine_Receptors

Cytokine_Receptors

Cytokine_Receptors

Cytokine_Receptors

Cytokine_Receptors

Cytokine_Receptors

Cytokine_Receptors

Cytokine_Receptors

Cytokine_Receptors

Cytokine_Receptors

Cytokine_Receptors

Cytokine_Receptors

Cytokine_Receptors

Cytokine_Receptors

Cytokine_Receptors

Cytokine_Receptors

Cytokine_Receptors

Cytokine_Receptors

Cytokine_Receptors

Cytokine_Receptors

Cytokine_Receptors

Cytokine_Receptors

Cytokine_Receptors

Cytokine_Receptors

Cytokine_Receptors

Cytokine_Receptors

Cytokine_Receptors

Cytokine_Receptors

Cytokine_Receptors

Cytokine_Receptors

Cytokine_Receptors

Cytokine_Receptors

Cytokine_Receptors

Cytokine_Receptors

Cytokine_Receptors

Cytokine_Receptors

Cytokine_Receptors

Cytokine_Receptors

Cytokine_Receptors

Cytokine_Receptors

Cytokine_Receptors

Cytokine_Receptors

Cytokine_Receptors

Cytokine_Receptors

Cytokine_Receptors

Cytokine_Receptors

Cytokine_Receptors

Cytokine_Receptors

Cytokine_Receptors

Cytokine_Receptors

Cytokine_Receptors

Cytokine_Receptors

Cytokine_Receptors
Cytokine_Receptors

Cytokine_Receptors

Cytokine_Receptors

Cytokine_Receptors

Cytokine_Receptors

Cytokine_Receptors

Cytokine_Receptors

Cytokine_Receptors

Cytokine_Receptors

Cytokine_Receptors

Cytokine_Receptors

Cytokine_Receptors

Cytokine_Receptors

Cytokine_Receptors

Cytokine_Receptors

Cytokine_Receptors

Cytokine_Receptors

Cytokine_Receptors

Cytokine_Receptors

Cytokine_Receptors

Cytokine_Receptors

Cytokine_Receptors

Cytokine_Receptors

Cytokine_Receptors

Cytokine_Receptors

Cytokine_Receptors

Cytokine_Receptors

Cytokine_Receptors

Cytokine_Receptors

Cytokine_Receptors

Cytokine_Receptors

Cytokine_Receptors

Cytokine_Receptors

Cytokine_Receptors

Cytokine_Receptors

Cytokine_Receptors

Cytokine_Receptors

Cytokine_Receptors

Cytokine_Receptors

Cytokine_Receptors

Cytokine_Receptors

Cytokine_Receptors

Cytokine_Receptors

Cytokine_Receptors

Cytokine_Receptors

Cytokine_Receptors

Cytokine_Receptors

Cytokine_Receptors

Cytokine_Receptors

Cytokine_Receptors

Cytokine_Receptors

Cytokine_Receptors

Cytokine_Receptors

Cytokine_Receptors

Cytokine_Receptors

Cytokine_Receptors

Cytokine_Receptors

Cytokine_Receptors

Cytokine_Receptors

Cytokine_Receptors

Cytokine_Receptors

Cytokine_Receptors

Cytokine_Receptors

Cytokine_Receptors

Cytokine_Receptors

Cytokine_Receptors

Cytokine_Receptors

Cytokine_Receptors

Cytokine_Receptors

Cytokine_Receptors

Cytokine_Receptors

Cytokine_Receptors

Cytokine_Receptors

Cytokine_Receptors

Cytokine_Receptors

Cytokine_Receptors

Cytokine_Receptors

Cytokine_Receptors

Cytokine_Receptors

Cytokine_Receptors

Cytokine_Receptors

Cytokine_Receptors

Cytokine_Receptors

Cytokine_Receptors

Cytokine_Receptors

Cytokine_Receptors

Cytokine_Receptors

Cytokine_Receptors

Cytokine_Receptors

Cytokine_Receptors

Cytokine_Receptors

Cytokine_Receptors

Cytokine_Receptors

Cytokine_Receptors

Cytokine_Receptors

Cytokine_Receptors

Cytokine_Receptors

Cytokine_Receptors

Cytokine_Receptors

Cytokine_Receptors

Cytokine_Receptors

Cytokine_Receptors

Cytokine_Receptors

Cytokine_Receptors

Cytokine_Receptors

Cytokine_Receptors

Cytokine_Receptors

Cytokine_Receptors

Cytokine_Receptors

Cytokine_Receptors

Cytokine_Receptors

Cytokine_Receptors

Cytokine_Receptors

Cytokine_Receptors

Cytokine_Receptors

Cytokine_Receptors

Cytokine_Receptors

Cytokine_Receptors

Cytokine_Receptors

Cytokine_Receptors

Cytokine_Receptors

Cytokine_Receptors

Cytokine_Receptors

Cytokine_Receptors

Cytokine_Receptors

Cytokine_Receptors

Cytokine_Receptors

Cytokine_Receptors

Cytokine_Receptors

Cytokine_Receptors

Cytokine_Receptors

Cytokine_Receptors

Cytokine_Receptors

Cytokine_Receptors

Cytokine_Receptors

Cytokine_Receptors

Cytokine_Receptors

Cytokine_Receptors

Cytokine_Receptors

Cytokine_Receptors

Cytokine_Receptors

Cytokine_Receptors

Cytokine_Receptors

Cytokine_Receptors

Cytokine_Receptors

Cytokine_Receptors

Cytokine_Receptors

Cytokine_Receptors

Cytokine_Receptors

Cytokine_Receptors

Cytokine_Receptors

Cytokine_Receptors

Cytokine_Receptors

Cytokine_Receptors

Cytokine_Receptors

Cytokine_Receptors

Cytokine_Receptors

Cytokine_Receptors

Cytokine_Receptors

Cytokine_Receptors

Cytokine_Receptors

Cytokine_Receptors

Cytokine_Receptors

Cytokine_Receptors

Cytokine_Receptors

Cytokine_Receptors

Cytokine_Receptors

Cytokine_Receptors

Cytokine_Receptors

Cytokine_Receptors

Cytokine_Receptors

Cytokine_Receptors

Cytokine_Receptors

Cytokine_Receptors

Cytokine_Receptors

Cytokine_Receptors

Cytokine_Receptors

Cytokine_Receptors

Cytokine_Receptors

Cytokine_Receptors

Cytokine_Receptors

Cytokine_Receptors

Cytokine_Receptors

Cytokine_Receptors

Cytokine_Receptors

Cytokine_Receptors

Cytokine_Receptors

Cytokine_Receptors

Cytokine_Receptors

Cytokine_Receptors

Cytokine_Receptors

Cytokine_Receptors

Cytokine_Receptors

Cytokine_Receptors

Cytokine_Receptors

Cytokine_Receptors

Cytokine_Receptors

Cytokine_Receptors

Cytokine_Receptors

Cytokine_Receptors

Cytokine_Receptors

Cytokine_Receptors

Cytokine_Receptors

Cytokine_Receptors

Cytokine_Receptors

Cytokine_Receptors

Cytokine_Receptors

Cytokine_Receptors

Cytokine_Receptors

Cytokine_Receptors

Cytokine_Receptors

Cytokine_Receptors

Cytokine_Receptors

Cytokine_Receptors

Cytokine_Receptors

Interferons
Interferons

Interferons

Interferons

Interferons

Interferons

Interferons

Interferons

Interferons

Interferons
Interferons
Interferons

Interferons

Interferons

Interferons
Interferons
Interferons

Interferon_Receptor

Interferon_Receptor

Interferon_Receptor

Interleukins

Interleukins

Interleukins

Interleukins
Interleukins

Interleukins

Interleukins

Interleukins

Interleukins
Interleukins

Interleukins

Interleukins

Interleukins

Interleukins

Interleukins

Interleukins

Interleukins

Interleukins

Interleukins

Interleukins

Interleukins

Interleukins

Interleukins

Interleukins

Interleukins

Interleukins

Interleukins

Interleukins

Interleukins

Interleukins

Interleukins

Interleukins

Interleukins

Interleukins

Interleukins

Interleukins

Interleukins

Interleukins

Interleukins

Interleukins

Interleukins

Interleukins

Interleukins

Interleukins

Interleukins

Interleukins
Interleukins

Interleukins_Receptor

Interleukins_Receptor

Interleukins_Receptor

Interleukins_Receptor

Interleukins_Receptor

Interleukins_Receptor

Interleukins_Receptor

Interleukins_Receptor

Interleukins_Receptor

Interleukins_Receptor

Interleukins_Receptor

Interleukins_Receptor

Interleukins_Receptor

Interleukins_Receptor

Interleukins_Receptor

Interleukins_Receptor

Interleukins_Receptor

Interleukins_Receptor

Interleukins_Receptor

Interleukins_Receptor

Interleukins_Receptor

Interleukins_Receptor

Interleukins_Receptor

Interleukins_Receptor

Interleukins_Receptor

Interleukins_Receptor

Interleukins_Receptor

Interleukins_Receptor

Interleukins_Receptor

Interleukins_Receptor

Interleukins_Receptor

Interleukins_Receptor

Interleukins_Receptor

Interleukins_Receptor

Interleukins_Receptor

Interleukins_Receptor

Interleukins_Receptor

Interleukins_Receptor

Interleukins_Receptor

Interleukins_Receptor

Interleukins_Receptor

Interleukins_Receptor

NaturalKiller_Cell_Cytotoxicity

NaturalKiller_Cell_Cytotoxicity

NaturalKiller_Cell_Cytotoxicity

NaturalKiller_Cell_Cytotoxicity

NaturalKiller_Cell_Cytotoxicity

NaturalKiller_Cell_Cytotoxicity

NaturalKiller_Cell_Cytotoxicity

NaturalKiller_Cell_Cytotoxicity

NaturalKiller_Cell_Cytotoxicity

NaturalKiller_Cell_Cytotoxicity

NaturalKiller_Cell_Cytotoxicity

NaturalKiller_Cell_Cytotoxicity

NaturalKiller_Cell_Cytotoxicity

NaturalKiller_Cell_Cytotoxicity

NaturalKiller_Cell_Cytotoxicity

NaturalKiller_Cell_Cytotoxicity

NaturalKiller_Cell_Cytotoxicity

NaturalKiller_Cell_Cytotoxicity

NaturalKiller_Cell_Cytotoxicity

NaturalKiller_Cell_Cytotoxicity

NaturalKiller_Cell_Cytotoxicity

NaturalKiller_Cell_Cytotoxicity

NaturalKiller_Cell_Cytotoxicity

NaturalKiller_Cell_Cytotoxicity

NaturalKiller_Cell_Cytotoxicity

NaturalKiller_Cell_Cytotoxicity

NaturalKiller_Cell_Cytotoxicity

NaturalKiller_Cell_Cytotoxicity

NaturalKiller_Cell_Cytotoxicity

NaturalKiller_Cell_Cytotoxicity

NaturalKiller_Cell_Cytotoxicity

NaturalKiller_Cell_Cytotoxicity

NaturalKiller_Cell_Cytotoxicity

NaturalKiller_Cell_Cytotoxicity

NaturalKiller_Cell_Cytotoxicity

NaturalKiller_Cell_Cytotoxicity

NaturalKiller_Cell_Cytotoxicity

NaturalKiller_Cell_Cytotoxicity

NaturalKiller_Cell_Cytotoxicity

NaturalKiller_Cell_Cytotoxicity

NaturalKiller_Cell_Cytotoxicity

NaturalKiller_Cell_Cytotoxicity

NaturalKiller_Cell_Cytotoxicity

NaturalKiller_Cell_Cytotoxicity

NatualKiller_Cell_Cytotoxicity

NaturalKiller_Cell_Cytotoxicity

NaturalKiller_Cell_Cytotoxicity

NaturalKiller_Cell_Cytotoxicity

NaturalKiller_Cell_Cytotoxicity

NaturalKiller_Cell_Cytotoxicity

NaturalKiller_Cell_Cytotoxicity

NaturalKiller_Cell_Cytotoxicity

NaturalKiller_Cell_Cytotoxicity

NaturalKiller_Cell_Cytotoxicity

NaturalKiller_Cell_Cytotoxicity

NaturalKiller_Cell_Cytotoxicity

NaturalKiller_Cell_Cytotoxicity

NaturalKiller_Cell_Cytotoxicity

NaturalKiller_Cell_Cytotoxicity

NaturalKiller_Cell_Cytotoxicity

NaturalKiller_Cell_Cytotoxicity

NaturalKiller_Cell_Cytotoxicity

NaturalKiller_Cell_Cytotoxicity

NaturalKiller_Cell_Cytotoxicity

NaturalKiller_Cell_Cytotoxicity

NaturalKiller_Cell_Cytotoxicity

NaturalKiller_Cell_Cytotoxicity

NaturalKiller_Cell_Cytotoxicity

NaturalKiller_Cell_Cytotoxicity

NaturalKiller_Cell_Cytotoxicity

NaturalKiller_Cell_Cytotoxicity

NaturalKiller_Cell_Cytotoxicity

NaturalKiller_Cell_Cytotoxicity

NaturalKiller_Cell_Cytotoxicity

NaturalKiller_Cell_Cytotoxicity

NaturalKiller_Cell_Cytotoxicity

NaturalKiller_Cell_Cytotoxicity

NaturalKiller_Cell_Cytotoxicity

NaturalKiller_Cell_Cytotoxicity

NaturalKiller_Cell_Cytotoxicity

NaturalKiller_Cell_Cytotoxicity

NaturalKiller_Cell_Cytotoxicity

NaturalKiller_Cell_Cytotoxicity

NaturalKiller_Cell_Cytotoxicity

NaturalKiller_Cell_Cytotoxicity

NaturalKiller_Cell_Cytotoxicity

NaturalKiller_Cell_Cytotoxicity

NaturalKiller_Cell_Cytotoxicity

NaturalKiller_Cell_Cytotoxicity

NaturalKiller_Cell_Cytotoxicity

NaturalKiller_Cell_Cytotoxicity

NaturalKiller_Cell_Cytotoxicity

NaturalKiller_Cell_Cytotoxicity

NaturalKiller_Cell_Cytotoxicity

NaturalKiller_Cell_Cytotoxicity

NaturalKiller_Cell_Cytotoxicity

NaturalKiller_Cell_Cytotoxicity

NaturalKiller_Cell_Cytotoxicity
NaturalKiller_Cell_Cytotoxicity
NaturalKiller_Cell_Cytotoxicity
NaturalKiller_Cell_Cytotoxicity
NaturalKiller_Cell_Cytotoxicity

NaturalKiller_Cell_Cytotoxicity

NaturalKiller_Cell_Cytotoxicity

NaturalKiller_Cell_Cytotoxicity

NaturalKiller_Cell_Cytotoxicity

NaturalKiller_Cell_Cytotoxicity

NaturalKiller_Cell_Cytotoxicity

NaturalKiller_Cell_Cytotoxicity

NaturalKiller_Cell_Cytotoxicity

NaturalKiller_Cell_Cytotoxicity

NaturalKiller_Cell_Cytotoxicity

NaturalKiller_Cell_Cytotoxicity

NaturalKiller_Cell_Cytotoxicity

NaturalKiller_Cell_Cytotoxicity

NaturalKiller_Cell_Cytotoxicity

NaturalKiller_Cell_Cytotoxicity

NaturalKiller_Cell_Cytotoxicity
NaturalKiller_Cell_Cytotoxicity

NaturalKiller_Cell_Cytotoxicity

TCRsignalingPathway

TCRsignalingPathway

TCRsignalingPathway

TCRsignalingPathway

TCRsignalingPathway
TCRsignalingPathway

TCRsignalingPathway

TCRsignalingPathway

TCRsignalingPathway

TCRsignalingPathway

TCRsignalingPathway

TCRsignalingPathway

TCRsignalingPathway

TCRsignalingPathway

TCRsignalingPathway

TCRsignalingPathway

TCRsignalingPathway

TCRsignalingPathway

TCRsignalingPathway

TCRsignalingPathway

TCRsignalingPathway

TCRsignalingPathway

TCRsignalingPathway

TCRsignalingPathway

TCRsignalingPathway

TCRsignalingPathway

TCRsignalingPathway

TCRsignalingPathway

TCRsignalingPathway

TCRsignalingPathway

TCRsignalingPathway

TCRsignalingPathway

TCRsignalingPathway

TCRsignalingPathway

TCRsignalingPathway

TCRsignalingPathway

TCRsignalingPathway

TCRsignalingPathway

TCRsignalingPathway

TCRsignalingPathway

TCRsignalingPathway

TCRsignalingPathway

TCRsignalingPathway

TCRsignalingPathway

TCRsignalingPathway

TCRsignalingPathway

TCRsignalingPathway

TCRsignalingPathway

TCRsignalingPathway

TCRsignalingPathway

TCRsignalingPathway

TCRsignalingPathway

TCRsignalingPathway

TCRsignalingPathway

TCRsignalingPathway

TCRsignalingPathway

TCRsignalingPathway

TCRsignalingPathway

TCRsignalingPathway

TCRsignalingPathway

TCRsignalingPathway

TCRsignalingPathway

TCRsignalingPathway

TCRsignalingPathway

TCRsignalingPathway

TCRsignalingPathway

TCRsignalingPathway

TCRsignalingPathway

TCRsignalingPathway

TCRsignalingPathway

TCRsignalingPathway

TCRsignalingPathway

TCRsignalingPathway

TCRsignalingPathway

TCRsignalingPathway

TCRsignalingPathway

TCRsignalingPathway

TCRsignalingPathway

TCRsignalingPathway

TCRsignalingPathway

TCRsignalingPathway

TCRsignalingPathway

TCRsignalingPathway

TCRsignalingPathway

TCRsignalingPathway

TCRsignalingPathway

TCRsignalingPathway

TCRsignalingPathway

TCRsignalingPathway

TCRsignalingPathway

TCRsignalingPathway

TCRsignalingPathway

TCRsignalingPathway

TCRsignalingPathway

TCRsignalingPathway

TCRsignalingPathway

TCRsignalingPathway

TCRsignalingPathway

TCRsignalingPathway

TCRsignalingPathway

TCRsignalingPathway

TCRsignalingPathway

TCRsignalingPathway

TCRsignalingPathway

TCRsignalingPathway

TCRsignalingPathway

TCRsignalingPathway

TCRsignalingPathway

TCRsignalingPathway

TCRsignalingPathway

TCRsignalingPathway

TCRsignalingPathway

TCRsignalingPathway

TCRsignalingPathway

TCRsignalingPathway

TCRsignalingPathway

TCRsignalingPathway

TCRsignalingPathway

TCRsignalingPathway

TCRsignalingPathway

TCRsignalingPathway

TCRsignalingPathway

TCRsignalingPathway

TCRsignalingPathway

TCRsignalingPathway

TCRsignalingPathway

TCRsignalingPathway

TCRsignalingPathway

TCRsignalingPathway

TCRsignalingPathway

TCRsignalingPathway

TCRsignalingPathway

TCRsignalingPathway

TCRsignalingPathway

TCRsignalingPathway

TCRsignalingPathway

TCRsignalingPathway

TCRsignalingPathway

TCRsignalingPathway

TCRsignalingPathway

TCRsignalingPathway

TCRsignalingPathway

TCRsignalingPathway

TCRsignalingPathway

TCRsignalingPathway

TCRsignalingPathway

TCRsignalingPathway

TCRsignalingPathway

TCRsignalingPathway

TCRsignalingPathway

TCRsignalingPathway

TCRsignalingPathway

TCRsignalingPathway

TCRsignalingPathway

TCRsignalingPathway

TCRsignalingPathway

TCRsignalingPathway

TCRsignalingPathway

TCRsignalingPathway

TCRsignalingPathway

TCRsignalingPathway

TCRsignalingPathway

TCRsignalingPathway

TCRsignalingPathway

TCRsignalingPathway

TCRsignalingPathway

TCRsignalingPathway

TCRsignalingPathway

TCRsignalingPathway

TCRsignalingPathway

TCRsignalingPathway

TCRsignalingPathway

TCRsignalingPathway

TCRsignalingPathway

TCRsignalingPathway

TCRsignalingPathway

TCRsignalingPathway

TCRsignalingPathway

TCRsignalingPathway

TCRsignalingPathway

TCRsignalingPathway

TCRsignalingPathway

TCRsignalingPathway

TCRsignalingPathway

TCRsignalingPathway

TCRsignalingPathway

TCRsignalingPathway

TCRsignalingPathway

TCRsignalingPathway

TCRsignalingPathway

TCRsignalingPathway

TCRsignalingPathway

TCRsignalingPathway

TCRsignalingPathway

TCRsignalingPathway

TCRsignalingPathway

TCRsignalingPathway

TCRsignalingPathway

TCRsignalingPathway

TCRsignalingPathway

TCRsignalingPathway

TCRsignalingPathway

TCRsignalingPathway

TCRsignalingPathway

TCRsignalingPathway

TCRsignalingPathway

TCRsignalingPathway

TCRsignalingPathway

TCRsignalingPathway

TCRsignalingPathway

TCRsignalingPathway

TCRsignalingPathway

TCRsignalingPathway

TCRsignalingPathway

TCRsignalingPathway

TCRsignalingPathway

TCRsignalingPathway

TCRsignalingPathway

TCRsignalingPathway

TCRsignalingPathway

TCRsignalingPathway

TCRsignalingPathway

TCRsignalingPathway

TCRsignalingPathway

TCRsignalingPathway

TCRsignalingPathway

TCRsignalingPathway

TCRsignalingPathway

TCRsignalingPathway

TCRsignalingPathway

TCRsignalingPathway

TCRsignalingPathway

TCRsignalingPathway

TCRsignalingPathway

TCRsignalingPathway

TCRsignalingPathway

TCRsignalingPathway

TCRsignalingPathway

TCRsignalingPathway

TCRsignalingPathway

TCRsignalingPathway

TCRsignalingPathway

TCRsignalingPathway

TCRsignalingPathway

TCRsignalingPathway

TCRsignalingPathway

TCRsignalingPathway

TCRsignalingPathway

TCRsignalingPathway

TCRsignalingPathway

TCRsignalingPathway

TCRsignalingPathway

TCRsignalingPathway

TCRsignalingPathway

TCRsignalingPathway

TCRsignalingPathway

TCRsignalingPathway

TCRsignalingPathway

TCRsignalingPathway

TCRsignalingPathway

TCRsignalingPathway

TCRsignalingPathway

TCRsignalingPathway

TCRsignalingPathway

TCRsignalingPathway

TCRsignalingPathway

TCRsignalingPathway

TCRsignalingPathway

TCRsignalingPathway

TCRsignalingPathway

TCRsignalingPathway

TCRsignalingPathway

TCRsignalingPathway

TCRsignalingPathway

TCRsignalingPathway

TCRsignalingPathway

TCRsignalingPathway

TCRsignalingPathway

TCRsignalingPathway

TCRsignalingPathway

TCRsignalingPathway

TCRsignalingPathway

TCRsignalingPathway

TCRsignalingPathway

TCRsignalingPathway

TCRsignalingPathway

TCRsignalingPathway

TCRsignalingPathway

TGFb_Family_Member

TGFb_Family_Member

TGFb_Family_Member

TGFb_Family_Member

TGFb_Family_Member

TGFb_Family_Member

TGFb_Family_Member

TGFb_Family_Member

TGFb_Family_Member

TGFb_Family_Member

TGFb_Family_Member

TGFb_Family_Member

TGFb_Family_Member

TGFb_Family_Member

TGFb_Family_Member

TGFb_Family_Member

TGFb_Family_Member

TGFb_Family_Member

TGFb_Family_Member

TGFb_Family_Member

TGFb_Family_Member

TGFb_Family_Member

TGFb_Family_Member

TGFb_Family_Member

TGFb_Family_Member

TGFb_Family_Member

TGFb_Family_Member

TGFb_Family_Member

TGFb_Family_Member

TGFb_Family_Member

TGFb_Family_Member

TGFb_Family_Member

TGFb_Family_Member

TGFb_Family_Member_Receptor

TGFb_Family_Member_Receptor

TGFb_Family_Member_Receptor

TGFb_Family_Member_Receptor

TGFb_Family_Member_Receptor

TGFb_Family_Member_Receptor

TGFb_Family_Member_Receptor

TGFb_Family_Member_Receptor

TGFb_Family_Member_Receptor

TGFb_Family_Member_Receptor

TGFb_Family_Member_Receptor

TGFb_Family_Member_Receptor

TNF_Family_Members

TNF_Family_Members

TNF_Family_Members

TNF_Family_Members

TNF_Family_Members

TNF_Family_Members

TNF_Family_Members

TNF_Family_Members

TNF_Family_Members

TNF_Family_Members

TNF_Family_Members

TNF_Family_Members

TNF_Family_Members_Receptors

TNF_Family_Members_Receptors

TNF_Family_Members_Receptors

TNF_Family_Members_Receptors

TNF_Family_Members_Receptors

TNF_Family_Members_Receptors

TNF_Family_Members_Receptors

TNF_Family_Members_Receptors

TNF_Family_Members_Receptors

TNF_Family_Members_Receptors

TNF_Family_Members_Receptors

TNF_Family_Members_Receptors

TNF_Family_Members_Receptors

TNF_Family_Members_Receptors

TNF_Family_Members_Receptors

TNF_Family_Members_Receptors

TNF_Family_Members_Receptors

TNF_Family_Members_Receptors

TNF_Family_Members_Receptors
