# Supplementary material for: An immune-related prognostic signature associated with immune landscape and therapeutic responses in gastric cancer
Source: Aging (Albany NY). 2023 Feb 22;15(4):1074–106. doi: 10.18632/aging.204534 (PMC10008502; doi:10.18632/aging.204534)
Supplement: Supplementary Table 4 [file aging-15-204534-s004.pdf]

**Supplementary Table 4. Detailed information of the immune-related genes in InnateDB database.**

| Database ID | Gene Symbol | PubMED ID | Annotation |
|---|---|---|---|
| 131550 | Id2 | 25902484 | Group 3 innate lymphoid cells (ILC3s) can mediate immune surveillance, which constantly maintains a proper microbiota, to facilitate early colonization resistance through an Id2-dependent regulation of Il22. |
| 192083 | Iltifb | 21469124 | Iltifb (Il22b) increases the TNF-alpha-dependent induction and secretion of several immune-modulatory molecules such as initial complement factors, antimicrobial peptides and chemokines in primary keratinocytes. Iltifb-mediated induction of innate immunity is crucial for the maintenance of epidermal integrity during infection with Candida albicans. (Demonstrated in human) |
| 192083 | Iltifb | 21874025 | Iltifb (Il22) is produced by lymphoid tissue-inducer cells where it regulates the maintenance of colonic lymphoid structures during Citrobacter rodentium infection, a mechanism that bridges the lymphotoxin pathway to mucosal epithelial defense mechanisms. |
| 192083 | Iltifb | 22921121 | ILTIFB (IL22) protects intestinal stem cells from inflammatory tissue damage and regulates sensitivity to graft versus host disease. (Demonstrated in human) |
| 192083 | Iltifb | 25395539 | Flagellin induces Tlr5-dependent Il22 production and Nlrc4-dependent Il18 production to promote a protective gene expression program in intestinal epithelial cells and elimination of rotavirus-infected cells. |
| 192083 | Iltifb | 25456160 | Cxcl16-Cxcr6 crosstalk coordinates the intestinal topography of Il22 secretion required for mucosal defence against Citrobacter rodentium infection. |
| 192083 | Iltifb | 25680273 | Il22 augments the expression of Il18 mRNA and inactive precursor protein (proIL-18) in intestinal epithelial cells after Toxoplasma gondii or Citrobacter rodentium infection and maintains the homeostatic amount of proIL-18 in the ileum. |
| 192083 | Iltifb | 26649819 | Innate lymphoid cells, potent producers of Il22 after intestinal injury, increase the growth of mouse small intestine organoids in an Il22-dependent fashion. |
| 192083 | Iltifb | 25902484 | Group 3 innate lymphoid cells (ILC3s) can mediate immune surveillance, which constantly maintains a proper microbiota, to facilitate early colonization resistance through an Id2-dependent regulation of Il22. |
| 76030 | PRKRA | 25784705 | The G3BP1-CAPRIN1-PRKRA complex represents a new mode of PRKRA activation and links stress responses with innate immune activation through PRKRA without a requirement for foreign double-stranded RNA pattern recognition. |
| 76030 | PRKRA | 26608320 | A defective interfering RNA isolated from the Hu-191 vaccine strain of measles virus is sensed by PRKRA and DDX58 to initiate an innate antiviral response. |

| | | | |
|---|---|---|---|
| 76030 | PRKRA | 26454002 | DDX3X participates in antiviral innate immunity by controlling translation of PRKRA. |
| 229341 | DDX3X | 18583960 | DDX3X is a critical component of the TANK-binding kinase 1 (TBK1)-dependent innate immune response. |
| 229341 | DDX3X | 20127681 | DDX3X is an antiviral MAVS (IPS-1) enhancer, where it can bind viral RNA to join it in the MAVS complex and this augments virus-mediated IFN-beta induction and host cell protection against virus infection. |
| 229341 | DDX3X | 20657822 | DDX3X RNA helicase can augment TBK1/IKKepsilon activity and hepatitis B virus (HBV) polymerase (HBV Pol) inhibits this TBK1/IKBKE activity by disrupting the interaction between IKBKE and DDX3X, providing an explanation for how HBV evades innate immune response in the early phase of the infection. |
| 229341 | DDX3X | 25740981 | DDX3X initiates a multifaceted cellular program involving dynamic associations with hepatitis C virus (HCV) RNA and proteins, CHUK, stress granules, and lipid droplet surfaces for its crucial role in the HCV life cycle. |
| 229341 | DDX3X | 26454002 | DDX3X participates in antiviral innate immunity by controlling translation of PRKRA. |
| 55854 | DDX58 | 15208624 | DDX58 (RIG-I) is a cytoplasmic RNA helicase that functions as an intracellular sensor of dsRNA leading to the induction of Interferon (IFN) production independently of TLR signalling. |
| 55854 | DDX58 | 18948594 | DDX58 (RIG-I) first and second caspase recruitment domains (CARDs) have distinct roles in TRIM25-mediated RIG-I ubiquitination, which leads to initiation of an antiviral signalling cascade. |
| 55854 | DDX58 | 19074283 | DDX58 serves as a critical link between TLR3 and type-II-IFN signalling pathways in innate antiviral immune responses. |
| 55854 | DDX58 | 19683681 | DDX58 plays an essential role in Toll-like receptor (TLR)-stimulated phagocytosis, demonstrating that DDX58 plays a role not only in antiviral responses but in antibacterial responses as well. |
| 55854 | DDX58 | 18523264 | DDX58 plays a key role in the expression of TNF-alpha in macrophages in response to LPS stimulation, mainly for the late phase LPS-induced expression of TNF-alpha. |
| 55854 | DDX58 | 19915568 | DDX58 is a sensor able to activate the inflammasome in response to certain RNA viruses by binding to the adaptor PYCARD to trigger the caspase-1 (CASP1)-dependent inflammasome activation and IL-1-beta production. |
| 55854 | DDX58 | 20403326 | DDX58 binds specifically to K63-polyubiquitin chains through its tandem caspase recruitment domains (CARDs) that act as a ubiquitin sensor in a manner that depends on RNA and ATP, demonstrate that un-anchored K63-polyubiquitin chains are signalling molecules in antiviral innate immunity. |

| | | | |
|---|---|---|---|
| 55854 | DDX58 | 20406818 | DDX58 (RIG-I) phosphorylation on serine 8 operates as a negative switch of RIG-I activation by suppressing TRIM25 interaction. |
| 55854 | DDX58 | 20511549 | DDX58 innate immune response to viral infection of human cells is modified by a functional polymorphism in the RIG-I caspase recruitment domain (CARD). |
| 55854 | DDX58 | 19936053 | DDX58 (RIG-I) is responsible for the cytosolic recognition of Legionella pneumophila RNA and the subsequent induction of type I IFN response. (Demonstrated in murine model) |
| 55854 | DDX58 | 21690088 | DDX58 and NOD2 colocalize to cellular ruffles and cell-cell junctions to form a protein complex via the CARD domains. DDX58 negatively regulates ligand-induced NFkB signalling mediated by NOD2, and conversely, NOD2 negatively regulates type I interferon induction by DDX58. |
| 55854 | DDX58 | 21695051 | DDX58, through the TRAIL pathway, initiates apoptosis in hepatocytes infected with hepatitis C Virus to suppress viral replication. HCV envelope proteins counteract the antiviral host defence by inhibiting the expression of DDX58. |
| 55854 | DDX58 | 22072774 | DDX58 (RIG-I) ubiquitination is inhibited by arterivirus and nairovirus deubiquitinating enzymes (DUBs), resulting in the inhibition of RIG-I-like receptor (RLR)-mediated innate immune signalling. (Demonstrated in mice) |
| 55854 | DDX58 | 22912779 | Antiviral stress granules containing DDX58 (RIG-I) and EIF2AK2 (PKR) have a critical role in viral detection and innate immunity. (Demonstrated in mouse) |
| 55854 | DDX58 | 23744645 | DDX58 (RIG-I) stimulation with a synthetic ligand inhibits HIV replication in macrophages. |
| 55854 | DDX58 | 23950712 | RNF135 is essential for the association of DDX58 (RIG-I) and TRIM25, resulting in the activation of RIG-I signalling. |
| 55854 | DDX58 | 24448099 | ISG15 does not directly alter human rhinovirus replication but modulates immune signalling via the viral sensor protein DDX58 to impact production of CXCL10, which has been linked to innate immunity to viruses. |
| 55854 | DDX58 | 24448099 | Human rhinovirus infection of epithelial cells induces the expression and secretion of ISG15, which modulates immune responses via effects on DDX58, and by regulating CXCL10 production. |
| 55854 | DDX58 | 24550253 | The antisense L region of encephalomyocarditis virus associates with DDX58 and is a key determinant of IFIH1 stimulation of infected cells. |
| 55854 | DDX58 | 25002588 | IFI16 transcriptionally regulates type I interferons and DDX58 (RIG-I) and controls the interferon response to both DNA and RNA viruses. |

| | | | |
|---|---|---|---|
| 55854 | DDX58 | 25520509 | Paramyxoviruses trigger the DNA-damage response, a pathway required for RPS6KA5 activation of phospho Ser 276 RELA formation to trigger the IRF7-DDX58 amplification loop necessary for mucosal interferon production. |
| 55854 | DDX58 | 25557055 | DDX58 dually functions as an hepatitis B virus sensor activating innate signalling and as a direct antiviral factor by counteracting the viral polymerase in hepatocytes. |
| 55854 | DDX58 | 26074083 | DDX58 is the primary pattern recognition receptor (PRR) for influenza A virus (IAV), but IFIH1 is a significant contributor to the cellular defense against IAV. |
| 55854 | DDX58 | 25880109 | Signalling through both DDX58 and TLR3 is important for interferon induction by influenza A virus in alveolar epithelial cells. |
| 55854 | DDX58 | 27210312 | Hepatitis B virus-induced MIR146A attenuates cell-intrinsic anti-viral innate immunity through targeting DDX58 and IFIT3. |
| 55854 | DDX58 | 26645583 | MIR485 exhibits bispecificity, targeting DDX58 in cells with a low abundance of H5N1 virus and viral PB1 in cells with increased amounts of the H5N1 virus. |
| 55854 | DDX58 | 26608320 | A defective interfering RNA isolated from the Hu-191 vaccine strain of measles virus is sensed by PRKRA and DDX58 to initiate an innate antiviral response. |
| 55854 | DDX58 | 26371557 | ATP binding is required for DDX58 signalling on viral RNA. ATP hydrolysis provides an important function by recycling DDX58 and promoting its dissociation from non-pathogenic RNA. |
| 55854 | DDX58 | 26378160 | Influenza B virus induces IRF3 activation and IL29 (IFNL1) gene expression without a requirement for viral protein synthesis or replication and DDX58 is the critical pattern recognition receptor needed for IRF3 activation. |
| 55854 | DDX58 | 25878102 | EFTUD2 is a novel innate immune antiviral regulator that restricts hepatitis C virus infection through a DDX58/IFIH1-mediated, JAK-STAT-independent pathway. |
| 55854 | DDX58 | 26450567 | MIR136 exhibits potent antiviral activity against H5N1 influenza A virus and acts as an immune agonist of DDX58. |
| 126353 | MIR136 | 26450567 | MIR136 exhibits potent antiviral activity against H5N1 influenza A virus and acts as an immune agonist of DDX58. |
| 223927 | Mir146 | 22545247 | Mir146 directly targets Relb to modulate the amplitude of monocyte responses to inflammatory challenges. |

| | | | |
|---|---|---|---|
| 223927 | Mir146 | 22593544 | Mir146 is a mechanosensitive miRNA that modulates mechanotransduction and pressure-induced inflammation in small airway epithelium. (Demonstrated in human) |
| 223927 | Mir146 | 24092752 | Nod2 driven inflammation is regulated by nitric oxide responsive Mir146 that facilitates activation of sonic hedgehog (SHH) signalling by targeting Numb expression. |
| 223927 | Mir146 | 25024218 | T-cell-intrinsic Mir155 is required for type-2 immunity, in part through regulation of S1pr1, whereas T-cell-intrinsic Mir146 is required to prevent overt Th1/Th17 skewing. |
| 223927 | Mir146 | 26048146 | Mir146 attenuates sepsis-induced cardiac dysfunction by preventing NF Î º B activation, inflammatory cell infiltration, and inflammatory cytokine production via targeting of Irak1 and Traf6 in both cardiomyocytes and inflammatory monocytic cells. |
| 223927 | Mir146 | 26456940 | Mir146 has a role in constraining intestinal barrier function, a process that alters gut homeostasis and increases susceptibility to dextran sodium sulphate-induced colitis. |
| 179050 | Nod2 | 18981137 | Nod2-deficient mice have impaired resistance to Mycobacterium tuberculosis infection through defective innate and adaptive immunity. |
| 179050 | Nod2 | 20679225 | Nod2 functions in non-hematopoietic cells of the small intestinal crypts and this is critical for protecting mice from a Th1-driven granulomatous inflammation in the ilum. |
| 179050 | Nod2 | 20685341 | Nod2 and Nod1 account for neutrophil recruitment to the lungs of mice infected with Legionella pneumophila. |
| 179050 | Nod2 | 17705131 | Nod2 and Nod1 activation results in substantial secretion of Ccl5 by murine macrophages and induces binding of NF-kappaB subunits to Ccl5 promoter. |
| 179050 | Nod2 | 21072876 | Nod2 and Nod1 can detect Legionella pneumophila and these receptors modulate the in vivo pulmonary immune response differently. |
| 179050 | Nod2 | 21199260 | Nod2 is both a positive and negative regulator of Tlr4 - the effect it exerts is dependent on the presence of MDP. Nod2 upon engagement with its ligand, MDP, positively regulates Tlr4-mediated signaling; in the absence of MDP, Nod2 negatively regulates the Tlr4 pathway. |

| | | | |
|---|---|---|---|
| 179050 | Nod2 | 21236705 | Nod2 is a peripheral peptidoglycan intracellular sensor and is important for the progression and pathogenesis of experimental autoimmune encephalomyelitis (animal model of multiple sclerosis). |
| 179050 | Nod2 | 21108472 | Nod2 detects heat-killed Legionella pneumophila and stimulates NFkB and IFN-beta promoter activity. Nod2 deficiency results in increased proinflammatory cytokine expression at 4hrs and greater neutrophil recruitment to the lung. |
| 179050 | Nod2 | 21690088 | Ddx58 and Nod2 colocalize to cellular ruffles and cell-cell junctions to form a protein complex via the CARD domains. Ddx58 negatively regulates ligand-induced NFkB signalling mediated by Nod2, and conversely, Nod2 negatively regulates type I interferon induction by Ddx58. (Demonstrated in human) |
| 179050 | Nod2 | 22750073 | Nod2 recognition of muramyl dipeptide, a component of bacterial cell walls, improves the barrier function of intestinal epithelial cells. |
| 179050 | Nod2 | 22531915 | Nod2 enhances the innate immune response of alveolar macrophages to Mycobacterium tuberculosis in human. (Demonstrated in human) |
| 179050 | Nod2 | 24163408 | Nod1 and Nod2 synergize with Tlr4 in dendritic cells to increase IL12 production and enhance invariant natural killer T (iNKT) cell activation, and are important regulators of the IFN gamma response by iNKT cells during S. typhimurium and L. monocytogenes infections. |
| 179050 | Nod2 | 25423082 | Salmonella enterica serovar Typhimurium Î 👪msbB that possesses a modified lipid A triggers exacerbated colitis in the absence of Nod1 and/or Nod2, which is likely due to increased Tlr2 stimulation. |
| 179050 | Nod2 | 25817335 | Nod2 regulates type-1 cytokine responses to Mycobacterium avium but is not required for the control of M. avium infection in vivo. |
| 179050 | Nod2 | 26444420 | Leukotriene B4 acts on Nod2 pathway to enhance the immune response against influenza A infection. |
| 205910 | Il1a | 24022484 | Under acidic conditions both pro-inflammatory forms of Il1a and Il1b are regulated independently of the NLRP3 inflammasome. |
| 205910 | Il1a | 24651866 | In response to adenovirus infection, the IL1A-IL1R1-CXCR2 signalling axis cooperates with complement to recruit Ly-6G+7/4+ polymorphonuclear leukocytes to the splenic marginal zone (MZ) in the proximity of virus-containing MARCO+ residential MZ macrophages, which are subsequently eliminated. |
| 205910 | Il1a | 26536497 | NLRP3 inflammasome formation is dispensable for alum-induced innate immunity but Il1a and Il1b are both necessary for alum-induced neutrophil influx in vivo. |

| | | | |
|---|---|---|---|
| 205910 | Il1a | 26439902 | Il1a directly senses DNA damage and acts as signal for genotoxic stress without loss of cell integrity. |
| 66482 | IL1A | 25463072 | Interleukin-1 (IL1A/IL1B) plays a key role in the interaction between local vessel wall cells and invading monocytes to multiply cholesterol-triggered inflammation in the vessel wall. |
| 66482 | IL1A | 25474109 | IFNG interferes with the IL-1/NFKBIZ axis in Î ²-glucan-activated dendritic cells and promotes T cell-mediated immune responses with increased release of IFNG and IL22, and diminished production of IL17A. |
| 66482 | IL1A | 25964352 | CASP4 is a critical regulator of noncanonical inflammasome activation that initiates defence against bacterial pathogens in primary macrophages by mediating cell death and IL1A release |
| 66482 | IL1A | 26439902 | IL1A directly senses DNA damage and acts as signal for genotoxic stress without loss of cell integrity. |
| 73750 | IFIH1 | 16785313 | IFIH1 (MDA5) recognizes distinct RNA viruses and plays a major role in the elimination of RNA viruses in vivo. |
| 73750 | IFIH1 | 15563593 | IFIH1 binds V proteins of paramyxoviruses and this inhibit its activation of the IFNB1 (IFN-beta) promoter. |
| 73750 | IFIH1 | 11805321 | IFIH1 is a double-stranded RNA-dependent ATPase that contains both a caspase recruitment domain and RNA helicase motifs. IFIH1 may also function as a mediator of interferon (IFN)-induced growth inhibition and/or apoptosis. |
| 73750 | IFIH1 | 20107606 | IFIH1 is indispensable for sustained expression of IFN in response to paramyxovirus infection and provide the first evidence of MDA5-dependent containment of in vivo infections caused by (-) sense RNA viruses. |
| 73750 | IFIH1 | 21245317 | IFIH1 is an RNA helicase and is a key component in activating the expression of type I IFN in response to viral infection. Viral mRNA with 5' cap and 3' poly(A) from parainfluenza virus 5 is able to activate IFN expression through RNASEL-IFIH1 signalling pathway. |
| 73750 | IFIH1 | 19936053 | IFIH1 (MDA5) is responsible for the cytosolic recognition of Legionella pneumophila RNA and the subsequent induction of type I IFN response. (Demonstrated in murine model) |
| 73750 | IFIH1 | 21637773 | IFIH1 deficiency results in a delayed type I IFN and attenuated type III IFN response to rhinovirus infection, leading to a transient increase in viral titer. Upon recognition of viral dsRNA, IFIH1 synergizes with TLR3 to induce pro-inflammatory signals leading to airways inflammation and hyper-responsiveness. (Demonstrated in murine model) |
| 73750 | IFIH1 | 23328395 | Paramyxovirus V proteins bind to IFIH1 (MDA5) to disrupt viral RNA recognition and induction of antiviral immunity. |

| | | | |
|---|---|---|---|
| 73750 | IFIH1 | 24550253 | The antisense L region of encephalomyocarditis virus associates with DDX58 and is a key determinant of IFIH1 stimulation of infected cells. |
| 73750 | IFIH1 | 25463548 | IFIH1 recognizes hepatitis C virus (HCV) to initiate host interferon response during HCV infection. |
| 73750 | IFIH1 | 26074083 | DDX58 is the primary pattern recognition receptor (PRR) for influenza A virus (IAV), but IFIH1 is a significant contributor to the cellular defense against IAV. |
| 73750 | IFIH1 | 25878102 | EFTUD2 is a novel innate immune antiviral regulator that restricts hepatitis C virus infection through a DDX58/IFIH1-mediated, JAK-STAT-independent pathway. |
| 54412 | EFTUD2 | 25878102 | EFTUD2 is a novel innate immune regulator that restricts hepatitis C virus infection through an RIG-I/MDA5-mediated, JAK-STAT-independent pathway. |
| 54412 | EFTUD2 | 25878102 | EFTUD2 is a novel innate immune antiviral regulator that restricts hepatitis C virus infection through a DDX58/IFIH1-mediated, JAK-STAT-independent pathway. |
| 154818 | Tlr2 | 18768838 | Tlr2 expression in astrocytes is induced by TNF alpha and NF Kappa B-dependent pathways. |
| 154818 | Tlr2 | 18621910 | Tlr2 recognition of Staphylococcal peptidoglycan leads to induction of beta-defensin-2. |
| 154818 | Tlr2 | 19019963 | Tlr2 is involved in Respiratory syncytial virus (RSV) recognition and subsequent innate immune activation by promoting neutrophil migration and dendritic cell activation within the lung. |
| 154818 | Tlr2 | 19865078 | Tlr4, Tlr2, or Tlr3 cooperate with proteinase-activated receptors (PARs) for activation of nuclear factor-kappaB-dependent signaling in mucosal epithelial cell lines. |
| 154818 | Tlr2 | 9751057 | Tlr2 is a signaling receptor that is activated by lipopolysaccharide (LPS) in a response that depends on LPS-binding protein and is enhanced by CD14. |
| 154818 | Tlr2 | 10364168 | Tlr2 is a signal transducer for soluble Peptidoglycan and lipoteichoic acid (LTA) in addition to LPS. |
| 154818 | Tlr2 | 10384090 | Tlr2 recognizes Gram-positive bacterial cell wall components, leading to the activation of the innate immune system. |
| 154818 | Tlr2 | 10426996 | Tlr2 is a molecular link between microbial products, apoptosis, and host defense mechanisms. |
| 154818 | Tlr2 | 20167866 | TLR2/MyD88/PI3K/Rac1/Akt pathway mediates the activation of LTA-induced mitogen-activated protein kinases (MAPKs), which in turn initiates the activation of NF-kappaB, and ultimately induces cPLA2/COX-2-dependent PGE2 and IL-6 generation. |
| 154818 | Tlr2 | 20385881 | Tlr2 and Tlr4 activate murine macrophages and this is negatively regulated by a Lyn/PI3K module and promoted by SHIP1. |

| | | | |
|---|---|---|---|
| 154818 | Tlr2 | 20407745 | Tlr2 is a molecular link between increased dietary lipid intake and the regulation of glucose homeostasis, via regulation of energy substrate utilization and tissue inflammation. |
| 154818 | Tlr2 | 20422028 | Tlr2 activation induces type I interferon responses from endolysosomal compartments where it is translocated to upon ligand engagement. |
| 154818 | Tlr2 | 20368346 | Tlr2 and Myd88-dependent phosphatidylinositol 3-kinase and Rac1 activation facilitates the phagocytosis of Listeria monocytogenes by murine macrophages. |
| 154818 | Tlr2 | 21439957 | Tlr1 :: Tlr2 dimeric pairs recognize malarial glycosylphosphatidylinositols (GPI) to initiates intracellular signalling and the production of pro-inflammatory cytokines. |
| 154818 | Tlr2 | 21454596 | Tlr2 recognizes Thermus aquaticus extracellular polysacchride, YT-1, and induces the production of cytokines TNF and IL6 in peritoneal macrophages. |
| 154818 | Tlr2 | 21482737 | Tlr2::Tlr6 synergistically interacts with Tlr9 in lung epithelium to induce rapid pathogen killing, and can be used as a therapeutic target to treat otherwise lethal pneumonia. |
| 154818 | Tlr2 | 21512004 | Tlr2 is activated by gut commensal microbe, Bacteroides fragilis, to establish host-microbial symbiosis by promoting immunological tolerance. |
| 154818 | Tlr2 | 21566133 | Tlr2 and Tnfsf11 signalling pathways are modulated by Porphromonas gingivalis to alter the differentiation states of osteoclasts resulting in bacteria-mediated bone loss. |
| 154818 | Tlr2 | 21602496 | Tlr2 is expressed by Muller cells, principal glia of retina, and is responsible for generating robust bactericidal activity against Staphylococcus aureusand contributing to retinal innate defence. |
| 154818 | Tlr2 | 21698237 | Tlr2 is required for rapid inflammasome activation in response to infection by cytosolic bacterial pathogens such as Francisella novicida. |
| 154818 | Tlr2 | 21862586 | Tlr2-driven integration of inducible nitric oxide synthase (iNOS), Wnt-beta-Catenin and Notch1 signalling contributes to its capacity to regulate a battery of genes associated with T regulatory cell lineage commitment and towards modulation of defined set of effector functions in macrophages. |
| 154818 | Tlr2 | 21873606 | Tlr2 directly recognizes glycogen, resulting in the activation of immunocytes such as macrophages to enhance the production of nitric oxide and inflammatory cytokines. |

| | | | |
|---|---|---|---|
| 154818 | Tlr2 | 22096480 | Tlr2 and Tlr4 are crucial for in vivo recognition of Chlamydia pneumoniae. Tlr2/4 double-deficient mice were unable to control pneumonia caused by C. pneumoniae. |
| 154818 | Tlr2 | 22102818 | Tlr2 signalling promotes protective vaccine-enhancing Th17 cell responses when cells are stimulated with early secreted antigenic target protein 6 (ESAT-6) expressed by the virulent Mycobacterium tuberculosis strain H37Rv but not by tuberculosis vaccine Bacillus Calmette-GuÃ©rin (BCG). |
| 154818 | Tlr2 | 22174456 | Tlr2 recognizes Mycobacterium tuberculosis H37Rv cell surface lipoprotein MPT83, which induces the production of Tnf, Il6, and Il12b cytokines by macrophages and upregulates macrophage function. |
| 154818 | Tlr2 | 22216191 | Mycobacterium abscessus glycopeptidolipid (GPL) prevents Tlr2-mediate induction of Il8 and Defb4a in respiratory epithelial cells. (Demonstrated in human) |
| 154818 | Tlr2 | 23994200 | Tlr2 is expressed in the enteric nervous system (ENS) and intestinal smooth muscle layers. Its absence induces architectural and neurochemical coding changes in the ENS, leading to gut dysmotility and to higher inflammatory bowel diseases susceptibility |
| 154818 | Tlr2 | 25423082 | Salmonella enterica serovar Typhimurium Î 👪msbB that possesses a modified lipid A triggers exacerbated colitis in the absence of Nod1 and/or Nod2, which is likely due to increased Tlr2 stimulation. |
| 154818 | Tlr2 | 25505250 | Adaptor proteins Ticam1 and Ticam2 have a novel function in Tlr2-mediated signal transduction. |
| 154818 | Tlr2 | 25826367 | Retinoic acid treatment enhances Tlr2-dependent Il10 production from T cells and this, in turn, potentiates T regulatory cell generation without the need for activation of antigen presenting cells. |
| 154818 | Tlr2 | 25586105 | Proline-proline-glutamic acid 57 (PPE57), located on the mycobacterial cell surface, induces a T helper 1 immune response via Tlr2-mediated macrophage functions. |
| 154818 | Tlr2 | 26078314 | Tlr2 is essential for the immune responses to cholera vaccination. |
| 154818 | Tlr2 | 26283364 | Staphylococcal superantigen-like protein 3 (SSL3) interferes with Tlr2 activation at two stages. First by binding to Tlr2 and blocking ligand binding and second by interacting with an already formed Tlr2-lipopeptide complex, thus preventing TLR heterodimerization and downstream signalling. |
| 154818 | Tlr2 | 26310831 | Peli3 is involved in endotoxin tolerance and functions as a negative regulator of Tlr2/4 signalling. |
| 154818 | Tlr2 | 26423153 | Cytokine activation as a result of Tlr2 stimulation is mediated by the phagosomal trafficking molecule Ap3b1 (AP-3). |

| | | | |
|---|---|---|---|
| 173348 | Ap3b1 | 21045126 | Ap3b1 (AP-3), a lysosome-related organelle trafficking and biogenesis protein, is required for the production of pro-inflammatory cytokines in plasmacytoid dendritic cells upon recognition of viral nucleic acids by endosomal Tlr7 or Tlr9. |
| 173348 | Ap3b1 | 21119105 | Ap3b1 is crucial for the trafficking of Tlr9 to specific endosomal compartments for the induction of type I interferon. |
| 173348 | Ap3b1 | 26423153 | Cytokine activation as a result of Tlr2 stimulation is mediated by the phagosomal trafficking molecule Ap3b1 (AP-3). |
| 195818 | Tlr9 | 11286707 | Tlr9 binds CpG DNA and induces dendritic cell maturation in a MyD88-dependent manner in mice. |
| 195818 | Tlr9 | 19923461 | Tlr9 engages in signalling crosstalk with Tlr4 during activation, and as a result, amplifies the inflammatory response of murine macrophages. |
| 195818 | Tlr9 | 17452530 | Tlr9, 7, and 3 interact with the endoplasmic reticulum (ER) membrane protein Unc93b and this is essential for proper TLR signaling. |
| 195818 | Tlr9 | 21376231 | Tlr9 signalling enhances the rate of acidification of the Salmonella-containing phagosome, and this acidification induces the expression of Salmonella pathogenecity genes that are necessary for intracellular survival, growth, and systemic infection. Tlr9 deficiency rescues the high Salmonella susceptibility phenotype observed in Tlr2,Tlr4 double mutant mice. |
| 195818 | Tlr9 | 21389263 | Tlr9 triggers plasmacytoid dendritic cells in Systemic Lupus Erythematosus patients upon recognition of self-antigens such as neutrophil extracellular traps (NETs). (Demonstrated in human) |
| 195818 | Tlr9 | 21402738 | Tlr9 requires proteolytic processing in endolysosome by asparagine endopeptidase and cathepsin in the endolysosome to initiate signalling in response to DNA. |
| 195818 | Tlr9 | 21439959 | Tlr9 deficiency reduced pancreatic edema, inflammation and pro-Il1b expression in pacreatitis. |
| 195818 | Tlr9 | 21482737 | Tlr9 synergistically interacts with Tlr2::Tlr6 in lung epithelium to induce rapid pathogen killing, and can be used as a therapeutic target to treat otherwise lethal pneumonia. |
| 195818 | Tlr9 | 21604257 | Tlr9 is proteolytically cleaved in the endosome to form a soluble Tlr9 (sTlr9), which inhibits Tlr9-dependent signalling and contributes to the prevention of autoimmune disease. |
| 195818 | Tlr9 | 21721026 | Tlr9 activation is enhanced by increased levels of circulating histones, serving as a crucial link between initial damage and activation of innate immunity during sterile inflammation |

| | | | |
|---|---|---|---|
| 195818 | Tlr9 | 21860217 | Tlr9 promotes macrophage Hif1a levels, oxidative burst and nitric oxide production in response to group A Streptococcus (GAS), contributing to GAS clearance in vivo in both localized cutaneous and systemic infection models. |
| 195818 | Tlr9 | 21947771 | Tlr9 is selectively compartmentalized to fungal phagosomes and negatively modulates macrophage anti-fungal effector functions. |
| 195818 | Tlr9 | 22342842 | Tlr9 expression and signalling capacity oscillates with the circadian clock. |
| 195818 | Tlr9 | 22535248 | Mitochondrial DNA that escapes from autophagy induces Tlr9 inflammatory responses in cardiomyocytes and is capable of inducing myocarditis and dilated cardiomyopathy. |
| 195818 | Tlr9 | 23071157 | TLR9 activation by endogenous self-ligands generated during oxidative stress promotes platelet hyper-reactivity and thrombosis. |
| 195818 | Tlr9 | 23142781 | Tlr9 contributes to the control of activated endogenous retroviruses (ERVs) and ERV-induced tumours. |
| 195818 | Tlr9 | 23752491 | The N-terminal region of TLR9 is cleaved in the endolysosome to form an intracellular DNA sensor with the C-terminal TLR9. |
| 195818 | Tlr9 | 24270517 | Mir126-Kdr axis is an important regulator of the innate response. Mir126 controls the survival and function of plasmacytoid dendritic cells and regulates gene expression of Tlr7, Tlr9, Nfkb1 and Kdr. |
| 195818 | Tlr9 | 25600358 | Dnase2a is required for Tlr9 activation by bacterial genomic DNA. |
| 195818 | Tlr9 | 26957214 | Tyrosine phosphorylation is essential for Tlr9 protein stability and signalling. |
| 195818 | Tlr9 | 26416273 | Tlr9 activation requires a single CpG positioned 4-6 nucleotides from the 5'-end and this activation is augmented by a 5'TCC sequence one to three nucleotides from the CG. |
| 162198 | Ifnb1 | 21408089 | Ifnb1 deficiency results in a partial suppression of the sterol pathway in macrophages during viral infections, thereby linking the regulation of lipid metabolism pathway with interferon anti-viral defence responses. |
| 162198 | Ifnb1 | 21602824 | Ifnb1 secretion is greater upon viable E. coli infection in comparison to heat killed E. coli vaccine or LPS. The induction of Ifnb1 is dependent on Ticam1-Irf3 signalling. |
| 162198 | Ifnb1 | 22291574 | Ifnb1 expression pattern during viral infection is a highly stochastic process influenced by cell-to-cell variability in viral induction processes. |
| 162198 | Ifnb1 | 22912878 | Ifnb1 production is fundamental to the efficient control of Listeria monocytogenes during the early innate phase of infection. NK cells treated with Ifnb1 during early infection were able to reduce bacterial titer in the spleen and significantly improve survival of infected mice. |

| | | | |
|---|---|---|---|
| 162198 | Ifnb1 | 24656046 | The noncanonical NF Î º B pathway regulates histone modifications at the Ifnb1 promoter resulting in attenuated recruitment of Rela and histone demethylase, Kdm4a, to the Ifnb1 promoter. This provides a mechanism for regulating the induction of type I interferons . |
| 162198 | Ifnb1 | 25517615 | The innate immune system plays a role in immunogenic tumour recognition. Tumor-cell-derived DNA triggers Ifnb1 production and dendritic cell activation via Tmem173 and Irf3 cytosolic DNA sensing pathways. |
| 162198 | Ifnb1 | 26202980 | Ifnb1 selectively restricts the transcriptional responses mediated by both the TLRs and the NOD-like receptors in Salmonella enterica serovar Typhimurium infection in macrophages. |
| 162198 | Ifnb1 | 26416280 | Atf3 plays an important role in modulating IFN responses in macrophages by controlling basal and inducible levels of Ifnb1, as well as the expression of genes downstream of IFN signalling. |
| 208712 | Atf3 | 24317040 | Atf3 has been identified as a high-density lipoprotein â € inducible negative regulator of macrophage activation. |
| 208712 | Atf3 | 26416280 | Atf3 plays an important role in modulating IFN responses in macrophages by controlling basal and inducible levels of Ifnb1, as well as the expression of genes downstream of IFN signalling. |
| 178924 | Nlrp3 | 19362020 | Nlrp3 mediated immune responses can be activated by RNA and mice lacking Nlrp3, or other inflammasome components, exhibit increased mortality and a reduced immune response after influenza virus exposure. |
| 178924 | Nlrp3 | 19362023 | Nlrp3(-/-) mice exhibit increased morbidity after infection with a pathogenic influenza A virus correlating with decreased neutrophil and monocyte recruitment and reduced IL-1beta, IL-18, TNF-alpha, IL-6, KC, MIP2, and IFN-alpha production. |
| 178924 | Nlrp3 | 21278344 | Nlrp3 is directly activated by certain antibiotics and plays an important role in the antibiotic-mediated secretion of Il1b. In the case of polymyxin B, Nlrp3 was also required for the neutrophil influx into the peritoneal cavity. |
| 178924 | Nlrp3 | 21289120 | Nlrp3 inflammasome is essential for host defence against influenza and other RNA viruses (i.e. EMCV, VSV). |
| 178924 | Nlrp3 | 21385879 | Nlrp3 recruits adaptor protein Pycard and Casp1 to form an Nlrp3 inflammasome complex in response to Varicella-Zoster Virus (VZV) infection. |
| 178924 | Nlrp3 | 21439959 | Nlrp3 is a component of the inflammasome and is required for inflammation in acute pancreatitis. |

| | | | |
|---|---|---|---|
| 178924 | Nlrp3 | 21602824 | Nlrp3 is necessary to illicit Il1b response specific to viable, but not heat-killed, E. coli infections. |
| 178924 | Nlrp3 | 22174673 | Nlrp3 inflammasome plays a role in innate immune responses against mucosal Candida infection. Nlrp3 limits the severity of infection when present in either the hematopoietic or stromal compartments. |
| 178924 | Nlrp3 | 22295065 | Nlrp3/Pycard inflammasome activation following human respiratory syncytial virus infection is dependent on the activation of Tlr2/Myd88/NF-kB and reactive oxygen species/potassium efflux. (Demonstrated in human) |
| 178924 | Nlrp3 | 22984081 | MIR223 and EBV miR-BART15 regulate the NLRP3 inflammasome and IL-1beta production. |
| 178924 | Nlrp3 | 23809161 | Potassium efflux is a common trigger of NLRP3 Inflammasome activation by bacterial toxins and particulate matter |
| 178924 | Nlrp3 | 24127597 | Mitochondrial membrane potential is required for the association of Nlrp3 and Mfn2. Mfns2 is required for the activation of Nlrp3 inflammasomes. |
| 178924 | Nlrp3 | 24265316 | 3, 4-methylenedioxy- Î ² -nitrostyrene is a potent and specific inhibitor of the NLRP3 inflammasome. |
| 178924 | Nlrp3 | 24549849 | Both Nlrp3 and Nlrp1a are important regulators of Toxoplasma proliferation and that IL18 signaling is required to mediate host resistance to acute toxoplasmosis. |
| 178924 | Nlrp3 | 24692555 | Group B streptococcus induces Il1b, and activates the NLRP3 inflammasome by a mechanism that requires hemolysin-mediated lysosomal leakage, which enhances the interaction of bacterial RNA with NLRP3. |
| 178924 | Nlrp3 | 25689249 | Activation of the Nlrp3 inflammasome is detrimental during leishmaniasis. Mice lacking the inflammasome components Nlrp3, Pycard, Casp1 exhibit defective Il1b and Il18 production at the infection site and are resistant to cutaneous Leishmania major infection. |
| 178924 | Nlrp3 | 26045547 | Nlrp3 is a novel molecular target for melatonin which requires Rora to blunt the NFkB/ NLRP3 connection during sepsis. |
| 178924 | Nlrp3 | 27214553 | Uropathogenic Escherichia coli protein TcpC attenuates activation of the Nlrp3 inflammasome by binding both Nlrp3 and Casp1. |
| 178924 | Nlrp3 | 26305961 | Nlrp3 deficiency protects mice from the development of type 1 diabetes by suppressing Th1 responses and impairing T-cell migration to pancreatic islets through the down-regulation of chemokine expression (Ccl5, Cxcl10, Irf1) in islets. |
| 178924 | Nlrp3 | 26341399 | Nlrp3 and Casp2 are required for endoplasmic reticulum stress-induced inflammation. |

| | | | |
|---|---|---|---|
| 178924 | Nlrp3 | 26416893 | Impairment of the mitochondrial electron transport chain by rotenone primes Nlrp3 inflammasome activation upon costimulation with ATP. |
| 63225 | IRF3 | 9463386 | IRF3 is a transcription factor that activates type-1 interferon (IFN) and IFN responsive genes. |
| 63225 | IRF3 | 11991981 | IRF3 is directly activated after virus infection and functions as a key activator of the intermediate/early alpha/beta interferon (IFN) genes as well as the RANTES chemokine gene. |
| 63225 | IRF3 | 18818105 | IRF3 transcription factor induces type I interferons (IFNs) and elicits innate antiviral response. TMEM173 (MITA) is a critical mediator of virus-triggered IRF3 activation and IFN expression. |
| 63225 | IRF3 | 9566918 | IRF3 phosphorylation is virus-inducible and results in IRF3 alteration of protein conformation to permit nuclear translocation, association with transcriptional partners, and primary activation of interferon (IFN)- and IFN-responsive genes. |
| 63225 | IRF3 | 21768204 | IRF3 is strongly phosphorylated at the late stages of a Sindbis virus infection to mount antiviral responses in human embryonic kidney cells. |
| 63225 | IRF3 | 21820332 | IRF3 is involved in the innate immune recognition of Plasmodium falciparum AT-rich DNA and in the subsequent induction of type I IFNs. Mice lacking Irf3/Irf7 are resistant to otherwise lethal cerebral malaria. (Demonstrated in mouse) |
| 63225 | IRF3 | 22170100 | IRF3 suppresses neuroinflammation through regulation of immunomodulatory MIR155 microRNA expression in astrocytes. |
| 63225 | IRF3 | 22593165 | HIV accessory protein Vpu targets IRF3 to endolysosome for proteolytic degradation to avoid antiviral immune responses. |
| 63225 | IRF3 | 23994473 | During the transcriptional response to Sendai virus infection, POLR2F(RNA Pol II) is recruited by IRF3 and NFÎºB to control virus induced gene activation. |
| 63225 | IRF3 | 25352594 | Vpu, an accessory protein encoded by HIV-1, contributes to the attenuation of the anti-viral response by partial inactivation of IRF3 while host cells undergo apoptosis. |
| 63225 | IRF3 | 25505063 | Hepatitis B virus (HBV) polymerase inhibits TMEM173-stimulated IRF3 activation and IFNB1 induction. |
| 63225 | IRF3 | 25792739 | Stimulation of TMEM173-dependent IRF3 activation by ultraviolet radiation is due to apoptotic signalling-dependent disruption of ULK1, a pro-autophagic protein that negatively regulates TMEM173. |
| 63225 | IRF3 | 26046437 | PQBP1 directly binds to reverse-transcribed HIV-1 DNA and interacts with MB21D1 to initiate an IRF3-dependent innate response |

| | | | |
|---|---|---|---|
| 63225 | IRF3 | 26646986 | 4-(2-chloro-6-fluorobenzyl)-N-(furan-2-ylmethyl)-3-oxo-3,4-dihydro-2H-benzo[b][1,4]thiazine-6-carboxamide (G10) requires STING to trigger IRF3/IFN-associated transcription in human fibroblasts and subsequently blocking replication of Chikungunya virus, Venezuelan Encephalitis virus, and Sindbis virus. |
| 63225 | IRF3 | 26378160 | Influenza B virus induces IRF3 activation and IL29 (IFNL1) gene expression without a requirement for viral protein synthesis or replication and DDX58 is the critical pattern recognition receptor needed for IRF3 activation. |
| 50119 | IL29 | 21499846 | IL29 is able to efficiently inhibit Herpes Simplex Virus Type-1 replication in neuronal cells by inducing the expression of TLR as well as activate the TLR-mediated interferon antiviral pathway. |
| 50119 | IL29 | 24041672 | IFNL1 (IL29), IFNL2 and IFNL3 have different effects on Toll-like receptor-related gene expression in HepG2 cells. |
| 50119 | IL29 | 26130701 | IFN-Î» 1 is able to augment TLR-mediated B cell activation, partially attributed to an upregulation of TLR7 expression |
| 50119 | IL29 | 26378160 | Influenza B virus induces IRF3 activation and IL29 (IFNL1) gene expression without a requirement for viral protein synthesis or replication and DDX58 is the critical pattern recognition receptor needed for IRF3 activation. |
| 40102 | TRAF6 | 16184196 | TRAF6 is an adapter protein linking kinases to TNF receptor, and IL-1 receptor signalling pathways, and also has E3 ubiquitin ligase activity. |
| 40102 | TRAF6 | 18617513 | TRAF6 autoubiquitination and its interaction with Ubc13 are dependent on zinc finger 1 (ZF1) motif and an intact RING domain, necessary elements in signalling by IL-1, LPS and RANKL. |
| 40102 | TRAF6 | 18922473 | TRAF6 is specifically required for the Smad-independent activation of JNK and p38, and its carboxyl TRAF homology domain physically interacts with TGF-beta receptors that activate JNK and p38 through a mechanism similar to that operating in the interleukin-1beta/Toll-like receptor pathway. |
| 40102 | TRAF6 | 18984593 | TRAF6 and MEK kinase 1 (MEKK1) play a pivotal role in the retinoic-acid-inducible gene-I (RIG-I)-like helicase antiviral pathway, where TRAF6 and MEKK1 activate NF-kappaB and mitogen-activated protein kinases via MAVS and this is critical for the optimal induction of type I interferons. |
| 40102 | TRAF6 | 19091594 | TRAF6 negatively regulates TNFalpha-induced NF-kappaB activation through its ubiquitin ligase activity. |
| 40102 | TRAF6 | 12140561 | TRAF6 regulates several signalling cascades in innate immunity, adaptive immunity and bone homeostasis. |

| | | | |
|---|---|---|---|
| 40102 | TRAF6 | 20449947 | TRAF6 competes with TRAF2 for CD40 binding to regulate NF-kappaB activation in human B lymphocytes, thereby limiting the capacity of CD40 engagement to induce NF-kappaB activation. |
| 40102 | TRAF6 | 20512936 | TRAF6 is autoinhibited by an intramolecular interaction which is counteracted by trans-ubiquitination and TRAF auto-ubiquitination is a means of sustaining an open conformation active in downstream signalling. |
| 40102 | TRAF6 | 20622119 | TRAF6 interacts with CSF2RB to mediate NF-kappaB signalling, demonstrating a novel TRAF6-dependent signalling pathway association with a type I cytokine receptor. |
| 40102 | TRAF6 | 21185369 | TRAF6 is a E3 ubiquitin ligase that activates NFKB pathway in response to innate and adaptive immunity stimuli. TRAF6 protein contains a highly conserved TRAF-C domain that contributes to oligomerization and its interaction to upstream signalling molecules and a RING domain dimerization interface that is functionally important for ubiquitination and the activation of NFKB. |
| 40102 | TRAF6 | 21220427 | TRAF6 is polyubiquitinated and disassembled during endotoxin tolerization; a process which impairs the production of LPS-induced pro-inflammatory cytokines without inhibition expression of anti-inflammatory or anti-microbial mediators. |
| 40102 | TRAF6 | 22033459 | TRAF6 is degraded in the proteasome upon TLR stimulation in macrophages. (Demonstrated in mice) |
| 40102 | TRAF6 | 24670424 | Following NOD2 activation, IRF4 interacts with MYD88, TRAF6, and RIPK2 and downregulates K63-linked polyubiquitinylation of RICK and TRAF6 leading to disruption of NFkB activation pathways. |
| 40102 | TRAF6 | 24670381 | MIR146A is a potent negative regulator of the innate immune response in keratinocytes through downregulation of the IRAK1/TRAF6/NFÎºB pathway. |
| 40102 | TRAF6 | 25027037 | STAT1 is directly recruited to TRAF6, demonstrating cross-talk between the TLR and JAK/STAT signalling pathways, and this direct activation of STAT1 by TLR signalling suggests a crucial role for STAT1 in TLR-induced inflammation. Demonstrated in mice. |
| 40102 | TRAF6 | 25371197 | ECSIT binds to MAP3K7 and TRAF6 to form a complex that plays a pivotal role in activating TLR4-mediated NF-kB signalling. |
| 40102 | TRAF6 | 26221041 | Reversible arginine methylation of TRAF6 is regulated by PRMT1 and JMJD6 and this in turn regulates TRAF6-dependent TLR signalling. |
| 40102 | TRAF6 | 26385923 | MAVS directly interacts with TRAF6 through its potential TRAF6-binding motif 2. |

| | | | |
|---|---|---|---|
| 49080 | MAVS | 19036819 | MAVS oligomer is essential in the formation of a multiprotein membrane-associated signalling complex that enables downstream activation of IRF3 and NF-kappaB in antiviral innate immunity. |
| 49080 | MAVS | 16177806 | MAVS is a caspase recruitment domain (CARD)-containing adaptor protein that interacts with DDX58 (RIG-I) and recruits CHUK (IKKalpha), IKBKB (IKKbeta) and IKBKE (IKKepsilon) kinases by means of its C-terminal region, leading to the activation of NF-kappaB and IRF3. |
| 49080 | MAVS | 16125763 | MAVS is an adaptor protein that contains an N-terminal caspase recruitment domain (CARD)-like domain and a C-terminal transmembrane domain, both of which are essential for MAVS signalling, while the transmembrane domain also targets MAVS to the mitochondria. |
| 49080 | MAVS | 20032188 | MAVS facilitates cell death by disrupting the mitochondrial membrane potential and by activating caspases. |
| 49080 | MAVS | 20140199 | MAVS-dependent RIG-I-like receptor (RLR) signalling regulates the quantity, quality, and balance of the immune response to West Nile virus (WNV) infection. |
| 49080 | MAVS | 20451243 | MAVS in peroxisomes induces a rapid interferon-independent expression of defence factors that provide short-term protection, whereas mitochondrial MAVS activates an interferon-dependent signalling pathway with delayed kinetics, which amplifies and stabilizes the antiviral response. |
| 49080 | MAVS | 20554965 | MAVS interacts with hepatitis B virus X protein and this promotes the degradation of MAVS via Lys(136) ubiquitination, preventing the induction of IFN-beta. |
| 49080 | MAVS | 20661427 | MAVS (IPS-1) interacts with MFN1 upon virus-infection or 5'ppp-RNA activation through redistribution of MAVS to form speck-like aggregates in cells. |
| 49080 | MAVS | 20699220 | MAVS (IPS1) plays an important role in regulating the host anti-viral response by binding to viral polymerase and inhibiting IFN-beta production. |
| 49080 | MAVS | 21110072 | MAVS negatively regulates the stability of voltage-dependent anion channel 1 (VDAC1) and thereby inhibits apoptosis in the response to release of cytochrome c. |
| 49080 | MAVS | 21454965 | The MAVS signalling pathway in non-myeloid cells is crucial for dsRNA-mediated natural killer cell activation. (Demonstrated in murine model) |
| 49080 | MAVS | 22844514 | Tyrosine phosphorylation of MAVS at amino acid residue Tyr9 is critical for the induction of IFNB signalling. |

| | | | |
|---|---|---|---|
| 49080 | MAVS | 23028806 | MAVS mRNA is degraded in response to foreign RNA and poly(I:C) to suppress hyper-immune reaction in late-phase antiviral signalling. |
| 49080 | MAVS | 23582325 | MAVS is required for optimal NLRP3 inflammasome activity by mediating mitochondrial recruitment of NLRP3. |
| 49080 | MAVS | 23555247 | MAVS is targeted by enterovirus protease to evade antiviral immunity. |
| 49080 | MAVS | 23951545 | The binding of MAVS to Traf2, Traf5, and Traf6 is dependent on virus infection and MAVS polymerization. The TRAF proteins promote ubiquitination that recruits IKBKG binding to the MAVS signalling complex. |
| 49080 | MAVS | 24651600 | Upon viral infection, MAVS recruits MKK7 onto mitochondria, leading to the induction of apoptosis by MAP2K7 activated MAPK9 |
| 49080 | MAVS | 25463536 | Macrocyclic NS3-4A resistance-associated amino acid variants (RAVs) with substitutions at residue D168 of the hepatitis C virus protease result in an increased capacity of NS3-4A to cleave MAVS and suppress IFNB1 induction. |
| 49080 | MAVS | 25816776 | RNA cleavage products, catalyzed by RNASEL, bind to DHX33 to facilitate the formation of a complex with MAVS and NLRP3 during viral infection. |
| 49080 | MAVS | 27213432 | HACE1 plays an inhibitory role in virus-induced signalling by disrupting the MAVS-TRAF3 complex. |
| 49080 | MAVS | 26246171 | MARCH5 modulates MAVS-mediated antiviral signalling, preventing excessive immune reactions. |
| 49080 | MAVS | 26385923 | MAVS directly interacts with TRAF6 through its potential TRAF6-binding motif 2. |
| 19987 | IKBKB | 18692471 | IKBKB regulates late-phase allergic reactions promoted by the release of pro-inflammatory cytokines in an NF-kappaB-dependent manner. |
| 19987 | IKBKB | 20627395 | Phosphorylated IKBKB is conjugated with a monoubiquitin by the E3 ubiquitin ligase TRIM21 leading to down-regulatioin of IKBKB-induced NF-kappaB signalling. The TRIM21-mediated monoubiquitination is involved in the translocation of active IKBKB to autophagosomes. |
| 19987 | IKBKB | 21138416 | IKBKB and other IKK kinases regulate each other by an intricate network involving phosphorylation of their catalytic and regulatory (NEMO, TANK) subunits to balance their activities during innate immunity. |
| 19987 | IKBKB | 24323043 | GNB2L1 (RACK1) negatively regulates NF Î º B activation by interacting with CHUK and IKBKB. The interaction interferes with the recruitment of the IKK complex to TRAF2 . |
| 19987 | IKBKB | 26620909 | The reversible ubiquitin editing of NLRC5 determines NLRC5â€ IKBKB interaction dynamics and plays a crucial role in precisely regulating NFÎºB signalling |

| | | | |
|---|---|---|---|
| 19987 | IKBKB | 26394554 | Enterovirus 71 2C protein binds to RELA and IKBKB to inhibit NF-kB activation and evade innate immune defenses. |
| 57543 | RELA | 11980335 | RELA, NF-kappaB p65 subunit, is involved in the transcription regulation of many genes including those genes involved in apoptosis, response to stress and inflammation. |
| 57543 | RELA | 21209118 | RELA is a subunit of NFKB and is not essential for virus-stimulated IFNB expression, instead, RELA sustains autocrine IFNB signalling prior to infection. The absence of RELA causes significant delays in IFNB induction and consequently defective secondary antiviral gene expression. RELA maintains autocrine IFNB signalling in uninfected cells, facilitates inflammatory and adaptive immune responses following infection, and promotes infected cell survival during this process. |
| 57543 | RELA | 21216972 | RELA is critical for pulmonary host defence during Streptococcus pneumoniae pneumonia in alveolar macrophages. During pneumococcal pneumonia, only the earliest induction of cytokines depends on transcription regulated by RELA in myeloid cells, and this transcriptional activity contributes to effective immunity. (Demonstrated in murine model) |
| 57543 | RELA | 21419662 | RELA is required for IL17A production in T cell in response to bacterial infection. RELA deficient T cells resulted in a diminished innate immune response to E. coli infection. (Demonstrated in murine model) |
| 57543 | RELA | 23271966 | A RELA isoform, p43, lacks the transactivation domain but is still able to potentiate anti-viral innate immunity. |
| 57543 | RELA | 23994473 | During the transcriptional response to Sendai virus infection, POLR2F(RNA Pol II) is recruited by IRF3 and NFÎºB to control virus induced gene activation. |
| 57543 | RELA | 25520509 | Paramyxoviruses trigger the DNA-damage response, a pathway required for RPS6KA5 activation of phospho Ser 276 RELA formation to trigger the IRF7-DDX58 amplification loop necessary for mucosal interferon production. |
| 57543 | RELA | 26055519 | Human papillomaviruses impair the acetylation of NFÎºB/RelA K310 in keratinocytes by augmenting the expression of interferon-related developmental regulator 1 (IFRD1) in an EGFR-dependent manner. |
| 57543 | RELA | 26642243 | Haploinsufficiency of A20 (HA20) is caused by high-penetrance loss-of-function germline mutations in TNFAIP3 with increased degradation of NFKBIA, nuclear translocation of RELA, increased expression of NF Î º B mediated proinflammatory cytokines, and defective deubiquitinating activity. |

| | | | |
|---|---|---|---|
| 57543 | RELA | 26296289 | MIR223 regulates macrophage function by modulating cytokine production and NF-Îº B activation through inhibition of RELA phosphorylation and nuclear translocation. |
| 57543 | RELA | 26394554 | Enterovirus 71 2C protein binds to RELA and IKBKB to inhibit NF-kB activation and evade innate immune defenses. |
| 181123 | Irf3 | 21768204 | Irf3 is strongly phosphorylated at the late stages of a Sindbis virus infection to mount antiviral responses in human embryonic kidney cells. (Demonstrated in human) |
| 181123 | Irf3 | 21820332 | Irf3 is involved in the innate immune recognition of Plasmodium falciparum AT-rich DNA and in the subsequent induction of type I IFNs. Mice lacking Irf3/Irf7 are resistant to otherwise lethal cerebral malaria. |
| 181123 | Irf3 | 22170100 | Irf3 suppresses neuroinflammation through regulation of immunomodulatory mmu-mir-155 microRNA expression in astrocytes. (Demonstrated in human) |
| 181123 | Irf3 | 22593165 | HIV accessory protein Vpu targets Irf3 to endolysosome for proteolytic degradation to avoid antiviral immune responses. (Demonstrated in human) |
| 181123 | Irf3 | 23028052 | Endoplasmic reticulum stress primes macrophages to respond to innate immunity stimuli by activating IRF3. |
| 181123 | Irf3 | 24362933 | Plasmodium RNA is a pathogen-associated molecular pattern (PAMP) capable of activating a type I IFN response via the cytosolic pattern recognition receptors Ifih1 and Mavs, as well as via transcription factors Irf3 and Irf7. |
| 181123 | Irf3 | 25431490 | Il28ra (Ifnlr1), Stat1 and Irf3 are required for antibiotics to prevent persistent murine norovirus infection. |
| 181123 | Irf3 | 25517615 | The innate immune system plays a role in immunogenic tumour recognition. Tumor-cell-derived DNA triggers Ifnb1 production and dendritic cell activation via Tmem173 and Irf3 cytosolic DNA sensing pathways. |
| 181123 | Irf3 | 25642965 | Aberrant mitochondrial DNA (mtDNA) packaging promotes escape of mtDNA into the cytosol, where it engages the DNA sensor Mb21d1 and promotes Tmem173-Irf3-dependent signalling to elevate IFN-stimulated gene expression, potentiate type I IFN responses and confer broad viral resistance. |
| 181123 | Irf3 | 26363053 | Ppp4c, a serine/threonine phosphatase, directly binds to Tbk1 upon virus infection to dephosphorylate Tbk1 and inhibit Tbk1 activation, and subsequently restrain Irf3 activation, resulting in suppressed production of type I IFN and IFN-stimulated genes. |
| 192882 | Tbk1 | 21820332 | Tbk1 is involved in the innate immune recognition of Plasmodium falciparum AT-rich DNA and in the subsequent induction of type I IFNs. Mice lacking Tbk1 are resistant to otherwise lethal cerebral malaria. |

| | | | |
|---|---|---|---|
| 192882 | Tbk1 | 22921120 | Tbk1 is a key regulator of immunological autophagy and is responsible for autophagosome maturation into bactericidal organelles. |
| 192882 | Tbk1 | 26363053 | Ppp4c, a serine/threonine phosphatase, directly binds to Tbk1 upon virus infection to dephosphorylate Tbk1 and inhibit Tbk1 activation, and subsequently restrain Irf3 activation, resulting in suppressed production of type I IFN and IFN-stimulated genes. |
| 209838 | Ppp4c | 26363053 | Ppp4c, a serine/threonine phosphatase, directly binds to Tbk1 upon virus infection to dephosphorylate Tbk1 and inhibit Tbk1 activation, and subsequently restrain Irf3 activation, resulting in suppressed production of type I IFN and IFN-stimulated genes. |
| 204565 | Myd88 | 20881045 | Myd88 restricts West Nile virus (WNV) by inhibiting replication in subsets of cells and modulating expression of chemokines that regulate immune cell migration into the central nervous system. |
| 204565 | Myd88 | 20943980 | Myd88 signalling plays an important role for resisting primary influenza virus infection but is dispensable for protection against a secondary lethal challenge. |
| 204565 | Myd88 | 21248248 | Myd88 is essential in restricting Tlr3 signaling and the host protection from unwanted immunopathologies associated with excessive production of Ifnb1. Myd88 inhibits Tlr3 signalling by impairing Ikbke-mediated induction of Irf3, and consequently the expression of Ifnb1 and Ccl5. |
| 204565 | Myd88 | 21283748 | Myd88 is activated by MHC class II in response to staphylococcal enterotoxins and is crucial for the induction of pro-inflammatory cytokines. |
| 204565 | Myd88 | 21325272 | MYD88 is a key signalling adapter in TLR signalling. MYD88 aggregates in the cell as distinct foci and co-localizes with IRAK4 in these Myddosomes - the formation of which is required for MYD88 function. (Demonstrated in human) |
| 204565 | Myd88 | 21353603 | Myd88 is required in dendritic cells stimulated with Tlr9 ligand for the enhancement of T cell-dependent antibody response. In addition, Myd88 is required in B cells to facilitate strong anti-viral antibody responses. |
| 204565 | Myd88 | 21422180 | Myd88 deficient macrophages displayed impaired interaction with fungal yeast cells and produced low levels of pro-inflammatory cytokines. Myd88 signalling is important in the activation of fungicidal mechanisms and the induction of protective innate immune responses against P. brasiliensis. |
| 204565 | Myd88 | 22028692 | Myd88 mediates cytoskeletal remodelling and late spreading of lipopolysaccharide (LPS)-stimulated macrophages. |

| | | | |
|---|---|---|---|
| 204565 | Myd88 | 22025508 | Myd88-dependent recruitment of inflammatory monocytes and dendritic cells to the lungs are key initial cellular responses required for early protection from Burkholderia mallei infection. |
| 204565 | Myd88 | 22386951 | Myd88 deficiency results in delayed recruitment of phagocytes and defective production of proinflammatory cytokines in response to Salmonella infection. |
| 204565 | Myd88 | 22491177 | Myd88 signalling in intestinal epithelial cells is crucial for the maintenance of gut microbiota homeostasis. |
| 204565 | Myd88 | 22536449 | Myd88 mediated production of reactive oxygen species (ROS) is essential for the induction of Il12 by lactic acid bacteria. |
| 204565 | Myd88 | 24442437 | Flagellin-specific IgG1 antibody response is induced through a Tlr5-, inflammasome-, and Myd88-independent pathway. |
| 204565 | Myd88 | 24204290 | The SF3A/SF3B mRNA splicing complexes regulate the innate immune response in part by regulating Myd88S levels, which modulate the extent of the innate immune response through Tlr4. |
| 204565 | Myd88 | 24375488 | Mir149 negatively regulates TLR/Myd88 mediated inflammatory responses in macrophages by targeting Myd88 mRNA. |
| 204565 | Myd88 | 25448706 | Myd88 and Ticam1 pathways differently regulate Tlr4-induced immune responses in B cells. |
| 204565 | Myd88 | 25548220 | Ticam1 but not Myd88 signalling is critical for the Trl4 protective adjuvant effect in neonates; where Ticam1(-/-) but not Myd88(-/-) neonates are highly susceptible to Escherichia coli peritonitis and bacteremia. |
| 204565 | Myd88 | 25848864 | Treml4 is an essential positive regulator of Tlr7 signalling. Treml4(-/-) macrophages are hyporesponsive to Tlr7 agonists and fail to produce type I interferons due to impaired phosphorylation of Stat1 by Mapk14 and decreased recruitment of Myd88 to Tlr7. |
| 204565 | Myd88 | 25808990 | Intracellular Sef/IL-17R (SEFIR) domain of Il17rd targets TIR adaptor proteins Myd88, Tirap, Ticam1, Ticam2 and Traf6 to inhibit TLR downstream signalling. |
| 204565 | Myd88 | 26565030 | Map1s (Mtap1s) controls bacterial phagocytosis through TLR signalling by interacting directly with Myd88. |
| 204565 | Myd88 | 27196572 | Hepatocyte Myd88 affects bile acids, gut microbiota and metabolome contributing to regulation of glucose and lipid metabolism. |
| 204565 | Myd88 | 26363072 | Extracellular RNA of cardiac origin exhibits a potent pro-inflammatory property in vitro and in vivo and induces cytokine production through Tlr7-Myd88 signalling. |
| 185532 | Tlr7 | 17452530 | Tlr7, 9, and 3 interact with the endoplasmic reticulum (ER) membrane protein UNC93B and this is essential for proper TLR signaling. |

| | | | |
|---|---|---|---|
| 185532 | Tlr7 | 21037581 | Tlr7 is expressed in C-fiber primary sensory neurons and is important for inducing itch (pruritus), but is not necessary for eliciting mechanical, thermal, inflammatory and neuropathic pain in mice |
| 185532 | Tlr7 | 21282509 | Tlr7 signaling pathway plays a pivotal role in fungal pathogen recognition and is essential for the subsequent Ifnb signaling. |
| 185532 | Tlr7 | 21402738 | Tlr7 requires proteolytic processing in endolysosome by asparagine endopeptidase and cathepsin in the endolysosome to initiate signalling. |
| 185532 | Tlr7 | 21487111 | Tlr7 agonist, such as imidazoquinolines, accumulate in the MHC class II loading compartment - this pH-dependent localization is required for the activation of plasmacytoid dendritic cells. (Demonstrated in human) |
| 185532 | Tlr7 | 21730058 | TLR7 inflammatory signalling leads to cardiac fibrosis in autoimmune associated congenital heart block.. (Demonstrated in human) |
| 185532 | Tlr7 | 21734241 | Tlr7 and Tlr8 are translocated from the endoplasmic reticulum to the endosome in the presence of antiphospholipid antibodies, as a consequence, plasmacytoid dendritic cells become dramatically sensitized to Tlr7/8 agonists and this may play a role in systemic autoimmunity. |
| 185532 | Tlr7 | 21998589 | Tlr7 is responsible for the detection of retroviruses and serves as a key checkpoint controlling the development of germinal center B cells. |
| 185532 | Tlr7 | 22396599 | Tlr7 signalling induces autophagy in HIV-infected plasmacytoid dendritic cells; this process is necessary for the induction of IFN-alpha. (Demonstrated in mice) |
| 185532 | Tlr7 | 22753494 | Tlr7 binds to exosomal Mir21 and Mir29a secreted by tumour cells and initiates a prometastatic inflammatory response. |
| 185532 | Tlr7 | 22952664 | Aberrant TLR7 activation induces Epstein-Barr viral protein LMP1 expression, which exacerbates IFN production in lupus patients. (Demonstrated in human) |
| 185532 | Tlr7 | 23142781 | Tlr7 contributes to the control of activated endogenous retroviruses (ERVs) and ERV-induced tumours. |
| 185532 | Tlr7 | 24270517 | Mir126-Kdr axis is an important regulator of the innate response. Mir126 controls the survival and function of plasmacytoid dendritic cells and regulates gene expression of Tlr7, Tlr9, Nfkb1 and Kdr. |
| 185532 | Tlr7 | 25848864 | Treml4 is an essential positive regulator of Tlr7 signalling. Treml4(-/-) macrophages are hyporesponsive to Tlr7 agonists and fail to produce type I interferons due to impaired phosphorylation of Stat1 by Mapk14 and decreased recruitment of Myd88 to Tlr7. |

| | | | |
|---|---|---|---|
| 185532 | Tlr7 | 25917529 | Neuronal Tlr7 recognizes endogenous ligands such as the miRNAs Let7c and miR21 and plays a negative role in controlling neuronal growth in a cell-autonomous manner. |
| 185532 | Tlr7 | 26363072 | Extracellular RNA of cardiac origin exhibits a potent pro-inflammatory property in vitro and in vivo and induces cytokine production through Tlr7-Myd88 signalling. |
| 134008 | Tslp | 26371187 | Epithelial cell-intrinsic Chuk expression and Tslp regulate group 3 innate lymphoid cell responses required to maintain intestinal barrier immunity. |
| 169132 | Chuk | 21765415 | Chuk has a key role in the negative feedback of NF-kB canonical signalling by orchestrating the assembly of the A20 ubiquitin-editing complex to limit inflammatory gene activation in response to proinflammatory stimuli such as Tnf and Il1. |
| 169132 | Chuk | 26371187 | Epithelial cell-intrinsic Chuk expression and Tslp regulate group 3 innate lymphoid cell responses required to maintain intestinal barrier immunity. |
| 81992 | IFIT5 | 26334375 | IFIT5 is a positive regulator in IKK phosphorylation and NF-Î°B activation. |
| 197036 | Stat2 | 26335850 | Transcriptional activation of Adar by IFN occurs in the absence of Stat1 by a non-canonical Stat2-dependent pathway. |
| 153162 | Stat1 | 22065572 | Stat1 phosphorylation at Ser708 is a key event in the IFN signalling pathway that imparts anti-viral immunity to restrict West Nile virus infection. |
| 153162 | Stat1 | 22425562 | Histone deacetylase inhibitors prevent Ifng-mediated phosphorylation of Stat1. (Demonstrated in human) |
| 153162 | Stat1 | 25431490 | Il28ra (Ifnlr1), Stat1 and Irf3 are required for antibiotics to prevent persistent murine norovirus infection. |
| 153162 | Stat1 | 25972472 | Cd81 inhibits Rac1/Stat1 activation and negatively regulates the defence mechanisms to Listeria monocytogenes infection. |
| 153162 | Stat1 | 25848864 | Treml4 is an essential positive regulator of Tlr7 signalling. Treml4(-/-) macrophages are hyporesponsive to Tlr7 agonists and fail to produce type I interferons due to impaired phosphorylation of Stat1 by Mapk14 and decreased recruitment of Myd88 to Tlr7. |
| 153162 | Stat1 | 26335850 | Transcriptional activation of Adar by IFN occurs in the absence of Stat1 by a non-canonical Stat2-dependent pathway. |
| 164791 | Adar | 21809195 | Adar destabalizes RNA structure by the deamination of adenosine to inosine, and therefore is able to disrupt replication of dsRNA viruses in the host. |
| 164791 | Adar | 26335850 | Transcriptional activation of Adar by IFN occurs in the absence of Stat1 by a non-canonical Stat2-dependent pathway. |

| | | | |
|---|---|---|---|
| 191912 | Ticam1 | 21454965 | The Ticam1 signalling pathway in murine dendritic cells is crucial for dsRNA-mediated natural killer cell activation. |
| 191912 | Ticam1 | 21494017 | Ticam1 deficiency results in the impairment of LPS-stimulated TNF-alpha protein translation. |
| 191912 | Ticam1 | 21602824 | Ticam1 is crucial for Nlrp3 inflammasome activation in response specific to viable, but not heat-killed, E. coli infections. |
| 191912 | Ticam1 | 21697485 | Ticam1 is proteolytically cleaved by Enterovirus 71 to inhibit the induction of innate immunity by Tlr3-signalling. Ticam1 cleavage results in the inhibition of NFkB and IFNB promoter activation. (Demonstrated in human) |
| 191912 | Ticam1 | 21703541 | Ticam1 forms a dsRNA sensor complex with components Ddx1, Ddx21 and Dhx36 to trigger the type I interferon and cytokine response to poly I:C, influenza A virus, and reovirus. |
| 191912 | Ticam1 | 21760953 | Ticam1 is a potent negative regulator of TLR agonist-triggered immune responses, specifically suppressing Il12 in dendritic cells and Ifng in natural killer cells. |
| 191912 | Ticam1 | 22072781 | Ticam1-Tlr3-mediated signalling pathway plays an essential role in the anti-viral response against poliovirus infection. |
| 191912 | Ticam1 | 22124111 | Ticam1 plays a role in host resistance to Gram-negative enteropathogens. Ticam1-mediated protective immunity is orchestrated by macrophage-induced IFN-beta and natural killer cell production of IFN-gamma. |
| 191912 | Ticam1 | 22123964 | Ticam1 forms a complex with Ripk3 upon Toll-like receptors (TLR) 3 and 4 activation resulting in Ripk3-dependent but TNF-independent necrosis in macrophages. |
| 191912 | Ticam1 | 25389373 | High-potency Tlr4 agonists can act as clinically useful vaccine adjuvants by selectively activating Ticam1-dependent immunostimulatory signalling events and only weakly activating potentially harmful Myd88-dependent inflammatory responses. |
| 191912 | Ticam1 | 25448706 | Myd88 and Ticam1 pathways differently regulate Tlr4-induced immune responses in B cells. |
| 191912 | Ticam1 | 25505250 | Adaptor proteins Ticam1 and Ticam2 have a novel function in Tlr2-mediated signal transduction. |
| 191912 | Ticam1 | 25548220 | Ticam1 but not Myd88 signalling is critical for the Trl4 protective adjuvant effect in neonates; where Ticam1(-/-) but not Myd88(-/-) neonates are highly susceptible to Escherichia coli peritonitis and bacteremia. |
| 191912 | Ticam1 | 25736436 | Wdfy1 is a crucial adaptor protein in the Tlr3/4 signalling pathway. Wdfy1 interacts with Tlr3 and Tlr4 and mediates the recruitment of Ticam1 to these receptors. |

| | | | |
|---|---|---|---|
| 191912 | Ticam1 | 25808990 | Intracellular Sef/IL-17R (SEFIR) domain of Il17rd targets TIR adaptor proteins Myd88, Tirap, Ticam1, Ticam2 and Traf6 to inhibit TLR downstream signalling. |
| 191912 | Ticam1 | 26651944 | Yersinia pseudotuberculosis type III secretion system effector, YopJ, suppresses Trif(Ticam1)-dependent responses during infection of primary phagocytic cells, including dendritic cells and macrophages. |
| 191912 | Ticam1 | 26351279 | Ticam1-dependent type I interferon signalling in T cells is essential to Th1 lineage differentiation and reactivation of memory T cells. Ticam1 activated memory T cells facilitate local neutrophil influx and enhance bacterial elimination. |
| 32341 | CAMP | 19625657 | CAMP represents a potent antimicrobial and cell-stimulating agent, most abundantly expressed in peripheral organs such as lung and skin during inflammation. |
| 32341 | CAMP | 19748465 | CAMP, a protein that has direct antimicrobial activity, serves as a mediator of vitamin D3-induced autophagy. |
| 32341 | CAMP | 19812202 | CAMP (LL-37) modulates IFN-gamma responses during both the innate and adaptive phases of immune responses, indicating an immunomodulatory role for this endogenous peptide. |
| 32341 | CAMP | 19797203 | CAMP has both antimicrobial and regenerative capabilities and promotes high glucose-attenuated epithelial wound healing via EGFR transactivation in organ cultured corneas. |
| 32341 | CAMP | 18923446 | CAMP is involved in various aspects of skin biology, including protection against infection, wound healing, and also in psoriasis where it suppresses apoptosis in keratinocytes. |
| 32341 | CAMP | 18397922 | CAMP induces endothelium-dependent relaxation in human omental veins, or vasodilation, mediated via an effect on endothelial ALX. |
| 32341 | CAMP | 20042575 | CAMP enhances delivery of CpG oligodeoxynucleotides to stimulate immune cells and this is independent of its amphipathic structure and its bactericidal property. |
| 32341 | CAMP | 20036634 | CAMP has dual function as an antimicrobial agent against bacterial target cells and a cell penetrating peptide that can deliver nucleic acids into the host cells. |
| 32341 | CAMP | 20163451 | CAMP decreases collagen expression at mRNA and protein levels in human dermal fibroblasts (HDFs) and this inhibition is dependent on phosphorylation of extracellular signal-regulated kinase (ERK). Vitamin C attenuates ERK signalling to inhibit the regulation of collagen production by CAMP in HDFs. |

| | | | |
|---|---|---|---|
| 32341 | CAMP | 20190140 | CAMP and human beta-defensins (hBDs) antimicrobial peptides induce the secretion of a pruritogenic cytokine IL-31 by human mast cells. |
| 32341 | CAMP | 20610648 | CAMP (LL37) directs macrophage differentiation toward macrophages with a pro-inflammatory signature and this requires internalization of the peptide, resulting in low production of IL-10 and profound production of IL-12p40 upon LPS stimulation. |
| 32341 | CAMP | 19703986 | CAMP (LL37) converts self-RNA into a trigger of TLR7 and TLR8 in human dendritic cells (DC), leading to production of TNF-alpha and IL6 and the differentiation of myeloid DCs into mature DCs. |
| 32341 | CAMP | 21393634 | CAMP (LL-37) attenuates lethal sepsis/endotoxin shock by suppressing the LPS-induced apoptosis of vascular and hepatic endothelial cells. LL-37 was found to inhibit the binding of LPS to the LPS receptors expressed on the cells. |
| 32341 | CAMP | 21389264 | CAMP (LL37) is found in high concentrations within neutrophil extracellular traps (NETs). CAMP is a neutrophil protein that facilitates the uptake and recognition of mammalian DNA by plasmacytoid dendritic cells, and may play a role in Systemic Lupus Erthermatosus autoimmunity. |
| 32341 | CAMP | 21441450 | CAMP (LL-37) dramatically reduced TNFA and nitric oxide levels produced by LPS and IFNG-polarized M1 macrophages, in addition LL-37-treated M1 macrophages were more efficient at suppressing tumour growth in vitro. This demonstrates the selective ability of LL-37 to decrease production of LPS-induced pro-inflammatory cytokines in macrophages, while leaving other crucial anti-inflammatory M1 and M2 macrophage functions unaltered. |
| 32341 | CAMP | 21448240 | CAMP (LL-37), at sufficiently low concentrations, is able to reduce fungal infectivity by inhibiting C. albicans adhesion to plastic surfaces, oral epidermoid cells, and the urinary bladders of female mice. The inhibitory effects of LL-37 on cell adhesion and aggregation were mediated by its preferential binding to mannan and chitin in the fungal cell wall. |
| 32341 | CAMP | 21464330 | CAMP (LL-37) translocates across the E. coli outer membrane and halts bacterial growth by interfering cell wall biogenesis. |
| 32341 | CAMP | 21562230 | CAMP (LL-37) confers protective immunity against psoriasis by neutralizing cytosolic DNA in keratinocytes and blocking the formation of AIM2 inflammasomes. |
| 32341 | CAMP | 21762664 | CAMP protects against colitis induction in mice. The increased expression of CAMP in monocytes involves the activation of TLR9/ERK signalling pathway by bacterial DNA. (Demonstrated in mouse) |

| | | | |
|---|---|---|---|
| 32341 | CAMP | 21832078 | CAMP expression is induced upon endoplasmic reticulum stress via NF-kB-C/EBP-alpha activation. |
| 32341 | CAMP | 22031815 | LL-37 (CAMP) reduces influenza A viral load and disease severity in mice. |
| 32341 | CAMP | 23328115 | CAMP (LL-37) is downregulated during septic shock. |
| 32341 | CAMP | 26113114 | (S)-methyl 2-(hexanamide)-3-(4-hydroxyphenyl) propanoate (MHP) activates SPHK1 to stimulate CAMP production and enhance epidermal antimicrobial defence. |
| 32341 | CAMP | 25884905 | Cleavage of CAMP by cathepsins CTSS and CTSK impairs its antimicrobial activity against Pseudomonas aeruginosa and Staphylococcus aureus. |
| 32341 | CAMP | 26878866 | Carbamylation of CAMP affects its bactericidal, cytotoxic and immunomodulatory function. |
| 32341 | CAMP | 26351280 | CAMP modulates the response of macrophages during mycobacterial infection controlling the expression of pro-inflammatory and anti-inflammatory cytokines. |
| 141522 | Casp2 | 26341399 | Nlrp3 and Casp2 are required for endoplasmic reticulum stress-induced inflammation. |
| 207066 | Mavs | 21454965 | The Mavs signalling pathway in non-myeloid cells is crucial for dsRNA-mediated natural killer cell activation. |
| 207066 | Mavs | 22844514 | Tyrosine phosphorylation of Mavs at amino acid residue Tyr9 is critical for the induction of Ifnb signalling. (Demonstrated in human) |
| 207066 | Mavs | 23499490 | Upon infection with encephalitic Bunyavirus, RIG-I/MAVS signalling activates SARM1 to mediate neuronal cell death. |
| 207066 | Mavs | 23754752 | MAVS binds to VDAC1 to trigger viral-induced apoptosis. |
| 207066 | Mavs | 24362933 | Plasmodium RNA is a pathogen-associated molecular pattern (PAMP) capable of activating a type I IFN response via the cytosolic pattern recognition receptors Ifih1 and Mavs, as well as via transcription factors Irf3 and Irf7. |
| 207066 | Mavs | 26079065 | Antiviral response to rotavirus in infected macrophages is fully Mavs-dependent. |
| 207066 | Mavs | 25897172 | Alveolar macrophages detect respiratory syncytial virus (RSV) via the Mavs /Ddx58 pathway and are a major source of type I interferons upon RSV infection. |
| 207066 | Mavs | 26633895 | Transgenic picornavirus RNA-dependent RNA polymerase (RdRP) expression in mice produces a quantitatively dramatic, sustained, effective antiviral interferon-stimulated genes (ISG) network, which requires the MDA5-MAVS pathway. |
| 207066 | Mavs | 26246171 | March5 modulates Mavs-mediated antiviral signalling, preventing excessive immune reactions. |
| 207066 | Mavs | 26348439 | Baiap2l1 recruits Ube2i to sumoylate Pcbp2, which causes its cytoplasmic translocation during viral infection and the sumoylated Pcbp2 associates with Mavs to initiate its degradation, leading to downregulation of antiviral responses. |

| | | | |
|---|---|---|---|
| 188762 | Pcbp2 | 22105485 | Pcbp2 synergizes with Pcbp1 in Mavs inhibition but Pcbp2 shows low basal expression with rapid induction after infection while Pcbp1 is stably and abundantly expressed. (Demonstrated in human) |
| 188762 | Pcbp2 | 26348439 | Baiap2l1 recruits Ube2i to sumoylate Pcbp2, which causes its cytoplasmic translocation during viral infection and the sumoylated Pcbp2 associates with Mavs to initiate its degradation, leading to downregulation of antiviral responses. |
| 151396 | Ube2i | 26348439 | Baiap2l1 recruits Ube2i to sumoylate Pcbp2, which causes its cytoplasmic translocation during viral infection and the sumoylated Pcbp2 associates with Mavs to initiate its degradation, leading to downregulation of antiviral responses. |
| 208134 | Baiap2l1 | 26348439 | Baiap2l1 recruits Ube2i to sumoylate Pcbp2, which causes its cytoplasmic translocation during viral infection and the sumoylated Pcbp2 associates with Mavs to initiate its degradation, leading to downregulation of antiviral responses. |
| 69618 | CASP1 | 15039421 | CASP1 activates NF-kappaB independent of its enzymatic activity and contributes to inflammation by proteolysis of pro-IL1B (IL-1 beta) and RIPK2 activation of NF-kappaB and MAPK1. |
| 69618 | CASP1 | 19124602 | CASP1 is part of the inflammasome complex, along with pathogen-specific nucleotide oligomerization and binding domain (NOD)-like receptors (NLRs) and in some cases the scaffolding protein ASC. Formation of the membrane-associated inflammasome complex in murine macrophages, results in cleavage of cytosolic CASP1 substrates and cell death. |
| 69618 | CASP1 | 20823203 | CASP1 activity is required for discrimination between translocon-positive and -negative bacteria in bone-marrow derived cells and interleukin-1 receptor signalling. Activation of CASP1 by bacteria expressing Type 3 secretion systems allows for rapid recognition of bacteria expressing conserved functions associated with virulence. |
| 69618 | CASP1 | 21057511 | CASP1 clears intracellular bacteria in vivo independently of IL1B (IL-1-beta) and IL18 and establishes pyroptosis as an efficient mechanism of bacterial clearance by the innate immune system. CASP1-induced pyroptotic cell death releases bacteria from macrophages and exposes the bacteria to uptake and killing by reactive oxygen species in neutrophils. |
| 69618 | CASP1 | 21439959 | CASP1 is a component of the inflammasome and is required for inflammation in acute pancreatitis. (Demonstrated in murine model) |
| 69618 | CASP1 | 21602824 | CASP1-dependent inflammatory cell death, or pyroptosis, is only induced by viable, but not heat-killed, E. coli. (Demonstrated in murine model) |

| | | | |
|---|---|---|---|
| 69618 | CASP1 | 22833538 | Naturally occurring variants of CASP1 differ considerably in structure and the ability to activate IL1B. |
| 69618 | CASP1 | 24481253 | The precursor and mature forms of IL37 are secreted from activated cells upon inflammasome activation and CASP1 processing of IL37 is important for its anti-inflammatory activity. |
| 69618 | CASP1 | 26324708 | 20-kDa IL1B generated from CASP1 cleaved pro-IL1B limits the available pro-IL1B for generation of CASP1 cleaved 17-kDa IL1B, thus reducing inflammation. |
| 66519 | IL1B | 20195505 | IL1B is an important proinflammatory cytokine that activates monocytes, macropages, and neutrophils. IL1B processing during infection is a complex process in which the inflammasomes are only one of several activation mechanisms. |
| 66519 | IL1B | 20401526 | Mature IL1B production requires, in addition to the synthesis of pro-IL1B, cleavage of the precursor protein by the inflammatory CASP1 (Caspase-1) which is controlled within the NLRP3 inflammasome. |
| 66519 | IL1B | 20620944 | IL1B-producing conventional dendritic cells preserves and expands IL-22(+)AHR(+) immature human natural killer cells in the secondary lymphoid tissue. |
| 66519 | IL1B | 21170027 | IL1B acts as a growth factor for neutrophil progenitors and as a survival factor for mature neutrophils. In the absence of IKBKB, the IL1B production is enhanced and provides a compensatory mechanism for maintaining antibacterial defense when NFKB is inhibited. (Demonstrated in murine model) |
| 66519 | IL1B | 21228274 | IL1B secretion in macrophages is regulated by autophagy by two mechanisms; sequestering of pro-IL1B in autophagosome during TLR stimulation, and processing,secretion of IL1B in a NLRP3- and TRIF-dependent manner. |
| 66519 | IL1B | 21602824 | IL1B secretion is induced only during viable E. coli infection (as oppose to heat-killed E. coli or LPS); Viable bacteria specifically elicit cleavage of pro-IL1B. (Demonstrated in murine model) |
| 66519 | IL1B | 21270399 | IL1B derived from alveolar macrophages is the critical mediator which induces chemokine production in non-hematopoietic cells in the lung, resulting in swift and robust recruitment of infection-controlling neutrophils into the airways. (Demonstrated in murine model) |

| | | | |
|---|---|---|---|
| 66519 | IL1B | 21628463 | IL1B secretion is tightly regulated by the redox status in myeloid cells. TLR engagement in monocytes induces ROS generation followed by a sustained antioxidant response and efficient IL1B secretion. In macrophages, the antioxidant systems are in an upregulated state, and therefore buffers the TLR induction of the redox response, which results in low IL1B processing and secretion. |
| 66519 | IL1B | 22158745 | IL1B is an important component of the cellular network involving macrophages and epithelial cells, which facilitates IL8 chemokine expression and aids neutrophil recruitment during pneumococcal pneumonia. |
| 66519 | IL1B | 22426547 | IL1B is an inflammatory cytokine that binds to its primary receptor, IL1R1, that then recruits the accessory protein IL1RAP to form a signalling-competent heterotrimeric complex. |
| 66519 | IL1B | 24277153 | TLR8 plays a pathogenic role in disease whereby its expression is increased in patients with systemic arthritis and is correlated with the elevation of IL1B levels and disease status. |
| 66519 | IL1B | 24323452 | Protein-bound polysaccharide-K can activate the NLRP3 inflammasome and induce IL1B in a TLR2- and NLRP3-dependent manner. |
| 66519 | IL1B | 25463072 | Interleukin-1 (IL1A/IL1B) plays a key role in the interaction between local vessel wall cells and invading monocytes to multiply cholesterol-triggered inflammation in the vessel wall. |
| 66519 | IL1B | 25474109 | IFNG interferes with the IL-1/NFKBIZ axis in Î²-glucan-activated dendritic cells and promotes T cell-mediated immune responses with increased release of IFNG and IL22, and diminished production of IL17A. |
| 66519 | IL1B | 25964352 | CASP4 is a critical regulator of noncanonical inflammasome activation that initiates defence against bacterial pathogens in primary macrophages by mediating cell death and IL1A release |
| 66519 | IL1B | 25637949 | DEFB103A and RNASE7 are induced in human umbilical endothelial cells (HUVECs) by classical inflammatory cytokines such as: IFNG, IL1B and TNF. |
| 66519 | IL1B | 26032420 | Antibody-dependent enhancement (ADE) of Dengue virus serotype 2 (DENV-2) elevates mature IL1B secretion via SYK signalling pathway in primary monocytes. |
| 66519 | IL1B | 26187413 | Differentiation of Type 3 innate lymphoid cells (ILC3) to IL7R(+) ILC1 is reversible whereas IL7R(+) ILC1 can differentiate to ILC3 in the presence of IL2, IL23A, and IL1B dependent on the transcription factor RORC, and this process is enhanced in the presence of retinoic acid. |
| 66519 | IL1B | 26324708 | 20-kDa IL1B generated from CASP1 cleaved pro-IL1B limits the available pro-IL1B for generation of CASP1 cleaved 17-kDa IL1B, thus reducing inflammation. |

| | | | |
|---|---|---|---|
| 82738 | TLR4 | 18326860 | TLR4 is activated by LPS and this recognition activates the Src family kinases, Src, Fyn and Yes, which in turn contribute to tyrosine phosphorylation of Zonula adherens proteins to open the endothelial paracellular pathway. |
| 82738 | TLR4 | 15852007 | TLR4 binding to microbial ligands can be inhibited by CD180 and its helper molecule, LY86, via direct interactions with the TLR4 signalling complex. |
| 82738 | TLR4 | 10196138 | TLR4 is involved in lipopolysaccharide (LPS) signaling and serves as a cell-surface co-receptor for CD14, leading to LPS-mediated NF-kappaB activation and subsequent cellular events. |
| 82738 | TLR4 | 20037584 | TLR4-TLR6-Cd36 activation is a common molecular mechanism by which atherogenic lipids and amyloid-beta stimulate sterile inflammation. |
| 82738 | TLR4 | 20133493 | TLR4 dimerize and enable rapid signal transduction against LPS stimulation on membrane-associated CD14-expressing cells. |
| 82738 | TLR4 | 20360853 | TLR4 and TLR9 have both non-redundant and cooperative roles in lung innate responses during Gram-negative bacterial pneumonia and are both critical for IL-17 driven antibacterial host response. |
| 82738 | TLR4 | 20826541 | TLR4 mediates LPS-induced muscle catabolism via coordinate activation of the ubiquitin-proteasome and the autophagy-lysosomal pathways. TLR4 activation by LPS induces C2C12 myotube atrophy via up-regulating autophagosome formation and the expression of ubiquitin ligase atrogin-1/MAFbx and MuRF1. |
| 82738 | TLR4 | 21442393 | TLR4 transfection of eukaryotic host cells using bacterial vectors, or bactofection, was shown to reduce E. coli colonization in the kidney and the bladder in an animal model of urinary tract infection. (Demonstrated in murine model) |
| 82738 | TLR4 | 21464300 | TLR4 is involved in the transmission of ER stress from tumour cells to macrophages, promoting a pro-inflammatory program in the tumour microenvironment, thus facilitating tumour progression. (Demonstrated in murine model) |
| 82738 | TLR4 | 21518783 | TLR4 deficient murine macrophages results in the complete abrogation of TNF-alpha production during Leishmania panamensis infection. The endosomal TLR4 plays a crucial role in the activation of host macrophages and controlling the early stages of parasitic infection. (Demonstrated in murine model) |
| 82738 | TLR4 | 21615666 | Epithelial TLR4 activation facilitates the transcytosis of non-cytolytic uropathogenic E. coli across intact collecting duct cell layers to invade the renal interstitium in experimental urinary tract infections. |

| | | | |
|---|---|---|---|
| 82738 | TLR4 | 21712422 | TLR4:LY96 functions as intracellular LPS sensor and triggers a unique set of LPS responses upon recognition of phagocytosed bacteria in macrophages. (Demonstrated in murine model) |
| 82738 | TLR4 | 21730052 | TLR4 on dendritic cell surfaces binds to HSPA14 and induces a robust Th1 response via the MAPK and NFkB signalling pathways. (Demonstrated in mouse) |
| 82738 | TLR4 | 21738466 | TLR4 recognizes Clostridium difficile surface layer proteins and induces the maturation of dendritic cells to activate the innate and adaptive immune response. (Demonstrated in mouse) |
| 82738 | TLR4 | 21775438 | TLR4 and HSPD1 mediate myocardial ischemia-activated innate immune signalling, which plays an important role in mediating apoptosis and inflammation during ischemia/reperfusion (I/R). (Demonstrated in murine model) |
| 82738 | TLR4 | 22096480 | TLR4 and TLR2 are crucial for in vivo recognition of Chlamydia pneumoniae. Tlr4/2 double-deficient mice were unable to control pneumonia caused by C. pneumoniae. (Demonstrated in mice) |
| 82738 | TLR4 | 22354030 | TLR4 translocates to membrane lipid rafts in a ceramide-dependent manner in Helicobacter pylori infected gastric epithelial cells. |
| 82738 | TLR4 | 22396536 | TLR4 is involved in cell-cell contact signalling between activated apoptotic lymphocytes and dendritic cells (DC) during the maturation of DCs. |
| 82738 | TLR4 | 22433865 | Synthetic triacylated lipid A-molecules have the potent ability to selectively antagonize TLR4 and inhibit anti-bacterial immunity. |
| 82738 | TLR4 | 22593572 | The poxviral protein A46 directly inhibits TLR4 signalling by disrupting receptor complex formation. |
| 82738 | TLR4 | 22962435 | A human TLR4 polymorphism (D299G/T399I) impairs TLR4::LY96 dimerization and results in a dampened host response to bacterial lipids. |
| 82738 | TLR4 | 22951730 | TLR4 is an important regulator of wound inflammation and is essential for early skin wound healing. (Demonstrated in mice) |
| 82738 | TLR4 | 24265315 | TIR domain-contaning protein from Brucella melitensis, TcpB, disrupts the receptor-adaptor interaction between TLR4 and TIRAP. |
| 82738 | TLR4 | 25371197 | ECSIT binds to MAP3K7 and TRAF6 to form a complex that plays a pivotal role in activating TLR4-mediated NF-kB signalling. |
| 82738 | TLR4 | 25505274 | The TLR4/S100A8 axis is important in the activation of monocytes. |
| 82738 | TLR4 | 26082489 | Endotoxin tolerance re-programs TLR4 signalling via suppression of PELI1, a positive regulator of MyD88- and TIR domain-containing adapter inducing IFN-Î² (TRIF)-dependent signalling that promotes K63-linked polyubiquitination of IRAK1, TBK1, and TAK1. |

| | | | |
|---|---|---|---|
| 82738 | TLR4 | 26610398 | H. pylori infection induces the expression and activation of components of NLRP3 inflammasomes in neutrophils and this activation is independent of a functional type IV secretion system, TLR2 and TLR4. |
| 82738 | TLR4 | 26310831 | PELI3 is involved in endotoxin tolerance and functions as a negative regulator of TLR2/4 signalling. |
| 41789 | TLR2 | 20505832 | TLR2 plays a critical role in the ability of innate immunity to determine M. pulmonis numbers in the lung, and early after respiratory infection TLR2 recognition of M. pulmonis triggers initial cytokine responses of host cells. |
| 41789 | TLR2 | 20927103 | TLR2 functions as a sensor of oxidation-associated molecular patterns, providing a key link connecting inflammation, oxidative stress, innate immunity and angiogenesis. |
| 41789 | TLR2 | 21439957 | TLR1 :: TLR2 dimeric pairs recognize malarial glycosylphosphatidylinositols (GPI) to initiates intracellular signalling and the production of pro-inflammatory cytokines. |
| 41789 | TLR2 | 21454596 | TLR2 recognizes Thermus aquaticus extracellular polysacchride, YT-1, and induces the production of cytokines TNF and IL6 in peritoneal macrophages. (Demonstrated in murine model) |
| 41789 | TLR2 | 21482737 | TLR2::TLR6 synergistically interacts with TLR9 in lung epithelium to induce rapid pathogen killing, and can be used as a therapeutic target to treat otherwise lethal pneumonia. |
| 41789 | TLR2 | 21512004 | TLR2 is activated by gut commensal microbe, Bacteroides fragilis, to establish host-microbial symbiosis by promoting immunological tolerance. (Demonstrated in murine model) |
| 41789 | TLR2 | 21566133 | TLR2 and TNFSF11 signalling pathways are modulated by Porphromonas gingivalis to alter the differentiation states of osteoclasts resulting in bacteria-mediated bone loss. (Demonstrated in murine model) |
| 41789 | TLR2 | 21602496 | TLR2 is expressed by Muller cells, principal glia of retina, and is responsible for generating robust bactericidal activity against Staphylococcus aureus and contributing to retinal innate defence. |
| 41789 | TLR2 | 21698237 | TLR2 is required for rapid inflammasome activation in response to infection by cytosolic bacterial pathogens such as Francisella novicida. (Demonstrated in murine model) |

| | | | |
|---|---|---|---|
| 41789 | TLR2 | 21862586 | TLR2-driven integration of inducible nitric oxide synthase (iNOS), Wnt-beta-Catenin and NOTCH1 signalling contributes to its capacity to regulate a battery of genes associated with T regulatory cell lineage commitment and towards modulation of defined set of effector functions in macrophages. (Demonstrated in murine model) |
| 41789 | TLR2 | 21873606 | TLR2 directly recognizes glycogen, resulting in the activation of immunocytes such as macrophages to enhance the production of nitric oxide and inflammatory cytokines. |
| 41789 | TLR2 | 22096480 | TLR2 and TLR4 are crucial for in vivo recognition of Chlamydia pneumoniae. Tlr2/4 double-deficient mice were unable to control pneumonia caused by C. pneumoniae. (Demonstrated in mice) |
| 41789 | TLR2 | 22102818 | TLR2 signalling promotes protective vaccine-enhancing Th17 cell responses when cells are stimulated with early secreted antigenic target protein 6 (ESAT-6) expressed by the virulent Mycobacterium tuberculosis strain H37Rv but not by tuberculosis vaccine Bacillus Calmette-Gu Ã © rin (BCG). (Demonstrated in mice) |
| 41789 | TLR2 | 22174456 | TLR2 recognizes Mycobacterium tuberculosis H37Rv cell surface lipoprotein MPT83, which induces the production of TNF, IL6, and IL12B cytokines by macrophages and upregulates macrophage function. (Demonstrated in mouse) |
| 41789 | TLR2 | 22216191 | Mycobacterium abscessus glycopeptidolipid (GPL) prevents TLR2-mediate induction of IL8 and DEFB4A in respiratory epithelial cells. |
| 41789 | TLR2 | 25353353 | Interaction of filamentous hemagglutinin (FHA) with TLR2 induces an innate immune response against Bordetella pertussis and the TLR2-binding domain of FHA may contribute to immunoprotection against pertussis infection. |
| 41789 | TLR2 | 25456159 | Cutaneous bacteria can negatively regulate skin-driven immune responses by inducing Gr1(+)CD11b(+) myeloid-derived suppressor cells via TLR2-6 activation. |
| 41789 | TLR2 | 25531754 | Soluble TLR2 (sTLR2) generated by metalloproteinase activation inhibits TLR2-induced cytokine production in THP-1 cell line. |
| 41789 | TLR2 | 25977263 | TLR10 is a functional receptor involved in the innate immune response to H. pylori infection and the TLR2/TLR10 heterodimer functions in H. pylori lipopolysaccharide recognition. |
| 41789 | TLR2 | 25955717 | Human Cytomegalovirus (HCMV) miR-UL112-3p efficiently targets TLR2 during HCMV infection, resulting in the inhibition of TLR2-mediated NF Î º B signalling. |

| | | | |
|---|---|---|---|
| 41789 | TLR2 | 26610398 | H. pylori infection induces the expression and activation of components of NLRP3 inflammasomes in neutrophils and this activation is independent of a functional type IV secretion system, TLR2 and TLR4. |
| 41789 | TLR2 | 26283364 | Staphylococcal superantigen-like protein 3 (SSL3) interferes with TLR2 activation at two stages. First by binding to TLR2 and blocking ligand binding and second by interacting with an already formed TLR2-lipopeptide complex, thus preventing TLR heterodimerization and downstream signalling. |
| 41789 | TLR2 | 26310831 | PELI3 is involved in endotoxin tolerance and functions as a negative regulator of TLR2/4 signalling. |
| 59060 | PELI3 | 17997719 | PELI3 and other pellino isoforms are the E3 ubiquitin ligases that mediate the IL-1-stimulated formation of K63-pUb-IRAK1 in cells, which may contribute to the activation of IKBKB and NF-kappaB, as well as other signalling pathways dependent on IRAK1 and IRAK4. |
| 59060 | PELI3 | 25483963 | Autophagy causes PELI3 degradation during Tlr4-signalling, subsequently inhibiting Il1b expression and impairing the hyperinflammatory phase during sepsis. |
| 59060 | PELI3 | 26310831 | PELI3 is involved in endotoxin tolerance and functions as a negative regulator of TLR2/4 signalling. |
| 156729 | Tlr4 | 19923461 | Signaling crosstalk during sequential Tlr4 and Tlr9 activation amplifies the inflammatory response of mouse macrophages. |
| 156729 | Tlr4 | 20385881 | Tlr4 and Tlr2 activate murine macrophages and this activation is negatively regulated by a Lyn/PI3K module and promoted by SHIP1. |
| 156729 | Tlr4 | 21442393 | Tlr4 transfection of eukaryotic host cells using bacterial vectors, or bactofection, was shown to reduce E. coli colonization in the kidney and the bladder in an animal model of urinary tract infection. |
| 156729 | Tlr4 | 21464300 | Tlr4 is involved in the transmission of ER stress from tumour cells to macrophages, promoting a pro-inflammatory program in the tumour microenvironment, thus facilitating tumour progression. |
| 156729 | Tlr4 | 21518783 | Tlr4 deficient murine macrophages results in the complete abrogation of TNF-alpha production during Leishmania panamensis infection. The endosomal Tlr4 plays a cruical role in the activation of host macrophages and controlling the early stages of parasitic infection. |
| 156729 | Tlr4 | 21615666 | Epithelial Tlr4 activation facilitates the transcytosis of non-cytolytic uropathogenic E. coli across intact collecting duct cell layers to invade the renal interstitium in experimental urinary tract infections. |

| | | | |
|---|---|---|---|
| 156729 | Tlr4 | 21712422 | Tlr4:Ly96 functions as intracellular LPS sensor and triggers a unique set of LPS responses upon recognition of phagocytosed bacteria in macrophages. |
| 156729 | Tlr4 | 21730052 | Tlr4 on dendritic cell surfaces binds to Hspa14 and induces a robust Th1 response via the MAPK and NFkB signalling pathways. |
| 156729 | Tlr4 | 21738466 | Tlr4 recognizes Clostridium difficile surface layer proteins and induces the maturation of dendritic cells to activate the innate and adaptive immune response. |
| 156729 | Tlr4 | 21775438 | Tlr4 and Hspd1 mediate myocardial ischemia-activated innate immune signalling, which plays an important role in mediating apoptosis and inflammation during ischemia/reperfusion (I/R). |
| 156729 | Tlr4 | 22096480 | Tlr4 and Tlr2 are crucial for in vivo recognition of Chlamydia pneumoniae. Tlr4/2 double-deficient mice were unable to control pneumonia caused by C. pneumoniae. |
| 156729 | Tlr4 | 22354030 | Tlr4 translocates to membrane lipid rafts in a ceramide-dependent manner in Helicobacter pylori infected gastric epithelial cells. (Demonstrated in human) |
| 156729 | Tlr4 | 22396536 | Tlr4 is involved in cell-cell contact signalling between activated apoptotic lymphocytes and dendritic cells (DC) during the maturation of DCs. (Demonstrated in human) |
| 156729 | Tlr4 | 22433865 | Synthetic triacylated lipid A-molecules have the potent ability to selectively antagonize Tlr4 and inhibit anti-bacterial immunity. (Demonstrated in human) |
| 156729 | Tlr4 | 22593572 | The poxviral protein A46 directly inhibits Tlr4 signalling by disrupting receptor complex formation. (Demonstrated in human) |
| 156729 | Tlr4 | 22962435 | A human TLR4 polymorphism (D299G/T399I) impairs TLR4::LY96 dimerization and results in a dampened host response to bacterial lipids. (Demonstrated in human) |
| 156729 | Tlr4 | 22951730 | Tlr4 is an important regulator of wound inflammation and is essential for early skin wound healing. |
| 156729 | Tlr4 | 24101501 | Milk oligosaccharide sialyl( Î ± 2,3)lactose modulates mucosal immunity by inducing inflammation through TLR4 signaling |
| 156729 | Tlr4 | 24423728 | Itgam (Cd11b) fine tunes the balance between adaptive and innate immune responses initiated by LPS by modulating the trafficking and signalling functions of Tlr4 in a cell-type-specific manner. |
| 156729 | Tlr4 | 24163408 | Nod1 and Nod2 synergize with Tlr4 in dendritic cells to increase IL12 production and enhance invariant natural killer T (iNKT) cell activation, and are important regulators of the IFN gamma response by iNKT cells during S. typhimurium and L. monocytogenes infections. |
| 156729 | Tlr4 | 25022365 | Rab8a interacts with Pik3cg to regulate Akt signalling generated by surface Tlr4. |

| | | | |
|---|---|---|---|
| 156729 | Tlr4 | 25389373 | High-potency Tlr4 agonists can act as clinically useful vaccine adjuvants by selectively activating Ticam1-dependent immunostimulatory signalling events and only weakly activating potentially harmful Myd88-dependent inflammatory responses. |
| 156729 | Tlr4 | 25448706 | Myd88 and Ticam1 pathways differently regulate Tlr4-induced immune responses in B cells. |
| 156729 | Tlr4 | 25483963 | Autophagy causes PELI3 degradation during Tlr4-signalling, subsequently inhibiting Il1b expression and impairing the hyperinflammatory phase during sepsis. |
| 156729 | Tlr4 | 25549946 | Tmem126a upregulates genes involved in antigen presentation; such as Icam1, MHC II, Cd86 and Cd40, via the Tlr4 signal transduction pathway. |
| 156729 | Tlr4 | 25548220 | Ticam1 but not Myd88 signalling is critical for the Trl4 protective adjuvant effect in neonates; where Ticam1(-/-) but not Myd88(-/-) neonates are highly susceptible to Escherichia coli peritonitis and bacteremia. |
| 156729 | Tlr4 | 25736436 | Wdfy1 is a crucial adaptor protein in the Tlr3/4 signalling pathway. Wdfy1 interacts with Tlr3 and Tlr4 and mediates the recruitment of Ticam1 to these receptors. |
| 156729 | Tlr4 | 25801433 | Lipopolysaccharide-mediated myeloid Anpep (CD13) expression governs internalization of Tlr4 and negatively regulates Tlr4 signalling, thereby balancing the innate response by maintaining the inflammatory equilibrium critical to innate immune regulation. |
| 156729 | Tlr4 | 26081153 | Psen2 deficiency is paralleled by reduced transcription of Tlr4 mRNA and loss of LPS-induced Tlr4 mRNA transcription regulation. |
| 156729 | Tlr4 | 26310831 | Peli3 is involved in endotoxin tolerance and functions as a negative regulator of Tlr2/4 signalling. |
| 130436 | Peli3 | 26310831 | Peli3 is involved in endotoxin tolerance and functions as a negative regulator of Tlr2/4 signalling. |
| 189070 | Atf7 | 26322480 | Atf7, a stress-response transcription factor, mediates epigenetic changes in macrophages involved in innate immunological memory in response to lipopolysaccharide. |
| 127311 | MIR223 | 22984082 | MIR223 negatively regulates NLRP3 inflammasome activity. |
| 127311 | MIR223 | 22984081 | MIR223 regulates the NLRP3 inflammasome and IL-1Î² production. |
| 127311 | MIR223 | 26296289 | MIR223 regulates macrophage function by modulating cytokine production and NF-Îº B activation through inhibition of RELA phosphorylation and nuclear translocation. |
| 285 | COLEC12 | 26290605 | Soluble COLEC12 can recognize Aspergillus fumigatus leading to activation of the alternative pathway of complement. |

| | | | |
|---|---|---|---|
| 47294 | TLR3 | 19074283 | TLR3-type II IFN signalling cooperates with the RIG-I/MDA5-type I IFN axes for efficient innate antiviral immune responses. |
| 47294 | TLR3 | 19865078 | TLR3-dependent antiviral pathway is negatively regulated by activated F2RL1 leading to blunted expression of TLR3/IRF3 driven genes, as well as activation of IRF3 and STAT1. |
| 47294 | TLR3 | 19865078 | TLR3, TLR2, or TLR4 cooperate with proteinase-activated receptors (PARs) for activation of nuclear factor-kappaB-dependent signalling in mucosal epithelial cell lines. |
| 47294 | TLR3 | 20019748 | TLR3-induced proapoptotic signalling involves TICAM1 (TRIF)-dependent activation of CASP8 and is under the control of inhibitor of apoptosis proteins (IAPs) in melanoma cells. |
| 47294 | TLR3 | 11607032 | TLR3 is activated by poly(I:C) and this induces cytokine production through a signalling pathway dependent on MyD88. |
| 47294 | TLR3 | 14982987 | TLR3-mediated activation of NF-kappaB and IRF3 diverges at Toll-IL-1 receptor domain-containing adapter 1 (TICAM1) inducing IFN-beta. |
| 47294 | TLR3 | 20861016 | TLR3 dimerizes when it binds dsRNA and this is essential for ligand binding. Although the three TLR3 contact sites individually interact weakly with their binding partners, together they form a high affinity ligand-receptor complex. |
| 47294 | TLR3 | 21220319 | TLR3 deletion dramatically enhanced the development of elastic lamina damage after collar-induced injury, indicating that TLR3 signalling plays a protective role in arterial vessel wall. |
| 47294 | TLR3 | 21367858 | TLR3 activation by Poly(I:C) in the endothelial cells induces Poly(I:C) dose- and time-dependent cell apoptosis. Specifically, TLR3 stimulation triggered the signalling of both extrinsic and intrinsic apoptotic pathways. |
| 47294 | TLR3 | 21402738 | TLR3 requires proteolytic processing in endolysosome by asparagine endopeptidase and cathepsin in the endolysosome to initiate signalling in response to DNA. (Demonstrated in murine model) |
| 47294 | TLR3 | 21498625 | TLR3 expression is inducible by LPS via TLR4-MYD88-IRAK-TRAF6-NFKB dependent signalling pathway. |
| 47294 | TLR3 | 21695051 | TLR3 is necessary to establish an antiviral state in hepatocytes infected with hepatitis C Virus. HCV envelope proteins counteract the antiviral host defence by inhibiting the expression of TLR3. |
| 47294 | TLR3 | 22016778 | TLR3 signalling is enhanced by the presence of viral double-strand RNA-binding proteins. |
| 47294 | TLR3 | 22072781 | TLR3-TICAM1-mediated signalling pathway plays an essential role in the anti-viral response against poliovirus infection. (Demonstrated in mice) |

| | | | |
|---|---|---|---|
| 47294 | TLR3 | 22262694 | TLR3 is constitutively expressed in spermatogonia and spermatocytes, and has the ability to activate anti-viral responses. (Demonstrated in mice) |
| 47294 | TLR3 | 22421964 | Upon engagement with its ligand, dsRNA, TLR3 possesses the ability to recruit CASP8 and RIPK1 to induce apoptosis. |
| 47294 | TLR3 | 22754655 | Activation of TLR3 with poly(I:C) mediates antiviral immunity that diminishes coronavirus production in macrophages. (Demonstrated in mice) |
| 47294 | TLR3 | 22570612 | Upregulation of TLR3 in intestinal epithelia during infancy may contribute to age-dependent susceptibility to rotavirus infection. (Demonstrated in mice) |
| 47294 | TLR3 | 22986631 | TLR3 activation differentially regulates phagocytosis of bacteria and apoptotic neutrophils by peritoneal macrophages. (Demonstrated in mice) |
| 47294 | TLR3 | 23035017 | TLR3 is more efficiently activated by high molecular mass than by low molecular weight poly(I:C). |
| 47294 | TLR3 | 25452586 | TLR3-mediated antibody response to Chikungunya virus plays a key role in its infection, replication and pathology. |
| 47294 | TLR3 | 25641411 | Intracellular/endocytic TLR3 interacts with SCARF 1 in the presence of Poly I:C to boost TLR3-mediated inflammatory signalling and stimulate cytokine production in macrophages. |
| 47294 | TLR3 | 25880109 | Signalling through both DDX58 and TLR3 is important for interferon induction by influenza A virus in alveolar epithelial cells. |
| 47294 | TLR3 | 26296370 | Bluetongue virus activates TLR3/interferons signalling pathway resulting in the inhibition of human immunodeficiency virus in macrophages. |
| 17212 | CLEC4E | 21267996 | CLEC4E, a C-type lectin receptor, is a pattern recognition receptor critical for immune responses to fungi. CLEC4E is coupled to SYK kinase and signals via CARD9 to activate NFKB, which in turns induces both innate and adaptive immunity. |
| 17212 | CLEC4E | 24733387 | Glycerol monomycolate is a unique ligand for CLEC4E (Mincle). |
| 17212 | CLEC4E | 26296894 | Cholesterol crystals are an endogenous ligand for CLEC4E and their binding activates innate immune responses. |
| 193222 | Traf6 | 21185369 | Traf6 is a E3 ubiquitin ligase that activates NFKB pathway in response to innate and adaptive immunity stimuli. Traf6 protein contains a highly conserved TRAF-C domain that contributes to oligomerization and its interaction to upstream signalling molecules, and a RING domain dimerization interface that is functionally important for ubiquitination and the activation of NFKB. |

| | | | |
|---|---|---|---|
| 193222 | Traf6 | 21220427 | Traf6 is polyubiquitinated and disassembled during endotoxin tolerization; a process which impairs the production of LPS-induced pro-inflammatory cytokines without inhibition expression of anti-inflammatory or anti-microbial mediators. |
| 193222 | Traf6 | 22033459 | Traf6 is degraded in the proteasome upon TLR stimulation in macrophages. |
| 193222 | Traf6 | 23951545 | The binding of MAVS to Traf2, Traf5, and Traf6 is dependent on virus infection and MAVS polymerization. The TRAF proteins promote ubiquitination that recruits IKBKG binding to the MAVS signalling complex. |
| 193222 | Traf6 | 26048146 | Mir146 attenuates sepsis-induced cardiac dysfunction by preventing NF Î º B activation, inflammatory cell infiltration, and inflammatory cytokine production via targeting of Irak1 and Traf6 in both cardiomyocytes and inflammatory monocytic cells |
| 193222 | Traf6 | 25808990 | Intracellular Sef/IL-17R (SEFIR) domain of Il17rd targets TIR adaptor proteins Myd88, Tirap, Ticam1, Ticam2 and Traf6 to inhibit TLR downstream signalling. |
| 193222 | Traf6 | 26503954 | Trim12c interacts with Traf6, a key protein in pathogen recognition receptor signalling, and reciprocally enhances its ubiquitination, leading to cooperative activation of IFN and NF-kB pathways. |
| 193222 | Traf6 | 26305951 | Usp25 physically associates with Traf3 and Traf6 after infection by RNA or DNA viruses and promotes innate antiviral responses by protecting virus-induced proteasome-dependent or independent degradation of Traf3 and Traf6. |
| 172558 | Traf3 | 16306937 | Traf3 is a component of Toll/interleukin-1 receptor (TIR) signalling complexes that is recruited along with Traf6. Traf3 is essential for the induction of type I interferons (IFN) and the anti-inflammatory cytokine interleukin 10, but is dispensable for expression of pro-inflammatory cytokines. |
| 172558 | Traf3 | 21660053 | Traf3 is a highly versatile regulator that positively controls type I interferon production, and negatively regulates MAP kinase activation and alternative NFkB signalling. |
| 172558 | Traf3 | 22792062 | Upon sensing dsRNA or dsDNA, Traf3 interacts with ER-to-Golgi transport proteins to induce Mavs-associated innate immune responses. (Demonstrated in human) |
| 172558 | Traf3 | 25422508 | Traf3 expressed in myeloid cells regulates immune responses in myeloid cells and acts to inhibit inflammation and tumor development. |

| | | | |
|---|---|---|---|
| 172558 | Traf3 | 26305951 | Usp25 physically associates with Traf3 and Traf6 after infection by RNA or DNA viruses and promotes innate antiviral responses by protecting virus-induced proteasome-dependent or independent degradation of Traf3 and Traf6. |
| 171582 | Usp25 | 26305951 | Usp25 physically associates with Traf3 and Traf6 after infection by RNA or DNA viruses and promotes innate antiviral responses by protecting virus-induced proteasome-dependent or independent degradation of Traf3 and Traf6. |
| 172251 | Irf1 | 21097874 | Irf1 transcriptionally inhibits the Il23a through the ISRE element and reduce the severity of chronic intestinal inflammation caused by LPS. |
| 172251 | Irf1 | 22266972 | Irf1 promotes immune cell apoptosis and inhibits autophagy in a murine endotoxemia model. |
| 172251 | Irf1 | 24675692 | Irf1 is an essential regulator of the host innate antiviral response in the brain by limiting viral replication at later stages of infection, but is not involved in the rapid induction of IFN. |
| 172251 | Irf1 | 26305961 | Nlrp3 deficiency protects mice from the development of type 1 diabetes by suppressing Th1 responses and impairing T-cell migration to pancreatic islets through the down-regulation of chemokine expression (Ccl5, Cxcl10, Irf1) in islets. |
| 179916 | Cxcl10 | 21124994 | Cxcl10 exert direct antimicrobial effects in vitro against Bacillus anthracis spore and bacilli in a receptor-independent manner and contributes to pulmonary innate immunity. |
| 179916 | Cxcl10 | 21518789 | Cxcl10 concentration in blood increases during neonatal polymicrobial sepsis, and the blockade of Cxcl10 not only worsens recruitment and phagocytic function of macrophages, but also the survival of neonatal mice. |
| 179916 | Cxcl10 | 26305961 | Nlrp3 deficiency protects mice from the development of type 1 diabetes by suppressing Th1 responses and impairing T-cell migration to pancreatic islets through the down-regulation of chemokine expression (Ccl5, Cxcl10, Irf1) in islets. |
| 205509 | Ccl5 | 26305961 | Nlrp3 deficiency protects mice from the development of type 1 diabetes by suppressing Th1 responses and impairing T-cell migration to pancreatic islets through the down-regulation of chemokine expression (Ccl5, Cxcl10, Irf1) in islets. |
| 68302 | ITGB1 | 20877569 | ITGB1 along with ITGA3 is a novel regulator for the recognition of bacterial lipopeptides. ITGB1/ITGA3 integrin regulates endosomal Toll-like receptor (TLR)-2/TLR1 signalling, serving as a mechanism for modulating inflammatory responses. |

| | | | |
|---|---|---|---|
| 68302 | ITGB1 | 26288256 | IFNG primes mast cells for enhanced anti-bacterial and pro-inflammatory responses to Staphylococcus aureus, partially mediated by ITGB1. |
| 45916 | IFNG | 20157607 | The combined treatment of IFNG with 1,25-dihydroxyvitamin D3 (1,25-D3) synergistically enhances nitric oxide (NO) synthesis and NOS2 expression induced by Mycobacterium tuberculosis (MTB) or by its purified protein derivatives in human monocyte-derived macrophages. |
| 45916 | IFNG | 20381453 | IFNG mediates DUOX2 dual oxidase expression via a STAT-independent signalling pathway and providing insights into a novel IFNG signalling pathway with potential importance for regulation of host defence responses. |
| 45916 | IFNG | 17981204 | IFNG is crucial for immunity against intracellular pathogens and for tumour control and it is produced predominantly by natural killer (NK) and natural killer T (NKT) cells as part of the innate immune response. |
| 45916 | IFNG | 24012417 | IFN gamma creates a primed chromatin environment in macrophages to augment TLR-induced gene transcription. |
| 45916 | IFNG | 25474109 | IFNG interferes with the IL-1/NFKBIZ axis in Î²-glucan-activated dendritic cells and promotes T cell-mediated immune responses with increased release of IFNG and IL22, and diminished production of IL17A. |
| 45916 | IFNG | 25732728 | Primary Î³Î´ T cells provide an early source of IFNG during dengue virus (DV) infection and target DV-infected cells. Monocytes also participate as accessory cells that sense DV infection and amplify the cellular immune response in an IL18-dependent manner. |
| 45916 | IFNG | 25637949 | DEFB103A and RNASE7 are induced in human umbilical endothelial cells (HUVECs) by classical inflammatory cytokines such as: IFNG, IL1B and TNF. |
| 45916 | IFNG | 26288256 | IFNG primes mast cells for enhanced anti-bacterial and pro-inflammatory responses to Staphylococcus aureus, partially mediated by ITGB1. |
| 43931 | DDX60L | 26269178 | DDX60L is an important effector protein of the innate immune response against hepatitis C virus. |
| 95591 | HDAC2 | 20798038 | Histone deacetylase 2 (HDAC2) is part of a repressor complex, along with key components that include HDAC1, RE-1 silencing transcription factor (REST), co-repressor of REST (CoREST), and lysine-specific demethylase (LSD) 1. The HDAC/CoREST/REST/LSD1 repressor complex is a significant component of host innate immunity. |
| 95591 | HDAC2 | 26287468 | TET2 selectively mediates active repression of IL6 transcription via NFKBIZ and HDAC2 during inflammation resolution in innate myeloid cells, including dendritic cells and macrophages. |
| 48252 | NFKBIZ | 16513645 | Inhibits the DNA binding of RELA and NFKB1 |

| | | | |
|---|---|---|---|
| 48252 | NFKBIZ | 19783680 | A key regulator of IL-6 production in human monocytes and plays an important role in both TLR and NOD-like receptor ligand induced inflammation |
| 48252 | NFKBIZ | 25474109 | IFNG interferes with the IL-1/NFKBIZ axis in Î ²-glucan-activated dendritic cells and promotes T cell-mediated immune responses with increased release of IFNG and IL22, and diminished production of IL17A. |
| 48252 | NFKBIZ | 26287468 | TET2 selectively mediates active repression of IL6 transcription via NFKBIZ and HDAC2 during inflammation resolution in innate myeloid cells, including dendritic cells and macrophages. |
| 9462 | IL6 | 21148800 | IL6 trans-signalling via STAT3 is a critical modulator of LPS-driven pro-inflammatory responses through cross-talk regulation of the TLR4/Mal signalling pathway broader mechanism that regulates the severity of the host inflammatory response. |
| 9462 | IL6 | 22096605 | IL6 synthesis is regulated by the opposing effects of prostaglandin (PG)E(2) and PGD(2) in human chondrocytes. IL6 synthesis is increased by PGE2 and decreased by PGD2 through the modulation of TLR4 synthesis. |
| 9462 | IL6 | 22030478 | Activation of either TLR4 or TLR2/6 significantly increased IL6 expression by U937 mononuclear cells. Co-activation of TLR4 and TLR2/6, led to a further augmentation on IL-6 expression. |
| 9462 | IL6 | 22426116 | IL6 is strategically upregulated by virulent Mycobacterium tuberculosis to inhibit the induction of innate immunity. |
| 9462 | IL6 | 23735697 | Hyperglycemia abrogates the ability of IL6 to induce neutrophil extracellular traps. |
| 9462 | IL6 | 24012417 | IFN gamma creates a primed chromatin environment in macrophages to augment TLR-induced IL6 transcription |
| 9462 | IL6 | 26287468 | TET2 selectively mediates active repression of IL6 transcription via NFKBIZ and HDAC2 during inflammation resolution in innate myeloid cells, including dendritic cells and macrophages. |
| 32690 | TET2 | 26287468 | TET2 selectively mediates active repression of IL6 transcription via NFKBIZ and HDAC2 during inflammation resolution in innate myeloid cells, including dendritic cells and macrophages. |
| 143980 | Hdac2 | 26287468 | Tet2 selectively mediates active repression of Il6 transcription via Nfkbiz and Hdac2 during inflammation resolution in innate myeloid cells, including dendritic cells and macrophages. |
| 165943 | Nfkbiz | 26287468 | Tet2 selectively mediates active repression of Il6 transcription via Nfkbiz and Hdac2 during inflammation resolution in innate myeloid cells, including dendritic cells and macrophages. |

| | | | |
|---|---|---|---|
| 144970 | Il6 | 21148800 | Il6 trans-signaling via Stat3 is a critical modulator of LPS-driven pro-inflammatory responses through cross-talk regulation of the Tlr4/Mal signaling pathway as a broader mechanism that regulates the severity of the host inflammatory response. |
| 144970 | Il6 | 22096605 | Il6 synthesis is regulated by the opposing effects of prostaglandin (PG)E(2) and PGD(2) in human chondrocytes. Il6 synthesis is increased by PGE2 and decreased by PGD2 through the modulation of Tlr4 synthesis. (Demonstrated in human) |
| 144970 | Il6 | 22030478 | Activation of either Tlr4 or Tlr2/6 significantly increased Il6 expression by U937 mononuclear cells. Co-activation of Tlr4 and Tlr2/6, led to a further augmentation on Il6 expression. (Demonstrated in human) |
| 144970 | Il6 | 22426116 | Il6 is strategically upregulated by virulent Mycobacterium tuberculosis to inhibit the induction of innate immunity. (Demonstrated in human) |
| 144970 | Il6 | 23359591 | Mycobacterium tuberculosis regulates host IL6 production to inhibit type I interferon-signalling. |
| 144970 | Il6 | 24412616 | Il6 causes compromised tissue repair by shifting acute inflammation into a more chronic profibrotic state through induction of T helper type1 cell responses as a consequence of recurrent inflammation. |
| 144970 | Il6 | 25398005 | Ybx1 controls intracellular Il6 mRNA levels in a cell type-specific manner, leading to functions that are dependent on the extracellular and intracellular distribution of Ybx1. |
| 144970 | Il6 | 25725105 | Cfp plays a role in intestinal homeostasis in response to an infectious challenge to activate Hc (C5a), which in turn provides protection through Il6 expression by the epithelium. |
| 144970 | Il6 | 25595775 | Defb1 is important for the control of early mucosal Candida infection and plays a critical role in the induction of innate inflammatory mediators including, Il1b, Il6, Cxcl1, Il17a, and Il17f. |
| 144970 | Il6 | 26287468 | Tet2 selectively mediates active repression of Il6 transcription via Nfkbiz and Hdac2 during inflammation resolution in innate myeloid cells, including dendritic cells and macrophages. |
| 195027 | Tet2 | 26287468 | Tet2 selectively mediates active repression of Il6 transcription via Nfkbiz and Hdac2 during inflammation resolution in innate myeloid cells, including dendritic cells and macrophages. |
| 161368 | March5 | 21625535 | March5 is a mitochondrial ubiquitin ligase that positively regulates Tlr7 signalling. March5 interacts with Tank to induce NFkB-mediated gene expression. |
| 161368 | March5 | 26246171 | March5 modulates Mavs-mediated antiviral signalling, preventing excessive immune reactions. |

| | | | |
|---|---|---|---|
| 82450 | MARCH5 | 21625535 | MARCH5 is a mitochondrial ubiquitin ligase that positively regulates TLR7 signalling. MARCH5 interacts with TANK to induce NFkB-mediated gene expression. |
| 82450 | MARCH5 | 26246171 | MARCH5 modulates MAVS-mediated antiviral signalling, preventing excessive immune reactions. |
| 175760 | Bnip3l | 26253785 | Mitophagy-inducing proteins Bnip3 and Bnip3l play a regulatory role in the generation of robust natural killer cell memory. |
| 269444 | Bnip3 | 26253785 | Mitophagy-inducing proteins Bnip3 and Bnip3l play a regulatory role in the generation of robust natural killer cell memory. |
| 137564 | Isg15 | 22022264 | Isg15 expression is induced during a hepatitis C virus infection in an Irf3-dependent manner. Isg15 acts as a negative modulator of the RIG-I pathway by mediating the level of Ddx58 (RIG-I) ubiquitination. (Demonstrated in human) |
| 137564 | Isg15 | 26259872 | Isg15 protects against Listeria monocytogenes infection. |
| 84312 | ISG15 | 19043203 | ISG15 (ISGylation) post-translational modification is mediated by a sequential reaction similar to ubiquitination and has been shown to negatively regulate the NF-kappaB pathway. |
| 84312 | ISG15 | 22022264 | ISG15 expression is induced during a hepatitis C virus infection in an IRF3-dependent manner. ISG15 acts as a negative modulator of the RIG-I pathway by mediating the level of DDX58 (RIG-I) ubiquitination. |
| 84312 | ISG15 | 24448099 | ISG15 does not directly alter human rhinovirus replication but modulates immune signalling via the viral sensor protein DDX58 to impact production of CXCL10, which has been linked to innate immunity to viruses. |
| 84312 | ISG15 | 24448099 | Human rhinovirus infection of epithelial cells induces the expression and secretion of ISG15, which modulates immune responses via effects on DDX58, and by regulating CXCL10 production. |
| 84312 | ISG15 | 26259872 | ISG15 protects against Listeria monocytogenes infection. |
| 159848 | Cdkn1a | 26259914 | During sepsis-induced generation of myeloid-derived suppressor cells, transcription factor Nfia represses Cdkn1a to arrest differentiation of Gr1+ CD11b+ cells. |
| 164923 | Nfia | 26259914 | During sepsis-induced generation of myeloid-derived suppressor cells, transcription factor Nfia represses Cdkn1a to arrest differentiation of Gr1+ CD11b+ cells. |
| 53485 | IFNB1 | 20174559 | IFNB1 has an essential role in the anti-viral response and optineurin (OPTN) has a role in the inhibition of virus-triggered IFNB1 induction. |
| 53485 | IFNB1 | 20501842 | Viral RNA-induced IFNB1 production is suppressed by oncogenic RAS through negative regulation of RIG-I signalling, leading to promotion of virus spread. |

| | | | |
|---|---|---|---|
| 53485 | IFNB1 | 20699220 | IFNB1 expression is inhibited by influenza A virus polymerase by binding to IFN-beta promoter stimulator 1 (MAVS). |
| 53485 | IFNB1 | 20298124 | IFNB1 is a type I interferon (IFN-I) and the IFN-I family comprises a wide number of cytokines with different modulatory effects on angiogenesis, cell growth, fibrosis, apoptosis and autoimmunity. |
| 53485 | IFNB1 | 21408089 | IFNB1 deficiency results in a partial suppression of the sterol pathway in macrophages during viral infections, thereby linking the regulation of lipid metabolism pathway with interferon anti-viral defence responses. (Demonstrated in murine model) |
| 53485 | IFNB1 | 21602824 | IFNB1 secretion is greater upon viable E. coli infection in comparison to heat killed E. coli vaccine or LPS. The induction of IFNB1 is dependent on TICAM1-IRF3 signalling. (Demonstrated in murine model). |
| 53485 | IFNB1 | 22291574 | IFNB1 expression pattern during viral infection is a highly stochastic process influenced by cell-to-cell variability in viral induction processes. (Demonstrated in mice) |
| 53485 | IFNB1 | 22912878 | IFNB1 production is fundamental to the efficient control of Listeria monocytogenes during the early innate phase of infection. NK cells treated with IFNB1 during early infection were able to reduce bacterial titer in the spleen and significantly improve survival of infected mice. (Demonstrated in mouse) |
| 53485 | IFNB1 | 25463536 | Macrocyclic NS3-4A resistance-associated amino acid variants (RAVs) with substitutions at residue D168 of the hepatitis C virus protease result in an increased capacity of NS3-4A to cleave MAVS and suppress IFNB1 induction. |
| 53485 | IFNB1 | 25505178 | Coronavirus engages papain-like proteases to escape from the innate antiviral response of the host by inhibiting TP53-IRF7-IFNB1 signalling. |
| 53485 | IFNB1 | 25505063 | Hepatitis B virus (HBV) polymerase inhibits TMEM173-stimulated IRF3 activation and IFNB1 induction. |
| 53485 | IFNB1 | 25678110 | ELAVL1 is required for the stabilization of IFNB1 mRNA, and suppression of ELAVL1 leads to impaired expression of IFNB1 in response to poly(I:C) treatment. |
| 53485 | IFNB1 | 26202980 | IFNB1 selectively restricts the transcriptional responses mediated by both the TLRs and the NOD-like receptors in Salmonella enterica serovar Typhimurium infection in macrophages. |
| 70010 | JMJD6 | 26221041 | Reversible arginine methylation of TRAF6 is regulated by PRMT1 and JMJD6 and this in turn regulates TRAF6-dependent TLR signalling. |

| | | | |
|---|---|---|---|
| 63299 | PRMT1 | 19136629 | PRMT1 is a protein arginine methyltransferase and a novel and crucial negative regulator of STAT1 activation that controls PIAS1-mediated repression by arginine methylation. |
| 63299 | PRMT1 | 26221041 | Reversible arginine methylation of TRAF6 is regulated by PRMT1 and JMJD6 and this in turn regulates TRAF6-dependent TLR signalling. |
| 102434 | RORC | 26187413 | Differentiation of Type 3 innate lymphoid cells (ILC3) to IL7R(+) ILC1 is reversible whereas IL7R(+) ILC1 can differentiate to ILC3 in the presence of IL2, IL23A, and IL1B dependent on the transcription factor RORC, and this process is enhanced in the presence of retinoic acid. |
| 40891 | IL23A | 20405269 | IL23A expression is regulated by MAP3K8 in lipopolysaccharide (LPS)-stimulated macrophages through ERK activation. |
| 40891 | IL23A | 20404142 | IL23A suppresses innate immune response independently of IL17A during carcinogenesis and metastasis (shown in mice). |
| 40891 | IL23A | 21097874 | IL23A is a LPS induced gene, and its expression in macrophages correlate with the severity of chronic intestinal inflammation. IL23A is transcriptionally inhibited by the binding of IRF1 to the ISRE element and serve as a homeostatic checkpoint in chronic intestinal inflammation (shown in mice). |
| 40891 | IL23A | 26187413 | Differentiation of Type 3 innate lymphoid cells (ILC3) to IL7R(+) ILC1 is reversible whereas IL7R(+) ILC1 can differentiate to ILC3 in the presence of IL2, IL23A, and IL1B dependent on the transcription factor RORC, and this process is enhanced in the presence of retinoic acid. |
| 36892 | IL2 | 26187413 | Differentiation of Type 3 innate lymphoid cells (ILC3) to IL7R(+) ILC1 is reversible whereas IL7R(+) ILC1 can differentiate to ILC3 in the presence of IL2, IL23A, and IL1B dependent on the transcription factor RORC, and this process is enhanced in the presence of retinoic acid. |
| 16161 | IL7R | 26187413 | Differentiation of Type 3 innate lymphoid cells (ILC3) to IL7R(+) ILC1 is reversible whereas IL7R(+) ILC1 can differentiate to ILC3 in the presence of IL2, IL23A, and IL1B dependent on the transcription factor RORC, and this process is enhanced in the presence of retinoic acid. |
| 160059 | Lcn2 | 26190773 | Macrophage iron regulatory proteins Aco1 and Ireb2 are protective against Salmonella by promoting the induction of Lcn2, a host antimicrobial factor that inhibits bacterial uptake of iron-laden siderophores, and by suppressing the ferritin iron pool. |

| | | | |
|---|---|---|---|
| 167179 | Ireb2 | 26190773 | Macrophage iron regulatory proteins Aco1 and Ireb2 are protective against Salmonella by promoting the induction of Lcn2, a host antimicrobial factor that inhibits bacterial uptake of iron-laden siderophores, and by suppressing the ferritin iron pool. |
| 135868 | Aco1 | 26190773 | Macrophage iron regulatory proteins Aco1 and Ireb2 are protective against Salmonella by promoting the induction of Lcn2, a host antimicrobial factor that inhibits bacterial uptake of iron-laden siderophores, and by suppressing the ferritin iron pool. |
| 47893 | TMEM173 | 18724357 | TMEM173 (STING) is an endoplasmic reticulum (ER) receptor that facilitates interferon (IFN) induction by binding to DDX58 (RIG-I) and to subunits of TRAP complex that facilitates translocation of proteins into the ER following translation. |
| 47893 | TMEM173 | 18818105 | TMEM173 is a critical mediator of virus-triggered type I IFN signalling and a critical mediator of virus-triggered IRF3 activation. |
| 47893 | TMEM173 | 19776740 | TMEM173 is essential for host defence against DNA pathogens such as HSV-1 and facilitates the adjuvant activity of DNA-based vaccines. |
| 47893 | TMEM173 | 19285439 | TMEM173 is an adaptor protein that links virus-sensing receptors to IRF3 activation. RNF5 negatively regulates this virus-triggered signaling by targeting TMEM173 for ubiquitination and degradation at the mitochondria. |
| 47893 | TMEM173 | 19926846 | TMEM173 is required for double stranded DNA-triggered innate immune responses where, upon sensing dsDNA, TMEM173 moves from the endoplasmic reticulum (ER) to the Golgi apparatus and finally reaches the cytoplasmic punctate structures to assemble with TANK-binding kinase 1 (TBK1). |
| 47893 | TMEM173 | 21820332 | TMEM173 is involved in the innate immune recognition of Plasmodium falciparum AT-rich DNA and in the subsequent induction of type I IFNs. (Demonstrated in mouse) |
| 47893 | TMEM173 | 22000020 | TMEM173 activates STAT6 during viral infection to induce genes responsible for immune cell homing. (Demonstrated in mice) |
| 47893 | TMEM173 | 22761576 | TMEM173 is cleaved by dengue viral protease to suppress IRF3 activation and subvert antiviral immunity. |
| 47893 | TMEM173 | 23055924 | Dengue viral NS2B3 protease complex selectively targets TMEM173 (STING) for degradation to inhibit type I IFN production in human dendritic cells. |
| 47893 | TMEM173 | 23542348 | TMEM173 (STING) is targeted by hepatitis C viral protease to disrupt interferon signalling. |
| 47893 | TMEM173 | 24131791 | Cyclic-di-GMP-induced levels of IFI16 suppress the expression of TMEM173 (STING). |

| | | | |
|---|---|---|---|
| 47893 | TMEM173 | 24449861 | In herpes simplex virus 1 (HSV-1) infected cells, the stability and function of IFI16 and TMEM173 are dependent on cell derivation and the functional integrity of HSV-1 proteins ICP0 and US3 protein kinase. |
| 47893 | TMEM173 | 24185615 | After viral infection, ELF4 binds to TMEM173 (STING) and induces type I interferon. ELF4 is critical for host antiviral defense. |
| 47893 | TMEM173 | 24449861 | The end result of the interplay between TMEM173 (STING), IFI16, and herpes simplex virus 1 (HSV-1) is determined by the genotype of the infected cells and the functional integrity of HSV-1 proteins infected cell protein 0 (ICP0) and US3 protein kinase. |
| 47893 | TMEM173 | 25401470 | Familial TMEM173 mutation is associated with inflammatory lupus-like manifestations. |
| 47893 | TMEM173 | 25425575 | Cytosolic RNA:DNA hybrids are sensed by the MB21D1-TMEM173 (cGAS-STING) pathway of the innate immune system. |
| 47893 | TMEM173 | 25505063 | Hepatitis B virus (HBV) polymerase inhibits TMEM173-stimulated IRF3 activation and IFNB1 induction. |
| 47893 | TMEM173 | 25526307 | Upon cytoplasmic DNA stimulation, the endoplasmic reticulum protein AMFR is recruited to and interacts with TMEM173 in an INSIG1-dependent manner. |
| 47893 | TMEM173 | 25792739 | Stimulation of TMEM173-dependent IRF3 activation by ultraviolet radiation is due to apoptotic signalling-dependent disruption of ULK1, a pro-autophagic protein that negatively regulates TMEM173. |
| 47893 | TMEM173 | 26646986 | 4-(2-chloro-6-fluorobenzyl)-N-(furan-2-ylmethyl)-3-oxo-3,4-dihydro-2H-benzo[b][1,4]thiazine-6-carboxamide (G10) requires STING to trigger IRF3/IFN-associated transcription in human fibroblasts and subsequently blocking replication of Chikungunya virus, Venezuelan Encephalitis virus, and Sindbis virus. |
| 47893 | TMEM173 | 26199418 | Viral interferon regulatory factor 1 (vIRF1), targets TMEM173 by preventing it from interacting with TBK1, thereby inhibiting TMEM173's phosphorylation and concomitant activation, resulting in an inhibition of the DNA sensing pathway. |
| 44993 | TBK1 | 18977754 | TBK1 alternative splicing negatively regulates virus-triggered IFN-beta signalling pathway by disrupting DDX58 and MAVS, and inhibiting IFN-beta signalling pathways. |
| 44993 | TBK1 | 16286919 | TBK1 and IKBKE (IKKi) kinases are required for innate immune activation by B-DNA, which may be important in antiviral innate immunity and other DNA-associated immune disorders. |
| 44993 | TBK1 | 12692549 | TBK1 and IKBKE have a pivotal role in coordinating the activation of IRF3 and NF-kappaB in the innate immune response. |

| | | | |
|---|---|---|---|
| 44993 | TBK1 | 21820332 | TBK1 is involved in the innate immune recognition of Plasmodium falciparum AT-rich DNA and in the subsequent induction of type I IFNs. Mice lacking Tbk1 are resistant to otherwise lethal cerebral malaria. (Demonstrated in mouse) |
| 44993 | TBK1 | 22921120 | TBK1 is a key regulator of immunological autophagy and is responsible for autophagosome maturation into bactericidal organelles. (Demonstrated in mouse) |
| 44993 | TBK1 | 23986588 | Herpes simplex virus 1 protein, UL36 ubiquitin-specific protease (UL36USP), deubiquitinates TRAF3 and inhibits recruitment of TBK1 and counteracts the IFNB pathway |
| 44993 | TBK1 | 25070846 | USP2 deubiquitinates K63-linked polyubiquitin chains from TBK1 to terminate TBK1 activation and negatively regulate IFNB1 signalling and antiviral immune response. |
| 44993 | TBK1 | 26082489 | Endotoxin tolerance re-programs TLR4 signalling via suppression of PELI1, a positive regulator of MyD88- and TIR domain-containing adapter inducing IFN-Î ² (TRIF)-dependent signalling that promotes K63-linked polyubiquitination of IRAK1, TBK1, and TAK1. |
| 44993 | TBK1 | 25855743 | HIV-1 accessory proteins Vpr and Vif bind to TBK1, inhibit its autophosphorylation, and prevent induction of type I and III interferon in myeloid cells. |
| 44993 | TBK1 | 26199418 | Viral interferon regulatory factor 1 (vIRF1), targets TMEM173 by preventing it from interacting with TBK1, thereby inhibiting TMEM173's phosphorylation and concomitant activation, resulting in an inhibition of the DNA sensing pathway. |
| 171519 | Nrip1 | 22388040 | NF-Î°B-mediated degradation of the coactivator NRIP1 (RIP140) regulates inflammatory responses and contributes to endotoxin tolerance. |
| 171519 | Nrip1 | 22388040 | Nrip1 is degraded by the NF-kB pathway to inactivate inflammatory gene expression and promotes endotoxin tolerance. |
| 171519 | Nrip1 | 26228026 | Nrip1 plays dual roles in regulating the M1-M2 phenotype switch in macrophages: in the nucleus as an M1 enhancer and in the cytosol as an M2 suppressor. |
| 170175 | Bcl11b | 25964371 | Bcl11b, a transcription factor essential in T cell lineage commitment and maintenance, is specifically expressed in progenitors committed to the group 2 innate lymphoid cells (ILC2) lineage and is required for ILC2 development. |
| 170175 | Bcl11b | 25964370 | Bcl11b is a factor in the differentiation of group 2 innate lymphoid cells (ILC2s). |
| 170175 | Bcl11b | 26231117 | Bcl11b sustains the genetic and functional programs, as well as lineage fidelity, of mature type 2 innate lymphoid cells. |

| | | | |
|---|---|---|---|
| 127807 | Map3k8 | 21135874 | Map3k8 is a MEK kinase that is require for the activation of MAP kinases in myeloid cells following TLR and TNF receptor stimulation. Map3k8 is critical for production of the pro-inflammatory cytokine TNF during inflammatory responses. |
| 127807 | Map3k8 | 26241898 | Map3k8 plays a role in facilitating both innate and adaptive antiviral responses by transducing Type I interferon (IFN) signals and promoting expression of IFN-stimulated genes (ISGs). |
| 151194 | Egfr | 26243875 | Il33-dependent group 2 innate lymphoid cells (ILC2s) signal via Areg-Egfr in damaged epithelia to restore epithelial barrier function and maintain tissue homeostasis. |
| 179096 | Areg | 26243875 | Il33-dependent group 2 innate lymphoid cells (ILC2s) signal via Areg-Egfr in damaged epithelia to restore epithelial barrier function and maintain tissue homeostasis. |
| 157638 | Il33 | 21623379 | Il33 is produced in alveolar macrophages that have been infected with Influenza A virus. The Il33-Il13 signalling axis is required for airway hyper-reactivity in asthma. |
| 157638 | Il33 | 22119406 | Innate lymphoid cells responding to Il33 mediate airway hyperreactivity independently of adaptive immunity. |
| 157638 | Il33 | 23960191 | IL33 predominantly elicits group 2 innate lymphoid cells (ILC2) responses, and IL25 simultaneously elicits phenotypically and functionally distinct ILC2 and multipotent progenitor type 2 cell populations at multiple tissue sites. |
| 157638 | Il33 | 24631157 | Chitin induces IL25, IL33, and TSLP which are required to stimulate ILC2 production of IL5 and IL13. IL5 and IL13, in turn, are required for the accumulation of eosinophils and alternatively activated macrophages that are associated with allergy. |
| 157638 | Il33 | 25659095 | Il33 prevents the development of experimental cerebral malaria by orchestrating a protective immune response via type-2 innate lymphoid cells, M2 macrophages and regulatory T cells. |
| 157638 | Il33 | 25786179 | Cigarette smoke decreases Il1rl1 expression on group 2 innate lymphoid cells while elevating Il1rl1 expression on macrophages and natural killer cells, thus altering Il33 responsiveness within the lung to infection. |
| 157638 | Il33 | 26092469 | Group 2 innate lymphoid cell (ILC2)-intrinsic Il33 signalling and Icosl expression promote regulatory T cell accumulation, whereas the inflammatory cytokine Ifng counter-regulates these effects, in part through direct effects on ILC2s. |
| 157638 | Il33 | 26243875 | Il33-dependent group 2 innate lymphoid cells (ILC2s) signal via Areg-Egfr in damaged epithelia to restore epithelial barrier function and maintain tissue homeostasis. |

| | | | |
|---|---|---|---|
| 148496 | Card9 | 20351059 | Card9 is critical for full activation of innate immunity by converging signals downstream of multiple pattern recognition receptors (PRRs) and plays a pivotal role in autonomous innate host defense against tuberculosis. |
| 148496 | Card9 | 24379290 | Card9 is largely dispensable for the innate immune response to oropharyngeal candidiasis whereas it is vital for the adaptive Th17 response. |
| 148496 | Card9 | 26627732 | Card9-mediated activation of the innate immune system exacerbates influenza pneumonia. Card9 deficiency improves mortality with reduced inflammatory cytokine/chemokines. |
| 141021 | Ikbkb | 21138416 | Ikbkb and other IKK kinases regulate each other by an intricate network involving phosphorylation of their catalytic and regulatory (NEMO, TANK) subunits to balance their activities during innate immunity. |
| 141021 | Ikbkb | 26620909 | The reversible ubiquitin editing of Nlrc5 determines Nlrc5 â € Ikbkb interaction dynamics and plays a crucial role in precisely regulating NFÎºB signalling |
| 182231 | Nlrc5 | 26620909 | The reversible ubiquitin editing of Nlrc5 determines Nlrc5 â € Ikbkb interaction dynamics and plays a crucial role in precisely regulating NFÎºB signalling |
| 32729 | NLRC5 | 19414032 | NLRC5 is nn NLR (nucleotide-binding domain and leucine-rich repeat containing family receptors) that is part of the family of pattern recognition receptors (PRRs) and is involved in immunity against intracellular pathogens. |
| 32729 | NLRC5 | 20061403 | NLRC5 represents a molecular switch of IFN-gamma activation sequence/IFN-specific response element signalling pathways contributing to antiviral defence mechanisms. |
| 32729 | NLRC5 | 20434986 | NLRC5 is a negative regulator that blocks two central components of the NF-kappaB and type I interferon signalling pathways and has an important role in homeostatic control of innate immunity. |
| 32729 | NLRC5 | 21148033 | NLRC5 is dispensable for cytokine induction in virus and bacterial infections under physiologic conditions. (Demonstrated in murine model) |
| 32729 | NLRC5 | 26620909 | The reversible ubiquitin editing of NLRC5 determines NLRC5â€ IKBKB interaction dynamics and plays a crucial role in precisely regulating NFÎºB signalling |
| 107914 | NLRP3 | 25978411 | Influenza A virus non-structural protein 1, NS1, physically interacts with endogenous NLRP3 downregulating NLRP3 inflammasome activation as well as NF-kB, leading to a reduction in the levels of inflammatory cytokines. |
| 107914 | NLRP3 | 25816776 | RNA cleavage products, catalyzed by RNASEL, bind to DHX33 to facilitate the formation of a complex with MAVS and NLRP3 during viral infection. |

| | | | |
|---|---|---|---|
| 107914 | NLRP3 | 26610398 | H. pylori infection induces the expression and activation of components of NLRP3 inflammasomes in neutrophils and this activation is independent of a functional type IV secretion system, TLR2 and TLR4. |
| 124824 | C2 | 25381436 | Staphylococcus aureus extracellular adherence protein (Eap) binds to C4B to inhibit binding of both full-length C2 and its C2b fragment, disrupting the formation of the C3 proconvertase (C4b2) and significantly diminishing the extent of S. aureus opsonophagocytosis and killing by neutrophils. |
| 124824 | C2 | 26608922 | Complement interfering protein (CIP) of group B Streptococcus (GBS) shows high affinity toward C4B and inhibits its interaction with C2, presumably preventing the formation of the C4BC2A convertase and GBS phagocytic killing in the absence of anti-GBS antibodies. |
| 301132 | C4B | 25381436 | Staphylococcus aureus extracellular adherence protein (Eap) binds to C4B to inhibit binding of both full-length C2 and its C2b fragment, disrupting the formation of the C3 proconvertase (C4b2) and significantly diminishing the extent of S. aureus opsonophagocytosis and killing by neutrophils. |
| 301132 | C4B | 26608922 | Complement interfering protein (CIP) of group B Streptococcus (GBS) shows high affinity toward C4B and inhibits its interaction with C2, presumably preventing the formation of the C4BC2A convertase and GBS phagocytic killing in the absence of anti-GBS antibodies. |
| 135957 | Ddx58 | 19936053 | Ddx58 (Rig-I) is responsible for the cytosolic recognition of Legionella pneumophila RNA and the subsequent induction of type I IFN response. |
| 135957 | Ddx58 | 21690088 | Ddx58 and Nod2 colocalize to cellular ruffles and cell-cell junctions to form a protein complex via the CARD domains. Ddx58 negatively regulates ligand-induced NFkB signalling mediated by Nod2, and conversely, Nod2 negatively regulates type I interferon induction by Ddx58. (Demonstrated in human) |
| 135957 | Ddx58 | 21695051 | Ddx58, through the TRAIL pathway, initiates apoptosis in hepatocytes infected with hepatitis C Virus. HCV envelope proteins counteract the antiviral host defence by inhibiting the expression of Ddx58. (Demonstrated in human) |
| 135957 | Ddx58 | 22072774 | Ddx58 (RIG-I) ubiquitination is inhibited by arterivirus and nairovirus deubiquitinating enzymes (DUBs), resulting in the inhibition of RIG-I-like receptor (RLR)-mediated innate immune signalling. |
| 135957 | Ddx58 | 22912779 | Antiviral stress granules containing Ddx58 (RIG-I) and Eif2ak2 (PKR) have a critical role in viral detection and innate immunity. |
| 135957 | Ddx58 | 23064150 | DDX58 (RIG-I) detects cytosolic Listeria monocytogenes infections by sensing secreted bacterial nucleic acids. |

| | | | |
|---|---|---|---|
| 135957 | Ddx58 | 23553835 | Ddx58 (RIG-I) is a positive regulator of NF-kB signalling via binding to Nfkb1 mRNA. |
| 135957 | Ddx58 | 23966395 | Ddx58 and Ifih1 are essential pattern recognition receptors for protection against West Nile virus infection in vivo. |
| 135957 | Ddx58 | 24692634 | Ddx58 preferentially binds to coding RNA from S. Typhimurium during infection leading to the expression of IFN beta andthis immunostimulatory activity depends on 5â€² triphosphorylation of RNA. |
| 135957 | Ddx58 | 26074083 | Ddx58 is the primary pattern recognition receptor (PRR) for influenza A virus (IAV), but Ifih1 is a significant contributor to the cellular defense against IAV. |
| 135957 | Ddx58 | 26146945 | Ddx58 acts in parallel with Zbp1 in an RNA polymerase III-dependent manner to initiate glial responses to herpes simplex virus-1. |
| 135957 | Ddx58 | 26645583 | Mir485 exhibits bispecificity, targeting Ddx58 in cells with a low abundance of H5N1 virus and viral PB1 in cells with increased amounts of the H5N1 virus. |
| 221446 | Mir485 | 26645583 | Mir485 exhibits bispecificity, targeting Ddx58 in cells with a low abundance of H5N1 virus and viral PB1 in cells with increased amounts of the H5N1 virus. |
| 127079 | MIR485 | 26645583 | MIR485 exhibits bispecificity, targeting DDX58 in cells with a low abundance of H5N1 virus and viral PB1 in cells with increased amounts of the H5N1 virus. |
| 92794 | PLA2G2A | 26644377 | IL22 induced at an early stage of L. monocytogenes infection enhances innate immunity against L. monocytogenes in the liver by stimulating hepatocytes to produce an antimicrobial molecule, PLA2G2A |
| 46046 | IL22 | 18978771 | IL22 plays a role in mucosal immunity where it helps constrain inflammation and protect mucosal sites. |
| 46046 | IL22 | 20870448 | IL22 is a member of the IL-10 cytokine family that is produced by special immune cell populations and ts primary effects on target cells include its role in innate immune defence against infections, in tumourigenesis, and in inflammatory diseases. |
| 46046 | IL22 | 21469124 | IL22 increases the TNF-alpha-dependent induction and secretion of several immune-modulatory molecules such as initial complement factors, antimicrobial peptides and chemokines in primary keratinocytes. IL22-mediated induction of innate immunity is crucial for the maintenance of epidermal integrity during infection with Candida albicans. |
| 46046 | IL22 | 21874025 | IL22 is produced by lymphoid tissue-inducer cells where it regulates the maintenance of colonic lymphoid structures during Citrobacter rodentium infection, a mechanism that bridges the lymphotoxin pathway to mucosal epithelial defense mechanisms. (Demonstrated in mice) |

| | | | |
|---|---|---|---|
| 46046 | IL22 | 22921121 | IL22 protects intestinal stem cells from inflammatory tissue damage and regulates sensitivity to graft versus host disease. |
| 46046 | IL22 | 24412612 | Stat3 mediates protection against intestinal infection by inducing innate lymphoid cell derived-Il22. (Demonstrated in mice) |
| 46046 | IL22 | 25476703 | IL22 protects against and IL22RA2 aggravates liver fibrosis and cirrhosis in chronic liver infections. |
| 46046 | IL22 | 25474109 | IFNG interferes with the IL-1/NFKBIZ axis in Î ²-glucan-activated dendritic cells and promotes T cell-mediated immune responses with increased release of IFNG and IL22, and diminished production of IL17A. |
| 46046 | IL22 | 25510212 | In alveolar epithelium, IL22 upregulates DEFB4A gene expression via STAT3. |
| 46046 | IL22 | 26644377 | IL22 induced at an early stage of L. monocytogenes infection enhances innate immunity against L. monocytogenes in the liver by stimulating hepatocytes to produce an antimicrobial molecule, PLA2G2A |
| 165137 | Cd47 | 26010544 | Cd47 plays a protective role against disseminated candidiasis and alters pro-inflammatory and immunosuppressive pathways known to regulate innate and T cell immunity. |
| 165137 | Cd47 | 27194758 | Cd47 plays a role as a negative regulator in inducing protective immune responses to influenza vaccination. |
| 16420 | SKP2 | 27194766 | IFI27 restricts viral infection by recruiting an E3 ligase, SKP2, for ubiquitination and degradation of viral protein. |
| 17962 | IFI27 | 20939681 | IFI27 is a mitochondrial protein and its expression sensitizes cells to apoptotic stimuli via mitochondrial membrane destabilization. |
| 17962 | IFI27 | 27194766 | IFI27 restricts viral infection by recruiting an E3 ligase, SKP2, for ubiquitination and degradation of viral protein. |
| 186283 | Clec4n | 21267996 | Clec4n, a C-type lectin receptor, is a pattern recognition receptor critical for immune responses to fungi. Clec4n is coupled to Syk kinase and signals via Card9 to activate NFKB, which in turns induces both innate and adaptive immunity. |
| 186283 | Clec4n | 21357742 | Clec4n is critical for the development of house dust mite (Dermatophagoides farinae) elicited eosinophilic and neutrophilic pulmonary inflammation. Clec4n was also found to be crucial for the Th2 cytokine induction in the lungs and re-stimulated lymph nodes. |
| 186283 | Clec4n | 21677049 | Clec4n is expressed mainly in DCs and macrophages. Clec4n recognizes alpha-mannans with its carbohydrate recognition domain and transduces signals through association with the ITAM-containing Fc receptor gamma chain, which recruits Syk and initiates the Card9/NFkB signalling cascade. |

| | | | |
|---|---|---|---|
| 186283 | Clec4n | 27194783 | Pik3cd regulates Clec4n signalling and the generation of Th2 and Th17 immunity. |
| 205331 | Pik3cd | 27194783 | Pik3cd regulates Clec4n signalling and the generation of Th2 and Th17 immunity. |
| 81919 | IFIT3 | 21813773 | IFIT3 triggers host antiviral responses by bridging TBK1 to MAVS, and IFIT3 plays an important role in the activation of IRF3. |
| 81919 | IFIT3 | 27210312 | Hepatitis B virus-induced MIR146A attenuates cell-intrinsic anti-viral innate immunity through targeting DDX58 and IFIT3. |
| 774160 | MIR146A | 25705792 | MIR146A upregulation by CXCR4 antagonist AMD3100 treatment or ZBTB16 silencing, decreases CXCR4 protein expression and prevents HIV-1 infection of leukemic monocytic cell line and CD4(+) T lymphocytes. |
| 774160 | MIR146A | 27210312 | Hepatitis B virus-induced MIR146A attenuates cell-intrinsic anti-viral innate immunity through targeting DDX58 and IFIT3. |
| 21240 | TRAF3 | 15708970 | TRAF3 serves as a negative regulator of the non-canonical NF-kappaB pathway by specifically blocking the activation of NF-kappaB via TRAF2/5. |
| 21240 | TRAF3 | 16858409 | TRAF3 is required for type I interferon production in response to intracellular double-stranded RNA. Similarly, a direct and specific interaction of the TRAF domain of TRAF3 with the TRAF-interaction motif (TIM) of MAVS is required for optimal MAVS-mediated antiviral responses. |
| 21240 | TRAF3 | 16306936 | TRAF3 is a major regulator of type I interferon (IFN) production and the innate antiviral response. |
| 21240 | TRAF3 | 20138174 | TRAF3 cooperates with ZMYND11 in the regulation of Epstein-Barr virus-derived LMP1/CTAR1-induced NF-kappaB activation. |
| 21240 | TRAF3 | 20832341 | TRAF3 functions downstream of multiple TNF receptors and receptors that induce interferon (IFN)-alpha, IFN-beta, and IFN-lambda production, including Toll-like receptor 3 (TLR3). TLR3-mediated immunity against primary infection by herpes simplex virus-1 (HSV-1) in the central nervous system is critically dependent on TRAF3. |
| 21240 | TRAF3 | 21660053 | TRAF3 is a highly versatile regulator that positively controls type I interferon production, and negatively regulates MAP kinase activation and alternative NFkB signalling. |
| 21240 | TRAF3 | 22792062 | Upon sensing dsRNA or dsDNA, TRAF3 interacts with ER-to-Golgi transport proteins to induce MAVS-associated innate immune responses. |
| 21240 | TRAF3 | 23986588 | Herpes simplex virus 1 protein, UL36 ubiquitin-specific protease (UL36USP), deubiquitinates TRAF3 and inhibits recruitment of TBK1 and counteracts the IFNB pathway |
| 21240 | TRAF3 | 27213432 | HACE1 plays an inhibitory role in virus-induced signalling by disrupting the MAVS-TRAF3 complex. |

| | | | |
|---|---|---|---|
| 94750 | HACE1 | 27213432 | HACE1 plays an inhibitory role in virus-induced signalling by disrupting the MAVS-TRAF3 complex. |
| 131590 | Casp1 | 19362023 | Casp1 is involved in key innate and healing responses to influenza A virus where Casp1(-/-) mice exhibited increased morbidity after infection with a pathogenic influenza A virus correlating with decreased neutrophil and monocyte recruitment and reduced Il1b (IL-1-beta), Il18 (IL-18), Tnf (TNF-alpha), Il6 (IL-6), Cxcl1 (KC), and Cxcl2 (MIP-2) production. |
| 131590 | Casp1 | 19124602 | Casp1 is part of the inflammasome complex, along with pathogen-specific nucleotide oligomerization and binding domain (NOD)-like receptors (NLRs) and in some cases the scaffolding protein ASC. Formation of the membrane-associated inflammasome complex in murine macrophages, results in cleavage of cytosolic Casp1 substrates and cell death. |
| 131590 | Casp1 | 21439959 | Casp1 is a component of the inflammasome and is required for inflammation in acute pancreatitis. |
| 131590 | Casp1 | 21602824 | Casp1-dependent inflammatory cell death, or pyroptosis, is only induced by viable, but not heat-killed, E. coli. |
| 131590 | Casp1 | 22833538 | Naturally occurring variants of Casp1 differ considerably in structure and the ability to activate Il1b.<br>(Demonstrated in human) |
| 131590 | Casp1 | 25689249 | Activation of the Nlrp3 inflammasome is detrimental during leishmaniasis. Mice lacking the inflammasome components Nlrp3, Pycard, Casp1 exhibit defective Il1b and Il18 production at the infection site and are resistant to cutaneous Leishmania major infection. |
| 131590 | Casp1 | 26056255 | Type 1 regulatory (Tr1) cells suppress Il1b transcription and Casp1 activation via an IL10R â€ dependent mechanism |
| 131590 | Casp1 | 27214553 | Uropathogenic Escherichia coli protein TcpC attenuates activation of the Nlrp3 inflammasome by binding both Nlrp3 and Casp1. |
| 164567 | Retnla | 23355735 | Retnla (RELMalpha) has a proinflammatory role in bacterial-induced colitis. |
| 164567 | Retnla | 27220602 | Expression of Cxcl13, Ifngr1, Retnla and Mrc1 distinguishes between large and small resident peritoneal macrophage subsets. |
| 135936 | Ifngr1 | 27220602 | Expression of Cxcl13, Ifngr1, Retnla and Mrc1 distinguishes between large and small resident peritoneal macrophage subsets. |
| 181227 | Cxcl13 | 27220602 | Expression of Cxcl13, Ifngr1, Retnla and Mrc1 distinguishes between large and small resident peritoneal macrophage subsets. |

| | | | |
|---|---|---|---|
| 138187 | Mrc1 | 27220602 | Expression of Cxcl13, Ifngr1, Retnla and Mrc1 distinguishes between large and small resident peritoneal macrophage subsets. |
| 4758 | NFKBIA | 9891086 | NFKBIA is a common component of the heterogeneous IKK complex that mediates an essential step of the NF-kappaB signal transduction cascade by acting as an inhibitor of NF-kappaB. |
| 4758 | NFKBIA | 12429743 | Tyrosine phosphorylation of NFKBIA is c-Src-dependent, leading to the subsequent activation of NF-kappaB. |
| 4758 | NFKBIA | 16931600 | Nuclear transport of the NFKBIA : RELA complex, required for the appropriate regulation of NF-kappaB signalling, is facilitated by 14-3-3 proteins. |
| 4758 | NFKBIA | 18503636 | NFKBIA ubiquitination and degradation is inhibited by ChlaDub1, a protein of Chlamydia trachomatis, suppressing NF-kappaB activation as a result. |
| 4758 | NFKBIA | 21454695 | NFKBIA degradation occurs through the TNF-stimulated formation of autophagosomes in epithelial cells, which results in the prolonged activation of NFKB activity. |
| 4758 | NFKBIA | 23487427 | Polymorphisms in the NFKBIA promoter are associated with pediatric lung diseases, including childhood asthma, bronchiolitis and bronchopulmonary dysplasia. |
| 4758 | NFKBIA | 26642243 | Haploinsufficiency of A20 (HA20) is caused by high-penetrance loss-of-function germline mutations in TNFAIP3 with increased degradation of NFKBIA, nuclear translocation of RELA, increased expression of NF Î º B mediated proinflammatory cytokines, and defective deubiquitinating activity. |
| 97033 | TNFAIP3 | 18268035 | TNFAIP3 restricts TLR signals by restricting ubiquitination of TRAF6 and restricts MyD88-independent TLR signals by inhibiting Toll/interleukin 1 receptor domain-containing adaptor inducing interferon (IFN) beta (TICAM1)-dependent nuclear factor kappaB signals but not IFN response factor 3 signalling. |
| 97033 | TNFAIP3 | 18342009 | TNFAIP3 restricts NOD2 triggered signals by deubiquitinating RIPK2. |
| 97033 | TNFAIP3 | 16306043 | TNFAIP3 negatively regulates the RIG-I antiviral state by blocking IRF- and NF-kappaB-mediated gene expression. |
| 97033 | TNFAIP3 | 18349075 | TNFAIP3 negatively regulates BCL10- and CARMA3-mediated activation of NF-kappaB by means of its deubiquitylation activity and preventing assembly of the complex containing CARMA3, BCL10 and IKBKG (NEMO). |
| 97033 | TNFAIP3 | 15258597 | TNFAIP3 removes lysine-63 linked ubiquitin chains from RIPK1 and polyubiquitinates RIPK1 with K48-linked ubiquitin chains, targeting it for proteosomal degradation, and hence down-regulating NF-kappaB signalling pathway. |

| | | | |
|---|---|---|---|
| 97033 | TNFAIP3 | 17709380 | TNFAIP3 is phosphorylated by IKBKB and this increases the ability of TNFAIP3 to inhibit the NF-kappaB signalling pathway. |
| 97033 | TNFAIP3 | 19008218 | TNFAIP3 is an early NF-kappaB-responsive gene that encodes a ubiquitin-editing protein that is involved in the negative feedback regulation of NF-kappaB signalling and thus is a central gatekeeper in inflammation and immunity. |
| 97033 | TNFAIP3 | 20304918 | TNFAIP3 (A20) and TAX1BP1 inhibit antiviral signaling by targeting TBK1/IKKi kinases and disrupting a TRAF3-TBK1-IKKi signalling complex. |
| 97033 | TNFAIP3 | 20798608 | TNFAIP3 and TRAF6 differentially regulate TLR4-induced autophagy during inflammatory responses by modulating K63-linked ubiquitination of BECN1 (Beclin 1). |
| 97033 | TNFAIP3 | 21119682 | TNFAIP3 is a deubiquitinase that counteracts E3 ligases and therefore play a prominent role in the down-regulation of NF-Î°B signalling and homeostasis. |
| 97033 | TNFAIP3 | 21220427 | TNFAIP3 expression and TNFAIP3-IRAK1interaction are important for endotoxin tolerance. TNFAIP3 over-expression inhibits LPS-induced activation of NFKB, and is mechanistically linked to endotoxin tolerance through the reprogramming of TLR4 signalling. |
| 97033 | TNFAIP3 | 22031828 | TNFAIP3 promotes intestinal epithelial barrier integrity and inhibits LPS-induced loss of the tight junction protein occludin. (Demonstrated in mice) |
| 97033 | TNFAIP3 | 26642243 | Haploinsufficiency of A20 (HA20) is caused by high-penetrance loss-of-function germline mutations in TNFAIP3 with increased degradation of NFKBIA, nuclear translocation of RELA, increased expression of NF Î º B mediated proinflammatory cytokines, and defective deubiquitinating activity. |
| 174129 | Ifih1 | 21245317 | Ifih1 is an RNA helicase and is a key component in activating the expression of type I IFN in response to viral infection. Viral mRNA with 5' cap and 3' poly(A) from parainfluenza virus 5 is able to activate IFN expression through Rnasel-Ifih1 signalling pathway. |
| 174129 | Ifih1 | 19936053 | Ifih1 (MDA5) is responsible for the cytosolic recognition of Legionella pneumophila RNA and the subsequent induction of type I IFN response. |
| 174129 | Ifih1 | 21637773 | Ifih1 deficiency results in a delayed type I IFN and attenuated type III IFN response to rhinovirus infection, leading to a transient increase in viral titer. Upon recognition of viral dsRNA, Ifih1 synergizes with Tlr3 to induce pro-inflammatory signals leading to airways inflammation and hyper-responsiveness. |
| 174129 | Ifih1 | 23328395 | Paramyxovirus V proteins bind to IFIH1 (MDA5) to disrupt viral RNA recognition and induction of antiviral immunity. |

| | | | |
|---|---|---|---|
| 174129 | Ifih1 | 23966395 | Ddx58 and Ifih1 are essential pattern recognition receptors for protection against West Nile virus infection in vivo. |
| 174129 | Ifih1 | 24362933 | Plasmodium RNA is a pathogen-associated molecular pattern (PAMP) capable of activating a type I IFN response via the cytosolic pattern recognition receptors Ifih1 and Mavs, as well as via transcription factors Irf3 and Irf7. |
| 174129 | Ifih1 | 25451939 | Arl5b negatively regulates the antiviral innate immune response by binding to Ifih1 and prevents the subsequent interaction of Ifih1 to poly(I:C). |
| 174129 | Ifih1 | 26074083 | Ddx58 is the primary pattern recognition receptor (PRR) for influenza A virus (IAV), but Ifih1 is a significant contributor to the cellular defense against IAV. |
| 174129 | Ifih1 | 26633895 | Transgenic picornavirus RNA-dependent RNA polymerase (RdRP) expression in mice produces a quantitatively dramatic, sustained, effective antiviral interferon-stimulated genes (ISG) network, which requires the MDA5-MAVS pathway. |
| 159374 | Il2rb | 26668377 | Selective loss of the histone-lysine N-methyltransferase Ezh2 (enhancer of zeste homolog 2) or inhibition of its enzymatic activity increases generation of the Il2rb (CD122) natural killer (NK) precursors and mature NK progeny. |
| 143636 | Ezh2 | 26668377 | Selective loss of the histone-lysine N-methyltransferase Ezh2 or inhibition of its enzymatic activity increases generation of the Il2rb (CD122) natural killer (NK) precursors and mature NK progeny. |
| 192196 | Ifng | 23754402 | IFNG is produced by neutrophils to mount host protection against intracellular pathogens. |
| 192196 | Ifng | 23818011 | IFNG produced by natural killer cells exacerbates Listeria monocytogenes infection by inhibiting granulocyte recruitment. |
| 192196 | Ifng | 26065469 | Ticam2 mediates antibacterial defence during Gram-negative pneumonia by inducing Ifng at the primary site of infection. |
| 192196 | Ifng | 26092469 | Group 2 innate lymphoid cell (ILC2)-intrinsic Il33 signalling and Icosl expression promote regulatory T cell accumulation, whereas the inflammatory cytokine Ifng counter-regulated the effects of Il33, in part through direct effects on ILC2s. |
| 192196 | Ifng | 26843324 | Irf8-Ifng circuit is a novel gastric innate immune mechanism in the host defense against infection with Helicobacter pylori. |
| 196509 | Irf8 | 22942423 | De-SUMOylation of IRF8 at residue Lys310 acts as a molecular mechanism to trigger innate immune responses in activated macrophages. |
| 196509 | Irf8 | 26843324 | Irf8-Ifng circuit is a novel gastric innate immune mechanism in the host defense against infection with Helicobacter pylori. |

| | | | |
|---|---|---|---|
| 193230 | Sppl3 | 26851218 | Sppl3 is an intramembrane aspartyl protease that controls natural killer cell maturation and cytotoxicity. |
| 134957 | Ahr | 21683686 | Ahr deficiency impairs TLR and NFkB-mediated proinflammatory gene expression after activation by a classical stimulus, such as LPS. |
| 134957 | Ahr | 23954130 | Innate expression of Ahr plays a protective role in T-cell-induced colitis by suppressing T helper 17 cells, thus inhibiting proinflammatory cytokine production. |
| 134957 | Ahr | 26857571 | Uropathogenic Escherichia coli suppresses neutrophil migration early in bacterial cystitis by eliciting an Ido1-mediated increase in local production of kynurenines, which act through the Ahr to impair neutrophil chemotaxis. |
| 142448 | Ido1 | 22355111 | Ido1 limits innate and adaptive immunity to apoptotic self-antigens. Ido1-mediated inhibition of inflammation plays a key role in suppressing systemic autoimmune diseases. |
| 142448 | Ido1 | 26857571 | Uropathogenic Escherichia coli suppresses neutrophil migration early in bacterial cystitis by eliciting an Ido1-mediated increase in local production of kynurenines, which act through the Ahr to impair neutrophil chemotaxis. |
| 154384 | Lgals3 | 22486577 | Lgals3 influences the course of malaria in a Plasmodium species-specific manner. |
| 154384 | Lgals3 | 26857579 | Lgals3 plays an important role in innate immunity to infection and colonization of Helicobacter pylori. |
| 212683 | Trpm5 | 26847546 | Tuft cells express Il25 and elicit group 2 innate lymphoid cells in a Trpm5-dependent manner in response to parasite colonization. |
| 162540 | Il25 | 24631157 | Chitin induces IL25, IL33, and TSLP which are required to stimulate ILC2 production of IL5 and IL13. IL5 and IL13, in turn, are required for the accumulation of eosinophils and alternatively activated macrophages that are associated with allergy. |
| 162540 | Il25 | 26847546 | Tuft cells express Il25 and elicit group 2 innate lymphoid cells in a Trpm5-dependent manner in response to parasite colonization. |
| 169968 | Rorc | 24419270 | Gata3 plays a generalized role in innate lymphoid cell (ILC) lineage determination and is critical for the development of gut Rorc+ group 3 ILCs subsets that maintain mucosal barrier homeostasis. |
| 169968 | Rorc | 24670648 | Retinoic acid (RA) induces the maturation of lymphoid tissue inducer cells in developing lymph nodes by inducing RA receptor binding to the promoter region of Rorc. |
| 169968 | Rorc | 26878233 | Rorc is differentially required in the maintenance of TH17 cell and group 3 innate lymphoid cell responses. |

| | | | |
|---|---|---|---|
| 186803 | Mb21d1 | 25642965 | Aberrant mitochondrial DNA (mtDNA) packaging promotes escape of mtDNA into the cytosol, where it engages the DNA sensor Mb21d1 and promotes Tmem173-Irf3-dependent signalling to elevate IFN-stimulated gene expression, potentiate type I IFN responses and confer broad viral resistance. |
| 186803 | Mb21d1 | 26944200 | The cationic polymer and vaccine adjuvant chitosan can engage the Tmem173/Mb21d1 (STING/cGAS) pathway to trigger innate and adaptive immune responses. |
| 137341 | Tmem173 | 21820332 | Tmem173 is involved in the innate immune recognition of Plasmodium falciparum AT-rich DNA and in the subsequent induction of type I IFNs. |
| 137341 | Tmem173 | 22000020 | Tmem173 activates Stat6 during viral infection to induce genes responsible for immune cell homing. |
| 137341 | Tmem173 | 23055924 | Dengue viral NS2B3 protease complex cannot degrade murine TMEM173, which confers protection against the viral infection. |
| 137341 | Tmem173 | 24119841 | Cyclic dinucleotides initiate the production of Tmem173(STING)-dependent proinflammatory genes and a negative-feedback to prevent sustained production that may otherwise lead to inflammation. |
| 137341 | Tmem173 | 24131791 | Cyclic-di-GMP-induced levels of Ifi202b suppress the expression of Tmem173 (STING). |
| 137341 | Tmem173 | 25517615 | The innate immune system plays a role in immunogenic tumour recognition. Tumor-cell-derived DNA triggers Ifnb1 production and dendritic cell activation via Tmem173 and Irf3 cytosolic DNA sensing pathways. |
| 137341 | Tmem173 | 25692705 | Unrepaired DNA lesions induce type I interferons via the Tmem173 pathway, resulting in enhanced anti-viral and anti-bacterial responses in Atm (-/-) mice. |
| 137341 | Tmem173 | 25646421 | Tmem173-deficient macrophages fail to express negative regulators of immune activation and are hyperresponsive to TLR ligands, producing abnormally high levels of proinflammatory cytokines. |
| 137341 | Tmem173 | 25642965 | Aberrant mitochondrial DNA (mtDNA) packaging promotes escape of mtDNA into the cytosol, where it engages the DNA sensor Mb21d1 and promotes Tmem173-Irf3-dependent signalling to elevate IFN-stimulated gene expression, potentiate type I IFN responses and confer broad viral resistance. |
| 137341 | Tmem173 | 26590319 | DNA vaccine-induced, Irf7-dependent signalling, as part of the Tmem173 (Sting) pathway, is critical for generation of both innate cytokine signalling and antigen-specific B and T cell responses. |
| 137341 | Tmem173 | 26944200 | The cationic polymer and vaccine adjuvant chitosan can engage the Tmem173/Mb21d1 (STING/cGAS) pathway to trigger innate and adaptive immune responses. |

| | | | |
|---|---|---|---|
| 106840 | TLR5 | 15069060 | TLR5 engagement with its ligand, flagellin, results in the activation of MAP kinases (ERK1/2, JNK, and p38) and degradation of NFKBIA (I-kappa-B-alpha). |
| 106840 | TLR5 | 17283206 | Toll-like receptor 5 (TLR5) is the TLR activated by bacterial flagellin and TLR5 residue 268 is responsible for human and mouse discrimination between flagellin molecules. |
| 106840 | TLR5 | 11323673 | TLR5 recognizes bacterial flagellin from both Gram-positive and Gram-negative bacteria, and its activation mobilizes the nuclear factor NF-kappaB and stimulates tumour necrosis factor-alpha (TNF) production. |
| 106840 | TLR5 | 11489966 | TLR5 expressed on the basolateral surface of intestinal epithelia mediates innate immune inflammatory responses to Salmonella by inducing epithelial pro-inflammatory gene expression. |
| 106840 | TLR5 | 12794153 | TLR5 forms heteromeric complexes with TLR4 as well as homomeric complexes. Signaling via heteromeric TLR5/TLR4 complexes is involved in the induction of macrophage nitric oxide (NO) production by Gram-negative flagellin. |
| 106840 | TLR5 | 22434932 | TLR5 forms a dimer upon binding to bacterial flagellin. |
| 106840 | TLR5 | 22545147 | TLR5 is highly expressed in mucosal dendritic cells and TLR5 signalling restricts regulatory T cell generation. (Demonstrated in mice) |
| 106840 | TLR5 | 22863420 | TLR5 deficiency in mice leads to a transient inability to manage proteobacteria which promotes chronic gut inflammation. (Demonstrated in mouse) |
| 106840 | TLR5 | 26950764 | CXCR1 regulates anti-Pseudomonas neutrophil responses through modulation of reactive oxygen species and interference with TLR5 expression. |
| 80821 | CXCR1 | 26950764 | CXCR1 regulates anti-Pseudomonas neutrophil responses through modulation of reactive oxygen species and interference with TLR5 expression. |
| 195602 | Stat6 | 22000020 | Stat6 is phosphorylated upon viral infection and translocates to the nucleus to induce genes responsible for immune cell homing. |
| 195602 | Stat6 | 26953325 | Stat6 signalling negatively regulates Î³Î´17 T cells, which plays a front-line role in mucosal immunity. |
| 212845 | Dhcr7 | 26938778 | Mir342 is involved in a novel post-transcriptional viral defence mechanism in IFN-activated macrophages by directly targeting Srebf2, Idi1, Dhcr7 and Sc4mol of the sterol pathway. |
| 158311 | Sc4mol | 26938778 | Mir342 is involved in a novel post-transcriptional viral defence mechanism in IFN-activated macrophages by directly targeting Srebf2, Idi1, Dhcr7 and Sc4mol of the sterol pathway. |

| | | | |
|---|---|---|---|
| 129163 | Idi1 | 26938778 | Mir342 is involved in a novel post-transcriptional viral defence mechanism in IFN-activated macrophages by directly targeting Srebf2, Idi1, Dhcr7 and Sc4mol of the sterol pathway. |
| 166203 | Srebf2 | 21408089 | Srebf2 is a key transcriptional regulator of sterol biosynthesis in lipid metabolism, and Srebp2 protein levels in macrophages are negatively regulated by type I interferon signalling during viral infection. |
| 166203 | Srebf2 | 26938778 | Mir342 is involved in a novel post-transcriptional viral defence mechanism in IFN-activated macrophages by directly targeting Srebf2, Idi1, Dhcr7 and Sc4mol of the sterol pathway. |
| 221078 | Mir342 | 26938778 | Mir342 is involved in a novel post-transcriptional viral defence mechanism in IFN-activated macrophages by directly targeting Srebf2, Idi1, Dhcr7 and Sc4mol of the sterol pathway. |
| 158292 | Tgfb1 | 26588780 | Epithelial cell-derived cytokine Tgfb1 has a central role in the generation of the pulmonary immune response by enhancing the chemoactivity of type 2 innate lymphoid cells. |
| 174662 | Sod1 | 26588782 | Sod1 is essential in protecting hepatocytes from virus-induced, interferon-driven oxidative damage in the liver. |
| 7247 | LGALS3 | 18825751 | LGALS3 exerts a regulatory role in innate immunity by diminishing IL-1beta production and thus affecting resistance to Rhodococcus equi infection. |
| 7247 | LGALS3 | 19951367 | LGALS3, an abundant protein in macrophages and epithelial cells, belongs to a family of beta-galactoside-binding proteins, the galectins, with many proposed functions in immune response, development, differentiation, cancer and infection. |
| 7247 | LGALS3 | 20208507 | LGALS3 is part of the galectin family of proteins that have emerged as autonomous bacteria-killing agents, pointing to a principal role of these proteins in innate immunity. |
| 7247 | LGALS3 | 22486577 | LGALS3 influences the course of malaria in a Plasmodium species-specific manner. (Demonstrated in mice) |
| 7247 | LGALS3 | 26589797 | LGALS1 and LGALS3 play opposing roles in the inflammatory responses to Trichomonas vaginalis infection. |
| 6929 | LGALS1 | 19561030 | LGALS1, the prototype of a family of beta-galactoside-binding proteins, is involved in monocyte chemoattraction at sites of inflammation where it stimulates monocyte migration in a dose-dependent manner via the p44/42 MAP kinase pathway. |
| 6929 | LGALS1 | 26589797 | LGALS1 and LGALS3 play opposing roles in the inflammatory responses to Trichomonas vaginalis infection. |

| | | | |
|---|---|---|---|
| 205873 | Aim2 | 20401524 | Aim2 is uniquely involved in sensing infection with the intracellular bacteria Francisella tularensis and subsequently triggering caspase-1-mediated pro-inflammatory cytokine production and macrophage cell death, which activate other components of the immune system and eliminate the infected macrophages. |
| 205873 | Aim2 | 20457908 | Aim2 is required for innate immune recognition of Francisella tularensis where Aim2-deficient mice display an increased susceptibility to F. tularensis infection compared with wild-type mice. |
| 205873 | Aim2 | 21562230 | Aim2-containing inflammasomes are activated in response to cytosolic DNA; this response is augmented in keratinocytes from psoriatic lesions and contributes to the auto-inflammatory disease. (Demonstrated in human) |
| 205873 | Aim2 | 26590313 | During influenza A virus infection, host-derived DNA accumulates in the lung microenvironment and is sensed by Aim2, which limits immune-mediated damage to infected tissues. |
| 212081 | Irf7 | 21820332 | Irf7 is involved in the innate immune recognition of Plasmodium falciparum AT-rich DNA and in the subsequent induction of type I IFNs. Mice lacking Irf3/Irf7 are resistant to otherwise lethal cerebral malaria. |
| 212081 | Irf7 | 24362933 | Plasmodium RNA is a pathogen-associated molecular pattern (PAMP) capable of activating a type I IFN response via the cytosolic pattern recognition receptors Ifih1 and Mavs, as well as via transcription factors Irf3 and Irf7. |
| 212081 | Irf7 | 25531441 | Genetic deletion of Eif4ebp1 or Eif4ebp2 potentiates innate antiviral immunity by enhancing translation of Irf7. |
| 212081 | Irf7 | 26590319 | DNA vaccine-induced, Irf7-dependent signalling, as part of the Tmem173 (Sting) pathway, is critical for generation of both innate cytokine signalling and antigen-specific B and T cell responses. |
| 164788 | Cxcr3 | 21518789 | Cxcr3 expression on recruited peritoneal macrophages and granulocytes increases following sepsis, and deletion of Cxcr3 significantly increases mortality to a septic challenge in neonatal mice. |

| | | | |
|---|---|---|---|
| 164788 | Cxcr3 | 24130498 | Inflammatory monocyte activation status, as measured by dual production of TNF-alpha and IL-12, was severely impaired in Cxcr3(-/-) mice. Deletion of Cxcr3 in mice resulted in selective loss of ability to control T. gondii infection specifically in the lamina propria compartment. |
| 164788 | Cxcr3 | 25466888 | Cxcr3 expression in innate CD8+ T cells defines protective antibacterial and cancer immunity upon Il15 stimulation. |
| 164788 | Cxcr3 | 26595890 | A Cxcr3-dependent innate antiviral pathway operates at epithelial surfaces to induce chemokines and neutrophil activity prior to the induction of interferons. |
| 204791 | Cd48 | 26564814 | Invariant natural killer T cell activation induced by mast cells exposed to alpha-galactosylceramide is regulated by costimulatory molecules Cd48 and Tnfsf4. |
| 201525 | Tnfsf4 | 26564814 | Invariant natural killer T cell activation induced by mast cells exposed to alpha-galactosylceramide is regulated by costimulatory molecules Cd48 and Tnfsf4. |
| 186402 | Clec4e | 21267996 | Clec4e, a C-type lectin receptor, is a pattern recognition receptor critical for immune responses to fungi. Clec4e is coupled to Syk kinase and signals via Card9 to activate NFKB, which in turns induces both innate and adaptive immunity. |
| 186402 | Clec4e | 24212132 | The expression of Clec4e (Mincle) and its downstream signal phospho-Syk/Syk increases after cerebral ischemia and reperfusion. |
| 186402 | Clec4e | 26558717 | Clec4d is an inducible myeloid-expressed C-type lectin receptor, whose expression is tightly linked to that of Clec4e. |
| 186345 | Clec4d | 26558717 | Clec4d is an inducible myeloid-expressed C-type lectin receptor, whose expression is tightly linked to that of Clec4e. |
| 164802 | Mtap1s | 26565030 | Map1s (Mtap1s) controls bacterial phagocytosis through TLR signalling by interacting directly with Myd88. |
| 205936 | Il1b | 21170027 | Il1b acts as a growth factor for neutrophil progenitors and as a survival factor for mature neutrophils. In the absence of Ikbkb, the Il1b production is enhanced and provides a compensatory mechanism for maintaining antibacterial defense when NFKB is inhibited. |
| 205936 | Il1b | 21228274 | Il1b secretion in macrophages is regulated by autophagy by two mechanisms; sequestering of pro-Il1b in autophagosome during TLR stimulation, and processing/secretion of Il1b in a Nlrp3- and TRIF-dependent manner. |

| | | | |
|---|---|---|---|
| 205936 | Il1b | 21602824 | Il1b secretion is induced only during viable E. coli infection (as oppose to heat-killed E. coli or LPS), viable bacteria specifically elicit cleavage of pro-Il1b. |
| 205936 | Il1b | 21270399 | Il1b derived from alveolar macrophages is the critical mediator which induces chemokine production in nonhematopoietic cells in the lung, resulting in swift and robust recruitment of infection-controlling neutrophils into the airways. |
| 205936 | Il1b | 21628463 | Il1b secretion is tightly regulated by the redox status in myeloid cells. TLR engagement in monocytes induces ROS generation followed by a sustained antioxidant response and efficient Il1b secretion. In macrophages, the antioxidant systems are in an upregulated state, and therefore buffers the TLR induction of the redox response, which results in low Il1b processing and secretion. (Demonstrated in human) |
| 205936 | Il1b | 22426547 | Il1b is an inflammatory cytokine that binds to its primary receptor, Il1r1, that then recruits the accessory protein Il1rap to form a signalling-competent heterotrimeric complex. (Demonstrated in human) |
| 205936 | Il1b | 24022484 | Under acidic conditions both pro-inflammatory forms of Il1a and Il1b are regulated independently of the NLRP3 inflammasome. |
| 205936 | Il1b | 24692555 | Group B streptococcus induces Il1b, and activates the NLRP3 inflammasome by a mechanism that requires hemolysin-mediated lysosomal leakage, which enhances the interaction of bacterial RNA with NLRP3. |
| 205936 | Il1b | 25422455 | Actin polymerization is required for Nlrc4-dependent regulation of intracellular bacterial burden, inflammasome assembly, pyroptosis, and Il1b production. |
| 205936 | Il1b | 25483963 | Autophagy causes PELI3 degradation during Tlr4-signalling, subsequently inhibiting Il1b expression and impairing the hyperinflammatory phase during sepsis. |
| 205936 | Il1b | 25689249 | Activation of the Nlrp3 inflammasome is detrimental during leishmaniasis. Mice lacking the inflammasome components Nlrp3, Pycard, Casp1 exhibit defective Il1b and Il18 production at the infection site and are resistant to cutaneous Leishmania major infection. |
| 205936 | Il1b | 25781937 | Escherichia coli toxin CNF1 promotes the maturation/secretion of Il1b while the Î±-hemolysin toxin inhibits Il1b secretion without affecting the recruitment of Ly6g+ cells. |
| 205936 | Il1b | 25683052 | Mirlet7f and its target Tnfaip3 regulate immune responses to Mycobacterium tuberculosis and control bacterial burden by augmenting the production of Tnf and Il1b. |

| | | | |
|---|---|---|---|
| 205936 | Il1b | 25595775 | Defb1 is important for the control of early mucosal Candida infection and plays a critical role in the induction of innate inflammatory mediators including, Il1b, Il6, Cxcl1, Il17a, and Il17f. |
| 205936 | Il1b | 26056255 | Type 1 regulatory (Tr1) cells suppress Il1b transcription and Casp1 activation via an IL10R â€ dependent mechanism. |
| 205936 | Il1b | 26536497 | NLRP3 inflammasome formation is dispensable for alum-induced innate immunity but Il1a and Il1b are both necessary for alum-induced neutrophil influx in vivo. |
| 163502 | Il18 | 22940097 | Il18 secreted by inflammatory monocytes is critical for the differentiation of CD8(+) T and NK lymphocytes into antimicrobial effector cells. |
| 163502 | Il18 | 24549849 | Both Nlrp3 and Nlrp1a are important regulators of Toxoplasma proliferation and Il18 signaling is required to mediate host resistance to acute toxoplasmosis. |
| 163502 | Il18 | 25395539 | Flagellin induces Tlr5-dependent Il22 production and Nlrc4-dependent Il18 production to promote a protective gene expression program in intestinal epithelial cells and elimination of rotavirus-infected cells. |
| 163502 | Il18 | 25689249 | Activation of the Nlrp3 inflammasome is detrimental during leishmaniasis. Mice lacking the inflammasome components Nlrp3, Pycard, Casp1 exhibit defective Il1b and Il18 production at the infection site and are resistant to cutaneous Leishmania major infection. |
| 163502 | Il18 | 25680273 | Il22 augments the expression of Il18 mRNA and inactive precursor protein (proIL-18) in intestinal epithelial cells after Toxoplasma gondii or Citrobacter rodentium infection and maintains the homeostatic amount of proIL-18 in the ileum. |
| 163502 | Il18 | 26638072 | Microbiota-associated metabolites modulate Nlrp6 inflammasome signalling, epithelial Il18 secretion, and anti-microbial pathways. |
| 163502 | Il18 | 26638073 | Colitis severity is controlled at the level of Il18 signalling in intestinal epithelial cells. |
| 211907 | Nlrp6 | 22763455 | Nlrp6 is a negative regulator of inflammatory signalling and impedes the clearance of both Gram-positive and -negative bacterial pathogens. |
| 211907 | Nlrp6 | 26494172 | Nlrp6 functions with Dhx15 as a viral RNA sensor to induce IFN-stimulated genes, and this effect is especially important in the intestinal tract. |
| 211907 | Nlrp6 | 26638072 | Microbiota-associated metabolites modulate Nlrp6 inflammasome signalling, epithelial Il18 secretion, and anti-microbial pathways. |

| | | | |
|---|---|---|---|
| 148568 | Atg5 | 17921696 | Atg12::Atg5 conjugate is a key regulator of the autophagic process to eliminate pathogens such as Streptococcus, M. tuberculosis, Listeria, and herpesvirus. Atg12::Atg5 also associates with components of the RIG-I pathway to negatively regulate type I IFN response and promote RNA virus replication. |
| 148568 | Atg5 | 25650776 | Atg5 knockout mice develop systemic and hepatic inflammation with high-fat diet and low-dose lipolysaccharide treatment. |
| 148568 | Atg5 | 26649827 | Atg5 plays a unique role in protection against M. tuberculosis by preventing polymorphic mononuclear cell (PMN)-mediated immunopathology. Loss of Atg5 in PMNs can cause susceptibility to M. tuberculosis. |
| 106270 | IL10 | 20154735 | IL10 expression is regulated in different immune cells has revealed some of the molecular mechanisms involved at the levels of signal transduction, epigenetics, transcription factor binding and gene activation. |
| 106270 | IL10 | 20435894 | IL10 is a potent anti-inflammatory cytokine that is crucial for down-regulating pro-inflammatory genes which are induced by Toll-like Receptor (TLR) signalling. It also plays a role in microRNA function, specifically its inhibitory effect on miR-155 expression in response to LPS. |
| 106270 | IL10 | 20883317 | IL10 is a pleiotropic cytokine released in many tissues that mediates anti-inflammatory effects. IL10 also has an immunomodulatory role by stimulating NKG2D ligand expression on macrophages, thereby rendering them susceptible to natural killer (NK) cell elimination. |
| 106270 | IL10 | 21849677 | IL10 contributes to antiviral innate immunity during acute infection by restricting activation-induced death in natural killer (NK) cells. Blockade of Il10 receptor during acute murine cytomegalovirus (CMV) infection markedly reduced the accumulation of cytotoxic NK cells in the spleen and lung. (Demonstrated in murine model) |
| 106270 | IL10 | 22268692 | IL10 has opposing functions in anti-microbial responses in its capacity to mediate protective immunity against some organisms but increase susceptibility to other infections. |
| 106270 | IL10 | 22876184 | IL10-mediated suppression of natural killer/dendritic cell crosstalk leads to prolonged mouse cytomegalovirus (MCMV) persistence due to poor priming of MCMV-specific T cells. (Demonstrated in mouse) |
| 106270 | IL10 | 23071313 | IL10 induces MIR187 to limit the expression of pro-inflammatory cytokines. |
| 106270 | IL10 | 23980205 | MIR145 directly targets HDAC11 to promote IL10 expression in TLR4-triggered macrophages. |

| | | | |
|---|---|---|---|
| 106270 | IL10 | 24265436 | HIV-1 infection of macrophages modulates host responses to co-infection with Mycobacterium tuberculosis by attenuating IL10 responses thus contributing to the pathogenesis of tuberculosis in HIV-1â€ infected patients. |
| 106270 | IL10 | 25005359 | Secreted CCNA2 (CCN1) promotes anti-inflammatory cytokine IL10 release from epithelial cells via integrin Î±VÎ²6-PKC, and this subsequently suppresses TNF, CXCL2 and neutrophil infiltration in the lungs. |
| 106270 | IL10 | 26535690 | MIR29A inhibits IL10-induced cytokine release by targeting JAK-STAT3 in monocytes during sepsis. |
| 50702 | STAT3 | 17971840 | STAT3 is a transcription factor that mediates interleukin-10 (IL-10) cytokine signalling. |
| 50702 | STAT3 | 19667404 | STAT3 and SRC have a role in the immunoregulation of dendritic cells (DCs) where apoptotic cells-induced inhibition of dendritic cells (DCs) requires MerTK-dependent activation of SRC and STAT3. |
| 50702 | STAT3 | 20040863 | STAT3 mediates mucosa-protective and anti-inflammatory functions in epithelial and myeloid cells and promotes inflammation in T cells. |
| 50702 | STAT3 | 20493732 | STAT3 binds to multiple genes involved in Th17 cell differentiation, cell activation, proliferation, and survival, regulating both expression, epigenetic modifications, and targets the T cell function in inflammation and homeostasis. |
| 50702 | STAT3 | 20581311 | STAT3 is an essential mediator of emergency granulopoiesis via its regulation of transcription factors CEBPA and CEBPB that direct granulocyte colony-stimulating factor (G-CSF)-responsive myeloid progenitor expansion. |
| 50702 | STAT3 | 21810606 | STAT3 is a negative regulator of type I IFN-mediated anti-viral responses. (Demonstrated in mouse) |
| 50702 | STAT3 | 25510212 | In alveolar epithelium, IL22 upregulates DEFB4A gene expression via STAT3. |
| 50702 | STAT3 | 26535690 | MIR29A inhibits IL10-induced cytokine release by targeting JAK-STAT3 in monocytes during sepsis. |
| 771638 | MIR29A | 26535690 | MIR29A inhibits IL10-induced cytokine release by targeting JAK-STAT3 in monocytes during sepsis. |
| 204141 | Trim12c | 26503954 | Trim12c interacts with Traf6, a key protein in pathogen recognition receptor signalling, and reciprocally enhances its ubiquitination, leading to cooperative activation of IFN and NF-kB pathways. |
| 3941 | MX1 | 18062906 | MX1 is an interferon-induced member of the dynamin superfamily of large GTPases, which inhibit a wide range of viruses by blocking an early stage of the replication cycle. |
| 3941 | MX1 | 20538602 | MX1 GTPase is a key mediator of cell-autonomous innate immunity against a broad range of viruses such as influenza and bunyaviruses. |
| 3941 | MX1 | 22531919 | MX1 expression is upregulated by alpha-defensins in gigival epithelial cell. |

| | | | |
|---|---|---|---|
| 3941 | MX1 | 24448803 | Antiviral specificity of MX1 against orthomyxoviruses (influenza A and Thogoto viruses) is determined by a few critical amino acids in the disordered loop L4 of MX1. |
| 3941 | MX1 | 25525793 | NRAV, a long noncoding RNA, modulates antiviral responses by negatively regulating the initial transcription of multiple critical interferon-stimulated genes, including IFITM3 and MX1, by affecting their histone modification. |
| 3941 | MX1 | 26507657 | MX1 prevents influenza A virus (IAV) replication by disassembling into dimers and binding to IAV nucleoprotein synthesized following primary transcription. |
| 128365 | Lyn | 20385881 | Lyn/PI3K module negatively regulates activation of murine macrophages while Inpp5d (SHIP-1) promotes it. |
| 128365 | Lyn | 22491248 | Overexpression of Lyn results in endotoxin hypersensitivity due to the increased activation of dendritic cells leading to an over-production of Ifng by natural killer cells. |
| 128365 | Lyn | 26517880 | Lyn regulates the macrophage basal-state signalling checkpoint, and the signalling reorganization initiated by receptor clustering allows cells to discriminate optimally between pathogens and nonpathogens. |
| 163641 | Dhx15 | 26494172 | Nlrp6 functions with Dhx15 as a viral RNA sensor to induce IFN-stimulated genes, and this effect is especially important in the intestinal tract. |
| 136575 | Il17a | 21822258 | Il17a signalling enhances the mRNA stability of chemokine Cxcl1 through Traf3ip2, Traf2-Traf5 and the RNA-binding protein Srsf1. |
| 136575 | Il17a | 22384827 | Il17a is significantly upregulated in both S. pyogenes inoculated and mock inoculated mice, indicating that the cytokine production can be triggered by inoculation trauma alone. |
| 136575 | Il17a | 23295184 | IL17A inhibits the release of IL23 during pulmonary inflammation and serves as part of negative feedback loop to control antibacterial immunity. |
| 136575 | Il17a | 24337746 | Il17a is an intrinsic regulator in coordinating neutrophil and macrophage antimicrobial activity to provide protection against acute pneumonic plague. |
| 136575 | Il17a | 24670684 | In mouse model of leukocyte adhesion deficiency type I disease, defective neutrophil recruitment is associated with dysregulated local overproduction of IL17, which drives inflammatory periodontal bone loss. |
| 136575 | Il17a | 25595775 | Defb1 is important for the control of early mucosal Candida infection and plays a critical role in the induction of innate inflammatory mediators including, Il1b, Il6, Cxcl1, Il17a, and Il17f. |
| 136575 | Il17a | 26488187 | Il17a is rapidly produced during lung injury and significantly contributes to early immunopathogenesis, a process that is orchestrated largely by a distinct population of pulmonary type 3 innate lymphoid cells. |

| | | | |
|---|---|---|---|
| 213476 | Ern1 | 22883233 | Hyperactivated Ern1 (IRE1Î±) increases Txnip mRNA stability by reducing levels of a Txnip destabilizing microRNA, miR-17. In turn, elevated Txnip protein activates the Nlrp3 inflammasome, causing procaspase-1 cleavage and interleukin 1 Î² (IL-1 Î²) secretion. (Demonstrated in human) |
| 213476 | Ern1 | 26173697 | ERN1-mediated persistent reactive oxygen species generation is a mechanism used by macrophages to kill bacterial pathogens that evade the initial oxidative burst. |
| 83167 | C5 | 18039528 | C5 is the fifth component of complement and is comprised of alpha (C5a) and beta (C5b) polypeptide chains, C5a is a complement anaphylatoxin that can stimulate the generation of nitric oxide along with the secretion of histamine and leukotriene LTC4 from several cell types and can bring about an increase in vascular permeability that facilitates eosinophil accumulation at sites of allergic inflammation. |
| 83167 | C5 | 10441679 | C5 is the fifth component of complement and is comprised of alpha (C5a) and beta (C5b) polypeptide chains. C5b first associates with complement component 6 (C6), which in turn initiates the assembly of the cytolytic membrane attack complex (MAC). |
| 83167 | C5 | 21063021 | C5 fragments are released from neutrophils upon their activation of the alternative complement pathway and this further amplifies neutrophil pro-inflammatory responses. |
| 83167 | C5 | 25539819 | C5 (C5a) is a critical mediator in blood during Candida albicans infection. |
| 83167 | C5 | 26176669 | Staphylococcus aureus-induced neutrophil dysfunction correlates with the loss of C5AR1 from the neutrophil cell surface and results in C5a (C5)-induced CEACAM8 overexpression. |
| 54589 | CEACAM8 | 26176669 | Staphylococcus aureus-induced neutrophil dysfunction correlates with the loss of C5AR1 from the neutrophil cell surface and results in C5a (C5)-induced CEACAM8 overexpression. |
| 59445 | C5AR1 | 20159852 | C5AR1 engage in crosstalk with TLR2 and Porphyromonas gingivalis, a major oral and systemic pathogen with complement C5 convertase-like activity, synergizes with C5a (fragment of complement protein C5) to increase cyclic adenosine monophosphate (cAMP) concentrations, resulting in suppression of macrophage immune function and enhanced pathogen survival. |

| | | | |
|---|---|---|---|
| 59445 | C5AR1 | 20457757 | C5AR1 is a receptor for C5a, a chief component of complement activation produced via all three complement pathways (i.e., lectin, classical, and alternative), stimulated tissue-resident macrophages, but not dendritic cells, to produce inflammatory cytokines including IL-6, in synergy with Toll-like receptor signalling or, notably, granulocyte/macrophage colony-stimulating factor (GM-CSF). |
| 59445 | C5AR1 | 23479227 | Increasing circulating C5AR1 serum concentration, coupled with decreased C5AR1 expression on neutrophils is correlated with lethality from septic shock. |
| 59445 | C5AR1 | 26176669 | Staphylococcus aureus-induced neutrophil dysfunction correlates with the loss of C5AR1 from the neutrophil cell surface and results in C5a (C5)-induced CEACAM8 overexpression. |
| 213369 | Zbp1 | 22423968 | Zbp1 interacts with Ripk3 to mediate virus-induced necrosis. |
| 213369 | Zbp1 | 26146945 | Ddx58 acts in parallel with Zbp1 in an RNA polymerase III-dependent manner to initiate glial responses to herpes simplex virus-1. |
| 197593 | Hs2st1 | 26150541 | Inactivation of Hs2st1 in neutrophils substantially reduces their bactericidal activity, and Hs2st1-deficient mice are more susceptible to systemic infection with the pathogenic bacterium group B Streptococcus. |
| 163345 | Zfp36 | 26019272 | Dusp1 modulates the phosphorylation status of mRNA-destabilizing protein Zfp36 to regulate macrophage immune response to lipopolysaccharide. |
| 155234 | Dusp1 | 21959016 | Dusp1 is a negative regulator of MAPK-dependent induction of Il6 and Il8 in response to the coronavirus infectious bronchitis virus (IBV). (Demonstrated in human) |
| 155234 | Dusp1 | 22464096 | Dusp1 antagonizes p38 MAPK activity to induce Il12b expression, and may play a role in the development of Th1 type immune response and anti-microbial defence. |
| 155234 | Dusp1 | 26019272 | Dusp1 modulates the phosphorylation status of mRNA-destabilizing protein Zfp36 to regulate macrophage immune response to lipopolysaccharide. |
| 189999 | Cxcr4 | 26026270 | Bacterial cell wall glucosaminyl-muramyl dipeptides (GMDPs) bind to the transcription factor Ybx1 to upregulate Nfkb2 and Cxcr4 gene expression. |
| 172544 | Nfkb2 | 26026270 | Bacterial cell wall glucosaminyl-muramyl dipeptides (GMDPs) bind to the transcription factor Ybx1 to upregulate Nfkb2 and Cxcr4 gene expression. |
| 182925 | Ybx1 | 25398005 | Ybx1 controls intracellular Il6 mRNA levels in a cell type-specific manner, leading to functions that are dependent on the extracellular and intracellular distribution of Ybx1. |
| 182925 | Ybx1 | 26026270 | Bacterial cell wall glucosaminyl-muramyl dipeptides (GMDPs) bind to the transcription factor Ybx1 to upregulate Nfkb2 and Cxcr4 gene expression. |

| | | | |
|---|---|---|---|
| 75571 | SYK | 26032420 | Antibody-dependent enhancement (ADE) of Dengue virus serotype 2 (DENV-2) elevates mature IL1B secretion via SYK signalling pathway in primary monocytes. |
| 130583 | Il7 | 26034215 | Il7 is produced by intestinal epithelial cells in response to Citrobacter rodentium infection and plays a critical role in the protective immunity against this intestinal attaching and effacing bacterium. |
| 259066 | Tlr5 | 22434932 | Tlr5 forms a dimer upon binding to bacterial flagellin. (Demonstrated in human) |
| 259066 | Tlr5 | 22545147 | Tlr5 is highly expressed in mucosal dendritic cells and Tlr5 signalling restricts regulatory T cell generation. |
| 259066 | Tlr5 | 22863420 | Tlr5 deficiency in mice leads to a transient inability to manage proteobacteria which promotes chronic gut inflammation. |
| 259066 | Tlr5 | 24442437 | Flagellin-specific IgG1 antibody response is induced through a Tlr5-, inflammasome-, and Myd88-independent pathway. |
| 259066 | Tlr5 | 25395539 | Flagellin induces Tlr5-dependent Il22 production and Nlrc4-dependent Il18 production to promote a protective gene expression program in intestinal epithelial cells and elimination of rotavirus-infected cells. |
| 259066 | Tlr5 | 26003491 | Indirect Tlr5-dependent stimulation of airway conventional dendritic cells is essential to flagellin's mucosal adjuvant activity. |
| 17225 | IRF7 | 19152337 | IRF7 increases the expression of a broad range of IFN-stimulated genes including immunomodulatory cytokines and genes involved in antigen processing and presentation. |
| 17225 | IRF7 | 15664995 | IRF7 is activated in response to virus infection and stimulates the transcription of a set of cellular genes involved in host antiviral defence. |
| 17225 | IRF7 | 15361868 | IRF7 forms a complex with MYD88 and TRAF6 and this complex formation, as well as TRAF6-dependent IRF7 ubiquitination, is required for TLR-mediated interferon (IFN)-alpha induction. |
| 17225 | IRF7 | 21820332 | IRF7 is involved in the innate immune recognition of Plasmodium falciparum AT-rich DNA and in the subsequent induction of type I IFNs. Mice lacking Irf3/Irf7 are resistant to otherwise lethal cerebral malaria. (Demonstrated in mouse) |
| 17225 | IRF7 | 25505178 | Coronavirus engages papain-like proteases to escape from the innate antiviral response of the host by inhibiting TP53-IRF7-IFNB1 signalling. |
| 17225 | IRF7 | 25520509 | Paramyxoviruses trigger the DNA-damage response, a pathway required for RPS6KA5 activation of phospho Ser 276 RELA formation to trigger the IRF7-DDX58 amplification loop necessary for mucosal interferon production. |
| 17225 | IRF7 | 25911105 | AIP is a novel inhibitor of IRF7 and a negative regulator of innate antiviral signalling. |

| | | | |
|---|---|---|---|
| 60515 | AIP | 25911105 | AIP is a novel inhibitor of IRF7 and a negative regulator of innate antiviral signalling. |
| 172144 | Ager | 22386596 | Ager is a native receptor for complement component C1qa. (Demonstrated in human) |
| 172144 | Ager | 25911757 | S100a8/S100a9 are proinflammatory proteins that activate natural killer cells via Ager signalling. |
| 168119 | S100a9 | 21239714 | S100a9 forms a complex with S100a8 and the complex is the site of interplay between extracellular Ca(2+) entry and intra-phagosomal reactive oxygen species production. S100a8 :: S100a9 acts as Ca(2+) sensor in phagosomal ROS production. |
| 168119 | S100a9 | 21382888 | S100a9-deficient murine neutrophils exhibited a reduce secretion of cytokines in response to Tlr4 stimulation. In contrast, S100a9-deficient dendritic cells showed an exacerbated release of cytokines after TLR stimulation. S100a9 has no effect on the inflammatory status of macrophages. |
| 168119 | S100a9 | 22423963 | S100a9 is strongly upregulated in neutrophils upon bacterial infection, and sequesters zinc as a mechanism of nutritional immunity. Salmonella typhimurium overcomes this defence mechanism by expressing a high affinity zinc transporter. |
| 168119 | S100a9 | 23133376 | S100A9 forms a heterodimer with S100A8 and is a key player in protective innate immunity during Klebsiella pneumonia infection. |
| 168119 | S100a9 | 25911757 | S100a8/S100a9 are proinflammatory proteins that activate natural killer cells via Ager signalling. |
| 168077 | S100a8 | 21239714 | S100a8 forms a complex with S100a9 and the complex is the site of interplay between extracellular Ca(2+) entry and intra-phagosomal reactive oxygen species production. S100A8 :: S100A9 acts as Ca(2+) sensor in phagosomal ROS production. |
| 168077 | S100a8 | 22423963 | S100a8 is strongly upregulated in neutrophils upon bacterial infection, and sequesters zinc as a mechanism of nutritional immunity. Salmonella typhimurium overcomes this defence mechanism by expressing a high affinity zinc transporter. |
| 168077 | S100a8 | 23133376 | S100A8 forms a heterodimer with S100A9 and is a key player in protective innate immunity during Klebsiella pneumonia infection. |
| 168077 | S100a8 | 25911757 | S100a8/S100a9 are proinflammatory proteins that activate natural killer cells via Ager signalling. |
| 161461 | Lcp2 | 25929249 | Lcp2 is a critical determinant of natural killer (NK)-cell development and NK cell mediated elimination of missing-self target cells. |
| 145267 | Ticam2 | 21494017 | Ticam2 deficiency results in the impairment of LPS-stimulated TNF-alpha protein translation. |
| 145267 | Ticam2 | 25505250 | Adaptor proteins Ticam1 and Ticam2 have a novel function in Tlr2-mediated signal transduction. |

| | | | |
|---|---|---|---|
| 145267 | Ticam2 | 26065469 | Ticam2 mediates antibacterial defence during Gram-negative pneumonia by inducing Ifng at the primary site of infection. |
| 145267 | Ticam2 | 25808990 | Intracellular Sef/IL-17R (SEFIR) domain of Il17rd targets TIR adaptor proteins Myd88, Tirap, Ticam1, Ticam2 and Traf6 to inhibit TLR downstream signalling. |
| 148134 | Tirap | 21705416 | Tirap Ser180Leu polymorphism is significantly associated with Behcet's disease in UK, but not Middle Eastern, patients. It is suggested that the Ser180Leu functional variant of Tirap will lead to greater cytokine production and tissue damage with persistence of mucosal lesions upon encounter with pathogens. (Demonstrated in human) |
| 148134 | Tirap | 25808990 | Intracellular Sef/IL-17R (SEFIR) domain of Il17rd targets TIR adaptor proteins Myd88, Tirap, Ticam1, Ticam2 and Traf6 to inhibit TLR downstream signalling. |
| 140856 | Il17rd | 25808990 | Intracellular Sef/IL-17R (SEFIR) domain of Il17rd targets TIR adaptor proteins Myd88, Tirap, Ticam1, Ticam2 and Traf6 to inhibit TLR downstream signalling. |
| 21550 | DHX33 | 25816776 | RNA cleavage products, catalyzed by RNASEL, bind to DHX33 to facilitate the formation of a complex with MAVS and NLRP3 during viral infection. |
| 105248 | RNASEL | 19075243 | RNASEL, antiviral endoribonuclease, is the terminal component of an RNA decay pathway that is an important mediator of IFN-induced antiviral activity and is required for the optimal induction of pro-inflammatory cytokines that play essential roles in host defence from bacterial pathogens. |
| 105248 | RNASEL | 19075243 | RNASEL regulates the expression of the endolysosomal protease, cathepsin-E, and endosome-associated activities, that function to eliminate internalized bacteria and may contribute to RNASEL antimicrobial action. |
| 105248 | RNASEL | 20833746 | RNASEL-mediated cleavage of Hepatitis C virus (HCV) RNA generates suppressor of virus RNA (svRNA) that activates DDX58 (RIG-I), thus propagating innate immune signalling to the interferon (IFN)-beta gene. |
| 105248 | RNASEL | 21190483 | RNASEL cleaves RNA during viral infections and the cleavage products induces the RIG-I pathway and production of IFNB gene. In addition, RNASEL is implicated in the protection of central nervous system against viral-induced demyelination. A broader role in innate immunity is suggested by involvement of RNASEL in cytokine induction and endosomal pathways that suppress bacterial infections. |

| | | | |
|---|---|---|---|
| | | | RNA cleavage products, catalyzed by RNASEL, bind to DHX33 to facilitate the formation of a complex with MAVS and NLRP3 during viral infection. |
| 105248 | RNASEL | 25816776 | |
| 11512 | IL32 | 25820174 | IL32 enhances host immunity to Mycobacterium tuberculosis. |
| 24117 | ELAVL1 | 14981256 | ELAVL1 is a protein that binds to specific mRNA subsets, preferentially within 3' untranslated regions, and is a pivotal posttranscriptional regulator of gene expression. |
| 24117 | ELAVL1 | 17534146 | ELAVL1 is an RNA-binding protein can stabilize and/or regulate the translation of target mRNAs, thereby affecting the cellular responses to immune, proliferative, and damaging agents. It has a broad anti-apoptotic function where it increases the stability of a target mRNA encoding the pro-survival deacetylase SIRT1 and promotes the expression of mRNAs encoding BCL2 and MCL1, two major anti-apoptotic effectors. |
| 24117 | ELAVL1 | 25678110 | ELAVL1 is required for the stabilization of IFNB1 mRNA, and suppression of ELAVL1 leads to impaired expression of IFNB1 in response to poly(I:C) treatment. |
| 30697 | CYLD | 18636086 | CYLD is a negative regulator of DDX58(RIG-I)-mediated antiviral response by removing Lys 63-linked polyubiquitin chains from RIG-I and TBK1. |
| 30697 | CYLD | 18643924 | CYLD is a crucial negative regulator of innate immune response in Escherichia coli pneumonia. |
| | | | CYLD is a deubiquitinase that counteracts E3 ligases and therefore play a prominent role in the downregulation of NF-Î°B signalling and homeostasis. |
| 30697 | CYLD | 21119682 | |
| 30697 | CYLD | 21498625 | CYLD is a deubiquitinase that act as a negative regulator of TLR3 induction in response to LPS. |
| 30697 | CYLD | 21946435 | CYLD plays a key role in Type I IFN receptor signalling during vesicular stomatitis virus (VSV) infection. In the absence of CYLD, IFN-beta is ineffective in the induction of antiviral genes. (Demonstrated in mice) |
| 30697 | CYLD | 22057290 | The E3 ligase ITCH and deubiquitinase CYLD act together to regulate TAK1 and inflammation. |
| 30697 | CYLD | 25909817 | MIR362 promotes natural killer-cell function by the down-regulation of CYLD. |
| 126785 | MIR362 | 25909817 | MIR362 promotes natural killer-cell function by the down-regulation of CYLD. |
| 200543 | Uvrag | 25917095 | Mir125a regulates the innate host defense by inhibiting the activation of autophagy and antimicrobial effects against Mycobacterium tuberculosis through targeting Uvrag. |

| | | | |
|---|---|---|---|
| 224267 | Mir125a | 25917095 | Mir125a regulates the innate host defense by inhibiting the activation of autophagy and antimicrobial effects against Mycobacterium tuberculosis through targeting Uvrag. |
| 187635 | Ptpn6 | 25876760 | Bordetella pertussis CyaA toxin plays a role in evading nitric oxide-mediated killing in macrophages through a cAMP-dependent activation of the Ptpn6 phosphatase. |
| 102192 | CTSK | 18762176 | CTSK is a cathepsins, which are key modulators of cell death and inflammatory responses. |
| 102192 | CTSK | 25884905 | Cleavage of CAMP by cathepsins CTSS and CTSK impairs its antimicrobial activity against Pseudomonas aeruginosa and Staphylococcus aureus. |
| 102189 | CTSS | 18762176 | CTSS is a member of the Cathepsins protein family, which are key modulators of cell death and inflammatory responses. |
| 102189 | CTSS | 21145045 | CTSS is an endosomal and lysosomal protease that is upregulated during various inflammatory disorders. TLR2, 3, 4 ligand engagement increases the proteolytic activities of CTSS in macrophages. (Demonstrated in murine model) |
| 102189 | CTSS | 25884905 | Cleavage of CAMP by cathepsins CTSS and CTSK impairs its antimicrobial activity against Pseudomonas aeruginosa and Staphylococcus aureus. |
| 223547 | Mir328 | 25894560 | Mir328 is a key element of the host response to pulmonary infection with non-typeable Haemophilus influenzae and its inhibition in macrophages augments phagocytosis, the production of reactive oxygen species, and microbicidal activity. |
| 126687 | MIR328 | 25894560 | MIR328 is a key element of the host response to pulmonary infection with non-typeable Haemophilus influenzae and its inhibition in macrophages augments phagocytosis, the production of reactive oxygen species, and microbicidal activity. |
| 201591 | Camp | 21448240 | CAMP (LL-37), at sufficiently low concentrations, is able to reduce fungal infectivity by inhibiting C. albicans adhesion to plastic surfaces, oral epidermoid cells, and the urinary bladders of female mice. The inhibitory effects of LL-37 on cell adhesion and aggregation were mediated by its preferential binding to mannan and chitin in the fungal cell wall. (Demonstrated in human) |
| 201591 | Camp | 21464330 | Camp (LL-37) translocates across the E. coli outer membrane and halts bacterial growth by interfering cell wall biogenesis. (Demonstrated in human) |
| 201591 | Camp | 21762664 | Camp protects against colitis induction in mice. The increased expression of Camp in monocytes involves the activation of Tlr9/ERK signalling pathway by bacterial DNA. |
| 201591 | Camp | 21832078 | Camp expression is induced upon endoplasmic reticulum stress via NF-kB-C/EBP-alpha activation. (Demonstrated in human) |

| | | | |
|---|---|---|---|
| 201591 | Camp | 22031815 | mCRAMP (Camp) reduces influenza A viral load and disease severity in mice. |
| 201591 | Camp | 25554785 | The transcription factor Zfp423 is necessary for adipocyte activation and impaired adipogenesis is observed in Zfp423(nur12) mice. |
| 201591 | Camp | 25896094 | Camp plays an important role in the innate immune response against pathogens in bacterial central nervous system infections. |
| 172024 | Il4 | 21566158 | Exogenous Il4 was sufficient to drive the accumulation of tissue macrophages through self-renewal revealing that the expansion of innate cells necessary for pathogen control or wound repair can occur without recruitment of potentially tissue-destructive inflammatory cells. |
| 172024 | Il4 | 23991011 | IL4 attenuates Th1-chemokines expression at the site of inflammation reducing Th1 lymphocyte recruitment and limits pathogen clearance |
| 172024 | Il4 | 25888258 | Nlrp12 is an intrinsic negative regulator of T-cell-mediated immunity. Altered NF-kB regulation and Il4 production are key mediators of Nlrp12-associated disease. |
| 264083 | Nlrp12 | 25888258 | Nlrp12 is an intrinsic negative regulator of T-cell-mediated immunity. Altered NF-kB regulation and Il4 production are key mediators of Nlrp12-associated disease. |
| 6693 | DEFB103B | 20385753 | DEFB103B inhibits cell wall biosynthesis in staphylococci by inhibiting those enzymes which use the bactoprenol bound cell wall building block Lipid II as substrate. |
| 6693 | DEFB103B | 21809339 | DEFB103B exerts anti-inflammatory activities by specifically targeting TLR signalling pathways to transcriptionally repress pro-inflammatory genes. |
| 6693 | DEFB103B | 22922323 | DEFB103B (hBD-3) is sequestered by extracellular DNA in Haemophilus influenzae biofilms to reduce its antimicrobial activity. |
| 6693 | DEFB103B | 22951718 | DEFB103B (hBD3) induces dendritic cell activation, migration and polarization in the skin. |
| 6693 | DEFB103B | 23133681 | DEFB103B (hBD3) is induced in leprosy type 1 reactions in keratinocytes. |
| 6693 | DEFB103B | 25847963 | Exposure to ambient air pollution particulate matter deregulates the ability of the human type II alveolar epithelial cells (A549) to express the antimicrobial peptides HBD-2 (DEFB4A/DEFB4B) and HBD-3 (DEFB103A/DEFB103B) upon infection with Mycobacterium tuberculosis and increases intracellular M. tuberculosis growth. |
| 6300 | DEFB103A | 20385753 | DEFB103A inhibits cell wall biosynthesis in staphylococci by inhibiting those enzymes which use the bactoprenol bound cell wall building block Lipid II as substrate. |

| | | | |
|---|---|---|---|
| 6300 | DEFB103A | 20483750 | DEFB103A is a beta-defensin with direct antimicrobial properties that contribute to local innate immune responses and it aids in combating microbial invasion by being chemotactic for a broad spectrum of leukocytes in a CCR6- and CCR2-dependent manner. |
| 6300 | DEFB103A | 21442129 | DEFB103A expression is inhibited in human epidermal keratinocytes under high glucose conditions, which in turn contributed to the frequent occurrences of infection associated with diabetic wounds. |
| 6300 | DEFB103A | 22922323 | DEFB103A (hBD-3) is sequestered by extracellular DNA in Haemophilus influenzae biofilms to reduce its antimicrobial activity. |
| 6300 | DEFB103A | 22951718 | DEFB103A (hBD3) induces dendritic cell activation, migration and polarization in the skin. |
| 6300 | DEFB103A | 23133681 | DEFB103A (hBD3) is induced in leprosy type 1 reactions in keratinocytes. |
| 6300 | DEFB103A | 25637949 | DEFB103A and RNASE7 are induced in human umbilical endothelial cells (HUVECs) by classical inflammatory cytokines such as: IFNG, IL1B and TNF. |
| 6300 | DEFB103A | 25847963 | Exposure to ambient air pollution particulate matter deregulates the ability of the human type II alveolar epithelial cells (A549) to express the antimicrobial peptides HBD-2 (DEFB4A/DEFB4B) and HBD-3 (DEFB103A/DEFB103B) upon infection with Mycobacterium tuberculosis and increases intracellular M. tuberculosis growth. |
| 6283 | DEFB4B | 25847963 | Exposure to ambient air pollution particulate matter deregulates the ability of the human type II alveolar epithelial cells (A549) to express the antimicrobial peptides HBD-2 (DEFB4A/DEFB4B) and HBD-3 (DEFB103A/DEFB103B) upon infection with Mycobacterium tuberculosis and increases intracellular M. tuberculosis growth. |
| 6711 | DEFB4 | 20483750 | DEFB4 is a beta-defensin with direct antimicrobial properties that contribute to local innate immune responses and it aids in combating microbial invasion by being chemotactic for a broad spectrum of leukocytes in a CCR6- and CCR2-dependent manner. |
| 6711 | DEFB4 | 22427634 | DEFB4A is upregulated in hypoxic microenvironments, which is characteristic of infected tissue. |
| 6711 | DEFB4 | 22500651 | DEFB4A induction in keratinocytes is inhibited by Pseudomonas aeruginosa rhamnolipids. |
| 6711 | DEFB4 | 25510212 | In alveolar epithelium, IL22 upregulates DEFB4A gene expression via STAT3. |

| | | | |
|---|---|---|---|
| 6711 | DEFB4 | 25847963 | Exposure to ambient air pollution particulate matter deregulates the ability of the human type II alveolar epithelial cells (A549) to express the antimicrobial peptides HBD-2 (DEFB4A/DEFB4B) and HBD-3 (DEFB103A/DEFB103B) upon infection with Mycobacterium tuberculosis and increases intracellular M. tuberculosis growth. |
| 158983 | Mapk14 | 21683629 | Mapk14 activation is blocked by Bacillus anthracis, resulting in the opening of a connexin ATP release channel and induction of macrophage death. Constitutive activation of Mapk14 interferes with inflammasome activation and Il1b production, which compromises antimicrobial immunity. |
| 158983 | Mapk14 | 21733175 | The Mapk14 pathway is an important contributor to microglial production of proinflammatory cytokines induced by LPS or beta-amyloid. |
| 158983 | Mapk14 | 22028692 | Mapk14 mediates cytoskeletal remodelling and early spreading of lipopolysaccharide (LPS)-stimulated macrophages. |
| 158983 | Mapk14 | 22447027 | Mapk1 (ERK) and Mapk14 (p38) control the dynamic balance regulating neutrophil migration. (Demonstrated in human) |
| 158983 | Mapk14 | 25783839 | Lysophosphatidic acid plays an anti-inflammatory role in macrophages by diminishing lipopolysaccharide-induced phosphorylation of Mapk14 and Akt1, as well as Rela nuclear translocation. |
| 158983 | Mapk14 | 25848864 | Treml4 is an essential positive regulator of Tlr7 signalling. Treml4(-/-) macrophages are hyporesponsive to Tlr7 agonists and fail to produce type I interferons due to impaired phosphorylation of Stat1 by Mapk14 and decreased recruitment of Myd88 to Tlr7. |
| 189814 | Treml4 | 25848864 | Treml4 is an essential positive regulator of Tlr7 signalling. Treml4(-/-) macrophages are hyporesponsive to Tlr7 agonists and fail to produce type I interferons due to impaired phosphorylation of Stat1 by Mapk14 and decreased recruitment of Myd88 to Tlr7. |
| 44230 | TLR7 | 18071655 | TLR7 recognizes long single-stranded RNA and short double-stranded RNA. |
| 44230 | TLR7 | 15034168 | TLR7 recognizes the single-stranded RNA viruses, vesicular stomatitis virus and influenza virus, resulting in activation of co-stimulatory molecules and production of cytokines. |
| 44230 | TLR7 | 14976261 | TLR7-dependent production of inflammatory cytokines can be induced by single-stranded RNA (ssRNA) molecules of non-viral origin. |
| 44230 | TLR7 | 14579267 | TLR7, 8 and 9 form a functional subgroup within the TLR family that recognizes pathogen-associated molecular patterns in endosomal/lysosomal compartments. |

| | | | |
|---|---|---|---|
| 44230 | TLR7 | 20034855 | TLR7/9-mediated innate immune responses via selected TLR pathways can be negatively regulated by a human microsatellite DNA-mimicking oligodeoxynucleotide with CCT repeats. |
| 44230 | TLR7 | 20007807 | TLR7-dependent innate immune response is induced by free human T-cell leukemia virus 1 (HTLV-1) in killer plasmacytoid dendritic cells, resulting in high production of IFN-alpha and TRAIL relocalization. |
| 44230 | TLR7 | 21037581 | TLR7 is expressed in C-fiber primary sensory neurons and is important for inducing itch (pruritus), but is not necessary for eliciting mechanical, thermal, inflammatory and neuropathic pain. (Demonstrated in murine model) |
| 44230 | TLR7 | 21282509 | TLR7 signalling pathway plays a pivotal role in fungal pathogen recognition and is essential for the subsequent IFNB signalling. (Demonstrated in murine model) |
| 44230 | TLR7 | 21402738 | TLR7 requires proteolytic processing in endolysosome by asparagine endopeptidase and cathepsin in the endolysosome to initiate signalling. (Demonstrated in murine model) |
| 44230 | TLR7 | 21487111 | TLR7 agonists, such as imidazoquinolines, accumulate in the MHC class II loading compartment - this pH-dependent localization is required for the activation of plasmacytoid dendritic cells. |
| 44230 | TLR7 | 21730058 | TLR7 inflammatory signalling leads to cardiac fibrosis in autoimmune associated congenital heart block. |
| 44230 | TLR7 | 21734241 | TLR7 and TLR8 are translocated from the endoplasmic reticulum to the endosome in the presence of antiphospholipid antibodies, as a consequence, plasmacytoid dendritic cells become dramatically sensitized to TLR7/8 agonists and this may play a role in systemic autoimmunity. |
| 44230 | TLR7 | 21998589 | TLR7 is responsible for the detection of retroviruses and serves as a key checkpoint controlling the development of germinal center B cells. (Demonstrated in mice) |
| 44230 | TLR7 | 22396599 | TLR7 signalling induces autophagy in HIV-infected plasmacytoid dendritic cells; this process is necessary for the induction of IFN-alpha. |
| 44230 | TLR7 | 22952664 | Aberrant TLR7 activation induces Epstein-Barr viral protein LMP1 expression, which exacerbates IFN production in lupus patients. |
| 44230 | TLR7 | 26130701 | IFN- Î » 1 is able to augment TLR-mediated B cell activation, partially attributed to an upregulation of TLR7 expression |
| 69650 | SPHK1 | 20634980 | SPHK1 is involved in toll-mediated human beta-defensin 2 (HBD-2) regulation in oral keratinocytes, which also involves the activation of PI3K, AKT, GSK3B (GSK-3beta) and ERK 1/2. |

| | | | |
|---|---|---|---|
| 69650 | SPHK1 | 20661259 | SPHK1 activation, mediated by TLR4, is found to be critical for the redox-dependent activation of HIF-1alpha and ASK1, as well as for the prevention of LPS-induced activation of caspase 3 and the expression of pro-inflammatory cytokine interleukin-6. |
| 69650 | SPHK1 | 26113114 | (S)-methyl 2-(hexanamide)-3-(4-hydroxyphenyl) propanoate (MHP) activates SPHK1, which also stimulates CAMP production and enhances epidermal antimicrobial defence. |
| 153059 | Peli1 | 26131354 | Peli1 is an innate immune regulator in the central nervous system that modulates the threshold of Type I interferon responses against viral infections. |
| 94882 | FCN3 | 20375620 | FCN3, as well as ficolins FCN1 and FCN2, in serum are associated with MBL-associated serine protease (MASP) to form a complex and this complex binds to carbohydrates present on the surface of a variety of Gram-positive and Gram-negative bacteria through ficolin, initiating complement activation via the lectin pathway. |
| 94882 | FCN3 | 26133042 | H-Ficolin (FCN3), an innate immune opsonin, participates in Aspergillus fumigatus defence through the activation of the lectin complement pathway, enhancement of fungus-host interactions and modulation of immune responses. |
| 168471 | Icosl | 26092469 | Group 2 innate lymphoid cell (ILC2)-intrinsic Il33 signalling and Icosl expression promote regulatory T cell accumulation, whereas the inflammatory cytokine Ifng counter-regulated the effects of Il33, in part through direct effects on ILC2s. |
| 194305 | Smpdl3b | 26095358 | The membrane-modulating enzyme SMPDL3B is a negative regulator of TLR signalling that functions at the interface of membrane biology and innate immunity. |
| 205243 | Ctnnb1 | 26100021 | Glycogen synthase kinase 3/Ctnnb1 (Î²-catenin) axis is required for optimal induction of antiviral innate immunity. |
| 156635 | Gsk3a | 26100021 | Glycogen synthase kinase 3/Ctnnb1 (Î²-catenin) axis is required for optimal induction of antiviral innate immunity. |
| 159859 | Gsk3b | 21515258 | Gsk3b is a regulator of LPS-mediated septic shock. Gsk3b deficiency results in the attenuation of endotoxemia. |
| 159859 | Gsk3b | 22218715 | Gsk3b functions downstream of Tlr2-stimulation to induce the expression of the monocyte chemoattractant protein 1, Ccl2. |
| 159859 | Gsk3b | 26100021 | Glycogen synthase kinase 3/Ctnnb1 (Î²-catenin) axis is required for optimal induction of antiviral innate immunity. |
| 27347 | CTNNB1 | 17704137 | CTNNB1 binds to NFKB1 and regulates expression of C-Reactive protein (CRP) after TNF (TNF-alpha) treatment. |

| | | | |
|---|---|---|---|
| 27347 | CTNNB1 | 20453844 | CTNNB1 interacts with LRRFIP1, promoting the activation of CTNNB1, which increases IFN-beta expression by binding to the C-terminal domain of the transcription factor IRF3 and recruiting the acetyltransferase EP300 to the IFN-beta enhanceosome via IRF3. |
| 27347 | CTNNB1 | 23785285 | Stabilization of CTNNB1 upon virus infection negatively regulates antiviral innate immunity. |
| 27347 | CTNNB1 | 26100021 | Glycogen synthase kinase 3/CTNNB1 (Î²-catenin) axis is required for optimal induction of antiviral innate immunity. |
| 51663 | GSK3B | 17912008 | GSK3B is a cytoplasmic serine/threonine protein kinase that regulates NF-kappaB activation and the proliferation and survival of pancreatic cancer cells. |
| 51663 | GSK3B | 17726008 | GSK3B inhibits MEKK4 activity and prevents its activation of JNK and p38, thus controlling MEKK4 dimerization both positively and negatively by regulating its interaction with specific proteins. |
| 51663 | GSK3B | 20497256 | GSK3B activation is accelerated by TLR4 which leads to deterioration of serum-deprivation-induced apoptosis and beta-arrestin 2 represents an inhibitory effect on the TLR4-mediated apoptotic cascade, through controlling the homeostasis of activation and inactivation of GSK3B. |
| 51663 | GSK3B | 21515258 | GSK3B is a regulator of LPS-mediated septic shock. GSK3B deficiency results in the attenuation of endotoxemia. (Demonstrated in murine model) |
| 51663 | GSK3B | 22218715 | GSK3B functions downstream of TLR2-stimulation to induce the expression of the monocyte chemoattractant protein 1, CCL2. (Demonstrated in mice) |
| 51663 | GSK3B | 26100021 | Glycogen synthase kinase 3/CTNNB1 (Î²-catenin) axis is required for optimal induction of antiviral innate immunity. |
| 53956 | GSK3A | 26100021 | Glycogen synthase kinase 3/CTNNB1 (Î²-catenin) axis is required for optimal induction of antiviral innate immunity. |
| 44288 | TLR8 | 18985539 | TLR8 expression and function is highly up-regulated in colonic epithelium from patients with active inflammatory bowel disease (IBD), suggesting that TLR8 signalling is important in the pathogenesis of IBD. |
| 44288 | TLR8 | 14579267 | Toll-like receptor 8 (TLR8), 7 and 9 form a functional subgroup within the TLR family that recognizes pathogen-associated molecular patterns in endosomal/lysosomal compartments. |
| 44288 | TLR8 | 14976262 | TLR8 and TLR7 bind single-stranded RNA, stimulating dendritic cells (DCs) and macrophages to secrete interferon-alpha and pro-inflammatory, as well as regulatory, cytokines. |

| | | | |
|---|---|---|---|
| 44288 | TLR8 | 17264163 | TLR8 signalling strongly promotes inflammatory lipid mediator biosynthesis, providing novel insights on innate immune response to viral infections. |
| 44288 | TLR8 | 20652908 | TLR8 is activated in human monocytic cells following Helicobacter pylori phagocytosis and TLR8 single nucleotide polymorphism play a role in the modulation of TLR8-dependent microbicidal response of infected macrophages. |
| 44288 | TLR8 | 21734241 | TLR7 and TLR8 are translocated from the endoplasmic reticulum to the endosome in the presence of antiphospholipid antibodies, as a consequence, plasmacytoid dendritic cells become dramatically sensitized to TLR7/8 agonists and this may play a role in systemic autoimmunity. |
| 44288 | TLR8 | 22393042 | TLR8 binding of HIV ssRNA induces endosomal acidification and chromatin remodeling at the TNF-alpha promoter to promote TNF-alpha release in infected macrophages. |
| 44288 | TLR8 | 22753494 | TLR8 binds to exosomal MIR21 and MIR29A secreted by tumour cells and initiates a prometastatic inflammatory response. |
| 44288 | TLR8 | 24277153 | TLR8 plays a pathogenic role in disease whereby its expression is increased in patients with systemic arthritis and is correlated with the elevation of IL1B levels and disease status. |
| 44288 | TLR8 | 25599397 | TLR8 binds degradation products of single-stranded RNA at two distinct sites. One site prefers uridine mononucleosides and the other site prefers short oligonucleotides. |
| 44288 | TLR8 | 26101323 | TLR8-dependent detection of bacterial RNA is critical for triggering monocyte activation in response to infection with Streptococcus pyogenes. |
| 21151 | UBE2V2 | 26101372 | TRIM5 requires UBE2W, UBE2N and UBE2V2 enzymatic activities to inhibit retroviral DNA synthesis |
| 51220 | UBE2N | 16862162 | UBE2N abalation resulted in defective B cell development and in impaired B cell and macrophage activation. |
| 51220 | UBE2N | 26101372 | TRIM5 requires UBE2W, UBE2N and UBE2V2 enzymatic activities to inhibit retroviral DNA synthesis |
| 25727 | UBE2W | 26101372 | TRIM5 requires UBE2W, UBE2N and UBE2V2 enzymatic activities to inhibit retroviral DNA synthesis |
| 27690 | TRIM5 | 21131187 | TRIM5 is upregulated by Type I and Type II interferons and have been found to restrict viral replication by modulating the RIG-I pathway. |
| 27690 | TRIM5 | 21734563 | TRIM5 is an innate intracellular HIV restriction factor that is upregulated by type I interferons. |
| 27690 | TRIM5 | 23438823 | TRIM5 was identified in a systematic screen for positive regulators of innate immune responses. |
| 27690 | TRIM5 | 26101372 | TRIM5 requires UBE2W, UBE2N and UBE2V2 enzymatic activities to inhibit retroviral DNA synthesis |

| | | | |
|---|---|---|---|
| 58353 | OAS3 | 21190483 | OAS3 is a pathogen recognition receptor for the viral pathogen-associated molecular pattern, double-stranded RNA. Upon recognition of dsRNA, the OAS3 activates RNASEL to cleave ssRNA. The OAS/RNASEL pathway triggers the RIG-I pathway and induce IFNB production. |
| 58353 | OAS3 | 26063222 | Antiviral activity of the 2 â € ² ,5 â € ² -oligoadenylate synthetase (OAS) gene variants against dengue virus shows strong and serotype-specific disparities. |
| 58231 | OAS1 | 21190483 | OAS1 is a pathogen recognition receptor for the viral pathogen-associated molecular pattern, double-stranded RNA. Upon recognition of dsRNA, the OAS1 activates RNASEL to cleave ssRNA. The OAS/RNASEL pathway triggers the RIG-I pathway and induce IFNB production. |
| 58231 | OAS1 | 23319625 | OAS1 is a cytoplasmic dsRNA sensor. |
| 58231 | OAS1 | 26063222 | Antiviral activity of the 2 â € ² ,5 â € ² -oligoadenylate synthetase (OAS) gene variants against dengue virus shows strong and serotype-specific disparities. |
| 210514 | Pycard | 21439959 | Pycard is a component of the inflammasome and is required for inflammation in acute pancreatitis. |
| 210514 | Pycard | 22295065 | Nlrp3/Pycard inflammasome activation following human respiratory syncytial virus infection is dependent on the activation of Tlr2/Myd88/NF-kB and reactive oxygen species/potassium efflux. (Demonstrated in human) |
| 210514 | Pycard | 23302887 | PYCARD is an essential regulator of inflammatory responses in West Nile virus encephalitis. |
| 210514 | Pycard | 24185614 | Phosphorylation of the inflammasome adaptor Pycard (ASC) controls inflammasome activity through the formation of ASC specks. The NLRP3 and AIM2 inflammasomes require Syk and Mapk8 (JNK) for their full activity . |
| 210514 | Pycard | 25689249 | Activation of the Nlrp3 inflammasome is detrimental during leishmaniasis. Mice lacking the inflammasome components Nlrp3, Pycard, Casp1 exhibit defective Il1b and Il18 production at the infection site and are resistant to cutaneous Leishmania major infection. |
| 210514 | Pycard | 26071556 | Mice lacking Pycard display attenuated pulmonary hypertension and right ventricle remodelling in response to hypoxia and this is accompanied by blunted inflammasome activation. |
| 207109 | Psen2 | 26081153 | Psen2 deficiency is paralleled by reduced transcription of Tlr4 mRNA and loss of LPS-induced Tlr4 mRNA transcription regulation. |
| 196306 | Stmn1 | 26082487 | Stmn1 overexpression impacts microtubule stability, impairs cell spreading, reduces activation-associated phenotypes andreduces complement receptor 3 (CR3)-mediated phagocytosis and cellular activation. |

| | | | |
|---|---|---|---|
| 94374 | MAP3K7 | 10187861 | MAP3K7 (TAK1) is a member of the mitogen-activated protein kinase kinase kinase family that, together with its activator TAK1-binding protein 1 (TAB1), activates the IKK signallosome and thus regulates NF-kappaB activation. |
| 94374 | MAP3K7 | 17965022 | MAP3K7 is an essential intermediate of NOD2 signalling where MAP3K7 deletion completely abolishes muramyl dipeptide (MDP)-NOD2 signalling, activation of NF-kappaB and MAPKs, and the subsequent induction of cytokines/chemokines in keratinocytes. |
| 94374 | MAP3K7 | 10675530 | MAP3K7 mediates the activation signal from TLRs to nuclear factor-kappaB in lipopolysaccharide-stimulated macrophages. |
| 94374 | MAP3K7 | 16260493 | MAP3K7 acts as an upstream activating kinase for IKBKB (IKK-beta) and MAPK8 (JNK), but not CHUK (IKK-alpha), revealing a specific role of MAP3K7 in inflammatory signalling pathways. |
| 94374 | MAP3K7 | 8533096 | MAP3K7 (TAK1) functions as a mediator in the signalling pathway of TGF-beta superfamily members. |
| 94374 | MAP3K7 | 9480845 | MAP3K7 induces NF-kappaB activation through a MAP3K14 (NIK)-independent signalling pathway. |
| 94374 | MAP3K7 | 10094049 | MAP3K7 links TRAF6 to the MAP3K14 (NIK)-IKK cascade in the IL-1 signalling pathway where activated MAP3K7 phosphorylates NIK, which stimulates IKK-alpha activity. |
| 94374 | MAP3K7 | 20065289 | MAP3K7 is a target for glucocorticoids that integrates their anti-inflammatory action in innate immunity signalling pathways. |
| 94374 | MAP3K7 | 20200282 | MAP3K7 plays a central role in controlling nuclear and cytoplasmic signalling cascades in primary neutrophils where it constitutively associates with the IKBKB (I-kappa-B kinase) complex in the nucleus and cytoplasm, impacting downstream signalling processes. |
| 94374 | MAP3K7 | 21220427 | MAP3K7 activation is impaired during endotoxin tolerization; a process which impairs the production of LPS-induced pro-inflammatory cytokines without inhibition expression of anti-inflammatory or anti-microbial mediators. |
| 94374 | MAP3K7 | 22069318 | MAP3K7 polyubiquitination is essential for the activation of NF-kB signalling downstream of TNF receptor, IL1 receptor and TLR4. |
| 94374 | MAP3K7 | 22843747 | MAP3K7 is necessary for the neutrophil priming effect of leukotriene B (4) to enhance TLR stimulation. (Demonstrated in mice) |
| 94374 | MAP3K7 | 25028512 | MAP3K7 (TAK1) Ser412 phosphorylation is regulated by PRKACA and PRKX, and is essential for proper signalling, as well as proinflammatory cytokine induction by TLR/IL-1R activation. |

| | | | |
|---|---|---|---|
| 94374 | MAP3K7 | 25371197 | ECSIT binds to MAP3K7 and TRAF6 to form a complex that plays a pivotal role in activating TLR4-mediated NF-kB signalling. |
| 94374 | MAP3K7 | 26082489 | Endotoxin tolerance re-programs TLR4 signalling via suppression of PELI1, a positive regulator of MyD88- and TIR domain-containing adapter inducing IFN-Î² (TRIF)-dependent signalling that promotes K63-linked polyubiquitination of IRAK1, TBK1, and TAK1. |
| 90782 | IRAK1 | 18276832 | IRAK1 plays an essential role in Toll-like receptor/interleukin-1 receptor (TLR/IL-1R) -associated NF-kappaB activation through its involvement in IKK activation, which then leads to subsequent IkappaB degradation and NF-kappaB nuclear translocation and both cytosolic and nuclear actions of IRAK1 participate in the activation of NF-kappaB-dependent transcriptional events. |
| 90782 | IRAK1 | 17997719 | IRAK1 and IRAK4 play key roles in a signalling pathway by which bacterial infection or interleukin-1 (IL1) trigger the production of inflammatory mediators. Pellino isoforms are the E3 ubiquitin ligases that mediate the IL1-stimulated formation of K63-pUb-IRAK1 in cells, which contribute to the activation of IKBKB and NF-kappaB, as well as other signalling pathways dependent on IRAK1 and IRAK4. |
| 90782 | IRAK1 | 10224059 | IRAK1 is a component of a novel signal transduction pathway through which TNF receptor activates NF-kappaB-dependent gene expression. |
| 90782 | IRAK1 | 9625767 | IRAK1 plays an essential proximal role in coordinating multiple IL1 signalling pathways for optimal induction of cellular responses. |
| 90782 | IRAK1 | 20044140 | IRAK1 functionally associates with PKC-epsilon and VASP in the regulation of macrophage migration. |
| 90782 | IRAK1 | 21057262 | IRAK1 and MYD88 autosomal recessive deficiencies impair Toll-like receptor (TLR)- and interleukin-1 receptor-mediated immunity and predispose patients to recurrent life-threatening bacterial diseases, such as invasive pneumococcal disease in particular, in infancy and early childhood, with weak signs of inflammation. |
| 90782 | IRAK1 | 21220427 | IRAK1 is polyubiquitinated and disassembled during endotoxin tolerization; a process which impairs the production of LPS-induced pro-inflammatory cytokines without inhibition expression of anti-inflammatory or anti-microbial mediators. |
| 90782 | IRAK1 | 22033459 | IRAK1 mediates the proteasome-dependent degradation of TRAF6 and acts as a negative regulator of TLR-signalling. (Demonstrated in mice) |
| 90782 | IRAK1 | 23143987 | IRAK1 accumulation triggers ischemia-induced inflammation in the small intestine. |
| 90782 | IRAK1 | 24670381 | MIR146A is a potent negative regulator of the innate immune response in keratinocytes through downregulation of the IRAK1/TRAF6/NFÎºB pathway. |

| | | | |
|---|---|---|---|
| 90782 | IRAK1 | 26082489 | Endotoxin tolerance re-programs TLR4 signalling via suppression of PELI1, a positive regulator of MyD88- and TIR domain-containing adapter inducing IFN-Î² (TRIF)-dependent signalling that promotes K63-linked polyubiquitination of IRAK1, TBK1, and TAK1. |
| 25713 | MYD88 | 11976320 | MYD88 is a Toll/IL-1R homology (TIR) domain containing adaptor which recruits IRAK1 possibly through IRAK4. |
| 25713 | MYD88 | 18327267 | MYD88 can interact with bacterial TIR domain containing-proteins (Tcps) identified in Escherichia coli CFT073 (TcpC) and Brucella melitensis (TcpB) and interfere with MYD88-dependent pathway, thus suppressing innate immunity and increasing virulence. |
| 25713 | MYD88 | 16354686 | MYD88 binding with interleukin-1 (IL-1) receptor (IL1R1) is required for inducing endocytosis of IL1R1 following ligand binding. |
| 25713 | MYD88 | 9734363 | MYD88 is a key adaptor/regulator molecule for the Toll/IL-1R family of receptors for innate immunity. |
| 25713 | MYD88 | 9430229 | MYD88 interacts with the IL-1 receptor and blocks NF-kappaB activation induced by IL-1, but not by TNF. |
| 25713 | MYD88 | 20167866 | TLR-2/MyD88/PI3K/Rac1/Akt pathway mediates LTA-induced MAPKs activation, which in turn initiates the activation of NF-kappaB, and ultimately induces cPLA2/COX-2-dependent PGE2 and IL-6 generation. |
| 25713 | MYD88 | 20519121 | MYD88 plays a critical role in reverse cholesterol transport in vitro and in vivo, in part through promoting ATP-binding cassette A1 transporter upregulation, coupling cholesterol trafficking to inflammation through MYD88 and identifying innate immunity as a physiologic signal in cholesterol homeostasis. |
| 25713 | MYD88 | 21057262 | MYD88 and IRAK1 autosomal recessive deficiencies impair Toll-like receptor (TLR)- and interleukin-1 receptor-mediated immunity and predispose patients to recurrent life-threatening bacterial diseases, such as invasive pneumococcal disease in particular, in infancy and early childhood, with weak signs of inflammation. |
| 25713 | MYD88 | 21248248 | MYD88 is essential in restricting TLR3 signalling and the host protection from unwanted immunopathologies associated with excessive production of IFNB1. MYD88 inhibits TLR3 signalling by impairing IKBKE-mediated induction of IRF3, and consequently the expression IFNB1 and CCL5. |
| 25713 | MYD88 | 21283748 | MYD88 is activated by MHC class II in response to staphylococcal enterotoxins and is crucial for the induction of pro-inflammatory cytokines. |

| | | | |
|---|---|---|---|
| 25713 | MYD88 | 21325272 | MYD88 is a key signalling adapter in TLR signalling. MYD88 aggregates in the cell as distinct foci and co-localizes with IRAK4 in these Myddosomes - the formation of which is required for MYD88 function. |
| 25713 | MYD88 | 21353603 | MYD88 is required in dendritic cells stimulated with TLR9 ligand for the enhancement of T cell-dependent antibody response. In addition, MYD88 is required in B cells to facilitate strong anti-viral antibody responses. (Demonstrated in murine model) |
| 25713 | MYD88 | 21422180 | MYD88 deficient macrophages displayed impaired interaction with fungal yeast cells and produced low levels of pro-inflammatory cytokines. MYD88 signalling is important in the activation of fungicidal mechanisms and the induction of protective innate immune responses against P. brasiliensis. (Demonstrated in murine model) |
| 25713 | MYD88 | 22028692 | MYD88 mediates cytoskeletal remodelling and late spreading of lipopolysaccharide (LPS)-stimulated macrophages. (Demonstrated in mice) |
| 25713 | MYD88 | 22025508 | MYD88-dependent recruitment of inflammatory monocytes and dendritic cells to the lungs are key initial cellular responses required for early protection from Burkholderia mallei infection. (Demonstrated in mice) |
| 25713 | MYD88 | 22386951 | MYD88 deficiency results in delayed recruitment of phagocytes and defective production of proinflammatory cytokines in response to Salmonella infection. (Demonstrated in mice) |
| 25713 | MYD88 | 22491177 | MYD88 signalling in intestinal epithelial cells is crucial for the maintenance of gut microbiota homeostasis. (Demonstrated in mice) |
| 25713 | MYD88 | 22536449 | MYD88 mediated production of reactive oxygen species (ROS) is essential for the induction of IL12 by lactic acid bacteria. (Demonstrated in mice) |
| 25713 | MYD88 | 24670424 | Following NOD2 activation, IRF4 interacts with MYD88, TRAF6, and RIPK2 and downregulates K63-linked polyubiquitinylation of RICK and TRAF6 leading to disruption of NFkB activation pathways. |
| 25713 | MYD88 | 26082489 | Endotoxin tolerance re-programs TLR4 signalling via suppression of PELI1, a positive regulator of MyD88- and TIR domain-containing adapter inducing IFN-Î² (TRIF)-dependent signalling that promotes K63-linked polyubiquitination of IRAK1, TBK1, and TAK1. |
| 53855 | PELI1 | 19734906 | PELI1 is a ubiquitin ligase that facilitates TRIF-dependent Toll-like receptor signalling and pro-inflammatory cytokine production. |
| 53855 | PELI1 | 17997719 | PELI1 (pellino) isoforms are E3 ubiquitin ligases that mediate the IL-1-stimulated formation of K63-pUb-IRAK1 in cells, contributing to the activation of IKBKB and NF-kappaB, as well as other signalling pathways dependent on IRAK1 and IRAK4. |

| | | | |
|---|---|---|---|
| 53855 | PELI1 | 20171181 | Discrete regions of PELI1 are bound by SMAD6 and SMAD7 via their MH2 domains to mediate TGF-beta1-induced negative regulation of IL-1R/TLR signalling. |
| 53855 | PELI1 | 21120624 | PELI1 is an adaptor protein involved in IL1R/TLR signaling. PELI1 is sumoylated by SUMO1at 5 lysine residues, and binds to the SUMO-conjugating enzyme UBE2I. |
| 53855 | PELI1 | 26082489 | Endotoxin tolerance re-programs TLR4 signalling via suppression of PELI1, a positive regulator of MyD88- and TIR domain-containing adapter inducing IFN-Î² (TRIF)-dependent signalling that promotes K63-linked polyubiquitination of IRAK1, TBK1, and TAK1. |
| 34438 | TIFA | 26068852 | Bacterial-derived monosaccharide heptose-1,7-bisphosphate (HBP) is a pathogen-associated molecular pattern (PAMP) that activates TIFA-dependent immunity to Gram-negative bacteria |
| 181478 | Rora | 22267218 | The transcription factor Rora is critical for the development of nuocytes and the mounting of innate type 2 immunity against parasitic worms. |
| 181478 | Rora | 26045547 | Nlrp3 is a novel molecular target for melatonin which requires Rora to blunt the NFkB/ NLRP3 connection during sepsis. |
| 154747 | Irak1 | 21220427 | Irak1 is polyubiquitinated and disassembled during endotoxin tolerization; a process which impairs the production of LPS-induced pro-inflammatory cytokines without inhibition expression of anti-inflammatory or anti-microbial mediators. |
| 154747 | Irak1 | 22033459 | Irak1 mediates the proteasome-dependent degradation of Traf6 and acts as a negative regulator of TLR-signalling. |
| 154747 | Irak1 | 23143987 | IRAK1 accumulation triggers ischemia-induced inflammation in the small intestine. |
| 154747 | Irak1 | 26048146 | Mir146 attenuates sepsis-induced cardiac dysfunction by preventing NF Î º B activation, inflammatory cell infiltration, and inflammatory cytokine production via targeting of Irak1 and Traf6 in both cardiomyocytes and inflammatory monocytic cells |
| 36786 | IFRD1 | 26055519 | Human papillomaviruses impair the acetylation of NFÎºB/RelA K310 in keratinocytes by augmenting the expression of interferon-related developmental regulator 1 (IFRD1) in an EGFR-dependent manner. |
| 16933 | EGFR | 11470832 | EGFR is the receptor for epidermal growth factor (EGF) and signalling through growth factor receptors controls diverse cell functions such as proliferation, migration, and differentiation. |
| 16933 | EGFR | 15284024 | EGFR-induced cell migration is mediated predominantly by the JAK-STAT pathway in primary esophageal keratinocytes. |

| | | | |
|---|---|---|---|
| 16933 | EGFR | 18772136 | EGFR and TLR4 are activated by neutrophil elastase (ELA2) to produce IL8 through a novel metalloprotease pathway. |
| 16933 | EGFR | 26055519 | Human papillomaviruses impair the acetylation of NFÎºB/RelA K310 in keratinocytes by augmenting the expression of interferon-related developmental regulator 1 (IFRD1) in an EGFR-dependent manner. |
| 41300 | PDE12 | 26055709 | PDE12 negatively regulates the innate immune response and inhibitors of PDE12 show increased IFN induced 2â€ ,5â€ -<br>oligoadenylate and antiviral activities. |
| 408826 | LYN | 20385881 | The LYN/PI3K module negatively regulates activation of murine macrophages while Inpp5d (SHIP-1) promotes it. |
| 408826 | LYN | 22491248 | Overexpression of LYN results in endotoxin hypersensitivity due to the increased activation of dendritic cells leading to an over-production of IFNG by natural killer cells. (Demonstrated in mice) |
| 408826 | LYN | 26055819 | LYN-dependent phosphorylation of the p110 catalytic subunit of PI 3-kinase is essential to the control of PI 3-kinase biological activity upstream of AKT and thereby to the transactivation of NFÎºB. |
| 92358 | MB21D1 | 23707061 | MB21D1 (cGAS) is a cytosolic DNA sensor and is required for the induction of the interferon response. |
| 92358 | MB21D1 | 24284630 | Cytosolic DNA sensor cyclic GMP-AMP synthase (cGAS, also known as MB21D1) is pivotal in protecting the host from both DNA and RNA viruses |
| 92358 | MB21D1 | 24269171 | Cytosolic DNA sensor cGAS (MB21D1) is essential in human dentritic cells for innate sensing of HIV-1 and HIV-2. |
| 92358 | MB21D1 | 25425575 | Cytosolic RNA:DNA hybrids are sensed by the MB21D1-TMEM173 (cGAS-STING) pathway of the innate immune system. |
| 92358 | MB21D1 | 25831530 | IFI16 and MB21D1 interact and cooperate during herpes simplex virus infection to initiate innate signalling. |
| 92358 | MB21D1 | 26046437 | PQBP1 directly binds to reverse-transcribed HIV-1 DNA and interacts with MB21D1 to initiate an IRF3-dependent innate response. |
| 64349 | PQBP1 | 26046437 | PQBP1 directly binds to reverse-transcribed HIV-1 DNA and interacts with MB21D1 to initiate an IRF3-dependent innate response. |
| 300259 | TNF | 16199883 | TNF (TNF-alpha) is an important mediator of inflammation, apoptosis, and the development of secondary lymphoid structures and LRRFIP1 represses TNF expression. |

| | | | |
|---|---|---|---|
| 300259 | TNF | 21602809 | TNF pre-treated macrophages exhibit endotoxin tolerance, i.e. less cytokine production, towards LPS challenge. TNF-mediated cross-tolerization is mediated by suppression of LPS-induced signalling and chromatin remodelling. |
| 300259 | TNF | 21611132 | TNF is essential to mount an acute inflammatory response to dsDNA in the endothelium. (Demonstrated in murine model) |
| 300259 | TNF | 24012417 | IFN gamma creates a primed chromatin environment in macrophages to augment TLR-induced TNF transcription |
| 300259 | TNF | 25005359 | Secreted CCNA2 (CCN1) promotes anti-inflammatory cytokine IL10 release from epithelial cells via integrin Î±VÎ²6-PKC, and this subsequently suppresses TNF, CXCL2 and neutrophil infiltration in the lungs. |
| 300259 | TNF | 25637949 | DEFB103A and RNASE7 are induced in human umbilical endothelial cells (HUVECs) by classical inflammatory cytokines such as: IFNG, IL1B and TNF. |
| 2528 | RNASE7 | 25712218 | RNASE7 is produced by airway epithelial basal cells in response to cigarette smoke exposure. |
| 2528 | RNASE7 | 25637949 | DEFB103A and RNASE7 are induced in human umbilical endothelial cells (HUVECs) by classical inflammatory cytokines such as: IFNG, IL1B and TNF. |
| 103863 | AIM2 | 19158675 | AIM2 recognizes cytosolic double stranded DNA and forms a caspase-1-activating inflammasome with PYCARD (ASC). |
| 103863 | AIM2 | 20351693 | AIM2 has a critical role in host innate immunity to intracellular pathogens where it is a crucial sensor of F. tularensis infection. |
| 103863 | AIM2 | 20351692 | AIM2 inflammasome is essential for host defence against cytosolic bacteria and DNA viruses. |
| 103863 | AIM2 | 20401524 | AIM2 is a newly discovered pattern recognition receptor (PRR) involved in the sensing of dangerous cytosolic DNA produced by infection with DNA viruses. |
| 103863 | AIM2 | 20457908 | AIM2 is required for innate immune recognition of Francisella tularensis where AIM2-deficient mice display an increased susceptibility to F. tularensis infection compared with wild-type mice. |
| 103863 | AIM2 | 21562230 | AIM2-containing inflammasomes are activated in response to cytosolic DNA, Â  this response is augmented in keratinocytes from psoriatic lesions and contributes to the auto-inflammatory disease. |
| 103863 | AIM2 | 25641891 | Single nucleotide polymorphisms in IFI16 and AIM2 are associated with BehÃ§et disease . |
| 103852 | IFI16 | 20890285 | IFI16, a PYHIN protein, is an intracellular DNA sensor that mediates the induction of interferon-beta (IFNB) by directly associating with IFNB-inducing viral DNA motifs. |

| | | | |
|---|---|---|---|
| 103852 | IFI16 | 21575908 | IFI16 acts as a nuclear pathogen sensor and interacts with PYCARD and CASP1 to form a functional inflammasome during KSHV infections. |
| 103852 | IFI16 | 24131791 | Cyclic-di-GMP-induced levels of IFI16 suppress the expression of TMEM173 (STING). |
| 103852 | IFI16 | 24367117 | IFI16 is essential for host defence by clustering into signalling foci with foreign DNA in a switch-like manner and is capable of using the size of naked double stranded DNA as a molecular ruler to distinguish self from nonself. |
| 103852 | IFI16 | 24449861 | In herpes simplex virus 1 (HSV-1) infected cells, the stability and function of IFI16 and TMEM173 are dependent on cell derivation and the functional integrity of HSV-1 proteins ICP0 and US3 protein kinase. |
| 103852 | IFI16 | 24154727 | IFI16 is a sensor for lentiviral reverse transcription products and restricts HIV-1 replication in macrophages. |
| 103852 | IFI16 | 24237704 | IFI16 oligomerizes upon viral DNA sensing in human cytomegalovirus (HCMV) infected-cells. The HCMV major tegument protein pUL83 blocks nuclear IFI16 oligomerization and inhibits IFI16-mediated antiviral cytokine expression |
| 103852 | IFI16 | 24449861 | The end result of the interplay between TMEM173 (STING), IFI16, and herpes simplex virus 1 (HSV-1) is determined by the genotype of the infected cells and the functional integrity of HSV-1 proteins infected cell protein 0 (ICP0) and US3 protein kinase. |
| 103852 | IFI16 | 24154727 | IFI16 is a sensor for lentiviral reverse transcription products and restricts HIV-1 replication in human macrophages. |
| 103852 | IFI16 | 25002588 | IFI16 transcriptionally regulates type-I interferons and DDX58 (RIG-I) and controls the interferon response to both DNA and RNA viruses. |
| 103852 | IFI16 | 25831530 | IFI16 and MB21D1 interact and cooperate during herpes simplex virus infection to initiate innate signalling. |
| 103852 | IFI16 | 25972554 | IFI16 restricts chromatinized human papillomaviruses DNA through epigenetic modifications, thus reducing both viral replication and transcription. |
| 103852 | IFI16 | 25641891 | Single nucleotide polymorphisms in IFI16 and AIM2 are associated with BehÃ§et disease. |
| 73479 | MAPK8 | 19153595 | MAPK8 phosphorylates IRF3 and is essential for IRF3 dimerization induced by polyinosinic-cytidylic acid (polyI:C). |
| 73479 | MAPK8 | 25642820 | Mycobacterium tuberculosis phosphatase PtpA suppresses innate immunity by binding to ubiquitin; which, in turn, activates it to dephosphorylate phosphorylated MAPK8 and MAPK14. |

| | | | |
|---|---|---|---|
| 84613 | MAPK14 | 15569672 | MAPK14 is dephosphorylated by PPP2R4, which then induces apoptosis in neutrophils and the resolution of inflammation. |
| 84613 | MAPK14 | 21683629 | MAPK14 activation is blocked by Bacillus anthracis, resulting in the opening of a connexin ATP release channel and induction of macrophage death. Constitutive activation of MAPK14 interferes with inflammasome activation and IL1B production, which compromises antimicrobial immunity. (Demonstrated in murine model) |
| 84613 | MAPK14 | 21733175 | The MAPK14 pathway is an important contributor to microglial production of proinflammatory cytokines induced by LPS or beta-amyloid. (Demonstrated in mouse) |
| 84613 | MAPK14 | 22028692 | MAPK14 mediates cytoskeletal remodelling and early spreading of lipopolysaccharide (LPS)-stimulated macrophages. (Demonstrated in mice) |
| 84613 | MAPK14 | 22447027 | MAPK1 (ERK) and MAPK14 (p38) control the dynamic balance regulating neutrophil migration. |
| 84613 | MAPK14 | 25642820 | Mycobacterium tuberculosis phosphatase PtpA suppresses innate immunity by binding to ubiquitin; which, in turn, activates it to dephosphorylate phosphorylated MAPK8 and MAPK14. |
| 158878 | Pten | 25646418 | Pten is an essential regulator of natural killer cell localization in vivo during both homeostasis and malignancy. |
| 135792 | Tnfaip3 | 21220427 | Tnfaip3 expression and Tnfaip3-Irak1interaction are important for endotoxin tolerance. Tnfaip3 overexpression inhibits LPS-induced activation of NFKB, and is mechanistically linked to endotoxin tolerance through the reprogramming of Tlr4 signaling. |
| 135792 | Tnfaip3 | 22031828 | Tnfaip3 promotes intestinal epithelial barrier integrity and inhibits LPS-induced loss of the tight junction protein occludin. |
| 135792 | Tnfaip3 | 24023826 | Tnfaip3 is regulated by both Nf-Î°B and p38-dependent Cebpb in response to LPS in macrophages. |
| 135792 | Tnfaip3 | 25683052 | Mirlet7f and its target Tnfaip3 regulate immune responses to Mycobacterium tuberculosis and control bacterial burden by augmenting the production of Tnf and Il1b. |
| 135792 | Tnfaip3 | 25609235 | Sqstm1 captures Tnfaip3, an NFkB inhibitor, and sequesters it in the autophagosome. This allows macrophages to release chemokines that recruit neutrophils and boost antifungal immunity. |
| 168358 | Sqstm1 | 21220332 | Sqstm1 is required for Tlr4-mediated autophagy. Tlr4-driven induction of Sqstm1 plays an essential role in the formation and the autophagy degradation of aggresome-like induced structures, which might be critical for regulating host defense. |

| | | | |
|---|---|---|---|
| 168358 | Sqstm1 | 22901810 | Sqstm1 and Calcoco2 are ubiquitin-autophagy receptors that are required for the recognition of extracelluar bacterial DNA by the Tmem173 (STING)-dependent cytosolic pathway, marking bacteria with ubiquitin, and delivery of bacilli to autophagosomes. |
| 168358 | Sqstm1 | 24011591 | Phosphorylation of Sqstm1 (p62) activates the Keap1-Nrf2 pathway during selective autophagy. |
| 168358 | Sqstm1 | 25609235 | Sqstm1 captures Tnfaip3, an NFkB inhibitor, and sequesters it in the autophagosome. This allows macrophages to release chemokines that recruit neutrophils and boost antifungal immunity. |
| 86629 | TREM1 | 20797396 | TREM1 is a superimmunoglobulin receptor present on neutrophils and monocytes, which plays an important role in the amplification of inflammation and its expression is inhibited by PGD(2) and PGJ(2) in macrophages. |
| 86629 | TREM1 | 21393102 | TREM1 is an activating receptor expressed on neutrophils and monocytes that amplifies inflammation induced by TLR4-signalling, specifically in the induction of TNFA production. |
| 86629 | TREM1 | 21555403 | TREM1 expression is upregulated following Pseudomonas aeruginosa infection in the cornea and the inhibition of TREM1 reduces the severity of corneal disease. TREM1 acts as an inflammatory amplifier in P. aeruginosa keratitis by modulating TLR signalling and Th1/Th2 responses. |
| 86629 | TREM1 | 21690199 | TREM1 expression is induced by vitamin D3 in human bronchial epithelial cells. Activation of TREM1 leads to the induction of human beta defensin 2 and TNF-alpha mRNA in the airway epithelium. |
| 86629 | TREM1 | 22459945 | TREM1 mediates endotoxin tolerance in monocytes through its ability to induce anti- or pro-inflammatory signals depending on its membrane-bound state. |
| 86629 | TREM1 | 23475790 | TREM1 expression and shedding are regulated by CpG-mediated TLR9 activation in macrophages. |
| 86629 | TREM1 | 25595774 | PGLYRP1 binds to bacterially derived peptidoglycan and these complexes constitute potent ligands capable of binding to TREM1 and inducing myeloid cell functions. |
| 58413 | PGLYRP1 | 20418257 | PGLYRP1 is a secreted innate immunity protein that is expressed in polymorphonuclear leukocytes and is conserved from insects to mammals, it recognizes bacterial peptidoglycan, and functions in antibacterial immunity and inflammation. |
| 58413 | PGLYRP1 | 21134971 | PGLYRP1 is a peptidoglycan recognition protein and play a role in innate immunity against L. monocytogenes infection by inducing TNFA. (Demonstrated in murine model) |

| | | | |
|---|---|---|---|
| 58413 | PGLYRP1 | 21439073 | PGLYRP1 is a member of the Peptidoglycan Recognition Proteins (PGRP) family and recognizes peptidoglycan, a structural component of bacterial cell walls, as a part of innate immune response against infections. |
| 58413 | PGLYRP1 | 21602801 | PGLYRP1 binds to Gram-positive bacterial wall and activates a protein-sensing two-component system to induce bacterial death. PLGYRP1-mediated activation results in membrane depolarization and cessation of peptidoglycan, protein, and RNA/DNA synthesis, as well as the production of hydroxyl radicals. |
| 58413 | PGLYRP1 | 25595774 | PGLYRP1 binds to bacterially derived peptidoglycan and these complexes constitute potent ligands capable of binding to TREM1 and inducing myeloid cell functions. |
| 136612 | Il17f | 25595775 | Defb1 is important for the control of early mucosal Candida infection and plays a critical role in the induction of innate inflammatory mediators including, Il1b, Il6, Cxcl1, Il17a, and Il17f. |
| 178943 | Cxcl1 | 25595775 | Defb1 is important for the control of early mucosal Candida infection and plays a critical role in the induction of innate inflammatory mediators including, Il1b, Il6, Cxcl1, Il17a, and Il17f. |
| 139170 | Defb1 | 21551252 | Defb1 is upregulated in plasmacytoid dendritic cells and monocyte during viral challenge Defb1-deficient mice infected with mouse-adapted HK18 (influenza) lost weight earlier and died sooner than WT mice, suggesting that Defb1 plays a role in early innate immune responses against influenza in vivo. However, lung virus titers were equal between the two mouse strains, indicating that the mechanism is not related to viral replication. |
| 139170 | Defb1 | 22102811 | Defb1 is a component of platelets that displays classic antimicrobial activity and signals polymorphonuclear leukocytes to extrude DNA lattices that capture and kill bacteria. (Demonstrated in human) |
| 139170 | Defb1 | 25595775 | Defb1 is important for the control of early mucosal Candida infection and plays a critical role in the induction of innate inflammatory mediators including, Il1b, Il6, Cxcl1, Il17a, and Il17f. |
| 175257 | Dnase2a | 25600358 | Dnase2a is required for Tlr9 activation by bacterial genomic DNA. |
| 66320 | FPR2 | 25605714 | FPR1 and FPR2 are G-protein-coupled receptors that recognize bacterial signal peptides, constituting a novel class of immune activators that contribute to mammalian immune defence against bacteria. |

| | | | |
|---|---|---|---|
| 66270 | FPR1 | 25605714 | FPR1 and FPR2 are G-protein-coupled receptors that recognize bacterial signal peptides, constituting a novel class of immune activators that contribute to mammalian immune defence against bacteria. |
| 18954 | PPARG | 20421464 | PPARG functions as an antimicrobial factor by maintaining constitutive epithelial expression of a subset of beta-defensin in the colon. |
| 18954 | PPARG | 21148557 | PPARG negatively regulates IFNB production in TLR3/4-stimulated macrophages by preventing IRF3 binding to the IFN-beta promoter. |
| 18954 | PPARG | 25595716 | MIR130A reduces hepatitis B virus (HBV) replication by down-regulating the expression of two major metabolic regulators PPARGC1A and PPARG, both of which can potently stimulate HBV replication. |
| 11756 | PPARGC1A | 21966468 | PPARGC1A is activated in Staphylococcus aureus-mediated sepsis via the TLR2-signalling pathway. (Demonstrated in mice) |
| 11756 | PPARGC1A | 25595716 | MIR130A reduces hepatitis B virus (HBV) replication by down-regulating the expression of two major metabolic regulators PPARGC1A and PPARG, both of which can potently stimulate HBV replication. |
| 126561 | MIR130A | 25595716 | MIR130A reduces hepatitis B virus (HBV) replication by down-regulating the expression of two major metabolic regulators PPARGC1A and PPARG, both of which can potently stimulate HBV replication. |
| 212409 | Tollip | 25586187 | Super-low dose lipopolysaccharide induces inhibitory phosphorylation of Pik3c3 leading to the disruption of endosome-lysosome fusion and low-grade inflammation in innate macrophages; a mechanism that depends on the clearance and relocation of Tollip. |
| 132410 | Pik3c3 | 22170068 | Class III phosphatidylinositol 3-kinases (PI3K) are required for downstream Arf6 regulation of CpG oligodeoxynucleotide uptake and thus have a role in Tlr9-mediated immune signalling. |
| 132410 | Pik3c3 | 25586187 | Super-low dose lipopolysaccharide induces inhibitory phosphorylation of Pik3c3 leading to the disruption of endosome-lysosome fusion and low-grade inflammation in innate macrophages; a mechanism that depends on the clearance and relocation of Tollip. |
| 177358 | Tnf | 21602809 | Tnf pre-treated macrophages exhibit endotoxin tolerance, i.e. less cytokine production, towards LPS challenge. Tnf-mediated cross-tolerization is mediated by suppression of LPS-induced signalling and chromatin remodelling. |
| 177358 | Tnf | 21611132 | Tnf is essential to mount an acute inflammatory response to dsDNA in the endothelium. |
| 177358 | Tnf | 25683052 | Mirlet7f and its target Tnfaip3 regulate immune responses to Mycobacterium tuberculosis and control bacterial burden by augmenting the production of Tnf and Il1b. |

| | | | |
|---|---|---|---|
| 129000 | Cav1 | 19949109 | Cav1 is an important component of the innate host immune response to the majority of non-cytotoxic strains of P. aeruginosa by promoting bacterial clearance during acute pneumonia and chronic colonization. |
| 129000 | Cav1 | 25665524 | The Akt1/Mir199a/Cav1 pathway is a regulator of innate immunity that is dysfunctional in cystic fibrosis macrophages contributing to lung hyper-inflammation. |
| 223741 | Mir199a-1 | 25665524 | The Akt1/Mir199a/Cav1 pathway is a regulator of innate immunity that is dysfunctional in cystic fibrosis macrophages contributing to lung hyper-inflammation. |
| 174623 | Akt1 | 21196185 | Akt1 is a Ser/Thr protein kinase that plays a pivotal role in functional activation in macrophages. Akt1 specifically functions in phagocytosis, intracellular bacterial infection, LPS tolerance, production of inflammatory cytokines/mediators, and migration during macrophage-mediate innate immunity. |
| 174623 | Akt1 | 21683629 | Akt1 activation is blocked by Bacillus anthracis, resulting in the opening of a connexin ATP release channel and induction of macrophage death. Constitutive activation of Akt1 interferes with inflammasome activation and Il1b production, which compromises antimicrobial immunity. |
| 174623 | Akt1 | 22218715 | Akt1 functions downstream of Tlr2-stimulation to induce the expression of the monocyte chemoattractant protein 1, Ccl2. |
| 174623 | Akt1 | 25783839 | Lysophosphatidic acid plays an anti-inflammatory role in macrophages by diminishing lipopolysaccharide-induced phosphorylation of Mapk14 and Akt1, as well as Rela nuclear translocation. |
| 174623 | Akt1 | 25665524 | The Akt1/Mir199a/Cav1 pathway is a regulator of innate immunity that is dysfunctional in cystic fibrosis macrophages contributing to lung hyper-inflammation. |
| 100329 | DR1 | 25589657 | DR1, a novel host susceptibility gene for influenza A virus replication, suppresses host innate immunity and enhances viral RNA replication. |
| 190697 | Il10 | 21849677 | Il10 contributes to antiviral innate immunity during acute infection by restricting activation-induced death in natural killer (NK) cells. Blockade of Il10 receptor during acute murine cytomegalovirus (CMV) infection markedly reduced the accumulation of cytotoxic NK cells in the spleen and lung. |
| 190697 | Il10 | 22268692 | Il10 has opposing functions in anti-microbial responses in its capacity to mediate protective immunity against some organisms but increase susceptibility to other infections. |
| 190697 | Il10 | 22876184 | Il10-mediated suppression of natural killer/dendritic cell crosstalk leads to prolonged mouse cytomegalovirus (MCMV) persistence due to poor priming of MCMV-specific T cells. |

| | | | |
|---|---|---|---|
| 190697 | Il10 | 25826367 | Retinoic acid treatment enhances Tlr2-dependent Il10 production from T cells and this, in turn, potentiates T regulatory cell generation without the need for activation of antigen presenting cells. |
| 190697 | Il10 | 25959063 | Intestinal macrophages that constitutively produce IL10, control excessive innate immune activation and prevent tissue damage after an acute bacterial infection. |
| 69557 | CASP4 | 25964352 | CASP4 is a critical regulator of noncanonical inflammasome activation that initiates defence against bacterial pathogens in primary macrophages by mediating cell death and IL1A release |
| 62945 | PTX3 | 19050261 | PTX3 is a multifunctional soluble molecule involved in inflammation and innate immunity. It is a unique factor H (FH) ligand in that it can bind both of the two hot-spots of FH and can participate in the localization of functionally active FH. |
| 62945 | PTX3 | 20208538 | PTX3, an essential component of humoral innate immunity, and immunoglobulins share functional outputs, including complement activation, opsonization and glycosylation-dependent regulation of inflammation. |
| 62945 | PTX3 | 20363749 | PTX3, an inflammation-associated long pentraxin, plays key roles in innate immunity, female fertility and vascular biology. PTX3 octamer contains two fibroblast growth factor 2 (FGF2) binding sites and this quaternary organization is required for the anti-angiogenic function of PTX3. |
| 62945 | PTX3 | 20683616 | PTX3 is produced by innate immunity cells (e.g. PMN, macrophages, dendritic cells) and it interacts with several ligands to play an essential role in innate immunity, tuning inflammation and matrix deposition. PTX3 provides a paradigm for the mode of action of humoral innate immunity. |
| 62945 | PTX3 | 21106539 | PTX3, serum amyloid P component (SAP) and C-reactive protein (CRP) belong to the pentraxin family of pattern recognition molecules involved in tissue homeostasis and innate immunity. PTX3 heterocomplexes with mannose-binding lectin (MBL) to trigger cross-activation of the complement system. |
| 62945 | PTX3 | 21465531 | PTX3 production is up-regulated in response to serum amyloid A, and contributes to the inflammatory pathogenesis of atherosclerosis. |
| 62945 | PTX3 | 21490156 | FCN3 and PTX3 are soluble oligomeric pattern-recognition molecules that interact with each other and act synergistically to activate the lectin complement pathway. |
| 62945 | PTX3 | 22278372 | PTX3 forms a complex with components of neutrophil extracellular traps in septic patients. |
| 62945 | PTX3 | 23475792 | Sputum PTX3 level are lower in cystic fibrosis patients due to proteolytic cleavage by Aspergillus fumigatus. |

| | | | |
|---|---|---|---|
| 62945 | PTX3 | 25679762 | PTX3 acts as an extrinsic oncosuppressor gene by regulating Complement-dependent, macrophage-sustained, tumor-promoting inflammation. |
| 62945 | PTX3 | 25964372 | PTX3 plays a non-redundant protective role in orchestrating tissue repair and remodeling by interacting with fibrin and plasminogen. |
| 102649 | S100A12 | 25964473 | S100A12 is induced in response to H. pylori infection and inhibits bacterial growth and viability in vitro by binding nutrient zinc. |
| 45673 | CXCL14 | 25964486 | CXCL14 is important for constitutive antimicrobial defences against pneumonia |
| 201810 | Myo18a | 25965346 | Myo18a isoforms differentially regulate trafficking, expression, and activation of innate immune receptors on macrophages |
| 207763 | Rac1 | 25972472 | Cd81 inhibits Rac1/Stat1 activation and negatively regulates the defence mechanisms to Listeria monocytogenes infection. |
| 212677 | Cd81 | 25972472 | Cd81 inhibits Rac1/Stat1 activation and negatively regulates the defence mechanisms to Listeria monocytogenes infection. |
| 38977 | CAPRIN1 | 25784705 | The G3BP1-CAPRIN1-PRKRA complex represents a new mode of PRKRA activation and links stress responses with innate immune activation through PRKRA without a requirement for foreign double-stranded RNA pattern recognition. |
| 54619 | G3BP1 | 25520508 | EIF2AK2 (PKR) is recruited to stress granules by G3BP1 to promote innate immune responses at both transcriptional and translational levels. |
| 54619 | G3BP1 | 25784705 | The G3BP1-CAPRIN1-PRKRA complex represents a new mode of PRKRA activation and links stress responses with innate immune activation through PRKRA without a requirement for foreign double-stranded RNA pattern recognition. |
| 65053 | ULK1 | 25792739 | Stimulation of TMEM173-dependent IRF3 activation by ultraviolet radiation is due to apoptotic signalling-dependent disruption of ULK1, a pro-autophagic protein that negatively regulates TMEM173. |
| 152923 | Nfil3 | 21383239 | Nfil3 is an Il12b transcriptional inhibitor in macrophages. Interactions of macrophages with the enteric microbiota induce Nfil3 to limit their inflammatory capacity. |
| 152923 | Nfil3 | 25801035 | Nfil3 is a key regulator of common helper-like innate lymphoid cell progenitors as they emerge during early lymphopoiesis. |
| 194391 | Anpep | 25801433 | Lipopolysaccharide-mediated myeloid Anpep (CD13) expression governs internalization of Tlr4 and negatively regulates Tlr4 signalling, thereby balancing the innate response by maintaining the inflammatory equilibrium critical to innate immune regulation. |
| 77255 | WFDC12 | 25770093 | WFDC12 plays a role in the regulation of lung inflammation. |

| | | | |
|---|---|---|---|
| 184766 | Ctnnd1 | 25773174 | Ctnnd1 expressed in alveolar type II epithelial cells plays a critical role in regulating the innate immunity of the entire lung. |
| 150795 | Ly6g | 25781937 | Escherichia coli toxin CNF1 promotes the maturation/secretion of Il1b while the Î±-hemolysin toxin inhibits Il1b secretion without affecting the recruitment of Ly6g+ cells. |
| 132298 | Rela | 21209118 | Rela is a subunit of NFKB and is not essential for virus-stimulated Ifnb expression, instead, Rela sustains autocrine Ifnb signaling prior to infection. The absence of Rela causes significant delays in Ifnb induction and consequently defective secondary antiviral gene expression. Rela maintains autocrine Ifnb signaling in uninfected cells, facilitates inflammatory and adaptive immune responses following infection, and promotes infected cell survival during this process. |
| 132298 | Rela | 21216972 | Rela is critical for pulmonary host defense during Streptococcus pneumoniae pneumonia in alveolar macrophages. During pneumococcal pneumonia, only the earliest induction of cytokines depends on transcription regulated by Rela in myeloid cells, and this transcriptional activity contributes to effective immunity. |
| 132298 | Rela | 21419662 | Rela is required for Il17a production in T cell in response to bacterial infection. Rela deficient T cells resulted in a diminished innate immune response to E. coli infection. |
| 132298 | Rela | 24023826 | Tnfaip3 is regulated by both Nf-Î°B and p38-dependent Cebpb in response to LPS in macrophages. |
| 132298 | Rela | 24656046 | The noncanonical NF Î°B pathway regulates histone modifications at the Ifnb1 promoter resulting in attenuated recruitment of Rela and histone demethylase, Kdm4a, to the Ifnb1 promoter. This provides a mechanism for regulating the induction of type I interferons . |
| 132298 | Rela | 25783839 | Lysophosphatidic acid plays an anti-inflammatory role in macrophages by diminishing lipopolysaccharide-induced phosphorylation of Mapk14 and Akt1, as well as Rela nuclear translocation. |
| 148423 | Il1rl1 | 22922442 | IL1RL1 (ST2) pre-treatment suppresses cytokine production and inhibits LPS signalling in dendritic cells. (Demonstrated in human) |
| 148423 | Il1rl1 | 25786179 | Cigarette smoke decreases Il1rl1 expression on group 2 innate lymphoid cells while elevating Il1rl1 expression on macrophages and natural killer cells, thus altering Il33 responsiveness within the lung to infection. |

| | | | |
|---|---|---|---|
| 55271 | HAVCR2 | 21637332 | HAVCR2 is constitutively expressed on human resting monocytes/macrophages and functions as a cap to block IL12, which is a key pro-inflammatory cytokine linking innate and adaptive immune responses. HAVCR2 plays a crucial role in the negative regulation of innate immune responses through crosstalk with PDCD1 and SOCS1 to limit STAT1 phosphorylation in HCV infection. |
| 55271 | HAVCR2 | 23967307 | TLR activation promotes HAVCR2 and LGALS9 association within the same macrophage to differentially regulate IL12/IL23 expressions via STAT3 phosphorylation. |
| 55271 | HAVCR2 | 25772938 | Hepatitis C virus (HCV)-induced, MIR155-regulated HAVCR2 expression regulates natural killer cell function, suggesting a novel mechanism for balancing immune clearance and immune injury during chronic viral infection. |
| 8510 | HIF1A | 18606657 | HIF1A transcription is induced by IFNA2 in human endothelial cells though a JAK-ISGF3 pathway under normoxic conditions, and that this response contributes to the anti-proliferative activity of this cytokine. |
| 8510 | HIF1A | 17965024 | HIF1A expression is regulated by intracellular calcium levels, resulting in modulation of PPP3CA (calcineurin) activity and RACK1 dimerization. |
| 8510 | HIF1A | 20517715 | Hypoxia-inducible factors (HIFs), including HIF1A, regulate glycolytic energy generation, optimize innate immunity, control pro-inflammatory gene expression, mediate bacterial killing and influence cell migration. HIFs contribute to inflammatory functions in various components of innate immunity, such as neutrophils, dendritic cells, mast cells, and epithelial cells. |
| 8510 | HIF1A | 20511350 | HIF1A, the most ubiquitously expressed hypoxia-inducible factor (HIF), in epithelial cells alters the lung's innate immune response and biases the tissue toward a Th2-mediated inflammation. |
| 8510 | HIF1A | 21685248 | HIF1A, under normoxic conditions, accumulates in dendritic cells via the TLR/MYD88/NFkB signalling pathway to induce a distinct subset of proinflammatory genes in comparison to hypoxia-induced HIF1A. (Demonstrated in murine model) |
| 8510 | HIF1A | 25746953 | HIF1A mediates the functional plasticity of monocytes during sepsis, wherein they transit from a pro-inflammatory to an immunosuppressive phenotype, while enhancing protective functions like phagocytosis, anti-microbial activity, and tissue remodelling . |
| 243385 | CHUK | 12351658 | CHUK is part of the inhibitor kappaB kinase (IKK) complex that phosphorylates IRS1 at Ser(312) and this contributes to the insulin resistance mediated by activation of inflammatory pathways. |
| 243385 | CHUK | 9891086 | CHUK (IKK-alpha) is part of the IKK signalosome that phosphorylates NFKBIA and NFKBIB, leading to activation of NF-kappaB. |

| | | | |
|---|---|---|---|
| 243385 | CHUK | 17434128 | CHUK (IKKalpha) phosphorylates CREBBP (CBP), regulating the CBP-mediated crosstalk between NF-kappaB and p53, a critical factor in the promotion of cell proliferation and tumor growth. |
| 243385 | CHUK | 20200270 | CHUK is a unique molecule involved in TLR7/9-MyD88-dependent type I IFN production through dendritic cell subset-specific mechanisms. |
| 243385 | CHUK | 21765415 | CHUK has a key role in the negative feedback of NF-kB canonical signalling by orchestrating the assembly of the A20 ubiquitin-editing complex to limit inflammatory gene activation in response to proinflammatory stimuli such as TNF and IL1. (Demonstrated in mouse) |
| 243385 | CHUK | 23422957 | CHUK is required in dendritic cells to prime adaptive immunity to Listeria monocytogenes. |
| 243385 | CHUK | 24323043 | GNB2L1 (RACK1) negatively regulates NF Î º B activation by interacting with CHUK and IKBKB. The interaction interferes with the recruitment of the IKK complex to TRAF2 . |
| 243385 | CHUK | 25740981 | DDX3X initiates a multifaceted cellular program involving dynamic associations with hepatitis C virus (HCV) RNA and proteins, CHUK, stress granules, and lipid droplet surfaces for its crucial role in the HCV life cycle. |
| 209781 | Mapk3 | 25538234 | Ptpn11 phosphatase function positively regulates Clec7a- and Itgam-stimulated reactive oxygen species production in macrophages by dephosphorylating and thus mitigating the inhibitory function of Sirpa and by promoting Mapk1/Mapk3 activation. |
| 209781 | Mapk3 | 25757564 | Prostaglandin E2 transactivates Csf1r and synergizes with its signalling at Mapk1/Mapk3 level in promoting macrophage migration. |
| 137672 | Mapk1 | 22447027 | Mapk1 (ERK) and Mapk14 (p38) control the dynamic balance regulating neutrophil migration. (Demonstrated in human) |
| 137672 | Mapk1 | 25538234 | Ptpn11 phosphatase function positively regulates Clec7a- and Itgam-stimulated reactive oxygen species production in macrophages by dephosphorylating and thus mitigating the inhibitory function of Sirpa and by promoting Mapk1/Mapk3 activation. |
| 137672 | Mapk1 | 25757564 | Prostaglandin E2 transactivates Csf1r and synergizes with its signalling at Mapk1/Mapk3 level in promoting macrophage migration. |
| 150998 | Csf1r | 25757564 | Prostaglandin E2 transactivates Csf1r and synergizes with its signalling at Mapk1/Mapk3 level in promoting macrophage migration. |
| 146254 | Traf2 | 22546736 | Traf2 deficiency results in the accumulation of Tnf-dependent, Il10-secreting neutrophils. Combined treatment of neutralizing antibodies against both Tnf and Il10 substantially ameliorated the colitis phenotype in the Traf2 null mice. |

| | | | |
|---|---|---|---|
| 146254 | Traf2 | 23951545 | The binding of MAVS to Traf2, Traf5, and Traf6 is dependent on virus infection and MAVS polymerization . The TRAF proteins promote ubiquitination that recruits IKBKG binding to the MAVS signalling complex. |
| 146254 | Traf2 | 25565375 | Traf2 mediates proteasome-dependent degradation of Irf5 and Rel as well as regulating macrophage polarization in tumour microenvironment and controlling tumour growth. |
| 146254 | Traf2 | 25752829 | Traf2-deficient macrophages produce reduced levels of inflammatory cytokines in response to lipopolysaccharide or flagellin stimulation and exhibit increased susceptibility to S. Typhimurium infection. |
| 135734 | Cfp | 25725105 | Cfp plays a role in intestinal homeostasis in response to an infectious challenge to activate Hc (C5a), which in turn provides protection through Il6 expression by the epithelium. |
| 163835 | Hc | 25725105 | Cfp plays a role in intestinal homeostasis in response to an infectious challenge to activate Hc (C5a), which in turn provides protection through Il6 expression by the epithelium. |
| 71390 | IL18 | 20195505 | IL18 and IL1B are important pro-inflammatory cytokines that activates monocytes, macropages, and neutrophils, as well as induce the Th1 and Th17 adaptive cellular responses. |
| 71390 | IL18 | 22940097 | IL18 secreted by inflammatory monocytes is critical for the differentiation of memory CD8(+) T and NK lymphocytes into antimicrobial effector cells. (Demonstrated in mice) |
| 71390 | IL18 | 25732728 | Primary Î³Î´ T cells provide an early source of IFNG during dengue virus (DV) infection and target DV-infected cells. Monocytes also participate as accessory cells that sense DV infection and amplify the cellular immune response in an IL18-dependent manner. |
| 152685 | Tlr3 | 21220319 | Tlr3 deletion dramatically enhanced the development of elastic lamina damage after collar-induced injury, indicating that Tlr3 signalling plays a protective role in arterial vessel wall. |
| 152685 | Tlr3 | 21367858 | Tlr3 activation by Poly(I:C) in the endothelial cells induces Poly(I:C) dose- and time-dependent cell apoptosis. Specifically, Tlr3 stimulation triggered the signalling of both extrinsic and intrinsic apoptotic pathways. |
| 152685 | Tlr3 | 21402738 | Tlr3 requires proteolytic processing in endolysosome by asparagine endopeptidase and cathepsin in the endolysosome to initiate signalling in response to DNA. |
| 152685 | Tlr3 | 21498625 | Tlr3 expression is inducible by LPS via Tlr4-Myd88-Irak-Traf6-NfkB dependent signalling pathway. (Demonstrated in human) |

| | | | |
|---|---|---|---|
| 152685 | Tlr3 | 21695051 | Tlr3 is necessary to establish an antiviral state in hepatocytes infected with hepatitis C Virus. HCV envelope proteins counteract the antiviral host defence by inhibiting the expression of Tlr3. (Demonstrated in human) |
| 152685 | Tlr3 | 22016778 | TLR3 signalling is enhanced by the presence of viral double-strand RNA-binding proteins. (Demonstrated in human) |
| 152685 | Tlr3 | 22072781 | Tlr3-Ticam1-mediated signalling pathway plays an essential role in the anti-viral response against poliovirus infection. |
| 152685 | Tlr3 | 22262694 | Tlr3 is constitutively expressed in spermatogonia and spermatocytes, and has the ability to activate anti-viral responses. |
| 152685 | Tlr3 | 22421964 | Upon engagement with its ligand, dsRNA, Tlr3 possesses the ability to recruit Casp8 and Ripk1 to induce apoptosis. (Demonstrated in human) |
| 152685 | Tlr3 | 22754655 | Activation of Tlr3 with poly(I:C) mediates antiviral immunity that diminishes coronavirus production in macrophages. |
| 152685 | Tlr3 | 22570612 | Upregulation of Tlr3 in intestinal epithelia during infancy may contribute to age-dependent susceptibility to rotavirus infection. |
| 152685 | Tlr3 | 22986631 | TLR3 activation differentially regulates phagocytosis of bacteria and apoptotic neutrophils by peritoneal macrophages. |
| 152685 | Tlr3 | 23142781 | Tlr3 contributes to the control of activated endogenous retroviruses (ERVs) and ERV-induced tumours. |
| 152685 | Tlr3 | 25736436 | Wdfy1 is a crucial adaptor protein in the Tlr3/4 signalling pathway. Wdfy1 interacts with Tlr3 and Tlr4 and mediates the recruitment of Ticam1 to these receptors. |
| 172895 | Wdfy1 | 25736436 | Wdfy1 is a crucial adaptor protein in the Tlr3/4 signalling pathway. Wdfy1 interacts with Tlr3 and Tlr4 and mediates the recruitment of Ticam1 to these receptors. |
| 157290 | Ear11 | 25713137 | Ear11 has prominent chemoattractant activity for F4/80(+)CD11c(-) tissue macrophages. |
| 166389 | Tnfrsf13c | 25673724 | Tnfrsf13c (BAFFR) expression is critical for innate immune activation and antiviral immunity. Tnfrsf13c deficiency results in reduced enforced viral replication, limited type I interferon production, and reduced adaptive immunity. |
| 149887 | Ptx3 | 21465531 | Ptx3 production is up-regulated in response to serum amyloid A, and contributes to the inflammatory pathogenesis of atherosclerosis. (Demonstrated in human) |
| 149887 | Ptx3 | 22278372 | Ptx3 forms a complex with components of neutrophil extracellular traps in septic patients. (Demonstrated in human) |

| | | | |
|---|---|---|---|
| 149887 | Ptx3 | 25679762 | Ptx3 acts as an extrinsic oncosuppressor gene by regulating Complement-dependent, macrophage-sustained, tumor-promoting inflammation. |
| 142284 | Osm | 25692402 | Stat3 is required for maximal Osm-induced lung Cxcl5 expression and subsequent neutrophil recruitment during bacterial pneumonia. |
| 178768 | Cxcl5 | 25692402 | Stat3 is required for maximal Osm-induced lung Cxcl5 expression and subsequent neutrophil recruitment during bacterial pneumonia. |
| 211637 | Stat3 | 21810606 | Stat3 is a negative regulator of type I IFN-mediated anti-viral responses. |
| 211637 | Stat3 | 23460517 | STAT3 mediates protein transport and secretion in hepatocytes during the acute phase of pneumonia. |
| 211637 | Stat3 | 24412612 | Stat3 mediates protection against intestinal infection by inducing innate lymphoid cell derived-Il22. |
| 211637 | Stat3 | 25692402 | Stat3 is required for maximal Osm-induced lung Cxcl5 expression and subsequent neutrophil recruitment during bacterial pneumonia. |
| 165511 | Atm | 25692705 | Unrepaired DNA lesions induce type I interferons via the Tmem173 pathway, resulting in enhanced anti-viral and anti-bacterial responses in Atm (-/-) mice. |
| 71032 | CXCR4 | 21094463 | CXCR4 is a chemokine receptor that is essential for homing of stem cells and more mature neutrophils to the bone marrow. |
| 71032 | CXCR4 | 25705792 | MIR146A upregulation by CXCR4 antagonist AMD3100 treatment or ZBTB16 silencing, decreases CXCR4 protein expression and prevents HIV-1 infection of leukemic monocytic cell line and CD4(+) T lymphocytes. |
| 72004 | ZBTB16 | 25705792 | MIR146A upregulation by CXCR4 antagonist AMD3100 treatment or ZBTB16 silencing, decreases CXCR4 protein expression and prevents HIV-1 infection of leukemic monocytic cell line and CD4(+) T lymphocytes. |
| 53480 | IL1RAPL1 | 25654981 | IL37 acts as an extracellular cytokine to inhibit innate inflammation by binding to IL18R1 and then using IL1RAPL1 for its anti-inflammatory properties. |
| 64199 | IL18R1 | 25654981 | IL37 acts as an extracellular cytokine to inhibit innate inflammation by binding to IL18R1 and then using IL1RAPL1 for its anti-inflammatory properties. |
| 66559 | IL1F7 | 20935647 | IL1F7 (IL-37) is a natural suppressor of innate inflammatory and immune responses. IL1F7 expression in macrophages or epithelial cells suppresses production of pro-inflammatory cytokines and the abundance of these cytokines increases with silencing of endogenous IL1F7 in human blood cells. |

| | | | |
|---|---|---|---|
| 66559 | IL1F7 | 21873195 | IL1F7 functions as a key modulator of intestinal inflammation where transgenic mice expressing IL1F7 subjected to dextran sulfate sodium-induced colitis showed reduced inflammation which was associated with decreased leukocyte recruitment into the colonic lamina propria. (Demonstrated in murine model) |
| 66559 | IL1F7 | 24481253 | The precursor and mature forms of IL37 are secreted from activated cells upon inflammasome activation and CASP1 processing of IL37 is important for its anti-inflammatory activity. |
| 66559 | IL1F7 | 25654981 | IL37 acts as an extracellular cytokine to inhibit innate inflammation by binding to IL18R1 and then using IL1RAPL1 for its anti-inflammatory properties. |
| 141954 | Sf3a1 | 25658809 | Sf3a1 affects the mRNA splicing of genes in the TLR signalling pathway to modulate the innate immune response in macrophages. |
| 17145 | CLEC6A | 20493731 | CLEC6A (Dectin-2) recognition of alpha-mannans and induction of Th17 cell differentiation is essential for host defence against Candida albicans. |
| 17145 | CLEC6A | 21267996 | CLEC6A, a C-type lectin receptor, is a pattern recognition receptor critical for immune responses to fungi. CLEC6A is coupled to SYK kinase and signals via CARD9 to activate NFKB, which in turns induces both innate and adaptive immunity. |
| 17145 | CLEC6A | 21357742 | CLEC6A is critical for the development of house dust mite (Dermatophagoides farinae) elicited eosinophilic and neutrophilic pulmonary inflammation. CLEC6A was also found to be crucial for the Th2 cytokine induction in the lungs and re-stimulated lymph nodes. |
| 17145 | CLEC6A | 21677049 | CLEC6A is expressed mainly in DCs and macrophages. CLEC6A recognizes alpha-mannans with its carbohydrate recognition domain and transduces signals through association with the ITAM-containing Fc receptor gamma chain, which recruits SYK and initiates the CARD9/NFkB signalling cascade. |
| 17145 | CLEC6A | 23911656 | CLEC4D and CLEC6A form a heterodimer complex which confers innate cells high ability to sense C. albicans infection by facilitating the activation of NFÎºB dependent inflammatory responses. |
| 17145 | CLEC6A | 25659141 | Plasmacytoid dendritic cells (pDCs) directly recognize Aspergillus fumigatus hyphae via CLEC6A; this interaction results in antifungal activity and the formation of pDC extracellular traps. |
| 162436 | Cd200r1 | 25654642 | Cd200-Cd200r1 pathway is an important regulator of antiviral immunity during cytomegalovirus infection that is exploited by murine cytomegalovirus to establish chronicity within mucosal tissue. |

| | | | |
|---|---|---|---|
| 162936 | Cd200 | 25654642 | Cd200-Cd200r1 pathway is an important regulator of antiviral immunity during cytomegalovirus infection that is exploited by murine cytomegalovirus to establish chronicity within mucosal tissue. |
| 28318 | ANO6 | 25651887 | Stimulation of P2RX7 receptors activates ANO6 to enhance bacterial phagocytosis and killing by macrophages. |
| 61253 | P2RX7 | 18765670 | P2X7 activity is modulated by human cathelicidin CAMP (LL-37) receptor in a structure-dependent manner and is involved in the proliferative cell response to CAMP. |
| 61253 | P2RX7 | 21988719 | P2RX7 activation in lipopolysaccharide (LPS)-primed myeloid cells results in secretion of pro-inflammatory cytokines IL1B and IL18. In addition, P2RX7 functions in the recognition and phagocytosis of non-opsonized bacteria and apoptotic cells. |
| 61253 | P2RX7 | 23479230 | P2RX7 engagement with ATP analog initiates cutaneous inflammation, dendritic cell differentiation, and induction of Th17 immunity. |
| 61253 | P2RX7 | 25651887 | Stimulation of P2RX7 receptors activates ANO6 to enhance bacterial phagocytosis and killing by macrophages. |
| 178353 | Zfp423 | 25554785 | The transcription factor Zfp423 is necessary for adipocyte activation and impaired adipogenesis is observed in Zfp423(nur12) mice. |
| 155648 | Rel | 25565375 | Traf2 mediates proteasome-dependent degradation of Irf5 and Rel as well as regulating macrophage polarization in tumour microenvironment and controlling tumour growth. |
| 132026 | Irf5 | 22412986 | Phosphorylation of Irf5 at ser451 and ser462 is the primary trigger of Irf5 function in nuclear accumulation, transcription and apoptosis. (Demonstrated in human) |
| 132026 | Irf5 | 25031348 | Irf5 modulates West Nile virus pathogenesis and host immune responses by shaping the early pro-inflammatory cytokine response in the draining lymph node. |
| 132026 | Irf5 | 25565375 | Traf2 mediates proteasome-dependent degradation of Irf5 and Rel as well as regulating macrophage polarization in tumour microenvironment and controlling tumour growth. |
| 98707 | SLC22A3 | 25561729 | SLC22A3 deficiency significantly decreases lipopolysaccharide- induced monocytic inflammatory response by interrupting NF-kB and MAPKs (mitogen-activated protein kinases) signalling cascades in a histamine dependent manner. |
| 48458 | TPP2 | 25525876 | TPPII controls the balance between intracellular amino acid availability, lysosome number, and glycolysis, which is vital for adaptive and innate immunity and neurodevelopmental health. |

| | | | |
|---|---|---|---|
| 44867 | EIF2AK2 | 12954221 | EIF2AK2 is a protein kinase that is regulated by RNA and is an important mediator of the antiviral and anti proliferative actions of interferon (IFN). |
| 44867 | EIF2AK2 | 12954221 | EIF2AK2 binds both SP1 and SP3 but only SP3 functions as part of the interferon-inducible complex with ISGF-3 proteins, consisting of STAT1, STAT2, and IRF9. |
| 44867 | EIF2AK2 | 19028691 | EIF2AK2 enhances the induction of interferon-beta and apoptosis mediated by cytoplasmic RNA sensors where in addition to MAVS and IRF3 but not TRIF, it is required for maximal type I IFN-beta induction and the induction of apoptosis by both transfected T7 phage polymerase-synthesized RNAs (PRNAs) and polyinosinic-polycytidylic acid. |
| 44867 | EIF2AK2 | 20485506 | EIF2AK2 is used by hepatitis C virus to restrain its ability to induce IFN through the RIG-I/MAVS pathway. |
| 44867 | EIF2AK2 | 22912779 | Antiviral stress granules containing DDX58 (RIG-I) and EIF2AK2 (PKR) have a critical role in viral detection and innate immunity. (Demonstrated in mouse) |
| 44867 | EIF2AK2 | 23115300 | Herpes simplex virus evades antiviral host defence by interacting with EIF2AK2 (PKR) to inhibit autophagy. |
| 44867 | EIF2AK2 | 23236554 | Bacterial dsRNA or poly(I:C) induces tyrosine phosphorylation of PKR activation by JAK kinases. |
| 44867 | EIF2AK2 | 23372823 | EIF2AK2 binds to dsRNA to inhibit IFN induction triggered by dengue virus. |
| 44867 | EIF2AK2 | 25520508 | EIF2AK2 (PKR) is recruited to stress granules by G3BP1 to promote innate immune responses at both transcriptional and translational levels. |
| 16084 | RPS6KA5 | 18690222 | RPS6KA5 negatively regulates TLR-pathway driven inflammation by preventing the binding of phosphorylated transcription factors CREB and ATF1 to IL10 and DUSP1 promoters. |
| 16084 | RPS6KA5 | 19922413 | RPS6KA5 regulates the transcription of IL1RA in response to TLR activation in macrophages. |
| 16084 | RPS6KA5 | 25520509 | Paramyxoviruses trigger the DNA-damage response, a pathway required for RPS6KA5 activation of phospho Ser 276 RELA formation to trigger the IRF7-DDX58 amplification loop necessary for mucosal interferon production. |
| 16268 | IFITM3 | 20064371 | IFITM3 is an antiviral restriction factor that mediates cellular resistance to influenza A H1N1 virus, West Nile virus, and Dengue virus. |
| 16268 | IFITM3 | 20601941 | IFITM3 is post-translationally regulated by S-palmitoylation on membrane-proximal cysteines, which controls its clustering in membrane compartments and its antiviral activity against influenza virus. |

| | | | |
|---|---|---|---|
| 16268 | IFITM3 | 25525793 | NRAV, a long noncoding RNA, modulates antiviral responses by negatively regulating the initial transcription of multiple critical interferon-stimulated genes, including IFITM3 and MX1, by affecting their histone modification. |
| 50271 | INSIG1 | 25526307 | Upon cytoplasmic DNA stimulation, the endoplasmic reticulum protein AMFR is recruited to and interacts with TMEM173 in an INSIG1-dependent manner. |
| 31785 | AMFR | 25526307 | Upon cytoplasmic DNA stimulation, the endoplasmic reticulum protein AMFR is recruited to and interacts with TMEM173 in an INSIG1-dependent manner. |
| 155976 | Eif4ebp2 | 25531441 | Genetic deletion of Eif4ebp1 or Eif4ebp2 potentiates innate antiviral immunity by enhancing translation of Irf7. |
| 145947 | Eif4ebp1 | 25531441 | Genetic deletion of Eif4ebp1 or Eif4ebp2 potentiates innate antiviral immunity by enhancing translation of Irf7. |
| 151723 | Anxa1 | 25533809 | Anxa1-/- mice are more susceptible to Mycobacterium tuberculosis infection as evidenced by a transient increase in pulmonary bacterial burden, exacerbated and disorganized granulomatous inflammation and impaired ability of Anxa1-/- dendritic cells to activate naÃ¯ve T cells. |
| 206000 | Sirpa | 25538234 | Ptpn11 phosphatase function positively regulates Clec7a- and Itgam-stimulated reactive oxygen species production in macrophages by dephosphorylating and thus mitigating the inhibitory function of Sirpa and by promoting Mapk1/Mapk3 activation. |
| 210531 | Itgam | 20639876 | Itgam (Cd11b integrin) is activated via Toll-like receptors (TLRs) and engages in crosstalk with the Myd88 and Ticam1 (TRIF) pathways inhibiting TLR signalling in innate immune responses. |
| 210531 | Itgam | 21245270 | Itgam :: Itgb2 is the principal leukocyte receptor involved in the recognition of the fungus Candida albicans. Recognition of Pra1p protein of C. albicans by Itgam :: Itgb2 plays a pivotal role in determining fungal virulence, and host response/protection against C. albicans infection. |
| 210531 | Itgam | 24423728 | Itgam (Cd11b) fine tunes the balance between adaptive and innate immune responses initiated by LPS by modulating the trafficking and signalling functions of Tlr4 in a cell-type-specific manner. |
| 210531 | Itgam | 25538234 | Ptpn11 phosphatase function positively regulates Clec7a- and Itgam-stimulated reactive oxygen species production in macrophages by dephosphorylating and thus mitigating the inhibitory function of Sirpa and by promoting Mapk1/Mapk3 activation. |

| | | | |
|---|---|---|---|
| 256772 | Clec7a | 21267996 | Clec7a, a C-type lectin receptor, is a pattern recognition receptor critical for immune responses to fungi. Clec7a is coupled to Syk kinase and signals via Card9 to activate NFKB, which in turns induces both innate and adaptive immunity. |
| 256772 | Clec7a | 21677049 | Clec7a is expressed mainly in DCs and macrophages. Clec7a recognizes beta-glucans with its carbohydrate recognition domain and transduces signals through its ITAM-like motif in the cytoplasmic region, which recruits Syk and initiates the Card9/NFkB signalling cascade. |
| 256772 | Clec7a | 22267217 | Clec7a acts as an extracellular sensor for fungi and mycobacteria that induce both Il1b production and maturation for protective immunity. (Demonstrated in human) |
| 256772 | Clec7a | 22543832 | The functional activity of Clec7a in mucosal immunity to Candida albicans is dependent on the genetic background of the host; this was specifically observed in two strains of mice, C57BL/6 and BALB/c. |
| 256772 | Clec7a | 23386437 | Clec7a is necessary for macrophage activation and resistance to pathogenic fungus Coccidioidesimmitis. |
| 256772 | Clec7a | 25538234 | Ptpn11 phosphatase function positively regulates Clec7a- and Itgam-stimulated reactive oxygen species production in macrophages by dephosphorylating and thus mitigating the inhibitory function of Sirpa and by promoting Mapk1/Mapk3 activation. |
| 196834 | Ptpn11 | 25538234 | Ptpn11 phosphatase function positively regulates Clec7a- and Itgam-stimulated reactive oxygen species production in macrophages by dephosphorylating and thus mitigating the inhibitory function of Sirpa and by promoting Mapk1/Mapk3 activation. |
| 177236 | Irak4 | 20595480 | IRAK4-mediated innate immune inflammatory responses play critical roles in divergent clinical outcomes in murine malaria models when Irak4 (-/-) mice were used to study two experimental models of malaria. |
| 177236 | Irak4 | 24717937 | Irak4 activation is impaired during endotoxin tolerization; a process which impairs the production of LPS-induced pro-inflammatory cytokines without inhibition expression of anti-inflammatory or anti-microbial mediators. |
| 177236 | Irak4 | 23027530 | Irak4 deficient mice show impaired innate immunity, leading to defective Type 1 T-cell responses, B-cell expansion, and are more susceptible to Toxoplasma gondii infection. |
| 177236 | Irak4 | 24717937 | Mir302b expression is up-regulated upon bacterial infection and is a crucial regulator of NFÎºB signalling by directly targeting IRAK4 |

| | | | |
|---|---|---|---|
| 177236 | Irak4 | 25548221 | Irak4 is a molecular target of chlorogenic acid in the treatment of innate immunity-related shock and organ dysfunction following insult of various Toll-like receptor pathogens from bacteria and viruses. |
| 212533 | Cd40 | 25549946 | Tmem126a upregulates genes involved in antigen presentation; such as Icam1, MHC II, Cd86 and Cd40, via the Tlr4 signal transduction pathway. |
| 158071 | Cd86 | 25549946 | Tmem126a upregulates genes involved in antigen presentation; such as Icam1, MHC II, Cd86 and Cd40, via the Tlr4 signal transduction pathway. |
| 138668 | Icam1 | 25549946 | Tmem126a upregulates genes involved in antigen presentation; such as Icam1, MHC II, Cd86 and Cd40, via the Tlr4 signal transduction pathway. |
| 198859 | Tmem126a | 25549946 | Tmem126a upregulates genes involved in antigen presentation; such as Icam1, MHC II, Cd86 and Cd40, via the Tlr4 signal transduction pathway. |
| 26364 | TP53 | 21483755 | TP53 binds to canonical and non-canonical promoter regions of the human TLR gene family and up-regulates the expression of TLRs. The activation of TP53 can directly influence the TLR-mediated induction of cytokines. |
| 26364 | TP53 | 22105999 | TP53 serves as a host antiviral factor and enhances both the innate and adaptive immune responses to influenza A virus. (Demonstrated in mice) |
| 26364 | TP53 | 25505178 | Coronavirus engages papain-like proteases to escape from the innate antiviral response of the host by inhibiting TP53-IRF7-IFNB1 signalling. |
| 178961 | Atg7 | 25512546 | Atg7 plays an essential role for autophagy during invariant natural killer T cell development. |
| 138680 | Arl5b | 25451939 | Arl5b negatively regulates the antiviral innate immune response by binding to Ifih1 and prevents the subsequent interaction of Ifih1 to poly(I:C). |
| 282076 | MIR548G | 25499200 | Overexpression of MIR548G suppresses multiplication and translation of dengue virus (DENV) 1, 2, 3 and 4. |
| 102654 | S100A8 | 12626582 | S100A8 is an intracellular calcium-binding protein that promotes neutrophil/monocyte recruitment at inflamed tissues by enhancing attachment to endothelial cells. |
| 102654 | S100A8 | 18714033 | S100A8 is a myeloid-related protein that rapidly modulates macrophage nitric oxide production during innate immune response. |
| 102654 | S100A8 | 20555353 | S100A8 (calgranulin A) and S100A9 (calgranulin B) form an antimicrobial heterodimeric complex known as calprotectin. Bacterial flagellin induces the upregulation of S100A8/S100A9 heterodimer via a TLR5-dependent mechanism in epidermal keratinocytes. |
| 102654 | S100A8 | 21239714 | S100A8 forms a complex with S100A9 and the complex is the site of interplay between extracellular Ca(2+) entry and intra-phagosomal reactive oxygen species production. S100A8 :: S100A9 acts as Ca(2+) sensor in phagosomal ROS production. |

| | | | |
|---|---|---|---|
| 102654 | S100A8 | 22423963 | S100A8 is strongly upregulated in neutrophils upon bacterial infection, and sequesters zinc as a mechanism of nutritional immunity. Salmonella typhimurium overcomes this defence mechanism by expressing a high affinity zinc transporter. (Demonstrated in mice) |
| 102654 | S100A8 | 23431180 | S100A8::S100A9 heterodimer sequesters Mn(2+) and Zn(2+) to starve bacteria of these essential nutrients. |
| 102654 | S100A8 | 25505274 | The TLR4/S100A8 axis is important in the activation of monocytes. |
| 90504 | IL17A | 19144317 | IL17A is a cytokine produced by T helper 17 (Th17) cells that plays important roles in the development of inflammatory diseases. |
| 90504 | IL17A | 20083670 | IL17A is an innate-adaptive immunomodulatory cytokine that is produced by gammadelta cells and is a key mediator for the innate immune response to urinary tract infections (UTIs) caused by uropathogenic Escherichia coli. |
| 90504 | IL17A | 21074482 | IL17A is a Th17-related cytokine, traditionally thought of as an adaptive responder, has been shown to have various innate sources and functions as a rapidly produced pro-inflammatory mediator. Innate IL17A-producing cells also employ many of the cytokine and transcriptional regulators utilized by Th17 cells. |
| 90504 | IL17A | 21822258 | IL17A signalling enhances the mRNA stability of chemokine CXCL1 through TRAF3IP2, TRAF2-TRAF5 and the RNA-binding protein SRSF1. |
| 90504 | IL17A | 22384827 | IL17A is significantly upregulated in both S. pyogenes inoculated and mock inoculated mice, indicating that the cytokine production can be triggered by inoculation trauma alone. (Demonstrated in mice) |
| 90504 | IL17A | 24194936 | Local production of IL17A in the airways drives early neutrophil infiltration into respiratory syncytial virus infected infant lungs. |
| 90504 | IL17A | 25474109 | IFNG interferes with the IL-1/NFKBIZ axis in Î²-glucan-activated dendritic cells and promotes T cell-mediated immune responses with increased release of IFNG and IL22, and diminished production of IL17A. |
| 96992 | IL22RA2 | 25476703 | IL22 protects against and IL22RA2 aggravates liver fibrosis and cirrhosis in chronic liver infections. |
| 178955 | Cxcl2 | 21757770 | Cxcl2 is a chemokine secreted by kidney dendritic cells to recruit neutrophils to sites of uropathogenic bacterial infection. |
| 178955 | Cxcl2 | 25447519 | Cebpa suppresses granule formation in mast cells and increases Cxcl2 production from mast cells upon bacterial stimulation. |
| 173713 | Cebpa | 21326902 | Cebpa is a member of the CCAAT enhancer binding protein family and is a transcriptional factor regulating genes in innate immunity and inflammation. The activities of CEBP are regulated via methylation of arginine and lysine side chains. |

| | | | |
|---|---|---|---|
| 173713 | Cebpa | 25447519 | Cebpa suppresses granule formation in mast cells and increases Cxcl2 production from mast cells upon bacterial stimulation. |
| 13675 | TLR6 | 15661917 | TLR6 co-expressed withTLR2 at the cell surface is crucial for recognition of diacylated lipopeptide and peptidoglycan derived from mycoplasma and to activate the NF-kappaB signalling cascades in human cells. |
| 13675 | TLR6 | 12697090 | TLR6 and TLR1 are involved in the discrimination of a subtle difference between triacyl and diacyl lipopeptides through interaction with TLR2. |
| 13675 | TLR6 | 15690042 | TLR6/2 heterodimer signalling is used by CD36, a selective and non-redundant sensor of microbial diacylglycerides. |
| 13675 | TLR6 | 20037584 | TLR6-CD36-TLR4 activation is a common molecular mechanism by which atherogenic lipids and amyloid-beta stimulate sterile inflammation. |
| 13675 | TLR6 | 21482737 | TLR2::TLR6 synergistically interacts with TLR9 in lung epithelium to induce rapid pathogen killing, and can be used as a therapeutic target to treat otherwise lethal pneumonia. |
| 13675 | TLR6 | 25456159 | Cutaneous bacteria can negatively regulate skin-driven immune responses by inducing Gr1(+)CD11b(+) myeloid-derived suppressor cells via TLR2-6 activation. |
| 206490 | Cxcr6 | 25456160 | Cxcl16-Cxcr6 crosstalk coordinates the intestinal topography of Il22 secretion required for mucosal defence against Citrobacter rodentium infection. |
| 194380 | Cxcl16 | 20874518 | Cxcl16 is a chemoattractant in cerebrospinal fluid in early pneumococcal meningitis where Cxcl16 was found to be upregulated in RAW264.7 macrophages (but not in neutrophils and endothelial cells) upon pneumococcal stimulation. Cxcl16 upregulation in vivo was dependent on Toll-like receptor (TLR) 2/TLR4 and MyD88 signaling. |
| 194380 | Cxcl16 | 25456160 | Cxcl16-Cxcr6 crosstalk coordinates the intestinal topography of Il22 secretion required for mucosal defence against Citrobacter rodentium infection. |
| 6012 | DEFA6 | 19024344 | DEFA6 (HD-6) and DEFA5 (HD-5) are two Paneth cell alpha-defensins found in the gut and alpha-defensins have multiple functions in the immune system. |
| 6012 | DEFA6 | 21560070 | DEFA6 is secreted by paneth cells in the small intestine and is secreted in response to cholinergic and microbial stimuli. DEFA5A confer immunity to oral infection by Salmonella and is a major determinant of the small intestinal microbiome composition. |

| | | | |
|---|---|---|---|
| 6012 | DEFA6 | 22722251 | DEFA6 is involved in mucosal innate immunity by protecting the small intestine against invasion by diverse enteric pathogens. In response to bacterial surface proteins, DEFA6 undergoes ordered self-assembly to form fibrils and nanonets that surround and entangle bacteria. |
| 6012 | DEFA6 | 25433720 | NOD2 plays a role in intestinal innate immunity by regulating the expression of DEFA5 and DEFA6 through the NF-kB and MAPK pathways. |
| 6199 | DEFA5 | 19024344 | DEFA5 and DEFA6 are two Paneth cell alpha-defensins found in the gut that have multiple functions in the immune system. |
| 6199 | DEFA5 | 21468224 | DEFA5 expression in mice showed significant loss of segmented filamentous bacteria, thus demonstrating a novel role for Paneth cell defensins in intestinal homeostasis by regulating the small intestinal microbiome. |
| 6199 | DEFA5 | 21560070 | DEFA5 is secreted by paneth cells in the small intestine and is secreted in response to cholinergic and microbial stimuli. DEFA5A confer immunity to oral infection by Salmonella and is a major determinant of the small intestinal microbiome composition. |
| 6199 | DEFA5 | 25433720 | NOD2 plays a role in intestinal innate immunity by regulating the expression of DEFA5 and DEFA6 through the NF-kB and MAPK pathways. |
| 30654 | NOD2 | 17690884 | NOD2 and NOD1 represent central players in the control of the immune responses to bacterial infections and inflammation. |
| 30654 | NOD2 | 18240302 | NOD2 is an intracellular receptor of muramyl dipeptide (MDP), a component of peptidoglycan present in the cell wall of Gram-positive (G+) and Gram-negative (G-) bacteria. |
| 30654 | NOD2 | 17705131 | NOD2 and NOD1 can induce CCL5 (RANTES) through NF-kappaB pathway, orchestrating the global Nod-dependent immune defence during bacterial infections. |
| 30654 | NOD2 | 19541630 | NOD2-dependent recognition of S. aureus and muramyl dipeptide is facilitated by alpha-toxin (alpha-hemolysin), a pore-forming toxin and virulence factor of the pathogen and is dependent on IL-1beta-amplified production of IL-6. |
| 30654 | NOD2 | 19898471 | NOD2 and NOD1 direct autophagy by recruiting ATG16L1 to the plasma membrane at the site of bacterial entry. |
| 30654 | NOD2 | 19966812 | NOD2 stimulation induces autophagy in dendritic cells influencing bacterial handling and antigen presentation. |
| 30654 | NOD2 | 20200479 | NOD2 recruits the critical autophagy protein ATG16L1 to the plasma membrane during bacterial invasion. |

| | | | |
|---|---|---|---|
| 30654 | NOD2 | 19701189 | NOD2 functions as a cytoplasmic viral pattern-recognition receptor (PRR) and activates an innate immune responses to viral ssRNA by triggering activation of interferon-regulatory factor 3 (IRF3) and production of interferon-beta (IFNB1). |
| 30654 | NOD2 | 21199260 | NOD2 is both a positive and negative regulator of TLR4 - the effect it exerts is dependent on the presence of MDP. NOD2 upon engagement with its ligand, MDP, positively regulates TLR4-mediated signalling; in the absence of MDP, NOD2 negatively regulates the TLR4 pathway. (Demonstrated in murine model) |
| 30654 | NOD2 | 21236705 | NOD2 is a peripheral peptidoglycan intracellular sensor and is important for the progression and pathogenesis of experimental autoimmune encephalomyelitis (animal model of multiple sclerosis). |
| 30654 | NOD2 | 21108472 | NOD2 detects heat-killed Legionella pneumophila and stimulates NFkB and IFN-beta promoter activity. NOD2 deficiency results in increased proinflammatory cytokine expression at 4hrs and greater neutrophil recruitment to the lung. (Demonstrated in murine model) |
| 30654 | NOD2 | 21690088 | DDX58 and NOD2 colocalize to cellular ruffles and cell-cell junctions to form a protein complex via the CARD domains. DDX58 negatively regulates ligand-induced NFkB signalling mediated by NOD2, and conversely, NOD2 negatively regulates type I interferon induction by DDX58. |
| 30654 | NOD2 | 22750073 | NOD2 recognition of muramyl dipeptide, a component of bacterial cell walls, improves the barrier function of intestinal epithelial cells. (Demonstrated in mice) |
| 30654 | NOD2 | 22531915 | NOD2 enhances the innate immune response of alveolar macrophages to Mycobacterium tuberculosis in human. |
| 30654 | NOD2 | 24670424 | Following NOD2 activation, IRF4 interacts with MYD88, TRAF6, and RIPK2 and downregulates K63-linked polyubiquitinylation of RICK and TRAF6 leading to disruption of NFkB activation pathways. |
| 30654 | NOD2 | 25433720 | NOD2 plays a role in intestinal innate immunity by regulating the expression of DEFA5 and DEFA6 through the NF-kB and MAPK pathways. |
| 148186 | Il1r2 | 25461404 | Adam17 affects sensitivity to interleukin-1 by changing the balance between Il1r1 and Il1r2 receptors. |
| 148254 | Il1r1 | 22426547 | Il1r1 is the primary receptor for the inflammatory cytokine Il1b. (Demonstrated in human) |
| 148254 | Il1r1 | 23440414 | IL1R1 upregulates Mir135b as a negative feedback regulatory mechanism to resolve cigarette smoke-induced inflammation in the lung. |

| | | | |
|---|---|---|---|
| 148254 | Il1r1 | 24651866 | In response to adenovirus infection, the IL1A-IL1R1-CXCR2 signalling axis cooperates with complement to recruit Ly-6G+7/4+ polymorphonuclear leukocytes to the splenic marginal zone (MZ) in the proximity of virus-containing MARCO+ residential MZ macrophages, which are subsequently eliminated. |
| 148254 | Il1r1 | 25461404 | Adam17 affects sensitivity to interleukin-1 by changing the balance between Il1r1 and Il1r2 receptors. |
| 130508 | Adam17 | 25461404 | Adam17 affects sensitivity to interleukin-1 by changing the balance between Il1r1 and Il1r2 receptors. |
| 42125 | IRF1 | 12759449 | IRF1 is required for the up-regulation of the CD40-NF-kappaB activator 1 (TRAF3IP2 ) axis during airway inflammation. |
| 42125 | IRF1 | 12420214 | IRF1 is controlled by two distinct signalling pathways; a JAK/STAT-signalling pathway in viral infected cells and an ATM-signalling pathway in DNA damaged cells. |
| 42125 | IRF1 | 3409321 | IRF1 specifically binds to the upstream regulatory region of the human IFN-beta (IFNB1) gene and mediates IFNB1 virus-induced transcription and is involved in the regulation of other genes such as IFN-alpha and MHC class I genes. |
| 42125 | IRF1 | 20308629 | IRF1 mediated type I IFN independent mechanism of enhanced RSAD2 (viperin) expression provides a redundant mechanism to protect cells from viral infections. |
| 42125 | IRF1 | 21097874 | IRF1 transcriptionally inhibits the IL23A through the ISRE element and reduce the severity of chronic intestinal inflammation caused by LPS (shown in mice). |
| 42125 | IRF1 | 22266972 | IRF1 promotes immune cell apoptosis and inhibits autophagy in a murine endotoxemia model. (Demonstrated in mice) |
| 42125 | IRF1 | 25461762 | MIR23A contributes to human herpes simplex virus type 1 replication through the regulation of the IRF1-mediated antiviral signal pathway. |
| 126931 | MIR23A | 25461762 | MIR23A contributes to human herpes simplex virus type 1 replication through the regulation of the IRF1-mediated antiviral signal pathway. |
| 171492 | Il15 | 22084435 | Il15 regulates homeostasis and terminal maturation of NKT cells. |
| 171492 | Il15 | 22940097 | IL15 secreted by inflammatory monocytes is critical for the differentiation of memory CD8(+) T and NK lymphocytes into antimicrobial effector cells. |
| 171492 | Il15 | 25466888 | Cxcr3 expression in innate CD8+ T cells defines protective antibacterial and cancer immunity upon Il15 stimulation. |
| 197156 | Il28ra | 25431490 | Il28ra (Ifnlr1), Stat1 and Irf3 are required for antibiotics to prevent persistent murine norovirus infection. |

| | | | |
|---|---|---|---|
| 197890 | Nlrc4 | 22174673 | The Nlrc4 inflammasome is important for control of mucosal Candida infection, impacting on inflammatory cell recruitment to infected tissues, as well as protecting against the systemic dissemination of infection. |
| 197890 | Nlrc4 | 22231517 | Flagellin-induced Nlrc4 inflammasome activation in splenic dendritic cells triggers antigen-independent IFN-gamma production by memory T cells. |
| 197890 | Nlrc4 | 22547706 | Nlrc4 is important for host survival and bacterial clearance, as well as neutrophil-mediated inflammation in the lungs following Klebsiella pneumoniae infection. |
| 197890 | Nlrc4 | 22484733 | Nlrc4-dependent production of Il1b by intestinal phagocytes is a mechanism that discriminates pathogenic from commensal bacteria in the intestinal host defence. |
| 197890 | Nlrc4 | 22885697 | Nlrc4 Ser â € 533 phosphorylation is essential for procaspase-1 recruitment to the Nlrc4 inflammasome complex after S. typhimurium infection. |
| 197890 | Nlrc4 | 23355222 | Microglial cells employ the NAIP5/NLRC4 inflammasome to monitor and clear central nervous system infections by flagellated bacteria. |
| 197890 | Nlrc4 | 25395539 | Flagellin induces Tlr5-dependent Il22 production and Nlrc4-dependent Il18 production to promote a protective gene expression program in intestinal epithelial cells and elimination of rotavirus-infected cells. |
| 197890 | Nlrc4 | 25422455 | Actin polymerization is required for Nlrc4-dependent regulation of intracellular bacterial burden, inflammasome assembly, pyroptosis, and Il1b production. |
| 149298 | Nod1 | 17705131 | Nod1 and Nod2 activation results in substantial secretion of Ccl5 by murine macrophages and induces binding of NF-kappaB subunits to Ccl5 promoter. |
| 149298 | Nod1 | 20389019 | Nod1 can activate the ISGF3 signaling pathway that is usually associated with protection against viral infection to provide mice with robust type I IFN-mediated protection from H. pylori and possibly other mucosal infections. |
| 149298 | Nod1 | 20685341 | Nod1 and Nod2 account for neutrophil recruitment to the lungs of mice infected with Legionella pneumophila. |
| 149298 | Nod1 | 21072876 | Nod1 and Nod2 can detect Legionella pneumophila and these receptors modulate the in vivo pulmonary immune response differently. |

| | | | |
|---|---|---|---|
| 149298 | Nod1 | 21236705 | Nod1 is a peripheral peptidoglycan intracellular sensor and is important for the progression and pathogenesis of experimental autoimmune encephalomyelitis (animal model of multiple sclerosis). |
| 149298 | Nod1 | 21108472 | Nod1 detects heat-killed Legionella pneumophila and stimulates NFkB and IFN-beta promoter activity. Nod1 deficiency results in impaired bacterial clearance and increased proinflammatory cytokine at 24hrs post-infection. |
| 149298 | Nod1 | 21677137 | Nod1 is expressed by trophoblast cells across gestation and may have a role in mediating infection-associated inflammation and prematurity. Activation of Nod1 by bacterial peptidoglycan-derived peptide induces maternal-fetal inflammation and preterm labour. |
| 149298 | Nod1 | 21715553 | Nod1 KO mice were protected from high-fat diet induced inflammation, lipid accumulation, and peripheral insulin intolerance. Ex vivo, Nod1 activation by bacterial peptidoglycan mimetics induces proinflammatory cytokine secretion and impaired insulin-stimulated glucose uptake in adipocytes. Hence, Nod1 is a plausible, new link between innate immunity and metabolism. |
| 149298 | Nod1 | 24163408 | Nod1 and Nod2 synergize with Tlr4 in dendritic cells to increase IL12 production and enhance invariant natural killer T (iNKT) cell activation, and are important regulators of the IFN gamma response by iNKT cells during S. typhimurium and L. monocytogenes infections. |
| 149298 | Nod1 | 25423082 | Salmonella enterica serovar Typhimurium Î [illegible]msbB that possesses a modified lipid A triggers exacerbated colitis in the absence of Nod1 and/or Nod2, which is likely due to increased Tlr2 stimulation. |
| 3485 | SMAD4 | 25410655 | TGF-Î² signalling upregulates MIR181A2 expression through SMAD3/4-dependent promoter activation. |
| 18366 | SMAD3 | 25410655 | TGF-Î² signalling upregulates MIR181A2 expression through SMAD3/4-dependent promoter activation. |
| 126715 | MIR181A2 | 25410655 | TGF-Î² signalling upregulates MIR181A2 expression through SMAD3/4-dependent promoter activation. |
| 87415 | LCN2 | 25398327 | LCN2 is secreted by the urinary tract mucosa in response to uropathogenic E. coli challenge and acts in innate immune defences as a colonization barrier. |
| 168769 | Tyrobp | 25402298 | Tyrobp impairs host defence during pneumococcal pneumonia at the primary site of infection by inhibiting phagocytosis by alveolar macrophages. |
| 151419 | Relb | 21419662 | Relb is required for Il17a production in T cell in response to bacterial infection. Relb deficient T cells resulted in a diminished innate immune response to E. coli infection. |
| 151419 | Relb | 25367573 | Ripk3 has a novel function in NF-kB activation, dendritic cell biology, innate inflammatory-cytokine expression, and injury-induced tissue repair. |

| | | | |
|---|---|---|---|
| 195445 | Nfkb1 | 21343618 | Nfkb1 enforces specificity of cellular response to pathogens by binding to a subset of IRE sequences in IFN-inducible genes. Nfkb1 deficiency results in the inappropriate production of Ifnb in response to bacterial DNA sensed by Tlr9. |
| 195445 | Nfkb1 | 24270517 | Mir126-Kdr axis is an important regulator of the innate response. Mir126 controls the survival and function of plasmacytoid dendritic cells and regulates gene expression of Tlr7, Tlr9, Nfkb1 and Kdr. |
| 195445 | Nfkb1 | 25367573 | Ripk3 has a novel function in NF-kB activation, dendritic cell biology, innate inflammatory-cytokine expression, and injury-induced tissue repair. |
| 165974 | Ripk3 | 22123964 | Ripk3 forms a complex with Ticam1 upon Toll-like receptors (TLR) 3 and 4 activation resulting in Ripk3-dependent but TNF-independent necrosis in macrophages. |
| 165974 | Ripk3 | 22423968 | Ripk3 interacts with Zbp1 to mediate virus-induced necrosis. |
| 165974 | Ripk3 | 25367573 | Ripk3 has a novel function in NF-kB activation, dendritic cell biology, innate inflammatory-cytokine expression, and injury-induced tissue repair. |
| 75609 | ATF2 | 25368329 | SCN5A is a novel pathogen sensor that initiates anti-viral signalling and transcription through ADCY8 and ATF2. |
| 35780 | ADCY8 | 25368329 | SCN5A is a novel pathogen sensor that initiates anti-viral signalling and transcription through ADCY8 and ATF2. |
| 26059 | SCN5A | 25368329 | SCN5A is a novel pathogen sensor that initiates anti-viral signalling and transcription through ADCY8 and ATF2. |
| 29564 | ECSIT | 10465784 | ECSIT is specific for the Toll/IL-1 pathways and is a regulator of MAP3K1 (MEKK1) processing. |
| 29564 | ECSIT | 25371197 | ECSIT binds to MAP3K7 and TRAF6 to form a complex that plays a pivotal role in activating TLR4-mediated NF-kB signalling. |
| 166877 | Slc11a1 | 25350459 | Slc11a1 confers temporal and anatomical host resistance to chronic Salmonella infection. |
| 74471 | USP2 | 25070846 | USP2 deubiquitinates K63-linked polyubiquitin chains from TBK1 to terminate TBK1 activation and negatively regulate IFNB1 signalling and antiviral immune response. |
| 139889 | Prl | 25049387 | Pituitary hormone prolactin (Prl) constrains tumor-promoting liver inflammation by inhibiting Map3k1-dependent activation of Myc at the level of the trafasome. |

| | | | |
|---|---|---|---|
| 144556 | Myc | 25049387 | Pituitary hormone prolactin (Prl) constrains tumor-promoting liver inflammation by inhibiting Map3k1-dependent activation of Myc at the level of the trafasome. |
| 96496 | ARG1 | 18978793 | ARG1 expression induced by intracellular pathogens in mouse classically activated macrophages (CAMs) through the TLR pathway, suppressing nitric oxide production and thwarting effective immunity independent of the STAT6 pathway. |
| 96496 | ARG1 | 25015834 | PI3K/PTEN-regulated extracellular ARG1 acts as a paracrine regulator of inflammation and immunity. Demonstrated in mice. |
| 81409 | PTEN | 25015834 | PI3K/PTEN-regulated extracellular ARG1 acts as a paracrine regulator of inflammation and immunity. Demonstrated in mice. |
| 133881 | Pik3cg | 25022365 | Rab8a interacts with Pik3cg to regulate Akt signalling generated by surface Tlr4. |
| 167493 | Rab8a | 25022365 | Rab8a interacts with Pik3cg to regulate Akt signalling generated by surface Tlr4. |
| 77617 | STAT1 | 17971840 | STAT1 mediates IFNG cytokine signalling, and as a member of the STAT family protein, STAT1 has a significant impact on the innate immunity during sepsis. |
| 77617 | STAT1 | 18678606 | STAT1 serine phosphorylation is induced by TNF-alpha and PGE2 and this activates expression of the STAT1 and NF-kappaB target gene IFN regulatory factor 1 (IRF1), which contributes to IFN responses. |
| 77617 | STAT1 | 7657660 | STAT1 phosphorylation and DNA binding activity is under the control of Janus protein-tyrosine kinases (JAKs) and the epidermal growth factor receptor (EGFR). |
| 77617 | STAT1 | 16481475 | Acetylation of STAT1 modulates NF-kappaB activity and thus ultimately apoptosis where, as a result, RELA DNA binding, nuclear localization, and expression of anti-apoptotic NF-kappaB target genes decrease. |
| 77617 | STAT1 | 8943351 | STAT1 is part of the interferon-stimulated gene factor 3 (ISGF3) transcription complex which is composed of a STAT1:2 heterodimer and a weak DNA-binding protein, IRF9. |
| 77617 | STAT1 | 20829351 | STAT1 transcription factor enhances TLR8 functionality by binding of to GAS elements on the TLR8 promoter in an IFN-gamma-dependent manner. |
| 77617 | STAT1 | 22065572 | STAT1 phosphorylation at Ser708 is a key event in the IFN signalling pathway that imparts anti-viral immunity to restrict West Nile virus infection. (Demonstrated in mice) |
| 77617 | STAT1 | 22425562 | Histone deacetylase inhibitors prevent IFNG-mediated phosphorylation of STAT1. |

| | | | |
|---|---|---|---|
| 77617 | STAT1 | 24449862 | TNK1 is a component of the IFN-JAK-STAT signalling cascade and is a critical antiviral host factor where its abundance is inversely correlated to viral replication and contributes to the hepatocytic response to antiviral treatment. |
| 77617 | STAT1 | 25027037 | STAT1 is directly recruited to TRAF6, demonstrating cross-talk between the TLR and JAK/STAT signalling pathways, and this direct activation of STAT1 by TLR signalling suggests a crucial role for STAT1 in TLR-induced inflammation. Demonstrated in mice. |
| 41523 | PRKX | 25028512 | MAP3K7 (TAK1) Ser412 phosphorylation is regulated by PRKACA and PRKX, and is essential for proper signalling, as well as proinflammatory cytokine induction by TLR/IL-1R activation. |
| 32929 | PRKACA | 25028512 | MAP3K7 (TAK1) Ser412 phosphorylation is regulated by PRKACA and PRKX, and is essential for proper signalling, as well as proinflammatory cytokine induction by TLR/IL-1R activation. |
| 189693 | S1pr1 | 25024218 | T-cell-intrinsic Mir155 is required for type-2 immunity, in part through regulation of S1pr1, whereas T-cell-intrinsic Mir146 is required to prevent overt Th1/Th17 skewing. |
| 24257 | CXCL2 | 21757770 | CXCL2 is a chemokine secreted by kidney dendritic cells to recruit neutrophils to sites of uropathogenic bacterial infection. (Demonstrated in mouse) |
| 24257 | CXCL2 | 25005359 | Secreted CCNA2 (CCN1) promotes anti-inflammatory cytokine IL10 release from epithelial cells via integrin Î±VÎ²6-PKC, and this subsequently suppresses TNF, CXCL2 and neutrophil infiltration in the lungs. |
| 36372 | CCNA2 | 25005359 | Secreted CCNA2 (CCN1) promotes anti-inflammatory cytokine IL10 release from epithelial cells via integrin Î±VÎ²6-PKC, and this subsequently suppresses TNF, CXCL2 and neutrophil infiltration in the lungs. |
| 126971 | MIR124-1 | 24995397 | MIR124-1 plays a negative regulatory role in fine-tuning the inflammatory response in alveolar macrophages upon mycobacterial infection, in part by directly targetting TLR signalling. |
| 66003 | PIK3CA | 17827709 | PIK3CA, as the alpha subunit of PI3K, functions as a negative regulator of TLR signalling. The activation of PIK3CA results in the inhibition of pro-inflammatory events such as expression of IL12 and TNFA. |
| 66003 | PIK3CA | 20953381 | PIK3CA and PIK3CB isoforms of class IA phosphatidylinositol 3-kinase (PI3K) are both required for the pro-inflammatory response to flagellin. |
| 66003 | PIK3CA | 24996183 | MIR203A accelerates apoptosis in LPS-stimulated alveolar epithelial cells by targeting PIK3CA. |
| 126601 | MIR203 | 22917968 | MIR203A post-transcriptionally regulate the pro-inflammatory cytokines TNFalpha and IL24 in keratinocytes. |
| 126601 | MIR203 | 23785202 | MIR203A can be induced by IFN in virally infected cells to dampen IFN anti-viral signalling. |

| | | | |
|---|---|---|---|
| 126601 | MIR203 | 24996183 | MIR203A accelerates apoptosis in LPS-stimulated alveolar epithelial cells by targeting PIK3CA. |
| 189794 | Trem1 | 21555403 | Trem1 expression is upregulated following Pseudomonas aeruginosa infection in the cornea and the inhibition of Trem1 reduces the severity of corneal disease. Trem1 acts as an inflammatory amplifier in P. aeruginosa keratitis by modulating TLR signalling and Th1/Th2 responses. |
| 189794 | Trem1 | 21690199 | Trem1 expression is induced by vitamin D3 in human bronchial epithelial cells. Activation of TREM1 leads to the induction of human beta defensin 2 and TNF-alpha mRNA in the airway epithelium. (Demonstrated in human) |
| 189794 | Trem1 | 22459945 | Trem1 mediates endotoxin tolerance in monocytes through its ability to induce anti- or pro-inflammatory signals depending on its membrane-bound state. (Demonstrated in human) |
| 189794 | Trem1 | 24752755 | Trem1 offers protective innate immunity during pneumococcal pneumonia by enhancing the early immune response of alveolar macrophages. |
| 73653 | SAMHD1 | 21720370 | SAMHD1 is specifically targeted for degradation by human immunodeficiency virus 1 (HIV-1) viral protein VPX, and mutations in SAMHD1 causes Aicardi-Goutieres syndrome, which mimics symptoms of congenital viral infection. |
| 73653 | SAMHD1 | 21613998 | SAMHD1 is expressed in dendritic and myeloid cells and acts as an anti-retroviral protein that inhibits the early stages of the viral life cycle. Silencing of SAMHD1 leads to significant accumulation of human immunodeficiency virus 1 (HIV-1) DNA in infected cells. |
| 73653 | SAMHD1 | 22327569 | SAMHD1 inhibits HIV replication by depleting intracellular dNTPs, which are required for viral reverse transcriptase to synthesize viral DNA. |
| 73653 | SAMHD1 | 24753578 | Full activation of SAMHD1 involves ordered binding of GTP and substrate dNTPs to activator and substrate sites on the enzyme. |
| 166395 | Cxcr2 | 24651866 | In response to adenovirus infection, the IL1A-IL1R1-CXCR2 signalling axis cooperates with complement to recruit Ly-6G+7/4+ polymorphonuclear leukocytes to the splenic marginal zone (MZ) in the proximity of virus-containing MARCO+ residential MZ macrophages, which are subsequently eliminated. |
| 166395 | Cxcr2 | 24755316 | Fibrates protect against bacteriaâ€ induced sepsis by inhibiting LPSâ€ mediated ERK phosphorylation and the expression of Adrbk1 (GRK2), thereby preventing Cxcr2 down-regulation on neutrophils. |
| 129419 | Adrbk1 | 24755316 | Fibrates protect against bacteriaâ€ induced sepsis by inhibiting LPSâ€ mediated ERK phosphorylation and the expression of Adrbk1 (GRK2), thereby preventing Cxcr2 down-regulation on neutrophils. |

| | | | |
|---|---|---|---|
| 126375 | MIR122 | 23580661 | Circulating MIR122 activates natural killer cells via the TLR1 signalling pathway. |
| 126375 | MIR122 | 24672032 | Reduction of MIR122 expression may play a role in the progression of fibrosis in patients with chronic hepatitis C. |
| 127039 | hsa-mir-146a | 19840932 | MIR146A is critical for in vitro monocytic cell-based endotoxin tolerance. |
| 127039 | hsa-mir-146a | 16885212 | MIR146A is involved in control of Toll-like receptor and cytokine signaling through a negative feedback regulation loop involving down-regulation of IL-1 receptor-associated kinase 1 (IRAK1) and TNF receptor-associated factor 6 (TRAF6) protein levels. |
| 127039 | hsa-mir-146a | 18801740 | MIR146A-mediated and NF-kappaB-sensitive inflammatory circuit was found in Alzheimer disease and in stressed human brain cells. |
| 127039 | hsa-mir-146a | 19021527 | MIR146A expression is driven by the transcription factor NF-kappaB and changes in the expression of hsa-mir-146a have been implicated in both the development of multiple cancers and in the negative regulation of inflammation induced via the innate immune response. |
| 127039 | hsa-mir-146a | 19021527 | MIR146A negatively regulates the IL-1-beta-induced release of chemokines IL-8 and RANTES. |
| 127039 | hsa-mir-146a | 19333922 | MIR146A negatively regulates the type I IFN pathway by directly targeting IRF5 and STAT1. |
| 127039 | hsa-mir-146a | 19596990 | MIR146A feedback inhibits RIG-I-dependent type I IFN production in macrophages by targeting TRAF6, IRAK1, and IRAK2. |
| 127039 | hsa-mir-146a | 20616571 | MIR146A significantly increases after 24 h stimulation in monocytes from neonate cord blood compared to those derived from adults, suggesting the involvement of microRNA in immune regulation of the innate immune system of neonates. |
| 127039 | hsa-mir-146a | 20937840 | MIR146A-mediated down-regulation of IRAK-1 coupled to an NF-Î°B-induced up-regulation of IRAK-2 expression drives an extensively sustained inflammatory response. |
| 127039 | hsa-mir-146a | 20951969 | MIR146A mediates the intestinal epithelial innate immune tolerance during the neonatal period, characterizing tolerance as an active condition involved in the establishment of intestinal mucosal homeostasis. |
| 127039 | hsa-mir-146a | 21329689 | MIR146A negatively regulates both oxidized low-density lipoprotein accumulation and the inflammatory response in macrophages by inhibiting TLR4 and the activation of TLR4-dependent signalling pathways. |
| 127039 | hsa-mir-146a | 21469088 | MIR146A is highly expressed in patients with Sjogren's syndrome and was found to increase the phagocytic activity and suppressing the inflammatory cytokine production in human monocytes. |

| | | | |
|---|---|---|---|
| 127039 | hsa-mir-146a | 21652514 | MIR146A transcription is induced by NFkB in response to stimulations such as LPS, TNF-alpha and IL1B. MIR146A targets TRAF6 and IRAK1 and act s as a negative regulator fine-tuning the immune response. |
| 127039 | hsa-mir-146a | 22545247 | MIR146A directly targets RELB to modulate the amplitude of monocyte response to inflammatory challenges. |
| 127039 | hsa-mir-146a | 22593544 | MIR146A is a mechanosensitive miRNA that modulates mechanotransduction and pressure-induced inflammation in small airway epithelium. |
| 127039 | hsa-mir-146a | 22363497 | MIR146A is overexpressed during prion disease and modulates the innate immune response and the microglial activation state. (Demonstrated in mice) |
| 127039 | hsa-mir-146a | 22822053 | MIR146A is upregulated in lung cells in response to infection with influenza A virus. |
| 127039 | hsa-mir-146a | 23028621 | MIR146A is a key regulator of astrocyte-mediated inflammation responses in neurological disorders. |
| 127039 | hsa-mir-146a | 23143987 | MIR146A alleviates ischemia-induced inflammation in the small intestine by downregulating IRAK1. |
| 127039 | hsa-mir-146a | 23343627 | In the course of influenza A infection MIR146A transcription increases to limit viral propagation. |
| 127039 | hsa-mir-146a | 23733368 | MIR146A targets components of the NF-kB and EGR pathways to repress inflammation. |
| 127039 | hsa-mir-146a | 24670381 | MIR146A is a potent negative regulator of the innate immune response in keratinocytes through downregulation of the IRAK1/TRAF6/NFÎºB pathway. |
| 27941 | RIPK2 | 17277144 | RIPK2 functions in innate immunity by mediating NOD1 and NOD2 signalling but not TLR-mediated immune responses. |
| 27941 | RIPK2 | 18079694 | RIPK2 polyubiquitination mediates the recruitment of MAP3K7 (TAK1) and the subsequent activation of NF-kappaB signalling via IKK complex in NOD signalling. |
| 27941 | RIPK2 | 18342009 | RIPK2 deubiquitination by TNFAIP3 (A20) restricts NF-kappaB activation via NOD2 signalling. |
| 27941 | RIPK2 | 18775659 | RIPK2 is sequestered by MAP3K4 to inhibit the NOD2:RIPK2 complex from activating NF-kappaB signalling pathways. |
| 27941 | RIPK2 | 21236705 | RIPK2 is a downstream adaptor molecule in the NOD1/2 signaling pathway and is important for the progression and pathogenesis of experimental autoimmune encephalomyelitis (animal model of multiple sclerosis). RIPK2 was found to be critical for the activation of CNS-infiltrating dendritic cells. |
| 27941 | RIPK2 | 21469090 | RIPK2 is essential for NOD1 and NOD2-signalling upon recognition of bacterial peptidoglycan. RIPK2 is crucial for inflammatory cytokine secretion, activation and recruitment of macrophage and neutrophils as well as the capacity to activate the adaptive immune response. (Demonstrated in murine model) |

| | | | |
|---|---|---|---|
| 27941 | RIPK2 | 24670424 | Following NOD2 activation, IRF4 interacts with MYD88, TRAF6, and RIPK2 and downregulates K63-linked polyubiquitinylation of RICK and TRAF6 leading to disruption of NFkB activation pathways. |
| 55681 | IRF4 | 12566414 | IRF4 participates in the regulation of lymphoid cell apoptosis by modulating the efficiency of the Fas-mediated death signal. |
| 55681 | IRF4 | 16272311 | IRF4 provides a positive feedback signal for its own gene expression in dendritic cells (DCs) and the expression of IRF4 mRNA, but not of other IRFs, is specifically up-regulated during DC differentiation. |
| 55681 | IRF4 | 24670424 | Following NOD2 activation, IRF4 interacts with MYD88, TRAF6, and RIPK2 and downregulates K63-linked polyubiquitinylation of RICK and TRAF6 leading to disruption of NFkB activation pathways. |
| 193159 | Il20rb | 24677051 | Astrocytes produce IL19 and express IL19 receptors (Il20ra and Il20rb) in response to bacterial pathogens. IL19 can significantly attenuate bacterially induced inflammatory astrocyte responses |
| 136067 | Il20ra | 24677051 | Astrocytes produce IL19 and express IL19 receptors (Il20ra and Il20rb) in response to bacterial pathogens. IL19 can significantly attenuate bacterially induced inflammatory astrocyte responses |
| 190663 | Il19 | 24677051 | Astrocytes produce IL19 and express IL19 receptors (Il20ra and Il20rb) in response to bacterial pathogens. IL19 can significantly attenuate bacterially induced inflammatory astrocyte responses |
| 157032 | Ptges | 21497116 | Ptges (prostaglandin E2) is produced by LPS-primed macrophages upon treatment with silica crystal and aluminum salts, and is important for the production of IgE in Th2 cells. |
| 157032 | Ptges | 24726877 | Influenza A virus infection targets the Ptges and prostaglandin E2 pathway to evade host type I IFN-dependent antiviral immunity |
| 82567 | MFF | 24733894 | Hepatitis C infection stimulates the expression of DNM1L and MFF and promotes DNM1L recruitment to mitochondria by stimulating the phosphorylation of DNM1L, leading to mitochondrial fission. |
| 26348 | DNM1L | 24733894 | Hepatitis C infection stimulates the expression of DNM1L and MFF and promotes DNM1L recruitment to mitochondria by stimulating the phosphorylation of DNM1L, leading to mitochondrial fission. |
| 36619 | BST2 | 19564354 | BST2 directly binds to purified LILRA4 (ILT7) protein, initiates signalling via the ILT7-FcepsilonRIgamma complex, and strongly inhibits production of IFN and pro-inflammatory cytokines in plamsmacytoid dendritic cells |
| 36619 | BST2 | 20702620 | BST2 (CD317) is a broadly acting and conserved mediator of innate control of retroviral infection and pathogenesis that restricts the release of retroviruses and lentiviruses in rodents by limiting replication of HIV-1 and MLV. |

| | | | |
|---|---|---|---|
| 36619 | BST2 | 21734563 | BST2 is an innate intracellular HIV restriction factor that is upregulated by type I interferons. |
| 36619 | BST2 | 22072710 | BST2 is a broad spectrum effector of the innate immune response to viral infection. BST2 is antagonized by the human immunodeficiency virus (HIV) Vpu protein to evade innate immune system detection. |
| 36619 | BST2 | 24733916 | HIV-1 viral protein U, Vpu, protects HIV-infected cells from antibody-dependent cell-mediated cytotoxicity as a function of its ability to counteract BST2 (tetherin). |
| 221396 | Mir302b | 24717937 | Mir302b expression is up-regulated upon bacterial infection and is a crucial regulator of NFÎºB signalling by directly targeting IRAK4 |
| 62183 | MAPK9 | 19153595 | MAPK9 phosphorylates IRF3 and is essential for IRF3 dimerization induced by polyinosinic-cytidylic acid (polyI:C). |
| 62183 | MAPK9 | 24651600 | Upon viral infection, MAVS recruits MKK7 onto mitochondria, leading to the induction of apoptosis by MAP2K7 activated MAPK9 |
| 24000 | MAP2K7 | 24651600 | Upon viral infection, MAVS recruits MKK7 onto mitochondria, leading to the induction of apoptosis by MAP2K7 activated MAPK9 |
| 172199 | Il5 | 24631157 | Chitin induces IL25, IL33, and TSLP which are required to stimulate ILC2 production of IL5 and IL13. IL5 and IL13, in turn, are required for the accumulation of eosinophils and alternatively activated macrophages that are associated with allergy. |
| 172064 | Il13 | 22154382 | Il13 dampens the innate immune response in airway epithelial cells via Irak3-mediated inhibition of Tlr2 signalling. (Demonstrated in human) |
| 172064 | Il13 | 24613091 | IL13 produced by activated innate lymphoid cells type 2 (ILC2s) is critical for promoting the migration of activated lung dendritic cells to the draining lymph node, and in the differentiation of naive CD4+ T cells into Th2 cells |
| 180637 | Kdm4a | 24656046 | The noncanonical NF Î º B pathway regulates histone modifications at the Ifnb1 promoter resulting in attenuated recruitment of Rela and histone demethylase, Kdm4a, to the Ifnb1 promoter. This provides a mechanism for regulating the induction of type I interferons . |
| 155240 | Vegfc | 24656836 | Flt4 signalling represents a negative feedback mechanism by immune cells to overreacting during bacterial infection; ligation of Flt4 by Vegfc reduces TLR4-triggered production of proinflammatory cytokines in macrophages. |
| 167138 | Flt4 | 24656836 | Flt4 signalling represents a negative feedback mechanism by immune cells to overreacting during bacterial infection; ligation of Flt4 by Vegfc reduces TLR4-triggered production of proinflammatory cytokines in macrophages. |

| | | | |
|---|---|---|---|
| 45463 | VEGFC | 24656836 | FLT4 signalling represents a negative feedback mechanism by immune cells to overreacting during bacterial infection; ligation of FLT4 by VEGFC reduces TLR4-triggered production of proinflammatory cytokines in macrophages. |
| 62355 | FLT4 | 24656836 | FLT4 signalling represents a negative feedback mechanism by immune cells to overreacting during bacterial infection; where ligation of FLT4 by VEGFC reduces TLR4-triggered production of proinflammatory cytokines in macrophages. |
| 195914 | Nlrp1a | 24549849 | Both Nlrp3 and Nlrp1a are important regulators of Toxoplasma proliferation and IL18 signaling is required to mediate host resistance to acute toxoplasmosis. |
| 191058 | Hdac1 | 24550390 | Daxx interacts with Hdac1 and represses the transcription of Il6 in TLR-triggered macrophages. |
| 168138 | Daxx | 24550390 | Daxx interacts with Hdac1 and represses the transcription of Il6 in TLR-triggered macrophages. |
| 164545 | Il12b | 22888135 | Il12, consisting of Il12a and Il12b subunits, induces Il2ra to form high-affinity Il2 receptors on natural killer cells in response to mouse cytomegalovirus infection. |
| 164545 | Il12b | 24163408 | Nod1 and Nod2 synergize with Tlr4 in dendritic cells to increase IL12 production and enhance invariant natural killer T (iNKT) cell activation, and are important regulators of the IFN gamma response by iNKT cells during S. typhimurium and L. monocytogenes infections. |
| 25212 | CXCL10 | 21124994 | CXCL10 exert direct antimicrobial effects in vitro against Bacillus anthracis spore and bacilli in a receptor-independent manner and contributes to pulmonary innate immunity. (Demonstrated in murine model) |
| 25212 | CXCL10 | 21518789 | CXCL10 concentration in blood increases during neonatal polymicrobial sepsis, and the blockade of CXCL10 not only worsens recruitment and phagocytic function of macrophages, but also the survival of neonatal mice. (Demonstrated in murine model) |
| 25212 | CXCL10 | 23998932 | MIR21 inhibition enhances CCL5 (RANTES) and CXCL10 (IP-10) release in MCF-7 cancer cells and resulted in increased lymphocyte migration . PIAS3 is a target of MIR21 in MCF-7 cells. |
| 25212 | CXCL10 | 24448099 | ISG15 does not directly alter human rhinovirus replication but modulates immune signalling via the viral sensor protein DDX58 to impact production of CXCL10, which has been linked to innate immunity to viruses. |
| 25212 | CXCL10 | 24448099 | Human rhinovirus infection of epithelial cells induces the expression and secretion of ISG15, which modulates immune responses via effects on DDX58, and by regulating CXCL10 production. |

| | | | |
|---|---|---|---|
| 148617 | Prdm1 | 24477914 | Prdm1 (Blimp1) modulates host defences by suppressing Ccl8-induced inflammation. |
| 5777 | MYH9 | 24489676 | The calpain-MYH9-RAB7B axis regulates TLR4 containing alpha-granules trafficking in thrombin-stimulated platelets. |
| 49631 | HNRNPL | 24371310 | THRIL, a large intergenic noncoding RNA, binds to heterogenous nuclear ribonucleoprotein L (HNRNPL) and forms a functional THRIL-HNRNPL complex that regulates transcription of TNF by binding to its promoter. |
| 81960 | IFIT1 | 20386592 | IFIT1 expression activates the IFN response during C. pneumoniae infection, mediated by intracellular nucleotide-sensing pattern recognition receptors (PRRs), which operate through a mechanism dependent on the bacterial type III secretion system (T3SS). |
| 81960 | IFIT1 | 21642987 | IFIT1 recognizes and directly binds to 5'-triphosphate RNA and functions as an innate inhibitor of viral replication and pathogenicity. (Demonstrated in murine model) |
| 81960 | IFIT1 | 22745654 | IFIT1 is an innate immune network bottleneck with the ability to suppress induction of TLR4 response genes in LPS-stimulated macrophages. (Demonstrated in mice) |
| 81960 | IFIT1 | 24098121 | IFIT1 binds mRNAs that lack 2â€²O methylation on the first ribose, thus inhibiting translation by impairing binding of eukaryotic translation initiation factors to 2â€²O-unmethylated RNA templates |
| 81960 | IFIT1 | 24371270 | IFIT1 binds with high affinity to the cap-proximal regions of cap0-mRNAs abrogating 48S complex formation in an in vitro reconstituted translation system. |
| 79651 | BTK | 12724322 | BTK is a Toll/interleukin-1 receptor domain-binding protein that participates in NF-kappaB activation by TLR4. |
| 79651 | BTK | 21441935 | BTK interacts with intracellular MHC class II molecules to activate adaptor molecules MYD88 and TICAM1 to promote TLR signalling. (Demonstrated in murine model) |
| 79651 | BTK | 21659545 | BTK is a positive regulator in the ITAM-mediated TREM1/TYROBP pathway, which induces pro-inflammatory cytokines such as TNF-alpha, IL8, and activation/differentiation cell surface markers. Patients suffering from X-linked agammaglobulinemia (XLA), which is a rare hereditary disease caused by mutation in the BTK gene, show reduced TNF-alpha induction in PBMCs upon TREM1 engagement. |
| 79651 | BTK | 22454496 | BTK directly phosphorylates TLR3 and plays a critical role in the induction of inflammatory cytokines and IFNB. (Demonstrated in mice) |
| 79651 | BTK | 22589540 | BTK is required for the activation of natural killer cells. (Demonstrated in mice) |

| | | | |
|---|---|---|---|
| 79651 | BTK | 22366891 | BTK is a negative regulator of TLR- or TNF-stimulated reactive oxygen species (ROS) production in neutrophils. |
| 79651 | BTK | 24375473 | BTK regulates TLR9-mediated induction of cytokines in plasmacytoid dendritic cells, but has no role in TLR7 signalling. |
| 78146 | TRIM14 | 23438823 | TRIM14 was identified in a systematic screen for positive regulators of innate immune responses. |
| 78146 | TRIM14 | 24379373 | TRIM14 is a mediator of mitochondrial antiviral immunity facilitating the immune responses mediated by retinoic acid-inducible gene-I-like (RIG-I)-like receptors. |
| 221120 | Mir149 | 24375488 | Mir149 negatively regulates TLR/Myd88 mediated inflammatory responses in macrophages by targeting Myd88 mRNA. |
| 152695 | Sykb | 22031919 | Sykb promotes wound healing in human rhinovirus-infected airway epithelial cells. (Demonstrated in human) |
| 152695 | Sykb | 14699155 | SYK is a tyrosine protein kinase that is an upstream activator of c-Jun N-terminal kinase (JNK). |
| 152695 | Sykb | 16449524 | SYK interacts with PLCG2 and plays a role in complement mediated phagocytosis by regulating both actin dynamics and the RhoA activation pathway. |
| 152695 | Sykb | 19151749 | SYK interacts with TRAF-interacting protein (TIRAP), two proteins with opposing effects on tumour necrosis factor (TNF) signalling where SYK enhances the activation of NF-kappaB by TNF and this is antagonized by TIRAP. |
| 152695 | Sykb | 19339971 | SYK operating downstream of ITAM-coupled fungal pattern recognition receptors by controlling both pro-IL-1beta synthesis and inflammasome activation after fungal infection. |
| 152695 | Sykb | 19913447 | SYK and MYD88 adaptor protein pathways activation by bacteria promotes regulatory properties of neutrophils. |
| 152695 | Sykb | 20401526 | SYK is activated by several ITAM-containing or ITAM-coupled C-type lectin receptors on myeloid cells leading to the production of pro-inflammatory cytokines including IL-1-beta to initiate antifungal responses. |
| 152695 | Sykb | 22031919 | SYK promotes wound healing in human rhinovirus-infected airway epithelial cells. |
| 152695 | Sykb | 24185614 | Phosphorylation of the inflammasome adaptor Pycard (ASC) controls inflammasome activity through the formation of ASC specks. The NLRP3 and AIM2 inflammasomes require Syk and Mapk8 (JNK) for their full activity . |
| 152695 | Sykb | 24212132 | The expression of Clec4e (Mincle) and its downstream signal phospho-Syk/Syk increases after cerebral ischemia and reperfusion. |

| | | | |
|---|---|---|---|
| 101710 | TXNIP | 22883233 | Hyperactivated ERN1 (IRE1 Î ± ) increases TXNIP mRNA stability by reducing levels of a TXNIP destabilizing microRNA, miR-17. In turn, elevated TXNIP protein activates the NLRP3 inflammasome, causing procaspase-1 cleavage and interleukin 1Î² (IL-1Î²) secretion. |
| 101710 | TXNIP | 24217221 | Endoplasmic reticulum (ER) stress-mediated reactive oxygen species accumulation leads to activation of NLRP3 inflammasome through enhanced secretion of IL1B and binding of TXNIP. |
| 211290 | Krt16 | 24218583 | Krt16 participates in the regulation of early inflammation and innate immunity in a broad range of settings involving skin, such as pachyonychia congenita and psoriasis. |
| 157061 | Cd5l | 21730133 | Cd5l (Aim) is required for obesity-associated recruitment of inflammatory macrophages into adipose tissue. |
| 157061 | Cd5l | 24223991 | Cd5l contributes to the macrophage autophagy mechanisms that lead to Mycobacterium. tuberculosis killing. |
| 273442 | MIR208B | 24241692 | The IFNL3 SNP rs4803217 is critical for the outcome of hepatitis C virus (HCV) infec Â¬ tion by controlling the stability of IFNL3 mRNA. HCV induces two microRNAs, MIR499A and MIR208B, that target the polymorphic region of the IFNL3 3â€² UTR. |
| 126967 | MIR499A | 24241692 | The IFNL3 SNP rs4803217 is critical for the outcome of hepatitis C virus (HCV) infec Â¬ tion by controlling the stability of IFNL3 mRNA. HCV induces two microRNAs, MIR499A and MIR208B, that target the polymorphic region of the IFNL3 3â€² UTR. |
| 50088 | IL28B | 24041672 | IFNL1, IFNL2 and IFNL3 (IL28B) have different effects on Toll-like receptor-related gene expression in HepG2 cells. |
| 50088 | IL28B | 24241692 | The IFNL3 SNP rs4803217 is critical for the outcome of hepatitis C virus (HCV) infec Â¬ tion by controlling the stability of IFNL3 mRNA. HCV induces two microRNAs, MIR499A and MIR208B, that target the polymorphic region of the IFNL3 3â€² UTR. |
| 207523 | Fscn1 | 24244012 | Fscn1 contributes to the survival of dendritic cells against Listeria monocytogenes infection. |
| 17253 | AICDA | 24244169 | Kaposi's sarcoma-associated herpesvirus (KSHV) infection results in upregulation of AICDA in primary human tonsillar B cells. Two KSHV miRNAs, K12-11 and K12-5 interact with the 3â€²UTR of AICDA to translationally repress its expression. |

| | | | |
|---|---|---|---|
| 69007 | PARD3 | 24244864 | Atypical PKC and PARD3 are inhibitors of the canonical NF-ÎºB activation pathway in epithelial cells. |
| 103733 | CD1D | 24248359 | Lipid transfer proteins saposins play an essential role in modulating human invariant natural killer T cell reactivity to antigen-presenting cells activated by inflammatory stimuli. Lipid-loaded Saposin B mediates lipid transfer onto CD1D and accelerates dissociation of CD1D-bound lipids, promoting lipid exchange. |
| 406966 | TICAM2 | 21494017 | TICAM2 deficiency results in the impairment of LPS-stimulated TNF-alpha protein translation. (Demonstrated in murine model) |
| 406966 | TICAM2 | 24297182 | ARF6 regulates LPS internalization and LPS-induced relocation of TICAM2 (TRAM), which is required for the MyD88-independent TLR4 signalling cascade. |
| 406966 | TICAM2 | 24255114 | The homotypic interaction of TICAM2 Toll/interleukin-1 receptor (TIR) domain is essential to form a scaffold for recruiting the TICAM1 TIR domain. |
| 19505 | TICAM1 | 19412184 | TICAM1 adaptor protein is displaced from TICAM2 by a splice variant of TICAM2, TAG, resulting in the negative regulation of the MyD88-independent TLR4 pathway. |
| 19505 | TICAM1 | 20019748 | TICAM1 (TRIF)-dependent activation of CASP8 is involved in pro-apoptotic signalling through TLR3 and this under the control of inhibitor of apoptosis proteins in melanoma cells. |
| 19505 | TICAM1 | 12471095 | TICAM1 preferentially activates the IFN-beta promoter in the Toll-like receptor signalling, particularly in the MyD88-independent pathway. |
| 19505 | TICAM1 | 14982987 | TICAM1 (TRIF) recruits TRAF6-TAK1-TAB2 to TLR3 through its TRAF6-binding site, which is required for NF-kappaB but not IRF3 activation. TLR3/TICAM1-mediated NF-kappaB and IRF3 activation is induced by double-stranded RNA. |
| 19505 | TICAM1 | 12539043 | TICAM-1 is an adaptor molecule that participates in TLR3-mediated interferon-beta induction. |
| 19505 | TICAM1 | 14517278 | TICAM1 and TICAM2 both function in LPS-TLR4 signalling to regulate the MyD88-independent pathway during the innate immune response to LPS. |
| 19505 | TICAM1 | 14519765 | TICAM1 and TICAM2 form an adaptor complex that plays a crucial role in LPS-TLR4-mediated activation of IFN-beta. |
| 19505 | TICAM1 | 21454965 | The TICAM1 signalling pathway in murine dendritic cells is crucial for dsRNA-mediated natural killer cell activation. (Demonstrated in murine model) |
| 19505 | TICAM1 | 21494017 | TICAM1 deficiency results in the impairment of LPS-stimulated TNF-alpha protein translation. (Demonstrated in murine model) |

| | | | |
|---|---|---|---|
| 19505 | TICAM1 | 21602824 | TICAM1 is crucial for NLRP3 inflammasome activation in response specific to viable, but not heat-killed, E. coli infections. (Demonstrated in murine model) |
| 19505 | TICAM1 | 21697485 | TICAM1 is proteolytically cleaved by Enterovirus 71 3C to inhibit the induction of innate immunity by TLR3-signalling. TICAM1 cleavage results in the inhibition of NFkB and IFN-beta promoter activation. |
| 19505 | TICAM1 | 21703541 | TICAM1 forms a dsRNA sensor complex with components DDX1, DDX21 and DHX36 to trigger the type I interferon and cytokine response to poly I:C, influenza A virus, and reovirus. (Demonstrated in murine model) |
| 19505 | TICAM1 | 21760953 | TICAM1 is a potent negative regulator of TLR agonist-triggered immune responses, specifically suppressing IL12 in dendritic cells and IFNG in natural killer cells. (Demonstrated in mouse) |
| 19505 | TICAM1 | 22072781 | TICAM1-TLR3-mediated signalling pathway plays an essential role in the anti-viral response against poliovirus infection. (Demonstrated in mouse) |
| 19505 | TICAM1 | 22124111 | TICAM1 plays a role in host resistance to Gram-negative enteropathogens. TICAM1-mediated protective immunity is orchestrated by macrophage-induced IFN-beta and natural killer cell production of IFN-gamma. (Demonstrated in mice) |
| 19505 | TICAM1 | 22123964 | TICAM1 forms a complex with RIPK3 upon Toll-like receptors (TLR) 3 and 4 activation resulting in RIPK3-dependent but TNF-independent necrosis in macrophages. (Demonstrated in mouse) |
| 19505 | TICAM1 | 23723066 | TICAM1 (TRIF) enhances expression of Kaposi â€ s sarcoma-associated herpesviral protein RTA. |
| 19505 | TICAM1 | 24255114 | The homotypic interaction of TICAM2 Toll/interleukin-1 receptor (TIR) domain is essential to form a scaffold for recruiting the TICAM1 TIR domain. |
| 136838 | Cd36 | 24257313 | MSR1 and Cd36 share several ligands and are involved in largely overlapping physiological and pathological processes, but they differ significantly in their effects on proinflammatory and immunoregulatory functions of macrophages. |
| 8289 | MSR1 | 19914718 | MSR1 is required for sensing human cytomegalovirus (HCMV) by endosomal TLR3 and TLR9 in monocytic THP-1 cells. |
| 8289 | MSR1 | 20162551 | MRS1 (SR-A) is upregulated in TLR4-mediated LPS responses and these receptors contribute to the efficient capturing and clearance of invading microbial pathogens. |
| 8289 | MSR1 | 21460221 | MSR1 deficiency leads to greater sensitivity to LPS-induced endotoxic shock. MSR1 down-regulates inflammatory gene expression in dendritic cells by suppressing TLR4-mediated activation of NFKB. |

| | | | |
|---|---|---|---|
| 8289 | MSR1 | 21756882 | MSR1, one of the principal receptors expressed on macrophages, suppresses macrophage activation by inhibiting the binding of lipopolysaccharide (LPS) to TLR4 in a competitive manner; thus playing a pivotal role in the regulation of the LPS-induced inflammatory response. (Demonstrated in murine model) |
| 8289 | MSR1 | 22751446 | Deletion of MSR1 results in protection from septic shock and modulation of TLR4 signalling in peritoneal macrophages. (Demonstrated in mice) |
| 8289 | MSR1 | 24257313 | MSR1 and Cd36 share several ligands and are involved in largely overlapping physiological and pathological processes, but they differ significantly in their effects on proinflammatory and immunoregulatory functions of macrophages. |
| 21100 | HMGB1 | 19890330 | HMGB1 functions as universal sentinel for nucleic-acid-mediated innate immune responses. |
| 21100 | HMGB1 | 19914413 | HMGB1 activates innate immunity mechanisms as a complex with DNA, lipids and/or pro-inflammatory cytokines. |
| 21100 | HMGB1 | 20419158 | HMGB1 is an alarmin and a key mediator of natural killer (NK)-dendritic cell (DC) cross-talk and plays a pivotal role in the escape of Human Immunodeficiency Virus (HIV)-infected dendritic cells from TRAIL-mediated NK cell cytotoxicity during NK-DC cross-talk by upregulating c-FLIP and c-IAP2 expression. |
| 21100 | HMGB1 | 21372296 | HMGB1 is an endogenous TLR4 ligand in macrophages and its release in wounds initiates TLR4-dependent responses that contribute to neovascularization. (Demonstrated in murine model) |
| 21100 | HMGB1 | 21389264 | HMGB1 is found in high concentrations within neutrophil extracellular traps (NETs). HMGB1 is a neutrophil protein that facilitates the uptake and recognition of mammalian DNA by plasmacytoid dendritic cells, and may play a role in Systemic Lupus Erthermatosus autoimmunity. |
| 21100 | HMGB1 | 21860212 | HMGB1 plays a key regulatory role in polymorphonuclear neutrophil (PMN) recruitment to inflammatory tissues. Low concentrations of HMGB1 (50-100 ng/ml) reduce baseline PMN migration as well as formyl-methionyl-leucyl-phenylalanine- and IL8-induced PMN chemotaxis, whereas higher HMGB1 concentrations (5000 ng/ml) have a chemoattractant effect on PMN through IL8 production. |
| 21100 | HMGB1 | 21871094 | HMGB1 has a pathogenic role in arthritis, where in complex with lipopolysaccharide, IL1A or IL1B, HMGB1 boosts the production of proinflammatory cytokines and MMP3 as demonstrated in synovial fibroblasts from rheumatoid arthritis and osteoarthritis patients. |
| 21100 | HMGB1 | 22396017 | Nuclear HMGB1 translocates to the cytoplasm in LPS-stimulated macrophages to potentiate inflammatory responses. (Demonstrated in mice) |

| | | | |
|---|---|---|---|
| 21100 | HMGB1 | 24257755 | Mast cell chymase CMA1 contributes to the control of inflammation by degrading the virulence factor Hsp70 of Trichinella spiralis, as well as several alarmins such as endogenous HSPA1A, BGN, and HMGB1.. |
| 89505 | BGN | 24257755 | Mast cell chymase CMA1 contributes to the control of inflammation by degrading the virulence factor Hsp70 of Trichinella spiralis, as well as several alarmins such as endogenous HSPA1A, BGN, and HMGB1.. |
| 79274 | HSPA1A | 21448922 | HSPA1A is secreted into the extracellular space during exercise-induced stress and increases the intracellular levels of cAMP, which acts as an "intracellular danger signal" to activate neutrophils. |
| 79274 | HSPA1A | 24257755 | Mast cell chymase CMA1 contributes to the control of inflammation by degrading the virulence factor Hsp70 of Trichinella spiralis, as well as several alarmins such as endogenous HSPA1A, BGN, and HMGB1. |
| 3997 | CMA1 | 24257755 | Mast cell chymase CMA1 contributes to the control of inflammation by degrading the virulence factor Hsp70 of Trichinella spiralis, as well as several alarmins such as endogenous HSPA1A, BGN, and HMGB1.. |
| 75811 | TIRAP | 11526399 | TIRAP is a Toll/Interleukin-1 receptor (TIR) domain containing adapter protein that binds to TLR4, serving as a bridge for MYD88 recruitment. |
| 75811 | TIRAP | 19717524 | TIRAP is dispensable in TLR2 signalling at high ligand concentrations in macrophages and dendritic cells, with MyD88 probably coupling to the TLR2 receptor complex at sufficient levels to allow activation but has an inhibitory role in the signalling of TLR3 to JNK. |
| 75811 | TIRAP | 11544529 | TIRAP is an adapter in Toll-like receptor 4 (TLR4) signal transduction. |
| 75811 | TIRAP | 12447442 | TIRAP is differentially involved in signalling by members of the Toll-like receptor (TLR) family and may account for specificity in the downstream signalling of individual TLRs. |
| 75811 | TIRAP | 12447441 | TIRAP has a crucial role in the MyD88-dependent signalling pathway shared by TLR2 and TLR4. |
| 75811 | TIRAP | 20400509 | TIRAP is a substrate for IRAK1 and IRAK4 with phosphorylation promoting its ubiquitination and degradation. |
| 75811 | TIRAP | 2095775 | TIRAP is an activator of TLR2/4 signalling and a negative regulator of TLR3/TRIF signalling. TIRAP is essential in restricting TLR3 signalling thereby protecting the host from unwanted immunopathologies associated with excessive IFN-beta production. |
| 75811 | TIRAP | 21705416 | TIRAP Ser180Leu polymorphism is significantly associated with Behcet's disease in UK, but not Middle Eastern, patients. It is suggested that the Ser180Leu functional variant of TIRAP will lead to greater cytokine production and tissue damage with persistence of mucosal lesions upon encounter with pathogens. |

| | | | |
|---|---|---|---|
| 75811 | TIRAP | 24265315 | TIR domain-contaning protein from Brucella melitensis, TcpB, disrupts the receptor-adaptor interaction between TLR4 and TIRAP. |
| 193788 | C3 | 24273177 | C3a and its receptor C3ar1 are critical for defense against C. psittaci in mouse lung infection. |
| 185906 | C3ar1 | 24273177 | C3a and its receptor C3ar1 are critical for defense against C. psittaci in mouse lung infection. |
| 142234 | Rheb | 24130493 | Mycobacterial infection induces expression of Mir155, which promotes the maturation of phagosomes and represses the expression of Rheb by targeting its 3â€ UTR |
| 142234 | Rheb | 24282297 | The interplay between Tsc1-Rheb-mTOR complex 1 signalling and Myc-dependent metabolism are dynamically regulated during dentritic cell (DC) development, whereas uncontrolled mTORC1 activation impairs DC development. |
| 152579 | Tsc1 | 22412198 | Tsc1 inhibits TLR response and endotoxin tolerance through repression of mTORC1 and JNK1/2 signalling pathways. |
| 152579 | Tsc1 | 23776173 | TSC1 is a critical regulator of dendritic cell function in both innate and adaptive immunity. |
| 152579 | Tsc1 | 24282297 | The interplay between Tsc1-Rheb-mTOR complex 1 signalling and Myc-dependent metabolism are dynamically regulated during dentritic cell (DC) development, whereas uncontrolled mTORC1 activation impairs DC development. |
| 204403 | Mtor | 21368289 | Inhibition of mTOR blocks the anti-inflammatory potency of glucocorticoids both in human monocytes and myeloid dendritic cells. (Demonstrated in human) |
| 204403 | Mtor | 24282297 | The interplay between Tsc1-Rheb-mTOR complex 1 signalling and Myc-dependent metabolism are dynamically regulated during dentritic cell (DC) development, whereas uncontrolled mTORC1 activation impairs DC development. |
| 172240 | Kdr | 24270517 | Mir126-Kdr axis is an important regulator of the innate response. Mir126 controls the survival and function of plasmacytoid dendritic cells and regulates gene expression of Tlr7, Tlr9, Nfkb1 and Kdr. |
| 172240 | Kdr | 24270517 | Mir126 controls the IFN-Î±/Î² responses to pathogen-associated nucleic acids through regulating the homeostasis and function of plasmacytoid dendritic cells (pDCs). Mir126 operates in part by regulating Kdr which encodes the growth factor receptor VEGFR2 in pDCs |
| 224249 | Mir126 | 24270517 | Mir126-Kdr axis is an important regulator of the innate response. Mir126 controls the survival and function of plasmacytoid dendritic cells and regulates gene expression of Tlr7, Tlr9, Nfkb1 and Kdr. |

| | | | |
|---|---|---|---|
| 224249 | Mir126 | 24270517 | Mir126 controls the IFN-Î±/Î² responses to pathogen-associated nucleic acids through regulating the homeostasis and function of plasmacytoid dendritic cells (pDCs). Mir126 operates in part by regulating Kdr which encodes the growth factor receptor VEGFR2 in pDCs |
| 152917 | Stat4 | 15356157 | Stat4 is required for the generation of an effective innate host defense against bacterial pathogens of the lung. |
| 152917 | Stat4 | 24285835 | Ity14 (Immunity to Typhimurium locus 14) mice with a mutation in Stat4 have an increased innate susceptibility following sublethal invasive S. Typhimurium challenge. |
| 5864 | ARF6 | 22170068 | ARF6 has a pivotal role in TLR9-mediated immune signaling by regulating the cellular uptake of CpG oligodeoxynucleotides. |
| 5864 | ARF6 | 24297182 | ARF6 regulates LPS internalization and LPS-induced relocation of TICAM2 (TRAM), which is required for the MyD88-independent TLR4 signalling cascade. |
| 194120 | Dusp16 | 24311790 | Dusp16 has a dual function in the innate immune system shaping the output of cytokines and other TLR-induced gene products by macrophages, most notably IL12, and regulating the responsiveness of bone marrow progenitor cells to the growth factor GM-CSF |
| 21798 | C3 | 16875735 | Central protein of the complement system; Classical, lectin and alternative pathways of complement all converge at the activation of C3 yielding a diverse set of biological responses |
| 21798 | C3 | 24315997 | T cell-expressed CTSL1 cleaves C3 into active C3a and C3b fragments mediating the intracellular and extracellular C3 activation in T cells. |
| 74981 | CTSL1 | 20926012 | CTSL1 enhances angiogenesis by increasing extracellular matrix degradation and remodelling. |
| 74981 | CTSL1 | 24315997 | T cell-expressed CTSL1 cleaves C3 into active C3a and C3b fragments mediating the intracellular and extracellular C3 activation in T cells. |
| 92817 | TRAF2 | 11479302 | TRAF2 and TRAF5 are signal transducers for the TNF receptor superfamily and are involved in TNF-induced NF-kappaB activation and protection from cell death. |
| 92817 | TRAF2 | 19150425 | TRAF2 plays a critical role in TNF signalling by directing the IKK complex to the membrane, promoting TRAF2 K63-linked ubiquitination, and positioning the IKKalpha and IKKbeta chains with the TAK1/TAB kinase. |
| 92817 | TRAF2 | 20449947 | TRAF2 competes with TRAF6 for CD40 binding, thereby limiting the capacity of CD40 engagement to induce NF-kappaB activation in human lymphocytes. |

| | | | |
|---|---|---|---|
| 92817 | TRAF2 | 22546736 | TRAF2 deficiency results in the accumulation of TNF-dependent, IL10-secreting neutrophils. Combined treatment of neutralizing antibodies against both TNF and IL10 substantially ameliorated the colitis phenotype in the Traf2 null mice. (Demonstrated in mice) |
| 92817 | TRAF2 | 24323043 | GNB2L1 (RACK1) negatively regulates NF Î º B activation by interacting with CHUK and IKBKB. The interaction interferes with the recruitment of the IKK complex to TRAF2 . |
| 63034 | GNB2L1 | 24323043 | GNB2L1 (RACK1) negatively regulates NF Î º B activation by interacting with CHUK and IKBKB. The interaction interferes with the recruitment of the IKK complex to TRAF2 . |
| 199298 | Rnasel | 21190483 | Rnasel cleaves RNA during viral infections and the cleavage products induces the RIG-I pathway and production of Ifnb gene. In addition, Rnasel is implicated in the protection of central nervous system against viral-induced demyelination. A broader role in innate immunity is suggested by involvement of Rnasel in cytokine induction and endosomal pathways that suppress bacterial infections. |
| 199298 | Rnasel | 24324683 | RNASEL contributes to innate immunity through regulating macrophage functions. |
| 148417 | Mapk8 | 24185614 | Phosphorylation of the inflammasome adaptor Pycard (ASC) controls inflammasome activity through the formation of ASC specks. The NLRP3 and AIM2 inflammasomes require Syk and Mapk8 (JNK) for their full activity . |
| 85951 | ELF4 | 24185615 | After viral infection, ELF4 binds to TMEM173 (STING) and induces type I interferon. ELF4 is critical for host antiviral defense. |
| 211512 | Dhx58 | 24434553 | Cytotoxic stress exemplified by ionizing radiation induces IFN-beta and enhances the expression of Dhx58 which in turn suppresses the interferon stimulated genes associated with cytotoxic stress by turning off the expression of IFN-beta. |
| 99443 | JAK1 | 8041779 | JAK1 physically associates with IL2RB and tyrosine phosphorylation of JAK1 is induced upon IL2 stimulation, suggesting that regulation of JAK1 may be linked to IL2 induced signal transduction. |
| 99443 | JAK1 | 15988755 | JAK1 and JAK2 are tyrosine kinases involved in the regulation of cell proliferation, differentiation, and survival and simultaneous activation of both JAK1 and JAK2 fusion proteins, but not either one alone, leads to the tyrosine phosphorylation of IL3RB, the activation of downstream signalling molecules, including STAT5, AKT, and MAPK, and the conferring of factor-independent growth to IL-3-dependent Ba/F3 cells. |

| | | | |
|---|---|---|---|
| 99443 | JAK1 | 24449862 | TNK1 is a component of the IFN-JAK-STAT signalling cascade and is a critical antiviral host factor where its abundance is inversely correlated to viral replication and contributes to the hepatocytic response to antiviral treatment. |
| 24740 | TNK1 | 24449862 | TNK1 is a component of the IFN-JAK-STAT signalling cascade and is a critical antiviral host factor where its abundance is inversely correlated to viral replication and contributes to the hepatocytic response to antiviral treatment. |
| 158682 | Reg3g | 24345802 | Reg3g has a protective role against mucosal infection by binding to Gram-negative and Gram-positive bacteria, and influences mucus distribution in the ileum and contributes to mucosal protection. |
| 156238 | Nlrx1 | 21703540 | Nlrx1 deletion leads to constitutive interaction of Mavs and Ddx58, resulting in increased inflammation. Nlrx1 knockout mice showed increased expression of antiviral signalling molecules such as Ifnb, Stat2, Oas1 and Il6 after influenza infection. In addition to attenuating the antiviral response, Nlrx1 directly associates with Traf6 and inhibits NFkB pathway in LPS-activated macrophages. |
| 156238 | Nlrx1 | 21703539 | Nlrx1 negatively regulates TLR-mediated NFkB activation by directly interacting with Traf6 or IKK kinase. Nlrx1 knockdown in mice enhances their susceptibility to LPS-induced septic shock and increases plasma Il6 levels. |
| 156238 | Nlrx1 | 22749352 | Nlrx1 attenuates type I IFN production and promotes autophagy during viral infection. (Demonstrated in human) |
| 156238 | Nlrx1 | 24366868 | Nlrx1 attenuates macrophage/microglial inflammatory activities and protects against experimental autoimmune encephalomyelitis caused by autoreactive T cells. |
| 12161 | SLX4 | 24412650 | HIV auxiliary protein Vpr directly activates SLX4 to promote cell cycle arrest at the G2/M, a process that requires VPRBP-DDB1-CUL4 E3-ligase complex, to escape from innate immune sensing. |
| 209867 | Bcl2l1 | 24412611 | Microbiota-driven Il17c induces Bcl2 and Bcl2l1 expression in intestinal epithelial cells in an autocrine manner to promote cell survival and tumorigenesis in both chemically induced and spontaneous intestinal tumor models. |
| 185673 | Bcl2 | 21850221 | Bcl2 is a multifunctional regulator of cell survival that inhibits the innate immune response during early stages of pathogenesis. Muscle-specific expression of Bcl2 in Lama2-deficient mice resulted in the inhibition of Tlr4, Tlr6, Tlr7, Tlr8 and Tlr9 induction, leading to reduced infiltration of eosinophils, the principal death effector cells. |

| | | | |
|---|---|---|---|
| 185673 | Bcl2 | 24412611 | Microbiota-driven Il17c induces Bcl2 and Bcl2l1 expression in intestinal epithelial cells in an autocrine manner to promote cell survival and tumorigenesis in both chemically induced and spontaneous intestinal tumor models. |
| 197277 | Il17c | 21993848 | Il17c is an essential autocrine cytokine that regulates innate epithelial immune responses. (Demonstrated in human) |
| 197277 | Il17c | 24412611 | Microbiota-driven Il17c induces Bcl2 and Bcl2l1 expression in intestinal epithelial cells in an autocrine manner to promote cell survival and tumorigenesis in both chemically induced and spontaneous intestinal tumor models. |
| 209673 | Il27 | 22156348 | Il27 priming results in an enhanced LPS-induced proinflammatory response in primary monocytes, specifically increasing expression of Il6, Tnf, Ccl3, and Ccl4. (Demonstrated in human) |
| 209673 | Il27 | 24408967 | Influenza virus infection induces pulmonary Il27 production in a type I IFN-alpha/beta receptor (IFNAR) signalling-dependent manner, which sensitizes mice to secondary pneumococcal infection downstream of IFNAR pathway. |
| 135340 | Gata3 | 23733962 | Gata3 is a critical regulator of innate lymphoid cell development from hematopoietic stem cells. |
| 135340 | Gata3 | 24419270 | Gata3 plays a generalized role in innate lymphoid cell (ILC) lineage determination and is critical for the development of gut Rorc+ group 3 ILCs subsets that maintain mucosal barrier homeostasis. |
| 728297 | EGLN2 | 24145445 | Regulation of IL1B-induced NF-Î°B by hydroxylases links key hypoxic and inflammatory signaling pathways in a manner that is dependent upon the combinatorial blockade of both EGLN2 and HIF1AN. |
| 86613 | HIF1AN | 24145445 | Regulation of IL1B-induced NF-Î°B by hydroxylases links key hypoxic and inflammatory signaling pathways in a manner that is dependent upon the combinatorial blockade of both EGLN2 and HIF1AN. |
| 206063 | Ifi202b | 24131791 | Cyclic-di-GMP-induced levels of Ifi202b suppress the expression of Tmem173 (STING). |
| 157258 | Il9 | 21983833 | Il9 production is largely restricted to innate lymphoid cells during papain-induce lung inflammation and serves as an as important bridging link to induce type 2 helper T cell responses. |
| 157258 | Il9 | 24138883 | Th9 cells and IL-9 have a critical and nonredundant role in host-protective type 2 immunity against parasitic worm infection |
| 1833 | CRKL | 9872990 | A type-I-IFN receptor that functions as a nuclear adapter protein and associates with STAT5 to regulate gene transcription through DNA binding |
| 1833 | CRKL | 24145029 | Enterohemorrhagic E. coli NleH1 effector targets CRKL to inhibit ribosomal protein S3 nuclear translocation and the NFÎ°B pathway. |

| | | | |
|---|---|---|---|
| 155803 | Numb | 24092752 | Nod2 driven inflammation is regulated by nitric oxide responsive Mir146 that facilitates activation of sonic hedgehog (SHH) signalling by targeting Numb expression. |
| 80616 | XRCC5 | 24098118 | Vaccinia virus protein C16 influences the immune response by binding to the XRCC6/XRCC5 (Ku70/80) complex thus blocking PRKDC-dependent DNA sensing in fibroblasts |
| 9323 | XRCC6 | 24098118 | Vaccinia virus protein C16 influences the immune response by binding to the XRCC6/XRCC5 (Ku70/80) complex thus blocking PRKDC-dependent DNA sensing in fibroblasts |
| 408612 | PRKDC | 24098118 | Vaccinia virus protein C16 influences the immune response by binding to the XRCC6/XRCC5 (Ku70/80) complex thus blocking PRKDC-dependent DNA sensing in fibroblast. |
| 159854 | Ifit1 | 21642987 | Ifit1 recognizes and directly binds to 5'-triphosphate RNA and functions as an innate inhibitor of viral replication and pathogenicity. |
| 159854 | Ifit1 | 22745654 | Ifit1 is an innate immune network bottleneck with the ability to suppress induction of Tlr4 response genes in LPS-stimulated macrophages. |
| 159854 | Ifit1 | 24098121 | IFIT1 binds mRNAs that lack 2â€²O methylation on the first ribose, thus inhibiting translation by impairing binding of eukaryotic translation initiation factors to 2â€²O-unmethylated RNA templates |
| 203813 | Mfn2 | 21285412 | Mfn2 deletion causes inability of the cell to undergo mitochondrial fusion, and therefore exhibit impaired induction of interferons and proinflammatory cytokines in response to viral infection - which results in increased viral replication. |
| 203813 | Mfn2 | 24127597 | Mitochondrial membrane potential is required for the association of Nlrp3 and Mfn2. Mfns2 is required for the activation of Nlrp3 inflammasomes. |
| 29713 | F2RL1 | 18622013 | F2RL1 and TLR4 interact in a synthetic agonist peptide-dependent manner and may support a novel paradigm of receptor cooperativity in inflammatory responses. |
| 29713 | F2RL1 | 19675284 | F2RL1 is involved in inflammation and is specifically activated by mast cell-derived tryptase. |
| 29713 | F2RL1 | 11907122 | Activation of F2RL1 stimulates IL6, IL8, and PTGER2 (prostaglandin E2) release from human respiratory epithelial cells, thus having an important role in modulating inflammation in the lung. |
| 29713 | F2RL1 | 19865078 | F2RL1 cooperates with TLR2, TLR3, or TLR4 for activation of nuclear factor-kappaB-dependent signalling in mucosal epithelial cell lines. |
| 29713 | F2RL1 | 19865078 | Activation of F2RL1 negatively regulates TLR3-dependent antiviral pathway, blunting the expression of TLR3/IRF3 driven genes, as well as activation of IRF3 and STAT1. |

| | | | |
|---|---|---|---|
| 29713 | F2RL1 | 21029417 | F2RL1 (PAR2) activates MAPK14 (p38) to trigger production of innate immunity markers in oral kertainocytes. |
| 29713 | F2RL1 | 24129891 | IL32 interacts with PR3 and F2RL1, triggering the PAR2-TRIF signalling axis, and IL32 may have a potential role in the transition from innate to adaptive immunity. |
| 13084 | PRTN3 | 24129891 | IL32 interacts with PRTN2 (PR3) and F2RL1, triggering the PAR2-TRIF signalling axis, and IL32 may have a potential role in the transition from innate to adaptive immunity. |
| 149582 | Msr1 | 21460221 | Msr1 deficiency leads to greater sensitivity to LPS-induced endotoxic shock. Msr1 down-regulates inflammatory gene expression in dendritic cells by suppressing Tlr4-mediated activation of NFKB. |
| 149582 | Msr1 | 21756882 | Msr1, one of the principal receptors expressed on macrophages, suppresses macrophage activation by inhibiting the binding of lipopolysaccharide (LPS) to Tlr4 in a competitive manner; thus playing a pivotal role in the regulation of the LPS-induced inflammatory response. |
| 149582 | Msr1 | 24035364 | Autophagy regulates phagocytosis by modulating the expression of scavenger receptors, Marco and Msr1 |
| 188086 | Marco | 24035364 | Autophagy regulates phagocytosis by modulating the expression of scavenger receptors, Marco and Msr1 |
| 80858 | CEBPB | 18820298 | CEBPB, one of the CEBP family members, is a crucial regulator of gene expression during innate immunity, inflammatory responses and adipogenesis. All of the individual CEBPB isoforms, LAP1, LAP2 and LIP, attenuate EGF-induced PTGS2 (COX-2) promoter activity. |
| 80858 | CEBPB | 21326902 | CEBPB is a member of the CCAAT enhancer binding protein family and is a transcriptional factor regulating genes in innate immunity and inflammation. The activities of CEBP are regulated via methylation of arginine and lysine side chains. |
| 80858 | CEBPB | 24038085 | CREB1 and CEBPB are involved in cytokine production in neutrophils in response to LPS and TNF. |
| 79573 | CREB1 | 19585511 | CREB1 and AP1 transcription factor activation regulates LPS- or M-CSF-induced activation of the MAPK phosphatase-1 (MKP-1) gene, a protein phosphatase that plays a crucial role in innate immunity. |
| 79573 | CREB1 | 18034423 | CREB1 has a key role in transcriptional regulation of macrophage migration inhibitory factor (MIF) gene expression and MIF-dependent host antimicrobial innate immune defence. |
| 79573 | CREB1 | 24038085 | CREB1 and CEBPB are involved in cytokine production in neutrophils in response to LPS and TNF. |

| | | | |
|---|---|---|---|
| 203203 | Nos2 | 16893173 | Nos2 (iNOS) and Calm1 (CaM) coordinately function to form a stable complex that is part of a rapid host response that functions within the first 30 min following bacterial infection to upregulate the innate immune system involving macrophage activation. |
| 203203 | Nos2 | 22542147 | Nitric oxide production by Nos2 promotes Listeria monocytogenes dissemination in the host. |
| 203203 | Nos2 | 24040305 | Inorganic polyphosphate suppresses Nos2 expression and nitric oxide release but not TNF release in LPS-activated macrophages |
| 50102 | IL28A | 21499846 | IL28A is able to efficiently inhibit Herpes Simplex Virus Type-1 replication in neuronal cells by inducing the expression of TLR as well as activate the TLR-mediated interferon antiviral pathway. |
| 50102 | IL28A | 24041672 | IFNL1, IFNL2 (IL28A) and IFNL3 have different effects on Toll-like receptor-related gene expression in HepG2 cells. |
| 181483 | Nfe2l2 | 21220332 | Nfe2l2 plays an important role in Tlr4-mediated autophagy. Nfe2l2 is activated by reactive oxygen species-Mapk14 axis-dependent Tlr4 signaling, and induces the accumulation of Sqstm1 and aggresome-like induced structures. |
| 181483 | Nfe2l2 | 24011591 | Phosphorylation of Sqstm1 (p62) activates the Keap1-Nrf2 pathway during selective autophagy |
| 139531 | Keap1 | 24011591 | Phosphorylation of Sqstm1 (p62) activates the Keap1-Nrf2 pathway during selective autophagy |
| 55893 | IL12B | 20145200 | IL12B production in response to Toll-like receptor (TLR) stimulation is regulated by the tyrosine phosphatase activity of PTPN6 and this is a novel mechanism for host regulation of IL12, a cytokine important in both innate and adaptive immunity. |
| 55893 | IL12B | 20935648 | IL12, consisting of IL12A and IL12B subunits, initiates local antitumor immunity by stimulating lymphoid tissue-inducer (LTi) cells bearing the natural cytotoxicity receptor NCR1 (NKp46). |
| 55893 | IL12B | 22888135 | IL12, consisting of IL12A and IL12B subunits, induces IL2RA to form high-affinity IL2 receptors on natural killer cells in response to mouse cytomegalovirus infection. (Demonstrated in mouse) |
| 55893 | IL12B | 23990628 | Macrophages cultured with bile acids produce lower levels of IL12 and display an anti-inflammatory phenotype characterized by an increased ratio of IL10 to IL12 |
| 55893 | IL12B | 24012417 | IFN gamma creates a primed chromatin environment in macrophages to augment TLR-induced IL12B transcription |
| 213090 | Cebpb | 21326902 | Cebpb is a member of the CCAAT enhancer binding protein family and is a transcriptional factor regulating genes in innate immunity and inflammation. The activities of CEBP are regulated via methylation of arginine and lysine side chains. |

| | | | |
|---|---|---|---|
| 213090 | Cebpb | 23472114 | LPS stimulation promotes the formation of CEBPB complexes on the Serpinb2 promoter to drive transcription. |
| 213090 | Cebpb | 24023826 | Tnfaip3 is regulated by both Nf-Î°B and p38-dependent Cebpb in response to LPS in macrophages. |
| 60223 | TRIM25 | 17392790 | TRIM25 is essential for RIG-I-mediated antiviral activity by inducing Lys 63-linked ubiquitination of DDX58 which is crucial for RIG-I signalling pathway. |
| 60223 | TRIM25 | 23438823 | TRIM25 was identified in a systematic screen for positive regulators of innate immune responses. |
| 60223 | TRIM25 | 23950712 | RNF135 is essential for the association of DDX58 (RIG-I) and TRIM25, resulting in the activation of RIG-I signalling. |
| 39370 | RNF135 | 23950712 | RNF135 is essential for the association of DDX58 (RIG-I) and TRIM25, resulting in the activation of RIG-I signalling. |
| 777831 | IKBKG | 10968790 | IKBKG is the regulatory subunit of the IKK-signalosome complex that is required for proinflammatory activation of the I-kappaB-kinase (IKK) complex. |
| 777831 | IKBKG | 19136968 | IKBKG is linearly polyubiquitinated by the LUBAC ligase complex, consisting of RNF31 and RBCK1 proteins, to regulate the canonical NF-kappaB activation pathway. |
| 777831 | IKBKG | 18080803 | IKBKG regulates TNF alpha signaling by coordinating cell responses mediated by the AP-1 and NF-kappa B pathways. |
| 777831 | IKBKG | 20010814 | IKBKG is essential for NF-kappaB activation and the polyubiquitylation and the degradation of IKBKG during Shigella infection is a new bacterial strategy to modulate host inflammatory responses. |
| 777831 | IKBKG | 23951545 | The binding of MAVS to Traf2, Traf5, and Traf6 is dependent on virus infection and MAVS polymerization. The TRAF proteins promote ubiquitination that recruits IKBKG binding to the MAVS signalling complex |
| 208924 | Traf5 | 23951545 | The binding of MAVS to Traf2, Traf5, and Traf6 is dependent on virus infection and MAVS polymerization. The TRAF proteins promote ubiquitination that recruits IKBKG binding to the MAVS signalling complex |
| 205458 | Mertk | 23954153 | Viruses engage Tyro3/Axl/Mertk (TAM) receptors in order to inhibit host type I IFN signalling. |
| 158468 | Axl | 23954153 | Viruses engage Tyro3/Axl/Mertk (TAM) receptors in order to inhibit host type I IFN signalling. |
| 199993 | Tyro3 | 23954153 | Viruses engage Tyro3/Axl/Mertk (TAM) receptors in order to inhibit host type I IFN signalling. |
| 35872 | LGALS9 | 21562126 | LGALS9 attenuates acute lung injury by preferentially suppressing pro-inflammatory functions in pDC-like macrophages through TLR2/TLR4 down-regulation. |

| | | | |
|---|---|---|---|
| 35872 | LGALS9 | 23408620 | LGALS9 engagement impairs the cytotoxicity function and cytokine production of natural killer cells. |
| 35872 | LGALS9 | 23967307 | TLR activation promotes HAVCR2 and LGALS9 association within the same macrophage to differentially regulate IL12/IL23 expressions via STAT3 phosphorylation. |
| 17159 | CLEC4D | 23911656 | CLEC4D and CLEC6A form a heterodimer complex which confers innate cells high ability to sense C. albicans infection by facilitating the activation of NFÎºB dependent inflammatory responses. |
| 19657 | HDAC11 | 23980205 | MIR145 directly targets HDAC11 to promote IL10 expression in TLR4-triggered macrophages. |
| 126739 | MIR145 | 23392170 | MIR145 targets SOCS7 to promote IFNB induction in bladder cancer cells. |
| 126739 | MIR145 | 23980205 | MIR145 directly targets HDAC11 to promote IL10 expression in TLR4-triggered macrophages |
| 98830 | PARK2 | 20457763 | PARK2 was recently shown to promote the clearance of impaired mitochondria by autophagy, termed mitophagy; PARK2 promotes mitophagy by catalyzing mitochondrial ubiquitination, which in turn recruits ubiquitin-binding autophagy components, HDAC6 and p62, leading to mitochondrial clearance. |
| 98830 | PARK2 | 24005326 | PARK2 is important for innate defence against M. tuberculosis for having a role in ubiquitin-mediated autophagy of M. tuberculosis |
| 7203 | POLR2F | 23994473 | During the transcriptional response to Sendai virus infection, POLR2F(RNA Pol II) is recruited by IRF3 and NFÎºB to control virus induced gene activation. |
| 18750 | IDO1 | 22355111 | IDO1 limits innate and adaptive immunity to apoptotic self-antigens. IDO1-mediated inhibition of inflammation plays a key role in suppressing systemic autoimmune diseases. (Demonstrated in mice) |
| 18750 | IDO1 | 23986532 | DNA nanoparticle is sensed selectively by myeloid dendritic cells (DCs) via the STING (for stimulator of interferon genes) /type I IFN pathway to induce Ido1 in DCs, which activate regulatory T cells. |
| 71595 | CXCL12 | 24000293 | MIR141 directly regulates CXCL12 expression and CXCL12 mediated leukocyte migration during colonic inflammation. |
| 126529 | MIR141 | 24000293 | MIR141 directly regulates CXCL12 expression and CXCL12 mediated leukocyte migration during colonic inflammation |
| 101753 | PIAS3 | 23998932 | MIR21 inhibition enhances CCL5 (RANTES) and CXCL10 (IP-10) release in MCF-7 cancer cells and resulted in increased lymphocyte migration . PIAS3 is a target of MIR21 in MCF-7 cells. |
| 42204 | CCL5 | 20386592 | CCL5 expression activates the IFN response during C. pneumoniae infection is mediated by intracellular nucleotide-sensing pattern recognition receptors (PRRs), which operate through a mechanism dependent on the bacterial type III secretion system (T3SS). |

| | | | |
|---|---|---|---|
| 42204 | CCL5 | 20523058 | CCL5 expression in airway epithelial cells is regulated by cross-talk between TLR3 signalling and inflammatory cytokines where TNF-alpha activates NF-kappaB, in cooperation with TLR3 signalling. |
| 42204 | CCL5 | 23998932 | MIR21 inhibition enhances CCL5 (RANTES) and CXCL10 (IP-10) release in MCF-7 cancer cells and resulted in increased lymphocyte migration . PIAS3 is a target of MIR21 in MCF-7 cells. |
| 126673 | MIR21 | 21652514 | MIR21 targets PDCD4, which acts a molecular switch between the pro-inflammatory (NFkB) and anti-inflammatory (IL10) response. MIR21-mediated reduction of PDCD4 is essential for protecting against the lethal effects of LPS. |
| 126673 | MIR21 | 22286305 | MIR21 is upregulated during Mycobacterium leprae infection of monocytes to escape from vitamin D-dependent antimicrobial pathways. |
| 126673 | MIR21 | 23580661 | Circulating MIR21 activates natural killer cells via the TLR1 signalling pathway. |
| 126673 | MIR21 | 23998932 | MIR21 inhibition enhances CCL5 (RANTES) and CXCL10 (IP-10) release in MCF-7 cancer cells and resulted in increased lymphocyte migration . PIAS3 is a target of MIR21 in MCF-7 cells. |
| 64056 | UCP2 | 23988448 | MIR133A1 enhances CASP1 activation and IL1B processing and suppresses inflammasome activation by suppressing its target UCP2 |
| 127431 | MIR133A1 | 23988448 | MIR133A1 enhances CASP1 activation and IL1B processing and suppresses inflammasome activation by suppressing its target UCP2 |
| 63368 | IL12A | 20935648 | IL12, consisting of IL12A and IL12B subunits, initiates local anti-tumour immunity by stimulating lymphoid tissue-inducer (LTi) cells bearing the natural cytotoxicity receptor NCR1 (NKp46). |
| 63368 | IL12A | 22888135 | IL12, consisting of IL12A and IL12B subunits, induces IL2RA to form high-affinity IL2 receptors on natural killer cells in response to mouse cytomegalovirus infection. (Demonstrated in mouse) |
| 63368 | IL12A | 23990628 | Macrophages cultured with bile acids produce lower levels of IL12 and display an anti-inflammatory phenotype characterized by an increased ratio of IL10 to IL12 |
| 2177 | RNF125 | 17460044 | RNF125 is an ubiquitin ligase which negatively regulates RIG-I signalling by conjugating ubiquitin to DDX58 and MDA5 and IPS1, tagging them for proteosomal degradation |
| 2177 | RNF125 | 23772026 | RNF125 is inhibited by human Bocavirus protein, VP2, to induce the IFNB signalling pathway. |
| 161026 | Zbtb20 | 23776228 | ZBTB20 inhibits transcription of IkBa to promote full TLR-triggered immunity. Zbtb20 null mice are resistant to endotoxin shock and sepsis. |
| 94666 | ASCC3 | 23781071 | ASCC3 inhibits IFN-signalling to dampen antiviral innate immunity. |

| | | | |
|---|---|---|---|
| 89176 | MASP2 | 18596036 | MASP2 binds to and is activated by MBL or ficolin in response to pathogen-associated molecular patterns (PAMPs). |
| 89176 | MASP2 | 16189649 | MASP2, upon activation, can cleave complement component 4 (C4) and C2 to generate the C3 convertase, C4bC2b. |
| 89176 | MASP2 | 22792067 | MASP2 deficient mice are defective in the lectin pathway of complement activation and are highly susceptible to pneumococcal infection. (Demonstrated in mice) |
| 89176 | MASP2 | 23785123 | MASP2 forms a complex with MASP1 to activate the complement system. |
| 69257 | MASP1 | 20038603 | MASP1 is an essential protease of both the lectin and alternative complement pathways, essential components of innate immunity, participating in the pathogenesis of inflammatory diseases and in host defence. |
| 69257 | MASP1 | 22966085 | MASP1 is crucial for classical complement activation, but is not required for the alternative pathway function. |
| 69257 | MASP1 | 23785123 | MASP1 forms a complex with MASP2 to activate the complement system. |
| 55716 | WNT9B | 23785285 | Secretion of WNT9B upon virus infection negatively regulates antiviral innate immunity. |
| 101110 | WNT2B | 23785285 | Secretion of WNT2B upon virus infection negatively regulates antiviral innate immunity. |
| 102045 | VPS45 | 23738510 | Mutations in VPS45 are associated with congenital neutrophil defect syndrome. |
| 202876 | Sarm1 | 23499490 | Upon infection with encephalitic Bunyavirus, SARM1 is activated by RIG-I/MAVS signalling to mediate neuronal cell death. |
| 202876 | Sarm1 | 23751821 | Sarm1 is expressed in neurons and regulates the expression of inflammatory and anti-viral cytokines. |
| 202876 | Sarm1 | 23749635 | Sarm1 null mice are resistant to central nervous system infection with vesicular stomatitis virus. |
| 407857 | TLR9 | 11130078 | TLR9 acts as a receptor for unmethylated CpG-DNA and bacterial DNA to initiate a variety of immune responses. |
| 407857 | TLR9 | 18262306 | TLR9 recognizes unmethylated DNA with CpG-motifs and the LR9 mediated signalling pathway is not only responsible for activation of innate immune cells, but also for mounting acquired responses. |
| 407857 | TLR9 | 14579267 | TLR9, 8 and 7 form a functional subgroup within the TLR family that recognizes pathogen-associated molecular patterns in endosomal/lysosomal compartments. |
| 407857 | TLR9 | 11867692 | TLR9 acts at the cell surface and engages an intracellular signalling pathway that includes MyD88, IRAK, and TRAF6. |

| | | | |
|---|---|---|---|
| 407857 | TLR9 | 20034855 | TLR9/7-mediated innate immune responses are negatively regulated via selected TLR pathways by a human microsatellite DNA-mimicking oligodeoxynucleotide with CCT repeats. |
| 407857 | TLR9 | 20360853 | TLR9 and TLR4 have both non-redundant and cooperative roles in lung innate responses during Gram-negative bacterial pneumonia and are both critical for IL-17 driven antibacterial host response. |
| 407857 | TLR9 | 18820679 | TLR9 ectodomain is cleaved to generate a functional receptor; although both the full-length and cleaved forms of TLR9 are capable of binding ligand, only the processed form recruits MyD88 on activation, indicating that this truncated receptor, rather than the full-length form, is functional. |
| 407857 | TLR9 | 21376231 | TLR9 signalling enhances the rate of acidification of Salmonella-containing phagosomes, and this acidification induces the expression of Salmonella pathogenecity genes that are necessary for intracellular survival, growth, and systemic infection. TLR9 deficiency rescues the high Salmonella susceptibility phenotype observed in TLR2,TLR4 double mutant mice . (Demonstrated in murine model) |
| 407857 | TLR9 | 21389263 | TLR9 triggers plasmacytoid dendritic cells in Systemic Lupus Erythematosus patients upon recognition of self-antigens such as neutrophil extracellular traps (NETs). |
| 407857 | TLR9 | 21402738 | TLR9 requires proteolytic processing in endolysosome by asparagine endopeptidase and cathepsin in the endolysosome to initiate signalling in response to DNA. (Demonstrated in murine model) |
| 407857 | TLR9 | 21439959 | TLR9 deficiency reduced pancreatic edema, inflammation and pro-IL1B expression in pacreatitis. (Demonstrated in murine model) |
| 407857 | TLR9 | 21482737 | TLR9 synergistically interacts with TLR2::TLR6 in lung epithelium to induce rapid pathogen killing, and can be used as a therapeutic target to treat otherwise lethal pneumonia. |
| 407857 | TLR9 | 21604257 | TLR9 is proteolytically cleaved in the endosome to form a soluble TLR9 (sTLR9), which inhibits TLR9-dependent signalling and contributes to the prevention of autoimmune disease. |
| 407857 | TLR9 | 21721026 | TLR9 activation is enhanced by increased levels of circulating histones, serving as a crucial link between initial damage and activation of innate immunity during sterile inflammation. (Demonstrated in murine model) |
| 407857 | TLR9 | 21860217 | TLR9 promotes macrophage HIF1A levels, oxidative burst and nitric oxide production in response to group A Streptococcus (GAS), contributing to GAS clearance in vivo in both localized cutaneous and systemic infection models. (Demonstrated in murine model) |

| | | | |
|---|---|---|---|
| 407857 | TLR9 | 21947771 | TLR9 is selectively compartmentalized to fungal phagosomes and negatively modulates macrophage anti-fungal effector functions. (Demonstrated in mice) |
| 407857 | TLR9 | 22342842 | TLR9 expression and signalling capacity oscillate with the circadian clock. (Demonstrated in mice) |
| 407857 | TLR9 | 22535248 | Mitochondrial DNA that escapes from autophagy induces TLR9 inflammatory responses in cardiomyocytes and is capable of inducing myocarditis and dilated cardiomyopathy. (Demonstrated in mice) |
| 407857 | TLR9 | 23071157 | TLR9 activation by endogenous self-ligands generated during oxidative stress promotes platelet hyper-reactivity and thrombosis. |
| 407857 | TLR9 | 23752229 | Human papillomavirus E7 protein forms a transcriptionally repressive complex over the promoter of TLR9 to suppress the interferon response. |
| 23294 | ANKRD17 | 23711367 | ANKRD17 binds to NOD2 and contributes to inflammatory responses against Shigella flexneri. |
| 15224 | UCHL1 | 23717208 | Human papillomavirus exploits UCHL1 to suppress interferon, pro-inflammatory cytokines and chemokines production and to dampen NF-kB signalling. |
| 148689 | Klf4 | 23733190 | KLF4 mediates the repression of Ciita/MHC-II in M. bovis BCG infected macrophages to evade antigen presentation. |
| 64539 | ERN1 | 20533428 | ERN1 (IRE1)/XBP1-mediated signalling plays roles in the coordination of metabolic and immune responses by acting as a regulatory hub, linking endoplasmic reticulum homeostasis with innate immunity and metabolism. |
| 64539 | ERN1 | 22883233 | Hyperactivated ERN1 (IRE1 Î ± ) increases TXNIP mRNA stability by reducing levels of a TXNIP destabilizing microRNA, miR-17. In turn, elevated TXNIP protein activates the NLRP3 inflammasome, causing procaspase-1 cleavage and interleukin 1Î² (IL-1Î²) secretion. |
| 64539 | ERN1 | 23684307 | ERN1 binds to cholera toxin in the endoplasmic reticulum to activate RIG-I innate immune signalling. |
| 138038 | Coch | 23684986 | COCH is secreted by follicular dendritic cells to promote antibacterial innate immunity. |
| 198471 | Cdc42 | 23690402 | Cdc42 has a critical role in mediating innate immunity against upper airway infections. |
| 74081 | SRC | 19667404 | SRC and STAT3 play a role in apoptotic cell-mediated MerTK-dependent immunoregulation of dendritic cells. |
| 49157 | CD14 | 19776284 | CD14 co-receptor participates with toll-like receptors (TLRs) in the response of microglial cells to fibrillar forms of beta-amyloid, contributing to microglial activation. |

| | | | |
|---|---|---|---|
| 49157 | CD14 | 19840871 | CD14 recognizes necrotic cells in addition to LPS, PG, apoptotic cells, and lipids, suggesting that it might be a universal adaptor for damage-associated molecular pattern (DAMP) and pathogen-associated molecular patterns (PAMP). |
| 49157 | CD14 | 10652232 | CD14 is a molecule that binds to lipopolysaccharide (LPS) and facilitates its signalling by helping TLR4-LY96 to sense and signal the presence of LPS. |
| 49157 | CD14 | 21078886 | CD14 contributes to nucleic acid uptake in macrophages and acts as a co-receptor for endosomal TLR7/TLR9 activation. |
| 49157 | CD14 | 23548899 | CD14 acts as an adaptor molecule for the immune recognition of salmonella curli fibers. |
| 22826 | IL27 | 20435892 | IL27, a member of the IL-12 cytokine family, acts as a pro-inflammatory cytokine that regulates the differentiation of naive T helper cells and also possesses anti-inflammatory properties. |
| 22826 | IL27 | 20519510 | IL27 induces a STAT1/3 and NFkappaB dependent pro-inflammatory cytokine profile in human monocytes. |
| 22826 | IL27 | 20519510 | IL27 is a strong inducer of pro-inflammatory cytokine and chemokine expression, including enhancement of IL6, CXCL10 (IP-10), CCL3 (MIP-1alpha), CCL4 (MIP-1beta), and TNF (TNF-alpha) expression in human primary monocytes, the production of which is mediated by STAT1, STAT3, and NF-kappaB activation. |
| 22826 | IL27 | 22156348 | IL27 priming results in enhanced LPS-induced proinflammatory response in human primary monocytes, specifically increasing expression of IL6, TNF, CCL3, and CCL4. |
| 22826 | IL27 | 23557795 | IL27 reduces vacuolar ATPase levels to inhibit phagosome acidification in macrophages. |
| 127561 | MIR15B | 23580661 | Circulating MIR15B activates natural killer cells via the TLR1 signalling pathway. |
| 195566 | Lgr4 | 23589304 | Lgr4 deletion potentiates TLR2/4-mediated cytokine production and Lgr4 null mice are more susceptible to septic shock. |
| 65161 | TNFSF10 | 23498957 | TNFSF10 is targeted by human cytomegalovirus glycoprotein to protect infected cells from natural killer cell cytotoxicity. |
| 197968 | Ppp1cc | 23499489 | PPP1CC dephosphorylates RNA sensors, RIG-I (DDX58) and MDA5 (IFIH1), to induce anti-viral IFNB production. |
| 57303 | PPP1CC | 23499489 | PPP1CC dephosphorylates RNA sensors, RIG-I (DDX58) and MDA5 (IFIH1), to induce anti-viral IFNB production. |
| 129195 | Ppp1ca | 23499489 | PPP1CA dephosphorylates RNA sensors, RIG-I (DDX58) and MDA5 (IFIH1), to induce anti-viral IFNB production. |

| | | | |
|---|---|---|---|
| 60210 | PPP1CA | 18949366 | PPP1CA positively regulates the TNF-alpha-induced NF-kappaB pathway at the level of IKK activation. |
| 60210 | PPP1CA | 23499489 | PPP1CA dephosphorylates RNA sensors, RIG-I (DDX58) and MDA5 (IFIH1), to induce anti-viral IFNB production. |
| 221154 | Mir203 | 23522925 | Mir203 post-transcriptionally downregulates Myd88 to inhibit the TLR signalling pathway in macrophages. |
| 170702 | Psmb8 | 23527234 | PSMB8 (LMP7) inhibits phagocytosis and enhances susceptibility of red blood cells to malarial parasites. |
| 127401 | MIR517C | 23448136 | MIR517C activates NF-kB signalling and directly targets TNIP1 mRNA to promote apoptosis. |
| 127271 | MIR517A | 23448136 | MIR517A activates NF-kB signalling and directly targets TNIP1mRNA to promote apoptosis. |
| 162815 | Ifne | 23449591 | IFNE is constitutively expressed by epithelial cells of the female reproductive tract to confer protection against sexually transmitted infections. |
| 172875 | Csf2 | 23453633 | CSF2 is a negative regulator of IL21-mediated apoptosis in conventional dendritic cells. |
| 140267 | Il21 | 23453633 | IL21 induces apoptosis of conventional dendritic cells. |
| 776557 | IKBKE | 17003035 | IKBKE interacts with NKFB2 (p52) and promotes transactivation via RELA (p65)-regulation of the alternative NF-kappaB activation pathway involving p52 and p65. |
| 776557 | IKBKE | 10421793 | IKBKE is an IkappaB kinase that is expressed mainly in immune cells, and is induced in response to pro-inflammatory cytokines such as tumour necrosis factor (TNF)-alpha, IL-1-beta and IL-6, in addition to LPS. |
| 776557 | IKBKE | 12692549 | IKBKE and TBK1 have a pivotal role in coordinating the activation of IRF3 and NF-kappaB in the innate immune response. |
| 776557 | IKBKE | 21138416 | IKBKE and other IKK kinases regulate each other by an intricate network involving phosphorylation of their catalytic and regulatory (NEMO, TANK) subunits to balance their activities during innate immunity. |
| 776557 | IKBKE | 21822257 | IKBKE kinase modulates IL-17 signalling through TRAF3IP2 adaptor protein phosphorylation, resulting in the induction of neutrophilia and pulmonary inflammation. (Demonstrated in mouse) |
| 776557 | IKBKE | 22065572 | IKBKE is an important signalling molecule in the IFN pathway and is necessary to mount anti-viral immunity against West Nile virus. (Demonstrated in mice) |
| 776557 | IKBKE | 22171011 | IKBKE plays a critical role in regulating the balance between the type I and type II interferon (IFN) signalling pathways. |
| 776557 | IKBKE | 22532683 | IKBKE is sequestered by arenavirus nucleoproteins to block its autocatalytic activity and its ability to active IRF3. |
| 776557 | IKBKE | 23453969 | IKBKE is poly-ubiquitinated at Lys30 and 401 to promote NF-kB activation in macrophages stimulated with TNFA and IL1B. |

| | | | |
|---|---|---|---|
| 193646 | Mfge8 | 23454767 | MFGE8 inhibits inflammasome-induced IL1B production in macrophages. |
| 11085 | NOD1 | 17690884 | NOD1 and NOD2 represent central players in the control of the immune responses to bacterial infections and inflammation. |
| 11085 | NOD1 | 17705131 | NOD1 and NOD2 can induce CCL5 (RANTES) through NF-kappaB pathway, orchestrating the global Nod-dependent immune defence during bacterial infections. |
| 11085 | NOD1 | 19898471 | NOD1 and NOD2 direct autophagy by recruiting ATG16L1 to the plasma membrane at the site of bacterial entry. |
| 11085 | NOD1 | 20039881 | NOD1 plays an important role in host defence against bacterial infection by regulating direct killing of Helicobacter pylori bacteria by antimicrobial peptides. |
| 11085 | NOD1 | 20042586 | NOD1-dependent responses account for host resistance against T. cruzi infection by mechanisms independent of cytokine production. |
| 11085 | NOD1 | 20081863 | NOD1 plays a role in priming innate defences, facilitating a rapid response to infection by recognizing peptidoglycan from microbiota and enhancing killing of pathogens by bone marrow-derived neutrophils. |
| 11085 | NOD1 | 21236705 | NOD1 is a peripheral peptidoglycan intracellular sensor and is important for the progression and pathogenesis of experimental autoimmune encephalomyelitis (animal model of multiple sclerosis). |
| 11085 | NOD1 | 21108472 | NOD1 detects heat-killed Legionella pneumophila and stimulates NFkB and IFN-beta promoter activity. NOD1 deficiency results in impaired bacterial clearance and increased proinflammatory cytokine at 24hrs post-infection. (Demonstrated in murine model) |
| 11085 | NOD1 | 21677137 | NOD1 is expressed by trophoblast cells across gestation and may have a role in mediating infection-associated inflammation and prematurity. Activation of NOD1 by bacterial peptidoglycan-derived peptide induces maternal-fetal inflammation and preterm labour. |
| 11085 | NOD1 | 21715553 | Nod1 KO mice were protected from high-fat diet induced inflammation, lipid accumulation, and peripheral insulin intolerance. Ex vivo, NOD1 activation by bacterial peptidoglycan mimetics induces proinflammatory cytokine secretion and impaired insulin-stimulated glucose uptake in adipocytes. Hence, NOD1 is a plausible, new link between innate immunity and metabolism. (Demonstrated in murine model) |
| 11085 | NOD1 | 23460743 | Helicobacter pylori infection of gastric epithelial cells activates NOD1 to enhance IFN-gamma signalling. |
| 705497 | MIR3148 | 23468661 | MIR3148 modulates allelic expression of TLR7 variant, rs3853839G, associate with systemic lupus erythematosus. |

| | | | |
|---|---|---|---|
| 210271 | Plunc | 21054862 | Plunc is a protein able to inhibit Mycoplasma pneumoniae (Mp) growth and its production following Mp infection is regulated through Toll-like receptor 2 (TLR2) signalling. |
| 210271 | Plunc | 21787346 | Plunc is a secretory protein that exhibits antimicrobial activity against Gram-negative bacteria and anti-inflammatory functions in respiratory infections. (Demonstrated in human) |
| 210271 | Plunc | 23470624 | PLUNC1 is suppressed in pneumonic epithelium in the respiratory tract. |
| 186363 | Serpinb2 | 23472114 | LPS stimulation promotes the formation of CEBPB complexes on the Serpinb2 promoter to drive transcription. |
| 80144 | ᶠTPA1B;SFTP⁄ | 20413160 | SFTPA1B;SFTPA1 is a carbohydrate pattern recognition molecule of innate immunity that significantly enhances phagocytosis and killing of Aspergillus fumigatus, a pathogenic fungus, by neutrophils and macrophages. |
| 80144 | ᶠTPA1B;SFTP⁄ | 20418258 | SFTPA1B;SFTPA1 can attenuate bacterial and viral infection and inflammation by acting as an opsonin and by regulating innate immune cell functions. |
| 80144 | ᶠTPA1B;SFTP⁄ | 21821801 | SFTPA1 binds to Mycobacterium avium lipid and it promotes the agglutination of the pathogen. The presence of SFTPA1 inhibits the growth of M. avium in culture. |
| 80144 | ᶠTPA1B;SFTP⁄ | 23475791 | SPA4 peptide, derived from SFTPA1, associates with TLR4 to inhibit LPS-induced inflammation and to alleviate endotoxic shock. |
| 94466 | TRIM63 | 23438823 | TRIM63 was identified in a systematic screen for positive regulators of innate immune responses. |
| 43425 | TRIM61 | 23438823 | TRIM61 was identified in a systematic screen for positive regulators of innate immune responses. |
| 43470 | TRIM60 | 23438823 | TRIM60 was identified in a systematic screen for positive regulators of innate immune responses. |
| 23653 | TRIM55 | 23438823 | TRIM55 was identified in a systematic screen for positive regulators of innate immune responses. |
| 67515 | TRIM49 | 23438823 | TRIM49 was identified in a systematic screen for positive regulators of innate immune responses. |
| 101433 | TRIM45 | 23438823 | TRIM45 was identified in a systematic screen for positive regulators of innate immune responses. |
| 58977 | TRIM42 | 23438823 | TRIM42 was identified in a systematic screen for positive regulators of innate immune responses. |
| 66673 | TRIM38 | 23056470 | TRIM38 targets TICAM1 (TRIF) for degradation to negatively regulateTLR3-mediated IFNB signalling. |
| 66673 | TRIM38 | 23438823 | TRIM38 was identified in a systematic screen for positive regulators of innate immune responses. |
| 61551 | TRIM37 | 23438823 | TRIM37 was identified in a systematic screen for positive regulators of innate immune responses. |
| 37599 | TRIM36 | 23438823 | TRIM36 was identified in a systematic screen for positive regulators of innate immune responses. |

| | | | |
|---|---|---|---|
| 72253 | TRIM27 | 23438823 | TRIM27 was identified in a systematic screen for positive regulators of innate immune responses. |
| 43164 | TRIM24 | 23438823 | TRIM24 was identified in a systematic screen for positive regulators of innate immune responses. |
| 24045 | TRIM23 | 20724660 | TRIM23 mediates K(lys)-27-ubiquitin conjugation to IKBKG (NEMO) which is essential for TLR3- and RIG-I/MDA5-mediated antiviral innate and inflammatory responses. |
| 24045 | TRIM23 | 23438823 | TRIM23 was identified in a systematic screen for positive regulators of innate immune responses. |
| 27325 | TRIM6 | 23438823 | TRIM6 was identified in a systematic screen for positive regulators of innate immune responses. |
| 81697 | MID2 | 23438823 | MID2 was identified in a systematic screen for positive regulators of innate immune responses. |
| 107395 | TRIM67 | 23438823 | TRIM67 was identified in a systematic screen for positive regulators of innate immune responses. |
| 30708 | TRIM66 | 23438823 | TRIM66 was identified in a systematic screen for positive regulators of innate immune responses. |
| 69081 | TRIM65 | 23438823 | TRIM65 was identified in a systematic screen for positive regulators of innate immune responses. |
| 32956 | TRIM56 | 21074459 | TRIM56 is a regulator of double-stranded DNA-mediated type I interferon induction where it acts as an interferon-inducible E3 ubiquitin ligase that modulates STING to confer double-stranded DNA-mediated innate immune responses. |
| 32956 | TRIM56 | 21289118 | TRIM56 is an anti-viral host factor that restricts pestivirus infection, specifically it was found to impair bovine viral diarrhoea virus replication. |
| 32956 | TRIM56 | 22948160 | TRIM56 is an essential component of the TLR3 anti-viral signalling pathway. |
| 32956 | TRIM56 | 23438823 | TRIM56 was identified in a systematic screen for positive regulators of innate immune responses. |
| 19813 | TRIM50 | 23438823 | TRIM50 was identified in a systematic screen for positive regulators of innate immune responses. |
| 34165 | TRIM13 | 23438823 | TRIM13 was identified in a systematic screen for positive regulators of innate immune responses. |
| 6467 | TRIM9 | 23438823 | TRIM9 was identified in a systematic screen for positive regulators of innate immune responses. |
| 88134 | TRIM8 | 23438823 | TRIM8 was identified in a systematic screen for positive regulators of innate immune responses. |
| 23894 | TRIM71 | 23438823 | TRIM71 was identified in a systematic screen for positive regulators of innate immune responses. |
| 107983 | TRIM58 | 23438823 | TRIM58 was identified in a systematic screen for positive regulators of innate immune responses. |
| 69064 | TRIM47 | 23438823 | TRIM47 was identified in a systematic screen for positive regulators of innate immune responses. |
| 82716 | TRIM32 | 22745133 | TRIM32 targets TMEM173 for ubiquitination and enhances the induction of IFNB against RNA and DNA viruses. |
| 82716 | TRIM32 | 23438823 | TRIM32 was identified in a systematic screen for positive regulators of innate immune responses. |

| | | | |
|---|---|---|---|
| 300948 | TRIM26 | 23438823 | TRIM26 was identified in a systematic screen for positive regulators of innate immune responses. |
| 24923 | TRIM21 | 20627395 | TRIM21 (Ro52) conjugates phosphorylated IKBKB with monoubiquitin and the IKBKB-induced NFkappa B signalling is downregulated. TRIM21-mediated monoubiquitination is involved in the subcellular translocation of active IKBKB to autophagosomes. |
| 24923 | TRIM21 | 20668674 | TRIM21 is a member of the TRIM family of single-protein E3 ligases and TRIM21-mediated ubiquitination promotes the degradation of IRF7 following TLR7 and TLR9 stimulation. As TRIM21 is also IFN-inducible, this system constitutes a negative-feedback loop that acts to protect the host from the prolonged activation of the immune response. |
| 24923 | TRIM21 | 22479513 | Tyrosine phosphorylation of the E3 ubiquitin ligase TRIM21 positively regulates interaction with IRF3 and hence TRIM21 activity. |
| 24923 | TRIM21 | 23438823 | TRIM21 was identified in a systematic screen for positive regulators of innate immune responses. |
| 43046 | MID1 | 23438823 | MID1 was identified in a systematic screen for positive regulators of innate immune responses. |
| 75795 | TRIM15 | 23077300 | TRIM15 is required for RIG-I mediated interferon production to inhibit viral replication. |
| 75795 | TRIM15 | 23438823 | TRIM15 was identified in a systematic screen for positive regulators of innate immune responses. |
| 62871 | TRIM7 | 23438823 | TRIM7 was identified in a systematic screen for positive regulators of innate immune responses. |
| 40906 | STAT2 | 8943351 | STAT2 is part of STAT-containing transcription factor, the alpha-interferon-induced ISGF3, that is composed of a STAT1:2 heterodimer and a weak DNA-binding protein, IRF9. |
| 40906 | STAT2 | 16689942 | STAT2 is a critical transactivator component of the interferon-stimulated gene factor 3 (ISGF3) complex that drives the expression of many interferon (IFN)-inducible genes. |
| 40906 | STAT2 | 23391734 | A loss-of-function mutation in STAT2 is associated with increased susceptibility to childhood viral diseases. |
| 64053 | IL1R2 | 23395675 | IL1R2 binds to IL1A to limit post-necrotic inflammation. |
| 92888 | MUL1 | 23399697 | MUL1 limits inflammation by regulating the RIG-I anti-viral response. |
| 161359 | Creb1 | 23405224 | Creb1 over-expression in myeloid cells results in increased abscess formation and aberrant cytokine response. |
| 203274 | Lgals9 | 21562126 | Lgals9 attenuates acute lung injury by preferentially suppressing pro-inflammatory functions in pDC-like macrophages through TLR2/TLR4 down-regulation. |
| 203274 | Lgals9 | 23408620 | LGALS9 engagement impairs the cytotoxicity function and cytokine production of natural killer cells. |

| | | | |
|---|---|---|---|
| 193040 | Gabarap | 23427251 | Gabarap deficient mice are more susceptible to sepsis due to enhanced inflammasome activation. |
| 102644 | S100A9 | 12626582 | S100A9 is an intracellular calcium-binding protein that promotes neutrophil/monocyte recruitment at inflamed tissues by enhancing attachment to endothelial cells. |
| 102644 | S100A9 | 18714033 | S100A9 is a myeloid-related protein that rapidly modulates macrophage nitric oxide production during innate immune response. |
| 102644 | S100A9 | 20555353 | S100A9 (calgranulin B) and S100A8 (calgranulin A) form an antimicrobial heterodimeric complex known as calprotectin. Bacterial flagellin induces the upregulation of S100A9/S100A8 heterodimer via a TLR5-dependent mechanism in epidermal keratinocytes. |
| 102644 | S100A9 | 21239714 | S100A9 forms a complex with S100A8 and the complex is the site of interplay between extracellular Ca(2+) entry and intra-phagosomal reactive oxygen species production. S100A8 :: S100A9 acts as Ca(2+) sensor in phagosomal ROS production. |
| 102644 | S100A9 | 21382888 | S100A9-deficient murine neutrophils exhibited a reduce secretion of cytokines in response to TLR4 stimulation. In contrast, S100A9-deficient dendritic cells showed an exacerbated release of cytokines after TLR stimulation. S100A9 has no effect on the inflammatory status of macrophages. (Demonstrated in murine model) |
| 102644 | S100A9 | 22423963 | S100A9 is strongly upregulated in neutrophils upon bacterial infection, and sequesters zinc as a mechanism of nutritional immunity. Salmonella typhimurium overcomes this defence mechanism by expressing a high affinity zinc transporter. (Demonstrated in mice) |
| 102644 | S100A9 | 23133376 | S100A9 forms a heterodimer with S100A8 and is a key player in protective innate immunity during Klebsiella pneumonia infection. |
| 102644 | S100A9 | 23431180 | S100A8::S100A9 heterodimer sequesters Mn(2+) and Zn(2+) to starve bacteria of these essential nutrients. |
| 126513 | MIRLET7B | 23437218 | MIRLET7B post-transcriptionally suppresses TLR4 and regulates NF-kB-mediated responses in H. pylori infection. |
| 221470 | Mir135b | 23440414 | IL1R1 upregulates Mir135b as a negative feedback regulatory mechanism to resolve cigarette smoke-induced inflammation in the lung. |
| 165889 | Tlr1 | 21439957 | Tlr1 :: Tlr2 dimeric pairs recognize malarial glycosylphosphatidylinositols (GPI) to initiates intracellular signalling and the production of pro-inflammatory cytokines. |
| 165889 | Tlr1 | 22778390 | Tlr1 is a critical innate receptor for protective intestinal T(H)17 immunity against Yersinia enterocolitica. |
| 165889 | Tlr1 | 23443468 | TLR1 in intestinal epithelium mediates activation and recruitment of dendritic cells to mount mucosal immunity against Yersinia enterocolitica. |

| | | | |
|---|---|---|---|
| 39587 | LEP | 23341537 | LEP upregulates TLR2 expression in monocytes, and may potentiate innate immunity and inflammation in hyperleptinemia conditions, such as obesity and type 2 diabetes mellitus. |
| 39587 | LEP | 23341537 | LEP (leptin) upregulates TLR2 in human monocytes. |
| 178155 | Scaf11 | 22895188 | Caspase-11 (SCAF11) increases susceptibility to Salmonella infection in the absence of caspase-1. |
| 178155 | Scaf11 | 23348507 | SCAF11 (Caspase 11) is required for innate surveillance against bacteria that escape the vacuole. |
| 177193 | Naip5 | 21282416 | Naip5 regulates the specificity of the Nlrc4 inflammasome for distinct bacterial ligands such as flagelllin. |
| 177193 | Naip5 | 21918512 | Naip5 is a NOD-like receptor protein that directly and specifically binds to bacterial flagellin. Naip5 is a universal component of the flagellin-Nlrc4 inflammasome pathway. |
| 177193 | Naip5 | 23355222 | Microglial cells employ the NAIP5/NLRC4 inflammasome to monitor and clear central nervous system infections by flagellated bacteria. |
| 194638 | Fer | 23355730 | FER is an inhibitory kinase for neutrophil chemotaxis. |
| 174863 | Rad23a | 23357418 | RAD23A is a negative regulator of the RIG-I anti-viral signalling pathway by mediating the ubiquitin-dependent proteasomal degradation of TRAF2. |
| 31509 | RAD23A | 23357418 | RAD23A is a negative regulator of the RIG-I anti-viral signalling pathway by mediating the ubiquitin-dependent proteasomal degradation of TRAF2. |
| 187527 | Lum | 23358433 | Lum facilitates early innate immune responses and orchestrates bacterial clearance and inflammation resolution in the cornea. |
| 158953 | Tacr1 | 23365459 | Agonists of TACR1 promote immunostimulatory dendritic cells by inhibiting IL10 synthesis and secretion. |
| 184281 | Nr1h4 | 23372731 | The bile acid sensor Nr1h4 (FXR) Is required for immune-regulatory activities of TLR9 in intestinal inflammation. |
| 176715 | Siglecg | 21208791 | Siglecg is a member of the SIGLEC protein family that recognize sialoside-based patterns and responds selectively to danger associated molecular patterns (DAMPs) to initiate limited innate response. |
| 176715 | Siglecg | 23374343 | Siglecg expression is upregulated by RNA viruses to suppress the RIG-I anti-viral signalling pathway. |
| 55066 | CNOT8 | 23386060 | CNOT8 negatively regulates interferon IFN/STAT1 signalling pathway and its depletion results in enhanced response to viral infection. |
| 147704 | Chat | 23297238 | Chat is expressed by lymphocytes to regulate local recruitment of neutrophils. |
| 62465 | COX5B | 23308066 | COX5B coordinates with the autophagy pathway to control MAVS aggregation and antiviral signalling. |
| 158216 | Abl1 | 23325923 | ABL1 kinase is required for neutrophil migration. |

| | | | |
|---|---|---|---|
| 89599 | ABL1 | 23325923 | ABL1 kinase is required for neutrophil migration. |
| 142854 | Prkcd | 22265677 | Prkcd is an essential signalling kinase in C-type lectin receptor-mediated innate immunity and host protection. |
| 142854 | Prkcd | 23326354 | Suppression of Prkcd expression inhibited nitric oxide production in activated macrophages. |
| 210153 | Tbx21 | 11752460 | Tbx21 (T-bet) is a member of the T-box transcription factor family that plays a critical role in interferon (IFN)-gamma regulation and promotion of cell-mediated immune responses against intracellular pathogens. |
| 210153 | Tbx21 | 23063332 | Tbx21 (T-bet) regulates the interplay between mucosal dendritic cells, innate lymphoid cells, and the intestinal microbiota. |
| 210153 | Tbx21 | 23334414 | Gradual expression of Tbx21 controls the fate and function of innate lymphoid cells. |
| 223707 | Mir212 | 23264652 | Mir212 targets Irak4 to dampen the inflammatory response in LPS-stimulated monocytes. |
| 126933 | MIR212 | 23264652 | MIR212 targets IRAK4 to dampen the inflammatory response in LPS-stimulated monocytes. |
| 221348 | Mir132 | 23264652 | Mir132 targets Irak4 to dampen the inflammatory response in LPS-stimulated monocytes. |
| 127301 | hsa-mir-132 | 20418869 | Hsa-mir-132 regulates antiviral innate immunity through suppression of the EP300 transcriptional co-activator, rather than a transcription factor or signaling protein. Furthermore, EP300 regulates hsa-mir-132 levels, revealing a dynamic equilibrium between hsa-mir-132 and EP300. |
| 127301 | hsa-mir-132 | 22822053 | MIR132 is upregulated in lung cells in response to infection with influenza A virus. |
| 127301 | hsa-mir-132 | 23264652 | MIR132 targets IRAK4 to dampen the inflammatory response in LPS-stimulated monocytes. |
| 164070 | Cryab | 23242137 | CRYAB is a suppressor of neuroinflammation, and the suppression is mediated by DRD2 in astrocytes. |
| 162803 | Drd2 | 23242137 | DRD2 activation in astrocytes suppresses neuroinflammation in the central nervous system through CRYAB. |
| 186197 | Tph1 | 23243271 | Tph1-deficient mice lack non-neuronal serotonin, and show impairment in neutrophil recruitment to sites of acute inflammation. |
| 547297 | ITGB3 | 23150579 | ITGAV::ITGB3 is a sensor and activator of innate immunity to herpes simplex virus-1. |
| 77054 | ITGAV | 23150579 | ITGAV::ITGB3 is a sensor and activator of innate immunity to herpes simplex virus-1. |
| 151717 | Fcnb | 23150716 | Fcnb is crucial for the induction of innate immunity against pneumococcal infection through the lectin complement pathway. |

| | | | |
|---|---|---|---|
| 146780 | Fcna | 20375621 | Fcna interact with mannose-binding lectin and fibrinogen/fibrin to augment the lectin complement pathway suggesting that this pathway collaborates with the coagulation system in the first-line host defence against pathogens under conditions such as injury and inflammation. |
| 146780 | Fcna | 23150716 | Fcna is crucial for the induction of innate immunity against pneumococcal infection through the lectin complement pathway. |
| 168209 | Glrx | 23159440 | Glrx deficiency impairs neutrophil polarization, chemotaxis, adhesion, and phagocytosis. |
| 606687 | Hspa1b | 23135720 | HSPA1B is released extracellularly upon measles virus infection, and induces Ifnb transcription in microglial cells. |
| 221234 | Mir497 | 23092882 | MIR497 inhibits IL1-induced IL6 transcription by targeting MAPK/ERK pathway. |
| 101151 | MOV10 | 23093941 | MOV10 associates with LINE1 ribonucleoprotein particles and restricts endogenous retrovirus transposition. |
| 207742 | Daglb | 23103940 | DAGLB is a key metabolic hub within a lipid network that regulates proinflammatory responses in peritoneal macrophages. |
| 199014 | Eif2ak2 | 22912779 | Antiviral stress granules containing Ddx58 (RIG-I) and Eif2ak2 (PKR) have a critical role in viral detection and innate immunity. |
| 199014 | Eif2ak2 | 23115300 | Herpes simplex virus evades antiviral host defence by interacting with EIF2AK2 (PKR) to inhibit autophagy. |
| 22709 | AKT1 | 21196185 | AKT1 is a Ser/Thr protein kinase that plays a pivotal role in functional activation in macrophages. AKT1 specifically functions in phagocytosis, intracellular bacterial infection, LPS tolerance, production of inflammatory cytokines/mediators, and migration during macrophage-mediate innate immunity. |
| 22709 | AKT1 | 21683629 | AKT1 activation is blocked by Bacillus anthracis, resulting in the opening of a connexin ATP release channel and induction of macrophage death. Constitutive activation of AKT1 interferes with inflammasome activation and IL1B production, which compromises antimicrobial immunity. (Demonstrated in murine model) |
| 22709 | AKT1 | 22218715 | AKT1 functions downstream of TLR2-stimulation to induce the expression of the monocyte chemoattractant protein 1, CCL2. (Demonstrated in mice) |
| 22709 | AKT1 | 23060458 | AKT1 is important for establishing the inflammatory microenvironment in the airway upon exposure to rhinovirus. |
| 52651 | AXL | 18083102 | AXL is a pleiotropic inhibitor of the innate immune response in dendritic cells, this tyrosine protein kinase is induced by IFNAR/STAT1 signalling and TLR ligation predominantly through TLR activation of the feed-forward cytokine pathway |

| | | | |
|---|---|---|---|
| 52651 | AXL | 19657094 | AXL regulates survival and migration of human dendritic cells by an IFN-alpha-inducible AXL/GAS6 pathway. |
| 52651 | AXL | 23071254 | TGF-Î²1-induced AXL enhances apoptotic cell uptake and blocks proinflammatory cytokine production in the skin. |
| 127585 | MIR187 | 23071313 | MIR187 directly targets TNFA mRNA stability and translation and acts as an effector of IL10-downstream signalling to inhibit pro-inflammatory cytokines. |
| 179253 | Pparg | 21148557 | Pparg negatively regulates Ifnb production in Tlr3/4 stimulated macrophages by preventing Irf3 binding to the Ifnb promoter. |
| 179253 | Pparg | 23071818 | The loss of PPARÎ³ in T cells increased colitis disease activity and colonic inflammatory lesions following Clostridium difficile infection. |
| 95888 | TRIM62 | 23077300 | TRIM62 is required for TRIF-mediated NFkB, AP-1 and interferon production after LPS challenge in macrophages. |
| 272448 | Mir466l | 23042536 | Mir466l directly targets Ifna expression to inhibit host antiviral immune responses. |
| 205966 | AI607873 | 23045604 | Pyhina is an activator of the STING-dependent IFN and ASC-dependent inflammasome in antiviral innate immunity. |
| 205951 | Pydc3 | 23045604 | Pydc3 is an activator of the STING-dependent IFN and ASC-dependent inflammasome in antiviral innate immunity. |
| 205927 | Pyhin1 | 23045604 | Pyhin1 is an activator of the STING-dependent IFN and ASC-dependent inflammasome in antiviral innate immunity. |
| 205894 | BC094916 | 23045604 | Pyhinb is an activator of the STING-dependent IFN and ASC-dependent inflammasome in antiviral innate immunity. |
| 205885 | Gm4955 | 23045604 | Pyblhinc is an activator of the STING-dependent IFN and ASC-dependent inflammasome in antiviral innate immunity. |
| 73191 | TRIM28 | 22995936 | TRIM28 interacts with IRF5 and mediates heterochromatinization at M1 macrophage markers (e.g. TNF) to suppress transcription. |
| 57242 | CALCOCO2 | 19820708 | CALCOCO2 recognizes ubiquitin-coated Salmonella enterica in human cells and binds to the adaptor proteins AZI2 and TBKBP1 to recruit TBK1. CALCOCO2 also recruits LC3, an autophagosomal marker, to activate autophagy against bacteria attempting to colonize their cytosol. |
| 57242 | CALCOCO2 | 20104023 | CALCOCO2 is a novel receptor for the selective autophagy of cytosolic bacteria. CALCOCO2 (NDP52) binds to the bacterial ubiquitin coat as well as to ATG8/LC3 and delivers cytosolic bacteria into autophagosomes. |

| | | | |
|---|---|---|---|
| 57242 | CALCOCO2 | 19841643 | CALCOCO2 directly binds to ubiquitinated bacteria and facilitates the assembly of an autophagic membrane that surrounds bacterial invaders. |
| 57242 | CALCOCO2 | 22901810 | CALCOCO2 and SQSTM1 are ubiquitin-autophagy receptors that are required for the recognition of extracelluar bacterial DNA by the TMEM173 (STING)-dependent cytosolic pathway, marking bacteria with ubiquitin, and delivery of bacilli to autophagosomes. (Demonstrated in mouse) |
| 57242 | CALCOCO2 | 23022382 | MAP1LC3C (LC3C) and its receptor CALCOCO2 (NDP52) are essential for antibacterial autophagy. |
| 107725 | MAP1LC3C | 23022382 | MAP1LC3C (LC3C) and its receptor CALCOCO2 (NDP52) are essential for antibacterial autophagy. |
| 147382 | Foxo3 | 22531926 | Foxo3 is an Ikbke-controlled checkpoint of IRF activation and regulation of Ifnb expression. (Demonstrated in human) |
| 147382 | Foxo3 | 22982991 | FOXO3 is a negative regulator of Irf7 transcription, and forms a coherent feed-forward regulatory circuit with Irf7 and Ifnb to maximize antiviral responses. |
| 95024 | FOXO3 | 19050264 | FOXO3 is a transcriptional activator which targets genes FAS ligand and TNFSF10 (TRAIL), involved in the extrinsic apoptotic pathway, and BBC3 (PUMA), PMAIP1 (Noxa), and BCL2L11 (Bim), which are part of the intrinsic apoptotic pathway. |
| 95024 | FOXO3 | 20739833 | FOXO3, along with AKT, is involved in TLR9-mediated anti-apoptosis and is a distinct regulator for FLICE-like inhibitory protein (FLIP) expression. |
| 95024 | FOXO3 | 22531926 | FOXO3 is an IKBKE-controlled checkpoint of IRF activation and regulation of IFNB expression. |
| 95024 | FOXO3 | 22982991 | OXO3 is a negative regulator of IRF7 transcription, and forms a coherent feed-forward regulatory circuit with IRF7 and IFNB to maximize antiviral responses. (Demonstrated in mice) |
| 115355 | VTRNA2-1 | 22986343 | VTRNA2-1 attenuates EIF2AK2 (PKR) activation, and it may act as a negative regulator of innate immune response to dsRNA viruses. |
| 149533 | Adipoq | 22948153 | ADIPOQ can induce pro-inflammatory functions in macrophages and T cells. (Demonstrated in human) |
| 69167 | ADIPOQ | 18179772 | ADIPOQ binds to C1Q and activates the classical pathway of complement. |
| 69167 | ADIPOQ | 22948153 | ADIPOQ can induce pro-inflammatory functions in macrophages and T cells. |
| 204815 | Trim56 | 21289118 | Trim56 is an anti-viral host factor that restricts pestivirus infection, specifically it was found to impair bovine viral diarrhoea virus replication. |
| 204815 | Trim56 | 22948160 | TRIM56 is an essential component of the TLR3 anti-viral signalling pathway. (Demonstrated in human) |
| 171590 | Tnfaip8l2 | 22904303 | Tnfaip8l2 is a negative regulator of immunity that controls innate immunity to RNA by targeting the PI3K-Rac pathway. |
| 171590 | Tnfaip8l2 | 22949657 | Tnfaip8l2 (TIPE2) serves as a negative regulator of phagocytosis and oxidative burst during infection. |

| | | | |
|---|---|---|---|
| 102300 | TNFAIP8L2 | 18455983 | TNFAIP8L2 is a negative regulator of innate and adaptive immunity that maintains immune homeostasis by binding to CASP8 and inhibiting nuclear factor kappa B activation while promoting apoptosis. |
| 102300 | TNFAIP8L2 | 22904303 | TNFAIP8L2 is a negative regulator of immunity that controls innate immunity to RNA by targeting the PI3K-Rac pathway. (Demonstrated in mouse) |
| 102300 | TNFAIP8L2 | 22949657 | TNFAIP8L2 (TIPE2) serves as a negative regulator of phagocytosis and oxidative burst during infection. (Demonstrated in mice) |
| 147642 | Abca1 | 22955730 | Abca1 deletion in the myeloid lineage enhances host immune response and clearance of Listeria monocytogenes. |
| 79441 | ABCA1 | 20472936 | ABCA1 promotes the efflux of bacterial lipopolysaccharide (LPS) from macrophages and accelerates recovery from LPS-induced tolerance. |
| 79441 | ABCA1 | 20650929 | ABCA1 in macrophages dampens inflammation by reducing MYD88-dependent TLR trafficking to lipid rafts, thus selectively reducing free cholesterol content in lipid rafts. |
| 79441 | ABCA1 | 22955730 | ABCA1 deletion in the myeloid lineage enhances host immune response and clearance of Listeria monocytogenes. (Demonstrated in mice) |
| 224167 | Mir467b | 22963823 | Mir467b regulates lipid accumulation and proinflammatory cytokine secretion in macrophages by targeting the LPL gene. |
| 206900 | Trp73 | 22976836 | Trp73 is required for macrophage-mediated innate immunity and the resolution of inflammatory responses. |
| 86757 | TP73 | 22976836 | TP73 is required for macrophage-mediated innate immunity and the resolution of inflammatory responses. (Demonstrated in mouse) |
| 127521 | MIR10B | 22915757 | MIR10B directly targets the 3'UTR of MICB and down-regulates its expression, which may aid tumour cells in immune escape from natural killer cells. |
| 64166 | IL1RL1 | 20400705 | IL1RL1 acts as a negative regulator of TLR2 signalling whereby over-expression of IL1RL1 can dose-dependently attenuate bacterial lipoprotein (BLP)-induced NF-kappaB activation, but is not required for BLP-induced tolerance. |
| 64166 | IL1RL1 | 20427273 | IL1RL1 is the receptor for IL33, a factor shown to modulate tryptase expression in mesenchymal cells (MCs), identifying a novel pathway by which MCs exposed to inflammatory cytokines modulate the phenotype of local MCs to shape their immune responses. |
| 64166 | IL1RL1 | 22922442 | IL1RL1 (ST2) pre-treatment suppresses cytokine production and inhibits LPS signalling in dendritic cells. |

| | | | |
|---|---|---|---|
| 39028 | IL15 | 20026737 | IL15 is a pluripotent anti-apoptotic cytokine that signals to cells of both the innate and adaptive immune system and is regarded as a highly promising immunomodulatory agent in cancer therapy. IL15 prevents two immunopathologic hallmarks of sepsis, namely, apoptosis and immunosuppression, and improves survival in two different models of sepsis. |
| 39028 | IL15 | 22084435 | IL15 regulates homeostasis and terminal maturation of NKT cells. (Demonstrated in mice) |
| 39028 | IL15 | 22940097 | IL15 secreted by inflammatory monocytes is critical for the differentiation of memory CD8(+) T and NK lymphocytes into antimicrobial effector cells. (Demonstrated in mice) |
| 45278 | IRF8 | 16484229 | IRF8 interacts with TRAF6 to modulate TLR signalling and may also contribute to the cross-talk between IFN-gamma and TLR signalling pathways. |
| 45278 | IRF8 | 18469857 | IRF8 belongs to a family of interferon (IFN) regulatory factors that modulate various important physiologic processes including host defence, cell growth and differentiation and immune regulation. |
| 45278 | IRF8 | 22942423 | De-SUMOylation of IRF8 at residue Lys310 acts as a molecular mechanism to trigger innate immune responses in activated macrophages. (Demonstrated in mice) |
| 209835 | Calcoco2 | 22901810 | Calcoco2 and Sqstm1 are ubiquitin-autophagy receptors that are required for the recognition of extracelluar bacterial DNA by the Tmem173 (STING)-dependent cytosolic pathway, marking bacteria with ubiquitin, and delivery of bacilli to autophagosomes. |
| 61811 | SQSTM1 | 19812211 | SQSTM1 (p62) targets invading bacteria to the autophagy pathway and its expression is required for efficient autophagy of bacteria, as well as restriction of their intracellular replication. |
| 61811 | SQSTM1 | 19850933 | SQSTM1 is a key intracellular target of innate defence regulator-1 (IDR-1), a synthetic peptide. |
| 61811 | SQSTM1 | 20206555 | SQSTM1 has bactericidal properties where it brings cytosolic proteins to autolysosomes where they are processed from innocuous precursors into neo-antimicrobial peptides. |
| 61811 | SQSTM1 | 20837465 | SQSTM1 and HDAC6 are important determinants of aggregated localization of MyD88 and MyD88 activation initiates a polyubiquitinated protein accumulating pathway that modulates MyD88-dependent signal transduction. |
| 61811 | SQSTM1 | 21220332 | SQSTM1 is required for TLR4-mediated autophagy. TLR4-driven induction of SQSTM1 plays an essential role in the formation and the autophagy degradation of aggresome-like induced structures, which might be critical for regulating host defence. |

| | | | |
|---|---|---|---|
| 61811 | SQSTM1 | 22901810 | SQSTM1 and CALCOCO2 are ubiquitin-autophagy receptors that are required for the recognition of extracelluar bacterial DNA by the TMEM173 (STING)-dependent cytosolic pathway, marking bacteria with ubiquitin, and delivery of bacilli to autophagosomes. (Demonstrated in mouse) |
| 166604 | Mcpt4 | 22901752 | The chymase mouse mast cell protease 4 (Mcpt4) degrades TNF, limits inflammation, and promotes survival in a model of sepsis. |
| 28456 | SCAF11 | 22895188 | Caspase-11 (SCAF11) increases susceptibility to Salmonella infection in the absence of caspase-1. (Demonstrated in mouse) |
| 175493 | Txnip | 22883233 | Hyperactivated Ern1 (IRE1Î±) increases Txnip mRNA stability by reducing levels of a Txnip destabilizing microRNA, miR-17. In turn, elevated Txnip protein activates the Nlrp3 inflammasome, causing procaspase-1 cleavage and interleukin 1 Î² (IL-1 Î²) secretion. (Demonstrated in human) |
| 43398 | NLRC4 | 18487086 | NLRC4 is part of the NOD-like receptor family of proteins that consists of more than 20 related family members, that is present in the cytosol and recognizes intracellular ligands. |
| 43398 | NLRC4 | 18280719 | NLR4C and other NLR proteins function to control IL-1, NF-kappaB, and host response to pathogens, including distinct forms of cell death. NLR4C is important for CASP1 activation and IL-1 processing. |
| 43398 | NLRC4 | 15107016 | NLRC4 expressed in monocytes associates with NOD2 following exposure to bacterial peptidoglycan, implying a regulatory role for interaction of NACHT-domain containing proteins in the innate immune response. |
| 43398 | NLRC4 | 20133635 | NLR4C detects the basal body rod component of the T3SS apparatus (rod protein) from S. typhimurium (PrgJ), Burkholderia pseudomallei (BsaK), Escherichia coli (EprJ and EscI), Shigella flexneri (MxiI), and Pseudomonas aeruginosa (PscI) and the specific detection of the virulence machinery permits the discrimination between pathogenic and non-pathogenic bacteria. |
| 43398 | NLRC4 | 22174673 | The NLRC4 inflammasome is important for control of mucosal Candida infection, impacting on inflammatory cell recruitment to infected tissues, as well as protecting against the systemic dissemination of infection. (Demonstrated in mouse) |
| 43398 | NLRC4 | 22231517 | Flagellin-induced NLRC4 inflammasome activation in splenic dendritic cells triggers antigen-independent IFN-gamma production by memory T cells. (Demonstrated in mouse) |
| 43398 | NLRC4 | 22547706 | NLRC4 is important for host survival and bacterial clearance, as well as neutrophil-mediated inflammation in the lungs following Klebsiella pneumoniae infection. (Demonstrated in mouse) |

| | | | |
|---|---|---|---|
| 43398 | NLRC4 | 22484733 | NLRC4-dependent production of IL1B by intestinal phagocytes is a mechanism that discriminates pathogenic from commensal bacteria in the intestinal host defence. (Demonstrated in mouse) |
| 43398 | NLRC4 | 22885697 | NLRC4 Ser â € 533 phosphorylation is essential for procaspase-1 recruitment to the NLRC4 inflammasome complex after S. typhimurium infection. (Demonstrated in mouse) |
| 150825 | Il12a | 22888135 | Il12, consisting of Il12a and Il12b subunits, induces Il2ra to form high-affinity Il2 receptors on natural killer cells in response to mouse cytomegalovirus infection. |
| 166933 | Tlr13 | 22821982 | Tlr13 recognizes a conserved 23S rRNA sequence that is the binding site of macrolide, lincosamide, and streptogramin group (MLS) antibiotics in bacteria. This reveals that specific mechanisms of antibiotic resistance are potent bacterial immune evasion strategies for avoiding recognition via Tlr13. |
| 129350 | Nlrc3 | 22863753 | Nlrc3 inhibits Toll-like receptor (TLR)-dependent activation of NF-kB by interacting with the TLR signalling adaptor Traf6 to attenuate ubiquitination of Traf6 and activation of NF-kB. |
| 12133 | NLRC3 | 22863753 | NLRC3 inhibits Toll-like receptor (TLR)-dependent activation of NF-kB by interacting with the TLR signalling adaptor TRAF6 to attenuate ubiquitination of TRAF6 and activation of NF-kB. (Demonstrated in mouse) |
| 126947 | MIR378 | 22855601 | MIR378 is specifically induced by IL4 via the IL-4-Receptor/PI3K/AKT signalling pathway to drive macrophage proliferation. (Demonstrated in mouse ) |
| 129229 | Cftr | 21712022 | Cftr deficiency alters the innate immunity of the biliary epithelium and reduces endotoxin tolerance, resulting in increased inflammatory response mediated by Tlr4 and NFkB. |
| 129229 | Cftr | 22859830 | Cftr is involved in myeloid cell function and its absence from myeloid-derived cells slows resolution of inflammation and infection of the lung. |
| 37713 | CFTR | 21712022 | CFTR deficiency alters the innate immunity of the biliary epithelium and reduces endotoxin tolerance, resulting in increased inflammatory response mediated by TLR4 and NFkB. |
| 37713 | CFTR | 22859830 | CFTR is involved in myeloid cell function and its absence from myeloid-derived cells slows resolution of inflammation and infection of the lung. (Demonstrated in mouse) |
| 212451 | Mmp9 | 21873432 | Mmp9 and Neu1 cross-talk in alliance with Tlr4 on the cell surface is a novel membrane sialidase-controlling mechanism that depends on ligand binding to its Toll-like receptor (TLR) to induce Neu1 activity, to influence receptor desialylation and subsequently to induce TLR receptor activation and the production of nitric oxide and pro-inflammatory cytokines in dendritic and macrophage cells. |

| | | | |
|---|---|---|---|
| 212451 | Mmp9 | 22496659 | Mmp9 inhibits influenza virus pathogenesis by mediating neutrophil migration to the respiratory tract. |
| 212451 | Mmp9 | 22860023 | Mmp9 cleaves the pulmonary collectin Sftpd (SP-D) leading to loss of its innate immune functions. |
| 78722 | MMP9 | 21873432 | MMP9 and NEU1 cross-talk in alliance with TLR4 on the cell surface is a novel membrane sialidase-controlling mechanism that depends on ligand binding to its Toll-like receptor (TLR) to induce NEU1 activity, to influence receptor desialylation and subsequently to induce TLR receptor activation and the production of nitric oxide and pro-inflammatory cytokines in dendritic and macrophage cells. (Demonstrated in murine model) |
| 78722 | MMP9 | 22496659 | MMP9 inhibits influenza virus pathogenesis by mediating neutrophil migration to the respiratory tract. (Demonstrated in mice) |
| 78722 | MMP9 | 22860023 | MMP9 cleaves the pulmonary collectin SFTPD (SP-D) leading to loss of its innate immune functions. (Demonstrated in mouse) |
| 148706 | Ahsg | 22842477 | Ahsg is an endogenous ligand of Tlr4 that promotes inflammatory signalling leading to lipid-induced insulin resistance. |
| 68916 | AHSG | 22842477 | AHSG is an endogenous ligand of TLR4 that promotes inflammatory signalling leading to lipid-induced insulin resistance. (Demonstrated in mice) |
| 132962 | Map3k7 | 21220427 | Map3k7 activation is impaired during endotoxin tolerization; a process which impairs the production of LPS-induced pro-inflammatory cytokines without inhibition expression of anti-inflammatory or anti-microbial mediators. |
| 132962 | Map3k7 | 22069318 | Map3k7 polyubiquitination is essential for the activation of NF-kB signalling downstream of TNF receptor, IL1 receptor and Tlr4. |
| 132962 | Map3k7 | 22843747 | Map3k7 is necessary for the neutrophil priming effect of leukotriene B (4) to enhance TLR stimulation. |
| 183156 | Olfm4 | 22844115 | Olfm4 is a negative regulator of neutrophil bactericidal activity by restricting cathepsin C-mediated protease activities. |
| 36123 | OLFM4 | 20534456 | OLFM4 exerts considerable influence on the host defence against H. pylori infection acting through NOD1 and NOD2 mediated NF-kappaB activation and subsequent cytokines and chemokines production, which in turn inhibit host immune response and contribute to persistence of H. pylori colonization. |
| 36123 | OLFM4 | 22844115 | OLFM4 is a negative regulator of neutrophil bactericidal activity by restricting cathepsin C-mediated protease activities. (Demonstrated in mice) |

| | | | |
|---|---|---|---|
| 167243 | Itgb2 | 21245270 | Itgam :: Itgb2 is the principal leukocyte receptor involved in the recognition of the fungus Candida albicans. Recognition of Pra1p protein of C. albicans by Itgam :: Itgb2 plays a pivotal role in determining fungal virulence, and host response/protection against C. albicans infection. |
| 167243 | Itgb2 | 22396536 | Itgb2 is involved in cell-cell contact signalling between activated apoptotic lymphocytes and dendritic cells (DC) during the maturation of DCs. (Demonstrated in human) |
| 167243 | Itgb2 | 22844116 | Itgax::Itgb2 is a leukocyte receptor for Candida albicans and is essential for protection against fungal infections. |
| 5590 | ITGB2 | 21245270 | ITGAM :: ITGB2 is the principal leukocyte receptor involved in the recognition of the fungus Candida albicans. Recognition of Pra1p protein of C. albicans by ITGAM :: ITGB2 plays a pivotal role in determining fungal virulence, and host response/protection against C. albicans infection. (Demonstrated in murine model) |
| 5590 | ITGB2 | 22396536 | ITGB2 is involved in cell-cell contact signalling between activated apoptotic lymphocytes and dendritic cells (DC) during the maturation of DCs. |
| 5590 | ITGB2 | 22844116 | ITGAX::ITGB2 is a leukocyte receptor for Candida albicans and is essential for protection against fungal infections. |
| 210550 | Itgax | 21775452 | Itgax is the main marker for identification of dendritic cells (DCs). Itgax expression negatively orchestrates both adaptive and innate immunity against herpes simplex virus type 1 (HSV-1) ocular infection via higher activities of type 1 interferon and CD8(+) T cell responses. |
| 210550 | Itgax | 22844116 | Itgax::Itgb2 is a leukocyte receptor for Candida albicans and is essential for protection against fungal infections. |
| 28212 | ITGAX | 21775452 | ITAGX is the main marker for identification of dendritic cells (DCs). ITGAX expression negatively orchestrates both adaptive and innate immunity against herpes simplex virus type 1 (HSV-1) ocular infection via higher activities of type 1 interferon and CD8(+) T cell responses. (Demonstrated in murine model) |
| 28212 | ITGAX | 22844116 | ITGAX::ITGB2 is a leukocyte receptor for Candida albicans and is essential for protection against fungal infections. |
| 190983 | Ikbke | 21138416 | Ikbke and other IKK kinases regulate each other by an intricate network involving phosphorylation of their catalytic and regulatory (NEMO, TANK) subunits to balance their activities during innate immunity. |

| | | | |
|---|---|---|---|
| 190983 | Ikbke | 21822257 | Ikbke kinase modulates Il-17 signalling through Traf3ip2 adaptor protein phosphorylation, resulting in the induction of neutrophilia and pulmonary inflammation. |
| 190983 | Ikbke | 22065572 | Ikbke is an important signalling molecule in the IFN pathway and is necessary to mount anti-viral immunity against West Nile virus. |
| 190983 | Ikbke | 22171011 | Ikbke plays a critical role in regulating the balance between the type I and type II interferon (IFN) signalling pathways. |
| 190983 | Ikbke | 22532683 | Ikbke is sequestered by arenavirus nucleoproteins to block its autocatalytic activity and its ability to active Irf3. (Demonstrated in human) |
| 204703 | Masp2 | 22792067 | Masp2 deficient mice are defective in the lectin pathway of complement activation and are highly susceptible to pneumococcal infection. |
| 145846 | Ms4a8a | 22806454 | Ms4a8a expression is induced by TLR signalling in M2-like macrophages. Ms4a8a is expressed at the late stages of Trypanosoma congolense and Taenia crassiceps infections. |
| 49293 | MS4A8B | 22806454 | MS4A8B expression is induced by TLR signalling in M2-like macrophages. MS4A8B is expressed at the late stages of Trypanosoma congolense and Taenia crassiceps infections. (Demonstrated in mice) |
| 178813 | Casp7 | 22807671 | Host activation of Casp7 in response to pore formation during Listeria monocytogene infection represents an adaptive mechanism by which host cells can protect membrane integrity during infection. |
| 90066 | CASP7 | 18667412 | CASP7 is a substrate of the CASP1 inflammasomes, demonstrating the existence of a nucleotide binding and oligomerization domain-like receptor/CASP1/CASP7 cascade and the existence of distinct activation mechanisms for CASP3 and CASP7 in response to microbial stimuli and bacterial infection. |
| 90066 | CASP7 | 19343209 | CASP7 activation by the NLRC4 inflammasome restricts Legionella pneumophila infection in mice. |
| 90066 | CASP7 | 22807671 | Host activation of CASP7 in response to pore formation during Listeria monocytogene infection represents an adaptive mechanism by which host cells can protect membrane integrity during infection. (Demonstrated in mice) |
| 281264 | MIR1275 | 22822053 | MIR1275 is upregulated in lung cells in response to infection with influenza A virus. |
| 127093 | MIR200C | 22822053 | MIR200C is upregulated in lung cells in response to infection with influenza A virus. |
| 126579 | MIRLET7C | 22835429 | MIRLET7C down-regulates IL10 expression. |
| 137597 | Pura | 22835829 | Pura expression is repressed by endogenous miRNA to restrict HIV-1 infection in monocytes. (Demonstrated in human) |
| 48322 | PURA | 22835829 | PURA expression is repressed by endogenous miRNA to restrict HIV-1 infection in monocytes. |

| | | | |
|---|---|---|---|
| 184519 | Ace2 | 22837003 | Ace2 deficiency results in increased susceptibility to intestinal inflammation induced by epithelial damage. Ace2 links amino acid malnutrition to altered gut microbiota, which leads to colitis susceptibility. |
| 46534 | ACE2 | 22837003 | ACE2 deficiency results in increased susceptibility to intestinal inflammation induced by epithelial damage. ACE2 links amino acid malnutrition to altered gut microbiota, which leads to colitis susceptibility. (Demonstrated in mice) |
| 196687 | Eif4e | 22544393 | The regulated phosphorylation of Eif4e has a key role in antiviral host defense by selectively controlling the translation of Nfkbia mRNA, which encodes a critical suppressor of the innate antiviral response. As a result, Eif4e null mice exhibit lower susceptibility to viral infections. |
| 30970 | EIF4E | 22544393 | The regulated phosphorylation of EIF4E has a key role in antiviral host defense by selectively controlling the translation of NFKBIA mRNA, which encodes a critical suppressor of the innate antiviral response. As a result, EIF4E null mice exhibit lower susceptibility to viral infections. (Demonstrated in mice) |
| 170705 | Edil3 | 22447028 | The leukocyte integrin antagonist (Edil3) Del-1 inhibits IL-17-mediated inflammatory bone loss. |
| 32547 | EDIL3 | 22447028 | The leukocyte integrin antagonist (EDIL3) Del-1 inhibits IL-17-mediated inflammatory bone loss. (Demonstrated in mice) |
| 2147 | MAPK1 | 22447027 | MAPK1 (ERK) and MAPK14 (p38) control the dynamic balance regulating neutrophil migration. |
| 71189 | NLRP4 | 15107016 | NLRP4 binds with NLRC4 through NACHT domain, which by mediating hetero-oligomerization, creates protein-interaction networks that potentially modulate immune responses to invading pathogens. |
| 71189 | NLRP4 | 22388039 | NLRP4 regulates the activation of type I interferon triggered by dsRNA or dsDNA. |
| 788 | NRIP1 | 22388040 | NF-Î°B-mediated degradation of the coactivator NRIP1 (RIP140) regulates inflammatory responses and contributes to endotoxin tolerance. (Demonstrated in mice) |
| 788 | NRIP1 | 22388040 | NRIP1 is degraded by the NF-kB pathway to inactivate inflammatory gene expression and promotes endotoxin tolerance. (Demonstrated in mice) |
| 171427 | Btk | 21441935 | Btk interacts with intracellular MHC class II molecules to activate adaptor molecules Myd88 and Ticam1 to promote TLR signalling. |
| 171427 | Btk | 21659545 | Btk is a positive regulator in the ITAM-mediated Trem1/Tyrobp pathway, which induces pro-inflammatory cytokines such as TNF-alpha, Il8, and activation/differentiation cell surface markers. |

| | | | |
|---|---|---|---|
| 171427 | Btk | 22454496 | Btk directly phosphorylates Tlr3 and plays a critical role in the induction of inflammatory cytokines and Ifnb. |
| 171427 | Btk | 22589540 | Btk is required for the activation of natural killer cells. |
| 171427 | Btk | 22366891 | Btk is a negative regulator of TLR- or TNF-stimulated reactive oxygen species (ROS) production in neutrophils. (Demonstrated in human) |
| 187492 | Zc3h12a | 22037600 | Zc3h12a prevents autoimmunity by controlling the stability of cytokine mRNA, such as Il6. Zc3h12a protein degradation is facilitated by IKK complex, which acts downstream of TLR stimulation. |
| 187492 | Zc3h12a | 22777400 | Zc3h12a is a potent regulator of innate immunity, which can be strongly engaged in the pathogenesis of acute and chronic infective diseases. (Demonstrated in human) |
| 96402 | ZC3H12A | 22037600 | ZC3H12A prevents autoimmunity by controlling the stability of cytokine mRNA, such as IL6. ZC3H12A protein degradation is facilitated by IKK complex, which acts downstream of TLR stimulation. (Demonstrated in mice) |
| 96402 | ZC3H12A | 22777400 | ZC3H12A is a potent regulator of innate immunity, which can be strongly engaged in the pathogenesis of acute and chronic infective diseases. |
| 13627 | TLR1 | 18635889 | TLR1 polymorphisms affect innate immune responses and outcomes in sepsis. |
| 13627 | TLR1 | 11932926 | TLR1 restrains potentially dangerous innate response to LPS by binding to TLR4 and preventing the formation of active signalling complexes. |
| 13627 | TLR1 | 12077222 | TLR1 interacts with TLR2 to recognize the lipid configuration of the native mycobacterial lipoprotein as well as several triacylated lipopeptides. |
| 13627 | TLR1 | 12697090 | TLR1 and TLR6 are involved in the discrimination of a subtle difference between triacyl and diacyl lipopeptides through interaction with TLR2. |
| 13627 | TLR1 | 21439957 | TLR1 :: TLR2 dimeric pairs recognize malarial glycosylphosphatidylinositols (GPI) to initiates intracellular signalling and the production of pro-inflammatory cytokines. |
| 13627 | TLR1 | 22778390 | TLR1 is a critical innate receptor for protective intestinal T(H)17 immunity against Yersinia enterocolitica. (Demonstrated in mice) |
| 147059 | P2ry14 | 22778393 | Deletion of P2ry14 inhibits macrophage recruitment and tissue inflammation, which mitigate diet-induced insulin resistance. |
| 61501 | P2RY14 | 22778393 | Deletion of P2RY14 inhibits macrophage recruitment and tissue inflammation, which mitigate diet-induced insulin resistance. (Demonstrated in mice) |
| 207813 | Dusp10 | 22307906 | Dusp10 protects against sepsis-induced acute lung injury. |
| 106785 | DUSP10 | 22307906 | DUSP10 protects against sepsis-induced acute lung injury. (Demonstrated in mice) |

| | | | |
|---|---|---|---|
| 210416 | E2f1 | 21131441 | E2f1 is important for normal inflammatory response to systemic LPS by enhancing the production of Il6 and Tnfa in murine macrophages. |
| 210416 | E2f1 | 22310660 | E2f1 directly binds to the promoter of Tlr3 to inhibit transcription. |
| 67214 | E2F1 | 21131441 | E2F1 is important for normal inflammatory response to systemic LPS by enhancing the production of IL6 and TNFA in macrophages. (Demonstrated in murine model) |
| 67214 | E2F1 | 22310660 | E2F1 directly binds to the promoter of TLR3 to inhibit transcription. (Demonstrated in mice) |
| 179829 | Rb1 | 22310660 | Rb1 positively regulates Tlr3 expression by modulating the transcription factor E2f1. |
| 32092 | RB1 | 22310660 | RB1 positively regulates TLR3 expression by modulating the transcription factor E2F1. (Demonstrated in mice) |
| 137812 | Angpt1 | 22015631 | Angpt1 promotes IL8 synthesis and release in neutrophils. (Demonstrated in human) |
| 32292 | ANGPT1 | 22015631 | ANGPT1 promotes IL8 synthesis and release in neutrophils. |
| 156241 | Dok3 | 22761938 | Dok3 is a negative regulator of TLR signalling by limiting LPS-induced ERK activation and cytokine responses in macrophages. |
| 60370 | DOK3 | 22761938 | DOK3 is a negative regulator of TLR signalling by limiting LPS-induced ERK activation and cytokine responses in macrophages. (Demonstrated in mice) |
| 168384 | Dicer1 | 22252463 | Down-regulation of Dicer1 elicits an interferon response in endometrial cancer cells. |
| 18522 | DICER1 | 22252463 | Down-regulation of DICER1 elicits an interferon response in endometrial cancer cells. |
| 16041 | NLRP6 | 12633874 | Regulator of NF-kappaB and CASP1, angiotensin II and vasopressin receptor |
| 16041 | NLRP6 | 22763455 | NLRP6 is a negative regulator of inflammatory signalling and impedes the clearance of both Gram-positive and -negative bacterial pathogens. (Demonstrated in mice) |
| 156706 | Trim32 | 22745133 | Trim32 targets Tmem173 for ubiquitination and enhances the induction of Ifnb against RNA and DNA viruses. (Demonstrated in human) |
| 209544 | Tufm | 22749352 | Tufm inhibits RLR-induced IFN-I and promotes autophagy during viral infection. (Demonstrated in human) |
| 23469 | TUFM | 22749352 | TUFM inhibits RLR-induced IFN-I and promotes autophagy during viral infection. |
| 74346 | NLRX1 | 18311173 | NLRX1 is an amplifier of ROS generation and an inhibitor of antiviral mitochondrial signalling with markedly increased IFN- Î ² responses in cells with down-regulated NLRX1 expression. |

| | | | |
|---|---|---|---|
| 74346 | NLRX1 | 18200010 | NLRX1 depletion via siRNA promotes virus-induced type 1 interferon production and decreases viral replication. |
| 74346 | NLRX1 | 18280719 | NLRX1 is an important regulator of the type I interferon (IFN) response, and prevents MAVS from signalling the activation of a type I IFN response by the RNA helicases |
| 74346 | NLRX1 | 21703540 | NLRX1 deletion leads to constitutive interaction of MAVS and DDX58, resulting in increased inflammation. In addition to attenuating the antiviral response, NLRX1 directly associates with TRAF6 and inhibits NFkB pathway in LPS-activated macrophages. |
| 74346 | NLRX1 | 21703539 | NLRX1 negatively regulates TLR-mediated NFkB activation by directly interacting with TRAF6 or IKK kinase. Nlrx1 knockdown in mice enhances their susceptibility to LPS-induced septic shock and increases plasma IL6 levels. |
| 74346 | NLRX1 | 22749352 | NLRX1 attenuates type I IFN production and promotes autophagy during viral infection. |
| 221242 | mmu-mir-29a | 21785411 | mmu-mir-29a suppresses immune responses to intracellular pathogens by targeting IFN-gamma mRNA. Mice infected with Listeria monocytogenes or Mycobacterium bovis bacillus Calmette-Gu Ã © rin (BCG) downregulated miR-29 expression. |
| 221242 | mmu-mir-29a | 22179202 | mmu-mir-29a reduces the expression of IFN-alpha receptor and is critical for dampening the sensitivity of thymic epithelium to infection signals. |
| 221242 | mmu-mir-29a | 22753494 | Mir29a is secreted by tumour cells in the form of exosomes, and binds to intracellular Tlr7 in immune cells to trigger a prometastatic inflammatory response. |
| 198032 | Gnai2 | 21255617 | Gnai2 is regulated by Tlr signaling and plays an anti-inflammatory role in endotoxema and polymicrobial sepsis. |
| 198032 | Gnai2 | 22581266 | Sustained activation of Gnai2 dampens Tnf and Il6 production stimulated by Tlr2/4 ligands. |
| 36105 | GNAI2 | 21255617 | GNAI2 is regulated by TLR signalling and plays an anti-inflammatory role in endotoxema and polymicrobial sepsis. (Demonstrated in murine model) |
| 36105 | GNAI2 | 22581266 | Sustained activation of GNAI2 dampens TNF and IL6 production stimulated by TLR2/4 ligands. (Demonstrated in mice) |
| 144109 | Spink5 | 22588119 | Spink5 binds to antimicrobial peptide dermcidin (DCD) and is involved in the trafficking of DCD in the epidermis. (Demonstrated in human) |
| 52343 | SPINK5 | 22588119 | SPINK5 binds to antimicrobial peptide dermcidin (DCD) and is involved in the trafficking of DCD in the epidermis. |
| 177285 | Lst1 | 22589543 | Lst1 recruits tyrosine-phosphorylated Ptpn6 and Ptpn11 to the plasma membrane in myeloid leukocytes. (Demonstrated in human) |

| | | | |
|---|---|---|---|
| 77937 | LST1 | 22589543 | LST1 recruits tyrosine-phosphorylated PTPN6 and PTPN11 to the plasma membrane in myeloid leukocytes. |
| 201281 | Scarb1 | 22589557 | Scarb1 regulates the macrophage inflammatory response to LPS. |
| 63952 | SCARB1 | 22589557 | SCARB1 regulates the macrophage inflammatory response to LPS. (Demonstrated in mice) |
| 274007 | Spag11a | 22535201 | Spag11a is a beta-defensin expressed in epididymis. Overexpression of Spag11a confers resistance to E. coli infection in epididymis. |
| 174941 | Tsc22d3 | 22539300 | Tsc22d3 is downregulated following TLR activation in macrophages. |
| 81583 | TSC22D3 | 22539300 | TSC22D3 is downregulated following TLR activation in macrophages. |
| 36000 | NOS2 | 20157607 | NOS2 is a nitric oxide synthase (iNOS) protein and combined treatment with 1,25-dihydroxyvitamin D3 (1,25-D3) and IFN-gamma has been shown to synergistically enhance NO synthesis and NOS2 expression induced by Mycobacterium tuberculosis (MTB) or by its purified protein derivatives in human monocyte-derived macrophages. |
| 36000 | NOS2 | 18799752 | NOS2 is an inducible nitric oxide (NO) synthase present in innate immune cells that produces NO in response to certain infections or upon stimulation with cytokines such as IFN-gamma and TNF. For optimal induction of NOS2 during Chlamydophila pneumoniae infection, the concerted action of the MyD88-dependent transcription factors NF-kappaB and AP-1 and of the MyD88-independent transcription factors phosphorylated STAT1 and IRF1 is required. |
| 36000 | NOS2 | 16893173 | NOS2 (iNOS) and CALM1 (CaM) coordinately function to form a stable complex that is part of a rapid host response that functions within the first 30 min following bacterial infection to up-regulate the innate immune system involving macrophage activation. |
| 36000 | NOS2 | 22542147 | Nitric oxide production by NOS2 promotes Listeria monocytogenes dissemination in the host. (Demonstrated in mice) |
| 18428 | CLEC7A | 15956283 | CLEC7A (Dectin-1) is a C-type lectin receptor that binds beta-glucan and is the primary receptor on macrophages for phagocytosis of various fungi. |
| 18428 | CLEC7A | 17698636 | CLEC7A synergizes with TLR2 to increase pro-inflammatory cytokine responses to pathogens. |
| 18428 | CLEC7A | 19633936 | CLEC7A has a critical role in the innate immune response against Mycobacterium tuberculosis in non-phagocytic cells in type II airway epithelial cells. |
| 18428 | CLEC7A | 20100861 | CLEC7A-dependent immune-cell recognition of beta glucan on the fungal cell-wall is modulated by the Candida albicans ERK-like-1 (CEK1)-mediated MAPK pathway, disruption of which causes enhanced cell-wall beta-glucan exposure triggering immune responses more efficiently than wild-type yeast. |

| | | | |
|---|---|---|---|
| 18428 | CLEC7A | 21267996 | CLEC7A, a C-type lectin receptor, is a pattern recognition receptor critical for immune responses to fungi. CLEC7A is coupled to SYK kinase and signals via CARD9 to activate NFKB, which in turns induces both innate and adaptive immunity. |
| 18428 | CLEC7A | 21677049 | CLEC7A is expressed mainly in dendritic cells and macrophages. CLEC7A recognizes beta-glucans with its carbohydrate recognition domain and transduces signals through its ITAM-like motif in the cytoplasmic region, which recruits SYK and initiates the CARD9/NFkB signalling cascade. |
| 18428 | CLEC7A | 22267217 | CLEC7A acts as an extracellular sensor for fungi and mycobacteria that induce both IL1B production and maturation for protective immunity. |
| 18428 | CLEC7A | 22543832 | The functional activity of CLEC7A in mucosal immunity to Candida albicans is dependent on the genetic background of the host. (Demonstrated in mice) |
| 171775 | Hamp | 20530874 | Hamp is a peptide hormone that regulates iron homeostasis and acts as an antimicrobial peptide, functioning in modulating acute inflammatory responses by mediating transcriptional changes. |
| 171775 | Hamp | 21572427 | Hamp (hepcidin), an iron regulatory hormone, inhibits the liver-stage infection in malarial parasitemia by arresting Plasmodium sporozoites in liver hepatocytes and prevents their development into blood-stage parasites. |
| 171775 | Hamp | 21685415 | Hamp expression in LPS or Tnfa-stimulated peripheral blood leukocytes is dependent on the NFkB signalling pathway. (Demonstrated in human) |
| 171775 | Hamp | 22544439 | Hamp expression is induced by Tlr2/Tlr4 ligands and may regulate pro-inflammatory cytokine production through maintenance of iron homeostasis in macrophages. |
| 43921 | HAMP | 21572427 | HAMP (hepcidin), an iron regulatory hormone and antimicrobial peptide, inhibits the liver-stage infection in malarial parasitemia by arresting Plasmodium sporozoites in liver hepatocytes and prevents their development into blood-stage parasites. (Demonstrated in murine model) |
| 43921 | HAMP | 21685415 | HAMP expression in LPS or TNFA-stimulated peripheral blood leukocytes is dependent on the NFkB signalling pathway. |
| 43921 | HAMP | 22544439 | HAMP expression is induced by TLR2/TLR4 ligands and may regulate pro-inflammatory cytokine production through maintenance of iron homeostasis in macrophages. (Demonstrated in mice) |
| 214493 | Rhbdf2 | 22550345 | Rhbdf2 is required for the secretion of Tnf in macrophages. |

| | | | |
|---|---|---|---|
| 69739 | RHBDF2 | 22550345 | RHBDF2 is required for the secretion of TNF in macrophages. (Demonstrated in mice) |
| 191069 | Plscr1 | 22453241 | Plscr1 regulates Tlr9 trafficking to endosomal compartment and plays an important role in the induction of type I IFN response in plasmocytoid dendritic cells. |
| 60169 | PLSCR1 | 22453241 | PLSCR1 regulates TLR9 trafficking to endosomal compartment and plays an important role in the induction of type I IFN response in plasmocytoid dendritic cells. |
| 57812 | DUSP1 | 18504304 | DUSP1 inhibits MAPK pathways and decreases TLR signalling. |
| 57812 | DUSP1 | 19436832 | DUSP1 is an essential feedback regulator of the innate immune response, it plays a critical role in preventing septic shock and multi-organ dysfunction during pathogenic infection and plays a pivotal role in the deactivation of MAPK1 and MAPK8. |
| 57812 | DUSP1 | 21959016 | DUSP1 is a negative regulator of MAPK-dependent induction of IL6 and IL8 in response to the coronavirus infectious bronchitis virus (IBV). |
| 57812 | DUSP1 | 22464096 | DUSP1 antagonizes p38 MAPK activity to induce IL12B expression, and may play a role in the development of Th1 type immune response and anti-microbial defence. (Demonstrated in mice) |
| 135441 | Ly96 | 21712422 | Tlr4::Ly96 functions as intracellular LPS sensor and triggers a unique set of LPS responses upon recognition of phagocytosed bacteria in macrophages. |
| 135441 | Ly96 | 22433852 | Amino acid Phe126 of Ly96 acts as a hydrophobic switch driving agonist-dependent contacts necessary for Tlr4 dimerization and activation. (Demonstrated in human) |
| 135441 | Ly96 | 22474354 | Morphine binds to Ly96, triggering Tlr4 oligomerization and induces neuroinflammation within the central nervous system. (Demonstrated in human) |
| 25842 | LY96 | 19840871 | LY96 (MD-2) recognizes only exogenous pathogen-associated molecular patterns (PAMPs) when complexed with TLR2-CD14 or TLR4-CD14. |
| 25842 | LY96 | 15852007 | LY86 and CD180 interact directly with the TLR4 signalling complex, inhibiting its ability to bind microbial ligand. |
| 25842 | LY96 | 10359581 | LY96 is physically associated with TLR4 on the cell surface and confers responsiveness to lipopolysaccharide (LPS). |
| 25842 | LY96 | 21712422 | TLR4::LY96 functions as intracellular LPS sensor and triggers a unique set of LPS responses upon recognition of phagocytosed bacteria in macrophages. (Demonstrated in murine model) |
| 25842 | LY96 | 22433852 | Amino acid Phe126 of LY96 acts as a hydrophobic switch driving agonist-dependent contacts necessary for TLR4 dimerization and activation. |

| | | | |
|---|---|---|---|
| 25842 | LY96 | 22474354 | Morphine binds to LY96, triggering TLR4 oligomerization and induces neuroinflammation within the central nervous system. |
| 409454 | LILRA2 | 22479404 | LILRA2-activation of monocytes is distinct from LPS activation, as assessed by the secreted cytokine profile. LILRA2 cross-linking also results in inhibition of FCGR1A-dependent phagocytosis in monocytes. |
| 202909 | Trim21 | 22479513 | Tyrosine phosphorylation of the E3 ubiquitin ligase Trim21 positively regulates interaction with Irf3 and hence Trim21 activity. |
| 191954 | Clec9a | 22483800 | Clec9a is restricted to dendritic cells, and specifically recognizes filamentous actin from necrotic cells. Clec9a ligand engagement is necessary for the priming of cytotoxic T cells against necrotic cell antigens. (Demonstrated in human) |
| 18377 | CLEC9A | 22483800 | CLEC9A is restricted to dendritic cells, and specifically recognizes filamentous actin from necrotic cells. CLEC9A ligand engagement is necessary for the priming of cytotoxic T cells against necrotic cell antigens. |
| 157012 | Pacsin1 | 22488361 | Pacsin1 regulates the Tlr7/9-mediated type I interferon response in plasmacytoid dendritic cells. |
| 83573 | PACSIN1 | 22488361 | PACSIN1 regulates the TLR7/9-mediated type I interferon response in plasmacytoid dendritic cells. |
| 192499 | Irak3 | 22154382 | Irak3 dampens the innate immune response in airway epithelial cells by inhibiting Tlr2 signalling in an Il13-dependent manner. (Demonstrated in human) |
| 192499 | Irak3 | 22492852 | Irak3 impairs host defence during pneumococcal pneumonia at the primary site of infection. |
| 45584 | IRAK3 | 17982103 | IRAK3 upregulation by lipopolysaccharide (LPS) is largely dependent on TNF-alpha and LPS tolerance in human endotoxemia models is associated with IRAK3 up-regulation. |
| 45584 | IRAK3 | 17379480 | IRAK3 selectively attenuates Pam3CSK4-induced MAPK14 (p38) activation through an IRAK1 independent and MKP1 dependent pathway. |
| 45584 | IRAK3 | 12620219 | IRAK3 is part of the interleukin-1 receptor-associated kinase (IRAK) family of proteins that mediate activation of nuclear factor-kappaB (NF-kappaB) and mitogen-activated protein kinase (MAPK) pathways. |
| 45584 | IRAK3 | 19809574 | IRAK3 is one of the negative regulators that contribute to the attenuation of NF-kappaB activation and it negatively regulates the alternative NF-kappaB pathway in a ligand-specific manner. |
| 45584 | IRAK3 | 14660668 | IRAK3 induction and inhibition of kinase activity of IRAK1 are crucial to peptidoglycan-induced tolerance in macrophages. |
| 45584 | IRAK3 | 12150927 | IRAK3 regulates Toll-like receptor (TLR) signalling and innate immune homeostasis where IRAK3 is induced upon TLR stimulation and negatively regulates TLR signalling. |

| | | | |
|---|---|---|---|
| 45584 | IRAK3 | 20042589 | IRAK3 is critical to preventing deleterious neutrophil-dependent lung injury during influenza infection of the respiratory tract. |
| 45584 | IRAK3 | 21098228 | IRAK3 is strongly expressed in resting alveolar macrophages and blunts TNF expression to protect the host from the injurious effect of bacterial-induced inflammation. The inhibitory function of IRAK3 is abolished when it is cleaved by CASP6, which allows the induction of the NFKB pathway and the expression of TNF. |
| 45584 | IRAK3 | 22154382 | IRAK3 dampens the innate immune response in airway epithelial cells by inhibiting TLR2 signalling in an IL13-dependent manner. |
| 45584 | IRAK3 | 22492852 | IRAK3 impairs host defence during pneumococcal pneumonia at the primary site of infection. (Demonstrated in mice) |
| 195386 | Eps8 | 22493489 | Eps8 is a key regulator of the LPS-induced Tlr4-Myd88 interaction and directly contributes to phagocytosis in macrophages. |
| 21563 | EPS8 | 22493489 | EPS8 is a key regulator of the LPS-induced TLR4-MYD88 interaction and directly contributes to phagocytosis in macrophages. (Demonstrated in mice) |
| 163574 | Atf4 | 22496230 | Murine cytomegalovirus targets transcription factor ATF4 to exploit the unfolded protein response |
| 8409 | ATF4 | 22496230 | Murine cytomegalovirus targets transcription factor ATF4 to exploit the unfolded protein response (Demonstrated in mice) |
| 190689 | Nfat5 | 20685965 | Nfat5 is a transcription factor that is upregulated by hypertonicity. Activated Nfat5 enhances LPS-mediated NFkB activity by complexing with NFkB and kB elements in downstream genes. |
| 190689 | Nfat5 | 22496647 | Nfat5 expression is strongly induced by Mycobacterium tuberculosis, and plays an essential role in stimulating HIV replication in co-infected macrophages. (Demonstrated in human) |
| 39265 | NFAT5 | 20685965 | NFAT5 is a transcription factor that is upregulated by hypertonicity. Activated NFAT5 enhances LPS-mediated NFkB activity by complexing with NFkB and kB elements in downstream genes. (Demonstrated in murine model) |
| 39265 | NFAT5 | 22496647 | NFAT5 expression is strongly induced by Mycobacterium tuberculosis, and plays an essential role in stimulating HIV replication in co-infected macrophages. |
| 174783 | Ehmt2 | 22412156 | Ehmt2 catalyzes di-methylation of histone H3 at the promoter of interferon (IFN) and IFN-inducible antiviral genes as an epigenetic silencing mechanism in fibroblasts. Ablation of Ehmt2 results in phenotypic conversion to potent IFN-producing cells and renders resistance to RNA viruses. |

| | | | |
|---|---|---|---|
| 79489 | EHMT2 | 22412156 | EHMT2 catalyzes di-methylation of histone H3 at the promoter of interferon (IFN) and IFN-inducible antiviral genes as an epigenetic silencing mechanism in fibroblasts. Ablation of EHMT2 results in phenotypic conversion to potent IFN-producing cells and renders resistance to RNA viruses. (Demonstrated in mice) |
| 90470 | TSC1 | 22412198 | TSC1 inhibits TLR response and endotoxin tolerance through repression of mTORC1 and JNK1/2 signalling pathways. (Demonstrated in mice) |
| 40393 | IRF5 | 18332133 | IRF5 is activated by both TBK1 and MYD88 to form a homodimer that binds to and activates transcription of type I interferon and inflammatory cytokine genes. |
| 40393 | IRF5 | 12600985 | IRF5 can act as both an activator and a repressor of interferon (IFN) gene induction dependent on the IRF-interacting partner, and may be a part of the regulatory network that ensures timely expression of the immediate early inflammatory genes. |
| 40393 | IRF5 | 22412986 | Phosphorylation of IRF5 at ser451 and ser462 is the primary trigger of IRF5 function in nuclear accumulation, transcription and apoptosis. |
| 151763 | Sftpa1 | 20413160 | Sftpa1 is a carbohydrate pattern recognition molecule of innate immunity, that significantly enhances phagocytosis and killing of Aspergillus fumigatus, a pathogenic fungus, by neutrophils and macrophages. |
| 151763 | Sftpa1 | 21821801 | Sftpa1 binds to Mycobacterium avium lipid and it promotes the agglutination of the pathogen. The presence of Sftpa1 inhibits the growth of M. avium in culture. (Demonstrated in human) |
| 151763 | Sftpa1 | 22418431 | Sftpa1 confers protection in lung epithelium against the cytotoxic effects of human beta-defensin 3, DEFB103B. |
| 154810 | 1700021K19Ril | 22423967 | Rubicon is a physiological feedback inhibitor of pattern recognition receptor signalling, preventing unbalanced proinflammatory responses. (Demonstrated in human) |
| 154810 | 1700021K19Ril | 22423966 | Rubicon positively regulates the NADPH oxidase complex to induce phagosomal trafficking of p22phox-gp91phox (which are subunits of NADPH oxidase), and the induction of reactive oxygen species and inflammatory cytokines. (Demonstrated in human) |
| 71731 | KIAA0226 | 22423967 | RUBICON is a physiological feedback inhibitor of pattern recognition receptor signalling, preventing unbalanced proinflammatory responses. |
| 71731 | KIAA0226 | 22423966 | RUBICON positively regulates the NADPH oxidase complex to induce phagosomal trafficking of p22phox-gp91phox (which are subunits of NADPH oxidase), and the induction of reactive oxygen species and inflammatory cytokines. |
| 82385 | ZBP1 | 17618271 | ZBP1 is a cytosolic DNA sensor that upon binding to DNA, associates with IRF3 transcription factor and TBK1 serine/threonine kinase, selectively enhancing DNA mediated induction of type-I-IFN. |

| | | | |
|---|---|---|---|
| 82385 | ZBP1 | 19846511 | ZBP1 is a cytosolic pattern recognition receptor of double-stranded DNA and is essential for IRF3 activation and interferon beta expression triggered by human cytomegalovirus (HCMV), as well as being sufficient to enhance HCMV-stimulated beta interferon transcription and secretion. |
| 82385 | ZBP1 | 20599623 | ZBP1 (DAI) enhanced HIV-1 replication, which is largely impaired by mutations at kappaB sites in HIV-1 long terminal repeat (LTR) or by suppressing activation of NF-kappaB. |
| 82385 | ZBP1 | 22423968 | ZBP1 interacts with RIPK3 to mediate virus-induced necrosis. (Demonstrated in mice) |
| 3902 | RIPK3 | 22123964 | RIPK3 forms a complex with TICAM1 upon Toll-like receptors (TLR) 3 and 4 activation resulting in RIPK3-dependent but TNF-independent necrosis in macrophages. (Demonstrated in mouse) |
| 3902 | RIPK3 | 22423968 | RIPK3 interacts with ZBP1 to mediate virus-induced necrosis. (Demonstrated in mice) |
| 145299 | Tmed7 | 22426228 | Tmed7 inhibits LPS-mediated Tlr4 signalling in late endosomes. (Demonstrated in human) |
| 37819 | 'MED7;TICAM | 19412184 | TMED7;TICAM2 (TICAM2) splice variant TAG negatively regulates the adapter MYD88-independent TLR4 pathway by displacing TICAM1 adapter from TICAM2. |
| 37819 | 'MED7;TICAM | 14517278 | TICAM2 and TICAM1 both function in LPS-TLR4 signaling to regulate the MYD88-independent pathway during the innate immune response to lipopolysaccharide (LPS). |
| 37819 | 'MED7;TICAM | 14519765 | TICAM2 and TICAM1 form an adapter complex that plays a crucial role for LPS-TLR4-mediated activation of IFN-beta. |
| 37819 | 'MED7;TICAM | 17277119 | TMED7;TICAM2 is an adapter protein for both TLR4 and TLR2/6 signaling in synovial fibroblasts, HUVECs, and murine embryonic fibroblasts. |
| 37819 | 'MED7;TICAM | 14556004 | TICAM2 provides specificity for the MYD88-independent component of TLR4 signalling. |
| 37819 | 'MED7;TICAM | 22426228 | TMED7 inhibits LPS-mediated TLR4 signalling in late endosomes. |
| 150439 | Il1rap | 22426547 | Il1rap is recruited to the Il1b::Il1r1 ligand-receptor complex to initiate the Il1b signalling cascade. (Demonstrated in human) |
| 69682 | IL1RAP | 22426547 | IL1RAP is recruited to the IL1B::IL1R1 ligand-receptor complex to initiate the IL1B signalling cascade. |
| 64088 | IL1R1 | 18996842 | IL1R1 is a substrate for presenilin-dependent gamma-secretase cleavage and intramembrane proteolysis may be a control mechanism for IL1R1-mediated signalling. |
| 64088 | IL1R1 | 21115691 | IL1R1 is essential for TLR9-dependent activation of tumour necrosis factor receptor-associated factor 3 (TRAF3) and for production of the anti-inflammatory cytokines IL-10 and type I interferon (IFN). |

| | | | |
|---|---|---|---|
| 64088 | IL1R1 | 22426547 | IL1R1 is the primary receptor for the inflammatory cytokine IL1B. |
| 212144 | Sdc4 | 22427536 | Sdc4 acts in the early inflammatory response to LPS and functions to limit the extent of pulmonary inflammation and lung injury. |
| 77503 | SDC4 | 22427536 | SDC4 acts in the early inflammatory response to LPS and functions to limit the extent of pulmonary inflammation and lung injury. (Demonstrated in mice) |
| 185523 | Tlr8 | 21734241 | Tlr7 and Tlr8 are translocated from the endoplasmic reticulum to the endosome in the presence of antiphospholipid antibodies, as a consequence, plasmacytoid dendritic cells become dramatically sensitized to Tlr7/8 agonists and this may play a role in systemic autoimmunity. |
| 185523 | Tlr8 | 22393042 | Tlr8 binding of HIV ssRNA induces endosomal acidification and chromatin remodeling at the TNF-alpha promoter to promote TNF-alpha release in infected macrophages. (Demonstrated in human) |
| 209311 | Hmgb1 | 21372296 | Hmgb1 is an endogenous Tlr4 ligand in macrophages and its release in wounds initiates Tlr4-dependent responses that contribute to neovascularization. |
| 209311 | Hmgb1 | 21860212 | Hmgb1 plays a key regulatory role in polymorphonuclear neutrophil (PMN) recruitment to inflammatory tissues. Low concentrations of Hmgb1 (50-100 ng/ml) reduce baseline PMN migration as well as formyl-methionyl-leucyl-phenylalanine- and Il8-induced PMN chemotaxis, whereas higher Hmgb1 concentrations (5000 ng/ml) have a chemoattractant effect on PMN through Il8 production . (Demonstrated in human) |
| 209311 | Hmgb1 | 21871094 | Hmgb1 has a pathogenic role in arthritis, where in complex with lipopolysaccharide, Il1a or Il1b, Hmgb1 boosts the production of proinflammatory cytokines and Mmp3 as demonstrated in synovial fibroblasts from rheumatoid arthritis and osteoarthritis patients. (Demonstrated in human) |
| 209311 | Hmgb1 | 22396017 | Nuclear Hmgb1 translocates to the cytoplasm in LPS-stimulated macrophages to potentiate inflammatory responses. (Demonstrated in mice) |
| 130586 | Cd209a | 20130211 | Cd209a has a role in the regulation of inflammation in a model of experimental colitis and is a critical innate factor in response to LPS. |
| 130586 | Cd209a | 22396536 | Cd209a is involved in cell-cell contact signalling between activated apoptotic lymphocytes and dendritic cells (DC) during the maturation of DCs. (Demonstrated in human) |
| 23414 | CD209 | 18998127 | CD209, upon pathogen binding, induces an intracellular signalling pathway with a central role for the serine/threonine kinase RAF1 and modulates TLR-induced activation. |

| | | | |
|---|---|---|---|
| 23414 | CD209 | 22396536 | CD209 is involved in cell-cell contact signalling between activated apoptotic lymphocytes and dendritic cells (DC) during the maturation of DCs. |
| 211114 | Samhd1 | 21720370 | Samhd11 is specifically targeted for degradation by human immunodeficiency virus 1 (HIV-1) viral protein VPX, and mutations in SAMHD11 causes Aicardi-Goutieres syndrome, which mimics symptoms of congenital viral infection. (Demonstrated in human) |
| 211114 | Samhd1 | 21613998 | Samhd1 is expressed in dendritic and myeloid cells and acts as an anti-retroviral protein that inhibits the early stages of the viral life cycle. Silencing of SAMHD1 leads to significant accumulation of human immunodeficiency virus 1 (HIV-1) DNA in infected cells. (Demonstrated in human) |
| 211114 | Samhd1 | 22327569 | Samhd1 inhibits HIV replication by depleting intracellular dNTPs, which are required for viral reverse transcriptase to synthesize viral DNA. (Demonstrated in human) |
| 188640 | Ntn1 | 22231519 | Ntn1 inhibits Ccl2 and Ccl19-mediated chemotaxis in macrophages and promotes atherosclerosis by retaining macrophages in the artery wall. |
| 29663 | NTN1 | 22231519 | NTN1 inhibits CCL2 and CCL19-mediated chemotaxis in macrophages and promotes atherosclerosis by retaining macrophages in the artery wall. (Demonstrated in mice) |
| 134049 | Nampt | 22377803 | Nampt secretion is enhanced by extracellular ATP in LPS-primed monocytes. (Demonstrated in human) |
| 35259 | NAMPT | 22377803 | NAMPT secretion is enhanced by extracellular ATP in LPS-primed monocytes. |
| 80463 | AGER | 22386596 | AGER is a native receptor for complement component C1QA. |
| 166648 | Jak3 | 22359619 | Jak3 inhibition is sufficient to counteract lung injury and protect immunocytes from hypercytokinemia following challenge with avian influenza H5N1 hemagglutinin. |
| 37201 | JAK3 | 14976041 | JAK3 is a primary response gene for IL6 in macrophage differentiation and ectopic overexpression of JAK3 accelerates monocytic differentiation of normal mouse bone marrow cells stimulated with cytokines. |
| 37201 | JAK3 | 22359619 | JAK3 inhibition is sufficient to counteract lung injury and protect immunocytes from hypercytokinemia following challenge with avian influenza H5N1 hemagglutinin. |
| 154756 | Sharpin | 21709223 | Sharpin is an essential adaptor downstream of Ikbkg in the TLR signalling pathway. A mutation in the Sharpin gene impairs Il12 production in TLR-stimulated macrophages. |
| 154756 | Sharpin | 22348129 | Sharpin deficient dendritic cells show reduced inflammatory cytokine production, defective NF-kB signalling and skewing towards a Th2-response. |

| | | | |
|---|---|---|---|
| 40065 | SHARPIN | 21709223 | SHARPIN is an essential adaptor downstream of IKBKG in the TLR signalling pathway. A mutation in the SHARPIN gene impairs IL12 production in TLR-stimulated macrophages. |
| 40065 | SHARPIN | 22348129 | SHARPIN deficient dendritic cells show reduced inflammatory cytokine production, defective NF-kB signalling and skewing towards a Th2-response. (Demonstrated in mice) |
| 198659 | Camkk2 | 22334678 | Camkk2-null macrophages exhibited deficiency to spread, phagocytize bacteria and synthesize cytokines in response to the Toll Like Receptor 4 (TLR4) agonist lipopolysaccharide (LPS). |
| 61470 | CAMKK2 | 22334678 | CAMKK2-null macrophages exhibited deficiency to spread, phagocytize bacteria and synthesize cytokines in response to the Toll Like Receptor 4 (TLR4) agonist lipopolysaccharide (LPS). (Demonstrated in mice) |
| 107120 | WNT3A | 22328140 | WNT3A in human chondrocytes counteracts IL1B induced NF-kB mediated matrix metalloproteinases expression in a negative feedback loop. |
| 130090 | Was | 22253930 | Was interacts with Btk to induce the LPS signalling cascade in macrophages. |
| 63703 | WAS | 22253930 | WAS interacts with BTK to induce the LPS signalling cascade in macrophages. (Demonstrated in mice) |
| 126583 | MIR16-2 | 22292036 | MIR16-2 expression is induced upon LPS stimulation and acts to promote NF-kB-mediated transcription of IL8 by suppressing the translation of SMRT. |
| 127053 | MIR16-1 | 22292036 | MIR16-1 expression is induced upon LPS stimulation and acts to promote NF-kB-mediated transcription of IL8 by suppressing the translation of SMRT. |
| 28012 | PYCARD | 18487086 | PYCARD protein contains CARD and Pyrin domains and is required for assembly of inflammasome. |
| 28012 | PYCARD | 14634131 | PYCARD prevents oligomerization of CASP1 mediated by RIPK2 by out-competing RIPK2 for binding, and thus, preventing CASP1 autoactivation. PYCARD also recruits CASP1 into PYCARD-formed cytosolic specks, separating it from RIPK2. |
| 28012 | PYCARD | 16585594 | PYCARD directs CASP1 away from RIPK2-mediated NF-kappaB activation, toward CASP1-mediated processing of pro-IL1B by interfering with CASP1-RIPK2 interaction. |
| 28012 | PYCARD | 19158675 | PYCARD binds to AIM2 to form a CASP1 and NF-kappaB activating inflammasome. |
| 28012 | PYCARD | 19759850 | PYCARD (ASC) splice variant protein (vASC) lacking the PGR domain regulates IL1B release and aggregates differently from intact PYCARD. |
| 28012 | PYCARD | 21439959 | PYCARD is a component of the inflammasome and is required for inflammation in acute pancreatitis. (Demonstrated in murine model) |
| 28012 | PYCARD | 22295065 | NLRP3/PYCARD inflammasome activation following human respiratory syncytial virus infection is dependent on the activation of TLR2/MYD88/NF-kB and reactive oxygen species/potassium efflux. |

| | | | |
|---|---|---|---|
| 159685 | Frem1 | 22262840 | Frem1 selectively amplifies NF-kB responses, such as cell survival and inflammatory responses, through changes in receptor conformation and adapter protein recruitment. (Demonstrated in human) |
| 50859 | FREM1 | 22262840 | FREM1 selectively amplifies NF-kB responses, such as cell survival and inflammatory responses, through changes in receptor conformation and adapter protein recruitment. |
| 199676 | Usp4 | 22262844 | Usp4 is a potent negative regulator of Tlr/Il1r-signalling through deubiquitination of Traf6 to prevent the activation of inflammatory responses. |
| 34595 | USP4 | 22262844 | USP4 is a potent negative regulator of TLR/IL1Rsignalling through deubiquitination of TRAF6 to prevent activation of inflammatory responses. |
| 38275 | DCD | 19014393 | Activates normal human keratinocytes and display in vitro microbicidal activities against bacteria and viruses |
| 38275 | DCD | 22262861 | DCD encodes the anionic amphiphilic peptide DCD-1L that interacts with negatively charged bacterial phospholipids to form ion channels in the bacterial membrane. |
| 40082 | PRKCD | 19150425 | Following TNF stimulation, PRKCD phosphorylates TRAF2 leading to CHUK (IKK alpha) and IKBKB (IKK beta) recruitment to the TNF receptor |
| 40082 | PRKCD | 22265677 | PRKCD is an essential signalling kinase in C-type lectin receptor-mediated innate immunity and host protection. (Demonstrated in mice) |
| 159696 | Fstl1 | 22265692 | Fstl1 acts as an endogenous Tlr4 agonist. Similar to LPS, Fstl1 induces tolerance to subsequent LPS stimulations. |
| 51777 | FSTL1 | 22265692 | FSTL1 acts as an endogenous TLR4 agonist and has the ability to induce IL6 and IL8 production. Similar to LPS, FSTL1 induces tolerance to subsequent LPS stimulations. |
| 14920 | RORA | 22267218 | The transcription factor RORA is critical for the development of nuocytes and the mounting of innate type 2 immunity against parasitic worms. (Demonstrated in mice) |
| 6083 | DEFA1 | 19024344 | DEFA1 (HNP-1) is a cationic alpha-defensin peptide present in human neutrophils and alpha-defensins have multiple functions in the immune system. |
| 6083 | DEFA1 | 19897717 | Defensins, such as DEFA1, contribute to innate immunity through diverse actions, including microbial killing and high concentrations are present in the lung in response to inflammation. |
| 6083 | DEFA1 | 20214904 | DEFA1, an alpha-defensin, binds to the cell wall precursor lipid II and reduction of lipid II levels in the bacterial membrane significantly reduces bacterial killing, suggesting that the inhibition of cell wall synthesis is a novel antibacterial mechanism of this important class of host defence peptides. |

| | | | |
|---|---|---|---|
| 6083 | DEFA1 | 22270360 | The dimerization of DEFA1 is crucial for the ability of the alpha defensin to kill S. aureus, inhibit anthrax lethal factor and bind HIV-1 protein gp120. |
| 6083 | DEFA1 | 22268819 | DEFA1 activates platelets and induces the formation of amyloid-like proteins, which entrap bacteria and fungi. |
| 129207 | Trib2 | 22271508 | Trib2 expression is induced by Tlr5 stimulation to inhibit NF-kB signalling in epithelial cells. |
| 30297 | TRIB2 | 22271508 | TRIB2 expression is induced by TLR5 stimulation to inhibit NF-kB signalling in epithelial cells. |
| 161189 | Apoa1 | 22271762 | Apoa1 has anti-inflammatory properties in macrophages through the stabilization of ATP-binding cassette transporter A1 (Abca1) and the down-regulation of Tlr4 signalling pathway. (Demonstrated in human) |
| 72644 | APOA1 | 20519121 | APOA1 is a serum apolipoprotein that induces antiatherogenic efflux of macrophage cholesterol and is anti-inflammatory due to its ability to neutralize bacterial lipopolysaccharide (LPS). APOA1 also signals in the macrophage through Toll-like receptor (TLR)2, TLR4, and CD14, utilizing MYD88-dependent and -independent pathways, to activate nuclear factor-kappaB and induce cytokines. |
| 72644 | APOA1 | 22271762 | APOA1 has anti-inflammatory properties in macrophages through the stabilization of ATP-binding cassette transporter A1 (ABCA1) and the down-regulation of TLR4 signalling pathway. |
| 156977 | Jak2 | 20393690 | Jak2 inhibition prevents innate immune responses and rescues animals from sepsis by specifically preventing LPS-induced STAT3 tyrosine phosphorylation without affecting serine phosphorylation in macrophages and by preventing the activation of the canonical p65RelA/p50NF-kappaB1 pathway but not the other NF-kappaB proteins. |
| 156977 | Jak2 | 22218715 | Jak2 functions downstream of Tlr2-stimulation to induce the expression of the monocyte chemoattractant protein 1, Ccl2. |
| 47039 | JAK2 | 15121872 | JAK2 binds to the majority of the known members of the cytokine family of receptors and ligand-receptor binding leads to activation of the associated JAK2 molecules, resulting in rapid autophosphorylation of multiple tyrosines within JAK2. Tyrosine 813 is a site of autophosphorylation in JAK2 and is the SH2-B beta-binding site within JAK2 that is required for SH2-B beta to enhance activation of JAK2. |
| 47039 | JAK2 | 8041779 | JAK2 and JAK1 tyrosine kinases physically associate with the gamma chain and beta chain of the interleukin 2 (IL-2) receptor , respectively, suggesting that regulation of the kinases may be linked to IL-2-induced signal transduction. |

| | | | |
|---|---|---|---|
| 47039 | JAK2 | 20393690 | JAK2 inhibition prevents innate immune responses and rescues animals from sepsis by specifically preventing LPS-induced STAT3 tyrosine phosphorylation without affecting serine phosphorylation in macrophages and by preventing the activation of the canonical p65RelA/p50NF-kappaB1 pathway but not the other NF-kappaB proteins. |
| 47039 | JAK2 | 22218715 | JAK2 functions downstream of TLR2-stimulation to induce the expression of the monocyte chemoattractant protein 1, CCL2. (Demonstrated in mice) |
| 182078 | Hsp90b1 | 22109526 | Hsp90b1 on the surface of polymorphonuclear leukocytes (PMNs) serves as a receptor for Escherichia coli K1 (EC-K1) entry. EC-K1 exploits surface-expressed Hsp90b1 in PMNs to prevent oxidative burst for the onset of neonatal meningitis. |
| 182078 | Hsp90b1 | 22223641 | Hsp90b1 is an essential endoplasmic reticulum chaperone protein for Toll-like receptors and integrins. |
| 54092 | HSP90B1 | 16754684 | HSP90B1 amplifies innate and adaptive immune responses via interaction with TLR2 and TLR4 ligands. |
| 54092 | HSP90B1 | 11584270 | Absence of HSP90B1 results in a defect in the formation of some cell surface receptors including Toll-like receptors (TLRs) which, as a result, are retained intracellularly. |
| 54092 | HSP90B1 | 19965672 | HSP90B1 is an endoplasmic reticulum master chaperone for multiple TLRs and is also essential for expression of multiple hematopoietic system-specific integrins. |
| 54092 | HSP90B1 | 20351288 | HSP90B1, an endoplasmic reticulum chaperone, is required in vesicular stomatitis virus (VSV) infection and in innate immunity via Toll-like receptors (TLRs). TLR-mediated immunity maintains the broad host range of the envelope glycoprotein of VSV (VSV-G) by positively selecting for the ubiquitous expression of HSP90B1. |
| 54092 | HSP90B1 | 20865800 | HSP90B1 chaperones multiple Toll-like receptors (TLRs), but not TLR3, in a manner that is dependent on another endoplasmic reticulum (ER) luminal protein, CNPY3. |
| 54092 | HSP90B1 | 22109526 | HSP90B1 on the surface of polymorphonuclear leukocytes (PMNs) serves as a receptor for Escherichia coli K1 (EC-K1) entry into the cell. EC-K1 exploits surface-expressed HSP90B1 in PMNs to prevent oxidative burst for the onset of neonatal meningitis. (Demonstrated in mice) |
| 54092 | HSP90B1 | 22223641 | HSP90B1 is an essential endoplasmic reticulum chaperone protein for Toll-like receptors (TLR) and integrins. (Demonstrated in mice) |
| 167747 | Trpm2 | 21709234 | Trpm2 deficient mice are extremely susceptible to infection with Listeria monocytogenes, exhibiting an inefficient innate immune response. |

| | | | |
|---|---|---|---|
| 167747 | Trpm2 | 22101731 | Trpm2 inhibits reactive oxygen species (ROS) production in phagocytic cells and prevents endotoxin-induced lung inflammation in mice. |
| 5302 | TRPM2 | 21709234 | Trpm2 deficient mice are extremely susceptible to infection with Listeria monocytogenes, exhibiting an inefficient innate immune response. (Demonstrated in murine model) |
| 5302 | TRPM2 | 22101731 | TRPM2 inhibits reactive oxygen species (ROS) production in phagocytic cells and prevents endotoxin-induced lung inflammation. (Demonstrated in mice) |
| 179176 | Cyld | 21498625 | Cyld is a deubiquitinase that act as a negative regulator of TLR3 induction in response to LPS. (Demonstrated in human) |
| 179176 | Cyld | 21946435 | Cyld plays a key role in Type I IFN receptor signalling during vesicular stomatitis virus (VSV) infection. In the absence of Cyld, IFN-beta is ineffective in the induction of antiviral genes. |
| 179176 | Cyld | 22057290 | The E3 ligase Itch and deubiquitinase Cyld act together to regulate Tak1 and inflammation. |
| 210517 | Itch | 22057290 | The E3 ligase Itch and deubiquitinase Cyld act together to regulate Tak1 and inflammation. |
| 68143 | ITCH | 18246070 | ITCH is an E3 ligase that negatively regulates inflammatory signalling pathways by controlling the function of the ubiquitin-editing enzyme TNFAIP3. |
| 68143 | ITCH | 19881509 | ITCH is a HECT domain-containing E3 ligase that negatively regulates MAVS-mediated antiviral response by catalyzing the K48-linked polyubiquitination and degradation of MAVS. |
| 68143 | ITCH | 22057290 | The E3 ligase ITCH and deubiquitinase CYLD act together to regulate TAK1 and inflammation. |
| 46304 | IL17C | 21993848 | IL17C is an essential autocrine cytokine that regulates innate epithelial immune responses. |
| 176930 | Il17re | 21993849 | Il17re is a receptor specific to Il17c that regulates early innate immunity to intestinal pathogens in colon epithelial cells. |
| 16793 | IL17RE | 21993849 | IL17RE is a receptor specific to IL17C that regulates early innate immunity to intestinal pathogens in colon epithelial cells. (Demonstrated in mice) |
| 45710 | IL9 | 21983833 | IL9 production is largely restricted to innate lymphoid cells during papain-induce lung inflammation and IL9 serves as an important bridging link to induce type 2 helper T cell responses. (Demonstrated in mice) |
| 175625 | Lrrk2 | 21983832 | Lrrk2 acts as a potent negative regulator of the transcription factor NFAT, and plays an important role in modulating inflammatory bowel disease. Lrrk2 deficiency conferred enhanced susceptibility to experimental colitis in mice. |
| 27257 | LRRK2 | 21983832 | LRRK2 acts as a potent negative regulator of the transcription factor NFAT, and plays an important role in modulating inflammatory bowel disease. |

| | | | |
|---|---|---|---|
| 2711 | PIK3C3 | 22170068 | Class III phosphatidylinositol 3-kinases (PI3K) are required for downstream ARF6 regulation of CpG oligodeoxynucleotide uptake and thus have a role in TLR9-mediated immune signalling. |
| 144539 | Arf6 | 22170068 | Arf6 has a pivotal role in Tlr9-mediated immune signaling by regulating the cellular uptake of CpG oligodeoxynucleotides. |
| 42599 | IL13 | 21097505 | IL13 is a pro-M2/Th2 cytokine that induces alternative activation of macrophages. IL13 associates with IL13RA1 to activate the transcription factor STAT6. |
| 42599 | IL13 | 22154382 | IL13 dampens the innate immune response in airway epithelial cells via IRAK3-mediated inhibition of TLR2 signalling. |
| 191235 | Hp | 22156194 | Haptoglobin activates innate immunity to enhance acute transplant rejection. |
| 41488 | HP | 22156194 | Haptoglobin activates innate immunity to enhance acute transplant rejection. (Demonstrated in mouse) |
| 190027 | Unc5cl | 22158417 | Unc5cl is a factor in epithelial inflammation where it activates the pro-inflammatory IRAK signalling cascade in a Myd88-independent manner. |
| 86449 | UNC5CL | 22158417 | UNC5CL is a factor in epithelial inflammation where it activates the pro-inflammatory IRAK signalling cascade in a MYD88-independent manner. |
| 195070 | Plcg2 | 22158869 | Plcg2, 1,4,5-triphosphate and intracellular calcium are required for the LPS-induced innate immune response pathway, where release of intracellular calcium mediates Tlr4 trafficking and subsequent activation of Irf3. |
| 43723 | PLCG2 | 22158869 | PLCG2, 1,4,5-triphosphate and intracellular calcium are required for the LPS-induced innate immune response pathway, where release of intracellular calcium mediates TLR4 trafficking and subsequent activation of IRF3. (Demonstrated in mouse) |
| 135345 | Cnot4 | 22159038 | Cnot4 enhances JAK/STAT pathway-dependent gene expression by positively regulating IFN-gamma- and IL4-induced STAT-mediated gene responses. (Demonstrated in human) |
| 42604 | CNOT4 | 22159038 | CNOT4 enhances JAK/STAT pathway-dependent gene expression by positively regulating IFN-gamma- and IL4-induced STAT-mediated gene responses. |
| 128581 | Unc93b1 | 21097503 | Unc93b1 is a resident endoplasmic reticulum protein that interacts with Tlr11 to regulate the dendritic cell activation in response to T. gondii profilin and parasitic infection. |
| 128581 | Unc93b1 | 21683627 | Unc93b1 controls homeostatic Tlr7 activation by balancing Tlr9 to Tlr7 trafficking from endoplasmic reticulum to endolysosomes. D34A mutation in Unc93b1 causes Tlr7-dependent systemic lethal inflammation. |

| | | | |
|---|---|---|---|
| 128581 | Unc93b1 | 21683627 | Unc93B1 is a multitransmembrane endoplasmic reticulum (ER)-resident protein that controls homeostatic Tlr7 activation by balancing Tlr9 to Tlr7 trafficking. Mice harboring a D34A mutation in Unc93b1 show Tlr7-dependent, systemic lethal inflammation. |
| 128581 | Unc93b1 | 22164301 | Unc93b1 participates in intracellular trafficking and signalling for all nucleotide-sensing Toll-like receptors (TLRs ). (Demonstrated in human) |
| 61027 | UNC93B1 | 18305481 | UNC93B1 is a polytopic membrane protein that delivers the nucleotide sensing receptors TLR7 and TLR9 from the endoplasmic reticulum to endolysosomes. |
| 61027 | UNC93B1 | 19451267 | UNC93B1 biases Toll-like receptor responses to nucleic acid in dendritic cells toward DNA- but against RNA-sensing. |
| 61027 | UNC93B1 | 20865117 | UNC93B1 associates with Toll-Like Receptor (TLR) 3, TLR7 and TLR9, mediating their translocation from the endoplasmic reticulum to the endolysosome, hence allowing proper activation by nucleic acid ligands. UNC93B1 has a critical role on induction of IL-12/IFN-gamma production as well as autonomous control of Toxoplasma replication by macrophages. |
| 61027 | UNC93B1 | 21683627 | UNC93B1 controls homeostatic TLR7 activation by balancing TLR9 to TLR7 trafficking from endoplasmic reticulum to endolysosomes. D34A mutation in UNC93B1 causes TLR7-dependent systemic lethal inflammation. (Demonstrated in murine model) |
| 61027 | UNC93B1 | 21683627 | UNC93B1 is a multitransmembrane endoplasmic reticulum (ER)-resident protein that controls homeostatic TLR7 activation by balancing TLR9 to TLR7 trafficking. Mice harboring a D34A mutation in Unc93b1 show Tlr7-dependent, systemic lethal inflammation. |
| 61027 | UNC93B1 | 22164301 | UNC93B1 participates in intracellular trafficking and signalling for all nucleotide-sensing Toll-like receptors (TLRs). |
| 142836 | Serpinb9 | 22167597 | Serpinb9 expression in tubular epithelial cells (TECs) is induced by triggering of the viral double-stranded RNA sensors Tlr3, Ifih1 and Ddx58. Serpinb9 upregulation increases the threshold for granzyme B-mediated apoptosis in TECs and protects the kidney against cytotoxic insults during viral infection. (Demonstrated in human) |

| | | | |
|---|---|---|---|
| 56650 | SERPINB9 | 22167597 | SERPINB9 expression in human tubular epithelial cells (TECs) is induced by triggering of the viral double-stranded RNA sensors TLR3, IFIH1 and DDX58. SERPINB9 upregulation increases the threshold for granzyme B-mediated apoptosis in TECs and protects the kidney against cytotoxic insults during viral infection. |
| 182955 | Igf1 | 21262348 | Igf11 is a PI3K-activating ligand that increases the secretion of Il6 and Tnf in lipopolysaccharide (LPS)-stimulated mast cells, as well as attenuating the production of Il1b. |
| 182955 | Igf1 | 21756999 | Igf1 induces Hif1a-Tlr9 cross talk that regulates inflammatory responses in glioma cells and this regulation functions in both positive and negative feedback loops. (Demonstrated in human) |
| 182955 | Igf1 | 22039371 | Igf1 suppresses the expression of Tlr4 to exert an anti-inflammatory effect of exercising. |
| 53609 | IGF1 | 21262348 | IGF1 is a PI3K-activating ligand that increases the secretion of IL6 and TNF in lipopolysaccharide (LPS)-stimulated mast cells, as well as attenuating the production of IL1B. (Demonstrated in murine model) |
| 53609 | IGF1 | 21756999 | IGF1 induces HIF1A-TLR9 cross talk that regulates inflammatory responses in glioma cells and this regulation functions in both positive and negative feedback loops. |
| 53609 | IGF1 | 22039371 | IGF1 suppresses the expression of TLR4 to exert an anti-inflammatory effect of exercising. (Demonstrated in mice) |
| 155311 | Hsf1 | 22042134 | Hsf1 is necessary to initiate host defence against Mycoplasma pneumoniae infection in the lungs through Tlr2 signalling activation. |
| 40498 | HSF1 | 20018623 | HSF1 activated by heat shock induces the expression of ATF3, a negative regulator of IL-6, and ATF3 is necessary for heat-mediated suppression of IL-6, indicating a fever-mediated feedback loop consisting of HSF1 and ATF3. |
| 40498 | HSF1 | 22042134 | HSF1 is necessary to initiate host defence against Mycoplasma pneumoniae infection in the lungs through TLR2 signalling activation. (Demonstrated in mice) |
| 170363 | Pros1 | 22043818 | Pros1 works with Gas6 to synergistically suppress the basal and TLR-triggered production of inflammatory cytokines in macrophages. |
| 46049 | PROS1 | 22043818 | PROS1 works with GAS6 to synergistically suppress the basal and TLR-triggered production of inflammatory cytokines in macrophages. (Demonstrated in mice) |
| 135497 | Gas6 | 22043818 | Gas6 works with Pros1 to synergistically suppress the basal and TLR-triggered production of inflammatory cytokines in macrophages. |

| | | | |
|---|---|---|---|
| 53634 | GAS6 | 11948660 | GAS6 is a ligand for all members of the TAM receptor tyrosine kinase family, each of which has different affinities to GAS6. |
| 53634 | GAS6 | 16723520 | GAS6 signals through the AXL receptor and the PI3-kinase/Akt1 survival pathway, protecting oligodendrocytes from growth factor withdrawal and TNF-alpha-mediated cell death. |
| 53634 | GAS6 | 22043818 | GAS6 works with PROS1 to synergistically suppress the basal and TLR-triggered production of inflammatory cytokines in macrophages. (Demonstrated in mice) |
| 214212 | Cd300lf | 22043923 | Cd300lf inhibits Myd88 and/or TRIF-mediated TLR signalling pathway through the dual activation of Ptpn6 and Ptpn11. (Demonstrated in human) |
| 67452 | CD300LF | 22043923 | CD300LF inhibits MYD88 and/or TRIF-mediated TLR signalling pathway through the dual activation of PTPN6 and PTPN11. |
| 214150 | Cd300a | 22043923 | Cd300a inhibits Myd88 and/or TRIF-mediated TLR signalling pathway through activation of Ptpn6. (Demonstrated in human) |
| 67271 | CD300A | 22043923 | CD300A inhibits MYD88 and/or TRIF-mediated TLR signalling pathway through activation of PTPN6. |
| 128168 | Rictor | 22045807 | Rictor reduces Tlr4-mediated inflammation by regulating the cellular localization of Foxo1. |
| 17688 | RICTOR | 22045807 | RICTOR reduces TLR4-mediated inflammation by regulating the cellular localization of FOXO1. (Demonstrated in mice) |
| 182659 | Ccl17 | 22057112 | Ccl17 is required for the induction of intestinal inflammation in mice. Ccl17 has an autocrine effect on dendritic cells that promotes production of inflammatory cytokines and activation of Th1 and Th17 cells and reduces expansion of Treg cells. |
| 33236 | CCL17 | 18191727 | IL4 (Interleukin-4) induces CCL17 expression via two STAT6 motifs in the proximal promoter and a distal tandem STAT6 element |
| 33236 | CCL17 | 22057112 | CCL17 is required for the induction of intestinal inflammation in mice. CCL17 has an autocrine effect on dendritic cells that promotes production of inflammatory cytokines and activation of Th1 and Th17 cells and reduces expansion of Treg cells. (Demonstrated in mice) |
| 159756 | Ifit2 | 22065572 | Ifit2 expression imparts anti-viral immunity to restrict West Nile virus infection and control viral pathogenesis. |
| 81895 | IFIT2 | 19108715 | IFIT2 belongs to type 1 interferon response genes and is highly induced after stimulation with LPS and selectively affects LPS induced protein expression by regulation at different post-transcriptional levels. |
| 81895 | IFIT2 | 22065572 | IFIT2 expression imparts anti-viral immunity to restrict West Nile virus infection and control viral pathogenesis. (Demonstrated in mice) |

| | | | |
|---|---|---|---|
| 170894 | Yy1 | 22065573 | Yy1 negatively regulates TLR3-induced expression of IFN-beta and acts downstream of TLR3 to limit the level and duration of the anti-viral response. (Demonstrated in human) |
| 20038 | YY1 | 22065573 | YY1 negatively regulates TLR3-induced expression of IFN-beta and acts downstream of TLR3 to limit the level and duration of anti-viral response. |
| 223693 | mmu-mir-10a | 22068236 | mmu-mir-10a expression is negatively regulated by intestinal microbiota, which contributes to the maintenance of intestinal homeostasis by targeting Il12b. |
| 127395 | MIR10A | 22068236 | MIR10A expression is negatively regulated by intestinal microbiota, which contributes to the maintenance of intestinal homeostasis by targeting IL12B. (Demonstrated in mice) |
| 35412 | MRGPRX2 | 22069323 | MRGPRX2 is a G protein-coupled receptor (GPCR) for the host defence peptide LL-37 in mast cells. |
| 48350 | IL33 | 20427273 | IL33, a member of the IL-1-related cytokines, is the first factor shown to modulate tryptase expression in mast cells (MCs). Synovial fibroblasts promote the expression and granule accumulation of tryptase via IL33 and its receptor ST-2 (IL1RL1). |
| 48350 | IL33 | 20473304 | IL33 attenuates sepsis by enhancing neutrophil influx to the site of infection by preventing the down-regulation of CXCR2 and reverses the TLR4-induced reduction of CXCR2 expression in neutrophils via the inhibition of expression of G protein-coupled receptor kinase-2 (GRK2). |
| 48350 | IL33 | 20937871 | IL33 is a crucial amplifier of mucosal and systemic innate, rather than acquired, immune responses, where it is essential for manifestation of T cell-independent protease allergen-induced airway inflammation as well as OVA-induced allergic topical airway inflammation, without affecting acquisition of antigen-specific memory T cells. |
| 48350 | IL33 | 21623379 | IL33 is produced in alveolar macrophages that have been infected with Influenza A virus. The IL33-IL13 signalling axis is required for airway hyper-reactivity in asthma. (Demonstrated in murine model) |
| 48350 | IL33 | 22119406 | Innate lymphoid cells responding to IL33 mediate airway hyperreactivity independently of adaptive immunity. (Demonstrated in mice) |
| 197102 | Rgs2 | 22120521 | Rgs2 negatively regulates Nox1 expression and consequently inhibits reactive oxygen species production in TLR-mediate innate immune responses. |
| 105451 | RGS2 | 22120521 | RGS2 negatively regulates NOX1 expression and consequently inhibits reactive oxygen species production in TLR-mediated innate immune responses. (Demonstrated in mice) |

| | | | |
|---|---|---|---|
| 170699 | Nox1 | 22120521 | Nox1 is essential for reactive oxygen species production in the TLR-mediated innate immune response. Nox1 expression is enhanced by Tlr2 signalling through the Jak1/3-Stat3 pathway and is negatively regulated by Rgs2. |
| 79287 | NOX1 | 20110267 | NOX1 activity is regulated through MAP kinase (MAPK), protein kinase C (PKC), and protein kinase A (PKA)-dependent phosphorylation on Ser-282 and Ser-172 of NOXA1. |
| 79287 | NOX1 | 18511861 | NOX1 is highly expressed in the colon epithelium and can be induced by LPS or IFN-gamma. |
| 79287 | NOX1 | 22120521 | NOX1 is essential for reactive oxygen species production in the TLR-mediated innate immune response. NOX1 expression is enhanced by TLR2 signalling through the JAK1/3-STAT3 pathway and is negatively regulated by RGS2. (Demonstrated in mice) |
| 151772 | Ppp3r1 | 22116828 | Ppp3r1 plays an important role in activation of peritoneal macrophages. Ppp3r1 induces Tnfsf10 (TRAIL) gene expression in peritoneal macrophages in an Itgam-dependent manner. |
| 281607 | PPP3R1 | 22116828 | PPP3R1 plays an important role in activation of peritoneal macrophages. PPP3R1 induces TNFSF10 (TRAIL) gene expression in peritoneal macrophages in an ITGAM-dependent manner. (Demonstrated in mice) |
| 36771 | PCBP2 | 19881509 | PCBP2 is a negative regulator in MAVS-mediated antiviral signalling that recruits the HECT domain-containing E3 ligase ITCH (AIP4) to polyubiquitinate and degrade MAVS. |
| 36771 | PCBP2 | 22105485 | PCBP2 synergizes with PCBP1 in MAVS inhibition but PCBP2 shows low basal expression with rapid induction after infection while PCBP1 is stably and abundantly expressed. |
| 164861 | Pcbp1 | 22105485 | Pcbp1 is critical in regulating Mavs degradation for both fine-tuning antiviral immunity and preventing inflammation. (Demonstrated in human) |
| 55131 | PCBP1 | 22105485 | PCBP1 is critical in regulating MAVS degradation for both fine-tuning antiviral immunity and preventing inflammation. |
| 191036 | Trp53 | 22105999 | Tpr53 serves as a host antiviral factor and enhances both the innate and adaptive immune responses to influenza A virus. |
| 160833 | Fancc | 22106009 | Fancc is involved in lipopolysaccharide (LPS)-induced peritoneal macrophage inflammatory response. Fancc null mice had impaired monocyte/macrophage trafficking and cytoskeletal rearrangements following LPS treatment. |
| 77041 | FANCC | 22106009 | FANCC is involved in lipopolysaccharide (LPS)-induced peritoneal macrophage inflammatory response. Fancc null mice had impaired monocyte/macrophage trafficking and cytoskeletal rearrangements following LPS treatment. (Demonstrated in mice) |

| | | | |
|---|---|---|---|
| 7897 | APOBEC3B | 22108670 | Deletion of the APOBEC3B gene strongly impacts susceptibility to Plasmodium falciparum malaria. |
| 5986 | DEFB1 | 21248850 | DEFB1 is ubiquitiously expressed by all human epithelial cells, and upon reduction of the disulfide-bridges DEFB1 becomes a potent antimicrobial peptide against opportunistic pathogenic fungi and anaerobic Gram+ bacteria. |
| 5986 | DEFB1 | 21551252 | DEFB1 is upregulated in plasmacytoid dendritic cells and monocytes during viral challenge. Defb1-deficient mice infected with mouse-adapted HK18 (influenza) lost weight earlier and died sooner than WT mice, suggesting that Defb1 plays a role in early innate immune responses against influenza in vivo. However, lung virus titers were equal between the two mouse strains, indicating that the mechanism is not related to viral replication. |
| 5986 | DEFB1 | 22102811 | DEFB1 is a component of platelets that displays classic antimicrobial activity and signals polymorphonuclear leukocytes to extrude DNA lattices that capture and kill bacteria. |
| 168189 | Pglyrp3 | 21439073 | Pglyrp3 is a member of the Peptidoglycan Recognition Proteins (PGRP) family and recognizes peptidoglycan, a structural component of bacterial cell walls, as a part of innate immune response against infections. |
| 168189 | Pglyrp3 | 21602801 | Pglyrp3 binds to Gram-positive bacterial wall and activates a protein-sensing two-component system to induce bacterial death. PLGYRP1-mediated activation results in membrane depolarization and cessation of peptidoglycan, protein, and RNA/DNA synthesis, as well as the production of hydroxyl radicals. (Demonstrated in human) |
| 168189 | Pglyrp3 | 22099350 | Pglyrp3, when stimulated with peptidoglycan, has an anti-inflammatory effect on intestinal epithelial cells. PGLYRP3 knocking down enhanced the expression of PGN-induced inflammatory cytokines. (Demonstrated in human) |
| 102636 | PGLYRP3 | 20418257 | PGLYRP3 is a secreted innate immunity protein that is expressed on body surfaces, mucous membranes, and in secretions (saliva, sweat) and is conserved from insects to mammals, it recognizes bacterial peptidoglycan, and functions in antibacterial immunity and inflammation. |
| 102636 | PGLYRP3 | 21439073 | PGLYRP3 is a member of the Peptidoglycan Recognition Proteins (PGRP) family and recognizes peptidoglycan, a structural component of bacterial cell walls, as a part of innate immune response against infections. |

| | | | |
|---|---|---|---|
| 102636 | PGLYRP3 | 21602801 | PGLYRP3 binds to Gram-positive bacterial wall and activates a protein-sensing two-component system to induce bacterial death. PLGYRP3-mediated activation results in membrane depolarization and cessation of peptidoglycan, protein, and RNA/DNA synthesis, as well as the production of hydroxyl radicals. |
| 102636 | PGLYRP3 | 22099350 | PGLYRP3, when stimulated with peptidoglycan, has an anti-inflammatory effect on intestinal epithelial cells. PGLYRP3 knocking down enhanced the expression of PGN-induced inflammatory cytokines. |
| 162905 | Cdkn2a | 22095712 | The tumour suppressor Cdkn2a plays a role in innate immunity as a regulator of inflammatory cell signalling. Cdkn2a null mice were unable to trigger a proper inflammatory response in experimental peritonitis or in induced edema and were resistant to LPS-induced endotoxic shock. |
| 54547 | CDKN2A | 22095712 | The tumour suppressor CDKN2A plays a role in innate immunity as a regulator of inflammatory cell signalling. Cdkn2a null mice were unable to trigger a proper inflammatory response in experimental peritonitis or in induced edema and were resistant to LPS-induced endotoxic shock. (Demonstrated in mice) |
| 187478 | Dcn | 22087031 | Dcn (Decorin) is an extracellular matrix proteoglycan that stimulates proinflammatory Pdcd4 and decreases the abundance of mmu-mir-21, boosting inflammatory activity in sepsis and suppressing tumour growth. |
| 50872 | DCN | 22087031 | DCN (Decorin) is an extracellular matrix proteoglycan that stimulates proinflammatory PDCD4 and decreases the abundance of MIR21, boosting inflammatory activity in sepsis and suppressing tumour growth. |
| 126493 | MIR125B2 | 22071331 | MIR125B2 may enhance type I IFN expression by suppressing EIF4EBP1 protein expression in airway epithelial cells, which potentially contributes to mucosal eosinophilia in eosinophilic chronic rhinosinusitis with nasal polyps (CRSwNP). |
| 127195 | MIR125B1 | 22071331 | MIR125B1 may enhance type I IFN expression by suppressing EIF4EBP1 protein expression in airway epithelial cells, which potentially contributes to mucosal eosinophilia in eosinophilic chronic rhinosinusitis with nasal polyps (CRSwNP). |
| 165924 | Bst2 | 22072710 | Bst2 is a broad spectrum effector of the innate immune response to viral infection that is antagonized by the human immunodeficiency virus (HIV) Vpu protein to evade innate immune system detection. (Demonstrated in human) |
| 171234 | Dlk1 | 22072963 | Dlk1 is a Notch ligand that plays a critical role in the development of anti-viral immunity. Dlk1 expression in macrophages specifically regulates IFN-gamma levels from CD4(+) and CD8(+)T cells in response to influenza A H1N1 virus infection. |

| | | | |
|---|---|---|---|
| 20347 | DLK1 | 22072963 | DLK1 is a Notch ligand that plays a critical role in the development of anti-viral immunity. DLK1 expression in macrophages specifically regulates IFN-gamma levels from CD4(+) and CD8(+)T cells in response to influenza A H1N1 virus infection. (Demonstrated in mice) |
| 174207 | Tnip1 | 21606507 | Tnip1 interacts with polyubiquitin to limit the activation of Tlr-Myd88 signalling pathway and prevents autoimmunity. |
| 174207 | Tnip1 | 22011580 | Tnip1 is an essential anti-inflammatory component of TLR-signalling pathways that controls Cebpb activity. Tnip1 null mice exhibit progressive, lupus-like inflammatory disease. |
| 54098 | TNIP1 | 21606507 | TNIP1 interacts with polyubiquitin to limit the activation of TLR-MYD88 signalling pathway and prevents autoimmunity. (Demonstrated in murine model) |
| 54098 | TNIP1 | 22011580 | TNIP1 is an essential anti-inflammatory component of TLR-signalling pathways that controls CEBPB activity. Tnip1 null mice exhibit progressive, lupus-like inflammatory disease. (Demonstrated in mice) |
| 159514 | Rac2 | 22018470 | Rac2 is modified by Escherichia coli protein CNF1 , which then interacts with the innate immune adaptors Ripk1 and Ripk2 to trigger an anti-bacterial immune response. This response was protective and increased the ability of the host to restrict pathogen growth, thus defining a mechanism of effector-triggered immunity that contributes to how metazoans defend against microbes with pathogenic potential. (Demonstrated in human) |
| 6499 | RAC2 | 22018470 | RAC2 is modified by Escherichia coli protein CNF1, which then interacts with the innate immune adaptors RIPK1 and RIPK2 to trigger an anti-bacterial immune response. This response was protective and increased the ability of the host to restrict pathogen growth, thus defining a mechanism of effector-triggered immunity that contributes to how metazoans defend against microbes with pathogenic potential. |
| 162642 | Pklr | 22022264 | Pklr is recruited by hepatitis C virus early in infection as a sensor to trigger the induction of Irf3-dependent genes. (Demonstrated in human) |
| 103234 | PKLR | 22022264 | PKLR is recruited by hepatitis C virus early in infection as a sensor to trigger the induction of IRF3-dependent genes. |
| 147701 | Senp2 | 22028379 | Senp2 acts as a negative regulator of virus-triggered IFN-beta induction by deSUMOylating Irf3 and conditioning it for ubiquitination and degradation. |

| | | | |
|---|---|---|---|
| 68570 | SENP2 | 22028379 | SENP2 acts as a negative regulator of virus-triggered IFN-beta induction by deSUMOylating IRF3 and conditioning it for ubiquitination and degradation. (Demonstrated in mice) |
| 42122 | STAT6 | 17971840 | Signal transducer and activator of transcription 6 (STAT6) is a transcription factor that mediates IL-13 cytokine signaling. |
| 42122 | STAT6 | 16433852 | STAT6 plays a role in the interleukin-4 (IL-4)-mediated modulation of TLR4 expression and the responsiveness of cells to lipopolysaccharide (LPS). |
| 42122 | STAT6 | 22000020 | STAT6 is phosphorylated upon viral infection and translocates to the nucleus to induce genes responsible for immune cell homing. (Demonstrated in mice) |
| 212910 | Fadd | 21183682 | Fadd interacts with Trim21 to negatively regulate the late Ifna pathway in response to viral infections. Fadd enhances Trim21 ubiquitin ligase activity to repress Ifna activation in SeV infected cells. In addition, Fadd with Trim21 can ubiquitinate Irf7 and change its phosphorylation state, consequently interfering with the activity of Traf6. |
| 212910 | Fadd | 21804564 | Fadd preserves epithelial barrier integrity and antibacterial defence, maintains homeostasis and prevents chronic intestinal inflammation. |
| 212910 | Fadd | 21979465 | Upon cleavage, Fadd oligomerizes and activates the caspase cascade to induce killing of tumour cells and intracellular pathogens by innate effector natural killer cells. (Demonstrated in human) |
| 212910 | Fadd | 22000287 | Fadd deficiency induces severe inflammatory skin lesions in mice, revealing a protective role for Fadd in epidermal keratinocytes. |
| 62144 | FADD | 17785432 | FADD is an adaptor protein involved in death receptor-mediated apoptosis and a physiological negative regulator of IRAK1/MyD88-dependent responses in innate immune signalling. |
| 62144 | FADD | 15711932 | FADD is part of the viral product dsRNA-triggered death inducing signalling complexes (dsRNA-DISCs) containing TRADD and CASP8. |
| 62144 | FADD | 21183682 | FADD interacts with TRIM21 to negatively regulate the late IFNA pathway in response to viral infections. FADD enhances TRIM21 ubiquitin ligase activity to repress IFNA activation in SeV infected cells. In addition, FADD with TRIM21 can ubiquitinate IRF7 and change its phosphorylation state, consequently interfering with the activity of TRAF6. |
| 62144 | FADD | 21804564 | FADD preserves epithelial barrier integrity and antibacterial defence, maintains homeostasis and prevents chronic intestinal inflammation. (Demonstrated in mouse) |

| | | | |
|---|---|---|---|
| 62144 | FADD | 21979465 | Upon cleavage, FADD oligomerizes and activates the caspase cascade to induce killing of tumour cells and intracellular pathogens by innate effector natural killer cells. |
| 62144 | FADD | 22000287 | FADD deficiency induces severe inflammatory skin lesions in mice, revealing a protective role for FADD in epidermal keratinocytes. (Demonstrated in mice) |
| 197265 | Pla2g4a | 22003202 | Pla2g4a and its metabolite lipid mediators induce autophagy in macrophages and monocytes. This autophagy is Atg5 dependent and independent of changes in mTOR or autophagic flux. |
| 105429 | PLA2G4A | 22003202 | PLA2G4A and its metabolite lipid mediators induce autophagy in macrophages and monocytes. This autophagy is ATG5 dependent and independent of changes in mTOR or autophagic flux. (Demonstrated in mice) |
| 198410 | P2rx7 | 21988719 | P2rx7 activation in lipopolysaccharide (LPS)-primed myeloid cells results in secretion of pro-inflammatory cytokines Il1b and Il18. In addition, P2rx7 functions in the recognition and phagocytosis of non-opsonized bacteria and apoptotic cells. (Demonstrated in human) |
| 154827 | Plaur | 21998707 | Plaur is required for optimal TLR2-induced neutrophil activation. |
| 55442 | PLAUR | 21998707 | PLAUR is required for optimal TLR2-induced neutrophil activation. (Demonstrated in mice) |
| 170467 | Gzmm | 21979465 | Gzmm cleaves Fadd to potentiate the killing efficacy of innate effector natural killer cells against tumor cells and intracellular pathogens. (Demonstrated in human) |
| 12497 | GZMM | 20406824 | GZMM (granzyme M) cleaves BIRC5 and this triggers degradation of the BIRC5-XIAP complex to free caspase activity, leading to cytolysis of target cells. |
| 12497 | GZMM | 21979465 | GZMM cleaves FADD to potentiate the killing efficacy of innate effector natural killer cells against tumor cells and intracellular pathogens. |
| 40608 | SELK | 21849499 | SELK is a novel target for calpain protease and its cleavage is regulated by the calpain/calpastatin system during Toll-like receptor (TLR)-induced activation of macrophages. (Demonstrated in murine model) |
| 168233 | Aire | 21628060 | Aire transcriptionally upregulates Tlr1/3/8 via direct interaction with the TLR promoters. In addition, Aire also mediates the induction of Il1a, Tnf, Nos2 and IFN-alpha expression upon Tlr1 and Tlr3 stimulation. |
| 168233 | Aire | 21962774 | Aire participates in Dectin-1 signalling, an anticandidal innate immune signalling pathway that specifically recognizes fungal beta-glucan. Aire formes a transient complex with the known Dectin-1 pathway components phosphorylated Sykb and Card9 after receptor ligation and localizes with Clec7a (Dectin-1) at the cell membrane (Demonstrated in human) |

| | | | |
|---|---|---|---|
| 5152 | AIRE | 21628060 | AIRE transcriptionally upregulates TLR1/3/8 via direct interaction with the TLR promoters. In addition, AIRE also mediates the induction of IL1A, TNF, NOS2 and IFN-alpha expression upon TLR1 and TLR3 stimulation. (Demonstrated in murine model) |
| 5152 | AIRE | 21962774 | AIRE participates in Dectin-1 signalling, an anticandidal innate immune signalling pathway that specifically recognizes fungal beta-glucan. AIRE formes a transient complex with the known Dectin-1 pathway components phosphorylated SYK and CARD9 after receptor ligation and localizes with CLEC7A (Dectin-1) at the cell membrane. |
| 213761 | Apoh | 21965665 | Apoh is a plasma protein that specifically interacts with lipopolysaccharide (LPS) and Tlr4 to induce Tnf-alpha production in macrophages. (Demonstrated in human) |
| 65172 | APOH | 21965665 | APOH is a plasma protein that specifically interacts with lipopolysaccharide (LPS) and TLR4 to induce TNF-alpha production in macrophages. |
| 151805 | Ppargc1b | 21966468 | Ppargc1b is activated in Staphylococcus aureus-mediated sepsis via the Tlr2-signalling pathway. |
| 53101 | PPARGC1B | 21966468 | PPARGC1B is activated in Staphylococcus aureus-mediated sepsis via the TLR2-signalling pathway. (Demonstrated in mice) |
| 163551 | Ppargc1a | 21966468 | Ppargc1a is activated in Staphylococcus aureus-mediated sepsis via the Tlr2-signalling pathway. |
| 190728 | Mapkapk2 | 21969554 | Mapkapk2 phosphorylation is suppressed by lipomannan derived from virulent Mycobacterium tuberculosis (M.tb), resulting in the potent suppression of Tnf biosynthesis and allowing M.tb to subvert host immunity and potentially to increase its virulence. (Demonstrated in human) |
| 106265 | MAPKAPK2 | 21969554 | MAPKAPK2 phosphorylation is suppressed by lipomannan derived from virulent Mycobacterium tuberculosis (M.tb), resulting in the potent suppression of TNF biosynthesis and allowing M.tb to subvert host immunity and potentially to increase its virulence. |
| 137408 | Zfpm2 | 21971825 | Zfpm2 inhibits the transcription of hepcidin antimicrobial peptide by suppressing the GATA4 and GATA6 transcription factors. (Demonstrated in human) |
| 32130 | ZFPM2 | 21971825 | ZFPM2 inhibits the transcription of hepcidin antimicrobial peptide by suppressing the GATA4 and GATA6 transcription factors. |
| 197075 | Zfpm1 | 21971825 | Zfpm1 inhibits the transcription of hepcidin antimicrobial peptide by suppressing the GATA4 and GATA6 transcription factors. (Demonstrated in human) |
| 46137 | ZFPM1 | 21971825 | ZFPM1 inhibits the transcription of hepcidin antimicrobial peptide by suppressing the GATA4 and GATA6 transcription factors. |

| | | | |
|---|---|---|---|
| 128584 | Gata6 | 21971825 | Gata6 activates the transcription of hepcidin antimicrobial peptide in hepatocytes. Friend of GATA (FOG)-proteins ZFPM1 and ZFPM2 moderate this transcription by suppressing the GATA transactivation of hepcidin promoter. (Demonstrated in human) |
| 1469 | GATA6 | 21971825 | GATA6 activates the transcription of hepcidin antimicrobial peptide in hepatocytes Friend of GATA (FOG)-proteins ZFPM1 and ZFPM2 moderate this transcription by suppressing the GATA transactivation of hepcidin promoter. |
| 172452 | Gata4 | 21971825 | Gata4 activates the transcription of hepcidin antimicrobial peptide in hepatocytes. Friend of GATA (FOG)-proteins ZFPM1 and ZFPM2 moderate this transcription by suppressing the GATA transactivation of hepcidin promoter. (Demonstrated in human) |
| 7434 | GATA4 | 21971825 | GATA4 activates the transcription of hepcidin antimicrobial peptide in hepatocytes. Friend of GATA (FOG) proteins, ZFPM1 and ZFPM2, moderate this transcription by suppressing the GATA transactivation of hepcidin promoter. |
| 139044 | Tyk2 | 21787891 | Tyk2 functions at the molecular interface between innate immunity and cellular metabolism and is involved in the regulation of lipid and carbohydrate metabolism in macrophages stimulated with poly(I:C). |
| 139044 | Tyk2 | 21757742 | Tyk2 is necessary for Socs1-mediated suppression of Type I IFN signalling. |
| 27182 | TYK2 | 12391177 | TYK2 is directly involved in IFN-alpha signalling for the induction and translocation of Daxx nuclear protein, which may result in B lymphocyte growth arrest and/or apoptosis. |
| 27182 | TYK2 | 20338026 | TYK2 modulates the relationship between immunity and metabolism where it is essential for the full LPS response, its function mainly being required for baseline expression and not LPS-induced upregulation of IFN-inducible genes, as well as its critical role in the downregulation of metabolic genes upon immune challenge, in particular genes involved in lipid metabolism. |
| 27182 | TYK2 | 21787891 | TYK2 functions at the molecular interface between innate immunity and cellular metabolism and is involved in the regulation of lipid and carbohydrate metabolism in macrophages stimulated with poly(I:C). (Demonstrated in mouse) |
| 27182 | TYK2 | 21757742 | TYK2 is necessary for SOCS1-mediated suppression of Type I IFN signalling. (Demonstrated in mice) |
| 142259 | Nr3c1 | 21750107 | The Glucocorticoid receptor, Nr3c1 exerts anti-inflammatory action in part by antagonizing pro-inflammatory transcription factors such as Rela of the NFkB complex. (Demonstrated in human) |

| | | | |
|---|---|---|---|
| 142259 | Nr3c1 | 21940629 | Nr3c1, the glucocorticoid receptor, is necessary for glucocorticoid-mediated induction of Nlrp3 and the subsequent formation of inflammasomes, demonstrating a novel role for glucocorticoids in sensitizing the initial inflammatory response by the innate immune system. |
| 51637 | NR3C1 | 21750107 | The Glucocorticoid receptor, NR3C1, exerts anti-inflammatory action in part by antagonizing pro-inflammatory transcription factors such as RELA of the NFkB complex. |
| 51637 | NR3C1 | 21940629 | NR3C1, the glucocorticoid receptor, is necessary for glucocorticoid-mediated induction of NLRP3 and the subsequent formation of inflammasomes, demonstrating a novel role for glucocorticoids in sensitizing the initial inflammatory response by the innate immune system. (Demonstrated in mice) |
| 153401 | Stub1 | 21911421 | Stub1 facilitates the formation of a TLR signalling complex by recruiting, ubiquitinating, and activating Src and Prkcz. Knockdown of Stub1 inhibits Tlr4- and Tlr9-signalling pathways. |
| 8030 | STUB1 | 21911421 | STUB1 facilitates the formation of a TLR signalling complex by recruiting, ubiquitinating, and activating Src and PRKCZ. Knockdown of Stub1 in mice inhibits Tlr4- and Tlr9-signalling pathways. (Demonstrated in mice) |
| 204786 | Serpine1 | 21768189 | Serpine1 is a critical mediator that controls the development of the early lung inflammation induced by Pseudomonas aeruginosa infections. |
| 204786 | Serpine1 | 21945446 | Serpine1 plays a critical role in early host defense response against Haemophilus influenzae infection. Serpine1 knockout mice show reduced bacterial clearance and prolonged pneumonia. |
| 32959 | SERPINE1 | 21768189 | SERPINE1 is a critical mediator of the pulmonary host response that controls the development of the early lung inflammation induced by Pseudomonas aeruginosa infections. (Demonstrated in mouse) |
| 32959 | SERPINE1 | 21945446 | SERPINE1 plays a critical role in early host defense response against Haemophilus influenzae infection. Serpine1 knockout mice show reduced bacterial clearance and prolonged pneumonia. (Demonstrated in mice) |
| 173742 | Tank | 21949249 | Tank plays a key role in the cross-talk between the IKK-related and the canonical IKK kinases, a mechanism required to limit the strength of TLR-signalling and prevent autoimmunity. |
| 73552 | TANK | 19668221 | TANK is involved in interferon responses and is a negative regulator of pro-inflammatory cytokine production induced by TLR signalling. |

| | | | |
|---|---|---|---|
| 73552 | TANK | 21949249 | TANK plays a key role in the cross-talk between the IKK-related and the canonical IKK kinases, a mechanism required to limit the strength of TLR-signalling and prevent autoimmunity. (Demonstrated in mice) |
| 26953 | NAIP | 18280719 | NAIP is important for CASP1 activation and IL-1 processing. |
| 26953 | NAIP | 21918512 | NAIP specifically recognizes the Chromobacterium violaceum type III secretion system protein, Cprl, and activates the NLRC4 inflammasome in macrophages. |
| 262522 | Naip2 | 21918512 | Naip2 functions as a specific inflammasome receptor for the type III secretion system rod component of Salmonella and Burkholderia bacterial species. |
| 189871 | Trem2 | 21956652 | Trem2 inhibits the induction of inflammatory cytokines and type I IFN production in TLR-stimulated dendritic cells. |
| 86566 | TREM2 | 21956652 | TREM2 inhibits the induction of inflammatory cytokines and type I IFN production in TLR-stimulated dendritic cells. (Demonstrated in mice) |
| 402349 | APOBEC3G | 21734563 | APOBEC3G is an innate intracellular HIV restriction factor that is upregulated by type I interferons. |
| 402349 | APOBEC3G | 21874023 | APOBEC3G, an intrinsic antiviral factor, promotes the recognition of human immunodeficiency virus (HIV) through the activation of the DNA-damage response pathway and the expression of natural killer cell-activating ligands on HIV-infected cells. |
| 156279 | Ddx41 | 21892174 | Ddx41 is a helicase responsible for sensing intracellular DNA in myeloid dendritic cells. Ddx41 expression knockdown blocked the induction of type I interferon and cytokine responses to DNA and DNA viruses. |
| 60413 | DDX41 | 21892174 | DDX41 is a helicase responsible for sensing intracellular DNA in myeloid dendritic cells. DDX41 expression knockdown blocked the induction of type I interferon and cytokine responses to DNA and DNA viruses. (Demonstrated in mice) |
| 148305 | Tax1bp1 | 21765415 | Tax1bp1 phosphorylation by Chuk is pivotal for the proinflammatory cytokine-dependent assembly of the A20 ubiquitin-editing complex to limit inflammatory gene activation. |
| 10692 | TAX1BP1 | 17283140 | TAX1BP1 is a nuclear receptor co-activator that forms a complex with the glucocorticoid receptor (NR3C1). TAX1BP1 can bind Tax, a human T-cell leukemia virus type 1 oncoprotein, directly and this induces the dissociation of TAX1BP1 from the glucocorticoid receptor-containing protein complex, and represses the co-activator function of TAX1BP1. |
| 10692 | TAX1BP1 | 20304918 | TAX1BP1 and TNFAIP3 (A20) inhibit antiviral signalling by targeting TBK1/IKKi kinases and disrupting the TRAF3-TBK1-IKKi signalling complex. |

| | | | |
|---|---|---|---|
| 10692 | TAX1BP1 | 21765415 | TAX1BP1 phosphorylation by CHUK is pivotal for the proinflammatory cytokine-dependent assembly of the A20 ubiquitin-editing complex to limit inflammatory gene activation. (Demonstrated in mouse) |
| 175208 | Neu1 | 21873432 | Neu1 and Mmp9 cross-talk in alliance with Tlr4 on the cell surface is a novel membrane sialidase-controlling mechanism that depends on ligand binding to its Toll-like receptor (TLR) to induce Neu1 activity, to influence receptor desialylation and subsequently to induce TLR receptor activation and the production of nitric oxide and pro-inflammatory cytokines in dendritic and macrophage cells. |
| 79398 | NEU1 | 21873432 | NEU1 and MMP9 cross-talk in alliance with TLR4 on the cell surface is a novel membrane sialidase-controlling mechanism that depends on ligand binding to its Toll-like receptor (TLR) to induce NEU1 activity, to influence receptor desialylation and subsequently to induce TLR receptor activation and the production of nitric oxide and pro-inflammatory cytokines in dendritic and macrophage cells. (Demonstrated in murine model) |
| 137002 | Aqp3 | 21865318 | Aqp3 is an aquaporin that functions in macrophage immunity by a cellular mechanism involving facilitated water and glycerol transport, and consequent phagocytic and migration activity. Aqp3, therefore, is a novel therapeutic target in modulating the innate immune response in various infectious and inflammatory conditions. |
| 57960 | AQP3 | 21865318 | AQP3 is an aquaporin that functions in macrophage immunity by a cellular mechanism involving facilitated water and glycerol transport, and consequent phagocytic and migration activity. AQP3, therefore, is a novel therapeutic target in modulating the innate immune response in various infectious and inflammatory conditions. |
| 182872 | Elf1 | 21867680 | Elf1 transcription factor negatively regulates Tollip, a negative regulator of Toll-like receptor (TLR) signalling, in response to O-linked N-acetylglucosamine (O-GlcNAc) modification. In intestinal epithelial cells insufficient O-GlcNAc modification prevents Elf1-mediated transcriptional repression and thereby suppresses TLR signalling via upregulated Tollip gene expression. (Demonstrated in human) |
| 26868 | ELF1 | 21867680 | ELF1 transcription factor negatively regulates TOLLIP, a negative regulator of Toll-like receptor (TLR) signalling, in response to O-linked N-acetylglucosamine (O-GlcNAc) modification. In intestinal epithelial cells, insufficient O-GlcNAc modification prevents ELF1-mediated transcriptional repression and thereby suppresses TLR signalling via upregulated TOLLIP gene expression. |

| | | | |
|---|---|---|---|
| 155582 | Cd8a | 21867928 | Cd8a-positive dendritic cells play a critical role in cross-presentation of antigens during intracellular pathogen infections, specifically by activating an innate immune response through Il12 production. |
| 59987 | CD8A | 21867928 | CD8A-positive dendritic cells play a critical role in cross-presentation of antigens during intracellular pathogen infections, specifically by activating an innate immune response through IL12 production. (Demonstrated in murine model) |
| 163463 | Muc1 | 21868711 | Muc1 controls the inflammatory response in airway epithelial cells during nontypeable Haemophilus influenzae infection, mainly through suppression of Tlr2 signalling and decreased Il8 production. |
| 103122 | MUC1 | 20375631 | MUC1 regulates innate immune responses of dendritic cells (DC) where deletion of MUC1 promotes a heightened functional response of DC in response to TLR4 and TLR5 signalling pathways. |
| 103122 | MUC1 | 21868711 | MUC1 controls the inflammatory response in airway epithelial cells during nontypeable Haemophilus influenzae infection, mainly through suppression of TLR2 signalling and decreased IL8 production. (Demonstrated in murine model) |
| 148344 | Il1rl2 | 21860022 | Il1rl2 binds members of the interleukin (IL)-1 family of cytokines that includes Il1f6, Il1f8 and Il1f9. Binding of these ligands to Il1rl2 plays a critical role in the interface between innate and adaptive immunity, leading to the stimulation of dendritic and T helper cell responses. |
| 64113 | IL1RL2 | 21860022 | IL1RL2 binds members of the interleukin (IL)-1 family of cytokines that includes IL1F6, IL1F8 and IL1F9. Binding of these ligands to IL1RL2 plays a critical role in the interface between innate and adaptive immunity, leading to the stimulation of dendritic and T helper cell responses. (Demonstrated in murine model) |
| 210447 | Rpl19 | 21860608 | Rpl19 inhibits Irf3 activation and Cxcl10 production to facilitate viral multiplication in cells that express Tlr3 in endosomes, and Rpl19 inhibits viral multiplication in cells bearing Tlr3 on their cell membrane. (Demonstrated in human) |
| 45490 | RPL19 | 21860608 | RPL19 inhibits IRF3 activation and CXCL10 production to facilitate viral multiplication in cells that express TLR3 in endosomes, and RPL19 inhibits viral multiplication in cells bearing TLR3 on their cell membrane. |
| 142010 | Selk | 21849499 | Selk is a novel target for calpain protease and its cleavage is regulated by the calpain/calpastatin system during Toll-like receptor (TLR)-induced activation of macrophages. |

| | | | |
|---|---|---|---|
| 4354 | BCL2 | 21850221 | BCL2 is a multifunctional regulator of cell survival that inhibits the innate immune response during early stages of pathogenesis. Muscle-specific expression of BCL2 in Lama2-deficient mice resulted in the inhibition of TLR4, TLR6, TLR7, TLR8 and TLR9 induction, leading to reduced infiltration of eosinophils, the principal death effector cells. (Demonstrated in murine model) |
| 209275 | Il4ra | 21825018 | The Il4ra signalling pathway contributes to the heightened susceptibility of mice co-infected with Mycobacterium tuberculosis and the intestinal helminth, Nippostrongylus brasiliensis |
| 21808 | IL4R | 8609418 | IL4R is utilized by IL13 to induce phosphorylation and activation of JAK2 in human colon carcinoma cell lines. |
| 21808 | IL4R | 21825018 | The IL4R signalling pathway contributes to the heightened susceptibility of mice co-infected with Mycobacterium tuberculosis and the intestinal helminth, Nippostrongylus brasiliensis. (Demonstrated in mice) |
| 151871 | Sftpd | 21821801 | Sftpd binds to Mycobacterium avium lipoarabinomannan and resulting in the agglutination of the pathogen. The presence of Sftpd promotes the phagocytosis of M. avium by macrophages. (Demonstrated in human) |
| 80187 | SFTPD | 18990700 | SFTPD inhibits lipopolysaccharide (LPS)-induced inflammatory cell responses by altering LPS binding to its receptors where it binds to a complex of TLR4/LY96 (MD-2) with high affinity and significantly reduces LY96 binding to both serotypes of LPS. |
| 80187 | SFTPD | 19007302 | SFTPD, upon S-nitrosylation, controls inflammatory function by acting as a chemoattractant for macrophages and inducing p38 MAPK phosphorylation. |
| 80187 | SFTPD | 20413160 | SFTPD is a carbohydrate pattern recognition molecule of innate immunity that significantly enhances phagocytosis and killing of Aspergillus fumigatus, a pathogenic fungus, by neutrophils and macrophages. |
| 80187 | SFTPD | 20418258 | SFTPD can attenuate bacterial and viral infection and inflammation by acting as an opsonin and by regulating innate immune cell functions. |
| 80187 | SFTPD | 21821801 | SFTPD binds to Mycobacterium avium lipoarabinomannan and resulting in the agglutination of the pathogen. The presence of SFTPD promotes the phagocytosis of M. avium by macrophages. |
| 159817 | Ifit3 | 21813773 | Ifit3 triggers host antiviral responses by bridging Tbk1 to Mavs, and Ifit3 plays an important role in the activation of Irf3. (Demonstrated in human) |

| | | | |
|---|---|---|---|
| 224213 | mmu-mir-29b-1 | 21785411 | mmu-mir-29b-1 suppresses immune responses to intracellular pathogens by targeting IFN-gamma mRNA. Mice infected with Listeria monocytogenes or Mycobacterium bovis bacillus Calmette-Gu Ã © rin (BCG) downregulated miR-29 expression. |
| 212419 | Pltp | 21787334 | Pltp may play a pivotal role in inflammation and innate immunity through its ability to accelerate the 'reverse LPS transport' pathway. |
| 78556 | PLTP | 21787334 | PLTP may play a pivotal role in inflammation and innate immunity through its ability to accelerate the 'reverse LPS transport' pathway. (Demonstrated in mouse) |
| 66205 | PLUNC | 21054862 | PLUNC is a protein able to inhibit Mycoplasma pneumoniae (Mp) growth and its production following Mp infection is regulated through Toll-like receptor 2 (TLR2) signalling, as determined in mice. |
| 66205 | PLUNC | 21787346 | PLUNC is a secretory protein that exhibits antimicrobial activity against Gram-negative bacteria and anti-inflammatory functions in respiratory infections. |
| 189788 | Ltbr | 21767811 | Ltbr signalling in the gut lymphoid follicles regulates Il22 production by innate lymphoid cells in response to mucosal pathogen challenge. |
| 13973 | LTBR | 20226692 | LTBR signalling in intestinal epithelial cells orchestrates innate immune responses against mucosal bacterial infection. |
| 13973 | LTBR | 21767811 | LTBR signalling in the gut lymphoid follicles regulates IL22 production by innate lymphoid cells in response to mucosal pathogen challenge. (Demonstrated in mouse) |
| 193190 | Rag1 | 21746813 | Rag1 knockout mice display deficient early inflammatory responses and reduced survival during sepsis, which demonstrates a novel role of B cells in the early innate immune response. |
| 40149 | RAG1 | 21746813 | Rag1 knockout mice display deficient early inflammatory responses and reduced survival during sepsis, which demonstrates a novel role of B cells in the early innate immune response. (Demonstrated in mice) |
| 193693 | Tnfsf9 | 21747409 | The expression of ligand-receptor pair Tnfsf9-Tnfrsf9 on monocytes and NK cells is induced by Mycobacterium tuberculosis. Blockage of the Tnfrsf9 pathway enhanced the level of Ifng and Tnfa producing lymphocytes against the pathogen. (Demonstrated in human) |
| 21688 | TNFSF9 | 21747409 | The expression of ligand-receptor pair TNFSF9-TNFRSF9 on monocytes and NK cells is induced by Mycobacterium tuberculosis. Blockage of the TNFRSF9 pathway enhanced the level of IFNG and TNFA producing lymphocytes against the pathogen. |

| | | | |
|---|---|---|---|
| 205902 | Tnfrsf9 | 21747409 | The expression of ligand-receptor pair Tnfsf9-Tnfrsf9 on monocytes and NK cells is induced by Mycobacterium tuberculosis. Blockage of the Tnfrsf9 pathway enhanced the level of Ifng and Tnfa producing lymphocytes against the pathogen. (Demonstrated in human) |
| 88268 | TNFRSF9 | 21747409 | The expression of the ligand-receptor pair TNFSF9-TNFRSF9 on monocytes and natural killer cells is induced by Mycobacterium tuberculosis. Blockage of the TNFRSF9 pathway enhanced the level of IFNG and TNFA producing lymphocytes against the pathogen. |
| 152558 | Snca | 21747756 | Snca acts as a danger-associated molecular pattern that activates the expression of TLRs to initiate the proinflammatory pathway and microglial activation. (Demonstrated in human) |
| 30077 | SNCA | 21747756 | SNCA acts as a danger-associated molecular pattern that activates the expression of TLRs to initiate the proinflammatory pathway and microglial activation. |
| 152055 | F11 | 21807745 | F11 modulates the inflammatory response of PMN leukocytes by reducing chemotaxis triggered by Il8 or fMLP, highlighting the interplay between inflammation and coagulation. |
| 47627 | F11 | 21807745 | F11 modulates the inflammatory response of PMN leukocytes by reducing chemotaxis triggered by IL8 or fMLP, highlighting the interplay between inflammation and coagulation (Demonstrated in mouse) |
| 102968 | ADAR | 21809195 | ADAR destabalizes RNA structure by the deamination of adenosine to inosine, and therefore is able to disrupt replication of dsRNA viruses in the host. |
| 189844 | Tnfrsf1a | 21802165 | Tnfrsf1a modulates nitric oxide production in peritoneal macrophages in response to Yersinia LPS stimulation through the Il6 and NFkB signalling pathway. |
| 13807 | TNFRSF1A | 12753742 | Recruitment of TNF receptor 1 to lipid rafts is essential for TNFalpha-mediated NF-kappaB activation |
| 13807 | TNFRSF1A | 19150425 | TNFR1 mediates TRAF2 phosphorylation and recruitment of the IKK complex |
| 13807 | TNFRSF1A | 21802165 | TNFRSF1A modulates nitric oxide production in peritoneal macrophages in response to Yersinia LPS stimulation through the IL6 and NFkB signalling pathway. (Demonstrated in mouse) |
| 189835 | Treml2 | 21804015 | Tlt2 enhances neutrophil functions such as antibacterial activity and chemotaxis by potentiating the response to the G protein-coupled receptor-signalling pathway. |
| 86610 | TREML2 | 21804015 | TLT2 enhances neutrophil functions such as antibacterial activity and chemotaxis by potentiating the response to the G protein-coupled receptor-signalling pathway. |
| 155970 | Cbl | 21799517 | Cbl, an E3 ligase, has a crucial role in regulating dendritic cell maturation by facilitating the regulatory functions of Nfkb1. |

| | | | |
|---|---|---|---|
| 74417 | CBL | 21799517 | CBL, an E3 ligase, has a crucial role in regulating dendritic cell maturation by facilitating the regulatory functions of NFKB1. (Demonstrated in mouse) |
| 155162 | Hspd1 | 21145789 | Hspd1 plays a dual role as an immune modulator and a biomarker, and is a target to modulate immunity for therapeutic purposes, and to monitor the immune response in health and disease. |
| 155162 | Hspd1 | 21775438 | Hspd1 and Tlr4 mediate myocardial ischemia-activated innate immune signalling, which plays an important role in mediating apoptosis and inflammation during ischemia/reperfusion (I/R). |
| 77925 | HSPD1 | 18575269 | HSPD1 is part of the heat shock family of proteins that have many roles in inflammation and regulation of the immune system. |
| 77925 | HSPD1 | 21145789 | HSPD1 plays a dual role as an immune modulator and a biomarker, and is a target to modulate immunity for therapeutic purposes, and to monitor the immune response in health and disease. |
| 77925 | HSPD1 | 21775438 | HSPD1 and TLR4 mediate myocardial ischemia-activated innate immune signalling, which plays an important role in mediating apoptosis and inflammation during ischemia/reperfusion (I/R). (Demonstrated in murine model) |
| 171712 | Pml | 21131187 | Pml, a member of the Trim protein family, is upregulated by Type I and Type II interferons and have been found to restrict viral replication by modulating the RIG-I pathway. |
| 171712 | Pml | 21779477 | Pml(-/-) mice are resistant to LPS-induced septic shock as a result of an ineffective production of cytokines and chemokines, suggesting a role for PML in the innate immune Toll-like receptor (TLR)/NF-kB pathway. Pml(-/-) mice also exhibit impaired function of macrophages and are thus unable to clear pathogenic microorganisms. |
| 21462 | PML | 21131187 | PML, a member of the TRIM protein family, is upregulated by Type I and Type II interferons and have been found to restrict viral replication by modulating the RIG-I pathway. |
| 21462 | PML | 21779477 | Pml(-/-) mice are resistant to LPS-induced septic shock as a result of an ineffective production of cytokines and chemokines, suggesting a role for PML in the innate immune Toll-like receptor (TLR)/NF-kB pathway. Pml(-/-) mice also exhibit impaired function of macrophages and are thus unable to clear pathogenic microorganisms. (Demonstrated in murine model) |
| 137359 | Pin1 | 21743479 | Pin1 is necessary to mount TLR-mediated, interferon-dependent innate and adaptive immune response. Pin1 is activated by Tlr7 and Tlr9, which binds to Irak1 to activate Irf7 which then induce type I interferons. |

| | | | |
|---|---|---|---|
| 26087 | PIN1 | 16699525 | PIN1 negatively regulates IRF3-dependent innate antiviral response by binding to phosphorylated IRF3 for proteasome-dependent degradation. |
| 26087 | PIN1 | 21743479 | PIN1 is necessary to mount TLR-mediated, interferon-dependent innate and adaptive immune response. PIN1 is activated by TLR7 and TLR9, which binds to IRAK1 to activate IRF7 which then induce type I interferons. (Demonstrated in mouse) |
| 148872 | Hrg | 21757718 | Hrg binds fibrinogen with high affinity and competes with thrombin for binding. This interaction may provide a novel link between coagulation, innate immunity and inflammation. (Demonstrated in human) |
| 149571 | Notch1 | 21737799 | Notch1 signalling modulates the microglia innate response to post-ischemic brain damage inflammation. Notch1 deficient mice exhibit significantly lower levels of activated microglia and reduced proinflammatory cytokine expression. |
| 69039 | HRG | 21757718 | HRG binds fibrinogen with high affinity and competes with thrombin for binding. This interaction may provide a novel link between coagulation, innate immunity and inflammation. |
| 92445 | NOTCH1 | 21737799 | NOTCH1 signalling modulates the microglia innate response to post-ischemic brain damage inflammation. Notch1 deficient mice exhibit significantly lower levels of activated microglia and reduced proinflammatory cytokine expression. (Demonstrated in mouse) |
| 197330 | Gbp2 | 21757726 | Gbp2 localizes to intracellular compartments positive for macroautophagy markers and Immunity-Related GTPases (IRGs) indirectly modulate Gbp2 localization. |
| 100168 | GBP2 | 21757726 | GBP2 localizes to intracellular compartments positive for macroautophagy markers and Immunity-Related GTPases (IRGs) indirectly modulate GBP2 localization. (Demonstrated in murine model) |
| 133420 | Hspa14 | 21730052 | Hspa14 binds directly to Tlr4 on dendritic cell surfaces and induces a robust Th1 response via the MAPK and NFkB signalling pathways. |
| 55933 | HSPA14 | 21730052 | HSPA14 binds directly to TLR4 on dendritic cell surfaces and induces a robust Th1 response via the MAPK and NFkB signalling pathways. |
| 262889 | Tgtp1 | 21757726 | Tgtp1 is part of the Immunity-Related GTPases (IRGs) family of proteins that are induced by interferon-gamma (Ifng) and play a crucial role in innate resistance to intracellular pathogens. Tgtp1 also influences the localization of Gbp2 by modulating macroautophagy. |
| 263693 | Igtp | 21731484 | Igtp is responsible for the IFN-gamma mediated induction of the antimicrobial defence system against Chlamydia trachomatis in mouse. Irgm1/Igtp knockout mice develop high bacterial burden post intrauterine infection, but subsequently clear the infection more efficiently than wt mice due to a compensatory T cell response. |

| | | | |
|---|---|---|---|
| 263693 | Igtp | 21757726 | Igtp is part of the Immunity-Related GTPases (IRGs) family of proteins that are induced by interferon-gamma (Ifng) and play a crucial role in innate resistance to intracellular pathogens. Igtp also influences the localization of Gbp2 by modulating macroautophagy. |
| 147056 | Edn1 | 21730058 | Edn1 links Tlr7 inflammatory signalling to cardiac fibrosis in autoimmune associated congenital heart block. (Demonstrated in human) |
| 132667 | Socs1 | 21263070 | Socs1 is a negative immunomodulator that is upregulated by Hepatitis C virus to deliver negative signaling to Tlr-mediated pathways controlling expression of Il12, a key cytokine linking innate and adaptive immunity. |
| 132667 | Socs1 | 21606371 | Socs1 is targeted by endogenous and pharmacologic glucocorticoids to limit Tlr3/Tlr4-mediated Stat1 activation which results in the suppression of inflammation. |
| 132667 | Socs1 | 21757742 | Socs1 inhibits type I interferon (IFN) signaling through an interaction with the Ifnar1 associated kinase, Tyk2, resulting in a reduced IFN response and reduced Ifnar1 surface expression. (Demonstrated in human) |
| 61847 | EDN1 | 21730058 | EDN1 links TLR7 inflammatory signalling to cardiac fibrosis in autoimmune associated congenital heart block. |
| 103716 | CD5L | 21730133 | CD5L (AIM) is required for obesity-associated recruitment of inflammatory macrophages into adipose tissue. (Demonstrated in mouse) |
| 14621 | SOCS1 | 16451196 | SOCS1 is a potent and multifaceted regulator of cytokines and cell-mediated inflammation. |
| 14621 | SOCS1 | 19017994 | SOCS1 regulates the IFN but Not NF{kappa}B Pathway in TLR-Stimulated Human monocytes and macrophages. |
| 14621 | SOCS1 | 21263070 | SOCS1 is a negative immunomodulator that is upregulated by Hepatitis C virus to deliver negative signalling to TLR-mediated pathways controlling expression of IL12, a key cytokine linking innate and adaptive immunity. |
| 14621 | SOCS1 | 21606371 | SOCS1 is targeted by endogenous and pharmacologic glucocorticoids to limit TLR3/TLR4-mediated STAT1 activation which results in the suppression of inflammation. (Demonstrated in murine model) |
| 14621 | SOCS1 | 21757742 | SOCS1 inhibits type I interferon (IFN) signaling through an interaction with the IFNAR1 associated kinase, TYK2, resulting in a reduced IFN response and reduced IFNAR1 surface expression. |

| | | | |
|---|---|---|---|
| 180975 | Nras | 21757746 | Nras is a small GTPase that, in response to microbial activation, mediates cholangiocyte pro-inflammatory cytokine production and induction of cholangiocyte proliferation. (Demonstrated in human) |
| 166425 | Irgm1 | 21731484 | Irgm1 is responsible for the IFN-gamma mediated induction of the antimicrobial defence system against Chlamydia trachomatis in mouse. Irgm1/Igtp knockout mice develop high bacterial burden post intrauterine infection, but subsequently clear the infection more efficiently than wt mice due to a compensatory T cell response. |
| 243969 | NRAS | 21757746 | NRAS is a small GTPase that, in response to microbial activation, mediates cholangiocyte pro-inflammatory cytokine production and induction of cholangiocyte proliferation. |
| 194580 | Aimp1 | 21711348 | Aimp1 is a pleiotropic cytokine expressed in the salivary glands, small intestine and large intestine. Aimp1 not only induces the maturation and activation of bone marrow-derived dendritic cells, but also induces the expression of Tlr1, 2, 3 and 7. |
| 401910 | MIF | 17435771 | MIF is a chemokine-like inflammatory mediator that triggers leukocyte recruitment by binding to CXCR2 and CXCR4. (Demonstrated in murine model) |
| 163956 | Gm16379 | 17435771 | Gm16379 (Mif) is a chemokine-like inflammatory mediator that triggers leukocyte recruitment by binding to Cxcr2 and Cxcr4. |
| 202825 | Duox2 | 21714724 | Duox2 plays pivotal roles in the Tlr5-dependent inflammatory response of nasal airway epithelium. Duox2 activation is required for flagellin-induced reactive oxygen species production, as well as the induction of mucin and MIP-2alpha in nasal epithelial cells. |
| 10041 | DUOX2 | 19759286 | DUOX2 is a member of the NAD(P)H oxidase family and is involved in NOD2-dependent reactive oxygen species (ROS) production. |
| 10041 | DUOX2 | 18511861 | DUOX2 and DUOX1 localize to the apical plasma membrane of epithelial cells in major airways, salivary glands, and the gastrointestinal tract, and provide extracellular hydrogen peroxide to lactoperoxidase to produce antimicrobial hypothiocyanite ions. Expression of dual oxidases DUOX2 and DUOX1 is regulated by Th1 and Th2 cytokines in human airways. |
| 10041 | DUOX2 | 20381453 | DUOX2 expression is mediated by IFN-gamma via a STAT-independent signalling pathway, providing insights into a novel IFN-gamma signalling pathway with potential importance for regulation of host defence responses. |

| | | | |
|---|---|---|---|
| 10041 | DUOX2 | 21714724 | DUOX2 plays pivotal roles in the TLR5-dependent inflammatory response of nasal airway epithelium. DUOX2 activation is required for flagellin-induced reactive oxygen species production, as well as the induction of mucin and MIP-2alpha in nasal epithelial cells. |
| 145192 | Rarres2 | 21715684 | Rarres2 (Chemerin) requires C-terminal proteolytic processing by cysteine cathepsins to selectively attract a specific subset of immunoregulatory APCs, such as immature plasmacytoid dendritic cells. In addition, truncated Rarres2 also displays antibacterial activity against Enterobacteriaceae. (Demonstrated in human) |
| 47881 | RARRES2 | 21715684 | RARRES2 (Chemerin) requires C-terminal proteolytic processing by cysteine cathepsins to selectively attract a specific subset of immunoregulatory APCs, such as immature plasmacytoid dendritic cells. In addition, truncated RARRES2 also displays antibacterial activity against Enterobacteriaceae. |
| 196988 | Kcnj8 | 21719711 | Kcnj8 is an ATP-sensitive potassium channel that restricts cardiotropic RNA virus replication. |
| 166063 | Nfatc4 | 21726630 | Nfatc4 is a transcription factor required for the regulation of the Toll-like receptor-activated innate inflammatory response in monocytes/macrophages. |
| 23052 | KCNJ8 | 21719711 | KCNJ8 is an ATP-sensitive potassium channel that restricts cardiotropic RNA virus replication. (Demonstrated in murine model) |
| 3927 | NFATC4 | 21726630 | NFATC4 is a transcription factor required for the regulation of the Toll-like receptor-activated innate inflammatory response in monocytes/macrophages. (Demonstrated in murine model) |
| 189544 | Nfatc3 | 21726630 | Nfatc3 is a transcription factor required for the regulation of the Toll-like receptor-activated innate inflammatory response in monocytes/macrophages. |
| 38028 | NFATC3 | 21726630 | NFATC3 is a transcription factor required for the regulation of the Toll-like receptor-activated innate inflammatory response in monocytes/macrophages. (Demonstrated in murine model) |
| 191926 | Clec1b | 21728173 | Clec1b is expressed in myeloid cells and acts as a Syk-coupled C-type lectin receptor (CLR) able to modulate Toll-like receptor (TLR) signaling and inflammatory responses. |
| 18309 | CLEC1B | 21728173 | CLEC1B is expressed in myeloid cells and acts as a SYK-coupled C-type lectin receptor (CLR) able to modulate Toll-like receptor (TLR) signaling and inflammatory responses. ( Demonstrated in murine model) |
| 147779 | Dhx36 | 21703541 | Dhx36 is a component of a cytosolic viral sensor and is recruited to a complex consisting of Ddx1, Ddx21 and Ticam1, which triggers type I interferon and cytokine response to dsRNA. |

| | | | |
|---|---|---|---|
| 62011 | DHX36 | 20696886 | DHX36 interacts with CpG-A and is associated with IFN-alpha production and IRF7 nuclear translocation upon CpG-A stimulation. DHX36 localizes within the cytosol and directly binds to the TLR domain of MYD88. |
| 62011 | DHX36 | 21703541 | DHX36 is a component of a cytosolic viral sensor and is recruited to a complex consisting of DDX1, DDX21 and TICAM1, which triggers type I interferon and cytokine response to dsRNA. (Demonstrated in murine model) |
| 157719 | Ddx21 | 21703541 | Ddx21 is a component of a cytosolic viral sensor and is recruited to a complex consisting of Ddx1, Dhx36 and Ticam1, which triggers type I interferon and cytokine response to dsRNA. |
| 76283 | DDX21 | 21703541 | DDX21 is a component of a cytosolic viral sensor and is recruited to a complex consisting of DDX1, DHX36 and TICAM1, which triggers type I interferon and cytokine response to dsRNA. (Demonstrated in murine model) |
| 128974 | Ddx1 | 21703541 | Ddx1 is a cytosolic viral sensor in dendritic cells that binds to dsRNA through its Helicase A domain. Ddx1 then recruits Ddx21, Dhx36 and Ticam1 to mount type I interferon and cytokine response to poly I:C, influenza A virus, and reovirus. |
| 30761 | DDX1 | 21703541 | DDX1 is cytosolic viral sensor in dendritic cells that binds to dsRNA through its Helicase A domain. DDX1 then recruits DDX21, DHX36 and TICAM1 to mount type I interferon and cytokine response to poly I:C, Influenza A virus and reovirus. (Demonstrated in murine model) |
| 172600 | Cd97 | 21706400 | Cd97 plays an important role in recruiting granulocytes and possibly macrophages to the sites of infection. |
| 33120 | CD97 | 21706400 | CD97 plays an important role in recruiting granulocytes and possibly macrophages to the sites of infection. |
| 152615 | Adrb2 | 21683614 | Activation of Adrb2 on Nod2/Tlr2-stimulated dendritic cells biases the cell priming ability towards an Th17 immune response. |
| 52685 | ADRB2 | 21683614 | Activation of ADRB2 on NOD2/TLR2-stimulated dendritic cells biases the cell priming ability towards an Th17 immune response. (Demonstrated in murine model) |
| 145910 | Jam3 | 21706006 | Jam3 is a key regulator of polarized neutrophil transendothelial migration in vivo. |
| 76413 | JAM3 | 21706006 | JAM3 is a key regulator of polarized neutrophil transendothelial migration in vivo. (Demonstrated in murine model) |
| 8995 | AHR | 21683686 | AHR deficiency imapirs TLR and NFkB-mediated proinflammatory gene expression after activation by a classical stimulus, such as LPS. (Demonstrated in murine model) |

| | | | |
|---|---|---|---|
| 148620 | Hif1a | 21685248 | Hif1a, under normoxic conditions, accumulates in dendritic cells via the TLR/Myd88/NFkB signalling pathway to induce a distinct subset of proinflammatory genes in comparison to hypoxia-induced Hif1a. |
| 157923 | Ubqln1 | 21695056 | Ubqln1 strongly suppresses the transcriptional activation of Ifnb promoter and is an inhibitor of the Tlr3/Ticam1 anti-viral pathway by reducing Ticam1 protein levels. (Demonstrated in human) |
| 73620 | UBQLN1 | 21695056 | UBQLN1 strongly suppresses the transcriptional activation of the IFNB promoter and is an inhibitor of the TLR3/TICAM1 anti-viral pathway by reducing TICAM1 protein levels. |
| 155006 | Pmaip1 | 21698224 | Pmaip1 is induced by the RIG-I/MDA5 axis during viral infection and activates apoptosis in macrophages, dendritic cells and primary lymphocytes. |
| 4142 | PMAIP1 | 21698224 | PMAIP1 is induced by the RIG-I/MDA5 axis during viral infection and activates apoptosis in macrophages, dendritic cells and primary lymphocytes. |
| 198951 | Ip6k1 | 21685907 | Ip6k1 disruption results in the augmentation of downstream phosphatidylinositol-(3,4,5)-triphosphate signalling in neutrophils. As a result, these neutrophils exhibited greater phagocytic and bactericidal ability and amplified NADPH oxidase-mediated production of superoxide. |
| 35427 | IP6K1 | 21685907 | IP6K1 disruption results in the augmentation of downstream phosphatidylinositol-(3,4,5)-triphosphate signalling in neutrophils. As a result, these neutrophils exhibited greater phagocytic and bactericidal ability and amplified NADPH oxidase-mediated production of superoxide. |
| 193424 | Khsrp | 21690298 | Khsrp directly interacts with AU-rich elements in the 3' UTR of Ifn mRNA to negatively regulate the transcript levels. Khsrp deficient mouse embryonic fibroblast produced higher levels of Ifna4 and Ifnb mRNAs in response to viral infections as a result of decreased mRNA decay. |
| 21386 | KHSRP | 21690298 | KHSRP directly interacts with AU-rich elements in the 3' UTR of IFN mRNA to negatively regulate the transcript levels. Khsrp deficient mouse embryonic fibroblast produced higher levels of Ifna4 and Ifnb mRNAs in response to viral infections as a result of decreased mRNA decay. |
| 176038 | Ifnar1 | 21408089 | Ifnar1 deficiency completely abolishes the reduction of sterol biosynthetic activity of macrophages during viral infections, thereby linking the regulation of lipid metabolism pathway with interferon anti-viral defence responses. |
| 176038 | Ifnar1 | 21695243 | Ifnar1 is phosphorylated by p38 MAP kinase in response to pathogen-recognition receptor stimulation. This phosphorylation promotes Ifnar1 ubiquitination and accelerates the proteolytic turnover of the receptor, which leads to attenuation of type I IFN signalling. |

| | | | |
|---|---|---|---|
| 2095 | IFNAR1 | 8605876 | IFNAR1 acts as a docking site for the latent form of STAT2 and mediates the interaction between JAK kinases and the STAT transcription factors. |
| 2095 | IFNAR1 | 9461596 | IFNAR1 interacts with the amino-terminal half of TYK2 and this interaction is required for interferon (IFN)-alpha signal transduction. |
| 2095 | IFNAR1 | 12220192 | The interferon alpha receptor is composed of two subunits: IFNAR1 and IFNAR2;IL10RB. IFNAR1 binds to STAT2 to initiate interferon (IFN) signalling. |
| 2095 | IFNAR1 | 20483775 | Type I interferons (IFNs) play an important role in innate immunity to protozoan parasites by binding the IFN alpha receptor, composed of IFNAR1 and IFNAR2;IL10RB, and regulating neutrophil/monocyte recruitment, neutrophil turnover, and Leishmania infection. |
| 2095 | IFNAR1 | 21408089 | IFNAR1 deficiency completely abolishes the reduction of sterol biosynthetic activity of macrophages during viral infections, thereby linking the regulation of lipid metabolism pathway with interferon anti-viral defence responses. (Demonstrated in murine model) |
| 2095 | IFNAR1 | 21695243 | IFNAR1 is phosphorylated by p38 MAP kinase in response to pathogen-recognition receptor stimulation. This phosphorylation promotes IFNAR1 ubiquitination and accelerates the proteolytic turnover of the receptor, which leads to attenuation of type I IFN signalling. |
| 202215 | Tpst1 | 21665277 | Tpst1 silencing reduces the Il6 production in LPS-stimulated macrophages. |
| 18615 | TPST1 | 21665277 | TPST1 silencing reduces the IL6 production in LPS-stimulated macrophages. (Demonstrated in murine model) |
| 153069 | Plec | 21665277 | Plec silencing reduces the Il6 production in LPS-stimulated macrophages. |
| 39382 | PLEC | 21665277 | PLEC silencing reduces the IL6 production in LPS-stimulated macrophages. (Demonstrated in murine model) |
| 93605 | VENTX | 21670496 | VENTX plays a pivotal role in human macrophage terminal differentiation and proinflammatory function. VENTX is upregulated during monocyte to macrophage differentiation, and ablation of VENTX in monocytes profoundly impairs their differentiation to macrophages. |
| 206550 | Ccr3 | 21660963 | Epithelial Ccr3 is involved in the progression of LPS-induced lung inflammation by mediating the release of Il8. |
| 30125 | CCR3 | 21660963 | Epithelial CCR3 is involved in the progression of LPS-induced lung inflammation by mediating the release of IL8. |
| 155959 | Vldlr | 21642441 | Vldlr is exploited by rhinovirus for viral entry. Vldlr protein levels are negatively regulated by IFN/RIG-I signalling via MIR23B. (Demonstrated in human) |

| | | | |
|---|---|---|---|
| 44752 | VLDLR | 21642441 | VLDLR is exploited by rhinovirus for viral entry. VLDLR protein levels are negatively regulated by IFN/RIG-I signalling via MIR23B. |
| 127077 | MIR23B | 21642441 | MIR23B expression is induced by RIG-I signalling and targets VLDLR and LRP5. MIR23B specifically inhibits rhinovirus infection which utilizes VLDLR for viral entry. |
| 157342 | Cflar | 21635783 | Cflar confers protection against cytoplasmic dsRNA-mediated cell death and down-regulates Irf3- and NFkB-mediated gene expression. In addition, Cflar also negatively regulates LPS-induced Tlr4-signalling in endothelial cells and protects against Tlr4-mediated apoptosis. |
| 78386 | CFLAR | 21635783 | CFLAR confers protection against cytoplasmic dsRNA-mediated cell death and down-regulates IRF3- and NFkB-mediated gene expression. In addition, CFLAR also negatively regulates LPS-induced TLR4-signalling in endothelial cells and protects against TLR4-mediated apoptosis. (Demonstrated in murine model) |
| 165782 | Havcr2 | 21637332 | Havcr2 is constitutively expressed on human resting monocytes/macrophages and functions as a cap to block Il12, which is a key pro-inflammatory cytokine linking innate and adaptive immune responses. Havcr2 plays a crucial role in the negative regulation of innate immune responses through crosstalk with Pdcd1 and Socs1 to limit Stat1 phosphorylation in HCV infection. (Demonstrated in human) |
| 131439 | Cdk6 | 21628465 | Cdk6 expression is induced upon LPS stimulation via Tlr4-signalling pathway. Cdk6 mediates the increased adhesion of macrophages in response to LPS challenge, and is important for LPS lethality. |
| 26773 | CDK6 | 21628465 | CDK6 expression is induced upon LPS stimulation via TLR4-signalling pathway. CDK6 mediates the increased adhesion of macrophages in response to LPS challenge, and is important for LPS lethality. (Demonstrated in murine model) |
| 126455 | MIR107 | 21628465 | MIR107 is a negative regulator of macrophage adhesion by targeting the degradation of CDK6 RNA, which confers protection against the lethal effect of LPS. The expression of MIR107 is inhibited by TLR4 signalling. |
| 146283 | Gpr77 | 21630250 | Gpr77 is an alternate receptor for C5a and is negatively regulated by the TLR-signalling pathway. TLR-mediated inhibition of Gpr77 results in the amplification of complement pro-inflammatory responses. |
| 59470 | GPR77 | 21630250 | GPR77 is an alternate receptor for C5a and is negatively regulated by the TLR-signalling pathway. TLR-mediated inhibition of GPR77 results in the amplification of complement pro-inflammatory responses. (Demonstrated in murine model) |

| | | | |
|---|---|---|---|
| 136357 | Lilrb3 | 20398663 | Lilrb3 negatively regulates macrophage functions in response to pathogenic bacteria and chronic intestinal inflammatory responses, as demonstrated by increased production of proinflammatory cytokines (IL-6, IL-1beta and TNF-alpha) and activation of MAPK and NFkappaB in Lilrb3(-/-) macrophages following bacterial activation. |
| 165835 | Pik3ap1 | 20728433 | Pik3ap1 acts as a negative regulator of TLR-induced production of IL6 and IL10 in macrophages in response to LPS stimulation. |
| 83923 | PIK3AP1 | 20728433 | PIK3AP1 acts as a negative regulator of TLR-induced production of IL6 and IL10 in macrophages in response to LPS stimulation. (Demonstrated in murine model) |
| 206576 | Avp | 21615666 | Avp regulates water absorption in the collecting duct and acts as a potent modulator of the TLR4-mediated intrarenal innate response caused by uropathogenic E. coli. |
| 45432 | AVP | 21615666 | AVP regulates water absorption in the collecting duct and acts as a potent modulator of the TLR4-mediated intrarenal innate response caused by uropathogenic E. coli. |
| 157464 | Casp8 | 21419663 | Casp8 restricts the RIG-I-mediated activation of Irf3 by catalytically cleaving Ripk1, and subsequently converting Ripk1 from a signalling enhancer to a signalling inhibitor. |
| 78534 | CASP8 | 17213198 | CASP8 has an essential role in the regulation of NF-kappaB function in response to TLR4 stimulation, whereby CASP8 recruitment to IKBKB leads to delayed NF-kappaB nuclear translocation and impaired NF-kappaB transcriptional activity. |
| 78534 | CASP8 | 20019748 | CASP8 is involved in pro-apoptotic signalling through TLR3 and its activation is TICAM1 (TRIF)-dependent. |
| 78534 | CASP8 | 21419663 | CASP8 restricts the RIG-I-mediated activation of IRF3 by catalytically cleaving RIPK1, and subsequently converting RIPK1 from a signalling enhancer to a signalling inhibitor. |
| 33208 | SREBF1 | 21531336 | SREBF1 deficient mice are resistant to endotoxic shock and systemic inflammatory response syndrome induced by cecal ligation and puncture. SREBF1 is not only a necessary transcription factor for lipogenesis genes in macrophages, but is also responsible for the expression of NLRP1A, which is a core inflammasome component. (Demonstrated in murine model) |
| 179865 | Srebf1 | 21531336 | Srebf1 deficient mice are resistant to endotoxic shock and systemic inflammatory response syndrome induced by cecal ligation and puncture. Srebf1 is not only necessary for transcription of lipogenesis genes in macrophages, but is also responsible for the expression of Nlrp1a, which is a core inflammasome component. |

| | | | |
|---|---|---|---|
| 137011 | Zc3hav1 | 21102435 | Zc3hav1 isoform ZAPS is selectively induced by 5'-triphosphate-modified RNA and functions as a potent stimulator of interferon responses via the RIG-I signalling pathway. |
| 43487 | ZC3HAV1 | 21102435 | ZC3HAV1 isoform ZAPS is selectively induced by 5'-triphosphate-modified RNA and functions as a potent stimulator of interferon responses via the RIG-I signalling pathway. |
| 94856 | ATG5 | 17709747 | ATG5 forms a conjugate with ATG12 to directly associate with DDX58 and MAVS and negatively regulate the antiviral IFN production pathway by mediating autophagy. |
| 94856 | ATG5 | 17921696 | ATG12::ATG5 conjugate is a key regulator of the autophagic process to eliminate pathogens such as Streptococcus, M. tuberculosis, Listeria, and herpesvirus. ATG12::ATG5 also associates with components of the RIG-I pathway to negatively regulate type I IFN response and promote RNA virus replication. |
| 145432 | Atg12 | 17921696 | Atg12::Atg5 conjugate is a key regulator of the autophagic process to eliminate pathogens such as Streptococcus, M. tuberculosis, Listeria, and herpesvirus. Atg12::Atg5 also associates with components of the RIG-I pathway to negatively regulate type I IFN response and promote RNA virus replication. |
| 37900 | ATG12 | 17921696 | ATG12::ATG5 conjugate is a key regulator of the autophagic process to eliminate pathogens such as Streptococcus, M. tuberculosis, Listeria, and herpesvirus. ATG12::ATG5 also associates with components of the RIG-I pathway to negatively regulate type I IFN response and promote RNA virus replication. |
| 126503 | MIR373 | 21608007 | MIR373 is upregulated in hepatitis B virus-infected liver tissues to target NFIB mRNA for degradation and promotes viral replication. |
| 127071 | MIR372 | 21608007 | MIR372 is upregulated in hepatitis B virus-infected liver tissues to target NFIB mRNA for degradation and promotes viral replication. |
| 154783 | Akna | 21606955 | Akna targeted deletion results in susceptibility to pathogen-induced systemic inflammation and causes sudden neonatal death. |
| 82103 | AKNA | 21606955 | AKNA targeted deletion results in susceptibility to pathogen-induced systemic inflammation and causes sudden neonatal death. (Demonstrated in murine model) |
| 17914 | IRAK2 | 17878161 | IRAK2 has a role in TRAF6 ubiquitination in the Toll-like receptor (TLR) pathway and thus plays a more central role than IRAK1 in TLR signalling to NFkappaB. |

| | | | |
|---|---|---|---|
| 17914 | IRAK2 | 18438411 | IRAK2 is critical in late-phase Toll-like receptor (TLR) responses, and IRAK1 and IRAK2 are essential for the initial responses to TLR stimulation. |
| 17914 | IRAK2 | 19224918 | IRAK2 is required for LPS-mediated post-transcriptional control of cytokine and chemokine expression, which plays an essential role in TLR4-induced septic shock. |
| 17914 | IRAK2 | 21291324 | IRAK2 induces cytokine and chemokine mRNA stability and translation in response to LPS stimulation in macrophages. The kinase activity of IRAK2 is required for the optimal activation of mitogen activated protein kinase signaling, which regulates cytokine/chemokine production at posttranscriptional levels. |
| 17914 | IRAK2 | 21606490 | IRAK2 is required for both TLR4 and TLR8-mediated activation of NFkB and p38 MAP kinase, and the induction of TNF mRNA. In addition, IRAK2 is required for regulating MYD88-dependent TNF-alpha mRNA stability. (Phenotype was not observed in murine ortholog, Irak2) |
| 40784 | CCL1 | 18981157 | The JAK2/STAT5/CIS pathway suppresses CCL1 but not CCL7 and CCL8 chemokine expression |
| 40784 | CCL1 | 21601943 | CCL1 secretion in freshly isolated monocytes is induced by the combined engagement of TLR3/TLR4/TLR8. As monocytes differentiate, the capacity to induce CCL1 is lost via a IL23-dependent mechanism. |
| 168143 | Pglyrp4 | 21439073 | Pglyrp4 is a member of the Peptidoglycan Recognition Proteins (PGRP) family and recognizes peptidoglycan, a structural component of bacterial cell walls, as a part of innate immune response against infections. |
| 168143 | Pglyrp4 | 21602801 | Pglyrp4 binds to Gram-positive bacterial wall and activates a protein-sensing two-component system to induce bacterial death. PLGYRP1-mediated activation results in membrane depolarization and cessation of peptidoglycan, protein, and RNA/DNA synthesis, as well as the production of hydroxyl radicals. (Demonstrated in human) |
| 102639 | PGLYRP4 | 20418257 | PGLYRP4 is a secreted innate immunity protein that is expressed on body surfaces, mucous membranes, and in secretions (saliva, sweat) and is conserved from insects to mammals, it recognizes bacterial peptidoglycan, and functions in antibacterial immunity and inflammation. |

| | | | |
|---|---|---|---|
| 102639 | PGLYRP4 | 21439073 | PGLYRP4 is a member of the Peptidoglycan Recognition Proteins (PGRP) family and recognizes peptidoglycan, a structural component of bacterial cell walls, as a part of innate immune response against infections. |
| 102639 | PGLYRP4 | 21602801 | PGLYRP4 binds to Gram-positive bacterial wall and activates a protein-sensing two-component system to induce bacterial death. PLGYRP1-mediated activation results in membrane depolarization and cessation of peptidoglycan, protein, and RNA/DNA synthesis, as well as the production of hydroxyl radicals. |
| 148760 | Pglyrp1 | 21134971 | Pglyrp1 is a peptidoglycan recognition protein and play a role in innate immunity against L. monocytogenes infection by inducing Tnfa. |
| 148760 | Pglyrp1 | 21439073 | Pglyrp1 is a member of the Peptidoglycan Recognition Proteins (PGRP) family and recognizes peptidoglycan, a structural component of bacterial cell walls, as a part of innate immune response against infections. |
| 148760 | Pglyrp1 | 21602801 | Pglyrp1 binds to Gram-positive bacterial wall and activates a protein-sensing two-component system to induce bacterial death. PLGYRP1-mediated activation results in membrane depolarization and cessation of peptidoglycan, protein, and RNA/DNA synthesis, as well as the production of hydroxyl radicals. (Demonstrated in human) |
| 185854 | Gbp10 | 21551061 | Gbp10 is an IFN gamma-inducible gene that confers cell-autonomous immunity to listerial or mycobacterial infection within macrophages. Gbp10 interacts with host defense proteins, including phagocyte oxidase, antimicrobial peptides and autophagy effectors, to kill intracellular bacteria. |
| 197215 | Gbp7 | 21551061 | Gbp7 is an IFN gamma-inducible gene that confers cell-autonomous immunity to listerial or mycobacterial infection within macrophages. Gbp7 interacts with host defense proteins, including phagocyte oxidase, antimicrobial peptides and autophagy effectors, to kill intracellular bacteria. |
| 255448 | Gbp6 | 21551061 | Gbp6 is an IFN gamma-inducible gene that confers cell-autonomous immunity to listerial or mycobacterial infection within macrophages. Gbp6 interacts with host defense proteins, including phagocyte oxidase, antimicrobial peptides and autophagy effectors, to kill intracellular bacteria. |

| | | | |
|---|---|---|---|
| 197289 | Gbp1 | 21551061 | Gbp1 is an IFN gamma-inducible gene that confers cell-autonomous immunity to listerial or mycobacterial infection within macrophages. Gbp1 interacts with host defense proteins, including phagocyte oxidase, antimicrobial peptides and autophagy effectors, to kill intracellular bacteria. |
| 182101 | Tnfsf11 | 21566133 | Tlr2 and Tnfsf11 signalling pathways are modulated by Porphromonas gingivalis to alter the differentiation states of osteoclasts resulting in bacteria-mediated bone loss. |
| 28393 | TNFSF11 | 21566133 | TLR2 and TNFSF11 signalling pathways are modulated by Porphromonas gingivalis to alter the differentiation states of osteoclasts resulting in bacteria-mediated bone loss. (Demonstrated in murine model) |
| 42701 | IL4 | 21566158 | Exogenous IL4 was sufficient to drive the accumulation of tissue macrophages through self-renewal revealing that the expansion of innate cells necessary for pathogen control or wound repair can occur without recruitment of potentially tissue-destructive inflammatory cells. (Demonstrated in murine model) |
| 168042 | Arhgap15 | 21551229 | Arhgap15 negatively modulates Akt1 activity and thereby negatively regulates neutrophil function. Arhgap15 deficiency results in increased neutrophil recruitment to the site of infection and offers protection against an experimental model of severe abdominal sepsis. |
| 71605 | ARHGAP15 | 21551229 | ARHGAP15 negatively modulates AKT1 activity and thereby negatively regulates neutrophil function. ARHGAP15 deficiency results in increased neutrophil recruitment to the site of infection and offers protection against an experimental model of severe abdominal sepsis. (Demonstrated in murine model) |
| 184076 | Bid | 21552281 | Bid is a critical component of the NOD signalling pathway and is crucial for the peptidoglycan inflammation response. Bid deficient mice are unresponsive to local or systemic exposure to NOD agonist or their protective effects in experimental colitis. |

| | | | |
|---|---|---|---|
| 1050 | BID | 21552281 | BID is a critical component of the NOD signalling pathway and is crucial for the peptidoglycan inflammation response. BID deficient mice are unresponsive to local or systemic exposure to NOD agonist or their protective effects in experimental colitis. (Demonstrated in murine model) |
| 197807 | Thbs1 | 21573017 | Thbs1 expression is associated with decreased phagocytosis and bacterial clearance, resulting in increased peritoneal inflammation and mortality from sepsis. |
| 5926 | THBS1 | 21573017 | THBS1 expression is associated with decreased phagocytosis and bacterial clearance, resulting in increased peritoneal inflammation and mortality from sepsis. (Demonstrated in murine model) |
| 208049 | Tecpr1 | 21575909 | Tecpr1 activity is necessary for efficient autophagic targeting of bacteria and the suppression of Shigella intracellular replication. |
| 28493 | TECPR1 | 21575909 | TECPR1 activity is necessary for efficient autophagic targeting of bacteria and the suppression of Shigella intracellular replication. (Demonstrated in murine model) |
| 94677 | HMGN2 | 21518253 | HMGN2 acts as a positive modulator of NF-kB signalling to promote LPS-induced beta-defensin expression. HMGN2 prolongs the retention time and enhances the accumulation of NF-kB p65 and synergistically bind to the DEFB4A (HBD-2 ) promoter to induce expression. |
| 76333 | CXCR3 | 21518789 | CXCR3 expression on recruited peritoneal macrophages and granulocytes increases following sepsis, and deletion of CXCR3 significantly increases mortality to a septic challenge in neonatal mice. (Demonstrated in murine model) |
| 136255 | Map3k5 | 21515258 | Map3k5 is required for LPS-induced activation of p38, which is a crucial determinant of the production of pro-inflammatory cytokines in endotoxemia. |
| 96934 | MAP3K5 | 21515258 | MAP3K5 is required for LPS-induced activation of p38, which is a crucial determinant of the production of pro-inflammatory cytokines in endotoxemia. (Demonstrated in murine model) |
| 89226 | PTGES | 21497116 | PTGES (prostaglandin E2) is produced by LPS-primed macrophages upon treatment with silica crystal and aluminum salts, and is important for the production of IgE in Th2 cells. (Demonstrated in murine model) |

| | | | |
|---|---|---|---|
| 212642 | Grn | 21497117 | Grn is an essential secreted cofactor that potentiates Tlr9-driven response to CpG olignonucletides by binding directly to CpG oligos and Tlr9. Grn deficient murine macrophages showed impaired delivery of CpG oligos to endolysosomal compartments and reduced response to CpG. |
| 53782 | GRN | 21497117 | GRN is an essential secreted cofactor that potentiates TLR9-driven response to CpG olignonucletides by binding directly to CpG oligos and TLR9. GRN deficient murine macrophages showed impaired delivery of CpG oligos to endolysosomal compartments and reduced response to CpG. (Demonstrated in murine model) |
| 165921 | Tlr6 | 21482737 | Tlr2::Tlr6 synergistically interacts with Tlr9 in lung epithelium to induce rapid pathogen killing, and can be used as a therapeutic target to treat otherwise lethal pneumonia. |
| 209330 | Cd46 | 10233938 | Cd46 enhances nitric oxide production in mouse macrophages in response to measles virus infection in the presence of gamma interferon. |
| 209330 | Cd46 | 21488871 | Cd46 is expressed ubiquitously and functions as a co-factor in the factor I-mediated proteolytic cleavage of C3b and C4b. Cd46 has a vital role in preventing complement deposition on host tissue, and the resulting auto-immunity. |
| 106372 | CD46 | 20087059 | CD46 is a cell surface pathogen receptor that induces autophagy upon pathogen recognition and this CD46-dependent autophagy is critical for early control of the infection. |
| 106372 | CD46 | 15919905 | CD46 is a ubiquitously expressed membrane protein that regulates complement activation, as a cellular attachment receptor of several pathogens, including measles virus, Neisseria gonorrhea, adenovirus and human herpesvirus 6. |
| 106372 | CD46 | 18573902 | CD46 enhances bacterial survival and represents a novel pathogenic mechanism that contributes to the severity of group A streptococcal disease. |
| 106372 | CD46 | 16888016 | CD46 acts as a human epithelial cell receptor for internalization of opsonized uropathogenic Escherichia coli. |
| 106372 | CD46 | 16087667 | CD46 plays a key role in tailoring innate immune recognition of apoptotic and necrotic cells, whereby the complement innate immune system is using two synergistic strategies with the recognition of altered self-nucleic acids (NA) and missing self-CD46 signals to instruct and tailor the efficient removal of apoptotic and necrotic cells in immunoprivileged sites. |
| 106372 | CD46 | 21488871 | CD46 is expressed ubiquitously and functions as a co-factor in the factor I-mediated proteolytic cleavage of C3b and C4b. CD46 has a vital role in preventing complement deposition on host tissue, and the resulting auto-immunity. |

| | | | |
|---|---|---|---|
| 91827 | FCN1 | 20375620 | FCN1, as well as ficolins FCN2 and FCN3, in serum are associated with MBL-associated serine protease (MASP) to form a complex and this complex binds to carbohydrates present on the surface of a variety of Gram-positive and Gram-negative bacteria through ficolin, initiating complement activation via the lectin pathway. |
| 91827 | FCN1 | 16116205 | FCN1, like its family members, functions as a recognition molecule of the lectin complement pathway and plays an important role in innate immunity. |
| 91827 | FCN1 | 21490156 | FCN3 and PTX3 are soluble oligomeric pattern-recognition molecules that interact with each other and act synergistically to activate the lectin complement pathway. |
| 190792 | Nr1h3 | 21492741 | Nr1h3 repressed Irf3- or Irf7-induced transactivation of the interferon-beta promoter and NDV infection further potentiated the repressive effect in dendritic cells. |
| 42877 | NR1H3 | 21492741 | NR1H3 repressed IRF3- or IRF7-induced transactivation of the interferon-beta promoter and NDV infection further potentiated the repressive effect in dendritic cells. (Demonstrated in murine model) |
| 146207 | Nr4a3 | 21492741 | Nr4a3 repressed Irf3- or Irf7-induced transactivation of the interferon-beta promoter and NDV infection further potentiated the repressive effect in dendritic cells. |
| 78601 | NR4A3 | 21492741 | NR4A3 repressed IRF3- or IRF7-induced transactivation of the interferon-beta promoter and NDV infection further potentiated the repressive effect in dendritic cells. (Demonstrated in murine model) |
| 170861 | H2-Ab1 | 21441935 | H2-Ab1 is an intracellular MHC class II molecule that can act as an adaptor to promote the full activation of the TLR-triggered innate immune response. |
| 170982 | H2-Aa | 21441935 | H2-Aa is an intracellular MHC class II molecule that can act as an adaptor to promote the full activation of the TLR-triggered innate immune response. |
| 209605 | Srxn1 | 21466852 | Srxn1 is transcriptionally regulated by Nfe2l2 in immunostimulated primary macrophages that produce both reactive oxygen species and nitric oxide. The nitric oxide/Nfe2l2/Srxn1 pathway participates in the maintenance of redox homeostasis in cytokine-activated macrophages and other inflammatory settings. |
| 38297 | SRXN1 | 21466852 | SRXN1 is transcriptionally regulated by NFE2L2 in immunostimulated primary macrophages that produce both reactive oxygen species and nitric oxide. The nitric oxide/NFE2L2/SRXN1 pathway participates in the maintenance of redox homeostasis in cytokine-activated macrophages and other inflammatory settings. (Demonstrated in murine model) |

| | | | |
|---|---|---|---|
| 207909 | Gp2 | 21468225 | Gp2 on M cells (specialized epithelial antigen-transporting cells) functions as an uptake receptor for a subset of commensal and pathogenic bacteria. Gp2 interacts with type 1 pilus of gram-negative enterobacilli such as E. coli and Salmonella enterica. |
| 18165 | GP2 | 21468225 | GP2 on M cells (specialized epithelial antigen-transporting cells) functions as an uptake receptor for a subset of commensal and pathogenic bacteria. GP2 interacts with type 1 pilus of gram-negative enterobacilli such as E. coli and Salmonella enterica. (Demonstrated in murine model) |
| 130745 | Ripk2 | 21236705 | Ripk2 is a downstream adaptor molecule in the Nod1/2 signalling pathway and is important for the progression and pathogenesis of experimental autoimmune encephalomyelitis (animal model of multiple sclerosis). Ripk2 was found to be critical for the activation of CNS-infiltrating dendritic cells. |
| 130745 | Ripk2 | 21469090 | Ripk2 is essential for Nod1 and Nod2-signalling upon recognition of bacterial peptidoglycan. Ripk2 is crucial for inflammatory cytokine secretion, activation and recruitment of macrophage and neutrophils as well as the capacity to activate the adaptive immune response. |
| 129289 | Hrh4 | 21469095 | Hrh4 is a histamine receptor that can mediate cytokine production (e.g. Il6) from mast cells. Hrh4 is also able to synergize with other inflammatory signals, such as LPS, to potentiate cytokine production and contribute to inflammation. |
| 1754 | HRH4 | 21469095 | HRH4 is a histamine receptor that can mediate cytokine production (e.g. IL6) from mast cells. HRH4 is also able to synergize with other inflammatory signals, such as LPS, to potentiate cytokine production and contribute to inflammation. (Demonstrated in murine model) |
| 136615 | Plg | 21464960 | Plg is a serum protein that interacts with B. anthracis spores and cleaves complement 3 molecules, resulting in a decrease in macrophage phagocytosis. (Demonstrated in human) |
| 98749 | PLG | 21464960 | PLG is a serum protein that interacts with B. anthracis spores and cleaves complement 3 molecules, resulting in a decrease in macrophage phagocytosis. |
| 204473 | Ccl2 | 21458307 | Ccl2 is secreted by bone marrow mesenchymal stem cells in response to circulating TLR ligands or bacterial infection, which then induces monocyte trafficking into the bloodstream. |
| 40504 | CCL2 | 21458307 | CCL2 is secreted by bone marrow mesenchymal stem cells in response to circulating TLR ligands or bacterial infection, which then induces monocyte trafficking into the bloodstream. (Demonstrated in murine model) |

| | | | |
|---|---|---|---|
| 200174 | Impdh2 | 21460227 | Impdh2 proteins are rapidly recruited to the lipid raft of monocytes after lipopeptide stimulation, and play an essential role in the negative regulation of Tlr2 signalling by modulating PI3K activity. (Demonstrated in human) |
| 34157 | IMPDH2 | 21460227 | IMPDH2 proteins are rapidly recruited to the lipid raft of monocytes after lipopeptide stimulation, and play an essential role in the negative regulation of TLR2 signalling by modulating PI3K activity. |
| 198256 | C1qc | 21450789 | C1qa :: C1qb :: C1qc (C1q) is a versatile innate immune molecule that recognizes an array of self, non-self and altered-self ligands. The broad-spectrum of ligand specificity is facilitated by the modular organization of the heterotrimeric globular region and its ability to change its confirmation. |
| 93737 | C1QC | 15207504 | C1QC is the C-chain of the C1Q recognition subunit of Complement component 1 (C1), and acts a multimolecular protease that triggers the classical pathway of complement and has a major role in the host defence against pathogens. |
| 93737 | C1QC | 20381531 | C1Q is involved in the modulation of various immune cells such as dendritic cells, platelets, microglia cells and lymphocytes. C1Q has roles in clearance of apoptotic cells as well as a range of cell processes such as differentiation, chemotaxis, aggregation and adhesion, and pathogenesis of neurodegenerative diseases. |
| 93737 | C1QC | 21450789 | C1QA :: C1QB :: C1QC (C1Q) is a versatile innate immune molecule that recognizes an array of self, non-self and altered-self ligands. The broad-spectrum of ligand specificity is facilitated by the modular organization of the heterotrimeric globular region and its ability to change its confirmation. |
| 198224 | C1qb | 21450789 | C1qa :: C1qb :: C1qc (C1q) is a versatile innate immune molecule that recognizes an array of self, non-self and altered-self ligands. The broad-spectrum of ligand specificity is facilitated by the modular organization of the heterotrimeric globular region and its ability to change its confirmation. |
| 93747 | C1QB | 15207504 | C1QB is the B-chain of the C1Q, the recognition subunit of Complement component 1 (C1), is a multimolecular protease that triggers the classical pathway of complement and has a major role in the host defence against pathogens. |
| 93747 | C1QB | 20381531 | C1Q is involved in the modulation of various immune cells such as dendritic cells, platelets, microglia cells and lymphocytes. C1Q has roles in clearance of apoptotic cells as well as a range of cell processes such as differentiation, chemotaxis, aggregation and adhesion, and pathogenesis of neurodegenerative diseases. |

| | | | |
|---|---|---|---|
| 93747 | C1QB | 21450789 | C1QA :: C1QB :: C1QC (C1Q) is a versatile innate immune molecule that recognizes an array of self, non-self and altered-self ligands. The broad-spectrum of ligand specificity is facilitated by the modular organization of the heterotrimeric globular region and its ability to change its confirmation. |
| 198277 | C1qa | 21450789 | C1qa :: C1qb :: C1qc (C1q) is a versatile innate immune molecule that recognizes an array of self, non-self and altered-self ligands. The broad-spectrum of ligand specificity is facilitated by the modular organization of the heterotrimeric globular region and its ability to change its confirmation. |
| 93718 | C1QA | 15207504 | C1QA is the A-chain of the C1Q recognition subunit of Complement component 1 (C1), and acts a multimolecular protease that triggers the classical pathway of complement and has a major role in the host defence against pathogens. |
| 93718 | C1QA | 20381531 | C1Q is involved in the modulation of various immune cells such as dendritic cells, platelets, microglia cells and lymphocytes. C1Q has roles in clearance of apoptotic cells as well as a range of cell processes such as differentiation, chemotaxis, aggregation and adhesion, and pathogenesis of neurodegenerative diseases. |
| 93718 | C1QA | 21450789 | C1QA :: C1QB :: C1QC (C1Q) is a versatile innate immune molecule that recognizes an array of self, non-self and altered-self ligands. The broad-spectrum of ligand specificity is facilitated by the modular organization of the heterotrimeric globular region and its ability to change its confirmation. |
| 205741 | Ccbp2 | 21450903 | Ccbp2 is a marker for innate-like B cells, which are a heterogeneous collection of cells that control infection and suppress inflammation. Ccbp2 act as a chemokine scavenger receptor by internalizing chemokines without inducing calcium fluxes or chemotaxis in innate B cells. |
| 28131 | CCBP2 | 21450903 | CCBP2 is a marker for innate-like B cells, which are a heterogeneous collection of cells that control infection and suppress inflammation. CCBP2 act as a chemokine scavenger receptor by internalizing chemokines without inducing calcium fluxes or chemotaxis in innate B cells. (Demonstrated in murine model) |
| 140708 | Nfkbia | 21454695 | Nfkbia degradation occurs through the TNF-stimulated formation of autophagosomes in epithelial cells, which results in the prolonged activation of NFKB activity. |
| 141324 | Xiap | 21447281 | Xiap facilitates ubiquitin-dependent signalling activated by pattern recognition receptors, such as TLR and NOD, to mediate the activation of NFKB transcription. |
| 85142 | XIAP | 18769721 | XIAP regulates cytosol-specific innate immunity to Listeria infection. |

| | | | |
|---|---|---|---|
| 85142 | XIAP | 19667203 | XIAP interacts with NOD1 and NOD2 and mediates NOD signalling via interaction with RIPK2. |
| 85142 | XIAP | 19531477 | XIAP and its E3 ligase activity promote transforming growth factor-{beta}-mediated NF-kappaB activation during breast cancer progression. |
| 85142 | XIAP | 20406824 | X-linked inhibitor of apoptosis protein (XIAP) in a complex with survivin, a physiological substrate for granzyme M (GzmM), act to inhibit caspase activation. |
| 85142 | XIAP | 21447281 | XIAP facilitates ubiquitin-dependent signalling activated by pattern recognition receptors, such as TLR and NOD, to mediate the activation of NFKB transcription. |
| 133177 | Birc3 | 21447281 | Birc3 facilitates ubiquitin-dependent signalling activated by pattern recognition receptors, such as TLR and NOD, to mediate the activation of NFKB transcription. |
| 133125 | Birc2 | 21447281 | Birc2 facilitates ubiquitin-dependent signalling activated by pattern recognition receptors, such as TLR and NOD, to mediate the activation of NFKB transcription. |
| 69075 | BIRC2 | 18697935 | BIRC2 regulates TNF-alpha(TNF)-mediated NF-kappaB activation by binding to TNFRSF1A (TNF receptor 1). |
| 69075 | BIRC2 | 17220297 | BIRC2 is the ubiquitin protein ligase for ASK1 ubiquitination, a protein that plays an essential role in tumour necrosis factor alpha (TNF-alpha)-induced mitogen-activated protein kinase signalling. BIRC2 is also responsible for regulating the duration of TNF signalling in primary cells expressing TNFR2. |
| 69075 | BIRC2 | 19464198 | BIRC2 and BIRC3 are required for innate immunity signalling by the pattern recognition receptors NOD1 and NOD2. |
| 69075 | BIRC2 | 20458734 | BIRC2 plays a role in lipopolysaccharide (LPS)-induced autophagy in vascular endothelial cells (VECs). |
| 69075 | BIRC2 | 21447281 | BIRC2 facilitates ubiquitin-dependent signalling activated by pattern recognition receptors, such as TLR and NOD, to mediate the activation of NFKB transcription. |
| 175332 | Hspa1a | 21448922 | Hspa1a is secreted into the extracellular space during exercise-induced stress and increases the intracellular levels of cAMP, which acts as an "intracellular danger signal" to activate neutrophils. (Demonstrated in human) |
| 251732 | Pglyrp2 | 21439073 | Pglyrp2 is a member of the Peptidoglycan Recognition Proteins (PGRP) family and recognizes peptidoglycan, a structural component of bacterial cell walls, as a part of innate immune response against infections. |
| 34754 | PGLYRP2 | 20418257 | PGLYRP2 is a secreted innate immunity protein that is expressed in the liver and is conserved from insects to mammals, it recognizes bacterial peptidoglycan, and functions in antibacterial immunity and inflammation. |

| | | | |
|---|---|---|---|
| 34754 | PGLYRP2 | 21439073 | PGLYRP2 is a member of the Peptidoglycan Recognition Proteins (PGRP) family and recognizes peptidoglycan, a structural component of bacterial cell walls, as a part of innate immune response against infections. |
| 137962 | Defb3 | 21442129 | Defb3 expression is inhibited in keratinocytes under high glucose conditions, which in turn contributed to the frequent occurrences of infection associated with diabetic wounds. (Demonstrated in rat model and in human) |
| 137529 | Raet1c | 21411527 | Raet1c expression on erythroblast surface is induced early after Friend virus inoculation, and is recognized by Klrk1 expressed on the NK cells to trigger cytotoxic activities. |
| 270707 | Raet1a | 21411527 | Raet1a expression on erythroblast surface is induced early after Friend virus inoculation, and is recognized by Klrk1 expressed on the NK cells to trigger cytotoxic activities. |
| 205690 | Crp | 21415385 | Crp promotes the differentiation of human monocytes toward a pro-inflammatory M1 macrophage phenotype. In addition, Crp treatment of M2 macrophages induced the expression of pro-inflammatory genes and a M1 phenotype. (Demonstrated in human) |
| 103911 | CRP | 16751408 | The major acute phase protein in humans that interacts with CFH of the alternative pathway of complement and C4BP of the classical complement pathway, limiting excessive complement activation |
| 103911 | CRP | 21383176 | CRP induces FCAR surface expression, phagocytosis, and TNF secretion in neutrophils. In addition, CRP physically interacts with FCAR, and induces ERK phosphorylation, cytokine production, and degranulation in mast cells. |
| 103911 | CRP | 21415385 | CRP promotes the differentiation of human monocytes toward a pro-inflammatory M1 macrophage phenotype. In addition, CRP treatment of M2 macrophages induced the expression of pro-inflammatory genes and a M1 phenotype. |
| 165744 | Lgmn | 21402738 | Lgmn is an asparagine endopeptidase that removes the majority of the Tlr9 ectodomain, and this catalytic cleavage is required for Tlr9 endolysosome signalling in response to DNA. |
| 17093 | LGMN | 21402738 | LGMN is an asparagine endopeptidase that removes the majority of the TLR9 ectodomain, and this catalytic cleavage is required for TLR9 endolysosome signalling in response to DNA. (Demonstrated in murine model) |
| 9448 | SREBF2 | 21408089 | SREBF2 is a key transcriptional regulator of sterol biosynthesis in lipid metabolism, and SREBP2 protein levels in macrophages are negatively regulated by type I interferon signalling during viral infection. (Demonstrated in murine model) |

| | | | |
|---|---|---|---|
| 133028 | Cops5 | 21403132 | Cops5 is required for activation of pro-inflammatory kinases, p38 and ErK, and the down-regulation of Nfe2l2 gene targets. Mice with Cops5 deficiency have lower mortality in polymicrobial sepsis. |
| 24191 | COPS5 | 21403132 | COPS5 is required for the activation of pro-inflammatory kinases, p38 and ErK, and the down-regulation of NFE2L2 gene targets. Mice with Cops5 deficiency have lower mortality in polymicrobial sepsis. (Demonstrated in murine model) |
| 57021 | RELB | 16951372 | RELB is a NF-kappaB subunit that participates in endotoxin tolerance by repressing pro-inflammatory gene expression. |
| 57021 | RELB | 17823304 | RELB mediates transcription of chemokines like IL8 via activation of AHR and Protein kinase A, and its expression is inhibited by Vitamin D3 analog in DCs. |
| 57021 | RELB | 12657634 | RELB acts as both transcription factor as well as a repressor of NF-kappaB gene expression by forming heterodimers with NFKB1 (p50 subunit) and NFKB2 (p100 subunit) or inhibiting RELA DNA binding activity, respectively. |
| 57021 | RELB | 19020113 | RELB sustains NFKBIA (IkappaB alpha) expression during endotoxin tolerance, RelB transcription activation requires binding to the (IkappaB alpha) proximal promoter along with NFKB1 (p50), and is associated with an apparent dimer exchange with RELA (p65). |
| 57021 | RELB | 19020113 | RELB functions as a dual transcription regulator during LPS tolerance and human severe systemic inflammation (SSI) by activating and repressing innate immunity genes. |
| 57021 | RELB | 21419662 | RELB is required for IL17A production in T cell in response to bacterial infection. RELB deficient T cells resulted in a diminished innate immune response to E. coli infection. (Demonstrated in murine model) |
| 144745 | Xbp1 | 20351694 | Xbp1 transcription factor regulates innate immune responses in macrophages via Tlr2 activation and its deficiency results in a much greater bacterial burden in mice infected with the Tlr2-activating human intracellular pathogen Francisella tularensis. |
| 144745 | Xbp1 | 21400498 | Xbp1 is an important regulator in ER stress response and may function collaboratively with innate immunity to maintain cellular homeostasis. Xbp1 is essential in poly(I:C)-signalling and ER stress-amplified IFNB production in dendritic cells, and the over-expression of Xbp1 synergistically augments the poly(I:C)-induced inflammatory response. |
| 3430 | XBP1 | 20351694 | XBP1 transcription factor regulates innate immune responses in macrophages via TLR2 activation and its deficiency results in a much greater bacterial burden in mice infected with the TLR2-activating human intracellular pathogen Francisella tularensis. |

| | | | |
|---|---|---|---|
| 3430 | XBP1 | 20533428 | XBP1/ERN1 (IRE1)-mediated signalling plays roles in the coordination of metabolic and immune responses by acting as a regulatory hub, linking endoplasmic reticulum homeostasis with innate immunity and metabolism. |
| 3430 | XBP1 | 20660350 | XBP1 interacts with EP300 to augment IFN-beta induction via a cis-acting enhancer in macrophages under endoplasmic reticulum stress. |
| 3430 | XBP1 | 21400498 | XBP1 is an important regulator in ER stress response and may function collaboratively with innate immunity to maintain cellular homeostasis. XBP1 is essential in poly(I:C)-signalling and ER stress-amplified IFNB production in dendritic cells, and the over-expression of XBP1 synergistically augments the poly(I:C)-induced inflammatory response. (Demonstrated in murine model) |
| 75722 | NFIL3 | 19749763 | NFIL3 is essential for generation of the natural killer (NK) cell lineage. |
| 75722 | NFIL3 | 21383239 | NFIL3 is an IL12B transcriptional inhibitor in macrophages. Interactions of macrophages with the enteric microbiota induce NFIL3 to limit their inflammatory capacity. (Demonstrated in murine model) |
| 158106 | Mbl2 | 20375621 | Mbl2 interacts with Fcna and fibrinogen/fibrin to augment the lectin complement pathway, which collaborates with the coagulation system in the first-line host defence against pathogens under conditions such as injury and inflammation. |
| 158106 | Mbl2 | 21383675 | Mbl2 treatment inhibits the activity of NFKB and consequently suppresses the production Tnf and Il12 production in human monocytes stimulated with LPS. In addition, Mbl2 was found to bind to Tlr4 and attenuate the binding of LPS to cell surfaces. |
| 74690 | MBL2 | 16105157 | MBL2 is a major recognition molecule of the lectin pathway of complement. |
| 74690 | MBL2 | 16911830 | MBL2 binds directly to a wide range of repeating sugar moieties on microbial surfaces via its lectin domain, resulting in neutralization and opsonization. |
| 74690 | MBL2 | 19840833 | MBL2 binds toll-like receptor 4 (TLR4) and modulates cellular responses by altering signals through TLRs. |
| 74690 | MBL2 | 21383675 | MBL2 treatment inhibits the activity of NFKB and consequently suppresses the production TNF and IL12 production in human monocytes stimulated with LPS. In addition, MBL2 was found to bind to TLR4 and attenuate the binding of LPS to cell surfaces. |
| 135484 | Ccr6 | 21042003 | Ccr6 mobilizes TCR-alpha/beta+, Ccr6+ innate and adaptive effector T cells in the airway in response to mycobacterial infection. Ccr6 is not required for induction of the adaptive antimycobacterial response |

| | | | |
|---|---|---|---|
| 135484 | Ccr6 | 21376174 | Ccr6 is a chemokine receptor that only binds to a single chemokine ligand, Ccl20. Ccr6 is an important receptor that is involved in regulating mucosal immunity by mediating the recruitment of dendritic cells and APCs to the sites of epithelial inflammation. |
| 99015 | CCR6 | 21042003 | CCR6 mobilizes TCR-alpha/beta+, CCR6+ innate and adaptive effector T cells in the airway in response to mycobacterial infection. CCR6 is not required for induction of the adaptive antimycobacterial response |
| 99015 | CCR6 | 21376174 | CCR6 is a chemokine receptor that only binds to a single chemokine ligand, CCL20. CCR6 is an important receptor that is involved in regulating mucosal immunity by mediating the recruitment of dendritic cells and APCs to the sites of epithelial inflammation. |
| 150133 | Trp63 | 21367858 | Trp63 is a crucial regulator downstream of Tlr3 in Poly(I:C)-induced signalling. Trp63 activates the signalling of both extrinsic and intrinsic apoptosis pathways in endothelial cells through death receptors and mitochondria. |
| 69487 | TP63 | 21367858 | TP63 is a crucial regulator downstream of TLR3 in Poly(I:C)-induced signalling. TP63 activates the signalling of both extrinsic and intrinsic apoptosis pathways in endothelial cells through death receptors and mitochondria. |
| 89258 | MTOR | 18758466 | MTOR is the signalling molecule involved in TLR-mediated IFN-alpha production by plasmacytoid dendritic cells (pDCs). |
| 89258 | MTOR | 18924132 | MTOR is an indispensable component of pathogen recognition receptor (PRR) signal pathways that orchestrates the defence program of innate immune cells. |
| 89258 | MTOR | 19131641 | MTOR plays a central role in cell growth and cellular responses to metabolic stress and its activation is essential in TLR2- and TLR4-induced neutrophil activation, as well as in the development and severity of acute lung injury. |
| 89258 | MTOR | 19331815 | MTOR signalling is one major mechanism in a tightly regulated network of intracellular signal pathways including the JAK/STAT system to regulate invasion in human trophoblast cells by secretion of enzymes that remodel the extra-cellular matrix (ECM) such as MMP2, MMP9, PLAU and SERPINE1. |
| 89258 | MTOR | 21368289 | Inhibition of mTOR blocks the anti-inflammatory potency of glucocorticoids both in human monocytes and myeloid dendritic cells. |
| 207019 | Cltc | 21364927 | Cltc functions as a built-in molecular brake that ensures a tight control of basal NFKB activation and gene expression in un-stimulated cells. Defects in Cltc expression could potentially lead to chronic inflammation disorder. |

| | | | |
|---|---|---|---|
| 61767 | CLTC | 21364927 | CLTC functions as a built-in molecular brake that ensures a tight control of basal NFKB activation and gene expression in un-stimulated cells. Defects in CLTC expression could potentially lead to chronic inflammation disorder. |
| 203973 | Fgf7 | 21343299 | Fgf7 enhances alveolar host defence through GM-CSF-stimulated macrophage activation. Intrapulmonary Fgf7 injection enhanced the clearance of E. coli or P. aeruginosa via the augmented recruitment, phagocytic activity and oxidant responses of macrophages. |
| 11473 | FGF7 | 21343299 | FGF7 enhances alveolar host defence through GM-CSF-stimulated macrophage activation. Intrapulmonary FGF7 injection enhanced the clearance of E. coli or P. aeruginosa via the augmented recruitment, phagocytic activity and oxidant responses of macrophages. (Demonstrated in murine model) |
| 31974 | NFKB1 | 14593105 | NFKB1 (p50) is a distinct form of NF-kappaB that interacts with STAT3 and cooperates with STAT3 bound to GAS sites. |
| 31974 | NFKB1 | 8152812 | NFKB1 is a subunit of the NF-kappaB transcriptional regulator complex. NF-kappaB is an inducible transcription factor that regulates the expression of numerous genes involved in immune and inflammation responses and in cellular growth control. |
| 31974 | NFKB1 | 21343618 | NFKB1 enforces specificity of cellular response to pathogens by binding to a subset of IRE sequences in IFN-inducible genes. NFKB1 deficiency results in the inappropriate production of IFNB in response to bacterial DNA sensed by TLR9. (Demonstrated in murine model) |
| 162142 | Cebpe | 21326902 | Cebpe is a member of the CCAAT enhancer binding protein family and is a transcriptional factor regulating genes in innate immunity and inflammation. The activities of CEBP are regulated via methylation of arginine and lysine side chains. |
| 216094 | oleculeID 2160 | 21326902 | Cebpd is a member of the CCAAT enhancer binding protein family and is a transcriptional factor regulating genes in innate immunity and inflammation. The activities of CEBP are regulated via methylation of arginine and lysine side chains. |
| 3098 | CEBPE | 21326902 | CEBPE is a member of the CCAAT enhancer binding protein family and is a transcriptional factor regulating genes in innate immunity and inflammation. The activities of CEBP are regulated via methylation of arginine and lysine side chains. |
| 281563 | CEBPD | 20829351 | CEBPD is involved in TLR8 mediated innate immune response by binding to C/EBP cis-acting elements within the TLR8 promoter increasing its transcriptional activity. |

| | | | |
|---|---|---|---|
| 281563 | CEBPD | 21326902 | CEBPD is a member of the CCAAT enhancer binding protein family and is a transcriptional factor regulating genes in innate immunity and inflammation. The activities of CEBP are regulated via methylation of arginine and lysine side chains. |
| 145441 | Coro2a | 21331046 | Coro2a mediates transcriptional activation of TLR-responsive genes through a Coro2a-actin-dependent mechanism to remove nuclear receptor co-repressor (NCoR) complexes from the promoters of target genes. |
| 78227 | CORO2A | 21331046 | CORO2A mediates transcriptional activation of TLR-responsive genes through a CORO2A-actin-dependent mechanism to remove nuclear receptor co-repressor (NCoR) complexes from the promoters of target genes. |
| 168890 | Hmox1 | 21307647 | Hmox1, a downstream signalling molecule in the Tlr4 pathway, is necessary for LPS-induced autophagy signalling in macrophages. |
| 5374 | HMOX1 | 21307647 | HMOX1, a downstream signalling molecule in the TLR4 pathway, is necessary for LPS-induced autophagy signalling in macrophages. |
| 178161 | Irak2 | 21291324 | Irak2 regulates cytokine and chemokine mRNA stability and translation in response to LPS stimulation in macrophages. The kinase activity of Irak2 is required for the optimal activation of mitogen activated protein kinase signalling, which regulates cytokine/chemokine production at post-transcriptional levels. |
| 56118 | TBKBP1 | 17568778 | A novel component of innate antiviral immunity, it shares a TBK1-binding domain with AZI2 and TANK |
| 61301 | SIAH2 | 12411493 | SIAH2 decreases TNF-alpha dependent induction of MAPK8 (JNK) activity and transcriptional activation of NF kappa B by mediating ubiquitination of TRAF2 under stress conditions. |
| 62489 | CD37 | 17182550 | CD37 is important for CLEC7A (Dectin-1) stabilization in APC membranes and controls Dectin-1-mediated IL6 production. |
| 64443 | SLC15A4 | 19570976 | SLC15A4 is an oligopeptide transporter expressed in early endosomes which is involved in transportation of NOD1 ligands. |
| 64443 | SLC15A4 | 21045126 | SLC15A4, a peptide/histidine transporter in organelle trafficking, is required for the production of pro-inflammatory cytokines in plasmacytoid dendritic cells upon recognition of viral nucleic acids by endosomal TLR7 or TLR9. |
| 65720 | SYP | 7559603 | SYP is a protein tyrosine phosphatase that associates with IL6ST and JAK2 in IL-11 signal transduction pathway. |
| 66030 | MERTK | 18083102 | MERTK is a tyrosine protein kinase, it acts with TYRO3 and AXL as pleiotropic inhibitor of the innate immune response in DCs. |
| 66516 | MAP2K6 | 9841871 | MAP2K6 activates MAPK14 (p38) MAP kinases. |
| 67727 | NLRP12 | 17418609 | NLRP12 is a negative regulator of the NF-Î°B response in monocytes. |

| | | | |
|---|---|---|---|
| 67727 | NLRP12 | 18280719 | NLRP12 negatively regulates non-canonical NF-Î°B pathway by inducing NIK degradation. |
| 67727 | NLRP12 | 16203735 | NLRP12 is an antagonist of toll-like receptor-, tumour necrosis factor alpha-, and Mycobacterium tuberculosis-induced pro-inflammatory signals. |
| 69118 | MMP7 | 19181662 | MMP7 is responsible for cleavage of several mouse (Defb1 and Defb2) and human (DEFA1, DEFB1, DEFB4) defensins from pro- to active forms. |
| 69319 | MMP12 | 19536155 | MMP12 has antimicrobial activity where upon bacterial infection, MMP12 is mobilized to macrophage phagolysosomes and adheres to bacterial cell walls where it disrupts cellular membranes resulting in bacterial death. |
| 69883 | NLRP2 | 18056399 | NLRP2 is an inhibitor of the NF-kappaB pathway. |
| 69883 | NLRP2 | 15030775 | NLRP2 associates with PYCARD, RIPK2, and CASP1, forming an inflammasome with high proIL-1beta-processing activity. |
| 71104 | NLRP9 | 18648497 | NLRP9 is a part of the NLRP (Nucleotide-binding oligomerization domain, Leucine rich Repeat and Pyrin domain containing) family, has a role in apoptosis and inflammation. |
| 71154 | NLRP11 | 18648497 | NLRP11 is a part of the NLRP (Nucleotide-binding oligomerization domain, Leucine rich Repeat and Pyrin domain containing) family, has a role in apoptosis and inflammation. |
| 71230 | NLRP13 | 18648497 | NLRP13 is a part of the NLRP (Nucleotide-binding oligomerization domain, Leucine rich Repeat and Pyrin domain containing) family, has a role in apoptosis and inflammation. |
| 71259 | NLRP8 | 18648497 | NLRP8 is a part of the NLRP (Nucleotide-binding oligomerization domain, Leucine rich Repeat and Pyrin domain containing) family, has a role in apoptosis and inflammation. |
| 71285 | NLRP5 | 18648497 | NLRP5 is a part of the NLRP (Nucleotide-binding oligomerization domain, Leucine rich Repeat and Pyrin domain containing) family, has a role in apoptosis and inflammation. |
| 83912 | PSMA7 | 19734229 | PSMA7 physically associates with and is involved in the stability of MAVS, which thus provides negative regulation of the innate antiviral response against infection by RNA viruses. |
| 84584 | TNFRSF18 | 19760073 | TNFRSF18 is expressed in several cells and tissues, including T and Natural Killer (NK) cells and is activated by its ligand, GITRL, mainly expressed on Antigen Presenting Cells (APCs) and endothelial cells and is a modulator of immune response and inflammation. |
| 86437 | CDK9 | 18728388 | CDK9 heterodimerizes with CCNT1 to form the positive transcriptional elongation factor b (P-TEFb) and plays a role in the activation of a subset of NF-kappaB dependent targets. |

| | | | |
|---|---|---|---|
| 95161 | PTAFR | 11309383 | PTAFR is involved in G-protein-independent activation of TYK2. |
| 99144 | C8A | 12220191 | C8A is the alpha subunit of complement factor 8 (C8) and is one of five components that interact to form the cytolytic membrane attack complex (MAC). |
| 104701 | SELE | 9006914 | SELE is upregulated by TNF alpha during inflammatory responses and activated by RELA, NFKB2, JUN and ATF2. |
| 125942 | C4A | 2650988 | C4A is one of two isotypes of the fourth complement component. |
| 125942 | C4A | 15476920 | C4A is a soluble complement anaphylatoxin that greatly controls the local pro-inflammatory response. |
| 151802 | Mbl1 | 20375621 | Mbl1 interacts with Fcna and fibrinogen/fibrin to augment the lectin complement pathway, which collaborates with the coagulation system in the first-line host defence against pathogens under conditions such as injury and inflammation. |
| 296590 | DEFA3 | 19024344 | DEFA3 transcribes a large amount cationic alpha-defensin peptides, HNP-3, in human neutrophils, these alpha-defensins have multiple functions in the immune system. |
| 205704 | Apcs | 21278351 | Apcs is a key negative regulator for innate immune responses to DNA and may be partly responsible for the insufficient immune responses after DNA vaccination in humans. Murine Apcs exhibited a similar, albeit very weak, activity. |
| 196803 | Cfh | 21285368 | Cfh, a complement regulatory factor, interacts with host cell surfaces as well as C3d part of C3b and plays a major in the distinguishing host from non-host surfaces during the alternatively activated complement pathway. |
| 1165 | PTPN2 | 14600148 | PTPN2 pays a novel role in the regulation of type 1 interferon-stimulated gene expression in cells previously desensitized to Type 1 interferons. |
| 1165 | PTPN2 | 11773439 | PTPN2 is a nuclear protein tyrosine phosphatase, a potential negative regulator of the PRL-mediated signalling pathway. |
| 2140 | IFNGR2 | 9001223 | IFNGR2 associates with JAK2 and this is required for IFN-gamma signalling. |
| 2140 | IFNGR2 | 16467883 | IFNRG2 complex is activated by STAT1, leading to the transcription of a significant portion of IFN-gamma induced genes, many of which are responsible for the induction of an apoptotic state in response to IFN-gamma. |
| 2768 | RCAN1 | 19716405 | RCAN1 (DSCR1) short isoform positively modulates IL-1R-mediated signalling pathways by regulating TOLLIP/IRAK1/TRAF6 complex formation. |

| | | | |
|---|---|---|---|
| 3195 | SMAD7 | 12589052 | Inhibitory SMAD7, a direct target gene for transforming growth factor-beta (TGF-beta), mediates TGF-beta1-induced apoptosis in several cell types and acts as a scaffolding protein to facilitate TAK1- and MKK3-mediated activation of p38. |
| 3195 | SMAD7 | 20171181 | SMAD7 acts as a critical mediator for effective TGF-beta1-mediated suppression of IL-1R/TLR signalling, by simultaneous binding to discrete regions of Pellino-1. |
| 3930 | MALT1 | 18192506 | MALT1 forms a complex with CARM1 and BCL10 to activate NF-kappaB. |
| 3930 | MALT1 | 20804738 | MALT1 is a paracaspase that has arginine-directed proteolytic activity. MALT1 cleaves TNFAIP3, a dual ubiquitin-editing enzyme involved in termination of NF-kappaB signalling, inducing cytosolic release of TNFAIP3 and dampening its inhibitory function. |
| 4231 | ABCG1 | 20395559 | ABCG1 regulates innate immunity in a tissue-selective manner and Abcg1(-/-) mice have an enhanced pulmonary host defence response driven predominantly by hematopoietic cells. |
| 4847 | SOCS6 | 14707129 | SOCS6 associates with KIT and regulates KIT receptor signalling and leads to MAPK activation. |
| 6178 | CSF2RB | 15988755 | CSF2RB (IL3RB) is tyrosine phosphorylated by the simultaneous activation of both JAK1 and JAK2 fusion proteins, but not either one alone. Phosphorylated CSF2RB then induces the activation of downstream signaling molecules, including STAT5, AKT, and MAPK, and the conferring of factor-independent growth to IL-3-dependent Ba/F3 cells |
| 6418 | IL2RB | 8700888 | IL2RB is phosphorylated by JAK1and different IL2RB tyrosines couple to at least two signalling pathways (JAK-STAT and SHC-coupled) and synergistically mediate IL2-induced proliferation. |
| 6418 | IL2RB | 8041779 | IL2RB physically associates with JAK1 suggesting that regulation of JAK1 may be linked to IL2 induced signal transduction. |
| 6696 | LGALS2 | 15356130 | LGASL2 binds to T cells in a beta-galactoside-specific manner and acts as pro-apoptotic effector for activated T cells. |
| 7865 | APOBEC3A | 20615867 | APOBEC3A has deaminase activity in monocytes and macrophages and this induces high levels of TC-specific deaminase activity in IFN-alpha signalling. |
| 8281 | MAP3K7IP1 | 15590691 | MAP3K7IP1 induces MAP3K7 autophosphorylation/activation and inhibits MAP3K7 interaction with the IKK signalosome. |
| 8281 | MAP3K7IP1 | 8638164 | MAP3K7IP1 functions as an activator of the MAP3K7 (TAK1) in TGF-beta signal transduction. |

| | | | |
|---|---|---|---|
| 8281 | MAP3K7IP1 | 14592977 | MAP3K&IP1 participates in a SAPK2a/p38alpha-mediated feedback control of MAP3K7, which not only limits the activation of SAPK2a/p38alpha but synchronizes its activity with other signalling pathways that lie downstream of MAP3K7 (JNK and IKK). |
| 8281 | MAP3K7IP1 | 16407200 | MAP3K7IP1 modulates intracellular localization of MAPK14 (p38) and downstream signalling. |
| 10142 | MLST8 | 18755269 | MLST8 regulates TNF-alpha-induced NF-kappaB signalling by directly inhibiting the activation of IkappaB kinase. |
| 10956 | TCEB2 | 9918119 | TCEB2 is a part of Elongin BC complex, is a component of a multiprotein SOCS1 complex that attenuates JAK/STAT signaling by binding to JAK2 and inhibiting JAK2 kinase and by interacting with SOCS box, the Elongin BC complex can increase expression of the SOCS1 protein by inhibiting its degradation. |
| 10956 | TCEB2 | 10051596 | TCEB2, as a part of the Elongin B and C complex, is bound by SOCS protein for proteasomal degradation. |
| 11729 | MEFV | 126461680 | MEFV inhibits the formation of CASP8, PYCARD and NLRC4 inflammasome formation by competing with CASP8 to bind to PYCARD. |
| 11729 | MEFV | 12615073 | MEFV inhibits binding of NLRP3 with PYCARD. |
| 11729 | MEFV | 17964261 | PYCARD-MEFV pyroptosome is induced by PSTPIP1 in a pyrin-dependent manner. |
| 11729 | MEFV | 17964261 | MEFV homodimerization is important for its ability to induce PYCARD oligomerization and CASP1 activation. |
| 11729 | MEFV | 14514692 | MEFV gene is bound constitutively by CEBPB, while RELA binds to the MEFV gene upon TNFA stimulation. |
| 13899 | PTK2B | 10228162 | PTK2B mediates the JAK-dependent activation of MAPK and STAT1 in interferon (IFN)-gamma, but not IFN-alpha signalling. |
| 13899 | PTK2B | 14963038 | PTK2B amplifies epidermal growth factor receptor (EGFR) and SRC-induced STAT3 activation, implicating PTK2B activation as a potential co-mediator in triggering STAT3-induced oncogenesis. |
| 15015 | MACR;C1QTN | 20739398 | C1q/TNF-related protein-3 (AMACR;C1QTNF3), a protein secreted by adipocytes (adipokine), inhibits three basic and common pro-inflammatory pathways involved in obesity and type 2 diabetes mellitus (adipo-inflammation) by acting as an endogenous LPS antagonist of the adipose tissue. |
| 15121 | SCARF1 | 19237602 | SCARF1 mediates host defence against Cryptococcus neoformans and Candida albicans through cytokine production and is required for macrophage binding to C. neoformans to control the infection in mice. |

| | | | |
|---|---|---|---|
| 15719 | PTPN6 | 9520455 | PTPN6 (SHP-1) normally functions to antagonize the IL-2 signal transduction pathway and human T-lymphotropic virus type I (HTLV-I) infection and oncogenic transformation can lead to loss of SHP-1 expression, resulting in constitutive activation of IL-2 regulated T cell responses. |
| 15719 | PTPN6 | 8524272 | PTPN6 reversibly associates with the IFN-alpha receptor complex upon IFN stimulation and selectively regulates distinct components of JAK/STAT signal transduction pathways. |
| 15719 | PTPN6 | 17079228 | PTPN6 specifically downregulates MAP3K7 (TAK1) through dephosphorylation, suppressing inflammatory responses via TAK1 signalling pathways. |
| 15719 | PTPN6 | 18391954 | PTPN6 promotes TLR- and RIG-I-activated production of type I interferon by inhibiting the kinase IRAK1. |
| 15719 | PTPN6 | 20145200 | PTPN6 tyrosine phosphatase activity plays a critical role in induction of IL2B production in macrophages in response to TLR ligands. |
| 16472 | CLEC4C | 20673884 | CLEC4C (BDCA-2) signalling inhibits TLR-9-agonist-induced plasmacytoid dendritic cell activation (through the inhibition of CD86 and CD40) and antigen presentation. |
| 16551 | SIGIRR | 12925853 | SIGIRR is a negative modulator of TLR-IL-1R signalling where it binds to the TLR-IL-1R signalling components in a ligand-dependent way. |
| 16551 | SIGIRR | 20060329 | SIGIRR controls Th17 cell expansion and effector function through the IL-1-induced mTOR signalling pathway. |
| 16551 | SIGIRR | 20130217 | SIGIRR regulates innate responses in differentiated human intestinal epithelial cells (IECs), modulating epithelial involvement in infectious and inflammatory bowel diseases. |
| 16551 | SIGIRR | 20364327 | SIGIRR can inhibit Toll-like receptor (TLR) 4, 5, and 9-mediated immune responses by attenuating production of the inflammatory mediators IL-6 and TNF-alpha and this attenuation was not the result of decreased expression of TLR4, 5 or 9, but rather a sequestration of MYD88 to the TLRs. |
| 16878 | HRAS | 12867418 | HRAS participates in CpG oligodeoxynucleotide signalling through association with TLR9 and promotion of IRAK1/TRAF6 complex formation in macrophages. |
| 16878 | HRAS | 11744690 | HRAS participates in the activation of MAPK1 by IL1A (IL-1) through association with IRAK1, IRAK2, TRAF6 and MAP3K7. |
| 16878 | HRAS | 20501842 | HRAS promotes virus spread by suppressing viral RNA-induced IFN-beta production through negative regulation of RIG-I signalling. |
| 17610 | C19orf29 | 20829348 | C19orf29 (Cactin) is a novel negative regulator of TLR signalling that targets the MHC class III protein I{kappa}B like (I{kappa}BL) and inhibits NFkappaB and IRF signalling pathways. |

| | | | |
|---|---|---|---|
| 17892 | C9 | 16189651 | C9, Complement factor nine, is one of five components that interact to form the cytolytic membrane attack complex (MAC), composed of a C5B-C8 complex attached to a transmembrane C9 oligomer. |
| 18183 | PIAS4 | 21199872 | PIAS4 is a member of the Protein Inhibitor of Activated STAT protein family, which regulate innate immune response by controlling transcription induced by TLR, RLR and JAK/STAT signalling pathways. PIAS4 specifically negatively regulate both IFN transcription and IFN stimulated gene expression through multiple mechanisms utilizing the function of different domains. |
| 177127 | Pias4 | 21199872 | Pias4 is a member of the Protein Inhibitor of Activated STAT protein family, which regulate innate immune response by controlling transcription induced by Tlr, Rlr and Jak/Stat signalling pathways. Pias4 specifically negatively regulate both Ifn transcription and Ifn stimulated gene expression through multiple mechanisms utilizing the function of different domains. |
| 18293 | MAP2K2 | 20837746 | MAP2K2 (MEK2)/PI3CD is a novel IFN-beta triggered signalling cascade that regulates secreted IL-1Ra (sIL-1Ra) expression in monocytes and this provides a rationale for an alternative, interferon (IFN)-beta-mediated pathway to induce/enhance sIL-1Ra production, dampening inflammation. |
| 18304 | SMAD6 | 16951688 | SMAD6 is a critical mediator of the TGF-beta-BMP pathway that mediates anti-inflammatory activity and negatively regulates IL-1R-Toll-like receptor signals. |
| 18304 | SMAD6 | 20171181 | SMAD6 acts a critical mediator for effective TGF-beta1-mediated suppression of IL-1R/TLR signalling, by simultaneous binding to discrete regions of Pellino-1. |
| 18753 | STAP2 | 18573890 | STAP2 acts as an endogenous negative regulator of EBV LMP1-mediated signalling through TRAF3 and TRADD. |
| 19140 | TOLLIP | 11751856 | TOLLIP is a negative regulator of toll-like receptor (TLR) mediated signalling which serves to limit the production of pro-inflammatory mediators during inflammation and infection. |
| 19140 | TOLLIP | 11751856 | TOLLIP over-expression inhibits activation of NF-kappaB in response to IL1, the TLR2 and TLR4 ligands. |
| 19140 | TOLLIP | 19716405 | TOLLIP/IRAK1/TRAF6 complex formation is positively regulated by RCAN1 (DSCR1) short isoform, resulting in the modulation of IL-1R-mediated signalling pathways. |
| 19263 | ARRB2 | 19783052 | ARRB2 acts to limit JNK/ERK activation and survival in macrophages and is required for basal and TLR-inducible complement C1q expression. |

| | | | |
|---|---|---|---|
| 19263 | ARRB2 | 20497256 | ARRB2 regulates TLR4-mediated apoptotic signalling through GSK3B where ARRB2 represents an inhibitory effect on the TLR4-mediated apoptotic cascade, through controlling the homeostasis of activation and inactivation of GSK3B. |
| 20380 | REST | 20798038 | RE-1 silencing transcription factor (REST) is part of a repressor complex, along with key components that include histone deacetylase (HDAC) 1 or 2, co-repressor of REST (CoREST), and lysine-specific demethylase (LSD) 1. The HDAC/CoREST/REST/LSD1 repressor complex is a significant component of host innate immunity. |
| 20861 | HSP90AA1 | 17420470 | HSP90AA1 (HSP90) positively regulates NOD1 activation. |
| 20861 | HSP90AA1 | 18950863 | HSP90AA1 regulates the stability of transforming growth factor beta-activated kinase 1 (TAK1) in interleukin-1beta (IL1B)-induced cell signalling and IL1B-induced signalling by interacting with and maintaining the stability of TAK1, suggesting that HSP90AA1 might act as the chaperone of TAK1 in immune and inflammatory responses related with IL1A (IL-1) signal cascades. |
| 21216 | RCOR1 | 20798038 | Co-repressor of REST (RCOR1) is part of a repressor complex, along with key components that include histone deacetylase 1 or 2, RE-1 silencing transcription factor (REST), and lysine-specific demethylase (LSD) 1. The HDAC/RCOR1/REST/LSD1 repressor complex is a significant component of host innate immunity. |
| 21805 | KAT2B | 10022868 | KAT2B is a histone acetylase and a phorbol ester-inducible co-activator of the interferon (IFN) regulatory factor (IRF) proteins which contributes to the establishment of type I IFN responsiveness. |
| 21836 | NLRP1 | 18280719 | NLRP1 forms a biochemical complex, or inflammasome, with NALP1, CARD7, and DEFCAP. |
| 21836 | NLRP1 | 21098108 | NLRP1 forms inflammasome upon cellular infection by Toxoplasma gondii - this process is critical in mediating the innate immune response to T. gondii infection and pathogenesis. |
| 22332 | THRB | 20018842 | THRB signalling plays a role in modulating dendritic cell (DC) physiology and has immunoregulatory effects. THRB contains an NF-kappaB consensus site in its promoter region that controls its expression, which in turn signals DCs to promote maturation and function via an Akt-dependent, but PI3K independent pathway. |
| 23411 | MTA1 | 20699220 | MTA1 binds to the MYD88 promoter to regulate LPS-induced NFkappa B signaling via MYD88-dependent signalling in murine macrophages. |

| | | | |
|---|---|---|---|
| 175870 | Mta1 | 20699220 | Mta1 binds to the Myd88 promoter to regulate LPS-induced NFkappa B signaling via Myd88-dependent signalling in murine macrophages. |
| 23908 | CCR4 | 18624303 | CCR4 modulates TLR9-mediated innate immunity and signalling. |
| 23954 | IL8 | 17220369 | IL8 is produced by airway epithelial cells in response to invading bacteria and mediates airway epithelial defence against bacterial infection via the DUOX1-TACE-TGF-alpha-EGFR signalling pathway. |
| 24023 | CD36 | 19237602 | CD36 mediates host defence against Cryptococcus neoformans and Candida albicans through cytokine production. |
| 24023 | CD36 | 19847289 | CD36 ectodomain binds negatively charged diacylglycerol ligands and CD36, along with CD14, has a non-redundant role for loading ligands onto TLR2 in the plasma-membrane. |
| 24023 | CD36 | 15690042 | CD36 is a selective and non-redundant sensor of microbial diacylglycerides that signal via the TLR2/6 heterodimer. |
| 24023 | CD36 | 20037584 | CD36-TLR4-TLR6 activation is a common molecular mechanism by which atherogenic lipids and amyloid-beta stimulate sterile inflammation. |
| 24968 | CD180 | 19154986 | CD180 is an accessory molecule for TLR2, forming part of the receptor complex for innate immune recognition of mycobacterial lipoproteins. |
| 24968 | CD180 | 15852007 | CD180 and its helper molecule, LY86, interact directly with the TLR4 signalling complex, inhibiting its ability to bind microbial ligands. |
| 24968 | CD180 | 20133206 | CD180 dramatically enhances CpG DNA-induced proliferation/survival by naive B cells but not by memory B cell. This enhancement that is mediated by CD180-induced TLR9 upregulation, leading to Akt activation and sustained NF-kappaB activation. |
| 25591 | BCL2A1 | 21167304 | BCL2A1 negatively regulates autophagy and expression of BCL2A1 in Mycobacterium tuberculosis infected macrophages provides the bacteria a survival strategy to overcome host defences. |
| 25766 | TCEB1 | 9918119 | TCEB1 is a part of Elongin BC complex, and is a component of a multiprotein SOCS1 complex that attenuates JAK/STAT signalling by binding to JAK2 and inhibiting JAK2 kinase and by interacting with SOCS box, the Elongin BC complex can increase expression of the SOCS1 protein by inhibiting its degradation. |
| 28022 | IRAK4 | 18794297 | IRAK4 is required for interleukin-1 receptor/toll-like receptor-induced MAP3K7 (TAK1)-dependent NF-kappaB activation. |
| 28022 | IRAK4 | 17997719 | IRAK4 and IRAK1 play key roles in a signalling pathway by which bacterial infection or IL-1 trigger the production of inflammatory mediators. |

| | | | |
|---|---|---|---|
| 28022 | IRAK4 | 11923871 | IRAK4 is indispensable for the responses of animals and cultured cells to IL-1 and ligands that stimulate various Toll-like receptors (TLRs). |
| 28022 | IRAK4 | 12682231 | IRAK4 plays a critical role in IL-1R and TLR signalling cascades and is an essential component of the IL-18 signalling cascade. |
| 28022 | IRAK4 | 21220427 | IRAK4 activation is impaired during endotoxin tolerization; a process which impairs the production of LPS-induced pro-inflammatory cytokines without inhibition expression of anti-inflammatory or anti-microbial mediators. |
| 28139 | ITGAM | 20639876 | ITGAM (CD11b integrin) is activated via Toll-like receptors (TLRs) and engages in crosstalk with the MYD88 and TICAM1 (TRIF) pathways inhibiting TLR signalling in innate immune responses. |
| 28139 | ITGAM | 21245270 | ITGAM :: ITGB2 is the principal leukocyte receptor involved in the recognition of the fungus Candida albicans. Recognition of Pra1p protein of C. albicans by ITGAM :: ITGB2 plays a pivotal role in determining fungal virulence, and host response/protection against C. albicans infection. (Demonstrated in murine model) |
| 29237 | VDR | 19931640 | VDR, vitamin D receptor, deletion leads to reduced level of NFKBIA protein through protein translation, protein-protein interaction, and post-translational modification. |
| 30112 | SIAH1 | 11742346 | SIAH1 is a ubiquitin ligase structurally related to TRAF and modulates TNF-alpha signalling. |
| 31324 | LPCAT2 | 20663880 | LPCAT2 is highly expressed in inflammatory cells and is activated by lipopolysaccharide (LPS) treatment through Toll-like receptor 4 (TLR4) and LPCAT2 phosphorylation through LPS-TLR4 signalling may directly depend on MAPK-activated protein kinase 2 (MAPKAPK2). |
| 32513 | ACHE | 18385943 | ACHE (acetylcholinesterase) expression is induced by hydrogen peroxide (H2O2) via the JNK/AP1/ ATF2 signalling pathway. |
| 32847 | loleculeID 328[illegible] | 21187075 | PALM3 is a LPS inducible gene that functions as an adaptor protein for TLR4 signalling. PALM3 interacts with SIGIRR to negatively regulate TLR signalling. |
| 35609 | TRAIP | 19151749 | TRAIP is a SYK antagonist in TNF signaling - overexpression of TRAIP sensitize cells to TNF-induced apoptosis. |
| 36010 | TRADD | 18439848 | TRADD is recruited to MAVS and orchestrated complex formation with TRAF3 and TANK and with FADD and RIPK1, leading to the activation of IRF3 and NF-kappaB-TRADD is not only essential in TNFR1 signalling but also in RIG-I antiviral pathway. |

| | | | |
|---|---|---|---|
| 36076 | TNIP3 | 17088249 | TNIP3 is a novel lipopolysaccharide-inducible inhibitor of NF-kappaB activation, binds to A20 and inhibits NF-kappaB activation induced by tumour necrosis factor, interleukin-1, and 12-O-tetradecanoylphorbol-13-acetate. |
| 36895 | MAP3K12 | 20940047 | MAP3K12 (DLK) and its downstream kinases contribute to the finely tuned regulation of CREB-dependent effects. MAP3K12 inhibits CREB activity by affecting the interaction of CREB with its second co-activator TORC. |
| 37147 | PTK2 | 18353772 | PTK2 induces KLF8 expression in human ovarian cancer cells by activating the PI3K-Akt signalling pathway. |
| 37314 | CAV1 | 19949109 | CAV1 is an important component of the innate host immune response to the majority of non-cytotoxic strains of P. aeruginosa by promoting bacterial clearance during acute pneumonia and chronic colonization. |
| 37314 | CAV1 | 20304961 | CAV1 is a scaffolding protein of caveolae that plays an important role in host defence and inflammation and CAV1 deficiency dampens Toll-like receptor 4 signalling through NOS3 (eNOS) activation. |
| 38838 | ELMOD2 | 19966137 | ELMOD2 regulates the TLR3 signalling pathway where silencing of ELMOD2 in human macrophages inhibited TLR3-dependent expression of type I and type III interferon genes. |
| 40313 | RNF41 | 19483718 | RNF41 is an E3 ligase that can negatively regulate MyD88-dependent production of pro-inflammatory cytokines but can promote TRIF-dependent production of type I interferon. |
| 40694 | SIRPA | 18233962 | SIRPA negatively regulates TLR4 or TLR3 dependent cytokine production through inhibition of NF-kappaB dependent signalling. |
| 40694 | SIRPA | 17954568 | SIRPA down-regulation is lipopolysaccharide (LPS) inducible and contributes to innate immune activation in macrophages. |
| 42468 | BCAR1 | 17129785 | BCAR1 acts as a primary force sensor, transducing force into mechanical extension and thereby priming phosphorylation and activation of downstream signalling whereby tyrosine phosphorylation of BCRA1 in a cytoskeletal complex is involved in force-dependent activation of the small GTPase RAP1A. |
| 43779 | ZMYND11 | 19795416 | Nuclear ZMYND11 is recruited by oligomerized, cytoplasmic TICAM1 to enhance NF-kappaB activation and type-I-interferon induction. ZMYND11 harbours dual modes of cytoplasmic NF-kappaB regulation, positively in the TICAM1 pathway and negatively in the PDLIM7 (LMP1) pathway. |
| 43779 | ZMYND11 | 20138174 | ZMYND11 cooperates with TRAF3 in the regulation of Epstein-Barr virus-derived LMP1/CTAR1-induced NF-kappaB activation. |

| | | | |
|---|---|---|---|
| 44001 | CD22 | 17562860 | CD22 (Siglec-2), like other sialic acid-binding immunoglobulin-like lectins (siglecs), is predominately expressed on immune cells. CD22 exhibits hallmarks of clathrin-mediated endocytosis and traffics to recycling compartment, reflecting its role in cell signalling and innate immunity. |
| 44001 | CD22 | 12055217 | CD22 expression and function are differentially regulated in B-1 and conventional B-2 cells, which are implicated in innate and adaptive immunity, respectively. |
| 47487 | CD274 | 21097698 | CD274 expression in macrophages is induced upon exposure to HIV virions and TLR stimulation. In addition, IL10 also up-regulates CD274 expression. |
| 47595 | PDCD1LG2 | 21097698 | PDCD1LG2 expression in macrophages is induced upon exposure to HIV virions, and is transcriptionally down-regulated by IL10. |
| 47801 | CCR7 | 20176793 | CCR7 is a chemokine receptor expressed on the surfaces of T cells, B cells, and mature dendritic cells that controls cell migration in response to the cognate ligands CCL19 and CCL21. CCR7 deficiency results in a heightened pro-inflammatory environment in response to acute pulmonary P. aeruginosa infection and contributes to more efficient clearance. |
| 47847 | SMARCE1 | 17669635 | SMARCE1 is a transcriptional modulator that is known to repress viral replication. |
| 47847 | SMARCE1 | 16135788 | SMARCE1 is associated with up-regulation of proapoptotic genes, including the tumour suppressor familial cylindromatosis (CYLD). |
| 50200 | NKIRAS2 | 20351193 | NKIRAS2 is a key inhibitor of NF-kappaB signalling and estradiol represses NF-kappaB activation through the induction of NKIRAS2. |
| 50252 | DHX58 | 17190814 | DHX58 (LGP2) helicase is related to DDX58 (RIG-I) and IFIH1 (MDA5) but lacks caspase activation and recruitment domains (CARDs), and functions as a negative regulator of innate host defence. |
| 50252 | DHX58 | 16210631 | DHX58 lacks the caspase recruitment domain (CARD) homology and functions as a negative regulator by interfering with the recognition of viral RNA by DDX58 (RIG-I) and IFIH1 (MDA5). |
| 50252 | DHX58 | 16116171 | DHX58 functions as a negative regulator of antiviral innate immunity by interfering with the recognition of viral RNA by DDX58 (RIG-I) and IFIH1 (MDA5). |
| 51299 | SOCS2 | 14645718 | SOCS2 can enhance IL-2 and IL-3 signalling by accelerating SOCS3 degradation. |
| 51769 | BECN1 | 18772134 | BECN1 is recruited into the mouse Myd88 and Ticam TLR-signalling complex thereby reducing its binding to Bcl2, leading to autophagy. |
| 52025 | NUMBL | 18299187 | NUMBL interacts with TAB2 (MAP3K7IP2) and inhibits TNF alpha and IL-1Beta-induced NF-kappaB activation. |

| | | | |
|---|---|---|---|
| 52430 | CCDC88A | 21209316 | CCDC88A, an activator of GNAI3, plays a key role in regulating autophagy; the dynamic interplay between GNAI3, GPSM1 and CCDC88A determines whether autophagy is promoted or inhibited. When stimulated by growth factors, CCDC88A disrupts the GNAI3::GPSM1 complex, subsequently enhancing anti-autophagy signalling pathways and inhibits autophagy by activating GNAI3. |
| 53332 | RPS19 | 19155217 | RPS19 interacts with macrophage migration inhibitory factor (MIF) and attenuates its pro-inflammatory function by inhibiting the MIF-CD74 interaction and MIF triggered adhesion of monocytes. |
| 53452 | CAMK2A | 18818394 | CAMK2A promotes TLR-triggered pro-inflammatory cytokine and type-I-IFN production by directly binding and activating MAP3K7 and IRF3 in macrophages. (Demonstrated in murine model) |
| 150657 | Camk2a | 18818394 | Camk2a promotes Tlr-triggered pro-inflammatory cytokine and type-I-IFN production by directly binding and activating Map3k7 and Irf3 in mouse macrophages. |
| 54199 | IFNA2 | 18729739 | IFNA2 controls chemotaxis by regulating the CXC receptor ligand interaction between CXCL10 and CXCR3A. |
| 54199 | IFNA2 | 18027911 | IFNA2 interacts differentially with IFNAR2, and influences IFNA1 interaction with IFNAR2. |
| 54199 | IFNA2 | 18606657 | IFNA2 induces the transcription of HIF1A in human endothelial cells though a JAK-ISGF3 pathway under normoxic conditions, and this response contributes to the anti-proliferative activity of this cytokine. |
| 54276 | IFNA1 | 15883164 | IFNA1 activates NF-kappaB in JAK1-deficient cells through a TYK2-dependent pathway where for the IFN signalling pathway leading to STAT activation, both JAK1 and TYK2 are essential, but NF-kappaB activation requires only TYK2. |
| 54276 | IFNA1 | 12817009 | IFNA1 induces the human anti-inflammatory cytokine IL-10 gene via a module consisting of interdependent IRF1 and STAT3 motifs. |
| 54276 | IFNA1 | 15699120 | IFNA1 sensitizes cells to microbial recognition by up-regulating the expression of several TLRs as well as adapter molecules and kinases involved in TLR signalling. |
| 54276 | IFNA1 | 9737881 | IFNA1 is a type I interferon (IFN) that binds to the IFN receptor (IFNAR), composed of two transmembrane polypeptides, IFNAR1 and IFNAR2;IL10RB. |
| 54829 | MAP3K14 | 18550535 | MAP3K14 associates with DDX58 (RIG-I) and its downstream adaptor, mitochondrial antiviral signaling (MAVS) and the MAP3K14-DDX58 signalling pathway induces RELA release from NFKB2 complexes in response to respiratory syncytial virus (RSV) infection. |

| | | | |
|---|---|---|---|
| 54829 | MAP3K14 | 16552041 | MAP3K14 binds to NOD2 and mediates induction of specific changes induced by NOD2 activator muramyl dipeptide (MDP) and this occurs in settings where both the NOD2 and TLR4 pathways are activated by their respective agonists. |
| 54978 | ANXA4 | 20237821 | ANXA4 differentially modulates the NF-kappaB signalling pathway and this is dependent on its interactions with NFKB1 and the intracellular Ca(2+) ion level. |
| 55775 | CYBB | 20421951 | CYBB (NOX2) functions in reactive oxygen species (ROS) generation to induce TNF-alpha-mediated host cell apoptosis and it function in sensing of persistent intracellular pathogens for subsequent induction of host cell apoptosis as a second line of defence. |
| 55775 | CYBB | 19339495 | CYBB is an NADPH oxidases which plays a central role in microbial killing by phagocytes through the generation of reactive oxygen species (ROS) and CYBB-generated ROS are necessary for LC3 recruitment to phagosomes, coupling oxidative and non-oxidative killing activities of the CYBB NADPH oxidase in phagocytes through autophagy. |
| 55775 | CYBB | 20532218 | CYBB and reactive oxygen species (ROS) are required for the host cell to trigger an efficient RIG-I-mediated IRF-3 activation and downstream antiviral IFNbeta and IFIT1 gene expression and CYBB is critical for the expression of the central mitochondria-associated adaptor MAVS. |
| 56568 | BCL3 | 21228348 | BCL3 is critically involved in lung defence against Gram-negative bacteria Klebsiella pneumoniae, modulating functions of several cells to facilitate efficient clearance of bacteria. Loss of BCL3 incurred dramatic cytokine imbalance in the lungs, failure to clear bacteria and increased susceptibility to K. pneumoniae pneumonia. (Demonstrated in murine model) |
| 57326 | RIPK1 | 16543241 | RIPK1 ubiquitination on Lys377 is required for tumour necrosis factor (TNF-alpha) induced NF-kappaB activation. |
| 57326 | RIPK1 | 16825191 | RIPK1 is a death domain kinase that is one of the critical components involved in mediating DNA damage-induced, p53-independent cell death. |
| 57921 | TRAFD1 | 18849341 | TRAFD1 deficiency reveals its negative regulatory role in the TLR and RIG-I-like helicases signalling pathway and was found to interact with TRIF, IPS-1, TRAF3 and TRAF6 in mice |
| 57921 | TRAFD1 | 16221674 | TRAFD1 is an interferon and LPS inducible gene acting as a negative regulator of Toll-like receptor signalling. |

| | | | |
|---|---|---|---|
| 58012 | ITGA3 | 20877569 | ITGA3 along with ITGB1 is a novel regulator for the recognition of bacterial lipopeptides. ITGA3/ITGB1 integrin regulates endosomal Toll-like receptor (TLR)-2/TLR1 signalling, serving as a mechanism for modulating inflammatory responses. |
| 58309 | IRF2BP1 | 18671972 | IRF2BP1 is a transcriptional co-repressor of IRF2 and, through physically association, enhances JDP2 polyubiquitination. IRF2BP1 has also been shown to repress ATF2-mediated transcriptional activation from a CRE-containing promoter. |
| 58598 | PIK3CB | 20953381 | PIK3CA and PIK3CB isoforms of class IA phosphatidylinositol 3-kinase (PI3K) are both required for the pro-inflammatory response to flagellin. |
| 59283 | LY86 | 15852007 | LY86 and CD180 interact directly with the TLR4 signalling complex, inhibiting its ability to bind microbial ligand. |
| 62534 | RANBP9 | 18040864 | RANBP9 is located in the Microtubule-Organizing Center it contains protein-interaction motifs, a cytoskeletal-binding domain, and multiple canonical docking sites for signalling intermediates. RANBP9 acts as an scaffolding protein and is important for maintaining cellular functions in the immune and nervous system. |
| 63405 | NUP153 | 15210729 | NUP153 and NUP214 are nucleoporins that control the nucleo-cytoplasmic shuttling and, along with XPO1-dependent nuclear export, the subcellular distribution of latent STAT1. |
| 64831 | OTUD5 | 17991829 | OTUD5 is a deubiquitinase that regulates Type I interferon (IFN) production where OTUD5 selectively cleaves the lysine-63-linked polyubiquitin chains on TRAF3, resulting in its dissociation from the downstream signalling complex containing TBK1. |
| 66703 | MAP3K8 | 19667062 | MAP3K8 negatively regulates interferon-beta production in macrophages and myeloid dendritic cells. |
| 66703 | MAP3K8 | 19933865 | MAP3K8 is essential for IL-1beta production from both macrophages and dendritic cells and an important mediator for collaboration of pattern recognition receptors with danger-associated molecular patterns (DAMPs) to induce TNF and IL-1beta production and optimal host defence. |
| 66703 | MAP3K8 | 20405269 | MAP3K8 regulates IL23A expression in lipopolysaccharide (LPS)-stimulated macrophages through extracellular signal-regulated kinase (ERK) activation. |
| 66703 | MAP3K8 | 21135874 | MAP3K8 is a MEK kinase that is require for the activation of MAP kinases in myeloid cells following TLR and TNF receptor stimulation. MAP3K8 is critical for production of the pro-inflammatory cytokine TNF during inflammatory responses. |

| | | | |
|---|---|---|---|
| 68562 | LILRA4 | 20193018 | LILRA4 couples with a signalling adapter to activate a prominent immunoreceptor tyrosine-based activation motif (ITAM)-mediated signalling pathway in human plasmacytoid dendritic cells. |
| 69045 | BIRC3 | 18697935 | BIRC3 is required for proper RIPK1 polyubiquitination and NF-kappaB activation upon TNF (TNF-alpha) treatment and regulates TNF-mediated NF-kappaB activation by binding to TNFRSF1A (TNFR1). |
| 69045 | BIRC3 | 19464198 | BIRC3 and BIRC2 are required for innate immunity signalling by the pattern recognition receptors NOD1 and NOD2. |
| 69466 | PROCR | 20543103 | PROCR confers anti-inflammatory properties when bound by activated protein C and when PROCR (EPCR) is blocked, F. tularensis loses the ability to suppress the pro-inflammatory response of endothelial cells. |
| 69532 | CASP12 | 18329614 | Rat CASP12 binds to Human RIPK2 and displaces Human TRAF6 from a complex in human cells, inhibiting its ubiquitin ligase activity, and blunting NF-kappaB activation. |
| 69532 | CASP12 | 18329614 | CASP12 deficiency enhances production of antimicrobial peptides, cytokines, and chemokines dependent on bacterial type III secretion and the Nod pathway. |
| 69714 | CARD18 | 11051551 | CARD18 is an inhibitor of LPS-induced IL1B (IL-1 beta) generation by preventing RIPK2-mediated oligomerization and auto-activation of CASP1. |
| 69714 | CARD18 | 11536016 | CARD18 is an intracellular regulator of CASP1 (caspase-1) activation and plays a role in the regulation of IL1B (IL-1beta) secretion and NF-kappaB activation during the pro-inflammatory cytokine response. |
| 70676 | SOCS3 | 10882725 | SOCS3 inhibits the JAK/STAT pathway and act as a negative regulator of fetal liver erythropoiesis (EPO) by binding the JAK2 and EPO receptor, respectively. |
| 70676 | SOCS3 | 10373548 | SOCS3 is rapidly induced by IL-2 in T cells where it acts to inhibit IL-2 responses in a classical negative feedback loop by suppressing STAT5 phosphorylation and lymphocyte proliferation. |
| 70676 | SOCS3 | 18989459 | SOCS3 protein is a potent endogenous inhibitor of Janus kinase (JAK)/STAT signalling and influenza A virus inhibits type I IFN signalling via NF-kappaB-dependent induction of SOCS3 expression. |
| 70676 | SOCS3 | 20631305 | SOCS3 is a molecular inhibitor of IFN signalling and SOCS3 expression, induced by stimuli present in the Human immunodeficiency virus (HIV)-1-infected brain, such as transactivator of transcription, inhibits antiviral IFN-beta signalling to enhance HIV-1 replication in macrophages, allowing HIV-1 to evade the protective innate immune response within the central nervous system. |

| | | | |
|---|---|---|---|
| 72874 | CYTIP | 17577583 | CYTIP co-localizes with SNX27 at the early endosomal compartment of lymphocytes suggesting a role for this interaction in endocytic trafficking and/or signalling. |
| 75124 | MAFB | 20581830 | MAFB binds to IRF3, impairing the recruitment of co-activators to IRF3 and antagonizing antiviral responses. |
| 159441 | Sirt1 | 21245135 | Sirt1 is a histone deacetylase enzyme that has been found to accumulate at the promoters of Tnfa and Il1b in response to Tlr4 signalling. Sirt1 promotes termination of NFKB-dependent transcription and recruits Relb to assemble transcription repressor complex that generates endotoxin tolerance. |
| 75847 | SIRT1 | 21245135 | SIRT1 is a histone deacetylase enzyme that has been found to accumulate at the promoters of TNFA and IL1B in response to TLR4 signalling. SIRT1 promotes termination of NFKB-dependent transcription and recruits RELB to assemble transcription repressor complex that generates endotoxin tolerance. |
| 75863 | NFE2L2 | 21220332 | NFE2L2 plays an important role in TLR4-mediated autophagy. NFE2L2 is activated by reactive oxygen species-MAPK14 axis-dependent TLR4 signalling, and induces the accumulation of SQSTM1 and aggresome-like induced structures. |
| 75950 | FLI1 | 20879862 | FLI1 contributes to lipopolysaccharide (LPS)-induced expression of matrix metalloprotease 1 (MMP-1), MMP-3, MMP-10, and interleukin-10 (IL-10) and rapid down-regulation of FLI1 expression after LPS stimulation attenuates the induction of various MMPs and IL-10 under inflammatory conditions. |
| 77092 | PTCH1 | 21131441 | PTCH1 is a member of the Hedgehog signalling pathway and is important for LPS-induced inflammatory response in macrophages. (Demonstrated in Murine model) |
| 77625 | STAT4 | 17971840 | STAT4 is a transcription factor that mediates IL12 cytokine signalling, and as a member of the STAT protein family, STAT4 have significant impact on innate immunity during sepsis. |
| 77625 | STAT4 | 14704793 | STAT4 relies on the phosphorylation of it's N-domains to form STAT4 tetramer in response to cytokine-induced activation. |
| 79215 | CTLA4 | 18824539 | CTLA4 blockade abrogates protection by regulatory T cells in a mouse model of microbe-induced innate immune-driven colitis. |
| 80038 | CTNNAL1 | 17952117 | CTNNAL1 is an alpha-catenin-related protein that shares structural similarities with cytoskeleton linker proteins that augments NF-kappaB activity, promotes cell migration and increases resistance to apoptosis. |

| | | | |
|---|---|---|---|
| 80804 | IL8RB | 20818377 | IL8RB is a G protein-coupled receptor that mediates NADPH oxidase-independent neutrophil extracellular trap (NET) formation and this G protein-coupled receptor (GPCR) pathway is operative and drug-sensitive in cystic fibrosis lung disease. |
| 80875 | AAMP | 19535145 | AAMP over-expression inhibits NOD1 and NOD2 mediated NF-kappaB signalling |
| 81223 | NFATC2 | 20557936 | NFATC2 interacts with JUN to synergistically activate interleukin-2 (IL2) transcription in T cells, a cytokine that acts as an autocrine growth factor during an immune response to bacterial and viral infection, as well as tumourigenesis. |
| 84173 | COPS8 | 20074051 | COPS8 is part of the COP9 signallosome that functions to control NF-kappaB activation. |
| 84322 | LRRFIP1 | 20453844 | LRRFIP1 interacts with and activates beta-catenin, which increases IFN-beta expression by binding to the C-terminal domain of the transcription factor IRF3 and recruiting the acetyltransferase EP300 to the IFN-beta enhanceosome via IRF3. |
| 84322 | LRRFIP1 | 16199883 | LRRFIP1 (GCF2) acts as a repressor and occupies the -308 site of the TNF-alpha promoter in cells that do not make TNF-alpha. Other proteins may bind to the promoter, particular to the -308 site to transition from repressed to active transcription. |
| 84322 | LRRFIP1 | 21102652 | LRRFIP1 is a regulator of toll-like receptor (TLR) pathway signaling and it co-localized with dsRNA in monocyte lysosomal structures. |
| 87893 | NFKB2 | 7925301 | NFKB2 (p49/p100 subunit) associates efficiently with RELB and up-regulates the synthesis of NFKBIA (IKappaB-alpha). |
| 87893 | NFKB2 | 18025196 | NFKB2 plays a key role in the regulation of RELA activation, suggesting an overlap in the function of NF-kappaB members in canonical and non-canonical pathway signalling. |
| 87893 | NFKB2 | 18377428 | NFKB2 rearrangement gene product (p58) localizes in the nucleus to form a complex with RELA or RELB, suggesting that such NFKB2 gene rearrangement may therefore be a factor in the constitutive activation of NF-kappaB in adult T-cell leukemia (ATL), and thereby playing a role in the ATL pathogenesis. |
| 87893 | NFKB2 | 17548614 | NFKB2 negatively regulates TCR signaling by binding with RELA, RELB, REL and NFKB1 (p50) in the cytoplasm and inhibiting these proteins from entering nucleus. |
| 87893 | NFKB2 | 19770515 | NFKB2 limits TNF-induced bone resorption in mice by a TRAF3-dependent mechanism. |
| 88716 | VEGFA | 21278304 | VEGFA-induced tissue response, e.g. angiogenesis, is inhibited during RSV and Influenza viral infections. These effects were mediated by RIG-I and IFNR-dependent pathways, and consequently inhibited the Th2 inflammation response. |

| | | | |
|---|---|---|---|
| 89810 | NUP214 | 15210729 | NUP214 and NUP153 are nucleoporins that control the nucleo-cytoplasmic shuttling and, along with XPO1-dependent nuclear export, the subcellular distribution of latent STAT1. |
| 89999 | MFN2 | 21285412 | MFN2 deletion causes inability of the cell to undergo mitochondrial fusion, and therefore exhibit impaired induction of interferons and pro-inflammatory cytokines in response to viral infection - which results in increased viral replication. |
| 90091 | TNFRSF1B | 17220297 | TNFRSF1B signalling induces selective BIRC2-dependent ASK1 ubiquitination and terminates mitogen-activated protein kinase signalling. |
| 90517 | IL17F | 19144317 | IL17A and IL17F are required for the induction of beta-defensin in vivo in mice. |
| 90517 | IL17F | 19244213 | IL17F signalling inhibits expression of pro-inflammatory genes though sequential phosphorylation of CEBPB regulatory 2 domain. |
| 90517 | IL17F | 21074482 | IL17F is a Th17-related cytokine, traditionally thought of as an adaptive responder, has been shown to have various innate sources and functions as a rapidly produced pro-inflammatory mediator. Innate IL17F-producing cells also employ many of the cytokine and transcriptional regulators utilized by Th17 cells. |
| 91131 | GRK5 | 20945396 | GRK5 is a serine/threonine kinase has a role in the regulation of G-protein coupled receptor (GPCR) signalling and is also an important regulator of signalling pathways stimulated by non-GPCRs. GRK5 also is a positive regulator of TLR4-induced I-kappa-B-alpha-NFkappaB pathway as well as a key modulator of lipopolysaccharide-induced inflammatory response. |
| 91946 | DMBT1 | 20418254 | DMBT1 encodes alternatively spliced proteins involved in mucosal innate immunity and two other molecules, a glycoprotein with a molecular mass of 340 kDa (GP340) and salivary agglutinin (DMBT1(SAG)). GP340 is secreted into broncho-alveolar surface lining fluid whereas DMBT1(SAG) is present in the saliva. Both interact with and agglutinate several Gram-negative and Gram-positive bacteria, as well as some viruses. |
| 92256 | GPSM1 | 21209316 | GPSM1, an inhibitor of GNAI3, plays a key role in regulating autophagy; the dynamic interplay between GNAI3, GPSM1, and CCDC88A determines whether autophagy is promoted or inhibited. During starving conditions, GPSM1 binds to GNAI3 in MAP1LC3A-positive membranes to promote autophagy. When stimulated by growth factors, CCDC88A promotes the dissociation of GPSM1::GNAI3 complex to initiate anti-autophagy signalling pathways. |

| | | | |
|---|---|---|---|
| 92266 | CARD9 | 19076343 | CARD9 signalling mediates mammalian innate immune responses against selected fungi, bacteria, and viruses and can prime and shape adaptive immunity. CARD9 plays an essential role in downstream signalling of the antifungal pattern-recognition receptor CLEC7A (Dectin-1). |
| 92266 | CARD9 | 20351059 | CARD9 is critical for full activation of innate immunity by converging signals downstream of multiple pattern recognition receptors (PRRs) and plays a pivotal role in autonomous innate host defence against tuberculosis. |
| 92843 | FBXW5 | 19232515 | FBXW5, an F-box family protein, negatively regulates MAP3K7 (TAK1) in the IL1B (IL-1beta) signalling pathway. |
| 93345 | IRAK1BP1 | 20534545 | IRAK1BP1 down-regulates Toll-like receptor-mediated transcription of several pro-inflammatory cytokines by changing the transcriptional profile of activated cells, leading to an increase in IL-10 production and promoting LPS tolerance. |
| 93808 | KDM1 | 20798038 | Lysine-specific demethylase 1 (KDM1) is part of a repressor complex, along with key components that include histone deacetylase 1 or 2, RE-1 silencing transcription factor (REST), and co-repressor of REST (RCOR1). The KDM1/HDAC/REST/RCOR1 repressor complex is a significant component of host innate immunity. |
| 94257 | RUNX3 | 16164020 | RUNX3 is capable of activating the CD11a gene promoter that directs CD11a/CD18 integrin expression as well as trans-activating the CD49d gene promoter. The leukocyte integrins CD11a/CD18 (LFA-1, alphaLbeta2) and CD49d (VLA-4, alpha4beta1, alpha4beta7) mediate leukocyte transendothelial migration during immune and inflammatory responses and provide co-stimulatory signals for the activation of T lymphocytes. |
| 95712 | HDAC1 | 14645718 | HDAC1 Inhibition decreases IFN-alpha responsiveness whereas its expression augments the IFN-alpha response, demonstrating that it modulates IFN-alpha-induced transcription. |
| 95712 | HDAC1 | 20798038 | Histone deacetylase 1 (HDAC1) is part of a repressor complex, along with key components that include HDAC2, RE-1 silencing transcription factor (REST), co-repressor of REST (CoREST), and lysine-specific demethylase (LSD) 1. The HDAC/CoREST/REST/LSD1 repressor complex is a significant component of host innate immunity. |
| 95984 | GJA1 | 19528242 | GJA1 plays an important role in innate immune control of commensal-mediated intestinal epithelial wound repair. |

| | | | |
|---|---|---|---|
| 97690 | MAP3K7IP2 | 11259596 | MAP3K7IP2 (TAB2) interacts with both MAP3K7 (TAK1) and TRAF6 and promotes their association, thereby triggering subsequent interleukin-1 signalling events. |
| 97690 | MAP3K7IP2 | 15327770 | MAP3K7IP2 (TAB2) and MAP3K7IP2 (TAB3) activate the NF-kappaB pathway through binding to K63-linked polyubiquitin chains. |
| 97690 | MAP3K7IP2 | 19150425 | MAP3K7IP2 binds to K-63 polyubiquitinated TRAF2 and this association is required for activation of downstream IKK and JNK kinases. |
| 97690 | MAP3K7IP2 | 19955178 | MAP3K7IP2 (TAB2) activates MAP3K7 (TAK1) and also plays an essential role in the deactivation of TAK1 by recruiting PP6 through a polyubiquitin chain-dependent mechanism. |
| 97690 | MAP3K7IP2 | 10882101 | MAP3K7IP2 is an adapter linking MAP3K7 (TAK1) and TRAF6 and also functions as a mediator of TAK1 activation in the IL-1 signalling pathway. |
| 97690 | MAP3K7IP2 | 14633987 | MAP3K7IP2 (TAB2) and MAP3K7IP3 (TAB3) function redundantly as mediators of TAK1 activation in IL-1 and TNF signal transduction. |
| 98137 | MKNK1 | 21149447 | MKNK1 plays an important role in IFNG induced IRF1 expression and is essential for generation of anti-proliferative responses. |
| 98770 | MAP3K4 | 17726008 | MAP3K4 (MEKK4) dimerization is regulated both positively and negatively by its interaction with specific proteins. GSK3B negatively regulates MAP3K4 stimulation of MAPK14 (P38) and MAPK8 (JNK) activity. |
| 98770 | MAP3K4 | 18775659 | MAP3K4 sequesters RIPK2 to inhibit the NOD2:RIPK2 complex from activating NF-kappaB signalling pathways. |
| 99221 | JUN | 20557936 | JUN interacts with NFATC2 to synergistically activate interleukin-2 (IL2) transcription in T cells, a cytokine that acts as an autocrine growth factor during an immune response to bacterial and viral infection, as well as tumourigenesis. |
| 100849 | GNAI3 | 21209316 | GNAI3 is a G protein involved in autophagy signalling pathways and is tightly regulated through its interaction with its GEF activator (CCDC88A) and GDI inhibitor (GPSM1). During starving conditions, GPSM1 binds to GNAI3 in MAP1LC3A-positive membranes to promote autophagy. When stimulated by growth factors, CCDC88A promotes the dissociation of GPSM1::GNAI3 complex to initiate anti-autophagy signalling pathways. |

| | | | |
|---|---|---|---|
| 186305 | Gnai3 | 21209316 | Gnai3 is a G protein involved in anti-autophagy signalling pathways and is tightly regulated through its interaction with its GEF activator (Ccdc88a) and GDI inhibitor (Gpsm1). During starving conditions, Gpsm1 binds to Gnai3 in Map1lc3a-positive membranes to promote autophagy. When stimulated by growth factors, Ccdc88a promotes the dissociation of Gpsm1::Gnai3 complex to initiate anti-autophagy signalling pathways. |
| 101004 | CD53 | 20407468 | CD53 is an important regulator of innate tumour necrosis factor (TNF)-alpha levels. |
| 102394 | SNX27 | 17577583 | SNX27 co-localizes with CYTIP at the early endosomal compartment of lymphocytes suggesting a role for this interaction in endocytic trafficking and/or signalling |
| 103247 | RUSC1 | 19365808 | RUSC1 binds to IKBKG and TRAF6, representing a molecular link between NGF signalling and IKK complex activity. |
| 104282 | FCGR2A | 12857726 | FCGR2A is a receptor for immunoglobulin G, clustering induces shape change, secretion and aggregation |
| 104282 | FCGR2A | 21044955 | FCGR2A (FcgammaRIIA) is a receptor that recognizes IgG opsonized particles and initiates phagocytosis in immune clearance. FCGR2A is also a positive regulator of complement-mediated phagocytosis. |
| 105504 | CFH | 18190458 | CFH is a major regulatory protein that down-regulates alternative complement activation. |
| 105504 | CFH | 21285368 | CFH, a complement regulatory factor, interacts with host cell surfaces as well as C3d part of C3b and plays a major in the distinguishing host from non-host surfaces during the alternative activated complement pathway. |
| 105609 | PTPRC | 11201744 | PTPRC (CD45) suppresses JAK (Janus kinase) kinases and negatively regulates cytokine receptor signaling by regulating interleukin-3-mediated cellular proliferation, erythropoietin-dependent haematopoiesis and antiviral responses in vitro and in vivo. |
| 106494 | TRAF5 | 11479302 | TRAF5 is a signal transducer for the TNF receptor superfamily that is involved in TNF-induced NF-kappaB activation and protection from cell death. |
| 106494 | TRAF5 | 19017969 | TRAF5, along with other TRAFs, is recruited to many TNF-receptor (TNF-R) superfamily members and is an important modulator of the proximal signaling events that occur at the time of receptor engagement and activation. TRAF5 has been shown to be a positive regulator of a number of these receptors that are involved in T cell co-stimulation. |

| | | | |
|---|---|---|---|
| 106494 | TRAF5 | 20161788 | TRAF5, a ubiquitin ligase, is a key molecule in the innate response against viral infection where it mediates the activation of IRF3 and NF-kappaB downstream of MAVS through the recruitment of NEMO. |
| 127623 | hsa-mir-146b | 16885212 | Hsa-mir-146b targets IRAK1 and TRAF6 mRNA and their expression is knocked down by co-expression of hsa-mir-146b, showing that hsa-mir-146b can post-transcriptionally repress IRAK1 and TRAF6. |
| 66114 | MFN1 | 21285412 | MFN1 deletion causes inability of the cell to undergo mitochondrial fusion, and therefore exhibit impaired induction of interferons and pro-inflammatory cytokines in response to viral infection - which results in increased viral replication. |
| 136722 | Mfn1 | 21285412 | Mfn1 deletion causes inability of the cell to undergo mitochondrial fusion, and therefore exhibit impaired induction of interferons and pro-inflammatory cytokines in response to viral infection - which results in increased viral replication. |
| 138525 | Cd14 | 21078886 | Cd14 contributes to nucleic acid uptake in macrophages and acts as a co-receptor for endosomal Tlr7/Tlr9 activation. |
| 148192 | Gpsm1 | 21209316 | Gpsm1, an inhibitor of Gnai3, plays a key role in regulating autophagy; the dynamic interplay between Gnai3, Gpsm1, and Ccdc88a determines whether autophagy is promoted or inhibited. During starving conditions, Gpsm1 binds to Gnai3 in Map1lc3a-positive membranes to promote autophagy. When stimulated by growth factors, Ccdc88a promotes the dissociation of Gpsm1::Gnai3 complex to initiate anti-autophagy signaling pathways. |
| 157020 | Ccdc88a | 21209316 | Ccdc88a, an activator of Gnai3, plays a key role in regulating autophagy; the dynamic interplay between Gnai3, Gpsm1 and Ccdc88a determines whether autophagy is promoted or inhibited. When stimulated by growth factors, Ccdc88a disrupts the Gnai3::Gpsm1 complex, subsequently enhancing anti-autophagy signaling pathways and inhibits autophagy by activating Gnai3. |
| 176847 | Mknk1 | 21149447 | Mknk1 plays an important role in Ifng induced Irf1 expression and is essential for generation of anti-proliferation responses. |

| | | | |
|---|---|---|---|
| 29322 | SPP1 | 21136203 | SPP1 exist in secreted and intracellular form; intracellular SPP1, or iSPP1, is involved in cytoskeleton rearrangement and signal transduction downstream of innate immunity receptors (e.g. TLR). iSPP1 may also function as an adaptor or scaffolding protein. |
| 185485 | Spp1 | 21136203 | Spp1 exist in secreted and intracellular form; intracellular Spp1, or iSpp1, is involved in cytoskeleton rearrangement and signal transduction downstream of innate immunity receptors (e.g. TLR). iSPP1 may also function as an adaptor or scaffolding protein. |
| 185847 | Vegfa | 21278304 | Vegfa-induced tissue response, e.g. angiogenesis, is inhibited during RSV and Influenza viral infections. These effects were mediated by RIG-I and IFNR-dependent pathways, and consequently inhibited the Th2 inflammation response. |
| 190275 | C4bp | 21283780 | C4bp plays an inhibitory role in antimicrobial response by delaying the classical complement activation and attenuating the lectin pathway activation. The major inhibitory role of C4bp is to facilitate the decay of C3 convertase. |
| 106321 | C4BPB | 21283780 | C4BPB plays an inhibitory role in antimicrobial response by delaying the classical complement activation and attenuating the lectin pathway activation. The major inhibitory role of C4BPB is to facilitate the decay of C3 convertase. |
| 106328 | C4BPA | 21283780 | C4BPA plays an inhibitory role in antimicrobial response by delaying the classical complement activation and attenuating the lectin pathway activation. The major inhibitory role of C4BPA is to facilitate the decay of C3 convertase. |
| 103905 | APCS | 21278351 | APCS is a key negative regulator for innate immune responses to DNA and may be partly responsible for the insufficient immune responses after DNA vaccination in humans. APCS-DNA complex showed significant defects in innate immune activation, and specifically inhibits the functions of HMGB1 and antimicrobial peptide LL37. |
| 187862 | Kitl | 21262348 | Kitl is a PI3K-activating ligand that increases the secretion of Il6 and Tnfa in LPS-stimulated mast cells, as well as the attenuating the production of Il1b. |

| | | | |
|---|---|---|---|
| 50398 | KITLG | 21262348 | KITLG is a PI3K-activating ligand that increases the secretion of IL6 and TNFA in LPS-stimulated mast cells, as well as the attenuating the production of IL1B. (Demonstrated in murine model) |
| 184148 | Pdcd1 | 21263073 | Pdcd1 expression in T cells is induced by Ifna stimulation and provides feedback inhibition of T cell activation. It is proposed that strong innate inflammatory response (i.e. Ifna secretion) cause an attenuated T cell response by Pdcd1 in sustained immune reaction. |
| 184148 | Pdcd1 | 21263070 | Pdcd1 is a negative immunomodulator that is upregulated by Hepatitis C virus to deliver negative signaling to Tlr-mediated pathways controlling expression of Il12, a key cytokine linking innate and adaptive immunity. |
| 85346 | PDCD1 | 21091911 | PDCD1 mediates functional impairment of early immune responses during HCV infection by limiting STAT1 phosphorylation, which consequently inhibits IL12 expression in monocytes / macrophages. |
| 85346 | PDCD1 | 21263073 | PDCD1 expression in T cells is induced by IFNA stimulation and provides feedback inhibition of T cell activation. It is proposed that strong innate inflammatory response (i.e. IFNA secretion) cause an attenuated T cell response by PDCD1 in sustained immune reaction. |
| 85346 | PDCD1 | 21263070 | PDCD1 is a negative immunomodulator that is upregulated by Hepatitis C virus to deliver negative signaling to TLR-mediated pathways controlling expression of IL12, a key cytokine linking innate and adaptive immunity. |
| 190364 | Rftn1 | 21266579 | Rftn1 cooperates with the uptake receptor to mediate cell entry of poly(I:C), which is critical for activation of Tlr3 and the subsequent production of IFN and inflammatory cytokines. |
| 21351 | RFTN1 | 21266579 | RFTN1 cooperates with the uptake receptor to mediate cell entry of poly(I:C), which is critical for activation of TLR3 and the subsequent production of IFN and inflammatory cytokines. |
| 138595 | Hspbp1 | 21247889 | Hspbp1 interacts with Pglyrp1 and inhibits the cytotoxic activity of the Hspa1a :: Pglyrp1 complex secreted by lymphocytes. |

| | | | |
|---|---|---|---|
| 70495 | HSPBP1 | 21247889 | HSPBP1 interacts with PGLYRP1 and inhibits the cytotoxic activity of the HSPA1A :: PGLYRP1 complex secreted by lymphocytes. |
| 183923 | Akap10 | 21247892 | Akap10 is required to induce Ptges2 in the synthesis of NO upon LPS stimulation, in addition Akap10 also mediates the Ptges2-induced expression of cytokines Il10 and Il6 in alveolar macrophages. (Demonstrated in rat models) |
| 35162 | AKAP10 | 21247892 | AKAP10 is required to induce PTGES2 in the synthesis of NO upon LPS stimulation, in addition AKAP10 also mediates the PTGES2-induced expression of cytokines IL10 and IL6 in alveolar macrophages. (Demonstrated in rat models) |
| 160124 | Ptges2 | 21247892 | Ptges2 is responsible for nearly half of the increment in NO production by alveolar macrophages in response to LPS stimulation. The enhancing effect of Ptges2 on NO production is mediated through the ligation of Ptger2 and acting via PKA to induce cAMP production. In addition Ptges2 induces expression of cytokines Il10 and Il6, while inhibiting Tnfa. (Demonstrated in rat models) |
| 87283 | PTGES2 | 21247892 | PTGES2 is responsible for nearly half of the increment in NO production by alveolar macrophages in response to LPS stimulation. The enhancing effect of PTGES2 on NO production is mediated through the ligation of PTGER2 and acting via PKA to induce cAMP production. In addition PTGES2 induces expression of cytokines IL10 and IL6, while inhibiting TNFA. (Demonstrated in rat models) |
| 202769 | Stim1 | 21239714 | Stim1 supports the influx of extracellular Ca(2+), which is required for production of reactive oxygen species in phagocytosis. |
| 24705 | STIM1 | 21239714 | STIM1 supports the influx of extracellular Ca(2+), which is required for production of reactive oxygen species in phagocytosis. |
| 199057 | Orai1 | 21239714 | Orai1 supports the influx of extracellular Ca(2+), which is required for production of reactive oxygen species in phagocytosis. |
| 61853 | ORAI1 | 21239714 | ORAI1 supports the influx of extracellular Ca(2+), which is required for production of reactive oxygen species in phagocytosis. |

| | | | |
|---|---|---|---|
| 156294 | Itpr3 | 21239714 | Itpr3 facilitates depletion of intracellular Ca(2+) for the internalization phase of FcgR-mediated phagocytosis. |
| 82972 | ITPR3 | 21239714 | ITPR3 facilitates depletion of intracellular Ca(2+) for the internalization phase of FcgR-mediated phagocytosis. |
| 174302 | Itpr1 | 21239714 | Itpr1 facilitates depletion of intracellular Ca(2+) for the internalization phase of FcgR-mediated phagocytosis. |
| 14187 | ITPR1 | 21239714 | ITPR1 facilitates depletion of intracellular Ca(2+) for the internalization phase of FcgR-mediated phagocytosis. |
| 152576 | Bcl3 | 21228348 | Bcl3 is critically involved in lung defense against Gram-negative bacteria Klebsiella pneumoniae, modulating functions of several cells to facilitate efficient clearance of bacteria. Loss of Bcl3 incurred dramatic cytokine imbalance in the lungs, failure to clear bacteria and increased susceptibility to K. pneumoniae pneumonia. |
| 202692 | Lrrfip2 | 21220426 | Lrrfip2, upon phosphorylation, interacts with Myd88 during LPS stimulation and induces NFKB activity. |
| 24724 | LRRFIP2 | 21220426 | LRRFIP2, when phosphorylated at serine 202, interacts with MYD88 during LPS stimulation to induces NFKB activity. |
| 209676 | Trib3 | 21220698 | TRIB3 is an inhibitor of TLR2 mediated NFKB activation and chemokine induction. Helicobacter pylori LPS stimulation decrease expression of TRIB3, which may be an important mechanism during H. pylori-associated pathogenesis. |
| 37170 | TRIB3 | 21220698 | TRIB3 is an inhibitor of TLR2 mediated NFKB activation and chemokine induction. Helicobacter pylori LPS stimulation decrease expression of TRIB3, which may be an important mechanism during H. pylori-associated pathogenesis. |
| 165906 | Chga | 21214543 | Chga undergoes proteolytic cleavage to give rise to biologically active peptides, including catestatin. Catestatin is a neuroendocrine peptide with effects on human autonomic function and has recently been found to be a cutaneous antimicrobial peptide by inducing mast cell activation. In mast cells, catestatin plays a role in the migration, degranulation and release of leukotriene and prostaglandins; in addition, catestatin also induces production of pro-inflammatory cytokines to prime the innate immune response. |

| | | | |
|---|---|---|---|
| 17248 | CHGA | 21214543 | CHGA undergoes proteolytic cleavage to give rise to biologically active peptides, including catestatin. Catestatin is a neuroendocrine peptide with effects on human autonomic function and has recently been found to be a cutaneous antimicrobial peptide by inducing mast cell activation. In mast cells, catestatin plays a role in the migration, degranulation and release of leukotriene and prostaglandins; in addition, catestatin also induces production of pro-inflammatory cytokines to prime the innate immune response. |
| 188669 | Siglech | 21208791 | Siglech is a member of the SIGLEC protein family that recognize sialoside-based patterns and plays a role in the attenuation of innate immunity to avoid autoimmune destruction. |
| 177258 | Siglece | 19933851 | Siglece is upregulated and phosphorylated following lipopolysaccharide stimulation in order to limit TLR-driven cytokine production and help maintain a healthy cytokine balance following infection. |
| 177258 | Siglece | 21208791 | Siglece is a member of the SIGLEC protein family that recognize sialoside-based patterns and plays a role in the attenuation of innate immunity to avoid autoimmune destruction. |
| 176568 | Siglec5 | 17562860 | Siglec5 is a member of the rapidly evolving CD33-related siglec subfamily that are expressed on cells of the innate immune system. Siglec5 mediates endocytosis of anti-Siglec5 and sialoside ligands and Sigelc5 endocytosis is clathrin and dynamin independent, requires ADP ribosylation factor 6, and traffics to lysosomes. |
| 176568 | Siglec5 | 21208791 | Siglec5 is a member of the SIGLEC protein family that recognize sialoside-based patterns and plays a role in the attenuation of innate immunity to avoid autoimmune destruction. |
| 206881 | Siglec1 | 21208791 | Siglec1 is a member of the SIGLEC protein family that recognize sialoside-based patterns and plays a role in the attenuation of innate immunity to avoid autoimmune destruction. |
| 2836 | SIGLEC15 | 21208791 | SIGLEC15 is a member of the SIGLEC protein family that recognize sialoside-based patterns and plays a role in the attenuation of innate immunity to avoid autoimmune destruction. |
| 63914 | SIGLEC11 | 21208791 | SIGLEC11 is a member of the SIGLEC protein family that recognize sialoside-based patterns and plays a role in the attenuation of innate immunity to avoid autoimmune destruction. |

| | | | |
|---|---|---|---|
| 65664 | SIGLEC9 | 21208791 | SIGLEC9 is a member of the SIGLEC protein family that recognize sialoside-based patterns and plays a role in the attenuation of innate immunity to avoid autoimmune destruction. |
| 65955 | SIGLEC8 | 21208791 | SIGLEC8 is a member of the SIGLEC protein family that recognize sialoside-based patterns and plays a role in the attenuation of innate immunity to avoid autoimmune destruction. |
| 65680 | SIGLEC7 | 21208791 | SIGLEC7 is a member of the SIGLEC protein family that recognize sialoside-based patterns and plays a role in the attenuation of innate immunity to avoid autoimmune destruction. |
| 66055 | SIGLEC6 | 21208791 | SIGLEC6 is a member of the SIGLEC protein family that recognize sialoside-based patterns and plays a role in the attenuation of innate immunity to avoid autoimmune destruction. |
| 66180 | SIGLEC5 | 19596804 | SIGLEC5 is bound by group B Streptococcus (GBS) beta protein and this functions to impair human leukocyte phagocytosis, oxidative burst, and extracellular trap production, promoting bacterial survival. |
| 66180 | SIGLEC5 | 21208791 | SIGLEC5 is a member of the SIGLEC protein family that recognize sialoside-based patterns and plays a role in the attenuation of innate immunity to avoid autoimmune destruction. |
| 47695 | SIGLEC1 | 21208791 | SIGLEC1 is a member of the SIGLEC protein family that recognize sialoside-based patterns and plays a role in the attenuation of innate immunity to avoid autoimmune destruction. |
| 65888 | SIGLEC10 | 21208791 | SIGLEC10 is a member of the SIGLEC protein family that recognize sialoside-based patterns and responds selectively to danger associated molecular patterns (DAMPs) to initiate limited innate response. |
| 140416 | Rgmb | 21187450 | Rgmb is highly expressed in macrophages and negatively regulates Il6 expression in a BMP ligand-dependent manner via MAPK and ERK pathway. |
| 35165 | RGMB | 21187450 | RGMB is highly expressed in macrophages and negatively regulates IL6 expression in a BMP ligand-dependent manner via MAPK and ERK pathway. (Demonstrated in murine model) |

| | | | |
|---|---|---|---|
| 173238 | 4432412L15Rik | 21187075 | Palm3 is a LPS inducible gene that functions as an adaptor protein for Tlr4 signaling. Palm3 interacts with Sigirr to negatively regulate TLR signaling. |
| 196327 | Oas3 | 21190483 | Oas3 is a pathogen recognition receptor for the viral pathogen-associated molecular pattern, double-stranded RNA. Upon recognition of dsRNA, the Oas3 activates Rnasel to cleave ssRNA. The Oas/Rnasel pathway triggers the RIG-I pathway and induce Ifnb production. |
| 196257 | Oas2 | 21190483 | Oas2 is a pathogen recognition receptor for the viral pathogen-associated molecular pattern, double-stranded RNA. Upon recognition of dsRNA, the Oas2 activates Rnasel to cleave ssRNA. The Oas/Rnasel pathway triggers the RIG-I pathway and induce Ifnb production. |
| 196607 | Oas1h | 21190483 | Oas1h is a pathogen recognition receptor for the viral pathogen-associated molecular pattern, double-stranded RNA. Upon recognition of dsRNA, the Oas1h activates Rnasel to cleave ssRNA. The Oas/Rnasel pathway triggers the RIG-I pathway and induce Ifnb production. |
| 196639 | Oas1g | 21190483 | Oas1g is a pathogen recognition receptor for the viral pathogen-associated molecular pattern, double-stranded RNA. Upon recognition of dsRNA, the Oas1g activates Rnasel to cleave ssRNA. The Oas/Rnasel pathway triggers the RIG-I pathway and induce Ifnb production. |
| 196587 | Oas1f | 21190483 | Oas1f is a pathogen recognition receptor for the viral pathogen-associated molecular pattern, double-stranded RNA. Upon recognition of dsRNA, the Oas1f activates Rnasel to cleave ssRNA. The Oas/Rnasel pathway triggers the RIG-I pathway and induce Ifnb production. |
| 196409 | Oas1e | 21190483 | Oas1e is a pathogen recognition receptor for the viral pathogen-associated molecular pattern, double-stranded RNA. Upon recognition of dsRNA, the Oas1e activates Rnasel to cleave ssRNA. The Oas/Rnasel pathway triggers the RIG-I pathway and induce Ifnb production. |
| 196726 | Oas1d | 21190483 | Oas1d is a pathogen recognition receptor for the viral pathogen-associated molecular pattern, double-stranded RNA. Upon recognition of dsRNA, the Oas1d activates Rnasel to cleave ssRNA. The Oas/Rnasel pathway triggers the RIG-I pathway and induce Ifnb production. |
| 196449 | Oas1c | 21190483 | Oas1c is a pathogen recognition receptor for the viral pathogen-associated molecular pattern, double-stranded RNA. Upon recognition of dsRNA, the Oas1c activates Rnasel to cleave ssRNA. The Oas/Rnasel pathway triggers the RIG-I pathway and induce Ifnb production. |

| | | | |
|---|---|---|---|
| 196543 | Oas1b | 21190483 | Oas1b is a pathogen recognition receptor for the viral pathogen-associated molecular pattern, double-stranded RNA. Upon recognition of dsRNA, the Oas1b activates Rnasel to cleave ssRNA. The Oas/Rnasel pathway triggers the RIG-I pathway and induce Ifnb production. |
| 196676 | Oas1a | 21190483 | Oas1a is a pathogen recognition receptor for the viral pathogen-associated molecular pattern, double-stranded RNA. Upon recognition of dsRNA, the Oas1a activates Rnasel to cleave ssRNA. The Oas/Rnasel pathway triggers the RIG-I pathway and induce Ifnb production. |
| 58392 | OAS2 | 21190483 | OAS2 is a pathogen recognition receptor for the viral pathogen-associated molecular pattern, double-stranded RNA. Upon recognition of dsRNA, the OAS2 activates RNASEL to cleave ssRNA. The OAS/RNASEL pathway triggers the RIG-I pathway and induce IFNB production. |
| 172850 | Ctss | 21145045 | Ctss is an endosomal and lysosomal protease that is upregulated during various inflammatory disorders. Tlr2, 3, 4 ligand engagement increases the proteolytic activities of Ctss in macrophages. |
| 172314 | Ctsb | 21145045 | Ctsb is an endosomal and lysosomal protease that is upregulated during various inflammatory disorders. Tlr2, 3, 4 ligand engagement increases the proteolytic activities of Ctsb in macrophages. |
| 7654 | CTSB | 18762176 | CTSB is a member of the Cathepsins protein family which are key modulators of cell death and inflammatory responses. |
| 7654 | CTSB | 21145045 | CTSB is an endosomal and lysosomal protease that is upregulated during various inflammatory disorders. TLR2, 3, 4 ligand engagement increases the proteolytic activities of CTSB in macrophages without changing the mRNA expression or the endogenous inhibitors of CTSB. (Demonstrated in murine model) |
| 182126 | Nlrc5 | 21148033 | Nlrc5 is dispensable for cytokine induction in virus and bacterial infections under physiologic conditions. |
| 137073 | Lair1 | 21151495 | Lair1 play an inhibition role in the mechanisms controlling Ifna production by pDCs both in normal and pathological innate immune responses. |
| 68573 | LAIR1 | 21151495 | LAIR1 play an inhibition role in the mechanisms controlling IFNA production by pDCs both in normal and pathological innate immune responses. |

| | | | |
|---|---|---|---|
| 106273 | IL19 | 21157117 | IL19 cytokine is a member of the interleukin 10 (IL10) family that includes IL20, IL22, IL24, and IL26 and has been shown to negatively regulate the innate immune system. |
| 99157 | C8B | 12220191 | C8B is the beta subunit of complement factor 8 (C8) and is one of five components that interact to form the membrane attack complex (MAC). |
| 6052 | DEFA4 | 19024344 | DEFA4 along with DEFA1 and DEFA3 are cationic alpha-defensin peptides that have multiple functions in the immune system. |
| 25037 | PIK3R1 | 17827709 | PIK3R1 (p85-alpha) is a subunit of phosphoinositide 3-kinase (PI3K) and PI3K is activated upon Toll-like receptor (TLR) ligation. |
| 25037 | PIK3R1 | 16921024 | PIK3R1 interacts with SYK during Fc-gamma receptor phagocytosis and endocytosis. |
| 30412 | NLRP10 | 15096476 | NLRP10 oligomerizes and binds to PYCARD, inhibits PYCARD mediated NF-kappaB activation and apoptosis, as well as CASP1-mediated IL1B maturation. |
| 300862 | NEU1 | 19796680 | NEU1 desialylation of sialyl alpha-2,3-linked beta-galactosyl residues of TLR4 is essential for receptor activation and cellular signaling. |
| 69692 | CARD16 | 11536016 | CARD16 is an intracellular regulator of CASP1 activation and plays a role in the regulation of IL1B secretion and NF-kappaB activation during the pro-inflammatory cytokine response. |
| 58105 | PTPN11 | 12615921 | PTPN11 is a STAT5A phosphatase that specifically interacts with STAT5A in vivo in a tyrosine phosphorylation-dependent manner, leading to the down-regulation of active STAT5A. |
| 58105 | PTPN11 | 8995399 | PTPN11 (SHP-2) phosphotyrosine phosphatase interacts with JAK tyrosine kinases to induce cytokine signal transduction. |
| 20236 | DUSP16 | 11489891 | DUSP16 mitogen activated phosphotase that negatively regulates MAPK activity and acts as a shuttle protein, determining the localization of MAPKs in the cytoplasm. |
| 100044 | BCL10 | 18806265 | BCL10 and MALT1 are essential mediators of NF-kappaB activation in response to the triggering of a diverse array of transmembrane receptors, including antigen receptors. |
| 100044 | BCL10 | 17540779 | BCL10 mediates LPS-induced activation of NF-kappaB and IL8 in human intestinal epithelial cells. |
| 89543 | NFKBIE | 11152669 | NFKBIE is part of the IkappaB family of proteins that regulates NF-kappaB-dependent transcription by inhibiting DNA binding and localizing these factors to the cell cytoplasm, specifically by sequestering REL and RELA in the cytoplasm and inhibiting nuclear export. |
| 102040 | OTUD7B | 18178551 | OTUD7B is a deubiquitinating enzyme that participate in the resolution of inflammatory responses by suppressing NF-kappaB. |

| | | | |
|---|---|---|---|
| 79667 | CFB | 17921140 | CFB is a crucial catalytic component of the C3 convertase enzyme that activates the alternative pathway of complement-mediated immunity. |
| 28713 | ISG20 | 15064795 | ISG20 is an IFN-induced 3'-5' exonuclease that is strongly induced by viral double stranded RNA and is activated by RELA/NFKB1. |
| 74884 | IL2RG | 8041779 | IL2RG physically associates with JAK2, suggesting that regulation of JAK2 may be linked to IL2 induced signal transduction. |
| 35720 | MST1R | 18684919 | MST1R regulates endotoxin-induced innate immune responses by regulating the production of and response to IFNG (IFN-gamma). |
| 14155 | ADAM10 | 18981120 | ADAM10 associates with tetraspanins and regulates cleavage of TNF (TNF-alpha) and EGF (epidermal growth factor). |
| 63432 | MAP3K3 | 18206350 | MAP3K3 in its unphosphorylated form can form a complex with unphosphorylated MAP3K7 (TAK1) where non-phosphorylated MAP3K7 interferes with MAP3K3 phosphorylation and NF-kappaB reporter activity. |
| 22073 | NKIRAS1 | 15024091 | NKIRAS1, in both GDP-bound and GTP-bound states, inhibits phosphorylation of NFKBIB by IKBKB and makes it resistant to degradation. |
| 64886 | KLK1 | 18577888 | KLK1 is part of the kallikrein-kinin system (KKS) that is involved in cancer and angiogenesis. |
| 64886 | KLK1 | 18725990 | KLK1 is involved in the enzymatic cascade known as the contact system in the blood which leads to an inflammatory response. |
| 15857 | C1S | 16177097 | C1S associates with C1R and C1Q to form the first component (C1) of the classical complement pathway. |
| 15857 | C1S | 16177097 | C1S is the modular serine protease responsible for cleavage of C4 and C2, the protein substrates for the first component (C1). |
| 25572 | PPP4C | 18634786 | PPP4C negatively regulates lipopolysacharide (LPS)-induced and TRAF6-mediated NF-kappaB activation by inhibiting the ubiquitination of TRAF6. |
| 22000 | MAP3K1 | 18984593 | MAP3K1 and TRAF6 play a pivotal role in the retinoic-acid-inducible gene-I (RIG-I)-like helicase antiviral pathway. |
| 105426 | PTGS2 | 18955820 | PTGS2 (COX-2) is suppressed through inhibiting the NF-kappaB activation by LPS and this may be associated with the anti-inflammatory effects of L. casei on Raw264.7 cells. |
| 52534 | KPNA1 | 16298512 | KPNA1 (IPOA5) regulates the import of STAT proteins into the nucleus through nuclear pores. |
| 52534 | KPNA1 | 12740372 | KPNA1 contains a nuclear localization signal binding site for STAT1 homodimers, STAT1-STAT2 heterodimers and influenza A virus nucleoprotein. |
| 92880 | C8G | 11058761 | C8G is the gamma subunit of complement factor 8 (C8) and is one of five components that interact to form the membrane attack complex (MAC). |

| | | | |
|---|---|---|---|
| 239499 | LAT | 12857726 | LAT dephosphorylation in human platelets is integrin-mediated and involves PTPN1 protein tyrosine phosphatase and both PTPN1 activation and LAT dephosphorylation processes were involved in the control of irreversible platelet aggregation upon FcgammaRIIa stimulation. |
| 15930 | C1R | 11445589 | C1R is the protease that mediates activation of the classical complement C1 complex and this catalytic property is mediated by C1R C-terminal region, comprising two complement control protein (CCP) modules followed by a serine protease (SP) domain. |
| 104095 | LY9 | 18501771 | LY9 (SLAMF3) is part of the SLAM and SAP gene families that control innate and adaptive immune responses. |
| 161037 | Ptch1 | 21131441 | Ptch is a member of the Hedgehog signaling pathway and is important for LPS-induced inflammatory response in macrophages. |
| 61793 | CFP | 18579773 | CFP binds to early apoptotic T cells and initiates complement activation, leading to C3B opsonization and ingestion by phagocytic cells. |
| 26804 | ICAM1 | 10924857 | ICAM1 is an inducible surface glycoprotein belonging to the immunoglobulin superfamily that is involved in a wide range of inflammatory and immune responses. |
| 26804 | ICAM1 | 3349522 | ICAM1 binds to ITGAL/ITGB2 (CD11a/CD18) and ITGAM/ITGB2 (CD11b/CD18) expressed by leukocytes and promotes their adhesion and transendothelial migration. |
| 299069 | HLA-E | 19000151 | HLA-E is a non-classical major histocompatibility complex (MHC) class I molecule that is the least polymorphic of all the MHC class I molecules and acts as a ligand for receptors of both the innate and the adaptive immune systems. |
| 47191 | EGR1 | 15545275 | EGR1 is an immediate early gene that is up-regulated by a multitude of growth factors, cytokines and environmental stresses to regulate LPS-induced SOCS1 transcription. |
| 21516 | IL6ST | 10829066 | IL6ST (GP130) is a common cytokine receptor subunit that is preferentially bound by SOCS3 on its SHP2-binding site, suggesting that some of the negative regulatory roles previously attributed to the phosphatase SHP-2 might in fact be caused by the action of SOCS3. |
| 31432 | IGF1R | 10747872 | IGF1R is able to mediate activation of STAT3 in vitro and in vivo and the JAKs are essential for this process of activation. |
| 2565 | ELP2 | 10954736 | ELP2 is a STAT3-interacting protein that regulates cytokine signal transduction and may regulate the activation of STAT3. |
| 29585 | F2RL2 | 18264801 | F2RL2 is able to signal autonomously to induce IL8 release mediated by ERK1/2 phosphorylation, which contributes actively to inflammatory responses. |

| | | | |
|---|---|---|---|
| 35882 | F2RL3 | 11907122 | F2RL3 modulates inflammation in the lung where its activation stimulates IL6, IL8, and PTGER2 (prostaglandin E2) release from human respiratory epithelial cells. |
| 14014 | CD27 | 9582383 | CD27 is a member of the tumour necrosis factor receptor superfamily that activates NF-kappaB and MAPK9 (stress-activated protein kinase/c-Jun N-terminal kinase) via TRAF2, TRAF5 and MAP3K14 (NIK). |
| 10490 | HOXA9 | 18981407 | HOXA9 plays a role in the innate immune response to bacterial infection as a modulator of NF-kappaB-dependent transcription. |
| 60718 | GSTP1 | 18962899 | GSTP1 suppresses LPS (lipopolysaccharide)-induced excessive production of pro-inflammatory factors by inhibiting LPS-stimulated MAPKs (mitogen-activated protein kinases) as well as NF-kappaB activation. |
| 29281 | EPOR | 8068943 | EPOR, a member of the cytokine receptor superfamily, associates with the JAK2 protein tyrosine kinase upon erythropoietin stimulation, inducing tyrosine phosphorylation of cellular substrates, including the EPOR, to transduce a growth signal. |
| 88472 | WDR34 | 19521662 | WDR34 is a MAP3K7 (TAK1)-associated inhibitor of the IL-1R/TLR3/TLR4-induced NF-kappaB activation pathway. |
| 24566 | ACAP1 | 17005562 | ACAP1 (CENTB1) selectively down-regulates NF-kappaB activation via NODs pathways, creating a feedback loop and suggesting a novel role of ACAP1 in innate immune responses to bacteria and inflammatory responses. |
| 34697 | ERAP1 | 18445477 | ERAP1 associates with RBMX and a heterogeneous nuclear ribonucleoprotein to regulate both the constitutive release of TNFR1 exosome-like vesicles and the inducible proteolytic cleavage of TNFR1 ectodomains. |
| 25238 | CXCL11 | 21124994 | CXCL11 exert direct antimicrobial effects in vitro against Bacillus anthracis spore and bacilli in a receptor-independent manner and contributes to pulmonary innate immunity. (Demonstrated in murine model) |
| 179944 | Cxcl11 | 21124994 | Cxcl11 exert direct antimicrobial effects in vitro against Bacillus anthracis spore and bacilli in a receptor-independent manner and contributes to pulmonary innate immunity. |
| 25111 | CXCL9 | 21124994 | CXCL9 exert direct antimicrobial effects in vitro against Bacillus anthracis spore and bacilli in a receptor-independent manner and contributes to pulmonary innate immunity. (Demonstrated in murine model) |

| | | | |
|---|---|---|---|
| 179870 | Cxcl9 | 21124994 | Cxcl9 exert direct antimicrobial effects in vitro against Bacillus anthracis spores and bacilli in a receptor-independent manner and contributes to pulmonary innate immunity. |
| 30467 | AP3B1 | 21045126 | AP3B1 (AP-3), a lysosome-related organelle trafficking and biogenesis protein, is required for the production of pro-inflammatory cytokines in plasmacytoid dendritic cells upon recognition of viral nucleic acids by endosomal TLR7 or TLR9. |
| 30467 | AP3B1 | 21119105 | AP3B1 is crucial for the trafficking of TLR9 to specific endosomal compartments for the induction of type I interferon. |
| 169560 | S100a10 | 21115493 | S100a10 :: Anxa2 is a key profibrinolytic complex that assembles plasminogen and tissue plasminogen activator, and promotes plasmin generation. As a negative feedback regulation, plasmin can induce disassociation of the heterotetramer and ubiquitin-mediated degradation of S100a10. |
| 102454 | S100A10 | 21115493 | S100A10 :: ANXA2 is a key profibrinolytic complex that assembles plasminogen and tissue plasminogen activator, and promotes plasmin generation. As a negative feedback regulation, plasmin can induce disassociation of the heterotetramer and ubiquitin-mediated degradation of S100A10. |
| 181662 | Anxa2 | 21115493 | Anxa2 :: S100a10 is a key profibrinolytic complex that assembles plasminogen and tissue plasminogen activator, and promotes plasmin generation. As a negative feedback regulation, plasmin can induce serine phosphorylation of Anxa2 and prevents the translocation to cell surface. |
| 14711 | ANXA2 | 19965653 | ANXA2 is found in a heterotetrameric complex with S100A10, serving as a key extracellular binding partner for pathogens and host proteins alike and can also can be shed or secreted. ANXA2 tetramer can activate human and murine macrophages through TLR4 demonstrating an important role for ANXA2 in the detection of danger to the host, whether from injury or invasion. |
| 14711 | ANXA2 | 21115493 | ANXA2 :: S100A10 is a key profibrinolytic complex that assembles plasminogen and tissue plasminogen activator, and promotes plasmin generation. As a negative feedback regulation, plasmin can induce serine phosphorylation of ANXA2 and prevents the translocation to cell surface. |

| | | | |
|---|---|---|---|
| 54381 | CEACAM1 | 18836450 | CEACAM1 inhibits TLR2 induced pro-inflammatory immune responses by inducing PTPN6-(SHP-1)-mediated inhibition of the PI3K-NF-kappaB signal transduction pathway in pulmonary epithelial cells. |
| 156030 | Tlr11 | 19801549 | Tlr11 expression in epithelial cells is regulated by epithelium-specific Ets factors, ELF3 (ESE-1), EHF (ESE-3), via its Ets element and by IRF8 through its IRF motif. |
| 156030 | Tlr11 | 21097503 | Tlr11 is an intracellular receptor in the endoplasmic reticulum, and acts as an innate sensor for Toxoplasma protein profilin. |
| 83434 | IL13RA1 | 21097505 | IL13RA1 is a receptor for IL13. Upon ligand engagement, IL13RA1 activates the transcription factor STAT6 to induce M2 expression profile in macrophages. |
| 197114 | Il23a | 21097874 | Il23a is a LPS induced gene, and its expression in macrophages correlate with the severity of chronic intestinal inflammation. Il23a is transcriptionally inhibited by the binding of Irf1 to the ISRE element and serve as a homeostatic checkpoint in chronic intestinal inflammation. |
| 135492 | Sema3a | 21098092 | Sema3a is a ligand for Plxna4, and the overexpression of Sema3a exacerbate cytokine storm caused by Tlr agonist and bacterial sepsis. The expression of Sema3a can be induced by Tlr engagement, and thus completes an autocrine loop. |
| 24546 | SEMA3A | 21098092 | SEMA3A is a ligand for PLXNA4, and the overexpression of SEMA3A exacerbate cytokine storm caused by TLR agonist and bacterial sepsis. The expression of SEMA3A can be induced by TLR engagement, and thus completes an autocrine loop. |
| 134005 | Plxna4 | 21098092 | Plxna4 is a receptor protein and its expression in macrophages is required for optimal cytokine production upon Tlr stimulation. Plxna4 is a crucial component of sepsis-induced cytokine storm by activating Rac1, Mapk8 and Nfkb. |
| 281690 | PLXNA4 | 21098092 | PLXNA4 is a receptor protein and its expression in macrophages is required for optimal cytokine production upon TLR stimulation. PLXNA4 is a crucial component of sepsis-induced cytokine storm by activating RAC1, MAPK8 and NFKB. |
| 33873 | CASP6 | 21098228 | CASP6 plays an important role in the activation of alveolar macrophages by neutrophils. The cleavage of IRAK3 by CASP6 mediates the degradation of NFKBIA and the induction of TNF upon stimulation by bacterial products. |
| 138822 | Defa20 | 21099205 | Defa20 is a mouse paneth cell alpha-defensins that has selective bactericidal activities against intestinal microbiota and these activities are dependent on the disulfide bonds. |

| | | | |
|---|---|---|---|
| 7478 | PELI2 | 17997719 | PELI2 and other pellino isoforms are the E3 ubiquitin ligases that mediate the IL-1-stimulated formation of K63-pUb-IRAK1 in cells, which may contribute to the activation of IKBKB and NF-kappaB, as well as other signalling pathways dependent on IRAK1 and IRAK4. |
| 126853 | hsa-mir-126 | 19843690 | Hsa-mir-126 antagonism suppresses the effector function of TH2 cells and the development of allergic airways disease. |
| 44260 | FFAR2 | 19865172 | FFAR2 binding of short chain fatty acids (SCFAs) provides a molecular link between diet, gastrointestinal bacterial metabolism, and immune and inflammatory responses. |
| 52872 | NR1H4 | 19864602 | NR1H4 is an essential component of a network of nuclear receptors that regulate intestinal innate immunity and homeostasis. |
| 126843 | hsa-mir-98 | 19592657 | Hsa-mir-98 and hsa-let-7e confer cholangiocyte expression of CISH in response to microbial challenge, a process that may be relevant to the regulation of TLR-mediated epithelial innate immune response. |
| 37175 | CISH | 19592657 | CISH is a negative regulator for inflammatory cytokine signalling that enhances NFKBIA (IKappaB-alpha) degradation and regulation and activates NF-kappaB in cholangiocytes in response to LPS stimulation. |
| 37175 | CISH | 19592657 | CISH expression in cholangiocytes is conferred by hsa-mir-98 and hsa-let-7e in response to microbial challenge, a process that may be relevant to the regulation of TLR-mediated epithelial innate immune response. |
| 126519 | hsa-let-7e | 19592657 | Hsa-let-7e and hsa-mir-98 confer cholangiocyte expression of CISH in response to microbial challenge, a process that may be relevant to the regulation of TLR-mediated epithelial innate immune response. |
| 88858 | HMGB3 | 19890330 | HMGB3 functions as a universal sentinel for nucleic-acid-mediated innate immune responses. |
| 44657 | HMGB2 | 19890330 | HMGB2 functions as a universal sentinel for nucleic-acid-mediated innate immune responses. |
| 22673 | SIVA1 | 19584092 | SIVA1 diminishes NFkappaB and enhances JNK activity to favour apoptosis by physically interacting with MAP3K7 (TAK1) and XIAP. |
| 22673 | SIVA1 | 19392652 | SIVA1 promotes K-48 polyubiquitination of TRAF2 and inhibits TCR-mediated activation of NF-kappaB. |
| 7946 | SNAP23 | 18692471 | SNAP23 phosphorylation by IKBKB regulates mast cell degranulation and anaphylactic reactions. |
| 46310 | IRF2 | 18514056 | IRF2 is a transcriptional repressor that functions by competing with the transcriptional activator IRF1 and has also been shown to functions as a transcriptional activator for several genes. IRF2 transcriptional activity is regulated by sumoylation whereby covalent modification of IRF2 by SUMO1 results in enhanced transcriptional repression activity on IRF1. |

| | | | |
|---|---|---|---|
| 20740 | PLK1 | 18957422 | PLK1 is a IKKgamma/NEMO-binding domain (gammaBD) kinase, which negatively regulates TNF-induced IKK activation and cyclin D1 expression. |
| 83153 | TRAF1 | 19698991 | TRAF1 is a positive regulator of the NF-kappaB alternative pathway. |
| 83153 | TRAF1 | 19540595 | TRAF1 mediates both the CD30 signaling-dependent and independent NF-kappaB activation, which prevents lymphoma cells from spontaneous and induced apoptosis. |
| 83153 | TRAF1 | 18429822 | TRAF1 phosphorylation by the ubiquitously expressed kinase PKN1 plays a critical role in the negative regulation of tonic activity of JNK and NF-kappaB signalling pathways. |
| 33268 | PKN1 | 18429822 | PKN1 is a ubiquitously expressed kinase that, when phosphorylated by TRAF1, plays a critical role in the negative regulation of tonic activity of JNK and NF-kappaB signalling pathways. |
| 66609 | FXR1 | 19864602 | FXR1 is an essential component of a network of nuclear receptors that regulate intestinal innate immunity and homeostasis. |
| 24405 | ERBB2IP | 16203728 | ERBB2IP ccts as a negative regulator of the MDP-mediated activation of NF-kappaB by NOD2. |
| 24405 | ERBB2IP | 16203728 | ERBB2IP overexpression inhibits NOD2-dependent cytokine secretion in mouse embryonic fibroblasts. |
| 37270 | RBCK1 | 19136968 | RBCK1 (LUBAC) complexes with RNF31 and is involved in the physiological regulation of the canonical NF-kappaB activation pathway through linear polyubiquitylation of IKBKG. |
| 3579 | RNF31 | 19136968 | RNF31 complexes with RBCK1 (LUBAC) and is involved in the physiological regulation of the canonical NF-kappaB activation pathway through linear polyubiquitylation of IKBKG. |
| 18826 | PIAS1 | 12855578 | PIAS1 and other PIAS proteins have been shown to function as E3-type small ubiquitin-like modifier (SUMO) ligases, and sumoylation is a modulatory mechanism for several transcription factors. |
| 18826 | PIAS1 | 19136629 | PIAS1 is a protein inhibitor of activated STAT1 and arginine methylation of PIAS1 is essential for the repressive function of PRMT1 in interferon (IFN)-dependent transcription as well as for the recruitment of PIAS1 to STAT1 target gene promoters in the late phase of the IFN response. |
| 46287 | WDR62 | 19910486 | WDR62 is recruited to stress granules and mediates a non-classical MAPK8 (JNK) activation. |
| 83597 | DAB2IP | 19948740 | DAB2IP functions as ARF6-GTPase activating protein to negatively regulate phosphatidylinositol 4, 5-bisphosphate (PIP2)-dependent TLR4-TIRAP-MyD88 signaling. |

| | | | |
|---|---|---|---|
| 39983 | YJEFN3 | 15753091 | YJEFN3 is a downstream effector of anti-bacterial function in intestinal epithelial cells that is required for NOD2-mediated NF-kappaB activation following NOD2 mediated recognition of bacterial muramyl dipeptide (MDP). |
| 39983 | YJEFN3 | 12163600 | YJEFN3 (NDUFA13) interacts with viral interferon regulatory factor 1 of Kaposi's sarcoma-associated herpesvirus and inhibits interferon/retinoic acid-induced cell death. |
| 39983 | YJEFN3 | 17928352 | YJEFN3 belongs to the family of genes associated with retinoid-interferon mortality and serves as an essential component of the oxidative phosphorylation system. YJEFN3 interacts with human herpesvirus 6B U95 protein and the resulting silencing of U95 expression reduces viral load and abrogates loss of mitochondrial membrane potential. |
| 39983 | YJEFN3 | 17823279 | YJEFN3 blocks SRC-induced gene expression through STAT3 and activation of cell adhesion molecules. |
| 101319 | RP5-1000E10.4 | 16281057 | RP5-1000E10.4 (SIKE) is a physiological suppressor of IKKepsilon and TBK1 and plays an inhibitory role in virus- and TLR3-triggered IRF3 but not NF-kappaB activation pathways. |
| 204202 | Trim30 | 18345001 | Trim30 negatively regulates TLR mediated NF-kappaB activation by targeting Map3k7ip2 (TAB2) and Map3k7ip3 (TAB3) for degradation. |
| 23171 | AZI2 | 17142768 | AZI2 participates in both the TLR3-mediated and the cytoplasmic DDX58 (RIG-I) dsRNA recognition pathways in type-1 interferon (IFN) induction by binding to MAVS, DDX58 and IFIH1 (MDA5). |
| 53967 | MAP3K7IP3 | 15327770 | MAP3K7IP3 (TAB3) and MAP3K7IP2 (TAB2) activate the NF-kappaB pathway through binding to K63-linked polyubiquitin chains. |
| 53967 | MAP3K7IP3 | 14670075 | MAP3K7IP3 (TAB3) and MAP3K7IP2 (TAB2) phosphorylation is mediated by SAPK2a/p38alpha and this contributes to the SAPK2a/p38alpha-mediated feedback control of MAP3K7 (TAK1) activity that also involves the phosphorylation of MAP3K7IP1 (TAB1). |
| 53967 | MAP3K7IP3 | 14633987 | MAP3K7IP3 (TAB3) and MAP3K7IP2 (TAB2) function redundantly as mediators of MAP3K7 (TAK1) activation in IL-1 and TNF signal transduction. |
| 34038 | GLRX | 21078302 | GLRX (GRX-1), a degluationylation enzyme, activates TRAF6 and is responsible for IL1R / TLR-dependent induction of NFkB pathway. |
| 7998 | BEC3G;APOB | 21078663 | APOBEC3G;APOBEC3F (A3G) is an innate restriction factor that inhibits human immunodeficiency virus type 1 (HIV-1) replication. |
| 127645 | hsa-mir-152 | 21068402 | Hsa-mir-152, hsa-mir-148a, and hsa-mir-148b, are negative regulators of the innate response and Ag-presenting capacity of dendritic cells (DCs), which may contribute to the immune homeostasis and immune regulation. |

| | | | |
|---|---|---|---|
| 126249 | hsa-mir-148b | 21068402 | Hsa-mir-148b, hsa-mir-148a, and hsa-mir-152, are negative regulators of the innate response and Ag-presenting capacity of dendritic cells (DCs), which may contribute to the immune homeostasis and immune regulation. |
| 126833 | hsa-mir-148a | 21068402 | Hsa-mir-148a, hsa-mir-148b, and hsa-mir-152, are negative regulators of the innate response and Ag-presenting capacity of dendritic cells (DCs), which may contribute to the immune homeostasis and immune regulation. |
| 49131 | TRAT1 | 21074459 | TRAT1 (TRIM56) is an interferon-inducible E3 ubiquitin ligase that modulates TMEM173 (STING) to confer double-stranded DNA-mediated innate immune responses. |
| 29671 | F2R | 17977790 | A coagulation factor II (thrombin) receptor belonging to a family of G protein-coupled receptors, protease-activated receptors (PARs), and during inflammation, microorganisms as well as host immune cells release various proteases activating protease-activated receptors (PARs) |
| 29671 | F2R | 11907122 | Activation of F2R stimulates IL6, IL8, and PTGER2 (prostaglandin E2) release from human respiratory epithelial cells |
| 29671 | F2R | 21029417 | F2R (PAR1) activates MAPK14 (p38) and MAPK3/1 (ERK1/2) kinases to trigger production of innate immunity markers in oral kertainocytes. |
| 201520 | Slc15a4 | 21045126 | Slc15a4, a peptide/histidine transporter in organelle trafficking, is required for the production of pro-inflammatory cytokines in plasmacytoid dendritic cells upon recognition of viral nucleic acids by endosomal Tlr7 or Tlr9. |
| 101961 | FCGR1A | 21044955 | FCGR1A (FcgammaRI) is a receptor that recognizes IgG opsonized particles and initiates phagocytosis in immune clearance. FCGR1A is also a negative regulator of complement-mediated phagocytosis. |
| 236915 | UBD | 19959714 | UBD mediates NF-kappaB activation and may promote tubulointerstitial inflammation in chronic kidney diseases. |
| 67366 | CD300E | 20039296 | CD300E functions as an activating receptor capable of regulating the innate immune response in myeloid cells. |
| 65194 | PRKCA | 19950169 | PRKCA is a key component that controls MyD88-dependent cytokine gene expression in human and mouse but differentially regulates production of TICAM1 (TRIF)-dependent cytokines. |
| 78470 | CASP10 | 17822854 | CASP10 is an initiator caspase in the death receptor (DR)-dependent apoptotic pathway with multiple identified splice variants. The prodomain-only isoform of CASP10, Caspase-10g, may play a regulatory role in the NF-kappaB pathways. |

| | | | |
|---|---|---|---|
| 106452 | IRF6 | 12219090 | IRF6 belongs to a family of transcription factors that share a highly conserved helix-turn-helix DNA-binding domain and a less conserved protein-binding domain. |
| 236583 | IRF9 | 9242679 | IRF9 is a DNA-binding protein that is a member of the alpha-interferon-induced ISGF3 complex. Bipartite complexes of STAT2:IRF9 and STAT2:STAT1 translocate to the nucleus and associate on DNA target sites as ISGF3. |
| 236583 | IRF9 | 15194680 | IRF9 functions to recruit RNA polymerase II to the promoter of interferon (IFN)-stimulated genes and this function of IRF9 requires the activity of histone deacetylases. |
| 57716 | VASP | 14679200 | VASP is an important component of the cellular microfilament system that plays a major role in the regulation of serum response element (SRE)-dependent transcription. VASP activity is regulated by GMP-dependent protein kinase (G-kinase). |
| 16232 | IFITM1 | 20064371 | IFITM1 is an antiviral restriction factor that mediates cellular resistance to influenza A H1N1 virus, West Nile virus, and dengue virus by inhibiting their early replication. |
| 16199 | IFITM2 | 20064371 | IFITM2 is an antiviral restriction factor that mediates cellular resistance to influenza A H1N1 virus, West Nile virus, and dengue virus by inhibiting their early replication. |
| 301516 | C4B | 2650988 | C4B is one of two isotypes of the fourth component of complement. |
| 301516 | C4B | 12440962 | C4B assembles with C2A to form the C3/C5 convertase that goes on to cleave complement C5. |
| 33417 | RAD21 | 12198550 | RAD21 is part of the cohesin complex which is the cellular machinery involved in sister chromatid cohesion and that which requires access to the nucleosomal DNA to perform its function in chromosome segregation. |
| 26684 | RASGEF1B | 20090772 | RASGEF1B is a guanine-nucleotide exchange factor (GEF), whose expression is induced in macrophages on stimulation with toll-like receptor (TLR) agonists. |
| 93355 | NOXA1 | 20110267 | NOXA1 together with NADPH oxidase organizer 1 (NOXO1) are key regulatory subunits of the NADPH oxidase NOX1, the activity of which is regulated through MAP kinase (MAPK), protein kinase C (PKC), and protein kinase A (PKA)-dependent phosphorylation on Ser-282 and Ser-172 of NOXA1. |
| 32212 | TRPV2 | 20118928 | TRPV2 has fundamental importance in innate immunity by participating in macrophage particle binding and early phagocytosis. |
| 46334 | CYBA | 20142487 | CYBA is an NADPH oxidase and CYBA-dependent reactive oxygen species (ROS) are key regulators of neutrophil chemotactic migration. |

| | | | |
|---|---|---|---|
| 33289 | CALCA | 20141542 | CALCA can be produced by immune cells such as monocytes/macrophages following inflammatory stimulation and has a positive or negative reciprocal effect on the production of other pro- and anti-inflammatory mediators, playing both facilitating and suppressing roles in immune and inflammatory responses as shown in mice. |
| 31818 | PPP3CA | 17965024 | PPP3CA (calcineurin) is a serine/threonine phosphatase that is activated by calcium and calmodulin that promotes HIF1A expression by dephosphorylating RACK1 and blocking RACK1 dimerization. |
| 247106 | KIR3DL2 | 20147700 | KIR3DL2 functions as a CpG oligodeoxynucleotide (ODN) receptor at the cell surface, facilitating the encounter of CpG ODN with TLR9 in early endosomes. |
| 54466 | OPTN | 20174559 | OPTN (optineurin) has a role in the inhibition of virus-triggered IFNB1 induction. |
| 3218 | IL25 | 20200520 | IL25, a member of the IL17 cytokine family, promotes the accumulation of a lineage-negative multipotent progenitor (MPP) cell population in the gut-associated lymphoid tissue that promotes T(H)2 cytokine responses, presenting an innate immune pathway that promotes T(H)2 cytokine responses at mucosal sites. |
| 49542 | LGALS4 | 20154696 | LGALS4 (Gal-4), expressed in the intestinal tract, recognizes and kills human blood group antigen-expressing Escherichia coli while failing to alter the viability of other E. coli strains or other Gram-negative or Gram-positive organisms. |
| 49542 | LGALS4 | 20208507 | LGALS4 is part of the galectin family of proteins that have emerged as autonomous bacteria-killing agents, pointing to a principal role of these proteins in innate immunity. |
| 107605 | LGALS8 | 20154696 | LGALS8 (Gal-8), expressed in the intestinal tract, recognizes and kills human blood group antigen-expressing Escherichia coli while failing to alter the viability of other E. coli strains or other Gram-negative or Gram-positive organisms. |
| 107605 | LGALS8 | 20208507 | LGALS8 is part of the galectin family of proteins that have emerged as autonomous bacteria-killing agents, pointing to a principal role of these proteins in innate immunity. |
| 8115 | RAC1 | 20167866 | RAC1 cooperates with TLR2, MyD88, and PI3K in lipoteichoic acid-induced cPLA2/COX-2-dependent airway inflammatory responses. |
| 62191 | IL31 | 20190140 | IL31 is a pruritogenic cytokine in human mast cells and its secretion is induced by antimicrobial peptides human beta-defensins and cathelicidin LL-37. |

| | | | |
|---|---|---|---|
| 26912 | RSAD2 | 20308629 | RSAD2 (viperin) is an antiviral protein whose expression is highly upregulated during viral infections via IFN-dependent and/or IFN-independent pathways and an IRF1 mediated type I IFN independent mechanism of enhanced RSAD2 expression provides a redundant mechanism to protect cells from viral infections. |
| 106549 | ATF3 | 16688168 | ATF3 is induced by lipopolysaccharide (LPS) and regulates TLR-stimulated inflammatory responses as part of a negative-feedback loop. |
| 29776 | HERC5 | 20308324 | HERC5 positively regulates the innate antiviral responses by sustaining IRF3 activation via a novel post-translational modification, ISGylation. |
| 91819 | FCN2 | 20375620 | FCN2, as well as ficolins FCN1 and FCN3, in serum are associated with MBL-associated serine protease (MASP) to form a complex and this complex binds to carbohydrates present on the surface of a variety of Gram-positive and Gram-negative bacteria through ficolin, initiating complement activation via the lectin pathway. |
| 10154 | DUOX1 | 18511861 | DUOX1 and DUOX2 localize to the apical plasma membrane of epithelial cells in major airways, salivary glands, and the gastrointestinal tract, and provide extracellular hydrogen peroxide to lactoperoxidase to produce antimicrobial hypothiocyanite ions. Expression of dual oxidases DUOX2 and DUOX1 is regulated by Th1 and Th2 cytokines in human airways. |
| 67316 | NOX4 | 18511861 | NOX4 is implicated in innate immunity since lipopolysaccharide (LPS) induces NOX4-dependent reactive oxygen species (ROS) generation. |
| 163644 | Abcg1 | 20395559 | Abcg1 regulates innate immunity in a tissue-selective manner and Abcg1(-/-) mice have an enhanced pulmonary host defense response driven predominantly by hematopoietic cells. |
| 22229 | LYN | 20385881 | The Lyn/PI3K module negatively regulates activation of murine macrophages while Inpp5d (SHIP-1) promotes it. |
| 178553 | Inpp5d | 20385881 | Inpp5d promotes while the Lyn/PI3K module negatively regulates activation in murine macrophages viaTlr2 and Tlr4 receptors. |
| 83441 | INPP5D | 20100929 | INPP5D (SHIP-1) is a critical negative regulator of IFN-beta production downstream of TLR3 through the regulation of TBK1 localization and activity. |
| 83441 | INPP5D | 20154203 | INPP5D is a negative regulator of GM-CSF-derived dendritic cell (DC) generation but a positive regulator of GM-CSF-derived DC maturation and function. |
| 87405 | CNPY3 | 18780723 | CNPY3 differentially interacts with TLR2, TLR4, and TLR9 and a single-nucleotide change in the CNPY3 gene can influence the strength of TLR responses and may also alter the relative activity of each TLR. |

| | | | |
|---|---|---|---|
| 87405 | CNPY3 | 17998391 | CNPY3 (PRAT4A) is required for TLR-dependent immune responses where it regulates the subcellular distribution and response of multiple TLRs, required for both innate and adaptive immune responses. |
| 70554 | BIRC5 | 20406824 | BIRC5 is cleaved by GZMM (granzyme M) and this triggers degradation of the BIRC5-XIAP complex to free caspase activity, leading to cytolysis of target cells. |
| 99054 | DHCR24 | 20406300 | DHCR24 (Seladin1) is a novel lipopolysaccharide (LPS)-responsive gene and inhibits the tumour necrosis factor-alpha production and osteoclast formation in response to LPS. DHCR24 is an LPS-responsible gene product that negatively regulates the LPS-induced inflammatory response. |
| 138962 | Gpr33 | 20399748 | Gpr33 is highly expressed in murine dendritic cells and its expression is regulated by the activity of toll-like receptors (TLR) and AP-1/NF-kappaB signaling pathways in cell culture and in vivo. |
| 231020 | GPR33 | 20399748 | GPR33 is an orphan member of the chemokine-like receptor family and is a pseudogene in most humans. |
| 80070 | SFTPA2 | 20413160 | SFTPA2 is a carbohydrate pattern recognition molecule of innate immunity, that significantly enhances phagocytosis and killing of Aspergillus fumigatus, a pathogenic fungus, by neutrophils and macrophages. |
| 97020 | IFNGR1 | 16785527 | IFNGR1, along with IFNGR2, are the receptor subunits for IFN-gamma that upon ligand binding, translocate to the nucleus together with STAT1-alpha, and associate with IFN-gamma-activated sequence (GAS) elements at the promoter sites of IFN-gamma-activated genes. |
| 2604 | MIF;SLC2A11 | 19155217 | MIF;SLC2A11 interacts with ribosomal protein S19 (RPS19) leading to attenuation of MIF pro-inflammatory function by inhibition of the MIF-CD74 interaction and MIF triggered adhesion of monocytes. |
| 2604 | MIF;SLC2A11 | 18034423 | MIF;SLC2A11 is a regulator of innate immunity and inflammation by having an important role in pro-inflammatory macrophage responses. Transcription factors specificity protein 1 (SP1) and cAMP response element-binding protein (CREB1) are critical positive regulators of its constitutive gene expression. |
| 27975 | ILF3 | 18490444 | ILF3 is an RNA-binding protein that influences mRNA turnover and/or translation by regulating mRNA stability. ILF3 can bind to mitogen-activated protein (MAP) kinase phosphatase 1 (MKP-1) and increase its mRNA stability and translation, resulting in increased MKP-1 dephosphorylation activity and thereby inactivation of MAP kinases extracellular signal-regulated kinase (ERK), c-Jun N-terminal kinase (JNK), and p38. |

| | | | |
|---|---|---|---|
| 81394 | ATG9A | 19926846 | ATG9A is an essential autophagy protein that functions as a regulator of innate immunity following double stranded DNA stimulation by controlling dsDNA-driven dynamic translocation of stimulator of IFN genes (STING), aka TMEM173, and TBK1. |
| 3922 | MX2 | 18062906 | MX2 is part of the Mx GTPase family of protein that are interferon-induced members of the dynamin superfamily of large GTPases, which inhibit a wide range of viruses by blocking an early stage of the replication cycle. |
| 8619 | TPSB2 | 20427273 | TPSB2 (tryptase) expression is modulated by IL33, a novel pathway by which mesenchymal cells exposed to inflammatory cytokines modulate the phenotype of local MCs to shape their immune responses. |
| 39257 | GAB1 | 20435932 | GAB1 inhibits vesicular stomatitis virus (VSV) replication and VSV infection-induced cell damage by inducing type I IFNs and IFN-inducible gene expression via the PI3K/Akt pathway. It is needed for full activation of TLR3/4- and RIG-I-triggered innate responses by promoting activation of PI3K/Akt, MAPKs, and NF-kappaB pathways. |
| 104073 | SLAMF1 | 18501771 | SLAMF1 is one of nine SLAM-family genes, a subfamily of the immunoglobulin superfamily, that encode differentially expressed cell-surface receptors of hematopoietic cells. SLAM and SLAM-associated protein (SAP) gene families control innate and adaptive immune responses |
| 104073 | SLAMF1 | 19079134 | SLAMF1 is one of nine SLAM-family genes and SLAM receptors and SLAM-associated proteins (SAPs) influence lymphocyte interactions, development and function. |
| 95826 | GOPC | 16878151 | GOPC (cystic fibrosis transmembrane regulator-associated ligand) interacts with human papillomavirus type 16 E6 protein to promote E6-associated protein (E6AP)-mediated ubiquitination and proteasomal degradation. |
| 14629 | YWHAE | 20462248 | YWHAE (14-3-3 epsilon) interacts with key components of mitogen-activated protein kinase (MAPK)signal module for selective modulation of the TNF-alpha-induced time course-dependent NF-kappaB activity. |
| 236774 | LTB4R | 20959460 | LTB4R (BLT1) is a high-affinity leukotriene B4 (LTB4) receptor that is expressed in a variety of immune cells such as neutrophils, macrophages and dendritic cells. LTB4-LTB4R signaling plays a pivotal role in macrophage phagocytosis and innate immunity. |
| 69364 | BTN3A3 | 20947169 | BTN3A3 is part of the BT3 family of immunoreceptors belonging to the extended B7 family that are expressed on the surface of resting and activated monocytes and monocyte-derived dendritic cells (iDC). BT3 molecules are involved in the regulation of the balance between immune activation and suppression. |

| | | | |
|---|---|---|---|
| 69145 | BTN3A2 | 20947169 | BTN3A2 is part of the BT3 family of immunoreceptors belonging to the extended B7 family that are expressed on the surface of resting and activated monocytes and monocyte-derived dendritic cells (iDC). BT3 molecules are involved in the regulation of the balance between immune activation and suppression. |
| 69277 | BTN3A1 | 20947169 | BTN3A1 is part of the BT3 family of immunoreceptors belonging to the extended B7 family that are expressed on the surface of resting and activated monocytes and monocyte-derived dendritic cells (iDC). BT3 molecules are involved in the regulation of the balance between immune activation and suppression. |
| 94975 | IFI6 | 15685448 | IFI6 has a function as a cell survival protein by inhibiting mitochondrial-mediated apoptosis. |
| 94975 | IFI6 | 20939681 | IFI6 (G1P3) gene encodes a low molecular weight mitochondrial protein that stabilizes mitochondrial function and opposes apoptosis. |
| 83127 | PTMA | 15242774 | PTMA interaction with STAT3 is IFN-induced and results in the nuclear translocation of the complex. |
| 83127 | PTMA | 20479248 | PTMA inhibits HIV-1 via Toll-like receptor 4-mediated type I interferon (IFN) induction by acting as a ligand for TLR4 and stimulating type I IFN production to potently suppress HIV-1 after entry into cells. |
| 151388 | Rxra | 20498053 | Rxra controls innate inflammatory responses through the up-regulation of chemokine expression. Mice lacking Rxra in myeloid cells exhibit reduced levels of CCL6 and CCL9, impaired recruitment of leukocytes to sites of inflammation, and lower susceptibility to sepsis. |
| 91747 | RXRA | 20498053 | RXRA controls innate inflammatory responses through the up-regulation of chemokine expression. Mice lacking RXRA in myeloid cells exhibit reduced levels of CCL6 and CCL9, impaired recruitment of leukocytes to sites of inflammation, and lower susceptibility to sepsis. |
| 213130 | Nfatc2 | 20557936 | Nfatc2 is a cytokine that acts as an autocrine growth factor during an immune response to bacterial and viral infection, as well as tumorigenesis. |
| 33233 | AIMP1 | 20510162 | AIMP1 is phosphorylated by JNK through the TLR-MyD88 pathway, leading to the loss of its regulatory activity for endoplasmic reticulum retention of gp96 and resulting in the increase of cell surface expression of gp96, thus providing a new molecular mechanism underlying TLR-mediated gp96 regulation. |
| 31798 | GNB2 | 20525682 | GNB2 is the major G protein isoform that mediates neutrophils directional cell migration and in vivo infiltration. |

| | | | |
|---|---|---|---|
| 43260 | SPI1 | 20510871 | SPI1 (PU.1) directly regulates FLT3 kinase in a concentration-dependent manner, and is a critical regulator of both conventional and plasmacytoid dendritic cell development. |
| 69828 | NLRP7 | 15817483 | NLRP7 is a member of the PYRIN-containing apoptotic protease-activating factor-1-like proteins that functions as a feedback regulator of CASP1-dependent interleukin-1beta secretion. |
| 42577 | GLI1 | 20547752 | GLI1 functions as an antagonist of NF-kB activity after LPS stimulation at the level of promoter binding. GLI1 interacts with RELA (p65) upon LPS stimulation and inhibits RELA-mediated transcriptional transactivation by interfering with RELA binding to target gene promoter DNA. |
| 67847 | MARCO | 20162551 | MARCO is upregulated in TLR4-mediated LPS responses and these receptors contribute to the efficient capturing and clearance of invading microbial pathogens. |
| 47323 | TOMM70A | 20628368 | TOMM70A, a mitochondrial import receptor, interacts with MAVS upon RNA virus infection acting as a critical adaptor bridging TBK1/IRF3 to MAVS. |
| 298136 | USP17 | 20368735 | USP17 is required for virus-induced RIG-I- and melanoma differentiation-associated protein-5 (MDA5)-mediated type I IFN signaling and functions through deubiquitination of RIG-I and MDA5 to regulate virus-induced type I IFN signaling. |
| 213293 | Ace | 20937811 | Ace and iNOS overexpression by myelomonocytic cells substantially boosts innate immunity and represents a new means to address serious bacterial infections such as L. monocytogenes and methicillin resistant S. Aureus. |
| 75897 | ETS1 | 20930145 | ETS1 transcription factor blocks terminal differentiation of keratinocytes and induces expression of matrix metalloproteases and innate immune mediators. |
| 17737 | RAB11A | 20933442 | RAB11A is an important regulator of Toll-like receptor 4 (TLR4) and TRAM transport to E. coli phagosomes thereby controlling IRF3 activation from this compartment. |
| 25942 | FZD1 | 20667980 | FZD1 has a role in the reciprocal regulation of the Toll-like receptor (TLR)/nuclear factor-kappaB (NF-kappaB) and the Wnt/beta-catenin pathway after aerosol infection of mice with Mycobacterium tuberculosis. |

| | | | |
|---|---|---|---|
| 132454 | Fzd1 | 20667980 | Fzd1 has a role in the reciprocal regulation of the Toll-like receptor (TLR)/nuclear factor-kappaB (NF-kappaB) and the Wnt/beta-catenin pathway after aerosol infection of mice with Mycobacterium tuberculosis. Fzd1 mRNA was significantly up-regulated during the course of infection in mice and its induction was dependent on TLRs, the myeloid differentiation response gene 88 (MyD88), and a functional NF-kappaB pathway. |
| 4005 | CTSG | 18762176 | CTSG, a cathepsin, is a key modulator of cell death and inflammatory responses. |
| 4005 | CTSG | 20676107 | ELANE and CTSG, together with externalized nucleosomes, promote coagulation and intravascular thrombus growth in vivo. During systemic infection, activation of coagulation fosters compartmentalization of bacteria in liver microvessels and reduces bacterial invasion into tissue. |
| 13111 | ELANE | 18772136 | ELANE is a pro-inflammatory protease that regulates IL8 production from airway epithelial cells and can activate both EGFR and TLR4. |
| 13111 | ELANE | 18802098 | ELANE mediates innate host protection against Pseudomonas aeruginosa by degrading the major outer membrane protein F, a protein involved in porin activity, maintenance of structural integrity, and sensing of host immune system activation. |
| 13111 | ELANE | 20676107 | ELANE and CTSG, together with externalized nucleosomes, promote coagulation and intravascular thrombus growth in vivo. During systemic infection, activation of coagulation fosters compartmentalization of bacteria in liver microvessels and reduces bacterial invasion into tissue. |
| 32554 | TNFRSF13B | 20676093 | TNFRSF13B (TACI) triggers class-switch recombination (CSR) via the DNA-editing enzyme AID by activating NF-kappaB through a Toll-like receptor (TLR)-like MyD88-IRAK1-IRAK4-TRAF6-TAK1 pathway. |
| 105280 | DHX9 | 20696886 | DHX9 interacts with CpG-B and was associated with TNF-alpha and IL-6 production and NF-kappaB activation upon CpG-B stimulation. DHX9 is localized in the cytosol and is found to bind to TLR domain of MYD88. |
| 18019 | MAP2K1 | 11134045 | MAP2K1 and MAP2K2 are necessary and sufficient for the direct binding of the mitogen-activated protein kinases (MAPKs) MAPK3 (ERK1) and MAPK1 (ERK2). |
| 42805 | DDIT3 | 20876114 | DDIT3 (CHOP) is an endoplasmic reticulum (ER) stress-induced transcription factor that targets the IL23 gene and this binding is enhanced in the context of both ER stress and Toll-like receptor (TLR) stimulation. |

| | | | |
|---|---|---|---|
| 83447 | ATG16L1 | 19898471 | ATG16L1 is involved in autophagy where it is recruited to the plasma membrane at the site of bacterial entry by NOD1 and NOD2. |
| 83447 | ATG16L1 | 20200479 | ATG16L1, a critical autophagy protein, is recruited to the plasma membrane by NOD2 during bacterial invasion. |
| 17807 | OTUB2 | 19996094 | OTUB2 and OTUB1 negatively regulate virus-triggered type I interferon (IFN) induction and cellular antiviral response by deubiquitinating TRAF3 and -6. |
| 52992 | OTUB1 | 19996094 | OTUB1 and OTUB2 negatively regulate virus-triggered type I interferon (IFN) induction and cellular antiviral response by deubiquitinating TRAF3 and -6. |
| 201304 | Padi4 | 20733033 | Padi4 plays a role in chromatin decondensation to form neutrophil extracellular traps (NETs) in an innate immune response to bacterial infection. |
| 36977 | IL21 | 20817119 | IL21 is a cytokine that has broad effects on both innate and adaptive immune responses. |
| 15159 | PPIA | 20829794 | PPIA (CYPA) is a cell-intrinsic sensor for human immunodeficiency virus 1 (HIV-1) that exists in dendritic cells and mediates an antiviral innate immune response. |
| 49799 | CD200 | 20833375 | CD200 is induced by TLR-, NOD2-, and NALP3-mediated pathways, limiting macrophage activation and protecting the host from excessive inflammation. |
| 71054 | ANXA1 | 20679535 | ANXA1 is cleaved by CAPN1 to generate a N-terminally truncated form of ANXA1 shown to be anti-inflammatory and able to activate ERK. This C-terminal ANXA1 peptide functions by increasing ICAM1 clustering around adherent neutrophils to anchor them to the endothelium and promote transmigration through the transcellular route. |
| 42778 | XDH | 20632067 | XDH (xanthine oxidase) is involved in TLR7/8-mediated activation of CASP1 and IL1B in an HIF1A-dependent manner. |
| 106349 | CR2 | 20558730 | CR2 (CD21) is a cell membrane receptor, with 15 or 16 extracellular short consensus repeats (SCRs), that promotes B lymphocyte responses and bridges innate and acquired immunity. |
| 1973 | FNAR2;IL10RI | 12220192 | The interferon alpha receptor is composed of two subunits: IFNAR1 and IFNAR2;IL10RB. Binding of interferon (IFN)-alpha to IFNAR2;IL10RB results in STAT2 binding and the initiation of the IFN signaling cascade. |
| 1973 | FNAR2;IL10RI | 20483775 | Type I interferons (IFNs) play an important role in innate immunity to protozoan parasites by binding the IFN alpha receptor, composed of IFNAR1 and IFNAR2;IL10RB, and regulating neutrophil/monocyte recruitment, neutrophil turnover, and Leishmania infection. |

| | | | |
|---|---|---|---|
| 208945 | Foxa2 | 20483781 | Foxa2 is expressed selectively in the respiratory epithelium where it plays a critical role in regulating genetic programs influencing Th2 cell-mediated pulmonary inflammation. |
| 59234 | FOXA2 | 20483781 | FOXA2 is expressed selectively in the respiratory epithelium where it plays a critical role in regulating genetic programs influencing Th2 cell-mediated pulmonary inflammation. |
| 137815 | Defb14 | 20483750 | Defb14 is a beta-defensin with direct antimicrobial properties that contribute to local innate immune responses and it aids in combating microbial invasion by being chemotactic for a broad spectrum of leukocytes in a CCR6- and CCR2-dependent manner. |
| 137844 | Defb4 | 20483750 | Defb4 is a beta-defensin with direct antimicrobial properties that contribute to local innate immune responses and it aids in combating microbial invasion by being chemotactic for a broad spectrum of leukocytes in a CCR6- and CCR2-dependent manner. |
| 157234 | Fcrl5 | 20519648 | Fcrl5 is an orphan immunoregulatory protein that is highly expressed by innate B lymphocytes, as a specific receptor for orthopoxvirus MHC class I-like protein (OMCP) and this strongly implicates it in contributing to host defense against zoonotic orthopoxviruses. |
| 145270 | Ly86 | 20534476 | Ly86 (MD-1) complexes with Toll-like receptor homolog Cd180 (RP105) to regulate the Ly96 (MD-2)/Tlr4-mediated lipopolysaccharide (LPS) response. Soluble Ly86 alone, in addition to its complex with Cd180, can regulate host LPS sensitivity. |
| 14716 | ING4 | 20534538 | ING4 is a member of the inhibitor of growth (ING) family of chromatin-modifying proteins that functions to negatively regulate the cytokine-mediated inflammatory response. In mice, it facilitates NF-kappaB activation of IkappaB promoters, thereby suppressing nuclear RelA (p65) levels and the activation of select NF-kappaB target cytokines. |
| 188872 | Ing4 | 20534538 | Ing4 is a member of the inhibitor of growth (ING) family of chromatin-modifying proteins that functions to negatively regulate the cytokine-mediated inflammatory response in mice by facilitating NF-kappaB activation of IkappaB promoters; thereby suppressing nuclear Rela (p65) levels and the activation of select NF-kappaB target cytokines. |
| 163852 | Calm1 | 16893173 | Calm1 (CaM) and Nos2 (iNOS) coordinately function to form a stable complex that is part of a rapid host response that functions within the first 30 min following bacterial infection to upregulate the innate immune system involving macrophage activation. |

| | | | |
|---|---|---|---|
| 15903 | CALM1 | 16893173 | CALM1 (CaM) and NOS2 (iNOS) coordinately function to form a stable complex that is part of a rapid host response that functions within the first 30 min following bacterial infection to upregulate the innate immune system involving macrophage activation. |
| 14319 | NXN | 20400501 | NXN subfamily of proteins form a link between MYD88 and FLII (flightless I) to mediate negative regulation of the TLR4/MYD88 pathway. |
| 6462 | SPON2 | 20205276 | SPON2 expression is upregulated during intestinal inflammation and may induce NF-kappaB promoter activation in a TLR-9 mediated manner. |
| 36980 | CTCF | 15670593 | CTCF regulates the transcription of the interleukin 1 receptor-associates kinase 2 (IRAK2) promoter. IRAK2 is part of a family of four IRAKs that regulate immune responsiveness to bacterial endotoxins. |
| 80442 | RNF5 | 19285439 | RNF5 negatively regulates virus-triggered signaling by targeting TMEM173, an adaptor protein that links virus-sensing receptors to IRF3 activation, for ubiquitination and degradation at the mitochondria. |
| 3013 | PIAS2 | 12764129 | Functions as an E3 ligase to promote STAT1 SUMO modification |
| 3611 | TCF4 | 18854153 | Transcription Factor that is an essential and specific regulator of Plasmacytoid dendritic cell development |
| 7206 | TYRO3 | 18083102 | Tyrosine protein kinase that acts with AXL and MERTK as pleiotropic inhibitor of the innate immune response in DCs |
| 11328 | TNFRSF12A | 12529173 | The FN14 cytoplasmic tail binds to tumour-necrosis-factor-receptor-associated factors 1, 2, 3 and 5 and mediates nuclear factor-kappaB activation |
| 13548 | TLR10 | 15728506 | Human TLR10 is an orphan member of the TLR family with no identified specific ligand |
| 13548 | TLR10 | 18487086 | NLRP3 has an important role in IL-1 beta and IL-18 secretion through the inflammasome and mediates responses to LPS, peptidoglycan, bacterial RNA and imidazoquinolines. |
| 13548 | TLR10 | 15728506 | Able to homodimerize, heterodimerize with TLR1 and TLR2, and directly associate with MYD88 |
| 13548 | TLR10 | 22043967 | mmu-mir-155 suppresses Socs1 protein expression and has a pro-inflammatory role in microglia. |
| 13548 | TLR10 | 18280719 | NLRP3 complexes with PYCARD (ASC), RIPK2, and CASP1 inflammasome to process IL-1 beta. |
| 13548 | TLR10 | 17911593 | Hsa-mir-155 is induced by bacterial and viral infections as well as pro-inflammatory cytokines and functions to suppress FADD, RIPK1 and IKKE expression. |
| 13548 | TLR10 | 19339971 | NLRP3 is a critical NOD-like receptor family member that transduces a fungal recognition signal to the inflammasome adaptor PYCARD for CASP1 activation and pro-IL-1beta processing. |
| 13548 | TLR10 | 22170100 | Mmu-mir-155 has a pro-inflammatory role in astrocytes and its expression is negatively regulated by Irf3. (Demonstrated in human) |

| | | | |
|---|---|---|---|
| | | | NLRP3-dependent CASP1 activation complex (inflammasome) is triggered when dying tumor cells release ATP, acting on P2X7 purinergic receptors from dendritic cells and allowing for the secretion of IL-1beta. |
| 13548 | TLR10 | 19767732 | |
| 13548 | TLR10 | 17242365 | Hsa-mir-155 induction in response to either poly(I : C) or TNF-alpha is blocked by pharmacological inhibition of JNK, suggesting that its inducing signals use the JNK pathway. |
| 13548 | TLR10 | 20610650 | NALP3 inflammasome is activated when CyaA, a virulence factor from B. pertussis, promotes IL-1beta production, which then polarizes T cell responses toward the Th17 subtype and promotes clearance of the bacteria from the respiratory tract. |
| 13548 | TLR10 | 22546503 | Mir155 antagonizes progesterone to reverse the inhibition of Tlr3/4 signalling. |
| | | | NLRP3 is directly activated by certain antibiotics and plays an important role in the antibiotic-mediated secretion of IL1B. In the case of polymyxin B, NLRP3 was also required for the neutrophil influx into the peritoneal cavity. (Demonstrated in murine models) |
| 13548 | TLR10 | 21278344 | |
| 13548 | TLR10 | 19008191 | Hsa-mir-155 expression in monocyte and macrophage cell lines is simulated by viral or bacterial infection in vitro. |
| 13548 | TLR10 | 21289120 | NLRP3 inflammasome is essential for host defence against influenza and other RNA viruses (i.e. EMCV, VSV). |
| 13548 | TLR10 | 23572582 | Mir155 targets Pmaip1 (Noxa) and Socs1 to mediate natural killer cell expansion during MCMV infection. |
| 13548 | TLR10 | 21385879 | NLRP3 recruits adaptor protein PYCARD and CASP1 to form an NLRP3 inflammasome complex in response to Varicella-Zoster Virus (VZV) infection. |
| 13548 | TLR10 | 20937844 | hsa-mir-155 feedback positively regulates host antiviral innate immune response by promoting type I IFN signaling via targeting suppressor of cytokine signaling 1 (SOCS1). |
| 13548 | TLR10 | 21439959 | NLRP3 is a component of the inflammasome and is required for inflammation in acute pancreatitis. (Demonstrated in murine model) |
| 13548 | TLR10 | 24130493 | Mycobacterial infection induces expression of Mir155, which promotes the maturation of phagosomes and represses the expression of Rheb by targeting its 3â€ UTR. |
| | | | NLRP3 is necessary to illicit IL1B response specific to viable, but not heat-killed, E. coli infections. (Demonstrated in murine model) |
| 13548 | TLR10 | 21602824 | |

| | | | |
|---|---|---|---|
| 13548 | TLR10 | 21097505 | hsa-mir-155 target IL13RA1 and reduces the IL13RA1 protein expression, and inhibits the expression of M2/pro-Th2 profile genes in macrophages. |
| 13548 | TLR10 | 22174673 | The NLRP3 inflammasome plays a role in innate immune responses against mucosal Candida infection. NLRP3 limits the severity of infection when present in either the hematopoietic or stromal compartments. (Demonstrated in mouse) |
| 13548 | TLR10 | 25024218 | T-cell-intrinsic Mir155 is required for type-2 immunity, in part through regulation of S1pr1, whereas T-cell-intrinsic Mir146 is required to prevent overt Th1/Th17 skewing. |
| 13548 | TLR10 | 22295065 | NLRP3/PYCARD inflammasome activation following human respiratory syncytial virus infection is dependent on the activation of TLR2/MYD88/NF-kB and reactive oxygen species/potassium efflux. |
| 13548 | TLR10 | 21652514 | MIR155 upregulation is a feature of the mammalian inflammatory response and MIR155 expression may exert both positive and negative regulatory effects on TLR and NFkB signalling. MIR155 expression is suppressed by IL10 as a part of negative-feedback loop in LPS-stimulated cells. |
| 13548 | TLR10 | 22984081 | MIR223 and EBV miR-BART15 regulate the NLRP3 inflammasome and IL-1beta production. |
| 13548 | TLR10 | 21762537 | hsa-mir-155 overexpression can enhance innate antiviral immunity by promoting the JAK/STAT signalling pathway to facilitate the clearance of hepatitis B virus in human hepatoma cells. |
| 13548 | TLR10 | 22997256 | Uromodulin nanoparticles activate the NLRP3 inflammasome in renal interstitial monocytes. |
| 13548 | TLR10 | 22043967 | hsa-mir-155 suppresses SOCS1 protein expression and has a pro-inflammatory role in microglia. (Demonstrated in mice) |
| 13548 | TLR10 | 24323452 | Protein-bound polysaccharide-K can activate the NLRP3 inflammasome and induce IL1B in a TLR2- and NLRP3-dependent manner . |
| 13548 | TLR10 | 22170100 | MIR155 has a pro-inflammatory role in human astrocytes and its expression is negatively regulated by IRF3. |
| 13548 | TLR10 | 24217221 | Endoplasmic reticulum (ER) stress-mediated reactive oxygen species accumulation leads to activation of NLRP3 inflammasome through enhanced secretion of IL1B and binding of TXNIP. |
| 13548 | TLR10 | 22546503 | MIR155 antagonizes progesterone to reverse the inhibition of TLR3/4 signalling. (Demonstrated in mice) |
| 13548 | TLR10 | 25355909 | Macrophages sense multiple types of bacterially derived RNA (mRNA, tRNA and rRNA) via the NLRP3 inflammasome. |

| | | | |
|---|---|---|---|
| 13548 | TLR10 | 23028330 | MIR155 exerts anti-HIV-1 effects by targeting several HIV-1 dependency factors involved in post-entry, pre-integration events. |
| 13548 | TLR10 | 25761061 | NLRP3 mediates NF-kB activation in both sterile and microbially induced inflammation. |
| 13548 | TLR10 | 23428672 | MIR155 is targeted by Borna disease virus (BDV) encoded protein to inhibit type I IFN induction. |
| 13548 | TLR10 | 23580661 | Circulating MIR155 activates natural killer cells via the TLR1 signalling pathway. |
| 13548 | TLR10 | 25772938 | Hepatitis C virus (HCV)-induced, MIR155-regulated HAVCR2 expression regulates natural killer cell function, suggesting a novel mechanism for balancing immune clearance and immune injury during chronic viral infection. |
| 13548 | TLR10 | 25995365 | Mir155 is a post-transcriptional repressor of Arntl (Bmal1), linking the molecular clock and innate immune response. |
| 13548 | TLR10 | 25978411 | Influenza A virus non-structural protein 1, NS1, physically interacts with endogenous NLRP3 downregulating NLRP3 inflammasome activation as well as NF-Î°B, leading to a reduction in the levels of inflammatory cytokines. |
| 13548 | TLR10 | 25977263 | TLR10 is a functional receptor involved in the innate immune response to H. pylori infection and the TLR2/TLR10 heterodimer functions in H. pylori lipopolysaccharide recognition. |
| 13958 | USP7 | 18952891 | Cytoplasmic USP7 binds to and deubiquitinates TRAF6 and IKBKG, thus terminating TLR-mediated NF-kappaB and JNK activation |
| 18462 | CARD6 | 16418290 | A microtubule-interacting protein that positively regulates NF-kappaB activation and modulates function of RIP family members |
| 18462 | CARD6 | 18160713 | Expression of CARD6 and RIPK2 in bone marrow-derived macrophages is rapidly induced by IFNB1 and IFNG |
| 18462 | CARD6 | 12775719 | Selectively modulates NF-kappaB activation by RIPK2 and NOD1 |
| 18519 | C7 | 11058761 | Complement component seven is one of five components that interact to form the cytolytic membrane attack complex (MAC) |
| 18604 | C6 | 11058761 | Complement component six is one of five components that interact to form the cytolytic membrane attack complex (MAC) |
| 18990 | BDKRB2 | 18810490 | Involved in the early immune response against Listeria infection by increasing the production of IL12A/IL12B in human monocyte-derived dentritic cells |
| 19613 | CTSD | 18762176 | Cathepsins: key modulators of cell death and inflammatory responses |

| | | | |
|---|---|---|---|
| 21497 | C1QBP | 12574814 | Binds proteins involved in complement, coagulation, and kinin systems, as well as viral and bacterial pathogens including S. aureus protein A and when expressed on activated platelets may contribute directly to thrombosis, inflammation and endovascular infections |
| 22594 | CSK | 15749833 | Plays a critical role in IL-1-induced NF-kappaB activation through the IKK complex |
| 22989 | SCAMP5 | 19234194 | Promotes calcium-regulated exocytosis of signal peptide-containing cytokine secretion (CCL5 but not IL-1beta) in human monocytes and mouse macrophages |
| 24106 | PSTPIP1 | 17964261 | Activates MEFV by binding to the B-Box domain of MEFV and unmasking its PYD domain |
| 24106 | PSTPIP1 | 17964261 | Enhances binding of MEFV to PYCARD |
| 24106 | PSTPIP1 | 14595025 | Tyrosine phosphorylation of PSTPIP1 significantly enhances its interaction with MEFV |
| 25289 | CTSH | 18762176 | Cathepsins: key modulators of cell death and inflammatory responses |
| 27924 | TRIM22 | 19218198 | An E3 ubiquitin ligase that ubiquitinates the viral 3C protease and acts as a component of an antiviral pathway induced by IFN against picornaviruses |
| 28592 | SMARCA4 | 19144648 | A member of the SWI/SNF family of proteins and is essential for the multiple changes in gene expression that occur during differentiation |
| 28592 | SMARCA4 | 19342595 | Required for maintaining expression of several smooth muscle-specific genes |
| 29985 | NLRP14 | 18648497 | Part of the NLRP (Nucleotide-binding oligomerization domain, Leucine rich Repeat and Pyrin domain containing) family, has a role in apoptosis and inflammation and several NLRPs have been indicated as being involved in reproduction as well |
| 30254 | CCNT1 | 18728388 | Heterodimerizes with CDK9 to form the positive transcriptional elongation factor b (P-TEFb) and plays a role in the activation of a subset of NF-kappaB dependent targets |
| 31021 | MAP2K4 | 10715136 | Activates JNK proteins |
| 31021 | MAP2K4 | 11343802 | Activated by MAP3K 2,3,4,5 (MEKK 2,3,4,5) |
| 31021 | MAP2K4 | 9841871 | Activated by MAP3K 2,3,4,5 (MEKK 2,3,4,5) |
| 31021 | MAP2K4 | 8940179 | Activated by MAP3K 2,3,4,5 (MEKK 2,3,4,5) |
| 35553 | MAP2K3 | 7839144 | Activates MAPK14 (p38) MAP kinases |
| 35701 | SUGT1 | 17420470 | Positively regulates NOD1 activation |
| 35701 | SUGT1 | 18818696 | Bridges the HSP90 molecular chaperone system to the substrate-specific arm of SCF ubiquitin ligase complexes, suggesting a role in SCF assembly and regulation, and providing multiple complementary routes for ubiquitination of Hsp90 client proteins |
| 36569 | SARM1 | 16964262 | Negatively regulates adaptor protein TICAM1-dependent Toll-like receptor signaling |

| | | | |
|---|---|---|---|
| 38002 | NCKAP1L | 19015308 | A point mutation in the murine Hem1 gene reveals an essential role for Hematopoietic Protein 1 in lymphopoiesis and innate immunity-T development is disrupted in Hem-1 deficient mice |
| 38974 | CD63 | 18930046 | Forms a complex with ELANE (neutrophil elastase 2) and plays a role in the targeting of ELANE to primary granules in neutrophils |
| 44200 | SMARCA2 | 19144648 | A member of the SWI/SNF family of proteins and is essential for the multiple changes in gene expression that occur during differentiation |
| 44200 | SMARCA2 | 19342595 | Required for maintaining expression of several smooth muscle-specific genes |
| 46340 | BMX | 18025155 | A tyrosine protein kinase that regulates TLR4 induced IL6 in macrophages independent of MAPK14 (p38-alpha) and NF-kappaB |
| 46853 | SERPING1 | 17709141 | A likely regulator of MASP2 |
| 49110 | RETNLB | 10921885 | A novel cysteine-rich secreted protein associated with pulmonary inflammation |
| 49787 | NFKBIB | 9346485 | Inhibitor of NF-kappaB, binds to both NF-kappaB subunit nuclear localization signals and NFkappaB |
| 50152 | PRKCE | 19150425 | Following TNF stimulation, PRKCE phosphorylates TRAF2 leading to CHUK (IKK alpha) and IKBKB (IKK beta) recruitment to the TNF receptor |
| 50532 | STAT5B | 9398404 | IL-2 and IL-7 induced heterodimerization of STAT5 isoforms in human peripheral blood T lymphoblast |
| 50625 | STAT5A | 9398404 | IL-2 and IL-7 induced heterodimerization of STAT5 isoforms in human peripheral blood T lymphoblast |
| 50628 | SOCS5 | 15590694 | SOCS4 and SOCS5 regulate EGFR signaling |
| 53133 | REL | 18523276 | Essential for the development of innate and T-cell-induced colitis through its ability to modulate expression of IL-12/23 family members |
| 53886 | RPS6KA4 | 18690222 | Negatively regulates TLR-pathway driven inflammation by preventing the binding of phosphorylated transcription factors CREB and ATF1 to IL-10 and DUSP1 promoters |
| 85368 | SH2D1A | 18501771 | The SLAM and SAP gene families control innate and adaptive immune responses |
| 85368 | SH2D1A | 19079134 | SLAM receptors and SAP influence lymphocyte interactions, development and function |
| 103936 | SLAMF8 | 18501771 | The SLAM and SAP gene families control innate and adaptive immune responses |
| 103966 | SLAMF9 | 18501771 | The SLAM and SAP gene families control innate and adaptive immune responses |
| 104051 | SLAMF6 | 18501771 | The SLAM and SAP gene families control innate and adaptive immune responses |
| 104051 | SLAMF6 | 19079134 | SLAM receptors and SAP influence lymphocyte interactions, development and function |
| 104088 | SLAMF7 | 18501771 | The SLAM and SAP gene families control innate and adaptive immune responses |
| 104088 | SLAMF7 | 19079134 | SLAM receptors and SAP influence lymphocyte interactions, development and function |

| | | | |
|---|---|---|---|
| 301101 | C2 | 12791093 | C2 is part of the classical and lectin complement pathways |
| 301101 | C2 | 11044372 | C2 molecule binds to C4B and is cleaved by C1S protease into C2A and C2B fragments and the resulting C4B2A complex (C3 convertase) is the active protease which cleaves C3 |
