# Supplementary material for: An immune-related prognostic signature associated with immune landscape and therapeutic responses in gastric cancer
Source: Aging (Albany NY). 2023 Feb 22;15(4):1074–106. doi: 10.18632/aging.204534 (PMC10008502; doi:10.18632/aging.204534)
Supplement: Supplementary Table 6 [file aging-15-204534-s005.pdf]

Supplementary Table 6. Differentially expressed genes.

Gene	logFC	pValue	FDR
ATIC	1.123355692	1.25E-16	1.27E-14
CDC25B	1.820530614	4.20E-15	1.82E-13
AP003419.3	1.704363081	1.17E-07	5.72E-07
RN7SL333P	1.474355473	7.67E-08	3.94E-07
PTTG3P	2.119335725	1.34E-11	1.86E-10
DYNC1I1	-1.45809121	0.033176124	0.045359658
DUXAP1	1.96869201	2.51E-05	7.01E-05
ARHGDIG	-1.129725317	3.82E-08	2.12E-07
LINC01433	1.847613252	1.39E-08	8.60E-08
LINC02253	5.774701726	8.97E-06	2.78E-05
RBM24	-1.656444358	1.25E-05	3.75E-05
FRY-AS1	1.333440219	0.00042584	0.00089129
CTSC	1.054388112	6.09E-08	3.20E-07
MMP12	2.585468903	1.30E-07	6.31E-07
RN7SL850P	1.247242277	0.000739432	0.00146697
CPM	1.174274358	0.005820332	0.009450015
LINC02584	2.60083187	2.00E-08	1.19E-07
DHCR7	1.00966805	1.52E-06	5.67E-06
SNORA74D	2.043014553	8.83E-08	4.47E-07
AC084125.2	1.057106949	5.38E-07	2.25E-06
AC138951.1	1.315254073	4.65E-06	1.54E-05
IGKV1-39	-2.810912153	0.012885926	0.019371419
AL139352.1	1.501341216	0.003424502	0.005853713
FCGR1A	2.449499591	1.12E-13	2.82E-12
ODCP	1.429850602	5.12E-10	4.69E-09
PARD6B	1.320696906	1.83E-09	1.42E-08
RPL7AP3	1.265273529	4.68E-08	2.53E-07
ASTL	1.304228734	0.008126658	0.0127863
AC127024.5	1.027388809	9.66E-08	4.84E-07
CHEK2	1.4825337	3.15E-15	1.45E-13
CCDC62	1.332722716	1.65E-07	7.79E-07
ANKRD45	1.559350039	0.000295957	0.000641466
PIGHP1	1.079543623	1.27E-08	7.95E-08
SMIM11P1	1.650647473	0.011063396	0.016892143
AC026124.2	1.053221479	4.17E-05	0.000110214
MSRB3	-1.578341456	0.000356625	0.000759307
BTBD16	3.310451159	1.61E-13	3.86E-12
CAVIN2	-1.241376455	3.17E-08	1.79E-07

AL161729.4	2.031893826	5.97E-07	2.47E-06
AL136115.1	1.666311151	1.20E-09	9.80E-09
APOBEC1	-2.615217764	0.031638202	0.043459911
SMTNL1	1.84882779	1.24E-08	7.76E-08
HTR1DP1	1.060993483	0.006192109	0.009992136
CES2	-1.456090365	0.030076278	0.041556487
RN7SL154P	1.092017208	0.001573432	0.002896384
LRP5L	1.005859524	1.38E-09	1.11E-08
TTC3-AS1	1.697913934	1.49E-08	9.17E-08
GNAO1	-1.883539794	4.58E-06	1.52E-05
MIS18A-AS1	1.383920179	1.76E-06	6.46E-06
TPM3P7	1.382765229	6.32E-07	2.60E-06
AL139120.1	1.560037924	1.24E-07	6.04E-07
HNRNPA3P3	1.323254134	1.95E-09	1.51E-08
ALG1L8P	1.085257763	0.000790552	0.001558408
RNU6-638P	1.080854651	0.000213589	0.000478149
MIR3174	2.073928341	1.50E-07	7.17E-07
AL139397.1	1.036142288	0.000938881	0.001824883
AC004466.2	1.043721633	5.21E-05	0.000134561
AC092198.1	4.159946293	6.05E-07	2.50E-06
P2RY14	-1.859475889	1.06E-11	1.51E-10
BNIP5	1.346897146	9.37E-06	2.89E-05
CCT2	1.402735663	1.50E-17	3.55E-15
IGKV1D-39	-3.074528791	0.011154364	0.017015435
AC022211.1	1.61514648	4.88E-11	5.81E-10
AL732366.1	1.681414401	6.07E-08	3.20E-07
NUTM2G	1.039461833	1.38E-05	4.11E-05
AL158196.1	1.289306168	7.23E-06	2.28E-05
FCHO1	1.755075042	1.76E-11	2.37E-10
HMGB3P32	1.829241737	7.35E-07	2.96E-06
RYR3	-1.827527614	0.000189153	0.000428813
BTBD10P2	1.175405729	4.53E-07	1.93E-06
RPL39P3	1.035031002	6.84E-06	2.17E-05
BMP6	-1.398308738	0.000363074	0.000771865
AC048337.1	1.448634139	0.001386785	0.002585496
AL049542.1	1.484134767	0.001087005	0.002081505
ITGA7	-1.233840124	0.010173729	0.015671166
AC015845.2	-1.607844698	0.022251577	0.031746091
NAT10	1.196912799	4.01E-17	6.44E-15
MIR548XHG	4.710464745	0.000186227	0.000422864
AC092991.1	1.134780353	0.005006326	0.008248519

AC016949.1	1.389161845	6.65E-09	4.45E-08
AC226118.1	2.319839958	0.00057569	0.001169019
IGLV2-8	-2.773580443	0.029022967	0.040243046
IL4I1	1.617890376	4.83E-09	3.34E-08
BET1-AS1	1.049859485	2.14E-06	7.69E-06
AC024568.1	1.190017167	1.80E-05	5.20E-05
CHIT1	3.846352941	2.34E-07	1.06E-06
CNTNAP3	-1.452121983	0.018425639	0.026778094
SNTB1	1.097196518	0.005159961	0.008474398
DMBX1	5.094592927	6.96E-15	2.74E-13
NUTF2P7	1.525351545	1.83E-07	8.54E-07
PTPRU	1.87048431	8.05E-08	4.11E-07
DNASE1L2	1.680640006	6.47E-10	5.75E-09
AC005180.2	-1.450024595	1.23E-08	7.75E-08
DCLRE1CP1	1.802949027	7.55E-05	0.000187849
HPGD	-2.153858194	1.89E-12	3.30E-11
BOLA3P1	1.827437752	0.010574798	0.016217693
TDRD5	3.140767934	1.56E-08	9.55E-08
IGHV1OR15-6	-2.446014399	0.000464604	0.00096492
POU5F1	1.714073646	1.71E-07	8.05E-07
AC078795.1	1.417800678	5.25E-11	6.19E-10
PLCD1	-1.008635452	1.97E-09	1.52E-08
AC020661.2	1.644827882	9.50E-08	4.77E-07
PIGU	1.152734894	1.22E-14	4.30E-13
FOXF2	-1.651499093	2.37E-06	8.40E-06
AC027644.1	1.44892843	1.95E-09	1.51E-08
AC243654.2	1.446905122	1.23E-07	6.01E-07
COL1A2	2.227494034	2.95E-12	4.90E-11
RNU1-129P	2.086759771	7.80E-05	0.000193093
SNORA72	1.467979623	8.45E-07	3.36E-06
AC018878.1	1.279334552	0.000207852	0.000466167
PDE1C	-1.157877369	1.27E-08	7.95E-08
AC079035.1	1.079082334	0.000799932	0.0015755
TPBGL	1.459639758	8.95E-07	3.54E-06
MIR6739	1.829771329	1.67E-09	1.31E-08
AC009127.1	1.18150367	0.000252025	0.000554958
SNORD111	2.493754777	3.59E-08	2.01E-07
AC254629.1	1.450250677	1.82E-06	6.67E-06
AC079866.1	1.310865569	6.48E-06	2.07E-05
SOX4	2.116741011	7.49E-17	9.04E-15
ESRRB	-1.595125762	0.007203237	0.011460461

KCNJ13	-1.806941377	0.017888853	0.026070508
MIR4284	1.881936118	2.35E-05	6.63E-05
AC105235.1	1.733833547	7.75E-09	5.12E-08
LINC02561	1.740099026	6.89E-06	2.19E-05
GFRA3	-1.33646207	1.01E-09	8.43E-09
LRRC45	1.025433389	2.51E-09	1.88E-08
AGRN	1.96247905	5.75E-17	7.74E-15
MIR6835	1.384146917	2.81E-05	7.76E-05
AC013553.2	1.908983195	2.06E-08	1.22E-07
AL049780.1	1.120926129	1.68E-06	6.20E-06
HSP90AA2P	1.171462714	7.46E-12	1.11E-10
AC092436.2	1.495120792	4.04E-07	1.74E-06
AC106037.3	1.113462057	5.99E-06	1.93E-05
JCHAIN	-3.523779554	0.001911014	0.003448939
AC024558.1	1.092441607	2.97E-05	8.13E-05
RN7SL306P	2.084818862	4.07E-05	0.000108053
CMTM1	1.384307032	2.75E-14	8.61E-13
AC009997.1	1.060947926	0.006306583	0.01016086
ADH7	-4.132838045	5.68E-12	8.78E-11
FBXO43	2.151214253	4.94E-12	7.77E-11
NCALD	-1.037783265	0.002654989	0.004645316
TIMM8A	1.181392382	6.74E-17	8.43E-15
AC025918.1	1.408340568	5.84E-05	0.000149015
AL031186.1	1.22575649	2.03E-07	9.36E-07
NUSAP1	1.782374567	1.02E-14	3.76E-13
LINC01091	1.000342006	0.002225796	0.003963881
IGFN1	2.840513388	4.96E-05	0.000128789
CAVIN4	1.246630739	2.35E-08	1.37E-07
IFITM3P4	1.300824087	3.49E-07	1.53E-06
PLEKHD1	2.46754581	1.06E-07	5.27E-07
SNORD91A	1.062634175	0.001437301	0.002669775
VWA5B1	3.549646818	0.018449278	0.026810979
RN7SL255P	2.757188498	1.17E-06	4.50E-06
AC090286.3	1.304935285	1.45E-07	6.95E-07
MXRA7P1	-1.360260681	0.001423565	0.002646474
MADD-AS1	1.480274725	6.97E-06	2.21E-05
KRT18P8	1.666560423	3.57E-09	2.56E-08
CELSR1	1.00355823	3.60E-07	1.57E-06
HMGN1P24	1.630388891	6.74E-06	2.14E-05
AC108727.1	1.566250061	1.71E-07	8.03E-07
AL033528.1	1.300795977	9.48E-05	0.000230398

SPAG5	2.091546273	8.32E-16	5.09E-14
AL731563.2	1.073238677	0.016470438	0.024172611
MT1M	-2.321130266	1.05E-13	2.68E-12
CAPN12	1.642681003	9.57E-06	2.94E-05
TNFSF11	2.724813939	2.50E-14	7.92E-13
MAGEA8	4.987800742	0.000510915	0.00105143
RN7SL239P	1.061276841	4.11E-05	0.000108911
SUMO2P16	1.120592951	0.016054031	0.023636999
AC096775.1	1.262733082	0.00114599	0.00218004
COCH	2.341784357	5.24E-06	1.71E-05
AC015853.1	1.453980215	1.62E-08	9.88E-08
COX6CP2	1.695548184	3.85E-05	0.000102863
MCM4	1.784158159	2.85E-15	1.34E-13
AL845321.1	1.128206671	0.01704537	0.024950429
PCAT14	4.882969064	0.031417346	0.043176567
HOXB-AS4	2.832913459	5.14E-12	8.03E-11
DYNLT2	1.300976512	6.21E-11	7.21E-10
AL031667.2	1.969925305	1.06E-05	3.22E-05
APOE	2.014540367	4.52E-09	3.15E-08
PLIN5	-1.487857334	6.89E-06	2.19E-05
AP002336.2	1.622891146	1.78E-10	1.84E-09
MYRF-AS1	1.667301917	4.98E-08	2.68E-07
LINC01558	2.154060761	2.38E-06	8.45E-06
IGKV1OR2-1	-3.198588642	0.000374908	0.00079455
AC092143.3	1.246419211	3.85E-06	1.30E-05
IGKV1-37	-3.557662179	0.000865696	0.001692637
GORAB	1.100650837	1.94E-14	6.43E-13
CYP2T3P	1.442434303	0.000231934	0.000515122
TGFB2-AS1	1.102200069	0.000399341	0.00084139
LACTB2-AS1	2.22571489	1.16E-14	4.12E-13
HOXC8	4.274436945	5.35E-16	3.61E-14
YWHAZP4	1.537772291	4.36E-09	3.05E-08
AC106873.1	1.313913654	0.000502577	0.001035949
TFDP1	1.016025157	1.09E-13	2.77E-12
LINC01301	1.545011945	2.67E-07	1.20E-06
NECAB1	-2.153592938	2.97E-05	8.13E-05
CPED1	-1.450286242	5.03E-05	0.0001304
AL133517.1	1.079982684	5.68E-08	3.00E-07
AUNIP	2.085346073	5.59E-16	3.73E-14
FAM180B	-3.200128229	2.00E-11	2.67E-10
FAT1	1.242661095	7.97E-10	6.88E-09

AL713899.1	4.206063641	5.16E-07	2.17E-06
BUD31P2	2.295964619	2.84E-05	7.83E-05
BIRC5	1.770492121	5.16E-13	1.06E-11
AC079922.1	1.163032766	3.26E-06	1.12E-05
NOX1	4.729361985	3.05E-12	5.03E-11
IGKV1-9	-2.392695728	0.033305606	0.045513333
RNU4-29P	2.005024678	0.000317918	0.000684277
CLLU1-AS1	1.608880266	0.007133788	0.01136441
RNA5SP429	1.127506827	0.001290675	0.002422198
HMGB1P5	1.385257719	1.74E-12	3.08E-11
NETO2	2.080605351	1.45E-09	1.15E-08
IYD	1.340940396	1.40E-05	4.16E-05
TIAF1	1.01162633	7.50E-09	4.97E-08
FCGR1CP	1.500078118	2.31E-06	8.22E-06
RASGRP3-AS1	1.232310863	4.59E-06	1.52E-05
AC114781.2	2.569806639	1.47E-09	1.17E-08
SLC29A2	1.453930557	3.94E-12	6.36E-11
AL442067.2	1.722405463	1.39E-06	5.22E-06
KCNMA1	-1.732842409	2.33E-05	6.56E-05
MELTF	3.250567281	5.52E-16	3.71E-14
LINC02519	2.547629381	8.36E-10	7.17E-09
HSP90AB1	1.256608094	1.48E-18	1.31E-15
AC011466.4	1.323430178	1.55E-06	5.78E-06
PIRT	-2.365759134	0.000111282	0.000265708
AC083822.1	1.825853151	3.70E-06	1.25E-05
CRYZL2P	1.567149641	8.49E-11	9.49E-10
STARD3	1.528619027	8.82E-05	0.000215862
GTF2IP5	1.577158643	4.40E-08	2.40E-07
AC008808.2	-1.722561242	8.63E-06	2.69E-05
DNAJA1P3	1.234986337	3.67E-08	2.05E-07
RPS27P21	1.114559142	0.010312489	0.015861979
LINC01814	2.057148915	1.12E-13	2.82E-12
PTCHD3P3	2.961887406	1.83E-06	6.69E-06
AC005154.4	1.281797263	4.39E-06	1.46E-05
Z84485.1	1.11861812	2.95E-06	1.03E-05
AC100803.4	1.088391196	0.000153354	0.000355121
H2AZP6	2.28354043	6.33E-09	4.26E-08
RNU6-574P	1.060655913	0.000691305	0.001379606
DDX18P1	1.116827739	1.50E-06	5.61E-06
KRT18P61	1.596273409	6.34E-10	5.65E-09
C20orf204	3.274967783	3.83E-11	4.69E-10

CCL4L2	1.165679133	1.19E-05	3.58E-05
AC084880.1	-1.401165451	9.53E-05	0.000231435
SLC22A1	1.549270422	3.45E-06	1.18E-05
AC068397.1	1.37315605	7.51E-05	0.000186965
TIMP1	1.798168726	5.47E-14	1.53E-12
NEK2	2.449672392	2.48E-16	1.96E-14
AC011933.1	1.255182487	6.25E-08	3.28E-07
AL118511.2	3.398751311	1.62E-05	4.74E-05
AC124852.1	-1.761730418	6.68E-07	2.72E-06
PCP2	1.53895936	1.01E-05	3.10E-05
AC007342.4	1.5562664	1.13E-06	4.34E-06
RPS3AP46	1.257449303	0.000584788	0.001184879
AC093826.1	1.281394811	0.00417558	0.00700408
PPM1N	1.597460419	9.91E-09	6.38E-08
AC006064.1	1.011971497	0.005388219	0.008811666
RPS6KA6	-1.116787166	4.55E-12	7.21E-11
LINC02605	1.29015442	4.34E-05	0.000114127
LZTS1	1.299323059	2.21E-09	1.68E-08
AC023855.1	1.377789399	0.00014863	0.000345202
AC009269.5	1.224592962	1.01E-05	3.09E-05
RN7SL236P	1.769328571	0.000351406	0.000749273
AC090044.1	-1.375538313	0.003149734	0.005429648
PHLDA2	1.201999622	2.40E-07	1.09E-06
HSPD1P1	1.645935705	5.74E-14	1.60E-12
BIRC7	4.566514166	6.16E-14	1.69E-12
PPIAP81	1.693683137	2.27E-05	6.43E-05
LINC00592	1.477031906	0.002316296	0.004110215
RN7SKP25	1.410427081	1.51E-05	4.46E-05
AC000111.2	1.711176153	0.001885222	0.003407468
RNU6-1337P	1.920285331	4.75E-05	0.000123745
AP000577.2	1.710871048	2.24E-09	1.70E-08
MCM10	2.627056158	6.74E-17	8.43E-15
GATA2-AS1	1.659920351	0.030554862	0.042139074
CILP2	3.99582583	3.25E-13	7.11E-12
RUFY4	1.805826629	3.77E-06	1.27E-05
CENPI	2.166740161	1.63E-17	3.68E-15
RN7SKP16	1.277948997	1.18E-06	4.51E-06
CEP72	1.901795463	6.36E-16	4.09E-14
AL390879.1	1.445781499	1.69E-12	2.99E-11
EGR1	-1.534420579	0.005848265	0.009490162
ANGPT1	-1.156852993	0.007069613	0.011265544

NPM2	1.433434647	0.003655396	0.006212097
HOXC-AS3	7.277285063	1.55E-14	5.35E-13
AL353705.3	2.205493228	2.62E-07	1.18E-06
ARHGEF37	-1.164465164	2.75E-11	3.53E-10
SNX5P2	1.180807751	8.93E-08	4.51E-07
LRFN5	-1.166469323	1.52E-05	4.47E-05
EIF4EP1	1.321550076	2.10E-07	9.65E-07
APLN	3.118100798	3.86E-14	1.14E-12
RBM34	1.003364657	1.47E-13	3.55E-12
AP001062.2	1.084435694	0.011522337	0.017521582
CDKN2A	3.092793207	2.89E-06	1.01E-05
AC108463.2	2.656381235	7.72E-16	4.82E-14
AP000919.2	1.097720727	0.002615229	0.004581465
AC103563.1	-2.756254678	0.025307474	0.035623275
ARHGEF39	1.32018711	6.73E-10	5.94E-09
LMOD1	-2.251363608	3.04E-09	2.23E-08
XIAP-AS1	1.186140846	5.29E-05	0.000136439
AL078459.1	1.599531733	1.02E-11	1.47E-10
RNU6-387P	2.023911422	2.33E-10	2.33E-09
AL049552.1	1.195164058	1.41E-06	5.31E-06
CDH11	1.604215321	1.02E-08	6.53E-08
LINC01106	1.495299241	4.49E-11	5.40E-10
CASC15	1.465163542	3.40E-08	1.91E-07
AC138819.1	-1.516002445	2.09E-05	5.96E-05
SPDL1	1.540218118	1.76E-16	1.57E-14
MCM3	1.370171611	3.56E-16	2.58E-14
MIR3651	1.061314976	0.006914229	0.011049661
AC244093.3	1.805931578	1.16E-08	7.33E-08
RNU6-1177P	1.846026876	3.81E-05	0.000101918
LINC00605	2.331319102	0.001384433	0.002582195
TDGF1P5	2.154877269	9.43E-11	1.04E-09
UTP18	1.11727014	4.23E-17	6.71E-15
AC022022.2	1.632951537	4.39E-06	1.46E-05
NPTX1	-2.461971035	6.99E-06	2.22E-05
SEMA4F	1.048429743	4.57E-10	4.23E-09
AP002981.1	1.625552412	3.72E-06	1.26E-05
FAM86B2	1.099168455	0.010254976	0.015786276
PABPN1P2	1.081378853	2.73E-09	2.02E-08
RPL36AP54	1.603452346	7.80E-07	3.13E-06
AC002558.2	1.278942833	2.86E-06	1.00E-05
AL121890.4	1.125582462	0.000625566	0.001259714

BZW2	1.152355856	1.35E-13	3.31E-12
RPL23AP6	1.034807231	0.00139053	0.002592114
AC133106.1	1.37158341	0.006157679	0.009945011
AL353622.2	1.425181398	2.35E-05	6.62E-05
CD86	1.046181	8.04E-06	2.51E-05
IGKV7-3	-2.934223691	0.00622833	0.010048152
ZFAS1	1.033007489	6.05E-09	4.08E-08
AL132639.3	1.123524764	8.86E-08	4.48E-07
AL034555.1	1.394486313	0.000165463	0.000380318
PPIAP43	1.373572994	1.19E-07	5.81E-07
RRAD	-1.27130468	0.002668688	0.004666219
MIR4261	3.51532762	3.56E-10	3.38E-09
FANCF	1.054968241	1.29E-12	2.37E-11
CCNJ	1.320090321	2.51E-17	4.99E-15
AL139021.2	1.241053705	5.17E-05	0.000133586
RNU6-476P	1.562954001	2.67E-06	9.37E-06
AL121845.3	1.731751812	7.48E-13	1.47E-11
RPL23AP55	1.418188033	1.58E-09	1.25E-08
MZT1	1.406221466	2.13E-15	1.06E-13
CHI3L1	2.679169916	3.59E-10	3.40E-09
AC010809.1	1.01539539	3.52E-06	1.20E-05
CLDN6	7.434967416	1.45E-06	5.45E-06
PPIAP74	1.678883266	9.54E-09	6.17E-08
AL355472.3	1.733407655	0.000538424	0.00110171
MFAP5	-1.210219482	8.71E-06	2.71E-05
ARL6IP1	1.16527166	4.97E-12	7.80E-11
AL121832.1	2.743280404	2.89E-05	7.93E-05
AC009093.2	1.499424293	8.62E-10	7.36E-09
RNY1P4	1.497372857	0.000485493	0.001003997
AP000487.1	1.309046403	6.26E-12	9.56E-11
ZSCAN18	-1.307273508	4.28E-07	1.84E-06
AC034105.1	-3.079860646	0.014841628	0.022019862
CCK	-1.04477734	7.68E-05	0.000190681
AC148476.1	1.641801901	0.000264458	0.000579798
AC073283.1	2.760324243	2.85E-13	6.38E-12
STMN1	1.435192716	6.62E-14	1.79E-12
MACC1-AS1	1.509605644	0.000201465	0.000453253
AC011377.1	2.611868525	7.17E-06	2.27E-05
SYPL1P2	2.096379479	5.59E-11	6.56E-10
AC116914.1	2.145274837	2.42E-09	1.82E-08
ZNF135	-1.05064111	6.66E-05	0.00016754

AC005481.1	2.066044403	5.25E-05	0.000135323
SCLY	1.268687406	1.03E-15	5.96E-14
PLK1	1.866532638	4.57E-14	1.32E-12
AL133373.1	4.299577713	1.69E-07	7.97E-07
RBPMSLP	1.598701704	3.06E-05	8.35E-05
TGIF1	1.382672537	2.03E-18	1.46E-15
SNORD15A	1.491658015	1.58E-05	4.63E-05
OLA1P1	1.151757431	7.03E-09	4.68E-08
GAPDHP76	1.546695043	3.82E-08	2.12E-07
LINC02086	3.323989608	1.89E-11	2.53E-10
AC026979.1	1.424267459	1.07E-05	3.24E-05
AC120193.1	1.37386974	9.53E-06	2.93E-05
AC125603.1	3.269480419	0.00400939	0.006754658
DEFA6	-4.286526663	0.017604702	0.025685897
HLA-DPB2	1.354818282	8.99E-05	0.000219685
RN7SL481P	1.749370686	3.20E-09	2.33E-08
LYNX1	-1.886481444	3.47E-05	9.36E-05
HLA-DQB1-AS	1.109227802	0.001797928	0.003266753
TPSP2	4.678484409	3.14E-08	1.78E-07
AP005899.1	1.647940353	8.49E-11	9.49E-10
CHRDL1	-1.949240392	2.39E-09	1.80E-08
RAD21	1.093261423	4.75E-15	2.01E-13
AC002044.2	1.079726658	7.35E-05	0.000183207
GCNT7	1.402184355	3.64E-09	2.60E-08
AP002967.1	1.259624734	0.01821099	0.026500831
HS6ST3	-1.26224495	3.12E-09	2.28E-08
AP000907.3	1.851573004	0.000123118	0.0002911
ACTP1	1.401647149	2.32E-05	6.56E-05
RN7SL45P	1.013731523	0.001267099	0.002385559
LAMA3	1.302876669	0.000392297	0.000827979
AC011455.1	1.34892153	0.000208795	0.000468124
AC008443.5	1.526104273	0.000175464	0.000401122
MTND4LP5	1.366731043	0.000636639	0.001280004
TCP1P1	1.123589324	4.11E-07	1.77E-06
METTL1	1.274820615	1.88E-15	9.51E-14
AL645608.3	1.100918925	0.001142721	0.002174754
AC090116.1	4.797169629	7.84E-09	5.18E-08
AC124944.1	2.024072782	5.28E-12	8.23E-11
LMNB2	1.447668917	6.09E-15	2.45E-13
AC233300.1	1.057241483	1.45E-05	4.30E-05
AC009097.2	1.809983608	7.10E-09	4.72E-08

NKILA	2.206960669	4.57E-09	3.17E-08
AC008115.1	1.440637825	0.003268306	0.005616242
SLC2A3P4	1.28143384	2.21E-06	7.90E-06
RNU1-103P	1.362008494	3.06E-07	1.36E-06
AC106886.2	1.436729192	1.11E-08	7.03E-08
SNORA2A	1.188164751	0.000140948	0.000328878
RN7SL610P	1.408057299	0.000358876	0.00076361
PSD	-2.696156859	0.000104352	0.000250804
FAM155A-IT1	1.828413581	0.006039016	0.009767572
IMPDH1P8	1.876148534	6.68E-13	1.33E-11
AL359881.1	2.522693293	3.07E-06	1.06E-05
LINC02739	1.004563758	0.001637093	0.003002784
AP001011.1	3.165128853	5.29E-08	2.82E-07
SNORA71C	2.431346432	1.55E-14	5.35E-13
NDUFB4P3	1.009836765	0.025870992	0.03634161
AL603839.4	1.861350216	4.53E-09	3.15E-08
IGLVI-63	-4.337446335	0.000287025	0.000623729
FEN1	1.436918188	1.52E-14	5.24E-13
AC007342.9	1.991194193	3.00E-08	1.71E-07
AL627309.6	1.492948939	0.00013733	0.000321146
AC127024.8	1.587258333	3.17E-10	3.06E-09
ACRP1	2.828170551	0.000884466	0.001727056
ATP5PFP1	-1.410152142	0.002080711	0.00372793
AC007128.2	5.14767018	3.04E-14	9.32E-13
COMP	4.513224748	2.67E-08	1.54E-07
MIR4525	2.314153235	3.76E-08	2.09E-07
PPIAP68	1.419861988	3.69E-06	1.25E-05
ASB11	-3.496561407	0.00502502	0.008275224
AL671986.1	1.113698611	7.87E-07	3.15E-06
YES1P1	1.61952356	3.07E-09	2.25E-08
LYG1	1.711928024	1.43E-09	1.14E-08
AL022345.1	1.377725483	0.000137242	0.000321074
ATP8B5P	1.043286959	4.72E-06	1.56E-05
AC091057.1	2.051630208	4.16E-16	2.95E-14
ZP3	1.496037002	2.26E-06	8.06E-06
SLC12A2	1.097784449	0.000538829	0.001101979
AC034102.8	1.064415858	1.58E-05	4.63E-05
SFRP4	3.44886689	2.58E-09	1.92E-08
RBMS3-AS3	-2.005300146	1.71E-08	1.04E-07
SNORD99	2.383422865	4.43E-13	9.27E-12
CTSG	-1.25416628	3.04E-11	3.83E-10

TAF5LP1	1.222141711	0.000380806	0.000805514
Z82190.1	1.12617927	0.000426267	0.000891694
AC027288.3	2.208068498	1.95E-10	1.99E-09
STAC3	1.382020398	5.08E-12	7.96E-11
ENO1P3	1.901002371	7.60E-08	3.91E-07
RPS2P16	1.252867579	4.46E-09	3.10E-08
AL163952.1	1.504790333	0.026983657	0.037715681
PRKAR2B	-1.336169222	2.51E-09	1.88E-08
MIR219A1	1.354317765	0.000220131	0.000491265
SEC14L4	2.296303272	0.029022109	0.040243046
LINC02588	4.155911275	0.000506264	0.001042824
AC096861.1	1.425683024	0.003385685	0.005792911
AC036176.3	1.134679331	0.003464812	0.005914623
LINC02163	6.692784102	4.39E-14	1.28E-12
AC005306.1	1.260742111	9.37E-07	3.68E-06
INKA2	-1.015826447	2.28E-05	6.44E-05
ALKAL2	-1.470531852	5.89E-09	3.99E-08
NPIPB14P	1.398104106	1.28E-07	6.22E-07
AP000785.1	3.073340084	0.001167187	0.002215325
AL513188.1	1.407765374	0.001095151	0.002093342
ESCO2	1.514323477	6.68E-11	7.69E-10
SLC4A9	1.409036688	5.24E-06	1.71E-05
HNRNPA3P11	1.069459236	1.06E-06	4.11E-06
U7	1.003272111	0.007908703	0.012476531
UICLM	3.716177904	0.000164555	0.000378426
AC006160.1	1.260813437	2.60E-06	9.17E-06
AC125611.2	1.546532202	6.58E-09	4.41E-08
NOTCH4	1.034904964	9.37E-09	6.08E-08
RNY4P10	1.393135801	1.10E-08	6.97E-08
PCSK9	2.451202006	2.46E-07	1.11E-06
AP003498.1	2.394126403	0.000124622	0.000294239
AL137002.1	1.802206461	2.84E-06	9.92E-06
WNT2	4.821745395	1.50E-17	3.55E-15
ANGPT2	2.210730822	5.17E-17	7.40E-15
IGLV3-29	-3.869846152	0.003059343	0.005288199
AC079915.1	1.356800942	0.000110212	0.00026346
OIP5	1.598503171	1.26E-12	2.32E-11
AC005550.2	1.254600628	0.001282952	0.002410466
CREBZF	1.048994951	1.49E-12	2.69E-11
ARHGEF4	-1.465103543	0.005601161	0.009123633
GRB14	1.371774946	0.000165913	0.00038122

SUGT1-DT	1.573854479	1.94E-10	1.97E-09
AC108174.1	3.673690523	0.000647893	0.001300571
SCIN	-1.56017694	1.30E-05	3.90E-05
RANP6	1.134988042	4.31E-06	1.44E-05
AC114730.1	1.339187682	3.85E-08	2.14E-07
AC007376.2	1.675074675	2.03E-06	7.32E-06
ASB9P1	1.540152737	1.61E-08	9.82E-08
MIR616	1.56635033	3.23E-06	1.11E-05
CT75	-1.743935795	0.002320461	0.004117089
GADD45B	-1.938686842	1.27E-05	3.80E-05
AC009268.2	2.237879796	2.39E-10	2.38E-09
C8orf76	1.160205254	1.65E-14	5.61E-13
GSTA1	-2.72288946	0.000378546	0.000801688
AF124730.1	2.910444378	1.58E-08	9.68E-08
RPL19P13	1.402061087	7.22E-05	0.000180225
AC025062.2	1.827575872	0.003682689	0.006254891
CLDN1	2.37849782	6.56E-13	1.31E-11
SLC25A22	1.012913212	2.21E-08	1.30E-07
RPL38P6	1.175121767	0.001252923	0.002361206
PRAME	6.63907748	2.65E-10	2.62E-09
CCL21	-1.313861675	1.02E-08	6.53E-08
AL138789.1	3.670271836	1.36E-11	1.88E-10
KRT18P27	2.055574018	8.35E-11	9.37E-10
AC087362.1	1.496897215	9.52E-05	0.000231139
ITCH-IT1	1.317397458	6.10E-06	1.96E-05
AC021876.1	3.52503648	5.02E-08	2.70E-07
ZDHHC4P1	2.509784123	2.30E-11	3.01E-10
ASS1P1	1.536609041	5.29E-08	2.82E-07
PINK1	-1.000051957	7.37E-14	1.96E-12
AC109460.1	1.457969075	6.41E-13	1.29E-11
PRKAA2	-1.55495029	4.72E-06	1.56E-05
KIF26B	2.955301753	1.19E-16	1.24E-14
IGHV1-12	-2.962919098	0.017065335	0.024978281
RPL35AP17	1.122795887	0.000777739	0.001536101
AC131009.3	2.230846769	4.46E-17	6.87E-15
AC011287.2	5.816622428	4.34E-07	1.86E-06
AC016027.3	1.484379732	0.000162835	0.000374794
TNFRSF10B	1.705094935	5.57E-18	2.15E-15
KREMEN2	2.229748332	9.05E-10	7.67E-09
AP000344.1	1.710826665	0.008944296	0.013946736
FGFRL1	1.070427521	0.000276888	0.000604017

RNU6-1340P	2.121231571	1.89E-07	8.82E-07
DPH2	1.044459755	1.02E-14	3.76E-13
AC124944.2	1.926339878	7.16E-14	1.92E-12
AP001052.1	2.166857108	5.59E-12	8.66E-11
TCOF1	1.235538989	3.51E-17	6.07E-15
C17orf98	1.72873544	0.00071681	0.001426018
AC010542.1	1.869951578	6.40E-07	2.62E-06
HMGN1P4	1.027964508	1.60E-05	4.69E-05
PHBP7	1.208021806	5.23E-06	1.71E-05
AL162595.1	1.880925973	1.28E-15	7.08E-14
RIC3	-1.367866952	3.92E-08	2.17E-07
AC022079.1	1.106638727	0.000317567	0.000683631
AL109923.1	1.262816603	2.45E-05	6.87E-05
CCND1	1.053342082	2.09E-06	7.54E-06
RPL23AP44	1.121850928	3.01E-05	8.24E-05
STC2	1.991329991	3.13E-11	3.92E-10
AC007879.3	1.518832707	2.38E-05	6.68E-05
NCAPH	1.997380115	5.59E-15	2.28E-13
AF107885.1	1.115504865	3.37E-05	9.12E-05
BHLHA15	-1.486722263	0.03584913	0.048659741
H2BC19P	1.091029201	1.38E-06	5.21E-06
AL035252.3	1.834422364	1.38E-12	2.52E-11
AC073257.2	1.061807921	0.000801134	0.001577169
SNRPGP14	1.003899894	0.000148097	0.000344165
CCDC87	1.086502641	4.40E-05	0.000115586
AC004466.3	1.572581524	1.32E-07	6.38E-07
AC053503.5	-2.031194857	1.95E-05	5.60E-05
AC087588.1	2.269811759	1.47E-12	2.66E-11
ZNF525	1.53319016	1.38E-10	1.47E-09
KLK1	1.34521997	0.021326317	0.030553653
SMG1P6	1.409698892	6.22E-10	5.55E-09
HOXA4	-1.593908749	7.03E-07	2.85E-06
AC116025.1	1.054129705	0.032671378	0.044745328
AC090425.1	2.238397491	2.28E-07	1.04E-06
H3P43	1.102136227	1.07E-07	5.31E-07
PIK3AP1	1.689007552	1.23E-11	1.72E-10
SAC3D1	1.076778191	1.67E-11	2.26E-10
AL137856.1	1.318295332	0.00013072	0.000307356
RTKN2	1.505121185	1.68E-10	1.74E-09
AL391095.1	2.323983639	3.16E-11	3.95E-10
CHSY3	1.658299763	1.47E-12	2.66E-11

COLGALT1	1.28224212	2.30E-18	1.57E-15
AC125807.2	1.476191812	3.15E-14	9.59E-13
RNU6-238P	1.706444365	7.00E-06	2.22E-05
MIR4530	-1.750897754	0.001562567	0.002878573
AC105219.1	3.291727887	1.25E-12	2.31E-11
FNDC5	-1.928998422	2.61E-09	1.94E-08
CYP2A6	1.272110701	0.003432168	0.005866416
AC064847.1	1.530714636	1.91E-07	8.87E-07
PLCL1	-1.305723397	0.028001563	0.038968744
SHROOM3-AS1	1.500512754	3.26E-07	1.44E-06
RN7SL141P	1.130065398	0.001334815	0.00249699
AC009704.2	1.544663659	4.59E-09	3.19E-08
AC007240.1	2.677535044	2.46E-09	1.84E-08
IGLV1-50	-3.621034076	0.001304852	0.002445845
MKRN9P	2.326863201	0.000198764	0.000447679
RPGRIP1L	1.136957319	2.66E-14	8.33E-13
AC022432.1	2.168665341	1.74E-05	5.06E-05
AC092681.1	2.103533216	1.40E-08	8.69E-08
LRRC69	1.624857701	2.42E-11	3.14E-10
PPIAP89	1.375840304	4.37E-05	0.000114717
SNHG31	1.285383837	5.86E-06	1.89E-05
GIP	-5.272939042	0.000193373	0.000437375
AC020658.4	2.014289257	1.72E-08	1.04E-07
SSTR5	2.293632371	0.015558697	0.022986458
AC131212.3	1.45260789	1.60E-09	1.26E-08
PA2G4P6	1.238892739	2.40E-11	3.11E-10
PLK5	1.099976018	0.035486496	0.048223985
AC003965.1	1.204996281	2.80E-05	7.74E-05
S100A3	1.365145151	1.65E-07	7.81E-07
PROSER1	1.398270334	4.65E-17	6.92E-15
SPRY4	1.180199362	2.35E-11	3.06E-10
UBE2T	1.992158003	2.88E-15	1.34E-13
H2AC14	1.428911006	4.05E-05	0.000107493
AC116025.2	2.627359302	2.06E-12	3.57E-11
FOXJ1	3.427017313	0.000265341	0.000581459
AC004898.1	1.476277419	2.56E-09	1.91E-08
SFMBT1	1.170065838	1.93E-15	9.68E-14
NME1	1.495223051	5.99E-13	1.21E-11
BX322650.1	1.514164742	5.47E-10	4.96E-09
AC011498.5	1.048150878	0.001748405	0.003187206
RUVBL1	1.253589118	4.52E-17	6.89E-15

MTND2P40	1.653456884	2.17E-07	9.96E-07
AP000695.1	2.431617764	7.55E-14	2.00E-12
AL022394.1	1.081956762	0.003730515	0.00632644
RN7SL636P	1.862299522	2.99E-05	8.20E-05
ACRV1	1.981352021	1.69E-12	2.99E-11
ZNF224	1.035778483	3.20E-11	3.99E-10
AC010422.2	1.094289782	8.82E-06	2.74E-05
RN7SL200P	1.41734094	2.04E-06	7.37E-06
NBEAP2	1.662066459	5.12E-15	2.13E-13
RN7SL472P	1.10277681	0.004378228	0.007308111
RNU7-128P	1.348069151	0.007934244	0.01251312
RNU4-8P	1.189862093	0.001613117	0.002961865
N4BP2L2-IT2	1.212770516	2.70E-08	1.55E-07
TOMM40P1	2.924598508	2.43E-12	4.14E-11
AC020928.1	1.65910173	0.007456132	0.011827702
CRYBB3	1.367393819	2.61E-08	1.51E-07
AL157871.2	1.478781374	6.66E-06	2.12E-05
SEPTIN5	-1.458709023	2.41E-05	6.76E-05
RN7SKP299	2.096006445	4.40E-09	3.07E-08
AC092691.1	-2.238672773	7.79E-14	2.06E-12
LINC02728	1.165186499	0.000136438	0.000319314
LINC01311	1.167536284	3.71E-09	2.65E-08
AC117489.1	-1.413938835	5.04E-06	1.66E-05
PHF2P2	5.272457508	0.009991341	0.01542143
RNA5SP474	1.319674529	3.65E-05	9.77E-05
AC018695.3	1.614896575	1.18E-07	5.80E-07
LINC00685	1.15826213	2.00E-05	5.72E-05
AP000593.3	1.686023849	1.29E-06	4.90E-06
C10orf88B	2.702470765	4.21E-12	6.74E-11
HLA-T	1.266900284	0.00139638	0.002601745
GDPD3	-1.147497975	9.78E-05	0.00023685
RN7SKP219	1.47494142	0.000781462	0.001542429
PPEF1	1.59454362	1.91E-08	1.14E-07
CDC25C	1.787201837	4.14E-13	8.73E-12
AL606534.4	1.881439542	9.29E-06	2.87E-05
RPL23AP59	1.001115802	0.000909116	0.001770516
SOX9-AS1	1.269487906	5.68E-05	0.00014517
AC106037.1	2.629644355	3.03E-05	8.29E-05
RN7SL454P	1.081968019	0.004976204	0.00820244
AC087741.1	1.53684883	3.63E-10	3.43E-09
AC116348.1	2.186093153	1.05E-06	4.09E-06

C4BPAP2	2.849642716	2.45E-05	6.85E-05
KIFC2	1.188730987	1.06E-08	6.75E-08
MT-TY	-1.414015351	7.44E-05	0.000185401
CPSF4L	1.125112394	0.000426076	0.00089166
AL049794.1	1.449974681	4.69E-07	1.99E-06
RN7SL351P	1.786880886	2.25E-07	1.03E-06
RNY3P12	1.795201287	0.00037496	0.000794598
AC008456.1	1.814375174	0.000102464	0.000246911
RPL21P110	1.209935276	6.56E-06	2.09E-05
ST6GAL2	-1.46118868	0.010982541	0.016778326
SP8	5.781280874	1.37E-09	1.10E-08
AC100791.2	4.158892815	5.25E-07	2.20E-06
RTN4RL2	2.024402909	9.32E-10	7.86E-09
MCM6	1.241937647	6.02E-15	2.42E-13
MIS18A	1.288046806	5.16E-13	1.06E-11
AL024507.2	1.015380785	3.22E-09	2.34E-08
RN7SL395P	1.053522203	0.000861121	0.001684063
AL031736.2	1.519262279	0.007280312	0.011574298
AL121772.1	2.377119042	5.91E-09	4.00E-08
VMP1	1.118506131	1.97E-13	4.59E-12
COX6B2	3.337334378	2.26E-12	3.87E-11
SUV39H1	1.138780824	9.35E-13	1.79E-11
MARS2	1.053541233	2.11E-11	2.79E-10
MIR624	1.385063828	0.001609468	0.002956388
MT1L	-1.056675851	2.66E-09	1.97E-08
AC068790.5	1.185027524	1.03E-06	4.01E-06
DNAJC18	-1.011347581	0.000444187	0.000926343
MPPED2	-1.504859628	0.007478265	0.011859282
H2AC17	1.764494475	7.13E-09	4.74E-08
CDC42P4	1.31372367	1.18E-05	3.55E-05
AC015908.3	-1.501028946	7.03E-09	4.68E-08
AC024451.4	1.887329741	1.63E-10	1.70E-09
AMTN	-2.553923594	8.65E-06	2.69E-05
AC087362.2	1.071781889	7.53E-05	0.000187333
HLA-DOA	1.057725837	0.012439759	0.018769694
AC005046.1	1.523823799	1.05E-08	6.70E-08
SMTN	-1.867003408	0.015207399	0.022506139
LINC02804	1.41018892	9.90E-06	3.04E-05
PLPP4	1.942359962	0.000655702	0.001314651
AL390719.2	1.590243398	9.42E-09	6.11E-08
AC005277.2	1.498729146	3.71E-05	9.93E-05

CDKN1A	-1.064087516	1.82E-06	6.65E-06
ZWINT	1.72534234	4.41E-14	1.28E-12
AC020915.1	1.349112989	3.92E-08	2.17E-07
AC104446.2	1.831327196	1.09E-10	1.19E-09
AC108676.2	2.05276791	0.000150889	0.000349989
NHS	1.488094965	2.05E-09	1.57E-08
AL591473.1	1.496273574	0.000960594	0.001863144
WNT9A	-1.350918042	0.000496589	0.001024796
DYRK3-AS1	1.600966446	5.40E-05	0.000138805
AC023794.3	-1.340410574	2.00E-08	1.19E-07
BEST3	3.537263364	9.58E-07	3.76E-06
EMX1	2.644288222	1.18E-05	3.57E-05
IGHV3-22	-2.624248636	0.016912336	0.024781567
C22orf23	-1.225661897	1.86E-08	1.12E-07
AL132800.1	1.032289281	9.77E-05	0.000236615
RN7SKP95	1.90049726	5.34E-06	1.74E-05
AC023632.5	1.240980286	0.000189731	0.000430038
NSD2	1.319052848	1.22E-16	1.25E-14
BPIFB9P	1.397943464	8.59E-05	0.000210649
AC034213.1	2.332020674	0.001475677	0.002733625
AL596247.1	1.293967688	3.41E-05	9.21E-05
RNU6-1300P	1.776837041	0.001423987	0.002647074
HS3ST6	-1.744480425	2.93E-07	1.31E-06
FOSL1P1	1.091982548	2.23E-05	6.32E-05
ID4	-1.216478887	1.76E-11	2.37E-10
LRRFIP1P1	1.436476472	1.72E-08	1.04E-07
RNU4-52P	1.30511941	4.20E-05	0.000110986
DNAJB5	-1.9340083	8.73E-07	3.46E-06
MRM1	1.124548538	2.81E-11	3.59E-10
KRT18P4	1.723618778	2.86E-14	8.87E-13
RNU7-123P	1.3832171	0.000111678	0.000266581
FLNC	-1.62467383	0.000508331	0.001046434
AC004461.1	1.05469065	2.04E-05	5.82E-05
MIR196A1	3.041253655	3.59E-09	2.58E-08
AC093249.2	1.086542796	5.81E-06	1.88E-05
AC092849.1	1.335993417	4.34E-05	0.000114109
KIT	-1.26722294	3.18E-10	3.07E-09
AC090181.3	1.716877517	2.30E-10	2.30E-09
IGKV1D-42	-3.811116935	0.005334051	0.008731784
AC103760.1	1.721014482	4.08E-09	2.89E-08
CORO6	-1.611338104	0.025121151	0.035383437

PTCH2	1.147970923	0.014437544	0.021468161
SNORD63B	1.12352892	1.67E-06	6.16E-06
NRBF2P5	1.471507599	8.79E-10	7.49E-09
MIR4664	1.441629171	6.05E-09	4.08E-08
RN7SL329P	1.210216016	1.98E-05	5.68E-05
DCN	-1.182950285	2.81E-07	1.26E-06
AC068533.3	1.530414097	2.55E-08	1.48E-07
NXPH4	2.836919738	1.70E-07	7.99E-07
CAPN11	1.425850492	0.000156799	0.000362279
MIR570	1.112675432	6.28E-06	2.01E-05
IGKV3OR2-268	-3.215209779	0.024366228	0.034416554
AC002351.1	3.488937993	0.002844195	0.004947121
SNORA63	1.591798348	6.62E-06	2.11E-05
ACTBP8	1.143815001	0.000883389	0.001725206
CARD11	1.189383678	3.45E-05	9.30E-05
AL512656.1	1.529129256	1.37E-07	6.59E-07
RETREG1	1.370907918	2.51E-05	7.02E-05
RN7SL37P	1.261328155	0.032735886	0.04483137
SNRPFP2	1.185592678	0.004843682	0.008003893
ACTG2	-1.993278501	2.21E-08	1.30E-07
VAC14-AS1	1.573940045	1.51E-12	2.71E-11
AC012354.2	5.502946749	5.91E-07	2.45E-06
FIRRE	3.679016326	1.40E-14	4.87E-13
IGHV3-42	-3.305346051	0.002810139	0.004893316
IFNL1	1.902273109	0.000378733	0.000802019
RNU6-795P	1.574916888	1.53E-05	4.51E-05
RPTN	-2.070093948	3.17E-06	1.10E-05
AMH	3.382191282	1.27E-09	1.03E-08
MIR5692C2	1.605020395	0.000343341	0.000733779
JTBP1	1.147791268	0.022894021	0.032559788
AC105910.1	1.590476474	3.45E-07	1.51E-06
HMGN2P18	1.144370287	0.009889309	0.015276336
DMRTA1	-1.660841901	1.09E-10	1.19E-09
MYMX	-1.564600524	9.23E-06	2.85E-05
CEMIP2	1.368363557	1.83E-11	2.46E-10
AP001207.1	1.488752409	2.05E-05	5.87E-05
AC073592.1	1.282759066	4.38E-07	1.88E-06
LRRN4CL	-1.438612795	3.40E-08	1.91E-07
BTBD19	1.114400399	4.29E-08	2.34E-07
TYMS	1.769143984	8.20E-15	3.10E-13
RNU6-57P	1.668039929	0.000429608	0.000898049

AC119800.1	1.201579689	0.000353324	0.000753001
PGC	-2.555190893	0.011717063	0.017784196
AKR1C3	-1.152738556	0.013702267	0.020462769
RANP8	1.507718286	3.44E-06	1.18E-05
AC009005.1	1.651187957	4.17E-08	2.29E-07
NUP188	1.006782203	3.03E-13	6.71E-12
MTHFD2P7	1.431648049	1.17E-07	5.73E-07
SNRPF	1.164724918	3.49E-12	5.70E-11
PHACTR2-AS1	1.350850755	8.13E-07	3.25E-06
PTPN2P1	1.268317705	1.69E-08	1.02E-07
PPIAP13	1.326228347	2.20E-08	1.30E-07
AP001267.3	-1.440350188	1.16E-06	4.47E-06
ANGPTL1	-2.450174425	5.58E-10	5.04E-09
LSM3P2	1.146378738	0.000247393	0.000545615
AC008870.4	1.39635423	3.59E-07	1.57E-06
PANK2-AS1	1.103307625	2.88E-07	1.29E-06
NPY	-1.739959688	2.63E-07	1.19E-06
JPH2	-1.883648425	2.98E-06	1.03E-05
AC092894.1	1.533580534	0.006478422	0.010414416
SULT1B1	-1.495860219	0.013405795	0.020067266
AP001025.1	2.413124551	0.005503932	0.008978433
IGHV3OR16-16	-2.783556961	0.027441164	0.038291851
ENDOU	-4.504710061	4.18E-06	1.40E-05
MIR4755	1.865130846	2.42E-06	8.59E-06
AL359510.2	1.013237107	0.000102967	0.00024793
AC005740.3	1.404860653	5.97E-06	1.93E-05
AC046143.2	1.417737859	9.84E-10	8.24E-09
AC079354.1	2.123600904	2.67E-07	1.20E-06
CDK6	2.155335299	2.60E-13	5.88E-12
TRAIP	1.563277895	6.02E-14	1.66E-12
GPR4	1.100587921	1.77E-09	1.38E-08
ZMYND15	1.437506134	4.71E-10	4.34E-09
AC005037.1	1.219620774	3.43E-08	1.93E-07
HNRNPA1P53	1.099428601	1.12E-06	4.33E-06
AC124283.2	1.513811787	8.81E-09	5.76E-08
AL157838.1	2.042661188	2.44E-14	7.79E-13
AC026369.2	2.817675716	5.45E-17	7.52E-15
AC107464.1	1.482658642	2.69E-05	7.46E-05
ZNF146	1.028812948	5.90E-17	7.79E-15
CPO	-2.287793078	2.90E-06	1.01E-05
AL049836.1	3.33226278	6.69E-10	5.91E-09

AC022400.8	1.291005903	0.000314137	0.000676958
CLIP4	-1.09298404	0.000698361	0.001391916
AL590133.1	1.751911947	3.66E-10	3.46E-09
TSPAN2	-1.419182593	0.000985189	0.001905576
AC004494.1	1.127888594	0.000591192	0.001196306
SNORD13D	1.1939693	9.92E-05	0.000239841
AL139246.5	1.026931154	4.69E-07	1.99E-06
EFCAB14-AS1	1.130863842	5.73E-06	1.86E-05
AL590096.1	1.061521751	0.001495309	0.002764034
BCAR4	5.50116764	1.33E-05	3.96E-05
PCDH17	1.0386173	5.89E-07	2.44E-06
TFF1	-2.797361674	0.005060825	0.008326468
SIGLEC11	-2.32067657	0.000154351	0.00035715
ADGRG1	1.399611492	1.56E-10	1.63E-09
IP6K3	-1.457846622	1.99E-09	1.53E-08
AC006449.3	1.801993863	1.99E-09	1.53E-08
RNU5B-1	1.373561129	0.000392604	0.000828495
AC073288.2	2.143383574	6.19E-08	3.25E-07
IGKV2D-29	-3.277285039	0.029253388	0.040538425
LINC01614	5.100158718	3.43E-15	1.55E-13
AL358232.1	2.417297128	4.51E-11	5.42E-10
TROAP	2.357639289	3.29E-16	2.47E-14
CHEK1	1.860475027	7.84E-18	2.58E-15
LCTL	1.713893286	1.22E-11	1.71E-10
AL109614.1	1.464322868	7.98E-08	4.08E-07
AC134050.1	1.675707097	2.92E-08	1.67E-07
AC025176.1	2.396133452	4.11E-10	3.85E-09
AC009093.1	2.256243414	1.10E-12	2.06E-11
IL17B	-1.124960889	0.000743534	0.001474348
AL136310.1	1.242513451	3.36E-05	9.08E-05
MIR7111	1.169301845	5.93E-05	0.000150858
NPM1P12	1.083348227	1.22E-05	3.67E-05
LINC00865	-1.01648236	3.24E-07	1.43E-06
Z98742.3	2.15463494	2.93E-06	1.02E-05
GIHCG	-1.018837696	3.95E-05	0.000105112
RPSAP74	1.842713692	1.44E-06	5.41E-06
UBAP2	1.002557667	1.16E-16	1.22E-14
AC011287.1	4.420186617	1.61E-06	5.98E-06
AIFM3	1.700993168	1.80E-07	8.43E-07
DDX39BP1	1.272528117	0.004987172	0.008219199
AC004837.2	1.579447438	6.15E-10	5.50E-09

CENPN	1.506582105	1.51E-15	8.01E-14
AC104971.3	-1.485310486	0.000608531	0.001227846
TAS2R20	1.387430544	2.57E-07	1.16E-06
RNU6-944P	1.433858886	0.006303395	0.010156953
AC114763.1	1.046721251	3.65E-08	2.04E-07
TEX45	1.483998956	2.22E-06	7.96E-06
AC005224.3	1.274737035	0.00050537	0.001041066
LINC00707	1.048190569	0.006124088	0.009893158
IGFL2	3.614673932	2.18E-11	2.87E-10
MUC21	-4.707578668	0.019414103	0.028035695
AC009121.3	1.299518371	3.46E-07	1.52E-06
AL161729.3	1.747536555	5.58E-08	2.96E-07
RNA5SP132	1.39970867	0.004417532	0.007364028
MED28P7	1.645126615	1.00E-05	3.06E-05
AC004691.1	1.458122927	6.10E-06	1.96E-05
HUNK	1.67432187	0.011953715	0.018102136
CHST13	2.858323564	1.72E-05	5.01E-05
DPP6	-3.244298953	1.25E-08	7.82E-08
IFITM8P	1.568861801	2.24E-05	6.36E-05
LINC01136	1.482634406	0.000145371	0.000338417
SNORA38	1.49338245	5.53E-05	0.000141749
MCM8	1.736004989	4.92E-18	2.02E-15
MAGEA10	7.753604404	0.000359315	0.000764485
TMEM26	2.187438672	5.45E-17	7.52E-15
LINC00114	2.786518892	0.026074842	0.036591306
C2orf49-DT	1.281484715	3.48E-10	3.32E-09
CT55	5.008210047	0.000323273	0.000694905
SNORA75	1.268587686	8.05E-08	4.11E-07
AL135844.1	1.499982185	1.52E-05	4.47E-05
AC007639.1	1.974222008	1.35E-09	1.09E-08
RNU7-3P	1.925501002	3.70E-06	1.25E-05
HNRNPA1P40	1.109898176	1.90E-06	6.92E-06
AC139720.2	2.438433917	0.015683794	0.023152019
AP000580.1	1.136034206	1.22E-07	5.95E-07
GJB7	3.014875157	0.000966795	0.001874489
ZNF789	1.438231101	1.13E-14	4.05E-13
AL645924.1	2.215684538	0.000242973	0.000537157
NSUN2	1.153260269	9.89E-17	1.08E-14
AP001469.3	1.151252666	9.23E-10	7.80E-09
AC137894.1	2.195499944	0.002983132	0.005167866
CKB	-2.220820635	2.50E-10	2.48E-09

APOA2	8.806732265	4.03E-05	0.00010705
AC020931.1	1.021380354	1.01E-06	3.93E-06
AKAP5	1.266094106	1.66E-09	1.30E-08
FKBP9	1.040355791	1.25E-14	4.40E-13
AC020913.3	1.213709116	1.21E-06	4.62E-06
NIPAL4	-2.176099855	2.51E-05	7.02E-05
RNU6-1281P	1.014816999	0.000294649	0.000638682
RRP9	1.094003834	1.91E-12	3.33E-11
AC092115.3	1.125100812	0.006829467	0.010925999
NDUFA3P4	1.344246085	0.001233702	0.002330093
ZNF331	-1.455332536	0.00787134	0.012419796
AKR1C2	-1.903413147	3.21E-13	7.04E-12
TFDP1P2	1.024945176	3.79E-05	0.000101343
AC009690.2	1.128085038	1.51E-12	2.71E-11
AC067750.1	-1.210569652	0.021861614	0.031248303
AC012173.1	1.244888982	4.74E-07	2.01E-06
AL136115.2	2.226905745	2.54E-12	4.30E-11
AC093001.1	5.038231948	1.99E-05	5.70E-05
AL035458.1	1.329754592	4.88E-07	2.06E-06
AC107959.1	-1.255797799	7.93E-07	3.18E-06
MIR4292	1.754944864	2.42E-11	3.14E-10
AL023755.1	2.627886105	7.53E-07	3.03E-06
RN7SKP237	1.023980492	1.28E-05	3.84E-05
AL049869.3	1.311341022	0.003738068	0.006338442
SETP8	1.347466289	3.05E-08	1.73E-07
PPIAP35	1.386633661	9.65E-08	4.84E-07
RPL21P106	1.066900747	0.00094357	0.001833462
LINC02416	1.590222026	9.15E-07	3.60E-06
RNU6-570P	2.159167306	0.000290666	0.000630613
G2E3-AS1	4.49168314	5.40E-08	2.88E-07
AC109322.1	1.084362049	7.43E-10	6.47E-09
NTN1	-1.779046603	9.51E-10	7.99E-09
MIR6774	1.601108775	4.99E-06	1.64E-05
AC092723.1	2.276580839	0.000195931	0.000442034
TBC1D31	1.730770227	1.44E-18	1.31E-15
CSRP3-AS1	2.181109802	5.98E-08	3.15E-07
LRRC8D-DT	1.693163747	2.24E-10	2.25E-09
GPR176	1.460411284	1.75E-10	1.81E-09
KCNS1	2.650657238	0.001846071	0.003344191
KIFC1	1.764893101	3.60E-13	7.77E-12
MS4A14	1.405411283	1.12E-07	5.51E-07

MASTL	1.473960147	2.51E-17	4.99E-15
RPS4XP5	1.170549015	3.82E-06	1.29E-05
JAM3	-1.038215349	0.006402469	0.01030102
AC022973.3	1.924165452	1.66E-10	1.73E-09
ZNF697	1.40465045	3.07E-14	9.39E-13
OR7E102P	1.125688879	0.008371278	0.013134723
RNU2-24P	1.744645829	0.000625602	0.001259714
LINC01691	5.506198556	1.68E-09	1.32E-08
AL354836.1	1.145279619	4.68E-08	2.53E-07
KIRREL3	-1.210522043	9.96E-06	3.05E-05
FAR2P1	6.8417975	2.05E-11	2.73E-10
AC018695.4	1.578078179	3.20E-11	3.99E-10
CCL15	1.230449431	4.48E-06	1.49E-05
SPDYE20P	1.593112188	0.000508761	0.001047158
CCDC14	1.485234421	3.80E-15	1.68E-13
AL109804.1	1.034040409	0.007329023	0.011645485
LINC01234	6.873979816	3.01E-13	6.68E-12
AL158068.2	1.632461037	1.17E-05	3.52E-05
UBL5P4	1.937070124	0.000392537	0.000828419
CITED2	-1.393163487	5.12E-15	2.13E-13
snoZ196	1.858073084	3.18E-06	1.10E-05
IGKV1-17	-3.255509941	0.027011058	0.037744412
KAT2B	-1.209087786	3.95E-13	8.42E-12
RN7SL589P	1.065487352	0.000163559	0.00037643
AC087257.1	1.512274139	4.75E-05	0.000123918
NEXMIF	-1.793893839	1.05E-09	8.72E-09
HOXA10-AS	4.545931575	1.49E-12	2.69E-11
AC078860.1	1.090471109	0.00417271	0.006999707
C10orf55	1.49356778	6.05E-09	4.08E-08
SNORD18	2.077371487	4.25E-07	1.82E-06
RNU6-1095P	1.27556053	4.50E-05	0.000117881
AP003390.1	1.273177047	0.005764349	0.009365602
AL355075.5	1.215140102	0.001082627	0.002073542
HOXA13	3.617172101	2.75E-13	6.19E-12
TSPEAR-AS2	2.466695391	5.67E-09	3.85E-08
AC020908.3	1.115373565	7.15E-05	0.000178892
OPA1-AS1	1.670457255	1.53E-08	9.39E-08
AC034102.5	1.217986467	0.000356252	0.000758998
AC009686.1	1.228859265	1.21E-05	3.63E-05
AL929236.1	1.558230184	6.55E-07	2.68E-06
PRR13P2	1.265053079	9.87E-08	4.94E-07

SNORD63	1.380479061	3.05E-08	1.73E-07
CDK12	1.253700284	8.65E-16	5.25E-14
S100A8	-3.126825236	0.001088695	0.002083244
AC008751.1	1.846239526	5.62E-05	0.000143818
EPSTI1	1.425776381	1.97E-08	1.17E-07
AL365223.1	1.730810895	8.61E-10	7.36E-09
AC026333.4	1.651797537	1.01E-08	6.47E-08
AC006273.1	2.436180929	1.73E-10	1.79E-09
GPR158	2.49726596	5.19E-05	0.00013402
AC093525.9	1.385295535	5.44E-08	2.89E-07
SAMD5	1.061408504	0.000710422	0.001414051
AC011676.2	3.181444579	1.05E-12	1.98E-11
AC011731.1	1.099867596	8.61E-07	3.42E-06
LINC00471	1.222409303	1.66E-05	4.85E-05
AC087481.1	1.34122505	5.71E-06	1.85E-05
IL17RB	2.262502094	4.64E-15	1.97E-13
CCNF	1.830348732	2.74E-15	1.30E-13
AC093787.1	-2.038675833	0.015096048	0.022357492
TMPO-AS1	1.281367423	6.15E-11	7.15E-10
ZNF888	1.493554983	3.44E-11	4.27E-10
LAPTM4BP2	1.07053208	5.41E-06	1.76E-05
MIR6832	2.083670258	3.78E-05	0.000100932
AC006017.1	1.32231649	6.62E-07	2.70E-06
CPLX1	1.907334016	1.43E-09	1.14E-08
AC004941.2	1.368683668	4.71E-06	1.56E-05
SEC61A2	1.163703542	9.18E-12	1.34E-10
AP002812.1	1.18104506	2.56E-05	7.13E-05
AP002340.1	1.18691721	1.64E-05	4.78E-05
TNS3	1.071412612	7.04E-11	8.04E-10
HRH2	-1.497027808	0.00042121	0.000882014
MIR3176	2.2286877	3.19E-12	5.25E-11
OBI1	1.270956161	2.99E-17	5.42E-15
H2AC16	1.52566908	1.03E-05	3.15E-05
RPS27P14	1.057233265	0.00764923	0.012106623
LGR4-AS1	1.325969044	2.27E-06	8.12E-06
AL353572.1	1.489122329	0.005929859	0.009609689
STRIP2	2.894002054	1.18E-17	3.20E-15
ENTPD3	-1.332041759	7.93E-09	5.23E-08
AC006480.2	1.200370667	1.36E-07	6.55E-07
KIF24	1.755556004	1.06E-15	6.05E-14
AC121493.1	1.097379803	1.88E-05	5.42E-05

LINC02575	2.269329679	6.74E-06	2.14E-05
MIR643	1.765921977	9.19E-06	2.84E-05
RN7SL684P	1.600558422	9.12E-06	2.82E-05
AC087491.1	2.016245181	8.87E-05	0.000217096
RNASE2	2.351035965	3.37E-07	1.48E-06
AC005632.3	1.04538817	8.41E-08	4.28E-07
AL360270.2	1.177956992	2.62E-05	7.29E-05
AL110115.2	1.185204448	0.000551352	0.001125173
H2BC11	1.574423222	4.31E-05	0.000113461
AC025165.1	-1.024034657	0.026043837	0.036560346
KCP	1.52675955	1.15E-06	4.43E-06
PEF1-AS1	1.632322515	1.19E-06	4.56E-06
XIRP1	3.148805997	7.57E-11	8.59E-10
MAGEA6	9.267447981	1.93E-07	8.99E-07
IGHV4-55	-2.778943928	0.021996755	0.031429666
CHADL	-1.114487608	4.19E-09	2.95E-08
AC005070.1	1.105783405	0.000664155	0.001330207
PPIAP36	1.248772792	4.16E-08	2.29E-07
SLC30A2	2.441716889	0.005628025	0.009161423
AC124319.1	1.296336601	1.70E-07	8.02E-07
NOP2	1.497126506	3.64E-18	1.72E-15
GLTP	-1.434487008	0.008437159	0.013223295
BAIAP2L1	1.030587626	9.93E-06	3.04E-05
RNA5SP111	1.307829534	0.028028569	0.03900429
WASIR2	1.623920086	1.02E-05	3.12E-05
CABYR	1.247758573	0.0001149	0.000273271
AC079907.1	1.495741425	1.70E-08	1.03E-07
CARNMT1-AS1	1.16200948	1.71E-05	4.98E-05
PGM5-AS1	-3.340864035	5.00E-13	1.03E-11
RNU4-36P	1.191639077	0.008562596	0.013400968
AC092687.3	1.190998337	2.91E-06	1.01E-05
AC024060.2	1.206982736	9.37E-09	6.08E-08
ARPC1B	1.121556982	1.58E-08	9.66E-08
ZNF775-AS1	1.271356581	0.000152536	0.000353473
AC006272.2	1.264452725	0.000226355	0.000503931
AL138963.1	1.292008562	8.01E-06	2.51E-05
AC024560.1	2.634792338	0.000426746	0.000892555
TMEM241	1.044626851	5.80E-12	8.93E-11
RHPN2P1	1.234956654	5.49E-05	0.000140903
MYOCD	-1.716323885	8.26E-08	4.21E-07
DUSP9	4.236000456	6.59E-06	2.10E-05

ZNF887P	1.915297044	2.01E-13	4.69E-12
SNRPGP17	1.049405608	0.020481616	0.02943528
AC010333.1	1.855530337	5.07E-06	1.66E-05
PRB4	3.220733786	0.000719276	0.001430178
RNA5SP108	1.755168381	1.41E-06	5.31E-06
RN7SL556P	1.971058386	0.000237366	0.000526115
CD84	1.372145457	2.65E-06	9.33E-06
MAB21L1	-1.753126117	2.80E-10	2.75E-09
AC020907.1	1.909610383	0.000271857	0.000594467
AC087685.1	1.102339639	0.025639185	0.036044492
AL359075.1	3.09272615	4.79E-12	7.57E-11
CNRIP1	-1.000106966	5.08E-06	1.67E-05
CENPQ	1.200940558	1.12E-12	2.10E-11
MAP3K20-AS1	2.521318818	0.00189089	0.003415395
AC125257.1	1.479565111	1.46E-15	7.82E-14
BRCA1P1	1.374242497	1.33E-06	5.05E-06
AC010463.2	1.20676645	0.009999412	0.0154321
TRIM31	1.552327926	3.54E-07	1.55E-06
ITIH2	4.742708562	5.73E-06	1.86E-05
IGSF11	-2.320971906	8.19E-07	3.27E-06
RPL23AP11	1.127907499	2.37E-05	6.68E-05
RNU6-97P	1.159827558	0.00085936	0.001680989
LINC01914	4.070022519	2.99E-15	1.39E-13
AC133065.3	1.0326546	0.000103017	0.00024793
AC026271.1	1.218405683	3.13E-13	6.90E-12
LINC01287	5.881434605	0.001710003	0.003124041
RNU6-571P	2.075052267	9.89E-05	0.000239286
DUTP2	2.179069199	2.17E-10	2.18E-09
AL035252.4	1.592844397	5.47E-09	3.73E-08
SEPTIN7P3	1.014785841	1.99E-05	5.71E-05
AC012170.1	1.195679068	9.66E-08	4.84E-07
IGLV3-13	-4.30792521	0.000723137	0.001437104
AL031716.1	1.540804803	4.83E-10	4.44E-09
AC067852.4	1.381678435	8.78E-08	4.44E-07
AVL9	1.183615693	7.70E-17	9.24E-15
DRAXIN	1.988670008	4.85E-10	4.45E-09
LINC02321	2.919555865	1.05E-09	8.72E-09
PAFAH1B3	1.306425765	4.78E-11	5.71E-10
WDR76	1.210909546	1.33E-13	3.27E-12
KIF18A	2.43541636	5.83E-17	7.76E-15
DDIAS	2.246690532	1.06E-15	6.05E-14

ADGRF5	1.002113604	1.24E-07	6.03E-07
TRAJ2	2.101154927	2.98E-05	8.17E-05
AC079336.6	1.153028418	1.17E-05	3.54E-05
AL354813.1	1.385398927	1.69E-06	6.22E-06
LRRC37A6P	1.753838536	1.82E-05	5.26E-05
LINC01556	1.381659955	0.007492141	0.01188058
RPS3AP19	1.971207191	1.06E-08	6.78E-08
AC009283.1	1.397306388	4.65E-05	0.00012129
GKN1	-4.413963865	5.78E-07	2.40E-06
AC036108.2	-1.075675683	0.005508854	0.008985911
RN7SKP97	1.78154612	7.96E-08	4.07E-07
HSPE1P6	1.952765933	1.17E-10	1.27E-09
PARP15	1.136298243	0.000136465	0.000319314
HSPA8P14	1.392353313	1.36E-06	5.14E-06
SNORA31	1.287136331	7.69E-06	2.42E-05
RPL28P2	1.298678994	8.93E-07	3.53E-06
AC009097.4	-1.077804258	0.000179189	0.000408834
NPFFR1	2.455565332	1.56E-09	1.24E-08
HMGA1P1	1.594125866	4.17E-09	2.94E-08
SLED1	1.44102732	0.000244994	0.000540963
NPM1P46	1.481760825	8.78E-09	5.74E-08
ULBP2	1.779910874	4.52E-10	4.19E-09
CYP4F12	-1.013838864	0.000786949	0.001551764
RPL35AP23	1.525137468	1.52E-05	4.48E-05
GTF2IP13	1.487103588	2.24E-09	1.70E-08
ITGA11	1.963339006	1.45E-09	1.15E-08
BBIP1P1	1.542714669	4.46E-06	1.48E-05
AC090844.1	1.463890116	1.85E-05	5.34E-05
ESM1	5.063219418	3.05E-20	4.50E-16
RNU6-790P	1.238766616	0.000377795	0.000800225
AC007193.2	-1.030867976	0.016176222	0.023784045
SLC7A3	-2.916607195	4.31E-10	4.01E-09
RIMS1	-1.104513204	1.83E-08	1.10E-07
AL391994.1	1.666282742	3.23E-07	1.42E-06
CENPK	1.816846426	5.59E-15	2.28E-13
AC015712.1	1.515336769	0.032149438	0.04410305
AL031985.3	1.294726312	3.52E-13	7.63E-12
AC044840.1	1.615417073	1.51E-06	5.63E-06
ADPGK	1.082857705	5.80E-15	2.35E-13
REP15	-2.126777391	0.00010104	0.000243733
STPG3	2.061666494	2.66E-08	1.53E-07

AL355863.1	1.211001886	0.002532511	0.004451485
CEP152	1.336196248	4.79E-14	1.36E-12
AF111169.3	1.471705405	2.78E-07	1.25E-06
NPIPB11	1.313658875	6.52E-09	4.38E-08
AL353807.2	2.019399211	1.66E-08	1.01E-07
BX539320.1	1.008712768	0.002956423	0.00512586
SSC5D	-1.126152625	0.010595072	0.016245046
LINC01285	2.049321651	1.76E-07	8.25E-07
BANF1P2	1.186532727	4.18E-05	0.000110517
ZNF165	1.028857746	2.18E-11	2.87E-10
RN7SL350P	1.275047825	0.00169574	0.003101388
ABHD11	1.56782182	6.20E-13	1.25E-11
MIR4804	1.126656598	0.016030207	0.023608445
CSTL1	2.603996432	7.06E-07	2.86E-06
U73169.1	1.265055069	5.75E-05	0.000146873
RPL17P15	1.164440378	0.000272456	0.000595615
IMMP1LP3	1.69914628	7.23E-06	2.28E-05
PCMTD1P3	2.806439387	8.79E-10	7.49E-09
TM4SF19	1.656973532	1.02E-08	6.53E-08
MNS1	1.260271137	3.60E-07	1.57E-06
KCTD8	-2.156778524	3.11E-09	2.28E-08
AC027607.1	1.190788085	1.78E-06	6.53E-06
KATNAL2	1.127759178	7.11E-05	0.000177803
GNG7	-1.985654293	4.05E-16	2.88E-14
TRAJ3	1.250724012	0.009534331	0.01477253
HESX1	1.051352053	3.40E-08	1.91E-07
AC024560.3	1.764540862	2.18E-11	2.87E-10
AC124067.4	3.243036236	2.60E-09	1.94E-08
AC048341.1	1.18237383	2.77E-08	1.59E-07
AC112484.3	2.317478214	3.85E-07	1.67E-06
RNU6-379P	1.536793916	2.00E-05	5.73E-05
NOL6	1.24761975	1.29E-14	4.55E-13
[illegible]MEM132D-AS	7.201334719	0.000219785	0.000490575
RN7SL615P	1.192754863	0.003727054	0.006323791
ZNF443	1.066968718	1.02E-12	1.94E-11
RNU6-343P	1.352683065	0.000127548	0.00030056
AL353807.4	1.931672196	1.73E-11	2.34E-10
AKR1E2	1.038141774	0.030195316	0.041695014
SND1-IT1	1.123817059	2.48E-05	6.93E-05
LINC00524	3.481807017	6.07E-05	0.000154203
AL033384.2	1.180605859	2.26E-06	8.06E-06

AHSA2P	1.042552573	2.23E-08	1.31E-07
DNAAF3	2.492615989	2.02E-11	2.69E-10
YWHAQP6	1.325667753	7.97E-08	4.08E-07
TCEAL2	-2.478378626	1.86E-08	1.12E-07
CCDC146	-1.291608255	0.01073893	0.016441962
APMAP	1.23885639	2.44E-16	1.95E-14
FBXO45	1.034346366	3.11E-14	9.48E-13
MIR1288	1.291891384	0.019232556	0.027803666
MIR5192	1.458156173	0.004071363	0.006849104
ALG1L2	1.365593777	2.36E-08	1.38E-07
HNRNPA1P10	1.216113307	9.91E-12	1.43E-10
RPL23AP20	3.131255937	1.56E-10	1.64E-09
ODF2-AS1	1.336542693	2.01E-06	7.27E-06
CDC42-IT1	1.245821534	1.97E-06	7.14E-06
CCR10	-1.186134117	3.40E-06	1.16E-05
AC009094.1	2.211620819	1.33E-07	6.43E-07
IGHV3OR16-11	-2.767794103	0.004508374	0.007497634
CBX7	-1.469079391	8.62E-09	5.64E-08
SNX25P1	2.049662287	5.99E-13	1.21E-11
AC087879.1	1.479349578	1.63E-05	4.78E-05
ANAPC1	1.129136691	3.91E-14	1.15E-12
DDN	1.882230398	0.002308559	0.00409816
AC006504.3	1.440745823	0.000159361	0.000367719
MIR4644	1.343783988	0.00221139	0.003942441
CPT1B	1.42655777	8.04E-10	6.93E-09
HNRNPA1P21	2.545408171	2.24E-17	4.66E-15
VTRNA2-1	-1.445103949	0.001939415	0.003496621
AL034349.1	2.335332854	6.40E-09	4.30E-08
AC104066.2	1.202944998	2.10E-06	7.58E-06
AC093620.1	1.1663135	2.89E-06	1.01E-05
AL353689.1	1.969396918	0.00010996	0.000262882
HK2-DT	1.928971091	2.24E-13	5.14E-12
H2BC18	1.574105552	4.40E-09	3.07E-08
AC130456.7	1.420144356	0.001480879	0.002740784
AC090772.3	1.020691227	0.001960345	0.003531033
HNRNPA1P12	1.021844074	3.60E-09	2.58E-08
MTATP6P11	1.007580149	0.001509273	0.002788299
AP002453.1	1.51667704	0.001617459	0.002969429
FIBCD1	3.74813918	6.31E-06	2.02E-05
RPS26P52	1.109640412	0.000787203	0.00155215
CPZ	1.296077464	0.000960776	0.001863361

AL136980.1	1.397219846	8.22E-05	0.000202522
PPIAP55	1.407108164	3.22E-09	2.34E-08
AC010967.1	3.14361926	9.89E-07	3.86E-06
AC092718.3	1.48717638	7.88E-13	1.53E-11
RN7SL280P	1.182978044	0.00156236	0.00287839
LINC02254	3.645558537	9.41E-05	0.000228745
DNM1	1.043991398	0.000574341	0.001166458
NPR3	-1.0312183	0.000150039	0.000348139
AC073592.2	2.747733102	2.80E-07	1.25E-06
AC237221.1	2.336426195	0.028663874	0.039809359
HMGN2P28	1.482730268	2.03E-08	1.20E-07
AC000061.1	3.803946096	2.52E-05	7.03E-05
ANKIB1	1.225044616	1.83E-13	4.32E-12
LARS2-AS1	1.649536116	1.49E-06	5.55E-06
CRYM-AS1	1.421259134	4.39E-11	5.29E-10
NAALADL2-AS	2.919867783	6.80E-05	0.000170931
AP003071.4	-1.44978598	0.017072956	0.024982644
RNU6-126P	2.451274297	1.79E-08	1.08E-07
AP1S3	1.197122048	7.79E-09	5.14E-08
FAAP24	1.207588428	2.98E-12	4.95E-11
SNORA26	1.308934851	3.03E-08	1.72E-07
PITPNM3	-1.748713118	7.64E-06	2.40E-05
AC009102.2	-1.321540394	0.001185232	0.002245712
LINC01337	1.57734388	3.62E-05	9.72E-05
PLAC8L1	1.071992496	2.78E-06	9.73E-06
AL590639.1	1.073856548	6.82E-06	2.17E-05
TM4SF1-AS1	1.685487906	4.77E-05	0.000124217
AL158151.2	1.431589257	0.01146464	0.017442804
IGKV2-28	-3.333394914	0.012216494	0.018472776
CXCL12	-1.208568066	1.03E-07	5.15E-07
RPL37A-DT	1.040258024	7.69E-05	0.000190737
HBA1	-2.112333187	2.76E-12	4.62E-11
OR7E128P	1.938883124	1.72E-11	2.33E-10
LINC02532	4.425227076	1.59E-06	5.91E-06
AC019206.1	2.047674784	7.30E-08	3.77E-07
HMGB3P6	1.867318082	1.65E-09	1.29E-08
TENT5B	-2.33635924	0.000655746	0.001314651
PRUNE2	-1.616048015	2.43E-05	6.81E-05
PRECSIT	1.720037425	3.25E-09	2.36E-08
AL049844.2	1.112387493	7.20E-07	2.91E-06
FGL1	5.988498883	0.003341937	0.005730571

CD46	1.026641015	3.21E-13	7.04E-12
SH3BGR	-2.132751652	8.53E-07	3.39E-06
KRTAP5-4	2.339752742	2.14E-08	1.26E-07
CCDC34	1.512855558	9.68E-13	1.84E-11
PRKAR1B-AS2	1.674612504	0.005640465	0.009180357
ITGA6	1.16270035	6.77E-09	4.53E-08
AC005839.1	1.168738768	3.03E-11	3.82E-10
ALPP	7.431848755	5.95E-14	1.64E-12
FOXL2	2.550422869	0.003504126	0.005975608
GAL3ST2	3.865512258	1.07E-11	1.53E-10
PGR	-1.748589002	5.99E-06	1.93E-05
RPL21P123	1.091514144	7.59E-05	0.000188659
LCN12	2.619579159	1.88E-08	1.13E-07
RNU6-25P	1.154049933	0.008843646	0.013798678
ADAMTS1	-1.682073549	1.21E-06	4.62E-06
EXOC6	1.006982608	1.81E-13	4.28E-12
ADAMTS7	1.378938281	5.92E-10	5.32E-09
FO393419.2	1.086998811	0.001982384	0.003566793
AC008957.2	1.695375298	2.91E-05	7.99E-05
AC138207.1	1.12404569	0.001041685	0.00200264
LOXL2	1.556236931	1.46E-12	2.63E-11
RNVU1-6	1.906396265	5.99E-06	1.93E-05
DOCK5	1.130420401	1.16E-09	9.50E-09
ARHGAP20	-1.009601352	9.10E-06	2.81E-05
EPYC	4.693207981	2.96E-07	1.32E-06
ADH1B	-2.146135765	1.59E-13	3.83E-12
DLEU2	1.912637429	3.85E-17	6.26E-15
LINC02014	1.595339535	1.69E-08	1.03E-07
CENPL	1.626389033	1.98E-18	1.46E-15
MAGI1-IT1	1.138186483	0.00011675	0.000277398
LINC01409	1.559714351	1.34E-11	1.86E-10
AHSG	5.065104258	0.000553332	0.001128866
KRT18P51	1.14398868	2.45E-05	6.87E-05
LOX	1.821629332	3.52E-11	4.34E-10
HMGA1	1.165529518	3.00E-08	1.71E-07
HOXC-AS1	4.063244808	1.24E-15	6.88E-14
CDCA5	2.174942375	1.27E-15	7.02E-14
TMSB10P2	1.283513062	0.000221233	0.000493477
AC069528.2	1.093858914	6.13E-07	2.53E-06
LINC01235	3.1203519	1.86E-14	6.22E-13
AL139288.1	1.314833761	3.00E-07	1.33E-06

AC120349.2	1.426142043	0.001024365	0.001972615
SHOX2	2.514177684	1.38E-08	8.57E-08
SLITRK3	-2.785715424	8.20E-12	1.22E-10
KRT18P17	1.09316936	1.06E-06	4.10E-06
ICAM1	1.301246592	1.96E-07	9.08E-07
FKBP10	2.408094642	1.09E-09	9.01E-09
FRGCA	2.921498988	5.32E-05	0.000137124
AL714022.1	1.2248732	1.29E-05	3.85E-05
AL031320.2	1.161739069	2.41E-07	1.09E-06
CYMP-AS1	2.216620351	0.003452139	0.005896013
VN1R84P	1.152842276	0.035752747	0.048541237
STX1A	1.13218416	1.91E-05	5.50E-05
AL645998.1	1.652757455	1.12E-06	4.31E-06
AL162578.1	1.057425798	0.000185628	0.000421673
MFAP4	-1.427346934	4.05E-10	3.79E-09
PTOV1-AS2	1.156701723	9.51E-10	7.99E-09
AC009533.2	1.664190466	2.37E-05	6.66E-05
ZNF32-AS1	1.148107837	3.02E-06	1.05E-05
AL133367.1	1.328651977	2.28E-09	1.73E-08
MIR544B	2.713543612	8.93E-06	2.77E-05
HSPE1P27	1.140624963	0.000781648	0.001542681
Z69666.1	1.40115131	1.85E-07	8.62E-07
FAM135A-AS1	1.531094326	2.77E-06	9.70E-06
RANBP17	1.806462308	2.22E-06	7.96E-06
Z97633.1	1.26134391	1.25E-09	1.02E-08
CGB5	7.940121123	5.21E-07	2.19E-06
AJUBA	1.410793661	4.83E-11	5.77E-10
KIF22	1.204663003	1.00E-13	2.57E-12
RN7SKP243	2.038571032	2.00E-07	9.25E-07
PRR5L	1.494173355	6.41E-10	5.69E-09
TUBB8P1	1.842454983	4.14E-09	2.92E-08
RNU6-87P	1.650154013	0.000343957	0.000734879
AC025034.1	1.389679957	5.11E-08	2.74E-07
CLPB	1.022617965	9.24E-13	1.77E-11
AC012640.5	1.217794206	0.001959659	0.003530036
RN7SL834P	1.35920328	9.57E-08	4.80E-07
AL009178.2	1.643577307	4.74E-09	3.28E-08
AC100827.5	1.064718931	0.000628678	0.001265335
SEC13P1	1.232569704	1.30E-05	3.90E-05
TGFB1I1	-1.507125647	0.003390199	0.005798773
RPL23AP69	1.45832151	1.77E-05	5.14E-05

MAPT	-1.576412162	0.0001463	0.000340412
WDR72	3.76127831	1.95E-10	1.98E-09
MIR3680-2	1.699396663	5.68E-05	0.000145289
SLC35G5	1.211319745	0.001982747	0.003566793
IGKV1OR2-108	-3.598727995	0.024716894	0.034865829
AC113143.1	1.872451753	4.13E-08	2.27E-07
AC004839.2	1.014554585	0.000178882	0.000408169
MIR3682	1.237883156	1.23E-05	3.70E-05
RPL23AP92	2.018382687	6.92E-07	2.81E-06
CDIPTOSP	2.53822417	3.31E-06	1.14E-05
CFAP92	1.415881352	3.74E-05	0.000100085
AP001893.1	1.265314454	5.06E-08	2.72E-07
METTL7A	-1.261610597	3.22E-14	9.76E-13
AL451050.2	2.021263334	2.72E-14	8.52E-13
AL136162.1	1.186140997	3.94E-05	0.000104818
MTND4P7	1.868586847	0.002909126	0.005052144
ZNF630-AS1	1.217289921	0.00021374	0.000478446
EML4	1.268685245	3.03E-15	1.40E-13
ANKRD61	1.849104575	8.51E-15	3.21E-13
AC002553.1	1.209211789	5.30E-07	2.22E-06
AL592494.2	1.099254438	0.015124069	0.022396501
SPATA32	1.502261251	2.27E-10	2.28E-09
SH2D5	3.202037228	1.97E-09	1.52E-08
ZNF69	1.224328686	5.34E-08	2.85E-07
HOXA11	5.213917884	1.87E-14	6.25E-13
H4C2	1.535731444	1.36E-07	6.55E-07
AL513174.1	1.776320216	1.59E-05	4.66E-05
PACC1	1.402518251	4.58E-17	6.90E-15
AC011379.2	1.068467095	0.003919789	0.006617936
RN7SL168P	1.129891619	0.003608241	0.006135483
KLF2P1	7.916624539	2.90E-11	3.69E-10
SHTN1	1.09734122	1.10E-10	1.20E-09
AL512326.2	1.005015381	0.006753242	0.010815742
AL163192.1	1.439077388	6.82E-05	0.000171286
TMPOP2	2.180162549	6.40E-11	7.40E-10
GORAB-AS1	2.308775388	1.14E-13	2.88E-12
EIF5P1	1.028418018	0.000254257	0.000559275
AC022126.1	2.588175737	3.02E-09	2.21E-08
AC008635.1	1.191933672	4.37E-06	1.46E-05
AC087752.2	1.53768173	1.39E-06	5.23E-06
CIDEA	-3.333106163	1.60E-11	2.18E-10

AC099489.2	1.250262322	0.000807103	0.001588097
AP003555.1	2.779895781	7.53E-13	1.47E-11
CGNL1	-1.597162499	1.34E-07	6.45E-07
CYCSP4	1.497715641	4.26E-05	0.000112211
[illegible]ABGAP1L-AS	1.862669576	5.67E-09	3.85E-08
MTCYBP21	-1.596353925	0.017988148	0.026209474
ANOS1	1.865962787	3.13E-09	2.28E-08
AP001178.1	1.882163209	3.64E-06	1.24E-05
AC124312.3	-1.482851124	2.65E-06	9.32E-06
AC100821.2	1.20488294	5.67E-06	1.84E-05
CFAP74	2.149009294	3.94E-06	1.33E-05
AL357874.2	1.75648237	3.98E-07	1.72E-06
AC012400.1	1.323796528	0.010476916	0.016083318
AP001160.1	1.129642036	3.60E-09	2.58E-08
AC019254.1	1.411973473	1.46E-07	6.98E-07
AC027237.3	1.423318362	6.76E-12	1.02E-10
MIR4701	1.868960192	0.000576067	0.001169695
DNMT3B	2.73792006	1.53E-16	1.47E-14
AL513534.1	1.544420679	4.08E-08	2.25E-07
TRIM10	1.419853698	2.85E-08	1.63E-07
BX255925.2	1.224667522	0.000412016	0.000864939
CCKAR	-2.167754434	2.38E-06	8.45E-06
IGHV3OR16-7	-3.232654056	0.005970611	0.009667499
H2BU1	1.913862468	1.21E-06	4.64E-06
ZNF738	1.263545602	4.16E-09	2.93E-08
SKIL	1.116544685	3.10E-11	3.89E-10
ABHD17C	1.027386083	1.47E-07	7.03E-07
IGFBP3	1.575691285	1.92E-09	1.48E-08
ASPN	1.49830084	4.68E-06	1.55E-05
POT1-AS1	1.448237011	1.89E-07	8.82E-07
AL008723.2	2.293671401	2.94E-08	1.68E-07
RNU6-724P	1.380581939	0.009532638	0.014771625
RNF150	-1.809309455	0.000113438	0.000270106
LINC02373	1.412466704	0.005414148	0.008850937
AL353150.1	1.371683614	0.000669032	0.001338969
GP6	1.146489737	0.00024435	0.000539846
QRSL1	1.685234089	1.05E-09	8.72E-09
AC005086.2	1.269241444	1.47E-06	5.51E-06
LANCL3	-1.146618841	8.53E-07	3.39E-06
AL031770.1	1.193577726	2.17E-05	6.17E-05
FAM86HP	1.072780144	2.10E-07	9.65E-07

MSTO1	1.023522383	1.99E-13	4.64E-12
AL109976.1	1.043044181	1.23E-06	4.69E-06
RN7SL330P	1.619691381	0.000289639	0.000628697
ATXN2-AS	1.283255094	1.34E-09	1.08E-08
PLPPR3	2.085698366	0.001234332	0.002331118
AC068587.2	1.451317202	0.001663752	0.003047521
KHDC4	1.028221621	1.78E-12	3.14E-11
ZNF300P1	-1.040097607	6.31E-05	0.000159623
RNU6-1004P	1.399867084	0.001337219	0.002501136
KRT18P33	1.988697777	2.30E-11	3.01E-10
ANKRD1	4.305162471	8.39E-08	4.27E-07
RNA5SP278	1.33486953	0.010193626	0.015699999
AC005776.2	1.044174889	2.91E-06	1.01E-05
KRT18P52	1.726103055	3.48E-10	3.32E-09
LINC00920	1.899681381	7.50E-09	4.97E-08
CAV1	-1.17518645	0.000107763	0.000258138
AC007849.1	1.687399627	0.000421155	0.000882014
AP006545.2	1.16481881	0.000164328	0.000377969
LEXM	-2.857375779	0.000124036	0.00029295
AP003484.1	1.364196928	0.001706478	0.003118672
AC090571.1	2.826700407	3.43E-08	1.93E-07
AC005736.1	1.355606306	0.001804738	0.003276891
PPIAL4A	1.016183429	1.81E-05	5.23E-05
DDX18P5	1.118741744	7.31E-06	2.30E-05
AC097381.3	1.019672951	0.009068777	0.014124304
AC002401.4	1.520860472	0.017345112	0.025350182
AC114488.1	2.06663863	6.15E-11	7.15E-10
LINC01611	4.822091153	0.000214899	0.000480677
SCRT1	3.146579941	0.001830388	0.003318941
ZNF217	1.293247744	1.70E-12	3.02E-11
MTND5P25	1.662919745	7.21E-07	2.91E-06
PHF5CP	1.870788303	3.69E-09	2.64E-08
OR2AO1P	2.685069022	1.33E-06	5.05E-06
DDX11	1.554930348	5.12E-15	2.13E-13
Z97832.1	1.52028835	6.58E-05	0.000165955
TRMT2B-AS1	1.698028538	1.25E-06	4.77E-06
ABCA17P	2.105128211	5.66E-05	0.000144721
KLF8	-1.679364186	0.000230522	0.000512352
RPL39P38	1.216966401	4.55E-06	1.51E-05
AC106900.1	2.292786509	2.57E-08	1.49E-07
CHAD	-1.907535181	9.64E-06	2.96E-05

SNORA70B	1.010164486	0.00313805	0.005411257
IGHD3-16	-1.945750851	0.017862635	0.026037989
RN7SL219P	1.65344386	4.39E-05	0.000115425
NAP1L4P1	1.736330503	4.70E-11	5.63E-10
PPIAP76	1.710829098	2.20E-06	7.87E-06
MIR7851	1.484760334	0.002032149	0.00364902
AKAP6	-1.878773435	0.000126448	0.000298074
PPIAP65	1.145778146	7.82E-05	0.000193494
ZFP42	4.98899224	9.67E-05	0.000234441
AL591926.6	1.283301241	0.000674418	0.001348532
AC005102.1	2.645072836	1.02E-07	5.09E-07
LINC01644	-1.525517014	0.014848418	0.022028709
AL512652.1	1.660685549	1.28E-07	6.23E-07
H4C4	1.098937957	0.002565996	0.004502322
AC112206.4	2.495290454	0.001170235	0.002220138
AL121933.1	1.489637475	0.000248353	0.000547415
ALPG	6.951456649	3.21E-10	3.09E-09
PTMAP1	2.009461839	3.00E-11	3.79E-10
IFI6	1.739378694	5.07E-08	2.72E-07
ADAMTS9-AS2	-1.92570188	5.58E-08	2.96E-07
PTMAP10	1.014458588	1.77E-07	8.30E-07
AL078645.1	1.086031292	0.006051834	0.009784146
LHFPL3	2.532704986	0.001039324	0.001998822
LINC01978	1.594717848	3.19E-06	1.10E-05
HSPD1P4	1.23708681	3.23E-11	4.03E-10
PPIAP59	1.922802755	1.31E-09	1.06E-08
TMPRSS15	-4.246762528	0.00876656	0.013692032
SPIC	1.365532555	0.034975653	0.047590467
AC005072.1	1.173579796	2.30E-05	6.48E-05
CLDN9	3.185446965	1.14E-07	5.62E-07
GPT	-1.836567347	0.001423565	0.002646474
AC027018.1	1.597928408	3.64E-07	1.58E-06
PON2	1.15519711	1.60E-08	9.73E-08
IRX3	-1.213259869	4.23E-05	0.000111515
SLC7A11	1.128301425	0.000567726	0.001154168
PABPC1P10	1.08581163	1.31E-06	4.97E-06
AL096855.2	2.415662581	7.50E-08	3.87E-07
USP41	1.628327928	0.014537175	0.021603046
LINC01484	1.950694405	6.00E-06	1.93E-05
AC008147.2	1.387262768	2.44E-09	1.83E-08
SNHG15	1.113640104	1.13E-12	2.12E-11

TNFAIP8L3	-1.473340976	8.54E-09	5.59E-08
SOCS5P4	1.20714614	5.36E-08	2.86E-07
SLC28A1	-4.379726371	0.006487211	0.010427916
AC234644.1	1.040992676	0.000881619	0.001721874
CST2	3.778416595	3.81E-12	6.18E-11
IGKV3OR2-5	-4.388005153	1.64E-06	6.07E-06
PRICKLE2	-1.483981394	0.001812322	0.003288869
ATP6V0E1P2	1.189597724	8.78E-06	2.73E-05
ARHGAP26-AS	1.059276552	0.032221151	0.044190049
NCOA7	1.06847655	6.58E-09	4.41E-08
SALL2	-1.251343101	7.95E-05	0.000196366
HOXC13	5.845159556	4.32E-10	4.02E-09
ADAM22	1.239639152	0.000356625	0.000759307
AC104843.2	1.376815691	0.001125764	0.002144934
AGAP5	1.181489412	6.59E-08	3.44E-07
POLE2	1.674049281	9.34E-16	5.58E-14
PABPC1P1	1.147916003	1.88E-09	1.46E-08
SEC61G	1.36762428	1.01E-09	8.43E-09
MAP3K5-AS1	1.226203656	6.33E-07	2.60E-06
PPIAP6	1.254371067	3.03E-10	2.94E-09
AC007435.1	1.275629025	6.55E-05	0.000165129
IPO4	1.405167273	1.63E-17	3.68E-15
AC126696.3	1.550471322	1.40E-05	4.15E-05
ALG13-AS1	1.270249997	3.36E-06	1.15E-05
LINC02652	2.00568463	0.002829311	0.004924446
ARHGEF3-AS1	1.161120558	7.60E-05	0.000188875
DKK1	2.773785869	0.015808124	0.023299645
AD001527.1	1.029907842	0.000149096	0.000346132
TRIB3	2.475762576	1.66E-10	1.73E-09
C5orf58	2.366069694	2.23E-08	1.31E-07
MAOA	-1.238537705	4.01E-10	3.76E-09
AC104971.1	1.048400678	4.48E-05	0.000117376
ELOVL3	2.629293553	7.88E-10	6.82E-09
RNFT2	2.47724673	1.50E-17	3.55E-15
ZNF320	1.0879898	7.43E-10	6.47E-09
MRPL53P1	1.37545598	1.49E-07	7.13E-07
CKS1BP2	2.03400478	2.94E-09	2.16E-08
ACAP2-IT1	1.447474872	1.64E-08	9.98E-08
ENPP7P14	2.254756408	2.80E-07	1.26E-06
AC090236.2	1.086555859	0.000287707	0.000624956
RNU6-100P	1.542451352	4.16E-07	1.79E-06

AC011595.1	1.71254814	6.23E-07	2.56E-06
KLHL35	2.211278531	1.23E-10	1.33E-09
RPSAP58	1.028065419	7.26E-07	2.93E-06
GSN	-1.59527766	7.29E-14	1.95E-12
PRKCG	3.738441504	1.37E-09	1.10E-08
HNRNPCP9	1.491787612	0.001372149	0.002560782
KCNB1	-2.562275663	6.90E-09	4.60E-08
AC005006.1	2.358812304	0.006476622	0.010412151
TMEM200A	1.427878042	1.38E-05	4.11E-05
AIDAP2	1.750511736	3.62E-08	2.02E-07
LINC02777	-1.034143425	4.39E-07	1.88E-06
AC011933.2	1.582369833	1.86E-08	1.12E-07
SNHG1	1.323365678	8.23E-12	1.22E-10
EML1	-1.345864742	0.002381772	0.004211813
DSN1	1.431387747	1.03E-15	5.96E-14
TIGD4	1.261147502	1.13E-08	7.15E-08
MIR3162	1.58663808	0.001021552	0.001968193
AL354941.1	1.644164583	3.06E-05	8.36E-05
KRT18P18	1.530984525	4.13E-09	2.92E-08
PUS1	1.358903116	8.67E-17	1.01E-14
AC022167.4	1.319985257	0.021748498	0.031101636
RNY1P10	2.810816907	1.92E-06	6.98E-06
RN7SKP26	2.18194587	3.00E-05	8.21E-05
RNF148	1.051896616	0.000200998	0.000452432
GHRL	-2.54541629	0.008321168	0.013062253
AC091492.1	1.206859008	2.76E-06	9.66E-06
FAM183A	1.415843494	0.019184904	0.027745316
AC106028.3	1.262350115	3.31E-10	3.17E-09
LRRC56	1.076374195	0.000163845	0.000376893
RCCD1	1.14988911	2.96E-14	9.13E-13
SCARNA4	1.472889259	5.98E-06	1.93E-05
H2BC16P	3.714721737	5.55E-05	0.000142403
ACER2	-1.36339347	0.0233466	0.033136228
CRP	5.72484944	0.000115316	0.00027421
AC005355.2	2.084735052	2.56E-07	1.16E-06
FAXC	-1.540658179	0.000374064	0.000792825
PLEKHS1	2.000360999	4.25E-08	2.32E-07
CCT6A	1.369317328	4.53E-18	1.89E-15
SUSD4	-1.316807907	1.38E-06	5.21E-06
IGKV1OR2-3	-3.601333787	0.000666518	0.001334739
SNORD69	1.314249658	1.72E-07	8.08E-07

XDC2P-NPIPB	1.097215262	4.06E-08	2.24E-07
AC116407.1	-1.841637199	0.001543668	0.002846314
PPIAP72	1.174349253	2.13E-08	1.26E-07
AC011120.1	1.32959402	2.80E-05	7.74E-05
LINC02595	1.929420005	7.34E-11	8.34E-10
AL035090.1	1.691970163	0.000177457	0.000405229
H2AZP3	1.223658999	9.05E-10	7.67E-09
SNORD71	1.635595568	6.16E-06	1.98E-05
ENC1	2.293190968	8.22E-17	9.61E-15
RPSAP47	1.055568617	0.000880913	0.001720747
TUBG1P	1.550922869	2.91E-09	2.14E-08
AC012254.3	1.440614372	1.83E-05	5.29E-05
AC243829.4	1.48369434	0.000106986	0.00025667
AC007546.1	1.58207504	5.43E-08	2.89E-07
AC005993.1	5.334109238	1.06E-08	6.78E-08
AC009065.8	1.076968231	2.79E-10	2.74E-09
RPL10P19	1.372879858	3.49E-08	1.96E-07
LINC02405	2.799176827	1.71E-06	6.31E-06
RNU6-1315P	1.087122628	0.011291285	0.017204599
C5orf46	3.15253301	1.88E-10	1.92E-09
MT1A	-1.569611874	3.16E-11	3.95E-10
WDHD1	1.813465789	5.31E-17	7.48E-15
AL512430.3	1.346096986	6.83E-05	0.000171525
AL354696.1	1.178627	8.60E-07	3.41E-06
AC024267.4	1.516090742	3.92E-07	1.70E-06
IGKV1OR9-2	-3.436736124	0.001669496	0.003056536
ARMC3	3.040712486	4.43E-08	2.42E-07
RPS27AP3	1.384579068	7.81E-08	4.00E-07
KIF15	2.211210176	5.18E-16	3.53E-14
AC007919.1	1.370261787	2.62E-05	7.30E-05
RPS3AP34	1.322771736	0.002482655	0.004371081
AC009121.2	2.847584122	8.48E-13	1.63E-11
AC010615.3	1.528404043	0.004963918	0.0081832
AC108516.2	1.61099514	8.15E-07	3.25E-06
CLPSL1	3.020667846	0.000365547	0.000776874
SERBP1P3	1.086535718	0.000279916	0.000610122
CLDN16	2.116188226	5.06E-09	3.49E-08
AC108112.1	3.743930954	1.14E-07	5.60E-07
AL133243.4	1.263535923	1.57E-06	5.83E-06
AC022558.1	1.2369045	5.23E-08	2.80E-07
HERC2P10	1.30607706	2.55E-06	9.02E-06

ECEL1	2.352109035	1.28E-06	4.86E-06
GRTP1-AS1	1.072619469	1.79E-05	5.18E-05
FIGNL1	1.513008053	1.79E-15	9.15E-14
SMPD5	1.604463383	4.10E-09	2.90E-08
SIX3	4.178019601	1.30E-05	3.88E-05
PRELP	-1.09346094	7.31E-06	2.30E-05
AC015983.1	1.489657542	0.0001946	0.000439666
AC091729.2	2.692416657	7.05E-09	4.69E-08
AC092953.1	1.479066059	4.54E-05	0.000118814
ETV5	1.069488251	3.19E-06	1.10E-05
RPL36P16	1.920823846	9.31E-09	6.05E-08
AL592071.1	1.812325511	5.91E-06	1.91E-05
AL138756.1	1.113004275	2.98E-09	2.19E-08
AL512413.1	2.7503127	9.65E-12	1.40E-10
RNVU1-3	1.027724255	0.000684226	0.001367016
AC008870.3	1.663881437	3.99E-06	1.34E-05
ARPC3P4	1.094244802	1.78E-07	8.33E-07
AC083806.2	1.479252077	2.24E-09	1.70E-08
AC005391.1	2.546501331	3.47E-10	3.31E-09
AC103706.1	1.378625574	2.20E-06	7.88E-06
AC010320.4	1.184320393	0.000102904	0.000247889
AC025423.5	1.888615645	0.001298891	0.002435947
CD274	1.696068043	0.000294194	0.000637748
PHF5GP	1.307332132	0.000961823	0.001865256
AL157402.2	1.009141927	0.033706	0.046020359
PMP2	-2.943554147	4.52E-11	5.44E-10
MIR4676	1.898673907	4.75E-05	0.000123813
H3-3A-DT	1.234351226	4.56E-06	1.51E-05
RN7SL47P	1.372038001	0.003397092	0.005809444
TMEM256P1	1.263655673	0.001625141	0.002982915
KCNH8	2.083997306	7.79E-05	0.000193017
SGO1	2.281114775	1.03E-16	1.12E-14
KLHL6-AS1	1.058488347	0.007786367	0.012300291
AC090192.2	2.826781396	4.40E-07	1.88E-06
RNU6-94P	1.064024095	0.000588774	0.001192317
AC107294.2	1.132786356	0.001333738	0.002495177
CYCSP6	2.676613287	0.000160729	0.000370504
AC134312.5	3.498101305	4.73E-14	1.35E-12
MIR4641	2.135106115	8.22E-06	2.57E-05
MSH5-SAPCD1	1.668270676	2.08E-10	2.10E-09
AL596223.1	1.307123962	5.22E-08	2.79E-07

AC245100.5	1.212680484	0.000425638	0.000890937
AC018529.2	1.062700083	1.39E-08	8.63E-08
GPATCH4	1.310339096	3.00E-18	1.63E-15
PASK	1.178049174	1.76E-11	2.37E-10
RNU6-850P	2.015767112	3.40E-11	4.22E-10
CLDN14	1.772131042	8.26E-08	4.21E-07
ADD3-AS1	1.118396347	0.000310631	0.000669837
TUBBP2	1.16941612	3.75E-08	2.09E-07
LANCL1-AS1	1.144145756	0.000158744	0.000366449
USP2	-2.040541769	3.31E-09	2.39E-08
AC024560.2	1.809175459	1.72E-09	1.35E-08
AC010601.1	1.602392593	0.011378183	0.017325116
LINC02244	1.761026623	1.56E-05	4.59E-05
UNC93B4	1.698338294	1.69E-05	4.94E-05
DCLRE1A	1.187060017	4.52E-14	1.30E-12
IGKV2OR2-1	-4.185427264	0.000816167	0.001604274
RIPK2	1.65081666	2.84E-19	1.26E-15
AC067863.2	1.193037492	0.013746023	0.020525813
HBB	-2.067060865	4.97E-12	7.80E-11
AL512430.2	1.42994174	0.000976226	0.001891123
TSPYL2	-1.373955187	0.007169851	0.011414352
IFITM3	1.032094949	1.13E-07	5.56E-07
SLC6A3	3.966805544	0.00334613	0.005736654
AL390729.1	1.618186515	2.76E-07	1.24E-06
LINC00862	2.114183992	6.82E-09	4.56E-08
MIR194-1	1.336595763	0.001516325	0.002799968
AL512506.1	1.776327093	4.59E-07	1.95E-06
NMB	1.295725964	1.29E-08	8.08E-08
ULBP3	1.500180466	1.27E-06	4.83E-06
RPSAP55	1.121268781	1.64E-06	6.08E-06
FOSB	-2.584227861	0.000170121	0.000389848
POLD2P1	1.673414013	2.73E-06	9.59E-06
ZNF695	3.298752953	5.89E-16	3.85E-14
TRIM60P18	1.210343648	0.029368425	0.040688914
AC026368.1	2.157588168	8.27E-09	5.43E-08
AL512506.2	1.106382291	0.000157163	0.000363089
LINC02367	1.207253105	0.000156799	0.000362279
LRATD2	1.412804018	9.99E-10	8.36E-09
IL9RP3	1.068220428	1.87E-06	6.81E-06
MIEN1	2.00689858	0.005628144	0.009161423
HNRNPA1P26	1.760090232	3.15E-07	1.39E-06

ONECUT2	1.732621199	1.28E-09	1.03E-08
MIR3145	1.15402669	0.000475412	0.000985368
NUAK1	1.153960734	2.13E-09	1.63E-08
DCST2	1.139875031	4.83E-06	1.59E-05
PLEKHO1	-1.169604892	0.00693792	0.011082867
AC096949.1	1.561412836	0.001269352	0.002389397
MYRIP	-1.470621848	5.41E-10	4.91E-09
INTS13	1.108322527	1.78E-12	3.14E-11
NPR1	-1.403978613	4.00E-06	1.35E-05
PPIAP64	1.284914879	3.94E-07	1.71E-06
LINC01967	4.335457139	2.35E-06	8.34E-06
AC091053.2	1.878631715	7.98E-10	6.89E-09
AC126120.1	1.131888932	0.000673327	0.001346452
ZNF730	1.50635201	0.027658846	0.038552212
STK31	3.829247486	3.38E-14	1.02E-12
CXCL8	3.524712247	4.08E-11	4.97E-10
DNAH5	2.046934854	1.28E-07	6.22E-07
IGLV8OR8-1	-2.954785421	0.003554436	0.0060552
ELOCP33	1.144998863	0.000433864	0.000906233
LRGUK	1.982810922	8.62E-10	7.36E-09
AC008514.1	3.224230415	1.21E-05	3.63E-05
COX6CP1	1.198671756	2.20E-06	7.88E-06
RNU6-884P	1.201289504	0.015322249	0.022659748
AC008379.1	1.248970398	4.03E-06	1.35E-05
AL353583.1	1.346576331	3.94E-07	1.71E-06
RNU2-22P	1.518516192	0.000456196	0.000949455
AL138807.1	1.815793873	3.15E-06	1.09E-05
ACTA2	-1.499635925	0.000130535	0.000306947
KLHL11	1.108316612	2.72E-08	1.56E-07
CDCA3	1.128992429	5.93E-08	3.13E-07
GJB4	2.330446422	5.76E-08	3.04E-07
IGKV1D-12	-3.049920311	0.017135801	0.025070401
LSR	1.001864905	1.14E-06	4.37E-06
COX7A1	-1.654057306	3.75E-08	2.09E-07
HNRNPA1P16	1.156551972	5.41E-10	4.91E-09
AC087465.1	1.468727887	0.000544464	0.001112734
SDR9C7	-2.911798405	0.011712981	0.017779013
PSAT1	1.56559796	1.15E-07	5.64E-07
RRM2	1.700717088	7.79E-13	1.52E-11
C1QL1	-1.944907396	3.13E-13	6.90E-12
GDF10	-1.297842271	1.19E-06	4.55E-06

H2AC12	1.277770925	5.82E-06	1.88E-05
IL23A	1.213146158	1.53E-10	1.61E-09
AC044781.1	1.556177011	0.000383044	0.000809999
MAPK4	-2.148807136	5.52E-08	2.93E-07
ADAMTSL3	-1.456235093	1.34E-05	4.00E-05
GLIS3-AS1	2.061562325	0.000145215	0.000338095
FENDRR	-1.292864071	3.72E-07	1.61E-06
RNU6-46P	1.62490841	0.000169164	0.000387854
ZNF587B	1.256783507	2.30E-15	1.13E-13
RNF32-AS1	2.791235232	5.06E-13	1.05E-11
AC012085.2	-1.728143498	0.000304085	0.000656945
NEMP1	1.295891586	1.47E-13	3.55E-12
AP000721.2	-2.35457878	0.000719633	0.001430781
AGT	2.348766356	2.01E-06	7.27E-06
HAL	1.844715375	0.000947457	0.001839807
CR936218.1	1.085719642	1.02E-05	3.12E-05
PTPRG-AS1	1.212476776	0.014219532	0.021180631
FCER1G	1.148739456	6.99E-06	2.22E-05
IGSF10	-1.062584214	2.57E-06	9.08E-06
ASS1P2	1.312292874	1.21E-08	7.64E-08
KRT18P35	1.857708541	1.82E-09	1.41E-08
SPATA13	1.234110303	1.44E-12	2.61E-11
LY6H	-1.855823915	5.44E-08	2.89E-07
AL109935.1	1.006252729	0.005859238	0.009507389
MPP7	-1.063133776	0.000219449	0.000489867
AC083795.1	1.029697095	0.023954739	0.033907245
AL592301.1	1.641305863	7.23E-09	4.80E-08
INTS4P1	1.489847575	7.49E-05	0.000186464
UBE2V1P1	1.070207899	4.90E-06	1.61E-05
AC008760.1	1.010906341	2.26E-06	8.06E-06
NEFM	-2.401851319	1.18E-06	4.53E-06
OSTCP3	1.529280283	0.000814885	0.001601872
CTBP1-AS	1.016218607	1.15E-07	5.64E-07
NME1-NME2	1.088531546	4.48E-06	1.49E-05
IGHV2OR16-5	-2.403663856	0.003124678	0.005390291
CLDN5	-1.153977329	1.42E-10	1.51E-09
RN7SL279P	1.35317213	2.27E-05	6.41E-05
TOP2A	2.862726353	2.69E-17	5.18E-15
AL355796.1	3.518847237	2.28E-07	1.04E-06
NEGR1	-2.00324582	5.11E-09	3.51E-08
MCRIP2P1	2.25795376	4.17E-08	2.29E-07

RN7SL8P	1.506000549	0.003030842	0.005244038
PPIAP31	1.167401873	1.63E-09	1.28E-08
MIR331	1.034197338	7.86E-05	0.000194512
IGKV1-33	-3.461033444	0.004138222	0.00694895
IGKV2-30	-2.565032982	0.028907887	0.040118974
CKS1BP7	1.632427227	4.39E-09	3.07E-08
LGI4	-1.931551498	2.79E-10	2.74E-09
AC010201.1	1.121366798	5.57E-05	0.000142633
PARP9	1.090703771	1.14E-11	1.62E-10
DIAPH3	1.706709104	1.92E-13	4.50E-12
AC010627.1	2.216699848	5.79E-05	0.000147685
IGHV3-72	-2.103556554	0.018659873	0.027071397
AC090695.1	1.572363291	5.80E-09	3.94E-08
IKBIP	1.120997352	4.63E-14	1.33E-12
AC138832.1	1.331999464	0.000402086	0.000846426
SNORD116-1	-1.46949607	0.007746002	0.012244511
RNU6-711P	1.783223107	8.91E-06	2.76E-05
AL136231.1	1.69656923	7.90E-06	2.48E-05
ARC	-3.30347918	1.19E-06	4.56E-06
CKS1BP3	1.912773657	1.33E-10	1.43E-09
CHAF1A	1.307660459	2.62E-14	8.25E-13
IQCH	1.379390247	1.65E-07	7.81E-07
RAD51AP1P1	1.94878709	1.61E-05	4.71E-05
H2BC15	1.371106035	2.61E-09	1.94E-08
AC112694.2	1.50457681	1.26E-09	1.02E-08
AC091117.2	1.178870099	0.001798194	0.003267013
RNU7-12P	1.160119699	0.000726344	0.001443369
MICU3	-1.234329264	4.68E-05	0.000122048
EBLN2	1.044315515	2.65E-08	1.53E-07
MTND4P6	2.016055916	0.00082624	0.001622637
IGHV3OR16-13	-3.152476877	0.027429723	0.038284926
AC138409.2	1.135414775	5.16E-09	3.54E-08
SNORD83A	1.095701897	9.42E-05	0.000228912
AC008543.1	1.201570765	3.31E-09	2.39E-08
PCDH10	-1.554620769	0.000418696	0.000877573
AC006023.1	1.060528853	0.001796723	0.003265008
ATAD2	2.351654018	9.99E-19	1.27E-15
TUB	-1.065859931	5.07E-05	0.000131186
AC092803.1	1.111596211	1.03E-05	3.14E-05
AC100793.4	-1.245421668	0.000139355	0.000325561
SLCO4A1-AS1	2.97322509	4.56E-08	2.48E-07

AC108676.1	3.03927078	8.85E-11	9.84E-10
SH3TC2	1.187004493	1.60E-07	7.58E-07
FAM201A	1.280200113	0.000520331	0.001068168
MYO1B	1.62379386	1.45E-16	1.40E-14
MIR1296	1.057287466	0.005880284	0.00953515
AL136418.1	2.688754748	4.61E-07	1.96E-06
ASS1	1.390943211	7.47E-06	2.35E-05
SCAT2	2.276259672	1.17E-13	2.94E-12
PPIAP91	1.807962763	3.99E-07	1.72E-06
ARHGEF2-AS1	1.604359162	0.001856596	0.003361886
PARP14	1.650870326	2.01E-16	1.73E-14
KIF23	2.504138852	7.94E-18	2.58E-15
SRPK1	1.144103498	1.19E-14	4.21E-13
AC018665.1	1.300587813	2.27E-09	1.72E-08
AC008429.4	1.053420003	0.000422088	0.000883784
AC023813.3	1.515064284	1.03E-05	3.15E-05
RPL6P2	1.195960917	1.32E-05	3.94E-05
AL031666.3	1.447305882	6.74E-05	0.000169473
AC010326.4	1.29000429	9.08E-12	1.33E-10
SPATA3-AS1	2.722083932	1.77E-11	2.38E-10
TFAP2A-AS1	1.610381685	4.28E-07	1.84E-06
SFRP5	-1.537270673	1.03E-07	5.15E-07
STYXL2	6.932062701	0.002143061	0.003830879
AC124916.2	1.327817337	0.001747949	0.003186592
AC006529.1	1.821714685	7.31E-10	6.39E-09
MYL6	-1.067644374	3.78E-09	2.69E-08
ISG15	1.298569326	4.09E-06	1.37E-05
AC008870.2	1.422965682	4.70E-11	5.63E-10
AC114755.1	1.339873868	0.002189198	0.003905492
TYMSOS	1.159761294	6.74E-06	2.14E-05
AOC4P	-1.123100638	0.020501908	0.029462853
RNY3P13	1.060328087	0.005500809	0.008973888
CSF2RA	1.051736569	2.44E-05	6.85E-05
MMP3	5.098421144	7.29E-10	6.38E-09
TCEAL7	-1.470272399	3.38E-05	9.13E-05
ISM2	2.301932091	0.035705171	0.048489874
KYNU	1.010777592	0.001169607	0.002219104
AC067930.4	2.27750169	4.95E-10	4.54E-09
AL359837.1	1.281533646	1.57E-05	4.62E-05
AL161669.3	1.961212951	0.010586072	0.016234048
SFTPA2	2.2914539	0.016286746	0.023933342

KRR1P1	1.388053373	1.22E-07	5.95E-07
AC097493.1	1.671463342	0.000531666	0.00108917
LINC01474	1.710400343	0.00140149	0.002609988
AC245884.12	1.982162529	8.42E-05	0.000207031
AC007598.1	1.046622052	0.013013711	0.019540737
AC008083.2	2.454425392	1.11E-06	4.28E-06
MUC16	2.84390398	8.26E-05	0.000203454
AC026771.1	1.241783958	1.19E-05	3.58E-05
RPL29P25	1.059967994	0.002246517	0.003998377
RNU6-882P	1.177069559	0.002094006	0.00374948
ASAP1-IT2	1.444179878	9.74E-08	4.88E-07
H4C13	1.422543617	0.000313169	0.000674927
AC144831.1	-1.16625741	1.72E-05	5.01E-05
PPP1R26-AS1	1.634081784	6.95E-14	1.87E-12
RCN1P2	1.551724503	9.38E-12	1.36E-10
AC015921.1	1.105936963	0.000196324	0.000442722
SNRPN	-1.302853393	2.33E-10	2.33E-09
RNU6-834P	1.64992343	4.05E-06	1.36E-05
STK32B	-1.028546063	3.79E-06	1.28E-05
AL121906.2	1.086778639	3.22E-05	8.75E-05
PPIAP8	1.167904112	3.92E-05	0.00010448
RPL23AP50	1.124335993	4.34E-05	0.000114052
AL445223.1	1.719403542	9.71E-07	3.80E-06
AL157871.3	1.371195864	3.08E-07	1.36E-06
TMEM108	-1.276213263	8.63E-08	4.38E-07
AC006946.1	1.869948035	3.74E-08	2.09E-07
STRA8	4.151957603	0.00649274	0.010434914
AC245100.2	1.555130916	1.76E-10	1.81E-09
MRGBP	1.344834223	4.75E-15	2.01E-13
AP002907.1	1.453026277	2.01E-09	1.55E-08
AC141557.2	2.834667456	7.15E-13	1.41E-11
LY6G5B	1.026239016	1.00E-06	3.90E-06
CHRM4	-1.217684708	2.34E-05	6.60E-05
PMEPA1	2.440783748	3.68E-14	1.10E-12
AC012360.1	1.021829265	0.003056012	0.005282784
PPIAP73	1.614578118	1.24E-07	6.06E-07
MRAP-AS1	1.223868534	3.27E-05	8.89E-05
SNRPA1	1.087780744	1.85E-15	9.43E-14
BNIP3P26	1.033930718	0.002297346	0.004079849
ENPP7P7	1.21110146	0.000740393	0.001468768
SHISA3	-1.001320886	7.20E-06	2.27E-05

MTND5P28	2.284810149	4.11E-12	6.61E-11
Z98884.2	1.515271015	8.89E-12	1.30E-10
NCLP1	1.603357902	2.91E-11	3.69E-10
RN7SL706P	2.787984604	1.29E-06	4.90E-06
AC092171.5	1.185513063	9.02E-07	3.56E-06
CENPJ	1.076973714	1.92E-10	1.95E-09
RNU6-82P	1.050141305	0.022201081	0.031691019
LINC00649	1.032776592	1.07E-06	4.16E-06
CENPM	1.102518822	5.34E-07	2.23E-06
GUCA2B	-4.021390807	0.000239212	0.00052959
GPRASP1	-1.391262306	0.000698361	0.001391916
LDC1P	2.360680167	8.48E-06	2.64E-05
Z82173.1	1.005931793	0.006410606	0.010313489
KCNC3	1.802467455	1.17E-10	1.27E-09
CHCHD2P11	1.102219733	0.000315586	0.000679752
RN7SL19P	1.489821614	1.99E-06	7.19E-06
CRHBP	-1.437738673	2.25E-05	6.36E-05
ADH1C	-1.522639238	0.002272762	0.004041308
RN7SL449P	1.280553193	0.000178379	0.000407194
RNA5SP395	1.694559345	1.76E-06	6.46E-06
VIP	-2.667953752	2.63E-05	7.31E-05
YTHDF2P1	1.364925844	8.82E-08	4.46E-07
GOLGA8B	1.131600735	7.06E-05	0.000176737
AC002546.1	-1.714127043	1.82E-05	5.26E-05
RNU6-748P	2.679688182	1.34E-06	5.06E-06
LINC00857	1.168205233	7.44E-07	2.99E-06
AC005790.1	-1.30291539	2.32E-05	6.55E-05
DBR1	1.066955713	6.16E-10	5.50E-09
LINC02195	2.240835315	2.11E-08	1.25E-07
AC129507.2	1.031687803	0.000470933	0.000976921
PDGFRB	1.581836719	5.96E-11	6.94E-10
SP6	1.077499264	0.000936925	0.001821214
RPL23AP48	1.105004163	1.65E-05	4.82E-05
TMSB10P1	1.187460546	1.81E-05	5.23E-05
ACYP1	1.275404533	1.12E-12	2.10E-11
TFAP2A	1.025522842	3.57E-07	1.56E-06
AC004009.3	2.183252461	0.000475574	0.000985628
ANKRD10-IT1	1.864321011	2.59E-14	8.17E-13
TREH	-3.537178453	0.000104352	0.000250804
CBX3P9	1.297967079	4.93E-11	5.86E-10
MTND3P5	3.347629133	1.11E-07	5.47E-07

MIR6719	1.458062762	0.002251538	0.004006777
AC079447.1	1.152835241	4.04E-11	4.93E-10
AC032044.2	1.019756026	0.000212031	0.00047482
BICC1	1.126520712	0.000131367	0.000308576
MMS22L	1.758629739	3.80E-17	6.26E-15
BOLA2P2	1.126084669	5.85E-06	1.89E-05
EIF4A1P6	1.18202452	3.84E-05	0.000102538
TBL1XR1	1.118177438	2.24E-15	1.10E-13
AC003965.2	2.718015017	5.33E-11	6.27E-10
RNU6-388P	1.466527723	4.20E-05	0.000110863
IGHV3-16	-3.301380776	0.004709755	0.007802344
AC034229.4	1.630378479	1.53E-06	5.70E-06
RNU6-762P	2.244258102	4.73E-10	4.36E-09
SLAMF8	1.5730481	1.97E-08	1.17E-07
TMEM81	1.036385904	4.09E-13	8.65E-12
IGBP1-AS2	1.27229326	2.08E-06	7.51E-06
CHKB-CPT1B	1.058489487	6.21E-06	1.99E-05
MXRA7	-1.180896047	0.014065666	0.020959644
AL022316.1	2.46573595	1.58E-09	1.25E-08
ZNF468	1.382526852	1.44E-12	2.61E-11
AC026740.1	1.99918059	1.30E-09	1.05E-08
AC011933.4	1.581525856	4.59E-05	0.000120074
PFN1P3	1.445035302	4.90E-07	2.07E-06
RNU6-199P	1.831536955	3.61E-08	2.02E-07
AC116917.1	-1.566076827	0.031216449	0.042935912
AL604028.2	1.226144136	7.36E-10	6.43E-09
HMGA1P2	1.213066281	3.59E-10	3.40E-09
PDGFB	1.132810693	2.13E-09	1.63E-08
SRCIN1	2.181768033	1.26E-06	4.79E-06
AL021807.1	1.866121352	1.27E-05	3.80E-05
DBIP1	1.111616163	0.001769598	0.00322253
MFSD4A	-2.023565702	0.030554862	0.042139074
RTL5	-1.21610342	5.99E-06	1.93E-05
AC027601.1	1.147903688	5.36E-10	4.87E-09
RAB9B	-1.59249028	1.18E-05	3.56E-05
RNU6-1128P	1.846857142	1.74E-05	5.05E-05
RN7SL541P	2.47312208	2.11E-09	1.61E-08
RNU6-1157P	1.701973516	3.71E-08	2.07E-07
AC135050.4	1.453165806	3.29E-07	1.45E-06
SYNGR1	-1.217350852	2.84E-09	2.10E-08
AC116914.2	1.130522882	1.91E-06	6.92E-06

C10orf95	1.236808493	1.57E-08	9.59E-08
RPP40	1.081022544	7.50E-11	8.51E-10
TCEA1P4	1.016739668	1.35E-06	5.10E-06
PRRT4	-2.51376056	4.39E-09	3.07E-08
HOXB7	1.20301152	6.18E-07	2.54E-06
ELOCP29	1.235941346	0.003327778	0.005709232
RN7SL735P	1.68966898	0.00030503	0.000658727
PPP1R12B	-1.431001697	0.001012987	0.001953853
BMS1P4	1.088610356	2.79E-09	2.06E-08
CD70	1.586857448	0.000278575	0.000607299
AL136380.1	1.146246776	8.48E-05	0.000208307
AC108472.1	-1.189088578	0.00025863	0.000568189
TBL1XR1-AS1	1.719289858	1.08E-07	5.36E-07
TMTC4	1.385751472	6.87E-16	4.36E-14
MOV10L1	1.470061169	1.11E-07	5.47E-07
PDK4	-2.429272672	3.55E-10	3.38E-09
AC087632.1	1.727483912	1.73E-05	5.03E-05
YEATS2-AS1	1.479891919	2.58E-11	3.33E-10
RTKN	1.401774551	1.19E-12	2.20E-11
ZNF239	1.669131174	9.81E-14	2.52E-12
PTPN12	1.023790443	4.55E-12	7.21E-11
PPIHP1	1.315298448	3.15E-07	1.39E-06
SLC35E4	1.314809165	6.41E-10	5.69E-09
CKAP2	1.902017067	3.17E-16	2.39E-14
FRAS1	1.499832351	0.001001782	0.00193472
BLM	2.128391706	2.54E-16	2.00E-14
DSG2-AS1	1.326433386	4.63E-06	1.53E-05
RNU4-21P	1.622342285	1.29E-05	3.86E-05
AL390838.2	1.330588814	0.004358444	0.007279648
AC233280.1	1.539964096	1.98E-07	9.18E-07
BHMT2	-1.779035445	2.63E-07	1.18E-06
AL161725.1	1.227106783	1.70E-05	4.95E-05
ITFG1-AS1	1.057685454	3.48E-07	1.53E-06
AC103810.5	1.484304302	0.000209105	0.000468623
RNU6-597P	1.205027393	7.07E-05	0.000176866
RN7SL204P	1.054892584	0.001224568	0.002313333
AP000347.1	1.435821809	1.39E-10	1.48E-09
ZNF471	-1.328238162	2.57E-06	9.08E-06
POLH-AS1	1.341050909	2.47E-10	2.46E-09
AC087620.1	1.435416064	2.42E-05	6.78E-05
AC008750.4	1.145220717	2.66E-06	9.36E-06

MFAP1P1	1.155836541	0.001694306	0.003098977
AC009509.1	-1.40190236	7.79E-05	0.000193017
AC116903.2	1.529289279	2.80E-05	7.74E-05
AC005726.2	2.071562544	4.04E-13	8.57E-12
CD177	4.040387285	0.003785495	0.006411517
RPL12P1	1.136202964	2.90E-07	1.30E-06
AC004134.1	2.133442339	8.35E-10	7.17E-09
RN7SL614P	1.248456565	1.65E-05	4.82E-05
GAPDHP39	2.060750396	5.42E-12	8.44E-11
GUCA1A	1.462957541	0.01270567	0.01912995
DAAM2	-1.539247645	3.21E-06	1.11E-05
RPL31P58	1.652197564	2.10E-07	9.68E-07
PFKFB2	1.147924541	6.86E-07	2.79E-06
IGHVIII-67-2	-3.023340113	0.002239447	0.003986593
RPS21P2	1.585339083	0.000464103	0.000963954
AL117187.1	1.316425934	4.19E-05	0.000110795
AC139720.1	2.350020311	1.94E-06	7.02E-06
DOK5	1.053119886	0.000288899	0.000627239
RNU7-79P	1.441111964	0.003701361	0.006283398
SNAPC4	1.007961147	1.52E-13	3.67E-12
KCNJ16	-2.66413515	0.000591098	0.001196306
IGKV3D-15	-3.38599769	0.016289717	0.023935067
XRN2	1.076816556	3.74E-18	1.72E-15
AL356218.1	1.822423899	0.001101119	0.00210309
AC092755.2	1.127878885	4.67E-07	1.98E-06
AC011479.2	1.666218122	6.50E-09	4.37E-08
FANCD2	1.955520114	2.14E-16	1.81E-14
AC092881.1	1.900675901	7.00E-10	6.17E-09
RPS3P2	1.235503434	3.78E-06	1.28E-05
C4A	1.283184665	0.000215427	0.000481494
AL513218.1	1.359145748	3.81E-09	2.71E-08
LRRIQ4	2.138568874	2.01E-08	1.20E-07
KRT18P25	2.12994457	2.58E-12	4.37E-11
AC007663.2	2.507533121	0.007794302	0.012312095
CCNE1	3.363673093	7.07E-15	2.77E-13
RPL32P33	1.105574866	0.002611392	0.004575947
TPX2	2.574601597	4.29E-17	6.76E-15
PSMC3IP	1.059876221	2.69E-13	6.06E-12
MTND4P14	1.329350095	4.68E-06	1.55E-05
AC012531.1	4.928496009	8.09E-16	4.99E-14
ELL2	-1.138865263	9.70E-10	8.14E-09

NDUFA3P1	1.074405345	0.000585271	0.001185763
AC002128.2	1.799761285	4.85E-11	5.79E-10
ADAM33	-1.499830662	6.03E-07	2.49E-06
PEBP4	-2.871859884	6.21E-13	1.25E-11
MRPL40P1	3.348255954	2.60E-10	2.58E-09
MROH3P	1.168662666	0.000190325	0.000431047
AC068946.1	1.38506453	4.42E-07	1.89E-06
THEM6	1.389754418	7.46E-12	1.11E-10
AC009087.1	1.291644908	2.75E-06	9.63E-06
UHRF1	2.242935023	1.63E-16	1.50E-14
AL353801.3	1.185586748	2.95E-06	1.03E-05
KCNA5	-2.825325996	4.98E-08	2.68E-07
LINC00659	1.97827489	3.31E-08	1.87E-07
AC024588.1	1.882449072	6.42E-07	2.63E-06
ADGRE2	1.453265758	9.49E-08	4.76E-07
PPIAP14	1.175681378	0.000902805	0.00175977
MAMDC4	1.611155626	2.31E-08	1.35E-07
CRYAB	-2.231759634	7.81E-11	8.83E-10
AC022211.2	1.281517749	1.06E-10	1.16E-09
AURKC	1.028949505	0.0004995	0.001030007
ZBTB40-IT1	1.118236717	1.43E-05	4.22E-05
HAND2	-2.299515737	0.000911063	0.001773929
AMPD1	-2.638865398	9.59E-05	0.000232733
TMEM238L	-1.839493145	0.001370519	0.002558033
RN7SL151P	1.649543113	0.01889691	0.02736745
PTH2R	3.316792445	0.005698063	0.009269006
RXRG	-2.881196551	8.09E-14	2.12E-12
PODXL2	1.512069633	1.21E-06	4.62E-06
AL133353.1	1.163520366	0.000290375	0.000630238
AC130456.1	1.935210669	4.87E-05	0.000126678
RNU6-875P	3.133351049	3.97E-09	2.82E-08
LINC02525	3.909986262	0.017032799	0.024934767
RAB5CP1	1.437143513	2.43E-06	8.60E-06
ARHGAP8	2.279104125	2.29E-16	1.87E-14
AACSP1	3.695739709	5.93E-05	0.000151014
PDZK1P1	1.283093899	4.25E-07	1.82E-06
ZNF724	1.798439589	2.68E-10	2.65E-09
AC125611.1	1.251969607	0.001361172	0.002542904
QTRT2	1.291308291	4.29E-19	1.26E-15
PI16	-3.144836293	1.48E-13	3.59E-12
SNHG12	1.190995233	3.16E-11	3.95E-10

RNU4-22P	1.455069865	0.000346388	0.000739521
KLF2P4	2.598655156	0.02975531	0.041174933
LINC02365	4.184060989	4.37E-09	3.06E-08
LBP	4.769498658	7.14E-07	2.89E-06
NNAT	-1.896190467	0.002852739	0.004959392
RNU6-833P	1.809075474	0.001221839	0.002309044
LINC01954	-1.274798602	0.001668692	0.003055484
AC013437.1	1.716286105	0.03570136	0.048489874
CDC6	3.081411606	1.63E-16	1.50E-14
FRMD1	-1.973563267	0.002695314	0.00470814
P3H4	2.034024542	4.10E-14	1.20E-12
FSIP2-AS1	2.341293768	7.22E-07	2.92E-06
EPHA10	1.287721995	4.46E-07	1.90E-06
AC006272.1	1.714867786	3.49E-06	1.19E-05
SAMD9L	1.083286419	0.000523373	0.001073501
SMC4	1.31682664	2.79E-12	4.66E-11
RMI1	1.044778305	5.14E-12	8.03E-11
AL356740.1	1.376461724	0.001383709	0.002581025
KRTAP5-9	1.380255208	0.000652797	0.001309526
GPR19	1.816552946	1.12E-11	1.59E-10
MIR497HG	-1.53265765	2.33E-06	8.28E-06
SDCBPP1	1.619466735	1.24E-05	3.73E-05
SKAP1-AS1	1.748985746	5.55E-07	2.31E-06
RPS29P14	1.095097044	3.43E-05	9.26E-05
AL357134.1	1.526434974	0.000358013	0.0007622
CCL26	2.4714125	0.001664114	0.003047521
MAP3K2-DT	1.099745332	1.11E-06	4.28E-06
AP000437.1	1.026458503	1.03E-05	3.14E-05
AC018809.1	1.340183934	4.85E-06	1.60E-05
IFT80	1.071317503	2.13E-12	3.68E-11
E2F5	1.158725785	1.30E-11	1.81E-10
FGF14-AS2	-1.450088342	1.00E-08	6.43E-08
KCTD5P1	1.246843422	6.83E-06	2.17E-05
AL158827.1	1.028682582	0.009393869	0.014576939
TNXB	-1.800608903	1.23E-09	9.99E-09
AC011753.1	2.259119394	0.000657955	0.001318782
AL365436.2	1.335356453	5.93E-07	2.45E-06
AL442125.2	1.280238336	5.07E-08	2.72E-07
STAT1	1.624185661	9.58E-14	2.47E-12
AL450344.3	1.182539777	5.05E-05	0.000130778
AC009950.1	1.014514022	0.000103017	0.00024793

AP000894.4	1.190875969	0.000485098	0.00100334
MIR8055	1.240078016	0.000282533	0.000614922
FGF19	8.294004292	8.05E-12	1.20E-10
SLC12A5-AS1	2.207267695	3.07E-10	2.98E-09
RNU6-925P	1.950851773	6.35E-11	7.35E-10
AC079466.1	7.054806703	1.76E-07	8.25E-07
AC073073.2	1.09819688	2.58E-11	3.33E-10
FAM96AP2	1.915231443	6.59E-07	2.69E-06
PNMA5	6.725214345	0.013586215	0.020305394
AC004112.1	1.076987316	9.90E-05	0.00023943
AC016027.5	2.008958698	1.79E-08	1.08E-07
CCDC114	1.479256	0.001607148	0.002952737
CSAG1	7.133033615	6.20E-07	2.55E-06
CTNNA3	-2.700378716	0.001385404	0.002583254
KIF20B	1.716323101	1.16E-16	1.22E-14
HMGN2P2	1.533124499	1.97E-05	5.65E-05
PAGE1	5.577329908	4.37E-05	0.00011474
AL731563.1	1.697571278	0.000157843	0.000364407
AC211469.1	1.234425958	0.000486172	0.001005323
AL023803.3	1.693195277	1.99E-06	7.19E-06
GAPDHP25	1.404262263	1.10E-05	3.33E-05
AC074044.1	1.617456867	4.38E-06	1.46E-05
CENPO	1.692486964	7.52E-18	2.50E-15
BRIP1	2.013607094	5.81E-16	3.83E-14
AL117328.1	2.16869628	7.82E-06	2.45E-05
PCP4L1	-2.617161827	1.63E-07	7.70E-07
AC099667.1	1.887219901	8.58E-08	4.36E-07
ZNF26	1.168585213	8.92E-14	2.31E-12
RNU6-1010P	1.039018736	0.00138724	0.002586162
SNX2P1	1.833904952	4.67E-09	3.24E-08
VNN3	1.662478994	0.010405519	0.015982634
SNORA38B	1.26596602	7.74E-05	0.000191934
GRAMD2A	1.487880361	0.000184503	0.000419449
LEF1	1.517158364	8.70E-10	7.42E-09
AC005013.1	1.530986015	0.011101242	0.016945073
RNU4-80P	1.949535705	3.63E-05	9.74E-05
AC012435.3	1.128118217	2.14E-05	6.09E-05
NFE2L3	2.607520007	9.05E-19	1.26E-15
AC009948.1	1.290189571	8.46E-06	2.64E-05
RNA5SP457	1.130378472	0.031698947	0.0435254
FLJ12825	1.979150379	8.52E-06	2.65E-05

MIR924HG	1.556743476	0.000315315	0.000679222
SCNN1G	-2.546970471	7.88E-09	5.19E-08
RIPOR3-AS1	1.4965858	3.29E-05	8.94E-05
EFCAB8	1.605499041	0.0008957	0.00174728
PDE6C	1.270817386	4.92E-07	2.08E-06
RNU6ATAC16F	1.827520942	1.06E-07	5.29E-07
FEZF1	5.458981738	9.34E-15	3.49E-13
AP000346.3	2.223227401	0.000543348	0.001110624
NPIPB13	1.033803115	4.17E-05	0.000110214
RPS20P33	1.536219814	1.02E-10	1.12E-09
AC135352.1	1.46415175	0.029909832	0.041358682
MAP6	-1.666658558	1.32E-06	5.00E-06
TMEM44	1.276945191	8.46E-14	2.21E-12
KIF4A	2.302241887	2.20E-16	1.84E-14
KRT18P7	1.268275491	1.66E-10	1.73E-09
PIWIL4	1.491492859	9.33E-11	1.03E-09
CNTN1	-1.806099924	4.76E-07	2.02E-06
RNU6-547P	1.970877602	3.29E-07	1.45E-06
RPL32P26	1.408671258	3.23E-07	1.42E-06
ETNPPL	-2.967793775	3.45E-05	9.31E-05
AC011815.2	1.298670645	4.61E-07	1.96E-06
ASIP	1.384806655	1.24E-07	6.05E-07
AC018797.2	1.747313128	6.00E-10	5.38E-09
PIMREGP3	1.64292964	3.74E-09	2.67E-08
RAD54B	1.961993935	1.11E-16	1.21E-14
AC005224.2	1.445092187	6.60E-05	0.000166419
SFTPB	3.566401451	0.001103679	0.002107678
AC092964.1	1.685499064	7.09E-09	4.72E-08
AC018845.3	1.239898117	5.36E-06	1.75E-05
LINC00106	1.546454314	6.43E-09	4.32E-08
SEPTIN7P12	1.372371591	1.06E-06	4.10E-06
B3GNTL1	1.348178109	9.63E-17	1.07E-14
ATP1B2	-1.726075506	1.35E-06	5.10E-06
DARS2	1.497555154	2.71E-15	1.29E-13
MIR1276	1.203546741	0.005817551	0.009447226
AC007922.2	1.643192442	2.00E-05	5.73E-05
AC011472.4	-1.665003211	0.001983009	0.003566793
AC068790.3	1.315543026	7.16E-08	3.70E-07
AC096887.2	1.142164918	8.78E-08	4.44E-07
OSMR	1.443990344	6.54E-10	5.79E-09
AC127024.2	1.300594308	9.30E-06	2.87E-05

AC145146.1	2.092947866	4.98E-06	1.64E-05
ARHGAP11A	2.244630829	3.75E-17	6.26E-15
MIR4639	1.087007413	0.036812319	0.049862144
ACTG1P24	1.121748309	2.64E-08	1.52E-07
AEN	1.039485707	1.39E-10	1.48E-09
AC103740.1	-2.019947539	0.002882057	0.005007101
AL136228.1	1.708486055	0.000933224	0.00181455
PDCL3P6	1.319624314	0.000133248	0.000312581
HORMAD1	1.74400156	1.43E-07	6.84E-07
IGFBP6	-2.09482831	3.71E-11	4.56E-10
KDM4A-AS1	1.439495002	4.44E-13	9.28E-12
AP003170.3	1.344231215	1.70E-06	6.24E-06
EEF1E1P1	2.144721199	6.61E-12	1.00E-10
DKK2	1.87848065	3.90E-08	2.16E-07
HOXA9	3.674605389	3.05E-11	3.84E-10
SLC7A8	-1.366489891	2.38E-08	1.39E-07
RANBP1P1	1.241949482	6.41E-11	7.41E-10
MYBPH	3.786483714	0.00036775	0.000781002
AC091390.2	1.390131544	7.86E-09	5.18E-08
RNU2-11P	1.33097604	0.000330861	0.000709586
H2AW	1.227143668	1.94E-05	5.57E-05
DMD	-1.697643142	3.59E-05	9.65E-05
FOXS1	2.913217341	9.51E-17	1.07E-14
PLOD3	1.622657703	4.85E-16	3.35E-14
KCNJ15	-1.780392733	0.000418726	0.000877573
SBK1	1.957566276	0.000832702	0.001633882
IQANK1	1.758079666	2.32E-11	3.03E-10
AL157385.1	1.175028024	0.001863574	0.003373146
VGLL1	3.681980915	0.029678497	0.041083577
LINC00939	2.963777506	3.38E-09	2.44E-08
AL356575.1	1.327087092	0.000407695	0.000857014
AC016559.1	1.703095473	0.001023098	0.00197103
AC138466.5	1.669913523	4.43E-09	3.09E-08
TTK	2.233726109	6.99E-15	2.74E-13
ODF3L1	-1.061626782	0.001144138	0.002176827
AC134349.2	1.2855542	2.02E-05	5.77E-05
IGLCOR22-1	-3.082625541	0.014307401	0.021302634
HGH1	1.403353104	7.71E-15	2.97E-13
AC024896.1	1.061415282	9.01E-08	4.55E-07
RPL7P21	1.07265082	0.000138179	0.000323046
ADIPOQ	-1.515685389	6.20E-10	5.54E-09

AC079316.2	1.278178673	0.001321807	0.002475438
AC009927.1	1.333027909	3.45E-09	2.49E-08
TEDC1	1.068504506	1.34E-09	1.08E-08
LINC02542	1.765876988	0.025427984	0.035768348
SH3KBP1	1.188996229	1.10E-11	1.57E-10
AL356652.1	1.794553202	3.44E-10	3.29E-09
AP005136.3	1.623848635	5.34E-13	1.10E-11
ISM1-AS1	1.998482825	1.17E-05	3.53E-05
SOX15	-2.305461111	5.30E-07	2.22E-06
TMEM191A	1.320877845	1.23E-07	5.99E-07
AC015813.6	1.063576511	1.34E-09	1.08E-08
CIP2A	2.098060993	2.92E-18	1.63E-15
TSACC	1.695442999	9.69E-14	2.49E-12
EIF5AP3	1.921893342	2.33E-11	3.04E-10
AC006947.1	1.091351206	0.002226065	0.003964095
MIR5094	2.083717199	1.37E-08	8.54E-08
SAMD12	1.366308697	1.21E-10	1.30E-09
ACTR3B	1.056306753	6.71E-13	1.34E-11
FUT4	1.147686808	1.55E-07	7.35E-07
AC113174.1	3.649487844	7.05E-07	2.86E-06
AC007448.3	2.157322474	9.61E-06	2.96E-05
CXCL11	3.404377295	5.41E-10	4.91E-09
TMEM74B	2.053362059	1.18E-07	5.77E-07
CD44-AS1	1.742840631	1.25E-07	6.08E-07
AC005920.1	1.138321687	0.000171187	0.00039216
AL133243.3	1.011580591	6.70E-07	2.73E-06
ARL4D	-1.951036389	5.30E-07	2.22E-06
PRSS33	3.952255856	3.79E-06	1.28E-05
KRT18P37	1.339537304	3.18E-10	3.07E-09
AC020907.3	1.931913143	4.45E-07	1.90E-06
CASQ1	-1.408411689	0.022360827	0.031895125
AC084024.1	1.372328466	4.02E-06	1.35E-05
C2CD4D	1.572645495	1.66E-09	1.30E-08
CCDC58P5	1.528297511	0.000185544	0.0004216
RPL9P30	1.435100704	1.13E-06	4.34E-06
AC245884.1	1.616394809	0.000120524	0.000285396
TASOR2	1.046577387	1.06E-14	3.88E-13
ITGAX	2.009393293	8.93E-13	1.71E-11
PRAC2	2.567757176	6.52E-08	3.41E-07
AL359921.1	1.151000841	3.40E-08	1.91E-07
HSPE1P10	1.477305721	2.30E-06	8.19E-06

AC007552.1	2.642652202	2.04E-07	9.40E-07
HNRNPA1P30	1.509812038	1.81E-08	1.09E-07
MET	2.491116347	1.68E-15	8.74E-14
MIR548L	1.888850097	2.72E-06	9.53E-06
AC010536.2	1.236791915	0.000244166	0.000539526
AC109361.2	1.203064638	9.06E-05	0.000221066
AC092757.2	1.062085911	4.52E-05	0.000118359
AC112225.1	1.100150323	0.003819105	0.0064631
AL157938.2	1.167120399	3.40E-05	9.19E-05
AC093458.1	1.62127492	0.000275947	0.000602407
AC010148.1	2.104447364	2.51E-09	1.88E-08
CDCA8	1.768287839	2.79E-14	8.71E-13
LINC02864	4.187925145	1.31E-05	3.91E-05
CCDC18	1.386871093	1.39E-14	4.85E-13
IFIT3	1.131043219	4.43E-05	0.000116288
AL354989.1	1.133613484	1.16E-05	3.51E-05
SERTAD4-AS1	-1.265914912	0.00046833	0.000971975
MICE	1.42917028	2.33E-07	1.06E-06
LINC01833	5.103972964	5.74E-10	5.18E-09
AC114737.1	1.890736952	4.79E-07	2.03E-06
AC117386.2	3.080260092	1.64E-06	6.07E-06
MIR3944	1.392413115	0.000531973	0.001089715
RPL23AP90	1.025866928	0.009770677	0.015101837
CCT5	1.164226611	4.47E-15	1.91E-13
KRT7	2.859644308	6.54E-07	2.67E-06
KRT18P15	1.816747325	4.34E-13	9.10E-12
AC005014.3	-1.252600659	5.27E-06	1.72E-05
RPL6P25	1.186901696	1.82E-05	5.26E-05
AC117490.2	1.015224707	2.67E-05	7.42E-05
PCDHGB9P	1.07799556	0.000905977	0.00176479
RGMB	-1.094444711	7.64E-08	3.93E-07
LYAR	1.340975005	1.32E-15	7.22E-14
NT5DC4	2.972471429	2.31E-13	5.29E-12
ENPP7P8	2.317539256	1.34E-05	4.00E-05
BCL2L1-AS1	1.7425177	1.54E-08	9.43E-08
AP002812.2	1.385909173	6.97E-07	2.83E-06
ETV5-AS1	1.292041814	0.000222422	0.000495763
AL355916.1	2.141788852	1.51E-06	5.63E-06
INSL6	2.152080211	0.013479795	0.020167838
PRRC2C	1.047666828	5.67E-17	7.71E-15
CST1	7.429488997	1.06E-18	1.29E-15

MARCKSL1	1.247855141	3.85E-10	3.62E-09
PDSS1P1	1.070832953	0.000328898	0.000705827
SNORA71A	1.201465923	2.48E-09	1.86E-08
Z98752.2	1.281536554	0.000320445	0.000689047
AHNAK	-1.134491359	2.26E-07	1.03E-06
WDR93	1.747363264	1.11E-07	5.47E-07
AC005291.1	2.030907346	3.19E-06	1.10E-05
NPM1P18	1.421964636	1.25E-07	6.10E-07
IGHV3OR16-17	-3.011532503	0.019969596	0.028769263
AC007038.1	1.609764696	2.61E-11	3.36E-10
CCDC69	-1.785916318	1.63E-11	2.22E-10
AC008267.5	1.291430633	0.000506747	0.001043739
AL513185.1	1.8583079	5.65E-05	0.000144604
RPS12P27	1.016652997	6.74E-05	0.000169556
HOXA11-AS	3.985983293	3.08E-13	6.82E-12
S100B	-2.05708412	1.06E-11	1.51E-10
PPIAP80	2.118101781	3.45E-09	2.48E-08
CKLF	1.150545653	1.63E-10	1.70E-09
BACH1-IT2	1.310099984	6.83E-08	3.55E-07
SNRPCP2	1.181579412	3.65E-06	1.24E-05
RN7SL57P	1.550242411	2.45E-05	6.85E-05
FOXH1	3.954474681	3.08E-12	5.08E-11
PLCXD3	-1.45135224	5.49E-12	8.52E-11
AL512326.3	1.198402928	0.003593106	0.006112869
CRYZP1	1.279793751	2.24E-07	1.03E-06
CNKSR2	-1.893077491	7.16E-05	0.000178892
ZNF670-ZNF69:	2.364541606	1.33E-15	7.28E-14
GNAZ	-1.073556919	3.74E-05	0.000100085
PPIAP67	1.395020803	0.015007482	0.022242344
PSPC1-AS2	1.377549774	1.53E-08	9.36E-08
CACYBPP1	1.476241445	1.20E-05	3.60E-05
AP006259.1	1.284660966	5.47E-05	0.000140496
ZNF321P	1.282257557	6.09E-08	3.20E-07
AL353796.1	1.040738479	4.70E-09	3.26E-08
TEX19	5.402978922	2.31E-08	1.35E-07
ASS1P10	1.361864376	2.14E-06	7.69E-06
ECE2	1.301596187	1.53E-11	2.09E-10
RPL35AP19	1.160808155	0.000518974	0.001066366
CEND1	-1.158299577	0.001222124	0.002309044
AL359092.2	1.107995065	1.78E-05	5.15E-05
AC138811.2	1.290148642	0.000501292	0.001033621

MARK2P8	1.08219884	1.90E-05	5.47E-05
AL139099.2	1.476975619	2.22E-07	1.02E-06
SCUBE1	-1.947122224	3.14E-08	1.78E-07
SKA1	2.093370952	4.69E-15	1.99E-13
RNU6-403P	3.039396028	2.77E-06	9.72E-06
IGLV2-33	-3.713235512	0.007227748	0.011496904
RPS20P9	1.307218333	2.73E-06	9.59E-06
AC007342.8	1.83277346	1.01E-07	5.06E-07
AC012676.3	1.223776644	7.63E-06	2.40E-05
AC073316.2	1.835441105	0.000692161	0.001381212
AC012615.4	1.428112541	3.08E-07	1.36E-06
AC008115.3	1.346871815	4.19E-09	2.95E-08
RN7SL430P	3.078234359	1.23E-07	6.01E-07
CERS3-AS1	-2.487014458	6.76E-06	2.15E-05
NFE2L1-DT	1.374979212	1.83E-08	1.10E-07
RN7SL840P	2.59874086	1.03E-06	4.00E-06
AP000892.4	-1.318926011	0.008093457	0.012735567
NPAS4	-2.649538552	5.88E-05	0.000149785
AC023825.2	1.795285334	2.46E-09	1.84E-08
DHRS2	4.992584782	8.32E-07	3.32E-06
KRTAP5-1	2.161210762	5.99E-11	6.98E-10
RNU4-5P	1.366160559	0.001511697	0.002791808
AURKB	1.766400743	1.63E-12	2.91E-11
BMERB1	-1.036633143	5.45E-05	0.000140169
RNU6-181P	1.393724649	0.001704276	0.003115074
LZTS3	1.300105415	2.90E-05	7.98E-05
AC010761.3	1.454545113	5.48E-08	2.91E-07
PAICS	1.441200262	9.38E-17	1.06E-14
MIR4802	1.009909673	0.002617744	0.004585569
IGF2BP2-AS1	2.376879797	1.44E-10	1.52E-09
AL359740.1	1.006741436	0.005954733	0.009645307
AC002059.1	1.189714099	5.49E-05	0.000140905
NME8	1.226042202	0.000348147	0.000742781
GFRA2	-2.235385504	9.92E-11	1.09E-09
CCDC162P	2.930597373	3.07E-10	2.98E-09
AC087269.1	3.482758194	1.19E-10	1.28E-09
BX255925.1	1.271767539	1.41E-06	5.29E-06
AC020978.7	1.015537632	3.72E-07	1.61E-06
MTBP	2.251252795	9.44E-19	1.26E-15
TNFRSF10C	1.028446833	0.009122169	0.01419833
EIF4E2P1	1.420882242	0.000715289	0.0014231

AC104365.1	2.599005062	3.76E-07	1.63E-06
FBXW11P1	1.143722872	1.76E-06	6.47E-06
NR5A2	1.051476826	1.40E-05	4.16E-05
NPPA	1.001581633	0.002088706	0.003741498
AC093583.1	-1.444317819	0.007236643	0.01151017
AC125603.2	3.921305229	0.003152919	0.005434788
POLR3G	1.250034889	7.98E-08	4.08E-07
RNU6-254P	1.546193759	0.002614682	0.00458081
RNU6-136P	1.622916424	0.000289642	0.000628697
EDAR	1.544407577	0.028565088	0.039676337
NKD2	2.825843153	2.38E-06	8.45E-06
CDK6-AS1	1.472081659	0.008266058	0.01298569
NRADDP	1.108280197	9.76E-06	3.00E-05
AP000866.3	1.163672322	3.05E-07	1.35E-06
ODF2	1.141366467	1.70E-17	3.74E-15
IGLV3-24	-2.920694311	3.21E-05	8.73E-05
ARL5B	1.005692629	1.66E-10	1.73E-09
LINC02544	4.414802231	3.11E-12	5.12E-11
PHBP9	1.028369024	1.15E-06	4.43E-06
AC103739.1	1.37560921	1.70E-07	8.02E-07
AATF	1.123052698	5.80E-15	2.35E-13
AL355922.1	1.542321466	0.005835086	0.009472238
GTPBP4	1.399641483	2.54E-19	1.26E-15
IGKV2-4	-2.320443469	0.000287649	0.000624882
AC145423.1	1.018122888	7.89E-05	0.000195208
AC133528.1	1.407697154	5.04E-06	1.66E-05
AC009296.1	1.640466839	8.20E-05	0.000202009
RAI14	1.182588883	1.48E-11	2.03E-10
PRC1-AS1	1.534388316	3.64E-09	2.60E-08
DSCR9	1.422169255	1.29E-06	4.90E-06
SLC11A1	2.007428581	5.55E-12	8.60E-11
AC092338.1	1.15087086	1.51E-06	5.63E-06
AC104534.1	2.027477909	3.36E-06	1.15E-05
TACR2	-2.046868446	2.11E-05	6.02E-05
C9orf50	1.550534118	1.33E-06	5.04E-06
AL031717.1	1.379905872	5.91E-09	4.00E-08
AC022558.3	1.155909116	1.08E-07	5.35E-07
AC100823.2	1.750664059	2.80E-06	9.81E-06
MIR23B	-2.074149794	0.003376817	0.00577848
AC027544.2	1.314342856	5.19E-06	1.70E-05
INO80-AS1	1.143063539	1.30E-05	3.89E-05

ABCA13	2.320747708	4.65E-07	1.97E-06
SOX12	1.133506682	1.35E-06	5.10E-06
AC083863.1	1.032069948	0.004046594	0.006811014
AC009054.1	1.286358158	5.69E-05	0.000145553
HCG15	1.122763336	0.024816463	0.034992773
AC243967.2	2.574005194	3.44E-06	1.18E-05
AC127024.4	1.048697998	3.57E-06	1.22E-05
LINC01342	1.860316119	3.87E-10	3.64E-09
AC011754.1	-1.136968335	0.000671928	0.001343957
TMC5	1.159879039	5.01E-06	1.65E-05
ANKRD35	-2.240054316	3.16E-09	2.30E-08
ZMYND10	1.176914173	1.18E-06	4.53E-06
MC1R	1.590378176	2.48E-13	5.63E-12
PDILT	-3.041776252	0.000143053	0.000333265
PLA2G5	-1.359037265	0.000350284	0.00074694
MIR623	1.283091017	0.001149415	0.002186243
SNORD53B	1.768133212	2.42E-06	8.58E-06
TAF4	1.167130185	9.89E-17	1.08E-14
CAND2	-1.330815241	0.0002451	0.000540963
AC036222.2	1.639389413	0.000134232	0.00031464
SLC26A8	1.679992826	2.13E-09	1.63E-08
IFITM3P1	1.59981806	1.08E-09	8.94E-09
AC113410.3	1.796299762	7.54E-09	4.99E-08
FLRT1	-2.083230428	0.000184503	0.000419449
LYPLAL1-DT	-1.341181767	0.000531015	0.00108792
ADAP1	1.469388138	3.99E-11	4.88E-10
AP002336.1	1.709122754	6.76E-07	2.75E-06
MIR98	1.490801548	2.30E-05	6.49E-05
CFAP251	1.802617398	1.61E-09	1.27E-08
KANK2	-1.305823998	0.000538829	0.001101979
IGHV3-65	-3.1648801	0.014928513	0.022137674
AC107959.3	1.599016482	8.71E-06	2.71E-05
COL21A1	-1.864265697	0.006744509	0.010802406
TCP1P3	1.244565678	9.23E-10	7.80E-09
RNU6-50P	1.329386606	0.000162522	0.000374269
AC104958.2	1.937534752	0.002357142	0.004174076
RNA5SP284	1.42340425	0.024820541	0.034995176
EIF2S2P2	2.087401851	8.65E-12	1.27E-10
SRMS	1.924315538	2.28E-07	1.04E-06
LINC01913	4.377753942	2.24E-09	1.70E-08
DCAF13	1.635014096	2.12E-18	1.49E-15

CWH43	-3.834080856	3.24E-11	4.04E-10
RNU6-125P	1.000802952	3.61E-05	9.70E-05
AC245052.3	1.140553093	6.65E-05	0.00016754
SOX21	-2.31614276	4.78E-07	2.02E-06
BNC2-AS1	-1.135114054	0.000173284	0.000396685
AL450263.1	1.068494838	5.08E-06	1.67E-05
AL355480.2	1.323314632	0.002913117	0.005056438
IQGAP3	2.434749755	7.11E-17	8.73E-15
AC004678.1	1.328800478	1.94E-09	1.50E-08
STC1	1.289294134	1.20E-07	5.85E-07
AOC3	-1.60342552	2.07E-06	7.48E-06
AMZ1	1.227907044	3.03E-07	1.34E-06
ZNF81	1.007244196	3.34E-11	4.14E-10
LCN6	-1.752846751	1.73E-11	2.34E-10
GPC2	2.137887385	8.12E-10	6.99E-09
SMKR1	2.411443564	1.28E-10	1.38E-09
AP005131.4	1.355263628	0.000947315	0.001839807
AC107072.1	1.089443112	0.002659507	0.00465261
PRR7	1.883713486	1.57E-14	5.40E-13
LNCAROD	4.557725209	0.010806616	0.01653514
JAKMIP2	-1.570815468	0.00411915	0.006919008
RNA5SP298	1.870992495	2.35E-05	6.62E-05
TRAJ37	2.753064244	3.55E-05	9.54E-05
AC133561.2	-2.333517656	0.007416509	0.011771857
AC016542.1	2.335178408	1.27E-09	1.03E-08
METTL27	2.001654246	6.83E-08	3.55E-07
BORA	1.578006027	5.47E-14	1.53E-12
CHORDC1P4	1.468998995	2.73E-09	2.02E-08
MIR3198-1	2.249512467	7.73E-09	5.11E-08
AC005330.1	1.321324989	0.002459445	0.004336214
AC005363.2	1.266599194	0.000459193	0.000954947
AL021707.6	1.280654696	6.71E-09	4.49E-08
AGBL2	1.701933388	5.89E-11	6.89E-10
AL031123.3	1.755820272	3.48E-10	3.32E-09
HSPB8	-2.471514549	4.34E-10	4.04E-09
RRN3P1	1.099428909	6.77E-08	3.52E-07
AL078604.2	2.419334814	3.45E-10	3.29E-09
E2F7	2.486172183	5.52E-16	3.71E-14
LINC02331	3.624487662	7.93E-06	2.48E-05
RN7SL431P	1.085792083	2.45E-05	6.86E-05
KRT23	3.85242936	3.74E-06	1.27E-05

FAM189A1	-1.005868716	9.03E-06	2.80E-05
ITGB1-DT	1.623607359	4.58E-06	1.52E-05
CENPE	2.350841274	9.89E-17	1.08E-14
FAM107A	-2.020171035	1.81E-13	4.28E-12
INCENP	1.033701661	1.56E-09	1.24E-08
SNRPCP11	1.174247995	0.01198088	0.018142242
PPIAP39	1.322287022	9.92E-06	3.04E-05
AC002091.1	1.057009894	0.001514627	0.002797025
ROR2	-1.297599602	0.001088695	0.002083244
SNORD70B	1.727885602	0.000399225	0.00084139
KRT7-AS	2.936214408	3.12E-10	3.02E-09
LCN1	6.03897712	4.09E-09	2.89E-08
SNORA9B	1.778218081	2.16E-08	1.27E-07
LARP7P2	1.450768841	0.000583469	0.001182922
MCIDAS	1.566248604	1.51E-07	7.21E-07
IFNG	2.121420953	2.84E-07	1.27E-06
AQP2	5.630319005	4.97E-06	1.63E-05
HSPA8P3	2.033321155	1.69E-10	1.75E-09
AC103996.1	1.494847015	0.000648191	0.001301018
MDK	1.304767595	5.43E-07	2.27E-06
ARHGEF38-IT1	2.459655606	1.11E-10	1.21E-09
DNAAF4	1.317223324	1.76E-08	1.06E-07
SULF2	1.341754151	3.96E-09	2.81E-08
LINC01705	2.744543605	7.83E-10	6.78E-09
MYH11	-2.426161455	7.03E-09	4.68E-08
MIR100HG	-1.474225314	0.004891471	0.008075261
TOB2P1	1.013162839	0.009723123	0.015032698
C2CD4A	2.927956994	3.09E-10	2.99E-09
RNU6-106P	1.538677311	0.002585123	0.0045326
AC008474.1	2.327406944	7.78E-08	3.99E-07
LMLN	1.053688676	9.46E-13	1.80E-11
LRRC37A9P	2.667810127	7.70E-13	1.50E-11
COL5A1	1.658636236	4.04E-09	2.86E-08
AC087521.1	-2.169233393	0.000665153	0.001332105
NCAPD3	1.51954005	3.13E-16	2.37E-14
AC119403.1	2.053676984	4.09E-06	1.37E-05
AL359385.1	2.252490098	9.28E-07	3.65E-06
DSCR8	7.386615767	9.56E-06	2.94E-05
AL359880.1	2.416467421	3.02E-10	2.94E-09
PAX8	1.67992636	4.40E-09	3.07E-08
AL034550.1	2.158454889	9.58E-14	2.47E-12

AC027796.2	1.140063807	2.38E-06	8.44E-06
PRELID1P5	1.10525203	5.20E-08	2.79E-07
UBASH3B	1.177794418	1.65E-07	7.81E-07
ECEL1P1	4.787684086	1.68E-08	1.02E-07
KRT8P34	1.366828086	2.19E-05	6.23E-05
CSE1L-AS1	2.002707119	1.43E-05	4.24E-05
AC015910.1	1.290611076	0.002313768	0.00410655
HS3ST2	2.220582217	1.63E-06	6.04E-06
LINC01357	2.157739376	1.05E-09	8.75E-09
ARL13A	1.042027353	8.13E-07	3.25E-06
ATP5PBP3	1.722873833	0.002330405	0.004132773
H4C11	1.010255954	3.62E-05	9.70E-05
POLE	1.209513874	3.82E-14	1.13E-12
BCL2L12	1.088016852	2.96E-10	2.89E-09
AC129492.1	2.020432741	1.23E-05	3.69E-05
RCAN2	-1.80269879	1.42E-08	8.77E-08
RPL23AP64	1.021660669	1.28E-06	4.86E-06
AC007688.1	1.278046268	0.000195996	0.000442144
LGALS17A	3.934973331	1.20E-08	7.57E-08
AC244093.2	1.804018437	3.56E-07	1.56E-06
PLCD4	-1.668332592	0.023925632	0.033867844
AL034418.1	1.554364576	1.88E-06	6.86E-06
ENAM	-1.779322852	1.16E-07	5.68E-07
PALB2	1.116702024	6.65E-15	2.64E-13
TRIM59	1.79963586	2.32E-17	4.78E-15
AC083809.1	4.190254957	3.07E-05	8.38E-05
KIAA1549L	2.088436071	2.58E-05	7.20E-05
MLXP1	3.714228498	3.67E-07	1.60E-06
SNORD121A	2.916103896	1.31E-08	8.17E-08
ASH1L-IT1	1.596141804	8.54E-07	3.39E-06
RPP25	1.342698565	8.98E-12	1.32E-10
AL391056.1	2.868997215	8.13E-07	3.25E-06
AC046143.1	1.245047182	9.69E-12	1.40E-10
MTND1P11	1.168582636	3.66E-05	9.82E-05
MLXIPL	1.823747099	2.25E-05	6.36E-05
PDE1B	-1.168514278	0.001150456	0.002187287
UPF3AP3	1.00128387	4.37E-05	0.000114832
RNU4ATAC18F	1.118576089	7.78E-05	0.0001929
MIR7848	1.943855355	0.000202979	0.000456505
AP003071.3	-1.478593409	0.026528667	0.037167478
AC021739.2	1.149596744	4.01E-07	1.73E-06

FKBP9P1	1.298326389	0.000101044	0.000243733
CAPN13	-1.22443196	0.016359792	0.024022202
SLC28A3	2.380513013	4.81E-08	2.60E-07
ZNF469	2.706642038	2.71E-15	1.29E-13
NUP205	1.050758934	8.31E-14	2.17E-12
KCNA1	-2.907315412	0.009070391	0.014125259
AC083843.3	1.121418472	0.001228837	0.002321074
UBFD1	1.019556339	4.73E-16	3.28E-14
LINC01050	7.679021055	6.60E-16	4.22E-14
ZKSCAN2-DT	1.263109614	3.30E-11	4.11E-10
RDM1	1.953089898	9.38E-12	1.36E-10
PAIP1P1	1.31977999	9.66E-08	4.84E-07
PRAM1	1.053217372	5.75E-05	0.000146889
MIR589	1.484495158	6.04E-07	2.49E-06
VAX2	1.854924485	0.015141801	0.022417908
RN7SL459P	1.040621508	0.008394965	0.013169421
ONECUT1	2.891964564	0.035179676	0.047836315
AL121839.1	2.523083084	1.82E-08	1.10E-07
GZMB	1.220667147	0.013943599	0.020792867
PLAAT3	1.09477202	0.00022911	0.000509553
GPR17	-1.440736904	6.57E-05	0.000165577
EFNA3	1.261955815	5.94E-09	4.02E-08
ULBP1	3.699662389	1.86E-12	3.27E-11
AP000346.1	1.536123323	3.93E-06	1.32E-05
AC120053.1	1.2000835	4.10E-08	2.26E-07
PPIAP10	2.222196297	1.18E-05	3.56E-05
CPHL1P	3.601454165	1.59E-09	1.25E-08
FANCI	2.200336752	1.75E-17	3.78E-15
TRAJ31	1.144467943	0.031062304	0.042745966
RN7SL81P	1.221738666	2.94E-06	1.02E-05
CLDN2	3.340666925	3.40E-07	1.49E-06
H4C9	1.188141085	0.0006299	0.001267221
AC106897.1	-1.219121816	1.45E-09	1.15E-08
LMO7-AS1	1.812505137	2.15E-08	1.27E-07
PPM1H	1.956280147	7.03E-14	1.89E-12
AL139274.1	1.516681459	2.38E-05	6.70E-05
AL162377.2	1.068385964	0.00451523	0.007508099
AL359922.2	1.456581514	9.33E-09	6.06E-08
KNSTRN	1.492433663	1.33E-14	4.65E-13
FNDC1	3.639623021	4.19E-13	8.83E-12
AC011447.7	2.051349947	0.000101044	0.000243733

PPIAP54	1.383659281	8.94E-07	3.53E-06
MIR4787	-1.873972538	0.003470104	0.005922519
PPP1R1A	-3.686285853	9.32E-10	7.86E-09
CPB2	2.911490549	5.87E-06	1.90E-05
AC022960.2	1.485915297	0.000169714	0.000389049
AL121845.4	1.34178291	1.01E-06	3.95E-06
AC083880.1	1.075435716	9.42E-07	3.70E-06
KRT24	-2.205999865	4.36E-13	9.13E-12
ST8SIA6-AS1	4.649718921	0.01016543	0.015667441
PLEKHG4	2.640995165	1.12E-14	4.01E-13
SFRP1	-2.322146951	1.41E-12	2.57E-11
U47924.3	1.026431285	0.000193004	0.000436615
PDLIM4	-1.150232335	3.09E-05	8.43E-05
CCT6P2	1.664514287	2.11E-11	2.79E-10
GAPDHP66	1.200821309	0.011142574	0.016998423
RRS1	1.130543758	1.74E-12	3.08E-11
CFAP52	1.570001855	1.72E-08	1.04E-07
ZNF814	1.134018886	5.09E-11	6.01E-10
CARD11-AS1	1.66499167	1.88E-05	5.41E-05
AC114284.1	1.134732531	3.38E-05	9.13E-05
SNRPCP6	2.159217571	0.001754788	0.003197967
SNORD7	2.253193039	8.59E-10	7.35E-09
BTNL9	1.11397366	7.95E-05	0.000196366
AC079926.1	1.433918902	0.000970957	0.001881874
UBE2C	2.398197579	9.51E-15	3.54E-13
AC138956.1	1.250538501	1.90E-07	8.86E-07
AF111167.2	-1.105174592	4.26E-10	3.98E-09
HNRNPA3P10	1.109377985	4.86E-09	3.35E-08
AL512306.2	1.230291094	0.012886327	0.019371419
AP001784.1	2.394364619	0.000898734	0.001752604
TNNC1	2.769588174	1.55E-07	7.35E-07
CST4	6.989281692	5.50E-17	7.55E-15
POLD1	1.452478689	1.30E-13	3.21E-12
MORF4L1P3	1.088907472	4.69E-05	0.000122279
CACNA1E	2.962361401	6.62E-06	2.11E-05
AC087857.1	4.613420255	7.05E-05	0.000176481
AC126773.5	1.303016347	1.61E-05	4.71E-05
AL391845.2	3.892613892	2.60E-16	2.04E-14
KRT18P13	2.157941146	1.74E-12	3.08E-11
ALG8	1.243906755	9.83E-16	5.77E-14
PIF1	1.545329892	2.60E-13	5.88E-12

AC004912.1	1.87496525	0.006108402	0.009868417
LINC00355	5.481771219	2.39E-07	1.09E-06
PDE11A-AS1	4.525436129	1.33E-06	5.03E-06
GPR137C	1.347739267	3.52E-10	3.34E-09
ERI1	1.357644006	7.20E-17	8.80E-15
RNU6-1141P	1.567538623	0.001837104	0.00332908
RNU7-75P	2.271307214	7.51E-09	4.98E-08
IRAK2	1.211470698	2.83E-07	1.27E-06
LINC02156	1.991780597	2.31E-06	8.23E-06
RPSAP43	1.328745593	3.09E-06	1.07E-05
AC104964.3	1.641125518	3.70E-05	9.92E-05
HMGA1P4	1.477729014	9.92E-07	3.87E-06
AP000866.6	1.462142835	5.02E-10	4.60E-09
TCAM1P	4.763986518	9.36E-11	1.03E-09
MIR5692A1	1.070239349	0.013791695	0.020588241
DGKB	-2.627915854	0.018659348	0.027071397
RNA5SP498	1.48108053	0.009018157	0.014051216
AC008114.1	2.988049497	1.21E-11	1.70E-10
FOXN2	1.067089764	8.41E-15	3.18E-13
AC015912.2	1.16937945	0.000560316	0.001141454
CDK3	1.183455939	1.76E-08	1.06E-07
FAM91A1	1.561570282	1.58E-18	1.34E-15
CYP2C18	-1.706072638	6.19E-05	0.000156871
TOP1MT	1.401602911	1.35E-15	7.33E-14
CKS2	1.670199067	1.11E-12	2.08E-11
RN7SL748P	1.112700928	0.000112179	0.000267418
AC011462.4	1.478565914	4.39E-09	3.07E-08
C2	1.747130343	1.46E-09	1.16E-08
FOXC2	1.282137766	0.000564446	0.001148111
KRT18P31	1.088335464	2.44E-08	1.42E-07
HMGB1P40	1.434741798	8.66E-09	5.66E-08
AC138932.4	1.28119406	4.76E-07	2.02E-06
THAP12P7	1.204936132	2.73E-09	2.02E-08
AL049758.1	1.041611321	0.000488535	0.001009663
TAS2R5	1.542971	4.04E-09	2.86E-08
AC025575.2	3.288155258	1.29E-07	6.26E-07
PPIAP77	1.744494952	1.00E-08	6.46E-08
MIR548AA1	2.47917114	1.82E-09	1.42E-08
AC013244.2	1.06951483	0.001781242	0.003240856
AC105118.1	2.493634791	5.26E-05	0.00013556
AC093607.1	-1.600167717	0.006386322	0.010280006

RN7SL812P	1.325179625	3.92E-05	0.000104446
RPS14P5	2.295419402	2.31E-07	1.05E-06
TPM2	-2.007616764	0.000404111	0.000850085
P3H2	-1.077818031	1.20E-06	4.59E-06
NDUFB4P10	1.225029122	0.027509433	0.038372058
KIF20A	2.129019278	9.58E-16	5.69E-14
AL593851.1	1.413916746	0.004254916	0.007121913
RPS29P9	1.346829998	0.000567994	0.001154623
CD36	-1.707240351	5.07E-09	3.49E-08
AC010973.2	1.335037817	7.27E-13	1.43E-11
AL353748.1	1.178322107	0.003087929	0.005332422
LINC01948	1.187141996	0.000101461	0.000244715
FBXO36-IT1	1.158607181	0.000141693	0.000330444
AC022167.2	1.042113484	1.19E-09	9.75E-09
CYP51A1P1	1.180581696	0.000278554	0.000607299
GMNN	1.057910813	8.97E-10	7.61E-09
AP2B1P1	1.168788275	1.38E-07	6.66E-07
ALMS1P1	1.351207718	4.96E-05	0.000128714
RPL17P17	1.050142861	0.000689908	0.001377541
AC140658.7	-1.000490666	0.004280027	0.007158996
EIF2B5-DT	1.095146981	5.98E-07	2.47E-06
TPTE2P5	1.181062658	3.85E-06	1.30E-05
ZNF683	1.361733688	0.010933441	0.016712877
Z97652.1	1.688217257	1.76E-05	5.09E-05
IGKV1D-8	-3.064186905	0.022779046	0.032411145
ITPK1P1	1.137942387	9.02E-07	3.56E-06
MIR499A	1.382944893	0.00439439	0.007331413
DLGAP4-AS1	1.010426862	1.52E-05	4.47E-05
MTND4P23	2.02384013	4.72E-09	3.27E-08
RAB6D	1.562852173	2.48E-07	1.12E-06
IGHV3OR16-9	-2.164148868	0.027197935	0.037995248
AL022322.1	2.062736419	2.56E-14	8.09E-13
CC2D2A	-1.028339037	0.007003488	0.011177555
AC135050.3	1.016998911	3.05E-05	8.32E-05
RTL3	-1.261914173	0.017687506	0.025801059
LGR5	3.144521251	1.12E-07	5.51E-07
MFSD2B	1.412674524	3.16E-11	3.95E-10
AC069277.1	2.83661695	0.0010267	0.001976969
C1orf195	1.443519719	6.74E-08	3.51E-07
AC002310.3	1.90063205	2.48E-08	1.44E-07
AC073657.2	1.31088929	0.000134504	0.000315224

COX19	1.185295427	1.76E-16	1.57E-14
TM4SF19-AS1	1.268614946	2.24E-06	8.01E-06
AC007991.4	4.425374127	3.87E-10	3.64E-09
AL121772.2	2.265869129	2.12E-06	7.64E-06
SPINK14	1.878629864	0.002714526	0.004738904
ZNF600	1.305893684	5.99E-13	1.21E-11
LINC02830	5.18548785	2.65E-06	9.32E-06
PIK3CD-AS1	1.324191256	0.000113347	0.000269962
COL19A1	-1.75402359	0.000875844	0.001711484
LHFPL5	2.010047275	7.58E-06	2.39E-05
AC027237.2	1.356744757	2.07E-05	5.91E-05
IGKV1OR10-1	-3.529162556	0.01032174	0.015874375
HNRNPA1P59	1.225583869	7.58E-10	6.58E-09
RNU6-1322P	2.202779645	1.35E-05	4.01E-05
RBM20	-1.84402252	2.71E-05	7.51E-05
RNU6-618P	1.286954849	4.29E-05	0.000112862
AC093582.1	1.534251947	1.07E-06	4.13E-06
AL592295.2	1.705920062	3.64E-06	1.24E-05
NUDCD1	1.641131871	6.74E-19	1.26E-15
AC004009.1	2.183501995	0.000172492	0.00039494
STAG3L5P	1.544245863	2.26E-12	3.87E-11
FSCN1P1	1.578598558	1.48E-06	5.55E-06
TRBV26OR9-2	-1.321617632	1.40E-08	8.68E-08
TIMELESS	1.853647626	1.61E-16	1.50E-14
TNFRSF9	1.630672776	3.81E-10	3.59E-09
GABRA5	-2.817896104	1.63E-06	6.03E-06
AL162390.1	1.091214045	0.000164876	0.000379002
CKM	-2.479645866	1.30E-05	3.90E-05
AC108693.1	1.349204738	2.50E-06	8.83E-06
AC040904.1	1.079917538	3.87E-05	0.000103323
PLXND1	1.099815839	3.43E-08	1.93E-07
LRRC43	1.077946575	0.011639113	0.017674945
AC103988.1	1.02498807	2.80E-07	1.25E-06
CLCN4	1.878476891	4.16E-09	2.93E-08
RNU6-1301P	1.099439053	0.007828612	0.012362627
AQP3	-2.056412988	0.008554591	0.013389231
AL928654.4	1.666639848	1.98E-14	6.57E-13
RFX8	2.413562112	1.01E-11	1.46E-10
Z98048.1	1.145233987	1.04E-05	3.16E-05
PXYLP1	1.295011026	8.02E-14	2.11E-12
RPS3AP38	1.104869513	1.75E-05	5.07E-05

AL133330.1	1.507430589	0.000111273	0.000265708
TAFA5	1.795222788	1.07E-07	5.31E-07
HK2	1.203953991	7.87E-07	3.15E-06
AC007621.1	1.777926253	5.45E-07	2.27E-06
PCDH9	-1.51554162	0.000963461	0.001868297
MIR378J	1.474288044	1.75E-06	6.44E-06
AC008267.4	2.129481042	1.14E-08	7.25E-08
ELF2P1	1.527256689	0.000285562	0.000620652
AC005786.1	1.046505125	0.000136871	0.000320236
RNU2-46P	1.60995514	0.010500756	0.016115273
RNA5SP310	1.599578699	2.03E-05	5.81E-05
AC027455.1	1.169628676	0.000140552	0.000328098
MTMR11	1.100093058	5.17E-07	2.17E-06
AC100788.2	1.053248248	0.001256042	0.002365751
PHOX2A	-2.791996808	0.002994799	0.00518538
AC022080.1	1.098611008	0.000109834	0.000262687
HSPB3	-2.760100487	3.52E-05	9.46E-05
HAPLN3	1.948009483	5.72E-13	1.17E-11
AL139384.1	1.260568376	2.67E-07	1.20E-06
COL6A4P2	1.465430869	0.000186779	0.00042398
IGLV7-35	-3.591537659	0.007040174	0.011229379
GRIN2A	-1.305144031	8.66E-07	3.43E-06
PRRG1	1.077524721	1.03E-07	5.15E-07
IGKV2-18	-3.159810864	0.000437769	0.000913888
AF131215.1	1.899867099	0.001041355	0.002002446
DGCR5	2.079194628	7.64E-06	2.40E-05
IGKV2D-30	-3.724686794	0.00166388	0.003047521
RPS2P1	1.022905757	2.31E-06	8.23E-06
CA15P1	1.085654735	0.004787714	0.007918199
RNU6-1283P	1.944786254	0.003176157	0.005471656
AC091132.1	1.236349577	0.001566014	0.002884722
RPL39P6	1.342620265	4.42E-06	1.47E-05
CLMN	1.149034835	6.60E-10	5.84E-09
POLG2	1.201472833	6.04E-16	3.92E-14
IFIT2	1.172952195	4.43E-05	0.000116288
PSLNR	4.761794709	1.80E-05	5.20E-05
SMC2	1.517611462	2.48E-16	1.96E-14
TTF2	1.316372197	8.99E-16	5.42E-14
GDF7	-1.437296943	2.92E-08	1.67E-07
CNTN3	-1.312504008	1.76E-05	5.10E-05
SOX9	1.973297698	1.61E-16	1.50E-14

AC138150.2	1.895523273	5.84E-07	2.42E-06
LINC00174	1.319353539	3.37E-09	2.43E-08
AK4P3	1.91901382	0.000102642	0.000247316
ANKRD44-IT1	1.31480455	0.00222574	0.003963881
AC073869.1	1.020751542	0.001007389	0.001944281
HPCAL4	-2.846454287	2.18E-13	5.03E-12
LMNTD2-AS1	2.185418437	2.88E-10	2.81E-09
AC244093.1	1.072227857	7.03E-07	2.85E-06
RELL2	1.193195498	1.30E-07	6.31E-07
LNCOG	3.35237974	2.14E-15	1.06E-13
FZD2	1.634992312	5.67E-09	3.85E-08
CASQ2	-2.651405076	2.37E-10	2.37E-09
LINC00896	1.502718427	4.05E-06	1.36E-05
C1orf147	1.667647114	2.99E-10	2.92E-09
ADAM8	1.564029013	6.71E-09	4.49E-08
LRRC8B	1.219501736	3.11E-15	1.43E-13
C2orf66	1.39372592	0.028330667	0.039390369
AC009093.6	1.445784453	5.54E-06	1.80E-05
AL162171.2	1.07159972	7.83E-05	0.000193869
EPM2A	-1.414919668	0.000380806	0.000805514
MIR181B2	1.812069639	6.91E-05	0.000173206
GFOD1	-1.361585449	0.007981707	0.012578294
PTGDR2	-2.192059963	0.000367433	0.000780447
ABCG2	-2.372371587	0.021681897	0.031014716
AL596223.2	1.423828117	8.12E-06	2.54E-05
SERPINH1P1	1.59433637	2.39E-11	3.10E-10
AC060773.1	1.794414451	1.52E-06	5.67E-06
CBFB	1.336456916	8.11E-17	9.52E-15
GGH	2.180710159	3.13E-11	3.92E-10
AC016550.2	4.245310163	0.000116843	0.000277594
AC021028.1	1.312039819	7.06E-05	0.000176713
AC022494.1	1.350659241	3.70E-06	1.26E-05
SLCO4A1	1.517917275	4.32E-08	2.36E-07
FBN3	1.817296239	0.002822058	0.004913427
SCD	1.284751026	6.97E-05	0.000174608
AC025048.4	1.150832852	7.98E-08	4.08E-07
RPS2P41	1.414588014	8.64E-06	2.69E-05
HSP90AB2P	1.502311295	7.55E-14	2.00E-12
AC073964.1	1.874857663	0.00114659	0.002181026
SETP5	2.000318838	3.65E-10	3.45E-09
AL031719.2	1.083488206	1.07E-08	6.80E-08

Z98742.2	1.351995598	0.000830634	0.001630304
RNU6-1306P	1.158197471	0.007949537	0.012532045
CNIH3-AS2	1.711757011	0.00013628	0.000319132
AC005261.2	1.264308496	2.35E-05	6.62E-05
LINC01876	1.93378826	4.58E-05	0.000119781
PTGR1	-1.56013348	6.48E-08	3.39E-07
TRIM72	3.069840144	1.04E-05	3.17E-05
PPIAP20	1.440956136	1.97E-06	7.14E-06
GINS4	2.005448989	6.04E-16	3.92E-14
AL022331.1	1.141999786	0.000122298	0.00028947
AC020891.3	1.671568361	1.00E-04	0.000241517
RNY4P17	1.0773489	0.00415178	0.006967667
LINC01232	1.325035714	2.63E-10	2.60E-09
AC023632.1	1.336685381	4.80E-05	0.00012498
AL591806.1	1.673878617	2.20E-05	6.25E-05
SCAT8	1.535466116	0.029104639	0.040352092
RNU6-218P	1.660100152	0.000770347	0.001522182
AC074029.3	1.318700865	4.99E-05	0.000129501
DNAJC2	1.413830456	4.29E-18	1.81E-15
AC010343.2	1.909454865	0.000142364	0.000331804
H2AX	1.063928537	3.11E-08	1.77E-07
IGHV3OR16-8	-2.957945556	0.017341095	0.025345702
AC009095.1	1.324095158	2.41E-09	1.82E-08
CXCL3	1.612221343	8.26E-07	3.29E-06
AC022730.2	1.151367829	0.009912472	0.015305905
NDUFAF4P4	1.167605051	0.000570621	0.001159786
PKMP3	-1.558654751	0.003239312	0.005569451
AL049612.1	1.66305272	0.016382656	0.024053054
CRTAC1	-1.616791261	4.01E-07	1.73E-06
AL513175.2	1.283172951	4.27E-06	1.43E-05
RPL23AP15	1.028525267	0.000675215	0.001349619
CXCL1	2.898342335	5.31E-11	6.25E-10
DSTN	-1.101306262	0.013882917	0.020715131
CARMIL1	1.052687168	4.80E-10	4.42E-09
RN7SL166P	1.872206161	6.01E-06	1.94E-05
MIR4999	1.2081284	0.001936835	0.003492464
AC099066.2	2.548420543	1.13E-10	1.23E-09
MTATP6P2	1.29383016	0.010405929	0.015982634
SMYD3-IT1	1.31881181	0.000119765	0.000283852
RTN4IP1	1.122433219	1.00E-08	6.43E-08
FAT3	-2.799958975	0.006252221	0.010082421

CCDC28A-AS1	1.257447407	8.78E-08	4.44E-07
AL442663.4	1.483784004	2.77E-08	1.59E-07
SNORA50A	1.348660124	0.000918404	0.001787168
MTND4LP14	3.07513156	1.93E-08	1.15E-07
RNU6-805P	1.267095292	0.024880286	0.035075701
C2orf88	-1.265312325	0.001774174	0.003228216
TACC3	1.535707599	1.02E-12	1.94E-11
AC244131.1	1.523455259	1.59E-05	4.67E-05
PHBP2	1.526283537	6.53E-10	5.79E-09
SCARA5	-3.022180204	1.37E-16	1.36E-14
AL596448.1	1.539260944	5.62E-05	0.000143967
GAPDHP22	2.097505122	1.04E-07	5.16E-07
BTC	-1.479631879	0.000175523	0.000401122
RPL37P1	1.122309513	2.83E-05	7.79E-05
IRAK1	1.02027373	9.72E-11	1.07E-09
TUFMP1	1.960644176	7.49E-05	0.000186464
KRT18P34	1.128872856	2.53E-09	1.89E-08
AC010273.2	1.061484436	8.46E-07	3.36E-06
ADAM9	1.053356219	1.26E-07	6.12E-07
PGM2L1	1.996149394	1.30E-18	1.31E-15
MUC20P1	1.34028306	0.033696649	0.046009949
AC005041.3	1.908654293	3.49E-12	5.70E-11
NDN	-1.225170239	3.82E-08	2.12E-07
CCDC198	4.952081813	5.00E-07	2.11E-06
FCF1P7	1.346097676	6.38E-07	2.62E-06
AP005230.1	4.18214984	2.99E-12	4.96E-11
SNRPGP15	1.153702946	1.37E-08	8.50E-08
DPY19L1P1	1.218876792	1.17E-11	1.65E-10
RPL7AP22	1.636082369	5.60E-08	2.97E-07
SLC25A13	1.012802333	2.15E-09	1.64E-08
UTP14A	1.06939413	5.45E-17	7.52E-15
CEP55	2.304792936	3.85E-17	6.26E-15
AC036103.1	1.235358009	9.27E-07	3.65E-06
AC116337.2	1.230495731	0.000131403	0.000308634
AC092118.2	1.089259439	0.00017434	0.000398786
AC013489.3	1.238792548	4.55E-07	1.94E-06
CDC20	1.679091618	5.67E-12	8.77E-11
OR52K3P	1.714435505	5.83E-06	1.89E-05
HROB	1.754626861	1.19E-14	4.21E-13
AL589765.3	1.388309569	0.000849531	0.001664206
CYP21A1P	1.090986116	0.019860054	0.028628479

RDM1P5	1.833066746	7.23E-11	8.23E-10
AL355598.1	1.38063916	0.00022952	0.000510413
ATP11A	2.288601022	6.65E-19	1.26E-15
AL138693.1	1.466347821	3.31E-08	1.87E-07
H3-4	1.909235869	1.86E-06	6.77E-06
LINC01694	2.940972992	0.000129707	0.000305136
RNA5SP435	2.38130569	2.26E-05	6.40E-05
AC135048.4	1.490649141	3.02E-12	4.99E-11
ATP8B3	1.213427653	0.000123266	0.000291321
C2orf92	1.165261343	1.44E-06	5.40E-06
PGM5	-2.465200822	2.87E-11	3.65E-10
ARMCX1	-1.186108747	3.49E-06	1.19E-05
GNMT	-1.296268511	0.010173729	0.015671166
RSPH14	1.440479371	0.000696356	0.001389062
AC087257.2	1.175241316	0.016999892	0.02489156
IGLJCOR18	-3.992079865	0.013162234	0.019748169
ZRANB3	1.02677744	6.12E-12	9.38E-11
TRIM24	1.083776802	1.37E-09	1.10E-08
HOXC-AS2	5.148544586	5.63E-18	2.15E-15
LGALS9B	-2.370842755	8.26E-07	3.29E-06
TFEC	1.369493855	1.62E-06	6.00E-06
AC006460.1	1.648048394	2.69E-08	1.55E-07
AC083982.1	1.016558664	0.003510415	0.005985949
ZNF774	1.334135752	2.28E-10	2.28E-09
AC087442.1	2.306329172	5.87E-06	1.90E-05
AL590282.1	1.693390374	6.81E-08	3.54E-07
AL132780.2	1.155611478	4.21E-08	2.31E-07
AC100782.1	3.481232481	0.000116721	0.000277353
AC005670.1	1.815882916	2.78E-05	7.68E-05
AC078962.1	1.12937813	0.001202107	0.002274104
PARPBP	1.801791116	7.81E-15	3.00E-13
BNIP3P17	2.123976623	1.62E-05	4.75E-05
AP001347.1	-1.522620338	0.000303212	0.000655187
SNORA80D	1.448253177	2.63E-06	9.27E-06
AC120498.10	-1.294494226	0.000217538	0.000485926
AC007879.4	1.098499168	0.026889297	0.037607468
AC091185.1	1.136566373	1.09E-05	3.32E-05
PRMT3	1.07407466	8.83E-15	3.31E-13
SNORA79	1.960667662	0.000904906	0.001763608
HIPK4	-1.321476652	0.000258942	0.000568717
NDC80	2.187437047	9.02E-17	1.03E-14

AC129507.1	-1.296608504	2.16E-12	3.72E-11
AC018644.1	1.227471862	2.04E-06	7.38E-06
AP005119.2	2.450727576	0.003149154	0.005429
LINC02806	4.072912076	0.000335721	0.00071882
KCNMB3	1.572390612	2.19E-08	1.29E-07
SNRPCP5	1.404437673	0.000222784	0.00049648
ATP6V0D2	1.868076136	4.64E-08	2.52E-07
APOBEC2	-1.76065108	1.32E-08	8.22E-08
IGLV5-37	-2.327139103	0.015833012	0.023334796
PGBD5	2.00213197	7.49E-05	0.000186464
AP002761.1	1.016549119	0.009895523	0.015285049
SLC5A4-AS1	2.813041166	1.49E-07	7.11E-07
SERPINH1	1.690917193	4.65E-17	6.92E-15
OTUD6B	1.077315355	4.65E-12	7.37E-11
F2	4.214066361	0.002098031	0.00375593
MIR3942	1.597302016	0.000524238	0.001075193
RNU6-742P	1.323497654	0.001691647	0.003094539
AL137802.2	2.184067315	9.07E-08	4.57E-07
HMGB1P9	1.714246463	2.31E-09	1.75E-08
KRT18P28	1.348531748	1.51E-07	7.18E-07
UGT1A10	-1.273498494	0.003024255	0.00523366
TNS1	-1.454844569	0.000385364	0.000814444
TEF	-1.22815993	1.60E-09	1.26E-08
ELFN1	1.014616306	5.13E-07	2.16E-06
MYBL2	2.395784215	1.25E-14	4.40E-13
AC092960.2	1.608174782	5.69E-05	0.000145508
RN7SL419P	1.56237332	0.000601871	0.001216251
AP002982.1	1.102302384	4.06E-06	1.36E-05
AC140479.2	2.852029331	4.16E-12	6.67E-11
GRM2	1.633840775	3.83E-11	4.69E-10
AC018638.3	1.209437564	0.004667119	0.007739893
AC103702.2	1.921921192	1.97E-09	1.52E-08
TNFAIP6	1.352432336	0.001276812	0.002400115
WDR43	1.26246716	5.01E-19	1.26E-15
CATSPERB	2.562692633	7.22E-10	6.32E-09
RNA5SP18	2.955814299	6.72E-08	3.50E-07
AC087284.1	1.465506143	2.95E-08	1.68E-07
IGHV7-27	-2.522997531	0.002659492	0.00465261
AL021392.1	1.289187024	1.47E-06	5.51E-06
TMEM68	1.073342721	3.99E-15	1.76E-13
AC136475.8	1.73107898	2.37E-11	3.09E-10

MBNL1-AS1	-1.496814343	0.001931335	0.003483019
ATAD5	1.920194501	2.00E-17	4.19E-15
PSMC2P1	1.130319981	3.35E-06	1.15E-05
ANAPC1P4	1.940069525	1.10E-14	3.97E-13
AL022238.1	1.175236598	7.90E-05	0.000195269
RGS9	-1.18833142	3.72E-08	2.07E-07
HMMR	2.18468986	1.74E-15	9.02E-14
AL158801.3	1.170485117	0.001448927	0.002688745
RNASEH2B-AS	1.320589696	1.75E-08	1.06E-07
ADAM10	1.156024517	1.40E-15	7.59E-14
KRT18	1.297632942	4.14E-08	2.27E-07
HOXC12	8.920969326	6.79E-09	4.54E-08
LINC02608	-1.183130835	0.001427655	0.002653338
RN7SL105P	1.161495912	0.005486095	0.008954819
LHX4	1.549426782	1.39E-12	2.54E-11
XAF1	1.263275164	6.77E-08	3.52E-07
LINC01730	1.860117755	9.17E-08	4.62E-07
AC129507.3	1.59366717	4.06E-07	1.75E-06
AC083843.2	1.379426456	3.06E-10	2.97E-09
AC004870.2	3.382650902	2.73E-05	7.56E-05
RPS24P17	1.039635997	0.000106369	0.000255364
AC133555.4	1.543845796	0.000304199	0.000657136
ARNTL2-AS1	1.017167796	0.001298572	0.002435519
TUBB4A	-1.310605205	0.001400603	0.002608885
BGN	2.909669059	1.16E-16	1.22E-14
SPACA4	1.165300838	0.000120159	0.000284585
AC090589.2	1.564754151	2.61E-05	7.26E-05
AC135279.2	1.623395803	2.44E-09	1.83E-08
CCDC15	1.005106473	5.51E-09	3.76E-08
H3C13	1.040987901	0.000995532	0.001924605
ZMIZ2	1.070354904	2.51E-16	1.98E-14
KIF26B-AS1	3.831236536	1.27E-14	4.46E-13
RPL36AP39	1.365418826	3.63E-07	1.58E-06
AC102945.1	1.692408853	4.28E-08	2.34E-07
AC006270.1	1.570434206	0.000227262	0.000505867
AC020907.4	1.312502328	2.74E-07	1.23E-06
IGHV3-50	-2.746394195	0.013009467	0.019535465
MRPL20-DT	1.470453345	6.87E-14	1.85E-12
GINS1	2.267447544	1.43E-16	1.40E-14
AL133260.1	2.42914225	1.65E-08	1.01E-07
COL6A3	1.245822492	5.84E-07	2.42E-06

TRRAP	1.086270097	1.59E-12	2.84E-11
AC008687.2	1.171196458	0.016808375	0.024634653
ITGAD	1.497063114	1.01E-05	3.10E-05
AC099811.3	1.204060238	2.57E-06	9.07E-06
LFNG	1.031392437	1.15E-05	3.49E-05
AL021920.2	2.150531897	6.92E-06	2.20E-05
AC007601.2	1.289738613	0.000293978	0.00063754
AC004836.1	-1.610555763	0.007434218	0.011797155
FRRS1L	-2.143232025	6.03E-07	2.49E-06
HMGA2	4.47019708	1.47E-13	3.55E-12
CTSV	1.981214358	2.20E-07	1.01E-06
OLR1	3.398129272	8.87E-16	5.36E-14
AC023794.2	-1.014806568	5.71E-05	0.000145971
HOXC13-AS	4.601817684	1.74E-05	5.05E-05
AC079949.2	2.420035037	0.001242374	0.002343149
AC013470.2	2.62671712	1.47E-08	9.06E-08
HMGA1P8	1.274479957	2.95E-08	1.68E-07
LRRC37A15P	1.328171385	7.40E-10	6.46E-09
RNA5SP247	1.913703333	1.36E-07	6.54E-07
C9orf43	1.163381997	2.85E-08	1.63E-07
COL27A1	1.656311904	4.32E-09	3.03E-08
AC022973.1	1.794362163	0.00085127	0.001667322
YRDCP1	1.748979346	1.89E-07	8.82E-07
AC093520.2	2.221705979	4.14E-09	2.92E-08
AC145207.8	1.020910365	7.15E-07	2.89E-06
RASGEF1C	-1.472618748	1.86E-06	6.78E-06
EIF1AX-AS1	1.605919807	1.41E-07	6.79E-07
AL132642.1	-1.341978344	6.19E-05	0.000156961
RNF144A	1.033425492	6.38E-07	2.62E-06
MTATP6P27	1.452096061	0.000177044	0.000404355
SNRPEP10	1.739849833	1.14E-06	4.38E-06
LINC00954	1.90713797	0.000181083	0.000412702
CASC9	3.197471744	2.22E-09	1.69E-08
RNU6-79P	1.990835015	0.000577511	0.001172157
PKHD1L1	-1.498212424	6.33E-12	9.65E-11
AC108751.3	2.168925997	1.19E-07	5.81E-07
AP000442.1	1.013477538	4.44E-08	2.42E-07
AL357054.4	-1.732458889	1.39E-05	4.13E-05
FOXC2-AS1	3.144333735	6.83E-05	0.000171481
FGF10	-1.065280828	6.11E-05	0.000154997
MFSD13A	1.170778816	3.56E-13	7.70E-12

GAPDHP51	1.163103411	0.000302705	0.000654388
AP001107.5	-2.95785858	3.28E-10	3.14E-09
AL160275.1	1.062294578	0.000874449	0.001708999
ERCC8-AS1	1.765544522	5.19E-07	2.18E-06
ENTR1	1.044074557	2.86E-16	2.18E-14
AL445686.1	1.086418496	0.002168431	0.003870776
TRAF5	1.104375673	2.15E-08	1.27E-07
ZFPM2-AS1	2.603548075	2.99E-05	8.18E-05
AL606834.1	1.037190212	3.26E-06	1.12E-05
AC007919.2	1.345282727	2.37E-05	6.66E-05
RNU2-37P	1.083417031	0.015635209	0.023091814
SNORD101	1.431226879	2.11E-09	1.61E-08
MT1X	-1.550106643	1.59E-10	1.66E-09
AC090912.3	3.539931281	1.61E-11	2.19E-10
KRAS	1.297854341	1.04E-06	4.04E-06
FAM215B	1.197917156	7.44E-05	0.000185401
DDX47	1.156675337	8.51E-12	1.26E-10
AC007036.2	1.06529524	0.000346668	0.000740057
AC007216.2	1.248051649	2.11E-07	9.72E-07
CDRT15	1.448999948	1.99E-07	9.22E-07
GAS1	-1.327778064	0.001070773	0.002053375
SYCE2	1.280978505	1.54E-05	4.52E-05
KRTAP4-1	6.691312502	3.82E-08	2.12E-07
AC023024.1	1.028803341	2.59E-06	9.14E-06
SNCG	-1.098359155	1.12E-09	9.25E-09
CDK2	1.162529853	6.61E-16	4.22E-14
RPL7P14	1.083280795	7.47E-05	0.000186004
MAPK10	-1.468220803	1.06E-07	5.27E-07
AC010538.1	1.422592677	5.93E-08	3.13E-07
P4HA3	2.473867273	1.83E-15	9.35E-14
GGT6	-1.101583287	0.000667122	0.001335447
IMM23B-AGAF	1.016772907	1.37E-06	5.16E-06
AC073136.1	1.566112725	2.92E-10	2.85E-09
MTHFD1L	2.208020404	3.38E-20	4.50E-16
AC007431.3	1.630650269	0.000122797	0.00029047
AC005796.1	-1.056329798	0.032454547	0.044480376
NKX2-3	-1.217658682	0.000219449	0.000489867
RNA5SP319	2.420276869	1.29E-05	3.87E-05
RPL11P1	1.555197864	1.31E-05	3.91E-05
AC078883.3	2.329504555	9.33E-09	6.06E-08
ZNF367	1.834543157	3.33E-17	5.87E-15

AL158835.2	1.203269519	0.000269302	0.000589591
RPS12P17	1.021077041	2.21E-05	6.26E-05
LINC02533	1.99682451	6.62E-05	0.00016683
HSPE1P4	1.799436653	1.95E-11	2.60E-10
MIR4435-2HG	2.306790291	2.89E-19	1.26E-15
PIWIL1	6.530786696	2.30E-12	3.94E-11
MRTO4	1.093044008	2.54E-15	1.22E-13
FBP2	-2.642621687	0.034623052	0.04715585
CRYBB2	1.490840845	0.002372769	0.004198679
GHR	-1.808369591	5.04E-11	5.96E-10
CCL3L3	1.89904269	1.55E-07	7.38E-07
IGLV3-22	-1.113862727	0.019507448	0.028161335
AC002398.2	-2.275471612	5.73E-06	1.86E-05
AC021739.1	1.302185813	0.000252815	0.000556377
ARL4AP5	1.754383237	1.28E-10	1.38E-09
AC007215.1	1.816121022	1.05E-08	6.69E-08
ZNF114	2.372600229	8.84E-06	2.74E-05
TMPO	1.30190426	8.10E-15	3.09E-13
HPX	2.200872605	8.04E-06	2.51E-05
RNY1P13	1.596896962	3.34E-06	1.15E-05
ILF2P1	1.459523773	4.10E-08	2.26E-07
AC113139.1	1.182314642	9.16E-06	2.83E-05
CPNE6	-1.411576715	1.83E-08	1.10E-07
PYDC1	1.662905264	0.035542253	0.048294828
MIR3177	1.03785949	0.002154578	0.003848885
ZNF731P	1.229306383	1.76E-12	3.11E-11
AFF3	-1.426686039	1.94E-10	1.97E-09
RNA5SP288	1.005917461	0.007187336	0.01144059
AC097639.2	2.50540709	1.09E-06	4.22E-06
MIR101-2	1.403897412	0.003773523	0.006393679
TBILA	1.594068011	7.04E-08	3.65E-07
AC098591.1	1.229151219	0.00555185	0.009053274
AC244197.2	1.426422801	5.64E-06	1.83E-05
MIR3161	1.554958737	8.05E-05	0.000198659
AL355862.1	1.9207388	0.029809168	0.041243033
SPEF1	1.272726421	0.001106825	0.002112454
RNU6-969P	1.155531855	0.004165655	0.006988752
MIR3648-2	2.357834698	0.00335659	0.005748667
AC009318.1	1.365259436	3.69E-07	1.60E-06
UHMK1	1.105675146	2.03E-14	6.70E-13
AC027373.1	1.033543001	3.74E-05	0.000100085

AC090739.1	1.359321226	2.90E-09	2.13E-08
POU5F1P4	1.359123742	1.24E-07	6.03E-07
FCGR2A	1.582316994	9.14E-10	7.74E-09
AC012568.1	1.016646663	0.003207584	0.005518304
AC007663.3	1.009804355	0.000134745	0.000315678
PPIAP19	1.00007942	1.85E-07	8.66E-07
SAA4	3.236144794	2.27E-05	6.42E-05
AL032819.1	2.315539108	4.41E-07	1.89E-06
RN7SL370P	1.34504678	0.000197113	0.000444288
IGKV1D-37	-3.874889816	0.001161536	0.002206778
POC1B-AS1	1.055219844	7.36E-10	6.43E-09
FAM72A	2.482907372	1.92E-17	4.09E-15
PPIAP50	1.468811293	1.52E-07	7.24E-07
RNU6V	1.257316543	9.46E-05	0.000229824
APOC2	1.15938328	0.000293299	0.000636118
APOC1	2.774555391	6.06E-13	1.22E-11
AC104843.1	1.18486987	9.26E-06	2.86E-05
LRRC10B	-1.925606016	0.011795484	0.017888955
URB2	1.574027813	9.61E-18	2.94E-15
RPS15AP30	1.300637882	1.03E-06	4.01E-06
AL133153.2	1.413484778	1.03E-06	4.01E-06
RN7SKP185	1.339502128	0.000296883	0.000643346
RN7SL809P	2.028947734	4.33E-11	5.23E-10
MIR3186	1.240092609	0.010145355	0.015637404
APELA	3.376098357	4.84E-11	5.78E-10
FNDC3B	1.047602715	4.81E-13	9.99E-12
AC004980.2	1.43496597	2.61E-07	1.18E-06
AC100843.1	2.085599336	1.98E-07	9.15E-07
AP001350.2	1.072697885	8.93E-08	4.51E-07
RN7SL764P	1.355257342	0.008253238	0.012967079
FAM86JP	1.280213058	6.26E-12	9.56E-11
ZNF280C	1.210814028	4.05E-13	8.57E-12
DIO1	2.046810276	0.019378504	0.027992146
HNRNPA3P5	1.045170781	2.85E-08	1.63E-07
AC007216.4	1.075718865	4.31E-06	1.44E-05
FOXD2-AS1	2.717453603	3.30E-18	1.71E-15
AC114730.3	1.189095802	4.54E-06	1.51E-05
AC018410.1	1.065018659	2.70E-05	7.49E-05
AC117503.4	1.131332128	1.57E-07	7.46E-07
TYRP1	1.627451879	0.006776401	0.010846317
AL645608.7	1.435669192	2.90E-08	1.66E-07

AC135068.5	-3.516351865	0.001668858	0.003055578
HSPB6	-2.441128118	2.13E-08	1.26E-07
AC006330.1	1.638568292	2.30E-10	2.30E-09
AC103957.2	1.4178878	0.003532576	0.006019885
:CDC144NL-AS	2.86243783	2.61E-07	1.18E-06
AL031432.2	1.244442948	0.000118139	0.000280472
HSPE1P7	1.856152532	2.75E-09	2.04E-08
AC007731.3	1.273476335	3.41E-05	9.21E-05
NEFL	-2.720637393	3.49E-05	9.41E-05
HMGN2P7	1.132916867	4.85E-06	1.60E-05
AC010168.1	1.260433959	2.84E-06	9.94E-06
PGF	1.47010561	6.54E-12	9.93E-11
AC006504.7	1.397893562	1.91E-05	5.50E-05
VCX	6.588613304	2.97E-05	8.14E-05
LMO3	-2.095335634	0.019788339	0.028528189
AC016575.1	1.558054642	9.02E-05	0.000220315
AP000346.4	1.663000767	0.001939534	0.003496621
IL2RA	1.414879546	6.71E-08	3.50E-07
HOXB6	1.246788698	3.92E-05	0.000104485
C20orf85	1.1824807	7.22E-05	0.000180312
RHPN1-AS1	1.963608394	3.34E-11	4.14E-10
SLC5A7	-3.061862051	1.68E-09	1.32E-08
SSB	1.071056408	5.06E-15	2.12E-13
AL117382.1	2.714523123	1.51E-10	1.59E-09
AC016877.3	1.740317512	2.89E-06	1.01E-05
SLC35F1	-1.357289103	0.007169851	0.011414352
ACTL8	7.318669473	1.39E-09	1.11E-08
OSGIN1	-1.452616987	6.08E-07	2.51E-06
TCF3	1.057878643	6.97E-11	7.97E-10
ASB5	-2.471548988	2.03E-06	7.32E-06
RNU7-186P	-1.417522298	0.031045292	0.04272697
GTF3C6P3	2.296933352	2.47E-09	1.86E-08
TC2N	1.119168456	4.72E-07	2.00E-06
LINC02889	2.673312136	3.47E-05	9.36E-05
RNU6-353P	1.094624251	0.002035773	0.003654614
RNU6-1255P	2.16405168	0.000486577	0.001005927
UTP25	1.259011281	1.20E-18	1.31E-15
AC106800.3	1.093749363	5.14E-06	1.68E-05
FMNL2	1.414540061	6.99E-15	2.74E-13
AC009237.8	1.330078658	1.86E-05	5.36E-05
GBX2	3.625753879	3.30E-06	1.13E-05

ADORA2A-AS1	1.547325662	0.012605288	0.018987896
RASA2-IT1	1.442489727	1.64E-06	6.08E-06
YJEFN3	1.28676239	1.24E-07	6.03E-07
OGN	-1.033643783	3.85E-06	1.30E-05
KLHL17	1.144955948	2.88E-10	2.81E-09
RTN4R	1.401412959	1.92E-09	1.48E-08
AC133065.1	1.091385976	0.003134648	0.005405741
AC092802.2	1.228140738	7.41E-07	2.99E-06
COL26A1	2.751170907	0.01179441	0.017888955
MIR644A	2.055798731	1.53E-06	5.70E-06
AARD	-2.115050525	4.93E-06	1.62E-05
BMP5	-1.138189879	4.09E-08	2.26E-07
AL442067.1	1.510852932	1.08E-07	5.36E-07
LINC00677	2.111906435	1.80E-07	8.42E-07
MIR4312	1.771566405	6.69E-06	2.13E-05
BOP1	1.562943744	5.18E-16	3.53E-14
AC083906.1	2.615390238	1.02E-08	6.53E-08
UBE2SP1	1.810466128	1.88E-13	4.42E-12
SSBL4P	1.598125732	4.15E-08	2.28E-07
AANAT	1.128430394	8.59E-05	0.000210746
AC005014.1	1.073897	1.02E-05	3.12E-05
RNU6-759P	1.408777931	5.90E-06	1.90E-05
GPRIN3	1.271473388	6.89E-06	2.19E-05
H1-3	1.182657762	0.000387662	0.00081904
AC091153.2	1.590712142	7.45E-07	3.00E-06
RN7SL20P	1.873996986	5.80E-05	0.000147955
RNU6-407P	1.194640361	8.23E-05	0.00020279
AP001992.1	1.304911906	5.16E-08	2.76E-07
AC023271.2	1.813001373	1.50E-08	9.25E-08
EPHA7	-2.621721946	8.82E-05	0.000215862
MHENCR	1.131261134	2.57E-10	2.55E-09
THAP5P1	1.37550339	4.41E-06	1.47E-05
AC060780.1	1.467169128	3.06E-10	2.97E-09
NOTUM	5.414003657	0.020888993	0.029977083
NOP58	1.324681545	2.48E-17	4.99E-15
CXCL9	3.443237622	4.08E-11	4.97E-10
PDZD4	-2.030870256	3.10E-09	2.26E-08
LRRC8D	1.063194955	2.08E-14	6.83E-13
AC099811.1	1.148994346	8.19E-07	3.27E-06
RN7SL192P	1.265075038	0.00019652	0.000443102
AC027514.2	1.134895713	2.04E-08	1.21E-07

AC100778.2	1.24888256	1.81E-08	1.09E-07
AC084864.1	2.306033693	2.50E-05	6.99E-05
RPL31P35	1.38217662	1.88E-05	5.41E-05
SMYD5	1.017669414	6.06E-17	7.88E-15
MTND5P14	1.04873414	0.002089647	0.003742888
MIR4698	2.082719045	0.000208171	0.000466842
TPM3P9	1.643654262	1.43E-14	4.96E-13
REPIN1-AS1	1.22325561	1.38E-09	1.11E-08
RPL39P33	1.353960952	0.001824991	0.003309606
AC006927.1	1.090810565	0.000197182	0.000444406
PPIAP41	1.003684979	3.95E-05	0.000105112
HNRNPA3P2	1.37052657	7.68E-05	0.00019072
SAA2	3.396887798	0.000737218	0.001463013
AC009318.4	1.090154244	0.001289976	0.002422125
CXCL6	3.03337399	3.59E-09	2.57E-08
RN7SL23P	1.483398497	8.52E-07	3.39E-06
ALG10	1.18558118	1.20E-13	3.00E-12
STX8P1	2.719215159	2.82E-07	1.26E-06
PINX1	1.01946721	8.97E-10	7.61E-09
AC090709.1	3.44747397	4.27E-05	0.000112528
GCNT4	-2.254637148	4.26E-12	6.80E-11
AL355802.2	2.142844522	5.91E-08	3.12E-07
AC104819.1	1.069388588	2.04E-05	5.83E-05
RPL23AP67	1.059007011	0.004531444	0.007529891
ZBTB12	1.023527708	6.81E-07	2.77E-06
AL121992.3	1.391713707	3.72E-08	2.07E-07
LINC02883	1.620731632	4.35E-08	2.38E-07
RGS2	-1.593590636	2.90E-09	2.13E-08
NRXN1	-2.59072176	3.20E-11	3.99E-10
NR2F1-AS1	-1.120934444	0.000670955	0.001342111
AC012435.2	1.348936962	1.55E-09	1.23E-08
SEMG2	4.294093281	2.81E-05	7.74E-05
SMG7-AS1	1.008511669	1.30E-09	1.05E-08
AC244205.1	-2.117283494	3.39E-05	9.15E-05
RPL32P28	1.520966486	0.00160763	0.00295342
5S_rRNA	1.411520418	9.60E-05	0.000232822
AC243732.1	1.036423096	6.67E-10	5.89E-09
AC092718.6	1.477502974	1.55E-09	1.23E-08
AC011700.1	3.319437685	3.12E-05	8.51E-05
AC084876.1	1.310009424	1.33E-06	5.05E-06
AC068134.2	4.647161102	7.41E-06	2.33E-05

LINC02604	1.635782604	1.46E-11	2.01E-10
PNPLA3	2.053171614	0.002381771	0.004211813
PAK1IP1	1.128975776	1.46E-14	5.07E-13
NSUN5P1	1.308313546	2.02E-11	2.69E-10
AC108865.1	3.381990494	0.026556805	0.037202986
HNF1B	1.801500836	2.12E-10	2.14E-09
AC012676.4	1.250619572	2.11E-05	6.00E-05
HMGB3P14	1.175305374	9.86E-05	0.000238578
SLC26A6	1.122201423	2.34E-08	1.36E-07
RPL23P10	1.683549798	0.000263267	0.000577614
MAGI1-AS1	1.025459716	0.005994535	0.009702109
EPHB4	1.434016079	3.70E-15	1.65E-13
RNVU1-4	1.624045203	6.84E-05	0.000171804
AL031710.2	1.72295995	1.48E-10	1.56E-09
LPCAT1	1.428635122	2.73E-12	4.58E-11
BMS1P8	1.64994203	0.015769012	0.023262348
SUPT16HP1	1.105156967	1.86E-10	1.91E-09
LINC02762	-1.226336991	1.78E-06	6.51E-06
ACADL	-1.470394381	2.73E-10	2.69E-09
AC013468.1	1.035978099	0.000174351	0.000398786
TREML2	1.373254404	0.018117188	0.026375884
MIR647	1.685396959	1.23E-10	1.33E-09
AL157827.1	1.80789228	1.81E-05	5.23E-05
TAC1	-1.890395465	0.000120023	0.000284439
MEIS1	-1.193144356	0.003305772	0.005674766
AC009486.1	1.26382955	0.000355715	0.000757914
IGHV3-71	-3.60563366	0.022833055	0.032483486
UTP6	1.010977923	1.65E-17	3.70E-15
RN7SL381P	1.460526546	3.37E-09	2.43E-08
STMN2	-1.902664911	0.000182174	0.000414963
ANLN	2.421700876	1.61E-17	3.68E-15
ARAP3	1.333351648	9.18E-12	1.34E-10
NEIL2	1.203700964	4.81E-08	2.60E-07
AC010301.1	1.188925115	0.001292831	0.002425607
RETN	1.389670192	0.000711185	0.001415463
AC066613.1	1.390983789	2.70E-09	2.00E-08
AC091887.1	1.47073042	3.98E-07	1.72E-06
RNU7-193P	1.156238519	0.005661194	0.009213533
ADAMTS18	3.650547576	2.30E-13	5.27E-12
SNHG17	1.145820844	4.85E-10	4.45E-09
AMOT	1.035110616	0.00106486	0.002042624

ACLY	1.11461725	9.04E-11	1.00E-09
SOBP	-1.266212669	8.48E-05	0.000208307
MXRA5	2.098936178	5.61E-12	8.68E-11
S100A7	2.220093975	9.20E-05	0.000224285
MPHOSPH6P1	1.624306115	1.36E-07	6.55E-07
AC106827.1	1.091762785	0.006304312	0.010157816
PDLIM3	-1.717418645	0.000190332	0.000431047
UBE2CP4	1.780596379	8.38E-08	4.27E-07
LTF	-2.51640745	0.019788339	0.028528189
AP005264.4	1.629052013	2.09E-05	5.97E-05
IFITM3P9	1.167341886	0.000186972	0.000424303
AC036214.3	1.055744479	3.15E-06	1.09E-05
PBK	2.219014091	3.00E-14	9.21E-13
AC009646.2	3.921291002	0.000606135	0.001223938
C17orf77	2.481200346	0.014811339	0.021978596
RNU6-998P	1.14408874	0.005876719	0.00952995
S100A7A	3.627548951	0.004148378	0.006962835
PLAU	2.363626031	2.81E-15	1.32E-13
IGHV7-56	-3.088766121	0.004093208	0.006881942
VPS9D1-AS1	2.188259342	1.51E-12	2.71E-11
LINC01348	1.879023712	8.05E-05	0.000198727
AQP9	1.697546224	0.000243604	0.00053833
IGKV1OR22-5	-3.334603773	0.005485457	0.008954325
HOXC11	4.86417197	8.21E-16	5.04E-14
SRMP2	1.752498626	1.66E-06	6.12E-06
SPATA46	1.417667684	1.20E-06	4.57E-06
AC005288.1	1.024466446	6.31E-14	1.73E-12
ADHFE1	-2.027302752	6.95E-13	1.37E-11
APLNR	1.180649673	3.77E-05	0.000100717
AC116348.2	1.875049037	0.000412294	0.000865454
SLC51B	-2.449041181	0.000348194	0.000742781
KIAA1549	1.728912584	6.54E-12	9.93E-11
PTMAP2	1.046958843	1.00E-08	6.43E-08
PRIM1	1.248836994	9.57E-13	1.82E-11
AC068189.1	1.730701062	1.33E-06	5.05E-06
SLX4IP	1.452552261	1.45E-16	1.40E-14
TYMP	1.381760406	1.43E-07	6.87E-07
TMEM89	1.910760017	3.10E-07	1.37E-06
AC131097.1	3.781638744	9.68E-07	3.79E-06
AC012213.3	1.493875284	1.83E-09	1.42E-08
MIR1972-1	1.127046432	0.000437477	0.000913351

AC005921.3	1.144023644	1.21E-06	4.62E-06
DSG2	1.257612139	3.69E-08	2.05E-07
LINC00189	1.544562342	5.78E-05	0.000147535
RNU6-1316P	2.330209766	7.96E-07	3.19E-06
IGHV1OR16-1	-3.868718702	0.020367756	0.029296931
SLC28A2	-1.730011169	0.010312486	0.015861979
AC022400.2	1.231923102	8.03E-06	2.51E-05
DKC1	1.626529958	7.76E-19	1.26E-15
TLR2	1.452516814	3.85E-10	3.62E-09
TERT	3.412831676	1.04E-09	8.65E-09
POU5F1B	2.486325659	5.11E-08	2.74E-07
GUCY1B2	3.692276646	2.35E-14	7.54E-13
SLITRK5	-1.273357387	0.000297722	0.000644618
SAPCD2P3	1.517018922	7.64E-08	3.93E-07
AC135068.1	-3.340608018	0.019445957	0.028080173
MIR657	2.891660133	7.70E-08	3.96E-07
RPL36AP16	1.378097915	5.93E-07	2.45E-06
AC106865.1	2.767804484	4.45E-10	4.13E-09
AL513497.1	1.208110012	4.31E-06	1.44E-05
YBX2	1.49701476	0.000133893	0.000313902
SNORD11	1.166852246	1.62E-06	6.01E-06
PIMREGP1	1.231848251	2.36E-05	6.64E-05
ABCA8	-1.525605734	2.30E-10	2.30E-09
SMIM10L2B	-1.176642705	1.99E-09	1.53E-08
HPN-AS1	1.738824221	7.76E-06	2.44E-05
RRH	1.06997427	0.000358474	0.000763121
AC023389.2	1.70881573	4.57E-10	4.23E-09
MYADML2	2.131615827	3.89E-05	0.000103849
HSPE1P9	1.44465363	6.14E-05	0.000155888
SAPCD1	2.542949661	5.24E-13	1.08E-11
LRRC4	-1.690021261	1.58E-08	9.66E-08
TOMM34	1.476256213	4.49E-16	3.15E-14
MUC12	3.065660695	3.56E-06	1.21E-05
LRPPRC	1.032112004	9.99E-15	3.69E-13
MINAR1	1.592313374	8.98E-12	1.32E-10
AL592437.1	1.327481445	1.80E-07	8.41E-07
TAGLN	-1.855227506	3.36E-06	1.15E-05
ANKEF1	1.249335004	6.91E-12	1.04E-10
UFM1P3	1.641101917	0.003967684	0.006690743
MIR3945HG	2.034760621	9.49E-08	4.76E-07
E2F3P1	1.273296165	0.003349857	0.005742673

NDRG2	-1.083605923	6.99E-15	2.74E-13
TAC3	-2.070443971	0.006905293	0.011036121
AP001429.1	1.502134024	1.06E-09	8.78E-09
CSMD2	1.913323637	3.19E-09	2.32E-08
AL121583.1	1.340183702	7.20E-06	2.27E-05
AC104984.2	1.472986662	1.23E-06	4.70E-06
TAFA4	-3.901732122	1.94E-05	5.58E-05
KRT75	2.703736985	0.015761029	0.023251859
AL589743.3	3.686674927	1.02E-07	5.09E-07
PPIAP58	2.156461863	5.21E-11	6.15E-10
LINC02119	5.585540782	0.003586676	0.00610271
RNA5SP311	1.055565937	2.22E-05	6.30E-05
COA7	1.120722054	1.81E-16	1.61E-14
PFN1P11	1.039737513	0.002546507	0.004473136
SLC25A32	1.104821635	5.31E-17	7.48E-15
MIR3192	1.907068022	6.45E-07	2.64E-06
AC093019.1	2.026127149	8.46E-07	3.37E-06
LINC01594	3.267867637	2.52E-06	8.90E-06
AC060766.1	1.065793636	6.26E-06	2.01E-05
MIR548I1	1.838027652	0.00660099	0.010592277
CHDH	1.453662039	5.80E-10	5.23E-09
RNU6-212P	1.264008707	0.00019555	0.000441287
AC020978.1	1.470968599	8.29E-07	3.31E-06
CKLF-CMTM1	1.463505175	4.24E-13	8.92E-12
MMP14	1.36833971	2.36E-12	4.03E-11
AC108860.2	1.713338752	1.22E-11	1.72E-10
PTGER3	-1.105005683	2.55E-05	7.11E-05
CAPN10-DT	1.45028624	1.97E-12	3.43E-11
MIR6812	2.326823535	1.71E-06	6.29E-06
TMEM132C	-1.562629147	3.16E-13	6.95E-12
SNORD12B	2.493353583	8.05E-15	3.08E-13
CALHM3	4.061897044	2.49E-07	1.13E-06
HSD3BP5	1.954698569	0.009160431	0.014255386
LDHC	1.108987141	0.004514437	0.007507249
SLC12A9	1.127995754	2.79E-13	6.25E-12
OBP2A	4.493550112	4.10E-07	1.77E-06
AC007773.1	1.382164646	1.02E-07	5.07E-07
RAB19	1.13637111	1.38E-07	6.66E-07
OTOP3	-4.533570247	1.83E-05	5.28E-05
RNA5SP28	3.599785547	1.03E-05	3.14E-05
HSPB7	-1.848749232	5.56E-07	2.32E-06

HMGN2P17	2.410256914	6.73E-10	5.94E-09
TKTL1	5.698133853	0.003352951	0.005747238
BUD23	1.000895272	5.32E-15	2.19E-13
RN7SKP78	1.350794302	3.37E-05	9.12E-05
AC090517.2	1.261249481	5.63E-08	2.98E-07
AC008752.1	1.201523863	0.000772557	0.001526323
TMEM132A	1.443574009	1.58E-09	1.25E-08
AP000424.2	1.970968198	4.51E-05	0.000118221
C8orf37-AS1	1.022210488	0.001774103	0.003228216
KLHDC8A	-1.085802357	0.001664114	0.003047521
TAP1	1.230887183	2.57E-06	9.08E-06
MRPS21P9	1.006281095	0.013264134	0.019882022
VEGFA	1.261171457	7.03E-09	4.68E-08
RABGAP1L-IT1	1.47690746	7.49E-05	0.000186464
RPS13P5	1.085148355	0.003458716	0.005905714
AC074121.1	1.347350552	0.002488977	0.004381033
KCND2	1.10999742	0.001746048	0.003183346
MAJIN	1.460217223	0.004764805	0.007884717
AC083906.3	3.16610915	7.85E-09	5.18E-08
LINC01169	1.485432119	0.001940302	0.003497769
AC090912.1	1.180266049	1.75E-05	5.09E-05
KLF4	-1.374098822	4.30E-12	6.87E-11
AC036108.3	-2.163169986	0.011382578	0.017327847
RYR2	-1.536860906	0.000782504	0.001543914
UNC5A	2.097637154	0.008340397	0.013091667
SLC44A3-AS1	1.357313451	3.66E-10	3.46E-09
LINC00336	1.274193258	0.000457494	0.000951488
MEST	2.510275612	6.23E-17	8.05E-15
AIF1L	-1.956430852	0.001058978	0.002032071
AL591516.1	1.750084874	1.94E-06	7.02E-06
AC100827.2	1.006014393	0.000100934	0.000243642
AIM2	1.184371393	0.001700083	0.003107623
BNIP3P25	1.315183307	2.13E-05	6.07E-05
TAF13P2	1.47193611	0.009397455	0.014580804
ANP32E	1.353027249	1.29E-16	1.28E-14
AC090772.2	1.42805478	0.001145463	0.002179193
MROH6	1.438296321	4.18E-07	1.79E-06
DGKI	1.279357645	4.41E-06	1.47E-05
PDHA1P1	1.416198111	2.23E-05	6.32E-05
AC019080.5	1.00241713	5.08E-07	2.14E-06
ASLP1	1.160565233	1.30E-07	6.31E-07

KRT18P11	1.35735935	9.74E-08	4.88E-07
AC091806.1	2.935856127	9.32E-10	7.86E-09
PLIN4	-2.53361368	2.07E-09	1.59E-08
AL357552.2	1.007906184	0.00101803	0.001962829
TLCD4-RWDD3	1.031579886	2.51E-08	1.46E-07
AC015911.3	1.560572453	3.51E-05	9.44E-05
IGHV3-60	-3.261354772	0.017634687	0.025727101
ANKRD10	1.070425622	2.67E-12	4.50E-11
GMPS	1.03053285	1.29E-16	1.28E-14
C20orf202	1.371821486	3.90E-05	0.000103872
AC234782.1	3.094905617	1.12E-06	4.30E-06
AP001273.1	1.294682513	3.25E-10	3.12E-09
AC004623.1	1.486481796	1.61E-09	1.27E-08
RN7SKP9	1.533018062	2.30E-06	8.21E-06
AL355877.1	2.125778	2.16E-08	1.27E-07
GRIK5	-1.767782372	8.37E-05	0.000205877
AC005387.2	1.33351878	3.69E-07	1.60E-06
AP003469.2	1.040489175	0.001035741	0.001992515
RPL30P13	1.244698975	0.000231212	0.000513801
TNNI2	1.569652571	0.000337917	0.000722901
H3C11	1.186788451	0.000315953	0.000680487
PDZD8	1.238942012	5.31E-12	8.28E-11
AL162724.1	1.137646479	0.000285058	0.000619959
KCNN3	-1.217049041	1.53E-06	5.70E-06
TRPM2-AS	3.53283962	5.65E-13	1.16E-11
RN7SL833P	1.057812907	0.00980335	0.015150579
RNU6-1099P	1.427983709	8.42E-08	4.28E-07
AP006748.1	2.981151774	2.56E-05	7.14E-05
PROX1	2.498192549	0.014690255	0.02180985
PRPH	-2.156967185	0.000126377	0.000297958
AL390066.1	1.520300716	1.35E-10	1.45E-09
AC010260.1	1.271796811	1.76E-07	8.24E-07
KLF15	-2.002246136	2.64E-11	3.39E-10
MIS18BP1	1.063322266	2.11E-13	4.89E-12
NANOGP4	1.192121062	0.000395118	0.000833338
HSPE1P13	1.128506252	0.001303986	0.00244515
AL035420.1	1.558723755	8.40E-05	0.000206449
AC012254.5	1.82169685	3.23E-05	8.76E-05
BNIP3P24	2.014496503	7.65E-06	2.40E-05
AL513175.1	1.510939948	2.57E-11	3.32E-10
DCAF12L2	-1.529183074	3.68E-06	1.25E-05

MYO16-AS1	2.983685403	0.001867299	0.003379429
GPM6A	-2.549182609	8.58E-11	9.58E-10
SRSF10P1	1.284386959	3.46E-05	9.32E-05
SLC22A9	1.994260292	0.000508458	0.001046614
AC007601.1	1.369962444	3.13E-06	1.08E-05
LINC02398	2.240519493	0.006436542	0.010352713
AL359878.2	1.622847134	6.67E-06	2.13E-05
AC008649.2	1.124587467	0.000172793	0.000395595
TEDC2	2.020228352	6.81E-15	2.69E-13
CCT4P2	1.440820484	0.000308459	0.000665567
AC087612.1	4.855271756	0.002638954	0.004620901
AP000763.3	1.590825715	1.78E-05	5.14E-05
ASF1B	1.588372991	8.93E-13	1.71E-11
PNMA2	1.434042519	0.006618276	0.0106149
RNU6-1152P	1.712238877	0.001418406	0.002638725
NR2C1	1.055384929	1.80E-14	6.05E-13
CTAGE7P	1.325438266	5.80E-10	5.23E-09
AC090772.4	1.051370314	6.54E-06	2.09E-05
RNA5SP123	1.331835204	2.80E-05	7.72E-05
AC016394.1	1.725418238	9.75E-15	3.61E-13
AC138393.1	1.741582581	0.000168087	0.00038565
AC004584.2	1.455438968	5.50E-07	2.30E-06
WSCD2	-2.137024861	1.06E-08	6.75E-08
IGLV7-43	-3.007907038	0.024071284	0.034046886
MIRLET7A1	-1.554064169	0.002678788	0.004682958
GMPSP1	1.430465287	3.82E-09	2.71E-08
PLSCR1	1.034437124	3.26E-12	5.35E-11
AP003086.1	1.749755268	1.19E-07	5.83E-07
AP002807.1	1.496584958	4.12E-12	6.61E-11
AC079209.1	1.424242268	6.49E-07	2.65E-06
AC068888.2	1.152192063	6.49E-07	2.65E-06
AC004594.1	1.089895721	0.000461501	0.000959222
AC099489.1	1.687928028	1.62E-06	6.02E-06
NOC2LP2	1.538030742	7.78E-07	3.12E-06
AC016717.2	2.896533376	0.005586037	0.009101223
AC025031.3	1.777004229	2.31E-06	8.21E-06
SUN3	3.048041656	1.44E-06	5.40E-06
CARTPT	-2.726166225	0.000122319	0.000289493
AC120498.4	3.032415869	8.20E-07	3.27E-06
CFAP45	1.257754953	6.70E-07	2.73E-06
MLN	-2.565656234	0.022066041	0.031516832

AKR1B15	-3.081207581	4.67E-07	1.98E-06
TAF2	1.032026239	1.06E-15	6.05E-14
MIR4729	1.246081143	0.012241878	0.018504858
AC005324.4	1.804118522	6.29E-10	5.61E-09
AL136531.1	1.117499533	2.14E-06	7.69E-06
NASPP1	1.423209833	4.78E-10	4.40E-09
AC027796.1	1.02489155	0.00011747	0.000279008
AC008735.2	1.428139987	3.06E-10	2.97E-09
CDK5RAP1	1.029006568	4.97E-17	7.23E-15
NCOA7-AS1	-1.707762299	0.013917663	0.020763488
ANKRD18A	1.532827232	5.07E-05	0.000131186
POC1A	1.182243668	3.10E-09	2.26E-08
SLC5A5	-3.185757874	0.000234807	0.000520835
LHX1	4.345471264	1.26E-06	4.79E-06
ELOCP31	1.012558432	0.00025939	0.000569577
AC092171.4	1.918084936	2.47E-12	4.20E-11
RBL1	1.623863473	2.99E-17	5.42E-15
RPSAP71	2.462974219	0.000119196	0.000282629
TSC22D3	-1.372220022	4.48E-09	3.12E-08
MAFG-DT	1.320042519	1.71E-07	8.05E-07
FTLP12	1.40223856	0.00115288	0.002191583
EIF4A1P5	1.265389494	6.30E-08	3.31E-07
AF279873.3	2.844728933	0.009534331	0.01477253
RBAKDN	2.382080708	0.003964632	0.006686442
RNU6-323P	1.118792753	0.001113546	0.002123781
LINC00853	1.441778216	2.53E-08	1.47E-07
RNU6ATAC24F	1.341949708	2.89E-05	7.94E-05
PPAN	1.016668715	5.52E-13	1.13E-11
AL031602.1	1.491288225	8.84E-07	3.50E-06
PUS7L	1.060862812	6.17E-15	2.48E-13
FAM86B3P	1.203284	4.39E-07	1.88E-06
CNOT7P1	1.200659424	1.00E-07	5.01E-07
UBE2CP1	1.430172967	2.28E-06	8.15E-06
AC104170.1	1.106232458	6.35E-05	0.000160575
SCARNA6	1.157640527	1.24E-06	4.72E-06
TDRKH	1.17555033	6.04E-10	5.41E-09
SLFN12L	1.014888244	0.000564446	0.001148111
IMPA1P1	1.413521469	0.030045004	0.041532662
NPM1P37	1.240168375	3.89E-08	2.15E-07
AC008494.2	1.508967737	2.05E-06	7.40E-06
RNU7-61P	1.531255234	0.012469971	0.018812286

AC131212.2	1.041704184	1.63E-05	4.76E-05
AC015660.1	1.339432564	0.000462842	0.000961524
RPS13P4	1.605293505	2.06E-05	5.90E-05
NTAN1P2	1.175166903	1.67E-08	1.01E-07
B3GNT6	-1.247732203	1.47E-06	5.51E-06
NUTF2P4	2.057832861	3.21E-07	1.42E-06
PDCL2	2.699643661	0.001137036	0.002164862
ZWILCH	1.787665227	3.74E-18	1.72E-15
LINC00494	2.007172367	0.000139889	0.000326779
MIR3142HG	1.028732163	2.67E-05	7.42E-05
AC105339.5	1.852357733	1.38E-08	8.59E-08
IGHV1-68	-1.435319244	0.028035223	0.039011513
MNX1-AS1	1.669210011	1.07E-09	8.87E-09
MIR1303	1.418839336	0.000517007	0.001062817
FXYD1	-2.364156466	2.44E-14	7.79E-13
C1RL-AS1	1.259681661	2.88E-10	2.81E-09
ASPDH	-2.272925139	0.00317455	0.005469438
CTCFL	5.07807674	0.011630654	0.017670158
RASGRF1	1.560694895	0.009635167	0.014910555
TCF3P1	1.105884083	0.001208788	0.002285967
AL391152.1	2.908749499	2.29E-06	8.17E-06
CSTP2	4.896392004	7.57E-10	6.57E-09
SNRPGP5	1.399833149	8.38E-07	3.34E-06
POLR1F	1.002082479	4.52E-14	1.30E-12
TNNI1	1.159136501	6.38E-07	2.62E-06
AC073592.10	3.006391553	3.23E-08	1.82E-07
AC073575.1	1.415867566	2.37E-05	6.66E-05
NUFIP1	1.078489849	2.81E-15	1.32E-13
MCM3AP-AS1	1.122883427	1.99E-12	3.47E-11
SNORD19	1.599003685	5.51E-07	2.30E-06
AC011465.1	1.875479964	1.00E-10	1.10E-09
MSX2	1.932669533	1.16E-08	7.32E-08
AC092756.1	1.007640297	0.000253469	0.00055777
IGHVII-51-2	-3.911225888	0.000347443	0.000741593
ARSLP1	1.322291715	0.000430648	0.000900151
SLC7A6	1.231406845	1.80E-12	3.17E-11
IGSF6	1.313908698	3.46E-08	1.94E-07
MIR4269	-1.80662437	0.012379505	0.018689566
LINC02816	1.79658667	1.35E-06	5.11E-06
RPL41P1	1.593817498	0.015339163	0.022679726
LINC02626	1.319685005	0.004704	0.007794132

RN7SL173P	1.044013836	0.001166233	0.002214137
IGLV1-41	-2.962046481	0.020613089	0.029613045
AL133410.1	2.113892675	2.64E-15	1.26E-13
GNRH2	1.24414437	0.000479019	0.000991996
AC091805.1	1.32375165	2.35E-05	6.62E-05
EN2	4.444836455	0.001246381	0.002350208
CETN4P	-1.137137459	1.14E-06	4.38E-06
AC012615.6	1.065200331	4.17E-08	2.29E-07
IGHV3-63	-3.234634793	0.030399658	0.041953261
LIG1	1.384834627	1.08E-13	2.74E-12
IGLL3P	-2.808970345	0.019523876	0.028181997
TNNT1	2.386564065	7.81E-07	3.13E-06
NPM1P35	1.542489084	2.84E-07	1.27E-06
AC060766.5	1.25942788	1.97E-05	5.65E-05
COLCA2	1.110782427	0.002614279	0.004580405
HNRNPA1P62	1.305026067	7.14E-06	2.26E-05
C4B	1.269733423	0.00016079	0.000370504
AC138207.4	1.080055025	3.28E-10	3.14E-09
RUNX1	1.731496133	2.89E-16	2.20E-14
AC000403.1	1.316635583	5.97E-06	1.92E-05
CIDEC	-3.312184356	5.35E-05	0.00013767
RNU6-10P	2.48751205	8.31E-07	3.31E-06
MGME1	1.275078989	6.65E-15	2.64E-13
ATP5MC1P4	2.603014611	2.50E-14	7.92E-13
RN7SL15P	1.119716443	0.000641481	0.001288669
HSPE1P8	1.410091195	1.49E-07	7.13E-07
AC005522.1	1.984561156	5.50E-08	2.92E-07
LURAP1	-1.148033065	3.51E-06	1.20E-05
AC015849.1	1.735994162	0.000375983	0.000796576
AC005479.1	1.017730522	0.000102957	0.00024793
AC016745.1	2.052095055	0.005092841	0.008372939
AC092919.1	1.918913444	0.001960589	0.003531233
PPIAP82	1.593430961	1.38E-08	8.57E-08
IL13RA2	2.995558668	2.74E-08	1.57E-07
AL352984.1	1.565912653	0.003405122	0.005822055
CCT5P1	1.155906313	1.22E-08	7.70E-08
RNU6-123P	1.325569707	1.50E-05	4.42E-05
ADH4	-2.047038821	0.018345747	0.026678507
AC008667.1	1.503989884	0.000382601	0.000809181
AL118511.1	1.789580205	1.52E-07	7.25E-07
AC073150.1	3.124878184	5.97E-12	9.18E-11

ZNF29P	1.785507027	5.75E-07	2.39E-06
AC092902.2	1.067731181	0.010333927	0.015891283
LINC01012	1.920611512	3.28E-09	2.38E-08
AL512326.1	1.02384553	0.017486417	0.025539889
AC010287.1	1.512641235	7.99E-06	2.50E-05
AC091046.2	1.970005738	2.49E-08	1.45E-07
DNAH2	2.855897792	1.28E-09	1.03E-08
AL133406.1	2.120248965	6.88E-09	4.59E-08
PCLAF	1.847574988	3.59E-14	1.08E-12
MSI2	1.137187721	4.81E-15	2.04E-13
AC015982.1	1.054937452	0.000270802	0.000592438
AC241585.1	1.069895056	1.50E-05	4.44E-05
AC093690.1	1.021951274	3.88E-06	1.31E-05
AL391244.1	-1.100280718	1.34E-07	6.45E-07
AC090246.1	1.769753035	4.47E-07	1.91E-06
JUNB	-1.088955951	2.41E-05	6.76E-05
H3C10	1.052215532	0.001415873	0.002634196
LINC01224	1.476539563	5.87E-05	0.000149503
AL590135.1	1.155361742	6.53E-05	0.000164581
PPIAP30	1.068952556	2.95E-06	1.03E-05
LIMK1	1.301632178	1.59E-14	5.46E-13
ANKRD18EP	1.098700335	6.09E-08	3.20E-07
SOX14	3.008097849	0.002970739	0.00514871
SKA3	2.341353151	2.06E-16	1.76E-14
RN7SL442P	1.816362387	0.001879899	0.003399
LY75	1.148791525	4.88E-09	3.37E-08
ANKS1B	-1.707775115	6.26E-06	2.01E-05
THAP9-AS1	1.19010504	4.97E-12	7.80E-11
LINC01068	1.400042535	2.67E-06	9.37E-06
CHRNA5	1.494539403	1.05E-11	1.50E-10
KRT18P20	1.53359101	9.77E-09	6.30E-08
KRT18P10	1.717383945	9.99E-10	8.36E-09
RPL7P46	1.146987599	3.28E-05	8.90E-05
AC004923.4	1.951851009	6.58E-12	9.98E-11
RN7SKP287	1.630638987	3.48E-07	1.53E-06
CATIP-AS2	1.777949472	3.08E-08	1.75E-07
RERG	-1.931765721	0.003441794	0.005879473
BIK	1.123135034	8.97E-06	2.78E-05
SLC6A15	-1.132319502	0.000216388	0.00048356
AC140076.1	1.014778049	0.000141893	0.000330764
BCL2A1	1.059604045	3.19E-06	1.10E-05

RNF213	1.238049984	1.77E-10	1.82E-09
AC115088.1	1.770130922	0.000106396	0.000255364
SPATA25	1.260755549	9.57E-06	2.94E-05
ZFHX2-AS1	1.005549627	3.34E-07	1.47E-06
WDR97	1.623295724	2.54E-07	1.15E-06
SRRM5	1.287939283	2.12E-12	3.67E-11
RPS15AP28	1.144006087	0.001478516	0.0027366
MIR3153	2.927228582	1.46E-10	1.55E-09
AC011899.2	1.130904985	0.000105752	0.000253961
RBPMS2	-2.346113118	3.71E-09	2.65E-08
AP4B1-AS1	1.183502191	3.59E-08	2.01E-07
SNORA30	2.460221727	1.59E-05	4.65E-05
MIR4427	2.634477542	1.51E-07	7.20E-07
KCNE2	-3.954571347	2.43E-05	6.81E-05
ZNF280A	5.0946826	9.19E-08	4.63E-07
NAA40	1.003627523	2.92E-13	6.51E-12
RPL21P13	1.296938817	9.14E-05	0.000222836
ADCY10P1	1.283872739	1.51E-06	5.63E-06
NME2P1	1.493701232	2.65E-10	2.62E-09
AC138470.1	1.112588503	1.97E-06	7.12E-06
DES	-1.889552131	6.23E-09	4.19E-08
PKP4-AS1	1.586195402	7.03E-09	4.68E-08
AC090772.1	1.174992584	1.35E-05	4.03E-05
LINC00412	1.87574573	1.14E-05	3.45E-05
USP32P3	1.849037159	3.85E-08	2.14E-07
SNORA15B-2	1.020057314	0.001049899	0.002017702
ACSL3-AS1	1.004362785	0.000279646	0.000609583
AC124798.1	2.069429806	1.36E-11	1.88E-10
AC020913.1	1.228532094	2.16E-06	7.75E-06
CNGA3	-1.552772953	9.99E-06	3.06E-05
AC007128.1	5.673553315	3.68E-15	1.64E-13
HJURP	2.306868519	4.98E-16	3.42E-14
AC022540.1	1.422404127	5.90E-06	1.90E-05
FTLP15	1.471401191	2.62E-08	1.52E-07
PLXNC1	1.021256004	2.58E-05	7.20E-05
RNA5SP221	1.880374122	0.000790296	0.001558019
ACTG1P17	1.000941145	2.08E-05	5.94E-05
LINC01376	1.191190955	9.02E-07	3.56E-06
TRAF2	1.200728086	6.04E-16	3.92E-14
CAB39L	-1.75589561	5.78E-09	3.92E-08
AC020917.2	1.657245646	7.35E-05	0.000183229

AC092910.3	1.405237728	1.46E-12	2.63E-11
LINC02068	1.721365008	4.03E-06	1.35E-05
JUN	-1.15421683	0.000215427	0.000481494
AC015849.5	2.845815718	4.88E-14	1.39E-12
RMI2	1.505674995	4.75E-12	7.51E-11
IGHV3OR16-10	-2.594298159	0.016108642	0.023705614
AC243654.3	1.250084731	3.62E-05	9.71E-05
STAM-AS1	1.629163591	4.21E-09	2.96E-08
AC006483.1	1.019404361	8.39E-07	3.34E-06
TIMMDC1-DT	2.092553968	5.35E-08	2.85E-07
AP002490.2	1.449907774	6.70E-06	2.13E-05
PNPT1	1.380472346	6.38E-18	2.30E-15
AC013652.1	1.922629359	1.02E-06	3.97E-06
NCS1	-1.412986816	0.025223073	0.035508222
AC079336.3	1.063343486	0.000263441	0.000577947
TRAJ1	1.51526094	0.012314288	0.018601648
NT5C3AP2	2.312936451	9.25E-07	3.64E-06
AC109460.2	1.436682087	1.48E-11	2.03E-10
GABRR1	2.924996783	1.21E-05	3.64E-05
AC137932.3	1.289871642	6.61E-11	7.62E-10
HECW2	1.006548421	1.93E-08	1.15E-07
SNORA68B	1.51423584	6.18E-05	0.000156854
ADTRP	-1.391971295	0.020037739	0.028847155
ANK2	-1.578905154	1.19E-05	3.58E-05
AC011825.2	1.521369032	1.02E-08	6.53E-08
ZNF485	1.43467586	3.29E-16	2.47E-14
HSPA2	-1.977199635	1.42E-05	4.21E-05
RNU6-1160P	2.278063937	3.72E-05	9.96E-05
SPATA45	1.203375756	0.00064633	0.001297627
FNTAP2	1.138738585	2.35E-06	8.34E-06
AL021407.2	2.252907772	3.92E-08	2.17E-07
AC093536.1	1.119621854	5.25E-08	2.80E-07
HSD3B7	1.354526763	7.19E-11	8.19E-10
KRT18P38	1.543818437	1.21E-09	9.91E-09
HSP90AA6P	1.376664867	8.08E-09	5.32E-08
AC093677.2	1.118313659	1.68E-06	6.20E-06
BLACAT1	3.399082992	8.51E-15	3.21E-13
MMP1	2.067690178	6.65E-08	3.47E-07
FOXE3	3.754605728	5.04E-12	7.91E-11
AC254562.1	1.553691068	7.06E-08	3.66E-07
MIR571	1.401988753	0.011431154	0.017398376

AC079360.1	1.532682326	0.000128494	0.000302655
BACH1-IT3	1.237227211	0.00015145	0.000351047
ZNF252P-AS1	1.886729776	6.95E-13	1.37E-11
MIR548AR	1.05516183	0.009204667	0.014313661
AL138962.1	3.894562001	8.34E-07	3.32E-06
INA	-1.535907176	3.57E-09	2.56E-08
KLF9	-1.471480305	2.26E-07	1.03E-06
LINC01293	2.0239331	0.000193605	0.000437789
BARX1	-2.032997783	8.26E-05	0.000203454
TUBBP1	1.057890023	2.71E-10	2.67E-09
BNIP3P16	1.405996909	0.003700727	0.006282722
AL008729.1	1.126593557	1.46E-07	6.98E-07
AC007431.1	1.645039253	9.40E-08	4.72E-07
RRBP1	1.042250312	2.60E-08	1.50E-07
TFRC	1.507642442	4.44E-11	5.35E-10
RHPN1	2.082954294	8.94E-15	3.35E-13
KCNQ1OT1	1.908529256	1.32E-08	8.22E-08
AL512306.1	1.198724862	6.74E-05	0.000169556
AC002056.1	1.379519643	5.36E-09	3.66E-08
MIR567	2.040715104	0.000244499	0.000540127
C11orf42	1.275346928	6.45E-06	2.06E-05
AC245884.3	1.793099925	2.82E-10	2.77E-09
RAB15	1.8116055	7.03E-13	1.39E-11
CDK2AP2P2	1.013881173	0.003655908	0.006212571
AL023803.1	1.207909172	0.000840052	0.001647697
GAPDHP55	1.60972068	3.05E-07	1.35E-06
OAS3	1.551225425	4.25E-11	5.15E-10
AC006141.1	1.052328492	1.80E-05	5.20E-05
INHBB	1.840921632	0.000726805	0.001443855
TAF4B	1.010709637	1.50E-08	9.21E-08
AC016582.2	-1.58526081	4.87E-07	2.06E-06
MIR5697	2.627298875	2.14E-05	6.09E-05
PABPN1P1	1.193914232	1.83E-09	1.42E-08
AC011444.3	-1.301205283	0.006233621	0.010056079
BEND5	-1.567313648	5.11E-09	3.51E-08
G3BP1P1	1.574689979	1.04E-10	1.15E-09
FAM133CP	1.047252895	0.010451012	0.016048349
VDAC1P8	1.290493095	2.33E-12	4.00E-11
H3C12	2.675687474	1.26E-12	2.32E-11
AC009226.1	1.42503504	3.59E-07	1.57E-06
AC005096.1	1.268659065	4.67E-06	1.54E-05

MTFR2	2.328418281	1.65E-16	1.52E-14
PSMD10P1	1.589553826	1.43E-10	1.52E-09
AL451069.3	1.458427472	0.002021836	0.003632216
KPNA2	1.749567654	1.29E-16	1.28E-14
KLRK1	1.084757444	0.005388312	0.008811666
AL133467.1	-1.502242832	0.007339177	0.011657666
KCNRG	1.146337198	2.90E-07	1.30E-06
AL356740.3	2.955068753	2.85E-06	9.97E-06
AC113410.1	1.088572993	2.33E-06	8.28E-06
LINC02803	1.475825793	3.54E-07	1.55E-06
ENPP3	-3.836072809	4.84E-07	2.05E-06
AC007347.1	1.518091106	3.68E-06	1.25E-05
RNLS	-1.022801402	2.27E-08	1.33E-07
FKTN	1.031886848	4.25E-15	1.83E-13
AC019186.1	1.378175146	1.32E-06	4.99E-06
PRELID3BP11	1.889330641	5.77E-05	0.000147418
RFC3	2.122808381	2.96E-18	1.63E-15
GNL3	1.026212623	1.18E-11	1.66E-10
AC008875.1	1.473500022	2.81E-05	7.75E-05
TUBB4BP2	1.425650655	2.39E-07	1.09E-06
UBE2QL1	-1.780843025	6.97E-11	7.97E-10
CCDC86	1.195921763	8.20E-15	3.10E-13
RAD51D	1.150460527	8.93E-19	1.26E-15
SINHCAFP2	1.312022234	8.34E-08	4.24E-07
AC093895.1	5.851674341	1.39E-10	1.48E-09
STON1	-1.743376883	0.005011905	0.008255158
ITGB5-AS1	1.059610387	0.000300879	0.000650862
MIR1249	2.155497444	1.04E-07	5.16E-07
ZIC5	4.304021319	2.58E-08	1.49E-07
AL133230.1	1.457244596	1.37E-10	1.46E-09
KCNQ5	-1.349278006	5.00E-07	2.11E-06
AC092119.2	1.322127479	4.28E-08	2.34E-07
LINC00092	-1.175235195	2.73E-10	2.69E-09
GALNT15	-1.34207543	9.69E-07	3.79E-06
LINC02241	4.686396324	0.001338591	0.002503528
PRR20G	5.954756277	0.000728639	0.001447174
CALHM5	1.320440717	2.63E-07	1.18E-06
DLX6-AS1	3.33616224	3.18E-05	8.66E-05
IGKV3OR22-2	-3.478498237	7.88E-08	4.04E-07
AC005972.2	2.013844752	5.46E-05	0.000140262
HMGB1P37	1.287046	9.45E-09	6.13E-08

PHYHIP	-1.161526967	0.000655746	0.001314651
ALG1L7P	1.381463078	0.000333368	0.000714356
MTHFD1P1	1.392750156	4.75E-11	5.69E-10
NRG4	-1.264405723	0.000115638	0.000274903
DPT	-3.593672371	1.67E-16	1.52E-14
RCC2P6	1.287569314	2.41E-09	1.82E-08
DDX11-AS1	1.797257489	2.76E-13	6.19E-12
ANKLE1	1.281857215	0.000974269	0.001887469
PILRB	1.768436324	1.42E-11	1.95E-10
PPIAP2	1.159871522	1.63E-08	9.90E-08
IGKV2D-28	-3.244381881	0.006836785	0.010937049
RANP3	1.456735906	0.001627828	0.002987641
AC114495.2	1.236029387	7.00E-07	2.84E-06
FANCA	2.008043656	1.81E-15	9.25E-14
AL591178.1	2.37697826	3.09E-07	1.37E-06
RNU6-298P	1.295992448	0.00074535	0.001477831
AC011481.1	1.010394198	0.000224203	0.000499474
AC116049.1	5.319717293	7.99E-07	3.20E-06
PMAIP1	1.261742148	2.63E-07	1.18E-06
VIT	-2.551681404	2.76E-12	4.62E-11
TUBAP12	1.042165818	0.000627981	0.001264028
AC116407.4	1.112140611	2.08E-08	1.23E-07
KCNAB3	1.200622405	5.11E-08	2.74E-07
COX6CP14	1.548060688	0.000643473	0.001292574
RADIL	-1.374856589	1.40E-08	8.70E-08
STOML3	3.825971531	2.90E-08	1.66E-07
AGAP2-AS1	1.062444917	1.23E-07	5.99E-07
TMC7	2.040823814	5.09E-14	1.44E-12
AC051619.7	-2.700635621	0.000254162	0.000559248
RPL32P2	1.369091304	2.13E-08	1.26E-07
AC027808.2	1.661540311	7.89E-06	2.48E-05
TRAJ19	1.274452845	0.002231387	0.003973307
SLC25A34	-2.460301525	0.000137333	0.000321146
AL096828.3	2.30168279	1.63E-11	2.22E-10
RPS6KL1	1.306716487	2.35E-10	2.35E-09
LYPD8	3.307831653	0.00228576	0.004062252
AC027763.2	1.260624122	0.003044207	0.005264769
CYBRD1	-1.390478704	3.14E-08	1.78E-07
PRAG1	1.134702657	4.40E-09	3.07E-08
EZH2	1.810690332	1.95E-15	9.77E-14
NKAPL	-1.749865856	1.14E-05	3.45E-05

ESCO1	1.020564528	9.04E-13	1.73E-11
AC016526.2	1.627730461	5.13E-07	2.16E-06
AL691432.1	1.055377638	1.19E-06	4.56E-06
PPIAP42	1.309791353	2.23E-10	2.24E-09
AC068533.4	1.736183949	2.37E-06	8.41E-06
AC020923.1	1.429800685	0.000400301	0.000843202
AC068790.7	1.127470477	1.41E-05	4.19E-05
CYP24A1	3.747971173	1.73E-05	5.03E-05
HMGB1P14	1.038517802	1.14E-07	5.60E-07
CLDN12	1.311563669	3.55E-11	4.38E-10
ABCC4	1.115442341	1.75E-06	6.42E-06
LYVE1	-2.624305899	4.79E-14	1.36E-12
EMBP1	1.374905891	1.15E-05	3.49E-05
IGLV3-32	-2.944648693	0.000308059	0.000664774
RPS19P1	1.010390162	1.35E-06	5.10E-06
AC145124.1	-1.021076454	4.48E-06	1.49E-05
TUBB6	-1.145016214	0.002469844	0.004351381
OR5BA1P	2.569894676	1.07E-06	4.16E-06
HOXC6	2.39049769	1.24E-11	1.74E-10
AC005162.2	1.881706146	0.000486512	0.001005891
C12orf75	1.641340035	1.25E-10	1.34E-09
MORF4L2-AS1	1.047884646	1.45E-06	5.44E-06
AC104695.3	1.1858877	0.001478438	0.0027366
MIR5587	2.129426219	1.23E-09	1.01E-08
LINC01270	2.008634534	1.46E-08	9.02E-08
BRCA2	2.153491795	1.61E-16	1.50E-14
MIR554	2.154411153	1.45E-08	8.95E-08
IGHD2-15	-1.992123509	0.023869949	0.033806984
HSPE1P5	1.706930095	6.47E-10	5.75E-09
EFTUD2	1.057826222	5.59E-16	3.73E-14
H2BC9	1.567549683	2.68E-05	7.45E-05
SHISAL1	-1.575246768	0.00109473	0.002092688
HNRNPA1P41	1.285497773	3.20E-05	8.69E-05
SCARNA13	1.490466709	1.26E-09	1.02E-08
CHORDC1P1	1.057476911	0.000952582	0.001849035
RNA5SP217	1.726068217	3.60E-06	1.23E-05
DNAH6	1.522584465	2.65E-06	9.33E-06
CXCL5	2.570593174	0.000747777	0.001481981
AL590103.1	1.939029489	1.47E-05	4.35E-05
RNA5SP168	1.603183406	0.002339531	0.004146748
TPM3P8	1.156693073	1.05E-08	6.70E-08

AC015574.1	3.32559731	0.003043764	0.005264344
HPDL	2.230238158	4.98E-11	5.91E-10
RNF14P1	2.010507885	4.25E-09	2.99E-08
RECQL4	2.5040501	1.21E-16	1.24E-14
AC004637.1	-1.587883359	0.000389957	0.000823531
PILRA	1.100590015	3.33E-05	9.02E-05
RPL31P52	1.005249786	7.62E-05	0.000189256
NQO2-AS1	1.205765284	0.000128898	0.000303419
KCNMB2-AS1	5.409468322	1.03E-12	1.95E-11
TAF9P3	1.369052777	9.31E-05	0.000226575
FOXO6-AS1	1.452642173	0.006643462	0.01065273
MAT1A	3.72975768	0.001255531	0.002365619
RNA5SP477	1.464340853	0.011474042	0.017455116
MAGEA4	10.3157325	1.04E-05	3.16E-05
RSF1-IT1	1.446544816	5.49E-06	1.79E-05
AKR1B10	-2.107007086	5.38E-07	2.25E-06
AL031736.1	3.171082847	0.000616233	0.001242541
AC004847.1	1.076887122	0.000124848	0.000294614
RNF157-AS1	1.297960785	0.002019901	0.003629228
AC005154.1	1.049374865	8.36E-05	0.000205684
SNX18P3	1.735639313	0.000109576	0.000262128
IGFL2-AS1	1.414734514	2.05E-05	5.86E-05
AC022306.3	1.13794026	6.22E-06	2.00E-05
CAD	1.509287486	3.37E-17	5.87E-15
PPAT	1.752725434	1.61E-18	1.34E-15
CHORDC1	1.301599705	1.10E-15	6.24E-14
RNU6-702P	2.251991676	4.03E-06	1.35E-05
AC105935.2	1.283135939	5.64E-05	0.000144277
AC008758.2	1.644313193	3.44E-06	1.18E-05
CD80	1.787471913	6.75E-11	7.75E-10
FAM102B	1.159514563	2.76E-12	4.62E-11
KLK14	1.457203429	0.02845163	0.039529012
RPL21P65	2.089337204	7.79E-10	6.75E-09
DDX18	1.026641569	1.02E-17	3.00E-15
PID1	-1.116313017	6.17E-06	1.98E-05
PKMP4	1.433894845	1.56E-09	1.24E-08
MPRIP-AS1	1.118378786	0.002025226	0.003637384
AL035045.1	2.949373819	6.16E-06	1.98E-05
AL732366.2	1.298837683	1.75E-05	5.09E-05
RNA5SP39	1.845098057	0.000332109	0.000711773
SST	-1.879562346	5.00E-07	2.11E-06

AC008760.2	2.000871512	1.01E-07	5.03E-07
LINC01419	6.608129292	5.70E-06	1.85E-05
SCN8A	1.577825686	0.000428742	0.000896308
AC027307.1	1.196306528	2.96E-05	8.12E-05
KNG1	3.850331067	7.54E-10	6.55E-09
AC093525.4	1.463942682	1.80E-06	6.60E-06
ARFGEF3	1.250630775	4.44E-08	2.42E-07
MIR200A	1.10971529	0.00014849	0.000344906
PLAC4	1.720782146	0.009205938	0.014313661
HSF2BP	1.378973868	8.53E-07	3.39E-06
AC004801.2	1.885454922	1.27E-11	1.78E-10
DNAJC19P8	2.952992283	2.61E-09	1.94E-08
AC104619.1	1.244235783	1.19E-07	5.81E-07
AC022784.6	3.676448465	4.23E-10	3.95E-09
AC112907.2	1.350834238	0.002654196	0.004645316
RPL23AP95	1.411594582	3.25E-10	3.12E-09
KIF11	1.919759514	9.83E-16	5.77E-14
LINC02345	2.571966037	3.88E-11	4.76E-10
MTND4LP13	2.038421559	4.54E-06	1.51E-05
RRP12	1.346242768	1.21E-16	1.24E-14
ADPGK-AS1	1.066743265	2.30E-07	1.05E-06
BEND3	1.056482198	1.43E-08	8.84E-08
AC008543.3	1.534579935	5.89E-09	3.99E-08
RPS23P6	1.434893898	4.45E-06	1.48E-05
COPZ2	-1.106999788	0.000747777	0.001481981
MAFA-AS1	2.568207776	0.002021748	0.003632216
MIR1972-2	1.895933371	1.10E-06	4.26E-06
AL645608.8	2.494048515	9.59E-07	3.76E-06
AC106886.3	1.099880462	0.002552334	0.004481302
AC079601.2	1.081693719	1.20E-06	4.59E-06
AC091152.1	1.344416634	8.77E-08	4.44E-07
AL360091.2	1.247275217	0.004059729	0.006830826
AC090954.1	2.530575174	2.24E-05	6.34E-05
STK3	1.24926706	2.01E-16	1.73E-14
RN7SL334P	1.608256402	0.013661742	0.020409114
LHX2	2.203164158	1.10E-09	9.05E-09
TMEM59L	-1.263804847	6.33E-07	2.60E-06
AC010761.1	1.082322153	1.08E-10	1.18E-09
LDHAL6A	1.106722886	0.00109774	0.002098026
RNU6-481P	1.441661341	4.89E-06	1.61E-05
LRRC46	1.393346528	8.04E-10	6.93E-09

AC007599.2	1.243335054	0.009625674	0.014901058
AC091181.1	1.325514623	1.80E-06	6.60E-06
RNU1-56P	1.324930575	0.001450658	0.00269177
AC108463.1	2.508896936	7.06E-17	8.73E-15
AL356320.1	2.076093322	4.24E-09	2.98E-08
IBSP	7.344500952	5.21E-16	3.54E-14
AC112187.1	1.406298093	4.64E-07	1.97E-06
MT1E	-1.213725355	1.13E-06	4.34E-06
NRCAM	2.432386584	6.44E-05	0.00016259
PTMAP9	1.2002431	1.69E-12	2.99E-11
MORN5	-2.701643057	8.80E-05	0.000215435
CDKN3	1.724878889	6.06E-13	1.22E-11
MIR27B	-1.683411698	0.000111397	0.00026596
AC012020.1	1.918741024	1.02E-08	6.53E-08
AC073655.2	1.084832218	4.41E-06	1.47E-05
SLC6A7	2.412878456	8.69E-08	4.41E-07
AC087276.3	1.168822111	0.003779819	0.006403532
AC105036.1	1.775519011	1.60E-07	7.59E-07
AL354877.1	1.471193131	5.80E-06	1.88E-05
RPS12P31	1.556186298	3.73E-06	1.27E-05
KRT18P1	1.617406111	2.32E-09	1.76E-08
ALKAL1	2.470266208	1.87E-09	1.45E-08
AC105339.3	1.373465816	1.06E-06	4.10E-06
AL121989.1	2.046738451	5.00E-08	2.69E-07
LINC00624	1.920797903	2.04E-13	4.74E-12
AC008739.2	2.179301153	8.93E-05	0.00021839
CFD	-2.639893304	1.74E-16	1.57E-14
LINC02418	4.605230559	9.48E-06	2.92E-05
AC090617.6	1.076401699	0.004761238	0.007879303
NUP210	1.068307685	0.000591193	0.001196306
SPDYE19P	1.15014584	0.000361843	0.000769555
AL121772.3	1.503455189	1.36E-07	6.55E-07
MIR4479	1.894129104	2.97E-07	1.32E-06
PACRG	-1.10108855	3.04E-09	2.23E-08
CLEC19A	2.212767986	4.93E-05	0.000128079
WDR17	-1.100636579	2.17E-06	7.80E-06
RPS27AP10	1.216238121	0.000346091	0.000738997
TSPAN13	1.085980593	2.54E-07	1.15E-06
BMP3	-1.798112221	0.000133751	0.000313735
HNRNPA3P9	1.781216752	5.04E-13	1.04E-11
RNF122	-1.017702479	0.000270237	0.000591347

RNU6-1043P	1.686399336	0.000149416	0.000346846
LINC02570	1.900449222	0.004056225	0.006826654
RPL8P1	1.172224934	2.35E-06	8.34E-06
RNU7-181P	2.205284349	1.63E-09	1.28E-08
HSPE1P25	1.837893225	9.13E-05	0.000222678
AC091390.1	1.610954589	3.15E-07	1.39E-06
AL031123.2	1.728274926	4.50E-12	7.15E-11
BNIP3P11	2.483612462	1.79E-15	9.15E-14
AC005089.1	2.41180448	2.15E-08	1.27E-07
LGI3	-3.693475621	4.49E-10	4.17E-09
TOMM40	1.148595554	1.85E-11	2.48E-10
AC024580.1	1.361105252	5.16E-08	2.76E-07
VEGFD	-2.671207066	3.42E-16	2.53E-14
AL583832.1	1.463245491	5.46E-09	3.72E-08
ZNF33BP1	1.291172444	6.11E-05	0.000155022
AC117383.1	1.792807133	2.48E-06	8.78E-06
SPDYE6	1.242484588	1.46E-07	6.98E-07
TPM1	-1.819219591	0.002724129	0.004754733
CFL1P6	1.648235706	2.35E-05	6.61E-05
NXT2	1.163317497	1.59E-10	1.66E-09
AL049779.2	1.205264725	9.79E-07	3.83E-06
PTMAP8	1.214950552	1.18E-09	9.67E-09
AC099811.6	1.356706758	6.70E-07	2.73E-06
HOXA10	3.984599611	1.19E-13	2.99E-12
TLX1	2.668178426	1.12E-07	5.51E-07
GAPLINC	2.512508989	9.22E-16	5.52E-14
AC012467.1	2.938468583	2.88E-10	2.81E-09
RN7SKP255	1.195677096	0.002955715	0.005125368
AC093155.2	1.232655846	1.71E-06	6.30E-06
LINC01891	1.384103598	0.00180167	0.003273106
LINC02709	1.670173797	4.57E-10	4.23E-09
RPL23AP66	1.044447872	8.88E-05	0.000217234
MFSD13B	1.005319632	3.90E-05	0.000103872
SPARCL1	-1.312789525	7.16E-05	0.000178892
TGFBR3	-1.415587686	5.00E-05	0.000129605
AP001065.3	-2.194558821	6.81E-06	2.16E-05
AC002553.2	1.237211025	1.34E-07	6.48E-07
NMI	1.098464688	2.76E-12	4.62E-11
AC020978.9	1.074635575	1.47E-07	7.03E-07
TMEM35A	-2.801097254	5.52E-10	5.00E-09
IGHV1-67	-2.681253427	0.036810169	0.049862144

FBXL7	-1.082141523	0.001297896	0.002434424
SH3GL2	-2.493257461	1.28E-13	3.19E-12
LONRF2	-1.740523933	8.77E-06	2.72E-05
AC109992.2	2.031167538	4.08E-11	4.97E-10
MIR181A2HG	1.525590118	8.73E-07	3.46E-06
AC126118.1	1.092871102	3.04E-05	8.31E-05
DUTP1	1.953048078	2.21E-07	1.01E-06
COX6B1P5	1.503386607	1.51E-05	4.45E-05
AL590762.1	1.1022506	0.000249645	0.000549992
LAMC2	2.775381298	3.19E-15	1.46E-13
IGKV1-13	-2.340897713	0.015255306	0.022574532
HSPA9P1	1.052927515	1.86E-08	1.12E-07
KLK6	2.344737261	7.68E-08	3.94E-07
SLC1A5	1.54210311	4.50E-12	7.15E-11
MTPAP	1.062267701	3.38E-16	2.51E-14
MYLK2	1.587744228	1.24E-09	1.01E-08
MIR1285-1	1.439001258	0.000139195	0.000325244
RNU6-288P	1.62551083	0.000296897	0.000643346
AL391095.3	1.614190343	6.77E-08	3.52E-07
RNU5A-8P	2.489620389	4.33E-05	0.000113901
SEMG1	3.347942108	0.00229697	0.004079453
SUMO2P19	1.41623914	3.76E-05	0.000100597
GARS1	1.000094361	1.33E-14	4.65E-13
MIR5695	1.963304256	0.000206498	0.000463521
GABPB1-AS1	1.25509358	1.02E-06	3.98E-06
VDAC1P4	1.217977754	6.18E-07	2.54E-06
LINC02585	1.385194457	2.06E-09	1.58E-08
SLC25A5P5	1.232981065	3.44E-06	1.18E-05
RPS26P2	1.008118006	0.000149096	0.000346132
AL355488.1	1.923092637	2.69E-13	6.06E-12
C8orf31	3.147285299	1.20E-11	1.69E-10
AC024937.2	1.822945698	1.73E-06	6.35E-06
SAMD12-AS1	1.527722004	2.32E-09	1.76E-08
AL031666.2	1.804600916	1.75E-10	1.81E-09
RPL12P11	1.08007129	3.33E-05	9.02E-05
MTND1P8	1.045133792	0.000420257	0.000880641
TMEM100	-2.720050009	3.56E-12	5.81E-11
RPL35AP24	1.78771963	2.69E-05	7.47E-05
HSP90AA1	1.359119177	2.57E-18	1.59E-15
RNA5SP437	1.886624791	6.23E-05	0.000157862
PRSS56	6.746965297	0.000109927	0.000262826

SPATA17	1.861328281	1.18E-09	9.67E-09
PLK2	1.055901568	3.33E-05	9.02E-05
AC009148.1	1.614200636	7.47E-10	6.50E-09
AC096921.1	1.090675621	0.001705958	0.003117935
RNA5SP490	1.157716628	0.007017446	0.011197819
AC013470.1	2.784694712	1.82E-06	6.66E-06
LINC01411	2.200954103	0.001632155	0.002994138
GPR84	2.191324987	5.36E-10	4.87E-09
MAK16	1.067501635	5.25E-15	2.17E-13
AC004975.1	1.106124344	5.14E-06	1.68E-05
RHEBL1	1.817235322	1.61E-13	3.86E-12
AL137161.1	1.72040109	0.008667054	0.013551713
RNA5SP82	1.469587286	1.08E-07	5.36E-07
SNRPFP4	2.057926837	7.77E-07	3.12E-06
LRP8	2.647071488	2.32E-16	1.88E-14
CHN2-AS1	1.598789127	7.28E-06	2.30E-05
GPX3	-2.115781247	4.84E-17	7.08E-15
ENTPD6	1.061228521	5.98E-10	5.36E-09
RNU6-672P	1.005293634	0.003433607	0.005867746
MGAM2	1.299252102	5.34E-07	2.23E-06
TCEAL5	-1.636512118	6.49E-08	3.40E-07
NCAPGP1	1.901768414	3.44E-06	1.18E-05
SPON1	-1.207003559	0.000682575	0.00136382
AZGP1P1	1.821572568	0.003789852	0.006417673
JRKL	1.313361366	1.63E-17	3.68E-15
FOS	-1.172234022	4.03E-05	0.000106983
DBF4B	1.345368903	9.83E-16	5.77E-14
PAX8-AS1	1.3687128	1.05E-05	3.20E-05
EMSLR	2.214335246	3.16E-09	2.30E-08
LEMD1	3.390992871	1.17E-12	2.18E-11
SDS	2.831864919	1.68E-15	8.74E-14
AL121916.1	1.438273824	0.001694112	0.003098836
ARHGAP4	1.112014752	1.43E-06	5.36E-06
LINC01146	3.499173456	3.00E-11	3.79E-10
COL7A1	1.769449208	3.83E-11	4.69E-10
PKMP1	1.187419423	1.17E-09	9.58E-09
LINC02449	1.201791302	0.001681994	0.003077333
RPS12P20	1.794744805	3.84E-08	2.13E-07
AC002511.1	-1.369969391	9.76E-07	3.82E-06
LEKR1	1.334097676	3.52E-10	3.34E-09
AC039056.2	1.285634943	2.26E-07	1.03E-06

FAM122B	1.374302743	2.82E-16	2.16E-14
MIR613	2.747104341	0.000104816	0.000251895
TEL1-TNFRSF6	1.956224293	1.14E-14	4.08E-13
HSPB2	-1.375928164	3.64E-05	9.76E-05
FHOD1	1.162435784	3.90E-16	2.78E-14
NANOGP5	1.0410635	0.000101956	0.00024582
MIR6728	1.875577133	0.000372234	0.000789448
CCDC138	1.510258244	3.19E-15	1.46E-13
DSCAS	1.035843523	0.000280414	0.000610758
PEG3	-1.366398854	8.71E-06	2.71E-05
AC022211.3	1.580555413	7.04E-10	6.19E-09
AC000123.2	1.172914029	1.10E-09	9.09E-09
CSNK1G2-AS1	1.31171044	5.31E-06	1.73E-05
AL606489.2	2.193548918	7.01E-09	4.68E-08
AC020612.2	1.660071804	9.20E-09	5.99E-08
NELFCD	1.32475167	1.55E-16	1.48E-14
ATP2C2-AS1	1.057220227	2.87E-05	7.91E-05
RNU7-125P	1.745172007	9.16E-05	0.000223389
RNU1-16P	1.296128223	9.82E-07	3.84E-06
NANOGNBP3	1.675298256	4.89E-08	2.64E-07
PODNL1	1.727592121	1.84E-09	1.43E-08
AP001628.1	2.036211942	4.42E-09	3.08E-08
Z95327.1	1.170241724	0.000887637	0.001732611
DCDC2	2.719025102	8.21E-05	0.000202319
IGKV3D-11	-2.798975561	0.027667664	0.038556975
RNU6-879P	1.985709927	5.52E-06	1.80E-05
MIR4285	2.24228064	0.000844424	0.001655419
ADAT1	1.027155359	4.04E-15	1.77E-13
AC002064.2	1.195817454	0.000186722	0.000423951
AC011676.3	1.245850076	0.001073472	0.002058255
WDR75	1.120434372	2.00E-17	4.19E-15
CCL18	2.253514582	3.47E-05	9.36E-05
RN7SL505P	2.002854418	5.60E-08	2.96E-07
B4GALT4-AS1	1.333948672	0.000265786	0.000582325
SYCE3	1.848570523	3.23E-07	1.43E-06
LINC02320	2.288227312	7.00E-07	2.84E-06
H2BC13	1.252352451	7.36E-06	2.32E-05
NXF3	3.211899979	0.007762288	0.012265975
ENHO	-1.763623734	1.72E-06	6.33E-06
NRG3	-1.294656694	0.000412014	0.000864939
AL135924.1	2.25526622	2.32E-07	1.06E-06

PRR19	2.225246126	1.03E-13	2.63E-12
NCAPG	2.082517076	8.73E-15	3.28E-13
AL157834.1	1.389257245	0.001738646	0.003171585
SNRPEP2	1.362191749	1.12E-10	1.22E-09
SAMD3	1.255489894	7.20E-05	0.000179903
CYP11A1	-1.560924255	1.35E-06	5.10E-06
AC091516.1	3.096374845	5.75E-12	8.88E-11
ITGA2	1.489018731	6.10E-10	5.46E-09
AC024267.3	1.365020587	3.89E-08	2.15E-07
EPHX4	1.870868608	1.65E-05	4.82E-05
AC026348.1	1.062851871	4.72E-05	0.000123172
NOP56	1.387876134	5.88E-18	2.18E-15
KRT8P43	1.025732152	0.000984955	0.001905576
AC005326.1	1.365629061	0.000413095	0.00086693
SCGB1D2	-1.213917038	8.55E-05	0.000209859
AC092375.1	1.91641221	2.20E-05	6.24E-05
LINC01018	-1.591579068	1.76E-08	1.06E-07
CEP135	1.222776569	1.51E-15	8.01E-14
STEAP1	1.546646239	1.48E-09	1.17E-08
SLC5A2	1.998137341	7.84E-12	1.17E-10
RNU6-720P	1.016352965	0.000562435	0.001144546
SCNN1B	-3.363370804	5.87E-15	2.38E-13
PSME2P5	2.323435457	2.38E-08	1.39E-07
AC104823.1	4.947367222	1.64E-07	7.75E-07
TSSK3	1.040623997	2.11E-06	7.59E-06
RPL35AP28	1.005304294	0.000757942	0.001500118
AC025265.1	1.298452571	1.26E-06	4.81E-06
AC011375.1	1.309417096	0.000125865	0.00029683
AC011498.3	1.812481488	9.47E-09	6.13E-08
IGLV3-17	-3.598236145	5.63E-05	0.000144144
AC004943.2	1.237823183	2.72E-14	8.52E-13
AL513320.1	1.250952776	1.42E-06	5.32E-06
RCN3	1.408693305	6.58E-09	4.41E-08
AL360181.1	1.580624894	4.44E-05	0.000116504
AL162431.1	1.115369196	5.96E-07	2.46E-06
RNVU1-33	1.469749563	0.000240609	0.000532261
AC009220.3	1.067807502	0.000275175	0.0006009
IGKV1OR1-1	-3.346738359	0.001653589	0.003030954
AC087441.2	1.17309845	0.000413981	0.000868447
ARHGAP39	1.848431505	1.03E-15	5.96E-14
AC004672.2	1.216128476	0.001721619	0.003143754

DZANK1	1.068449653	2.21E-09	1.68E-08
AL158834.2	1.100729277	1.04E-05	3.18E-05
EPHA6	-2.632675021	8.10E-05	0.000199843
TMEM252	-2.139355507	2.83E-10	2.78E-09
FAM89A	1.007551949	5.07E-08	2.72E-07
KRTAP5-5	2.824882008	0.000656936	0.001316936
PRSS21	3.736137143	0.00428507	0.007164973
AC005726.1	1.113591834	1.17E-08	7.38E-08
LINC01503	1.126076956	0.000404111	0.000850085
SNORD19C	2.130604632	2.83E-11	3.61E-10
AJ009632.2	2.220593262	2.38E-09	1.80E-08
CDK1	2.177775472	5.89E-16	3.85E-14
RBFOX3	-1.871815124	0.000125243	0.000295521
MIR16-1	2.257765053	1.62E-05	4.75E-05
C3	1.154099081	0.012886407	0.019371419
INE1	1.117720346	1.06E-09	8.78E-09
ILDR2	1.326104919	4.55E-05	0.000119065
OGFR-AS1	1.126978955	7.50E-07	3.02E-06
HDC	-1.616336091	2.38E-07	1.08E-06
AC008268.1	-3.356249958	0.003651584	0.006206807
AL356752.1	2.262239466	1.12E-10	1.22E-09
AC090912.2	2.15744967	3.85E-08	2.14E-07
RBM28	1.07096451	6.87E-16	4.36E-14
AL590560.3	1.066149424	0.030484848	0.042057756
NPM1P6	1.015162508	9.25E-08	4.65E-07
POU2F1	1.175991001	3.21E-16	2.42E-14
AC004890.3	1.036929651	1.94E-06	7.02E-06
RPS18P13	1.033516934	4.57E-05	0.000119442
BARX1-DT	-1.744058217	0.000273315	0.000597396
RNU1-153P	1.112089689	0.000804609	0.001583658
HMGB1P51	1.790595867	5.39E-08	2.87E-07
RNU7-84P	1.340263876	9.59E-05	0.000232733
RNU6-558P	2.153877165	3.21E-06	1.11E-05
OVOL3	1.621861264	1.49E-07	7.10E-07
C1GALT1	1.068080867	7.73E-11	8.75E-10
AL360091.1	1.034823768	0.000406505	0.000854666
AC023389.1	1.139823928	1.42E-05	4.20E-05
UNC5B-AS1	-1.385338164	9.25E-08	4.65E-07
MTTP	-4.195568939	0.000752015	0.001489318
AC104986.1	1.027975671	1.02E-06	3.95E-06
TRIM74	-1.379704979	0.026948136	0.037669985

SUV39H2-DT	1.140866718	4.81E-09	3.33E-08
PNLIPRP1	3.049597132	0.007866858	0.012415664
ALG3	1.241531439	4.87E-15	2.05E-13
TTTY14	-1.179871703	0.004835347	0.007991026
CHP1P2	1.338864812	2.08E-10	2.10E-09
MIR3679	1.299228608	0.000131156	0.000308299
TBX15	2.13513488	7.36E-09	4.89E-08
SKP2	1.578690957	7.37E-14	1.96E-12
MCM2	1.863035755	1.51E-15	8.01E-14
SRRM1P3	1.437800737	1.41E-10	1.50E-09
CXCL10	3.21464345	6.28E-10	5.60E-09
SPTSSB	-1.492853349	0.001962189	0.003532445
CSPP1	1.336471106	1.67E-16	1.52E-14
OR2I1P	2.765007849	1.84E-09	1.43E-08
AC023511.1	-1.616260511	1.30E-06	4.93E-06
AC122688.1	1.773874762	5.88E-07	2.44E-06
AP001350.1	1.124343568	1.78E-05	5.15E-05
ZNF860	1.298860865	2.17E-08	1.28E-07
RNU6-840P	1.49773819	0.00427961	0.007158748
NPM1P32	1.131263867	1.60E-05	4.70E-05
AC013410.2	1.578886262	7.88E-05	0.00019481
AC010542.5	1.460973694	4.21E-11	5.11E-10
SUZ12	1.034515415	2.04E-16	1.74E-14
RNU6-190P	1.33187566	1.96E-05	5.64E-05
PTP4A2P1	1.080717255	1.60E-08	9.73E-08
CENPS	1.235800843	5.99E-12	9.21E-11
AC017074.1	1.378686893	7.20E-05	0.000179903
CKS1B	1.338396168	4.00E-13	8.49E-12
AC012513.2	1.031588766	6.84E-06	2.17E-05
IGLV7-46	-3.262236618	0.033630541	0.045938518
UBOX5-AS1	1.143104197	6.49E-07	2.65E-06
COL12A1	1.753457815	5.31E-10	4.83E-09
AL359382.1	1.667518473	0.002516751	0.004425827
AC004551.1	1.812712454	2.28E-08	1.34E-07
RNU6-302P	2.064513301	4.94E-06	1.62E-05
HDGFP1	1.108542378	0.000333473	0.000714523
AC253536.3	1.342528837	1.91E-06	6.92E-06
AC012150.2	2.372491021	6.90E-07	2.80E-06
E2F1	2.100134715	2.68E-16	2.08E-14
AL358472.3	1.035473234	7.23E-07	2.92E-06
FSIP2	-1.025484794	0.010547502	0.016177695

AC011939.1	1.052042886	8.19E-07	3.27E-06
RANBP3L	-1.604506666	2.60E-06	9.17E-06
AL021707.4	1.080474631	1.22E-05	3.67E-05
IL24	1.586752684	4.43E-06	1.47E-05
WFDC3	1.069997895	0.000344049	0.000734879
SEPTIN11	1.278602243	2.87E-17	5.31E-15
TTLL4	1.262443954	8.61E-14	2.24E-12
CORIN	2.695002677	4.24E-13	8.92E-12
RPS15AP6	1.489240228	5.32E-07	2.23E-06
FAP	3.246167732	1.22E-15	6.79E-14
HLF	-2.632741193	2.19E-08	1.29E-07
RPL26P29	3.079123419	5.80E-13	1.18E-11
NUDT5	1.038648116	2.93E-14	9.04E-13
BVES	-1.606329392	8.00E-05	0.000197543
VAV2	1.336893694	1.12E-12	2.10E-11
SP3P	3.40051032	5.11E-07	2.15E-06
AC040160.1	1.043136122	1.26E-07	6.12E-07
SULT2A1	-1.828131124	0.000505195	0.001041066
AC008267.7	1.24161382	0.000214592	0.000480071
AL109615.3	3.95605953	3.65E-16	2.64E-14
SMC3P1	1.044462967	1.08E-08	6.85E-08
MFHAS1	1.321843727	3.27E-15	1.49E-13
HIGD1C	1.269261425	0.006806436	0.010892424
RNU6-1147P	1.64760032	0.00183176	0.003319848
AL121949.1	2.671891519	0.026880605	0.037597285
RNF112	-1.197339643	3.17E-08	1.79E-07
RNU4-14P	1.345913283	0.014725664	0.021861202
CYTOR	1.309964956	6.08E-11	7.08E-10
RAD18	1.074609589	2.40E-13	5.46E-12
RNF207	1.675645497	4.50E-12	7.15E-11
IFITM9P	1.645336854	6.67E-10	5.89E-09
AADACL2-AS1	1.888389088	1.67E-05	4.88E-05
CASK-AS1	2.435503089	8.81E-10	7.50E-09
KIR2DS4	1.820978688	0.00061954	0.001248452
CPT1C	1.006962818	0.005286348	0.008660613
CST5	2.366457137	0.0001072	0.000256999
SNORA16B	1.420650305	1.00E-06	3.90E-06
AL049757.1	1.428522155	4.25E-06	1.42E-05
MFAP2	2.473701842	7.30E-12	1.09E-10
AC009237.3	1.679071611	4.52E-08	2.46E-07
AL356299.2	2.418841962	1.96E-13	4.59E-12

AC111170.3	1.127353247	7.09E-07	2.87E-06
SNRPEP11	1.660545805	0.000109663	0.000262313
ERVW-1	2.916474452	1.35E-06	5.09E-06
LGALS9C	-2.739688432	1.15E-06	4.43E-06
RPL7AP28	-1.603213424	2.01E-05	5.75E-05
PRDX3P2	1.349978643	8.37E-10	7.17E-09
PUM3	1.190827926	5.67E-14	1.58E-12
KRT18P39	1.768129106	1.80E-11	2.41E-10
C11orf53	1.702546116	0.008851671	0.013809581
RNU6-249P	2.346964171	0.000332647	0.000712869
POTEF	1.140318536	7.35E-05	0.000183207
SHANK2-AS2	1.954075692	6.29E-06	2.01E-05
STPG4	3.494295696	4.46E-14	1.29E-12
CCL14	-1.250606265	1.42E-07	6.82E-07
PRB3	1.804453165	4.15E-06	1.39E-05
AC245884.10	1.451334704	2.83E-06	9.91E-06
CCDC150	2.689602665	7.90E-17	9.44E-15
SIRPB1	1.440107634	0.000100395	0.000242429
CNR1	-1.109440012	2.34E-05	6.60E-05
B4GALNT2	-1.65973307	0.000485254	0.001003581
AF235103.1	2.049428041	9.13E-08	4.60E-07
LINC02428	3.805011257	0.004670361	0.007744788
AL031714.1	1.239133133	2.10E-10	2.12E-09
ACKR1	-1.432664596	4.25E-11	5.15E-10
CRYL1	-1.15197691	0.00100737	0.001944281
RPL7P27	1.209275882	5.02E-05	0.000130134
PFN1P8	1.576237007	2.95E-06	1.03E-05
PLAC1	2.54523474	0.000116219	0.000276234
TPD52L1	-1.184861399	0.00693792	0.011082867
AC015849.3	1.353784304	3.03E-10	2.94E-09
INTS7	1.148411806	5.74E-16	3.80E-14
ADAT2	1.464233559	2.06E-13	4.79E-12
UGT8	1.024187456	3.11E-05	8.48E-05
Z98257.1	3.480951831	1.99E-07	9.22E-07
SNORD111B	2.397138388	2.66E-05	7.39E-05
DCUN1D2-AS	1.523604679	2.33E-08	1.36E-07
CD300LF	1.486133964	5.99E-08	3.15E-07
SLFN13	1.237543506	0.000130535	0.000306947
ZNF841	1.069186846	4.80E-10	4.42E-09
GAS6-AS1	1.369752786	0.001119182	0.002133308
AL390728.3	1.437510891	0.000160475	0.000370002

MIR558	1.166866353	0.00191751	0.003460195
AL096855.1	1.212376061	0.00360781	0.006135142
MYLK	-1.756244103	0.000125646	0.000296339
VAT1L	-1.044630746	0.000520332	0.001068168
SNRPGP3	1.855705852	0.000201355	0.000453043
LINC01615	2.802542729	2.26E-13	5.19E-12
ARHGAP10	-1.43857398	0.000205038	0.000460592
NLGN1	-1.389228195	4.25E-08	2.32E-07
TMTC1	-1.130707723	0.000968851	0.001877929
PTX3	-1.243826361	3.24E-07	1.43E-06
AC130324.1	1.080171496	1.34E-06	5.07E-06
C2-AS1	2.451504353	3.21E-09	2.34E-08
GPRC5A	2.200883774	1.42E-12	2.59E-11
RNA5SP268	1.026647735	0.003995986	0.006734206
DIAPH2-AS1	1.012294164	6.97E-05	0.000174608
AC010478.1	-1.402129075	0.001423558	0.002646474
RASGEF1A	2.057937178	7.38E-07	2.97E-06
AC084024.2	1.656222804	1.78E-06	6.51E-06
RPL7P49	1.423465063	1.08E-06	4.16E-06
AC126474.1	1.240439838	2.15E-09	1.64E-08
DLX4	3.003243947	1.14E-09	9.37E-09
PPIAP45	3.129379379	1.18E-10	1.28E-09
AL031667.3	1.039285377	3.40E-06	1.16E-05
AC119424.1	-1.294693242	7.96E-06	2.49E-05
TMEM220-AS1	-1.619055388	2.81E-11	3.59E-10
SNRPEP4	1.035974231	8.79E-10	7.49E-09
LINC02126	2.351884864	1.18E-06	4.52E-06
RUNX3-AS1	1.008508401	6.34E-05	0.000160177
CIT	1.531997887	1.07E-15	6.10E-14
AC104451.1	1.24428739	0.000455185	0.000947573
CLEC12A	1.026741979	0.011031865	0.016844965
TSN	1.000959591	3.69E-18	1.72E-15
DCAF13P3	1.251995752	8.09E-08	4.13E-07
IGKV1D-33	-3.441864894	0.00764923	0.012106623
NPHP4	1.256972755	4.63E-14	1.33E-12
IGKV1OR2-11	-3.174122988	0.010265846	0.015797846
ONECUT3	1.791010418	0.015740506	0.023224154
C5orf34	2.061120251	3.89E-18	1.72E-15
RPL31P50	3.603141932	9.23E-09	6.01E-08
AC025031.4	1.986501582	1.94E-12	3.38E-11
SNORA74A	1.771369262	0.000100832	0.000243418

HS2ST1	1.094267535	3.51E-16	2.57E-14
IL17C	3.001440056	3.90E-05	0.000103872
ACTG1P3	1.592950184	6.02E-14	1.66E-12
AL139099.3	1.907567368	9.25E-08	4.65E-07
TRAJ35	1.507677681	0.007717595	0.012206849
CCN5	-2.762325034	5.38E-15	2.21E-13
CDCA7	2.092178407	4.87E-13	1.01E-11
MIR3125	2.268754228	0.00023964	0.000530493
GRIN3B	2.157724588	5.92E-06	1.91E-05
ZNF587P1	1.427517991	6.01E-05	0.000152663
TRAJ5	1.828367634	0.001555043	0.002866495
TMEM201	1.366592091	5.24E-16	3.56E-14
MKRN5P	1.430280599	0.000450911	0.000939263
NME5	-1.328488555	1.88E-06	6.83E-06
BTNL10	1.382495155	3.03E-05	8.27E-05
SPIN4	1.360857868	1.73E-10	1.79E-09
CCDC160	-1.013052595	3.81E-07	1.65E-06
AC007533.1	1.528680689	1.50E-08	9.25E-08
AL031600.1	1.712089205	4.30E-10	4.01E-09
AC002116.2	1.47989534	1.34E-11	1.86E-10
TAGLN3	-2.141986192	8.61E-07	3.42E-06
CPXM1	2.485566699	1.06E-12	2.00E-11
RNU6-1153P	1.143373393	0.00064717	0.001299216
AC079385.1	1.346992694	1.61E-06	5.95E-06
C20orf144	1.624380902	6.87E-13	1.36E-11
SEMA6D	-1.589232328	0.00017996	0.000410312
RNA5SP370	1.33084599	0.007426269	0.011785243
AC091179.1	3.654218112	0.015314196	0.022649095
RBP4	2.39136971	0.036265551	0.04917732
AC007001.1	1.275303667	0.003786526	0.006412856
TTC22	-1.012541527	0.032030049	0.043941533
DOC2A	2.766986339	7.16E-08	3.70E-07
RBBP8	1.159234813	2.13E-12	3.68E-11
NKX6-2	-3.724016525	2.42E-10	2.41E-09
HOTAIR	5.954109429	3.33E-16	2.49E-14
RPL23AP26	1.100538995	0.015652767	0.023115183
SLC4A11	1.905144302	1.97E-09	1.52E-08
AC069257.1	1.241268758	2.10E-06	7.58E-06
DLL3	3.617321992	0.014499398	0.021550741
AL121895.1	1.70861286	5.44E-08	2.89E-07
MIR579	2.084016246	1.64E-06	6.07E-06

DLEU7	2.253829824	4.12E-18	1.77E-15
ZNF670	1.092124856	5.95E-14	1.64E-12
KRT18P48	1.16988342	1.17E-06	4.50E-06
ASCC3	1.0159762	3.30E-12	5.40E-11
FBXO5	1.315856912	7.46E-14	1.98E-12
DPF1	1.848151438	1.51E-10	1.59E-09
ALOXE3	1.138911258	0.00090844	0.001769458
MIR4489	1.142426941	0.007645339	0.012101902
ZNF667-AS1	-1.212361462	4.92E-07	2.08E-06
AC009945.1	1.014894148	3.30E-05	8.94E-05
MTDHP1	1.058538028	3.83E-06	1.29E-05
AF001548.1	-2.009664904	3.51E-05	9.46E-05
LINC01711	5.227829113	2.82E-15	1.32E-13
PER1	-1.511524514	1.29E-08	8.08E-08
AL159159.1	1.023222664	0.000606976	0.001225451
NOC3L	1.113426827	2.50E-14	7.92E-13
RNU2-18P	2.79390606	2.22E-05	6.30E-05
FANCC	1.004380854	1.14E-14	4.08E-13
HAVCR2	1.674958119	1.53E-10	1.61E-09
AL353807.3	1.176449039	4.85E-08	2.62E-07
HNRNPA3P1	1.027146543	4.61E-07	1.96E-06
ELOVL2	1.940604245	0.000133046	0.000312135
IGKV1OR22-1	-3.580698663	0.001963566	0.003534684
AC090152.1	-1.190230858	8.87E-07	3.51E-06
IGLV3-30	-3.608099604	1.11E-05	3.37E-05
AL589674.1	1.25877826	2.32E-06	8.25E-06
HNF1A	1.33949824	7.71E-09	5.10E-08
E2F8	1.310456287	9.37E-09	6.08E-08
AC080080.1	1.888810723	3.46E-14	1.04E-12
AC009065.2	1.271921805	8.46E-07	3.36E-06
AP005233.2	2.713979764	9.21E-10	7.79E-09
AC068724.1	1.463458672	1.24E-06	4.75E-06
AP001453.1	1.237181275	0.000148156	0.000344165
P3H1	1.281824219	6.96E-16	4.39E-14
CT83	5.688829025	3.96E-07	1.71E-06
OACYLP	2.789950987	4.51E-14	1.30E-12
AC090181.2	1.980060683	1.59E-12	2.84E-11
RNU2-51P	1.587229526	0.000161334	0.000371726
RPL18P10	1.526583361	9.46E-09	6.13E-08
CLCNKA	-2.111884867	7.23E-06	2.28E-05
NALT1	-2.287007882	0.000244351	0.000539846

LINC01355	1.490324423	8.54E-09	5.59E-08
RN7SL180P	1.255805526	0.000150193	0.000348436
RNU6-422P	1.23210566	0.005397964	0.008825023
TMPRSS11CP	1.07015428	0.001317228	0.002468078
AL627309.7	1.145272405	0.00835951	0.013117285
CASC20	2.619962885	9.02E-05	0.000220315
KISS1	1.228991082	0.001294254	0.002428104
H4C10P	1.340630253	0.003201808	0.005511569
SLC22A20P	1.715255446	9.77E-07	3.82E-06
FGFBP2	-1.221616421	5.43E-07	2.27E-06
SNORD123	1.693804273	0.004213791	0.007058398
RAB1AP1	1.490618125	3.44E-09	2.48E-08
RAE1	1.200920614	1.24E-16	1.25E-14
U47924.1	1.786933362	3.01E-07	1.34E-06
PDCD11	1.28797981	6.65E-17	8.40E-15
ZNF385B	-1.342580665	3.41E-06	1.17E-05
SLC35G6	1.053401853	0.000470933	0.000976921
KIAA0319	1.237587193	0.003206988	0.005517636
AL513523.1	1.347662957	6.15E-08	3.23E-07
ESPNP	1.881202603	0.000156524	0.000361738
AC108134.3	1.768008617	1.44E-07	6.92E-07
AC027796.4	1.079074069	1.89E-07	8.82E-07
LIPF	-3.560362467	9.31E-05	0.000226587
SNORA58B	1.410429917	6.52E-05	0.000164508
CDH24	1.430317431	3.07E-15	1.42E-13
AL590438.1	2.136808834	3.76E-06	1.27E-05
IL18BP	1.430405581	5.86E-10	5.27E-09
MARCKSL1P1	1.162818817	7.58E-06	2.38E-05
AL035456.1	1.829961582	4.87E-15	2.05E-13
BICD1	1.295558951	4.54E-13	9.48E-12
AC005183.1	1.174143774	0.001539179	0.00283902
PRC1	2.091369969	1.17E-16	1.23E-14
PELATON	2.154112675	2.99E-13	6.66E-12
AC108704.1	1.180016663	0.00032723	0.000702786
CFAP43	1.907447351	1.03E-11	1.48E-10
PCNX2	1.165263477	1.00E-10	1.10E-09
SUV39H2	1.405230983	6.93E-18	2.40E-15
AP000345.2	1.643477902	6.45E-05	0.000162919
AC020978.3	1.338906446	6.43E-07	2.64E-06
MYOM2	-1.312204386	0.000280271	0.000610497
IFITM10	1.40242715	4.08E-09	2.89E-08

AC069282.1	1.398331134	7.27E-13	1.43E-11
NR4A3	-2.156697512	0.006776404	0.010846317
LARGE2	1.65922955	1.16E-05	3.51E-05
SYNC	-1.207156409	0.00131213	0.002458699
AC026356.2	1.630878669	8.85E-11	9.84E-10
AC012676.1	1.324704805	1.42E-11	1.95E-10
RPRM	-2.741480119	6.74E-11	7.75E-10
ZNF799	1.019036769	8.92E-14	2.31E-12
AGGF1P2	1.903997844	0.00045547	0.000948091
AC063948.1	1.01791678	0.000143473	0.000334186
RNY1	-1.605136025	0.028530034	0.039633808
GAD1	4.65044749	4.14E-15	1.81E-13
SLC16A9	-1.459276665	1.56E-06	5.79E-06
MIR4685	1.258515748	0.001909244	0.003447145
ACTC1	-1.605417021	0.009290392	0.014433182
HSPD1	1.65800331	8.16E-18	2.62E-15
AP000253.1	1.009100606	3.34E-06	1.15E-05
AC074091.1	1.269309722	0.006877434	0.010995476
IGKV3D-25	-3.824588219	1.97E-05	5.66E-05
RN7SL493P	1.045136461	0.00469423	0.007779528
FAM72D	2.578276021	5.12E-15	2.13E-13
AL163051.1	1.211674868	2.15E-09	1.64E-08
GAPDHP64	1.015944931	3.87E-05	0.000103294
ARAP1-AS2	1.363225048	6.10E-10	5.46E-09
DTX4	1.168241535	1.45E-09	1.15E-08
GPER1	-2.010034831	2.61E-09	1.94E-08
MTND2P31	2.109943283	0.00868522	0.013577727
PRDX2P4	1.158751803	0.000267483	0.000585801
AP001775.2	1.462063828	6.15E-06	1.98E-05
LAIR2	1.754849892	3.44E-06	1.18E-05
RNF223	-1.348717992	0.027228697	0.038024275
IFNL3	5.082695399	1.27E-06	4.82E-06
CPB1	-2.461482421	2.85E-07	1.27E-06
C18orf54	1.494887921	3.11E-15	1.43E-13
MT1G	-1.453671661	0.000301396	0.000651717
AC079949.1	2.149643045	0.008320926	0.013062253
AC019176.2	1.060625407	0.003813784	0.006454506
FAM83H	1.122335027	5.02E-08	2.70E-07
UBE2Q1-AS1	1.996120324	2.00E-10	2.03E-09
AC092614.1	1.297002134	1.80E-07	8.43E-07
TTC13	1.11654597	1.12E-14	4.01E-13

POFUT1	1.149360727	4.08E-11	4.97E-10
SYT8	2.200247885	0.022133619	0.031596415
PTF1A	-1.733147881	2.72E-05	7.53E-05
TARBP1	1.520237087	2.48E-16	1.96E-14
AP001107.3	1.444388603	3.17E-06	1.10E-05
IGKV3-7	-3.463463343	0.023771428	0.033683565
HMGA2-AS1	3.363147467	1.89E-14	6.30E-13
GART	1.06595043	1.61E-16	1.50E-14
SNORA55	1.381008535	2.57E-07	1.16E-06
RRM2P2	1.341371856	4.47E-07	1.91E-06
ATP11A-AS1	1.516158755	5.50E-06	1.79E-05
TEAD4	1.873602859	1.42E-18	1.31E-15
RPS29P20	1.255924033	0.00335659	0.005748667
AL137186.1	2.386807478	1.24E-06	4.75E-06
JAG2	1.214331465	2.50E-10	2.48E-09
HMGB1P10	1.541520815	2.56E-09	1.91E-08
WFDC10B	1.996372373	0.000649752	0.001303909
AC020658.1	1.213098493	3.06E-05	8.36E-05
BCAS2P1	2.032649416	0.036396648	0.049346676
TNNI3	3.937880742	5.92E-09	4.01E-08
BOLA3P3	1.357206499	0.006364776	0.010247182
AC091059.2	1.152392175	0.000481689	0.000996827
MLNR	-2.921876313	0.000440234	0.000918962
DENND2C	-2.153505668	0.000786949	0.001551764
ZFP41	1.115011909	1.40E-11	1.93E-10
ABCA11P	1.158009675	1.49E-07	7.14E-07
ERFL	1.044404623	0.000101044	0.000243733
DBF4	1.894178686	1.87E-17	4.01E-15
DHX34	1.39473748	1.33E-17	3.38E-15
CYP4A22-AS1	2.805392386	1.75E-14	5.92E-13
AC132812.1	1.049822926	3.82E-09	2.71E-08
IGHV3-52	-2.999816246	0.029191835	0.040464562
ZNF781	-1.037886097	0.007583683	0.012010015
AC104964.1	1.886361976	0.000795808	0.001567923
P2RX2	-2.254056879	9.50E-15	3.54E-13
AC132192.2	1.483114777	1.59E-10	1.66E-09
RPS24P7	1.139207694	4.03E-05	0.000106918
LDOC1	-1.015781483	3.85E-08	2.14E-07
AC016601.1	1.305614513	0.004706672	0.007797722
AC096720.1	1.2942241	4.78E-06	1.58E-05
CLDN20	1.024026302	3.57E-06	1.22E-05

AC067931.1	1.736913234	3.08E-11	3.88E-10
RPS26P34	1.169620075	0.003466928	0.005917855
SLC16A8	1.635140024	3.62E-08	2.02E-07
AC112721.2	2.058219499	2.19E-08	1.29E-07
RNA5SP33	1.326059171	0.002079523	0.003726051
KNOP1P2	1.194847193	2.50E-06	8.85E-06
AC105460.1	7.95020054	0.000215398	0.000481494
TMEM62	1.103620967	3.28E-13	7.18E-12
IGHV3OR16-12	-2.828684686	0.011328188	0.017257867
PART1	-2.186141765	2.78E-11	3.56E-10
AC010595.1	7.389643454	3.80E-07	1.65E-06
ANK1	1.861418017	0.002780621	0.004844765
AL160281.1	1.597997803	2.75E-05	7.60E-05
KCNH4	1.329857708	3.44E-06	1.18E-05
AC008761.2	1.178390996	0.002546206	0.004472901
ASS1P13	1.596481862	9.46E-07	3.71E-06
CPVL	1.111800173	0.001150456	0.002187287
GPR180	1.116741504	1.67E-14	5.67E-13
PDE11A	1.378799703	0.000856493	0.001675749
GASAL1	1.167137385	5.17E-07	2.17E-06
AC011466.2	2.070751787	3.61E-07	1.57E-06
PLN	-2.556958809	4.81E-08	2.60E-07
AC107993.1	1.32637883	1.76E-06	6.47E-06
RN7SL390P	1.45783365	0.007021053	0.011202905
CHTF18	1.752206734	5.45E-15	2.23E-13
GK-AS1	1.464462048	4.85E-08	2.62E-07
AC006449.5	1.463941027	5.58E-08	2.96E-07
HPSE2	-3.170152606	1.43E-11	1.97E-10
RUSC1-AS1	1.450785604	2.02E-12	3.50E-11
CPE	-1.111362706	1.53E-09	1.22E-08
SLC52A2	1.384723856	2.32E-13	5.29E-12
RPL4P7	1.093615352	0.00371949	0.006311762
PXDN	1.084060823	8.60E-07	3.41E-06
AC100791.3	1.245084478	4.64E-05	0.000121284
TPT1P7	1.352950245	0.000196975	0.000444051
YWHAEP1	1.017414583	6.13E-07	2.53E-06
AC092828.1	1.343287038	2.34E-05	6.60E-05
PLPP7	-1.119575555	0.005210185	0.008547924
PRRX1	1.430625972	8.46E-06	2.64E-05
FOXD3	-1.815319246	1.43E-07	6.88E-07
CCDC107	-1.023114543	4.40E-09	3.07E-08

COL4A1	1.569370387	1.63E-11	2.22E-10
RPL17P40	1.419753258	0.000239212	0.00052959
CCNE2	1.528250234	6.87E-13	1.36E-11
AC024361.1	1.143649433	5.00E-05	0.000129605
RPS14P4	2.116708061	8.58E-11	9.58E-10
WDR4	1.209041286	2.41E-14	7.72E-13
LINC01619	1.324946787	6.50E-05	0.0001641
LINC01836	2.262009359	1.02E-09	8.50E-09
AL139124.1	1.400071993	3.02E-06	1.05E-05
MELTF-AS1	1.553137416	6.33E-12	9.65E-11
PPIAP7	1.449630624	1.64E-06	6.06E-06
TREML3P	1.957359976	3.58E-05	9.62E-05
AC107031.1	2.180569888	0.000538449	0.00110171
LINC02528	2.518413134	5.90E-07	2.44E-06
RNU6-789P	1.383197502	0.001494251	0.002763012
TOMM40P2	2.360071789	2.17E-11	2.86E-10
SLC22A11	4.341106613	1.18E-07	5.78E-07
RPS18P1	1.159851846	0.001166407	0.002214137
AC107884.2	1.045042998	0.00280187	0.004879554
KIF12	1.424310611	1.63E-06	6.04E-06
AC034102.6	1.103612369	2.13E-08	1.26E-07
LINC02268	-2.30804692	0.020629943	0.029631475
RPL35AP16	1.567940169	1.32E-07	6.38E-07
GDPD5	2.17631215	3.22E-14	9.76E-13
RN7SKP292	1.367324489	7.05E-05	0.000176433
AC027807.2	-1.894850446	0.001115176	0.002126585
BAG4	1.044497382	1.30E-13	3.21E-12
AC093157.1	1.067842884	3.31E-10	3.17E-09
FAM86GP	1.438647235	9.89E-06	3.03E-05
AC010271.2	1.733695237	0.000466652	0.000968869
MIR22HG	-1.068793226	1.86E-12	3.27E-11
AL357033.2	3.205728531	2.67E-06	9.37E-06
AC018904.1	1.270026191	9.57E-13	1.82E-11
NDC1	1.655531285	4.84E-17	7.08E-15
MYOT	-2.264756984	4.12E-11	5.01E-10
RN7SL321P	1.359877639	0.000582123	0.001180552
CELSR3	2.894107799	1.32E-15	7.22E-14
AC144836.1	1.506303907	5.83E-06	1.89E-05
AC087163.2	1.634495011	1.03E-09	8.61E-09
FAM24B	1.544084527	1.06E-09	8.78E-09
AC108704.2	1.280988855	1.45E-09	1.15E-08

ILDR1	2.000237711	2.45E-13	5.58E-12
AC004223.1	1.127129369	1.88E-06	6.83E-06
APBB1	-1.075664422	5.35E-05	0.00013767
SMC1B	2.504775425	5.05E-06	1.66E-05
OR6L2P	1.203249038	0.001057094	0.002029773
MIR3136	2.017278578	0.000286779	0.000623246
AC098859.1	1.066744592	2.46E-07	1.11E-06
ZNF473	1.200217475	5.38E-17	7.52E-15
Z99127.1	1.910198452	2.64E-08	1.53E-07
TMC6	1.093797775	2.30E-09	1.74E-08
NAPSA	1.080660882	0.018425644	0.026778094
PUS7	1.881856601	7.52E-18	2.50E-15
SNORA5A	1.195715021	6.36E-08	3.33E-07
AL669818.1	1.906232441	7.03E-08	3.64E-07
FAM72C	2.958478353	1.15E-14	4.10E-13
IGLVI-20	-2.734346956	0.000195874	0.000441943
GBP1	1.078739774	0.000392297	0.000827979
AC022400.4	1.03147532	3.69E-07	1.60E-06
FOXP4-AS1	1.677439121	5.56E-07	2.32E-06
MRPL3P1	1.173415631	1.69E-09	1.32E-08
SP140L	1.023046983	1.76E-11	2.37E-10
AL138759.1	2.592860186	2.88E-09	2.12E-08
AC099314.1	1.443181145	4.50E-06	1.50E-05
ASS1P7	1.694375312	4.69E-07	1.99E-06
ANKRD20A11F	-2.796762428	0.00186067	0.003368838
RAD51AP1	2.291113277	3.56E-17	6.08E-15
CYP4F29P	-3.468444902	0.004884385	0.008066062
RNU6-1170P	1.635571175	3.26E-06	1.12E-05
EMILIN3	-1.885260955	4.00E-13	8.49E-12
RN7SL789P	1.684023755	0.000844291	0.001655279
MTND1P14	1.943257185	0.009611108	0.014880239
RNU6-1048P	1.9018651	7.41E-07	2.99E-06
AC063965.2	1.186252607	4.00E-06	1.35E-05
RPS3AP3	2.759670981	3.56E-09	2.56E-08
PCDHGA1	1.295338243	1.62E-05	4.73E-05
SNRPGP9	1.759269849	5.78E-06	1.87E-05
GGNBP1	1.527328469	6.11E-09	4.12E-08
POLR3GP1	2.623982734	1.95E-09	1.51E-08
AC007996.1	1.710411836	9.28E-12	1.35E-10
IGKV1OR2-9	-3.211687355	0.007063166	0.011262681
KCNG3	2.647957386	2.97E-06	1.03E-05

IGLV2-34	-3.976935923	0.026408002	0.037012047
AC074131.1	-1.33018772	5.10E-06	1.67E-05
SCARNA18	2.410289881	2.51E-05	7.03E-05
POLA1	1.145955103	2.74E-15	1.30E-13
AC090948.3	1.260582911	4.79E-08	2.59E-07
Z99127.2	1.739122866	8.24E-06	2.57E-05
RNU4ATAC11F	1.064859088	0.003688734	0.006264758
RNFT1P2	1.196681159	0.00078028	0.001540211
AC027281.1	1.707292517	1.06E-09	8.77E-09
LINC01697	-2.659414028	7.10E-06	2.25E-05
AC040975.1	2.136336907	3.42E-06	1.17E-05
CCNA2	1.737279007	3.91E-13	8.34E-12
AC091180.1	1.239296631	5.37E-05	0.000138383
NPSR1	5.239970179	9.12E-07	3.59E-06
H2AC18	1.054989695	0.003681454	0.006253191
AP001160.4	1.064179158	5.47E-05	0.000140496
SETSIP	1.109387371	3.82E-09	2.71E-08
PLSCR2	2.123646264	6.10E-10	5.46E-09
RAB3IP	1.725214198	1.76E-16	1.57E-14
MIR4728	3.337978291	1.96E-06	7.12E-06
AC008985.1	1.028007417	4.37E-05	0.000114832
AL928711.1	1.110496531	2.57E-05	7.16E-05
AC011472.1	-1.296777251	0.018896915	0.02736745
MAOB	-1.48164167	3.82E-09	2.71E-08
ITGB1BP2	-1.56104487	0.014250535	0.021220879
POLQ	2.341647814	3.80E-15	1.68E-13
AC211433.1	1.45746307	1.20E-09	9.83E-09
AL353708.3	1.260894775	3.88E-07	1.68E-06
AL049835.1	1.389535205	0.000156524	0.000361738
STPG3-AS1	2.099792189	1.25E-08	7.86E-08
SOX11	1.520660293	6.49E-07	2.65E-06
IFITM3P5	1.470745084	2.28E-09	1.73E-08
AL513185.2	2.284327412	4.48E-05	0.000117452
AL590729.1	1.900196537	3.65E-10	3.45E-09
EIF4HP2	1.611601058	1.84E-10	1.89E-09
ATP6V1G1P4	1.297090524	0.001597674	0.002936546
TNFRSF12A	1.19581959	2.17E-09	1.65E-08
AC118755.1	1.119029928	0.000518102	0.001064657
LINC01719	1.093614746	3.72E-05	9.95E-05
MORC4	1.385262264	2.93E-14	9.04E-13
LINC01569	1.032944081	1.07E-06	4.16E-06

PKD2L1	2.64067721	7.55E-14	2.00E-12
AC006512.1	1.365367565	0.000394091	0.000831568
NUCB1-AS1	1.081641713	0.002084028	0.00373337
AF127577.3	5.442586269	2.80E-10	2.76E-09
LASTR	1.888594414	0.000525281	0.001077249
FAM238B	4.62048819	8.69E-08	4.41E-07
ACP4	1.236696193	4.00E-05	0.000106324
LINC01943	1.074807816	1.11E-08	7.03E-08
AC004223.4	1.089936456	5.99E-05	0.000152392
KRT17	2.439005237	2.70E-08	1.55E-07
LINC00574	1.341789447	0.004523433	0.007517575
TAS2R4	1.50612869	2.77E-08	1.59E-07
C11orf96	-1.745189829	0.000186814	0.00042398
MIR222HG	1.43282533	4.14E-07	1.78E-06
AL158824.1	1.625080043	1.31E-10	1.41E-09
PTGS1	-1.51857605	1.80E-12	3.17E-11
RN7SL455P	1.735656793	0.000140431	0.000327844
AL592045.1	1.01009954	0.02365242	0.033529199
AC239800.2	1.267978847	0.015860996	0.023373453
RPS26P6	1.025856014	8.94E-05	0.000218403
LSAMP-AS1	2.157643567	5.46E-05	0.000140357
AC104564.5	1.414893651	1.02E-06	3.95E-06
AC021739.4	1.12181957	1.67E-05	4.88E-05
SELENOM	-1.266301565	1.94E-05	5.57E-05
CSAG3	7.640981716	5.57E-08	2.96E-07
ARL6IP1P2	1.129735541	1.90E-06	6.90E-06
STUM	-1.057013925	8.41E-08	4.28E-07
AC092834.1	-1.729030996	0.015306044	0.022639553
HM13-IT1	1.281199028	5.89E-09	3.99E-08
LINC01765	-1.173781493	0.000317852	0.00068419
ZNF556	1.661999664	0.000111993	0.000267024
GATAD1	1.08773444	3.28E-10	3.14E-09
IGLJ1	-2.156510613	0.001275062	0.002398517
ZBTB16	-2.45154655	7.73E-11	8.75E-10
RNU6-437P	1.367145898	0.00997949	0.015404924
AF279873.2	1.314925704	0.001004554	0.001939653
SH2D2A	1.085115859	5.48E-08	2.91E-07
PPP1R27	2.219210174	5.01E-08	2.70E-07
KCNA2	-1.45305729	1.88E-10	1.92E-09
DUSP5P1	3.369327238	6.12E-10	5.47E-09
PPIAP85	1.4568924	4.74E-05	0.000123573

FOXC1	1.495477959	7.01E-08	3.63E-07
CDC45	2.009507283	6.81E-15	2.69E-13
CCDC187	2.071012989	0.00061183	0.00123413
CKS1BP4	1.553458695	4.44E-05	0.000116391
IGKV3D-7	-3.761205919	0.000601123	0.001215292
AC073370.1	1.119747219	0.012440329	0.018769694
GDAP1	1.011006495	8.10E-05	0.000199843
AC008147.1	1.17348022	2.23E-06	7.99E-06
STEAP2	1.207941146	2.08E-07	9.57E-07
SLC35E1P1	1.269123329	1.03E-06	4.01E-06
KRT18P21	1.61819846	2.22E-08	1.30E-07
AC087749.2	1.029429197	0.000888286	0.001733752
AL662791.1	1.202977111	0.009391669	0.014574373
EFNA1	1.286935959	4.93E-11	5.86E-10
AP000344.2	2.781548203	0.001380129	0.002574526
CBLIF	-4.569346467	0.001485772	0.002749648
MUCL1	6.341010923	2.90E-06	1.01E-05
RNU6-808P	1.404048552	4.64E-06	1.54E-05
AC015660.2	2.05096736	0.000302422	0.00065388
ERMN	1.882468456	2.67E-09	1.98E-08
AADAC	-1.827897868	0.003373039	0.00577259
MMP20	2.929096828	0.000741953	0.001471752
AC007318.1	1.505657443	1.24E-05	3.71E-05
MIR25	1.708880834	2.06E-10	2.08E-09
RNU6-669P	2.388680672	1.05E-08	6.73E-08
GPAT3	-1.6135027	6.95E-08	3.60E-07
LINC02474	7.229046745	5.37E-09	3.67E-08
CHL1	-1.17434773	5.53E-08	2.94E-07
RNU6ATAC35F	1.445025365	0.000308476	0.000665567
MTND4P26	2.591315715	1.17E-13	2.94E-12
TAS2R15P	1.045753079	6.49E-06	2.07E-05
GBAP1	1.224107825	1.93E-12	3.37E-11
PLA2G4E	1.433827363	2.65E-05	7.38E-05
AC145423.2	1.003678092	6.85E-05	0.000171993
COL4A5	-1.825194207	2.95E-07	1.32E-06
ERFE	2.649039007	1.07E-12	2.02E-11
MRPS9-AS1	1.534526866	1.01E-08	6.48E-08
NUP160	1.130842264	2.71E-16	2.09E-14
CA11	-1.077774261	2.68E-10	2.65E-09
PPP1R3G	-1.267869651	2.54E-07	1.15E-06
PGAM5	1.138533992	6.09E-14	1.67E-12

SULF1	2.784556029	2.73E-13	6.13E-12
PBX4	1.508037101	2.18E-11	2.87E-10
AC007731.1	1.527412978	3.79E-11	4.65E-10
EVX1	5.167229212	4.60E-11	5.52E-10
AC115102.1	1.795956345	6.75E-08	3.52E-07
SEPHS1P4	1.206728664	2.31E-08	1.35E-07
FOXRED2	1.429819962	9.42E-10	7.93E-09
NSG1	-1.19862108	4.65E-09	3.23E-08
STIL	2.593395047	3.95E-18	1.72E-15
AMIGO2	1.054902645	0.0006299	0.001267221
AL139002.1	7.018504838	8.80E-05	0.000215393
PHBP21	1.025452931	1.17E-07	5.72E-07
GGTA1	-1.119209918	1.53E-09	1.22E-08
AC092279.2	1.027489651	0.00108269	0.002073542
SCAANT1	1.12513634	3.93E-05	0.000104641
CNBD2	1.54615879	1.06E-08	6.75E-08
PROCA1	1.014559	5.61E-07	2.33E-06
RPS4XP19	1.039636655	0.000327729	0.000703745
AC105429.1	1.650648703	7.97E-10	6.88E-09
AC106037.2	1.257561489	1.52E-06	5.67E-06
FAM221A	1.250385131	1.17E-08	7.38E-08
IFI44L	1.527089899	4.28E-06	1.43E-05
NCAM1	-2.516817809	1.85E-11	2.48E-10
STAMBPL1	1.382523906	2.79E-12	4.66E-11
RBM46	3.240468858	0.005154364	0.00846886
C1QBPP2	1.309743712	9.25E-08	4.65E-07
SNHG4	1.742183893	1.26E-13	3.13E-12
POLR1C	1.241022296	2.01E-16	1.73E-14
AL645608.4	2.051364842	0.004717915	0.007814891
TFF2	-2.565569349	0.002151369	0.003843411
AC100832.1	2.264416216	0.001710471	0.003124683
MIR559	2.268135577	1.80E-10	1.85E-09
SNORA58	1.660221743	3.42E-05	9.23E-05
AL365356.1	1.611573711	1.64E-09	1.29E-08
TRIM46	1.435470289	1.16E-08	7.32E-08
SORCS1	-2.188889637	1.15E-11	1.63E-10
AL391839.1	1.067637095	0.002873853	0.004994143
PLAGL2	1.095544366	3.54E-07	1.55E-06
AL583856.1	1.043650197	6.89E-08	3.58E-07
CLPSL2	4.426923161	9.56E-07	3.75E-06
ZNF536	-1.359199158	9.48E-12	1.38E-10

IGHV3OR16-15	-3.092134289	0.001425791	0.002650243
MIR3939	2.217969499	4.33E-07	1.86E-06
RPS4XP10	1.712168674	1.53E-05	4.51E-05
AC018641.1	3.789962642	3.23E-09	2.35E-08
LINC00944	1.228158557	0.000291535	0.000632447
GSTM2	-1.478941227	4.40E-12	7.01E-11
AGAP6	1.091719222	4.23E-09	2.97E-08
PATL2	1.158323037	3.60E-07	1.57E-06
AC090578.2	2.667742243	0.002161072	0.003859968
MRPL37P1	1.174815744	2.09E-06	7.54E-06
PGM5P4	-2.090244459	7.80E-10	6.76E-09
ARHGEF26	-1.848320189	1.06E-06	4.10E-06
AC105137.2	1.395369542	8.85E-08	4.48E-07
THRA1/BTR	1.026081509	0.01345511	0.020138823
BRCC3P1	1.464845702	6.34E-11	7.34E-10
LRFN4	2.360404628	2.72E-17	5.18E-15
POLR1B	1.331222978	6.47E-18	2.30E-15
MEGF6	1.070752443	4.77E-05	0.000124217
AC131097.2	3.589243497	1.68E-12	2.99E-11
AL355353.1	1.193533621	2.26E-05	6.40E-05
HLA-Z	2.613563064	1.99E-05	5.70E-05
C5orf34-AS1	1.495324666	2.45E-06	8.67E-06
RNU6-807P	1.505646166	5.41E-05	0.000139039
MRPS6P2	1.384230209	0.000549918	0.00112259
PVRIG	1.183874663	1.97E-05	5.66E-05
AL139147.1	1.916202655	1.13E-07	5.56E-07
TMEM52B	2.033356295	4.75E-12	7.51E-11
TMEM67	1.068128462	1.90E-10	1.94E-09
ARRDC5	1.509820457	0.000231939	0.000515122
HNRNPA1P36	1.204369942	1.23E-09	9.99E-09
IL11	3.60095304	3.07E-14	9.39E-13
AC022144.1	2.734243821	3.85E-12	6.23E-11
CA14	-1.099395731	0.013347186	0.019993013
FAM181B	-1.292608971	2.05E-09	1.57E-08
POLD3	1.392063225	3.48E-15	1.57E-13
KIF14	2.912786838	2.65E-18	1.60E-15
ST8SIA2	1.320883238	0.001085669	0.002079097
TTLL6	1.129644693	5.48E-06	1.78E-05
CREG2	3.108437238	3.39E-09	2.44E-08
TRAJ36	1.547869411	0.002578933	0.00452264
EXOSC3P2	1.541402975	0.001130739	0.002154104

RNA5SP345	1.164348382	0.000499244	0.001030007
AC007611.1	1.403482789	8.09E-08	4.13E-07
NUDCP1	1.345700094	2.00E-08	1.19E-07
PSMB3P1	1.435427736	2.34E-06	8.33E-06
ANGPTL7	-2.454322168	1.97E-06	7.12E-06
NDUFB8P2	1.12436088	2.99E-07	1.33E-06
AC092301.1	1.262893806	1.64E-08	9.98E-08
PLIN1	-1.632306374	2.75E-08	1.57E-07
AL158071.1	1.065542022	0.00020678	0.000464114
SNORA33	1.53800725	2.88E-10	2.81E-09
PRKDC	1.473915509	6.96E-16	4.39E-14
MTG2	1.00307797	5.92E-13	1.20E-11
AC084083.1	1.735286608	9.45E-09	6.13E-08
TNFSF12	-1.113752377	3.77E-05	0.000100717
AL354726.1	1.843457279	4.03E-08	2.23E-07
KRT18P55	1.998301252	2.48E-11	3.20E-10
IMMP1LP1	1.05535	1.50E-05	4.42E-05
MIR1245A	1.783931052	0.002275707	0.004046275
DAP3P1	1.906541142	8.64E-07	3.43E-06
DLGAP1-AS2	2.548989537	2.57E-15	1.24E-13
AC091179.4	3.391934318	5.98E-06	1.93E-05
SAPCD2	2.180028556	2.47E-14	7.85E-13
IGHV4OR15-8	-3.198249972	0.014617653	0.021716585
CR1L	1.374083711	8.45E-06	2.64E-05
FABP6	1.934107978	4.03E-06	1.35E-05
NARF-IT1	1.096492981	3.75E-06	1.27E-05
AC090204.1	1.69525488	0.011484583	0.017466165
AC012640.1	1.635026937	1.82E-07	8.53E-07
AC073842.1	-1.168814013	0.027296985	0.038117631
AP005329.1	1.089487376	5.30E-07	2.22E-06
TRMU	1.057397965	4.52E-14	1.30E-12
AC130371.2	-1.164734687	9.09E-08	4.58E-07
TMEM270	2.732398588	1.71E-08	1.04E-07
HMGN2P22	1.58063295	0.003365615	0.005763013
PCDHGA10	1.164150774	0.026156585	0.036690557
DPPA2	4.545933506	4.07E-05	0.000107895
MIR4326	3.106933787	4.67E-08	2.53E-07
PLP1	-3.349575907	9.74E-15	3.61E-13
AC127002.1	1.484135989	5.74E-06	1.86E-05
LSM12P1	1.16593337	1.72E-09	1.35E-08
AC012146.1	1.653189649	0.000289385	0.000628242

AC010201.2	1.076387121	2.63E-06	9.27E-06
ANO5	-1.157885761	2.77E-05	7.65E-05
MRPS31P5	1.255304816	8.19E-08	4.18E-07
AC093840.1	1.050878957	2.79E-07	1.25E-06
AC103705.1	1.925210545	4.54E-05	0.0001187
VLDLR	-1.010366476	2.97E-05	8.13E-05
AP000577.1	1.161365688	2.72E-05	7.53E-05
AP001372.1	1.929151368	1.30E-08	8.11E-08
RPL23AP91	1.101390694	0.002366008	0.00418922
MDFI	1.677023358	1.26E-10	1.35E-09
U62317.2	1.496327266	1.97E-05	5.65E-05
FNDC11	2.017859292	1.88E-08	1.13E-07
LINC00941	3.677884468	1.06E-10	1.16E-09
AL512637.1	1.918030825	1.43E-09	1.14E-08
AC009041.3	2.070523995	1.26E-13	3.15E-12
AC009065.5	1.755522743	1.81E-09	1.41E-08
AC134349.1	1.30369192	6.99E-06	2.22E-05
PTCD1	1.182752839	6.56E-17	8.37E-15
RNU5B-2P	1.27681129	2.76E-06	9.66E-06
ERVMER34-1	2.208317922	8.37E-05	0.000205877
COL10A1	7.328616929	5.94E-19	1.26E-15
AL359510.1	1.22658532	0.004152505	0.006968446
COL3A1	2.393909985	3.52E-13	7.63E-12
AC011458.2	1.195361188	0.023570895	0.033426082
AC007785.3	2.098034673	3.54E-08	1.98E-07
AP000619.1	2.971769884	6.00E-08	3.16E-07
MIR548N	4.316967453	2.54E-07	1.15E-06
SORBS2	-1.478041528	4.42E-07	1.89E-06
AC245884.9	2.59566495	0.000264067	0.000579081
CNN1	-2.185030178	2.27E-08	1.33E-07
NBEA	-1.302768505	0.002641356	0.004623587
CLEC5A	2.923202262	7.16E-15	2.79E-13
AC105219.3	1.766824431	1.34E-10	1.43E-09
AC092969.1	3.999309978	0.000203412	0.000457402
MMRN1	-1.658094851	5.49E-12	8.52E-11
PLAUR	1.149293268	2.28E-07	1.04E-06
YDJC	1.281639757	6.47E-12	9.84E-11
HMGN1P30	1.847975069	4.89E-07	2.07E-06
RN7SL233P	1.887314078	2.20E-06	7.87E-06
AC093012.1	1.230461315	3.76E-06	1.27E-05
AC009093.4	1.247034649	3.21E-05	8.74E-05

CDO1	-1.149751633	5.04E-07	2.12E-06
DDX50P1	1.679187385	5.52E-13	1.13E-11
ZNF582-AS1	-1.159004913	2.40E-06	8.52E-06
AC098818.1	2.364274009	3.34E-05	9.05E-05
AC009996.1	1.016604544	7.72E-06	2.42E-05
PIMREG	1.924207941	6.39E-14	1.74E-12
FKBP1AP3	1.330863769	1.75E-05	5.08E-05
SLC43A3	1.30970862	2.53E-09	1.89E-08
AL356432.2	1.56399066	6.90E-06	2.19E-05
CBX3	1.28302983	6.13E-18	2.24E-15
RN7SL138P	1.15699694	1.76E-06	6.47E-06
NXPE4	-1.514669996	6.02E-06	1.94E-05
NCBP2	1.014290527	4.61E-16	3.21E-14
RNU6-678P	1.206767533	0.000844726	0.001655889
IGHV3-38	-3.131533097	0.018686787	0.027107338
FHL1	-2.434849432	5.49E-12	8.52E-11
AP000943.3	1.750825606	5.04E-08	2.71E-07
AL359092.1	1.009749685	0.000837337	0.001642637
IGF2BP3	3.707500513	3.05E-12	5.03E-11
RAC3	1.453316634	0.002214194	0.003945066
RNU6-484P	1.169647593	0.001136344	0.002163698
SNHG14	-1.342858982	4.65E-06	1.54E-05
BAAT	6.690013358	1.07E-15	6.10E-14
AC024940.3	1.230935725	1.33E-05	3.97E-05
AL132780.5	1.889641388	1.13E-12	2.12E-11
RN7SL851P	1.595536025	0.000150077	0.000348197
BACH1-AS1	1.030507268	1.35E-05	4.03E-05
RNASEH2A	1.147885964	3.31E-10	3.17E-09
CHD7	1.424792936	1.19E-15	6.65E-14
GRIK3	-2.231944698	1.80E-10	1.85E-09
IGHV3-41	-3.190140041	0.003827906	0.006475527
LRRC36	1.985040188	6.37E-08	3.33E-07
BANCR	6.461700856	6.94E-07	2.82E-06
SIGLEC9	1.084904756	5.14E-05	0.000132786
H2AC9P	2.682473706	2.85E-05	7.84E-05
TUBA3D	1.020757206	1.86E-08	1.12E-07
AP002478.1	5.223735911	5.83E-14	1.61E-12
AL162231.2	-1.163615664	4.52E-05	0.000118359
HMGA1P3	1.513784442	2.38E-09	1.80E-08
AP000907.1	1.325425619	0.00070645	0.001406984
FAM110D	-1.156766236	1.26E-08	7.88E-08

ZNF678	1.194824956	3.80E-15	1.68E-13
RN7SL221P	1.956010996	2.66E-05	7.38E-05
TAP2	1.203728143	6.48E-11	7.48E-10
AL161725.2	1.759709068	6.21E-06	1.99E-05
PCDHB8	2.109072072	5.56E-05	0.000142615
GLB1L	1.15353756	2.27E-08	1.33E-07
RPL12P18	1.706598481	1.54E-05	4.53E-05
EMC3-AS1	1.429919837	2.11E-14	6.89E-13
SPINK7	-4.51919169	1.30E-05	3.88E-05
POGLUT2	1.218814297	1.14E-11	1.62E-10
AC006277.1	1.106158338	1.54E-08	9.43E-08
MMP2-AS1	2.448078874	3.11E-09	2.27E-08
PKHD1	2.041222908	0.000238423	0.000528063
AL139349.1	1.664637458	9.55E-09	6.17E-08
LINC01424	1.483792312	3.08E-06	1.07E-05
AC109479.1	3.002664545	5.65E-10	5.10E-09
C1QTNF4	-1.624954923	6.88E-05	0.000172502
AC131159.1	1.153734329	2.30E-05	6.50E-05
AC093249.4	1.397370325	4.38E-09	3.06E-08
FAR2P4	4.315648154	2.13E-05	6.07E-05
C21orf58	1.134439605	8.29E-10	7.11E-09
KRT18P19	1.720115627	7.88E-10	6.82E-09
AP003086.2	1.293748639	0.001210229	0.002288517
AC007683.2	2.082639097	2.24E-07	1.03E-06
RNA5-8SP5	-1.497012911	0.016432902	0.024118851
IGF2BP2	1.972862507	6.87E-13	1.36E-11
AL133406.2	1.439813657	7.79E-09	5.14E-08
CCNB3	1.427541895	9.57E-06	2.94E-05
RNU6-1025P	1.638435887	0.001107975	0.002114216
PPIAP33	1.481253532	2.98E-07	1.32E-06
ATAT1	1.216018534	8.32E-16	5.09E-14
H2BC17	1.8841411	2.01E-07	9.29E-07
SNORD62B	1.311450801	2.49E-08	1.44E-07
ADGRE4P	1.134272885	0.001988214	0.003575913
AC004000.1	1.250077058	1.53E-05	4.50E-05
TNFSF4	1.647776669	4.23E-09	2.97E-08
AC091564.1	1.091023114	0.000768258	0.001518618
AC103563.7	-3.281790453	3.35E-17	5.87E-15
AL512444.1	2.333545151	7.60E-08	3.91E-07
IGHV3-37	-3.572742355	0.00233406	0.004137603
LCN1P1	2.196369964	1.36E-05	4.05E-05

AC135279.3	1.097240803	1.54E-06	5.75E-06
DMP1	2.938440943	1.61E-08	9.79E-08
CDH3	2.283378052	1.20E-11	1.69E-10
TAS2R6P	1.678675859	7.40E-08	3.82E-07
MIR3133	2.15784412	0.000114984	0.000273445
PPIAP28	1.669900549	2.06E-05	5.89E-05
LINC01977	2.422043563	1.41E-07	6.79E-07
GNGT1	4.554447567	2.39E-06	8.47E-06
AC106875.1	8.18854724	6.51E-07	2.66E-06
AC096656.1	2.995072184	3.44E-08	1.93E-07
LINC02182	2.871350981	0.00303958	0.005257791
AC008763.1	1.466800801	3.49E-05	9.41E-05
PGA4	-4.078045101	3.39E-06	1.16E-05
ARFGAP1	1.044502124	4.65E-13	9.66E-12
AL162727.2	2.23016972	2.28E-11	2.98E-10
AC025171.4	1.459780999	1.14E-08	7.21E-08
AL354993.2	3.17396284	5.67E-07	2.36E-06
CEP43	1.078346762	2.44E-12	4.16E-11
IFITM5	1.69004936	0.018550351	0.026940226
FAM111A	1.247550238	7.71E-15	2.97E-13
AC091812.2	1.895977035	1.88E-05	5.42E-05
DLEU1	1.019655199	1.34E-11	1.86E-10
RPL7P57	1.787813009	8.66E-11	9.66E-10
AC008785.1	2.784311394	0.00339427	0.005804991
AC073347.1	4.5519606	0.003954566	0.006671577
AC134981.1	-2.593656371	0.003384311	0.005790933
PCMTD2	1.049671031	7.65E-11	8.68E-10
CDCA2	2.396198363	9.10E-16	5.46E-14
FABP4	-1.673283752	9.38E-12	1.36E-10
UPK3BP1	1.365539213	2.92E-06	1.02E-05
AC141586.5	1.385899282	1.30E-07	6.31E-07
AC106052.1	1.23075735	0.000193567	0.00043774
AC012354.8	6.092720284	9.21E-07	3.63E-06
AC092916.2	1.063961139	1.55E-05	4.55E-05
GAPDHP14	2.172501863	1.20E-09	9.82E-09
DMGDH	-1.150901156	5.53E-05	0.000141749
RAD54L	2.122398537	9.70E-16	5.73E-14
CFAP61-AS1	4.142594224	3.11E-07	1.38E-06
CENPF	2.679015291	5.83E-17	7.76E-15
LHFPL3-AS2	1.509960591	0.010082115	0.015545378
TWNK	1.568872858	2.80E-17	5.24E-15

ASPG	-1.740071484	0.001527132	0.00281836
AP005262.1	7.164706538	1.76E-06	6.45E-06
MMP8	5.012704552	8.56E-12	1.26E-10
ZGRF1	1.6158341	7.71E-15	2.97E-13
AC024361.3	2.029857676	4.64E-09	3.22E-08
AC025576.2	2.268416982	1.14E-07	5.60E-07
FOXP2	-1.696968965	4.28E-06	1.43E-05
AC004076.2	1.285781394	1.58E-08	9.66E-08
IGHV1OR16-3	-2.833970297	0.008563759	0.013402001
POLR1A	1.062603774	2.33E-15	1.14E-13
SMIM5	-1.639266293	4.43E-10	4.12E-09
SPARC	1.777353139	2.02E-12	3.50E-11
IDI2-AS1	1.156906604	3.53E-06	1.20E-05
CCNI2	1.477213017	8.08E-09	5.32E-08
ALDOB	-4.101355046	0.03475867	0.047316985
AC013262.1	1.34630329	0.009574208	0.014829141
LINC01082	-1.746815098	2.64E-12	4.46E-11
AL163953.1	2.217231659	8.50E-05	0.0002086
LINC02106	-2.309394274	5.92E-07	2.45E-06
AC005229.1	1.646463816	5.95E-06	1.92E-05
FXYD6	-2.26741286	1.75E-05	5.07E-05
DDX39B	1.004862083	4.10E-08	2.26E-07
MIR181A2	2.572760065	8.20E-06	2.56E-05
MYBBP1A	1.078889033	8.15E-13	1.57E-11
PHGR1	-2.631807856	0.011587384	0.017608458
RN7SL558P	1.55107038	6.09E-08	3.20E-07
CTTNBP2	-1.125968189	0.00071857	0.001428881
AL731571.1	1.062822135	6.84E-09	4.57E-08
TNFSF9	2.157843531	7.98E-06	2.50E-05
LENG8-AS1	1.155224916	6.05E-09	4.08E-08
AC004080.1	4.504738072	3.43E-06	1.18E-05
NPM1P21	1.144046059	6.01E-06	1.94E-05
SLC9C1	1.672575686	1.24E-08	7.79E-08
ISYNA1	1.448368208	9.64E-06	2.96E-05
AC100861.1	1.264029447	9.89E-10	8.28E-09
CCDC194	1.952216332	5.18E-07	2.17E-06
SNORD100	1.601855018	1.09E-08	6.94E-08
AZU1	3.959386436	0.00037538	0.000795361
AC087477.1	1.256084729	0.000461244	0.000958837
AC099518.2	1.975807169	4.60E-11	5.53E-10
ATP5MFP4	1.451324355	4.51E-08	2.46E-07

AC010976.1	1.60952021	1.37E-11	1.90E-10
IGLV3-6	-2.931777356	0.008172165	0.012847279
AL023284.4	1.44814901	5.73E-08	3.03E-07
RN7SKP173	2.228260459	1.50E-10	1.58E-09
LINC01798	-1.447678492	0.007457653	0.011829411
GLRX5P2	1.906086691	3.43E-08	1.93E-07
NLRC4	1.01739298	5.44E-08	2.89E-07
RCOR2	2.158132457	7.15E-10	6.27E-09
RFC4	1.50505356	7.01E-17	8.73E-15
AC068790.2	1.121732873	3.44E-07	1.51E-06
PSCA	-3.198621338	5.44E-06	1.77E-05
AC024937.1	1.186630406	0.000222973	0.00049686
AC005920.2	1.487036242	5.77E-06	1.87E-05
AL354993.1	1.696425516	0.000330876	0.000709586
MTHFD2	1.560418935	5.74E-16	3.80E-14
EVA1A	2.885207666	1.04E-12	1.96E-11
AC027801.1	1.510156299	4.90E-09	3.38E-08
SNORC	2.072832022	6.00E-09	4.05E-08
ZNF385C	1.4256944	0.000698361	0.001391916
AC025178.1	1.066257215	2.49E-05	6.98E-05
FDXACB1	1.090214026	3.85E-12	6.23E-11
FAM9C	5.825499551	1.23E-06	4.68E-06
PTBP1P	1.318499176	5.68E-08	3.00E-07
NRIR	2.113434869	2.09E-08	1.24E-07
AC024267.5	1.397771427	7.19E-07	2.91E-06
PCAT1	2.328108041	1.19E-12	2.20E-11
HMGB1P24	1.695222187	3.12E-10	3.02E-09
RN7SKP180	1.55851613	3.00E-06	1.04E-05
AL049539.1	1.873739781	3.30E-09	2.39E-08
AL118505.1	1.899028313	5.65E-11	6.63E-10
BEND3P1	1.265701795	0.0091849	0.014288457
AP002768.1	1.597314376	0.002765132	0.004820616
LINC00427	1.01115014	0.007932735	0.012511481
GAPDHP59	1.185567657	3.31E-06	1.14E-05
AC130456.3	1.782191043	3.47E-09	2.50E-08
RDH12	-2.731570628	9.29E-09	6.04E-08
GSTA3	-3.401161474	5.08E-05	0.00013153
ALDOC	-1.11129069	7.01E-10	6.17E-09
AC010210.1	1.123424882	1.65E-05	4.82E-05
AC004130.1	1.440704793	0.001319389	0.002471083
KCNIP2-AS1	1.160051053	2.69E-07	1.21E-06

PMFBP1	2.121292143	6.47E-10	5.75E-09
ELOVL4	-1.893587207	0.001370519	0.002558033
LINC00628	1.077009479	3.52E-05	9.47E-05
PROSER2-AS1	1.656518244	4.09E-06	1.37E-05
ALOX12P2	1.908657424	9.23E-05	0.00022482
MTND4P9	1.284684167	5.44E-06	1.77E-05
MT-TE	-1.628222102	9.68E-06	2.97E-05
PDCD2L	1.25673546	1.63E-13	3.89E-12
AP000424.1	3.505829978	1.92E-07	8.91E-07
AC083899.1	1.118600461	9.17E-15	3.43E-13
RNU7-151P	1.343176546	0.010375305	0.01594663
AC007405.2	5.911213966	3.72E-10	3.51E-09
VEZTP1	1.149824793	1.42E-05	4.21E-05
AC012676.5	1.542870295	4.03E-12	6.49E-11
AL390067.1	1.659899758	9.45E-06	2.91E-05
AL359697.1	1.082175351	2.03E-06	7.32E-06
AC013452.1	1.359027235	1.36E-05	4.05E-05
AC068790.1	1.81032839	2.46E-06	8.70E-06
AP000695.3	1.761504696	6.56E-13	1.31E-11
PAICSP4	1.722518887	1.90E-13	4.46E-12
AC026310.1	2.97020746	6.41E-07	2.63E-06
AL139095.4	1.204328258	5.82E-06	1.88E-05
SYCP2	1.781366666	0.013464632	0.020146284
RN7SL377P	1.026616818	0.036828589	0.049862144
SH3GL1P2	1.000117235	8.65E-05	0.000212004
BMPER	-2.004839335	1.18E-07	5.77E-07
AL356057.1	1.152808632	8.04E-05	0.000198428
SPEG	-1.925173114	0.000150987	0.000350005
AC129510.1	1.478520481	6.60E-10	5.84E-09
RPL7P13	1.186451918	1.19E-06	4.54E-06
CCNYL6	1.122794324	2.06E-07	9.50E-07
TTLL2	2.464652465	1.51E-07	7.21E-07
RN7SKP239	1.188727304	1.45E-05	4.30E-05
ELOCP27	1.20442569	1.42E-05	4.20E-05
AC008870.5	1.937046148	1.55E-06	5.78E-06
AP000786.1	1.809724646	5.70E-07	2.37E-06
LINC01731	2.210724679	0.03425115	0.046690501
NCR3LG1	1.999593631	1.82E-11	2.43E-10
HHLA3-AS1	2.256202248	5.84E-05	0.000148859
MIR302A	2.012832797	3.16E-05	8.60E-05
TMEM105	1.657271521	2.23E-05	6.32E-05

DTX3L	1.277753271	1.03E-15	5.96E-14
C19orf48	1.379775556	4.86E-12	7.64E-11
IGLVIVOR22-1	3.610268627	2.32E-06	8.25E-06
SGO2	1.935284165	3.47E-16	2.55E-14
TUBAP11	1.413515117	9.16E-09	5.96E-08
TPSB2	-1.020224694	6.31E-05	0.000159623
KRT18P16	1.602488255	7.66E-10	6.64E-09
MIR5009	1.831850787	0.000189538	0.00042965
HMGB1P6	2.079888449	4.00E-14	1.18E-12
IFITM3P7	2.049744659	1.74E-08	1.05E-07
AC079741.1	1.995033448	3.69E-06	1.25E-05
AC012531.6	5.456700546	4.93E-07	2.08E-06
THBS2	2.838929337	1.06E-11	1.51E-10
AC087277.2	2.337453973	9.90E-05	0.000239414
LINC01819	2.91001605	0.001407548	0.00262072
AC022405.1	2.354313991	1.42E-09	1.14E-08
AP002954.1	1.55146978	5.16E-05	0.000133449
MIR593	1.363226886	3.01E-07	1.34E-06
AC124947.2	1.820318071	2.45E-10	2.43E-09
FBXO41	1.716547055	3.56E-12	5.81E-11
SNORA51	2.194565047	4.53E-06	1.50E-05
MACC1	1.612479345	4.90E-10	4.49E-09
ZIC2	3.813886342	1.91E-07	8.88E-07
RNY1P9	1.2403615	5.82E-05	0.000148406
RPS24P18	1.261525544	0.000951737	0.001847848
SLC30A3	1.892113114	0.01945957	0.028095918
SNORA1B	1.514538009	0.000122935	0.000290743
SNORA15B-1	1.291217967	0.000336498	0.000720253
CBX5P1	1.209483299	4.84E-05	0.000125892
UBD	2.940480374	3.89E-10	3.65E-09
DUXAP8	3.953975109	1.76E-15	9.09E-14
RN7SL269P	1.205784678	0.001056893	0.002029533
DNAJC5B	1.442301999	1.16E-06	4.47E-06
AL731563.3	1.21950199	1.94E-07	9.02E-07
TCAP	1.923432437	3.91E-06	1.32E-05
AC022509.2	1.463838576	0.000226228	0.000503775
AC100821.1	1.302966371	2.03E-09	1.56E-08
RB1-DT	1.107073465	1.63E-05	4.76E-05
LINC02100	1.908660723	3.74E-09	2.67E-08
AC008434.1	1.086511476	1.42E-06	5.32E-06
AL831737.1	1.567874922	0.000189924	0.000430415

CATSPER2	1.303466611	1.19E-08	7.51E-08
MCF2L	1.188586132	8.23E-09	5.41E-08
PFN1P4	1.312894205	9.75E-06	2.99E-05
TMEM75	1.853620364	1.88E-08	1.13E-07
PGAM1P7	2.28941303	4.58E-11	5.50E-10
AC107081.1	1.516807482	8.72E-11	9.72E-10
LACTB2	1.46202464	1.17E-11	1.65E-10
GAPDHP35	1.11157886	7.74E-05	0.000191901
AC027020.2	1.019679281	0.000356625	0.000759307
GTSE1	2.152041832	1.13E-15	6.36E-14
SSC4D	1.851100264	6.91E-12	1.04E-10
RNA5SP425	1.153698861	0.000123113	0.0002911
LINC01655	3.160481844	1.08E-08	6.85E-08
ZNF765	1.212378745	2.44E-16	1.95E-14
LINC01356	2.377742307	5.04E-11	5.96E-10
NEXN	-1.600695355	0.005628144	0.009161423
NUP107	1.53653584	2.44E-18	1.59E-15
KDM1B	1.029050608	8.23E-12	1.22E-10
RPL22P20	1.436932539	3.61E-05	9.70E-05
CHRM2	-2.546079572	3.63E-05	9.73E-05
PFAS	1.248182643	2.61E-15	1.25E-13
PRIM2	1.423357109	8.68E-19	1.26E-15
Z95152.1	1.114257969	5.45E-05	0.000140169
CTSB	1.214858985	7.38E-12	1.11E-10
KCNJ10	1.439730413	0.023828271	0.033749749
RDH8	6.940427328	3.51E-13	7.61E-12
PLXNA3	1.299559066	3.06E-13	6.78E-12
RPL7AP65	1.586841907	2.62E-07	1.18E-06
AP001542.1	2.994233014	9.66E-09	6.24E-08
LINC01397	1.726982452	1.07E-07	5.33E-07
EFCAB10	1.234829995	3.99E-11	4.88E-10
AP001271.2	1.917508451	3.36E-07	1.48E-06
MIR5190	1.010622966	0.023156551	0.03289628
PABPC3	1.015331712	1.63E-08	9.90E-08
C16orf89	-3.212754561	2.32E-13	5.29E-12
GCC2-AS1	1.239815021	1.10E-08	7.00E-08
MCTS2P	1.682057198	3.11E-14	9.48E-13
AARSD1	1.018423918	2.44E-12	4.16E-11
GAS1RR	-1.995630431	3.14E-08	1.78E-07
MIR4635	2.037606297	2.09E-08	1.24E-07
KDELC1P1	2.625649269	2.59E-10	2.56E-09

H3C8	1.257093503	0.007136295	0.011365684
CARD9	1.074734736	1.76E-05	5.10E-05
MIR6769A	1.127432658	0.001817093	0.003297302
ALDH3A1	-2.559788333	3.29E-06	1.13E-05
PSMD3	1.231912345	1.59E-12	2.84E-11
CHGA	-1.343468862	3.34E-07	1.47E-06
IGLV4-3	-4.774302439	0.003343896	0.005733562
LINC01410	1.195497629	0.000369631	0.000784553
SS18L1	1.09770976	8.92E-14	2.31E-12
HMGN2P20	1.460288141	4.41E-08	2.40E-07
EIPR1-IT1	2.45951766	1.11E-10	1.21E-09
SCAT1	2.616404602	3.53E-11	4.36E-10
WDR27	1.073785383	1.53E-09	1.22E-08
AL606468.1	1.682003218	1.77E-05	5.14E-05
CENPW	1.460333135	1.17E-11	1.65E-10
FKBP5	-1.201712052	0.000548306	0.001119469
AC006978.2	1.967119715	8.48E-08	4.31E-07
AC016542.3	1.180834293	0.002428563	0.004289431
AC011466.3	2.509809094	8.72E-12	1.28E-10
PPIL1	1.041444554	1.09E-14	3.94E-13
DGKG	-1.769839118	6.37E-08	3.33E-07
AC073857.1	1.02323778	7.86E-09	5.18E-08
AL590666.2	1.133587063	2.73E-05	7.56E-05
LIF-AS1	1.934013418	1.41E-06	5.32E-06
TRAJ34	1.831773997	0.009437517	0.014632737
POU5F1P6	1.017233617	0.026753236	0.037442724
DLGAP5	2.092889892	1.70E-15	8.82E-14
AC087501.1	1.310467732	2.72E-05	7.53E-05
THORLNC	1.235261807	7.22E-06	2.28E-05
PHF6	1.26026121	1.53E-15	8.08E-14
AC061992.2	1.357582168	5.53E-08	2.94E-07
CSAG2	6.979019848	1.90E-08	1.14E-07
RNA5SP256	2.207165587	0.000228238	0.000507785
HACD1	-1.536419543	5.32E-06	1.74E-05
OSCAR	1.661442034	3.12E-10	3.02E-09
KHSRPP1	1.833803996	0.003095146	0.00534246
MSH5	2.298147943	2.16E-15	1.07E-13
SPINK2	-2.960020575	2.42E-05	6.78E-05
AC126124.2	1.127937546	8.10E-05	0.000199838
AC097639.1	1.382729677	2.19E-08	1.29E-07
TM4SF1	1.334799671	3.36E-06	1.15E-05

KRTAP5-AS1	1.209075909	2.54E-05	7.09E-05
AC091179.2	4.563227361	1.33E-08	8.31E-08
PRDM8	-1.528726208	3.15E-05	8.59E-05
MAGEA12	9.62805065	8.38E-09	5.49E-08
PPIAP3	1.657141771	5.89E-11	6.89E-10
TAS2R38	3.871844284	3.03E-11	3.82E-10
HOXC9	4.851250542	1.35E-17	3.39E-15
AC093423.1	1.088148901	0.028214191	0.039240054
SYNGR4	3.495252999	1.78E-07	8.33E-07
INHBA	3.765640856	2.88E-18	1.63E-15
NPM3	1.072639268	7.32E-08	3.78E-07
IGHV3-75	-3.71561598	0.000129258	0.000304234
AC004830.2	1.583804936	9.73E-08	4.87E-07
AL391988.1	1.680943555	3.28E-10	3.14E-09
TMEM33	1.010622118	2.32E-14	7.49E-13
AC100797.1	2.112194105	1.75E-06	6.41E-06
RNU6-1161P	3.064979099	1.44E-08	8.89E-08
PRR7-AS1	2.076534529	8.31E-14	2.17E-12
CHST1	1.783213388	9.04E-13	1.73E-11
CEP295	1.032494698	4.25E-11	5.15E-10
CBR1	-1.916691307	8.04E-10	6.93E-09
PRKX	1.053507475	2.58E-09	1.92E-08
HNRNPA1P49	1.263026618	8.04E-09	5.29E-08
ETF1P1	1.529973987	3.60E-08	2.01E-07
NF1P8	4.540032413	0.001180798	0.002238583
ASB2	-1.815782374	3.99E-08	2.21E-07
NPSR1-AS1	5.577198283	7.97E-15	3.05E-13
RN7SL376P	2.576979405	5.14E-06	1.68E-05
BUB3	1.061394397	2.78E-16	2.14E-14
AC117503.2	1.643227991	7.21E-10	6.32E-09
FLNA	-1.64947526	0.013882917	0.020715131
GAPDHP45	1.531413636	8.32E-06	2.60E-05
AC011479.3	1.24683554	2.99E-07	1.33E-06
IGKV2OR2-7	-2.3588685	0.000419755	0.00087966
GTF2IP23	1.84179314	4.19E-12	6.70E-11
SLC6A14	2.458786485	1.03E-05	3.14E-05
AL445649.1	2.602834027	0.001348224	0.002519952
BNIP3P40	1.033024706	0.024325268	0.034360521
RPF2P1	1.833518465	9.54E-09	6.17E-08
LINC00479	1.702937512	0.011634504	0.017673991
AC016954.1	1.703893909	5.66E-07	2.35E-06

GPR155	-1.459790589	2.04E-08	1.21E-07
Z97192.2	1.432907273	3.62E-05	9.70E-05
PTPRO	2.869046746	2.00E-08	1.19E-07
ATAD3A	1.083973225	1.30E-11	1.81E-10
MIR581	1.645919265	0.00054575	0.001114765
AMELX	2.561182771	3.36E-06	1.15E-05
LINC02185	-1.06838559	0.00014731	0.000342433
FAM186B	1.136640151	1.90E-08	1.13E-07
KRT18P68	1.455637851	1.67E-08	1.01E-07
SBSPON	-1.499420405	2.44E-07	1.11E-06
AC073529.1	1.244967783	1.63E-09	1.28E-08
LINC01952	1.102321421	0.003142425	0.005418113
IGBP1-AS1	1.537579064	3.64E-09	2.60E-08
AC120114.3	1.487491272	2.87E-11	3.65E-10
PPFIA1	1.089760194	3.80E-17	6.26E-15
AC126615.2	1.739391334	3.17E-08	1.80E-07
AL355987.4	1.52669319	4.25E-11	5.15E-10
FAM229B	-1.196223592	0.00026054	0.000571913
AC022150.2	1.065138748	0.000112713	0.000268477
LINC02577	4.33723236	3.56E-15	1.59E-13
FMN2	-1.210544446	0.001602723	0.002945216
IL37	4.215475971	2.97E-11	3.76E-10
AC008147.3	1.284815198	5.38E-07	2.25E-06
CTAGE3P	1.293937286	3.59E-05	9.65E-05
UPF3B	1.05333339	2.19E-10	2.20E-09
RNU6ATAC9P	1.876554464	7.72E-05	0.000191333
NPM1P26	1.24506331	5.31E-09	3.63E-08
KAT2A	1.348864942	6.74E-18	2.36E-15
IGLC5	-1.59587759	0.023478692	0.033309512
AGAP13P	1.176520554	1.86E-05	5.36E-05
MIR6859-4	1.04323837	0.030772292	0.042399431
AL139123.1	1.384808628	4.08E-06	1.37E-05
GUCY1A2	1.275862182	3.56E-08	1.99E-07
AC093458.2	1.945472809	1.12E-07	5.52E-07
RN7SL683P	1.759773339	3.81E-05	0.000101735
AC106820.5	1.059833394	0.000124777	0.000294551
AC018629.1	1.666080043	0.000610261	0.00123115
MIR6753	1.431437874	4.59E-08	2.49E-07
RR5-ARHGAP	3.070728299	2.60E-17	5.12E-15
LINC02700	5.357275424	7.03E-09	4.68E-08
HNRNPA1P28	1.074057136	6.67E-07	2.72E-06

AP001469.2	1.136380088	1.86E-07	8.69E-07
AC132192.1	1.323298501	1.85E-07	8.62E-07
H3P25	1.079036478	2.88E-05	7.92E-05
LINC02870	2.063894386	3.10E-07	1.37E-06
NOP56P3	2.281522585	8.00E-12	1.19E-10
HAUS6	1.270223928	4.64E-15	1.97E-13
CDCA4	1.014888164	8.12E-10	6.99E-09
CATSPER1	1.393223202	0.001131596	0.002154812
GTF2IP14	1.090846868	6.59E-07	2.69E-06
DNAH14	2.103898996	3.21E-06	1.11E-05
C6orf99	1.330680226	3.60E-07	1.57E-06
AC245517.5	-1.258657843	0.011768962	0.017856865
AC138409.1	1.745017523	5.15E-10	4.71E-09
PKMYT1	2.222142706	3.52E-15	1.58E-13
ALMS1-IT1	1.416393222	4.39E-10	4.08E-09
IGKV1OR-2	-4.198377055	0.002140003	0.00382567
AP001525.1	2.268272307	7.15E-05	0.000178892
ENPP7P4	1.15610838	0.000227681	0.000506642
AC093866.1	4.886789333	4.48E-07	1.91E-06
AC107909.1	2.201907881	2.17E-08	1.28E-07
DMPK	-1.602568618	7.11E-05	0.000177803
AL137186.3	2.230839328	8.46E-05	0.000207796
SPOCD1	1.999943284	5.25E-12	8.19E-11
NLRC5	1.442772179	3.09E-10	2.99E-09
CKS1BP6	1.582436545	5.45E-06	1.77E-05
PPIAP47	1.291959724	8.96E-07	3.54E-06
Z99943.2	1.210361972	8.67E-06	2.70E-05
DDX12P	1.912004732	1.36E-14	4.75E-13
RNU6-1156P	2.388468415	5.74E-05	0.000146517
BRCA1	1.757650456	2.20E-16	1.84E-14
AKR1C1	-1.935686204	9.79E-13	1.86E-11
AL021331.1	2.379848129	1.53E-06	5.70E-06
AC087878.1	1.565255537	0.000515795	0.001060406
AL023584.2	1.0252151	0.001447174	0.002685679
COL5A3	1.434739852	6.90E-09	4.60E-08
RNVU1-27	1.129715423	0.002028442	0.003642855
AQP6	1.302948864	0.036260902	0.04917732
MFSD12	1.121156962	7.29E-14	1.95E-12
AC092755.1	1.113401834	0.001092914	0.002090566
DDX10	1.104779033	8.50E-18	2.70E-15
AL049745.2	1.532303209	4.66E-06	1.54E-05

AC130456.2	1.162410159	0.020715019	0.029751457
AC005021.1	2.313656608	3.16E-08	1.79E-07
AP001781.1	1.36222435	0.000917866	0.001786383
MIR661	1.97279417	7.67E-05	0.000190372
AC024382.1	2.389857918	0.000141398	0.000329812
AC108463.3	3.359717485	1.22E-17	3.21E-15
AP005131.2	1.159607482	0.001772239	0.003226679
AL590233.1	1.230211985	0.00109899	0.00210023
AL606970.1	1.41037517	0.003352043	0.005746052
C1QTNF6	2.286836572	3.89E-18	1.72E-15
RPS26P47	1.096107723	0.013288803	0.019913398
AL160163.1	1.46835298	0.000226295	0.000503872
BDNF	1.164074385	0.001355701	0.002532861
AL390728.2	1.669513272	1.90E-07	8.85E-07
SLC6A6	1.232371042	7.06E-12	1.06E-10
AC011997.1	2.022897612	1.85E-06	6.74E-06
MIR548AT	1.133827393	0.000299236	0.000647676
RN7SL470P	1.474219414	0.002909535	0.005052524
MIR553	1.407335641	4.09E-05	0.000108464
TTLL10	1.415833609	9.16E-06	2.83E-05
LRRN4	2.800268765	2.98E-08	1.70E-07
AL590128.1	1.281757441	2.44E-05	6.83E-05
TONSL	1.867392731	1.12E-15	6.29E-14
IGHV7-34-1	-2.83955124	0.009451859	0.014654121
AP001065.4	2.502108891	2.20E-06	7.87E-06
DNAH3	1.821736825	2.03E-09	1.56E-08
HSP90AA3P	1.849259475	6.56E-13	1.31E-11
MXRA5Y	2.09877511	1.07E-05	3.27E-05
RPS26P41	1.058525226	0.016151794	0.023752062
AC087385.1	1.34884983	4.11E-07	1.77E-06
AC116407.3	1.212295216	3.85E-08	2.14E-07
AC079834.1	-1.284091839	0.007542248	0.011952211
AC015883.1	1.649155992	4.36E-08	2.38E-07
CD177P1	3.619987963	0.000155771	0.000360185
SORBS1	-1.828521969	2.74E-06	9.59E-06
AL121924.1	1.701055483	0.000824508	0.001619375
AL121790.1	1.7933639	0.000107385	0.000257418
PRELID3A	1.063978937	6.54E-07	2.67E-06
RNU6-1203P	1.302903516	0.010192989	0.015699925
TOMM20P2	1.167912479	0.001262879	0.002377883
AC114763.2	2.113363845	1.61E-09	1.27E-08

AC002128.1	1.503531362	1.42E-10	1.51E-09
DPY19L1	1.63358856	5.52E-17	7.55E-15
CYP3A4	-4.200709284	0.002338786	0.004145704
AC097484.2	1.269863455	0.004113258	0.006910418
AC023302.1	1.882132045	6.27E-08	3.29E-07
AL365181.3	1.836130887	2.65E-08	1.53E-07
AC134407.1	1.029099813	2.16E-05	6.15E-05
CPEB1	-1.636424084	7.45E-08	3.84E-07
PVR	1.488536039	6.57E-15	2.62E-13
AC134775.1	1.506445268	1.10E-05	3.33E-05
LINC01389	2.105773211	1.44E-12	2.61E-11
ZBTB12BP	1.103143113	6.81E-07	2.77E-06
HNRNPA1P37	1.113509412	4.55E-07	1.94E-06
AC053503.3	-2.085629679	2.66E-07	1.20E-06
RBP2	-5.545127996	0.03240732	0.044421488
PLK4	1.913156651	2.16E-15	1.07E-13
AL020995.1	1.491438034	1.53E-06	5.70E-06
VMA21	1.164805654	1.16E-15	6.51E-14
LINC01126	1.235796609	1.28E-07	6.22E-07
HIGD1AP6	2.519493949	1.39E-05	4.13E-05
AC018620.1	1.188452923	0.001407447	0.002620715
AC093166.2	1.417537476	0.000260901	0.000572658
RNU6-606P	1.125961909	0.011110903	0.016957875
AP005136.1	1.812539863	0.000341138	0.000729421
RPS15P9	1.260861747	0.000215151	0.0004812
AC004080.12	5.490724341	3.50E-10	3.33E-09
PPIAP23	1.873743171	5.04E-08	2.71E-07
CBX2	2.852978011	1.97E-13	4.59E-12
MAGEA1	8.396114865	6.68E-05	0.0001681
RN7SL270P	1.310489989	0.001058508	0.002032071
NPM1P31	1.709857045	7.81E-05	0.000193489
AC124067.3	3.556653512	5.78E-07	2.40E-06
AC114760.2	1.008008374	0.000610261	0.00123115
TUBBP6	1.532470573	1.02E-09	8.54E-09
NMRK2	-1.625607292	4.78E-05	0.000124396
AC244100.4	2.455727338	4.70E-05	0.000122645
C7orf61	1.087744444	5.47E-05	0.000140552
SLC9A4	-2.370793786	3.59E-05	9.65E-05
LINC02663	1.7766808	0.002902094	0.005040918
NPY6R	-2.736372286	9.54E-05	0.00023151
NSUN5P2	1.098146598	2.87E-08	1.64E-07

AC010275.1	2.727840431	2.31E-05	6.52E-05
THEM5	1.30445051	0.002035949	0.003654614
CLSPN	2.705789249	1.48E-18	1.31E-15
AC011442.1	1.330561039	5.15E-05	0.00013305
TMEM25	-1.294818037	7.36E-10	6.43E-09
MIR548C	1.379286913	4.89E-05	0.000127154
ATOH8	-1.435591167	8.76E-11	9.76E-10
RNU4-82P	1.394584279	1.78E-06	6.51E-06
ODF3L2	1.213408206	0.020372502	0.029297072
RSPO2	-2.361722397	4.68E-11	5.61E-10
AC068580.1	1.376832574	6.46E-09	4.34E-08
KRT2	-2.429130441	1.58E-05	4.64E-05
MIR17HG	2.155108325	1.10E-11	1.57E-10
RNASE1	-1.967160316	3.14E-06	1.09E-05
SSBL5P	1.423350268	9.72E-09	6.28E-08
AL096701.3	1.078020387	9.09E-07	3.58E-06
C4orf46	1.110606666	2.89E-14	8.96E-13
AC093297.2	1.066394419	1.34E-09	1.08E-08
LIPG	2.163026597	1.78E-11	2.39E-10
RELT	1.22687867	3.48E-11	4.31E-10
HPSE	1.115412364	3.11E-08	1.77E-07
ADAM12	3.987387431	3.17E-18	1.69E-15
AC025449.1	1.060242379	6.59E-08	3.44E-07
AC008742.1	1.500210353	5.68E-07	2.36E-06
RPS3AP54	2.000651972	0.000576601	0.00117069
ACTL6A	1.291566037	8.01E-17	9.44E-15
AL391832.1	2.705571688	1.20E-08	7.58E-08
TRABD2A	1.88153891	7.07E-08	3.66E-07
RNA5SP493	1.575789404	0.000119486	0.00028324
PSMG1	1.062713592	4.45E-12	7.08E-11
C4orf48	1.46705038	3.26E-07	1.44E-06
FARP1-AS1	1.055586917	0.006344639	0.010215996
RNU6-1079P	1.617463979	0.000102907	0.000247889
RNU6-549P	1.367641628	9.27E-05	0.000225631
CEACAM6	1.848321311	0.00029958	0.000648158
AC009118.2	1.137461213	0.000446462	0.000931014
SOCAR	1.696424071	1.17E-11	1.65E-10
ABL2	1.039560242	5.88E-14	1.62E-12
ZDHHC20-IT1	1.589423752	4.19E-09	2.95E-08
IL27	1.658890758	5.37E-07	2.25E-06
LINC00431	1.150444887	0.020799992	0.029867399

AC073365.1	7.853328103	1.67E-06	6.17E-06
AC007000.2	1.245142641	3.20E-06	1.10E-05
NUF2	2.424885752	1.63E-16	1.50E-14
SNORD14A	1.47015756	9.33E-08	4.69E-07
CCNB1	1.736457766	1.27E-13	3.16E-12
GHET1	1.239219803	5.21E-09	3.57E-08
RNU6-444P	1.733179475	0.00030251	0.000654019
CNOT10-AS1	1.607284479	5.05E-05	0.000130803
RNU6-890P	1.493078882	2.17E-05	6.16E-05
RPL36AP52	1.073859764	6.12E-06	1.97E-05
WASHC5-AS1	2.347916063	4.78E-14	1.36E-12
DCLRE1B	1.204454445	3.03E-17	5.45E-15
PRR22	1.194223183	7.62E-07	3.06E-06
HSPA8P15	1.182738771	1.07E-07	5.31E-07
AL117329.1	6.200438822	1.58E-09	1.25E-08
TFB2M	1.043801495	7.29E-14	1.95E-12
DDX21	1.455914433	9.35E-18	2.90E-15
HIP1	1.01148544	2.39E-09	1.80E-08
AC073320.1	1.25669774	5.40E-06	1.76E-05
CD300E	1.352597318	0.025946596	0.036430526
MCM7	1.609781769	6.57E-15	2.62E-13
COL11A1	6.29130539	3.38E-16	2.51E-14
PLXNA1	1.405623753	2.78E-15	1.31E-13
AC105036.2	1.171943359	0.000202007	0.000454397
AC092120.2	1.110004445	0.012930751	0.019433693
BCAT1	1.929937141	1.96E-07	9.08E-07
LINC00894	1.25733387	1.44E-08	8.91E-08
SNORD6	1.948636096	1.34E-10	1.43E-09
AC130651.1	1.265839416	2.21E-05	6.27E-05
AL161757.1	1.057092351	0.00058479	0.001184879
CACNA1D	2.040146119	7.42E-11	8.42E-10
ERBB2	2.112115022	0.003476586	0.005931681
AC084357.2	1.395008401	4.07E-07	1.75E-06
AC078909.2	1.299951722	0.000703145	0.001400821
KLHL7-DT	1.372803397	0.000551496	0.001125213
ADAMTSL2	1.447657973	2.15E-07	9.87E-07
CLMP	-1.161317211	0.000561183	0.001142259
AC106782.4	1.020444422	0.0004733	0.00098122
ELOVL7	1.191575092	1.31E-08	8.15E-08
RPL37P15	1.03343343	0.006326291	0.0101883
AC022973.5	2.007064838	1.37E-11	1.90E-10

AC091799.1	1.264787469	2.03E-07	9.36E-07
AC080129.2	1.954243291	2.74E-05	7.57E-05
MMP10	3.870840167	1.02E-10	1.12E-09
SNRPFP1	1.599220634	8.88E-10	7.55E-09
TDRD6	1.984185767	3.71E-11	4.56E-10
SEH1L	1.165006401	8.01E-17	9.44E-15
GAPDHP32	1.029339844	0.003271412	0.005620854
AC008619.1	2.17427306	0.000747874	0.001482062
AC083805.2	1.179332508	0.000600981	0.001215098
ARHGEF25	-1.7337417	1.91E-05	5.50E-05
UTS2	2.477140311	5.39E-06	1.76E-05
NARF-AS1	1.692965232	1.09E-09	8.98E-09
GOLGA8A	1.455051824	4.28E-07	1.84E-06
AL139184.1	2.291744791	8.99E-07	3.55E-06
RARRES1	2.041981778	1.78E-06	6.51E-06
KLF17P1	1.532563884	1.16E-05	3.51E-05
AC084757.2	1.299554479	6.14E-05	0.000155878
TATDN2P2	1.032105385	2.36E-08	1.38E-07
RNU6-1238P	4.094020488	1.96E-07	9.09E-07
AL138831.3	1.416684365	2.10E-07	9.68E-07
ADGRB3	-1.868002487	9.69E-07	3.79E-06
AC073107.2	1.604462329	2.09E-09	1.60E-08
TK1	1.185020708	1.93E-08	1.15E-07
PRR11	1.914539	1.67E-16	1.52E-14
MLEC	1.213075784	2.47E-12	4.20E-11
AL355864.2	1.144334384	9.14E-05	0.000222836
ZNF618	1.25434369	3.52E-11	4.34E-10
AC004471.1	1.106285733	2.80E-06	9.80E-06
TTI1	1.252737734	9.74E-18	2.95E-15
SEPTIN3	1.59169373	0.004078557	0.006857741
AL033504.1	1.374670119	0.000457141	0.000951347
KNOP1	1.32168864	9.88E-18	2.96E-15
LINP1	1.257055079	0.025599403	0.036001864
RPL7AP2	1.290534151	1.95E-06	7.07E-06
ACTN2	-2.687265561	0.006004067	0.009716945
RNU12-2P	1.331203706	0.002295836	0.004079039
WDR62	2.346803679	1.63E-16	1.50E-14
CPNE7	2.418769986	3.78E-09	2.69E-08
SPDYE2	1.234964578	9.92E-07	3.87E-06
PHF14	1.36284996	1.61E-17	3.68E-15
DDX55	1.172975138	1.13E-17	3.15E-15

LRP2	3.546984766	0.002132793	0.003814316
ALG1L3P	3.213436536	7.90E-08	4.05E-07
SNORD14E	2.420284478	1.45E-08	8.94E-08
DHRSX-IT1	1.119219685	2.09E-05	5.96E-05
RPL23AP89	1.552888075	1.93E-05	5.55E-05
RPL39P39	1.653323639	3.44E-06	1.18E-05
AL138760.1	1.59369743	5.62E-05	0.000143807
SPON2	1.332394048	5.16E-09	3.54E-08
AC008105.1	1.362634502	3.47E-09	2.50E-08
AP002762.1	2.011969302	0.001875208	0.003391209
LINC01909	1.431110127	3.89E-08	2.15E-07
MCOLN2	1.046991333	1.16E-05	3.51E-05
AC110801.1	1.386800266	0.005686715	0.009251676
AP002812.3	1.395357814	1.31E-06	4.96E-06
TRAJ23	1.751376857	0.001893911	0.003420388
LINC02582	7.391385554	3.55E-06	1.21E-05
CXorf65	1.751964803	2.51E-09	1.88E-08
AL645729.1	1.433649651	0.010766698	0.016483528
MYOM1	-1.785902558	9.29E-05	0.000226136
DLG2	-1.591885928	5.49E-05	0.000140903
SLC39A8	1.177422361	3.00E-06	1.04E-05
PITX1	-1.653837287	0.015808287	0.023299645
AL683807.2	1.606138398	0.014886802	0.022078277
AL136221.1	1.553288815	3.37E-08	1.90E-07
KRT18P26	1.576311589	3.71E-09	2.65E-08
ZBTB45P1	2.944122356	2.59E-12	4.38E-11
FDPSP1	1.005820561	1.66E-06	6.12E-06
RN7SKP74	1.083911264	0.000360908	0.000767637
AC124947.1	1.261422841	0.000464965	0.000965593
EIF4A1P12	1.516222945	0.028427724	0.03950816
PYCR1	1.014455476	4.06E-06	1.36E-05
MYZAP	-1.700184302	4.10E-14	1.20E-12
KANSL1L-AS1	1.244048755	2.63E-07	1.18E-06
AVIL	1.516354218	8.10E-05	0.000199843
AL008718.3	1.221874158	4.05E-05	0.0001075
RPS16P9	1.300098561	0.004204373	0.00704395
AL096701.2	1.822292228	1.11E-07	5.47E-07
NOP56P1	1.627080554	1.64E-05	4.79E-05
AC063952.1	3.367033452	1.36E-07	6.56E-07
ARL9	1.609220374	3.49E-07	1.53E-06
TUBB4BP3	1.574222004	9.05E-05	0.000221031

VIPR2	-1.531186555	1.68E-07	7.93E-07
CBY2	4.838571392	6.64E-08	3.47E-07
MESP2	2.603465037	1.92E-08	1.15E-07
GAPDHP2	1.11289994	3.34E-09	2.41E-08
AC007608.4	1.955484993	0.035756098	0.048543313
SNORA24B	2.032659989	2.04E-06	7.36E-06
RNU6-595P	1.437156908	4.22E-06	1.41E-05
RIMKLBP2	1.234853763	4.81E-08	2.60E-07
AC079684.1	2.113857511	2.96E-13	6.58E-12
AC097372.2	2.168293548	8.23E-10	7.08E-09
AC021739.3	1.741278275	3.31E-08	1.87E-07
GRIN3A	1.018862981	4.20E-05	0.000110863
GLIS3	1.521286799	8.32E-07	3.32E-06
NEU3	1.030782827	5.92E-13	1.20E-11
AC005840.2	1.631015668	3.21E-10	3.09E-09
APOA4	-5.923367969	0.011916284	0.018052649
C1orf131	1.096642583	1.09E-13	2.77E-12
LAMTOR5-AS1	1.194403546	5.49E-12	8.52E-11
FFAR4	1.228354035	0.024717022	0.034865829
BEX4	-1.349998166	4.60E-08	2.49E-07
AL138689.1	1.591459018	1.16E-05	3.50E-05
Z69720.2	1.8721553	3.60E-07	1.57E-06
EOMES	1.335445593	3.20E-05	8.70E-05
AC068790.4	1.118749456	4.93E-06	1.62E-05
RPL7P2	1.59822938	1.81E-07	8.48E-07
ALG1L13P	1.046082729	0.000171725	0.00039332
REEP2	-1.770751022	5.42E-05	0.000139291
MIR4768	1.798997781	0.000238827	0.000528913
AC073487.1	1.15335354	1.42E-06	5.34E-06
AL157938.3	1.20539649	3.42E-05	9.25E-05
RN7SL744P	1.729412797	0.000769296	0.001520245
AC087071.2	1.171141108	2.11E-06	7.59E-06
CCNYL7	1.263631105	1.58E-05	4.63E-05
RN7SL606P	1.505283831	5.04E-06	1.66E-05
IGKV1OR9-1	-3.434022791	4.22E-05	0.0001115
DUXAP10	4.005128094	2.30E-15	1.13E-13
AC104115.2	1.307708223	1.95E-07	9.05E-07
AC083902.1	-2.504916199	0.019064793	0.027583589
OSBPL3	1.695332999	3.56E-17	6.08E-15
ZBTB41	1.079200728	1.08E-12	2.04E-11
AC010609.1	1.783074471	0.003289875	0.00565039

KCNH2	-1.278840828	0.001070773	0.002053375
MMP7	3.717400618	8.87E-09	5.78E-08
FSCN1	1.506000546	2.62E-08	1.52E-07
AP003559.1	2.547392754	0.004806214	0.00794485
HCP5	1.002319159	9.03E-06	2.80E-05
HNRNPA1P42	1.327221142	7.84E-08	4.02E-07
NKAIN4	1.908173827	0.000178827	0.00040811
CEP170	1.122557811	6.34E-10	5.65E-09
AL033527.2	2.325891319	3.49E-11	4.32E-10
AL451062.1	1.111738136	7.82E-05	0.000193563
RNA5SP159	2.214085382	9.80E-05	0.000237157
PABPC1L	2.893210067	2.32E-16	1.88E-14
LINC02690	4.455208424	0.002245642	0.003997353
PRKCA-AS1	1.620935532	5.00E-07	2.11E-06
RNU6-322P	1.234112749	8.66E-05	0.000212227
PON1	3.130985577	0.000602978	0.001218396
CTPS1	1.293358108	2.71E-16	2.09E-14
MIR4420	1.057753751	0.019143982	0.027689141
OR7E38P	1.070587572	3.03E-07	1.34E-06
ATP6V1C1	1.118690011	5.34E-18	2.12E-15
AL133215.1	1.920488542	6.38E-09	4.29E-08
H3P38	1.303475378	6.78E-05	0.00017046
ECRG4	-3.449044651	1.69E-12	2.99E-11
CASP2	1.2980647	4.07E-17	6.49E-15
AC105384.2	1.231448681	7.44E-05	0.000185401
AL512770.1	1.166906014	4.44E-09	3.09E-08
AL138963.2	1.634926657	1.32E-05	3.94E-05
CKAP2L	2.090399327	9.58E-16	5.69E-14
CRYGS	1.051210596	8.32E-07	3.32E-06
AL355802.3	1.970824905	3.28E-13	7.18E-12
NUDT10	-1.515574361	9.23E-07	3.63E-06
AP000487.2	1.454156767	1.56E-05	4.57E-05
MAPK15	3.226014566	2.61E-15	1.25E-13
CYMP	2.36681733	0.010315053	0.015865007
AC021744.1	1.627777981	4.24E-05	0.00011175
HAUS7	1.317259199	1.82E-14	6.11E-13
PSAPL1	-3.163451504	9.71E-05	0.000235283
H3P42	1.769799277	2.65E-05	7.36E-05
GEN1	1.568732862	2.01E-14	6.64E-13
PAIP2B	-1.28774215	6.28E-11	7.28E-10
NLN	1.100162204	7.97E-13	1.55E-11

AC008443.2	3.932472315	1.94E-05	5.57E-05
SPDYC	4.783752663	1.53E-12	2.74E-11
AC008895.1	1.186477888	3.52E-10	3.34E-09
LDHD	-1.452365141	2.98E-07	1.32E-06
FERMT1	1.816422195	9.38E-12	1.36E-10
LINC01004	1.422577222	9.33E-11	1.03E-09
LINC01170	1.714270109	0.027421665	0.038275685
LINC02473	2.502212106	0.000720502	0.001432199
MTUS2	-2.815423682	1.67E-05	4.88E-05
RNU6-665P	2.234293567	6.47E-05	0.000163349
MAK	1.293864605	1.16E-07	5.68E-07
AL355490.2	1.405446668	6.18E-05	0.000156811
CEP83	1.204016381	3.57E-15	1.60E-13
MTHFD1	1.003633818	1.59E-13	3.83E-12
DAB1	-2.691284859	4.36E-09	3.05E-08
HNRNPA1P34	1.163842795	0.00010893	0.000260748
NUP85	1.029326415	1.68E-17	3.72E-15
DNAH17	1.435090973	6.47E-12	9.84E-11
AC078860.2	3.40774388	8.95E-07	3.54E-06
RN7SL30P	1.723447014	2.38E-05	6.69E-05
[illegible]CL2L2-PABPN	1.304639233	1.72E-08	1.04E-07
MIR3189	4.164838742	4.43E-14	1.29E-12
WDR38	1.307966239	0.000264735	0.000580357
AC023825.1	2.623867438	8.03E-13	1.56E-11
SNAI1	1.039587924	5.47E-07	2.28E-06
AC026410.2	1.266223202	3.35E-05	9.08E-05
AC016735.1	1.728379964	2.44E-08	1.42E-07
S100A12	-1.392638681	3.16E-07	1.39E-06
ATP4B	-4.951253252	1.39E-05	4.13E-05
GFRA1	-1.959605572	8.41E-11	9.41E-10
SYNPO2	-1.580932775	6.83E-08	3.55E-07
H3P1	1.289842594	8.39E-09	5.50E-08
TSPEAR	2.483590727	9.09E-07	3.58E-06
RBM12B	1.07690258	4.64E-15	1.97E-13
AC024884.1	1.966303553	5.96E-05	0.000151686
DDX27	1.191827986	6.02E-15	2.42E-13
MYADML	1.519831823	0.00296348	0.005136492
LINC02884	-1.120103834	2.72E-06	9.54E-06
KIAA1841	1.091172751	4.54E-13	9.48E-12
AP001001.1	1.582296671	3.81E-10	3.59E-09
AC009237.14	1.107706226	1.41E-05	4.19E-05

AL589765.6	2.581058645	8.98E-10	7.62E-09
AC012254.4	1.385051065	9.49E-06	2.92E-05
AC080037.1	1.196269337	2.48E-05	6.94E-05
AC027449.1	1.947441391	1.94E-05	5.57E-05
RNVU1-31	1.142602548	0.004247265	0.007109998
KMT5AP3	2.311734573	3.67E-08	2.05E-07
DLL4	1.260829435	2.45E-11	3.17E-10
RN7SL535P	1.691403614	1.40E-08	8.69E-08
MRPS18AP1	1.294572733	6.53E-07	2.67E-06
HSPD1P12	1.412721999	1.11E-07	5.47E-07
RPS26P45	2.272396281	2.14E-07	9.84E-07
BBOX1-AS1	1.5603501	7.58E-08	3.90E-07
RNU7-48P	1.189412407	0.021364927	0.030607323
AC006435.5	1.242307824	0.010382571	0.015956876
RNU4-30P	1.256765451	0.001646491	0.003018152
RPGRIP1	1.548015378	7.41E-08	3.82E-07
IFITM1	1.468197784	3.29E-07	1.45E-06
HNRNPA1P5	1.34548036	1.09E-08	6.94E-08
TAF1A	1.3012409	1.70E-15	8.82E-14
AC020611.1	1.386227366	1.59E-05	4.66E-05
AC011411.1	1.124379531	0.000205131	0.000460761
ADAMTS2	3.029619788	2.29E-16	1.87E-14
COL22A1	3.145108038	3.97E-10	3.72E-09
MSLNL	4.327251669	3.61E-10	3.42E-09
RN7SL3	1.156034649	0.030796597	0.042413178
ZNF121	1.269878807	8.83E-15	3.31E-13
C9orf116	1.119755594	3.24E-06	1.12E-05
STMN1P1	1.656769736	1.88E-07	8.79E-07
AC026956.1	1.015892105	0.015589499	0.023030689
HTR7	-1.512874432	3.07E-09	2.25E-08
RNU6-652P	1.024316136	2.76E-05	7.65E-05
AL117379.1	1.589174745	2.96E-10	2.89E-09
SMIM10L2A	-1.196307235	2.41E-09	1.82E-08
AC008507.1	1.069154176	4.83E-06	1.59E-05
AC233280.2	2.353972805	5.03E-11	5.96E-10
RAP2CP1	1.877684354	0.001871698	0.003385322
SIX4	1.853852854	4.36E-09	3.05E-08
AC016737.1	2.488230318	9.08E-12	1.33E-10
DAGLA	1.218073974	8.70E-09	5.69E-08
AC005165.1	-1.48530001	3.81E-07	1.65E-06
UTP20	1.279961889	2.14E-16	1.81E-14

AP000695.2	1.882233247	1.34E-09	1.08E-08
OR13Z2P	6.217797557	2.16E-09	1.65E-08
TRPM2	1.888795043	1.32E-13	3.24E-12
ENPP6	-1.628573747	3.13E-11	3.92E-10
AC006480.1	1.681642924	8.60E-09	5.63E-08
AC104785.1	1.176910981	6.99E-06	2.22E-05
AC004069.1	1.176649144	2.11E-06	7.61E-06
BANF2	2.491877905	0.005416809	0.008853658
AL161891.1	2.090336907	2.38E-16	1.92E-14
NEURL3	2.270087093	8.23E-07	3.28E-06
AC018714.1	2.041772563	0.000426518	0.000892148
SALL4	5.400403376	1.39E-17	3.39E-15
LINC01537	-1.493699127	1.66E-06	6.12E-06
IVNS1ABP	1.223508735	3.18E-14	9.66E-13
SPRY4-AS1	1.050118193	4.67E-06	1.54E-05
FERMT2	-1.374287078	0.024616874	0.034741115
RN7SL806P	1.405684051	0.000143107	0.000333362
AL162615.1	1.067773404	3.71E-06	1.26E-05
IMPG1	1.611530122	1.25E-09	1.02E-08
AC010615.1	1.186886965	2.44E-05	6.85E-05
PCAT18	-2.738543629	3.23E-08	1.83E-07
AC011483.1	1.722251977	0.00127725	0.002400768
RNU7-18P	2.198332452	1.06E-05	3.22E-05
AC020656.2	3.450289514	6.15E-11	7.15E-10
AC106028.4	1.515350875	1.15E-08	7.26E-08
HECW1	1.205885822	0.000162821	0.000374794
TCEAL6	-3.139299558	3.52E-05	9.48E-05
FOXP1-AS1	1.067403624	0.000258056	0.000567068
TMCO1-AS1	1.100218019	0.000103671	0.000249352
NRG2	-1.737949346	4.16E-09	2.93E-08
ASS1P11	1.354213763	8.24E-05	0.000202899
LINC00244	1.879181824	0.000898686	0.001752604
PRKCB	-1.045428683	0.000406513	0.000854666
AC091588.1	1.852987839	5.50E-08	2.92E-07
HASPIN	1.959304545	2.21E-14	7.19E-13
MATN3	3.04953361	1.07E-11	1.53E-10
STX16-NPEPL1	1.147993901	8.43E-06	2.63E-05
NDUFS5P1	1.35309643	0.002046407	0.00367266
RPL7P8	1.168750443	5.74E-07	2.39E-06
AC004009.2	2.047638437	0.001000795	0.001933797
AC091812.3	1.522623918	0.000503664	0.001038111

AC017083.1	1.46928349	2.41E-09	1.82E-08
AL731566.2	1.456239946	1.40E-11	1.93E-10
AC023043.2	3.030704679	1.71E-07	8.04E-07
ZFP69B	1.38899283	1.94E-13	4.55E-12
Z97200.1	1.507376507	4.34E-05	0.000114127
TUBB8P2	1.516041339	7.18E-05	0.00017943
AC010491.1	1.07008485	4.31E-05	0.000113461
ACTBL2	4.523331045	1.98E-11	2.64E-10
ECT2	2.766459953	1.12E-18	1.29E-15
AC004678.2	1.469856853	5.99E-08	3.15E-07
ESF1	1.357908146	4.46E-14	1.29E-12
AC092451.1	1.734513465	4.43E-05	0.000116198
AL158166.2	1.546771605	1.77E-09	1.38E-08
AC009159.3	-1.066736211	9.76E-07	3.82E-06
HIGD1AP17	1.152311646	0.001182914	0.002241639
TG	1.120749887	0.008398336	0.013169421
DLEU2L	1.086429434	2.19E-06	7.85E-06
RPS24P6	1.068711796	0.000179893	0.000410312
MRGPRF	-1.729095858	9.23E-05	0.00022482
RNA5SP37	1.474471332	3.34E-06	1.15E-05
ZNF281	1.113369019	3.54E-18	1.72E-15
AL024498.1	1.05059275	1.25E-08	7.86E-08
GRIN2D	2.964699339	1.01E-14	3.73E-13
RPL35AP5	1.095250711	2.11E-07	9.72E-07
DUSP26	-1.678088587	1.15E-08	7.26E-08
ATR	1.434444855	2.04E-16	1.74E-14
CARMN	-1.677104104	0.002738153	0.004776081
FADS6	-2.215466053	0.000519802	0.001067986
KRT18P23	1.871409034	3.48E-11	4.31E-10
DIRAS1	-1.234990664	4.98E-11	5.91E-10
IGHV1OR15-1	-2.717711273	0.007117049	0.011338421
AC008267.2	1.169790862	2.42E-08	1.41E-07
AC005840.3	1.366136292	9.35E-05	0.000227377
MAD2L1P1	1.136706802	2.24E-06	8.01E-06
SNRPB	1.052942365	1.50E-11	2.05E-10
AC130456.4	2.341249779	8.33E-11	9.35E-10
H3P4	-1.119745897	7.91E-06	2.48E-05
Z93403.1	2.648537356	1.65E-05	4.83E-05
IGLV2-28	-3.8498552	0.005572109	0.009082647
FAM86LP	1.510155333	8.20E-06	2.56E-05
CAMTA1-IT1	2.299248263	0.003482097	0.005940324

AC243571.2	1.723165963	0.000176193	0.000402585
HBA2	-1.449914649	2.21E-10	2.22E-09
NDUFA3P2	1.575154186	1.05E-05	3.19E-05
SNORA22B	1.684393868	4.44E-08	2.42E-07
PGA5	-3.545703762	6.10E-07	2.51E-06
AL139384.2	1.582310704	0.007136272	0.011365684
STK16P1	2.321564997	9.59E-12	1.39E-10
CDT1	1.773841292	3.64E-12	5.92E-11
SEM1	1.016807816	7.15E-10	6.27E-09
CXCL16	1.335591063	3.20E-11	3.99E-10
CSRP1	-1.694130336	8.00E-07	3.20E-06
TBRG4	1.016172621	4.57E-14	1.32E-12
MIR3657	1.351184427	0.000191133	0.000432822
ASAH2	-3.972234982	0.001568781	0.002888221
AL133373.2	2.237222297	2.42E-08	1.41E-07
AC114956.1	1.477544798	0.000140855	0.000328692
RNU6-26P	1.283737138	0.000944768	0.001835655
AC040173.1	1.654227184	3.93E-06	1.32E-05
AL691449.1	1.402604852	0.003863113	0.006532182
MST1P2	1.267122287	0.001774174	0.003228216
FAM111B	1.768298105	9.24E-13	1.77E-11
BICRA-AS1	1.03101344	0.000204767	0.000460166
ALG1L	1.577815512	1.53E-05	4.50E-05
SUMO1P1	4.264530054	8.80E-06	2.73E-05
AL645940.1	1.080120614	0.000107956	0.000258555
TAF7L	2.375184105	3.30E-05	8.96E-05
RNU6-1223P	1.23190445	7.25E-06	2.29E-05
MADCAM1	-1.044713988	0.002852739	0.004959392
ASS1P3	1.020349143	0.004784473	0.007913331
CNTN2	-1.617270765	6.40E-05	0.000161579
LINC01979	2.488086056	2.50E-12	4.24E-11
RPS29P10	1.266377343	0.004459909	0.007423986
ZNF107	1.258082465	3.40E-13	7.40E-12
HIGD1AP18	1.708065579	0.000147286	0.000342408
AC073534.1	1.378395443	4.42E-06	1.47E-05
MZT1P1	1.189772547	0.008000946	0.012607121
ASPHD1	1.824319291	8.46E-07	3.36E-06
RPS3AP27	1.171647321	3.97E-05	0.000105717
AL358178.1	1.100829794	1.26E-05	3.76E-05
AC004223.2	1.24647423	4.51E-05	0.000118176
CHCHD2P8	1.038175035	2.37E-05	6.67E-05

PKP4	1.067668301	1.32E-13	3.24E-12
HOXB9	3.094624827	1.25E-12	2.31E-11
MNX1	1.684337786	6.28E-11	7.28E-10
PM20D1	-1.552223237	0.001655232	0.003033132
AQP4	-4.478326476	8.56E-14	2.23E-12
AC012313.3	1.159182371	0.000574341	0.001166458
FAM81A	2.086150119	4.65E-13	9.66E-12
TIMM8AP1	1.147210021	0.004201347	0.007039476
E2F3-IT1	2.026072751	1.23E-07	6.01E-07
AC015813.1	1.148509089	3.02E-06	1.05E-05
TRGV6	1.680077138	9.13E-05	0.000222784
AL137060.3	1.720850956	7.42E-12	1.11E-10
AC139495.2	1.024473046	0.000156799	0.000362279
RPSAP16	1.454933648	8.77E-08	4.44E-07
OLFML2B	2.208513527	1.42E-11	1.95E-10
AC004951.1	1.258270413	2.53E-08	1.47E-07
AP001625.2	1.140926348	4.20E-05	0.000110863
AC120036.1	4.15845981	1.14E-09	9.38E-09
CCT6P3	1.055287438	8.62E-10	7.36E-09
AC007842.1	1.401230923	8.37E-10	7.17E-09
XPOT	1.268148692	1.45E-16	1.40E-14
LINC01748	2.854843162	1.49E-06	5.56E-06
AHR	1.027721779	6.97E-11	7.97E-10
RN7SL429P	1.123606848	0.012252057	0.018518143
COL5A2	2.246363551	3.31E-15	1.50E-13
BTG2	-2.145522582	4.31E-06	1.44E-05
PKNOX2	-1.564704895	8.76E-11	9.76E-10
RNU7-77P	1.108498606	0.012379131	0.018689566
RNU6-937P	2.173862829	2.65E-05	7.38E-05
KLHDC7B	1.620604859	0.000107763	0.000258138
SNORD70	1.558608982	1.54E-06	5.72E-06
RNU6-610P	2.875237713	1.02E-08	6.56E-08
COL8A1	1.981949109	1.22E-08	7.70E-08
PDZRN4	-3.098334844	3.06E-08	1.74E-07
UNC13A	2.051259554	0.005415523	0.008852103
MSLN	3.470194478	1.78E-08	1.07E-07
AL591895.1	1.567106272	9.91E-12	1.43E-10
JADE3	1.174389659	1.57E-14	5.40E-13
FOXP1-IT1	1.303261953	1.67E-07	7.87E-07
MMP9	1.712969523	7.58E-08	3.90E-07
AC004895.1	1.127710936	0.000797347	0.001570642

PPIAP66	1.288611701	2.15E-08	1.27E-07
AC099811.5	1.143347616	2.82E-05	7.78E-05
LRAT	-2.208798204	6.73E-06	2.14E-05
SLC12A8	1.485800269	2.47E-14	7.85E-13
HSPE1P2	1.318695338	3.79E-11	4.65E-10
TAS2R19	1.333822375	9.56E-07	3.75E-06
NALCN	1.652222302	0.000637186	0.001280718
FARSA-AS1	1.299853011	1.92E-06	6.97E-06
KRT8P12	1.2965847	1.01E-15	5.90E-14
MIR4482	1.060818077	0.000539762	0.001103803
AKR1B10P1	-2.69460965	6.99E-07	2.83E-06
U62317.1	1.202307593	8.79E-12	1.29E-10
LINC00870	2.059774175	1.48E-07	7.06E-07
AC069234.4	1.21556348	0.00060397	0.001220306
SLC9A9	-1.063532611	2.71E-09	2.01E-08
AC093157.2	1.103277205	6.20E-08	3.26E-07
KIF18B	2.627840541	4.58E-17	6.90E-15
AC010631.1	1.282572761	0.003235797	0.005564326
LIF	2.635640149	2.23E-16	1.86E-14
AL137244.1	1.546852855	2.03E-07	9.36E-07
PDCL3P3	2.663319468	0.000337925	0.000722901
AP001885.1	1.224098832	9.64E-06	2.96E-05
LINC01123	1.521319072	5.04E-11	5.96E-10
H2AC19	1.142277934	0.003196476	0.005503814
NOS1	-2.566159471	4.35E-07	1.86E-06
AC145343.1	1.332746323	3.73E-05	9.98E-05
FLG	-5.590766506	0.003189868	0.005494566
DBH-AS1	1.435185937	0.000236253	0.000523735
SEPTIN14	3.897303875	0.000459665	0.000955853
AC211433.2	1.264750046	8.58E-05	0.000210396
ATP2A1	1.912749244	1.51E-12	2.71E-11
AC136475.3	2.477767148	6.40E-09	4.30E-08
ATP6V1B1	1.669615186	1.37E-06	5.18E-06
AL591135.1	1.029186653	9.99E-08	4.99E-07
LINC01869	1.160987958	0.000276825	0.000604017
UBE2CP3	1.029629786	0.000249596	0.000549992
AL133153.1	3.201203954	1.32E-09	1.06E-08
SRSF10	1.023126782	1.26E-17	3.29E-15
EDN2	-1.919148651	6.07E-05	0.000154076
PHC2-AS1	1.127524831	0.000364075	0.000773932
UCK2	1.125208633	5.00E-15	2.10E-13

AL356124.1	1.945365413	1.51E-09	1.20E-08
LCLAT1	1.016947498	1.93E-12	3.37E-11
ZBTB33	1.066509621	3.86E-14	1.14E-12
AL109810.1	1.346433855	1.18E-06	4.51E-06
C5orf66-AS1	-2.961571825	4.86E-13	1.01E-11
TNIP3	1.349818969	1.91E-05	5.50E-05
KLK4	4.034541692	0.001073472	0.002058255
LINC02520	1.20736026	0.020375552	0.029297072
AC243964.2	1.35498727	0.001872526	0.003386588
AC009228.2	1.346934299	0.002314022	0.004106726
AL138847.1	1.189676726	3.71E-05	9.93E-05
MAL	-4.582220321	2.44E-17	4.97E-15
IGKV2-40	-3.374628045	0.000327301	0.000702882
AC005740.1	1.666644524	6.57E-08	3.43E-07
CDC42EP3P1	1.582912854	9.28E-06	2.87E-05
AC012618.2	1.055718297	1.35E-10	1.45E-09
AC048341.2	1.523388753	6.90E-11	7.90E-10
ACBD7	2.722002862	1.46E-12	2.63E-11
AC233976.1	-1.204590693	2.70E-05	7.48E-05
TAF1D	1.283353044	8.10E-15	3.09E-13
KRT18P3	1.946842837	2.80E-11	3.58E-10
MIR302D	1.958290509	8.59E-05	0.000210746
CHST4	2.704633306	2.65E-06	9.33E-06
AC022336.1	1.196321799	1.46E-07	6.98E-07
TNFRSF25	1.616570199	2.14E-10	2.16E-09
SRPX	-1.288714735	4.12E-11	5.01E-10
NAA25	1.193189563	1.28E-15	7.08E-14
PKD1L3	1.502233997	0.002406547	0.004252397
ARMC2-AS1	1.775663524	0.003484766	0.005944114
FOXL1	1.446000327	1.63E-07	7.70E-07
AL356740.2	2.457341299	7.63E-06	2.40E-05
C1QTNF7	-1.872273538	6.03E-07	2.49E-06
AC103702.1	1.176065713	0.001664106	0.003047521
STX16	1.012229164	3.85E-12	6.23E-11
RPL35P3	1.180560245	7.43E-07	2.99E-06
AC080013.2	1.40839189	3.75E-06	1.27E-05
AC011603.2	1.46494519	3.33E-08	1.87E-07
RN7SL566P	1.236484487	8.49E-07	3.37E-06
ZFPM2	-1.193924647	0.009418371	0.014603902
YEATS4	1.133721406	3.05E-12	5.03E-11
SNRPA1P1	1.400962065	8.89E-12	1.30E-10

AC027702.1	1.354787284	1.72E-08	1.04E-07
NIBAN1	-1.818095154	4.75E-06	1.57E-05
ELFN2	3.385238242	1.11E-06	4.29E-06
AC007342.5	1.572429213	5.81E-07	2.41E-06
CLCA1	-2.987610941	0.002699411	0.004714679
CLEC7A	1.041595508	1.54E-05	4.52E-05
AL590483.1	2.889758576	0.000108696	0.000260256
FADS2	1.126576765	0.001355701	0.002532861
LINC01778	-1.553724705	0.00066893	0.001338865
IGHJ1	-1.84373826	0.028978747	0.040206842
ASS1P9	1.880301626	6.88E-08	3.57E-07
LINC00884	1.589023993	8.47E-09	5.55E-08
BIRC6-AS2	1.226882383	1.71E-05	4.97E-05
DDX31	1.240914317	3.75E-17	6.26E-15
CABLES2	1.069196687	2.13E-08	1.26E-07
EEF1DP5	1.196807827	7.11E-05	0.000177747
TXLNB	-1.166925714	9.38E-07	3.69E-06
MASP1	-1.427424334	6.48E-08	3.39E-07
HAGHL	1.992651768	2.95E-12	4.90E-11
IGFL4	3.64861975	4.54E-08	2.46E-07
CXCR2P1	2.156939176	4.95E-06	1.63E-05
AL365181.2	2.320311683	5.20E-08	2.79E-07
AC068025.1	2.107851537	5.89E-09	3.99E-08
MYB	2.160939604	1.71E-10	1.78E-09
CXCR2	-1.763055352	0.000365247	0.0007763
FAM3D	-1.246297902	0.000303222	0.000655187
FO393415.1	1.192069756	2.69E-05	7.46E-05
NANOGP7	2.361472661	5.64E-08	2.99E-07
AC022559.1	3.568872672	0.030022258	0.041503373
SUB1P2	2.319912305	6.49E-05	0.000163884
CCNB2	1.796534441	1.10E-14	3.97E-13
PDP1	1.20984317	7.61E-13	1.49E-11
SNORD37	1.53378677	0.001000609	0.001933577
RNU6-562P	2.335513358	0.000359745	0.000765337
TMEM147-AS1	1.104304391	2.21E-09	1.68E-08
RNA5SP122	1.695702507	4.41E-06	1.47E-05
SLC20A1	1.176217218	3.00E-14	9.21E-13
PHYHD1	-1.249147413	5.14E-12	8.03E-11
AP000346.2	1.637761223	6.10E-10	5.46E-09
RPL7P7	1.084996904	3.76E-06	1.27E-05
PPP4R1L	1.060717334	2.76E-09	2.04E-08

BID	1.265331218	2.29E-13	5.25E-12
MYO7A	1.703818457	1.19E-11	1.68E-10
EIF4A2P2	1.046634629	6.87E-05	0.000172469
NHLRC3	1.025326522	1.59E-15	8.34E-14
NOVA1	-1.405624309	1.09E-07	5.39E-07
RNY4P19	1.873447837	0.00056137	0.001142552
CHAF1B	1.56652152	2.36E-15	1.15E-13
AL356488.2	1.527208948	3.52E-05	9.48E-05
CALY	-2.138136951	9.32E-08	4.68E-07
POM121B	1.005969379	1.10E-05	3.33E-05
AC131235.2	1.195961994	3.92E-05	0.000104354
AC020558.2	1.177740911	5.79E-07	2.40E-06
AC092422.1	-2.280607517	4.34E-08	2.37E-07
SLC25A4	-1.649493226	1.85E-11	2.48E-10
AC078778.1	1.523573815	5.36E-08	2.86E-07
AL031709.1	1.566714876	1.24E-07	6.05E-07
HOTTIP	3.401909028	8.35E-12	1.23E-10
MTCO1P45	1.158728948	0.006723938	0.010771401
EIF3MP1	1.615614554	0.000934525	0.001816946
AC073957.3	1.990191589	3.60E-13	7.77E-12
AC108114.1	1.51790984	7.93E-08	4.06E-07
MYH7B	1.778957338	6.83E-05	0.000171547
IGKV1D-27	-2.512749194	0.020386441	0.029309565
SVIL2P	-1.243365785	0.010982555	0.016778326
AC010530.1	1.221056569	3.39E-06	1.16E-05
CCN6	2.104289191	0.000974269	0.001887469
AC007099.1	6.185876655	9.29E-09	6.04E-08
AC004241.3	1.534651015	2.67E-11	3.42E-10
EEF1E1	1.136546433	2.91E-11	3.69E-10
BMP8A	2.273115076	7.90E-15	3.03E-13
TUBB1	1.14561832	1.39E-05	4.13E-05
LINC02753	2.085087905	2.11E-05	6.02E-05
PIGW	1.572951488	1.09E-17	3.15E-15
PPIAP9	1.103942788	8.55E-06	2.66E-05
AL356488.1	1.07895968	8.77E-06	2.72E-05
FCGR1B	1.874190954	7.01E-10	6.17E-09
NPIPB4	1.207034679	2.04E-08	1.21E-07
NT5C3A	1.006394496	5.92E-10	5.32E-09
AC010173.1	1.216022798	1.08E-07	5.33E-07
AL360219.1	1.327441765	3.66E-06	1.24E-05
RN7SL487P	1.391356765	3.23E-06	1.12E-05

IGKV2OR22-3	-4.170529909	0.00438042	0.007311312
LRRC3-DT	-2.601188421	0.003201199	0.005511233
TMEM211	-1.678101788	0.027707615	0.038604031
RPL12P25	1.862293664	3.19E-09	2.33E-08
THSD4	-1.426097279	1.78E-06	6.51E-06
AC022001.1	1.188223277	0.000187481	0.000425348
LINC01291	1.363878975	0.004448552	0.007408785
TPT1P10	1.000636105	0.000273515	0.000597504
CYP27B1	2.428688266	5.80E-15	2.35E-13
RPL32P22	1.200551078	0.000881314	0.001721405
AL449212.1	1.228358108	8.88E-10	7.55E-09
CLEC6A	1.442357141	0.000407471	0.000856613
CDC42P2	1.062006722	0.001255139	0.002365048
AC005393.1	1.361709268	9.50E-05	0.00023078
GTF2IP20	1.329665411	3.50E-09	2.52E-08
MMP25-AS1	1.603812238	1.97E-12	3.43E-11
TFAP2E	1.410929427	6.88E-05	0.000172502
PNO1	1.060642346	5.72E-18	2.15E-15
RN7SKP38	1.250511037	0.000927036	0.001802913
CBX8	1.226022664	3.63E-10	3.43E-09
CTLA4	1.294041489	5.88E-08	3.10E-07
AF131216.1	1.190304437	9.89E-06	3.03E-05
NFKBIE	1.036069911	2.70E-08	1.55E-07
AP003733.1	1.89241343	1.31E-07	6.32E-07
AC006252.1	1.442035272	3.13E-09	2.28E-08
QPCT	1.384119455	0.005848265	0.009490162
C8orf88	-2.018410501	7.50E-07	3.02E-06
HNRNPA1P4	1.167328267	1.92E-10	1.95E-09
AL121829.2	1.892992677	1.12E-05	3.40E-05
AP000851.1	1.895439443	3.67E-05	9.84E-05
GTF2IP9	1.481934973	4.76E-08	2.58E-07
CFTR	1.328608391	0.000567726	0.001154168
ELFN1-AS1	3.07845338	2.01E-09	1.55E-08
UBE2D3P3	1.657037247	0.000118406	0.000281055
BNIP3P39	2.38205123	0.000245855	0.000542583
AC005086.3	1.290014118	0.0014201	0.002641692
EDN1	1.170545427	2.53E-05	7.06E-05
ATP1A2	-2.790752551	8.88E-12	1.30E-10
EFNA4	1.151701666	2.82E-10	2.77E-09
AC011466.1	1.308807263	2.31E-06	8.24E-06
LINC01767	1.252279187	0.034732238	0.047295491

RNU6-519P	2.004310519	0.005021251	0.008269529
DNASE2B	2.061672956	3.56E-05	9.56E-05
AL365205.2	1.278500674	0.00414277	0.006953861
AC008750.6	2.287711802	5.21E-09	3.57E-08
AC117498.2	1.226692061	2.50E-05	6.99E-05
AC106772.1	2.70271123	5.92E-08	3.12E-07
RNU6-856P	1.393071241	1.45E-06	5.42E-06
LSG1	1.206026386	1.23E-18	1.31E-15
RPL39P36	1.309356715	2.02E-10	2.04E-09
MYOZ1	-1.246712819	0.024918371	0.035121966
TUBAP14	1.019757732	9.84E-07	3.85E-06
AL683807.1	1.2033268	0.001035382	0.001992515
AP003696.1	1.672537581	5.51E-06	1.79E-05
TMEM145	2.030237454	0.000112353	0.000267809
UFL1-AS1	1.740689734	0.000372028	0.000789075
S100A5	1.555066087	9.91E-08	4.95E-07
AC022493.1	1.120755115	0.019191304	0.027753066
BRIX1	1.452425595	1.41E-17	3.40E-15
GNL3LP1	1.596471439	1.28E-10	1.38E-09
AC005519.1	1.010704051	1.11E-06	4.28E-06
RNF113B	1.916569502	2.61E-07	1.18E-06
FXYD5	1.101995044	1.06E-07	5.27E-07
FRMD5	2.343750326	6.09E-14	1.67E-12
IGKV1OR-3	-3.182382748	0.001497704	0.00276827
SRSF6P2	1.025779312	2.13E-09	1.63E-08
KRT39	3.180655845	0.000110354	0.000263777
LINC02882	4.063035044	5.22E-10	4.76E-09
POLR1G	1.591867166	1.37E-17	3.39E-15
AC010883.1	1.118777158	4.61E-06	1.53E-05
MAL2-AS1	1.942785961	5.59E-11	6.56E-10
TXLNG	1.00459668	4.70E-13	9.77E-12
AL450344.2	1.233234135	3.64E-05	9.75E-05
AC007314.1	1.385102183	3.82E-06	1.29E-05
LINC02716	-1.460316714	6.11E-12	9.38E-11
AL731567.1	1.290023353	2.24E-05	6.34E-05
THUMPD3-AS1	1.017351889	8.97E-10	7.61E-09
GRB7	2.706328884	2.96E-10	2.89E-09
MIR5194	1.415292509	1.88E-05	5.41E-05
HIF1AP1	1.556091487	1.68E-06	6.21E-06
TPM3P6	1.65722342	8.39E-09	5.50E-08
RCC1	1.492976658	2.86E-14	8.87E-13

DNMT1	1.466268209	1.12E-17	3.15E-15
SNORD88A	2.061999708	4.00E-08	2.21E-07
FANCB	2.175120024	5.98E-17	7.85E-15
RNU6-658P	1.539594689	1.65E-06	6.08E-06
HNRNPA1P44	1.304586978	8.12E-07	3.25E-06
AL034405.1	1.403752003	0.000959626	0.001861538
RN7SL220P	1.013931335	0.000234739	0.000520813
ATP6V1FNB	1.004073645	4.46E-07	1.90E-06
AC110769.2	1.143987122	3.68E-06	1.25E-05
LRRTM1	-2.092834351	9.84E-10	8.24E-09
RNF165	-1.473771828	0.000181651	0.000413955
RNA5SP527	1.117456473	0.016175122	0.02378374
AL606534.1	1.573564858	5.53E-07	2.30E-06
XPO1	1.157648454	4.35E-17	6.81E-15
LINC02586	1.22267948	0.001490822	0.002757461
OTOAP1	2.138964694	1.51E-11	2.07E-10
SNORD1B	1.687763912	9.14E-07	3.60E-06
IGLV1-36	-1.792206055	0.035570113	0.04833022
SSX1	6.36469136	0.000373012	0.000791037
KCNK9	1.791800629	0.000730921	0.001451125
SHISA2	1.547054075	0.000640859	0.001287517
TMEM240	-1.211719577	0.003880985	0.006560319
CAMKV	2.942874156	0.02979966	0.04123202
STRIT1	1.210315654	0.02675131	0.037442724
AL928654.2	1.635956305	5.93E-12	9.13E-11
DTNA	-1.010848691	1.07E-05	3.24E-05
ZNF710-AS1	-1.258880728	1.06E-06	4.10E-06
AL353693.1	3.485091054	2.47E-07	1.12E-06
TAL2	1.745960943	6.31E-06	2.02E-05
EPB41L3	-1.057259855	1.66E-05	4.85E-05
AL359878.1	1.58924575	3.79E-10	3.58E-09
RPS24P12	1.670020134	1.85E-06	6.74E-06
AC091868.1	1.070850473	0.000703112	0.001400821
AC108059.1	1.162639176	0.025324716	0.035635261
AP002992.1	2.762185455	0.004358798	0.007279783
AC012186.3	1.984943672	5.32E-09	3.63E-08
RPL35P9	1.247191152	0.00010604	0.000254606
AC087741.2	1.032460997	0.00132799	0.002485271
PSTPIP2	1.081365609	0.000380806	0.000805514
ELOCP21	1.72032575	0.000811369	0.001595667
TRARG1	-1.742794441	8.25E-09	5.42E-08

CCDC58P3	1.159974614	4.20E-05	0.000110908
RPL7P28	2.193022343	3.86E-07	1.67E-06
RNF14P3	1.423888027	2.65E-07	1.19E-06
ATP6V0CP1	1.322197312	6.91E-07	2.81E-06
PPIAP49	1.403591679	1.25E-06	4.78E-06
ICAM5	1.490156655	0.000136465	0.000319314
H3P13	1.381988066	6.69E-07	2.73E-06
AC005787.1	2.436924957	0.000115858	0.000275399
AC090699.1	1.821165982	9.02E-07	3.56E-06
SNORD124	1.818256197	1.65E-06	6.09E-06
ACAN	3.981983607	1.72E-18	1.39E-15
ARTN	1.071917329	0.031904827	0.043781015
CDK8P2	1.681263433	0.000197995	0.000446011
GLP2R	-1.556935124	2.17E-06	7.80E-06
PCP4	-1.633526806	0.0001946	0.000439666
AK3P5	1.064853421	2.67E-05	7.42E-05
GTF3C2-AS1	1.368687511	5.96E-11	6.94E-10
EGFL6	1.743541035	2.87E-08	1.64E-07
TIGD1	1.732593588	4.84E-17	7.08E-15
MAB21L2	-1.620344838	5.86E-06	1.89E-05
AL360091.3	1.467025618	8.36E-05	0.000205645
CMA1	-1.955005622	1.26E-12	2.33E-11
AL132657.2	1.126003942	0.001785145	0.003245741
ASPM	2.435275475	3.80E-16	2.72E-14
SPDYE5	1.426853804	6.04E-10	5.41E-09
AC112721.1	1.644909745	3.51E-06	1.20E-05
RPL7P52	1.020976636	7.41E-06	2.33E-05
GRM8	3.073464494	2.16E-14	7.03E-13
ADRB3	-1.965769901	1.62E-07	7.66E-07
AL121657.1	1.006865094	0.004528869	0.00752608
SPP1	4.341971278	6.70E-14	1.81E-12
SERPINB9	1.04100697	1.88E-06	6.83E-06
FOXCUT	1.53365883	2.52E-06	8.90E-06
AC083798.1	1.525842967	2.21E-05	6.27E-05
TMOD1	-1.980877754	1.43E-09	1.14E-08
TRIM31-AS1	2.457732406	2.56E-13	5.79E-12
LINC01522	4.108404421	5.17E-06	1.69E-05
AC010789.1	4.861358836	1.65E-09	1.29E-08
AC087359.1	1.825348181	1.76E-08	1.06E-07
MIOX	2.971331438	9.36E-06	2.89E-05
IGHV1-14	-2.755502989	0.021946875	0.031363832

ZNF117	1.297994755	1.72E-05	5.01E-05
ZNF662	-1.01592689	4.46E-07	1.90E-06
EPCAM	1.027955591	5.16E-08	2.76E-07
RNU6-312P	1.196844161	0.000477783	0.000989743
LY6E	1.844198459	2.25E-11	2.95E-10
RNU6-623P	1.997157276	1.18E-05	3.56E-05
AC005757.1	1.390246231	3.02E-05	8.26E-05
GLI1	1.152098299	0.001494549	0.002763012
AC092814.2	1.461866482	3.45E-05	9.31E-05
DTL	2.343629181	2.72E-17	5.18E-15
AC025594.1	-1.155143939	0.015698497	0.023171156
RNU6-930P	1.619357711	1.04E-06	4.03E-06
PKMP5	1.261564762	1.16E-08	7.32E-08
YRDCP2	1.593683399	1.73E-06	6.37E-06
SNX10	2.121748954	1.03E-15	5.96E-14
HMGN2P40	1.160409133	0.00085483	0.001673356
AL162413.1	3.958576666	1.61E-06	5.98E-06
FOXD4	1.710038606	2.20E-07	1.01E-06
SPC24	1.426345139	2.64E-12	4.46E-11
HSD17B3	2.012679856	6.43E-07	2.64E-06
NPM1P40	1.109803372	4.15E-06	1.39E-05
SLC6A21P	1.945555247	0.01166431	0.0177122
B3GAT1	-1.58905384	0.002809266	0.004892115
AC096552.1	1.256659433	0.000824518	0.001619375
AC010435.1	1.434928404	5.38E-07	2.25E-06
AC008115.2	2.17231178	0.000890709	0.001738012
URI1	1.184694744	1.37E-10	1.46E-09
AC234772.1	1.220873243	0.000598557	0.001210381
NPIPB15	1.331331396	0.016928208	0.024792581
CYBB	1.009772343	0.000496589	0.001024796
SNORA69	1.605373703	0.000816808	0.001605414
DHCR24-DT	1.359325384	5.80E-08	3.06E-07
RNF43	1.808536875	1.57E-07	7.46E-07
AC009163.4	1.487205867	7.36E-08	3.80E-07
NFXL1	1.267354242	1.50E-15	7.95E-14
AC008738.2	1.053017283	0.025948314	0.036431017
ACOX2	-1.391133055	6.58E-09	4.41E-08
LINC00449	1.484187856	3.34E-09	2.41E-08
PDZRN3	-1.537683683	1.14E-06	4.37E-06
DNAAF5	1.091421123	6.06E-17	7.88E-15
R3HDML	3.393804941	1.86E-11	2.49E-10

ZNF816	1.10847879	2.61E-11	3.36E-10
AL162431.3	1.014647248	0.002500333	0.004398988
SLC17A9	2.117661236	6.90E-11	7.90E-10
AC002347.2	1.685783853	0.000804145	0.001582861
AC145207.9	1.093809667	1.18E-05	3.56E-05
AL133551.1	1.458267849	8.41E-08	4.28E-07
PCSK2	-2.125980902	5.92E-13	1.20E-11
INHBE	1.631792074	3.14E-08	1.78E-07
AC020629.1	1.45443484	5.35E-06	1.74E-05
FPR3	1.375692447	6.84E-06	2.17E-05
RPL13AP12	1.091973321	9.69E-07	3.79E-06
PWAR5	-1.723549384	6.28E-07	2.58E-06
PALLD	-1.169359427	0.016151057	0.023752062
PPP1R1B	2.320216128	0.000227706	0.000506642
PSORS1C3	2.336220755	3.33E-07	1.47E-06
RNASE3	2.675020467	0.001214375	0.002296194
AL451142.1	1.5831687	0.007623705	0.012069091
AL158166.1	1.933489875	3.41E-11	4.22E-10
PCAT7	1.211261942	7.47E-06	2.35E-05
AP001099.1	2.08475063	0.000727904	0.001445823
AC103691.1	1.99178752	7.19E-08	3.72E-07
SLC19A3	1.885619504	0.000601524	0.001215641
TTC26	1.352915785	8.76E-16	5.31E-14
MAP3K21	1.306408898	3.35E-10	3.20E-09
TMEM255A	-1.080440158	1.62E-05	4.73E-05
AC025287.2	1.278641469	9.34E-05	0.000227202
MAF	-1.258410492	4.90E-10	4.49E-09
GBP7	1.458884375	0.000259369	0.000569577
AC116158.2	1.220132283	0.002599573	0.004556436
MCF2L-AS1	1.559752051	3.54E-07	1.55E-06
TMEM74	-1.210789097	0.000133818	0.000313863
MIR3117	1.353014405	0.000791341	0.001559732
CDH16	1.982786849	0.003213304	0.005527431
TIPIN	1.242782821	1.22E-16	1.25E-14
AC026782.2	3.496499794	1.62E-07	7.68E-07
CACNA1H	-1.524305199	0.006163603	0.009949149
RNU7-70P	1.853139682	0.005752819	0.009348945
NR4A1	-1.32163122	0.027337972	0.038166864
KRT18P56	1.408884621	2.49E-05	6.98E-05
RNU6-80P	3.645727219	7.90E-08	4.05E-07
AL031665.1	3.108625818	9.74E-07	3.81E-06

AP002812.4	1.685110616	4.23E-05	0.000111698
AC127071.1	6.049861749	1.25E-06	4.75E-06
AC011290.2	1.545711686	1.11E-09	9.17E-09
AC079336.4	1.13002758	0.001106779	0.002112454
AC073575.2	1.59538485	3.97E-11	4.86E-10
CCSAP	1.233514006	4.47E-15	1.91E-13
RNU6-188P	1.541734559	0.001345467	0.002515151
LMNB1	1.58271073	1.06E-12	2.00E-11
PTCHD1	-2.716384033	5.25E-07	2.20E-06
RPL23AP46	1.045715079	0.000185401	0.000421311
LY6E-DT	1.381142785	6.74E-05	0.000169556
AC104135.1	1.777862967	0.004772518	0.007895937
TDO2	1.085216135	2.38E-05	6.68E-05
RN7SKP150	1.52145288	7.65E-07	3.07E-06
AC116552.1	1.250581988	8.87E-07	3.51E-06
AL138921.1	1.352827007	1.29E-08	8.08E-08
LINC02154	4.574827998	8.44E-11	9.44E-10
APOB	-4.336649738	0.000517258	0.001063105
RNA5SP219	1.535947139	1.58E-07	7.50E-07
RFWD3	1.38142646	2.44E-16	1.95E-14
CLN6	1.217890858	6.62E-14	1.79E-12
RPL34P22	1.139362865	0.005075103	0.008348928
GAS8-AS1	1.749459764	4.75E-08	2.57E-07
SYT12	1.264563877	0.022967372	0.032648422
CCNO	1.159568976	1.35E-06	5.10E-06
ATP10B	1.347048889	0.001431296	0.002659361
THY1	1.905665753	1.47E-12	2.66E-11
AC098591.2	1.041196457	2.91E-06	1.01E-05
AL391832.3	1.608842436	1.67E-06	6.16E-06
CYP2W1	3.973592651	1.93E-05	5.54E-05
MYO3B	1.967992449	0.007548397	0.011958398
AC107308.1	2.976624631	2.25E-07	1.03E-06
CFAP44	1.254062586	2.44E-09	1.83E-08
SLC39A10	2.105177303	5.01E-19	1.26E-15
AC135012.3	-1.289553953	6.08E-08	3.20E-07
AC006254.2	1.747548766	2.57E-05	7.18E-05
GK-IT1	1.772660216	1.33E-09	1.07E-08
NUTF2P6	1.555500708	1.60E-06	5.94E-06
PROM1	1.452206539	0.000270237	0.000591347
NUP58	1.097329159	2.54E-15	1.22E-13
SLC2A12	-1.143139621	1.56E-09	1.24E-08

SPATC1	1.218257753	1.15E-06	4.42E-06
COX5BP6	1.171344251	3.10E-07	1.37E-06
DSCC1	2.305894991	7.87E-19	1.26E-15
CPNE1	1.29182568	1.89E-14	6.30E-13
CCDC77	1.040016256	7.29E-14	1.95E-12
CDC42P3	1.002496745	0.00010209	0.00024612
AC016831.1	1.071417477	3.79E-06	1.28E-05
GPR15	-1.781811386	0.036102081	0.048993055
AC026412.2	1.579339669	1.20E-09	9.83E-09
MIR3155A	2.6491374	1.64E-06	6.05E-06
SLC12A9-AS1	1.629122313	2.02E-11	2.69E-10
HSPD1P6	2.252998087	1.15E-17	3.15E-15
OR7E126P	1.388715019	6.81E-07	2.77E-06
ATOH1	1.016436928	0.012753291	0.019194056
AC121336.1	1.408619015	0.001436545	0.002668556
AC024940.5	1.830159207	2.38E-11	3.10E-10
CYP51A1P2	1.360591488	1.24E-08	7.76E-08
RPS4XP23	1.583681415	0.000120458	0.000285266
HIGD1AP16	1.354144578	1.14E-05	3.45E-05
HMGN1P37	1.11200895	0.000448764	0.000935302
SLFNL1-AS1	1.067983667	5.11E-09	3.51E-08
AC083855.1	1.207458929	0.005310569	0.008696688
AC112191.3	1.428646701	9.11E-05	0.000222309
ARID3A	1.608316081	4.49E-05	0.000117669
LINC01671	2.534984061	0.000807872	0.001589467
POM121L9P	1.376446382	6.70E-07	2.73E-06
YWHAQP5	1.387013992	3.38E-05	9.13E-05
REEP1	-1.547842893	4.11E-07	1.77E-06
B3GNT8	-1.324455176	0.028001563	0.038968744
RNU1-108P	1.258264256	0.002796183	0.004869969
IGLV8-61	-2.019500911	0.026155487	0.036690557
AC006518.2	1.381806712	1.89E-05	5.44E-05
AC117500.2	1.196658967	1.85E-07	8.62E-07
TNFSF13B	1.063313559	1.26E-05	3.77E-05
BCL7A	1.113764042	3.73E-13	8.00E-12
AC011330.2	1.439304127	5.77E-08	3.05E-07
RNU4-48P	2.058462211	5.71E-05	0.000145945
HMGB1P3	1.257991028	4.07E-06	1.37E-05
PPIAP1	1.017692333	0.00051375	0.001056937
ADCYAP1R1	-2.862491338	1.30E-09	1.05E-08
RNU6-107P	1.288839895	0.000197044	0.000444171

SNRPGP10	1.048443776	5.72E-09	3.89E-08
FKBP14-AS1	1.225571001	1.33E-06	5.04E-06
RBM17P4	1.124687875	3.08E-06	1.07E-05
AC103834.1	1.106231321	0.000708703	0.001411261
AC128687.2	1.047738606	0.002002136	0.003599494
LINC00278	-1.076093232	0.004400387	0.007340959
SNRPCP14	1.96737174	0.000462847	0.000961524
KCNMB1	-2.100588636	4.04E-09	2.86E-08
AC090953.1	1.636195778	3.83E-06	1.29E-05
DEFB4A	-1.170188	0.000318045	0.000684447
EFCAB12	1.283159403	0.000383079	0.000809999
SAMD15	1.076653424	8.60E-07	3.41E-06
AL583810.1	1.858272464	1.16E-09	9.54E-09
AC009088.4	1.041940194	0.000584173	0.00118426
ASMTL-AS1	1.368490178	3.08E-07	1.36E-06
AC244093.4	1.838397869	7.26E-11	8.26E-10
AL691482.1	1.688467168	0.00066024	0.001322863
AC023509.5	1.209779819	2.59E-05	7.21E-05
SHCBP1	1.801471833	1.10E-14	3.97E-13
HHEX	1.174243664	1.47E-05	4.35E-05
HOXD13	5.189243901	0.018801495	0.027258893
PDCD5P1	1.309100924	6.81E-07	2.77E-06
AC012354.7	2.70121607	8.72E-06	2.71E-05
AC010487.1	2.064462114	5.52E-10	5.00E-09
HELLS	2.345364494	2.96E-18	1.63E-15
LINC01132	1.371618991	1.45E-10	1.54E-09
COL23A1	-1.261536013	7.87E-07	3.15E-06
AC079866.2	1.011184398	0.000278403	0.000607272
ITGA8	-1.733934096	6.48E-08	3.39E-07
CHRNG	1.051963717	0.000124054	0.00029295
AC005256.1	1.588115607	0.000845678	0.001657634
RPS3AP28	1.483541426	1.90E-06	6.91E-06
CACNA2D3	-1.45232443	2.17E-08	1.28E-07
RN7SKP130	2.12022204	0.000507522	0.001045255
SLC6A4	-3.325372343	1.05E-05	3.20E-05
MAP3K4-AS1	1.049569981	0.003002124	0.005197387
ANKRD13B	1.559309427	1.00E-11	1.44E-10
AC005104.1	1.208907609	8.32E-07	3.32E-06
TIMM9P1	2.042485649	6.43E-05	0.000162348
RPL21P136	1.185425219	3.17E-05	8.63E-05
CSE1L	1.487935166	5.24E-17	7.46E-15

RGS5	-1.170960711	0.001462607	0.002711864
AL035420.3	1.811125096	7.72E-06	2.42E-05
PFN1P6	1.460672349	4.03E-07	1.74E-06
TBX4	4.407749923	2.70E-05	7.48E-05
CHP1P3	2.150983397	2.57E-08	1.49E-07
AL121578.1	4.485495087	6.90E-06	2.19E-05
TCHH	1.489125526	0.000101044	0.000243733
LINC01762	2.375416343	1.03E-11	1.47E-10
SLCO1B3	4.797308697	1.34E-06	5.08E-06
APOBEC3B	1.750975323	4.25E-08	2.32E-07
SNHG20	1.038700051	6.26E-12	9.56E-11
IARS1	1.003776302	8.15E-13	1.57E-11
HSPA6	1.84677603	0.000131367	0.000308576
TNFRSF11B	3.395735904	6.21E-11	7.21E-10
AC092839.1	1.119283497	0.000738487	0.001465423
GFY	5.007503259	4.44E-06	1.48E-05
AC083900.1	1.316120068	0.000134504	0.000315224
AKAP8P1	3.379476255	1.29E-08	8.05E-08
LINC01579	-1.472418858	0.029718935	0.041130395
NKX2-5	2.93455573	0.007913877	0.012483215
PKD1L1	1.423270275	8.79E-09	5.74E-08
APOC1P1	2.389407234	1.99E-09	1.53E-08
LINC02696	-1.177742512	0.000523217	0.001073501
AC091965.5	1.42349374	0.009409532	0.014597842
RPLP0P2	2.312369252	1.02E-15	5.95E-14
MCMDC2	1.132276687	0.000314398	0.000677303
DOK7	1.043488196	0.034357114	0.046827761
RNU6-198P	1.2848136	0.010064593	0.0155246
HLA-DPA3	1.563407935	2.21E-06	7.91E-06
IGKV1D-17	-2.567640121	0.022292384	0.031800904
SNORD62A	2.330761556	1.46E-08	9.01E-08
AC091144.2	1.425574124	9.17E-05	0.000223551
AC000123.1	1.12798976	4.52E-10	4.19E-09
AC009237.15	1.496388473	4.86E-06	1.60E-05
LIMS1-AS1	1.176904914	2.53E-06	8.95E-06
RAET1K	2.960889658	3.13E-13	6.90E-12
TRIB2	1.141376708	1.10E-07	5.43E-07
IGHV3-35	-3.560077283	0.010996626	0.01679886
ZNF792	1.103784088	1.56E-12	2.79E-11
NOXRED1	1.267070448	4.06E-09	2.87E-08
ATP5MGP1	1.337960251	0.002280711	0.004054631

CFAP157	1.810421273	1.79E-18	1.41E-15
AR	-1.431080587	1.07E-05	3.24E-05
C14orf132	-1.266035545	5.17E-07	2.17E-06
GINS2	1.453796567	2.07E-09	1.59E-08
CKAP5	1.290698904	2.87E-17	5.31E-15
ABHD6	-1.042431681	6.07E-05	0.000154076
CDC42P1	1.053862538	1.91E-05	5.50E-05
MSH2	1.454861245	1.36E-16	1.34E-14
IGHA1	-3.063695171	0.003476586	0.005931681
AC093525.10	1.335433377	9.99E-06	3.06E-05
DDTP1	1.673209578	1.52E-05	4.49E-05
RPL35P4	1.139591994	3.17E-05	8.64E-05
GGCT	1.347685134	1.50E-15	7.95E-14
AC132219.1	1.22039435	4.77E-05	0.000124254
SYNDIG1	1.51699107	5.36E-06	1.75E-05
AC116351.1	1.520340255	0.000545123	0.001113668
H1-5	1.340596217	0.000564407	0.001148111
C2orf15	1.505368304	3.73E-13	8.00E-12
AC090774.2	3.08030305	0.000592071	0.001197991
G6PC	-4.629068916	0.005283822	0.008657539
IGHV3-19	-3.695235419	0.007452684	0.011822937
FOXD1	1.754380678	0.014818048	0.021986102
AC006028.1	1.655049926	8.35E-07	3.33E-06
CNTNAP2	2.388397459	0.00025271	0.000556191
AL049745.1	1.876596948	1.60E-05	4.69E-05
SCGB2A1	-2.522138719	0.000127497	0.000300466
LENEP	1.331130579	0.000301116	0.000651321
AC134407.3	1.1211497	5.38E-07	2.25E-06
PTTG4P	1.511303094	6.21E-06	1.99E-05
HLA-S	1.374119253	0.000572733	0.001163813
TSPEAR-AS1	2.225368045	4.13E-07	1.77E-06
LINC02065	2.570758788	3.54E-06	1.21E-05
AC246817.1	-1.203895743	3.90E-07	1.69E-06
AL356481.3	1.04254235	1.21E-05	3.65E-05
AC068987.3	1.054222178	0.003190789	0.005494731
AC084125.4	1.564010465	5.71E-10	5.16E-09
FAXDC2	-1.687549165	2.63E-10	2.60E-09
CPSF1P1	3.206181243	3.48E-10	3.32E-09
AL358075.1	1.029701586	8.47E-06	2.64E-05
KRT1	-5.476929988	0.002213001	0.003945051
LINC01010	2.191411723	2.13E-06	7.66E-06

AC092573.1	2.013487492	1.06E-06	4.11E-06
RNU6-1262P	1.198652561	0.000204274	0.000459147
RNA5SP464	1.124961255	0.004118958	0.006919008
PGAM1P11	2.092940639	5.54E-09	3.77E-08
ATG12P2	1.446001607	9.04E-05	0.000220806
AL356489.2	-1.515325193	6.14E-09	4.14E-08
RN7SL368P	2.113596075	2.44E-11	3.16E-10
AC112512.1	1.212796637	5.56E-07	2.32E-06
MIR4766	1.425759197	0.000338785	0.000724682
HMGN1P12	1.604360787	2.58E-05	7.18E-05
SLC35F2	1.025812541	3.37E-07	1.48E-06
AP003170.5	1.618243555	6.03E-07	2.49E-06
CLDN3	1.344664141	4.88E-07	2.06E-06
AC116337.3	-1.507920491	1.71E-05	4.98E-05
SLC5A6	1.649945033	4.79E-16	3.32E-14
AC022762.2	1.696796115	4.05E-10	3.79E-09
H4C14	1.292973073	0.001159994	0.002204163
ADAMTS12	3.442507526	1.21E-17	3.21E-15
LINC01597	1.936884495	0.000227005	0.000505337
PROZ	2.042724178	1.07E-06	4.13E-06
CEACAM8	2.148299344	9.82E-07	3.84E-06
ZGLP1	1.076570343	3.31E-08	1.87E-07
RNU6-48P	1.859112796	2.58E-07	1.17E-06
DLX6	3.753353196	0.001222739	0.002310042
RPL35AP4	1.267910099	0.00047163	0.000977986
HOXD9	1.268991218	3.05E-06	1.06E-05
PLA2G2E	3.018196373	0.000208192	0.000466851
ETV7-AS1	1.134348663	3.15E-05	8.58E-05
AL136982.3	1.528751234	7.22E-07	2.92E-06
KISS1R	2.34610852	0.003728533	0.006324666
SLC25A39P1	1.329478996	5.79E-07	2.40E-06
AC120349.3	1.342523323	5.52E-06	1.80E-05
AC092794.2	1.715843315	0.000242275	0.000535659
RNA5SP323	3.015270801	1.21E-09	9.87E-09
YTHDF1	1.045301065	2.91E-17	5.35E-15
RLN2	1.135840921	0.010475521	0.016082104
KIF2C	2.134499422	5.59E-16	3.73E-14
AP003072.2	1.390667683	0.000400134	0.000842918
TFAP4	1.271874045	1.88E-15	9.51E-14
RNU4-78P	1.297080157	2.08E-05	5.95E-05
NKRF	1.303260967	1.07E-17	3.14E-15

RNU6-541P	1.221854316	0.019389866	0.028006766
SCARNA8	1.446406761	3.24E-05	8.81E-05
AC024995.1	1.020209026	0.000350284	0.00074694
PARD6BP1	1.620629532	1.40E-08	8.65E-08
HSPD1P5	1.804493765	8.15E-13	1.57E-11
HAND2-AS1	-2.612074623	0.000163842	0.000376893
AC105129.3	1.677835636	1.47E-06	5.51E-06
PRSS22	1.105534411	3.29E-07	1.45E-06
AL606517.1	1.569062617	0.000252204	0.000555307
RNU6-122P	2.036055217	1.66E-07	7.82E-07
RNA5SP195	1.290836167	0.002388716	0.004223532
C6orf58	-1.356469729	0.000752024	0.001489318
AP001439.1	1.385623094	0.000413981	0.000868447
AL031600.2	1.698977651	1.55E-05	4.57E-05
CHODL	-2.517837556	4.00E-13	8.49E-12
IGLC6	-2.49810142	0.01369484	0.02045741
MIR548V	1.153466684	0.004480374	0.007453395
HMGB3P24	1.035732848	5.01E-06	1.65E-05
SPC25	1.718899513	6.55E-14	1.78E-12
AC010547.6	1.429978593	0.000185273	0.000421056
OCM	1.023751412	0.032165175	0.044117823
MANEAL	1.47869704	6.65E-07	2.71E-06
MYL9	-2.377499539	3.54E-07	1.55E-06
AL157400.4	1.121638926	4.34E-05	0.000114052
KNL1	1.934837239	4.74E-14	1.35E-12
AC000120.1	1.477530861	3.44E-06	1.18E-05
GAPDHP62	2.464923197	1.97E-14	6.52E-13
RHBDF2	1.310820644	4.35E-11	5.24E-10
AC008521.1	1.44361057	1.77E-05	5.13E-05
BX284668.5	1.300833976	0.0003428	0.000732682
BRDT	2.833941594	0.016293682	0.023939573
INSYN1	-1.809277348	7.50E-09	4.97E-08
SNORA71B	2.002814821	1.54E-13	3.71E-12
AL354928.1	2.009981235	1.06E-08	6.76E-08
RPS6P8	1.754075904	3.39E-05	9.16E-05
MIR4706	1.364339945	0.000241502	0.000534128
UBE2V2P4	1.155570435	0.001090759	0.002086893
CYCSP34	2.149789233	1.70E-11	2.30E-10
SEC62-AS1	1.15914771	1.58E-05	4.64E-05
AC011447.3	1.633623872	0.000433834	0.000906233
NCAPG2	1.79052648	5.38E-16	3.63E-14

CASK	1.287799758	1.39E-14	4.85E-13
AC005884.1	-1.308571072	3.02E-07	1.34E-06
AL031670.1	1.193157507	4.06E-08	2.24E-07
PKIG	-1.034222916	1.48E-05	4.38E-05
ZNF587	1.192886132	3.46E-14	1.04E-12
MRPL32P2	1.53662924	0.001168316	0.002217287
KIF21B	1.017633422	1.47E-06	5.51E-06
AC002398.1	1.207358513	3.28E-09	2.38E-08
OBP2B	6.959255506	1.83E-06	6.69E-06
AC002306.1	1.141668715	7.88E-05	0.000194956
MIR548I2	2.180411428	3.10E-07	1.37E-06
SNORD116-6	-1.181554317	0.000127088	0.000299557
CKMT2	-2.418893776	9.35E-14	2.41E-12
AC025031.1	1.441196685	1.49E-05	4.40E-05
M1AP	2.470849973	2.72E-06	9.53E-06
AC004217.2	1.861892116	8.77E-06	2.72E-05
AL355987.5	2.05955779	8.37E-08	4.26E-07
APOC3	-5.01383222	0.023056499	0.032771619
PA2G4P4	1.151018404	7.06E-12	1.06E-10
AL118522.1	1.536689094	0.018857146	0.02732025
AC091868.2	1.529829048	2.14E-05	6.09E-05
AL136307.1	1.530818041	0.011000844	0.01680434
AP000943.1	4.892190893	5.84E-10	5.26E-09
PENK	-2.822265122	0.003344305	0.005733895
RPS2P36	2.029782361	7.58E-08	3.90E-07
FILIP1	-1.507597316	0.014312632	0.021302634
NUP37	1.091351737	1.61E-14	5.51E-13
AC011462.3	1.205361303	0.004833794	0.007988953
AL162741.1	1.062738766	7.69E-06	2.42E-05
AC004066.1	1.058341494	8.13E-05	0.000200538
SETP20	1.299164757	6.25E-08	3.28E-07
COX7BP2	1.26222187	0.000197306	0.000444649
LUCAT1	2.039975971	1.18E-07	5.77E-07
C3P1	5.370452407	5.89E-13	1.20E-11
PURPL	4.486483733	9.75E-07	3.82E-06
MMP13	4.483820872	1.08E-11	1.54E-10
THOC2	1.17620777	2.20E-16	1.84E-14
RNA5SP479	1.595692538	9.38E-05	0.000228095
ZNF702P	1.145896348	0.000104352	0.000250804
AC007191.1	1.155155133	1.27E-08	7.95E-08
AL732372.2	1.722740494	0.000587783	0.001190407

AL078644.1	1.427091785	1.55E-10	1.62E-09
CENPN-AS1	1.24157018	9.37E-09	6.08E-08
AC048344.4	1.589730787	5.21E-09	3.57E-08
MIR135B	2.742017355	1.26E-08	7.88E-08
MTND2P13	2.929736832	0.012793268	0.019243351
AP000553.2	1.855714273	3.29E-06	1.13E-05
AC004908.2	1.135254152	1.09E-08	6.91E-08
COL18A1	1.281820404	1.04E-08	6.64E-08
H3P16	1.779521318	3.66E-07	1.59E-06
NOX4	1.948395229	4.55E-12	7.21E-11
AC132938.5	1.441888998	1.88E-10	1.92E-09
AL121832.3	2.414433268	1.63E-14	5.55E-13
NEK5	1.290264301	6.65E-09	4.45E-08
LAMP5	1.648931736	0.015740506	0.023224154
RPL7P50	1.235966038	3.63E-07	1.58E-06
SMIM35	2.619254602	4.44E-11	5.35E-10
IGKV1OR2-6	-3.169686204	0.02789569	0.038841694
USP12P1	1.005999584	0.001013009	0.001953853
AC011443.1	1.220124036	5.71E-05	0.000145935
AL136295.5	1.343863813	1.76E-09	1.37E-08
RN7SL760P	1.763003447	0.000204779	0.000460166
AC027763.1	1.577574855	0.000616069	0.001242303
DUS4L	1.166842774	1.96E-16	1.70E-14
PRMT1P1	1.155920451	2.94E-08	1.68E-07
AC079336.7	1.043460617	0.000859464	0.001681069
Z94721.1	1.718855618	3.30E-11	4.11E-10
CEBPD	-1.016087921	2.38E-05	6.68E-05
PPIAP78	1.445819664	3.01E-06	1.04E-05
DENND5B-AS1	1.249395446	1.58E-05	4.64E-05
MRPL35P3	1.204812581	0.000118716	0.000281565
DUXAP9	3.652306038	3.68E-14	1.10E-12
AL807757.2	1.545599253	2.38E-08	1.39E-07
CAB39P1	1.505318419	2.47E-05	6.91E-05
VN1R42P	1.235155022	3.01E-06	1.04E-05
AC069499.1	2.109450792	2.35E-14	7.54E-13
RNU6-757P	1.650758597	8.89E-05	0.000217542
RANBP1	1.041041811	3.44E-13	7.48E-12
AC246787.2	-1.631563732	0.023263169	0.033038933
AC121761.1	1.27973489	3.72E-07	1.61E-06
AL161733.1	3.156504101	7.38E-05	0.000183915
TFPI2-DT	3.13537844	0.000258173	0.000567277

IRAG1	-1.130351158	0.000378546	0.000801688
PPIAP11	1.069425852	9.64E-09	6.22E-08
CEACAM7	1.50567358	0.010933317	0.016712877
AC138904.1	2.179265163	1.40E-05	4.16E-05
AC103563.2	-3.03651063	1.19E-07	5.82E-07
KCNJ2	1.070299387	0.000136465	0.000319314
ORC6	2.215214155	2.32E-16	1.88E-14
RNA5SP179	1.802615709	0.000165683	0.000380791
LINGO1	1.922460311	3.22E-09	2.34E-08
MIR1-1HG-AS1	-2.414792018	2.89E-08	1.65E-07
IFNL2	4.322276553	1.98E-07	9.19E-07
ZNF28	1.329064517	3.23E-12	5.30E-11
AC011921.3	1.040095371	4.00E-06	1.35E-05
AC112694.1	1.583116371	4.30E-11	5.20E-10
AL031708.1	1.465081901	7.97E-09	5.25E-08
Z97192.1	2.573707775	3.12E-05	8.51E-05
FBXO39	1.368865029	0.000266966	0.000584722
AC012254.1	2.166910972	2.11E-05	6.03E-05
MEX3A	2.925158892	7.42E-16	4.64E-14
SETBP1	-1.110368896	1.63E-05	4.76E-05
RPS4XP21	1.069440412	0.00105114	0.002019796
AC126564.1	2.040815786	0.009356926	0.014528073
STRA6	4.856689985	1.39E-16	1.37E-14
CFL2	-1.609008682	0.000297775	0.000644618
RPSAP21	1.144029718	7.17E-07	2.90E-06
TESMIN	2.407155744	9.10E-16	5.46E-14
AL592148.1	1.228308279	4.98E-07	2.10E-06
AC127024.7	1.021018327	0.001280065	0.002405211
NPM1P5	1.329369427	6.59E-07	2.69E-06
AP000879.2	1.578049882	0.000106403	0.000255364
MIR6783	3.15115235	4.25E-06	1.42E-05
PPP1R14BP4	1.543810238	1.39E-06	5.22E-06
MSH4	1.984081157	8.36E-06	2.61E-05
AC027176.2	1.477390889	1.90E-09	1.47E-08
LAMP3	1.793323987	5.25E-10	4.79E-09
NME9	1.106034691	4.65E-07	1.97E-06
AC009120.3	1.191686179	4.78E-07	2.02E-06
SNORD53	1.717129708	0.002680217	0.004685148
AC010719.1	1.382402193	5.10E-05	0.000131965
SPDYE22P	1.399418498	3.49E-09	2.51E-08
AC004771.4	1.100458868	9.88E-07	3.86E-06

GABRD	3.328669465	6.20E-19	1.26E-15
VSTM2A	-3.26940597	2.00E-10	2.03E-09
SCN2B	-1.33056735	1.35E-07	6.50E-07
AC106782.5	1.417441501	3.55E-11	4.38E-10
SNHG3	1.131402944	3.31E-09	2.39E-08
AC099066.3	1.15260141	0.000187412	0.000425228
LINC01775	1.502229338	6.89E-07	2.80E-06
AC127070.3	1.132904034	6.31E-05	0.000159583
AGO2	1.112132685	3.91E-13	8.34E-12
CFAP161	1.406560284	4.80E-07	2.03E-06
CEMIP	4.125369531	1.63E-16	1.50E-14
GSTM1	-1.3621731	0.004545694	0.007551245
HRAT92	1.768753999	7.84E-05	0.000194103
RNU6-1280P	1.003118351	0.000411241	0.000863693
AC010536.1	1.419174651	8.37E-10	7.17E-09
SASS6	1.420338337	2.68E-16	2.08E-14
CASR	-1.069863435	0.002760991	0.004813712
CA6	5.097070665	0.002257531	0.004017174
GOT2P6	1.5984485	1.73E-05	5.03E-05
IGFBP1	5.519011329	1.35E-09	1.08E-08
AC074117.2	1.075673051	1.31E-05	3.91E-05
LINC01511	3.904470437	9.91E-05	0.000239537
CBX4	1.100389819	7.19E-11	8.19E-10
SLC27A6	-1.894157822	8.14E-07	3.25E-06
AC025580.1	1.500974	0.012494791	0.018840186
KY	-1.844556698	3.82E-05	0.000101962
BBC3	1.508424532	7.38E-12	1.11E-10
XPO4	1.116342682	4.87E-13	1.01E-11
AC016559.3	1.328228018	0.002891461	0.005023102
AP000317.1	-1.307309654	0.008386763	0.013158243
AL356234.2	2.587014645	7.83E-08	4.01E-07
KRIT1	1.090209706	1.37E-09	1.10E-08
AC012313.8	1.48918315	5.56E-09	3.79E-08
OAS2	1.361470833	1.47E-07	7.03E-07
AL590609.2	1.666237257	0.004322307	0.007223367
AC111170.1	1.305702753	3.33E-05	9.02E-05
RNU6-155P	1.095773155	0.016366151	0.024030146
PTMAP15	2.03454621	0.003580744	0.006096899
SOX17	-1.292631231	0.00012016	0.000284585
AC022150.4	1.222575044	7.74E-05	0.000191901
AC004938.1	1.035548299	0.000558898	0.001138653

CENPH	1.422034113	8.71E-14	2.27E-12
CBWD4P	1.411990697	1.86E-06	6.78E-06
PSMC1P2	2.776767866	3.99E-08	2.21E-07
ART3	-1.779408754	0.009354155	0.014524618
GCOM1	-1.178289171	6.31E-06	2.02E-05
RPL21P121	1.487592445	4.01E-05	0.000106383
AL031710.1	1.363671896	2.25E-05	6.36E-05
AC024901.1	2.176908004	0.002666924	0.004664666
PPIAP60	1.546571475	4.28E-08	2.34E-07
AC007014.2	2.292905035	4.53E-12	7.20E-11
LINCR-0001	2.822394799	1.86E-08	1.12E-07
AL353747.3	2.016914621	0.01190521	0.018038936
AL031768.1	1.021187355	7.76E-05	0.00019238
LINC01358	-1.436429889	0.007003936	0.011177601
MAGEA11	6.280929806	0.001036468	0.00199376
RNU6-282P	1.352085764	1.01E-05	3.09E-05
SSBL2P	1.273093329	9.29E-09	6.04E-08
WDR88	1.086931732	2.53E-06	8.95E-06
MKI67	2.253104818	2.29E-16	1.87E-14
AC112496.1	1.231320401	7.81E-08	4.00E-07
TRIM50	-2.971246991	2.91E-05	8.00E-05
AC112497.2	1.080416855	1.02E-12	1.94E-11
PLEKHA8	1.071252962	9.46E-16	5.64E-14
AC243964.4	1.100669736	1.99E-07	9.22E-07
REG3A	-1.730403621	0.008812783	0.013756161
CYP4F23P	2.347566739	9.74E-06	2.99E-05
ADORA1	1.132537569	0.000150039	0.000348139
LINC02886	1.313453579	5.49E-05	0.00014094
PMCH	2.617979758	7.40E-10	6.46E-09
AC004943.1	1.223610871	6.59E-08	3.44E-07
RNU4-89P	1.31736272	5.83E-06	1.89E-05
AC005730.3	1.210783285	1.10E-06	4.25E-06
AC113191.1	1.463810006	6.83E-12	1.03E-10
AC093702.1	-1.999910182	0.020316151	0.029225858
BPIFA2	4.426011705	2.55E-06	9.01E-06
SLC18A3	-2.738572964	0.000129267	0.000304234
FANCL	1.078012509	4.25E-15	1.83E-13
HMGB1P4	1.005984273	3.23E-06	1.12E-05
AC018653.3	1.528478031	2.37E-11	3.09E-10
USP43	1.243090006	1.24E-08	7.76E-08
MGC32805	1.487649776	0.011216395	0.017105163

SCUBE2	-1.673012565	3.00E-10	2.92E-09
LINC02820	3.642721972	0.000196777	0.000443643
FMR1-IT1	1.481288112	4.13E-08	2.27E-07
ITIH5	-1.488691727	7.69E-06	2.42E-05
RN7SL750P	1.106590467	0.001912259	0.003450952
CHIA	-1.55887386	0.000297727	0.000644618
RBP7	-1.167173237	3.20E-08	1.81E-07
HCFC1-AS1	-1.169288012	0.005069458	0.008340156
AL391832.2	1.87314142	8.15E-11	9.15E-10
AC020922.3	1.811213803	7.18E-09	4.77E-08
HPRT1	1.008115672	2.61E-12	4.41E-11
SNORA70G	1.07813755	0.000311458	0.000671565
GAS2L3	1.637112382	2.01E-14	6.64E-13
PTPRK-AS1	1.64260278	7.84E-08	4.02E-07
HELB	1.450032565	5.88E-14	1.62E-12
CD55	1.411395628	1.85E-06	6.74E-06
LINC02340	2.485351239	8.66E-08	4.40E-07
PDE2A	-1.447921284	4.16E-09	2.93E-08
ZNF812P	-2.688396434	0.000505347	0.001041066
SYT13	2.094384202	2.39E-05	6.72E-05
AC110768.2	1.146890634	0.000170617	0.000390916
RBM12B-AS1	1.768008751	1.17E-15	6.58E-14
TNFRSF17	-2.465916136	0.022362529	0.031895845
CASC8	2.08754119	1.01E-07	5.03E-07
RNVU1-21	1.658503532	2.91E-05	7.99E-05
CCL7	4.52854145	7.91E-13	1.54E-11
LMTK2	1.046569634	4.03E-08	2.22E-07
DDAH1	1.102941075	9.33E-11	1.03E-09
SNORA11	-1.6141758	0.021924452	0.03133476
AC092115.1	1.021891421	7.90E-11	8.91E-10
AL691482.3	2.185617396	3.77E-11	4.63E-10
FAM227A	1.856978047	3.26E-12	5.35E-11
AC005381.1	3.872276929	4.48E-05	0.000117384
AC244093.5	1.996491726	1.52E-10	1.60E-09
U73166.1	1.133270833	2.15E-07	9.87E-07
AC016866.3	2.215066876	2.05E-08	1.22E-07
MCEMP1	2.258149483	3.69E-07	1.60E-06
FTLP2	1.065317266	6.65E-07	2.71E-06
AC004264.1	2.622456764	1.50E-15	7.95E-14
DACT1	1.102069514	0.000177729	0.000405745
VRTN	2.342848008	0.000945439	0.001836826

PRSS51	3.650875762	8.36E-10	7.17E-09
AC016405.1	1.25285017	2.12E-06	7.61E-06
MIR2116	1.806974781	6.65E-07	2.71E-06
AL049869.2	1.137040819	5.63E-07	2.34E-06
AC012354.4	3.615583409	5.07E-07	2.14E-06
HMGB3	2.048871178	1.24E-16	1.25E-14
HSP90AB3P	1.461214046	2.47E-14	7.85E-13
LNCOC1	1.48416717	1.13E-06	4.35E-06
TTC34	1.132777848	0.002654988	0.004645316
AC005291.2	1.192008976	0.008847881	0.013804477
SUCNR1	1.831797429	1.94E-10	1.97E-09
ARL14EPP1	1.858340786	3.57E-10	3.39E-09
AC092447.2	1.157146902	4.58E-06	1.52E-05
TPM3P2	1.950812467	9.57E-05	0.000232289
NDUFAF6	1.017304793	3.18E-14	9.66E-13
PER3	-1.094716653	8.94E-05	0.000218403
PDZD2	-1.166801002	0.008793857	0.013728227
CGREF1	1.090998867	0.000404111	0.000850085
APOA1	-3.420695378	0.01064282	0.016309828
UBE2SP2	1.69181148	4.19E-12	6.70E-11
SNORD12C	1.686776617	7.63E-09	5.05E-08
RPS27AP8	1.087616569	0.00530869	0.008696681
RHOQ-AS1	1.12424849	2.94E-05	8.08E-05
TMSB15A	1.885125889	1.34E-08	8.36E-08
CDH19	-2.39641529	4.70E-10	4.34E-09
SLC38A7	1.128561968	3.35E-18	1.71E-15
AL035461.2	1.799228376	1.72E-12	3.05E-11
TUBA5P	1.897429816	3.63E-10	3.43E-09
AC127024.3	1.344108034	8.70E-06	2.71E-05
H3C7	1.119621989	0.003354427	0.005748667
RPSAP69	1.428814977	4.17E-09	2.94E-08
AC004231.1	3.049777118	0.000849904	0.001664814
RNU6-893P	1.526293248	0.000461456	0.000959202
LRRK2-DT	-1.12126933	3.52E-06	1.20E-05
LDB3	-2.314953582	5.31E-09	3.63E-08
AC092718.5	1.313284787	3.13E-05	8.52E-05
AL445490.1	1.680223103	1.11E-07	5.47E-07
AL021407.1	2.348142672	4.53E-07	1.93E-06
TGS1	1.173998136	2.72E-17	5.18E-15
FSD1L	1.084428483	2.63E-06	9.27E-06
AC027130.1	1.444194381	2.05E-06	7.38E-06

RPL7P25	1.168190594	6.43E-07	2.64E-06
NCAPD2P1	3.718410405	3.59E-12	5.84E-11
AC008622.2	1.011532685	0.000182787	0.000416187
RNU1-91P	1.11423972	0.010879427	0.016637948
AL138966.2	1.174492999	2.25E-06	8.05E-06
AL121890.5	1.091803063	0.003907954	0.006602557
SNORD51	1.41121739	0.000535205	0.001095662
PDC-AS1	1.12686493	2.10E-05	5.99E-05
AC015871.4	1.290309413	3.61E-06	1.23E-05
SIGLEC7	1.15037577	5.31E-05	0.000136807
RNU7-59P	1.155722194	0.007921466	0.012494447
RNU6-942P	1.114569746	0.000386691	0.000817118
MYOC	-4.805691164	1.74E-16	1.57E-14
PCNPP1	1.339462896	5.86E-10	5.27E-09
C3orf35	1.210285079	3.59E-08	2.01E-07
AC245884.8	1.563626072	1.56E-09	1.24E-08
PVT1	2.147162904	3.19E-15	1.46E-13
AC118755.2	1.785150234	0.001203551	0.002276535
BATF2	1.729734452	6.43E-07	2.64E-06
TRIM71	5.315716791	3.90E-05	0.000103872
MFAP3L	1.152695477	0.000710422	0.001414051
LINC00460	3.549581819	5.21E-10	4.75E-09
ZNF572	1.338659773	8.19E-08	4.18E-07
MIR320C1	1.920911879	2.40E-06	8.52E-06
RPS26P58	1.042900731	0.000394634	0.000832383
SLC30A7	1.081305053	2.10E-15	1.05E-13
AC023043.1	2.375633653	4.80E-09	3.33E-08
ERV3-1	1.159054014	0.00198301	0.003566793
MTCO3P44	2.814203581	1.07E-08	6.80E-08
EGR4	2.099710451	0.00033183	0.000711356
F2RL2	2.055547913	7.98E-11	8.99E-10
PLAC9	-1.698128031	1.37E-10	1.46E-09
GXYLT1	1.113778171	7.42E-16	4.64E-14
AC012363.1	5.05576157	9.63E-15	3.57E-13
AC011747.1	3.984251703	4.74E-05	0.000123681
SPATA13-AS1	1.017348913	4.24E-05	0.000111921
TEX30	1.119255301	4.30E-10	4.01E-09
FCF1P8	1.003334633	3.18E-05	8.64E-05
NCF2	1.009994102	0.000165913	0.00038122
TESC-AS1	1.331653676	0.00340989	0.005829833
AL031846.1	1.44497446	2.14E-06	7.68E-06

AC023983.1	1.080141708	0.000221914	0.000494914
AC104996.2	1.180083473	0.000154837	0.000358123
LINC00622	1.242016361	0.0004364	0.000911318
SGCG	-2.12665539	1.01E-08	6.51E-08
TMEM63A	1.378939609	2.89E-13	6.45E-12
CXCL17	-1.626986053	6.88E-05	0.000172502
AP003467.1	1.91446669	1.67E-07	7.88E-07
DONSON	1.346642042	1.83E-16	1.62E-14
GABRR2	1.253654399	3.24E-05	8.81E-05
HNRNPA1P39	1.137624696	1.00E-07	5.01E-07
RCC2	1.624288336	9.35E-18	2.90E-15
CYP19A1	2.970037578	5.49E-13	1.13E-11
LBX2	1.982854647	1.33E-12	2.44E-11
H2BC20P	1.142241382	1.57E-08	9.59E-08
PCNA	1.559200105	1.06E-14	3.88E-13
ACSM3	1.052190491	0.000952764	0.001849035
NOLC1	1.21747145	8.01E-16	4.96E-14
AC097634.1	1.020289869	1.41E-05	4.19E-05
AC093788.1	1.720563199	1.26E-10	1.35E-09
ARGFXP2	1.649277163	7.22E-10	6.32E-09
CBWD6	1.398846671	1.02E-11	1.47E-10
SNX29P1	1.046394653	0.022391075	0.031933142
HMGN2P25	1.475905686	2.32E-06	8.25E-06
PIANP	-1.254092888	5.47E-07	2.28E-06
AC005479.2	1.85665406	1.59E-08	9.69E-08
AL121832.2	1.085074	1.94E-07	9.02E-07
SMCO2	1.280330611	3.10E-07	1.37E-06
FCER1A	-1.465208133	1.96E-10	1.99E-09
AC096642.1	1.27744585	4.60E-06	1.52E-05
NFASC	-1.331254701	0.0006299	0.001267221
NCAPD2	1.381063693	4.30E-15	1.85E-13
PPP1R35-AS1	1.273702862	2.79E-05	7.71E-05
RNU6-45P	1.131166519	0.004467478	0.007435656
TPSG1	-1.720300531	3.57E-07	1.56E-06
GBP4	1.740202821	6.66E-05	0.00016754
RPL22L1	1.471160602	3.57E-06	1.22E-05
MIR3690	1.851275369	0.000386246	0.000816243
PLCH1	1.117351214	1.10E-06	4.25E-06
LINC01210	4.926484268	1.93E-05	5.55E-05
PYY2	2.582993878	2.73E-10	2.69E-09
ITPKB	-1.16102039	0.003978657	0.006705851

AC010533.1	1.5669075	2.16E-06	7.76E-06
MEIOC	1.422836744	4.32E-08	2.36E-07
RERGL	-1.37230396	6.97E-11	7.97E-10
SNORD11B	1.871539155	1.77E-06	6.50E-06
AC012531.4	6.498625481	5.24E-09	3.59E-08
SNORA19	1.855675001	0.000107789	0.000258177
OSBPL10	1.049764942	2.45E-10	2.43E-09
AL390961.2	1.448965155	7.09E-06	2.24E-05
FOXF1	-1.61089506	1.59E-05	4.67E-05
IGLV2-18	-2.684344569	0.015504523	0.022910234
VARS1	1.080043775	1.99E-13	4.64E-12
LINC02228	1.396541725	0.033649831	0.045960156
AL442663.2	1.071343391	0.000270802	0.000592438
ADCY3	1.018153112	2.94E-11	3.72E-10
GPM6B	-2.503476401	2.17E-09	1.65E-08
RNU6-501P	1.305302272	0.010507937	0.016125365
BX322635.1	1.18788428	0.002640831	0.004623587
FERP1	1.641991321	5.11E-09	3.51E-08
AC007497.1	2.108915929	1.50E-08	9.22E-08
VTRNA1-3	-1.117684099	0.033110809	0.04528895
AL353572.2	1.344047793	0.000167831	0.00038533
PRDM16-DT	-1.962561653	0.000561183	0.001142259
NPM1P33	1.300787061	2.62E-06	9.23E-06
NUP43	1.009260932	2.88E-15	1.34E-13
AC007406.3	2.309738004	2.28E-14	7.38E-13
AC108449.1	1.154383136	5.84E-05	0.000148982
PTRH2	1.033013853	6.02E-15	2.42E-13
AGTR1	-1.72575854	2.11E-07	9.71E-07
AC021242.3	1.23325397	1.42E-05	4.21E-05
RPS26P49	1.429863049	0.002580457	0.004525014
AC015911.7	1.172279558	6.25E-05	0.000158271
ASCL2	2.957623762	1.20E-06	4.59E-06
IGHV8-51-1	-3.603678948	0.019327338	0.027925535
SAP30-DT	-1.385102664	0.000591193	0.001196306
IGHVII-22-1	-1.916319004	0.031069111	0.042753125
NPM1P9	2.137829643	1.68E-14	5.69E-13
ARL6IP1P3	1.537981642	2.50E-07	1.13E-06
AC122688.3	1.302530059	9.51E-10	7.99E-09
SEM1P1	1.645376935	3.58E-06	1.22E-05
RN7SL652P	1.803598167	1.41E-05	4.19E-05
IGLV3-10	-2.432837382	0.035711112	0.048489874

AC090617.4	1.025663557	5.20E-05	0.000134352
TMEM267	1.184082524	1.85E-15	9.43E-14
AL589182.2	3.216627246	1.46E-09	1.16E-08
DRC1	1.57632754	0.013458256	0.0201424
AL360268.1	1.209259788	2.91E-05	7.99E-05
IGHV3-62	-2.947848323	0.008777462	0.013708256
RSF1-IT2	1.256772024	1.54E-05	4.52E-05
CYP2B6	1.800758699	6.23E-07	2.56E-06
GGT5	1.001249872	0.00032013	0.000688426
PABPC4-AS1	1.43921268	3.28E-09	2.38E-08
CRIP1P4	1.410452359	0.000204769	0.000460166
EDN3	-1.977621065	0.001053094	0.002022589
RNU6-780P	1.133456729	6.37E-05	0.000160882
AL606490.8	1.995996725	5.26E-05	0.000135547
YPEL5P2	1.05950556	0.000543971	0.001111813
AC060780.2	1.157807591	5.07E-08	2.72E-07
AL592435.1	1.194372425	0.000102346	0.000246672
AC104958.1	1.460180319	0.002488913	0.004381033
CYSLTR1	-1.062724856	0.000135603	0.000317602
AC027559.1	-1.226709425	0.000861117	0.001684063
AC131571.1	1.59164172	6.81E-06	2.16E-05
ORC1	1.978608374	1.94E-14	6.43E-13
AL445991.1	1.50524012	1.33E-06	5.03E-06
PTGES2-AS1	3.27202553	4.07E-12	6.55E-11
AL513008.1	1.283229939	0.027867212	0.038808126
LEFTY1	5.544755943	0.000199993	0.000450348
AC073323.1	5.966996716	2.53E-11	3.27E-10
RNA5SP129	1.09790421	0.006617283	0.0106149
AC012569.1	1.672204855	7.12E-08	3.69E-07
CYRIB	1.182788728	3.19E-15	1.46E-13
IER5L	1.836689888	5.92E-13	1.20E-11
HIF3A	-1.279355982	1.11E-05	3.35E-05
L1TD1	2.222965558	5.56E-06	1.81E-05
MIR200C	2.086171135	0.000185083	0.00042066
AC108058.1	1.035692619	0.000389973	0.000823531
AC010894.2	2.634054674	0.027864582	0.038806492
AC007285.1	1.561985665	1.57E-08	9.63E-08
H2AC4	1.632457235	1.47E-05	4.34E-05
AC233266.2	-1.784535716	0.013784568	0.020578755
ARHGAP26-IT1	1.388074201	0.000205582	0.000461737
AL513217.1	-2.193440667	4.87E-05	0.000126511

AL031668.1	2.01570958	3.13E-10	3.03E-09
RNA5SP212	1.403418088	0.000195612	0.000441391
LINC00216	1.215263254	6.52E-09	4.38E-08
AC006023.2	1.374672392	0.000550092	0.001122772
C6orf223	4.012296069	9.38E-12	1.36E-10
AL358075.2	1.522214012	7.56E-05	0.000187933
WFDC10A	3.432018929	7.28E-06	2.30E-05
CYP1A1	-2.682417337	0.000887412	0.001732299
DEPDC1B	2.151387333	3.35E-15	1.52E-13
RNA5SP277	1.23182775	0.002980456	0.005163566
RN7SKP271	1.196207995	4.12E-05	0.000108961
AC109460.3	1.088819562	5.94E-09	4.02E-08
TNFSF15	1.483018055	1.49E-09	1.19E-08
AC073517.1	1.139602722	6.01E-06	1.94E-05
PTN	-1.925389668	1.24E-11	1.74E-10
EXO1	2.534105987	2.38E-17	4.88E-15
AP002449.1	1.756859597	1.84E-10	1.89E-09
SEZ6L2	1.606366004	4.44E-09	3.09E-08
RNU6-703P	1.238096624	0.000830009	0.001629318
OVGP1	1.193742439	3.19E-06	1.10E-05
RPL39P16	1.717442869	6.84E-10	6.03E-09
ADAMTS9-AS1	-2.510345845	0.000457452	0.000951475
CCR1	1.047686175	2.85E-05	7.84E-05
SNORD121B	1.07560338	0.000582713	0.001181479
TICRR	2.062547598	5.81E-14	1.61E-12
C2orf50	1.832636251	2.27E-07	1.04E-06
AL390728.5	1.514118541	1.23E-11	1.72E-10
AC067945.2	1.099721113	2.86E-05	7.88E-05
AC005828.6	2.452090041	1.11E-07	5.47E-07
RPL36AP36	1.120834211	0.000493878	0.001019596
AC002563.1	1.122436804	0.000789087	0.00155575
FANCM	1.096864326	8.93E-13	1.71E-11
AC004148.1	1.303919235	3.89E-09	2.76E-08
AC097478.1	2.728521777	0.018006774	0.026233743
BRSK2	1.318748401	0.0233466	0.033136228
AC091153.3	1.715474442	5.25E-08	2.80E-07
CCKBR	-2.725269653	5.61E-09	3.82E-08
AC022098.4	1.120495392	0.001249614	0.002355303
AC073651.1	1.406704277	2.12E-05	6.04E-05
SINHCAF	1.592009774	1.57E-15	8.25E-14
UCN	1.533597132	3.12E-10	3.02E-09

RNA5SP383	1.264038872	2.17E-07	9.95E-07
STK32C	1.382560432	7.51E-10	6.52E-09
AC008740.1	1.18944261	4.44E-05	0.000116471
PTH1R	-1.55129281	2.35E-09	1.77E-08
ZNF8-ERVK3-1	1.473418815	7.06E-12	1.06E-10
PYGM	-2.75149171	7.36E-10	6.43E-09
AC079305.2	-1.700756648	1.45E-06	5.45E-06
PSRC1	1.452951172	6.40E-12	9.75E-11
PLA1A	1.728677446	1.00E-06	3.90E-06
ITGB8	1.330159205	1.72E-08	1.04E-07
DGAT2L7P	1.123303912	0.000222425	0.000495763
RNU6-1206P	1.579534784	0.000157639	0.000364124
AC034102.3	1.205116833	0.018743387	0.027177605
AC113346.1	7.297685956	2.43E-10	2.43E-09
LAGE3P1	1.485364111	1.52E-08	9.35E-08
FJX1	1.308895619	7.43E-10	6.47E-09
MNX1-AS2	1.765293569	6.40E-10	5.69E-09
ABI3BP	-2.256034193	3.66E-07	1.59E-06
PTPN13	-1.382651922	0.00201462	0.003619984
RNU6-854P	1.995934748	0.003026363	0.005236628
PPFIA3	1.006299086	6.54E-07	2.67E-06
TPSD1	-1.467850486	7.06E-05	0.000176737
RNU6-554P	2.005780863	7.80E-05	0.000193239
LGALS2	1.762069418	0.017073005	0.024982644
PROX1-AS1	2.658678162	0.023343154	0.033136228
RPL21P132	1.129587174	2.46E-06	8.72E-06
C1QTNF2	-1.611322705	4.69E-07	1.99E-06
AL137077.1	1.402260249	4.47E-06	1.49E-05
GRASLND	2.597557073	4.41E-14	1.28E-12
AC008735.1	1.478028221	1.50E-07	7.16E-07
FOXM1	2.238712024	6.44E-16	4.14E-14
TUBB3	2.338402881	1.54E-11	2.11E-10
TMEM38B	1.01955405	9.42E-10	7.93E-09
BNIP3P42	2.437801522	1.25E-09	1.02E-08
SHANK2-AS1	1.970349072	2.73E-07	1.23E-06
ITCH-AS1	1.030547429	0.003391023	0.00579981
NSG2	-1.382445752	5.23E-08	2.80E-07
NPPC	-2.737151863	4.20E-10	3.93E-09
AL445187.1	1.184226599	3.08E-06	1.07E-05
AL355472.2	1.542835815	0.001107189	0.002112866
C1QL4	2.408225481	0.003580487	0.006096851

ZNF474	1.162485042	0.00575004	0.009344999
AC072052.1	1.116385124	0.001642395	0.003011471
FOXP3	1.381684748	9.37E-09	6.08E-08
RN7SL404P	1.297469036	0.022367989	0.031901925
HMGB1P11	1.479889394	2.62E-08	1.51E-07
AC127526.1	2.092411531	2.93E-08	1.67E-07
AC089983.1	1.263075798	1.36E-06	5.12E-06
AC243773.2	1.682670889	0.000269715	0.000590447
AC011491.2	1.082355412	0.001278972	0.002403496
IGLV1-62	-1.976059748	0.001438839	0.002672258
PTPRJ-AS1	1.430984205	2.57E-06	9.08E-06
LINC02257	3.178785438	3.69E-09	2.64E-08
AC025871.1	1.660309375	0.000105774	0.00025399
H3P28	1.106065304	0.007290221	0.011588668
TTC36	-2.429305795	1.76E-08	1.06E-07
AL590383.1	1.057798407	0.008610517	0.013468055
CYP4F22	-3.010171392	0.000331888	0.000711356
RNU6-1278P	1.837196151	0.000164502	0.000378337
AC124067.2	3.355806696	4.65E-09	3.23E-08
NUDT19P5	1.872490958	0.000132087	0.000310212
HENMT1	1.711930213	6.26E-12	9.56E-11
AC103769.1	1.020703988	0.008456149	0.01325069
TNFRSF10A	1.276877514	6.40E-12	9.75E-11
RN7SL398P	1.018072591	0.016423742	0.024112048
HMGN1P15	1.014093578	0.000550036	0.001122744
AP001207.2	1.730210733	4.64E-06	1.54E-05
PRKN	-1.62601018	1.47E-10	1.55E-09
AL139041.1	1.582522823	2.71E-05	7.51E-05
ANKRD65	-1.017150386	6.34E-10	5.65E-09
NEK3	1.247360365	1.44E-08	8.91E-08
MAD2L1	2.133044283	8.01E-16	4.96E-14
AP001610.2	1.685533003	4.82E-06	1.59E-05
TLE6	1.132970155	7.69E-05	0.000190737
PPIAP70	1.470154309	2.58E-08	1.50E-07
CTHRC1	3.796555931	2.03E-18	1.46E-15
AP002784.1	5.523109229	3.83E-10	3.60E-09
AC023908.3	1.319161727	1.55E-07	7.35E-07
MND1	1.809385301	1.00E-13	2.57E-12
SLC8A2	-1.6765861	0.000604998	0.001221749
RNU2-7P	1.106549894	0.000174128	0.000398344
RNU4-53P	2.222613397	1.62E-05	4.74E-05

RNVU1-30	1.003444982	0.000380057	0.000804568
POU5F1P3	1.013275624	4.74E-05	0.000123579
ZBTB7C	-1.496885546	1.37E-06	5.18E-06
RN7SL246P	1.475685855	9.12E-07	3.59E-06
ARID3C	1.474398573	4.87E-05	0.000126671
AC073333.1	1.900649312	1.62E-14	5.54E-13
PTK7	1.812896887	1.03E-09	8.57E-09
AC025062.1	1.171006219	2.04E-06	7.38E-06
AC103987.1	1.900583181	2.74E-05	7.58E-05
AL662797.1	1.628154478	1.24E-13	3.10E-12
RNU2-68P	1.353458756	1.71E-05	4.99E-05
SNORD104	1.465067128	8.86E-08	4.48E-07
RN7SL745P	1.590419383	0.00042487	0.0008894
TMEM63C	-1.015869482	1.15E-08	7.26E-08
MTND3P10	2.970215209	5.73E-11	6.72E-10
AC015908.2	-1.541646599	4.55E-06	1.51E-05
FDPSP7	1.966408665	3.84E-10	3.61E-09
TREM1	1.699442965	2.53E-05	7.06E-05
PCDHB2	1.211566072	0.033565867	0.045854878
AL031009.1	1.951031351	1.03E-13	2.64E-12
AC007182.2	1.016549358	9.69E-07	3.79E-06
AC105052.2	1.437236902	9.50E-09	6.15E-08
AL139317.1	1.599380843	4.15E-05	0.000109837
JPH4	-1.095544868	1.75E-05	5.07E-05
RIBC2	1.742319367	4.84E-07	2.05E-06
AC027544.1	1.196646215	2.34E-05	6.59E-05
LINC01094	1.656757043	7.04E-11	8.04E-10
CDRT15P1	1.058624855	7.54E-05	0.00018756
SLC12A7	1.450286228	6.79E-13	1.35E-11
AC074135.1	1.566310912	0.029064171	0.040298082
NR6A1	1.518994278	1.34E-09	1.08E-08
AC069499.2	2.366108299	4.19E-08	2.30E-07
LINC01176	1.483924944	3.63E-10	3.43E-09
LRRC6	1.226694029	0.005210185	0.008547924
RCN1	1.365571221	3.77E-14	1.12E-12
AC011632.1	4.297061894	0.000135302	0.000316927
LINC00501	2.606250373	2.14E-09	1.64E-08
AC010285.1	1.627263753	3.32E-06	1.14E-05
AC084824.5	1.016883681	3.57E-06	1.22E-05
NAA15	1.011022816	3.61E-16	2.61E-14
AP000867.2	1.644986846	4.30E-05	0.000113177

SLAMF9	1.690136852	1.87E-05	5.40E-05
LINC01602	4.482009844	0.001608413	0.002954653
AP000525.1	3.495821311	1.59E-10	1.66E-09
AC026464.5	2.156945723	2.31E-08	1.35E-07
DCBLD1	1.355309116	5.12E-15	2.13E-13
AC010761.4	1.212996871	3.50E-06	1.19E-05
ARHGEF38	2.285485647	4.97E-14	1.41E-12
NAP1L2	-1.887878572	4.35E-11	5.24E-10
UCN2	2.517585237	2.07E-10	2.09E-09
AC068580.2	4.975503834	2.77E-11	3.55E-10
MIR6750	1.476010298	0.000487321	0.001007388
LAPTM4B	1.115551918	3.03E-08	1.72E-07
ERCC6L	2.056681596	4.41E-15	1.90E-13
HSP90AA4P	1.517438966	1.48E-10	1.56E-09
PDCD5P2	1.823291451	1.40E-08	8.69E-08
RNU4-4P	2.986324189	2.23E-07	1.02E-06
AL591212.2	1.375369074	0.001589301	0.002922769
MYH16	1.747495481	1.04E-05	3.18E-05
RNA5SP53	1.424041939	0.00180511	0.003277343
C2orf72	1.124156992	2.13E-05	6.05E-05
RNF32	1.525979603	2.64E-11	3.39E-10
AC007906.2	1.375653166	0.006018415	0.00973662
RPSAP31	1.205356712	7.47E-07	3.01E-06
MAGI2	-1.166320463	0.000739321	0.00146686
AL035420.2	1.787751692	7.20E-05	0.000179903
AC026191.1	1.429911254	4.32E-08	2.36E-07
AC020661.3	1.576759747	1.09E-07	5.38E-07
LSM3P5	1.53249325	1.24E-05	3.73E-05
AL356653.1	1.791284236	9.24E-13	1.77E-11
DCLK3	2.657162608	1.28E-16	1.28E-14
MPP1	-1.219677692	0.004201438	0.007039476
SAGE1	6.829131348	3.14E-05	8.56E-05
WARS1	1.708668626	5.44E-08	2.89E-07
ZMYM4-AS1	1.47845024	2.31E-05	6.51E-05
AC092757.3	1.329783722	4.09E-06	1.37E-05
RNA5SP317	1.338976012	3.17E-06	1.10E-05
AL391825.1	1.47851541	4.35E-11	5.24E-10
METTL24	-2.193579374	1.88E-07	8.76E-07
HELZ2	1.373367095	9.13E-11	1.01E-09
FGD6	1.068403746	1.08E-08	6.85E-08
AC008555.6	1.549977731	6.36E-16	4.09E-14

AL158063.1	1.598038375	1.87E-05	5.38E-05
AL137798.1	1.111925939	0.005898863	0.009564695
AC025280.1	-2.106621618	0.000483068	0.000999527
AC025161.1	1.508877916	9.98E-08	4.99E-07
AC105250.1	1.114266702	2.58E-09	1.92E-08
AC090018.1	1.471465956	0.000303665	0.00065609
FCGBP	-1.036936124	0.007834857	0.012367357
SERPINE1	2.399299923	2.67E-11	3.42E-10
ACTR5	1.123066295	3.51E-16	2.57E-14
AL109811.1	1.518547373	2.09E-09	1.60E-08
ANKRD63	-3.052287213	0.022289126	0.031797959
AL121782.1	2.438182884	3.16E-10	3.05E-09
CABP7	-1.28496839	0.012275834	0.018546715
AC021851.1	1.658901404	1.94E-10	1.97E-09
AC108063.1	1.518426851	1.78E-05	5.16E-05
RNY4P25	1.285279496	7.45E-05	0.000185446
AC122108.2	2.573174013	1.89E-06	6.88E-06
IGHV3-47	-3.609804166	0.006344096	0.010215739
PTTG1	1.339004017	1.24E-09	1.01E-08
KRT18P53	1.425321745	0.003483499	0.005942334
SINHCAFP1	1.109720814	1.02E-06	3.98E-06
RNU4-25P	2.546906934	6.51E-07	2.66E-06
HOXC10	6.98186728	1.34E-14	4.70E-13
LINC01593	3.214630754	1.07E-05	3.26E-05
AP000873.1	1.169628836	2.46E-08	1.43E-07
XPO5	1.580707079	1.13E-17	3.15E-15
AL121574.1	1.057067826	0.004742291	0.00785185
KLHL31	1.429148308	3.25E-08	1.84E-07
SERINC5	1.222059288	1.20E-12	2.23E-11
RFC5	1.04866086	6.02E-15	2.42E-13
RPL23AP8	1.0795662	6.73E-05	0.00016933
AC007216.3	1.211903274	3.98E-07	1.72E-06
MIR4653	1.490539848	3.51E-07	1.54E-06
AC090970.2	1.221503765	0.000123264	0.000291321
IGKV2D-18	-2.844824807	0.007714104	0.012202052
AC073896.3	1.099849091	8.29E-10	7.11E-09
CCNP	2.957404658	1.01E-09	8.43E-09
ITGBL1	1.427950991	0.005932791	0.00961035
PRMT5-AS1	1.275249399	4.12E-11	5.01E-10
PDE7A	1.099424555	5.37E-12	8.36E-11
AC007272.1	1.741271378	1.32E-07	6.36E-07

NEIL3	1.750227813	2.18E-12	3.75E-11
AL121949.2	3.713594057	0.000228978	0.000509346
MTCO2P27	1.505889047	0.001879024	0.003397649
HSPE1P28	1.200069493	0.000106717	0.000256072
AC253536.6	1.202510665	2.74E-06	9.59E-06
MYB-AS1	4.028498694	3.61E-10	3.42E-09
MAGEA3	9.039227002	4.17E-07	1.79E-06
AC020891.2	2.341868637	5.09E-07	2.14E-06
AP001086.1	1.093205354	8.95E-07	3.54E-06
BUB1B	2.314175925	1.91E-16	1.67E-14
SPRN	1.09351279	2.66E-13	6.01E-12
ACACB	-1.319733131	1.24E-07	6.03E-07
RN7SL769P	1.114346872	0.004566907	0.007585981
AL022393.1	1.27486097	0.006065966	0.009806399
AC079336.1	1.127310303	0.000208549	0.000467612
ABALON	1.669303598	3.10E-11	3.89E-10
RN7SL268P	1.04254741	2.80E-05	7.74E-05
AC018607.1	1.952720654	3.28E-10	3.14E-09
LRRC3	-1.471243815	5.17E-05	0.000133586
PCOLCE2	-2.386685081	2.95E-09	2.17E-08
IGHG4	1.562922384	0.00029958	0.000648158
JAM2	-1.578945479	4.08E-09	2.89E-08
AC011448.1	1.228052836	1.82E-07	8.49E-07
SUGCT-AS1	3.454371418	1.20E-15	6.67E-14
AC099811.4	1.180148008	0.000285247	0.000620322
DUSP5	-1.555895068	7.15E-06	2.26E-05
RNU1-72P	3.387680117	1.20E-06	4.61E-06
CGAS	1.799217362	9.14E-17	1.04E-14
SLC4A8	1.073698332	0.003978656	0.006705851
GATA3	1.127573741	4.03E-05	0.000106983
SEPTIN14P12	2.7825934	2.38E-10	2.37E-09
MIR1205	1.881440321	0.00143228	0.002661004
RHOT1P2	-3.017551226	0.011624597	0.017661963
CCN4	1.121034154	5.25E-08	2.80E-07
HTRA4	3.212381213	5.59E-15	2.28E-13
ANKRD37	-1.01325179	0.000203773	0.000458097
PSMA1P1	1.212819668	9.95E-07	3.88E-06
KRT17P3	1.228510638	0.000152896	0.000354134
AL137247.1	1.222896455	1.02E-05	3.13E-05
TREM2	3.061726227	1.55E-15	8.16E-14
TMED2-DT	1.323603234	7.36E-13	1.44E-11

RPS2P6	1.12981204	1.57E-06	5.85E-06
RN7SL798P	1.358482968	1.05E-05	3.19E-05
SLC2A6	1.011766151	3.85E-06	1.30E-05
TAAR3P	1.13801849	0.001292472	0.002425103
RACGAP1	1.419141689	3.77E-14	1.12E-12
OLFM4	1.8319706	0.011281375	0.017190487
HHIPL2	2.623950632	2.46E-08	1.43E-07
DZIP1L	1.223311515	0.014312632	0.021302634
FOXD2	2.380817163	1.46E-14	5.07E-13
AC099850.3	1.980206148	1.12E-15	6.29E-14
AC025423.1	2.029059253	5.38E-09	3.67E-08
AC112777.1	2.120363566	6.47E-14	1.76E-12
AC002480.2	1.419355794	0.001749991	0.003189878
SIX1	2.63937892	9.12E-09	5.94E-08
AL139035.1	1.096202627	1.28E-05	3.82E-05
'27P1-BPTFP1-]	1.004713058	1.86E-08	1.12E-07
SNORD116-4	-1.324779438	9.23E-05	0.000224915
F12	1.414046305	3.57E-06	1.22E-05
TRPM5	1.968956965	0.000206857	0.000464249
LINC01081	-1.46182191	4.41E-08	2.40E-07
ZNF267	1.291508477	5.05E-16	3.46E-14
VXN	-1.571958767	0.001150328	0.002187287
ZHX1-C8orf76	1.633313008	9.70E-16	5.73E-14
LYN	1.015131043	7.91E-08	4.05E-07
AL645608.1	1.866359607	3.40E-07	1.49E-06
LARP1P1	1.564691966	1.09E-05	3.32E-05
EME1	2.544799362	1.33E-17	3.38E-15
MIR4677	2.210974941	3.10E-07	1.37E-06
CCL20	2.245081711	6.27E-05	0.000158675
AL035458.2	2.041676253	3.56E-12	5.81E-11
UBE2V2P3	1.432042155	3.94E-07	1.70E-06
LIMS2	-1.955126201	7.09E-07	2.87E-06
AC107926.1	1.777693967	0.003967644	0.006690743
SLC22A17	-1.674036954	1.09E-08	6.91E-08
AC104461.1	1.836527577	8.53E-05	0.000209331
RPL7P24	1.157613011	1.32E-07	6.36E-07
BPIFA1	6.700236748	0.001237133	0.002334917
AL139289.1	1.162729257	1.51E-06	5.63E-06
AC104088.1	1.765020465	4.84E-05	0.000125758
AL356432.3	1.397820735	0.000221277	0.000493534
CDC25A	1.862999492	2.69E-13	6.06E-12

DRAM1	1.078713984	3.17E-08	1.79E-07
PF4V1	2.175312296	2.84E-05	7.83E-05
DNA2	2.276257581	3.07E-17	5.49E-15
PPP1R14A	-1.809766433	2.70E-08	1.55E-07
CHML	1.68919888	3.80E-16	2.72E-14
CSF2	4.688076547	3.84E-13	8.21E-12
AL592295.1	1.072129597	6.54E-10	5.79E-09
SNX8	1.06986191	2.13E-13	4.93E-12
THBS4-AS1	1.670772302	1.76E-06	6.45E-06
AC211476.2	1.467233961	5.94E-09	4.02E-08
MIR4648	1.313423562	0.000558351	0.001137626
CYP2D7	1.023444077	1.02E-06	3.98E-06
LAMB1	1.119369354	1.86E-10	1.91E-09
ROS1	4.133536077	4.76E-08	2.58E-07
COX5BP3	1.18704215	0.000347301	0.000741349
TRMT6	1.422817972	1.15E-17	3.15E-15
AC104794.4	Inf	4.00E-11	4.89E-10
HOOK1	1.101201012	4.06E-08	2.24E-07
RNU4-38P	3.319575273	5.04E-09	3.47E-08
LINC00205	1.103083255	5.41E-10	4.91E-09
MIR576	1.096348022	0.004197199	0.007035913
EVX1-AS	5.100424591	4.21E-06	1.41E-05
ZDHHC9	1.219732721	3.48E-13	7.56E-12
MIR3685	1.368407084	5.25E-08	2.80E-07
MMP11	4.568485374	2.50E-18	1.59E-15
BYSL	1.434755381	1.45E-16	1.40E-14
RNU6-418P	1.34815417	1.55E-06	5.76E-06
NXPH3	-1.528137495	1.95E-06	7.07E-06
AC104297.1	1.090311423	7.61E-08	3.91E-07
AL356608.2	2.688263724	4.43E-05	0.000116198
AC069271.1	1.172203896	1.48E-05	4.37E-05
SCGB3A2	1.592465235	0.033814562	0.046161486
IGFL3	3.722521704	1.96E-06	7.10E-06
AC022973.2	1.008608364	7.95E-05	0.000196366
MPZ	-1.046514207	1.87E-11	2.50E-10
AIRE	2.445756275	0.003595441	0.006116451
AL360169.1	1.732495403	0.000469357	0.00097403
KRT18P65	1.668666765	1.64E-08	9.98E-08
PTMAP4	1.263598008	3.16E-07	1.39E-06
UTP15	1.035475626	7.30E-17	8.88E-15
LURAP1L-AS1	2.036511312	1.13E-08	7.17E-08

AL645939.2	1.179501009	0.000169442	0.000388457
HSPE1P3	1.268236726	2.73E-09	2.02E-08
CABCOCO1	-1.555591903	4.68E-05	0.000122048
COL1A1	3.289901209	2.61E-17	5.12E-15
MTCO3P43	2.854091361	1.66E-13	3.96E-12
SLBP-DT	1.146427811	9.46E-09	6.13E-08
AC245052.2	1.71967828	0.000153166	0.000354716
AL356215.1	2.317466407	2.65E-05	7.37E-05
MIR3129	1.891403079	0.001237301	0.002335069
TTPAL	1.023172778	1.69E-13	4.02E-12
SLC2A4	-2.380775119	1.94E-10	1.97E-09
PLS3-AS1	1.293268259	1.05E-06	4.09E-06
AP001107.6	1.336260588	2.12E-06	7.62E-06
HK3	1.550050404	2.56E-07	1.16E-06
GAPDHP46	1.362941959	1.69E-05	4.92E-05
AC103725.1	1.430723355	0.005623811	0.009159407
AC003002.2	1.28093523	0.001751313	0.00319207
RNU6-1201P	1.485394016	8.47E-05	0.000208149
XRCC2	2.579211029	7.32E-18	2.50E-15
BNIP3P10	1.325415876	3.23E-05	8.79E-05
AC025766.1	1.243346994	4.02E-05	0.000106617
IGF2BP1	6.609908648	3.78E-09	2.69E-08
FDPSP3	1.423087463	1.51E-05	4.45E-05
SNORA5C	1.571607967	4.14E-12	6.65E-11
WDR90	1.4503835	1.30E-13	3.21E-12
LAMA1	1.053131191	0.009767369	0.015097601
DNAJC3-DT	1.000439833	9.53E-05	0.000231435
AC007619.2	1.546107104	2.30E-06	8.20E-06
IGHV3-36	-3.123095277	0.009055124	0.014103866
AC090907.1	1.477931389	0.00014033	0.000327638
MTERF3	1.130912164	4.04E-15	1.77E-13
MSR1	2.292961314	1.42E-12	2.59E-11
AC010768.2	1.161493085	2.27E-05	6.42E-05
MTND4P13	1.870852911	0.007187621	0.01144059
AL021026.1	-2.350212819	4.38E-06	1.46E-05
TEX11	2.069127252	0.01933795	0.027939353
RPS15AP10	1.224697208	2.53E-08	1.47E-07
MORN3	1.647934058	0.000818723	0.001608704
RNU6-237P	1.5779402	5.96E-05	0.000151686
AC114402.1	1.17257996	0.000106986	0.00025667
CLK2P1	1.316736686	3.92E-08	2.17E-07

SSBP2	-1.378268033	8.87E-09	5.78E-08
EYA4	1.255197065	0.032790252	0.044891989
HAVCR1	4.76471738	8.28E-08	4.22E-07
AL158071.2	1.235922701	0.000108799	0.000260473
AL391840.2	1.033616791	0.001092501	0.002089927
CEP85	1.216592603	2.36E-12	4.03E-11
RN7SL663P	1.493084238	1.84E-06	6.72E-06
MIR3646	1.664209599	1.30E-06	4.92E-06
IGKV6D-41	-2.85608969	0.001603975	0.00294711
LSM8	1.056843561	1.19E-13	2.97E-12
AL512306.3	1.326631448	0.00582312	0.009453964
AP001442.1	1.166991848	4.32E-07	1.85E-06
MIR597	1.170137924	0.024801956	0.034983785
XPNPEP2	-2.996697327	0.000138206	0.000323046
ELOCP2	1.077021237	6.98E-07	2.83E-06
PRPS1P2	1.061324842	2.87E-08	1.64E-07
RAD51	1.842119695	5.81E-16	3.83E-14
SLIT2	-1.120472149	0.001462607	0.002711864
LYG2	1.584509705	8.03E-06	2.51E-05
APOL4	1.304565857	5.08E-07	2.14E-06
AC093732.1	2.415948827	1.53E-10	1.61E-09
NANP	1.143694718	2.03E-14	6.70E-13
BCHE	-2.18244995	1.19E-09	9.75E-09
AC244034.2	1.541216254	5.02E-06	1.65E-05
FOCAD-AS1	1.134462705	0.004979161	0.008206806
PDCL3P5	1.190987717	2.51E-09	1.88E-08
TP53I11	1.239923215	1.38E-10	1.47E-09
HTR2C	6.998950034	2.37E-07	1.08E-06
ATP6V1G2	-1.665752175	0.000147226	0.000342299
RNY4P7	1.077582742	0.000901039	0.001756584
FGF3	6.631580031	2.60E-05	7.24E-05
CASC19	2.74138977	4.23E-09	2.97E-08
IGLVI-70	-1.663090541	0.021639773	0.030962773
AL353804.2	1.386053808	6.50E-06	2.07E-05
PTPRG	1.015738676	8.31E-09	5.45E-08
GPIHBP1	-1.079422589	9.82E-09	6.33E-08
AC099518.4	1.754845981	1.58E-11	2.15E-10
CCL3	2.103501668	1.09E-09	9.01E-09
AC245100.3	6.290166587	4.14E-05	0.000109604
AC130324.2	2.289205196	1.40E-09	1.12E-08
NCBP2-AS1	1.129842757	1.11E-06	4.28E-06

MIR3619	1.250296606	0.002693559	0.004705383
P4HA1	1.437034274	6.70E-16	4.27E-14
Z99916.1	1.156409842	0.001308876	0.002452945
GSTCD-AS1	1.295153364	4.10E-07	1.76E-06
AC120114.1	1.058172852	1.75E-06	6.42E-06
AC018552.3	1.767462236	2.79E-06	9.75E-06
ADCY5	-1.806722549	3.29E-06	1.13E-05
RN7SL146P	1.661775235	6.64E-06	2.11E-05
IGHA2	-2.773504664	0.003223263	0.005543488
SRGAP1	1.013463116	3.45E-10	3.29E-09
CYCSP27	1.669124975	0.00011216	0.000267397
FKBP1AP1	1.082341206	6.38E-07	2.62E-06
FBXL19-AS1	1.278086028	3.60E-12	5.86E-11
DRGX	3.806116865	4.75E-05	0.000123888
AC138207.6	1.329847951	6.98E-07	2.83E-06
IGKV2OR22-4	-3.292901968	0.00346878	0.005920637
LINC00634	1.58751368	1.76E-06	6.46E-06
AC007204.1	1.604495547	7.12E-06	2.25E-05
AC097641.2	1.232349319	1.94E-09	1.50E-08
ATP4A	-4.69394627	4.05E-06	1.36E-05
ATP13A3	1.474750427	1.39E-17	3.39E-15
LINC00299	2.179834689	2.36E-11	3.08E-10
AC026471.3	1.981283495	5.63E-08	2.98E-07
C1QTNF1-AS1	1.986846042	5.92E-05	0.000150795
STEAP2-AS1	1.651558653	2.52E-05	7.05E-05
AC092168.2	1.29278059	5.89E-07	2.44E-06
Z98200.1	1.570797225	1.15E-07	5.66E-07
CCDC78	1.995182526	6.54E-11	7.55E-10
AC090559.2	1.18809935	0.000258942	0.000568717
AP002812.5	1.087954548	5.89E-05	0.000149972
SUMO4	1.000104358	3.57E-07	1.56E-06
RASA4CP	-1.127496858	6.34E-11	7.34E-10
CUZD1	1.676485334	1.32E-06	5.00E-06
AC253536.5	-1.234285528	0.032850539	0.044972204
CASTOR1	-1.26954997	0.001783641	0.003243227
NUP62CL	2.217966329	1.10E-11	1.57E-10
PBX1	-1.004568962	7.75E-06	2.43E-05
RALGAPA2	1.036917896	5.16E-09	3.54E-08
POMK	1.349363649	3.18E-10	3.07E-09
SCML1	1.658245118	3.69E-13	7.94E-12
SAP25	1.420082778	9.31E-07	3.66E-06

RN7SL364P	1.049713869	0.000169058	0.000387649
CABP4	1.511052762	0.00333931	0.005726435
AL121839.2	1.570193539	2.91E-10	2.84E-09
RPS8P4	1.15377903	3.18E-05	8.64E-05
RN7SL788P	1.144144447	0.031025209	0.042708154
PMS2P12	1.226100906	0.010306254	0.015857881
AC106028.2	1.949318823	8.51E-10	7.29E-09
DTYMK	1.046440455	1.56E-10	1.63E-09
RGS17P1	1.060472056	2.39E-06	8.48E-06
RPL6P24	1.375370848	4.53E-06	1.51E-05
MIR5000	1.141837275	0.022699433	0.032305464
AC064836.1	1.085958341	0.001343539	0.002511722
IGLV2-5	-3.651984288	0.017313713	0.025307069
AC022973.4	1.897495514	3.59E-09	2.58E-08
CR769775.1	1.165416237	0.007044478	0.011234226
SNRPCP13	2.063908485	1.05E-06	4.08E-06
DRICH1	1.965815548	3.23E-09	2.35E-08
GYPC	-1.258832082	1.06E-08	6.75E-08
EEF1B2P4	1.039470308	0.008732235	0.013645622
AC138956.2	1.018907246	5.98E-07	2.47E-06
NIFK	1.001942884	1.90E-15	9.60E-14
CD276	1.310772544	8.20E-15	3.10E-13
MIR6772	1.487424403	0.000213861	0.000478675
RPS24P14	2.189760732	4.91E-05	0.000127497
AC084757.1	1.57170476	4.52E-08	2.46E-07
AL031587.5	-1.261988622	4.27E-09	3.00E-08
WNT8B	2.418170652	1.30E-09	1.05E-08
RAB3B	1.406621767	0.010452931	0.016048349
COX5BP4	2.277702161	4.82E-06	1.59E-05
C2CD4D-AS1	1.538162965	0.000979715	0.001896778
DOCK3	-1.628995284	0.023925632	0.033867844
AC008395.1	1.242435358	0.000220027	0.000491075
ABCE1	1.289343976	5.75E-17	7.74E-15
AC009533.1	1.30032064	8.76E-11	9.76E-10
HOGA1	1.298683881	0.006032607	0.009758392
MIR378G	2.554048448	3.62E-06	1.23E-05
MCTP2	1.067129603	3.89E-08	2.15E-07
HAGLROS	1.578243386	1.66E-05	4.85E-05
FAM3B	-1.514629087	1.50E-05	4.44E-05
RNU6-88P	1.105366006	0.000228582	0.000508507
VSIG2	-2.224201168	0.001137851	0.002165794

MYEOV	1.213083905	0.00029958	0.000648158
AC003070.1	1.032876815	7.69E-06	2.42E-05
ADAMDEC1	1.019476164	5.45E-05	0.000140169
AC053481.3	2.303454328	0.001463483	0.0027133
PPIAP34	1.606678213	4.34E-10	4.04E-09
AC009407.1	1.430144458	3.47E-05	9.36E-05
MESTP1	1.847177451	2.17E-09	1.65E-08
DHX37	1.152923302	1.35E-15	7.33E-14
PPP1R3C	-2.445623763	5.05E-10	4.62E-09
MIR770	-1.486329763	0.035040797	0.047662067
AL158825.2	1.627977788	4.67E-07	1.98E-06
CYS1	-1.187332546	0.00042121	0.000882014
RAPSN	1.140039335	0.009287835	0.014433182
ZNF711	1.360660224	1.66E-09	1.30E-08
CETP	1.126351992	5.58E-10	5.04E-09
OTC	-2.412763823	0.002323157	0.004121017
RNF2P1	2.422532184	2.85E-06	9.95E-06
RPL35AP	1.691872801	0.001884803	0.003406943
AL110115.1	1.207473851	6.39E-06	2.04E-05
CADM3-AS1	-1.094873476	1.33E-07	6.43E-07
KRT87P	2.530965048	3.57E-09	2.56E-08
KCNK3	-1.413435474	1.02E-09	8.50E-09
HSPH1	1.622163561	1.83E-16	1.62E-14
RN7SL165P	1.213353083	0.003656515	0.006213206
LRRC39	1.361178599	2.68E-09	1.99E-08
ASPA	-1.975136855	2.92E-15	1.36E-13
AC010368.1	1.840461188	0.000536299	0.001097479
INAFM2	-2.07009001	0.015808287	0.023299645
MTX1P1	1.27472701	2.11E-12	3.65E-11
AL138767.3	1.560557801	0.000194593	0.000439666
RNA5SP483	1.492888123	0.003315938	0.005691485
NDUFAF2P2	1.567263911	3.15E-05	8.57E-05
HMGB2P1	2.05064469	3.70E-09	2.64E-08
SNORA80C	2.633212001	7.37E-06	2.32E-05
SNORA46	1.423241127	0.000235208	0.000521678
CLDN4	1.377384718	4.23E-09	2.97E-08
SATB2	1.871170229	9.23E-11	1.02E-09
KNTC1	1.894583466	5.03E-17	7.25E-15
NDUFA3P6	1.385982164	0.002419873	0.004274365
ERVH48-1	1.24377466	0.002444251	0.004312795
ZNF761	1.677982986	1.13E-16	1.21E-14

HSPE1	1.393374114	1.08E-14	3.92E-13
'NFRSF10A-AS	1.267986593	1.80E-10	1.85E-09
HES6	1.440150932	5.05E-06	1.66E-05
AL118558.2	1.808578169	0.000143613	0.000334424
EIF1P4	1.825706171	0.000166602	0.00038277
FAM72B	2.374043158	1.93E-15	9.68E-14
AC011239.2	-2.364964014	0.000368235	0.000781963
PKDCC	1.478940139	5.25E-08	2.80E-07
AC110588.1	2.107324136	3.12E-07	1.38E-06
AC007950.1	2.708584078	4.14E-05	0.000109646
XRCC3	1.070877061	8.41E-14	2.20E-12
DIXDC1	-1.325343224	6.92E-05	0.00017356
CEACAM16	2.167715118	0.000328736	0.000705566
AK5	-1.198167658	0.000985189	0.001905576
AC100757.2	-1.780285373	0.021143444	0.03032427
AP005271.1	2.507134329	0.015300655	0.022632838
GSTM5	-2.376984826	4.97E-14	1.41E-12
CLCN5	1.535921155	2.11E-13	4.89E-12
RNA5SP38	5.75251356	3.22E-06	1.11E-05
SNORD72	2.243390291	1.07E-08	6.81E-08
SGCA	-2.349078579	9.72E-11	1.07E-09
EFS	-1.721463795	6.25E-08	3.28E-07
FEZF1-AS1	5.012598647	3.82E-15	1.69E-13
RNU6-1011P	1.620396199	1.30E-07	6.31E-07
SHOC1	1.129855504	0.000814112	0.001600471
AL158068.1	1.306120679	2.60E-05	7.24E-05
HRCT1	-1.21621368	0.001755377	0.003198166
DEFA5	-5.299361472	0.001615837	0.002966655
AC107294.3	1.205226175	0.001589248	0.002922769
AC022784.1	3.787078718	2.15E-08	1.27E-07
SNRPCP19	2.386078937	4.01E-06	1.35E-05
CH25H	-1.003352526	0.000103683	0.000249352
KRT9	2.550729513	0.00026545	0.000581637
P2RY6	1.664789213	2.33E-10	2.33E-09
BARD1	1.036014909	4.81E-12	7.57E-11
PGA3	-3.783641187	2.71E-07	1.22E-06
AC099518.1	2.126780233	2.73E-10	2.69E-09
AP3M2	1.209049407	4.20E-15	1.82E-13
ZC3HAV1L	1.212064861	1.56E-10	1.63E-09
SOSTDC1	-2.589423372	7.70E-13	1.50E-11
RNU4-39P	1.238313935	3.10E-05	8.45E-05

SPTBN5	1.452677276	2.29E-06	8.17E-06
CRISPLD1	1.321246158	0.000132204	0.000310296
RNA5SP160	1.174851658	0.000590017	0.001194742
AC092809.1	1.080220456	0.000707067	0.001408109
AURKA	2.190818762	3.35E-15	1.52E-13
ADAMTS8	-1.265590945	0.000139967	0.000326819
SNORA74C-1	1.835962361	8.18E-06	2.56E-05
DCSTAMP	3.996551316	8.10E-16	4.99E-14
CLIP3	-1.585948591	0.000551499	0.001125213
ZNF195	1.463628766	1.08E-16	1.18E-14
AC016292.1	1.763883351	2.22E-05	6.30E-05
CADM3	-2.726768689	4.46E-15	1.91E-13
UAP1L1	1.701715184	5.81E-14	1.61E-12
ANKRD49P2	1.585942138	0.000155625	0.00035991
AC073210.1	1.775468606	1.25E-09	1.02E-08
CXorf38	1.046803379	9.93E-14	2.55E-12
RNU6-640P	2.007224648	0.001822649	0.003305584
MYG1-AS1	1.335198101	2.54E-15	1.22E-13
DPRXP2	1.464212626	0.001091038	0.002087278
AC096631.2	1.091764275	1.21E-05	3.65E-05
AL109618.1	1.22288867	3.12E-10	3.02E-09
PPP1R14BP2	1.676809643	2.15E-09	1.64E-08
RNA5SP47	3.03437842	3.84E-05	0.000102588
ECEL1P2	3.544011734	4.23E-07	1.81E-06
RNVU1-22	2.366354491	1.76E-05	5.11E-05
CYP2D8P	1.394022857	1.02E-08	6.53E-08
RPL39P25	1.222694596	0.004805225	0.007943708
AL445250.1	2.043246769	0.000578854	0.001174459
RNU2-70P	1.292283004	0.002904555	0.005044534
AL645608.6	1.81565962	0.006338688	0.010207648
NAALADL1	-2.609074341	6.94E-06	2.20E-05
C4BPB	1.729163023	1.85E-05	5.33E-05
CYP4B1	-2.379365438	1.37E-10	1.46E-09
BCRP3	1.332466895	5.25E-08	2.80E-07
TCF23	2.456055292	0.000666988	0.001335447
GCSHP2	1.724726607	0.006103512	0.009863506
WDR12	1.086793462	2.57E-15	1.24E-13
AC007277.1	2.513174332	0.007042543	0.011231813
RPL23AP86	1.346161094	1.86E-06	6.78E-06
OR2B6	3.595290178	1.05E-10	1.15E-09
CYP4F24P	-1.08293176	0.011491786	0.017476121

TMEM182	1.06912976	5.73E-12	8.86E-11
MIR1293	1.419113052	1.66E-05	4.84E-05
AC091980.1	1.054646544	0.000422666	0.000884925
JPH1	1.497680094	6.99E-06	2.22E-05
AC092473.1	1.317724493	0.000590081	0.001194782
ITGB6	1.083832839	0.015273156	0.022593416
FAM71E1	1.00842811	0.023828271	0.033749749
OGT	1.057647739	2.04E-10	2.06E-09
PLBD1	1.123192288	2.17E-07	9.94E-07
MSX1	1.364673906	3.75E-07	1.63E-06
AC090617.8	1.263087992	0.001015625	0.001958616
RN7SL75P	1.451555921	2.12E-05	6.05E-05
H2BS1	1.41082581	0.008109672	0.012760328
CCR8	2.74256514	6.26E-13	1.26E-11
AC008569.2	1.673485445	3.04E-09	2.23E-08
AC007220.1	1.159974876	0.000127489	0.000300466
RHOB	-1.579669119	0.00230858	0.00409816
AC019257.2	1.454889557	1.18E-06	4.53E-06
ZNF93	1.119725381	2.48E-05	6.93E-05
GYG2	1.036698648	0.000584398	0.001184264
AC138393.2	1.408634356	1.19E-08	7.51E-08
C1orf112	1.803617961	3.84E-18	1.72E-15
RNU1-124P	1.586660736	0.000118831	0.000281813
MIR6512	1.2007876	0.003049039	0.005271072
TRBV29OR9-2	1.459446487	5.36E-06	1.75E-05
CEP78	1.093108995	3.30E-14	9.97E-13
KLHDC7B-DT	1.35736776	6.04E-08	3.18E-07
SERPINB11	-1.329985876	0.024239569	0.034259448
PI15	1.846859103	0.003919789	0.006617936
DUSP1	-1.518445415	2.90E-09	2.13E-08
AC087521.2	1.237577555	1.46E-07	6.98E-07
LINC00668	1.095491144	0.001575485	0.002899762
RN7SL838P	1.287471392	3.30E-05	8.95E-05
AC010343.3	4.342844064	7.10E-05	0.000177685
AL031848.1	1.099522814	0.000813528	0.001599794
MIR608	2.26767562	1.47E-06	5.51E-06
NCEH1	1.464342879	4.88E-11	5.81E-10
RN7SL612P	1.733565838	3.67E-05	9.83E-05
PPIAP75	1.989969309	2.96E-10	2.89E-09
AC015911.1	1.197786012	2.88E-05	7.93E-05
AC022165.1	1.277917753	2.43E-05	6.83E-05

AC003070.2	1.364458785	0.017508389	0.025569178
FBXL6	1.099184045	2.39E-09	1.80E-08
MELK	2.240467872	4.27E-16	3.02E-14
H2BC14	1.33723128	0.000203337	0.000457272
IGHV1OR16-4	-3.414085748	4.29E-05	0.000112834
NGB	-3.009584923	0.001896353	0.003424567
PDE4C	-1.028208616	5.28E-06	1.72E-05
FGL2	-2.125797077	6.35E-06	2.03E-05
AP000692.2	-1.291353048	2.11E-06	7.59E-06
OLAH	1.771000984	3.41E-05	9.22E-05
CALB1	5.402279353	0.012717274	0.019142018
AC132825.1	1.261539351	0.025325346	0.035635261
TXNIP	-1.137934967	1.09E-06	4.22E-06
PA2G4P2	1.290441236	5.09E-11	6.01E-10
BTF3P6	1.921122379	9.00E-07	3.55E-06
AC124283.3	1.063508281	4.86E-06	1.60E-05
AL513327.2	1.86575843	8.95E-08	4.52E-07
AC117465.1	3.540614209	4.47E-07	1.91E-06
AL592076.1	1.597282124	0.030885317	0.042528769
SLC16A7	-1.080347028	4.69E-07	1.99E-06
CASP8	1.025188777	1.23E-12	2.27E-11
ZDHHC23	1.377326667	9.28E-12	1.35E-10
ZFP36	-1.454624409	6.18E-07	2.54E-06
TNS4	1.866553221	7.64E-05	0.000189687
ATP2C2	1.138448799	0.000875978	0.001711484
MIR6793	1.472369114	0.004222016	0.007069442
LINC01942	1.097031314	0.007437601	0.01180182
AC145285.1	1.062503118	5.79E-07	2.40E-06
PPIAP79	1.555826464	2.70E-06	9.49E-06
RPL22P12	1.617712293	3.92E-12	6.33E-11
FMO1	1.729714587	7.92E-06	2.48E-05
MTATP6P26	2.454956908	1.52E-11	2.08E-10
AC055855.1	1.453408656	1.11E-08	7.04E-08
IGKV2D-26	-2.780136916	0.000258312	0.000567536
AP002993.1	1.061040852	0.000557009	0.001135673
RNU6-647P	1.311841931	0.010951155	0.016738994
AC004080.15	5.29449478	1.75E-10	1.81E-09
SLC9A5	1.521663712	2.76E-13	6.19E-12
WFDC1	-1.068876803	2.37E-06	8.40E-06
AL513123.1	5.480741735	1.71E-10	1.78E-09
TBC1D30	1.713784475	3.40E-13	7.40E-12

AC089999.2	1.016221582	1.55E-05	4.55E-05
AC062037.1	1.552740098	6.10E-05	0.000154997
VMO1	1.838326033	4.09E-06	1.37E-05
ETV4	3.0560783	2.19E-15	1.08E-13
RPL35AP32	1.042189134	2.19E-06	7.85E-06
TNFSF18	1.816965876	9.23E-05	0.00022482
MIR548AQ	2.009904241	0.000594098	0.001201911
RNU6-479P	1.529362215	0.002466129	0.004347423
SMYD3-AS1	2.180719402	2.31E-08	1.35E-07
AC068620.2	1.141200845	2.02E-06	7.29E-06
AC020916.1	1.081597086	3.93E-07	1.70E-06
MIR6740	2.104795049	9.85E-08	4.93E-07
AC016747.2	1.34751051	0.000321022	0.000690232
PAEP	4.964423022	6.30E-09	4.24E-08
PLA2G7	2.389921634	4.65E-13	9.66E-12
AC007151.1	1.635614777	1.75E-05	5.08E-05
RPL7P20	1.056005344	1.31E-05	3.92E-05
AC005281.1	-1.019089182	6.41E-06	2.05E-05
FAM27E3	1.278974565	2.86E-07	1.28E-06
RNASEH1P1	4.065751613	0.000234276	0.000519916
WT1	3.167971747	2.85E-07	1.27E-06
PLPPR4	1.779721806	4.46E-07	1.90E-06
PPIAP26	1.474200278	1.52E-05	4.48E-05
GDF15	2.960186821	5.12E-15	2.13E-13
KCNE3	1.432327815	2.09E-06	7.54E-06
RPS20P4	1.00266974	8.80E-07	3.49E-06
PRELID1P1	1.088928923	8.26E-08	4.21E-07
DPY19L1P2	1.36779543	1.81E-08	1.09E-07
UBE2R2-AS1	1.099145313	1.72E-05	5.01E-05
OSM	1.981886502	1.53E-06	5.70E-06
RNU6-988P	1.51159849	0.000686948	0.001371835
AP001330.3	1.222599209	0.000106541	0.000255671
TMEM97	1.021797268	9.84E-07	3.85E-06
AL954705.1	1.086140035	7.62E-07	3.06E-06
RNU5E-10P	1.223912936	0.00496597	0.008186077
F2R	1.849471614	2.39E-15	1.16E-13
AC015967.2	1.03508734	0.000697986	0.001391916
AC105129.1	1.145102201	9.31E-05	0.000226629
IGKV2D-24	-2.573925615	0.018249861	0.026555945
KMT5C	1.110691255	7.63E-12	1.14E-10
TOMM40P4	1.430579121	2.36E-15	1.15E-13

RAB39A	1.309503057	0.001962189	0.003532445
AL359183.1	1.012461248	2.57E-06	9.08E-06
KRT20	-2.033030618	0.002090026	0.003742888
AGPAT5	1.149680678	3.16E-11	3.95E-10
DFFBP1	1.148589822	2.24E-05	6.34E-05
CPN1	5.460805312	2.26E-06	8.08E-06
AC087273.2	3.668668724	1.18E-07	5.79E-07
AC022400.3	1.502908554	5.21E-06	1.70E-05
PGGHG	2.105467711	2.16E-12	3.72E-11
AC128687.1	1.953327421	6.04E-06	1.94E-05
LILRB4	1.747716979	7.57E-09	5.02E-08
RPL12P21	1.203672417	7.90E-07	3.16E-06
RPA4	1.170021527	0.000207585	0.000465618
GNPNAT1	1.097066055	2.23E-11	2.93E-10
AC048382.1	1.150279451	3.60E-06	1.22E-05
MICD	1.577963093	0.000128788	0.00030332
KCNJ14	1.676326586	3.73E-13	8.00E-12
AC000067.1	1.87152159	6.32E-07	2.60E-06
ANKRD53	-1.353464268	3.28E-09	2.38E-08
AC006349.1	1.58957161	0.000147779	0.000343493
AKR1C7P	2.046025077	8.10E-09	5.32E-08
LINC02875	1.301599034	0.000182218	0.000414963
LINC01572	1.832684122	2.92E-13	6.51E-12
ZBED6CL	1.232258964	1.47E-10	1.55E-09
AC013355.1	1.422521504	3.24E-06	1.12E-05
AL365436.3	1.447831883	8.93E-05	0.000218403
SYNM	-2.350952041	6.75E-07	2.75E-06
COL4A6	-2.794040594	2.58E-11	3.33E-10
MIR4295	2.383822584	0.00037965	0.000803833
TXNDC12-AS1	1.557814884	3.67E-07	1.60E-06
EPOP	1.747604894	8.31E-09	5.45E-08
AC022080.2	1.174272751	0.002278612	0.004051171
AC011676.1	2.795406279	4.07E-14	1.20E-12
AC079602.1	1.278649661	0.002553137	0.004482417
ZNF124	1.308080895	5.38E-15	2.21E-13
MSTO2P	1.080263195	2.68E-10	2.65E-09
DACT3	-1.698177308	4.72E-07	2.00E-06
DIP2A-IT1	1.126823102	1.30E-05	3.87E-05
AC074117.1	1.463060596	1.16E-16	1.22E-14
BMP1	1.530540561	1.21E-16	1.24E-14
RPL39P14	1.351890365	4.61E-06	1.53E-05

SNORD93	1.033625098	0.011942579	0.018091441
MYOM3	2.973145467	4.40E-09	3.07E-08
BTBD7P1	1.486260764	0.025729308	0.036165465
AC006254.3	1.127680862	0.000139333	0.000325537
VCAN	2.040681614	3.13E-11	3.92E-10
RDH16	1.842175003	3.94E-06	1.33E-05
RGMA	-1.49168442	7.26E-07	2.93E-06
INSYN2B	1.915029367	7.31E-08	3.77E-07
AC004865.1	1.154833733	0.001036662	0.00199399
ANXA2P3	1.171190572	7.80E-06	2.45E-05
AL022345.3	1.517530034	0.00761977	0.012063579
RPL35AP11	1.373581893	7.55E-05	0.000187724
AP003108.5	3.050478223	0.000461121	0.000958657
RPS12P2	1.46668168	1.25E-05	3.74E-05
ESRRG	-2.980857548	1.34E-12	2.46E-11
DBF4P1	1.937922741	5.21E-13	1.07E-11
AC021945.1	2.57047535	1.92E-13	4.50E-12
IGKV1OR2-118	-3.403923674	0.000904214	0.001762387
AC005546.1	1.323121236	2.25E-05	6.36E-05
AC108667.1	2.185920305	1.28E-05	3.84E-05
HAMP	1.98680152	1.34E-08	8.36E-08
P2RY1	-1.503894536	0.006872903	0.01098889
RNU6-704P	1.387872447	0.00040131	0.00084526
HMGN1P2	1.009877051	0.001004355	0.00193955
TNFAIP2	1.50950314	3.92E-08	2.17E-07
GBP5	2.352202596	9.17E-08	4.62E-07
AC010326.3	1.127563363	1.52E-09	1.21E-08
AC006042.1	1.996566284	5.83E-11	6.82E-10
KLF2	-1.452366734	2.55E-06	9.02E-06
MIR1203	2.030539253	0.000640216	0.001286517
AC253536.4	4.065280206	0.000118404	0.000281055
VASH1	1.080581039	2.09E-09	1.60E-08
AL355574.1	1.228550701	3.25E-10	3.12E-09
AC010327.5	1.233174862	1.45E-05	4.28E-05
AL162734.1	1.86558352	2.35E-07	1.07E-06
TUBAP13	3.170640614	0.000103754	0.000249499
GSTA2	-2.881468377	0.0078989	0.012462542
AL117327.1	1.365620929	0.000232923	0.000517171
AF107885.2	1.038306112	0.000241899	0.000534917
MTCO1P28	1.351992597	0.000111872	0.000267021
MAP4K4	1.086336627	1.50E-14	5.18E-13

RNF139-AS1	1.40885831	6.69E-12	1.01E-10
AP001363.2	-1.299046152	3.21E-06	1.11E-05
IFI30	1.328074443	6.04E-10	5.41E-09
FASN	1.000683318	1.06E-07	5.27E-07
ADAMTSL1	-1.651158193	1.17E-11	1.65E-10
DEPDC1	2.051594269	3.82E-14	1.13E-12
CACNB2	-1.696940726	0.003958946	0.006677275
FTLP8	1.169347669	8.12E-05	0.000200231
RNU6-689P	1.012508322	5.20E-05	0.000134349
FGF2	-1.489130262	0.000129708	0.000305136
AC005722.4	-2.055483223	5.08E-05	0.000131393
AC006511.2	2.00167685	5.00E-10	4.58E-09
NTSR1	2.382107937	0.02542799	0.035768348
RN7SL16P	1.142345352	0.030683719	0.042283952
LINC01518	6.032952508	3.41E-06	1.17E-05
RND2	-1.117256628	6.50E-06	2.07E-05
AC010547.2	3.085308219	1.41E-06	5.29E-06
LINC02160	2.711744774	0.00491757	0.008117845
PHBP19	1.588314647	4.74E-09	3.28E-08
AC008750.1	1.934814424	4.82E-09	3.33E-08
RN7SL344P	1.31418345	0.001904836	0.00343942
AP000919.3	1.153590606	7.69E-06	2.42E-05
RPS15AP39	1.787047281	1.01E-07	5.02E-07
SNAP91	-1.49664952	0.000263819	0.000578587
MIR3131	1.559855706	0.003196226	0.00550374
AL139246.2	1.739711272	7.22E-08	3.73E-07
ZSCAN12P1	1.217556903	3.97E-10	3.72E-09
ZDHHC11	1.361551013	0.00032013	0.000688426
AL390037.1	1.148868934	0.001663971	0.003047521
C7	-1.428345214	6.90E-09	4.60E-08
LMO1	-1.84732603	0.02334566	0.033136228
IFNL3P1	1.077328451	0.005429735	0.008874241
ZNF850	1.094594236	0.000514299	0.001057494
AC092112.1	3.471477958	2.53E-07	1.15E-06
AQP10	-5.30816315	1.65E-06	6.10E-06
C6orf15	7.64227421	1.40E-05	4.16E-05
AC017006.2	1.378341669	5.93E-05	0.000150858
AF111168.1	1.391511487	1.19E-06	4.55E-06
MYOSLID	2.739099954	5.30E-09	3.63E-08
AC131212.4	1.225056774	6.80E-05	0.000170911
AC004241.1	-1.017722965	9.64E-06	2.96E-05

AC016924.1	-1.20881406	9.00E-05	0.000219788
LINC02633	2.191907523	0.00010071	0.000243147
RAD17P1	1.029853624	0.00101844	0.001962854
AP000842.3	3.107243808	0.000913939	0.001779259
AL662890.1	2.193183518	9.74E-07	3.81E-06
SCN7A	-2.569506071	3.15E-10	3.04E-09
CACNA1F	1.624793481	4.11E-07	1.77E-06
HEATR1	1.522053354	8.01E-17	9.44E-15
RPL39P18	1.541940834	1.32E-06	5.02E-06
RIPPLY3	2.471854507	0.020367102	0.029296931
FAR2	1.548541653	2.26E-10	2.26E-09
SYCP2L	2.13924796	0.00043744	0.000913344
AC007991.2	4.157907144	1.60E-08	9.73E-08
CYCSP10	1.057411548	7.98E-06	2.50E-05
AC092535.5	2.026952481	1.99E-10	2.01E-09
CEACAM5	1.46991186	0.011180978	0.017052127
ZNF878	1.342626954	6.59E-06	2.10E-05
AC008494.1	1.001092299	5.24E-06	1.71E-05
BUB1	2.389328453	1.13E-16	1.21E-14
PMS2P3	1.045610989	8.06E-13	1.56E-11
SULT1C3	-1.385590261	0.007804289	0.012326409
RPL23AP23	1.389694321	1.99E-05	5.70E-05
AL353764.1	1.034803819	0.005210041	0.008547924
AL353622.1	1.769545198	4.98E-11	5.91E-10
SNRPD1	1.467852417	2.29E-16	1.87E-14
TMEM249	2.914147216	7.18E-12	1.08E-10
AL390726.4	2.075567855	9.83E-06	3.02E-05
MDM2	1.340470428	2.15E-11	2.85E-10
RNU6-450P	1.115884685	0.000775294	0.001531503
TSLP	-1.881405252	1.13E-09	9.33E-09
COL9A1	2.05475222	0.00026054	0.000571913
AC115619.1	-4.489773912	0.000229706	0.000510752
LIX1	-2.257169603	0.003270066	0.005618903
NADK2-AS1	1.016251424	1.21E-05	3.65E-05
TTYH3	1.928150892	2.30E-15	1.13E-13
RAI2	-1.112699304	6.43E-07	2.64E-06
KCNMB2	-1.317904754	4.04E-07	1.74E-06
AC132219.2	1.031582986	1.15E-07	5.64E-07
AC114781.3	2.196633893	9.38E-06	2.89E-05
GKN2	-4.355548968	1.32E-06	5.00E-06
LINC01451	1.244052706	0.001004486	0.001939653

AL024508.1	1.209486096	1.41E-05	4.19E-05
AL590004.3	1.497637329	0.014374951	0.021385856
CSRNP1	-1.639429097	1.23E-07	5.99E-07
AC016065.2	1.127977356	0.000554565	0.001131157
AC073089.1	1.362024453	7.90E-05	0.000195269
CYP2AB1P	-1.48241089	0.00014667	0.000341244
C3orf18	-1.38950614	3.60E-06	1.22E-05
AC067930.2	1.81150366	2.49E-07	1.13E-06
AL807752.1	1.419907471	5.98E-10	5.36E-09
RGPD2	1.659835951	8.31E-06	2.59E-05
AC104982.2	1.73413787	3.53E-08	1.98E-07
AC093323.2	1.031999594	0.007466125	0.011842145
AL117344.1	1.169135058	7.74E-05	0.000191934
AC139792.1	1.398399914	0.000367753	0.000781002
RPL23AP19	1.002334672	0.000884238	0.001726737
AC055876.2	2.402803613	3.37E-09	2.43E-08
AC004080.2	3.064402293	5.32E-11	6.27E-10
TLCD1	1.366958343	3.59E-08	2.01E-07
TPSAB1	-1.154449361	3.94E-06	1.33E-05
AL024507.1	1.89030209	0.001761127	0.003207982
SEMA5B	1.983463936	1.44E-12	2.61E-11
RPL7P56	1.386704557	1.42E-05	4.21E-05
AC131971.1	1.070061535	1.72E-06	6.33E-06
MTURN	-1.068019481	3.47E-06	1.18E-05
AC023043.3	1.672747433	1.43E-11	1.97E-10
SELENOOLP	6.469145069	1.84E-06	6.72E-06
RNU6-920P	1.442116145	0.001161536	0.002206778
RNU6-19P	2.213096116	1.58E-05	4.64E-05
EIF3FP1	1.581810266	0.002944949	0.005107033
AC004253.1	1.288079741	2.44E-07	1.11E-06
E2F3	1.685485303	1.33E-19	1.18E-15
AC055764.1	1.005805869	0.000167764	0.000385209
GREB1	1.272124828	0.001655232	0.003033132
ARSL	1.810735092	5.58E-08	2.96E-07
AC131953.1	1.395229389	1.46E-05	4.31E-05
B3GNT4	1.52871973	2.26E-09	1.71E-08
ASNSP1	5.120374955	1.08E-05	3.29E-05
CR391992.1	1.056616865	0.000381535	0.000806991
OSTCP8	1.92183102	3.38E-09	2.44E-08
NBPF20	1.081999054	3.10E-11	3.89E-10
CHRNA1	3.24972426	3.27E-15	1.49E-13

LIN28B	6.193277571	0.014487063	0.021534582
SOX21-AS1	-2.013135973	9.69E-06	2.98E-05
AC125238.2	1.089014031	1.32E-06	5.00E-06
CLEC3B	-3.060295471	1.19E-16	1.24E-14
RPS2P45	1.072326355	4.74E-05	0.000123573
SPDYA	1.083372881	4.61E-07	1.96E-06
IGHV3OR15-7	-3.561168681	0.00730514	0.011608777
EMILIN2	1.02856577	4.64E-08	2.52E-07
POP1	1.577802248	7.49E-17	9.04E-15
LINC00337	1.724460501	1.89E-07	8.82E-07
SPHK1	1.125066852	1.03E-07	5.15E-07
AC119674.1	1.232672033	5.69E-05	0.00014543
RPS10P9	1.000572101	0.000133893	0.000313902
HNRNPH1P1	1.294042111	7.32E-08	3.78E-07
MIR5581	1.810120814	1.18E-06	4.52E-06
SPDYE1	1.052029996	6.75E-07	2.75E-06
AC068580.4	1.408723248	8.07E-06	2.52E-05
ZNF133-AS1	2.962909623	1.77E-06	6.51E-06
PNCK	-2.777669352	4.04E-07	1.74E-06
F5	2.423271977	6.31E-06	2.02E-05
TOPBP1	1.411135578	9.02E-17	1.03E-14
RNA5SP282	1.521341662	0.000475288	0.000985188
AC055811.3	1.56656153	1.30E-06	4.92E-06
AC092747.2	1.846340035	1.04E-05	3.18E-05
FBXL22	-1.710552425	0.000152902	0.000354134
AL035448.1	1.194163189	2.40E-06	8.52E-06
A2M-AS1	-1.483188238	0.003206988	0.005517636
CDC7	2.048333698	3.96E-17	6.39E-15
AC110285.6	1.090770916	0.004413722	0.007358136
ZBTB46-AS1	4.015831237	0.00032961	0.00070727
PLXDC1	1.006299664	1.20E-07	5.85E-07
MIR5685	1.563037577	0.004523007	0.007517575
TGIF2	1.286777386	2.53E-14	8.00E-13
RNA5SP305	1.699167041	0.000222162	0.000495276
RNU1-134P	1.02308378	0.001654995	0.003033132
UCA1	3.88323025	9.77E-07	3.82E-06
AL713922.2	1.561812924	9.75E-06	2.99E-05
AC011773.1	2.044204603	7.21E-10	6.32E-09
MT-TA	-1.304863005	3.52E-05	9.47E-05
GPR141	1.146149353	0.000561183	0.001142259
HOXD10	3.985727892	0.001644451	0.003015034

GJA3	1.73965444	3.96E-06	1.33E-05
MIR548K	1.63007585	0.004039158	0.006799648
DPPA3	3.445767871	0.023976145	0.033935742
AC022509.3	1.148173251	0.01184802	0.017963518
PROC	1.975327479	5.49E-05	0.000140903
AC010320.3	1.444383396	1.88E-06	6.83E-06
LINC00858	4.733049396	4.55E-11	5.47E-10
MIR4713HG	2.134432321	8.82E-08	4.46E-07
LINC01980	5.057569894	2.40E-07	1.09E-06
LIFR	-1.025650958	6.09E-08	3.20E-07
RNA5SP207	1.354712898	0.000173541	0.000397104
UPK3A	3.997321134	0.000753451	0.001492005
LINC02266	3.049625943	3.83E-06	1.29E-05
MIR6797	2.317223544	2.99E-09	2.20E-08
AC011944.1	1.489604697	1.27E-05	3.81E-05
Z82243.1	1.447935293	1.71E-10	1.78E-09
HNRNPMP1	1.474180547	2.60E-05	7.23E-05
KRT18P5	1.356878004	1.37E-10	1.46E-09
AC023830.1	1.287640287	2.04E-07	9.43E-07
U2SURP	1.084244343	1.35E-15	7.33E-14
RAB42	1.133928226	5.43E-07	2.27E-06
PSMC1P5	1.084288769	0.00025074	0.000552357
RPSAP8	1.249754804	8.82E-05	0.000215862
GAPDHP43	1.298319688	1.51E-08	9.28E-08
UNC93B2	1.621777765	0.000113874	0.000271096
AL135937.1	1.08864924	0.001000199	0.001932926
FSIP2-AS2	2.299269679	0.000207045	0.00046463
AC005632.4	1.449403231	0.000206386	0.000463307
DNASE1L3	-1.946551131	7.51E-10	6.52E-09
PADI2	1.639716569	9.11E-05	0.000222309
GPX8	1.289572809	2.95E-08	1.68E-07
FCGR3A	2.513647467	1.91E-12	3.33E-11
ZNF137P	1.24813444	6.40E-06	2.04E-05
AC013356.2	1.060925307	2.40E-05	6.74E-05
RNU4ATAC16F	1.027990763	0.005209242	0.008547924
C4BPA	5.217518212	2.32E-11	3.03E-10
AC090519.1	1.135722341	7.87E-05	0.000194741
RNU6-1321P	1.179293849	0.001474138	0.002730964
RXFP4	1.127651424	0.000325847	0.00070017
AC087222.1	1.467159158	6.64E-08	3.47E-07
EIF1AXP2	1.66736035	0.000908719	0.001769872

NASP	1.127577774	2.44E-16	1.95E-14
ADRB2	-1.860139611	4.14E-15	1.81E-13
LDHAL6B	1.431838849	5.02E-08	2.70E-07
AC005387.1	1.144359245	1.54E-06	5.75E-06
RNU6-1209P	1.838486585	9.26E-07	3.64E-06
AC004232.2	1.17974083	2.62E-08	1.52E-07
PODXL	1.611503495	1.05E-15	6.02E-14
AC026704.1	1.39192286	1.75E-08	1.06E-07
ABCA9	-1.388726554	0.000283693	0.000617042
AC108451.2	2.483601083	0.002998189	0.005190911
Z93930.1	1.44604013	1.55E-05	4.55E-05
CFAP65	1.614886445	1.76E-05	5.10E-05
SRD5A3-AS1	1.022640251	2.29E-06	8.17E-06
KCNJ11	1.078981206	0.000175523	0.000401122
AC134879.2	-3.513649891	0.010850536	0.016596623
AC011676.5	1.753045935	2.52E-09	1.89E-08
MUC20	1.180425154	0.011331878	0.017258554
TRIM17	1.450957402	7.44E-07	2.99E-06
SVIL	-1.503040195	0.032918452	0.045046659
YWHABP2	1.014800657	6.53E-08	3.41E-07
MIR4777	1.076967314	0.003787376	0.006413887
RNU6-268P	1.26559778	5.56E-05	0.000142615
RPSAP52	3.670388543	2.57E-18	1.59E-15
AL357055.1	1.133486496	2.04E-07	9.40E-07
Z99129.1	1.04143686	0.00379131	0.006419733
AC013731.1	1.832341809	1.79E-09	1.40E-08
LPAR3	-1.577002981	9.66E-05	0.00023417
LIN9	1.317702271	8.20E-15	3.10E-13
AL031651.1	1.617708278	3.76E-05	0.000100589
AURKAP2	2.071403239	2.71E-06	9.50E-06
AC091046.1	1.087751607	0.00010543	0.00025328
AL031673.1	1.229254698	4.83E-06	1.59E-05
PRSS41	2.737777552	0.000562389	0.001144539
H3P12	1.162659892	3.96E-05	0.00010536
SCDP1	1.312484095	7.97E-05	0.000196971
CAPN9	-1.988309403	0.012033531	0.018215757
AC027801.5	2.101361062	5.29E-08	2.82E-07
AP000974.1	1.359155424	1.23E-07	6.01E-07
AL121748.1	1.27057877	5.27E-06	1.72E-05
CFB	1.06363506	0.003206988	0.005517636
AC106820.3	1.507973202	1.86E-09	1.45E-08

PEX1	1.14421747	4.75E-10	4.38E-09
FGFR4	2.295927369	1.31E-12	2.42E-11
C19orf84	3.79056634	1.85E-11	2.48E-10
EFCAB5	1.421890122	4.67E-07	1.98E-06
ENPP7P11	2.111277535	2.64E-05	7.35E-05
AL365436.1	1.116765882	2.72E-08	1.56E-07
AC011298.1	3.239720044	0.000230656	0.000512607
HMGN2P6	1.249818741	3.03E-08	1.72E-07
AL357500.2	1.114842773	1.33E-05	3.97E-05
AC012073.1	2.114955846	2.80E-17	5.24E-15
AL360169.2	1.371276041	9.40E-05	0.000228486
MAMDC2	-2.852795713	1.09E-14	3.94E-13
AC005180.1	-1.410163201	1.66E-07	7.85E-07
AKAP12	-1.083154031	0.009767369	0.015097601
SUB1P4	1.347305338	1.14E-07	5.60E-07
AP000845.1	1.582245471	2.18E-11	2.87E-10
AC026356.1	1.60947018	3.55E-14	1.06E-12
AC068491.2	3.197762713	3.17E-14	9.66E-13
AC008507.2	2.022960558	8.94E-07	3.53E-06
ASGR1	1.876309192	9.91E-05	0.000239537
MPHOSPH9	1.097032719	1.30E-13	3.21E-12
AP001574.1	1.953438278	4.44E-05	0.000116566
MIR3127	1.416786561	5.62E-05	0.000143934
HCN3	1.052972076	5.11E-08	2.74E-07
ARHGEF2-AS2	1.160928506	4.52E-08	2.46E-07
CACNG8	2.646713863	2.92E-09	2.15E-08
AL161431.1	4.483899483	5.83E-10	5.25E-09
RNVU1-32	2.41234101	1.55E-12	2.79E-11
AC124312.2	-1.167209409	0.000709896	0.001413531
CEP131	1.147535623	1.16E-12	2.16E-11
CNTFR	-1.357913438	1.21E-10	1.30E-09
LINC02829	-1.045888714	5.90E-07	2.44E-06
RPL30P2	1.329839416	1.71E-06	6.30E-06
EIF2S2P3	1.128156153	4.44E-09	3.09E-08
POLR2KP1	1.895812716	2.87E-07	1.28E-06
AC096741.1	1.516326196	9.38E-05	0.00022799
AL160175.1	3.948136077	2.69E-08	1.55E-07
AL589655.1	1.153336191	0.027686365	0.038580477
C3AR1	1.056681921	0.00046833	0.000971975
LUM	1.047550724	0.000306906	0.000662393
NPL	1.072144091	2.90E-09	2.13E-08

MICB	1.642175465	2.79E-10	2.74E-09
MTCO1P11	1.168565353	1.55E-05	4.55E-05
CADM2	-2.5959036	2.63E-11	3.38E-10
AC024561.1	1.020681782	0.005100073	0.008384312
SNORD56B	1.343778192	0.00881225	0.013756135
AC008810.1	1.546250383	9.30E-06	2.87E-05
MIR454	1.281925864	6.24E-05	0.000157987
UBE2S	1.470747446	3.71E-11	4.56E-10
MSL3P1	1.646952133	1.72E-09	1.35E-08
AL158211.3	1.681880383	0.000111654	0.000266548
AC010531.2	1.076812359	0.002744268	0.004786434
MIR429	1.309914857	7.41E-06	2.33E-05
AC017076.1	2.937416016	7.52E-10	6.53E-09
AL354919.2	2.109162536	5.17E-09	3.55E-08
PARK7P1	1.369263476	9.82E-09	6.33E-08
ALG1L12P	1.170368843	0.005309989	0.008696688
ELAVL4	-1.122450737	0.029022895	0.040243046
UBE2CP2	1.305948349	9.29E-06	2.87E-05
AL031289.1	1.074027862	0.001177109	0.002231907
MIR27A	-1.430818975	4.51E-08	2.45E-07
SOHLH1	5.985083286	0.000173884	0.000397824
RN7SL174P	1.328284579	3.78E-05	0.000101108
KCNK16	-2.020496122	0.000496444	0.001024736
BX284668.2	-1.434567307	7.48E-07	3.01E-06
AC010422.4	1.196225173	3.68E-06	1.25E-05
RN7SL14P	1.303392014	0.004994512	0.00823007
MIR4753	2.33131127	0.001270329	0.002390121
GCG	-1.103113626	6.95E-05	0.000174093
RPS15AP15	1.120084765	0.003251611	0.005589716
AP000753.3	1.502863295	0.000172446	0.00039487
AP000254.1	1.224621523	4.21E-05	0.000111174
AL031429.2	-1.64913532	0.00018556	0.000421602
3L5P-PVRIG2P-	1.361779054	7.04E-11	8.04E-10
CSTA	-4.284105316	0.001755378	0.003198166
DIRC1	3.414444773	3.41E-07	1.50E-06
KIF5A	-1.053929903	0.013523697	0.020221031
AL158801.1	1.295352896	1.02E-05	3.12E-05
PNOC	-1.857782912	2.03E-06	7.32E-06
AC007566.1	1.14877777	0.000333886	0.000715007
EPHB2	2.416685758	1.51E-11	2.07E-10
ANGPTL6	-1.426861318	0.026795389	0.037480062

AL354707.1	1.047004451	0.00216834	0.003870776
SNRPCP4	1.286591114	7.37E-05	0.000183783
HEYL	1.496571082	4.56E-08	2.48E-07
IGLV3-16	-3.244630722	0.022079465	0.031532625
RNU4-51P	1.201870307	0.003018196	0.005223513
AC068658.1	3.391374073	0.00101699	0.001960964
AC104024.2	1.211762934	0.00059394	0.001201682
NAP1L5	-1.321223621	0.000496589	0.001024796
RNU6-313P	1.151237689	0.020018048	0.028835947
WDR3	1.056626611	1.57E-13	3.79E-12
AC108673.2	1.43173098	1.21E-09	9.87E-09
BRI3BP	1.077252519	9.29E-09	6.04E-08
BOLA2P1	1.313612268	0.003156767	0.005441069
NDRG4	-1.597984197	1.08E-06	4.19E-06
PRSS30P	2.340833587	4.61E-10	4.26E-09
AC027288.2	2.215803192	1.54E-07	7.35E-07
ADAMTS6	1.067254375	4.46E-07	1.90E-06
QSOX2	1.525689687	6.48E-17	8.34E-15
NACAD	-1.588508353	1.40E-05	4.16E-05
SMOC2	-1.902993626	0.000557937	0.001136869
AC090527.3	1.299385395	1.77E-05	5.14E-05
AC004921.1	1.36893915	2.28E-05	6.44E-05
AC145285.3	1.079323138	1.81E-05	5.23E-05
AC011476.2	1.726684313	3.05E-08	1.73E-07
EHMT2-AS1	1.18831732	5.58E-06	1.81E-05
AC243967.1	1.906846652	0.00110889	0.00211581
LAPTM4BP1	1.117049359	0.000686396	0.001371025
RN7SL130P	1.353440209	2.65E-06	9.33E-06
ATP2C1	1.027908278	7.32E-16	4.60E-14
IZUMO2	2.76100959	3.00E-07	1.33E-06
FSTL3	1.102328205	0.00017996	0.000410312
SMS	1.001787045	5.45E-15	2.23E-13
AL136988.2	1.333802316	1.12E-06	4.31E-06
AC002059.2	1.499632502	1.21E-09	9.91E-09
ADAMTS14	2.862482553	5.34E-18	2.12E-15
CENPU	1.64244038	3.56E-12	5.81E-11
SNRPEP5	1.958646349	7.81E-07	3.13E-06
AC026401.2	1.651625591	4.61E-07	1.96E-06
RNU6-377P	1.647959513	1.20E-06	4.57E-06
PRIMA1	-2.837126198	7.19E-13	1.41E-11
OLFM2	1.395096201	0.000505371	0.001041066

SPATA12	1.254854548	2.61E-07	1.18E-06
BOLA2P3	2.269910865	9.03E-08	4.55E-07
TMPRSS5	2.574540776	4.75E-06	1.57E-05
PRKX-AS1	1.636671751	6.16E-06	1.98E-05
CENPA	2.042478022	7.81E-15	3.00E-13
IGHV1OR15-3	-1.609049619	0.020478229	0.029432
AC119150.1	3.727374736	2.21E-06	7.91E-06
SUMO2P7	1.416969209	5.04E-06	1.66E-05
H19	2.931664579	0.001169607	0.002219104
PYCR3	1.290310257	4.98E-11	5.91E-10
AC091133.5	4.810118751	6.36E-08	3.33E-07
MXD1	-1.190124887	0.009811795	0.015159234
AC061975.7	1.725764426	5.54E-06	1.80E-05
UNC93B6	1.830826052	3.84E-09	2.73E-08
FANCG	1.047452527	2.94E-11	3.72E-10
AC020910.2	2.147398935	6.72E-07	2.74E-06
AC092666.1	2.808505315	0.001323791	0.002478805
AC073842.2	1.354240759	8.30E-06	2.59E-05
LRRC15	2.328514159	6.21E-06	1.99E-05
PDC	1.317710673	1.69E-05	4.93E-05
IFITM3P3	1.457035461	4.61E-09	3.20E-08
C1GALT1P1	2.389904867	5.66E-09	3.85E-08
AC025188.1	1.777993373	6.62E-07	2.70E-06
VEPH1	1.143920163	0.033305606	0.045513333
RNU7-45P	1.158109587	0.014372992	0.021385856
ATAD3B	1.423248961	2.08E-14	6.83E-13
AL078587.1	2.566588445	1.13E-11	1.61E-10
ASCL5	1.9701265	2.05E-05	5.87E-05
RN7SL125P	1.375662801	2.87E-05	7.89E-05
CA2	-1.463835436	0.008753569	0.013672544
MCCC1-AS1	1.399903203	9.32E-08	4.69E-07
RNU6-314P	1.18260416	0.004208762	0.007050417
BMS1	1.031928482	9.63E-17	1.07E-14
RNU6-583P	2.945911424	2.71E-08	1.56E-07
LINC02613	-1.461617067	3.59E-07	1.57E-06
RN7SL118P	1.17369967	1.21E-05	3.65E-05
RNA5SP227	1.861738872	0.000954687	0.00185263
BST2	1.03896371	4.84E-05	0.000125748
MIR4659A	3.228495186	1.57E-05	4.60E-05
AL627308.1	1.428948689	2.12E-05	6.04E-05
AC005086.5	1.537291929	9.28E-05	0.000226008

IDO1	3.839826962	3.85E-08	2.14E-07
MIR320B2	2.087919774	5.59E-09	3.80E-08
AL049874.2	1.294801211	3.22E-05	8.74E-05
RNU6-483P	1.426349097	0.000574697	0.001167093
KLHL2P1	1.163793363	2.49E-06	8.82E-06
AC004584.1	1.294086147	4.00E-05	0.000106324
HNRNPA1P70	1.701481806	0.000187574	0.000425524
NEB	4.512829359	2.66E-14	8.33E-13
PROCR	1.01169303	0.000144463	0.000336374
IGKV1D-35	-4.405769942	0.000545507	0.001114353
OXTR	1.522828352	8.60E-12	1.27E-10
IFI44	1.268439753	1.42E-06	5.32E-06
TRIP13	2.601114369	1.33E-17	3.38E-15
AL035404.2	1.763617349	0.000234621	0.000520595
RSKR	1.559671895	6.79E-13	1.35E-11
AC004777.1	1.047415402	0.004678	0.007756006
AL356417.2	3.634588112	5.96E-12	9.18E-11
AL139128.1	1.818554091	8.00E-10	6.90E-09
RPL7AP73	1.167433507	5.78E-05	0.000147535
AF186192.3	1.405453805	0.012098702	0.018306089
BVES-AS1	-1.853715551	6.33E-08	3.32E-07
HNF1A-AS1	2.007334141	6.16E-10	5.50E-09
MYO19	1.047062919	2.40E-11	3.11E-10
BDKRB1	-1.322466517	1.57E-05	4.61E-05
PCDH12	1.056563424	4.21E-08	2.31E-07
CCDC148	1.428612652	5.99E-05	0.00015222
NUP155	1.386992799	3.47E-16	2.55E-14
BAMBI	3.18601849	4.16E-09	2.93E-08
RNU6-197P	1.210833843	0.001279206	0.002403767
HSBP1P1	1.340351637	0.006239794	0.010065428
NARS2	1.167347847	1.73E-14	5.87E-13
FAM169A	1.254109171	0.001393026	0.002595677
AC087721.1	2.26430656	4.49E-09	3.13E-08
AC092757.1	1.631031184	1.71E-05	4.99E-05
INTS8	1.260824154	9.02E-17	1.03E-14
AC116562.2	2.463173467	0.000100573	0.000242837
AC023449.1	1.54419402	2.93E-05	8.05E-05
TMEM220	-1.04284356	2.73E-09	2.02E-08
AC099509.1	1.201339559	0.013350009	0.019996118
STAG3	1.074798076	0.0002451	0.000540963
AC009120.1	1.146391119	1.66E-06	6.12E-06

KLK15	1.757695471	0.016606347	0.024358657
AKR1C4	1.720796467	0.004703178	0.007793872
THNSL1	1.243798391	3.05E-12	5.03E-11
RNU4ATAC12F	1.620556775	2.64E-07	1.19E-06
AL117350.1	1.097384265	2.89E-05	7.95E-05
AL356320.2	1.73761928	4.99E-09	3.44E-08
AC009084.2	1.20948495	0.000100391	0.000242429
BX470102.1	1.857518683	4.66E-10	4.30E-09
AC108860.1	1.621887086	1.01E-06	3.95E-06
E2F6P4	4.315615206	6.79E-05	0.000170694
AL031577.1	1.064056621	8.76E-05	0.000214562
AC124069.1	1.668390909	0.000186103	0.000422654
OTX1	2.628833056	4.49E-16	3.15E-14
RIF1	1.160660914	4.92E-16	3.39E-14
PIM1	-1.161137944	1.43E-07	6.87E-07
PTMAP5	1.082505738	1.75E-08	1.06E-07
ST7-OT4	1.380390779	6.28E-07	2.58E-06
AC127164.1	1.017477539	6.66E-05	0.00016754
AC012317.1	1.295751383	0.000672146	0.001344292
PAQR4	1.769443599	9.03E-14	2.34E-12
CPP	1.886180438	0.013852083	0.020676071
