# Supplementary material for: An immune-related prognostic signature associated with immune landscape and therapeutic responses in gastric cancer
Source: Aging (Albany NY). 2023 Feb 22;15(4):1074–106. doi: 10.18632/aging.204534 (PMC10008502; doi:10.18632/aging.204534)
Supplement: Supplementary Table 8 [file aging-15-204534-s007.pdf]

Supplementary Table 8. The immune-related gene list in the InnateDB database.

Gene
Id2
Iltifb
PRKRA
DDX3X
DDX58
MIR136
Mir146
Nod2
Il1a
IFIH1
EFTUD2
Tlr2
Ap3b1
Tlr9
Ifnb1
Atf3
Nlrp3
IRF3
IL29
TRAF6
MAVS
IKBKB
RELA
Tbk1
Ppp4c
Myd88
Tlr7
Tslp
Chuk
IFIT5
Stat2
Stat1
Adar
Ticam1
CAMP
Casp2
Pcbp2
Ube2i
Baiap2l1

CASP1
IL1B
TLR4
PELI3
Atf7
MIR223
COLEC12
TLR3
CLEC4E
Traf3
Usp25
Irf1
Cxcl10
Ccl5
ITGB1
IFNG
DDX60L
HDAC2
NFKBIZ
IL6
TET2
March5
Bnip3l
Bnip3
Isg15
Cdkn1a
Nfia
JMJD6
PRMT1
RORC
IL23A
IL2
IL7R
Lcn2
Ireb2
Aco1
TMEM173
Nrip1
Bcl11b
Map3k8
Egfr

Areg
Il33
Card9
Nlrc5
C2
C4B
Mir485
PLA2G2A
IL22
Cd47
SKP2
IFI27
Clec4n
Pik3cd
IFIT3
MIR146A
HACE1
Retnla
Ifngr1
Cxcl13
Mrc1
NFKBIA
TNFAIP3
Il2rb
Ezh2
Irf8
Sppl3
Ahr
Ido1
Lgals3
Trpm5
Il25
Mb21d1
TLR5
CXCR1
Stat6
Dhcr7
Sc4mol
Idi1
Srebf2
Mir342

Tgfb1
Sod1
LGALS1
Aim2
Irf7
Cxcr3
Cd48
Tnfsf4
Clec4d
Mtap1s
Il18
Nlrp6
Atg5
IL10
STAT3
MIR29A
Trim12c
MX1
Lyn
Dhx15
Il17a
Ern1
C5
CEACAM8
C5AR1
Zbp1
Hs2st1
Zfp36
Dusp1
Cxcr4
Nfkb2
Ybx1
SYK
Il7
AIP
Ager
S100a9
S100a8
Lcp2
Ticam2
Tirap

Il17rd
DHX33
RNASEL
IL32
ELAVL1
CYLD
MIR362
Uvrag
Mir125a
Ptpn6
CTSK
CTSS
Mir328
Il4
Nlrp12
DEFB103B
DEFB103A
DEFB4B
DEFB4
Mapk14
Treml4
SPHK1
Peli1
FCN3
Icosl
Smpdl3b
Ctnnb1
Gsk3a
Gsk3b
TLR8
UBE2V2
UBE2N
UBE2W
TRIM5
OAS3
OAS1
Pycard
Psen2
Stmn1
MAP3K7
IRAK1

TIFA
Rora
IFRD1
PDE12
PQBP1
TNF
RNASE7
IFI16
MAPK8
Pten
Sqstm1
TREM1
PGLYRP1
Il17f
Cxcl1
Defb1
Dnase2a
FPR2
FPR1
PPARG
PPARGC1A
MIR130A
Tollip
Pik3c3
Cav1
Mir199a-1
Akt1
DR1
CASP4
PTX3
S100A12
CXCL14
Myo18a
Rac1
Cd81
CAPRIN1
G3BP1
ULK1
Nfil3
Anpep
WFDC12

Ctnnd1
Ly6g
Il1rl1
HAVCR2
HIF1A
Mapk3
Mapk1
Csf1r
Traf2
Cfp
Hc
Wdfy1
Ear11
Tnfrsf13c
Osm
Cxcl5
Atm
ZBTB16
IL1RAPL1
IL18R1
IL1F7
Sf3a1
CLEC6A
Cd200r1
Cd200
ANO6
P2RX7
Zfp423
Rel
Irf5
SLC22A3
TPP2
EIF2AK2
RPS6KA5
IFITM3
INSIG1
AMFR
Eif4ebp2
Eif4ebp1
Anxa1
Sirpa

Itgam
Clec7a
Ptpn11
Irak4
Cd40
Cd86
Icam1
Tmem126a
TP53
Atg7
Arl5b
MIR548G
IL22RA2
Cxcl2
Cebpa
TLR6
Cxcr6
Cxcl16
DEFA6
DEFA5
Il1r2
Il1r1
Adam17
MIR23A
Il15
Il28ra
Nlrc4
Nod1
SMAD4
SMAD3
MIR181A2
Tyrobp
Relb
Nfkb1
Ripk3
ATF2
ADCY8
SCN5A
ECSIT
Slc11a1
USP2

Prl
Myc
ARG1
Pik3cg
Rab8a
PRKX
PRKACA
S1pr1
CCNA2
MIR124-1
PIK3CA
MIR203
SAMHD1
Cxcr2
Adrbk1
MIR122
hsa-mir-146a
RIPK2
IRF4
Il20rb
Il20ra
Il19
Ptges
MFF
DNM1L
BST2
Mir302b
MAPK9
MAP2K7
Il5
Il13
Kdm4a
Vegfc
Flt4
Nlrp1a
Hdac1
Daxx
Il12b
Prdm1
MYH9
HNRNPL

IFIT1
BTK
TRIM14
Mir149
Sykb
TXNIP
Krt16
Cd5l
MIR208B
MIR499A
IL28B
Fscn1
AICDA
PARD3
CD1D
Cd36
MSR1
HMGB1
BGN
HSPA1A
CMA1
C3
C3ar1
Rheb
Tsc1
Mtor
Kdr
Mir126
Stat4
ARF6
Dusp16
CTSL1
GNB2L1
ELF4
Dhx58
JAK1
TNK1
Reg3g
Nlrx1
SLX4
Bcl2l1

Bcl2
Il17c
Il27
Gata3
EGLN2
HIF1AN
Ifi202b
Il9
CRKL
Numb
XRCC5
XRCC6
PRKDC
Mfn2
F2RL1
PRTN3
Marco
CEBPB
CREB1
Nos2
IL28A
Nfe2l2
Keap1
TRIM25
RNF135
IKBKG
Traf5
Mertk
Axl
Tyro3
LGALS9
HDAC11
MIR145
PARK2
POLR2F
CXCL12
MIR141
PIAS3
MIR21
UCP2
MIR133A1

IL12A
RNF125
Zbtb20
ASCC3
MASP2
MASP1
WNT9B
WNT2B
VPS45
Sarm1
ANKRD17
UCHL1
Klf4
Coch
Cdc42
SRC
CD14
MIR15B
Lgr4
TNFSF10
Ppp1cc
Ppp1ca
Psmb8
MIR517C
MIR517A
Ifne
Csf2
Il21
IKBKE
Mfge8
MIR3148
Plunc
Serpinb2
SFTPA1B;SFTPA1
TRIM63
TRIM61
TRIM60
TRIM55
TRIM49
TRIM45
TRIM42

TRIM38
TRIM37
TRIM36
TRIM27
TRIM24
TRIM23
TRIM6
MID2
TRIM67
TRIM66
TRIM65
TRIM56
TRIM50
TRIM13
TRIM9
TRIM8
TRIM71
TRIM58
TRIM47
TRIM32
TRIM26
TRIM21
MID1
TRIM15
TRIM7
MUL1
Gabarap
MIRLET7B
Mir135b
Tlr1
LEP
Scaf11
Naip5
Fer
Rad23a
Lum
Tacr1
Nr1h4
Siglecg
CNOT8
Chat

COX5B
Abl1
Prkcd
Tbx21
Mir212
Mir132
hsa-mir-132
Cryab
Drd2
Tph1
ITGB3
ITGAV
Fcnb
Fcna
Glrx
Hspa1b
Mir497
MOV10
Daglb
MIR187
TRIM62
Mir466l
AI607873
Pydc3
Pyhin1
BC094916
Gm4955
TRIM28
CALCOCO2
MAP1LC3C
Foxo3
VTRNA2-1
Adipoq
Tnfaip8l2
Abca1
Mir467b
Trp73
TP73
MIR10B
Mcpt4
Tlr13

Nlrc3
MIR378
Cftr
Mmp9
Ahsg
Olfm4
Itgb2
Itgax
Ms4a8a
MS4A8B
Casp7
MIR1275
MIR200C
MIRLET7C
Pura
Ace2
Eif4e
Edil3
NLRP4
Zc3h12a
P2ry14
Dusp10
E2f1
Rb1
Angpt1
Dok3
Dicer1
Tufm
mmu-mir-29a
Gnai2
Spink5
Lst1
Scarb1
Spag11a
Tsc22d3
Hamp
Rhbdf2
Plscr1
Ly96
LILRA2
Clec9a

Pacsin1
Irak3
Eps8
Atf4
Nfat5
Ehmt2
Sftpa1
1700021K19Rik
KIAA0226
Tmed7
TMED7;TICAM2
Il1rap
Sdc4
Cd209a
CD209
Ntn1
Nampt
Jak3
Sharpin
Camkk2
WNT3A
Was
MIR16-2
MIR16-1
Frem1
Usp4
DCD
Fstl1
DEFA1
Trib2
Apoa1
Jak2
Hsp90b1
Trpm2
Itch
Il17re
Lrrk2
Hp
Unc5cl
Plcg2
Cnot4

Unc93b1
Serpinb9
Igf1
Hsf1
Pros1
Gas6
Cd300lf
Cd300a
Rictor
Ccl17
Ifit2
Yy1
mmu-mir-10a
MIR10A
MRGPRX2
Rgs2
Nox1
Ppp3r1
Pcbp1
Trp53
Fancc
APOBEC3B
Pglyrp3
Cdkn2a
Dcn
MIR125B2
MIR125B1
Dlk1
Tnip1
Rac2
Pklr
Senp2
Fadd
Pla2g4a
Plaur
Gzmm
SELK
Aire
Apoh
Ppargc1b
Mapkapk2

Zfpm2
Zfpm1
Gata6
Gata4
Tyk2
Nr3c1
Stub1
Serpine1
Tank
NAIP
Naip2
Trem2
APOBEC3G
Ddx41
Tax1bp1
Neu1
Aqp3
Elf1
Cd8a
Muc1
Il1rl2
Rpl19
Il4ra
IL4R
Sftpd
mmu-mir-29b-1
Pltp
Ltbr
Rag1
Tnfsf9
Tnfrsf9
Snca
F11
Tnfrsf1a
Treml2
Cbl
Hspd1
Pml
Pin1
Hrg
Notch1

Gbp2
Hspa14
Tgtp1
Igtp
Edn1
Socs1
Nras
Irgm1
Aimp1
MIF
Gm16379
Duox2
Rarres2
Kcnj8
Nfatc4
Nfatc3
Clec1b
Dhx36
Ddx21
Ddx1
Cd97
Adrb2
Jam3
Ubqln1
Pmaip1
Ip6k1
Khsrp
Ifnar1
Tpst1
Plec
VENTX
Ccr3
Vldlr
MIR23B
Cflar
Cdk6
MIR107
Gpr77
Lilrb3
Pik3ap1
Avp

Casp8
SREBF1
Zc3hav1
Atg12
MIR373
MIR372
Akna
IRAK2
CCL1
Pglyrp4
Gbp10
Gbp7
Gbp6
Gbp1
Tnfsf11
Arhgap15
Bid
Thbs1
Tecpr1
HMGN2
Map3k5
Grn
Cd46
FCN1
Nr1h3
Nr4a3
H2-Ab1
H2-Aa
Srxn1
Gp2
Hrh4
Plg
Ccl2
Impdh2
C1qc
C1qb
C1qa
Ccbp2
Xiap
Birc3
Birc2

Pglyrp2
Defb3
Raet1c
Raet1a
Crp
Lgmn
Cops5
Xbp1
Mbl2
Ccr6
Trp63
TP63
Cltc
Fgf7
Cebpe
MoleculeID 216094
CEBPD
Coro2a
Hmox1
TBKBP1
SIAH2
CD37
SLC15A4
SYP
MAP2K6
MMP7
MMP12
NLRP2
NLRP9
NLRP11
NLRP13
NLRP8
NLRP5
PSMA7
TNFRSF18
CDK9
PTAFR
C8A
SELE
C4A
Mbl1

DEFA3
Apcs
Cfh
PTPN2
IFNGR2
RCAN1
SMAD7
MALT1
ABCG1
SOCS6
CSF2RB
LGALS2
APOBEC3A
MAP3K7IP1
MLST8
TCEB2
MEFV
PTK2B
AMACR;C1QTNF3
SCARF1
CLEC4C
SIGIRR
HRAS
C19orf29
C9
PIAS4
MAP2K2
SMAD6
STAP2
ARRB2
REST
HSP90AA1
RCOR1
KAT2B
NLRP1
THRB
MTA1
CCR4
IL8
CD180
BCL2A1

TCEB1
VDR
SIAH1
LPCAT2
ACHE
MoleculeID 32847
TRAIP
TRADD
TNIP3
MAP3K12
PTK2
ELMOD2
RNF41
BCAR1
ZMYND11
CD22
CD274
PDCD1LG2
CCR7
SMARCE1
NKIRAS2
SOCS2
BECN1
NUMBL
CCDC88A
RPS19
CAMK2A
IFNA2
IFNA1
MAP3K14
ANXA4
CYBB
BCL3
RIPK1
TRAFD1
ITGA3
IRF2BP1
PIK3CB
LY86
RANBP9
NUP153

OTUD5
LILRA4
PROCR
CASP12
CARD18
SOCS3
CYTIP
MAFB
Sirt1
FLI1
PTCH1
CTLA4
CTNNAL1
IL8RB
AAMP
NFATC2
COPS8
LRRFIP1
VEGFA
NUP214
TNFRSF1B
GRK5
DMBT1
GPSM1
FBXW5
IRAK1BP1
KDM1
RUNX3
GJA1
MAP3K7IP2
MKNK1
MAP3K4
JUN
GNAI3
CD53
SNX27
RUSC1
FCGR2A
PTPRC
hsa-mir-146b
MFN1

SPP1
C4bp
C4BPB
C4BPA
Kitl
KITLG
Pdcd1
Rftn1
Hspbp1
Akap10
Ptges2
Stim1
Orai1
Itpr3
Itpr1
Lrrfip2
Trib3
Chga
Siglech
Siglece
Siglec5
Siglec1
SIGLEC15
SIGLEC11
SIGLEC9
SIGLEC8
SIGLEC7
SIGLEC6
SIGLEC10
Rgmb
4432412L15Rik
Oas2
Oas1h
Oas1g
Oas1f
Oas1e
Oas1d
Oas1c
Oas1b
Oas1a
Ctsb

Lair1
C8B
DEFA4
PIK3R1
NLRP10
CARD16
BCL10
NFKBIE
OTUD7B
CFB
ISG20
IL2RG
MST1R
ADAM10
MAP3K3
NKIRAS1
KLK1
C1S
MAP3K1
PTGS2
KPNA1
C8G
LAT
C1R
LY9
HLA-E
EGR1
IL6ST
IGF1R
ELP2
F2RL2
F2RL3
CD27
HOXA9
GSTP1
EPOR
WDR34
ACAP1
ERAP1
CXCL11
CXCL9

S100a10
Anxa2
CEACAM1
Tlr11
IL13RA1
Sema3a
Plxna4
CASP6
Defa20
PELI2
hsa-mir-126
FFAR2
hsa-mir-98
CISH
hsa-let-7e
HMGB3
HMGB2
SIVA1
SNAP23
IRF2
PLK1
TRAF1
PKN1
FXR1
ERBB2IP
RBCK1
RNF31
PIAS1
WDR62
DAB2IP
YJEFN3
RP5-1000E10.4
Trim30
AZI2
MAP3K7IP3
APOBEC3G;APOBEC3F
hsa-mir-152
hsa-mir-148b
hsa-mir-148a
TRAT1
F2R

FCGR1A
UBD
CD300E
PRKCA
CASP10
IRF6
IRF9
VASP
IFITM1
IFITM2
RAD21
RASGEF1B
NOXA1
TRPV2
CYBA
CALCA
PPP3CA
KIR3DL2
OPTN
LGALS4
LGALS8
IL31
RSAD2
HERC5
FCN2
DUOX1
NOX4
Inpp5d
CNPY3
BIRC5
DHCR24
Gpr33
SFTPA2
MIF;SLC2A11
ILF3
ATG9A
MX2
TPSB2
GAB1
SLAMF1
GOPC

YWHAE
LTB4R
BTN3A3
BTN3A2
BTN3A1
IFI6
PTMA
Rxra
GNB2
SPI1
NLRP7
GLI1
TOMM70A
USP17
Ace
ETS1
RAB11A
FZD1
CTSG
ELANE
TNFRSF13B
DHX9
MAP2K1
DDIT3
ATG16L1
OTUB2
OTUB1
Padi4
PPIA
XDH
CR2
IFNAR2;IL10RB
Foxa2
Defb14
Fcrl5
ING4
Calm1
NXN
SPON2
CTCF
RNF5

PIAS2
TCF4
TNFRSF12A
TLR10
USP7
CARD6
C7
C6
BDKRB2
CTSD
C1QBP
CSK
SCAMP5
PSTPIP1
CTSH
TRIM22
SMARCA4
NLRP14
CCNT1
MAP2K4
MAP2K3
SUGT1
NCKAP1L
CD63
SMARCA2
BMX
SERPING1
RETNLB
NFKBIB
PRKCE
STAT5B
STAT5A
SOCS5
RPS6KA4
SH2D1A
SLAMF8
SLAMF9
SLAMF6
SLAMF7
