# Supplementary material for: An immune-related prognostic signature associated with immune landscape and therapeutic responses in gastric cancer
Source: Aging (Albany NY). 2023 Feb 22;15(4):1074–106. doi: 10.18632/aging.204534 (PMC10008502; doi:10.18632/aging.204534)
Supplement: Supplementary Table 10 [file aging-15-204534-s009.pdf]

Supplementary Table 10. Differentially expressed immune-related genes.

Gene	logFC	pValue	FDR
CTSB	1.214858985	7.38E-12	1.11E-10
FCER1G	1.148739456	6.99E-06	2.22E-05
HLA-DOA	1.057725837	0.012439759	0.01876969
HSPA2	-1.977199635	1.42E-05	4.21E-05
HSPA6	1.84677603	0.000131367	0.00030858
HSP90AA1	1.359119177	2.57E-18	1.59E-15
HSP90AB1	1.256608094	1.48E-18	1.31E-15
ICAM1	1.301246592	1.96E-07	9.08E-07
IFNG	2.121420953	2.84E-07	1.27E-06
KIR2DS4	1.820978688	0.00061954	0.00124845
MICB	1.642175465	2.79E-10	2.74E-09
PSMD3	1.231912345	1.59E-12	2.84E-11
TAP1	1.230887183	2.57E-06	9.08E-06
TAP2	1.203728143	6.48E-11	7.48E-10
SEM1	1.016807816	7.15E-10	6.27E-09
IFI30	1.328074443	6.04E-10	5.41E-09
PROCR	1.01169303	0.000144463	0.00033637
ULBP3	1.500180466	1.27E-06	4.83E-06
ULBP2	1.779910874	4.52E-10	4.19E-09
ULBP1	3.699662389	1.86E-12	3.27E-11
HAMP	1.98680152	1.34E-08	8.36E-08
DEFB4A	-1.170188	0.000318045	0.00068445
CXCL16	1.335591063	3.20E-11	3.99E-10
CXCL8	3.524712247	4.08E-11	4.97E-10
CXCL10	3.21464345	6.28E-10	5.60E-09
CXCL9	3.443237622	4.08E-11	4.97E-10
CXCL5	2.570593174	0.000747777	0.00148198
CXCL11	3.404377295	5.41E-10	4.91E-09
CXCL6	3.03337399	3.59E-09	2.57E-08
CXCL1	2.898342335	5.31E-11	6.25E-10
CXCL12	-1.208568066	1.03E-07	5.15E-07
CXCL3	1.612221343	8.26E-07	3.29E-06
DEFA6	-4.286526663	0.017604702	0.0256859
DEFA5	-5.299361472	0.001615837	0.00296665
LCN1	6.03897712	4.09E-09	2.89E-08
S100A8	-3.126825236	0.001088695	0.00208324
LCN6	-1.752846751	1.73E-11	2.34E-10
S100A12	-1.392638681	3.16E-07	1.39E-06

CCR10	-1.186134117	3.40E-06	1.16E-05
PENK	-2.822265122	0.003344305	0.00573389
MMP12	2.585468903	1.30E-07	6.31E-07
TMSB15A	1.885125889	1.34E-08	8.36E-08
S100B	-2.05708412	1.06E-11	1.51E-10
S100A7	2.220093975	9.20E-05	0.00022429
LCN12	2.619579159	1.88E-08	1.13E-07
S100A5	1.555066087	9.91E-08	4.95E-07
S100A3	1.365145151	1.65E-07	7.81E-07
S100A7A	3.627548951	0.004148378	0.00696283
ZC3HAV1L	1.212064861	1.56E-10	1.63E-09
AZU1	3.959386436	0.00037538	0.00079536
LCN1P1	2.196369964	1.36E-05	4.05E-05
SLC22A17	-1.674036954	1.09E-08	6.91E-08
PF4V1	2.175312296	2.84E-05	7.83E-05
MMP9	1.712969523	7.58E-08	3.90E-07
ANOS1	1.865962787	3.13E-09	2.28E-08
FABP6	1.934107978	4.03E-06	1.35E-05
TLR2	1.452516814	3.85E-10	3.62E-09
PLAU	2.363626031	2.81E-15	1.32E-13
PAEP	4.964423022	6.30E-09	4.24E-08
OBP2A	4.493550112	4.10E-07	1.77E-06
IFNL1	1.902273109	0.000378733	0.00080202
SFTPA2	2.2914539	0.016286746	0.02393334
LBP	4.769498658	7.14E-07	2.89E-06
RBP4	2.39136971	0.036265551	0.04917732
NOX4	1.948395229	4.55E-12	7.21E-11
LTF	-2.51640745	0.019788339	0.02852819
FABP4	-1.673283752	9.38E-12	1.36E-10
R3HDML	3.393804941	1.86E-11	2.49E-10
RBP7	-1.167173237	3.20E-08	1.81E-07
OBP2B	6.959255506	1.83E-06	6.69E-06
RBP2	-5.545127996	0.03240732	0.04442149
CETP	1.126351992	5.58E-10	5.04E-09
BPIFA1	6.700236748	0.001237133	0.00233492
PI15	1.846859103	0.003919789	0.00661794
NOX1	4.729361985	3.05E-12	5.03E-11
PMP2	-2.943554147	4.52E-11	5.44E-10
CTSG	-1.25416628	3.04E-11	3.83E-10
AEN	1.039485707	1.39E-10	1.48E-09
CYBB	1.009772343	0.000496589	0.0010248

BPIFA2	4.426011705	2.55E-06	9.01E-06
TFRC	1.507642442	4.44E-11	5.35E-10
IDO1	3.839826962	3.85E-08	2.14E-07
GDF15	2.960186821	5.12E-15	2.13E-13
ADIPOQ	-1.515685389	6.20E-10	5.54E-09
STAT1	1.624185661	9.58E-14	2.47E-12
IFNL2	4.322276553	1.98E-07	9.19E-07
SEMG1	3.347942108	0.00229697	0.00407945
CCL20	2.245081711	6.27E-05	0.00015868
CHIT1	3.846352941	2.34E-07	1.06E-06
PGC	-2.555190893	0.011717063	0.0177842
VEGFA	1.261171457	7.03E-09	4.68E-08
ISG15	1.298569326	4.09E-06	1.37E-05
F2R	1.849471614	2.39E-15	1.16E-13
IL27	1.658890758	5.37E-07	2.25E-06
MAPT	-1.576412162	0.0001463	0.00034041
CST4	6.989281692	5.50E-17	7.55E-15
CSRP1	-1.694130336	8.00E-07	3.20E-06
JUN	-1.15421683	0.000215427	0.00048149
BST2	1.03896371	4.84E-05	0.00012575
TPM2	-2.007616764	0.000404111	0.00085008
AHNAK	-1.134491359	2.26E-07	1.03E-06
FGF2	-1.489130262	0.000129708	0.00030514
MSR1	2.292961314	1.42E-12	2.59E-11
DLL4	1.260829435	2.45E-11	3.17E-10
SLC11A1	2.007428581	5.55E-12	8.60E-11
SEMG2	4.294093281	2.81E-05	7.74E-05
DES	-1.889552131	6.23E-09	4.19E-08
TNFRSF10A	1.276877514	6.40E-12	9.75E-11
TNFRSF10B	1.705094935	5.57E-18	2.15E-15
TNFSF11	2.724813939	2.50E-14	7.92E-13
KNG1	3.850331067	7.54E-10	6.55E-09
KLRK1	1.084757444	0.005388312	0.00881167
KCNH2	-1.278840828	0.001070773	0.00205338
CLDN4	1.377384718	4.23E-09	2.97E-08
RNASE3	2.675020467	0.001214375	0.00229619
PTX3	-1.243826361	3.24E-07	1.43E-06
MASP1	-1.427424334	6.48E-08	3.39E-07
PROC	1.975327479	5.49E-05	0.0001409
ABCC4	1.115442341	1.75E-06	6.42E-06
PLSCR1	1.034437124	3.26E-12	5.35E-11

PDGFRB	1.581836719	5.96E-11	6.94E-10
PCSK2	-2.125980902	5.92E-13	1.20E-11
AQP9	1.697546224	0.000243604	0.00053833
BIRC5	1.770492121	5.16E-13	1.06E-11
TNFSF4	1.647776669	4.23E-09	2.97E-08
NOS1	-2.566159471	4.35E-07	1.86E-06
CCL15	1.230449431	4.48E-06	1.49E-05
CCL14	-1.250606265	1.42E-07	6.82E-07
CCL18	2.253514582	3.47E-05	9.36E-05
CCL26	2.4714125	0.001664114	0.00304752
CCR8	2.74256514	6.26E-13	1.26E-11
CCL21	-1.313861675	1.02E-08	6.53E-08
CCL7	4.52854145	7.91E-13	1.54E-11
CCL3	2.103501668	1.09E-09	9.01E-09
CCL3L3	1.89904269	1.55E-07	7.38E-07
CCL4L2	1.165679133	1.19E-05	3.58E-05
CCR1	1.047686175	2.85E-05	7.84E-05
TAFA5	1.795222788	1.07E-07	5.31E-07
TAFA4	-3.901732122	1.94E-05	5.58E-05
CRP	5.72484944	0.000115316	0.00027421
CD86	1.046181	8.04E-06	2.51E-05
OLR1	3.398129272	8.87E-16	5.36E-14
RNASE2	2.351035965	3.37E-07	1.48E-06
LYN	1.015131043	7.91E-08	4.05E-07
VAV2	1.336893694	1.12E-12	2.10E-11
RAC3	1.453316634	0.002214194	0.00394507
KRAS	1.297854341	1.04E-06	4.04E-06
FOS	-1.172234022	4.03E-05	0.00010698
CARD11	1.189383678	3.45E-05	9.30E-05
NFKBIE	1.036069911	2.70E-08	1.55E-07
PRKCB	-1.045428683	0.000406513	0.00085467
IFITM1	1.468197784	3.29E-07	1.45E-06
IGHA1	-3.063695171	0.003476586	0.00593168
IGHA2	-2.773504664	0.003223263	0.00554349
IGHD2-15	-1.992123509	0.023869949	0.03380698
IGHD3-16	-1.945750851	0.017862635	0.02603799
IGHG4	1.562922384	0.00029958	0.00064816
IGHJ1	-1.84373826	0.028978747	0.04020684
IGHV3-16	-3.301380776	0.004709755	0.00780234
IGHV3-35	-3.560077283	0.010996626	0.01679886
IGHV3-38	-3.131533097	0.018686787	0.02710734

IGHV3-72	-2.103556554	0.018659873	0.0270714
IGKV1-13	-2.340897713	0.015255306	0.02257453
IGKV1-17	-3.255509941	0.027011058	0.03774441
IGKV1-33	-3.461033444	0.004138222	0.00694895
IGKV1-37	-3.557662179	0.000865696	0.00169264
IGKV1-39	-2.810912153	0.012885926	0.01937142
IGKV1-9	-2.392695728	0.033305606	0.04551333
IGKV1D-12	-3.049920311	0.017135801	0.0250704
IGKV1D-17	-2.567640121	0.022292384	0.0318009
IGKV1D-33	-3.441864894	0.00764923	0.01210662
IGKV1D-37	-3.874889816	0.001161536	0.00220678
IGKV1D-39	-3.074528791	0.011154364	0.01701544
IGKV1D-42	-3.811116935	0.005334051	0.00873178
IGKV1D-8	-3.064186905	0.022779046	0.03241115
IGKV2-28	-3.333394914	0.012216494	0.01847278
IGKV2-30	-2.565032982	0.028907887	0.04011897
IGKV2-40	-3.374628045	0.000327301	0.00070288
IGKV2D-24	-2.573925615	0.018249861	0.02655595
IGKV2D-28	-3.244381881	0.006836785	0.01093705
IGKV2D-29	-3.277285039	0.029253388	0.04053842
IGKV2D-30	-3.724686794	0.00166388	0.00304752
IGKV3-7	-3.463463343	0.023771428	0.03368356
IGKV3D-11	-2.798975561	0.027667664	0.03855697
IGKV3D-15	-3.38599769	0.016289717	0.02393507
IGKV3D-7	-3.761205919	0.000601123	0.00121529
IGKV6D-41	-2.85608969	0.001603975	0.00294711
IGLC6	-2.49810142	0.01369484	0.02045741
IGLJ1	-2.156510613	0.001275062	0.00239852
IGLV1-36	-1.792206055	0.035570113	0.04833022
IGLV1-50	-3.621034076	0.001304852	0.00244585
IGLV2-18	-2.684344569	0.015504523	0.02291023
IGLV2-33	-3.713235512	0.007227748	0.0114969
IGLV2-8	-2.773580443	0.029022967	0.04024305
IGLV3-10	-2.432837382	0.035711112	0.04848987
IGLV3-16	-3.244630722	0.022079465	0.03153262
IGLV3-22	-1.113862727	0.019507448	0.02816133
IGLV3-32	-2.944648693	0.000308059	0.00066477
IGLV4-3	-4.774302439	0.003343896	0.00573356
IGLV5-37	-2.327139103	0.015833012	0.0233348
IGLV7-43	-3.007907038	0.024071284	0.03404689
IGLV7-46	-3.262236618	0.033630541	0.04593852

IGLV8-61	-2.019500911	0.026155487	0.03669056
C3	1.154099081	0.012886407	0.01937142
CKLF	1.150545653	1.63E-10	1.70E-09
CMA1	-1.955005622	1.26E-12	2.33E-11
CXCL17	-1.626986053	6.88E-05	0.0001725
EDN1	1.170545427	2.53E-05	7.06E-05
EDN2	-1.919148651	6.07E-05	0.00015408
EDN3	-1.977621065	0.001053094	0.00202259
FGF10	-1.065280828	6.11E-05	0.000155
SAA2	3.396887798	0.000737218	0.00146301
SEMA4F	1.048429743	4.57E-10	4.23E-09
SEMA5B	1.983463936	1.44E-12	2.61E-11
SEMA6D	-1.589232328	0.00017996	0.00041031
SLIT2	-1.120472149	0.001462607	0.00271186
TYMP	1.381760406	1.43E-07	6.87E-07
CYSLTR1	-1.062724856	0.000135603	0.0003176
ACKR1	-1.432664596	4.25E-11	5.15E-10
GPR17	-1.440736904	6.57E-05	0.00016558
PTGDR2	-2.192059963	0.000367433	0.00078045
CXCR2	-1.763055352	0.000365247	0.0007763
PLAUR	1.149293268	2.28E-07	1.04E-06
PLXNA1	1.405623753	2.78E-15	1.31E-13
PLXNA3	1.299559066	3.06E-13	6.78E-12
PLXNC1	1.021256004	2.58E-05	7.20E-05
PLXND1	1.099815839	3.43E-08	1.93E-07
AGT	2.348766356	2.01E-06	7.27E-06
AMELX	2.561182771	3.36E-06	1.15E-05
AMH	3.382191282	1.27E-09	1.03E-08
ANGPTL7	-2.454322168	1.97E-06	7.12E-06
APLN	3.118100798	3.86E-14	1.14E-12
ARTN	1.071917329	0.031904827	0.04378102
BDNF	1.164074385	0.001355701	0.00253286
BMP1	1.530540561	1.21E-16	1.24E-14
BMP3	-1.798112221	0.000133751	0.00031373
BMP5	-1.138189879	4.09E-08	2.26E-07
BMP6	-1.398308738	0.000363074	0.00077186
BMP8A	2.273115076	7.90E-15	3.03E-13
BTC	-1.479631879	0.000175523	0.00040112
CCK	-1.04477734	7.68E-05	0.00019068
CD70	1.586857448	0.000278575	0.0006073
CGB5	7.940121123	5.21E-07	2.19E-06

CHGA	-1.343468862	3.34E-07	1.47E-06
CMTM1	1.384307032	2.75E-14	8.61E-13
CSF2	4.688076547	3.84E-13	8.21E-12
DKK1	2.773785869	0.015808124	0.02329964
ESM1	5.063219418	3.05E-20	4.50E-16
FAM3B	-1.514629087	1.50E-05	4.44E-05
FAM3D	-1.246297902	0.000303222	0.00065519
FGF19	8.294004292	8.05E-12	1.20E-10
FGF3	6.631580031	2.60E-05	7.24E-05
VEGFD	-2.671207066	3.42E-16	2.53E-14
GCG	-1.103113626	6.95E-05	0.00017409
GDF10	-1.297842271	1.19E-06	4.55E-06
GDF7	-1.437296943	2.92E-08	1.67E-07
GHRL	-2.54541629	0.008321168	0.01306225
GIP	-5.272939042	0.000193373	0.00043738
GKN1	-4.413963865	5.78E-07	2.40E-06
GNRH2	1.24414437	0.000479019	0.000992
IL11	3.60095304	3.07E-14	9.39E-13
IL17B	-1.124960889	0.000743534	0.00147435
IL17C	3.001440056	3.90E-05	0.00010387
IL37	4.215475971	2.97E-11	3.76E-10
IL23A	1.213146158	1.53E-10	1.61E-09
IL24	1.586752684	4.43E-06	1.47E-05
IFNL3	5.082695399	1.27E-06	4.82E-06
INHBA	3.765640856	2.88E-18	1.63E-15
INHBB	1.840921632	0.000726805	0.00144385
INHBE	1.631792074	3.14E-08	1.78E-07
INSL6	2.152080211	0.013479795	0.02016784
JAG2	1.214331465	2.50E-10	2.48E-09
LEFTY1	5.544755943	0.000199993	0.00045035
LIF	2.635640149	2.23E-16	1.86E-14
MDK	1.304767595	5.43E-07	2.27E-06
MLN	-2.565656234	0.022066041	0.03151683
NMB	1.295725964	1.29E-08	8.08E-08
NPPA	1.001581633	0.002088706	0.0037415
NPPC	-2.737151863	4.20E-10	3.93E-09
NPY	-1.739959688	2.63E-07	1.19E-06
NRG2	-1.737949346	4.16E-09	2.93E-08
NRG3	-1.294656694	0.000412014	0.00086494
NRG4	-1.264405723	0.000115638	0.0002749
OGN	-1.033643783	3.85E-06	1.30E-05

OSGIN1	-1.452616987	6.08E-07	2.51E-06
OSM	1.981886502	1.53E-06	5.70E-06
ENDOU	-4.504710061	4.18E-06	1.40E-05
PDGFB	1.132810693	2.13E-09	1.63E-08
PGF	1.47010561	6.54E-12	9.93E-11
PMCH	2.617979758	7.40E-10	6.46E-09
PNOC	-1.857782912	2.03E-06	7.32E-06
PTN	-1.925389668	1.24E-11	1.74E-10
RETN	1.389670192	0.000711185	0.00141546
RLN2	1.135840921	0.010475521	0.0160821
SPP1	4.341971278	6.70E-14	1.81E-12
SST	-1.879562346	5.00E-07	2.11E-06
STC1	1.289294134	1.20E-07	5.85E-07
STC2	1.991329991	3.13E-11	3.92E-10
TAC1	-1.890395465	0.000120023	0.00028444
TG	1.120749887	0.008398336	0.01316942
TNFRSF11B	3.395735904	6.21E-11	7.21E-10
TNFSF12	-1.113752377	3.77E-05	0.00010072
TNFSF13B	1.063313559	1.26E-05	3.77E-05
TNFSF15	1.483018055	1.49E-09	1.19E-08
TNFSF18	1.816965876	9.23E-05	0.00022482
TNFSF9	2.157843531	7.98E-06	2.50E-05
TSLP	-1.881405252	1.13E-09	9.33E-09
UCN	1.533597132	3.12E-10	3.02E-09
UCN2	2.517585237	2.07E-10	2.09E-09
UTS2	2.477140311	5.39E-06	1.76E-05
VIP	-2.667953752	2.63E-05	7.31E-05
ADCYAP1R1	-2.862491338	1.30E-09	1.05E-08
ADRB2	-1.860139611	4.14E-15	1.81E-13
AGTR1	-1.72575854	2.11E-07	9.71E-07
ANGPT1	-1.156852993	0.007069613	0.01126554
ANGPTL1	-2.450174425	5.58E-10	5.04E-09
ANGPTL6	-1.426861318	0.026795389	0.03748006
APLNR	1.180649673	3.77E-05	0.00010072
AR	-1.431080587	1.07E-05	3.24E-05
C3AR1	1.056681921	0.00046833	0.00097198
CNTFR	-1.357913438	1.21E-10	1.30E-09
CSF2RA	1.051736569	2.44E-05	6.85E-05
ESRRB	-1.595125762	0.007203237	0.01146046
ESRRG	-2.980857548	1.34E-12	2.46E-11
FGFR4	2.295927369	1.31E-12	2.42E-11

FGFRL1	1.070427521	0.000276888	0.00060402
GHR	-1.808369591	5.04E-11	5.96E-10
GLP2R	-1.556935124	2.17E-06	7.80E-06
GPER1	-2.010034831	2.61E-09	1.94E-08
IL13RA2	2.995558668	2.74E-08	1.57E-07
IL17RB	2.262502094	4.64E-15	1.97E-13
IL2RA	1.414879546	6.71E-08	3.50E-07
LGR5	3.144521251	1.12E-07	5.51E-07
LIFR	-1.025650958	6.09E-08	3.20E-07
MC1R	1.590378176	2.48E-13	5.63E-12
MET	2.491116347	1.68E-15	8.74E-14
MLNR	-2.921876313	0.000440234	0.00091896
NPR1	-1.403978613	4.00E-06	1.35E-05
NPR3	-1.0312183	0.000150039	0.00034814
NR2C1	1.055384929	1.80E-14	6.05E-13
NR4A1	-1.32163122	0.027337972	0.03816686
NR4A3	-2.156697512	0.006776404	0.01084632
NR5A2	1.051476826	1.40E-05	4.16E-05
NR6A1	1.518994278	1.34E-09	1.08E-08
OSMR	1.443990344	6.54E-10	5.79E-09
OXTR	1.522828352	8.60E-12	1.27E-10
PGR	-1.748589002	5.99E-06	1.93E-05
PTGER3	-1.105005683	2.55E-05	7.11E-05
PTH1R	-1.55129281	2.35E-09	1.77E-08
PTH2R	3.316792445	0.005698063	0.00926901
RXRG	-2.881196551	8.09E-14	2.12E-12
SSTR5	2.293632371	0.015558697	0.02298646
TGFBR3	-1.415587686	5.00E-05	0.0001296
TNFRSF10C	1.028446833	0.009122169	0.01419833
TNFRSF12A	1.19581959	2.17E-09	1.65E-08
TNFRSF17	-2.465916136	0.022362529	0.03189585
TNFRSF25	1.616570199	2.14E-10	2.16E-09
TNFRSF9	1.630672776	3.81E-10	3.59E-09
TUBB3	2.338402881	1.54E-11	2.11E-10
VIPR2	-1.531186555	1.68E-07	7.93E-07
FCGR3A	2.513647467	1.91E-12	3.33E-11
PRKCG	3.738441504	1.37E-09	1.10E-08
GZMB	1.220667147	0.013943599	0.02079287
BID	1.265331218	2.29E-13	5.25E-12
CTLA4	1.294041489	5.88E-08	3.10E-07
TRAJ1	1.51526094	0.012314288	0.01860165

TRAJ2	2.101154927	2.98E-05	8.17E-05
TRAJ3	1.250724012	0.009534331	0.01477253
TRAJ5	1.828367634	0.001555043	0.0028665
TRAJ19	1.274452845	0.002231387	0.00397331
TRAJ23	1.751376857	0.001893911	0.00342039
TRAJ31	1.144467943	0.031062304	0.04274597
TRAJ34	1.831773997	0.009437517	0.01463274
TRAJ35	1.507677681	0.007717595	0.01220685
TRAJ36	1.547869411	0.002578933	0.00452264
TRAJ37	2.753064244	3.55E-05	9.54E-05
EFTUD2	1.057826222	5.59E-16	3.73E-14
NLRC5	1.442772179	3.09E-10	2.99E-09
C2	1.747130343	1.46E-09	1.16E-08
C4B	1.269733423	0.00016079	0.0003705
SKP2	1.578690957	7.37E-14	1.96E-12
IFIT3	1.131043219	4.43E-05	0.00011629
CEACAM8	2.148299344	9.82E-07	3.84E-06
SPHK1	1.125066852	1.03E-07	5.15E-07
OAS3	1.551225425	4.25E-11	5.15E-10
IRAK1	1.02027373	9.72E-11	1.07E-09
AIM2	1.184371393	0.001700083	0.00310762
TREM1	1.699442965	2.53E-05	7.06E-05
HAVCR2	1.674958119	1.53E-10	1.61E-09
ZBTB16	-2.45154655	7.73E-11	8.75E-10
CLEC6A	1.442357141	0.000407471	0.00085661
IFITM3	1.032094949	1.13E-07	5.56E-07
MIR181A2	2.572760065	8.20E-06	2.56E-05
USP2	-2.040541769	3.31E-09	2.39E-08
PRKX	1.053507475	2.58E-09	1.92E-08
CCNA2	1.737279007	3.91E-13	8.34E-12
RIPK2	1.65081666	2.84E-19	1.26E-15
TXNIP	-1.137934967	1.09E-06	4.22E-06
MIR499A	1.382944893	0.00439439	0.00733141
BGN	2.909669059	1.16E-16	1.22E-14
TRAF2	1.200728086	6.04E-16	3.92E-14
PRKDC	1.473915509	6.96E-16	4.39E-14
ASCC3	1.0159762	3.30E-12	5.40E-11
TRIM24	1.083776802	1.37E-09	1.10E-08
TRIM50	-2.971246991	2.91E-05	8.00E-05
TRIM71	5.315716791	3.90E-05	0.00010387
VTRNA2-1	-1.445103949	0.001939415	0.00349662

NLRC4	1.01739298	5.44E-08	2.89E-07
CFTR	1.328608391	0.000567726	0.00115417
AHSG	5.065104258	0.000553332	0.00112887
OLFM4	1.8319706	0.011281375	0.01719049
ITGAX	2.009393293	8.93E-13	1.71E-11
MIR200C	2.086171135	0.000185083	0.00042066
P2RY14	-1.859475889	1.06E-11	1.51E-10
E2F1	2.100134715	2.68E-16	2.08E-14
TSC22D3	-1.372220022	4.48E-09	3.12E-08
CLEC7A	1.041595508	1.54E-05	4.52E-05
RHBDF2	1.310820644	4.35E-11	5.24E-10
DUSP1	-1.518445415	2.90E-09	2.13E-08
NTN1	-1.779046603	9.51E-10	7.99E-09
MIR16-1	2.257765053	1.62E-05	4.75E-05
TRIB2	1.141376708	1.10E-07	5.43E-07
APOA1	-3.420695378	0.01064282	0.01630983
TRPM2	1.888795043	1.32E-13	3.24E-12
SERPINB9	1.04100697	1.88E-06	6.83E-06
CD300LF	1.486133964	5.99E-08	3.15E-07
IFIT2	1.172952195	4.43E-05	0.00011629
RGS2	-1.593590636	2.90E-09	2.13E-08
FANCC	1.004380854	1.14E-14	4.08E-13
APOBEC3B	1.750975323	4.25E-08	2.32E-07
CDKN2A	3.092793207	2.89E-06	1.01E-05
DCN	-1.182950285	2.81E-07	1.26E-06
AIRE	2.445756275	0.003595441	0.00611645
ZFPM2	-1.193924647	0.009418371	0.0146039
SERPINE1	2.399299923	2.67E-11	3.42E-10
TREM2	3.061726227	1.55E-15	8.16E-14
AQP3	-2.056412988	0.008554591	0.01338923
TREML2	1.373254404	0.018117188	0.02637588
HSPD1	1.65800331	8.16E-18	2.62E-15
DDX21	1.455914433	9.35E-18	2.90E-15
JAM3	-1.038215349	0.006402469	0.01030102
AHR	1.027721779	6.97E-11	7.97E-10
PMAIP1	1.261742148	2.63E-07	1.18E-06
VLDLR	-1.010366476	2.97E-05	8.13E-05
MIR23B	-2.074149794	0.003376817	0.00577848
CDK6	2.155335299	2.60E-13	5.88E-12
PIK3AP1	1.689007552	1.23E-11	1.72E-10
CASP8	1.025188777	1.23E-12	2.27E-11

IRAK2	1.211470698	2.83E-07	1.27E-06
CD46	1.026641015	3.21E-13	7.04E-12
CEBPD	-1.016087921	2.38E-05	6.68E-05
MMP7	3.717400618	8.87E-09	5.78E-08
C4A	1.283184665	0.000215427	0.00048149
LGALS2	1.762069418	0.017073005	0.02498264
KAT2B	-1.209087786	3.95E-13	8.42E-12
CD36	-1.707240351	5.07E-09	3.49E-08
BCL2A1	1.059604045	3.19E-06	1.10E-05
TRAIP	1.563277895	6.02E-14	1.66E-12
TNIP3	1.349818969	1.91E-05	5.50E-05
CAV1	-1.17518645	0.000107763	0.00025814
CD274	1.696068043	0.000294194	0.00063775
CARD9	1.074734736	1.76E-05	5.10E-05
FCGR2A	1.582316994	9.14E-10	7.74E-09
TRAF5	1.104375673	2.15E-08	1.27E-07
C4BPB	1.729163023	1.85E-05	5.33E-05
C4BPA	5.217518212	2.32E-11	3.03E-10
TRIB3	2.475762576	1.66E-10	1.73E-09
SIGLEC11	-2.32067657	0.000154351	0.00035715
SIGLEC9	1.084904756	5.14E-05	0.00013279
SIGLEC7	1.15037577	5.31E-05	0.00013681
RGMB	-1.094444711	7.64E-08	3.93E-07
OAS2	1.361470833	1.47E-07	7.03E-07
CFB	1.06363506	0.003206988	0.00551764
ADAM10	1.156024517	1.40E-15	7.59E-14
KLK1	1.34521997	0.021326317	0.03055365
EGR1	-1.534420579	0.005848265	0.00949016
F2RL2	2.055547913	7.98E-11	8.99E-10
HOXA9	3.674605389	3.05E-11	3.84E-10
HMGB3	2.048871178	1.24E-16	1.25E-14
PLK1	1.866532638	4.57E-14	1.32E-12
WDR62	2.346803679	1.63E-16	1.50E-14
YJEFN3	1.28676239	1.24E-07	6.03E-07
FCGR1A	2.449499591	1.12E-13	2.82E-12
UBD	2.940480374	3.89E-10	3.65E-09
CD300E	1.352597318	0.025946596	0.03643053
RAD21	1.093261423	4.75E-15	2.01E-13
TPSB2	-1.020224694	6.31E-05	0.00015962
IFI6	1.739378694	5.07E-08	2.72E-07
GLI1	1.152098299	0.001494549	0.00276301

SPON2	1.332394048	5.16E-09	3.54E-08
C7	-1.428345214	6.90E-09	4.60E-08
SLAMF8	1.5730481	1.97E-08	1.17E-07
SLAMF9	1.690136852	1.87E-05	5.40E-05
