# Supplementary material for: An immune-related prognostic signature associated with immune landscape and therapeutic responses in gastric cancer
Source: Aging (Albany NY). 2023 Feb 22;15(4):1074–106. doi: 10.18632/aging.204534 (PMC10008502; doi:10.18632/aging.204534)
Supplement: Supplementary Table 11 [file aging-15-204534-s010.pdf]

Supplementary Table 11. Gene list in turquoise module after WGCNA analysis.

Gene
HSPA2
HSPA6
HSP90AA1
HSP90AB1
PSMD3
SEM1
PROCR
ULBP3
ULBP2
ULBP1
DEFB4A
CXCL16
CXCL5
CXCL1
CXCL12
CXCL3
DEFA6
DEFA5
S100A8
LCN6
S100A12
CCR10
PENK
S100B
S100A7
LCN12
S100A5
S100A7A
ZC3HAV1L
SLC22A17
FABP6
OBP2A
LBP
RBP4
FABP4
RBP7
OBP2B
RBP2
NOX1

PMP2
AEN
TFRC
GDF15
ADIPOQ
CCL20
VEGFA
MAPT
CST4
CSRP1
JUN
TPM2
AHNAK
FGF2
DES
TNFRSF10A
TNFRSF10B
KNG1
KCNH2
CLDN4
PTX3
MASP1
PROC
ABCC4
PCSK2
BIRC5
NOS1
CCL15
CCL14
TAFA4
RNASE2
LYN
VAV2
RAC3
KRAS
FOS
NFKBIE
PRKCB
IGLV3-10
IGLV8-61
CKLF

CMA1
EDN2
FGF10
SAA2
SEMA4F
SEMA6D
SLIT2
CYSLTR1
ACKR1
GPR17
CXCR2
PLXNA1
PLXNA3
AGT
AMH
ANGPTL7
ARTN
BMP3
BMP5
BTC
CGB5
CMTM1
DKK1
ESM1
FAM3D
FGF19
FGF3
GDF10
GDF7
GHRL
GIP
GNRH2
IL17B
IL17C
IL23A
INHBB
INHBE
INSL6
JAG2
LEFTY1
LIF

MDK
MLN
NMB
NPPA
NPPC
NPY
NRG2
NRG3
OGN
OSGIN1
ENDOU
PTN
RLN2
SST
TAC1
TNFRSF11B
TNFSF12
TNFSF15
TNFSF9
TSLP
UCN
VIP
ADCYAP1R1
ADRB2
AGTR1
ANGPT1
ANGPTL1
AR
CNTFR
ESRRB
ESRRG
FGFR4
FGFRL1
GHR
GLP2R
GPER1
IL17RB
LGR5
LIFR
MET
MLNR

NPR1
NPR3
NR2C1
NR4A1
NR4A3
NR5A2
NR6A1
OXTR
PGR
PTGER3
PTH1R
PTH2R
RXRG
TGFBR3
TNFRSF12A
TNFRSF25
TUBB3
VIPR2
BID
CTLA4
EFTUD2
C2
C4B
SKP2
IRAK1
ZBTB16
USP2
PRKX
CCNA2
TXNIP
TRAF2
PRKDC
ASCC3
TRIM24
TRIM50
TRIM71
VTRNA2-1
CFTR
AHSG
OLFM4
MIR200C

P2RY14
E2F1
TSC22D3
DUSP1
NTN1
MIR16-1
TRIB2
APOA1
RGS2
FANCC
APOBEC3B
CDKN2A
DCN
AIRE
ZFPM2
AQP3
TREML2
HSPD1
DDX21
JAM3
AHR
PMAIP1
VLDLR
MIR23B
CDK6
CASP8
IRAK2
CD46
CEBPD
MMP7
C4A
LGALS2
KAT2B
CD36
TRAIP
TNIP3
CAV1
C4BPB
C4BPA
TRIB3
RGMB

CFB
ADAM10
EGR1
HOXA9
HMGB3
PLK1
WDR62
YJEFN3
RAD21
TPSB2
C7
